# Opportunities amid complexities in returning genetic results to black precision medicine research participants: Interview themes in context with open *all of us* data

**DOI:** 10.1017/cts.2025.67

**Published:** 2025-04-11

**Authors:** Rachele M. Hendricks-Sturrup, Nora Emmott, Maryam Nafie, Stephanie Argetsinger, Lauren Edgar, Tracey Johnson-Glover, Kurt D. Christensen

**Affiliations:** 1 Duke-Robert J Margolis, MD, Center for Health Policy Washington, DC, USA; 2 Department of Population Medicine, Harvard Pilgrim Health Care Institute, Boston, MA, USA; 3 Harvard Medical School, Boston, MA, USA; 4 Southern Nevada Black Nurses Association, Las Vegas, NV, USA

**Keywords:** Precision medicine, pharmacogenomics, return of results, health policy, African ancestry

## Abstract

**Objective:**

We sought to describe perspectives among Black nursing professionals and community leaders regarding the return of genetic test results, and place perspectives into context with aggregated findings in the *All of Us* Research Program’s Data Browser.

**Methods:**

Semi-structured, virtual interviews were held with adults (≥18 years of age) self-identifying as Black. A 2-step thematic analysis process was used to assess interviewee perspectives with (sub)themes identified in the literature across two topics: drug/medication response and hereditary disease risk. Themes were placed into context with Data Browser content, focusing on genes and their respective alleles with frequencies ≥0.10 in African ancestry populations in *All of Us*.

**Results:**

Interviewee perspectives aligned with previously identified major themes in the literature (motivations to engage or disengage; integrating research and care), with five (5) subthemes emerging across major themes. Seven (7) alleles were observed with frequencies ≥0.10 for three (3) pharmacogenomic (PGx) biomarkers in the Data Browser for African ancestry populations: *CYP2C19* (SNV, 10-94761900-C-T; SNV,10-94775367-A-G; SNV 10-94781859-G-A), *DPYD* (SNV, 1-97883329-A-G; SNV, 1-97515839-T-C), *UGT1A1* (insertion, 2-233760233-C-CAT; SNV, 2-233757136-G-A). Four (4) alleles were observed with frequencies ≥0.10 for three (3) genes implicated in hereditary disease risk, two of which contemporaneously hold PGx implications for African ancestry populations: *CACNA1S* (PGx, SNV, 1-201112815-C-T; SNV, 1-201110107-C-T), *SCN5A* (no PGx, SNV, 3-38603929-T-C), *TP53* (PGx, SNV, 17-7676154-G-C).

**Conclusions:**

Our findings convey important clinical and translational science considerations for individuals and community leaders of African ancestry and researchers seeking reputable, publicly available information to understand, communicate, and act on genomic findings.

## Introduction

The mission of the *All of Us* Research Program is to accelerate health research and medical breakthroughs by enrolling over one million demographically participants within the United States (US) and collecting health, environmental, and psychosocial data in an ongoing manner. As of September 2024, *All of Us* has registered over 839,000 participants and over 900 research institutions have Data Use and Registration Agreements in place with *All of Us* [1–2]. Over 16% of *All of Us* enrollees self-identify as Black, African American, or African individuals. As *All of Us* continues its work and expands its recruitment efforts, further engagement with participants will be integral to the program’s success.

The scope and design of *All of Us* create opportunities and challenges for specific populations, including individuals of African descent. Consistent with a core value to provide participants access to their information, *All of Us* discloses genomic information about how participants’ genomic information affects their predicted responses to certain medications and about whether participants have genetic risk factors for highly actionable diseases. Concurrently, based on a core value to make data broadly available, any internet user can examine frequencies of genetic variants associated with medication responses or disease risk, whether deleterious, protective, or otherwise, and stratify results by genetic ancestry among *All of Us* participants [3]. Given the expansive size of *All of Us*, the program will need to anticipate potential effects on participants, clinicians, and researchers from its protocols for results disclosure and open access to aggregated data.

Consideration of these issues is particularly important for populations, such as Americans of African descent, for whom the utility of genomic information, and engagement in genomic medicine research, may be less certain and for whom aggregated information poses a risk for misunderstandings or various stigma. For example, in our prior work focused on Black community engagement to devise strategies to engage Black communities in genomic medicine research, we encountered qualitative themes that highlighted this uncertainty and concern about potential misunderstandings or stigma within or about this community [4]. Specifically, key emerging theme descriptions among members of the Black community included but were not limited to: 1) ensuring that patients feel personally empowered and engaged as partners in the management of their health rather than used or undervalued; and 2) ensuring that researchers adhere to standards of research ethics and rigor and are held accountable for how they design, conduct, and report research findings or data about African Americans.

This study is a step to address these issues and concerns by documenting the perspectives of experts who have led efforts to educate individuals of African descent about precision medicine and the benefits and risks of receiving/disclosing individual genomic findings, and also recruit participants for the *All of Us* Research Program in an engaging and informed manner. We also report, based on observations among individuals of African genetic ancestry in the *All of Us* Research Program, the frequency of different types of variants, per the *All of Us* Data Browser, in genes addressed by the *All of Us* Research Program’s “Hereditary Disease Risk” and “Medicine and Your DNA” reports [3]. Our goal is to highlight opportunities and challenges for the *All of Us* program to consider to improve the return of results experience for participants of African descent, their clinicians, and collaborating researchers.

## Methods

### Interview participants

To document experts’ perspectives about the return of genomic information to participants of African descent within the *All of Us* Research Program, we recruited leaders of the National Black Nurses Association (NBNA) to complete semi-structured interviews. NBNA has led national, multi-year efforts to raise awareness and educate community members about precision medicine and *All of Us*. Initial questions asked NBNA members to respond based on personal experiences and experiences as a nursing professional, which was to encourage sharing of their holistic personal perspectives.

Eligible individuals were adults (≥18 years of age) who self-identified as being of African descent and practice within the nursing profession. Interviewees were recruited by members of the project team (R.M.H-S., L.E., T.J-G., and K.D.C.) via email and video calls, and by including invitations in weekly newsletters distributed to NBNA members to a web-based survey that ascertained interest in participating in an interview. Consistent with our prior work, chapter members were professionally or personally familiar with precision or genomic medicine research [4]. Incentives in the form of $25 to $50 gift cards were distributed to participants who completed the electronic survey and/or an interview.

### Interview guide development

In our prior work, we provided strategies and discussed preliminary themes in the literature to describe why addressing return of results needs and concerns among participants of self-reported African descent/ancestry or heritage in the US is critical for both scientific and social reasons [5]. These themes became reflected in our interview guide and centered on the actionability of genetic test results, trust and distrust, protection of privacy, eagerness among individuals to participate in research, and meaningful engagement strategies. The interview guide also addressed interviewees’ personal and professional experiences in the disclosure of both clinical and research-grade genetic findings. Additional questions were asked about attitudes towards specific types of genetic information that could be received through participation in *All of Us*, including results related to medication responses and hereditary disease risks. The interview guide was piloted among the research team members for quality assurance purposes. The final interview guide included 24 questions (see Supplement). Demographic data were not collected during interviews.

### Interviews and transcription

Semi-structured interviews were conducted virtually via Zoom, to minimize burdens to participants, between January and May 2024. Prior to each interview, each interviewee received a brief explanation of the study goals and aims. Oral informed consent was obtained from each interviewee exclusively at the commencement of each interview. Interviewees were informed that they could skip or refuse to answer any question if they wished and could stop the recording at any time. Interviews were recorded and transcribed verbatim for qualitative analysis by an independent third-party service provider. Interview transcriptions and recordings were securely stored within an online server at Duke University.

### Thematic analysis

A 2-step thematic analysis process was used to identify salient themes in the data [6]. The first author (R.M.H-S.) analyzed interview transcripts using NVivo software, carrying out inductive data coding to identify segments that aligned with known themes (“motivations to engage or disengage” and “integrating research and care”) and associated subthemes, organized using Microsoft Excel and manual queries (single layer, bottom-up topic modeling) to categorize illuminating quotes from interview transcripts [7]. The second author (K.D.C.) reviewed coded quotations, and disagreements were discussed until consensus (≥95% agreement) was achieved. Our study followed the Standards for Reporting Qualitative Research reporting guideline [8]. Exemplar quotes among interviewees were identified and lightly edited for inclusion in our results.

### All of us data collection and assessment

A search was conducted in July 2024 within the *All of Us* Research Program Data Browser, which contained participant data updated through February 15, 2023, from 409,420 participants. We searched for single nucleotide variants and short insertions/deletions in each gene listed in the *All of Us* Research Program’s “Hereditary Disease Risk” and “Medicine and Your DNA” reports, and filtered results to compare how allele frequencies vary across subpopulations [3,9]. Analyses focused on allele with frequencies (“hspAFs,” per the Data Browser) >0.10 for individuals with African ancestry (consistent with Masson et al.) to examine how knowledge about even relatively common variants differs by group [10]. Variants with drug response, risk factor, pathogenic, likely pathogenic, or uncertain ClinVar significance were documented in Microsoft Excel and analyzed descriptively among two authors (R.M.H-S. and K.D.C.) to identify opportunities for clinical implementation and further research inquiry. Genes included in the *All of Us* “Medicine and Your DNA” and “Hereditary Disease Risk” reports were cross-referenced with the current US Food and Drug Administration (FDA) “Table of Pharmacogenomic Biomarkers in Drug Labeling” (see Supplement Table 1) to identify variants with both hspAF specific to the African genetic ancestry *All of Us* population and drugs/medications with a current US FDA PGx labeling section [3,11]. Likewise, genes included in the *All of Us* “Hereditary Disease Risk” report were cross-referenced with the US Centers for Disease Control and Prevention (CDC) list of genes and associated conditions with Tier 1 genomic applications (i.e., clinical genetic testing for the following disease areas: hereditary breast and ovarian cancer syndrome [HBOC], Lynch syndrome [LS], and familial hypercholesterolemia [FH]) [3,12].

**Table 1.** Alignment between emerging themes in the literature and present interview findings on return of drug/medication response and hereditary disease risk genetic test results in African ancestry populations

*Categorical themes within the literature*	*Interview results with nursing professionals of self-reported African Ancestry* *(exemplar quote)*	
*Theme 1: Motivations to engage or disengage*	*Topic 1: Drug/Medication Response*	*Topic 2: Hereditary Disease Risk*
*Subtheme 1*: Participants hold a general interest in engaging in the return of results process.	Results may improve shared decision-making about medication selections. (“I like the approach of shared decision-making, where a person has given as much information as they can, not only in writing, but the highlights of things with conversation, to say, “We’re going to try this drug, but we know that it only has a 50% of the efficacy. But here’s why we want to try this drug as opposed to this other drug that’s been on the market for 100 years, and it has a 60% efficacy.” So, I think that information is helpful.”)	Participants want the opportunity to participate in education before and after disclosure of genetic risk information. Participants want to be able to opt in or out of receiving hereditary disease risk results at any point in the research process, including immediately before disclosure. (“I would like to see more of a follow-up or more of an almost like a pre-brief before they get those results or consent to have their genetic testing done.”)
*Subtheme 2*: Engagement in return of results likely depends on the clinical nature or actionability of the results being conveyed.	Results can be interesting, but aren’t necessarily useful in everyday life without specific guidance. Testing may help address issues with trial-and-error approaches to care. Participants may struggle with the complexity of information. (“I would say for me personally, when I got my results, and I’m a healthcare provider, I think it was very confusing and I do not think the layperson that’s not in the medical field may completely understand those results… I’m not sure of the link between getting the results and the practicality of what do I do with that in my everyday life.”)	Concern about possible liability risks associated with interpretation of results and responses to findings. Concern about emotional or psychological harms associated with learning genetic disease risk. (“I guess… it is sort of is individualized. I guess… the legal aspects of it and making sure that there are disclaimers that do not hold you or others liable. But if I have a clear understanding, and I signed a contract, I understand that it’s ultimately on me as the individual, but also having that opportunity that if that individual decides at the last minute that they do not want the information, to accept that desire.” “I think that’s a challenge because what do they do with that information? Are they going to not just be stressed that they might get this disease and there’s nothing they can do? And that woe is me? Or are they going to still participate in a healthy lifestyle and try their best? Yeah, it’s a sticky situation I think when dealing with that information.”)
*Subtheme 3*: Adults may equally engage themselves and their children in return of results.	(None)	Testing can impact personal decisions around family planning and education about genetic risk following return of results. (Ultimately, I think it’s great that people have this ability to find out if they’re at increased risk of certain diseases so that they can take actions ahead of time or that they’re just aware that they may have this gene and get their kids and their family members tested. I definitely think there’s a benefit to the testing. It’s just the implementation of it that I’m just a little bit concerned about.”)
*Theme 2: Integrating research and care*	*Topic 1: Drug/Medication Response*	*Topic 2: Hereditary Disease Risk*
*Subtheme 1*: The return of genetic research results may contribute to misconceptions around the clinical utility of the studies, especially if return of results occurs in usual care (vs research) settings.	Patients become empowered and informed to discuss treatment decisions with clinicians. Clinicians may not be well-informed of the latest research developments in pharmacogenomics. (“I think to me it’s like anything that’s going on now. People are more informed, whether we want them to go online looking up stuff, they’re going to do it. Like you say, what medication may be more likely to respond to this, hopefully, they will use that in their care. They will more than likely look it up themselves, they’ll more than likely say, “Well, doc, you have me on this medication. I went through this program and I had some genetic testing and blah, blah, blah. It basically said that this medication or something works better for me,” and just get their thoughts. I’ve done those kinds of things myself with some things I’ve gone through. I just asked the question. So, it gives you more information to better hopefully advocate for yourself, that type of thing.”	Concern about individual understanding of genetic test results. Researchers should be responsible for following up with both research participants and their health care providers with information about disease risk, treatment, and recommendation of lifestyle changes based on test results. (“I think in the beginning, they give you information and resources that maybe after you get the results. But my fear is that it’s not enough, to be perfectly honest. I think when it comes to genetic testing, people do not really understand that when a test result comes back, it doesn’t mean a hundred percent chance that you’re going to have X, *Y*, and Z disease. People do not know what to do with the information.” “What are we doing to support patients now that they have this information and we’ve identified a risk profile? Do we then have a next step for supporting them in that way? Because for me, there’s a level of irresponsibility… and we know that there’s a risk for this disease. And if we found that risk even more… I think there’s a responsibility on the research side to do some next steps and follow up.”)
*Subtheme 2*: Lack of access to follow-up health care may hinder participants’ ability to address actionable genetic testing results.	Options to refer to specialists may be needed. (“I think it needs to be really clear for genetic researchers to be relaying this information. It needs to be broken down into really simple terms, and then there needs to be some type of follow up - like, oh, here are your results. Here’s what we found about medications that are aligned with your DNA. But, like, what’s next? I think if they have questions, there needs to be proper referrals in place and things like that.”)	Test results should accompany information or guidance about next steps. (“I think there’s a responsibility on the research side to do some next steps and follow up. If nothing else, refer them to someone who might be able to help support if they have questions. Even if it’s primary care and maybe our primary care providers probably are not even well equipped. So, I do not know who that person is. But I think there is a responsibility for All of Us and those folks providing those results. It’s not just like, here you go. Have a great day. I think that if they have questions, what’s the next step for them?”)

### Ethical considerations and institutional review board review

The research protocol, interview guide, interviewee consent mechanisms, and study materials were reviewed and approved by the Duke University (protocol ID # 2022-0514) and Harvard Pilgrim Health Care Institute (registration #IRB00000560) institutional review boards in August 2022. Our analysis of *All of Us* Data Browser content was intended to produce generalizable knowledge based on an assessment of de-identified and aggregated information within the *All of Us* Data Browser. Our analysis solely involved data that is publicly available using the *All of Us* Data Browser and was not intended as human subjects research.

## Results

### Interview details

Interviews were conducted virtually with 5 NBNA chapter leaders in 4 different US states (Connecticut [n = 2], New York [ = 1], Virginia [n = 1], and Nevada [n = 1]), representing 776 local NBNA chapter members [13]. Interviews ranged in duration from 41 to 67 minutes in length.

### Thematic assessment 1: motivations to engage or disengage

Interviewees’ perspectives on the topic of returning results about medication responses highlighted generally positive attitudes about the benefits of disclosing pharmacogenomic information about a variety of medications and indications, as well as alignment with previously identified themes within the literature (see Table 1). Interviewees addressed how information may motivate participants to engage in shared decision-making with clinicians about medications to incorporate the latest and/or robust evidence of treatment efficacy. They also addressed how results may help address issues with trial and error medication/step therapy approaches to care (e.g., time to desired treatment effect), especially for individuals whose existing medications may not be working well and individuals who may be able to reduce the large number of medications that they’re currently taking.

Interviewee perspectives about returning results about hereditary disease risks also affirmed a general interest in engaging in the return of results that depended on the clinical nature and actionability of the results. Interviewees expressed that participants desire education both before and after disclosure of hereditary disease risks about the value of understanding family and hereditary disease risks. Interviewees also emphasized how participants value being able to opt in and out of receiving test results at any point in the research process, as well as the opportunity to learn about hereditary disease risks based on known or unknown ancestry. Interviewees also expressed concern that disclosure could introduce liabilities to clinicians and researchers regarding test result interpretation and implementation process, as well as possible negative emotional or psychological impacts associated with learning genetic disease risk. Regarding familial outcomes, interviewees emphasized how hereditary disease risk findings could impact personal decisions around family planning.

### Thematic assessment 2: integrating research and care

Like Thematic Assessment 1, we observed interviewee alignment with previously identified themes within the literature (see Table 1). Within the theme of “integrating research and care,” interviewees addressed how returning genetic research results may contribute to misconceptions around the clinical utility of the studies, especially if return of results occurs in usual care settings. Interviewees discussed how disclosure of drug/medication response information would allow *All of Us* participants to become empowered and informed to discuss treatment decisions with clinicians who may or may not be well-informed of the latest research developments.

Interviewees’ perspectives on the topic of returning results about hereditary disease risk affirmed that lack of access to follow-up health care may hinder participants’ ability to address actionable genetic testing results. Concerns about whether individuals would understand the genetic information disclosed to them were also expressed. Interviewees also expressed an opinion that researchers are responsible for following up with both research participants and their health care providers with information about disease risk, treatment, and recommended lifestyle changes based on disclosed risk information. To address these concerns, interviewees suggested that disclosure of hereditary disease risks should include information and guidance about recommended next steps.

### All of us data browser analysis

Self-reported race/ethnicity among *All of Us* study participants showed as follows: Asian (3.0%; *n* = 7,440); Black, African American, or African (20.4%; *n* = 50,080); Hispanic, Latino, or Spanish (17.1%; *n* = 41,940); White (51.3%; *n* = 125,860); more than once race/ethnicity (3.8%; *n* = 9,220); other (1.7%; *n* = 4,040); and prefer not to answer (2.8%; *n* = 6,880). We observed variants with ClinVar drug response significance and allele frequencies of at least 0.10 within the *All of Us* African genetic ancestry population for three of seven genes (43%) included in the *All of Us* “Medicine and Your DNA” report, including three *CYP2C19,* two *DPYD* variants, and two *UGT1A1* variants (Table 2 and Supplement Table 2). For *CYP2C19*, over half (n = 6; 55%) of drugs included in the *All of Us* “Medicine and Your DNA” report currently have regulatory labeling (i.e., US FDA PGx labeling section; see Table 2 and Supplement Tables 1-2): citalopram, clobazam, clopidogrel, doxepin, escitalopram, and voriconazole.[11] All drugs included in the *All of Us* “Medicine and Your DNA” report for *DPYD* (n = 2; 100%) currently have a US FDA PGx labeling section (capecitabine and fluorouracil). Lastly, no drugs (n = 0; 0%) included in the *All of Us* “Medicine and Your DNA” report for *UGT1A1* currently have a US FDA PGx labeling section. Among all variants across all three genes (*CYP2C19*, *DPYD*, *UGT1A1*), 99.3% to 99.9% have undefined/uncertain ClinVar significance as of July 2024.

**Table 2.** Summary of genes included in the *all of us* “Medicine and your DNA” report and variants with allele frequencies (hspAF) ≥0.10 in participants of African genetic ancestry and present drug response significance classification in clinVar^a^

PGx Biomarker	Drug interactions considered*	Variants Assigned Drug Response Significance Classification in ClinVar & hspAF in the *All of Us* African Ancestry Population (Variant Type; Allele Count; Allele Frequency)
*CYP2C19*	amitriptyline (Elavil®)	10-94761900-C-T (SNV; 107,890; 0.222523) 10-94775367-A-G (SNV; 107,886; 0.198450) 10-94781859-G-A (SNV; 107,890; 0.177876)
	citalopram (Celexa®)*	
	clobazam (Onfi®)*	
	clomipramine (Anafranil®)	
	clopidogrel (Plavix®)*	
	doxepin (Sinequan®)*	
	escitalopram (Lexapro®)*	
	imipramine (Tofranil®)	
	sertraline (Zoloft®)	
	trimipramine (Surmontil®)	
	voriconazole (Vfend®)*	
*DPYD*	capecitabine (Xeloda®)*	1-97883329-A-G (SNV; 44,373; 0.411265) 1-97515839-T-C (SNV; 17,056; 0.158081)
	fluorouracil (Adrucil®)*	
*UGT1A1*	atazanavir (Reyataz®)	2-233760233-C-CAT (insertion; 107,882; 0.401207) 2-233757136-G-A (SNV; 107,828; 0.305394)
	belinostat (Beleodaq®)	
	irinotecan (Camptosar®)	

a Genes are summarized with associated drug interactions, per FDA PGx biomarker labeling, and allele frequencies as of July 2024.

* Contains a United States Food and Drug Administration pharmacogenomic biomarker labeling section

SNV = single nucleotide variant.

We also observed that 12 genes listed in the *All of Us* “Hereditary Disease Risk” report also have variants associated with drug response significance in ClinVar or a US FDA PGx labeling section (Table 3 and Supplement Tables 1 and 3) [11]. Yet these same genes are not included in the *All of Us* “Medicine and Your DNA” report despite US FDA and ClinVar concordance in some cases. Specifically, we observed one gene (*APOB*) with ClinVar drug response significance but no current US FDA PGx labeling section. We also observed six genes (n = 6) without ClinVar drug response significance but a current US FDA PGx labeling section: *BRCA1/2* (oncology, five drugs [n = 5]), *APC* (oncology, one drug [n = 1]), LDLR (endocrinology, one drug [n = 1]), *LMNA* (inborn errors of metabolism, one drug [n = 1]), *PTEN* (oncology, one drug [n = 1]), *RET* (oncology, two drugs [n = 2]). Lastly, we observed five genes (n = 5) with assigned ClinVar drug response significance and a US FDA PGx labeling section: *WT1* (oncology, one drug [n = 1]), *CACNA1S* (anesthesiology, four drugs [n = 4]), *GLA* (inborn errors of metabolism, one drug [n = 1]), *RYR1* (anesthesiology, four drugs [n = 4]), *TP53* (oncology, three drugs [n = 3]). However, no genes included in the *All of Us* “Hereditary Disease Risk” report contain variants with allele frequencies ≥0.10 within the *All of Us* African genetic ancestry population and either an assigned ClinVar drug response significance and/or a US FDA PGx labeling section. Among all variants across all 12 genes (*APC, BRCA1, BRCA2, CACNA1S, GLA, LDLR, LMNA, PTEN, RET, RYR1, TP53, WT1*), 97.4% to 99.2% have undefined/uncertain ClinVar significance as of July 2024.

**Table 3.** Summary of genes included in the *all of us* “Hereditary disease risk” report with allele frequencies (hspAF) ≥0.10 in participants of African genetic ancestry for variants with present risk factor, likely pathogenic, or pathogenic classification in clinVar and regulatory drug labeling^a^

Gene	Condition associated with this gene	What it is	Variants with Risk Factor, Likely Pathogenic, or Pathogenic Significance Classification in ClinVar (Variant Type; Allele Count; Allele Frequency)	Variants with Drug Response Significance	FDA PGx Drug Labeling Section (Therapeutic Area)
*CACNA1S*	Malignant hyperthermia susceptibility	Hypermetabolic reaction to certain anesthetics and muscle relaxants	1-201112815-C-T (SNV; 73,407; 0.680362) 1-201110107-C-T (SNV; 30,531; 0.282972)	None	Desflurane, Isoflurane, Sevoflurane, Succinylcholine (Anesthesiology)
*SCN5A*	Brugada syndrome and long QT syndrome 3	Heart arrhythmias	3-38603929-T-C (SNV; 31,805; 0.294802)	None	None
*TP53*	Li-Fraumeni syndrome	Increased risk for multiple types of cancers	17-7676154-G-C (SNV; 41,433; 0.384037)	None	Pirtobrutinib, Venetoclax, Zanubrutinib (Oncology)

a Genes are summarized with their associated diseases, allele frequencies, assigned drug response significance, and FDA PGx biomarker labeling as of July 2024.

SNV = single nucleotide variant.

Lastly, we observed that among the three disease areas with CDC Tier 1 genomic applications (HBOC, LS, FH) [12] and included in the *All of Us* “Hereditary Disease Risk” report, no associated variants for genes implicated in those diseases (*BRCA1, BRCA2* for HBOC; *MLH1, MSH2, MSH6, PMS2* for LS; and *PCSK9, APOB, LDLR* for FH) were present within the *All of Us* population with African genetic ancestry at allele frequencies of at least 0.10 (see Table 3 and Supplement Table 3). Among all variants across all 9 genes (*BRCA1, BRCA2, MLH1, MSH2, MSH6, PMS2, PCSK9, APOB, LDLR)*, 94.9% to 99.2% have undefined/uncertain ClinVar significance as of July 2024. However, we observed three genes (n = 3) included in the *All of Us* “Hereditary Disease Risk” report with variants at an allele frequency≥0.10 in the *All of Us* African genetic ancestry population: *CACNA1S* (two gene variants [n = 2], malignant hyperthermia susceptibility), *SCN5A* (one variant [n = 1], Brugada syndrome and long QT syndrome 3), and *TP53* (one variant [n = 1], Li-Fraumeni syndrome; see Table 3 and Supplement Table 3). Among all variants across all 3 genes (*CACNA1S, SCN5A, TP53*), 97.4% to 98.3% have undefined/uncertain ClinVar significance as of July 2024.

## Discussion

Our qualitative and quantitative methods and findings, together, demonstrate the value and potential of precision medicine research and possible translation of findings for diverse ancestral subpopulations, including but not limited to those of African ancestry. Our interview findings confirmed how leaders who engage with their communities about precision medicine research had hopes that enrollment and disclosure of individual findings would improve patient care not just by improving medication decisions and identifying disease risks for participants and their families, but also by leading to more robust interactions with clinicians. This is especially true for participants seeking clinician perspectives to understand the complex relationship between health and genetics. Community leaders conveyed an understanding of potential risks to clinicians and researchers, in addition to participants, about adverse psychosocial responses and potential liabilities. They also addressed the potential for therapeutic misconceptions of research participation and the possibility that medical benefits could be compromised by misunderstandings and lack of access to medical care. Ultimately, respondents emphasized the need for *All of Us* to complement information with recommendations about what participants and their families should *do* with results outside of the study setting.

The diversity of the *All of Us* Research Program cohort is a major success. A February 2024 report described *All of Us* participants with self-reported African ancestry as (total *n* = 236,021) as: Black or African American (inclusive of admixed European and West Asian ancestry; 21.2%; *n* = 50,064); Middle Eastern or North African (inclusive of admixed West Asian, South Asian, African, and European ancestry; 0.06%; *n* = 1,301); Native Hawaiian or Other Pacific Islander (inclusive of admixed East Asian, South Asian, American, West Asian, and African ancestry; 0.01%; *n* = 237); and more than one population (admixed European, American, African, West Asian, East Asian, and South Asian; 3.9%; *n* = 9,216) [14]. Our quantitative findings also highlight the translational potential of publicly-available findings within *All of Us* for populations of African ancestry. Variants with drug response classifications and allele frequencies as high as 40% were observed among participants of African descent in *DPYD* and *UGT1A1* and thus may affect response to chemotherapeutic or antiretroviral medications in this subgroup (an observation that should be validated both scientifically and clinically). In addition, three variants with drug response classifications in *CYP2C19* were observed with more moderate allele frequencies of approximately 20%, which may affect response to medications for more common conditions (e.g., antidepressants, anti-fungal agents). Many of the medications associated with PGx findings in those three genes have FDA biomarker labeling sections that address how to use the PGx information, which if implemented could result in greater education of participants and their clinicians to ensure accurate translation of findings. Policymakers, including but not limited to those drafting and updating insurance coverage policies for PGx-based prescribing, and medical product regulators may also need to address how some of the medications with established PGx associations currently lack FDA labels. Nevertheless, the return of aggregated and individual genomic results was prioritized by *All of Us* to return value to communities and participants who are or have been underrepresented in genomics research. This includes individuals of African ancestry who may also identify as Black or African American, Middle Eastern or North African, and/or Native Hawaiian or Other Pacific Islander, thus creating tremendous scientific value and opportunity for these communities.

Our data browser findings also raise general concerns about present allele classifications in ClinVar. Frequencies of a few pathogenic or likely pathogenic variants were greater than 10% in *CACNA1S*, *SCN5A* and *TP53*, raising questions about their current variant classification in ClinVar. These allele frequencies exceed the minor allele frequency thresholds of 0.05 used by the Clinical Genome Resource Sequence Variant Interpretation Working Group, the American College of Medical Genetics and Genomics, and the Association of Molecular Pathologists as evidence of non-pathogenicity [15,16]. In addition, because the frequency of meaningful variants in genes associated with disease risk and medication risk is relatively rare, participants may be motivated to use the public data browser to understand the frequency of meaningful variants in individuals of African genetic descent. If so, they are likely to observe how the vast majority of variants are classified as having unknown or undefined clinical significance. Moreover, classifying alleles showing unusually high frequency (allele frequency >0.50, in particular) as a “variant” may be confusing to members of the public who are unlikely to understand that such language has been retained to accommodate existing laboratory bioinformatics pipelines. As *All of Us* considers the future of returning results, the program may need to ensure the scientific validity or robustness of current reference genomes used to classify alleles in ClinVar, the accuracy of individual findings for individuals of African and other genetic ancestry, the need for participants to receive guidance about what they should do next with their information, and the possibility for aggregated data to be confusing to public observers.

Our study is accompanied by two notable limitations. First, we experienced challenges in recruiting more than five interviewees due to scheduling constraints among NBNA members during the recruitment period. However, merging Black perspectives found within peer-reviewed scientific literature to date on return of results with the five perspectives obtained in our study may improve the reliability of our present findings. Nonetheless, future qualitative work is needed to strengthen the validity and reliability of our present findings. Second, work is needed to continue to evaluate variants found among *All of Us* participants of any significance level, based on ClinVar assessments, in a scientifically astute, clinically validated, timely, and ethical, legal, and social implications (ELSI)-informed manner for effective translation. In February 2024, *All of Us* announced that its researchers have identified or discovered more than 275 million previously unreported genetic variants. In May 2024, *All of Us* then announced its return of personalized health-related DNA results to more than 100,000 participants in the program (an initiative that is anticipated to be ongoing). Over 7,000 genetic variants described in the reports had never been observed among people with prior genetic testing yet are associated with certain serious health conditions. These developments have been captured, according to the program, within ClinVar to help inform health care providers and researchers who use the database to help diagnose and manage patients and identify new areas of research [17].

However, there are oftentimes unresolved conflicting classifications of variants, as well as upgrading and downgrading of variant classifications in ClinVar over time [16,18,19]. Thus, an overreliance on ClinVar without a close and independent assessment of possibilities for variant reclassifications could involve risk and/or become a source of confusion or misinterpretation of genetic information among its users. Altogether, we believe our present findings provide a much-needed form of preliminary evidence on how researchers should utilize genomic information based on the current state of evidence and share that information in a way that aligns with the values of and addresses concerns among individuals of African descent.

## Conclusion

As the *All of Us* Research Program continues its successful mission to accelerate and inform health research and medical breakthroughs by enrolling over one million participants of diverse ancestries. Community leaders who inform and engage their constituents about precision medicine research opportunities like *All of Us* anticipate that the disclosure of individual genomic research findings carry a promise or community expectation to help improve patient and patient family education and support the integration of research into care. In the case of populations of African ancestry with who carry alleles, at a moderate to high frequency, with current drug response significance and/or hereditary disease risk indications based on current ClinVar classifications, policymakers, and regulators should collaborate with patients, providers, researchers, and community leaders to ensure accurate, reliable, and trustworthy translation of genomic findings. Such value will be important for research communities including and like *All of Us* in their pursuits to return translational scientific value to communities and participants in a trusted, scientifically rigorous, and clinically sound manner.

## Supporting information

Hendricks-Sturrup et al. supplementary material 1 Hendricks-Sturrup et al. supplementary material

Hendricks-Sturrup et al. supplementary material 2 Hendricks-Sturrup et al. supplementary material

Hendricks-Sturrup et al. supplementary material 3 Hendricks-Sturrup et al. supplementary material

## Supplementary material

The supplementary material for this article can be found athttps://doi.org/10.1017/cts.2025.67.
