# Supplementary material for: Opportunities amid complexities in returning genetic results to black precision medicine research participants: Interview themes in context with open all of us data
Source: J Clin Transl Sci. 2025 Apr 11;9(1):e89. doi: 10.1017/cts.2025.67 (PMC12089846; doi:10.1017/cts.2025.67)
Supplement: Hendricks-Sturrup et al. supplementary material 1 — Hendricks-Sturrup et al. supplementary material [file S2059866125000676sup001.pdf]

Table of Pharmacogenomic Biomarkers in Drug Labeling

Last Updated: 06/2024

Inclusion criteria: germline or somatic gene variants (polymorphisms, mutations), functional deficiencies with a genetic etiology, gene expression differences, chromosomal abnormalities; selected proteins that are used for treatment selection are also included;

Exclusion criteria: non-human genetic factors (e.g., viral or bacterial), biomarkers used for disease diagnostic purposes that are not used to determine dosing or treatment selection within the diagnosed disease, and biomarkers that are related to a drug other than the referenced drug (e.g., influences the effect of the referenced drug as a perpetrator of an interaction with another drug)

NDA/ANDA/BLA Number, Label Version Date	Drug	Therapeutic Area*	Biomarker†	Labeling Sections	Labeling Text‡
020977, 03/20/2017	Abacavir	Infectious Diseases	HLA-B	Boxed Warning, Dosage and Administration, Contraindications, Warnings and Precautions	**BOXED WARNING**<br>WARNING: HYPERSENSITIVITY REACTIONS, and LACTIC ACIDOSIS, AND SEVERE HEPATOMEGALY<br>*Hypersensitivity Reactions*<br>Serious and sometimes fatal hypersensitivity reactions, with multiple organ involvement, have occurred with ZIAGEN® (abacavir).<br>Patients who carry the HLA-B*5701 allele are at a higher risk of a hypersensitivity reaction to abacavir; although, hypersensitivity reactions have occurred in patients who do not carry the HLA-B*5701 allele [see Warnings and Precautions (5.1)].<br>ZIAGEN is contraindicated in patients with a prior hypersensitivity reaction to abacavir and in HLA-B*5701-positive patients [see Contraindications (4), Warnings and Precautions (5.1)]. All patients should be screened for the HLA-B*5701 allele prior to initiating therapy with ZIAGEN or reinitiation of therapy with ZIAGEN, unless patients have a previously documented HLA-B*5701 allele assessment. Discontinue ZIAGEN immediately if a hypersensitivity reaction is suspected, regardless of HLA-B*5701 status and even when other diagnoses are possible [see Contraindications (4), Warnings and Precautions (5.1)].<br>Following a hypersensitivity reaction to ZIAGEN, NEVER restart ZIAGEN or any other abacavir-containing product because more severe symptoms, including death can occur within hours. Similar severe reactions have also occurred rarely following the reintroduction of abacavir-containing products in patients who have no history of abacavir hypersensitivity [see Warnings and Precautions (5.1)]. (…)<br><br>**2 DOSAGE AND ADMINISTRATION**<br>**2.1 Screening for HLA-B*5701 Allele Prior to Starting ZIAGEN**<br>Screen for the HLA-B*5701 allele prior to initiating therapy with ZIAGEN [see Boxed Warning, Warnings and Precautions (5.1)].<br><br>**4 CONTRAINDICATIONS**<br>ZIAGEN is contraindicated in patients:<br>• who have the HLA-B*5701 allele [see Warnings and Precautions (5.1)].<br><br>**5 WARNINGS AND PRECAUTIONS**<br>**5.1 Hypersensitivity Reactions**<br>Serious and sometimes fatal hypersensitivity reactions have occurred with ZIAGEN (abacavir). These hypersensitivity reactions have included multi-organ failure and anaphylaxis and typically occurred within the first 6 weeks of treatment with ZIAGEN (median time to onset was 9 days); although abacavir hypersensitivity reactions have occurred any time during treatment [see Adverse Reactions (6.1)]. Patients who carry the HLA B*5701 allele are at a higher risk of abacavir hypersensitivity reactions; although, patients who do not carry the HLA-B*5701 allele have developed hypersensitivity reactions. Hypersensitivity to abacavir was reported in approximately 206 (8%) of 2,670 patients in 9 clinical trials with abacavir-containing products where HLA-B*5701 screening was not performed. The incidence of suspected abacavir hypersensitivity reactions in clinical trials was 1% when subjects carrying the HLA-B*5701 allele were excluded. In any patient treated with abacavir, the clinical diagnosis of hypersensitivity reaction must remain the basis of clinical decision making.<br>Due to the potential for severe, serious, and possibly fatal hypersensitivity reactions with ZIAGEN:<br>• All patients should be screened for the HLA-B*5701 allele prior to initiating therapy with ZIAGEN or reinitiation of therapy with ZIAGEN, unless patients have a previously documented HLA-B*5701 allele assessment.<br>• ZIAGEN is contraindicated in patients with a prior hypersensitivity reaction to abacavir and in HLA-B*5701-positive patients.<br>• Before starting ZIAGEN, review medical history for prior exposure to any abacavir containing product. NEVER restart ZIAGEN or any other abacavir-containing product following a hypersensitivity reaction to abacavir, regardless of HLA-B*5701 status.<br>• To reduce the risk of a life-threatening hypersensitivity reaction, regardless of HLA-B*5701 status, discontinue ZIAGEN immediately if a hypersensitivity reaction is suspected, even when other diagnoses are possible (e.g., acute onset respiratory diseases such as pneumonia, bronchitis, pharyngitis, or influenza; gastroenteritis; or reactions to other medications).<br>• If a hypersensitivity reaction cannot be ruled out, do not restart ZIAGEN or any other abacavir-containing products because more severe symptoms which may include life-threatening hypotension and death, can occur within hours.<br>• If a hypersensitivity reaction is ruled out, patients may restart ZIAGEN. Rarely, patients who have stopped abacavir for reasons other than symptoms of hypersensitivity have also experienced life-threatening reactions within hours of reinitiating abacavir therapy. Therefore, reintroduction of ZIAGEN or any other abacavir containing product is recommended only if medical care can be readily accessed.<br>• A Medication Guide and Warning Card that provide information about recognition of hypersensitivity reactions should be dispensed with each new prescription and refill.
**208716, 03/03/2023**	**Abemaciclib (1)**	Oncology	ESR (Hormone Receptor)	Indications and Usage, Adverse	**1 INDICATIONS AND USAGE**<br>**1.1 Early Breast Cancer**

* Therapeutic areas do not necessarily reflect the CDER review division.

† Representative biomarkers are listed based on standard nomenclature as per the Human Genome Organization (HUGO) symbol and/or simplified descriptors using other common conventions. Listed biomarkers do not necessarily reflect the terminology used in labeling. The term "Nonspecific" is provided when labeling does not explicitly identify the specific biomarker(s) or when the biomarker is represented by a molecular phenotype or gene signature, and in some cases the biomarker was inferred based on the labeling language.

‡ Referenced figures and tables may be found in the labeling available at Drugs@FDA.

Blue text represents the most recent addtions and/or changes since last posted version.

NDA/ANDA/BLA Number, Label Version Date	Drug	Therapeutic Area*	Biomarker†	Labeling Sections	Labeling Text‡
				Reactions, Clinical Studies	VERZENIO® (abemaciclib) is indicated:<br>• in combination with endocrine therapy (tamoxifen or an aromatase inhibitor) for the adjuvant treatment of adult patients with hormone receptor (HR)-positive, human epidermal growth factor receptor 2 (HER2)-negative, nodepositive, early breast cancer at high risk of recurrence [see Clinical Studies (14.1)].<br>**1.2 Advanced or Metastatic Breast Cancer**<br>VERZENIO (abemaciclib) is indicated:<br>• in combination with an aromatase inhibitor as initial endocrine-based therapy for the treatment adult patients with hormone receptor (HR)-positive, human epidermal growth factor receptor 2 (HER2)-negative advanced or metastatic breast cancer.<br>• in combination with fulvestrant for the treatment of adult patients with hormone receptor (HR)-positive, human epidermal growth factor receptor 2 (HER2)-negative advanced or metastatic breast cancer with disease progression following endocrine therapy.<br>• as monotherapy for the treatment of adult patients with HR-positive, HER2-negative advanced or metastatic breast cancer with disease progression following endocrine therapy and prior chemotherapy in the metastatic setting.<br><br>**6 ADVERSE REACTIONS**<br>**Early Breast Cancer**<br>monarchE: VERZENIO in Combination with Tamoxifen or an Aromatase Inhibitor as Adjuvant Treatment<br>Adult patients with HR-positive, HER2-negative, node-positive early breast cancer at a high risk of recurrence The safety of VERZENIO was evaluated in monarchE, a study of 5591 adult patients receiving VERZENIO plus endocrine therapy (tamoxifen or an aromatase inhibitor) or endocrine therapy (tamoxifen or an aromatase inhibitor) alone [see Clinical Studies (14.1)]. Patients were randomly assigned to receive 150 mg of VERZENIO orally, twice daily, plus tamoxifen or an aromatase inhibitor, or tamoxifen or an aromatase inhibitor, for two years or until discontinuation criteria were met. The median duration of VERZENIO treatment was 24 months.<br>**Advanced or Metastatic Breast Cancer**<br>**MONARCH 3: VERZENIO in Combination with an Aromatase Inhibitor (Anastrozole or Letrozole) as Initial EndocrineBased Therapy**<br>*Postmenopausal Women with HR-positive, HER2-negative locoregionally recurrent or metastatic breast cancer with no prior systemic therapy in this disease setting*<br>MONARCH 3 was a study of 488 women receiving VERZENIO plus an aromatase inhibitor or placebo plus an aromatase inhibitor. Patients were randomly assigned to receive 150 mg of VERZENIO or placebo orally twice daily, plus physician's choice of anastrozole or letrozole once daily. Median duration of treatment was 15.1 months for the VERZENIO arm and 13.9 months for the placebo arm. Median dose compliance was 98% for the VERZENIO arm and 99% for the placebo arm. (…)<br>***MONARCH 2: VERZENIO in Combination with Fulvestrant***<br>*Women with HR-positive, HER2-negative advanced or metastatic breast cancer with disease progression on or after prior adjuvant or metastatic endocrine therapy*<br>The safety of VERZENIO (150 mg twice daily) plus fulvestrant (500 mg) versus placebo plus fulvestrant was evaluated in MONARCH 2. The data described below reflect exposure to VERZENIO in 441 patients with HR-positive, HER2-negative advanced breast cancer who received at least one dose of VERZENIO plus fulvestrant in MONARCH 2. (…)<br>MONARCH 1: VERZENIO Administered as a Monotherapy in Metastatic Breast Cancer<br>*Patients with HR-positive, HER2-negative breast cancer who received prior endocrine therapy and 1-2 chemotherapy regimens in the metastatic setting*<br>Safety data below are based on MONARCH 1, a single-arm, open-label, multicenter study in 132 women with measurable HR-positive, HER2-negative metastatic breast cancer. Patients received 200 mg VERZENIO orally twice daily until development of progressive disease or unmanageable toxicity. Median duration of treatment was 4.5 months. (…)<br><br>**14 CLINICAL STUDIES**<br>**14.1 Early Breast Cancer**<br>VERZENIO in Combination with Standard Endocrine Therapy (monarchE)<br>*Patients with HR-positive, HER2-negative, node-positive early breast cancer at high risk of recurrence*<br>monarchE (NCT03155997) was a randomized (1:1), open-label, two cohort, multicenter study in adult women and men with HR-positive, HER2-negative, node-positive, resected, early breast cancer with clinical and pathological features consistent with a high risk of disease recurrence. To be enrolled in cohort 1, patients had to have HR positive, HER2 negative early breast cancer with tumor involvement in at least 1 axillary lymph node (pALN) and either:<br>• ≥4 pALN or<br>• 1-3 pALN and at least one of: – tumor grade 3 – tumor size ≥ 50 mm. Patients with available untreated breast tumor samples were tested retrospectively at central sites using the Ki-67 IHC MIB-1 pharmDx (Dako Omnis) assay to establish if the Ki-67 score was ≥20%, specified in the protocol as "Ki-67 high".<br>**14.2 Advanced or Metastatic Breast Cancer**<br>*VERZENIO in Combination with an Aromatase Inhibitor (Anastrozole or Letrozole) (MONARCH 3)*<br>*Postmenopausal women with HR-positive, HER2-negative advanced or metastatic breast cancer with no prior systemic therapy in this disease setting*<br>MONARCH 3 was a randomized (2:1), double-blinded, placebo-controlled, multicenter study in postmenopausal women with HR-positive, HER2-negative advanced or metastatic breast cancer in combination with a nonsteroidal aromatase inhibitor as initial endocrine-based therapy, including patients not previously treated with systemic therapy for breast cancer. (…)<br>***VERZENIO in Combination with Fulvestrant (MONARCH 2)***

* Therapeutic areas do not necessarily reflect the CDER review division.
† Representative biomarkers are listed based on standard nomenclature as per the Human Genome Organization (HUGO) symbol and/or simplified descriptors using other common conventions. Listed biomarkers do not necessarily reflect the terminology used in labeling. The term "Nonspecific" is provided when labeling does not explicitly identify the specific biomarker(s) or when the biomarker is represented by a molecular phenotype or gene signature, and in some cases the biomarker was inferred based on the labeling language.
‡ Referenced figures and tables may be found in the labeling available at Drugs@FDA.
Blue text represents the most recent addtions and/or changes since last posted version.

NDA/ANDA/BLA Number, Label Version Date	Drug	Therapeutic Area*	Biomarker†	Labeling Sections	Labeling Text‡
					Patients with HR-positive, HER2-negative advanced or metastatic breast cancer with disease progression on or after prior adjuvant or metastatic endocrine therapy<br>MONARCH 2 (NCT02107703) was a randomized, placebo-controlled, multicenter study in women with HR-positive, HER2-negative metastatic breast cancer in combination with fulvestrant in patients with disease progression following endocrine therapy who had not received chemotherapy in the metastatic setting. (…)<br>***VERZENIO Administered as a Monotherapy in Metastatic Breast Cancer (MONARCH 1)***<br>*Patients with HR-positive, HER2-negative breast cancer who received prior endocrine therapy and 1-2 chemotherapy regimens in the metastatic setting*<br>MONARCH 1 (NCT02102490) was a single-arm, open-label, multicenter study in women with measurable HR-positive, HER2-negative metastatic breast cancer whose disease progressed during or after endocrine therapy, had received a taxane in any setting, and who received 1 or 2 prior chemotherapy regimens in the metastatic setting. (…)
**208716, 03/03/2023**	Abemaciclib (2)	Oncology	ERBB2 (HER2)	Indications and Usage, Adverse Reactions, Clinical Studies	**1 INDICATIONS AND USAGE**<br>**1.1 Early Breast Cancer**<br>VERZENIO® (abemaciclib) is indicated:<br>• in combination with endocrine therapy (tamoxifen or an aromatase inhibitor) for the adjuvant treatment of adult patients with hormone receptor (HR)-positive, human epidermal growth factor receptor 2 (HER2)-negative, nodepositive, early breast cancer at high risk of recurrence [see Clinical Studies (14.1)].<br>**1.2 Advanced or Metastatic Breast Cancer**<br>VERZENIO (abemaciclib) is indicated:<br>• in combination with an aromatase inhibitor as initial endocrine-based therapy for the treatment adult patients with hormone receptor (HR)-positive, human epidermal growth factor receptor 2 (HER2)-negative advanced or metastatic breast cancer.<br>• in combination with fulvestrant for the treatment of adult patients with hormone receptor (HR)-positive, human epidermal growth factor receptor 2 (HER2)-negative advanced or metastatic breast cancer with disease progression following endocrine therapy.<br>• as monotherapy for the treatment of adult patients with HR-positive, HER2-negative advanced or metastatic breast cancer with disease progression following endocrine therapy and prior chemotherapy in the metastatic setting.<br><br>**6 ADVERSE REACTIONS**<br>**Early Breast Cancer**<br>monarchE: VERZENIO in Combination with Tamoxifen or an Aromatase Inhibitor as Adjuvant Treatment<br>Adult patients with HR-positive, HER2-negative, node-positive early breast cancer at a high risk of recurrence The safety of VERZENIO was evaluated in monarchE, a study of 5591 adult patients receiving VERZENIO plus endocrine therapy (tamoxifen or an aromatase inhibitor) or endocrine therapy (tamoxifen or an aromatase inhibitor) alone [see Clinical Studies (14.1)]. Patients were randomly assigned to receive 150 mg of VERZENIO orally, twice daily, plus tamoxifen or an aromatase inhibitor, or tamoxifen or an aromatase inhibitor, for two years or until discontinuation criteria were met. The median duration of VERZENIO treatment was 24 months.<br>**Advanced or Metastatic Breast Cancer**<br>**MONARCH 3: VERZENIO in Combination with an Aromatase Inhibitor (Anastrozole or Letrozole) as Initial EndocrineBased Therapy**<br>*Postmenopausal Women with HR-positive, HER2-negative locoregionally recurrent or metastatic breast cancer with no prior systemic therapy in this disease setting*<br>MONARCH 3 was a study of 488 women receiving VERZENIO plus an aromatase inhibitor or placebo plus an aromatase inhibitor. Patients were randomly assigned to receive 150 mg of VERZENIO or placebo orally twice daily, plus physician's choice of anastrozole or letrozole once daily. Median duration of treatment was 15.1 months for the VERZENIO arm and 13.9 months for the placebo arm. Median dose compliance was 98% for the VERZENIO arm and 99% for the placebo arm.<br>**MONARCH 2: VERZENIO in Combination with Fulvestrant**<br>*Women with HR-positive, HER2-negative advanced or metastatic breast cancer with disease progression on or after prior adjuvant or metastatic endocrine therapy*<br>The safety of VERZENIO (150 mg twice daily) plus fulvestrant (500 mg) versus placebo plus fulvestrant was evaluated in MONARCH 2. The data described below reflect exposure to VERZENIO in 441 patients with HR-positive, HER2-negative advanced breast cancer who received at least one dose of VERZENIO plus fulvestrant in MONARCH 2. (…)<br>MONARCH 1: VERZENIO Administered as a Monotherapy in Metastatic Breast Cancer<br>*Patients with HR-positive, HER2-negative breast cancer who received prior endocrine therapy and 1-2 chemotherapy regimens in the metastatic setting*<br>Safety data below are based on MONARCH 1, a single-arm, open-label, multicenter study in 132 women with measurable HR-positive, HER2-negative metastatic breast cancer. Patients received 200 mg VERZENIO orally twice daily until development of progressive disease or unmanageable toxicity. Median duration of treatment was 4.5 months. (…)<br><br>**14 CLINICAL STUDIES**<br>**14.1 Early Breast Cancer**<br>VERZENIO in Combination with Standard Endocrine Therapy (monarchE)<br>Patients with HR-positive, HER2-negative, node-positive early breast cancer at high risk of recurrence monarchE (NCT03155997) was a randomized (1:1), open-label, two cohort, multicenter study in adult women and men with HR-positive, HER2-negative, node-positive, resected, early breast cancer with clinical and pathological features consistent with a high risk of disease recurrence. To be enrolled in cohort 1, patients had to have HR positive, HER2 negative early breast cancer with tumor involvement in at least 1 axillary lymph node (pALN) and either:

* Therapeutic areas do not necessarily reflect the CDER review division.
† Representative biomarkers are listed based on standard nomenclature as per the Human Genome Organization (HUGO) symbol and/or simplified descriptors using other common conventions. Listed biomarkers do not necessarily reflect the terminology used in labeling. The term "Nonspecific" is provided when labeling does not explicitly identify the specific biomarker(s) or when the biomarker is represented by a molecular phenotype or gene signature, and in some cases the biomarker was inferred based on the labeling language.
‡ Referenced figures and tables may be found in the labeling available at Drugs@FDA.
Blue text represents the most recent addtions and/or changes since last posted version.

NDA/ANDA/BLA Number, Label Version Date	Drug	Therapeutic Area*	Biomarker†	Labeling Sections	Labeling Text‡
					• ≥4 pALN or<br>• 1-3 pALN and at least one of:<br>– tumor grade 3<br>– tumor size ≥ 50 mm.<br>Patients with available untreated breast tumor samples were tested retrospectively at central sites using the Ki-67 IHC MIB-1 pharmDx (Dako Omnis) assay to establish if the Ki-67 score was ≥20%, specified in the protocol as "Ki-67 high".<br>**14.2 Advanced or Metastatic Breast Cancer**<br>**VERZENIO in Combination with an Aromatase Inhibitor (Anastrozole or Letrozole) (MONARCH 3)**<br>*Postmenopausal women with HR-positive, HER2-negative advanced or metastatic breast cancer with no prior systemic therapy in this disease setting*<br>MONARCH 3 was a randomized (2:1), double-blinded, placebo-controlled, multicenter study in postmenopausal women with HR-positive, HER2-negative advanced or metastatic breast cancer in combination with a nonsteroidal aromatase inhibitor as initial endocrine-based therapy, including patients not previously treated with systemic therapy for breast cancer.<br>***VERZENIO in Combination with Fulvestrant (MONARCH 2)***<br>*Patients with HR-positive, HER2-negative advanced or metastatic breast cancer with disease progression on or after prior adjuvant or metastatic endocrine therapy*<br>MONARCH 2 (NCT02107703) was a randomized, placebo-controlled, multicenter study in women with HR-positive, HER2-negative metastatic breast cancer in combination with fulvestrant in patients with disease progression following endocrine therapy who had not received chemotherapy in the metastatic setting. (…)<br>***VERZENIO Administered as a Monotherapy in Metastatic Breast Cancer (MONARCH 1)***<br>*Patients with HR-positive, HER2-negative breast cancer who received prior endocrine therapy and 1-2 chemotherapy regimens in the metastatic setting*<br>MONARCH 1 (NCT02102490) was a single-arm, open-label, multicenter study in women with measurable HR-positive, HER2-negative metastatic breast cancer whose disease progressed during or after endocrine therapy, had received a taxane in any setting, and who received 1 or 2 prior chemotherapy regimens in the metastatic setting. (…)
**208716, 10/12/2021**	Abemaciclib (3)	Oncology	MKI67	Clinical Studies	**14 CLINICAL STUDIES**<br>**14.1 Early Breast Cancer**<br>VERZENIO in Combination with Standard Endocrine Therapy (monarchE)<br>*Patients with HR-positive, HER2-negative, node-positive early breast cancer at high risk of recurrence*<br>monarchE (NCT03155997) was a randomized (1:1), open-label, two cohort, multicenter study in adult women and men with HR-positive, HER2-negative, node-positive, resected, early breast cancer with clinical and pathological features consistent with a high risk of disease recurrence. To be enrolled, patients had to have HR-positive HER2-negative early breast cancer with tumor involvement in at least 1 axillary lymph node (pALN) and to be enrolled in cohort 1 had to have either:<br>• ≥4 pALN or<br>• 1-3 pALN and at least one of:<br>– tumor grade 3 or<br>– tumor size ≥50 mm<br>Patients enrolled in cohort 2 could not have met the eligibility criteria for cohort 1. To be enrolled in cohort 2, patients had to have 1-3 pALN and Ki-67 score ≥20%. Breast tumor samples were tested at central sites using the Ki-67 IHC MIB-1 pharmDx (Dako Omnis) assay to establish if the Ki-67 score was ≥20%. (…)
**213871, 01/14/2022**	Abrocitinib	Dermatology	CYP2C19	Dosage and Administration, Use in Specific Populations, Clinical Pharmacology	**2 DOSAGE AND ADMINISTRATION**<br>**2.4 Recommended Dosage in CYP2C19 Poor Metabolizers**<br>In patients who are known or suspected to be CYP2C19 poor metabolizers, the recommended dosage of CIBINQO is 50 mg once daily [see Use in Specific Populations (8.8) and Clinical Pharmacology (12.5)]. If an adequate response is not achieved with CIBINQO 50 mg orally daily after 12 weeks, consider increasing dosage to 100 mg orally once daily. Discontinue therapy if inadequate response is seen after dosage increase to 100 mg once daily.<br><br>**8 USE IN SPECIFIC POPULATIONS**<br>**8.8 CYP2C19 Poor Metabolizers**<br>In patients who are CYP2C19 poor metabolizers, the AUC of abrocitinib is increased compared to CYP2C19 normal metabolizers due to reduced metabolic clearance. Dosage reduction of CIBINQO is recommended in patients who are known or suspected to be CYP2C19 poor metabolizers based on genotype or previous history/experience with other CYP2C19 substrates [see Dosage and Administration (2.4) and Clinical Pharmacology (12.5)].<br><br>**12 CLINICAL PHARMACOLOGY**<br>**12.5 Pharmacogenomics**<br>Patients who are CYP2C19 poor metabolizers have little to no CYP2C19 enzyme function compared to CYP2C19 normal metabolizers that have fully functional CYP2C19 enzymes.<br>After single doses of abrocitinib, CYP2C19 poor metabolizers demonstrated dose-normalized AUC of abrocitinib values that were 2.3-fold higher when compared to CYP2C19 normal metabolizers. Approximately 3-5% of Caucasians and Blacks and 15 to 20% of Asians are CYP2C19 poor metabolizers [see Dosage and Administration (2.4) and Use in Specific Populations (8.8)].
**216340, 06/21/2024**	**Adagrasib**	Oncology	KRAS	Indications and Usage, Dosage and Administration,	**1 INDICATIONS AND USAGE**<br>**1.1. KRAS G12C-Mutated Locally Advanced or Metastatic Non-Small Cell Lung Cancer**<br>KRAZATI, as a single-agent, is indicated for the treatment of adult patients with KRAS G12C-mutated locally advanced or metastatic non-small cell lung cancer (NSCLC), as determined by an FDA-approved test [see Dosage and Administration (2.1)], who have received at least one prior systemic therapy.

* Therapeutic areas do not necessarily reflect the CDER review division.
† Representative biomarkers are listed based on standard nomenclature as per the Human Genome Organization (HUGO) symbol and/or simplified descriptors using other common conventions. Listed biomarkers do not necessarily reflect the terminology used in labeling. The term "Nonspecific" is provided when labeling does not explicitly identify the specific biomarker(s) or when the biomarker is represented by a molecular phenotype or gene signature, and in some cases the biomarker was inferred based on the labeling language.
‡ Referenced figures and tables may be found in the labeling available at Drugs@FDA.
Blue text represents the most recent addtions and/or changes since last posted version.

NDA/ANDA/BLA Number, Label Version Date	Drug	Therapeutic Area*	Biomarker†	Labeling Sections	Labeling Text‡
				Adverse Reactions, Clinical Pharmacology, Clinical Studies	This indication is approved under accelerated approval based on objective response rate (ORR) and duration of response (DOR) [see Clinical Studies (14.1)]. Continued approval for this indication may be contingent upon verification and description of a clinical benefit in a confirmatory trial.<br>**1.2. KRAS G12C-Mutated Locally Advanced or Metastatic Colorectal Cancer**<br>KRAZATI in combination with cetuximab is indicated for the treatment of adult patients with KRAS G12C-mutated locally advanced or metastatic colorectal cancer (CRC), as determined by an FDA-approved test [see Dosage and Administration (2.1)], who have received prior treatment with fluoropyrimidine-, oxaliplatin-, and irinotecan-based chemotherapy.<br>This indication is approved under accelerated approval based on ORR and DOR [see Clinical Studies (14.2)]. Continued approval for this indication may be contingent upon verification and description of a clinical benefit in a confirmatory trial.<br><br>**2 DOSAGE AND ADMINISTRATION**<br>**2.1 Patient Selection**<br>Non-Small Cell Lung Cancer<br>Select patients for treatment of locally advanced or metastatic NSCLC with KRAZATI based on the presence of KRAS G12C mutation in plasma or tumor specimens [see Clinical Studies (14.1)]. If no mutation is detected in a plasma specimen, test tumor tissue.<br>Colorectal Cancer<br>Select patients for treatment of locally advanced or metastatic CRC with KRAZATI based on the presence of KRAS G12C mutation in tumor specimens [see Clinical Studies (14.2)].<br>Information on FDA-approved tests for the detection of a KRAS G12C mutation is available at: https://www.fda.gov/CompanionDiagnostics<br><br>**6 ADVERSE REACTIONS**<br>Non-Small Cell Lung Cancer<br>The safety of adagrasib was evaluated in patients with KRAS G12C-mutated, locally advanced or metastatic NSCLC in KRYSTAL-1 [see Clinical Studies (14)]. Patients received adagrasib 600 mg orally twice daily (n = 116). Among patients who received adagrasib, 45% were exposed for 6 months or longer and 4% were exposed for greater than one year. (see Tables 3 and 4)<br>Colorectal Cancer<br>The safety of adagrasib combined with cetuximab was evaluated in 94 patients with KRAS G12C-mutated, locally advanced or metastatic CRC in KRYSTAL-1 [see Clinical Studies (14.2)]. Patients started treatment with adagrasib 600 mg twice daily in combination with cetuximab weekly (n = 17) or every two weeks (n = 77). Among patients who received adagrasib in combination with cetuximab, 60% were exposed for greater than 6 months and 12% were exposed for greater than 12 months.<br><br>**12 CLINICAL PHARMACOLOGY**<br>**12.3 Pharmacokinetics**<br>The pharmacokinetics of adagrasib were studied in healthy subjects and in patients with KRAS G12C-mutated NSCLC and are presented as mean (percent coefficient of variation) unless otherwise specified.<br>Adagrasib AUC and Cmax increase dose proportionally over the dose range of 400 mg to 600 mg (0.67 to 1 times the approved recommended dose). Adagrasib steady-state was reached within 8 days following administration of the approved recommended dosage and accumulation was approximately 6-fold.<br><br>**14 CLINICAL STUDIES**<br>**14.1 Non-Small Cell Lung Cancer**<br>The efficacy of adagrasib was evaluated in KRYSTAL-1 (NCT03785249), a multicenter, single-arm, open-label expansion cohort study. Eligible patients were required to have locally advanced or metastatic KRAS G12C-mutated NSCLC who previously received treatment with a platinum-based regimen and an immune checkpoint inhibitor, an Eastern Cooperative Oncology Group Performance Status (ECOG PS) of 0 or 1, and at least one measurable lesion as defined by Response Evaluation criteria in Solid Tumors (RECIST v1.1). Identification of a KRAS G12C mutation was prospectively determined by local testing using tissue specimens. Patients received adagrasib 600 mg orally twice daily until unacceptable toxicity or disease progression. Tumor assessments were performed every 6 weeks. The major efficacy outcome measures were confirmed objective response rate (ORR) and duration of response (DOR) as evaluated by blinded independent central review (BICR) according to RECIST v1.1.<br>In the efficacy population, KRAS G12C mutation status was determined by prospective local testing using tumor tissue specimens. Of the 112 patients with KRAS G12C mutation, tissue samples from 88% (98/112) patients were tested retrospectively using the QIAGEN therascreen KRAS RGQ PCR Kit. While 89% (87/98) of patients were positive for KRAS G12C mutation, 11% (11/98) did not have a KRAS G12C mutation identified. In addition, plasma samples from 63% (71/112) patients were tested retrospectively using Agilent Resolution ctDx FIRST assay. While 66% (47/71) of patients were positive for KRAS G12C mutation, 34% (24/71) did not have a KRAS G12C mutation identified. (…)<br>**14.2 Colorectal Cancer**<br>The efficacy of adagrasib in combination with cetuximab was evaluated in KRYSTAL-1, a multicenter, single-arm, open-label expansion cohort study. Eligible patients were required to have locally advanced or metastatic KRAS G12C-mutated CRC and to have previously received therapy with fluoropyrimidine-, oxaliplatin-, and irinotecan-based chemotherapy, a VEGF inhibitor if eligible, and an ECOG PS of 0 or 1.<br>Patients initiated treatment with adagrasib 600 mg orally twice daily in combination with cetuximab administered either biweekly (77 patients with 500 mg/m2 every two weeks) or weekly (17 patients with 400 mg/m2 initial dose followed by 250 mg/m2 weekly). Treatment continued until unacceptable toxicity or disease progression. Tumor assessments were performed every 6 weeks. Adagrasib discontinuation required cetuximab discontinuation, however patients could continue to receive adagrasib if cetuximab was discontinued [see Dosage and Administration (2.3)]. Six patients continued with adagrasib single agent therapy

NDA/ANDA/BLA Number, Label Version Date	Drug	Therapeutic Area*	Biomarker†	Labeling Sections	Labeling Text‡
					after discontinuing cetuximab. The length of time these 6 patients received adagrasib alone ranged from 43 days to 3 years. Patient treatment with adagrasib after disease progression continued if a patient was clinically stable and considered to be deriving clinical benefit by the investigator.<br>The major efficacy outcome measures were confirmed ORR and DOR according to RECIST v1.1 as assessed by BICR.<br>In the efficacy population, KRAS G12C mutation status was determined by prospective local testing using tumor tissue specimens. Of the 94 patients with KRAS G12C mutation, tissue samples from 79% (74/94) patients were tested retrospectively using the QIAGEN therascreen KRAS RGQ PCR Kit. Of the 74 tissue samples submitted, 81% (60/74) yielded a result with 93% (56/60) positive for KRAS G12C and 7% (4/60) without a KRAS G12C mutation identified.
125427,<br>**05/03/2019**	**Ado-Trastuzumab Emtansine**	Oncology	ERBB2<br>(HER2)	Indications and Usage, Dosage and Administration, Adverse Reactions, Clinical Pharmacology, Clinical Studies	**1 INDICATIONS AND USAGE**<br>**1.1 Metastatic Breast Cancer (MBC)**<br>KADCYLA®, as a single agent, is indicated for the treatment of patients with HER2-positive, metastatic breast cancer who previously received trastuzumab and a taxane, separately or in combination.<br>Patients should have either:<br>• Received prior therapy for metastatic disease, or<br>• Developed disease recurrence during or within six months of completing adjuvant therapy.<br>**1.2 Early Breast Cancer (EBC)**<br>KADCYLA, as a single agent, is indicated for the adjuvant treatment of patients with HER2- positive early breast cancer who have residual invasive disease after neoadjuvant taxane and trastuzumab -based treatment.<br>Select patients for therapy based on an FDA-approved companion diagnostic for KADCYLA [see Dosage and Administration (2.1)]<br><br>**2 DOSAGE AND ADMINISTRATION**<br>**2.1 Patient Selection**<br>Select patients based on HER2 protein overexpression or HER2 gene amplification in tumor specimens [see Indications and Usage (1), Clinical Studies (14)].<br>Assessment of HER2 protein overexpression and/or HER2 gene amplification should be performed using FDA-approved tests specific for breast cancers by laboratories with demonstrated proficiency. Information on the FDA-approved tests for the detection of HER2 protein overexpression and HER2 gene amplification is available at: http://www.fda.gov/CompanionDiagnostics.<br>Improper assay performance, including use of sub-optimally fixed tissue, failure to utilize specified reagents, deviation from specific assay instructions, and failure to include appropriate controls for assay validation, can lead to unreliable results.<br><br>**6 ADVERSE REACTIONS**<br>**6.1 Clinical Trials Experience**<br>Because clinical trials are conducted under widely varying conditions, adverse reaction rates observed in the clinical trials of a drug cannot be directly compared to rates in the clinical trials of another drug and may not reflect the rates observed in practice.<br>The data in the WARNINGS AND PRECAUTIONS reflect exposure to KADCYLA as a single agent at 3.6 mg/kg given as an intravenous infusion every 3 weeks (21-day cycle) in 1624 patients including 884 patients with HER2-positive metastatic breast cancer and 740 patients with HER2- positive early breast cancer (KATHERINE trial).<br>Metastatic Breast Cancer<br>In clinical trials, KADCYLA has been evaluated as single-agent in 884 patients with HER2- positive metastatic breast cancer. The most common (≥ 25%) adverse reactions were fatigue, nausea, musculoskeletal pain, hemorrhage, thrombocytopenia, headache, increased transaminases, constipation and epistaxis.<br>The adverse reactions described in Table 3 were identified in patients with HER2-positive metastatic breast cancer treated in the EMILIA trial [see Clinical Studies (14.1)]. Patients were randomized to receive KADCYLA or lapatinib plus capecitabine. The median duration of study treatment was 7.6 months for patients in the KADCYLA-treated group and 5.5 months and 5.3 months for patients treated with lapatinib and capecitabine, respectively. (…)<br>Early Breast Cancer<br>KADCYLA has been evaluated as a single-agent in 740 patients with HER2-positive early breast cancer.<br>The adverse reactions described in Table 5 were identified in patients with HER2-positive early breast cancer treated in the KATHERINE trial [see Clinical Studies (14.2)]. Patients were randomized to receive KADCYLA or trastuzumab. The median duration of study treatment was 10 months for patients in the KADCYLA-treated group and 10 months for patients treated with trastuzumab. (…)<br><br>**12 CLINICAL PHARMACOLOGY**<br>**12.2 Pharmacodynamics**<br>Cardiac Electrophysiology<br>The effect of multiple doses of KADCYLA (3.6 mg/kg every 3 weeks) on the QTc interval was evaluated in an open label, single arm study in 51 patients with HER2-positive metastatic breast cancer. No large changes in the mean QT interval (i.e., > 20 ms) were detected in the study.<br>**12.3 Pharmacokinetics**<br>*Effect of Hepatic Impairment*<br>The liver is a primary organ for eliminating DM1 and DM1-containing catabolites. The pharmacokinetics of ado-trastuzumab emtansine and DM1-containing catabolites were evaluated after the administration of 3.6 mg/kg of KADCYLA to metastatic HER2-positive breast cancer patients with normal hepatic function (n=10), mild (Child-Pugh A; n=10) and moderate (ChildPugh B; n=8) hepatic impairment. (…)<br><br>**14 CLINICAL STUDIES**<br>**14.1 Metastatic Breast Cancer**

* Therapeutic areas do not necessarily reflect the CDER review division.
† Representative biomarkers are listed based on standard nomenclature as per the Human Genome Organization (HUGO) symbol and/or simplified descriptors using other common conventions. Listed biomarkers do not necessarily reflect the terminology used in labeling. The term “Nonspecific” is provided when labeling does not explicitly identify the specific biomarker(s) or when the biomarker is represented by a molecular phenotype or gene signature, and in some cases the biomarker was inferred based on the labeling language.
‡ Referenced figures and tables may be found in the labeling available at Drugs@FDA.
Blue text represents the most recent addtions and/or changes since last posted version.

Table of Pharmacogenomic Biomarkers in Drug Labeling
Last Updated: 06/2024

NDA/ANDA/BLA Number, Label Version Date	Drug	Therapeutic Area*	Biomarker†	Labeling Sections	Labeling Text‡
					The efficacy of KADCYLA was evaluated in a randomized, multicenter, open-label trial (EMILIA) (NCT00829166) of 991 patients with HER2-positive, unresectable locally advanced or metastatic breast cancer. Prior taxane and trastuzumab-based therapy was required before trial enrollment. Patients with only prior adjuvant therapy were required to have disease recurrence during or within six months of completing adjuvant therapy. Breast tumor samples were required to show HER2 overexpression defined as 3+ IHC or FISH amplification ratio ≥ 2.0 determined at a central laboratory. (…)<br>**14.2 Early Breast Cancer**<br>KATHERINE (NCT01772472) was a randomized, multicenter, open-label trial of 1486 patients with HER2-positive, early breast cancer. Patients were required to have had neoadjuvant taxane and trastuzumab-based therapy with residual invasive tumor in the breast and/or axillary lymph nodes. Patients received radiotherapy and/or hormonal therapy concurrent with study treatment as per local guidelines. Breast tumor samples were required to show HER2 overexpression defined as 3+ IHC or ISH amplification ratio ≥ 2.0 determined at a central laboratory using Ventana's PATHWAY anti-HER2-/neu (4B5) Rabbit Monoclonal Primary Antibody or INFORM HER2 Dual ISH DNA Probe Cocktail assays. Patients were randomized (1:1) to receive KADCYLA or trastuzumab. Randomization was stratified by clinical stage at presentation, hormone receptor status, preoperative HER2-directed therapy (trastuzumab, trastuzumab plus additional HER2-directed agent[s]), and pathological nodal status evaluation after preoperative therapy.<br>KADCYLA was given intravenously at 3.6 mg/kg on Day 1 of a 21-day cycle. Trastuzumab was given intravenously at 6 mg/kg on Day 1 of a 21-day cycle. Patients were treated with KADCYLA or trastuzumab for a total of 14 cycles unless there was recurrence of disease, withdrawal of consent, or unacceptable toxicity. At the time of the major efficacy outcome analysis, median treatment duration was 10 months for both KADCYLA- and trastuzumab-treated patients. Patients who discontinued KADCYLA for reasons other than disease recurrence could complete the remainder of the planned HER2-directed therapy with trastuzumab if appropriate based on toxicity considerations and investigator discretion.<br>(…) The majority of patients (77%) had received an anthracycline-containing neoadjuvant chemotherapy regimen. Twenty percent of patients received another HER2-targeted agent in addition to trastuzumab as a component of neoadjuvant therapy; 94% of these patients received pertuzumab. (…)
**761178,** 08/30/2023	**Aducanumab-avwa**	Neurology	APOE	Boxed Warning, Warnings and Precautions, Clinical Studies, Patient Counseling Information	**BOXED WARNING**<br>**WARNING: AMYLOID RELATED IMAGING ABNORMALITIES**<br>Monoclonal antibodies directed against aggregated forms of beta amyloid, including ADUHELM, can cause amyloid related imaging abnormalities (ARIA), characterized as ARIA with edema (ARIA-E) and ARIA with hemosiderin deposition (ARIA-H). Incidence and timing of ARIA vary among treatments. ARIA usually occurs early in treatment and is usually asymptomatic, although serious and life-threatening events rarely can occur. Serious intracerebral hemorrhages, some of which have been fatal, have been observed in patients treated with this class of medications [see Warnings and Precautions (5.1), Adverse Reactions (6.1)].<br>ApoE ε4 Homozygotes<br>Patients who are apolipoprotein E ε4 (ApoE ε4) homozygotes (approximately 15% of Alzheimer's disease patients) treated with this class of medications, including ADUHELM, have a higher incidence of ARIA, including symptomatic, serious, and severe radiographic ARIA, compared to heterozygotes and noncarriers. Testing for ApoE ε4 status should be performed prior to initiation of treatment to inform the risk of developing ARIA. Prior to testing, prescribers should discuss with patients the risk of ARIA across genotypes and the implications of genetic testing results. Prescribers should inform patients that if genotype testing is not performed they can still be treated with ADUHELM; however, it cannot be determined if they are ApoE ε4 homozygotes and at higher risk for ARIA [see Warnings and Precautions (5.1)].<br>Consider the benefit of ADUHELM for the treatment of Alzheimer's disease and potential risk of serious adverse events associated with ARIA when deciding to initiate treatment with ADUHELM [see Warnings and Precautions (5.1) and Clinical Studies (14)].<br><br>**5 WARNINGS AND PRECAUTIONS**<br>**5.1 Amyloid Related Imaging Abnormalities**<br>ARIA usually occurs early in treatment and is usually asymptomatic, although serious and life-threatening events, including seizure and status epilepticus, rarely can occur. When present, reported symptoms associated with ARIA may include headache, confusion, visual changes, dizziness, nausea, and gait difficulty. Focal neurologic deficits may also occur. Symptoms associated with ARIA usually resolve over time. The risk of ARIA, including symptomatic and serious ARIA, is increased in apolipoprotein E ε4 (ApoE ε4) homozygotes. In addition to ARIA, intracerebral hemorrhages greater than 1 cm in diameter have occurred in patients treated with ADUHELM. (…)<br>ApoE ε4 Carrier Status and Risk of ARIA<br>Approximately 15% of Alzheimer's disease patients are ApoE ε4 homozygotes. In Studies 1 and 2, among patients with a known ApoE ε4 genotype, 17% (182/1103) of patients in the ADUHELM group were ApoE ε4 homozygotes, 51% (564/1103) were heterozygotes, and 32% (357/1103) were noncarriers. The incidence of symptomatic ARIA was higher in ApoE ε4 homozygotes (16%) than in heterozygotes (11%) and noncarriers (5%) among patients treated with ADUHELM. However, the incidence of serious adverse reactions with ARIA-E, including risk of death, persistent or significant disability or incapacity, hospitalization, or other medically important event that may require intervention to prevent serious outcomes, was similar for ApoE ε4 carriers and noncarriers (2% in homozygotes, 1% in heterozygotes, 2% in noncarriers). The recommendations on management of ARIA do not differ between ApoE ε4 carriers and noncarriers [see Dosage and Administration (2.3)]. Testing for ApoE ε4 status should be performed prior to initiation of treatment to inform the risk of developing ARIA. Prior to testing, prescribers should discuss with patients the risk of ARIA across genotypes and the implications of genetic testing results. Prescribers should inform patients that if genotype testing is not performed they can still be treated with ADUHELM; however, it cannot be determined if they are ApoE ε4 homozygotes and at a higher risk for ARIA. An FDA-authorized test for detection of ApoE ε4 alleles to identify patients at risk of ARIA if treated with ADUHELM is not currently available. Currently available tests used to identify ApoE ε4 alleles may vary in accurary and design.<br>The majority of ARIA-E radiographic events occurred early in treatment (within the first 8 doses), although ARIA can occur at any time and patients can have more than 1 episode. The maximum radiographic severity of ARIA-E in patients treated with ADUHELM was mild in 10% (115/1105) of patients, moderate in 20% (223/1105) of patients, and severe in 4% (49/1105) of patients. Resolution on MRI occurred in 68% of ARIA-E patients by 12 weeks, 91% by 20 weeks, and 98% overall after detection. The maximum radiographic severity of ARIA-H microhemorrhage in patients treated with ADUHELM was mild in 14% (154/1105) of patients, moderate in 3% (29/1105) of patients, and severe in 3% (29/1105) patients. The maximum radiographic severity of ARIA-H superficial siderosis in

* Therapeutic areas do not necessarily reflect the CDER review division.
† Representative biomarkers are listed based on standard nomenclature as per the Human Genome Organization (HUGO) symbol and/or simplified descriptors using other common conventions. Listed biomarkers do not necessarily reflect the terminology used in labeling. The term "Nonspecific" is provided when labeling does not explicitly identify the specific biomarker(s) or when the biomarker is represented by a molecular phenotype or gene signature, and in some cases the biomarker was inferred based on the labeling language.
‡ Referenced figures and tables may be found in the labeling available at Drugs@FDA.
Blue text represents the most recent addtions and/or changes since last posted version.

Table of Pharmacogenomic Biomarkers in Drug Labeling
Last Updated: 06/2024

NDA/ANDA/BLA Number, Label Version Date	Drug	Therapeutic Area*	Biomarker†	Labeling Sections	Labeling Text‡
					patients treated with ADUHELM was mild in 7% (79/1105) of patients, moderate in 4% (47/1105) of patients, and severe in 3% (36/1105) of patients. Among patients treated with ADUHELM, the incidence of severe radiographic ARIA-E was highest in ApoE ε4 homozygotes 11% (20/182), compared to heterozygotes 4% (21/564) or noncarriers 2% (8/357). Among patients treated with ADUHELM, the incidence of severe radiographic ARIA-H (microhemorrhage or superficial siderosis) was highest in ApoE ε4 homozygotes 20% (36/182), compared to heterozygotes 4% (21/564) or noncarriers 2% (6/357).<br>Other Risk Factors for Intracerebral Hemorrhage<br>Patients were excluded from enrollment in Studies 1 and 2 for findings on neuroimaging that indicated an increased risk for intracerebral hemorrhage. These included findings suggestive of cerebral amyloid angiopathy (prior intracerebral hemorrhage greater than 1 cm in diameter, more than 4 microhemorrhages, superficial siderosis, and history of diffuse white matter disease). Vasogenic edema could also be suggestive of cerebral amyloid angiopathy. These and other lesions (aneurysm, vascular malformation) could potentially increase the risk of intracerebral hemorrhage.<br>The presence of an ApoE ε4 allele is also associated with cerebral amyloid angiopathy which has an increased risk for intracerebral hemorrhage.<br><br>**14 CLINICAL STUDIES**<br>In Studies 1 and 2, patients were randomized to receive ADUHELM low dose (3 or 6 mg/kg for ApoE ε4 carriers and noncarriers, respectively), ADUHELM high dose (10 mg/kg), or placebo every 4 weeks for 18 months, followed by an optional, dose-blind, long-term extension period. Both studies included an initial titration period of up to 6 months to the maximum target dose. At the beginning of the study, ApoE ε4 carriers were initially titrated up to a maximum of 6 mg/kg in the high dose group, which was later adjusted to 10 mg/kg.<br><br>**17 PATIENT COUNSELING INFORMATION**<br>**Advise the patient and/or caregiver to read the FDA-approved patient labeling (Medication Guide).**<br>Amyloid Related Imaging Abnormalities<br>Inform patients that ADUHELM may cause Amyloid Related Imaging Abnormalities or "ARIA". ARIA most commonly presents as temporary swelling in areas of the brain that usually resolves over time. Some people may also have small spots of bleeding in or on the surface of the brain. Inform patients that most people with swelling in areas of the brain do not experience symptoms, however, some people may experience symptoms such as headache, confusion, dizziness, vision changes, nausea, aphasia, weakness, or seizure. Instruct patients to notify their healthcare provider if these symptoms occur. Inform patients that events of intracerebral hemorrhage greater than 1 cm in diameter have been reported infrequently in patients taking ADUHELM, and that the use of anticoagulant or thrombolytic medications while taking ADUHELM may increase the risk of bleeding in the brain. Notify patients that their healthcare provider will perform MRI scans to monitor for ARIA [see Warnings and Precautions (5.1)].<br>Inform patients that although ARIA can occur in any patient treated with ADUHELM, there is an increased risk in patients who are ApoE ε4 homozygotes, and that testing for ApoE ε4 status should be performed prior to initiation of treatment to inform the risk of developing ARIA. Prior to testing, discuss with patients the risk of ARIA across genotypes and the implications of genetic testing results. Inform patients that if testing is not performed, it cannot be determined if they are ApoE ε4 homozygotes and at a higher risk for ARIA.
201292, **10/11/2019**	Afatinib	Oncology	EGFR	Indications and Usage, Dosage and Administration, Adverse Reactions, Clinical Studies	**1 INDICATIONS AND USAGE**<br>**1.1 EGFR Mutation-Positive, Metastatic Non-Small Cell Lung Cancer**<br>GILOTRIF is indicated for the first-line treatment of patients with metastatic non-small cell lung cancer (NSCLC) whose tumors have non-resistant epidermal growth factor receptor (EGFR) mutations as detected by an FDA-approved test [see Clinical Pharmacology (12.1) and Clinical Studies (14.1)].<br>Limitation of Use: The safety and efficacy of GILOTRIF have not been established in patients whose tumors have resistant EGFR mutations [see Clinical Studies (14.1)].<br><br>**2 DOSAGE AND ADMINISTRATION**<br>**2.1 Patient Selection for EGFR Mutation-Positive Metastatic NSCLC**<br>2.1 Patient Selection for Non-Resistant EGFR Mutation-Positive Metastatic NSCLC Select patients for first-line treatment of metastatic NSCLC with GILOTRIF based on the presence of nonresistant EGFR mutations in tumor specimens [see Indications and Usage (1.1) and Clinical Studies (14.1)]. Information on FDA-approved tests for the detection of EGFR mutations in NSCLC is available at: http://www.fda.gov/CompanionDiagnostics.<br><br>**6 ADVERSE REACTIONS**<br>**6.1 Clinical Trials Experience**<br>The data described below reflect exposure to GILOTRIF as a single agent in LUX-Lung 3, a randomized, active-controlled trial conducted in patients with EGFR mutation-positive, metastatic NSCLC, and in LUX-Lung 8, a randomized, active controlled trial in patients with metastatic squamous NSCLC progressing after platinum-based chemotherapy.(…)<br>*EGFR Mutation-Positive, Metastatic NSCLC*<br>(…) The data in Tables 1 and 2 below reflect the exposure of 229 EGFR-tyrosine kinase inhibitor-naïve, GILOTRIF-treated patients with EGFR mutation-positive, metastatic, non-squamous NSCLC enrolled in a randomized, multicenter, open-label trial (LUX-Lung 3). (…)<br><br>**14 CLINICAL STUDIES**<br>**14.1 EGFR Mutation-Positive Non-Small Cell Lung Cancer**<br>The efficacy and safety of GILOTRIF in the first-line treatment of 345 patients with EGFR mutation-positive, metastatic [Stage IV and Stage IIIb with pleural and/or pericardial effusion as classified by the American Joint Commission on Cancer (AJCC, 6th edition)] non-small cell lung cancer (NSCLC) were established in a randomized, multicenter, open-label trial (LUX-Lung 3 [NCT00949650]). Patients were randomized (2:1) to receive GILOTRIF 40 mg orally once daily (n=230) or up to 6 cycles of pemetrexed/cisplatin (n=115). Randomization was stratified according to EGFR mutation status (exon 19 deletion vs exon 21 L858R vs other) and race (Asian vs non-Asian). The major efficacy outcome was progression-free survival (PFS) as assessed by an independent review committee

* Therapeutic areas do not necessarily reflect the CDER review division.
† Representative biomarkers are listed based on standard nomenclature as per the Human Genome Organization (HUGO) symbol and/or simplified descriptors using other common conventions. Listed biomarkers do not necessarily reflect the terminology used in labeling. The term "Nonspecific" is provided when labeling does not explicitly identify the specific biomarker(s) or when the biomarker is represented by a molecular phenotype or gene signature, and in some cases the biomarker was inferred based on the labeling language.
‡ Referenced figures and tables may be found in the labeling available at Drugs@FDA.
Blue text represents the most recent addtions and/or changes since last posted version.

NDA/ANDA/BLA Number, Label Version Date	Drug	Therapeutic Area*	Biomarker†	Labeling Sections	Labeling Text‡
					(IRC). Other efficacy outcomes included overall response rate (ORR) and overall survival (OS). EGFR mutation status was prospectively determined for screening and enrollment of patients by a clinical trial assay (CTA). Tumor samples from 264 patients (178 randomized to GILOTRIF and 86 patients randomized to chemotherapy) were tested retrospectively by the companion diagnostic therascreen® EGFR RGQ PCR Kit, which is FDA-approved for selection of patients for GILOTRIF treatment.<br>Among the patients randomized, 65% were female, median age was 61 years, baseline ECOG performance status was 0 (39%) or 1 (61%), 26% were Caucasian and 72% were Asian. The majority of the patients had a tumor sample with an EGFR mutation categorized by the CTA as either exon 19 deletion (49%) or exon 21 L858R substitution (40%), while the remaining 11% had other mutations.<br>Pre-specified exploratory subgroup analyses were conducted according to the stratification factor of EGFR mutation category. See Figure 2 and text below Figure 2.<br>*Overall Response Rate In Other EGFR Mutations*<br>The efficacy of GILOTRIF in patients with NSCLC harboring non-resistant EGFR mutations (S768I, L861Q, and G719X) other than exon 19 deletions or exon 21 L858R substitutions was evaluated in a pooled analysis of such patients enrolled in one of three clinical trials (LUX-Lung 2 [NCT00525148], LUX-Lung 3 [NCT00949650], and LUX-Lung 6 [NCT01121393]). • LUX-Lung 2 was a single arm, multicenter study of afatinib 40 or 50 mg orally once daily until disease progression or intolerable side effects. EGFR status was determined by bi-directional Sanger sequencing of tumor tissue.<br>• LUX-Lung 3 was a randomized, multicenter study comparing treatment with afatinib 40 mg orally once daily to intravenous cisplatin 75 mg/m2 plus pemetrexed 500 mg/m2 every 21 days for up to 6 cycles. EGFR status was determined by the therascreen® EGFR RGQ PCR Kit.<br>• LUX-Lung 6 was a randomized, multicenter study comparing treatment with afatinib 40 mg to intravenous gemcitabine 1000 mg/m2 on day 1 and day 8 plus cisplatin 75 mg/m2 on day 1 of a 3-week schedule for up to 6 cycles. EGFR status was determined by the therascreen® EGFR RGQ PCR Kit.<br>Among the 75 GILOTRIF treated patients with uncommon EGFR mutations, 32 patients had a non-resistant EGFR mutation. Among the 32 patients with a confirmed non-resistant EGFR mutation, the median age was 60.5 years (range 32-79), 66% were female, 97% were Asian, 3% were other races, 38% had an ECOG PS of 0, 63% had an ECOG PS 1, 66% were never smokers, 28% were former smokers, and 6% were current smokers. Baseline disease characteristics were 97% Stage IV disease, 3% Stage IIIb disease, and 88% had received no prior systemic therapy for advanced or metastatic disease.<br>The number of patients, the number of responders, and durations of response in subgroups defined by identified mutation(s) are summarized in Table 6.
**208434, 04/18/2024**	**Alectinib**	Oncology	ALK	Indications and Usage, Dosage and Administration, Adverse Reactions, Clinical Pharmacology, Clinical Studies	**1 INDICATIONS AND USAGE**<br>**1.1 Adjuvant Treatment of Resected ALK-Positive Non-Small Cell Lung Cancer (NSCLC)**<br>ALECENSA is indicated as adjuvant treatment in adult patients following tumor resection of anaplastic lymphoma kinase (ALK)-positive non-small cell lung cancer (NSCLC) (tumors ≥ 4 cm or node positive), as detected by an FDA-approved test [see Dosage & Administration (2.1)].<br>**1.2 Treatment of Metastatic ALK-Positive NSCLC**<br>ALECENSA is indicated for the treatment of adult patients with ALK-positive metastatic NSCLC as detected by an FDA-approved test [see Dosage & Administration (2.1)].<br><br>**2 DOSAGE AND ADMINISTRATION**<br>**2.1 Patient Selection**<br>Select patients with resectable tumors for the adjuvant treatment of NSCLC with ALECENSA based on the presence of ALK positivity in tumor tissue [see Indications and Usage (1.1) and Clinical Studies (14.1)].<br>Select patients for the treatment of metastatic NSCLC with ALECENSA based on the presence of ALK positivity in tumor tissue or plasma specimens [see Indications and Usage (1.2) and Clinical Studies (14.2)]. If ALK rearrangements are not detected in a plasma specimen, test tumor tissue if feasible.<br>Information on FDA-approved tests for the detection of ALK rearrangements in NSCLC is available at http://www.fda.gov/CompanionDiagnostics.<br><br>**6 ADVERSE REACTIONS**<br>**6.1 Clinical Trials Experience**<br>Adjuvant Treatment of Resected ALK-Positive NSCLC<br>The safety of ALECENSA was evaluated in ALINA, a multi-center, open-label, randomized trial for the adjuvant treatment of patients with resected ALK-positive NSCLC [see Clinical Studies (14.1)]. At the time of DFS analysis, the median duration of exposure was 23.9 months for ALECENSA and 2.1 months for platinum-based chemotherapy.. (…)<br>Previously Untreated Metastatic ALK-Positive NSCLC<br>The safety of ALECENSA was evaluated in 152 patients with ALK-positive NSCLC in the ALEX study. The median duration of exposure to ALECENSA was 17.9 months. Patient characteristics of the ALEX study population (n=303) were: median age 56 years, age less than 65 (77%), female (56%), Caucasian (50%), Asian (46%), adenocarcinoma histology (92%), never smoker (63%), and ECOG PS 0 or 1 (93%).<br>Metastatic ALK-Positive NSCLC Previously Treated with Crizotinib<br>The safety of ALECENSA was evaluated in 253 patients with ALK-positive non-small cell lung cancer (NSCLC) treated with ALECENSA in two clinical trials, Studies NP28761 and NP28673. The median duration of exposure to ALECENSA was 9.3 months. One hundred sixty-nine patients (67%) were exposed to ALECENSA for more than 6 months, and 100 patients (40%) for more than one year. The population characteristics were: median age 53 years, age less than 65 (86%), female (55%), White (74%), Asian (18%), NSCLC adenocarcinoma histology (96%), never or former smoker (98%), ECOG Performance Status (PS) 0 or 1 (91%), and prior chemotherapy treatment (78%).<br><br>**12 CLINICAL PHARMACOLOGY**<br>**12.3 Pharmacokinetics**<br>The pharmacokinetics of alectinib and its major active metabolite M4 have been characterized in patients with ALK-positive NSCLC and healthy subjects.

* Therapeutic areas do not necessarily reflect the CDER review division.
† Representative biomarkers are listed based on standard nomenclature as per the Human Genome Organization (HUGO) symbol and/or simplified descriptors using other common conventions. Listed biomarkers do not necessarily reflect the terminology used in labeling. The term "Nonspecific" is provided when labeling does not explicitly identify the specific biomarker(s) or when the biomarker is represented by a molecular phenotype or gene signature, and in some cases the biomarker was inferred based on the labeling language.
‡ Referenced figures and tables may be found in the labeling available at Drugs@FDA.
Blue text represents the most recent addtions and/or changes since last posted version.

Table of Pharmacogenomic Biomarkers in Drug Labeling
Last Updated: 06/2024

NDA/ANDA/BLA Number, Label Version Date	Drug	Therapeutic Area*	Biomarker†	Labeling Sections	Labeling Text‡
					In patients with ALK-positive NSCLC, the geometric mean (coefficient of variation %) steady-state maximal concentration (Cmax,ss) for alectinib was 665 ng/mL (44%) and for M4 was 246 ng/mL (45%) with peak to trough concentration ratio of 1.2. (…)<br>*Absorption*<br>Alectinib reached maximal concentrations at 4 hours following administration of ALECENSA 600 mg twice daily under fed conditions in patients with ALK-positive NSCLC. (…)<br>*Distribution*<br>The apparent volume of distribution is 4,016 L for alectinib and 10,093 L for M4.<br>Alectinib and M4 are bound to human plasma proteins greater than 99%, independent of drug concentration.<br>Alectinib concentrations in the cerebrospinal fluid in patients with ALK-positive NSCLC approximate estimated alectinib free concentrations in the plasma. (…)<br>*Elimination*<br>The apparent clearance (CL/F) is 81.9 L/hour for alectinib and 217 L/hour for M4. The geometric mean elimination half-life is 33 hours for alectinib and 31 hours for M4 in patients with ALK-positive NSCLC.<br><br>**14 CLINICAL STUDIES**<br>**14.1 Adjuvant Treatment of Resected ALK-Positive NSCLC**<br>The efficacy of ALECENSA for the adjuvant treatment of patients with ALK-positive NSCLC following complete tumor resection was evaluated in a global, randomized open-label clinical trial (ALINA: NCT03456076). Eligible patients were required to have resectable ALK-positive NSCLC, Stage IB (tumors ≥ 4 cm) – IIIA per the Union for International Cancer Control/American Joint Committee on Cancer (UICC/AJCC) Staging System, 7th Edition. ALK rearrangements were identified by a locally performed FDA-approved ALK test or by a centrally performed VENTANA ALK (D5F3) CDx assay.<br>**14.2 Treatment of Metastatic ALK-Positive NSCLC**<br>*Previously Untreated Metastatic ALK-Positive NSCLC*<br>The efficacy of ALECENSA for the treatment of patients with ALK-positive NSCLC who had not received prior systemic therapy for metastatic disease was established in an open-label, randomized, active-controlled, multicenter study (ALEX: NCT02075840). Patients were required to have an ECOG performance status of 0-2 and ALK-positive NSCLC as identified by the VENTANA ALK (D5F3) CDx assay.<br>*Metastatic ALK-Positive NSCLC Previously Treated with Crizotinib*<br>The safety and efficacy of ALECENSA were established in two single-arm, multicenter clinical trials: NP28761 (NCT01588028) and NP28673 (NCT01801111). Patients with locally advanced or metastatic ALK-positive NSCLC, who have progressed on crizotinib, with documented ALK-positive NSCLC based on an FDA-approved test, and ECOG PS of 0-2 were enrolled in both studies.
125291, **02/18/2020**	**Alglucosidase Alfa**	Inborn Errors of Metabolism	GAA	Warnings and Precautions	**5 WARNINGS AND PRECAUTIONS**<br>**5.2 Immune-Mediated Reactions**<br>Immune-mediated cutaneous reactions have been reported with alglucosidase alfa including necrotizing skin lesions [see Adverse Reactions (6.3)]. Systemic immune-mediated reactions, including possible type III immune-mediated reactions have been observed with alglucosidase alfa. These reactions occurred several weeks to 3 years after initiation of alglucosidase alfa infusions. Skin biopsy in one patient demonstrated deposition of anti-rhGAA antibodies in the lesion. Another patient developed severe inflammatory arthropathy in association with pyrexia and elevated erythrocyte sedimentation rate. Nephrotic syndrome secondary to membranous glomerulonephritis was observed in some Pompe disease patients treated with alglucosidase alfa who had persistently positive anti-rhGAA IgG antibody titers. In these patients, renal biopsy was consistent with immune complex deposition. Patients improved following treatment interruption. Therefore, patients receiving alglucosidase alfa should undergo periodic urinalysis [see Adverse Reactions (6.3)]. (…)<br>**5.5 Risk of Antibody Development**<br>Patients with infantile-onset Pompe disease should have a cross-reactive immunologic material (CRIM) assessment early in their disease course and be managed by a clinical specialist knowledgeable in immune tolerance induction in Pompe disease to optimize treatment. Immune tolerance induction administered prior to and in conjunction with initiation of alglucosidase alfa has been reported to aide tolerability of alglucosidase alfa in CRIM-negative patients. CRIM status has been shown to be associated with immunogenicity and patients' responses to enzyme replacement therapies. CRIM-negative infants with infantile-onset Pompe disease treated with alglucosidase alfa have shown poorer clinical response in the presence of high sustained IgG antibody titers and positive inhibitory antibodies compared to CRIM-positive infants [see Adverse Reactions (6.2)]. In clinical studies, the majority of patients developed IgG antibodies to alglucosidase alfa, typically within 3 months of treatment. There is evidence to suggest that some patients who develop high and sustained IgG antibody titers, including CRIM-negative patients (i.e., patients in whom no endogenous GAA protein was detected by Western blot analysis and/or predicted based on the genotype), may experience reduced clinical alglucosidase alfa treatment efficacy, such as loss of motor function, ventilator dependence, or death.
12/16/2020, **020298**	**Allopurinol**	Oncology	HLA-B	Warnings	**WARNINGS**<br>WARNINGS: DISCONTINUE ALOPRIM AT THE FIRST APPEARANCE OF SKIN RASH OR OTHER SIGNS WHICH MAY INDICATE A HYPERSENSITIVITY REACTION. Serious and sometimes fatal dermatologic reactions, including toxic epidermal necrolysis (TEN), Stevens-Johnson syndrome (SJS), and drug reaction with eosinophilia and systemic symptoms (DRESS) have been reported in patients taking allopurinol. These reactions occur in approximately 5 in 10,000 (0.05%) patients taking allopurinol. Other serious hypersensitivity reactions that have been reported include exfoliative, urticarial and purpuric lesions; generalized vasculitis; and irreversible hepatotoxicity.<br>The HLA-B*58:01 allele is a genetic marker for severe skin reactions indicative of hypersensitivity to allopurinol. Patients who carry the HLA-B*58:01 allele are at a higher risk of allopurinol hypersensitivity syndrome (AHS), but hypersensitivity reactions have been reported in patients who do not carry this allele. The frequency of this allele is higher in individuals of African, Asian (e.g., Han Chinese, Korean, Thai), and Native Hawaiian/Pacific Islander ancestry. Prior to starting ALOPRIM, consider testing for the HLA-B*58:01 allele in genetically at-risk populations. The use of ALOPRIM is not recommended in HLA-B*58:01 positive patients unless the benefits clearly outweigh the risks.

* Therapeutic areas do not necessarily reflect the CDER review division.
† Representative biomarkers are listed based on standard nomenclature as per the Human Genome Organization (HUGO) symbol and/or simplified descriptors using other common conventions. Listed biomarkers do not necessarily reflect the terminology used in labeling. The term "Nonspecific" is provided when labeling does not explicitly identify the specific biomarker(s) or when the biomarker is represented by a molecular phenotype or gene signature, and in some cases the biomarker was inferred based on the labeling language.
‡ Referenced figures and tables may be found in the labeling available at Drugs@FDA.
Blue text represents the most recent addtions and/or changes since last posted version.

Table of Pharmacogenomic Biomarkers in Drug Labeling
Last Updated: 06/2024

NDA/ANDA/BLA Number, Label Version Date	Drug	Therapeutic Area*	Biomarker†	Labeling Sections	Labeling Text‡
212526, **05/04/2022**	Alpelisib (1)	Oncology	ERBB2 (HER2)	Indication and Usage, Dosage and Administration, Adverse Reactions, Clinical Studies	**1 INDICATIONS AND USAGE**<br>PIQRAY is indicated in combination with fulvestrant for the treatment of postmenopausal women, and men, with hormone receptor (HR)-positive, human epidermal growth factor receptor 2 (HER2)-negative, PIK3CAmutated, advanced or metastatic breast cancer as detected by an FDA-approved test following progression on or after an endocrine-based regimen.<br><br>**2 DOSAGE AND ADMINISTRATION**<br>**2.1 Patient Selection**<br>Select patients for the treatment of HR-positive, HER2-negative advanced or metastatic breast cancer with PIQRAY, based on the presence of one or more PIK3CA mutations in tumor tissue or plasma specimens [see Clinical Studies (14)]. If no mutation is detected in a plasma specimen, test tumor tissue. Information on FDAapproved tests for the detection of PIK3CA mutations in breast cancer is available at: http://www.fda.gov/CompanionDiagnostics.<br><br>**6 ADVERSE REACTIONS**<br>**6.1 Clinical Trial Experience**<br>Because clinical trials are conducted under widely varying conditions, adverse reaction rates observed in the clinical trials of a drug cannot be directly compared to rates in the clinical trials of another drug and may not reflect the rates observed in practice.<br>The safety of PIQRAY was evaluated in a randomized, double-blind, placebo-controlled trial (SOLAR-1) in 571 patients with HR-positive, HER2-negative, advanced or metastatic breast cancer enrolled into two cohorts, with or without a PIK3CA mutation [see Clinical Studies (14)]. (…)<br><br>**14 CLINICAL STUDIES**<br>SOLAR-1 (NCT02437318) was a randomized, double-blind, placebo-controlled trial of PIQRAY plus fulvestrant versus placebo plus fulvestrant in 572 patients with HR-positive, HER2-negative, advanced or metastatic breast cancer whose disease had progressed or recurred on or after an aromatase inhibitor-based treatment (with or without CDK4/6 combination). Patients were excluded if they had inflammatory breast cancer, diabetes mellitus Type 1 or uncontrolled Type 2, or pneumonitis. Randomization was stratified by presence of lung and/or liver metastasis and previous treatment with CDK4/6 inhibitor(s). Overall, 60% of enrolled patients had tumors with one or more PIK3CA mutations in tissue, 50% had liver/lung metastases, and 6% had previously been treated with a CDK4/6 inhibitor. (…)
212526, **05/04/2022**	Alpelisib (2)	Oncology	ESR (Hormone Receptor)	Indication and Usage, Dosage and Administration, Adverse Reactions, Clinical Studies	**1 INDICATIONS AND USAGE**<br>PIQRAY is indicated in combination with fulvestrant for the treatment of postmenopausal women, and men, with hormone receptor (HR)-positive, human epidermal growth factor receptor 2 (HER2)-negative, PIK3CAmutated, advanced or metastatic breast cancer as detected by an FDA-approved test following progression on or after an endocrine-based regimen.<br><br>**2 DOSAGE AND ADMINISTRATION**<br>**2.1 Patient Selection**<br>Select patients for the treatment of HR-positive, HER2-negative advanced or metastatic breast cancer with PIQRAY, based on the presence of one or more PIK3CA mutations in tumor tissue or plasma specimens [see Clinical Studies (14)]. If no mutation is detected in a plasma specimen, test tumor tissue. Information on FDAapproved tests for the detection of PIK3CA mutations in breast cancer is available at: http://www.fda.gov/CompanionDiagnostics.<br><br>**6 ADVERSE REACTIONS**<br>**6.1 Clinical Trial Experience**<br>Because clinical trials are conducted under widely varying conditions, adverse reaction rates observed in the clinical trials of a drug cannot be directly compared to rates in the clinical trials of another drug and may not reflect the rates observed in practice.<br>The safety of PIQRAY was evaluated in a randomized, double-blind, placebo-controlled trial (SOLAR-1) in 571 patients with HR-positive, HER2-negative, advanced or metastatic breast cancer enrolled into two cohorts, with or without a PIK3CA mutation [see Clinical Studies (14)]. (…)<br><br>**14 CLINICAL STUDIES**<br>SOLAR-1 (NCT02437318) was a randomized, double-blind, placebo-controlled trial of PIQRAY plus fulvestrant versus placebo plus fulvestrant in 572 patients with HR-positive, HER2-negative, advanced or metastatic breast cancer whose disease had progressed or recurred on or after an aromatase inhibitor-based treatment (with or without CDK4/6 combination). Patients were excluded if they had inflammatory breast cancer, diabetes mellitus Type 1 or uncontrolled Type 2, or pneumonitis. Randomization was stratified by presence of lung and/or liver metastasis and previous treatment with CDK4/6 inhibitor(s). Overall, 60% of enrolled patients had tumors with one or more PIK3CA mutations in tissue, 50% had liver/lung metastases, and 6% had previously been treated with a CDK4/6 inhibitor. (…)
212526, **05/04/2022**	Alpelisib (3)	Oncology	PIK3CA	Indication and Usage, Dosage and Administration, Adverse Reactions, Clinical Studies	**1 INDICATIONS AND USAGE**<br>PIQRAY is indicated in combination with fulvestrant for the treatment of postmenopausal women, and men, with hormone receptor (HR)-positive, human epidermal growth factor receptor 2 (HER2)-negative, PIK3CA-mutated, advanced or metastatic breast cancer as detected by an FDA-approved test following progression on or after an endocrine-based regimen.<br><br>**2 DOSAGE AND ADMINISTRATION**<br>**2.1 Patient Selection**<br>Select patients for the treatment of HR-positive, HER2-negative advanced or metastatic breast cancer with PIQRAY, based on the presence of one or more PIK3CA mutations in tumor tissue or plasma specimens [see Clinical Studies (14)]. If no mutation is detected in a plasma specimen, test tumor tissue. Information on FDAapproved tests for the detection of PIK3CA mutations in breast cancer is available at: http://www.fda.gov/CompanionDiagnostics.

* Therapeutic areas do not necessarily reflect the CDER review division.
† Representative biomarkers are listed based on standard nomenclature as per the Human Genome Organization (HUGO) symbol and/or simplified descriptors using other common conventions. Listed biomarkers do not necessarily reflect the terminology used in labeling. The term “Nonspecific” is provided when labeling does not explicitly identify the specific biomarker(s) or when the biomarker is represented by a molecular phenotype or gene signature, and in some cases the biomarker was inferred based on the labeling language.
‡ Referenced figures and tables may be found in the labeling available at Drugs@FDA.
Blue text represents the most recent addtions and/or changes since last posted version.

NDA/ANDA/BLA Number, Label Version Date	Drug	Therapeutic Area*	Biomarker†	Labeling Sections	Labeling Text‡
					**6 ADVERSE REACTIONS**<br>**6.1 Clinical Trial Experience**<br>Because clinical trials are conducted under widely varying conditions, adverse reaction rates observed in the clinical trials of a drug cannot be directly compared to rates in the clinical trials of another drug and may not reflect the rates observed in practice.<br><br>**14 CLINICAL STUDIES**<br>SOLAR-1 (NCT02437318) was a randomized, double-blind, placebo-controlled trial of PIQRAY plus fulvestrant versus placebo plus fulvestrant in 572 patients with HR-positive, HER2-negative, advanced or metastatic breast cancer whose disease had progressed or recurred on or after an aromatase inhibitor-based treatment (with or without CDK4/6 combination). Patients were excluded if they had inflammatory breast cancer, diabetes mellitus Type 1 or uncontrolled Type 2, or pneumonitis. Randomization was stratified by presence of lung and/or liver metastasis and previous treatment with CDK4/6 inhibitor(s). Overall, 60% of enrolled patients had tumors with one or more PIK3CA mutations in tissue, 50% had liver/lung metastases, and 6% had previously been treated with a CDK4/6 inhibitor.<br>There were 341 patients enrolled by tumor tissue in the cohort with a PIK3CA mutation and 231 enrolled in the cohort without a PIK3CA mutation. Of the 341 patients in the cohort with a PIK3CA mutation, 336 (99%) patients had one or more PIK3CA mutations confirmed in tumor tissue using the FDA-approved therascreen® PIK3CA RGQ PCR Kit. Out of the 336 patients with PIK3CA mutations confirmed in tumor tissue, 19 patients had no plasma specimen available for testing with the FDA-approved therascreen® PIK3CA RGQ PCR Kit. Of the remaining 317 patients with PIK3CA mutations confirmed in tumor tissue, 177 patients (56%) had PIK3CA mutations identified in plasma specimen, and 140 patients (44%) did not have PIK3CA mutations identified in plasma specimen. (…)<br>(…) Patient demographics for those with PIK3CA-mutated tumors were generally representative of the broader study population. The median duration of exposure to PIQRAY plus fulvestrant was 8.2 months with 59% of patients exposed for > 6 months. (…)<br>(…) The major efficacy outcome was investigator-assessed progression-free survival (PFS) in the cohort with a PIK3CA mutation per Response Evaluation Criteria in Solid Tumors (RECIST) v1.1. Additional efficacy outcome measures were overall response rate (ORR) and overall survival (OS) in the cohort with a PIK3CA mutation.<br>Efficacy results for the cohort with a PIK3CA mutation in tumor tissue are presented in Table 8 and Figure 1. PFS results for the cohort with a PIK3CA mutation by investigator assessment were supported by consistent results from a blinded independent review committee (BIRC) assessment. Consistent results were seen in patients with tissue or plasma PIK3CA mutations. At the time of final PFS analysis, 27% (92/341) of patients had died, and overall survival follow-up was immature.<br>No PFS benefit was observed in patients whose tumors did not have a PIK3CA tissue mutation (HR = 0.85; 95% CI: 0.58, 1.25). (See Table 8 and Figure 1)
208078, **11/28/2018**	**Amifampridine Phosphate**	Neurology	NAT2	Dosage and Administration, Use in Specific Populations, Clinical Pharmacology	**2 DOSAGE AND ADMINISTRATION**<br>**2.4 Known N-acetyltransferase 2 (NAT2) Poor Metabolizers**<br>The recommended starting dosage of FIRDAPSE in known N-acetyltransferase 2 (NAT2) poor metabolizers is 15 mg daily, taken orally in 3 divided doses [see Use in Specific Populations (8.8) and Clinical Pharmacology (12.3, 12.5)].<br><br>**8 USE IN SPECIFIC POPULATIONS**<br>**8.8 NAT2 Poor Metabolizers**<br>Exposure of FIRDAPSE is increased in patients who are N-acetyltransferase 2 (NAT2) poor metabolizers [see Clinical Pharmacology (12.5)]. Therefore, initiate FIRDAPSE in patients who are known NAT2 poor metabolizers at the lowest recommended starting dosage (15 mg/day) and monitor for adverse reactions [see Dosage and Administration (2.4)]. Consider dosage modification of FIRDAPSE for patients who are known NAT2 poor metabolizers as needed based on clinical effect and tolerability.<br><br>**12 CLINICAL PHARMACOLOGY**<br>**12.2 Pharmacodynamics**<br>The effect of FIRDAPSE on QTc interval prolongation was studied in a double blind, randomized, placebo and positive controlle d study in 52 healthy individuals who are slow acetylators. At an exposure 2-fold the expected maximum therapeutic exposure of amifampridine, FIRDAPSE did not prolong QTc to any clinically relevant extent.<br>**12.5 Pharmacogenomics**<br>Genetic variants in the N-acetyltransferase gene 2 (NAT2) affect the rate and extent of FIRDAPSE metabolism. Poor metabolizers, also referred to as “slow acetylators” (i.e., carriers of two reduced function alleles), have 3.5- to 4.5-fold higher Cmax, and 5.6- to 9- fold higher AUC than normal metabolizers, also referred to as “fast/rapid acetylators” (i.e., carriers of two normal function alleles). Therefore, FIRDAPSE should be initiated at the lowest recommended starting dosage (15 mg/day) in known NAT2 poor metabolizers, and such patients should be closely monitored for adverse reactions [see Dosage and Administration (2.4) and Use in Specific Populations (8.8)].<br>In the general population, the NAT2 poor metabolizer phenotype prevalence is 40–60% in the White and African American populations, and in 10–30% in Asian ethnic populations (individuals of Japanese, Chinese, or Korean descent).
**207356, 02/10/2023**	**Amikacin**	Infectious Diseases	MT-RNR1	Warnings and Precautions	**5 WARNINGS AND PRECAUTIONS**<br>**5.6 Ototoxicity**<br>Ototoxicity with use of ARIKAYCE<br>Ototoxicity has been reported with the use of ARIKAYCE in the clinical trials. Ototoxicity (including deafness, dizziness, presyncope, tinnitus, and vertigo) were reported with a higher frequency in patients treated with ARIKAYCE plus a background regimen (17%) compared to patients treated with background regimen

Table of Pharmacogenomic Biomarkers in Drug Labeling
Last Updated: 06/2024

NDA/ANDA/BLA Number, Label Version Date	Drug	Therapeutic Area*	Biomarker†	Labeling Sections	Labeling Text‡
					alone (9.8%). This was primarily driven by tinnitus (8.1% in ARIKAYCE plus background regimen vs. 0.9% in the background regimen alone arm) and dizziness (6.3% in ARIKAYCE plus background regimen vs. 2.7% in the background regimen alone arm) [see Adverse Reactions (6.1)].<br>Closely monitor patients with known or suspected auditory or vestibular dysfunction during treatment with ARIKAYCE. If ototoxicity occurs, manage the patient as medically appropriate, including potentially discontinuing ARIKAYCE.<br>Risk of Ototoxicity Due to Mitochondrial DNA Variants<br>Cases of ototoxicity with aminoglycosides have been observed in patients with certain variants in the mitochondrially encoded 12S rRNA gene (MT-RNR1), particularly the m.1555A>G variant. Ototoxicity occurred in some patients even when their aminoglycoside serum levels were within the recommended range. Mitochondrial DNA variants are present in less than 1% of the general US population, and the proportion of the variant carriers who may develop ototoxicity as well as the severity of ototoxicity is unknown. In case of known maternal history of ototoxicity due to aminoglycoside use or a known mitochondrial DNA variant in the patient, consider alternative treatments other than aminoglycosides unless the increased risk of permanent hearing loss is outweighed by the severity of infection and lack of safe and effective alternative therapies.
085971, **07/17/2014**	**Amitriptyline**	Psychiatry	CYP2D6	Precautions	**PRECAUTIONS**<br>**Drugs Metabolized by P450 2D6**<br>The biochemical activity of the drug metabolizing isozyme cytochrome P450 2D6 (debrisoquin hydroxylase) is reduced in a subset of the caucasian population (about 7 to 10% of Caucasians are so called “poor metabolizers”); reliable estimates of the prevalence of reduced P450 2D6 isozyme activity among Asian, African and other populations are not yet available. Poor metabolizers have higher than expected plasma concentrations of tricyclic antidepressants (TCAs) when given usual doses. Depending on the fraction of drug metabolized by P450 2D6, the increase in plasma concentration may be small, or quite large (8 fold increase in plasma AUC of the TCA).<br>In addition, certain drugs inhibit the activity of this isozyme and make normal metabolizers resemble poor metabolizers. (…)
**761210, 03/01/2024**	**Amivantamab-vmjw**	Oncology	EGFR	Indications and Usage, Dosage and Administration, Adverse Reactions, Clinical Studies	**1 INDICATIONS AND USAGE**<br>**1.1 First-Line Treatment of NSCLC with EGFRExon 20 Insertion Mutations**<br>RYBREVANT is indicated in combination with carboplatin and pemetrexed for the first-line treatment of adult patients with locally advanced or metastatic non-small cell lung cancer (NSCLC) with epidermal growth factor receptor (EGFR) exon 20 insertion mutations, as detected by an FDA-approved test [see Dosage and Administration (2.2)] .<br>**1.2 Previously Treated NSCLC with EGFRExon 20 Insertion Mutations**<br>RYBREVANT is indicated as a single agent for the treatment of adult patients with locally advanced or metastatic NSCLC with EGFR exon 20 insertion mutations, as detected by an FDA-approved test [see Dosage and Administration (2.2)] , whose disease has progressed on or after platinum-based chemotherapy.<br><br>**2 DOSAGE AND ADMINISTRATION**<br>**2.1 Patient Selection**<br>Select patients for treatment with RYBREVANT based on the presence of EGFR exon 20 insertion mutations in tumor or plasma specimens [see Clinical Studies (14)]. If no mutation is detected in a plasma specimen, test tumor tissue. Information on FDA-approved tests is available at: http://www.fda.gov/CompanionDiagnostics.<br><br>**6 ADVERSE REACTIONS**<br>**6.1 Clinical Trials Experience**<br>First-line Treatment of Non-Small Cell Lung Cancer (NSCLC) with Exon 20 Insertion Mutations<br>The data described below reflect exposure to RYBREVANT in combination with carboplatin and pemetrexed at the recommended dosage in the PAPILLON trial [see Clinical Studies (14.1)] in 151 patients with locally advanced or metastatic NSCLC with EGFR exon 20 insertion mutations. Among patients who received RYBREVANT in combination with carboplatin and pemetrexed the median exposure was 9.7 months (range: 0.0 to 26.9 months). In patients that received carboplatin and pemetrexed alone, the median exposure was 6.7 months (range 0.0 to 25.3). (…)<br>Previously Treated NSCLC Exon 20 Insertion Mutations<br>The data described below reflect exposure to RYBREVANT at the recommended dosage in 129 patients with locally advanced or metastatic NSCLC with EGFR exon 20 insertion mutations in the CHRYSALIS trial [see Clinical Studies (14.2)], whose disease had progressed on or after platinum-based chemotherapy. Among patients who received RYBREVANT, 44% were exposed for 6 months or longer and 12% were exposed for greater than one year.<br><br>**12 CLINICAL PHARMACOLOGY**<br>**12.2 Pharmacodynamics**<br>The exposure-response relationship and time-course of pharmacodynamic response of amivantamab-vmjw have not been fully characterized in patients with NSCLC with EGFR exon 20 insertion mutations.<br><br>**14 CLINICAL STUDIES**<br>**14.1 First Line Treatment of NSCLC with Exon 20 Insertion Mutations-PAPILLON**<br>The efficacy of RYBREVANT was evaluated in PAPILLON (NCT04538664), in a randomized, open-label, multicenter study. Eligible patients were required to have previously untreated locally advanced or metastatic NSCLC with EGFR Exon 20 insertion mutations measurable disease per RECIST v1.1, Eastern Cooperative Oncology Group (ECOG) performance status (PS) ≤1, and adequate organ and bone marrow function. Patients with brain metastases at screening were eligible for participation once they were definitively treated, clinically stable, asymptomatic, and off corticosteroid treatment for at least 2 weeks prior to randomization. Patients with a medical history of interstitial lung disease or active ILD were excluded from the clinical study.

* Therapeutic areas do not necessarily reflect the CDER review division.
† Representative biomarkers are listed based on standard nomenclature as per the Human Genome Organization (HUGO) symbol and/or simplified descriptors using other common conventions. Listed biomarkers do not necessarily reflect the terminology used in labeling. The term “Nonspecific” is provided when labeling does not explicitly identify the specific biomarker(s) or when the biomarker is represented by a molecular phenotype or gene signature, and in some cases the biomarker was inferred based on the labeling language.
‡ Referenced figures and tables may be found in the labeling available at Drugs@FDA.
Blue text represents the most recent addtions and/or changes since last posted version.

Table of Pharmacogenomic Biomarkers in Drug Labeling
Last Updated: 06/2024

NDA/ANDA/BLA Number, Label Version Date	Drug	Therapeutic Area*	Biomarker†	Labeling Sections	Labeling Text‡
					**14.2 Previously Treated NSCLC with Exon 20 Insertion Mutations-CHRYSALIS**<br>The efficacy of RYBREVANT was evaluated in patients with locally advanced or metastatic NSCLC with EGFR exon 20 insertion mutations in a multicenter, open-label, multi-cohort clinical trial (CHRYSALIS, NCT02609776). The study included patients with locally advanced or metastatic NSCLC with EGFR exon 20 insertion mutations whose disease had progressed on or after platinum-based chemotherapy. Patients with untreated brain metastases and patients with a history of ILD requiring treatment with prolonged steroids or other immunosuppressive agents within the last 2 years were not eligible for the study.<br>In the efficacy population, EGFR exon 20 insertion mutation status was determined by prospective local testing using tissue (94%) and/or plasma (6%) samples. Of the 81 patients with EGFR exon 20 insertion mutations identified by local testing, plasma samples from 78/81 (96%) patients were tested retrospectively using Guardant360 ®CDx, identifying 62/78 (79%) samples with an EGFR exon 20 insertion mutation; 16/78 (21%) samples did not have an EGFR exon 20 insertion mutation identified.<br>Patients received RYBREVANT at 1050 mg (for patient baseline body weight < 80 kg) or 1400 mg (for patient baseline body weight ≥80 kg) once weekly for 4 weeks, then every 2 weeks thereafter until disease progression or unacceptable toxicity. The major efficacy outcome measure was overall response rate (ORR) according to Response Evaluation Criteria in Solid Tumors (RECIST v1.1) as evaluated by Blinded Independent Central Review (BICR). An additional efficacy outcome measure was duration of response (DOR) by BICR.<br>The efficacy population included 81 patients with NSCLC with EGFR exon 20 insertion mutation with measurable disease who were previously treated with platinum-based chemotherapy. The median age was 62 (range: 42 to 84) years, 59% were female; 49% were Asian, 37% were White, 2.5% were Black; 74% had baseline body weight <80 kg; 95% had adenocarcinoma; and 46% had received prior immunotherapy. The median number of prior therapies was 2 (range: 1 to 7). At baseline, 67% had Eastern Cooperative Oncology Group (ECOG) performance status of 1; 53% never smoked; all patients had metastatic disease; and 22% had previously treated brain metastases.
072691, **07/17/2014**	**Amoxapine**	Psychiatry	CYP2D6	Precautions	**PRECAUTIONS**<br>**Drug Interactions**<br>**Drugs Metabolized by P450 2D6**<br>The biochemical activity of the drug metabolizing isozyme cytochrome P450 2D6 (debrisoquin hydroxylase) is reduced in a subset of the caucasian population (about 7 to 10% of caucasians are so called "poor metabolizers"); reliable estimates of the prevalence of reduced P450 2D6 isozyme activity among Asian, African and other populations are not yet available. Poor metabolizers have higher than expected plasma concentrations of tricyclic antidepressants (TCAs) when given usual doses. Depending on the fraction of drug metabolized by P450 2D6, the increase in plasma concentration may be small, or quite large (8 fold increase in plasma AUC of the TCA).<br>In addition, certain drugs inhibit the activity of this isozyme and make normal metabolizers resemble poor metabolizers. An individual who is stable on a given dose of TCA may become abruptly toxic when given one of these inhibiting drugs as concomitant therapy. (…)
204325, **09/15/2017**	**Amphetamine**	Psychiatry	CYP2D6	Clinical Pharmacology	**12 CLINICAL PHARMACOLOGY**<br>**12.3 Pharmacokinetics**<br>**Elimination**<br>**Metabolism and Excretion**<br>Amphetamine is reported to be oxidized at the 4 position of the benzene ring to form 4 hydroxyamphetamine, or on the side chain α or β carbons to form alpha-hydroxy-amphetamine or norephedrine, respectively. Norephedrine and 4-hydroxy-amphetamine are both active and each is subsequently oxidized to form 4-hydroxy-norephedrine. Alpha-hydroxy-amphetamine undergoes deamination to form phenylacetone, which ultimately forms benzoic acid and its glucuronide and the glycine conjugate hippuric acid. Although the enzymes involved in amphetamine metabolism have not been clearly defined, CYP2D6 is known to be involved with formation of 4-hydroxy-amphetamine. Since CYP2D6 is genetically polymorphic, population variations in amphetamine metabolism are a possibility.
**103950, 12/18/2020**	**Anakinra**	Rheumatology	NLRP3	Indications and Usage, Dosage and Administration, Warnings and Precautions, Adverse Reactions, Use in Specific Populations, Clinical Pharmacology, Clinical Studies	**1 INDICATIONS AND USAGE**<br>**1.2 Cryopyrin-Associated Periodic Syndromes (CAPS)**<br>KINERET is indicated for the treatment of Neonatal-Onset Multisystem Inflammatory Disease (NOMID).<br><br>**2 DOSAGE AND ADMINISTRATION**<br>**2.2 Cryopyrin-Associated Periodic Syndromes (CAPS)**<br>The recommended starting dose of KINERET is 1-2 mg/kg for NOMID patients. The dose can be individually adjusted to a maximum of 8 mg/kg daily to control active inflammation. Adjust doses in 0.5 to 1 mg/kg increments. Once daily administration is generally recommended, but the dose may be split into twice daily administrations. Each syringe is intended for a single use. A new syringe must be used for each dose. Any unused portion after each dose should be discarded.<br><br>**5 WARNINGS AND PRECAUTIONS**<br>**5.1 Serious Infections**<br>KINERET has been associated with an increased incidence of serious infections (2%) vs. Placebo (< 1%) in clinical trials in RA. Administration of KINERET in RA should be discontinued if a patient develops a serious infection. In KINERET treated NOMID and DIRA patients the risk of a disease flare when discontinuing KINERET treatment should be weighed against the potential risk of continued treatment. Treatment with KINERET should not be initiated in patients with active infections. The safety and efficacy of KINERET in immunosuppressed patients or in patients with chronic infections have not been evaluated. (…)<br>**5.6 Neutrophil Count**<br>Patients receiving KINERET may experience a decrease in neutrophil counts. Neutrophil counts should therefore be assessed prior to initiating KINERET treatment, and while receiving KINERET, monthly for 3 months, and thereafter quarterly for a period up to 1 year. In the placebo-controlled studies, 8% of RA patients receiving KINERET had decreases in neutrophil counts of at least one World Health Organization (WHO) toxicity grade compared with 2% in the placebo control group. Nine KINERET-treated patients (0.4%) experienced neutropenia (ANC < 1 x 109 /L). This is discussed in more detail in the Adverse

* Therapeutic areas do not necessarily reflect the CDER review division.
† Representative biomarkers are listed based on standard nomenclature as per the Human Genome Organization (HUGO) symbol and/or simplified descriptors using other common conventions. Listed biomarkers do not necessarily reflect the terminology used in labeling. The term "Nonspecific" is provided when labeling does not explicitly identify the specific biomarker(s) or when the biomarker is represented by a molecular phenotype or gene signature, and in some cases the biomarker was inferred based on the labeling language.
‡ Referenced figures and tables may be found in the labeling available at Drugs@FDA.
Blue text represents the most recent addtions and/or changes since last posted version.

NDA/ANDA/BLA Number, Label Version Date	Drug	Therapeutic Area*	Biomarker†	Labeling Sections	Labeling Text‡
					Reactions (6): Hematologic Events (6.1) section. In 43 NOMID patients followed for up to 60 months 2 patients experienced neutropenia that resolved over time during continued KINERET treatment. [see Adverse Reactions (6.2)].<br><br>**6 ADVERSE REACTIONS**<br>**6.2 Clinical Study Experience in NOMID**<br>The data described herein reflect an open-label study in 43 NOMID patients exposed to KINERET for up to 60 months adding up to a total exposure of 159.8 patient years. Patients were treated with a starting dose of 1 to 2 mg/kg/day and an average maintenance dose of 3-4 mg/kg/day adjusted depending on the severity of disease. Among pediatric NOMID patients, doses up to 7.6 mg/kg/day have been maintained for up to 15 months. (…)<br>Immunogenicity<br>The immunogenicity of KINERET in NOMID patients was not evaluated.<br><br>**8 USE IN SPECIFIC POPULATIONS**<br>**8.1 Pregnancy**<br>Risk Summary<br>Available data from retrospective studies and case reports on KINERET use in pregnant women are insufficient to identify a drug associated risk of major birth defects, miscarriage, or maternal and fetal adverse events. There are risks to the mother and fetus associated with active rheumatoid arthritis or Cryopyrin-Associated Periodic Syndromes (CAPS). In animal reproduction studies, subcutaneous administration of anakinra to pregnant rats and rabbits during organogenesis demonstrated no evidence of fetal harm at doses up to 25 times the maximum recommended human dose (MRHD). (…)<br>Clinical Considerations Disease-associated maternal and/or embryo/fetal risk Published data suggest the risk of adverse pregnancy outcomes in women with rheumatoid arthritis or CAPS is associated with increased disease activity. Adverse pregnancy outcomes include preterm delivery (before 37 weeks of gestation), low birth weight (<2500 grams), and small for gestational age at birth.<br>**8.4 Pediatric Use**<br>*Neonatal-Onset Multisystem Inflammatory Disease (NOMID)*<br>The NOMID study included 36 pediatric patients: 13 below 2 years, 18 between 2 and 11 years, and 5 between 12 and 17 years of age. A subcutaneous KINERET starting dose of 1–2 mg/kg/day was administered in all age groups. An average maintenance dose of 3–4 mg/kg/day was adequate to maintain clinical response throughout the study irrespective of age but a higher dose was, on occasion, required in severely affected patients. The prefilled syringe does not allow doses lower than 20 mg to be administered.<br><br>**12 CLINICAL PHARMACOLOGY**<br>**12.3 Pharmacokinetics**<br>(…) In NOMID patients, at a median SC dose of 3 mg/kg once daily and a median treatment time of 3.5 years, the median (range) steady-state serum exposure of anakinra was Cmax 3628 (655–8511) ng/mL (n=16) and C24h 203 (53–1979) ng/mL (n=16). The median (range) half-life of anakinra was 5.7 (3.1–28.2) hours (n=12). There was no obvious gender difference. (…)<br><br>**14 CLINICAL STUDIES**<br>**14.2 Clinical Studies in NOMID**<br>The efficacy of KINERET was evaluated in a prospective, long-term, open-label and uncontrolled study which incorporated a withdrawal period in a subset of 11 patients. This study included 43 NOMID patients 0.7 to 46 years of age treated for up to 60 months. Patients were given an initial KINERET dose of 1–2.4 mg/kg body weight. During the study, the dose was adjusted by 0.5 to 1 mg/kg increments to a protocol-specified maximum of 10 mg/kg daily, titrated to control signs and symptoms of disease. The maximum dose actually studied was 7.6 mg/kg/day. The average maintenance dose was 3 to 4 mg/kg daily. In general, the dose was given once daily, but for some patients, the dose was split into twice daily administrations for better control of disease activity.<br>NOMID symptoms were assessed with a disease-specific Diary Symptom Sum Score (DSSS), which included the prominent disease symptoms fever, rash, joint pain, vomiting, and headache. In addition, serum amyloid A (SAA), hsCRP, and ESR levels were monitored. Changes in clinical and laboratory parameters from baseline to Months 3 to 6 and from Month 3 (before withdrawal) to the end of the withdrawal period were assessed in the subset of patients who underwent withdrawal. The estimated changes from baseline in DSSS are summarized through Month 60 in Table 6. Results were consistent across all subgroups, including age, gender, presence of CIAS1 mutation, and disease phenotype. Improvements occurred in all individual disease symptoms comprising the DSSS (Table 7), as well as in the serum markers of inflammation. For the 11 patients who went through a withdrawal phase, disease symptoms and serum markers of inflammation worsened after withdrawal and promptly responded to reinstitution of KINERET therapy. Upon withdrawal of treatment, the median time until disease flare criteria were met was 5 days. (See Tables 6 and 7).<br>KINERET treatment also appeared to be associated with improvement of, or stability in, assessments of other NOMID disease manifestations, such as CNS, audiogram, and visual acuity data, up to Month 60.
020541,<br>**12/13/2018**	Anastrozole	Oncology	ESR, PGR<br>(Hormone Receptor)	Indications and Usage, Adverse Reactions, Drug Interactions, Clinical Studies	**1 INDICATIONS AND USAGE**<br>**1.1 Adjuvant Treatment**<br>ARIMIDEX is indicated for adjuvant treatment of postmenopausal women with hormone receptor-positive early breast cancer.<br>**1.2 First-Line Treatment**<br>ARIMIDEX is indicated for the first-line treatment of postmenopausal women with hormone receptor-positive or hormone receptor unknown locally advanced or metastatic breast cancer.<br>**1.3 Second-Line Treatment**<br>ARIMIDEX is indicated for the treatment of advanced breast cancer in postmenopausal women with disease progression following tamoxifen therapy. Patients with ER negative disease and patients who did not respond to previous tamoxifen therapy rarely responded to ARIMIDEX.

Table of Pharmacogenomic Biomarkers in Drug Labeling
Last Updated: 06/2024

NDA/ANDA/BLA Number, Label Version Date	Drug	Therapeutic Area*	Biomarker†	Labeling Sections	Labeling Text‡
					**6 ADVERSE REACTIONS**<br>**6.1 Clinical Trials Experience**<br>A post-marketing trial assessed the combined effects of ARIMIDEX and the bisphosphonate risedronate on changes from baseline in BMD and markers of bone resorption and formation in postmenopausal women with hormone receptor-positive early breast cancer. All patients received calcium and vitamin D supplementation. At 12 months, small reductions in lumbar spine bone mineral density were noted in patients not receiving bisphosphonates. Bisphosphonate treatment preserved bone density in most patients at risk of fracture. (…)<br><br>**7 DRUG INTERACTIONS**<br>**7.1 Tamoxifen**<br>Co-administration of anastrozole and tamoxifen in breast cancer patients reduced anastrozole plasma concentration by 27%. However, the co-administration of anastrozole and tamoxifen did not affect the pharmacokinetics of tamoxifen or N-desmethyltamoxifen. At a median follow-up of 33 months, the combination of ARIMIDEX and tamoxifen did not demonstrate any efficacy benefit when compared with tamoxifen in all patients as well as in the hormone receptor-positive subpopulation. This treatment arm was discontinued from the trial [see Clinical Studies (14.1)]. Based on clinical and pharmacokinetic results from the ATAC trial, tamoxifen should not be administered with anastrozole. (…)<br><br>**14 CLINICAL STUDIES**<br>**14.1 Adjuvant Treatment of Breast Cancer in Postmenopausal Women**<br>At a median follow-up of 33 months, the combination of ARIMIDEX and tamoxifen did not demonstrate any efficacy benefit when compared with tamoxifen in all patients as well as in the hormone receptor-positive subpopulation.<br>(…) Patients in the two monotherapy arms of the ATAC trial were treated for a median of 60 months (5 years) and followed for a median of 68 months. Disease-free survival in the intent-to-treat population was statistically significantly improved [Hazard Ratio (HR) = 0.87, 95% CI: 0.78, 0.97, p=0.0127] in the ARIMIDEX arm compared to the tamoxifen arm. In the hormone receptor-positive subpopulation representing about 84% of the trial patients, disease-free survival was also statistically significantly improved (HR = 0.83, 95% CI: 0.73, 0.94, p=0.0049) in the ARIMIDEX arm compared to the tamoxifen arm.<br>(See Figure 2) (…)<br>The frequency of individual events in the intent-to-treat population and the hormone receptor-positive subpopulation are described in Table 8. (see Tables 7 and 8)<br>A summary of the study efficacy results is provided in Table 9. (See Table 9, 10, and Figure 4) (…)<br>**14.2 First-Line Therapy in Postmenopausal Women with Advanced Breast Cancer**<br>Two double-blind, controlled clinical studies of similar design (0030, a North American study and 0027, a predominately European study) were conducted to assess the efficacy of ARIMIDEX compared with tamoxifen as first-line therapy for hormone receptor-positive or hormone receptor-unknown locally advanced or metastatic breast cancer in postmenopausal women. (See Table 11) (…)<br>**14.3 Second-Line Therapy in Postmenopausal Women with Advanced Breast Cancer who had Disease Progression following Tamoxifen Therapy**<br>Anastrozole was studied in two controlled clinical trials (0004, a North American study; 0005, a predominately European study) in postmenopausal women with advanced breast cancer who had disease progression following tamoxifen therapy for either advanced or early breast cancer. Some of the patients had also received previous cytotoxic treatment. Most patients were ER-positive; a smaller fraction were ER-unknown or ER negative; the ER-negative patients were eligible only if they had had a positive response to tamoxifen. (…)
**761123, 07/30/2021**	**Anifrolumab-fnia**	Rheumatology	Gene Signature (IFN)	Clinical Pharmacology, Clinical Studies	**12 CLINICAL PHARMACOLOGY**<br>**12.2 Pharmacodynamics**<br>In patients with SLE, following the administration of anifrolumab-fnia at 300 mg dose, via intravenous infusion every 4 weeks for 52 weeks, neutralization (≥80%) of a type I IFN gene signature was observed from Week 4 to Week 52 in blood samples of patients with elevated levels of type I IFN inducible genes and returned to baseline levels within 8 to 12 weeks following withdrawal of anifrolumab-fnia at the end of the 52-week treatment period. However, the clinical relevance of the type I IFN gene signature neutralization is unclear.<br>In SLE patients with positive anti-dsDNA antibodies at baseline (Trials 2 and 3), treatment with anifrolumab-fnia 300 mg led to numerical reductions in anti-dsDNA antibodies over time through Week 52.<br>In patients with low complement levels (C3 and C4), increases in complement levels were observed in patients receiving anifrolumab-fnia through Week 52.<br>Specific Populations<br>There was no clinically meaningful difference in systemic clearance based on age, race, ethnicity, region, gender, IFN status or body weight, that requires dose adjustment.<br><br>**14 CLINICAL STUDIES**<br>Randomization was stratified by disease severity (SLEDAI-2K score at baseline, <10 vs ≥10 points), OCS dose on Day 1 (<10 mg/day vs ≥10 mg/day prednisone or equivalent) and interferon gene signature test results (high vs low). (…)
021912, **05/29/2019**	**Arformoterol (1)**	Pulmonary	UGT1A1	Clinical Pharmacology	**12 CLINICAL PHARMACOLOGY**<br>**12.5 Pharmacogenomics**<br>Arformoterol is eliminated through the action of multiple drug metabolizing enzymes. Direct glucuronidation of arformoterol is mediated by several UGT enzymes and is the primary elimination route. O-Desmethylation is a secondary route catalyzed by the CYP enzymes CYP2D6 and CYP2C19. In otherwise healthy subjects with reduced CYP2D6 and/or UGT1A1 enzyme activity, there was no impact on systemic exposure to arformoterol compared to subjects with normal CYP2D6 and/or UGT1A1 enzyme activities.

* Therapeutic areas do not necessarily reflect the CDER review division.
† Representative biomarkers are listed based on standard nomenclature as per the Human Genome Organization (HUGO) symbol and/or simplified descriptors using other common conventions. Listed biomarkers do not necessarily reflect the terminology used in labeling. The term “Nonspecific” is provided when labeling does not explicitly identify the specific biomarker(s) or when the biomarker is represented by a molecular phenotype or gene signature, and in some cases the biomarker was inferred based on the labeling language.
‡ Referenced figures and tables may be found in the labeling available at Drugs@FDA.
Blue text represents the most recent addtions and/or changes since last posted version.

NDA/ANDA/BLA Number, Label Version Date	Drug	Therapeutic Area*	Biomarker†	Labeling Sections	Labeling Text‡
021912, **05/29/2019**	Arformoterol (2)	Pulmonary	CYP2D6	Clinical Pharmacology	**12 CLINICAL PHARMACOLOGY**<br>**12.5 Pharmacogenomics**<br>Arformoterol is eliminated through the action of multiple drug metabolizing enzymes. Direct glucuronidation of arformoterol is mediated by several UGT enzymes and is the primary elimination route. O-Desmethylation is a secondary route catalyzed by the CYP enzymes CYP2D6 and CYP2C19. In otherwise healthy subjects with reduced CYP2D6 and/or UGT1A1 enzyme activity, there was no impact on systemic exposure to arformoterol compared to subjects with normal CYP2D6 and/or UGT1A1 enzyme activities.
021436, **02/23/2017**	Aripiprazole	Psychiatry	CYP2D6	Dosage and Administration, Use in Specific Populations, Clinical Pharmacology	**2 DOSAGE AND ADMINISTRATION**<br>**2.7 Dosage Adjustments for Cytochrome P450 Considerations**<br>Dosage adjustments are recommended in patients who are known CYP2D6 poor metabolizers and in patients taking concomitant CYP3A4 inhibitors or CYP2D6 inhibitors or strong CYP3A4 inducers (see Table 2). When the coadministered drug is withdrawn from the combination therapy, ABILIFY dosage should then be adjusted to its original level. When the coadministered CYP3A4 inducer is withdrawn, ABILIFY dosage should be reduced to the original level over 1 to 2 weeks. Patients who may be receiving a combination of strong, moderate, and weak inhibitors of CYP3A4 and CYP2D6 (e.g., a strong CYP3A4 inhibitor and a moderate CYP2D6 inhibitor or a moderate CYP3A4 inhibitor with a moderate CYP2D6 inhibitor), the dosing may be reduced to one-quarter (25%) of the usual dose initially and then adjusted to achieve a favorable clinical response.<br>(See Table 2)<br><br>**8 USE IN SPECIFIC POPULATIONS**<br>**8.6 CYP2D6 Poor Metabolizers**<br>Dosage adjustment is recommended in known CYP2D6 poor metabolizers due to high aripiprazole concentrations. Approximately 8% of Caucasians and 3–8% of Black/African Americans cannot metabolize CYP2D6 substrates and are classified as poor metabolizers (PM) [see DOSAGE AND ADMINISTRATION (2.7) and CLINICAL PHARMACOLOGY (12.3)].<br><br>**12 CLINICAL PHARMACOLOGY**<br>**12.3 Pharmacokinetics**<br>(…) For CYP2D6 poor metabolizers, the mean elimination half-life for aripiprazole is about 146 hours.<br>*Drug Interaction Studies*<br>Effects of other drugs on the exposures of aripiprazole and dehydro-aripiprazole are summarized in Figure 1 and Figure 2, respectively. Based on simulation, a 4.5-fold increase in mean Cmax and AUC values at steady-state is expected when extensive metabolizers of CYP2D6 are administered with both strong CYP2D6 and CYP3A4 inhibitors. A 3-fold increase in mean Cmax and AUC values at steady-state is expected in poor metabolizers of CYP2D6 administered with strong CYP3A4 inhibitors. (…)<br>*Studies in Specific Populations*<br>Exposures of aripiprazole and dehydro-aripiprazole in specific populations are summarized in Figure 4 and Figure 5, respectively. In addition, in pediatric patients (10 to 17 years of age) administered with Abilify (20 mg to 30 mg), the body weight corrected aripiprazole clearance was similar to the adults. (See Figure 4 and 5)
207533, **11/30/2018**	Aripiprazole Lauroxil	Psychiatry	CYP2D6	Dosage and Administration, Use in Specific Populations, Clinical Pharmacology	**2 DOSAGE AND ADMINISTRATION**<br>**2.4 Dose Adjustments for CYP450 Considerations**<br>Refer to the prescribing information for oral aripiprazole for recommendations regarding dosage adjustments due to drug interactions, for the first 21 days when the patient is taking oral aripiprazole concomitantly with the first dose of ARISTADA.<br>Once stabilized on ARISTADA, refer to the dosing recommendations below for patients taking CYP 2D6 inhibitors, CYP 3A4 inhibitors, or CYP 3A4 inducers:<br>• No dosage changes recommended for ARISTADA, if CYP 450 modulators are added for less than 2 weeks.<br>• Make dose changes to ARISTADA if CYP 450 modulators are added for greater than 2 weeks. (See Table 4)<br><br>**8 USE IN SPECIFIC POPULATIONS**<br>**8.6 CYP2D6 Poor Metabolizers**<br>Dosage adjustment is recommended in known CYP 2D6 poor metabolizers due to high aripiprazole concentrations. Approximately 8% of Caucasians and 3-8% of Black/African Americans cannot metabolize CYP2D6 substrates and are classified as poor metabolizers (PM) [see Dosage and Administration (2.4), Clinical Pharmacology (12.3)].<br><br>**12 CLINICAL PHARMACOLOGY**<br>**12.3 Pharmacokinetics**<br>*Metabolism and Elimination*<br>(…) Elimination of aripiprazole is mainly through hepatic metabolism involving CYP 3A4 and CYP 2D6. Dosage adjustments are recommended in CYP 2D6 poor metabolizers due to high aripiprazole concentrations [see Dosage and Administration (2.4)]. (…)<br>*Drug Interaction Studies*<br>No specific drug interaction studies have been performed with ARISTADA. The drug interaction data provided below is obtained from studies with oral aripiprazole.<br>Effects of other drugs on the exposures of aripiprazole and dehydro-aripiprazole are summarized in Figure 1 and Figure 2, respectively. Based on simulation, a 4.5-fold increase in mean Cm ax and AUC values at steady-state is expected when extensive metabolizers of CYP2D6 are administered with both strong CYP 2D6 and CYP 3A4 inhibitors. After oral administration, a 3-fold increase in mean Cm ax and AUC values at steady-state is expected in poor metabolizers of CYP 2D6 administered with strong CYP 3A4 inhibitors. (See Figure 1, 2, and 3)

NDA/ANDA/BLA Number, Label Version Date	Drug	Therapeutic Area*	Biomarker†	Labeling Sections	Labeling Text‡
					Specific Population Studies<br>A population pharmacokinetic analysis showed no effect of sex, race or smoking on ARISTADA pharmacokinetics [see Use in Specific Populations (8.8)]. Exposures of aripiprazole and dehydro-aripiprazole using oral aripiprazole in specific populations are summarized in Figure 4 and Figure 5, respectively. (See Figure 4 and 5)
021248, **06/20/2019**	**Arsenic Trioxide**	Oncology	PML-RARA	Indications and Usage, Clinical Studies	**1 INDICATIONS AND USAGE**<br>**1.1. Newly-Diagnosed Low-Risk APL**<br>TRISENOX is indicated in combination with tretinoin for treatment of adults with newlydiagnosed low-risk acute promyelocytic leukemia (APL) whose APL is characterized by the presence of the t(15;17) translocation or PML/RAR-alpha gene expression.<br>**1.2. Relapsed or Refractory APL**<br>TRISENOX is indicated for induction of remission and consolidation in patients with APL who are refractory to, or have relapsed from, retinoid and anthracycline chemotherapy, and whose APL is characterized by the presence of the t(15;17) translocation or PML/RAR-alpha gene expression.<br><br>**14 CLINICAL STUDIES**<br>**14.1 Newly-Diagnosed Low-Risk APL**<br>(…) The trial enrolled 162 patients with a morphologic diagnosis of APL. The median age of patients was 45 years in the TRISENOX/tretinoin arm and 47 years in the chemotherapy/tretinoin arm, and 52% and 46% were male in the TRISENOX/tretinoin and chemotherapy/tretinoin arms, respectively. Baseline characteristics were balanced between treatment arms, including median WBC count, platelet count, PML-RARA isoform, and FLT3-ITD status. (…)
022466, **11/02/2018**	**Articaine and Epinephrine (1)**	Anesthesiology	G6PD	Warnings and Precautions	**5 WARNINGS AND PRECAUTIONS**<br>**5.4 Methemoglobinemia**<br>Cases of methemoglobinemia have been reported in association with local anesthetic use. Although all patients are at risk for methemoglobinemia, patients with glucose-6-phosphate dehydrogenase deficiency, congenital or idiopathic methemoglobinemia, cardiac or pulmonary compromise, infants under 6 months of age, and concurrent exposure to oxidizing agents or their metabolites are more susceptible to developing clinical manifestations of the condition. If local anesthetics must be used in these patients, close monitoring for symptoms and signs of methemoglobinemia is recommended. (…)
022466, **11/02/2018**	**Articaine and Epinephrine (2)**	Anesthesiology	Nonspecific (Congenital Methemoglobinemia)	Warnings and Precautions	**5 WARNINGS AND PRECAUTIONS**<br>**5.4 Methemoglobinemia**<br>Cases of methemoglobinemia have been reported in association with local anesthetic use. Although all patients are at risk for methemoglobinemia, patients with glucose-6-phosphate dehydrogenase deficiency, congenital or idiopathic methemoglobinemia, cardiac or pulmonary compromise, infants under 6 months of age, and concurrent exposure to oxidizing agents or their metabolites are more susceptible to developing clinical manifestations of the condition. If local anesthetics must be used in these patients, close monitoring for symptoms and signs of methemoglobinemia is recommended. (…)
761034, **01/21//2022**	**Atezolizumab (1)**	Oncology	CD274 (PD-L1)	Indications and Usage, Dosage and Administration, Adverse Reactions, Clinical Pharmacology, Clinical Studies	**1 INDICATIONS AND USAGE**<br>**1.1 Urothelial Carcinoma**<br>TECENTRIQ (atezolizumab) is indicated for the treatment of patients with locally advanced or metastatic urothelial carcinoma who:<br>• are not eligible for cisplatin-containing chemotherapy and whose tumors express PD-L1 (PD7 L1 stained tumor-infiltrating immune cells [IC] covering ≥ 5% of the tumor area), as determined by an FDA-approved test [see Dosage and Administration (2.1)], or<br>• are not eligible for any platinum-containing chemotherapy regardless of PD-L1 status, or<br>• have disease progression during or following any platinum-containing chemotherapy, or within 12 months of neoadjuvant or adjuvant chemotherapy.<br>**1.2 Non-Small Cell Lung Cancer**<br>• TECENTRIQ, as a single-agent, is indicated as adjuvant treatment following resection and platinum-based chemotherapy for adult patients with stage II to IIIA [see Clinical Studies (14.2)] non-small cell lung cancer (NSCLC) whose tumors have PD-L1 expression on ≥ 1% of tumor cells, as determined by an FDA-approved test [see Dosage and Administration (2.1)].<br>• TECENTRIQ, as a single agent, is indicated for the first-line treatment of adult patients with metastatic non-small cell lung cancer (NSCLC) whose tumors have high PD-L1 expression (PD-L1 stained ≥ 50% of tumor cells [TC ≥ 50%] or PD-L1 stained tumor-infiltrating immune cells [IC] covering ≥ 10% of the tumor area [IC ≥ 10%]), as determined by an FDAapproved test, with no EGFR or ALK genomic tumor aberrations [see Dosage and Administration (2.1)].<br><br>**2 DOSAGE AND ADMINISTRATION**<br>2.1 Patient Selection for Treatment of Urothelial Carcinoma, Non-Small Cell Lung Cancer and Melanoma<br>Select cisplatin-ineligible patients with previously untreated locally advanced or metastatic urothelial carcinoma for treatment with TECENTRIQ based on the PD-L1 expression on tumorinfiltrating immune cells [see Clinical Studies (14.1)].<br>Select patients with Stage II to IIIA non-small cell lung cancer for treatment with TECENTRIQ as a single agent based on PD-L1 expression on tumor cells [see Clinical Studies (14.2)]. Select patients with first-line metastatic non-small cell lung cancer for treatment with TECENTRIQ as a single agent based on the PD-L1 expression on tumor cells or on tumorinfiltrating immune cells [see Clinical Studies (14.2)].<br>Information on FDA-approved tests for the determination of PD-L1 expression in locally advanced or metastatic urothelial carcinoma or non-small cell lung cancer are available at: http://www.fda.gov/CompanionDiagnostics.<br><br>**6 ADVERSE REACTIONS**<br>**6.1 Clinical Trials Experience**<br>Non-small Cell Lung Cancer (NSCLC)<br>*IMpower110*

* Therapeutic areas do not necessarily reflect the CDER review division.
† Representative biomarkers are listed based on standard nomenclature as per the Human Genome Organization (HUGO) symbol and/or simplified descriptors using other common conventions. Listed biomarkers do not necessarily reflect the terminology used in labeling. The term "Nonspecific" is provided when labeling does not explicitly identify the specific biomarker(s) or when the biomarker is represented by a molecular phenotype or gene signature, and in some cases the biomarker was inferred based on the labeling language.
‡ Referenced figures and tables may be found in the labeling available at Drugs@FDA.
Blue text represents the most recent addtions and/or changes since last posted version.

NDA/ANDA/BLA Number, Label Version Date	Drug	Therapeutic Area*	Biomarker†	Labeling Sections	Labeling Text‡
					The safety of TECENTRIQ was evaluated in IMpower110, a multicenter, international, randomized, open-label study in 549 chemotherapy-naïve patients with stage IV NSCLC, including those with EGFR or ALK genomic tumor aberrations. Patients received TECENTRIQ 1200 mg every 3 weeks (n=286) or platinum-based chemotherapy consisting of carboplatin or cisplatin with either pemetrexed or gemcitabine (n=263) until disease progression or unacceptable toxicity [see Clinical Studies (14.2)]. IMpower110 enrolled patients whose tumors express PD-L1 (PD-L1 stained ≥ 1% of tumor cells [TC] or PD-L1 stained tumor-infiltrating immune cells [IC] covering ≥ 1% of the tumor area). The median duration of exposure to TECENTRIQ was 5.3 months (0 to 33 months). (…)<br><u>Previously Treated Metastatic NSCLC</u><br>(…) The safety of TECENTRIQ was evaluated in OAK, a multicenter, international, randomized, open-label trial in patients with metastatic NSCLC who progressed during or following a platinum-containing regimen, regardless of PD-L1 expression [see Clinical Studies (14.2)]. (…)<br>**6.2 Immunogenicity**<br>(…) Among 487 ADA-evaluable patients with NSCLC who received atezolizumab in IMpower010, 31% (n=152) tested positive for treatment-emergent ADA at one or more post-dose time points. Among 241 patients in the PD-L1 SP263 ≥1% TC Stage II-IIIA population, 28% (n=67) tested positive for treatment-emergent ADA at one or more post-dose time points. Patients who tested positive for treatment-emergent ADA had lower systemic atezolizumab exposure as compared to patients who were ADA-negative [see Clinical Pharmacology (12.3)]. There were insufficient numbers of patients and DFS events in the ADA-positive subgroup (19%; 39/207 by week 7) to determine whether ADA alters the efficacy of atezolizumab. The presence of ADA did not have a clinically significant effect on the incidence or severity of adverse reactions.<br><br>**12 CLINICAL PHARMACOLOGY**<br>**12.3 Pharmacokinetics**<br>*Specific Populations*<br>Age (21–89 years), body weight, gender, positive anti-therapeutic antibody (ATA) status, albumin levels, tumor burden, region or race, mild or moderate renal impairment (estimated glomerular filtration rate (eGFR) 30 to 89 mL/min/1.73 m), mild hepatic impairment (bilirubin ≤ ULN and AST > ULN or bilirubin < 1.0 to 1.5 × ULN and any AST), level of PD-L1 expression, or ECOG status had no clinically significant effect on the systemic exposure of atezolizumab. (…)<br><br>**14 CLINICAL STUDIES**<br>**14.1 Urothelial Carcinoma**<br>*Cisplatin-Ineligible Patients with Locally Advanced or Metastatic Urothelial Carcinoma*<br>(…) Tumor specimens were evaluated prospectively using the VENTANA PD-L1 (SP142) Assay at a central laboratory, and the results were used to define subgroups for pre-specified analyses. Of the 119 patients, 27% were classified as having PD-L1 expression of ≥ 5% (defined as PD-L1 stained tumor-infiltrating immune cells [IC] covering ≥ 5% of the tumor area). The remaining 73% of patients were classified as having PD-L1 expression of < 5% (PD-L1 stained tumor infiltrating IC covering < 5% of the tumor area).<br>Among the 32 patients with PD-L1 expression of ≥ 5%, median age was 67 years, 81% were male, 19% female, and 88% were White. Twenty-eight percent of patients had non-bladder urothelial carcinoma and 56% had visceral metastases. Seventy-two percent of patients had an ECOG PS of 0 or 1. Reasons for ineligibility for cisplatin-containing chemotherapy were: 66% had impaired renal function, 28% had an ECOG PS of 2, 16% had a hearing loss ≥ 25 dB, and 9% had Grades 2-4 peripheral neuropathy at baseline. Thirty-one percent of patients had disease progression following prior platinum-containing neoadjuvant or adjuvant chemotherapy.<br>Confirmed ORR in all patients and the two PD-L1 subgroups are summarized in Table 22. The median follow-up time for this study was 14.4 months. In 24 patients with disease progression following neoadjuvant or adjuvant therapy, the ORR was 33% (95% CI: 16%, 55%). (See Table 22) (…)<br>(…) Both cisplatin-eligible and cisplatin-ineligible patients are included in the study. Tumor specimens were evaluated prospectively using the VENTANA PD-L1 (SP142) Assay at a central laboratory. The independent Data Monitoring Committee (iDMC) for the study conducted a review of early data and found that patients classified as having PD-L1 expression of <5% when treated with TECENTRIQ monotherapy had decreased survival compared to those who received platinum-based chemotherapy. The iDMC recommended closure of the monotherapy arm to further accrual of patients with low PD-L1 expression, however, no other changes were recommended for the study, including any change of therapy for patients who had already been randomized to and were receiving treatment in the monotherapy arm.<br>*Previously Treated Patients with Locally Advanced or Metastatic Urothelial Carcinoma*<br>(…) Tumor specimens were evaluated prospectively using the VENTANA PD-L1 (SP142) Assay at a central laboratory and the results were used to define subgroups for pre-specified analyses. Of the 310 patients, 32% were classified as having PD-L1 expression of ≥ 5%. The remaining 68% of patients were classified as having PD-L1 expression of < 5%.<br>Confirmed ORR and median DOR in all patients and the two PD-L1 subgroups are summarized in Table 23. The median follow-up time for this study was 32.9 months. In 59 patients with disease progression following neoadjuvant or adjuvant therapy, the ORR was 22.0% (95% CI: 831 12.3%, 34.7%). (See Table 23) (…)<br>**14.2 Non-Small Cell Lung Cancer**<br>Adjuvant Treatment of Stage II-IIIA NSCLC with PD-L1 Expression ≥ 1%The median age was 62 years (range: 26 to 84), and 67% of patients were male. The majority of patients were White (73%) and Asian (24%). Most patients were current or previous smokers (78%) and baseline ECOG performance status in patients was 0 (55%) or 1 (44%). Overall, 12% of patients had Stage IB, 47% had Stage II and 41% had Stage IIIA disease. PD-L1 expression, defined as the percentage of tumor cells expressing PD-L1 as measured by the VENTANA PDL1 (SP263) assay, was ≥ 1% in 53% of patients, <1% in 44% and unknown in 2.6%.The primary efficacy outcome measure was disease-free survival (DFS) as assessed by the investigator. The primary efficacy analysis population (n = 476) was patients with Stage II – IIIA NSCLC with PD-L1 expression on ≥ 1% of tumor cells (PD-L1 ≥ 1% TC). DFS was defined as the time from the date of randomization to the date of occurrence of any of the following: first documented recurrence of disease, new primary NSCLC, or death due to any cause, whichever occurred first. A key secondary efficacy outcome measure was overall survival (OS) in the intentto-treat population. At the time of the interim DFS

NDA/ANDA/BLA Number, Label Version Date	Drug	Therapeutic Area*	Biomarker†	Labeling Sections	Labeling Text‡
					analysis, the study demonstrated a statistically significant improvement in DFS in the PD-L1 ≥ 1% TC, Stage II – IIIA patient population. Efficacy results are presented in Table 23 and Figure 1.<br>In a pre-specified secondary subgroup analysis of patients with PD-L1 TC ≥ 50% Stage II – IIIA NSCLC (n=229), the median DFS was not reached (95% CI: 42.3 months, NE) for patients in the TECENTRIQ arm and was 35.7 months (95% CI: 29.7, NE) for patients in the best supportive care arm, with a HR of 0.43 (95% CI: 0.27, 0.68). In an exploratory subgroup analysis of patients with PD-L1 TC 1-49% Stage II – IIIA NSCLC (n=247), the median DFS was 32.8 months (95% CI: 29.4, NE) for patients in the TECENTRIQ arm and 31.4 months (95% CI: 24.0, NE) for patients in the best supportive care arm, with a HR of 0.87 (95% CI: 0.60, 1.26). (See Figure 1)<br>At the time of the DFS interim analysis, 19% of patients in the PD-L1 ≥1% TC Stage II – IIIA patient population had died. An exploratory analysis of OS in this population resulted in a stratified HR of 0.77 (95% CI: 0.51, 1.17).<br>*Metastatic Chemotherapy-Naïve NSCLC with High PD-L1 Expression*<br>The efficacy of TECENTRIQ was evaluated in IMpower110 (NCT02409342), a multicenter, international, randomized, open-label trial in patients with stage IV NSCLC whose tumors express PD-L1 (PD-L1 stained ≥ 1% of tumor cells [TC ≥ 1%] or PD-L1 stained tumorinfiltrating immune cells [IC] covering ≥ 1% of the tumor area [IC ≥ 1%]), who had received no prior chemotherapy for metastatic disease. PD-L1 tumor status was determined based on immunohistochemistry (IHC) testing using the VENTANA PD-L1 (SP142) Assay. The evaluation of efficacy is based on the subgroup of patients with high PD-L1 expression (TC ≥ 50% or IC ≥ 10%), excluding those with EGFR or ALK genomic tumor aberrations. The trial excluded patients with a history of autoimmune disease, administration of a live attenuated vaccine within 28 days prior to randomization, active or untreated CNS metastases, administration of systemic immunostimulatory agents within 4 weeks or systemic immunosuppressive medications within 2 weeks prior to randomization.<br>Randomization was stratified by sex, ECOG performance status, histology (non-squamous vs. squamous) and PD-L1 expression (TC ≥ 1% and any IC vs. TC < 1% and IC ≥ 1%). Patients were randomized (1:1) to receive one of the following treatment arms:<br>• Arm A: TECENTRIQ 1200 mg every 3 weeks until disease progression or unacceptable toxicity<br>• Arm B: Platinum-based chemotherapy<br>Administration of TECENTRIQ was permitted beyond RECIST-defined disease progression. Tumor assessments were conducted every 6 weeks for the first 48 weeks following Cycle 1, Day 1 and then every 9 weeks thereafter. Tumor specimens were evaluated prospectively using the VENTANA PD-L1 (SP142) Assay at a central laboratory and the results were used to define subgroups for pre-specified analyses.<br>The major efficacy outcome measure was overall survival (OS) sequentially tested in the following subgroups of patients, excluding those with EGFR or ALK genomic tumor aberrations: TC ≥50% or IC ≥10%; TC ≥5% or IC ≥5%; and TC ≥1% or IC ≥1%.<br>Among the 205 chemotherapy-naïve patients with stage IV NSCLC with high PD-L1 expression (TC ≥ 50% or IC≥ 10%) excluding those with EGFR or ALK genomic tumor aberrations, the median age was 65.0 years (range: 33 to 87), and 70% of patients were male. The majority of patients were White (82%) and Asian (17%). Baseline ECOG performance status was 0 (36%) or 1 (64%); 88% were current or previous smokers; and 76% of patients had non-squamous disease while 24% of patients had squamous disease.<br>The trial demonstrated a statistically significant improvement in OS for patients with high PD-L1 expression (TC ≥50% or IC ≥10%) at the time of the OS interim analysis. There was no statistically significant difference in OS for the other two PD-L1 subgroups (TC ≥5% or IC ≥5%; and TC ≥1% or IC ≥1%) at the interim or final analyses. Efficacy results for patients with NSCLC with high PD-L1 expression are presented in Table 24 and Figure 1. (See Table 24 and Figure 2)<br>*Metastatic Chemotherapy-Naive Non-Squamous NSCLC*<br>IMpower150<br>The efficacy of TECENTRIQ with bevacizumab, paclitaxel, and carboplatin was evaluated in IMpower150 (NCT02366143), a multicenter, international, randomized (1:1:1), open-label trial in patients with metastatic non-squamous NSCLC. Patients with stage IV non-squamous NSCLC who had received no prior chemotherapy for metastatic disease but could have received prior EGFR or ALK kinase inhibitor if appropriate, regardless of PD-L1 or T-effector gene (tGE) status and ECOG performance status 0 or 1 were eligible. The trial excluded patients with a history of autoimmune disease, administration of a live attenuated vaccine within 28 days prior to randomization, active or untreated CNS metastases, administration of systemic immunostimulatory agents within 4 weeks or systemic immunosuppressive medications within 2 weeks prior to randomization, or clear tumor infiltration into the thoracic great vessels or clear cavitation of pulmonary lesions as seen on imaging. Randomization was stratified by sex, presence of liver metastases, and PD-L1 expression status on tumor cells (TC) and tumorinfiltrating immune cells (IC) as follows: TC3 and any IC vs. TC0/1/2 and IC2/3 vs. TC0/1/2 and IC0/1.<br>Tumor assessments were conducted every 6 weeks for the first 48 weeks following Cycle 1, Day 1 and then every 9 weeks thereafter. Tumor specimens were evaluated prior to randomization for PD-L1 tumor expression using the VENTANA PD-L1 (SP142) assay at a central laboratory. Tumor tissue was collected at baseline for expression of tGE signature and evaluation was performed using a clinical trial assay in a central laboratory prior to the analysis of efficacy outcome measures.<br>The major efficacy outcome measures for comparison of Arms B and C were progression free survival (PFS) by RECIST v1.1 in the tGE-WT (patients with high expression of T-effector gene signature [tGE], excluding those with EGFR- and ALK-positive NSCLC [WT]) and in the ITT WT subpopulations and overall survival (OS) in the ITT-WT subpopulation. Additional efficacy outcome measures for comparison of Arms B and C or Arms A and C were PFS and OS in the ITT population, OS in the tGE-WT subpopulation, and ORR/DoR in the tGE-WT and ITT-WT subpopulations.<br>A total of 1202 patients were enrolled across the three arms of whom 1045 were in the ITT-WT subpopulation and 447 were in the tGE-WT subpopulation. The demographic information is limited to the 800 patients enrolled in Arms B and C where efficacy has been demonstrated. The median age was 63 years (range: 31 to 90), and 60% of patients were male. The majority of patients were White (82%), 13% of patients were Asian, 10% were Hispanic, and 2% of patients were Black. Clinical sites in Asia (enrolling 13% of the study population) received paclitaxel at a dose of 175 mg/m2 while the remaining 87% received paclitaxel at a dose of 200 mg/m2 . Approximately 14% of patients had liver metastases at baseline, and most patients were current or previous smokers (80%). Baseline ECOG performance status was 0 (43%) or 1 (57%). PD-L1 was TC3 and any IC in 12%, TC0/1/2 and IC2/3 in 13%, and TC0/1/2 and IC0/1 in 75%. The demographics for the 696 patients in the ITT-WT subpopulation were similar to the ITT population except for the absence of patients with EGFR- or ALK-positive NSCLC.

* Therapeutic areas do not necessarily reflect the CDER review division.
† Representative biomarkers are listed based on standard nomenclature as per the Human Genome Organization (HUGO) symbol and/or simplified descriptors using other common conventions. Listed biomarkers do not necessarily reflect the terminology used in labeling. The term “Nonspecific” is provided when labeling does not explicitly identify the specific biomarker(s) or when the biomarker is represented by a molecular phenotype or gene signature, and in some cases the biomarker was inferred based on the labeling language.
‡ Referenced figures and tables may be found in the labeling available at Drugs@FDA.
Blue text represents the most recent addtions and/or changes since last posted version.

NDA/ANDA/BLA Number, Label Version Date	Drug	Therapeutic Area*	Biomarker†	Labeling Sections	Labeling Text‡
					The trial demonstrated a statistically significant improvement in PFS between Arms B and C in both the tGE-WT and ITT-WT subpopulations, but did not demonstrate a significant difference for either subpopulation between Arms A and C based on the final PFS analyses. In the interim analysis of OS, a statistically significant improvement was observed for Arm B compared to Arm C, but not for Arm A compared to Arm C. Efficacy results for the ITT-WT subpopulation are presented in Table 25 and Figure 3. (…)<br>*IMpower130*<br>The efficacy of TECENTRIQ with paclitaxel protein-bound and carboplatin was evaluated in IMpower130 (NCT02367781), a multicenter, randomized (2:1), open-label trial in patients with stage IV non-squamous NSCLC. Patients with Stage IV non-squamous NSCLC who had received no prior chemotherapy for metastatic disease, but could have received prior EGFR or ALK kinase inhibitor, if appropriate, were eligible. The trial excluded patients with history of autoimmune disease, administration of live attenuated vaccine within 28 days prior to randomization, administration of immunostimulatory agents within 4 weeks or systemic immunosuppressive medications within 2 weeks prior to randomization, and active or untreated CNS metastases. Randomization was stratified by sex, presence of liver metastases, and PD-L1 tumor expression according to the VENTANA PD-L1 (SP142) assay as follows: TC3 and any IC vs. TC0/1/2 and IC2/3 vs. TC0/1/2 and IC0/1. (…)<br>A total of 724 patients were enrolled; of these, 681 (94%) were in the ITT-WT population. The median age was 64 years (range: 18 to 86) and 59% were male. The majority of patients were white (90%), 2% of patients were Asian, 5% were Hispanic, and 4% were Black. Baseline ECOG performance status was 0 (41%) or 1 (58%). Most patients were current or previous smokers (90%). PD-L1 tumor expression was TC0/1/2 and IC0/1 in 73%; TC3 and any IC in 14%; and TC0/1/2 and IC2/3 in 13%. Efficacy results for the ITT-WT population are presented in Table 26 and Figure 3. (…)<br>*Previously Treated Metastatic NSCLC*<br>The efficacy of TECENTRIQ was evaluated in a multicenter, international, randomized (1:1), open-label study (OAK; NCT02008227) conducted in patients with locally advanced or metastatic NSCLC whose disease progressed during or following a platinum-containing regimen. Patients with a history of autoimmune disease, symptomatic or corticosteroid-dependent brain metastases, or requiring systemic immunosuppression within 2 weeks prior to enrollment were ineligible. Randomization was stratified by PD-L1 expression tumor-infiltrating immune cells (IC), the number of prior chemotherapy regimens (1 vs. 2), and histology (squamous vs. nonsquamous).<br>Patients were randomized to receive TECENTRIQ 1200 mg intravenously every 3 weeks until unacceptable toxicity, radiographic progression, or clinical progression or docetaxel 75 mg/m2 intravenously every 3 weeks until unacceptable toxicity or disease progression. Tumor assessments were conducted every 6 weeks for the first 36 weeks and every 9 weeks thereafter. The major efficacy outcome measure was overall survival (OS) in the first 850 randomized patients and OS in the subgroup of patients with PD-L1-expressing tumors (defined as ≥ 1% PDL1 expression on tumor cells [TC] or immune cells [IC]). Additional efficacy outcome measures were OS in all randomized patients (n = 1225), OS in subgroups based on PD-L1 expression, overall response rate (ORR), and progression free survival as assessed by the investigator per RECIST v.1.1.<br>Among the first 850 randomized patients, the median age was 64 years (33 to 85 years) and 47% were ≥ 65 years old; 61% were male; 70% were White and 21% were Asian; 15% were current smokers and 67% were former smokers; and 37% had baseline ECOG PS of 0 and 63% had a baseline ECOG PS of 1. Nearly all (94%) had metastatic disease, 74% had non-squamous histology, 75% had received only one prior platinum-based chemotherapy regimen, and 55% of patients had PD-L1-expressing tumors. (See Table 27 and Figure 5)<br>Tumor specimens were evaluated prospectively using the VENTANA PD-L1 (SP142) Assay at a central laboratory and the results were used to define the PD-L1 expression subgroups for prespecified analyses. Of the 850 patients, 16% were classified as having high PD-L1 expression, defined as having PD-L1 expression on ≥ 50% of TC or ≥ 10% of IC. In an exploratory efficacy subgroup analysis of OS based on PD-L1 expression, the hazard ratio was 0.41 (95% CI: 0.27, 0.64) in the high PD-L1 expression subgroup and 0.82 (95% CI: 0.68, 0.98) in patients who did not have high PD-L1 expression.
761034,<br>**01/21//2022**	Atezolizumab (2)	Oncology	Gene Signature (T-effector)	Clinical Studies	**14 CLINICAL STUDIES**<br>**14.2 Non-Small Cell Lung Cancer**<br>Metastatic Chemotherapy-Naive Non-Squamous NSCLC<br>*IMpower150*<br>The efficacy of TECENTRIQ with bevacizumab, paclitaxel, and carboplatin was evaluated in IMpower150 (NCT02366143), a multicenter, international, randomized (1:1:1), open-label trial in 1202 patients with metastatic non-squamous NSCLC. IMpower150 enrolled patients with stage IV non-squamous NSCLC who had received no prior chemotherapy for metastatic disease, but could have received prior EGFR or ALK kinase inhibitor if appropriate, regardless of PD-L1 or T-effector gene (tGE) status and ECOG performance status 0 or 1. (…)<br>(…) Tumor assessments were conducted every 6 weeks for the first 48 weeks following Cycle 1, Day 1 and then every 9 weeks thereafter. Tumor specimens were evaluated prior to randomization for PD-L1 tumor expression using the VENTANA PD-L1 (SP142) assay at a central laboratory. Tumor tissue was collected at baseline for expression of tGE signature and evaluation was performed using a clinical trial assay in a central laboratory prior to the analysis of efficacy outcome measures.<br>The major efficacy outcome measures for comparison of Arms B and C were progression free survival (PFS) by RECIST v1.1 in the tGE-WT (patients with high expression of T-effector gene signature [tGE], excluding those with EGFR- and ALK-positive NSCLC [WT]) and in the ITTWT subpopulations and overall survival (OS) in the ITT-WT subpopulation. Additional efficacy outcome measures for comparison of Arms B and C or Arms A and C were PFS and OS in the ITT population, OS in the tGE-WT subpopulation, and ORR/DoR in the tGE-WT and ITT-WT subpopulations.<br>A total of 1202 patients were enrolled across the three arms of whom 1045 were in the ITT-WT subpopulation and 447 were in the tGE-WT subpopulation. (…)<br>(…) The trial demonstrated a statistically significant improvement in PFS between Arms B and C in both the tGE-WT and ITT-WT subpopulations, but did not demonstrate a significant difference for either subpopulation between Arms A and C based on the final PFS analyses. In the interim analysis of OS, a statistically significant improvement was observed for Arm B compared to Arm C, but not for Arm A compared to Arm C. Efficacy results for the ITT-WT subpopulation are presented in Table 25 and Figure 3. (…)

* Therapeutic areas do not necessarily reflect the CDER review division.
† Representative biomarkers are listed based on standard nomenclature as per the Human Genome Organization (HUGO) symbol and/or simplified descriptors using other common conventions. Listed biomarkers do not necessarily reflect the terminology used in labeling. The term “Nonspecific” is provided when labeling does not explicitly identify the specific biomarker(s) or when the biomarker is represented by a molecular phenotype or gene signature, and in some cases the biomarker was inferred based on the labeling language.
‡ Referenced figures and tables may be found in the labeling available at Drugs@FDA.
Blue text represents the most recent addtions and/or changes since last posted version.

NDA/ANDA/BLA Number, Label Version Date	Drug	Therapeutic Area*	Biomarker†	Labeling Sections	Labeling Text‡
761034,<br>**01/21//2022**	Atezolizumab (3)	Oncology	EGFR	Indications and Usage, Adverse Reactions, Clinical Studies	**1 INDICATIONS AND USAGE**<br>**1.2 Non-Small Cell Lung Cancer**<br>• TECENTRIQ, in combination with bevacizumab, paclitaxel, and carboplatin, is indicated for the first-line treatment of adult patients with metastatic non-squamous NSCLC with no EGFR or ALK genomic tumor aberrations.<br>• TECENTRIQ, in combination with paclitaxel protein-bound and carboplatin, is indicated for the first-line treatment of adult patients with metastatic non-squamous NSCLC with no EGFR or ALK genomic tumor aberrations.<br>• TECENTRIQ, as a single-agent, is indicated for the treatment of adult patients with metastatic NSCLC who have disease progression during or following platinum-containing chemotherapy. Patients with EGFR or ALK genomic tumor aberrations should have disease progression on FDA-approved therapy for NSCLC harboring these aberrations prior to receiving TECENTRIQ.<br><br>**6 ADVERSE REACTIONS**<br>**6.1 Clinical Trials Experience**<br><u>Non-small Cell Lung Cancer (NSCLC)</u><br>*IMpower110*<br>The safety of TECENTRIQ was evaluated in IMpower110, a multicenter, international, randomized, open-label study in 549 chemotherapy-naïve patients with stage IV NSCLC, including those with EGFR or ALK genomic tumor aberrations.<br><br>**14 CLINICAL STUDIES**<br>**14.2 Non-Small Cell Lung Cancer**<br>*Metastatic Chemotherapy-Naïve NSCLC with High PD-L1 Expression*<br>The efficacy of TECENTRIQ was evaluated in IMpower110 (NCT02409342), a multicenter, international, randomized, open-label trial in patients with stage IV NSCLC whose tumors express PD-L1 (PD-L1 stained ≥ 1% of tumor cells [TC ≥ 1%] or PD-L1 stained tumorinfiltrating immune cells [IC] covering ≥ 1% of the tumor area [IC ≥ 1%]), who had received no prior chemotherapy for metastatic disease. PD-L1 tumor status was determined based on immunohistochemistry (IHC) testing using the VENTANA PD-L1 (SP142) Assay. The evaluation of efficacy is based on the subgroup of patients with high PD-L1 expression (TC ≥ 50% or IC ≥ 10%), excluding those with EGFR or ALK genomic tumor aberrations. The trial excluded patients with a history of autoimmune disease, administration of a live attenuated vaccine within 28 days prior to randomization, active or untreated CNS metastases, administration of systemic immunostimulatory agents within 4 weeks or systemic immunosuppressive medications within 2 weeks prior to randomization. (…)<br>The major efficacy outcome measure was overall survival (OS) sequentially tested in the following subgroups of patients, excluding those with EGFR or ALK genomic tumor aberrations: TC ≥50% or IC ≥10%; TC ≥5% or IC ≥5%; and TC ≥1% or IC ≥1%.<br>Among the 205 chemotherapy-naïve patients with stage IV NSCLC with high PD-L1 expression (TC ≥ 50% or IC≥ 10%) excluding those with EGFR or ALK genomic tumor aberrations, the median age was 65.0 years (range: 33 to 87), and 70% of patients were male. The majority of patients were White (82%) and Asian (17%). Baseline ECOG performance status was 0 (36%) or 1 (64%); 88% were current or previous smokers; and 76% of patients had non-squamous disease while 24% of patients had squamous disease. (See Table 24 and Figure 2) (…)<br><u>Metastatic Chemotherapy-Naive Non-Squamous NSCLC</u><br>*IMpower150*<br>**(…)** The major efficacy outcome measures for comparison of Arms B and C were progression free survival (PFS) by RECIST v1.1 in the tGE-WT (patients with high expression of T-effector gene signature [tGE], excluding those with EGFR- and ALK-positive NSCLC [WT]) and in the ITT- WT subpopulations and overall survival (OS) in the ITT-WT subpopulation. Additional efficacy outcome measures for comparison of Arms B and C or Arms A and C were PFS and OS in the ITT population, OS in the tGE-WT subpopulation, and ORR/DoR in the tGE-WT and ITT-WT subpopulations. (…)<br>**(…)** Baseline ECOG performance status was 0 (43%) or 1 (57%). PD-L1 was TC3 and any IC in 12%, TC0/1/2 and IC2/3 in 13%, and TC0/1/2 and IC0/1 in 75%. The demographics for the 696 patients in the ITT-WT subpopulation were similar to the ITT population except for the absence of patients with EGFR- or ALK-positive NSCLC. (…)<br>*IMpower130*<br>(…) Tumor assessments were conducted every 6 weeks for the first 48 weeks, then every 9 weeks thereafter. Major efficacy outcome measures were PFS by RECIST v1.1 and OS in the subpopulation of patients evaluated for and documented to have no EGFR or ALK genomic tumor aberrations (ITT-WT). (…)
761034,<br>**01/21//2022**	Atezolizumab (4)	Oncology	ALK	Indications and Usage, Adverse Reactions, Clinical Studies	**1 INDICATIONS AND USAGE**<br>**1.2 Non-Small Cell Lung Cancer**<br>• TECENTRIQ, in combination with bevacizumab, paclitaxel, and carboplatin, is indicated for the first-line treatment of adult patients with metastatic non-squamous NSCLC with no EGFR or ALK genomic tumor aberrations.<br>• TECENTRIQ, in combination with paclitaxel protein-bound and carboplatin, is indicated for the first-line treatment of adult patients with metastatic non-squamous NSCLC with no EGFR or ALK genomic tumor aberrations.<br>• TECENTRIQ, as a single-agent, is indicated for the treatment of adult patients with metastatic NSCLC who have disease progression during or following platinum-containing chemotherapy. Patients with EGFR or ALK genomic tumor aberrations should have disease progression on FDA-approved therapy for NSCLC harboring these aberrations prior to receiving TECENTRIQ.<br><br>**6 ADVERSE REACTIONS**<br>**6.1 Clinical Trials Experience**<br><u>Non-small Cell Lung Cancer (NSCLC)</u><br>*IMpower110*

NDA/ANDA/BLA Number, Label Version Date	Drug	Therapeutic Area*	Biomarker†	Labeling Sections	Labeling Text‡
					The safety of TECENTRIQ was evaluated in IMpower110, a multicenter, international, randomized, open-label study in 549 chemotherapy-naïve patients with stage IV NSCLC, including those with EGFR or ALK genomic tumor aberrations.<br><br>**14 CLINICAL STUDIES**<br>**14.2 Non-Small Cell Lung Cancer**<br>**14.2 Non-Small Cell Lung Cancer Metastatic Chemotherapy-Naïve NSCLC with High PD-L1 Expression**<br>The efficacy of TECENTRIQ was evaluated in IMpower110 (NCT02409342), a multicenter, international, randomized, open-label trial in patients with stage IV NSCLC whose tumors express PD-L1 (PD-L1 stained ≥ 1% of tumor cells [TC ≥ 1%] or PD-L1 stained tumorinfiltrating immune cells [IC] covering ≥ 1% of the tumor area [IC ≥ 1%]), who had received no prior chemotherapy for metastatic disease. PD-L1 tumor status was determined based on immunohistochemistry (IHC) testing using the VENTANA PD-L1 (SP142) Assay. The evaluation of efficacy is based on the subgroup of patients with high PD-L1 expression (TC ≥ 50% or IC ≥ 10%), excluding those with EGFR or ALK genomic tumor aberrations. The trial excluded patients with a history of autoimmune disease, administration of a live attenuated vaccine within 28 days prior to randomization, active or untreated CNS metastases, administration of systemic immunostimulatory agents within 4 weeks or systemic immunosuppressive medications within 2 weeks prior to randomization. (…)<br>The major efficacy outcome measure was overall survival (OS) sequentially tested in the following subgroups of patients, excluding those with EGFR or ALK genomic tumor aberrations: TC ≥50% or IC ≥10%; TC ≥5% or IC ≥5%; and TC ≥1% or IC ≥1%.<br>Among the 205 chemotherapy-naïve patients with stage IV NSCLC with high PD-L1 expression (TC ≥ 50% or IC≥ 10%) excluding those with EGFR or ALK genomic tumor aberrations, the median age was 65.0 years (range: 33 to 87), and 70% of patients were male. The majority of patients were White (82%) and Asian (17%). Baseline ECOG performance status was 0 (36%) or 1 (64%); 88% were current or previous smokers; and 76% of patients had non-squamous disease while 24% of patients had squamous disease. (See Table 24 and Figure 2) (…)<br><br>Metastatic Chemotherapy-Naive Non-Squamous NSCLC<br>*IMpower150*<br>**(…)** The major efficacy outcome measures for comparison of Arms B and C were progression free survival (PFS) by RECIST v1.1 in the tGE-WT (patients with high expression of T-effector gene signature [tGE], excluding those with EGFR- and ALK-positive NSCLC [WT]) and in the ITT- WT subpopulations and overall survival (OS) in the ITT-WT subpopulation. Additional efficacy outcome measures for comparison of Arms B and C or Arms A and C were PFS and OS in the ITT population, OS in the tGE-WT subpopulation, and ORR/DoR in the tGE-WT and ITT-WT subpopulations. (…)<br>**(…)** Baseline ECOG performance status was 0 (43%) or 1 (57%). PD-L1 was TC3 and any IC in 12%, TC0/1/2 and IC2/3 in 13%, and TC0/1/2 and IC0/1 in 75%. The demographics for the 696 patients in the ITT-WT subpopulation were similar to the ITT population except for the absence of patients with EGFR- or ALK-positive NSCLC. (…)<br>*IMpower130*<br>(…) Tumor assessments were conducted every 6 weeks for the first 48 weeks, then every 9 weeks thereafter. Major efficacy outcome measures were PFS by RECIST v1.1 and OS in the subpopulation of patients evaluated for and documented to have no EGFR or ALK genomic tumor aberrations (ITT-WT). (…)
761034, **01/21//2022**	Atezolizumab (5)	Oncology	BRAF	Indications and Usage, Dosage and Administration, Adverse Reactions, Clinical Studies	**1 INDICATIONS AND USAGE**<br>**1.5 Melanoma**<br>TECENTRIQ, in combination with cobimetinib and vemurafenib, is indicated for the treatment of patients with BRAF V600 mutation-positive unresectable or metastatic melanoma [see Dosage and Administration (2.1)].<br><br>**2 DOSAGE AND ADMINISTRATION**<br>**2.1 Patient Selection for Treatment of Urothelial Carcinoma, Triple-Negative Breast Cancer, or Non-Small Cell Lung Cancer and Melanoma**<br>Select patients with unresectable or metastatic melanoma for treatment with TECENTRIQ in combination with cobimetinib and vemurafenib after confirming the presence of a BRAF V600 mutation [see Clinical Studies (14.5)]. Information on FDA-approved tests for the detection of BRAF V600 mutations in melanoma is available at: http://www.fda.gov/CompanionDiagnostics.<br><br>**6 ADVERSE REACTIONS**<br>**6.1 Clinical Trials Experience**<br>Melanoma<br>The safety of TECENTRIQ, administered with cobimetinib and vemurafenib was evaluated in IMspire150, a double-blind, randomized (1:1), placebo-controlled study conducted in patients with previously untreated BRAF V600 mutation-positive metastatic or unresectable melanoma [see Clinical Studies (14.5)]. Patients received TECENTRIQ with cobimetinib and vemurafenib (N=230) or placebo with cobimetinib and vemurafenib (n=281). (…)<br><br>**14 CLINICAL STUDIES**<br>**14.5 Melanoma**<br>The efficacy of TECENTRIQ in combination with cobimetinib and vemurafenib was evaluated in a double-blind, randomized (1:1), placebo-controlled, multicenter trial (IMspire150; NCT02908672) conducted in 514 patients. Randomization was stratified by geographic location (North America vs. Europe vs. Australia, New Zealand, and others) and baseline lactate dehydrogenase (LDH) [less than or equal to upper limit of normal (ULN) vs. greater than ULN]. Eligible patients were required to have previously untreated unresectable or metastatic BRAF V600 mutation-positive melanoma as detected by a locally available test and centrally confirmed with the FoundationOneTM assay. (…)
021411, **05/19/2017**	Atomoxetine	Psychiatry	CYP2D6	Dosage and Administration,	**2 DOSAGE AND ADMINISTRATION**<br>**2.4 Dosing in Specific Populations**

* Therapeutic areas do not necessarily reflect the CDER review division.
† Representative biomarkers are listed based on standard nomenclature as per the Human Genome Organization (HUGO) symbol and/or simplified descriptors using other common conventions. Listed biomarkers do not necessarily reflect the terminology used in labeling. The term “Nonspecific” is provided when labeling does not explicitly identify the specific biomarker(s) or when the biomarker is represented by a molecular phenotype or gene signature, and in some cases the biomarker was inferred based on the labeling language.
‡ Referenced figures and tables may be found in the labeling available at Drugs@FDA.
Blue text represents the most recent addtions and/or changes since last posted version.

NDA/ANDA/BLA Number, Label Version Date	Drug	Therapeutic Area*	Biomarker†	Labeling Sections	Labeling Text‡
				Warnings and Precautions, Adverse Reactions, Drug Interactions, Use in Specific Populations, Clinical Pharmacology	Dosing adjustment for use with a strong CYP2D6 inhibitor or in patients who are known to be CYP2D6 PMs. In children and adolescents up to 70 kg body weight administered strong CYP2D6 inhibitors, e.g., paroxetine, fluoxetine, and quinidine, or in patients who are known to be CYP2D6 PMs, STRATTERA should be initiated at 0.5 mg/kg/day and only increased to the usual target dose of 1.2 mg/kg/day if symptoms fail to improve after 4 weeks and the initial dose is well tolerated.<br><br>**5 WARNINGS AND PRECAUTIONS**<br>**5.12 Laboratory Tests**<br>Routine laboratory tests are not required. CYP2D6 metabolism- Poor metabolizers (PMs) of CYP2D6 have a 10-fold higher AUC and a 5-fold higher peak concentration to a given dose of STRATTERA compared with extensive metabolizers (EMs). Approximately 7% of a Caucasian population are PMs. Laboratory tests are available to identify CYP2D6 PMs. The blood levels in PMs are similar to those attained by taking strong inhibitors of CYP2D6. The higher blood levels in PMs lead to a higher rate of some adverse effects of STRATTERA [see Adverse Reactions (6.1)].<br>**5.13 Concomitant Use of Potent CYP2D6 Inhibitors or Use in patients who are known to be CYP2D6 PMs**<br>Atomoxetine is primarily metabolized by the CYP2D6 pathway to 4 hydroxyatomoxetine. Dosage adjustment of STRATTERA may be necessary when coadministered with potent CYP2D6 inhibitors (e.g., paroxetine, fluoxetine, and quinidine) or when administered to CYP2D6 PMs. [See Dosage and Administration (2.4) and Drug Interactions (7.2)].<br><br>**6 ADVERSE REACTIONS**<br>**6.1 Clinical Trials Experience**<br>*Child and Adolescent Clinical Trials*<br>(…) The following adverse reactions occurred in at least 2% of child and adolescent CYP2D6 PM patients and were statistically significantly more frequent in PM patients compared with CYP2D6 EM patients: insomnia (11% of PMs, 6% of EMs); weight decreased (7% of PMs, 4% of EMs); constipation (7% of PMs, 4% of EMs); depression (7% of PMs, 4% of EMs); tremor (5% of PMs, 1% of EMs); excoriation (4% of PMs, 2% of EMs); middle insomnia (3% of PMs, 1% of EMs); conjunctivitis (3% of PMs, 1% of EMs); syncope (3% of PMs, 1% of EMs); early morning awakening (2% of PMs, 1% of EMs); mydriasis (2% of PMs, 1% of EMs); sedation (4% of PMs, 2% of EMs). (…)<br>*Adult Clinical Trials*<br>(…) The following adverse events occurred in at least 2% of adult CYP2D6 poor metaboliser (PM) patients and were statistically significantly more frequent in PM patients compared to CYP2D6 extensive metaboliser (EM) patients: vision blurred (4% of PMs, 1% of EMs); dry mouth (35% of PMs, 17% of EMs); constipation (11% of PMs, 7% of EMs); feeling jittery (5% of PMs, 2% of EMs); decreased appetite (23% of PMs, 15% of EMs); tremor (5% of PMs, 1% of EMs); insomnia (19% of PMs, 11% of EMs); sleep disorder (7% of PMs, 3% of EMs); middle insomnia (5% of PMs, 3% of EMs); terminal insomnia (3% of PMs, 1% of EMs); urinary retention (6% of PMs, 1% of EMs); erectile dysfunction (21% of PMs, 9% of EMs); ejaculation disorder (6% of PMs, 2% of EMs); hyperhidrosis (15% of PMs, 7% of EMs); peripheral coldness (3% of PMs, 1% of EMs). (…)<br><br>**7 DRUG INTERACTIONS**<br>**7.2 Effect of CYP2D6 Inhibitors on Atomoxetine**<br>In extensive metabolizers (EMs), inhibitors of CYP2D6 (e.g., paroxetine, fluoxetine, and quinidine) increase atomoxetine steady-state plasma concentrations to exposures similar to those observed in poor metabolizers (PMs). In EM individuals treated with paroxetine or fluoxetine, the AUC of atomoxetine is approximately 6- to 8-fold and Css, max is about 3- to 4-fold greater than atomoxetine alone.<br>In vitro studies suggest that coadministration of cytochrome P450 inhibitors to PMs will not increase the plasma concentrations of atomoxetine.<br><br>**8 USE IN SPECIFIC POPULATIONS**<br>**8.6 Hepatic Insufficiency**<br>Atomoxetine exposure (AUC) is increased, compared with normal subjects, in EM subjects with moderate (ChildPugh Class B) (2-fold increase) and severe (Child-Pugh Class C) (4-fold increase) hepatic insufficiency. Dosage adjustment is recommended for patients with moderate or severe hepatic insufficiency [see Dosage and Administration (2.3)].<br>**8.7 Renal Insufficiency**<br>EM subjects with end stage renal disease had higher systemic exposure to atomoxetine than healthy subjects (about a 65% increase), but there was no difference when exposure was corrected for mg/kg dose. STRATTERA can therefore be administered to ADHD patients with end stage renal disease or lesser degrees of renal insufficiency using the normal dosing regimen.<br>**8.9 Ethnic Origin**<br>Ethnic origin did not influence atomoxetine disposition (except that PMs are more common in Caucasians).<br><br>**12 CLINICAL PHARMACOLOGY**<br>**12.2 Pharmacodynamics**<br>*Cardiac Electrophysiology*<br>The effect of STRATTERA on QTc prolongation was evaluated in a randomized, double-blinded, positive-(moxifloxacin 400 mg) and placebo-controlled, cross-over study in healthy male CYP2D6 poor metabolizers. A total of 120 healthy subjects were administered STRATTERA (20 mg and 60 mg) twice daily for 7 days. No large changes in QTc interval (i.e., increases >60 msec from baseline, absolute QTc >480 msec) were observed in the study. However, small changes in QTc interval cannot be excluded from the current study, because the study failed to demonstrate assay sensitivity. There was a slight increase in QTc interval with increased atomoxetine concentration.<br>**12.3 Pharmacokinetics**

* Therapeutic areas do not necessarily reflect the CDER review division.
† Representative biomarkers are listed based on standard nomenclature as per the Human Genome Organization (HUGO) symbol and/or simplified descriptors using other common conventions. Listed biomarkers do not necessarily reflect the terminology used in labeling. The term “Nonspecific” is provided when labeling does not explicitly identify the specific biomarker(s) or when the biomarker is represented by a molecular phenotype or gene signature, and in some cases the biomarker was inferred based on the labeling language.
‡ Referenced figures and tables may be found in the labeling available at Drugs@FDA.
Blue text represents the most recent addtions and/or changes since last posted version.

NDA/ANDA/BLA Number, Label Version Date	Drug	Therapeutic Area*	Biomarker†	Labeling Sections	Labeling Text‡
					Atomoxetine is well-absorbed after oral administration and is minimally affected by food. It is eliminated primarily by oxidative metabolism through the cytochrome P450 2D6 (CYP2D6) enzymatic pathway and subsequent glucuronidation. Atomoxetine has a half-life of about 5 hours. A fraction of the population (about 7% of Caucasians and 2% of African Americans) are poor metabolizers (PMs) of CYP2D6 metabolized drugs. These individuals have reduced activity in this pathway resulting in 10-fold higher AUCs, 5-fold higher peak plasma concentrations, and slower elimination (plasma half-life of about 24 hours) of atomoxetine compared with people with normal activity [extensive metabolizers (EMs)]. (…)<br>*Absorption and distribution*<br>Atomoxetine is rapidly absorbed after oral administration, with absolute bioavailability of about 63% in EMs and 94% in PMs. Maximal plasma concentrations (Cmax) are reached approximately 1 to 2 hours after dosing. (…)<br>*Metabolism and elimination*<br>Atomoxetine is metabolized primarily through the CYP2D6 enzymatic pathway. People with reduced activity in this pathway (PMs) have higher plasma concentrations of atomoxetine compared with people with normal activity (EMs). For PMs, AUC of atomoxetine is approximately 10-fold and Css, max is about 5-fold greater than EMs. Laboratory tests are available to identify CYP2D6 PMs. Coadministration of STRATTERA with potent inhibitors of CYP2D6, such as fluoxetine, paroxetine, or quinidine, results in a substantial increase in atomoxetine plasma exposure, and dosing adjustment may be necessary [see Warnings and Precautions (5.13)]. Atomoxetine did not inhibit or induce the CYP2D6 pathway.<br>The major oxidative metabolite formed, regardless of CYP2D6 status, is 4-hydroxyatomoxetine, which is glucuronidated.<br>4-Hydroxyatomoxetine is equipotent to atomoxetine as an inhibitor of the norepinephrine transporter but circulates in plasma at much lower concentrations (1% of atomoxetine concentration in EMs and 0.1% of atomoxetine concentration in PMs). 4-Hydroxyatomoxetine is primarily formed by CYP2D6, but in PMs, 4-hydroxyatomoxetine is formed at a slower rate by several other cytochrome P450 enzymes. N-Desmethylatomoxetine is formed by CYP2C19 and other cytochrome P450 enzymes, but has substantially less pharmacological activity compared with atomoxetine and circulates in plasma at lower concentrations (5% of atomoxetine concentration in EMs and 45% of atomoxetine concentration in PMs).<br>Mean apparent plasma clearance of atomoxetine after oral administration in adult EMs is 0.35 L/hr/kg and the mean half-life is 5.2 hours. Following oral administration of atomoxetine to PMs, mean apparent plasma clearance is 0.03 L/hr/kg and mean half-life is 21.6 hours. For PMs, AUC of atomoxetine is approximately 10-fold and Css, max is about 5-fold greater than EMs. The elimination half-life of 4-hydroxyatomoxetine is similar to that of N- desmethylatomoxetine (6 to 8 hours) in EM subjects, while the half-life of N-desmethylatomoxetine is much longer in PM subjects (34 to 40 hours). (…)
215358, **10/29/2021**	**Asciminib**	Oncology	BCR-ABL1 (Philadelphia chromosome)	Indication and Usage, Dosage and Administration, Adverse Reactions, Use in Specific Populations, Clinical Studies	**1 INDICATIONS AND USAGE**<br>SCEMBLIX is indicated for the treatment of adult patients with:<br>• Philadelphia chromosome-positive chronic myeloid leukemia (Ph+ CML) in chronic phase (CP), previously treated with two or more tyrosine kinase inhibitors (TKIs).<br>This indication is approved under accelerated approval based on major molecular response (MMR) [see Clinical Studies (14.1)]. Continued approval for this indication may be contingent upon verification and description of clinical benefit in a confirmatory trial(s).<br>• Ph+ CML in CP with the T315I mutation.<br><br>**2 DOSAGE AND ADMINISTRATION**<br>**2.1 Recommended Dosage in Patients with Ph+ CML-CP, Previously Treated with Two or More TKIs**<br>The recommended dose of SCEMBLIX is 80 mg taken orally once daily at approximately the same time each day or 40 mg twice daily at approximately 12-hour intervals. The recommended dose of SCEMBLIX is taken orally without food. Avoid food consumption for at least 2 hours before and 1 hour after taking SCEMBLIX [see Clinical Pharmacology (12.2)]. Continue treatment with SCEMBLIX as long as clinical benefit is observed or until unacceptable toxicity occurs.<br>**2.2 Recommended Dosage in Patients with Ph+ CML-CP with the T315I Mutation**<br>The recommended dose of SCEMBLIX is 200 mg taken orally twice daily at approximately 12-hour intervals. The recommended dose of SCEMBLIX is taken orally without food. Avoid food consumption for at least 2 hours before and 1 hour after taking SCEMBLIX [see Clinical Pharmacology (12.2)]. (See Table 1)<br><br>**6 ADVERSE REACTIONS**<br>**6.1 Clinical Trials Experience**<br>Because clinical trials are conducted under widely varying conditions, adverse reaction rates observed in the clinical trials of a drug cannot be directly compared to rates in the clinical trials of another drug and may not reflect the rates observed in practice.<br>The pooled safety population described in the WARNING AND PRECAUTIONS reflect exposure to SCEMBLIX at 10 mg to 200 mg orally twice daily (between 0.25 to 5 times the recommended dosage for the 80 mg daily dosage and between 0.05 times and up to the recommended dosage for the 200 mg twice daily dosage) in 356 patients enrolled in one of two clinical trials , including patients with Ph+ CML in chronic (CP) receiving SCEMBLIX as monotherapy: study CABL001A2301 (ASCEMBL) and study CABL001X2101 [see Clinical Studies (14)]. Among the 356 patients receiving SCEMBLIX, the median duration of exposure to SCEMBLIX was 89 weeks (range, 0.1 to 342 weeks).<br><u>Adverse Reactions in Patients with Ph+ CML-CP, Previously Treated with Two or More TKIs</u><br>The clinical trial randomized and treated 232 patients with Ph+ CML-CP, previously treated with two or more TKIs to receive SCEMBLIX 40 mg twice daily or bosutinib 500 mg once daily (ASCEMBL) [see Clinical Studies (14.1)]. The safety population (received at least 1 dose of SCEMBLIX) included 156 patients with Ph+ CML-CP, previously treated with two or more TKIs. Among patients who received SCEMBLIX, 83% were exposed for 24 weeks or longer and 67% were exposed for 48 weeks or longer. (See Tables 3 and 4)<br><u>Adverse Reactions in Patients with Ph+ CML-CP with the T315I Mutation</u><br>The single-arm clinical trial enrolled patients with Ph+ CML-CP with the T315I mutation [see Clinical Studies (14.2)]. The safety population (received at least 1 dose of SCEMBLIX) included 48 patients with Ph+ CML-CP with the T315I mutation who received 200 mg of SCEMBLIX twice daily. Among these patients, 83% were exposed for 24 weeks or longer and 75% were exposed for 48 weeks or longer. (See Tables 5 and 6)

* Therapeutic areas do not necessarily reflect the CDER review division.
† Representative biomarkers are listed based on standard nomenclature as per the Human Genome Organization (HUGO) symbol and/or simplified descriptors using other common conventions. Listed biomarkers do not necessarily reflect the terminology used in labeling. The term “Nonspecific” is provided when labeling does not explicitly identify the specific biomarker(s) or when the biomarker is represented by a molecular phenotype or gene signature, and in some cases the biomarker was inferred based on the labeling language.
‡ Referenced figures and tables may be found in the labeling available at Drugs@FDA.
Blue text represents the most recent addtions and/or changes since last posted version.

Table of Pharmacogenomic Biomarkers in Drug Labeling
Last Updated: 06/2024

NDA/ANDA/BLA Number, Label Version Date	Drug	Therapeutic Area*	Biomarker†	Labeling Sections	Labeling Text‡
					**8 USE IN SPECIFIC POPULATIONS**<br>**8.5 Geriatric Use**<br>In the ASCEMBL study, 44 of the 233 (19%) patients were 65 years of age or older and 6 (2.6%) were 75 years of age or older.<br>In the X2101 study, 16 of the 48 (33%) patients with the T315I mutation were 65 years of age or older and 4 (8%) were 75 years of age or older.<br>Overall, no differences in safety or efficacy of SCEMBLIX were observed between patients 65 years of age or older compared to younger patients. There is an insufficient number of patients 75 years of age or older to assess whether there are differences in safety or efficacy.<br><br>**14 CLINICAL STUDIES**<br>**14.1 Ph+ CML-CP, Previously Treated With Two or More TKIs**<br>The efficacy of SCEMBLIX in the treatment of patients with Ph+ CML in chronic phase (Ph+ CML-CP), previously treated with two or more tyrosine kinase inhibitors was evaluated in the multi-center, randomized, active-controlled, and open-label study ASCEMBL (NCT 03106779). (See Table 8)<br>**14.2 Ph+ CML-CP with the T315I mutation**<br>The efficacy of SCEMBLIX in the treatment of patients with Ph+ CML-CP with the T315I mutation was evaluated in a multi-center open-label study CABL001X2101 (NCT02081378). Testing for T315I mutation utilized a qualitative p210 BCR-ABL mutation test using Sanger Sequencing. Efficacy was based on 45 patients with Ph+ CML-CP with the T315I mutation who received SCEMBLIX at a dose of 200 mg twice daily. Patients continued treatment until unacceptable toxicity or treatment failure occurred.
209112, 07/08/2022	Ascorbic Acid	Endocrinology	G6PD	Dosage and Administration, Warnings and Precautions, Adverse Reactions, Patient Counseling Information	**2 DOSAGE AND ADMINISTRATION**<br>**2.3 Dosage Reductions in Specific Populations**<br>Women who are pregnant or lactating and patients with glucose-6-dehydrogenase deficiency should not exceed the U.S. Recommended Dietary Allowance (RDA) or daily Adequate Intake (AI) level for ascorbic acid for their age group and condition [see Warnings and Precautions (5.2) and Use in Specific Populations (8.1, 8.2)].<br><br>**5 WARNINGS AND PRECAUTIONS**<br>**5.2 Hemolysis in Patients with Glucose-6-Phosphate Dehydrogenase Deficiency**<br>Hemolysis has been reported with administration of ascorbic acid in patients with glucose-6-phosphate dehydrogenase deficiency. Patients with glucose-6-phosphate dehydrogenase deficiency may be at increased risk for severe hemolysis during treatment with ascorbic acid. Monitor hemoglobin and blood count and use a reduced dose of ASCOR in patients with glucose-6-phosphate dehydrogenase deficiency [see Dosage and Administration (2.3)]. Discontinue treatment with ASCOR if hemolysis is suspected and treat as needed.<br><br>**6 ADVERSE REACTIONS**<br>• Hemolysis in patients with glucose-6-phosphate dehydrogenase deficiency [see Warnings and Precautions (5.2)] .<br>(…) Acute and chronic oxalate nephropathy have occurred with prolonged administration of high doses of ascorbic acid [see Warnings and Precautions (5.1)]. In patients with glucose-6- phosphate dehydrogenase deficiency, severe hemolysis has occurred [see Warnings and Precautions (5.2)].<br><br>**17 PATIENT COUNSELING INFORMATION**<br>• Inform patients with glucose-6-phosphate dehydrogenase deficiency that treatment with ASCOR may increase their risk of hemolysis [see Warnings and Precautions (5.2)].
021881, 05/14/2021	**Ascorbic Acid, PEG-3350, Potassium Chloride, Sodium Ascorbate, Sodium Chloride, and Sodium Sulfate**	Gastroenterology	G6PD	Warnings and Precautions, Adverse Reactions	**5 WARNINGS AND PRECAUTIONS**<br>**5.8 Glucose-6-phosphate dehydrogenase (G-6-PD) Deficiency**<br>Since MoviPrep contains sodium ascorbate and ascorbic acid, MoviPrep should be used with caution in patients with glucose-6-phosphate dehydrogenase (G-6-PD) deficiency, especially G-6-PD deficiency patients with an active infection, with a history of hemolysis, or taking concomitant medications known to precipitate hemolytic reactions.<br><br>**6 ADVERSE REACTIONS**<br>Glucose-6-Phosphate Dehydrogenase (G6PD) Deficiency [see Warnings and Precautions (5.8)]
212608, **05/22/2023**	Avapritinib (1)	Oncology	PDGFRA	Indications and Usage, Dosage and Administration, Clinical Studies	**1 INDICATIONS AND USAGE**<br>**1.1 PDGFRA Exon 18 Mutation-Positive Unresectable or Metastatic Gastrointestinal Stromal Tumor (GIST)**<br>AYVAKIT is indicated for the treatment of adults with unresectable or metastatic GIST harboring a platelet-derived growth factor receptor alpha (PDGFRA) exon 18 mutation, including PDGFRA D842V mutations [see Dosage and Administration (2.1)].<br><br>**2 DOSAGE AND ADMINISTRATION**<br>**2.1 Patient Selection for GIST Harboring PDGFRA Exon 18 Mutations**<br>Select patients for treatment with AYVAKIT based on the presence of a PDGFRA exon 18 mutation [see Clinical Studies (14.1)]. An FDA-approved test for the detection of exon 18 mutations is not currently available.<br><br>**14 CLINICAL STUDIES**<br>**14.1 Gastrointestinal Stromal Tumors**

* Therapeutic areas do not necessarily reflect the CDER review division.
† Representative biomarkers are listed based on standard nomenclature as per the Human Genome Organization (HUGO) symbol and/or simplified descriptors using other common conventions. Listed biomarkers do not necessarily reflect the terminology used in labeling. The term “Nonspecific” is provided when labeling does not explicitly identify the specific biomarker(s) or when the biomarker is represented by a molecular phenotype or gene signature, and in some cases the biomarker was inferred based on the labeling language.
‡ Referenced figures and tables may be found in the labeling available at Drugs@FDA.
Blue text represents the most recent addtions and/or changes since last posted version.

NDA/ANDA/BLA Number, Label Version Date	Drug	Therapeutic Area*	Biomarker†	Labeling Sections	Labeling Text‡
					Patients with GIST Harboring a PDGFRA Exon 18 Mutation<br>Patients with unresectable or metastatic GIST harboring a PDGFRA exon 18 mutation were identified by local or central assessment using a PCR- or NGS-based assay. The assessment of efficacy was based on a total of 43 patients, including 38 patients with PDGFRA D842V mutations. The median duration of follow up for patients with PDGFRA exon 18 mutations was 10.6 months (range: 0.3 to 24.9 months).<br>The study population characteristics were median age of 64 years (range: 29 to 90 years), 67% were male, 67% were White, 93% had an ECOG PS of 0-1, 98% had metastatic disease, 53% had largest target lesion >5 cm, and 86% had prior surgical resection. The median number of prior kinase inhibitors was 1 (range: 0 to 5).<br>Efficacy results in patients with GIST harboring PDGFRA exon 18 mutations including the subgroup of patients with PDGFRA D842V mutations enrolled in NAVIGATOR are summarized in Table 5. (See Table 8)
**212608, 05/22/2023**	**Avapritinib (2)**	Oncology	KIT	Clinical Studies	**14 CLINICAL STUDIES**<br>**14.2 Advanced Systemic Mastocytosis**<br>The efficacy of AYVAKIT was demonstrated in EXPLORER (NCT02561988) and PATHFINDER (NCT03580655), two multi-center, single-arm, open-label clinical trials. Response-evaluable patients include those with a confirmed diagnosis of AdvSM per World Health Organization (WHO) and deemed evaluable by modified international working group-myeloproliferative neoplasms research and treatmentEuropean competence network on mastocytosis (IWG-MRT-ECNM) criteria at baseline as adjudicated by an independent central committee, who received at least 1 dose of AYVAKIT, had at least 2 post-baseline bone marrow assessments, and had been on study for at least 24 weeks, or had an end of study visit. All enrolled patients had an ECOG performance status (PS) of 0 to 3 and 91% had a platelet count of ≥ 50 X 109 /L prior to initiation of therapy. (…)<br>The study population characteristics were median age of 67 years (range: 37 to 85 years), 58% were male, 98% were White, 68% had an ECOG PS of 0-1, 32% had an ECOG PS of 2-3, 40% had ongoing corticosteroid therapy use for AdvSM at baseline, 66% had prior antineoplastic therapy, 47% had received prior midostaurin, and 94% had a D816V mutation. The median bone marrow mast cell infiltrate was 50%, the median serum tryptase level was 255.8 ng/mL, and the median KIT D816V mutant allele fraction was 12.2%. (…)<br>**14.3 Indolent Systemic Mastocytosis**<br>The efficacy of AYVAKIT was demonstrated in PIONEER (NCT03731260), a randomized, double-blind, placebo-controlled trial conducted in adult patients with Indolent Systemic Mastocytosis (ISM) based on World Health Organization (WHO) classification. (…)<br>Additional supportive results included the proportion of AYVAKIT-treated patients achieving ≥50% reduction from baseline through Week 24 in TSS compared to placebo. Objective measures of mast cell burden were assessed including the proportion of AYVAKIT-treated patients with a ≥50% reduction from baseline through Week 24 in serum tryptase, peripheral blood KIT D816V allele fraction and bone marrow mast cells.<br>The median age of the patients who received AYVAKIT was 50 years (range: 18 to 77 years), 71% were female, 77% were White, <1% were Asian, 3% had other race and 19% had missing race. Ethnicities included 4% Hispanic or Latino. KIT D816V mutations were identified in 93% of patients. At baseline, the mean TSS was 50.17 (standard deviation: 19.15), the median serum tryptase level was 38.40 ng/mL, the median KIT D816V mutant allele fraction was 0.39% by ddPCR and the median bone marrow mast cell infiltrate was 7%. Study population characteristics were similar in the placebo group. (See Table 14) (…)
210238, **06/30/2019**	**Avatrombopag (1)**	Hematology	F2 (Prothrombin)	Warnings and Precautions	**5 WARNINGS AND PRECAUTIONS**<br>**5.1 Thrombotic/Thromboembolic Complications**<br>DOPTELET is a thrombopoietin (TPO) receptor agonist and TPO receptor agonists have been associated with thrombotic and thromboembolic complications in patients with chronic liver disease. Portal vein thrombosis has been reported in patients with chronic liver disease treated with TPO receptor agonists. In the ADAPT-1 and ADAPT-2 clinical trials, there was 1 treatment-emergent event of portal vein thrombosis in a patient (n=1/430) with chronic liver disease and thrombocytopenia treated with DOPTELET. Consider the potential increased thrombotic risk when administering DOPTELET to patients with known risk factors for thromboembolism, including genetic prothrombotic conditions (Factor V Leiden, Prothrombin 20210A, Antithrombin deficiency or Protein C or S deficiency). DOPTELET should not be administered to patients with chronic liver disease in an attempt to normalize platelet counts.
210238, **06/30/2019**	**Avatrombopag (2)**	Hematology	F5 (Factor V Leiden)	Warnings and Precautions	**5 WARNINGS AND PRECAUTIONS**<br>**5.1 Thrombotic/Thromboembolic Complications**<br>DOPTELET is a thrombopoietin (TPO) receptor agonist and TPO receptor agonists have been associated with thrombotic and thromboembolic complications in patients with chronic liver disease. Portal vein thrombosis has been reported in patients with chronic liver disease treated with TPO receptor agonists. In the ADAPT-1 and ADAPT-2 clinical trials, there was 1 treatment-emergent event of portal vein thrombosis in a patient (n=1/430) with chronic liver disease and thrombocytopenia treated with DOPTELET. Consider the potential increased thrombotic risk when administering DOPTELET to patients with known risk factors for thromboembolism, including genetic prothrombotic conditions (Factor V Leiden, Prothrombin 20210A, Antithrombin deficiency or Protein C or S deficiency). DOPTELET should not be administered to patients with chronic liver disease in an attempt to normalize platelet counts.
210238, **06/30/2019**	**Avatrombopag (3)**	Hematology	PROC	Warnings and Precautions	**5 WARNINGS AND PRECAUTIONS**<br>**5.1 Thrombotic/Thromboembolic Complications**<br>DOPTELET is a thrombopoietin (TPO) receptor agonist and TPO receptor agonists have been associated with thrombotic and thromboembolic complications in patients with chronic liver disease. Portal vein thrombosis has been reported in patients with chronic liver disease treated with TPO receptor agonists. In the ADAPT-1 and ADAPT-2 clinical trials, there was 1 treatment-emergent event of portal vein thrombosis in a patient (n=1/430) with chronic liver disease and thrombocytopenia treated with DOPTELET. Consider the potential increased thrombotic risk when administering DOPTELET to patients with known risk factors for thromboembolism, including genetic prothrombotic conditions (Factor V Leiden, Prothrombin 20210A, Antithrombin deficiency or Protein C or S deficiency). DOPTELET should not be administered to patients with chronic liver disease in an attempt to normalize platelet counts.
210238, **06/30/2019**	**Avatrombopag (4)**	Hematology	PROS1	Warnings and Precautions	**5 WARNINGS AND PRECAUTIONS**<br>**5.1 Thrombotic/Thromboembolic Complications**

* Therapeutic areas do not necessarily reflect the CDER review division.
† Representative biomarkers are listed based on standard nomenclature as per the Human Genome Organization (HUGO) symbol and/or simplified descriptors using other common conventions. Listed biomarkers do not necessarily reflect the terminology used in labeling. The term “Nonspecific” is provided when labeling does not explicitly identify the specific biomarker(s) or when the biomarker is represented by a molecular phenotype or gene signature, and in some cases the biomarker was inferred based on the labeling language.
‡ Referenced figures and tables may be found in the labeling available at Drugs@FDA.
Blue text represents the most recent addtions and/or changes since last posted version.

NDA/ANDA/BLA Number, Label Version Date	Drug	Therapeutic Area*	Biomarker†	Labeling Sections	Labeling Text‡
					DOPTELET is a thrombopoietin (TPO) receptor agonist and TPO receptor agonists have been associated with thrombotic and thromboembolic complications in patients with chronic liver disease. Portal vein thrombosis has been reported in patients with chronic liver disease treated with TPO receptor agonists. In the ADAPT-1 and ADAPT-2 clinical trials, there was 1 treatment-emergent event of portal vein thrombosis in a patient (n=1/430) with chronic liver disease and thrombocytopenia treated with DOPTELET. Consider the potential increased thrombotic risk when administering DOPTELET to patients with known risk factors for thromboembolism, including genetic prothrombotic conditions (Factor V Leiden, Prothrombin 20210A, Antithrombin deficiency or Protein C or S deficiency). DOPTELET should not be administered to patients with chronic liver disease in an attempt to normalize platelet counts.
210238, **06/30/2019**	Avatrombopag (5)	Hematology	SERPINC1 (Antithrombin III)	Warnings and Precautions	**5 WARNINGS AND PRECAUTIONS**<br>**5.1 Thrombotic/Thromboembolic Complications**<br>DOPTELET is a thrombopoietin (TPO) receptor agonist and TPO receptor agonists have been associated with thrombotic and thromboembolic complications in patients with chronic liver disease. Portal vein thrombosis has been reported in patients with chronic liver disease treated with TPO receptor agonists. In the ADAPT-1 and ADAPT-2 clinical trials, there was 1 treatment-emergent event of portal vein thrombosis in a patient (n=1/430) with chronic liver disease and thrombocytopenia treated with DOPTELET. Consider the potential increased thrombotic risk when administering DOPTELET to patients with known risk factors for thromboembolism, including genetic prothrombotic conditions (Factor V Leiden, Prothrombin 20210A, Antithrombin deficiency or Protein C or S deficiency). DOPTELET should not be administered to patients with chronic liver disease in an attempt to normalize platelet counts.
210238, 06/30/2019	Avatrombopag (6)	Hematology	CYP2C9	Clinical Pharmacology	**12 CLINICAL PHARMACOLOGY**<br>**12.5 Pharmacogenomics**<br>The CYP2C9*2 and CYP2C9*3 loss-of-function polymorphisms result in reduced CYP2C9 enzymatic activity. In a pooled pharmacogenomic analysis of avatrombopag studies, subjects heterozygous for CYP2C9 loss-of-function polymorphisms (intermediate metabolizers [n=24]) had approximately 1.4-fold higher exposure and subjects homozygous for CYP2C9 loss-of-function polymorphisms (poor metabolizers [n=2]) had approximately 2-fold higher exposure compared to subjects wild-type for CYP2C9 (normal metabolizers [n=94]).
761049, **06/30/2020**	Avelumab	Oncology	CD274 (PD-L1)	Clinical Studies	**14 CLINICAL STUDIES**<br>**14.1 Metastatic Merkel Cell Carcinoma**<br>(…) A total of 88 patients were enrolled. Baseline patient characteristics were a median age of 73 years (range: 33 to 88), 74% of patients were male, 92% were White, and the ECOG performance score was 0 (56%) or 1 (44%). Seventy-five percent of patients were 65 years or older, 35% were 75 or older and 3% were 85 or older. Sixty-five percent of patients were reported to have had one prior anti-cancer therapy for metastatic MCC and 35% had two or more prior therapies. Fifty-three percent of patients had visceral metastases. All patients had tumor samples evaluated for PD-L1 expression; of these, 66% were PD-L1-positive (≥ 1% of tumor cells), 18% were PD-L1 negative, and 16% had non-evaluable results by an investigational immunohistochemistry assay. Archival tumor samples were evaluated for Merkel cell polyomavirus (MCV) using an investigational assay; of the 77 patients with evaluable results, 52% had evidence of MCV. Efficacy results are presented in Table 8. Responses were observed in patients regardless of tumor PD-L1 expression or presence of MCV.<br>**14.2 Locally Advanced or Metastatic Urothelial Carcinoma**<br>*First-Line Maintenance Treatment of Urothelial Carcinoma*<br>(…) Fifty-six percent (56%) of patients received prior gemcitabine plus cisplatin, 38% of patients received prior gemcitabine plus carboplatin, and 6% of patients received prior gemcitabine plus cisplatin and gemcitabine plus carboplatin. Best response to first-line chemotherapy was CR or PR (72%) or SD (28%). Sites of metastasis prior to chemotherapy were visceral (55%) or nonvisceral (45%). Fifty-one (51%) of patients had PD-L1-positive-tumors, 39% of patients had PD-L1-negative tumors, and 10% of patients had unknown PD-L1 tumor status. Six percent (6%) of patients received another PD-1/PD-L1 checkpoint inhibitor after discontinuation of treatment in the BAVENCIO plus BSC arm and 44% of patients in the BSC arm.<br>The major efficacy outcome measure was overall survival (OS) in all randomized patients and patients with PD-L1-positive tumors. The trial demonstrated a statistically significant improvement in OS for patients randomized to BAVENCIO plus BSC as compared with BSC alone (Table 9 and Figure 1). Consistent results were observed across the pre-specified subgroup of CR/PR versus SD to first-line chemotherapy.<br>In the prespecified endpoint of OS among patients with PD-L1-positive tumors (n=358, 51%), the hazard ratio was 0.56 (95% CI: 0.40, 0.79; 2-sided p-value <0.001) for patients randomized to BAVENCIO plus BSC versus BSC alone. In an exploratory analysis of patients with PD-L1-negative tumors (n=271, 39%), the OS hazard ratio was 0.85 (95% CI: 0.62, 1.18).<br>*Previously-Treated Urothelial Carcinoma*<br>Patients were included regardless of their PD-L1 status. (…)<br>Efficacy results are presented in Table 10. The median time to response was 2.0 months (range: 1.3 to 11.0) among patients followed for either > 13 weeks or > 6 months. Using a clinical trial assay to assess PD-L1 staining, with 16% of patients not evaluable, there were no clear differences in response rates based on PD-L1 tumor expression. (…)<br>**14.3 Advanced Renal Cell Carcinoma**<br>The efficacy and safety of BAVENCIO in combination with axitinib was demonstrated in the JAVELIN Renal 101 trial (NCT02684006), a randomized, multicenter, open-label, study of BAVENCIO in combination with axitinib in 886 patients with untreated advanced RCC regardless of tumor PD-L1 expression [intent-to-treat (ITT) population]. (…)<br>The major efficacy outcome measures were progression-free survival (PFS), as assessed by an BICR using RECIST v1.1 and overall survival (OS) in patients with PD-L1-positive tumors using a clinical trial assay (PD-L1 expression level ≥ 1%). Since PFS was statistically significant in patients with PD-L1-positive tumors [HR 0.61 (95% CI: 0.48, 0.79)], it was then tested in the ITT population and a statistically significant improvement in PFS in the ITT population was also demonstrated.
**050794, 05/20/2022**	Azacitidine (1)	Oncology	CBL	Clinical Studies	**14 CLINICAL STUDIES**<br>**14.2 Juvenile Myelomonocytic Leukemia (JMML)**

* Therapeutic areas do not necessarily reflect the CDER review division.
† Representative biomarkers are listed based on standard nomenclature as per the Human Genome Organization (HUGO) symbol and/or simplified descriptors using other common conventions. Listed biomarkers do not necessarily reflect the terminology used in labeling. The term “Nonspecific” is provided when labeling does not explicitly identify the specific biomarker(s) or when the biomarker is represented by a molecular phenotype or gene signature, and in some cases the biomarker was inferred based on the labeling language.
‡ Referenced figures and tables may be found in the labeling available at Drugs@FDA.
Blue text represents the most recent addtions and/or changes since last posted version.

NDA/ANDA/BLA Number, Label Version Date	Drug	Therapeutic Area*	Biomarker†	Labeling Sections	Labeling Text‡
					AZA-JMML-001 (NCT02447666) was an international, multicenter, open-label study to evaluate the pharmacokinetics, pharmacodynamics, safety, and activity of VIDAZA prior to hematopoietic stem cell transplantation (HSCT) in a total of 18 pediatric patients (median age of 2.1 years, range 0.2-6.9 years; 61% male; 89% white) with juvenile myelomonocytic leukemia (JMML).<br>Patients with newly diagnosed JMML were included if they met the following criteria: diagnosis confirmed in peripheral blood and bone marrow and had one of the following: somatic mutation in PTPN11, KRAS, or NRAS and HbF % > 5 x normal value for age, or clinical diagnosis of neurofibromatosis Type 1 (NF-1). Additionally, patients included had no CNS involvement, isolated extramedullary disease, or germline molecular aberrations in CBL, PTPN11, NRAS, or KRAS. Eighteen patients with JMML (13 PTPN11, 3 NRAS, 1 KRAS somatic mutations and 1 clinical diagnosis of neurofibromatosis type 1 [NF 1]) were enrolled. (…)
**050794, 05/20/2022**	Azacitidine (2)	Oncology	PTPN11	Clinical Studies	**14 CLINICAL STUDIES**<br>**14.2 Juvenile Myelomonocytic Leukemia (JMML)**<br>AZA-JMML-001 (NCT02447666) was an international, multicenter, open-label study to evaluate the pharmacokinetics, pharmacodynamics, safety, and activity of VIDAZA prior to hematopoietic stem cell transplantation (HSCT) in a total of 18 pediatric patients (median age of 2.1 years, range 0.2-6.9 years; 61% male; 89% white) with juvenile myelomonocytic leukemia (JMML).<br>Patients with newly diagnosed JMML were included if they met the following criteria: diagnosis confirmed in peripheral blood and bone marrow and had one of the following: somatic mutation in PTPN11, KRAS, or NRAS and HbF % > 5 x normal value for age, or clinical diagnosis of neurofibromatosis Type 1 (NF-1). Additionally, patients included had no CNS involvement, isolated extramedullary disease, or germline molecular aberrations in CBL, PTPN11, NRAS, or KRAS. Eighteen patients with JMML (13 PTPN11, 3 NRAS, 1 KRAS somatic mutations and 1 clinical diagnosis of neurofibromatosis type 1 [NF 1]) were enrolled. (…)
**050794, 05/20/2022**	Azacitidine (3)	Oncology	RAS	Clinical Studies	**14 CLINICAL STUDIES**<br>**14.2 Juvenile Myelomonocytic Leukemia (JMML)**<br>AZA-JMML-001 (NCT02447666) was an international, multicenter, open-label study to evaluate the pharmacokinetics, pharmacodynamics, safety, and activity of VIDAZA prior to hematopoietic stem cell transplantation (HSCT) in a total of 18 pediatric patients (median age of 2.1 years, range 0.2-6.9 years; 61% male; 89% white) with juvenile myelomonocytic leukemia (JMML).<br>Patients with newly diagnosed JMML were included if they met the following criteria: diagnosis confirmed in peripheral blood and bone marrow and had one of the following: somatic mutation in PTPN11, KRAS, or NRAS and HbF % > 5 x normal value for age, or clinical diagnosis of neurofibromatosis Type 1 (NF-1). Additionally, patients included had no CNS involvement, isolated extramedullary disease, or germline molecular aberrations in CBL, PTPN11, NRAS, or KRAS. Eighteen patients with JMML (13 PTPN11, 3 NRAS, 1 KRAS somatic mutations and 1 clinical diagnosis of neurofibromatosis type 1 [NF 1]) were enrolled. (…)
016324, **12/20/2018**	Azathioprine (1)	Rheumatology	TPMT	Dosage and Administration, Warnings, Precautions, Drug Interactions, Adverse Reactions, Clinical Pharmacology	**DOSAGE AND ADMINISTRATION**<br>**Patients with TPMT and/or NUDT15 Deficiency**<br>Consider testing for TPMT and NUDT15 deficiency in patients who experience severe bone marrow toxicities. Early drug discontinuation may be considered in patients with abnormal CBC results that do not respond to dose reduction (see CLINICAL PHARMACOLOGY, WARNINGS: Cytopenias, and PRECAUTIONS: Laboratory Tests).<br>*Homozygous deficiency in either TPMT or NUDT15*<br>Because of the risk of increased toxicity, consider alternative therapies for patients who are known to have TPMT or NUDT15 deficiency (see CLINICAL PHARMACOLOGY, WARNINGS: Cytopenias, and PRECAUTIONS: Laboratory Tests).<br>*Heterozygous deficiency in TPMT and/or NUDT15*<br>Because of the risk of increased toxicity, dosage reduction is recommended in patients known to have heterozygous deficiency of TPMT or NUDT15. Patients who are heterozygous for both TPMT and NUDT15 deficiency may require more substantial dosage reductions (see CLINICAL PHARMACOLOGY, WARNINGS: Cytopenias, and PRECAUTIONS: Laboratory Tests).<br><br>**WARNINGS**<br>*Cytopenias*<br>*TPMT or NUDT15 Deficiency*<br>(…) Patients with thiopurine S-methyl transferase (TPMT) or nucleotide diphospatase (NUDT15) deficiency may be at an increased risk of severe and life-threatening myelotoxicity if receiving conventional doses of IMURAN (see CLINCIAL PHARMACOLOGY). Death associated with pancytopenia has been reported in patients with absent TPMT activity receiving azathioprine. In patients with severe myelosuppression, consider evaluation for TPMT and NUDT15 deficiency (see PRECAUTIONS: Laboratory Tests). Consider alternative therapy in patients with homozygous TPMT or NUDT15 deficiency and reduced dosages in patients with heterozygous deficiency (see DOSAGE AND ADMINISTRATION).<br><br>**PRECAUTIONS**<br>***TPMT and NUDT15 Testing:*** Consider genotyping or phenotyping patients for TPMT deficiency and genotyping for NUDT1 deficiency in patients with severe myelosuppression. TPMT and NUDT15 testing cannot substitute for complete blood count (CBC) monitoring in patients receiving IMURAN. Accurate phenotyping (red blood cell TPMT activity) results are not possible in patients who have received recent blood transfusions (see CLINICAL PHARMACOLOGY, WARNINGS: Cytopenias, ADVERSE REACTIONS and DOSAGE AND ADMINISTRATION sections). *Drug Interactions*<br>Use with Allopurinol: One of the pathways for inactivation of azathioprine is inhibited by allopurinol. Patients receiving IMURAN and allopurinol concomitantly should have a dose reduction of IMURAN, to approximately 1/3 to 1/4 the usual dose. It is recommended that a further dose reduction or alternative therapies be considered for patients with low or absent TPMT activity receiving IMURAN and allopurinol because both TPMT and XO inactivation pathways are affected. See CLINICAL PHARMACOLOGY, WARNINGS, PRECAUTIONS: Laboratory Tests and ADVERSE REACTIONS sections.<br><br>**ADVERSE REACTIONS**<br>*Hematologic*

Table of Pharmacogenomic Biomarkers in Drug Labeling
Last Updated: 06/2024

NDA/ANDA/BLA Number, Label Version Date	Drug	Therapeutic Area*	Biomarker†	Labeling Sections	Labeling Text‡
					(…) Patients with low or absent TPMT or NUDT15 activity are at increased risk for severe, life-threatening myelosuppression from IMURAN (see CLINICAL PHARMACOLOGY, WARNINGS: Cytopenias and PRECAUTIONS: Laboratory Tests, DOSAGE AND ADMINISTRATION).<br><br>**CLINICAL PHARMACOLOGY**<br>(…) 6-MP undergoes two major inactivation routes. One is thiol methylation, which is catalyzed by the enzyme thiopurine Smethyltransferase (TPMT), to form the inactive metabolite methyl-6-MP (6-MeMP). Another inactivation pathway is oxidation, which is catalyzed by xanthine oxidase (XO) to form 6-thiouric acid. The nucleotide diphosphatase (NUDT15) enzyme is involved in conversion of the 6-TGNs to inactive 6-TG monophosphates. TPMT activity correlates inversely with 6-TGN levels in erythrocytes and presumably other hematopoietic tissues, since these cells have negligible xanthine oxidase (involved in the other inactivation pathway) activities.<br>Genetic polymorphisms influence TPMT and NUDT15 activity. Several published studies indicate that patients with reduced TPMT or NUDT15 activity receiving usual doses of 6-MP or azathioprine, accumulate excessive cellular concentrations of active 6-TGNs, and are at higher risk for severe myelosuppression. Because of the risk of toxicity, patients with TPMT or NUDT15 deficiency require alternative therapy or dose modification (see DOSAGE and ADMINISTRATION).<br>Approximately 0.3% (1:300) of patients of European or African ancestry have two loss-of-function alleles of the TPMT gene and have little or no TPMT activity (homozygous deficient or poor metabolizers), and approximately 10% of patients have one loss-of-function TPMT allele leading to intermediate TPMT activity (heterozygous deficient or intermediate metabolizers). The TPMT*2, TPMT*3A, and TPMT*3C alleles account for about 95% of individuals with reduced levels of TPMT activity. NUDT15 deficiency is detected in <1% of patients of European or African ancestry. Among patients of East Asian ancestry (i.e., Chinese, Japanese, Vietnamese), 2% have two loss-of-function alleles of the NUDT15 gene, and approximately 21% have one loss-of-function allele. The p.R139C variant of NUDT15 (present on the *2 and *3 alleles) is the most commonly observed, but other less common loss-of-function NUDT15 alleles have been observed. (…)
016324, 12/20/2018	Azathioprine (2)	Rheumatology	NUDT15	Dosage and Administration, Warnings, Precautions, Adverse Reactions, Clinical Pharmacology	**DOSAGE AND ADMINISTRATION**<br>**Patients with TPMT and/or NUDT15 Deficiency**<br>Consider testing for TPMT and NUDT15 deficiency in patients who experience severe bone marrow toxicities. Early drug discontinuation may be considered in patients with abnormal CBC results that do not respond to dose reduction (see CLINICAL PHARMACOLOGY, WARNINGS: Cytopenias, and PRECAUTIONS: Laboratory Tests).<br>*Homozygous deficiency in either TPMT or NUDT15*<br>Because of the risk of increased toxicity, consider alternative therapies for patients who are known to have TPMT or NUDT15 deficiency (see CLINICAL PHARMACOLOGY, WARNINGS: Cytopenias, and PRECAUTIONS: Laboratory Tests).<br>*Heterozygous deficiency in TPMT and/or NUDT15*<br>Because of the risk of increased toxicity, dosage reduction is recommended in patients known to have heterozygous deficiency of TPMT or NUDT15. Patients who are heterozygous for both TPMT and NUDT15 deficiency may require more substantial dosage reductions (see CLINICAL PHARMACOLOGY, WARNINGS: Cytopenias, and PRECAUTIONS: Laboratory Tests).<br><br>**WARNINGS**<br>*Cytopenias*<br>*TPMT or NUDT15 Deficiency*<br>(…) Patients with thiopurine S-methyl transferase (TPMT) or nucleotide diphosphatase (NUDT15) deficiency may be at an increased risk of severe and life-threatening myelotoxicity if receiving conventional doses of IMURAN (see CLINCIAL PHARMACOLOGY). Death associated with pancytopenia has been reported in patients with absent TPMT activity receiving azathioprine. In patients with severe myelosuppression, consider evaluation for TPMT and NUDT15 deficiency (see PRECAUTIONS: Laboratory Tests). Consider alternative therapy in patients with homozygous TPMT or NUDT15 deficiency and reduced dosages in patients with heterozygous deficiency (see DOSAGE AND ADMINISTRATION).<br><br>**PRECAUTIONS**<br>***TPMT and NUDT15 Testing:*** Consider genotyping or phenotyping patients for TPMT deficiency and genotyping for NUDT15 deficiency in patients with severe myelosuppression. TPMT and NUDT15 testing cannot substitute for complete blood count (CBC) monitoring in patients receiving IMURAN. Accurate phenotyping (red blood cell TPMT activity) results are not possible in patients who have received recent blood transfusions (see CLINICAL PHARMACOLOGY, WARNINGS: Cytopenias, ADVERSE REACTIONS and DOSAGE AND ADMINISTRATION sections).<br><br>**ADVERSE REACTIONS**<br>*Hematologic*<br>(…) Patients with low or absent TPMT or NUDT15 activity are at increased risk for severe, life-threatening myelosuppression from IMURAN (see CLINICAL PHARMACOLOGY, WARNINGS: Cytopenias and PRECAUTIONS: Laboratory Tests, DOSAGE AND ADMINISTRATION).<br><br>**CLINICAL PHARMACOLOGY**<br>(…) The nucleotide diphosphatase (NUDT15) enzyme is involved in conversion of the 6-TGNs to inactive 6-TG monophosphates. TPMT activity correlates inversely with 6-TGN levels in erythrocytes and presumably other hematopoietic tissues, since these cells have negligible xanthine oxidase (involved in the other inactivation pathway) activities. Genetic polymorphisms influence TPMT and NUDT15 activity. Several published studies indicate that patients with reduced TPMT or NUDT15 activity receiving usual doses of 6-MP or azathioprine, accumulate excessive cellular concentrations of active 6-TGNs, and are at higher risk for severe myelosuppression. Because of the risk of toxicity, patients with TPMT or NUDT15 deficiency require alternative therapy or dose modification (see DOSAGE and ADMINISTRATION).

* Therapeutic areas do not necessarily reflect the CDER review division.
† Representative biomarkers are listed based on standard nomenclature as per the Human Genome Organization (HUGO) symbol and/or simplified descriptors using other common conventions. Listed biomarkers do not necessarily reflect the terminology used in labeling. The term "Nonspecific" is provided when labeling does not explicitly identify the specific biomarker(s) or when the biomarker is represented by a molecular phenotype or gene signature, and in some cases the biomarker was inferred based on the labeling language.
‡ Referenced figures and tables may be found in the labeling available at Drugs@FDA.
Blue text represents the most recent addtions and/or changes since last posted version.

Table of Pharmacogenomic Biomarkers in Drug Labeling
Last Updated: 06/2024

NDA/ANDA/BLA Number, Label Version Date	Drug	Therapeutic Area*	Biomarker†	Labeling Sections	Labeling Text‡
					Approximately 0.3% (1:300) of patients of European or African ancestry have two loss-of-function alleles of the TPMT gene and have little or no TPMT activity (homozygous deficient or poor metabolizers), and approximately 10% of patients have one loss-of-function TPMT allele leading to intermediate TPMT activity (heterozygous deficient or intermediate metabolizers). The TPMT*2, TPMT*3A, and TPMT*3C alleles account for about 95% of individuals with reduced levels of TPMT activity. NUDT15 deficiency is detected in <1% of patients of European or African ancestry. Among patients of East Asian ancestry (i.e., Chinese, Japanese, Vietnamese), 2% have two loss-of-function alleles of the NUDT15 gene, and approximately 21% have one loss-of-function allele. The p.R139C variant of NUDT15 (present on the *2 and *3 alleles) is the most commonly observed, but other less common loss-of-function NUDT15 alleles have been observed. (…)
206256, **01/08/2020**	Belinostat	Oncology	UGT1A1	Dosage and Administration, Clinical Pharmacology	**2 DOSAGE AND ADMINISTRATION**<br>**2.3 Patients with Reduced UGT1A1 Activity**<br>Reduce the starting dose of Beleodaq to 750 mg/m2 in patients known to be homozygous for the UGT1A1*28 allele [see Clinical Pharmacology (12.5)].<br><br>**12 CLINICAL PHARMACOLOGY**<br>**12.5 Pharmacogenomics**<br>UGT1A1 activity is reduced in individuals with genetic polymorphisms that lead to reduced enzyme activity such as the UGT1A1*28 polymorphism. Approximately 20% of the black population, 10% of the white population, and 2% of the Asian population are homozygous for the UGT1A1*28 allele. Additional reduced function alleles may be more prevalent in specific populations.<br>Because belinostat is primarily (80-90%) metabolized by UGT1A1, the clearance of belinostat could be decreased in patients with reduced UGT1A1 activity (e.g., patients with UGT1A1*28 allele). Reduce the starting dose of Beleodaq to 750 mg/m^2 in patients known to be homozygous for the UGT1A1*28 allele to minimize dose limiting toxicities.
21**5383, 12/14/2023**	Belzutifan (1)	Oncology	CYP2C19	Warnings and Precautions, Drug Interactions, Use in Specific Populations, Clinical Pharmacology	**5 WARNINGS AND PRECAUTIONS**<br>**5.1 Anemia**<br>(…) Monitor for anemia before initiation of, and periodically throughout, treatment with WELIREG. Closely monitor patients who are dual UGT2B17 and CYP2C19 poor metabolizers due to potential increases in exposure that may increase the incidence or severity of anemia [see Clinical Pharmacology (12.5)]. (…)<br><br>**7 DRUG INTERACTIONS**<br>**7.2 Effect of WELIREG on Other Drugs**<br>Sensitive CYP3A4 Substrates<br>Coadministration of WELIREG with CYP3A4 substrates decreases concentrations of CYP3A substrates [see Clinical Pharmacology (12.3)], which may reduce the efficacy of these substrates. The magnitude of this decrease may be more pronounced in patients who are dual UGT2B17 and CYP2C19 poor metabolizers [see Clinical Pharmacology (12.3)]. Avoid coadministration of WELIREG with sensitive CYP3A4 substrates, for which minimal decrease in concentration may lead to therapeutic failures of the substrate. If coadministration cannot be avoided, increase the sensitive CYP3A4 substrate dosage in accordance with its Prescribing Information.<br><br>**8 USE IN SPECIFIC POPULATIONS**<br>**8.8 Dual UGT2B17 and CYP2C19 Poor Metabolizers**<br>Patients who are dual UGT2B17 and CYP2C19 poor metabolizers have higher belzutifan exposures, which may increase the incidence and severity of adverse reactions of WELIREG. Closely monitor for adverse reactions in patients who are dual UGT2B17 and CYP2C19 poor metabolizers [see Warnings and Precautions (5), Adverse Reactions (6), Clinical Pharmacology (12.5)].<br><br>**12 CLINICAL PHARMACOLOGY**<br>**12.3 Pharmacokinetics**<br>Specific Populations<br>Patients who are poor metabolizers of UGT2B17 and CYP2C19 had higher belzutifan AUC [see Clinical Pharmacology (12.5)].<br>Drug Interaction Studies<br>*Clinical Studies and Model-Informed Approaches*<br>Effect of Belzutifan on CYP3A Substrates: Coadministration of WELIREG 120 mg once daily with midazolam (a sensitive CYP3A4 substrate) decreased the midazolam AUC by 40% and the Cmax by 34%. Midazolam AUC is predicted to decrease up to 70% in patients with higher belzutifan concentrations (e.g., dual poor metabolizers) [see Clinical Pharmacology (12.5)].<br>**12.5 Pharmacogenomics**<br>Patients who are UGT2B17, CYP2C19, or dual UGT2B17 and CYP2C19 poor metabolizers have 2-, 1.6-, or 3.2-fold higher belzutifan steady state AUC0-24h (respectively) compared to patients who are UGT2B17 normal (extensive) metabolizers and CYP2C19 non-poor (ultrarapid, rapid, normal, and intermediate) metabolizers [see Use in Specific Populations (8.7)].<br>UGT2B17 poor metabolizers who are homozygous for the UGT2B17*2 allele have no UGT2B17 enzyme activity. CYP2C19 poor metabolizers (such as *2/*2, *3/*3, *2/*3) have significantly reduced or absent CYP2C19 enzyme activity. Approximately 15% of White, 6% of Black or African American, and up to 77% of certain Asian populations are UGT2B17 poor metabolizers. Approximately 2% of White, 5% of Black or African American, and up to 19% of certain Asian populations are CYP2C19 poor metabolizers. Approximately 0.4% of White, 0.3% of Black or African American, and up to 15% of certain Asian populations are dual UGT2B17 and CYP2C19 poor metabolizers.

* Therapeutic areas do not necessarily reflect the CDER review division.
† Representative biomarkers are listed based on standard nomenclature as per the Human Genome Organization (HUGO) symbol and/or simplified descriptors using other common conventions. Listed biomarkers do not necessarily reflect the terminology used in labeling. The term “Nonspecific” is provided when labeling does not explicitly identify the specific biomarker(s) or when the biomarker is represented by a molecular phenotype or gene signature, and in some cases the biomarker was inferred based on the labeling language.
‡ Referenced figures and tables may be found in the labeling available at Drugs@FDA.
Blue text represents the most recent addtions and/or changes since last posted version.

Table of Pharmacogenomic Biomarkers in Drug Labeling
Last Updated: 06/2024

NDA/ANDA/BLA Number, Label Version Date	Drug	Therapeutic Area*	Biomarker†	Labeling Sections	Labeling Text‡
215383, **12/14/2023**	**Belzutifan (2)**	Oncology	UGT2B17	Warnings and Precautions, Drug Interactions, Use in Specific Populations, Clinical Pharmacology	**5 WARNINGS AND PRECAUTIONS**<br>**5.1 Anemia**<br>(…) Monitor for anemia before initiation of, and periodically throughout, treatment with WELIREG. Closely monitor patients who are dual UGT2B17 and CYP2C19 poor metabolizers due to potential increases in exposure that may increase the incidence or severity of anemia [see Clinical Pharmacology (12.5)].<br>(…)<br><br>**7 DRUG INTERACTIONS**<br>**7.2 Effect of WELIREG on Other Drugs**<br>Sensitive CYP3A4 Substrates<br>Coadministration of WELIREG with CYP3A4 substrates decreases concentrations of CYP3A substrates [see Clinical Pharmacology (12.3)], which may reduce the efficacy of these substrates. The magnitude of this decrease may be more pronounced in patients who are dual UGT2B17 and CYP2C19 poor metabolizers [see Clinical Pharmacology (12.3)]. Avoid coadministration of WELIREG with sensitive CYP3A4 substrates, for which minimal decrease in concentration may lead to therapeutic failures of the substrate. If coadministration cannot be avoided, increase the sensitive CYP3A4 substrate dosage in accordance with its Prescribing Information.<br><br>**8 USE IN SPECIFIC POPULATIONS**<br>**8.8 Dual UGT2B17 and CYP2C19 Poor Metabolizers**<br>Patients who are dual UGT2B17 and CYP2C19 poor metabolizers have higher belzutifan exposures, which may increase the incidence and severity of adverse reactions of WELIREG. Closely monitor for adverse reactions in patients who are dual UGT2B17 and CYP2C19 poor metabolizers [see Warnings and Precautions (5), Adverse Reactions (6), Clinical Pharmacology (12.5)].<br><br>**12 CLINICAL PHARMACOLOGY**<br>**12.3 Pharmacokinetics**<br>Specific Populations<br>Patients who are poor metabolizers of UGT2B17 and CYP2C19 had higher belzutifan AUC [see Clinical Pharmacology (12.5)].<br>Drug Interaction Studies<br>*Clinical Studies and Model-Informed Approaches*<br>Effect of Belzutifan on CYP3A Substrates: Coadministration of WELIREG 120 mg once daily with midazolam (a sensitive CYP3A4 substrate) decreased the midazolam AUC by 40% and the Cmax by 34%. Midazolam AUC is predicted to decrease up to 70% in patients with higher belzutifan concentrations (e.g., dual poor metabolizers) [see Clinical Pharmacology (12.5)].<br>**12.5 Pharmacogenomics**<br>Patients who are UGT2B17, CYP2C19, or dual UGT2B17 and CYP2C19 poor metabolizers have 2-, 1.6-, or 3.2-fold higher belzutifan steady state AUC0-24h (respectively) compared to patients who are UGT2B17 normal (extensive) metabolizers and CYP2C19 non-poor (ultrarapid, rapid, normal, and intermediate) metabolizers [see Use in Specific Populations (8.7)].<br>UGT2B17 poor metabolizers who are homozygous for the UGT2B17*2 allele have no UGT2B17 enzyme activity. CYP2C19 poor metabolizers (such as *2/*2, *3/*3, *2/*3) have significantly reduced or absent CYP2C19 enzyme activity. Approximately 15% of White, 6% of Black or African American, and up to 77% of certain Asian populations are UGT2B17 poor metabolizers. Approximately 2% of White, 5% of Black or African American, and up to 19% of certain Asian populations are CYP2C19 poor metabolizers. Approximately 0.4% of White, 0.3% of Black or African American, and up to 15% of certain Asian populations are dual UGT2B17 and CYP2C19 poor metabolizers.
215383, **12/14/2023**	**Belzutifan (3)**	Oncology	VHL	Clinical Studies	**14 CLINICAL STUDIES**<br>The efficacy of WELIREG was evaluated in Study 004 (NCT03401788), an open-label clinical trial in 61 patients with VHL-associated RCC diagnosed based on a VHL germline alteration and with at least one measurable solid tumor localized to the kidney as defined by response evaluation criteria in solid tumors (RECIST) v1.1. Enrolled patients had other VHL-associated tumors including CNS hemangioblastomas and pNET. (…)<br>The study population characteristics were: median age 41 years [range 19-66 years], 3.3% age 65 or older; 53% male; 90% were White, 3.3% were Black or African-American, 1.6% were Asian, and 1.6% were Native Hawaiian or other Pacific Islander; 82% had an ECOG PS of 0, 16% had an ECOG PS of 1, and 1.6% had an ECOG PS of 2; and 84% had VHL Type I Disease. The median diameter of RCC target lesions per central independent review committee (IRC) was 2.2 cm (range 1-6.1). Median time from initial radiographic diagnosis of VHL-associated RCC tumors that led to enrollment on Study 004 to the time of treatment with WELIREG was 17.9 months (range 2.8-96.7). Seventy-seven percent of patients had prior surgical procedures for RCC. The major efficacy endpoint for the treatment of VHL-associated RCC was overall response rate (ORR) measured by radiology assessment using RECIST v1.1 as assessed by IRC. Additional efficacy endpoints included duration of response (DoR), and time to response (TTR). Table 4 summarizes the efficacy results for VHL-associated RCC in Study 004. (See Table 4)<br>For VHL-associated RCC, median TTR was 8 months (range 2.7, 19). Table 5 summarizes the efficacy results for VHL-associated pNET or CNS hemangioblastomas in Study 004. (See Table 5)<br>For VHL-associated CNS hemangioblastomas, TTR was 3.1 months (range 2.5, 11). For VHL-associated pNET, median TTR was 8.1 months (range 2.7, 11). Decreases in size of CNS hemangioblastoma-associated peri-tumoral cysts and syringes were observed.
**211617, 03/22/2024**	**Bempedoic Acid and Ezetimibe**	Endocrinology	Nonspecific (Heterozygous Familial Hypercholesterolemia)	Indications and Usage, Adverse Reactions, Clinical Studies	**1 INDICATIONS AND USAGE**<br>NEXLIZET, a combination of bempedoic acid and ezetimibe, is indicated:<br>• As an adjunct to diet, alone or in combination with other low-density lipoprotein cholesterol (LDL-C) lowering therapies, to reduce LDL-C in adults with primary hyperlipidemia, including heterozygous familial hypercholesterolemia (HeFH).<br>The bempedoic acid component of NEXLIZET is indicated:

* Therapeutic areas do not necessarily reflect the CDER review division.
† Representative biomarkers are listed based on standard nomenclature as per the Human Genome Organization (HUGO) symbol and/or simplified descriptors using other common conventions. Listed biomarkers do not necessarily reflect the terminology used in labeling. The term "Nonspecific" is provided when labeling does not explicitly identify the specific biomarker(s) or when the biomarker is represented by a molecular phenotype or gene signature, and in some cases the biomarker was inferred based on the labeling language.
‡ Referenced figures and tables may be found in the labeling available at Drugs@FDA.
Blue text represents the most recent addtions and/or changes since last posted version.

NDA/ANDA/BLA Number, Label Version Date	Drug	Therapeutic Area*	Biomarker†	Labeling Sections	Labeling Text‡
					• To reduce the risk of myocardial infarction and coronary revascularization in adults who are unable to take recommended statin therapy (including those not taking a statin) with:<br>○ established cardiovascular disease (CVD), or<br>○ a high risk for a CVD event but without established CVD.<br><br>**6 ADVERSE REACTIONS**<br>Bempedoic acid<br>The data in Table 1 reflect exposure to bempedoic acid in two placebo-controlled primary hyperlipidemia trials that included 2,009 patients treated with bempedoic acid for 52 weeks (median treatment duration of 52 weeks) [see Clinical Studies (14.1)]. The mean age for bempedoic acid-treated patients was 65 years, 29% were female, 95% were White, 3% were Black or African American, 1% were Asian, and 1% were other races; 3% identified as Hispanic or Latino ethnicity. All patients received bempedoic acid 180 mg orally once daily plus maximally tolerated statin therapy alone or in combination with other lipid-lowering therapies. At baseline, 97% of patients had CVD and about 4% had a diagnosis of HeFH. Patients on simvastatin 40 mg/day or higher were excluded from the trials. (See Table 1)<br>NEXLIZET<br>In a 4-arm, 12-week, randomized, double-blind, placebo-controlled, parallel group, factorial trial, 85 patients received NEXLIZET (180 mg of bempedoic acid and 10 mg of ezetimibe) once daily [see Clinical Studies (14.1)]. The mean age for NEXLIZET-treated patients was 62 years, 51% were female, 78% were White, 19% were Black or African American, 2% were Asian, and 1% were American Indian or Alaska Native; 11% identified as Hispanic or Latino ethnicity. At baseline, 61% of patients had CVD and/or a diagnosis of HeFH. All patients received NEXLIZET plus maximally tolerated statin therapy. Patients taking simvastatin 40 mg/day or higher and patients taking non-statin lipid-lowering therapy (including fibrates, niacin, bile acid sequestrants, ezetimibe, and PCSK9 inhibitors) were excluded from the trial.<br><br>**14 CLINICAL STUDIES**<br>**14.1 Primary Hyperlipidemia Trials in Adults**<br>The efficacy of NEXLIZET was investigated in a single, multi-center, randomized, double-blind, placebo-controlled, parallel group trial that enrolled 301 patients with HeFH, established CVD, or multiple risk factors for CVD on maximally tolerated statin therapy. The efficacy of NEXLIZET in patients with multiple risk factors for cardiovascular disease has not been established.<br>Trial 1 (NCT03337308) was a 4-arm, 12-week trial that assessed the efficacy of NEXLIZET in 301 patients randomized 2:2:2:1 to receive either oral NEXLIZET (180 mg of bempedoic acid and 10 mg of ezetimibe) (n = 86), bempedoic acid 180 mg (n = 88), ezetimibe 10 mg (n = 86), or placebo (n = 41) once daily as add-on to maximally tolerated statin therapy. Patients were stratified by cardiovascular risk and baseline statin intensity. Patients on simvastatin 40 mg per day or higher and patients taking non-statin lipid-lowering therapy (including fibrates, niacin, bile acid sequestrants, ezetimibe, and PCSK9 inhibitors) were excluded from the trial.<br>Baseline Demographics and Disease Characteristics: Overall, the mean age at baseline was 64 years (range: 30 to 87 years), 50% were 65 years of age and older, 50% were female, 81% were White, 17% were Black or African American, 1% were Asian, and 1% were other races; 12% identified as Hispanic or Latino ethnicity. Sixty-two percent (62%) of patients had clinical CVD and/or a diagnosis of HeFH. The mean baseline LDL-C was 149.7 mg/dL. At the time of randomization, 65% of patients were receiving statin therapy; and 35% were receiving high intensity statin therapy.<br>Bempedoic Acid<br>*Primary Hyperlipidemia*<br>In the two primary hyperlipidemia (52-week) trials (Trials 2 and 3) that included 3,009 adult patients with HeFH or established CVD on maximally tolerated statin therapy, the difference between bempedoic acid and placebo in mean percent change in LDL-C from baseline to Week 12 was -17% to -18%. Bempedoic acid also significantly lowered non-HDL-C (-13%), apo B (-12% to -13%), and TC (-11%) compared with placebo.
020576, 11/06/2018	**Betaine**	Inborn Errors of Metabolism	CBS, MMADHC, MTHFR (Homocystinuria Deficiency)	Indications and Usage, Warnings and Precautions, Clinical Pharmacology, Clinical Studies	**1 INDICATIONS AND USAGE**<br>CYSTADANE® is indicated for the treatment of homocystinuria to decrease elevated homocysteine blood concentrations in pediatric and adult patients. Included within the category of homocystinuria are:<br>• Cystathionine beta-synthase (CBS) deficiency<br>• 5,10-methylenetetrahydrofolate reductase (MTHFR) deficiency<br>• Cobalamin cofactor metabolism (cbl) defect<br><br>**5 WARNINGS AND PRECAUTIONS**<br>**5.1 Hypermethioninemia in Patients with CBS Deficiency**<br>Patients with homocystinuria due to cystathionine beta-synthase (CBS) deficiency may also have elevated plasma methionine concentrations. Treatment with CYSTADANE may further increase methionine concentrations due to the remethylation of homocysteine to methionine. Cerebral edema has been reported in patients with hypermethioninemia, including patients treated with CYSTADANE [see Adverse Reactions (6.2)]. Monitor plasma methionine concentrations in patients with CBS deficiency. Plasma methionine concentrations should be kept below 1,000 micromol/L through dietary modification and, if necessary, a reduction of CYSTADANE dosage.<br><br>**12 CLINICAL PHARMACOLOGY**<br>**12.2 Pharmacodynamics**<br>CYSTADANE was observed to lower plasma homocysteine concentrations in three types of homocystinuria, including CBS deficiency; MTHFR deficiency; and cbl defect. Patients have taken CYSTADANE for many years without evidence of tolerance. There has been no demonstrated correlation between Betaine concentrations and homocysteine concentrations.

* Therapeutic areas do not necessarily reflect the CDER review division.
† Representative biomarkers are listed based on standard nomenclature as per the Human Genome Organization (HUGO) symbol and/or simplified descriptors using other common conventions. Listed biomarkers do not necessarily reflect the terminology used in labeling. The term “Nonspecific” is provided when labeling does not explicitly identify the specific biomarker(s) or when the biomarker is represented by a molecular phenotype or gene signature, and in some cases the biomarker was inferred based on the labeling language.
‡ Referenced figures and tables may be found in the labeling available at Drugs@FDA.
Blue text represents the most recent addtions and/or changes since last posted version.

NDA/ANDA/BLA Number, Label Version Date	Drug	Therapeutic Area*	Biomarker†	Labeling Sections	Labeling Text‡
					In CBS-deficient patients, large increases in methionine concentrations over baseline have been observed. CYSTADANE has also been demonstrated to increase low plasma methionine and S-adenosylmethionine (SAM) concentrations in patients with MTHFR deficiency and cbl defect.<br><br>**14 CLINICAL STUDIES**<br>CYSTADANE was studied in a double-blind, placebo-controlled, crossover study in 6 patients (3 males and 3 females) with CBS deficiency, ages 7 to 32 years at enrollment. CYSTADANE was administered at a dosage of 3 grams twice daily, for 12 months. Plasma homocysteine concentrations were significantly reduced (p<0.01) compared to placebo. Plasma methionine concentrations were variable and not significantly different compared to placebo. CYSTADANE has also been evaluated in observational studies without concurrent controls in patients with homocystinuria due to CBS deficiency, MTHFR deficiency, or cbl defect. A review of 16 case studies and the randomized controlled trial previously described was also conducted, and the data available for each study were summarized; however, no formal statistical analyses were performed. The studies included a total of 78 male and female patients with homocystinuria who were treated with CYSTADANE. This included 48 patients with CBS deficiency, 13 with MTHFR deficiency, and 11 with cbl defect, ranging in age from 24 days to 53 years. (…)
**210498, 10/11/2023**	**Binimetinib (1)**	Oncology	BRAF	Indications and Usage, Dosage and Administration, Warnings and Precautions, Adverse Reactions, Use in Specific Populations, Clinical Studies	**1 INDICATIONS AND USAGE**<br>**1.1 BRAF V600E or V600K Mutation-Positive Unresectable or Metastatic Melanoma**<br>MEKTOVI is indicated, in combination with encorafenib, for the treatment of patients with unresectable or metastatic melanoma with a BRAF V600E or V600K mutation, as detected by an FDA-approved test [see Dosage and Administration (2.1)].<br>**1.2 BRAF V600E Mutation-Positive Metastatic Non-Small Cell Lung Cancer (NSCLC)**<br>MEKTOVI is indicated, in combination with encorafenib, for the treatment of adult patients with metastatic non-small cell lung cancer (NSCLC) with a BRAF V600E mutation, as detected by an FDA-approved test. [see Dosage and Administration (2.1)].<br><br>**2 DOSAGE AND ADMINISTRATION**<br>**2.1 Patient Selection**<br>BRAF V600E or V600K Mutation-Positive Unresectable or Metastatic Melanoma<br>Confirm the presence of a BRAF V600E or V600K mutation in tumor specimens prior to initiating MEKTOVI [see Clinical Studies (14)]. Information on FDA-approved tests for the detection of BRAF V600E and V600K mutations in melanoma is available at: http://www.fda.gov/CompanionDiagnostics.<br>BRAF V600E Mutation-Positive Metastatic Non-Small Cell Lung Cancer (NSCLC)<br>Confirm the presence of a BRAF V600E mutation in tumor or plasma specimens prior to initiating MEKTOVI [see Clinical Studies (14.2)]. If no mutation is detected in a plasma specimen, test tumor tissue. Information on FDA-approved tests for the detection of BRAF V600E mutations in NSCLC is available at: http://www.fda.gov/CompanionDiagnostics.<br><br>**5 WARNINGS AND PRECAUTIONS**<br>**5.3 Ocular Toxicities**<br>*Retinal Vein Occlusion*<br>RVO is a known class-related adverse reaction of MEK inhibitors and may occur in patients treated with MEKTOVI in combination with encorafenib. In patients with BRAF mutation-positive melanoma receiving MEKTOVI with encorafenib (n=690), 1 patient experienced RVO (0.1%). (…)<br>**5.4 Interstitial Lung Disease**<br>In patients with BRAF mutation-positive melanoma receiving MEKTOVI with encorafenib (n=690), 2 patients (0.3%) developed interstitial lung disease (ILD), including pneumonitis.<br>**5.5 Interstitial Lung Disease**<br>In patients with BRAF mutation-positive melanoma receiving MEKTOVI with encorafenib (n=690), 2 patients (0.3%) developed interstitial lung disease (ILD), including pneumonitis. In PHAROS, 1 patient (1%) receiving MEKTOVI with encorafenib developed pneumonitis. Assess new or progressive unexplained pulmonary symptoms or findings for possible ILD. Withhold, reduce dose, or permanently discontinue based on severity of adverse reaction [see Dosage and Administration (2.3), Adverse Reactions (6.1)].<br>**5.6 Rhabdomyolysis**<br>Rhabdomyolysis can occur when MEKTOVI is administered in combination with encorafenib. In COLUMBUS, elevation of laboratory values of serum CPK occurred in 58% of patients treated with MEKTOVI in combination with encorafenib. In patients with BRAF mutation-positive melanoma receiving MEKTOVI with encorafenib (n=690), rhabdomyolysis was reported in 1 patient (0.1%). (…)<br><br>**6 ADVERSE REACTIONS**<br>**6.1 Clinical Trials Experience**<br>(…) The data described in Warnings and Precautions [see Warnings and Precautions (5)] reflect exposure of 192 patients with BRAF V600 mutation-positive unresectable or metastatic melanoma to MEKTOVI (45 mg twice daily) in combination with encorafenib (450 mg once daily) in a randomized open-label, activecontrolled trial (COLUMBUS) or, for rare events, exposure of 690 patients with BRAF V600 mutation- positive melanoma to MEKTOVI (45 mg twice daily) in combination with encorafenib at doses between 300 mg and 600 mg once daily across multiple clinical trials.<br>BRAF V600E or V600K Mutation-Positive Unresectable or Metastatic Melanoma<br>The data described below reflect exposure of 192 patients with BRAF V600 mutation-positive unresectable or metastatic melanoma to MEKTOVI (45 mg twice daily) in combination with encorafenib (450 mg once daily) in COLUMBUS. (…)

* Therapeutic areas do not necessarily reflect the CDER review division.
† Representative biomarkers are listed based on standard nomenclature as per the Human Genome Organization (HUGO) symbol and/or simplified descriptors using other common conventions. Listed biomarkers do not necessarily reflect the terminology used in labeling. The term “Nonspecific” is provided when labeling does not explicitly identify the specific biomarker(s) or when the biomarker is represented by a molecular phenotype or gene signature, and in some cases the biomarker was inferred based on the labeling language.
‡ Referenced figures and tables may be found in the labeling available at Drugs@FDA.
Blue text represents the most recent addtions and/or changes since last posted version.

NDA/ANDA/BLA Number, Label Version Date	Drug	Therapeutic Area*	Biomarker†	Labeling Sections	Labeling Text‡
					<u>BRAF V600E Mutation-Positive Metastatic Non-Small Cell Lung Cancer (NSCLC)</u><br>The safety of MEKTOVI in combination with encorafenib is described in 98 patients with BRAF V600E mutation-positive metastatic NSCLC who received MEKTOVI (45 mg twice daily) in combination with encorafenib (450 mg once daily) in an open-label, single-arm trial (PHAROS). (…)<br><br>**8 USE IN SPECIFIC POPULATIONS**<br>**8.5 Geriatric Use**<br>Of the 690 patients with BRAF mutation-positive melanoma who received MEKTOVI in combination with encorafenib across multiple clinical trials, 20% were aged 65 to 74 years and 8% were aged 75 years and older [see Clinical Pharmacology (12.3)].<br>Of the 98 patients with BRAF V600E mutation-positive metastatic NSCLC who received MEKTOVI in combination with encorafenib, 62 (63.2%) were 65 years of age and over and 20 (20.4%) were 75 years and over [see Clinical Studies (14.2)].<br>No overall differences in the safety or effectiveness of MEKTOVI plus encorafenib were observed in older patients as compared to younger patients.<br><br>**14 CLINICAL STUDIES**<br>**14.1 BRAF V600E or V600K Mutation-Positive Unresectable or Metastatic Melanoma**<br>MEKTOVI in combination with encorafenib was evaluated in a randomized, active-controlled, open-label, multicenter trial (COLUMBUS; NCT01909453). Eligible patients were required to have BRAF V600E or V600K mutation-positive unresectable or metastatic melanoma, as detected using the bioMerieux THxID™BRAF assay. Patients were permitted to have received immunotherapy in the adjuvant setting and one prior line of immunotherapy for unresectable locally advanced or metastatic disease. Prior use of BRAF inhibitors or MEK inhibitors was prohibited. (…)<br>(…) Based on centralized testing, 100% of patients' tumors tested positive for BRAF mutations; BRAF V600E (88%), BRAF V600K (11%), or both (< 1%). (…)<br>**14.2 BRAF V600E Mutation-Positive Metastatic Non-Small Cell Lung Cancer**<br>MEKTOVI in combination with encorafenib was evaluated in an open-label, multicenter, single-arm study in patients with BRAF V600E mutation-positive metastatic non-small cell lung cancer (NSCLC) (PHAROS; NCT03915951). Eligible patients had a diagnosis of histologically-confirmed metastatic NSCLC with BRAF V600E mutation that was treatment-naïve or had been previously treated with 1 prior line of systemic therapy in the metastatic setting (platinum-based chemotherapy and/or anti-PD-1/PD-L1 therapies), age 18 years or older, Eastern Cooperative Oncology Group (ECOG) performance status (PS) of 0 or 1, and measurable disease as defined by Response Evaluation Criteria in Solid Tumors (RECIST) v1.1. Prior use of BRAF inhibitors or MEK inhibitors was not allowed. (…)<br>In the efficacy population, BRAF V600E mutation status was determined by prospective local testing using tumor tissue (78%) or blood (22%) specimens. Of the 98 patients with BRAF V600E mutation, 6 patients were enrolled into the trial based on testing of their tumor tissue specimens with the FoundationOne CDx tissue test. Of the remaining 92 patients enrolled based on local testing, 68 patients had their tumor tissue specimens retrospectively confirmed as having BRAF V600E positive status by the FoundationOne CDx tissue test. The remaining patients had either BRAF V600E negative status (n=5) or had unevaluable results (n=19) by the FoundationOne CDx tissue test. In addition, plasma samples from 81 out of 98 patients were retrospectively tested using the FoundationOne Liquid CDx assay. Of the 81 patients, 48 were confirmed positive for BRAF V600E, while 33 patients were BRAF V600E mutation negative by FoundationOne Liquid CDx assay. The remaining 17 samples had unevaluable results with FoundationOne Liquid CDx assay.<br>Efficacy results for patients with BRAF V600E mutation-positive metastatic NSCLC are summarized in Table 8.
210498, **10/11/2023**	**Binimetinib (2)**	Oncology	UGT1A1	Clinical Pharmacology	**12 CLINICAL PHARMACOLOGY**<br>**12.3 Pharmacokinetics**<br>*Drug Interaction Studies*<br>*Clinical Studies*<br>Effect of UGT1A1 Inducers or Inhibitors on Binimetinib: UGT1A1 genotype and smoking (UGT1A1 inducer) do not have a clinically important effect on binimetinib exposure. Simulations predict similar Cmax of binimetinib 45 mg in the presence or absence of atazanavir 400 mg (UGT1A1 inhibitor).
**125557, 06/14/2024**	**Blinatumomab (1)**	Oncology	BCR-ABL1 (Philadelphia chromosome)	Indications and Usage, Adverse Reactions, Use in Specific Populations, Clinical Pharmacology, Clinical Studies	**1 INDICATIONS AND USAGE**<br>**1. 3 B-cell Precursor ALL in the Consolidation Phase**<br>BLINCYTO is indicated for the treatment of CD19-positive Philadelphia chromosome-negative B-cell precursor acute lymphoblastic leukemia (ALL) in the consolidation phase of multiphase chemotherapy in adult and pediatric patients one month and older.<br><br>**6 ADVERSE REACTIONS**<br>**6.1 Clinical Trials Experience**<br>The safety of BLINCYTO in adult and pediatric patients one month and older with MRD-positive B-cell precursor ALL (n = 137), relapsed or refractory B-cell precursor ALL (n = 267), and Philadelphia chromosome-negative B-cell precursor ALL in consolidation (n = 165) was evaluated in clinical studies. The most common adverse reactions (≥ 20%) to BLINCYTO in this pooled population were pyrexia, infusion-related reactions, headache, infection, musculoskeletal pain, neutropenia, nausea, anemia, thrombocytopenia, and diarrhea. (…)<br><u>Relapsed or Refractory B-cell Precursor ALL</u><br>The safety of BLINCYTO was evaluated in a randomized, open-label, active-controlled clinical study (TOWER Study) in which 376 adult patients with Philadelphia chromosome-negative relapsed or refractory B-cell precursor ALL were treated with BLINCYTO (n = 267) or standard of care (SOC) chemotherapy (n = 109). The median age of BLINCYTO-treated patients was 37 years (range: 18 to 80 years), 60% were male, 84% were White, 7% Asian, 2% were Black or African American, 2% were American Indian or Alaska Native, and 5% were Multiple/Other.<br><u>B-cell Precursor ALL in the Consolidation Phase</u><br>*Study E1910*

* Therapeutic areas do not necessarily reflect the CDER review division.
† Representative biomarkers are listed based on standard nomenclature as per the Human Genome Organization (HUGO) symbol and/or simplified descriptors using other common conventions. Listed biomarkers do not necessarily reflect the terminology used in labeling. The term "Nonspecific" is provided when labeling does not explicitly identify the specific biomarker(s) or when the biomarker is represented by a molecular phenotype or gene signature, and in some cases the biomarker was inferred based on the labeling language.
‡ Referenced figures and tables may be found in the labeling available at Drugs@FDA.
Blue text represents the most recent addtions and/or changes since last posted version.

NDA/ANDA/BLA Number, Label Version Date	Drug	Therapeutic Area*	Biomarker†	Labeling Sections	Labeling Text‡
					The safety of a consolidation regimen comprised of multiple cycles of BLINCYTO monotherapy in addition to multiple cycles of chemotherapy (BLINCYTO arm) was evaluated in a randomized trial in adult patients with newly diagnosed Philadelphia chromosome-negative B-cell precursor ALL (Study E1910) [NCT02003222] [see Clinical Studies (14.3)] which included 111 patients treated in the BLINCYTO arm and 112 patients treated in the chemotherapy alone arm. In the BLINCYTO arm, the median (range) of cycles was 8 (1-8) (4 cycles of BLINCYTO and 4 cycles of chemotherapy). In the chemotherapy alone arm, the median (range) of cycles was 4 (1-4).<br><br>**8 USE IN SPECIFIC POPULATIONS**<br>**8.4 Pediatric Use**<br>B-cell Precursor ALL in the Consolidation Phase<br>The safety and efficacy of BLINCYTO for the treatment of Philadelphia-chromosome negative B-cell precursor ALL in the consolidation phase have been established in pediatric patients one month and older. Use of BLINCYTO for this indication is supported by extrapolation from a randomized controlled study in adults (Study E1910, NCT02003222) and evidence from two randomized, controlled studies in pediatric patients (Study 20120215 and Study AALL1331) [see Adverse Reactions (6.1), Use in Specific Populations (8.4), Clinical Pharmacology (12.3), and Clinical Studies (14.3)].<br>**8.5 Geriatric Use**<br>There were 158 (7%) patients 65 years and older in clinical studies of BLINCYTO for patients with MRD positive, CD19 positive B-cell precursor ALL in first or second complete remission, relapsed or refractory CD19 positive B-cell precursor ALL, and CD19 positive, Philadelphia-chromosome negative B-cell precursor ALL in the consolidation phase. Of the total number of BLINCYTO-treated patients in these studies, 123 (8%) were 65 years of age and older and 21 (1%) were 75 years of age or older. No overall differences in safety or effectiveness were observed between these patients and younger patients, and other reported clinical experience has not identified differences in responses between the elderly and younger patients. However, elderly patients experienced a higher rate of serious infections and neurological toxicities, including cognitive disorder, encephalopathy, and confusion [see Warnings and Precautions (5.2, 5.3)].<br><br>**12 CLINICAL PHARMACOLOGY**<br>**12.3 Pharmacokinetics**<br>Specific Populations<br>There were no clinically meaningful differences in the pharmacokinetics of blinatumomab based on age (0.6 to 80 years of age), sex, race (72% White, 17% Asian, 3% Black), ethnicity, Philadelphia chromosome status or mild (total bilirubin ≤ upper limit of normal [ULN] and AST > ULN or total bilirubin > 1 to 1.5 × ULN and any AST) or moderate hepatic impairment (total bilirubin > 1.5 to 3 × ULN and any AST). The effect of other races or severe hepatic impairment (total bilirubin > 3 × ULN, any AST) on the pharmacokinetics of blinatumomab is unknown. Body surface area (0.4 to 2.9 m2) influences the pharmacokinetics of blinatumomab, supporting BSA-based dosing in patients < 45 kg.<br><br>**14 CLINICAL STUDIES**<br>**14.1 MRD-positive B-cell Precursor ALL**<br>*BLAST Study*<br>The efficacy of BLINCYTO was evaluated in an open-label, multicenter, single-arm study (BLAST Study) [NCT01207388] that included patients who were ≥ 18 years of age, had received at least 3 chemotherapy blocks of standard ALL therapy, were in hematologic complete remission (defined as < 5% blasts in bone marrow, absolute neutrophil count > 1 Gi/L, platelets > 100 Gi/L) and had MRD at a level of ≥ 0.1% using an assay with a minimum sensitivity of 0.01%. (…) (See Table 13) (…)<br>**14.2 Relapsed/Refractory B-cell Precursor ALL**<br>TOWER Study The efficacy of BLINCYTO was compared to standard of care (SOC) chemotherapy in a randomized, open-label, multicenter study (TOWER Study) [NCT02013167]. Eligible patients were ≥ 18 years of age with relapsed or refractory B-cell precursor ALL [> 5% blasts in the bone marrow and refractory to primary induction therapy or refractory to last therapy, untreated first relapse with first remission duration < 12 months, untreated second or later relapse, or relapse at any time after allogeneic hematopoietic stem cell transplantation (alloHSCT)]. (See Table 16) (…)<br>**14.2 Relapsed/Refractory B-cell Precursor ALL**<br>*Study MT103-211*<br>Study MT103-211 [NCT01466179] was an open-label, multicenter, single-arm study. Eligible patients were ≥ 18 years of age with Philadelphia chromosome-negative relapsed or refractory B-cell precursor ALL (relapsed with first remission duration of ≤ 12 months in first salvage or relapsed or refractory after first salvage therapy or relapsed within 12 months of alloHSCT and had ≥ 10% blasts in bone marrow). (See Table 17) (…)<br>*ALCANTARA Study*<br>The efficacy of BLINCYTO for treatment of Philadelphia chromosome-positive B-cell precursor ALL was evaluated in an open-label, multicenter, single-arm study (ALCANTARA Study) [NCT02000427]. Eligible patients were ≥ 18 years of age with Philadelphia chromosome-positive B-cell precursor ALL, relapsed or refractory to at least 1 second generation or later tyrosine kinase inhibitor (TKI), or intolerant to second generation TKI, and intolerant or refractory to imatinib mesylate. (See Table 19) (…)<br>**14.3 Philadelphia Chromosome-Negative B-cell Precursor ALL in the Consolidation Phase**<br>Study E1910<br>The efficacy of BLINCYTO was evaluated in a randomized, controlled study in adult patients with newly diagnosed Philadelphia chromosome-negative B-cell precursor ALL (Study E1910) [NCT02003222]. Eligible patients in hematologic complete remission (CR) or CR with incomplete peripheral blood count recovery (CRi) following induction and intensification chemotherapy were randomized 1:1 to receive a consolidation regimen comprised of multiple cycles of BLINCYTO monotherapy in addition to multiple cycles of intensive chemotherapy (BLINCYTO arm) or to intensive chemotherapy alone (chemotherapy arm). Randomization was stratified by age (< 55 years versus ≥ 55 years), CD20 status, rituximab use, and intent to undergo allogeneic stem cell transplantation (HSCT).<br>Study 20120215

* Therapeutic areas do not necessarily reflect the CDER review division.
† Representative biomarkers are listed based on standard nomenclature as per the Human Genome Organization (HUGO) symbol and/or simplified descriptors using other common conventions. Listed biomarkers do not necessarily reflect the terminology used in labeling. The term “Nonspecific” is provided when labeling does not explicitly identify the specific biomarker(s) or when the biomarker is represented by a molecular phenotype or gene signature, and in some cases the biomarker was inferred based on the labeling language.
‡ Referenced figures and tables may be found in the labeling available at Drugs@FDA.
Blue text represents the most recent addtions and/or changes since last posted version.

Table of Pharmacogenomic Biomarkers in Drug Labeling
Last Updated: 06/2024

NDA/ANDA/BLA Number, Label Version Date	Drug	Therapeutic Area*	Biomarker†	Labeling Sections	Labeling Text‡
					The efficacy of BLINCYTO compared to consolidation chemotherapy was evaluated in a randomized, controlled, open-label, multicenter study (Study 20120215) [NCT02393859]. Eligible patients were 28 days to 18 years old and had high-risk, first-relapsed, Philadelphia chromosome-negative B-cell precursor ALL with < 25% blasts in the bone marrow after induction and 2 cycles of consolidation chemotherapy. Patients were randomized 1:1 to receive BLINCYTO or the IntReALLHR2010 HC3 intensive combination chemotherapy as the third cycle of consolidation. Patients in the BLINCYTO arm received one cycle of BLINCYTO as a continuous intravenous infusion at 15 mcg/m2/day over 4 weeks (maximum daily dose was not to exceed 28 mcg/day). Randomization was stratified by age, minimal residual disease status determined at the end of induction based on local assessment, and bone marrow status determined at the end of the second block of consolidation chemotherapy. Patients were to proceed to HSCT after this cycle of consolidation.<br>There were 54 patients randomized to the BLINCYTO arm and 57 to the chemotherapy arm. The demographics and baseline characteristics are shown in Table 23.
**125557, 06/14/2024**	**Blinatumomab (2)**	Oncology	CD19	Indications and Usage, Use in Specific Populations	**1 INDICATIONS AND USAGE**<br>**1.1 MRD-positive B-cell Precursor ALL**<br>BLINCYTO is indicated for the treatment of CD19-positive B-cell precursor acute lymphoblastic leukemia (ALL) in first or second complete remission with minimal residual disease (MRD) greater than or equal to 0.1% in adult and pediatric patients one month and older.<br>**1.2 Relapsed or Refractory B-cell Precursor ALL**<br>BLINCYTO is indicated for the treatment of relapsed or refractory CD19-positive B-cell precursor acute lymphoblastic leukemia (ALL) in adult and pediatric patients one month and older.<br>**1. 3 B-cell Precursor ALL in the Consolidation Phase**<br>BLINCYTO is indicated for the treatment of CD19-positive Philadelphia chromosome-negative B-cell precursor acute lymphoblastic leukemia (ALL) in the consolidation phase of multiphase chemotherapy in adult and pediatric patients one month and older.<br><br>**8 USE IN SPECIFIC POPULATIONS**<br>**8.4 Pediatric Use**<br>The safety and efficacy of BLINCYTO in pediatric patients less than 1 month of age have not been established for any indication [see Indications and Usage (1)].<br>Minimal Residual Disease (MRD)-Positive B-cell Precursor ALL<br>The safety and efficacy of BLINCYTO for the treatment of CD19-positive B-cell precursor acute lymphoblastic leukemia (ALL) in first or second complete remission with minimal residual disease (MRD) greater than or equal to 0.1% have been established in pediatric patients one month and older. Use of BLINCYTO is supported by evidence from two randomized, controlled trials (Study AALL1331, NCT02101853 and Study 20120215, NCT02393859) [see Clinical Studies (14.3)] in pediatric patients with first relapsed B-cell precursor ALL. Both studies included pediatric patients with MRD-positive B-cell precursor ALL. The studies included pediatric patients treated with BLINCYTO in the following age groups: 6 infants (1 month up to less than 2 years), 165 children (2 years up to less than 12 years), and 70 adolescents (12 years to less than 17 years). In general, the adverse reactions in BLINCYTO-treated pediatric patients were similar in type to those seen in adult patients with MRD-positive ALL [see Adverse Reactions (6.1)], and no differences in safety were observed between the different pediatric age subgroups.<br>**8.5 Geriatric Use**<br>There were 158 (7%) patients 65 years and older in clinical studies of BLINCYTO for patients with MRD positive, CD19 positive B-cell precursor ALL in first or second complete remission, relapsed or refractory CD19 positive B-cell precursor ALL, and CD19 positive, Philadelphia-chromosome negative B-cell precursor ALL in the consolidation phase. Of the total number of BLINCYTO-treated patients in these studies, 123 (8%) were 65 years of age and older and 21 (1%) were 75 years of age or older. No overall differences in safety or effectiveness were observed between these patients and younger patients, and other reported clinical experience has not identified differences in responses between the elderly and younger patients. However, elderly patients experienced a higher rate of serious infections and neurological toxicities, including cognitive disorder, encephalopathy, and confusion [see Warnings and Precautions (5.2, 5.3)].
202258, **01/30/2017**	**Boceprevir**	Infectious Diseases	IFNL3 (IL28B)	Clinical Pharmacology	**12 CLINICAL PHARMACOLOGY**<br>**12.5 Pharmacogenomics**<br>A genetic variant near the gene encoding interferon-lambda-3 (IL28B rs12979860, a C to T change) is a strong predictor of response to PegIntron/REBETOL. IL28B rs12979860 was genotyped in 653 of 1048 (62%) subjects in SPRINT-2 (previously untreated) and 259 of 394 (66%) subjects in RESPOND-2 (previous partial responders and relapsers) [see Clinical Studies (14) for trial descriptions]. Among subjects that received at least one dose of placebo or VICTRELIS (Modified-Intent-to-Treat population), SVR rates tended to be lower in subjects with the C/T and T/T genotypes compared to those with the C/C genotype, particularly among previously untreated subjects receiving 48 weeks of PegIntron and REBETOL (see Table 9). Among previous treatment failures, subjects of all genotypes appeared to have higher SVR rates with regimens containing VICTRELIS. The results of this retrospective subgroup analysis should be viewed with caution because of the small sample size and potential differences in demographic or clinical characteristics of the substudy population relative to the overall trial population. (See Table 9)
**203341, 09/26/2023**	**Bosutinib**	Oncology	BCR-ABL1 (Philadelphia chromosome)	Indications and Usage, Dosage and Administration, Warnings and Precautions, Adverse Reactions, Use in Specific	**1 INDICATIONS AND USAGE**<br>BOSULIF is indicated for the treatment of:<br>• Adult and pediatric patients 1 year of age and older with chronic phase (CP) Philadelphia chromosome-positive chronic myelogenous leukemia (Ph+ CML), newly-diagnosed or resistant or intolerant to prior therapy [see Clinical Studies (14.1, 14.2, 14.3)].<br>• Adult patients with accelerated phase (AP), or blast phase (BP) Ph+ CML with resistance or intolerance to prior therapy [see Clinical Studies (14.2)]..<br><br>**2 DOSAGE AND ADMINISTRATION**<br>**2.1 Recommended Dosing**<br>Dosage in Adult Patients with Newly-Diagnosed CP Ph+ CML<br>The recommended dosage of BOSULIF is 400 mg orally once daily with food.

* Therapeutic areas do not necessarily reflect the CDER review division.
† Representative biomarkers are listed based on standard nomenclature as per the Human Genome Organization (HUGO) symbol and/or simplified descriptors using other common conventions. Listed biomarkers do not necessarily reflect the terminology used in labeling. The term "Nonspecific" is provided when labeling does not explicitly identify the specific biomarker(s) or when the biomarker is represented by a molecular phenotype or gene signature, and in some cases the biomarker was inferred based on the labeling language.
‡ Referenced figures and tables may be found in the labeling available at Drugs@FDA.
Blue text represents the most recent addtions and/or changes since last posted version.

NDA/ANDA/BLA Number, Label Version Date	Drug	Therapeutic Area*	Biomarker†	Labeling Sections	Labeling Text‡
				Populations, Clinical Studies	<u>Dosage in Adult Patients with CP, AP, or BP Ph+ CML with Resistance or Intolerance to Prior Therapy</u><br>The recommended dosage of BOSULIF is 500 mg orally once daily with food.<br><u>Dosage in Pediatric Patients with Newly-Diagnosed CP Ph+ CML or with CP Ph+ CML with Resistance or Intolerance to Prior Therapy</u><br>The recommended dose of BOSULIF for pediatric patients with newly-diagnosed CP Ph+ CML is 300 mg/m2 orally once daily with food and the recommended dosage for pediatric patients with CP Ph+ CML that is resistant or intolerant to prior therapy is 400 mg/m2 orally once daily with food and dose recommendations are provided in Table 1. As appropriate, the desired dose can be attained by combining different strengths of BOSULIF tablets or capsules. (See Table 1)<br>**2.2 Dose Escalation**<br>In clinical studies of adult patients with Ph+ CML, dose escalation by increments of 100 mg once daily to a maximum of 600 mg once daily was allowed in patients who did not achieve or maintain a hematologic, cytogenetic, or molecular response and who did not have Grade 3 or higher adverse reactions at the recommended starting dosage.<br>In pediatric patients with BSA <1.1 m2 and an insufficient response after 3 months consider increasing dose by 50 mg increments up to maximum of 100 mg above starting dose. Dose increases for insufficient response in pediatric patients with BSA ≥1.1 m2 can be conducted similarly to adult recommendations in 100 mg increments.<br>The maximum dose in pediatric and adult patients is 600 mg once daily.<br>**2.5 Dose Adjustments for Renal Impairment or Hepatic Impairment**<br>The recommended starting doses for patients with renal and hepatic impairment are described in Table 4 below. (See Table 4)<br><br>**5 WARNINGS AND PRECAUTIONS**<br>**5.1 Gastrointestinal Toxicity**<br>Diarrhea, nausea, vomiting, and abdominal pain occur with BOSULIF treatment. Monitor and manage patients using standards of care, including antidiarrheals, antiemetics, and fluid replacement. In the randomized clinical trial in patients with newly-diagnosed Ph+ CML, the median time to onset for diarrhea (all grades) was 3 days and the median duration per event was 3 days.<br>Among 49 pediatric patients with newly-diagnosed CP Ph+ CML or who had CP Ph+ CML that was resistant or intolerant to prior therapy, the median time to onset for diarrhea (all grades) was 2 days and the duration was 2 days. Among patients who experienced diarrhea, the median number of episodes of diarrhea per patient during treatment with BOSULIF was 2 (range 1 – 198). (…)<br>**5.3 Hepatic Toxicity**<br>**(…)** Among 49 pediatric patients with newly-diagnosed CP Ph+ CML or who had CP Ph+ CML that was resistant or intolerant to prior therapy, the incidence based on laboratory data that worsened from baseline of increased ALT was 59% and of increased AST 51%. Seventy-six percent of the patients experienced an increase in either ALT or AST. Most cases of increased transaminases occurred early in treatment; of patients who experienced increased transaminases of any grade, 84% of patients experienced their first increases within the first 3 months. The median time to onset for adverse reactions of increased ALT and AST was 22 and 15 days, respectively. The median duration for adverse reactions of Grade 3 or 4 increased ALT or AST was 26 and 12 days, respectively.<br>**5.4 Cardiovascular Toxicity**<br>(…) Among 49 pediatric patients with newly diagnosed CP Ph+ CML or who had CP Ph+ CML that was resistant or intolerant to prior therapy, 4 (8%) patients had Grade 1-2 cardiac events, including tachycardia (n=2), angina pectoris, right bundle branch block, and sinus tachycardia (n=1 each).<br>**5.4 Fluid Retention**<br>Among 546 adult patients in a single-arm study in patients with Ph+ CML who were resistant or intolerant to prior therapy, Grade 3 or 4 fluid retention was reported in 30 patients (6%). Some patients experienced more than one fluid retention event. Specifically, 24 patients experienced Grade 3 or 4 pleural effusions, 9 patients experienced Grade 3 or Grade 4 pericardial effusions, and 6 patients experienced Grade 3 edema. Among 49 pediatric patients with newly diagnosed CP Ph+ CML or who had CP Ph+ CML that was resistant or intolerant to prior therapy, Grade 1-2 pericardial effusion, peripheral edema, and face edema were reported in 1 patient each.<br>**5.6 Renal Toxicity**<br>Overall, 45% of the pediatric patients with newly diagnosed CP Ph+ CML or resistant or intolerant CP Ph+ CML who had normal eGFR at baseline shifted to a maximum of mild, and 40% pediatric patients who had mild eGFR at baseline shifted to a maximum of moderate during treatment.<br><br>**6 ADVERSE REACTIONS**<br>**6.1 Clinical Trials Experience**<br>The most common adverse reactions, in ≥20% of adults with newly diagnosed CP Ph+ CML or CP, AP, or BP Ph+ CML with resistance or intolerance to prior therapy (N=814) were diarrhea (80%), rash (44%), nausea (44%), abdominal pain (43%), vomiting (33%), fatigue (33%), hepatic dysfunction (33%), respiratory tract infection (25%), pyrexia (24%), and headache (21%). (…)<br><br>**8 USE IN SPECIFIC POPULATIONS**<br>**8.4 Pediatric Use**<br>The safety and effectiveness of BOSULIF have been established in pediatric patients 1 year of age and older with newly-diagnosed CP Ph+ CML and CP Ph+ CML that is resistant or intolerant to prior therapy.<br>Use of BOSULIF for these indications is based on data from BCHILD [NCT04258943]. The study included pediatric patients with newly diagnosed CP Ph+ CML in the following age groups: 2 patients 1 year of age to less than 6 years of age, 3 patients 6 years of age to less than 12 years of age, and 10 patients 12 years of age to less than 17 years of age. The study also included pediatric patients with CP Ph+ CML that was resistant or intolerant to prior therapy in the following age groups: 4 patients 1 year of age to less than 6 years of age, 10 patients 6 years of age to less than 12 years of age, and 10 patients 12 years of age to less than 17 years of age. [see Adverse Reactions (6.1) and Clinical Studies (14.3]. BSA-normalized apparent clearance in 27 pediatric patients aged 4 to <17 years

* Therapeutic areas do not necessarily reflect the CDER review division.
† Representative biomarkers are listed based on standard nomenclature as per the Human Genome Organization (HUGO) symbol and/or simplified descriptors using other common conventions. Listed biomarkers do not necessarily reflect the terminology used in labeling. The term “Nonspecific” is provided when labeling does not explicitly identify the specific biomarker(s) or when the biomarker is represented by a molecular phenotype or gene signature, and in some cases the biomarker was inferred based on the labeling language.
‡ Referenced figures and tables may be found in the labeling available at Drugs@FDA.
Blue text represents the most recent addtions and/or changes since last posted version.

NDA/ANDA/BLA Number, Label Version Date	Drug	Therapeutic Area*	Biomarker†	Labeling Sections	Labeling Text‡
					(141.3 L/h/m2) was 29% higher than BSA-normalized apparent clearance in adult patients with CP Ph+ CML (109.2 L/h/m2) [see Clinical Pharmacology (12.3)]. The recommended dosage of BOSULIF in pediatric patients is based on body-surface area (BSA) [see Dosage and Administration (2.1)].<br>The safety and effectiveness of BOSULIF in pediatric patients younger than 1 year of age with newly diagnosed CP Ph+ CML, pediatric patients younger than 1 year of age with CP Ph+ CML that is resistant or intolerant to prior therapy, and pediatric patients with AP Ph+ CML or BP Ph+ CML have not been established.<br>**8.5 Geriatric Use**<br>In the Phase 1/2 clinical trial of BOSULIF in patients with Ph+ CML, 20% were age 65 and over, 4% were 75 and over. No overall differences in safety or effectiveness were observed between these patients and younger patients, and other reported clinical experience has not identified differences in responses between the elderly and younger patients, but greater sensitivity of some older individuals cannot be ruled out.<br><br>**14 CLINICAL STUDIES**<br>**14.1 Adult Patients with Newly-Diagnosed CP Ph+ CML**<br>The efficacy of BOSULIF in patients with newly-diagnosed chronic phase Ph+ CML was evaluated in the Bosutinib trial in First-line chrOnic myelogenous leukemia tREatment (BFORE) Trial: “A Multicenter Phase 3, Open-Label Study of Bosutinib Versus Imatinib in Adult Patients With Newly Diagnosed Chronic Phase Chronic Myelogenous Leukemia” [NCT02130557].<br>The BFORE Trial is a 2-arm, open-label, randomized, multicenter trial conducted to investigate the efficacy and safety of BOSULIF 400 mg once daily alone compared with imatinib 400 mg once daily alone in adult patients with newly-diagnosed CP Ph+ CML. The trial randomized 536 patients (268 in each arm) with Ph+ or Ph- newly-diagnosed CP CML (intent-to-treat [ITT] population) including 487 patients with Ph+ CML harboring b2a2 and/or b3a2 transcripts at baseline and baseline BCR-ABL copies >0 (modified intent-to-treat [mITT] population). (…)<br>**14.2 Adult Patients with Imatinib-Resistant or -Intolerant Ph+ CP, AP, and BP CML**<br>Study 200 (NCT00261846), a single-arm, open-label, multicenter study in patients with CML who were resistant or intolerant to prior therapy was conducted to evaluate the efficacy and safety of BOSULIF 500 mg once daily in patients with imatinib-resistant or -intolerant CML with separate cohorts for CP, AP, and BP disease previously treated with 1 prior TKI (imatinib) or more than 1 TKI (imatinib followed by dasatinib and/or nilotinib). The definition of imatinib resistance included (1) failure to achieve or maintain any hematologic improvement within 4 weeks; (2) failure to achieve a CHR by 3 months, cytogenetic response by 6 months or major cytogenetic response (MCyR) by 12 months; (3) progression of disease after a previous cytogenetic or hematologic response; or (4) presence of a genetic mutation in the BCR-ABL gene associated with imatinib resistance. Imatinib intolerance was defined as inability to tolerate imatinib due to toxicity, or progression on imatinib and inability to receive a higher dose due to toxicity. (See Table 14) (…)<br>**14.3 Pediatric Patients with Newly-Diagnosed CP Ph+ CML or with CP Ph+ CML with Resistance or Intolerance to Prior Therapy**<br>The efficacy of BOSULIF in pediatric patients with newly-diagnosed (ND) chronic phase (CP) Ph+ CML and patients with resistant/intolerant (R/I) CP Ph+ CML was evaluated in the BCHILD trial [NCT04258943]. The BCHILD trial is a multicenter, non-randomized, open-label study conducted to identify a recommended dose of bosutinib administered orally once daily in pediatric patients with ND CP Ph+ CML and pediatric patients with R/I CP Ph+ CML who have received at least one prior TKI therapy, to estimate the safety and tolerability and efficacy, and to evaluate the PK of bosutinib in this patient population. The study enrolled 28 patients with R/I CP Ph+ CML treated with BOSULIF at 300 mg/m2 to 400 mg/m2 orally once daily, and 21 patients with ND CP Ph+ CML treated at 300 mg/m2 orally once daily. Efficacy outcomes included CCyR (defined as the absence of Ph+ metaphases in chromosome banding analysis of ≥20 metaphases, or <1 % BCR-ABL1–positive nuclei of at least 200 peripheral blood interphase nuclei analyzed by Fluorescence In Situ Hybridization (FISH), or MMR if an adequate cytogenetic assessment was unavailable), MCyR (defined as CCyR or partial cytogenetic response of 1% to 35% Ph+ metaphases), and MMR (defined as ≤0.1% BCR-ABL ratio on international scale [IS]) at any time on study.<br>Patients with ND CP Ph+ CML had a median age of 14 years (range 5 to 17 years); 68% were male; 81% were White, 14% were Black/African American, and 5% were race not reported.<br>The major (MCyR) and complete (CCyR) cytogenetic responses among patients with ND CP Ph+ CML were 76.2% (95% CI: 52.8, 91.8) and 71.4% (95% CI: 47.8, 88.7), respectively. The MMR among patients with ND CP Ph+ CML was 28.6% (95% CI: 11.3, 52.3). The median duration of follow-up was 14.2 months (range: 1.1, 26.3 months) in patients with ND CP CML. Patients with R/I CP Ph+ CML included n=6 treated at 300 mg/m2 (0.75 times the recommended dose), n=11 treated at 350 mg/m2 (0.875 times the recommended dose), and n=11 at 400 mg/m2 . Overall (n=28), patients had a median age of 11.5 years (range: 1 to 17 years); 57% were male; 43% were White, 7% were Black/African American, 14% were Asian, and 36% were race not reported.<br>The major (MCyR) and complete (CCyR) cytogenetic responses among patients with R/I CP Ph+ CML were 82.1% (95% CI: 63.1, 93.9) and 78.6% (95% CI: 59.0, 91.7), respectively. The MMR among patients with R/I CP Ph+ CML was 50.0% (95% CI: 30.6, 69.4). The MR4.5 (defined as BCR-ABL/ABL IS ≤ 0.0032%) was 17.9% (95% CI: 6.1, 36.9). Among 14 patients who achieved MMR, two patients lost MMR after 13.6 months and 24.7 months on treatment. The median duration of follow-up for overall survival was 23.2 months (range: 1.0, 61.5 months) in patients with R/I CP Ph+ CML.
125388, 11/10/2022	Brentuximab Vedotin (1)	Oncology	ALK	Use in Specific Populations, Clinical Studies	**8 USE IN SPECIFIC POPULATIONS**<br>**8.4 Pediatric Use**<br>Newly Diagnosed ALK+ ALCL<br>The safety and effectiveness of ADCETRIS in combination with alternating chemotherapy Courses A (dexamethasone, ifosfamide, methotrexate, etoposide, cytarabine) and B (dexamethasone, methotrexate, cyclophosphamide, doxorubicin) administered every 21 days for a total of 6 cycles was assessed but have not been established based on a study (NCT01979536) in 67 patients, which included 61 pediatric patients age 2 to less than 17 with newly diagnosed ALK+ ALCL. No new safety signals were identified in this study.<br><br>**14 CLINICAL STUDIES**<br>**14.2 Systemic Anaplastic Large Cell Lymphoma and Other CD30-Expressing Peripheral T-Cell Lymphomas**<br>**Randomized Clinical Trial in Previously Untreated Systemic Anaplastic Large Cell Lymphoma or Other CD30-Expressing Peripheral T-Cell Lymphomas (Study 6: ECHELON-2, NCT01777152)**

NDA/ANDA/BLA Number, Label Version Date	Drug	Therapeutic Area*	Biomarker†	Labeling Sections	Labeling Text‡
					The median age was 58 years (range: 18 to 85), 63% were male, 62% were White, 22% were Asian, and 78% had an ECOG performance status of 0-1. Of the 452 patients enrolled, the disease subtypes included patients with systemic ALCL [70%; 48% anaplastic lymphoma kinase (ALK) negative and 22% ALK positive], PTCL not otherwise specified (16%), angioimmunoblastic T-cell lymphoma (12%), adult T-cell leukemia/lymphoma (2%), and enteropathy-associated T-cell lymphoma (<1%). Most patients had Stage III or IV disease (81%) and a baseline international prognostic index of 2 or 3 (63%). (…)<br>**14.3 Systemic Anaplastic Large Cell Lymphoma**<br>*Clinical Trial in Relapsed sALCL (Study 2)*<br>(…) The 58 patients ranged in age from 14–76 years (median, 52 years) and most were male (57%) and white (83%). Patients had received a median of 2 prior therapies; 26% of patients had received prior autologous hematopoietic stem cell transplantation. Fifty percent (50%) of patients were relapsed and 50% of patients were refractory to their most recent prior therapy. Seventy-two percent (72%) were anaplastic lymphoma kinase (ALK)-negative. (…)
125388, 11/10/2022	**Brentuximab Vedotin (2)**	Oncology	TNFRSF8 (CD30)	Indications and Usage, Dosage and Administration, Adverse Reactions, Use in Specific Populations, Clinical Studies	**1 INDICATIONS AND USAGE**<br>**1.5 Previously untreated systemic anaplastic large cell lymphoma (sALCL) or other CD30-expressing peripheral T-cell lymphomas (PTCL), in combination with chemotherapy**<br>ADCETRIS is indicated for the treatment of adult patients with previously untreated sALCL or other CD30-expressing PTCL, including angioimmunoblastic T-cell lymphoma and PTCL not otherwise specified, in combination with cyclophosphamide, doxorubicin, and prednisone.<br>**1.7 Relapsed primary cutaneous anaplastic large cell lymphoma (pcALCL) or CD30- expressing mycosis fungoides (MF)**<br>ADCETRIS is indicated for the treatment of adult patients with pcALCL or CD30-expressing MF who have received prior systemic therapy.<br><br>**2 DOSAGE AND ADMINISTRATION**<br>**2.1 Recommended Dosage**<br>For dosing instructions of combination agents administered with ADCETRIS, see Clinical Studies (14.1 and 14.2) and the manufacturer's prescribing information. (See Table 1)<br><br>**6 ADVERSE REACTIONS**<br>**6.1 Clinical Trial Experience**<br>(…) Data summarizing ADCETRIS exposure are also provided for 347 patients with T-cell lymphoma, including 223 patients with PTCL who received ADCETRIS in combination with chemotherapy in a randomized, double-blind, controlled trial; 58 patients with sALCL who received ADCETRIS monotherapy in a single-arm trial; and 66 patients with pcALCL or CD30-expressing MF who received ADCETRIS monotherapy in a randomized, controlled trial. (…)<br>*Previously Untreated Systemic Anaplastic Large Cell Lymphoma or Other CD30- Expressing Peripheral T-Cell Lymphomas (Study 6, ECHELON-2)*<br>ADCETRIS in combination with CHP was evaluated in patients with previously untreated, CD30- expressing PTCL in a multicenter randomized, double-blind, double dummy, actively controlled trial. (See Table 7) (…)<br>*Primary Cutaneous Anaplastic Large Cell Lymphoma and CD30-Expressing Mycosis Fungoides (Study 4: ALCANZA)*<br>ADCETRIS was studied in 131 patients with pcALCL or CD30-expressing MF requiring systemic therapy in a randomized, open-label, multicenter clinical trial in which the recommended starting dose and schedule was ADCETRIS 1.8 mg/kg intravenously over 30 minutes every 3 weeks or physician's choice of either methotrexate 5 to 50 mg orally weekly or bexarotene 300 mg/m2 orally daily. (See Table 10) (…)<br><br>**8 USE IN SPECIFIC POPULATIONS**<br>**8.5 Geriatric Use**<br>(…) In the clinical trial of ADCETRIS in combination with CHP for patients with previously untreated, CD30-expressing PTCL (Study 6: ECHELON-2), 31% of ADCETRIS + CHP-treated patients were age 65 or older. (…)<br>(…) In the clinical trial of ADCETRIS in pcALCL or CD30-expressing MF (Study 4: ALCANZA), 42% of ADCETRIS-treated patients were age 65 or older. No meaningful differences in safety or efficacy were observed between these patients and younger patients.<br><br>**14 CLINICAL STUDIES**<br>**14.2 Systemic Anaplastic Large Cell Lymphoma and Other CD30-Expressing Peripheral T-Cell Lymphomas**<br>*Randomized Clinical Trial in Previously Untreated Systemic Anaplastic Large Cell Lymphoma or Other CD30-Expressing Peripheral T-Cell Lymphomas (Study 6: ECHELON-2, NCT01777152)*<br>The efficacy of ADCETRIS in combination with chemotherapy for the treatment of adult patients with previously untreated, CD30-expressing PTCL was evaluated in a multicenter, randomized, double-blind, double-dummy, actively controlled trial. For enrollment, the trial required CD30 expression ≥10% per immunohistochemistry. The trial excluded patients with primary cutaneous CD30-positive T-cell lymphoproliferative disorders and lymphomas. (See Table 13) (…)<br>**14.4 Primary Cutaneous Anaplastic Large Cell Lymphoma and CD30-Expressing Mycosis Fungoides**<br>*Randomized Clinical Trial in Primary Cutaneous Anaplastic Large Cell Lymphoma and CD30-expressing Mycosis Fungoides (Study 4: ALCANZA, NCT01578499)*<br>The efficacy of ADCETRIS in patients with primary cutaneous anaplastic large cell lymphoma (pcALCL) or mycosis fungoides (MF) requiring systemic therapy was studied in ALCANZA, a randomized, open-label, multicenter clinical trial. (…)<br>(…) Patients with pcALCL must have received prior radiation or systemic therapy, and must have at least 1 biopsy with CD30-expression of ≥10%. Patients with MF must have received prior systemic therapy and have had skin biopsies from at least 2 separate lesions, with CD30- expression of ≥10% in at least 1 biopsy.

* Therapeutic areas do not necessarily reflect the CDER review division.
† Representative biomarkers are listed based on standard nomenclature as per the Human Genome Organization (HUGO) symbol and/or simplified descriptors using other common conventions. Listed biomarkers do not necessarily reflect the terminology used in labeling. The term "Nonspecific" is provided when labeling does not explicitly identify the specific biomarker(s) or when the biomarker is represented by a molecular phenotype or gene signature, and in some cases the biomarker was inferred based on the labeling language.
‡ Referenced figures and tables may be found in the labeling available at Drugs@FDA.
Blue text represents the most recent addtions and/or changes since last posted version.

NDA/ANDA/BLA Number, Label Version Date	Drug	Therapeutic Area*	Biomarker†	Labeling Sections	Labeling Text‡
					A total of 131 patients were randomized (66 ADCETRIS, 65 physician's choice). The efficacy results were based on 128 patients (64 patients in each arm with CD30-expression of ≥10% in at least one biopsy). (See Table 17) (...)<br>(…) Supportive trials include 2 single-arm trials, which enrolled patients with MF who were treated with ADCETRIS 1.8 mg/kg intravenously over 30 minutes every 3 weeks. Out of 73 patients with MF from the 2 pooled supportive trials, 34% (25/73) achieved ORR4. Among these 73 patients, 35 had 1% to 9% CD30-expression and 31% (11/35) achieved ORR4.
205422, **02/09/2018**	Brexpiprazole	Psychiatry	CYP2D6	Dosage and Administration, Use in Specific Populations, Clinical Pharmacology	**2 DOSAGE AND ADMINISTRATION**<br>**2.5 Dosage Modifications for CYP2D6 Poor Metabolizers and for Concomitant use with CYP Inhibitors or Inducers**<br>Dosage adjustments are recommended in patients who are known cytochrome P450 (CYP) 2D6 poor metabolizers and in patients taking concomitant CYP3A4 inhibitors or CYP2D6 inhibitors or strong CYP3A4 inducers (see Table 1). If the coadministered drug is discontinued, adjust the REXULTI dosage to its original level. If the coadministered CYP3A4 inducer is discontinued, reduce the REXULTI dosage to the original level over 1 to 2 weeks [see Drug Interactions (7.1), Clinical Pharmacology (12.3)]. (See Table 1)<br><br>**8 USE IN SPECIFIC POPULATIONS**<br>**8.6 CYP2D6 Poor Metabolizers**<br>Dosage adjustment is recommended in known CYP2D6 poor metabolizers, because these patients have higher brexpiprazole concentrations than normal metabolizers of CYP2D6. Approximately 8% of Caucasians and 3–8% of Black/African Americans cannot metabolize CYP2D6 substrates and are classified as poor metabolizers (PM) [see Dosage and Administration (2.5), Clinical Pharmacology (12.3)].<br><br>**12 CLINICAL PHARMACOLOGY**<br>**12.3 Pharmacokinetics**<br>*Drug Interaction Studies*<br>Effects of other drugs on the exposures of brexpiprazole are summarized in Figure 2. Based on simulation, a 5.1-fold increase in AUC values at steady-state is expected when extensive metabolizers of CYP2D6 are administered with both strong CYP2D6 and CYP3A4 inhibitors. A 4.8-fold increase in mean AUC values at steady-state is expected in poor metabolizers of CYP2D6 administered with strong CYP3A4 inhibitors [see Drug Interactions (7.1)].
208772, **05/22/2020**	Brigatinib	Oncology	ALK	Indications and Usage, Dosage and Administration, Adverse Reactions, Clinical Studies	**1 INDICATIONS AND USAGE**<br>ALUNBRIG is indicated for the treatment of adult patients with anaplastic lymphoma kinase (ALK)- positive metastatic non-small cell lung cancer (NSCLC) as detected by an FDA-approved test [see Dosage and Administration (2.1)].<br><br>**2 DOSAGE AND ADMINISTRATION**<br>**2.1 Patient Selection**<br>Select patients for the treatment of metastatic NSCLC with ALUNBRIG based on the presence of ALK positivity in tumor specimens [see Clinical Studies (14)]. Information on FDA-approved tests for the detection of ALK rearrangements in NSCLC is available at http://www.fda.gov/CompanionDiagnostics.<br><br>**6 ADVERSE REACTIONS**<br>**6.1 Clinical Trial Experience**<br>*Advanced ALK-positive NSCLC Without Prior ALK-targeted Therapy*<br>In ALTA 1L, the safety of ALUNBRIG was evaluated in 136 patients with advanced ALK-positive NSCLC who had not previously received an ALK-targeted therapy [see Clinical Studies (14)]. (…)<br>*ALK-positive Advanced or Metastatic NSCLC Previously Treated with Crizotinib*<br>The safety of ALUNBRIG was evaluated in 219 patients with locally advanced or metastatic ALKpositive NSCLC who received at least 1 dose of ALUNBRIG in ALTA after experiencing disease progression on crizotinib. (…)<br><br>**14 CLINICAL STUDIES**<br>TKI-naïve Advanced ALK-positive NSCLC (ALTA 1L Study)<br>The efficacy of ALUNBRIG was demonstrated in a randomized (1:1), open-label, multicenter trial (ALTA 1L, NCT02737501) in adult patients with advanced ALK-positive NSCLC who had not previously received an ALK-targeted therapy. (…)<br>A total of 275 patients were randomized to receive ALUNBRIG 180 mg orally once daily with a 7-day lead-in at 90 mg once daily (n=137) or crizotinib 250 mg orally twice daily (n=138). Of the 275 enrolled patients, 239 had positive results using the companion diagnostic test, the Vysis ALK Break Apart FISH Probe Kit; central results were negative for 20 patients and unavailable for 16 patients. (…)<br>ALK-positive Advanced or Metastatic NSCLC Previously Treated with Crizotinib<br>The efficacy of ALUNBRIG was demonstrated in a two-arm, open-label, multicenter trial (ALTA, NCT02094573) in adult patients with locally advanced or metastatic ALK-positive non-small cell lung cancer (NSCLC) who had progressed on crizotinib. The study required patients to have a documented ALK rearrangement based on an FDA-approved test or a different test with adequate archival tissue to confirm ALK arrangement by the Vysis® ALK Break-Apart fluorescence in situ hybridization (FISH) Probe Kit test. (…)
205836, **05/12/2018**	Brivaracetam	Neurology	CYP2C19	Clinical Pharmacology	**12 CLINICAL PHARMACOLOGY**<br>**12.3 Pharmacokinetics**<br>*Metabolism*<br>Brivaracetam is primarily metabolized by hydrolysis of the amide moiety to form the corresponding carboxylic acid metabolite, and secondarily by hydroxylation on the propyl side chain to form the hydroxy metabolite. The hydrolysis reaction is mediated by hepatic and extra-hepatic amidase. The hydroxylation pathway is

* Therapeutic areas do not necessarily reflect the CDER review division.
† Representative biomarkers are listed based on standard nomenclature as per the Human Genome Organization (HUGO) symbol and/or simplified descriptors using other common conventions. Listed biomarkers do not necessarily reflect the terminology used in labeling. The term "Nonspecific" is provided when labeling does not explicitly identify the specific biomarker(s) or when the biomarker is represented by a molecular phenotype or gene signature, and in some cases the biomarker was inferred based on the labeling language.
‡ Referenced figures and tables may be found in the labeling available at Drugs@FDA.
Blue text represents the most recent addtions and/or changes since last posted version.

Table of Pharmacogenomic Biomarkers in Drug Labeling
Last Updated: 06/2024

NDA/ANDA/BLA Number, Label Version Date	Drug	Therapeutic Area*	Biomarker†	Labeling Sections	Labeling Text‡
					mediated primarily by CYP2C19. In human subjects possessing genetic variations in CYP2C19, production of the hydroxy metabolite is decreased 2-fold or 10-fold, while the blood level of brivaracetam itself is increased by 22% or 42%, respectively, in individuals with one or both mutated alleles. CYP2C19 poor metabolizers and patients using inhibitors of CYP2C19 may require dose reduction. An additional hydroxy acid metabolite is created by hydrolysis of the amide moiety on the hydroxy metabolite or hydroxylation of the propyl side chain on the carboxylic acid metabolite (mainly by CYP2C9). None of the 3 metabolites are pharmacologically active.
018692, **11/02/2018**	**Bupivacaine (1)**	Anesthesiology	G6PD	Warnings	**WARNINGS**<br>**Methemoglobinemia:** Cases of methemoglobinemia have been reported in association with local anesthetic use. Although all patients are at risk for methemoglobinemia, patients with glucose-6- phosphate dehydrogenase deficiency, congenital or idiopathic methemoglobinemia, cardiac or pulmonary compromise, infants under 6 months of age, and concurrent exposure to oxidizing agents or their metabolites are more susceptible to developing clinical manifestations of the condition. If local anesthetics must be used in these patients, close monitoring for symptoms and signs of methemoglobinemia is recommended.
018692, **11/02/2018**	**Bupivacaine (2)**	Anesthesiology	Nonspecific (Congenital Methemoglobinemia)	Warnings	**WARNINGS**<br>**Methemoglobinemia:** Cases of methemoglobinemia have been reported in association with local anesthetic use. Although all patients are at risk for methemoglobinemia, patients with glucose-6- phosphate dehydrogenase deficiency, congenital or idiopathic methemoglobinemia, cardiac or pulmonary compromise, infants under 6 months of age, and concurrent exposure to oxidizing agents or their metabolites are more susceptible to developing clinical manifestations of the condition. If local anesthetics must be used in these patients, close monitoring for symptoms and signs of methemoglobinemia is recommended.
**018644, 11/05/2019**	**Bupropion**	Psychiatry	CYP2D6	Clinical Pharmacology	**12 CLINICAL PHARMACOLOGY**<br>**12.3 Pharmacokinetics**<br>Potential for WELLBUTRIN to Affect Other Drugs<br>*Drugs Metabolized by CYP2D6:* In vitro, bupropion and its metabolites (erythrohydrobupropion, threohydrobupropion, hydroxybupropion) are CYP2D6 inhibitors. In a clinical trial of 15 male subjects (ages 19 to 35 years) who were extensive metabolizers of CYP2D6, bupropion 300 mg/day followed by a single dose of 50 mg desipramine increased the Cmax, AUC, and t1/2 of desipramine by an average of approximately 2-, 5-, and 2-fold, respectively. The effect was present for at least 7 days after the last dose of bupropion. Concomitant use of bupropion with other drugs metabolized by CYP2D6 has not been formally studied. (…)
**215430, 05/07/2024**	**Bupropion and Dextromethorphan**	Psychiatry	CYP2D6	Dosage and Administration, Use in Specific Populations, Clinical Pharmacology	**2 DOSAGE AND ADMINISTRATION**<br>**2.5 Dosage Recommendations for Known CYP2D6 Poor Metabolizers (PMs)**<br>The recommended dosage for patients known to be poor CYP2D6 metabolizers is one tablet once daily in the morning [see Use in Specific Populations (8.8), Clinical Pharmacology (12.3)].<br><br>**8 USE IN SPECIFIC POPULATIONS**<br>**8.8 CYP2D6 Poor Metabolizers**<br>Dosage adjustment is recommended in patients known to be poor CYP2D6 metabolizers because these patients have higher dextromethorphan concentrations than extensive/intermediate CYP2D6 metabolizers [see Dosage and Administration (2.5), Clinical Pharmacology (12.3)].<br><br>**12 CLINICAL PHARMACOLOGY**<br>**12.3 Pharmacokinetics**<br>*Excretion*<br>In CYP2D6 extensive metabolizers, approximately 37-52% of the orally administered dose of dextromethorphan is recovered in the urine. Less than 2% of the administered dose is excreted as unchanged parent drug in the urine. In CYP2D6 poor metabolizers, approximately 45-83% of the administered dose is recovered in the urine. Approximately 26% of the administered dose is excreted as unchanged parent drug in the urine.<br>Following oral administration of 200 mg of 14C-bupropion in humans, 87% and 10% of the radioactive dose were recovered in the urine and feces, respectively. Only 0.5% of the oral dose was excreted as unchanged bupropion.<br>Specific Populations<br>*Patients with Renal Impairment, Patients with Hepatic Impairment, and CYP2D6 Poor Metabolizers*<br>The effects of renal impairment, hepatic impairment, and CYP2D6 poor metabolizer status on the exposure to AUVELITY are summarized in Figure 1 [see Dosage and Administration (2.3, 2.5)].
009386, **12/24/2003**	**Busulfan**	Oncology	BCR-ABL1 (Philadelphia chromosome)	Clinical Studies	**14 CLINICAL STUDIES**<br>(…) Busulfan is clearly less effective in patients with chronic myelogenous leukemia who lack the Philadelphia (Ph) chromosome. Also, the so-called "juvenile" type of chronic myelogenous leukemia, typically occurring in young children and associated with the absence of a Philadelphia chromosome, responds poorly to busulfan. The drug is of no benefit in patients whose chronic myelogenous leukemia has entered a "blastic" phase. (…)
01/21/2021, **212888**	**Cabotegravir and Rilpivirine (1)**	Infectious Diseases	HLA-B	Clinical Studies	**14 CLINICAL STUDIES**<br>**14.1 Clinical Trials in Adults**<br>The efficacy of CABENUVA has been evaluated in two Phase 3 randomized, multicenter, activecontrolled, parallel-arm, open-label, non-inferiority trials:<br>• Trial 201584 (FLAIR, [NCT02938520]), (n = 629): HIV-1–infected, antiretroviral treatment (ART)- naive subjects received a dolutegravir INSTI-containing regimen for 20 weeks (either dolutegravir/abacavir/lamivudine or dolutegravir plus 2 other NRTIs if subjects were HLA B*5701 positive). (…)
01/21/2021, **212888**	**Cabotegravir and Rilpivirine (2)**	Infectious Diseases	UGT1A1	Clinical Pharmacology	**12 CLINICAL PHARMACOLOGY**<br>**12.3 Pharmacokinetics**<br>Specific Populations

* Therapeutic areas do not necessarily reflect the CDER review division.
† Representative biomarkers are listed based on standard nomenclature as per the Human Genome Organization (HUGO) symbol and/or simplified descriptors using other common conventions. Listed biomarkers do not necessarily reflect the terminology used in labeling. The term "Nonspecific" is provided when labeling does not explicitly identify the specific biomarker(s) or when the biomarker is represented by a molecular phenotype or gene signature, and in some cases the biomarker was inferred based on the labeling language.
‡ Referenced figures and tables may be found in the labeling available at Drugs@FDA.
Blue text represents the most recent addtions and/or changes since last posted version.

NDA/ANDA/BLA Number, Label Version Date	Drug	Therapeutic Area*	Biomarker†	Labeling Sections	Labeling Text‡
					No clinically significant differences in the pharmacokinetics of cabotegravir or rilpivirine were observed based on age, sex, race/ethnicity, body mass index, or UGT1A1 polymorphisms.
203756, **01/31/2020**	Cabozantinib	Oncology	RET	Clinical Studies	**14 CLINICAL STUDIES**<br>(…) Of 330 patients randomized, 67% were male, the median age was 55 years, 23% were 65 years or older, 89% were white, 54% had a baseline ECOG performance status of 0, and 92% had undergone a thyroidectomy. The RET mutation status determined by a research use assay was positive in 51%, negative in 14%, and was unknown in 35%. Twenty-five percent (25%) had two or more prior systemic therapies and 21% had been previously treated with a TKI. (…)
020896, 12/14/2022	Capecitabine (1)	Oncology	DPYD	Warnings and Precautions, Clinical Pharmacology, Patient Counseling Information	**5 WARNINGS AND PRECAUTIONS**<br>**5.2 Serious Adverse Reactions from Dihydropyrimidine Dehydrogenase (DPD) Deficiency**<br>Patients with certain homozygous or compound heterozygous variants in the DPYD gene known to result in complete or near complete absence of DPD activity (complete DPD deficiency) are at increased risk for acute early-onset toxicity and serious, including fatal, adverse reactions due to XELODA (e.g., mucositis, diarrhea, neutropenia, and neurotoxicity). Patients with partial DPD activity (partial DPD deficiency) may also have increased risk of serious, including fatal, adverse reactions.<br>XELODA is not recommended for use in patients known to have certain homozygous or compound heterozygous DPYD variants that result in complete DPD deficiency.<br>Withhold or permanently discontinue XELODA based on clinical assessment of the onset, duration, and severity of the observed adverse reactions in patients with evidence of acute earlyonset or unusually severe reactions, which may indicate complete DPD deficiency. No XELODA dose has been proven safe for patients with complete DPD deficiency. There are insufficient data to recommend a specific dose in patients with partial DPD deficiency.<br>Consider testing for genetic variants of DPYD prior to initiating XELODA to reduce the risk of serious adverse reactions if the patient's clinical status permits and based on clinical judgement [see Clinical Pharmacology (12.5)]. Serious adverse reactions may still occur even if no DPYD variants are identified.<br>An FDA-authorized test for the detection of genetic variants of DPYD to identify patients at risk of serious adverse reactions due to increased systemic exposure to XELODA is not currently available. Currently available tests used to identify DPYD variants may vary in accuracy and design (e.g., which DPYD variant(s) they identify).<br><br>**12 CLINICAL PHARMACOLOGY**<br>**12.5 Pharmacogenomics**<br>The DPYD gene encodes the enzyme DPD, which is responsible for the catabolism of >80% of fluorouracil. Approximately 3-5% of White populations have partial DPD deficiency and 0.2% of White populations have complete DPD deficiency, which may be due to certain genetic no function or decreased function variants in DPYD resulting in partial to complete or near complete absence of enzyme activity. DPD deficiency is estimated to be more prevalent in Black or African American populations compared to White populations. Insufficient information is available to estimate the prevalence of DPD deficiency in other populations.<br>Patients who are homozygous or compound heterozygous for no function DPYD variants (i.e., carry two no function DPYD variants) or are compound heterozygous for a no function DPYD variant plus a decreased function DPYD variant have complete DPD deficiency and are at increased risk for acute early-onset of toxicity and serious life-threatening, or fatal adverse reactions due to increased systemic exposure to XELODA. Partial DPD deficiency can result from the presence of either two decreased function DPYD variants or one normal function plus either a decreased function or a no function DPYD variant. Patients with partial DPD deficiency may also be at an increased risk for toxicity from XELODA.<br>Four DPYD variants have been associated with impaired DPD activity in White populations, especially when present as homozygous or compound heterozygous variants: c.1905+1G>A (DPYD *2A), c.1679T>G (DPYD *13), c.2846A>T, and c.1129-5923C>G (Haplotype B3). DPYD*2A and DPYD*13 are no function variants, and c.2846A>T and c.1129-5923C>G are decreased function variants. The decreased function DPYD variant c.557A>G is observed in individuals of African ancestry. This is not a complete listing of all DPYD variants that may result in DPD deficiency [see Warnings and Precautions (5.2)].<br><br>**17 PATIENT COUNSELING INFORMATION**<br><u>Serious Adverse Reactions from Dihydropyrimidine Dehydrogenase (DPD) Deficiency</u><br>Inform patients of the potential for serious and life-threatening adverse reactions due to DPD deficiency and discuss with your patient whether they should be tested for genetic variants of DPYD that are associated with an increased risk of serious adverse reactions from the use of XELODA. Advise patients to immediately contact their healthcare provider if symptoms of severe mucositis, diarrhea, neutropenia, and neurotoxicity occur [see Warnings and Precautions (5.2) and Clinical Pharmacology (12.5)].
020896, 12/14/2022	Capecitabine (2)	Oncology	ERBB2 (HER2)	Indications and Usage, Dosage and Administration, Adverse Reactions, Clinical Studies	**1 INDICATIONS AND USAGE**<br>**1.3 Gastric, Esophageal, or Gastroesophageal Junction Cancer**<br>XELODA is indicated for the:<br>• treatment of adults with unresectable or metastatic gastric, esophageal, or gastroesophageal junction cancer as a component of a combination chemotherapy regimen.<br>• treatment of adults with HER2-overexpressing metastatic gastric or gastroesophageal junction adenocarcinoma who have not received prior treatment for metastatic disease as a component of a combination regimen.<br><br>**2 DOSAGE AND ADMINISTRATION**<br>**2.3 Recommended Dosage for Gastric, Esophageal, or Gastroesophageal Junction Cancer**<br>The recommended dosage of XELODA for unresectable or metastatic gastric, esophageal, or gastroesophageal junction cancer is:<br>• 625 mg/m2 orally twice daily on days 1 to 21 of each 21-day cycle for a maximum of 8 cycles in combination with platinum-containing chemotherapy.

* Therapeutic areas do not necessarily reflect the CDER review division.
† Representative biomarkers are listed based on standard nomenclature as per the Human Genome Organization (HUGO) symbol and/or simplified descriptors using other common conventions. Listed biomarkers do not necessarily reflect the terminology used in labeling. The term "Nonspecific" is provided when labeling does not explicitly identify the specific biomarker(s) or when the biomarker is represented by a molecular phenotype or gene signature, and in some cases the biomarker was inferred based on the labeling language.
‡ Referenced figures and tables may be found in the labeling available at Drugs@FDA.
Blue text represents the most recent addtions and/or changes since last posted version.

NDA/ANDA/BLA Number, Label Version Date	Drug	Therapeutic Area*	Biomarker†	Labeling Sections	Labeling Text‡
					OR<br>• 850 mg/m2 or 1,000 mg/m2 orally twice daily for the first 14 days of each 21-day cycle until disease progression or unacceptable toxicity in combination with oxaliplatin 130 mg/m2 administered intravenously on day 1 of each cycle. Individualize the dose and dosing schedule of XELODA based on patient risk factors and adverse reactions.<br>The recommended dosage of XELODA for HER2-overexpressing metastatic gastric or gastroesophageal junction adenocarcinoma is 1,000 mg/m2 orally twic daily for the first 14 days of each 21-day cycle until disease progression or unacceptable toxicity in combination with cisplatin and trastuzumab.<br>Refer to the Prescribing Information for agents used in combination for additional dosing information as appropriate.<br><br>**6 ADVERSE REACTIONS**<br>Unresectable or Metastatic Gastric, Esophageal, or Gastroesophageal Junction Cancer<br>The safety of XELODA for the treatment of adults with unresectable or metastatic gastric, esophageal, or gastroesophageal junction cancer as a component of a combination chemotherapy regimen was derived from published literature [see Clinical Studies (14.3)]. The safety of XELODA for the treatment of adults with unresectable or metastatic gastric, esophageal, or gastroesophageal junction cancer as a component of a combination chemotherapy regimen was consistent with the known safety profile of XELODA. The safety of XELODA for the treatment of patients with HER2-overexpressing metastatic gastric or gastroesophageal junction adenocarcinoma who have not received prior treatment for metastatic disease as a component of a combination regimen was derived from the published literature [see Clinical Studies (14.3)]. The safety of XELODA for the treatment of patients with HER2-overexpressing metastatic gastric or gastroesophageal junction adenocarcinoma was consistent with the known safety profile of XELODA.<br><br>**14 CLINICA STUDIES**<br>**14.3 Gastric, Esophageal, or Gastroesophageal Junction Cancer**<br>The efficacy of XELODA for treatment of adults with unresectable or metastatic gastric, esophageal, or gastroesophageal junction cancer as a component of a combination chemotherapy regimen was derived from studies in the published literature. XELODA was evaluated in REAL2, a randomized non-inferiority, 2x2 factorial trial, where the major efficacy outcome measure was overall survival, and an additional randomized trial conducted by the North Central Cancer Treatment Group, where the major efficacy outcome measure was objective response rate.<br>The efficacy of XELODA for the treatment of adults with HER2-overexpressing metastatic gastric or gastroesophageal junction adenocarcinoma who have not received prior treatment for metastatic disease as a component of a combination regimen was derived from studies in the published literature. XELODA was evaluated in the ToGA trial [NCT01041404], an open-label, multicenter, randomized trial where the primary efficacy measure was overall survival.
**218197, 11/16/2023**	Capivasertib (1)	Oncology	AKT1	Indications and Usage, Dosage and Administration, Adverse Reactions, Clinical Studies	**1 INDICATIONS AND USAGE**<br>TRUQAP, in combination with fulvestrant, is indicated for the treatment of adult patients with hormone receptor (HR)-positive, human epidermal growth factor receptor 2 (HER2)-negative, locally advanced or metastatic breast cancer with one or more PIK3CA/AKT1/PTEN-alteration as detected by an FDA-approved test following progression on at least one endocrine-based regimen in the metastatic setting or recurrence on or within 12 months of completing adjuvant therapy.<br><br>**2 DOSAGE AND ADMINISTRATION**<br>**2.1 Patient Selection**<br>Select patients for the treatment of HR-positive, HER2-negative advanced or metastatic breast cancer with TRUQAP, based on the presence of one or more of the following genetic alterations in tumor tissue: PIK3CA/AKT1/PTEN [see Clinical Studies (14)].<br>Information on FDA-approved tests for the detection of PIK3CA, AKT1, and PTEN alterations is available at: http://www.fda.gov/CompanionDiagnostics.<br><br>**6 ADVERSE REACTIONS**<br>CAPItello-291<br>The safety of TRUQAP was evaluated in CAPItello-291, a clinical trial including 288 adult patients (155 patients in TRUQAP with fulvestrant arm and 133 patients in placebo with fulvestrant arm) whose breast cancer had one or more PIK3CA/AKT1/PTEN-alterations [see Clinical Studies (14)]. Among patients who received TRUQAP, 61% were exposed for 6 months or longer and 30% were exposed for greater than one year. (…)<br><br>**14 CLINICAL STUDIES**<br>The efficacy of TRUQAP with fulvestrant was evaluated in CAPItello-291 (NCT04305496), a randomized, double-blind, placebo-controlled, multicenter trial that enrolled 708 adult patients with locally advanced (inoperable) or metastatic HR-positive, HER2-negative (defined as IHC 0 or 1+, or IHC 2+/ISH-) breast cancer of which 289 patients had tumors with eligible PIK3CA/AKT1/PTEN-alterations. Eligible PIK3CA/AKT1 activating mutations or PTEN loss of function alterations were identified in the majority of FFPE tumor specimens using FoundationOne®CDx next-generation sequencing (n=686). All patients were required to have progression on an aromatase inhibitor (AI) based treatment in the metastatic setting or recurrence on or within 12 months of completing (neo)adjuvant treatment with an AI. Patients could have received up to two prior lines of endocrine therapy and up to 1 line of chemotherapy for locally advanced (inoperable) or metastatic disease. Patients were excluded if they had clinically significant abnormalities of glucose metabolism (defined as patients with diabetes mellitus Type 1, Type 2, requiring insulin treatment, or HbA1c ≥8% (63.9 mmol/mol)).<br>Patients were randomized (1:1) to receive either 400 mg of TRUQAP (n=355) or placebo (n=353), given orally twice daily for 4 days followed by 3 days off treatment each week of 28-day treatment cycle. Fulvestrant 500 mg intramuscular injection was administered on cycle 1 days 1 and 15, and then at day 1 of each subsequent 28-day cycle. Patients were treated until disease progression, or unacceptable toxicity. Randomization was stratified by presence of liver metastases (yes vs. no), prior treatment with CDK4/6 inhibitors (yes vs. no) and geographical region (region 1: US, Canada, Western Europe, Australia, and Israel vs region 2: Latin America, Eastern Europe and Russia vs Region 3: Asia).

NDA/ANDA/BLA Number, Label Version Date	Drug	Therapeutic Area*	Biomarker†	Labeling Sections	Labeling Text‡
					The major efficacy outcomes were investigator-assessed progression-free survival (PFS) in the overall population, and in the population of patients whose tumors have PIK3CA/AKT1/PTEN-alterations evaluated according to Response Evaluation Criteria in Solid Tumors (RECIST), version 1.1. Additional efficacy outcome measures were overall survival (OS), investigator assessed objective response rate (ORR) and duration of response (DoR).<br>A statistically significant difference in PFS was observed in the overall population and the population of patients whose tumors have PIK3CA/AKT1/PTEN-alteration. An exploratory analysis of PFS in the 313 (44%) patients whose tumors did not have a PIK3CA/AKT1/PTEN-alteration showed a HR of 0.79 (95% CI: 0.61, 1.02), indicating that the difference in the overall population was primarily attributed to the results seen in the population of patients whose tumors have PIK3CA/AKT1/PTEN-alteration.<br>Of the 289 patients whose tumors were PIK3CA/AKT1/PTEN-altered, the median age was 59 years (range 34 to 90); female (99%); White (52%), Asian (29%), Black (1%), American Indian/Alaska Native (0.7%), other races (17%) and 9% were Hispanic/Latino. Eastern Cooperative Oncology Group (ECOG) performance status was 0 (66%) or 1 (34%), and 18% were premenopausal or perimenopausal. Seventy-six percent of patients had an alteration in PIK3CA, 13% had an alteration in AKT1, and 17% had an alteration in PTEN. All patients received prior endocrine-based therapy (100% AI based treatment and 44% received tamoxifen). Seventy-one percent of patients were previously treated with a CDK4/6 inhibitor and 18% received prior chemotherapy for locally advanced (inoperable) or metastatic disease.<br>Efficacy results for PIK3CA/AKT1/PTEN-altered subgroup are presented in Table 7 and Figure 1. Results from the blinded independent review committee (BICR) assessment were consistent with the investigator assessed PFS results. Overall survival results were immature at the time of the PFS analysis (30% of the patients died). (See Table 7 nad Figure 1)
**218197, 11/16/2023**	Capivasertib (2)	Oncology	ERBB2 (HER2)	Indications and Usage, Dosage and Administration, Clinical Studies	**1 INDICATIONS AND USAGE**<br>TRUQAP, in combination with fulvestrant, is indicated for the treatment of adult patients with hormone receptor (HR)-positive, human epidermal growth factor receptor 2 (HER2)-negative, locally advanced or metastatic breast cancer with one or more PIK3CA/AKT1/PTEN-alteration as detected by an FDA-approved test following progression on at least one endocrine-based regimen in the metastatic setting or recurrence on or within 12 months of completing adjuvant therapy.<br><br>**2 DOSAGE AND ADMINISTRATION**<br>**2.1 Patient Selection**<br>Select patients for the treatment of HR-positive, HER2-negative advanced or metastatic breast cancer with TRUQAP, based on the presence of one or more of the following genetic alterations in tumor tissue: PIK3CA/AKT1/PTEN [see Clinical Studies (14)].<br>Information on FDA-approved tests for the detection of PIK3CA, AKT1, and PTEN alterations is available at: http://www.fda.gov/CompanionDiagnostics.<br><br>**14 CLINICAL STUDIES**<br>The efficacy of TRUQAP with fulvestrant was evaluated in CAPItello-291 (NCT04305496), a randomized, double-blind, placebo-controlled, multicenter trial that enrolled 708 adult patients with locally advanced (inoperable) or metastatic HR-positive, HER2-negative (defined as IHC 0 or 1+, or IHC 2+/ISH-) breast cancer of which 289 patients had tumors with eligible PIK3CA/AKT1/PTEN-alterations. Eligible PIK3CA/AKT1 activating mutations or PTEN loss of function alterations were identified in the majority of FFPE tumor specimens using FoundationOne®CDx next-generation sequencing (n=686). All patients were required to have progression on an aromatase inhibitor (AI) based treatment in the metastatic setting or recurrence on or within 12 months of completing (neo)adjuvant treatment with an AI. Patients could have received up to two prior lines of endocrine therapy and up to 1 line of chemotherapy for locally advanced (inoperable) or metastatic disease. Patients were excluded if they had clinically significant abnormalities of glucose metabolism (defined as patients with diabetes mellitus Type 1, Type 2, requiring insulin treatment, or HbA1c ≥8% (63.9 mmol/mol)). (…)
**218197, 11/16/2023**	Capivasertib (3)	Oncology	ESR (Hormone Receptor)	Indications and Usage, Dosage and Administration, Clinical Studies	**1 INDICATIONS AND USAGE**<br>TRUQAP, in combination with fulvestrant, is indicated for the treatment of adult patients with hormone receptor (HR)-positive, human epidermal growth factor receptor 2 (HER2)-negative, locally advanced or metastatic breast cancer with one or more PIK3CA/AKT1/PTEN-alteration as detected by an FDA-approved test following progression on at least one endocrine-based regimen in the metastatic setting or recurrence on or within 12 months of completing adjuvant therapy.<br><br>**2 DOSAGE AND ADMINISTRATION**<br>**2.1 Patient Selection**<br>Select patients for the treatment of HR-positive, HER2-negative advanced or metastatic breast cancer with TRUQAP, based on the presence of one or more of the following genetic alterations in tumor tissue: PIK3CA/AKT1/PTEN [see Clinical Studies (14)].<br>Information on FDA-approved tests for the detection of PIK3CA, AKT1, and PTEN alterations is available at: http://www.fda.gov/CompanionDiagnostics.<br><br>**14 CLINICAL STUDIES**<br>The efficacy of TRUQAP with fulvestrant was evaluated in CAPItello-291 (NCT04305496), a randomized, double-blind, placebo-controlled, multicenter trial that enrolled 708 adult patients with locally advanced (inoperable) or metastatic HR-positive, HER2-negative (defined as IHC 0 or 1+, or IHC 2+/ISH-) breast cancer of which 289 patients had tumors with eligible PIK3CA/AKT1/PTEN-alterations. Eligible PIK3CA/AKT1 activating mutations or PTEN loss of function alterations were identified in the majority of FFPE tumor specimens using FoundationOne®CDx next-generation sequencing (n=686). All patients were required to have progression on an aromatase inhibitor (AI) based treatment in the metastatic setting or recurrence on or within 12 months of completing (neo)adjuvant treatment with an AI. Patients could have received up to two prior lines of endocrine therapy and up to 1 line of chemotherapy for locally advanced (inoperable) or metastatic disease. Patients were excluded if they had clinically significant abnormalities of glucose metabolism (defined as patients with diabetes mellitus Type 1, Type 2, requiring insulin treatment, or HbA1c ≥8% (63.9 mmol/mol)). (…)
**218197, 11/16/2023**	Capivasertib (4)	Oncology	PIK3CA	Indications and Usage, Dosage and Administration,	**1 INDICATIONS AND USAGE**<br>TRUQAP, in combination with fulvestrant, is indicated for the treatment of adult patients with hormone receptor (HR)-positive, human epidermal growth factor receptor 2 (HER2)-negative, locally advanced or metastatic breast cancer with one or more PIK3CA/AKT1/PTEN-alteration as detected by an FDA-approved test following progression on at least one endocrine-based regimen in the metastatic setting or recurrence on or within 12 months of completing adjuvant therapy.

* Therapeutic areas do not necessarily reflect the CDER review division.
† Representative biomarkers are listed based on standard nomenclature as per the Human Genome Organization (HUGO) symbol and/or simplified descriptors using other common conventions. Listed biomarkers do not necessarily reflect the terminology used in labeling. The term "Nonspecific" is provided when labeling does not explicitly identify the specific biomarker(s) or when the biomarker is represented by a molecular phenotype or gene signature, and in some cases the biomarker was inferred based on the labeling language.
‡ Referenced figures and tables may be found in the labeling available at Drugs@FDA.
Blue text represents the most recent addtions and/or changes since last posted version.

NDA/ANDA/BLA Number, Label Version Date	Drug	Therapeutic Area*	Biomarker†	Labeling Sections	Labeling Text‡
				Adverse Reactions, Clinical Studies	**2 DOSAGE AND ADMINISTRATION**<br>**2.1 Patient Selection**<br>Select patients for the treatment of HR-positive, HER2-negative advanced or metastatic breast cancer with TRUQAP, based on the presence of one or more of the following genetic alterations in tumor tissue: PIK3CA/AKT1/PTEN [see Clinical Studies (14)].<br>Information on FDA-approved tests for the detection of PIK3CA, AKT1, and PTEN alterations is available at: http://www.fda.gov/CompanionDiagnostics.<br><br>**6 ADVERSE REACTIONS**<br>CAPItello-291<br>The safety of TRUQAP was evaluated in CAPItello-291, a clinical trial including 288 adult patients (155 patients in TRUQAP with fulvestrant arm and 133 patients in placebo with fulvestrant arm) whose breast cancer had one or more PIK3CA/AKT1/PTEN-alterations [see Clinical Studies (14)]. Among patients who received TRUQAP, 61% were exposed for 6 months or longer and 30% were exposed for greater than one year. (…)<br><br>**14 CLINICAL STUDIES**<br>The efficacy of TRUQAP with fulvestrant was evaluated in CAPItello-291 (NCT04305496), a randomized, double-blind, placebo-controlled, multicenter trial that enrolled 708 adult patients with locally advanced (inoperable) or metastatic HR-positive, HER2-negative (defined as IHC 0 or 1+, or IHC 2+/ISH-) breast cancer of which 289 patients had tumors with eligible PIK3CA/AKT1/PTEN-alterations. Eligible PIK3CA/AKT1 activating mutations or PTEN loss of function alterations were identified in the majority of FFPE tumor specimens using FoundationOne®CDx next-generation sequencing (n=686). All patients were required to have progression on an aromatase inhibitor (AI) based treatment in the metastatic setting or recurrence on or within 12 months of completing (neo)adjuvant treatment with an AI. Patients could have received up to two prior lines of endocrine therapy and up to 1 line of chemotherapy for locally advanced (inoperable) or metastatic disease. Patients were excluded if they had clinically significant abnormalities of glucose metabolism (defined as patients with diabetes mellitus Type 1, Type 2, requiring insulin treatment, or HbA1c ≥8% (63.9 mmol/mol)).<br>Patients were randomized (1:1) to receive either 400 mg of TRUQAP (n=355) or placebo (n=353), given orally twice daily for 4 days followed by 3 days off treatment each week of 28-day treatment cycle. Fulvestrant 500 mg intramuscular injection was administered on cycle 1 days 1 and 15, and then at day 1 of each subsequent 28-day cycle. Patients were treated until disease progression, or unacceptable toxicity. Randomization was stratified by presence of liver metastases (yes vs. no), prior treatment with CDK4/6 inhibitors (yes vs. no) and geographical region (region 1: US, Canada, Western Europe, Australia, and Israel vs region 2: Latin America, Eastern Europe and Russia vs Region 3: Asia).<br>The major efficacy outcomes were investigator-assessed progression-free survival (PFS) in the overall population, and in the population of patients whose tumors have PIK3CA/AKT1/PTEN-alterations evaluated according to Response Evaluation Criteria in Solid Tumors (RECIST), version 1.1. Additional efficacy outcome measures were overall survival (OS), investigator assessed objective response rate (ORR) and duration of response (DoR).<br>A statistically significant difference in PFS was observed in the overall population and the population of patients whose tumors have PIK3CA/AKT1/PTEN-alteration. An exploratory analysis of PFS in the 313 (44%) patients whose tumors did not have a PIK3CA/AKT1/PTEN-alteration showed a HR of 0.79 (95% CI: 0.61, 1.02), indicating that the difference in the overall population was primarily attributed to the results seen in the population of patients whose tumors have PIK3CA/AKT1/PTEN-alteration.<br>Of the 289 patients whose tumors were PIK3CA/AKT1/PTEN-altered, the median age was 59 years (range 34 to 90); female (99%); White (52%), Asian (29%), Black (1%), American Indian/Alaska Native (0.7%), other races (17%) and 9% were Hispanic/Latino. Eastern Cooperative Oncology Group (ECOG) performance status was 0 (66%) or 1 (34%), and 18% were premenopausal or perimenopausal. Seventy-six percent of patients had an alteration in PIK3CA, 13% had an alteration in AKT1, and 17% had an alteration in PTEN. All patients received prior endocrine-based therapy (100% AI based treatment and 44% received tamoxifen). Seventy-one percent of patients were previously treated with a CDK4/6 inhibitor and 18% received prior chemotherapy for locally advanced (inoperable) or metastatic disease.<br>Efficacy results for PIK3CA/AKT1/PTEN-altered subgroup are presented in Table 7 and Figure 1. Results from the blinded independent review committee (BICR) assessment were consistent with the investigator assessed PFS results. Overall survival results were immature at the time of the PFS analysis (30% of the patients died). (See Table 7 nad Figure 1)
**218197, 11/16/2023**	Capivasertib (5)	Oncology	PTEN	Indications and Usage, Dosage and Administration, Adverse Reactions, Clinical Studies	**1 INDICATIONS AND USAGE**<br>TRUQAP, in combination with fulvestrant, is indicated for the treatment of adult patients with hormone receptor (HR)-positive, human epidermal growth factor receptor 2 (HER2)-negative, locally advanced or metastatic breast cancer with one or more PIK3CA/AKT1/PTEN-alteration as detected by an FDA-approved test following progression on at least one endocrine-based regimen in the metastatic setting or recurrence on or within 12 months of completing adjuvant therapy.<br><br>**2 DOSAGE AND ADMINISTRATION**<br>**2.1 Patient Selection**<br>Select patients for the treatment of HR-positive, HER2-negative advanced or metastatic breast cancer with TRUQAP, based on the presence of one or more of the following genetic alterations in tumor tissue: PIK3CA/AKT1/PTEN [see Clinical Studies (14)].<br>Information on FDA-approved tests for the detection of PIK3CA, AKT1, and PTEN alterations is available at: http://www.fda.gov/CompanionDiagnostics.<br><br>**6 ADVERSE REACTIONS**<br>CAPItello-291<br>The safety of TRUQAP was evaluated in CAPItello-291, a clinical trial including 288 adult patients (155 patients in TRUQAP with fulvestrant arm and 133 patients in placebo with fulvestrant arm) whose breast cancer had one or more PIK3CA/AKT1/PTEN-alterations [see Clinical Studies (14)]. Among patients who received TRUQAP, 61% were exposed for 6 months or longer and 30% were exposed for greater than one year. (…)

* Therapeutic areas do not necessarily reflect the CDER review division.
† Representative biomarkers are listed based on standard nomenclature as per the Human Genome Organization (HUGO) symbol and/or simplified descriptors using other common conventions. Listed biomarkers do not necessarily reflect the terminology used in labeling. The term “Nonspecific” is provided when labeling does not explicitly identify the specific biomarker(s) or when the biomarker is represented by a molecular phenotype or gene signature, and in some cases the biomarker was inferred based on the labeling language.
‡ Referenced figures and tables may be found in the labeling available at Drugs@FDA.
Blue text represents the most recent addtions and/or changes since last posted version.

NDA/ANDA/BLA Number, Label Version Date	Drug	Therapeutic Area*	Biomarker†	Labeling Sections	Labeling Text‡
					**14 CLINICAL STUDIES**<br>The efficacy of TRUQAP with fulvestrant was evaluated in CAPItello-291 (NCT04305496), a randomized, double-blind, placebo-controlled, multicenter trial that enrolled 708 adult patients with locally advanced (inoperable) or metastatic HR-positive, HER2-negative (defined as IHC 0 or 1+, or IHC 2+/ISH-) breast cancer of which 289 patients had tumors with eligible PIK3CA/AKT1/PTEN-alterations. Eligible PIK3CA/AKT1 activating mutations or PTEN loss of function alterations were identified in the majority of FFPE tumor specimens using FoundationOne®CDx next-generation sequencing (n=686). All patients were required to have progression on an aromatase inhibitor (AI) based treatment in the metastatic setting or recurrence on or within 12 months of completing (neo)adjuvant treatment with an AI. Patients could have received up to two prior lines of endocrine therapy and up to 1 line of chemotherapy for locally advanced (inoperable) or metastatic disease. Patients were excluded if they had clinically significant abnormalities of glucose metabolism (defined as patients with diabetes mellitus Type 1, Type 2, requiring insulin treatment, or HbA1c ≥8% (63.9 mmol/mol)).<br>Patients were randomized (1:1) to receive either 400 mg of TRUQAP (n=355) or placebo (n=353), given orally twice daily for 4 days followed by 3 days off treatment each week of 28-day treatment cycle. Fulvestrant 500 mg intramuscular injection was administered on cycle 1 days 1 and 15, and then at day 1 of each subsequent 28-day cycle. Patients were treated until disease progression, or unacceptable toxicity. Randomization was stratified by presence of liver metastases (yes vs. no), prior treatment with CDK4/6 inhibitors (yes vs. no) and geographical region (region 1: US, Canada, Western Europe, Australia, and Israel vs region 2: Latin America, Eastern Europe and Russia vs Region 3: Asia).<br>The major efficacy outcomes were investigator-assessed progression-free survival (PFS) in the overall population, and in the population of patients whose tumors have PIK3CA/AKT1/PTEN-alterations evaluated according to Response Evaluation Criteria in Solid Tumors (RECIST), version 1.1. Additional efficacy outcome measures were overall survival (OS), investigator assessed objective response rate (ORR) and duration of response (DoR).<br>A statistically significant difference in PFS was observed in the overall population and the population of patients whose tumors have PIK3CA/AKT1/PTEN-alteration. An exploratory analysis of PFS in the 313 (44%) patients whose tumors did not have a PIK3CA/AKT1/PTEN-alteration showed a HR of 0.79 (95% CI: 0.61, 1.02), indicating that the difference in the overall population was primarily attributed to the results seen in the population of patients whose tumors have PIK3CA/AKT1/PTEN-alteration.<br>Of the 289 patients whose tumors were PIK3CA/AKT1/PTEN-altered, the median age was 59 years (range 34 to 90); female (99%); White (52%), Asian (29%), Black (1%), American Indian/Alaska Native (0.7%), other races (17%) and 9% were Hispanic/Latino. Eastern Cooperative Oncology Group (ECOG) performance status was 0 (66%) or 1 (34%), and 18% were premenopausal or perimenopausal. Seventy-six percent of patients had an alteration in PIK3CA, 13% had an alteration in AKT1, and 17% had an alteration in PTEN. All patients received prior endocrine-based therapy (100% AI based treatment and 44% received tamoxifen). Seventy-one percent of patients were previously treated with a CDK4/6 inhibitor and 18% received prior chemotherapy for locally advanced (inoperable) or metastatic disease.<br>Efficacy results for PIK3CA/AKT1/PTEN-altered subgroup are presented in Table 7 and Figure 1. Results from the blinded independent review committee (BICR) assessment were consistent with the investigator assessed PFS results. Overall survival results were immature at the time of the PFS analysis (30% of the patients died). (See Table 7 nad Figure 1)
213591, 08/10/2022	Capmatinib	Oncology	MET	Indications and Usage, Dosage and Administration, Clinical Studies	**1 INDICATIONS AND USAGE**<br>TABRECTA is indicated for the treatment of adult patients with metastatic non-small cell lung cancer (NSCLC) whose tumors have a mutation that leads to mesenchymal-epithelial transition (MET) exon 14 skipping as detected by an FDAapproved test.<br><br>**2 DOSAGE AND ADMINISTRATION**<br>**2.1 Patient Selection**<br>Select patients for treatment with TABRECTA based on the presence of a mutation that leads to MET exon 14 skipping in tumor or plasma specimens [see Clinical Studies (14)]. If a mutation that leads to MET exon 14 skipping is not detected in a plasma specimen, test tumor tissue if feasible. Information on FDA-approved tests is available at: http://www.fda.gov/CompanionDiagnostics.<br><br>**14 CLINICAL STUDIES**<br>Metastatic NSCLC with a Mutation that Leads to MET Exon 14 Skipping<br>The efficacy of TABRECTA was evaluated in GEOMETRY mono-1, a multicenter, non-randomized, open-label, multicohort study (NCT02414139). Eligible patients were required to have NSCLC with a mutation that leads to MET exon 14 skipping, epidermal growth factor receptor (EGFR) wild-type and anaplastic lymphoma kinase (ALK) negative status, and at least one measurable lesion as defined by Response Evaluation Criteria in Solid Tumors (RECIST) version 1.1. Patients with symptomatic CNS metastases, clinically significant uncontrolled cardiac disease, or who received treatment with any MET or hepatocyte growth factor (HGF) inhibitor were not eligible for the study. Out of the 97 patients enrolled in GEOMETRY mono-1 following the central confirmation of MET exon 14 skipping by a RNA-based clinical trial assay, 78 patient samples were retested with the FDA-approved FoundationOne® CDx (22 treatment-naïve and 56 previously treated patients) to detect mutations that lead to MET exon 14 skipping. Out of 78 samples retested with FoundationOne® CDx, 73 samples were evaluable (20 treatment-naïve and 53 previously treated patients), 72 (20 treatment-naïve and 52 previously treated patients) of which were confirmed to have a mutation that leads to MET exon 14 skipping, demonstrating an estimated positive percentage agreement of 99% (72/73) between the clinical trial assay and the FDA-approved assay.
016608, **03/20/2018**	Carbamazepine (1)	Neurology	HLA-B	Boxed Warning, Warnings, Precautions	**BOXED WARNING**<br>*Serious dermatologic reactions and HLA-B*1502 allele*<br>Serious and sometimes fatal dermatologic reactions, including toxic epidermal necrolysis (TEN) and Stevens-Johnson syndrome (SJS), have been reported during treatment with Tegretol. These reactions are estimated to occur in 1 to 6 per 10,000 new users in countries with mainly Caucasian populations, but the risk in some Asian countries is estimated to be about 10 times higher. Studies in patients of Chinese ancestry have found a strong association between the risk of developing SJS/TEN and the presence of HLA-B*1502, an inherited allelic variant of the HLA-B gene. HLA-B*1502 is found almost exclusively in patients with ancestry across broad areas of Asia. Patients with ancestry in genetically at-risk populations should be screened for the presence of HLA-B*1502 prior to

Table of Pharmacogenomic Biomarkers in Drug Labeling
Last Updated: 06/2024

NDA/ANDA/BLA Number, Label Version Date	Drug	Therapeutic Area*	Biomarker†	Labeling Sections	Labeling Text‡
					initiating treatment with Tegretol. Patients testing positive for the allele should not be treated with Tegretol unless the benefit clearly outweighs the risk (see WARNINGS AND PRECAUTIONS, Laboratory Tests). (…)<br><br>**WARNINGS**<br>*Serious Dermatologic Reactions*<br>Serious and sometimes fatal dermatologic reactions, including toxic epidermal necrolysis (TEN) and Stevens-Johnson syndrome (SJS), have been reported with Tegretol treatment. The risk of these events is estimated to be about 1 to 6 per 10,000 new users in countries with mainly Caucasian populations. However, the risk in some Asian countries is estimated to be about 10 times higher. Tegretol should be discontinued at the first sign of a rash, unless the rash is clearly not drug-related. If signs or symptoms suggest SJS/TEN, use of this drug should not be resumed and alternative therapy should be considered.<br>*SJS/TEN and HLA-B*1502 Allele*<br>Retrospective case-control studies have found that in patients of Chinese ancestry there is a strong association between the risk of developing SJS/TEN with carbamazepine treatment and the presence of an inherited variant of the HLA-B gene, HLA-B*1502. The occurrence of higher rates of these reactions in countries with higher frequencies of this allele suggests that the risk may be increased in allele-positive individuals of any ethnicity.<br>Across Asian populations, notable variation exists in the prevalence of HLA-B*1502. Greater than 15% of the population is reported positive in Hong Kong, Thailand, Malaysia, and parts of the Philippines, compared to about 10% in Taiwan and 4% in North China. South Asians, including Indians, appear to have intermediate prevalence of HLA-B*1502, averaging 2% to 4%, but higher in some groups. HLA-B*1502 is present in less than 1% of the population in Japan and Korea.<br>HLA-B*1502 is largely absent in individuals not of Asian origin (e.g., Caucasians, African-Americans, Hispanics, and Native Americans).<br>Prior to initiating Tegretol therapy, testing for HLA-B*1502 should be performed in patients with ancestry in populations in which HLA-B*1502 may be present. In deciding which patients to screen, the rates provided above for the prevalence of HLA-B*1502 may offer a rough guide, keeping in mind the limitations of these figures due to wide variability in rates even within ethnic groups, the difficulty in ascertaining ethnic ancestry, and the likelihood of mixed ancestry. Tegretol should not be used in patients positive for HLA-B*1502 unless the benefits clearly outweigh the risks. Tested patients who are found to be negative for the allele are thought to have a low risk of SJS/TEN (see BOXED WARNING and PRECAUTIONS, Laboratory Tests).<br>Over 90% of Tegretol treated patients who will experience SJS/TEN have this reaction within the first few months of treatment. This information may be taken into consideration in determining the need for screening of genetically at-risk patients currently on Tegretol.<br>The HLA-B*1502 allele has not been found to predict risk of less severe adverse cutaneous reactions from Tegretol such as maculopapular eruption (MPE) or to predict Drug Reaction with Eosinophilia and Systemic Symptoms (DRESS).<br>Limited evidence suggests that HLA-B*1502 may be a risk factor for the development of SJS/TEN in patients of Chinese ancestry taking other antiepileptic drugs associated with SJS/TEN, including phenytoin. Consideration should be given to avoiding use of other drugs associated with SJS/TEN in HLA-B*1502 positive patients, when alternative therapies are otherwise equally acceptable.<br><br>**PRECAUTIONS**<br>*Laboratory Tests*<br>For genetically at-risk patients (see WARNINGS), high-resolution ‘HLA-B*1502 typing’ is recommended. The test is positive if either one or two HLA-B*1502 alleles are detected and negative if no HLA B*1502 alleles are detected.
016608,<br>**03/20/2018**	Carbamazepine (2)	Neurology	HLA-A	Warnings	**WARNINGS**<br>*Hypersensitivity Reactions and HLA-A*3101 Allele*<br>Retrospective case-control studies in patients of European, Korean, and Japanese ancestry have found a moderate association between the risk of developing hypersensitivity reactions and the presence of HLAA*3101, an inherited allelic variant of the HLA-A gene, in patients using carbamazepine. These hypersensitivity reactions include SJS/TEN, maculopapular eruptions, and Drug Reaction with Eosinophilia and Systemic Symptoms (see DRESS/Multiorgan hypersensitivity below).<br>HLA-A*3101 is expected to be carried by more than 15% of patients of Japanese, Native American, Southern Indian (for example, Tamil Nadu) and some Arabic ancestry; up to about 10% in patients of Han Chinese, Korean, European, Latin American, and other Indian ancestry; and up to about 5% in African-Americans and patients of Thai, Taiwanese, and Chinese (Hong Kong) ancestry.<br>The risks and benefits of Tegretol therapy should be weighed before considering Tegretol in patients known to be positive for HLA A*3101.<br>Application of HLA genotyping as a screening tool has important limitations and must never substitute for appropriate clinical vigilance and patient management. Many HLA-B*1502-positive and HLA-A*3101-positive patients treated with Tegretol will not develop SJS/TEN or other hypersensitivity reactions, and these reactions can still occur infrequently in HLA-B*1502-negative and HLA-A*3101 negative patients of any ethnicity. The role of other possible factors in the development of, and morbidity from, SJS/TEN and other hypersensitivity reactions, such as antiepileptic drug (AED) dose, compliance, concomitant medications, comorbidities, and the level of dermatologic monitoring, have not been studied.
022562,<br>**12/23/2019**	Carglumic Acid	Inborn Errors of Metabolism	NAGS	Indications and Usage, Dosage and Administration, Warnings and Precautions, Use in Specific Populations, Clinical	**1 INDICATIONS AND USAGE**<br>**1.1 Acute hyperammonemia in patients with NAGS deficiency**<br>Carbaglu is indicated as an adjunctive therapy in pediatric and adult patients for the treatment of acute hyperammonemia due to the deficiency of the hepatic enzyme N-acetylglutamate synthase (NAGS). During acute hyperammonemic episodes concomitant administration of Carbaglu with other ammonia lowering therapies such as alternate pathway medications, hemodialysis, and dietary protein restriction are recommended.<br>**1.2 Chronic Hyperammonemia in Patients with NAGS Deficiency**<br>Carbaglu is indicated for maintenance therapy in pediatric and adult patients for chronic hyperammonemia due to the deficiency of the hepatic enzyme N-acetylglutamate synthase (NAGS). During maintenance therapy, the concomitant use of other ammonia lowering therapies and protein restriction may be reduced or discontinued based on plasma ammonia levels.

* Therapeutic areas do not necessarily reflect the CDER review division.
† Representative biomarkers are listed based on standard nomenclature as per the Human Genome Organization (HUGO) symbol and/or simplified descriptors using other common conventions. Listed biomarkers do not necessarily reflect the terminology used in labeling. The term “Nonspecific” is provided when labeling does not explicitly identify the specific biomarker(s) or when the biomarker is represented by a molecular phenotype or gene signature, and in some cases the biomarker was inferred based on the labeling language.
‡ Referenced figures and tables may be found in the labeling available at Drugs@FDA.
Blue text represents the most recent addtions and/or changes since last posted version.

NDA/ANDA/BLA Number, Label Version Date	Drug	Therapeutic Area*	Biomarker†	Labeling Sections	Labeling Text‡
				Pharmacology, Clinical Studies	**2 DOSAGE AND ADMINISTRATION**<br>**2.1 Recommended Dosage**<br>CARBAGLU should be initiated as soon as the diagnosis of NAGS deficiency is suspected, which may be as soon as at birth, and managed by a physician and medical team experienced in metabolic disorders. (…)<br><br>**5 WARNINGS AND PRECAUTIONS**<br>**5.1 Hyperammonemia**<br>(…) Since hyperammonemia in NAGS deficiency is the result of imbalance between ammonia detoxification capacity and protein catabolism, complete protein restriction during an acute hyperammonemic episode is recommended for no longer than 12 to 36 hours while maximizing caloric supplementation to reverse catabolism. Protein should be reintroduced as early as possible, following improvement of metabolic and clinical abnormalities in this setting. During long-term management, dietary protein restriction should be instituted to maintain blood ammonia level within an acceptable range for age.<br><br>**8 USE IN SPECIFIC POPULATIONS**<br>**8.1 Pregnancy**<br>There are no adequate and well controlled studies or available human data with Carbaglu in pregnant women. Decreased survival and growth occurred in offspring born to animals that received carglumic acid at doses similar to the maximum recommended starting human dose during pregnancy and lactation. Because untreated N-acetylglutamate synthase (NAGS) deficiency results in irreversible neurologic damage and death, women with NAGS must remain on treatment throughout pregnancy. (…)<br>**8.3 Nursing Mothers**<br>It is not known whether Carbaglu is excreted in human milk. Carglumic acid is excreted in rat milk, and an increase in mortality and impairment of body weight gain occurred in neonatal rats nursed by mothers receiving carglumic acid. Because many drugs are excreted in human milk and because of the potential for serious adverse reactions in nursing infants from Carbaglu, human milk-feeding is not recommended. Treatment is continuous and life-long for NAGS deficiency patients.<br>**8.4 Pediatric Use**<br>The efficacy of Carbaglu for the treatment of hyperammonemia in patients with NAGS deficiency from birth to adulthood was evaluated in a retrospective review of the clinical course of 23 NAGS deficiency patients who all began Carbaglu treatment during infancy or childhood. There are no apparent differences in clinical response between adults and pediatric NAGS deficiency patients treated with Carbaglu, however, data are limited.<br><br>**12 CLINICAL PHARMACOLOGY**<br>**12.2 Pharmacodynamics**<br>In a retrospective review of the clinical course in 23 patients with NAGS deficiency, carglumic acid reduced plasma ammonia levels within 24 hours when administered with and without concomitant ammonia lowering therapies. No dose response relationship has been identified. (…)<br><br>**14 CLINICAL STUDIES**<br>**14.1 Responses of Patients with NAGS Deficiency to Acute and Chronic Treatment**<br>The efficacy of Carbaglu in the treatment of hyperammonemia due to NAGS deficiency was evaluated in a retrospective review of the clinical course of 23 NAGS deficiency patients who received Carbaglu treatment for a median of 7.9 years (range 0.6 to 20.8 years). (See Table 2)<br>(…) Of the 23 NAGS deficiency patients who received treatment with Carbaglu, a subset of 13 patients who had both well documented plasma ammonia levels prior to Carbaglu treatment and after long-term treatment with Carbaglu were selected for analysis. (…)<br>(…) The mean plasma ammonia level at baseline and the decline that is observed after treatment with Carbaglu in 13 evaluable patients with NAGS deficiency is illustrated in Figure 1. (See Figure 1)
204370,<br>**05/24/2019**	Cariprazine	Psychiatry	CYP2D6	Clinical Pharmacology	**12 CLINCIAL PHARMACOLOGY**<br>**12.3 Pharmacokinetics**<br>*CYP2D6 Poor Metabolizers*<br>CYP2D6 poor metabolizer status does not have clinically relevant effect on pharmacokinetics of cariprazine, DCAR, or DDCAR.<br>*Drug Interaction Studies*<br>*CYP2D6 inhibitors*<br>CYP2D6 inhibitors are not expected to influence pharmacokinetics of cariprazine, DCAR or DDCAR based on the observations in CYP2D6 poor metabolizers.
011792,<br>**04/04/2019**	Carisoprodol	Rheumatology	CYP2C19	Use in Specific Populations, Clinical Pharmacology	**8 USE IN SPECIFIC POPULATION**<br>**8.8 Patients with Reduced CYP2C19 Activity**<br>Patients with reduced CYP2C19 activity have higher exposure to carisoprodol. Therefore, caution should be exercised in administration of SOMA to these patients [see Clinical Pharmacology (12.3)].<br><br>**12 CLINCIAL PHARMACOLOGY**<br>**12.3 Pharmacokinetics**<br>*Metabolism*<br>The major pathway of carisoprodol metabolism is via the liver by cytochrome enzyme CYP2C19 to form meprobamate. This enzyme exhibits genetic polymorphism (see Patients with Reduced CYP2C19 Activity below).

NDA/ANDA/BLA Number, Label Version Date	Drug	Therapeutic Area*	Biomarker†	Labeling Sections	Labeling Text‡
					Patients with Reduced CYP2C19 Activity<br>SOMA should be used with caution in patients with reduced CYP2C19 activity. Published studies indicate that patients who are poor CYP2C19 metabolizers have a 4-fold increase in exposure to carisoprodol, and concomitant 50% reduced exposure to meprobamate compared to normal CYP2C19 metabolizers. The prevalence of poor metabolizers in Caucasians and African Americans is approximately 3-5% and in Asians is approximately 15-20%.
020297, **09/14/2017**	Carvedilol	Cardiology	CYP2D6	Drug Interactions, Clinical Pharmacology	**7 DRUG INTERACTIONS**<br>**7.1 CYP2D6 Inhibitors and Poor Metabolizers**<br>Interactions of carvedilol with potent inhibitors of CYP2D6 isoenzyme (such as quinidine, fluoxetine, paroxetine, and propafenone) have not been studied, but these drugs would be expected to increase blood levels of the R(+) enantiomer of carvedilol [see Clinical Pharmacology (12.3)]. Retrospective analysis of side effects in clinical trials showed that poor 2D6 metabolizers had a higher rate of dizziness during up-titration, presumably resulting from vasodilating effects of the higher concentrations of the α-blocking R(+) enantiomer.<br><br>**12 CLINCIAL PHARMACOLOGY**<br>**12.3 Pharmacokinetics**<br>Carvedilol is subject to the effects of genetic polymorphism with poor metabolizers of debrisoquin (a marker for cytochrome P450 2D6) exhibiting 2- to 3-fold higher plasma concentrations of R(+)-carvedilol compared with extensive metabolizers. In contrast, plasma levels of S(-)-carvedilol are increased only about 20% to 25% in poor metabolizers, indicating this enantiomer is metabolized to a lesser extent by cytochrome P450 2D6 than R(+)-carvedilol. The pharmacokinetics of carvedilol do not appear to be different in poor metabolizers of S-mephenytoin (patients deficient in cytochrome P450 2C19).
02/25/2021, **213026**	Casimersen	Neurology	DMD	Indications and Usage, Adverse Reactions, Use in Specific Populations, Clinical Pharmacology, Clinical Studies	**1 INDICATIONS AND USAGE**<br>AMONDYS 45 is indicated for the treatment of Duchenne muscular dystrophy (DMD) in patients who have a confirmed mutation of the DMD gene that is amenable to exon 45 skipping. This indication is approved under accelerated approval based on an increase in dystrophin production in skeletal muscle observed in patients treated with AMONDYS 45 [see Clinical Studies (14)]. Continued approval for this indication may be contingent upon verification of a clinical benefit in confirmatory trials.<br><br>**6 ADVERSE REACTIONS**<br>**6.1 Clinical Trials Experience**<br>Because clinical trials are conducted under widely varying conditions, adverse reaction rates observed in clinical trials of a drug cannot be directly compared to rates in the clinical trials of another drug and may not reflect the rates observed in practice.<br>In the AMONDYS 45 clinical development program, 76 patients received at least one intravenous dose of AMONDYS 45 (30 mg/kg). All patients were male and had genetically confirmed Duchenne muscular dystrophy. Age at study entry was 7 to 20 years (mean 9.9 years). Most (88%) patients were White, and 9% were Asian.<br><br>**8 USE IN SPECIFIC POPULATIONS**<br>**8.4 Pediatric Use**<br>AMONDYS 45 is indicated for the treatment of DMD in patients who have a confirmed mutation of the DMD gene that is amenable to exon 45 skipping, including pediatric patients [see Clinical Studies (14)].<br><br>**12 CLINICAL PHARMACOLOGY**<br>**12.2 Pharmacodynamics**<br>In the interim analysis of muscle biopsy tissue obtained at baseline and at Week 48 from patients in Study 1, patients who received AMONDYS 45 (n=27) demonstrated a significant increase in skipping of exon 45 (p<0.001) compared to baseline, demonstrated by reverse transcription digital droplet polymerase chain reaction (RT-ddPCR). Patients who received placebo (n=16) did not demonstrate a significant increase in exon 45 skipping (p=0.808). The level of exon skipping is positively correlated with dystrophin protein expression [see Clinical Studies (14)].<br>In Study 1 [see Clinical Studies (14)], dystrophin levels as assessed by the Sarepta Western blot assay increased from 0.93% (SD 1.67) of normal at baseline to 1.74% (SD 1.97) of normal after 48 weeks of treatment with AMONDYS 45. The mean change from baseline in dystrophin after 48 weeks of treatment with AMONDYS 45 was 0.81% (SD 0.70) of normal levels (p<0.001). This increase in dystrophin protein expression after treatment with AMONDYS 45 positively correlated with the level of exon skipping. The mean change from baseline in dystrophin after 48 weeks of treatment with placebo was 0.22% (SD 0.49). Patients who received AMONDYS 45 showed a significantly greater increase in dystrophin protein levels from baseline to Week 48 compared to those who received placebo (mean difference of 0.59%; p = 0.004). Dystrophin levels assessed by Western blot can be meaningfully influenced by differences in sample processing, analytical technique, reference materials, and quantitation methodologies. Therefore, comparing dystrophin results from different assay protocols will require a standardized reference material and additional bridging studies. Correct localization of dystrophin to the sarcolemma in patients treated with AMONDYS 45 was demonstrated by immunofluorescence staining.<br>**12.3 Pharmacokinetics**<br>Specific Populations<br>*Age, Sex & Race*<br>The pharmacokinetics of AMONDYS 45 have been evaluated in male DMD patients 9 to 20 years of age. There is no experience with the use of AMONDYS 45 in DMD patients 65 years of age or older. AMONDYS 45 has not been studied in female patients. The potential impact of race on the pharmacokinetics of casimersen is unknown.<br>*Patients with Renal Impairment*

NDA/ANDA/BLA Number, Label Version Date	Drug	Therapeutic Area*	Biomarker†	Labeling Sections	Labeling Text‡
					The effect of renal impairment on the pharmacokinetics of casimersen was evaluated in nonDMD subjects aged 35 to 65 years with Stage 2 chronic kidney disease (CKD) (n=8, estimated glomerular filtration rate [eGFR] ≥60 and <90mL/min/1.73 m2) or Stage 3 CKD (n=8, eGFR ≥30 and <60mL/min/1.73 m2) and matched healthy subjects (n=9, eGFR ≥90 mL/min/1.73 m2). Subjects received a single 30 mg/kg intravenous dose of casimersen.<br>In subjects with Stage 2 or Stage 3 CKD, exposure (AUC) increased approximately 1.2-fold and 1.8-fold, respectively, compared with subjects with normal renal function. The Cmax in subjects with Stage 2 CKD was similar to Cmax in subjects with normal renal function; in subjects with Stage 3 CKD, there was a 1.2-fold increase in Cmax compared with subjects with normal renal function. The effect of Stage 4 or Stage 5 CKD on casimersen pharmacokinetics and safety has not been studied.<br>Estimated GFR values derived from MDRD equations and the threshold definitions for various CKD stages in otherwise healthy adults would not be generalizable to pediatric patients with DMD. Therefore, no specific dosage adjustment can be recommended for patients with renal impairment [see Use in Specific Populations (8.6)].<br><br>**14 CLINICAL STUDIES**<br>The effect of AMONDYS 45 on dystrophin production was evaluated in one study in male DMD patients who have a confirmed mutation of the DMD gene that is amenable to exon 45 skipping (Study 1; NCT02500381). (…)
020998, **05/03/2019**	**Celecoxib**	Rheumatology	CYP2C9	Dosage and Administration, Use in Specific Populations, Clinical Pharmacology	**2 DOSAGE AND ADMINISTRATION**<br>**2.7 Special Populations**<br>*Poor Metabolizers of CYP2C9 Substrates*<br>In adult patients who are known or suspected to be poor CYP2C9 metabolizers based on genotype or previous history/experience with other CYP2C9 substrates (such as warfarin, phenytoin), initiate treatment with half of the lowest recommended dose.<br>In patients with JRA who are known or suspected to be poor CYP2C9 metabolizers, consider using alternative treatments. [see Use in Specific populations (8.8), and Clinical Pharmacology (12.5)].<br><br>**8 USE IN SPECIFIC POPULATIONS**<br>**8.4 Pediatric Use**<br>Alternative therapies for treatment of JRA should be considered in pediatric patients identified to be CYP2C9 poor metabolizers [see Poor Metabolizers of CYP2C9 substrates (8.8)].<br><br>**8.8 Poor Metabolizers of CYP2C9 Substrates**<br>In patients who are known or suspected to be poor CYP2C9 metabolizers (i.e., CYP2C9*3/*3), based on genotype or previous history/experience with other CYP2C9 substrates (such as warfarin, phenytoin) administer CELEBREX starting with half the lowest recommended dose. Alternative management should be considered in JRA patients identified to be CYP2C9 poor metabolizers. [see Dosage and Administration (2.6) and Clinical Pharmacology (12.5)].<br><br>**12 CLINICAL PHARMACOLOGY**<br>**12.5 Pharmacogenomics**<br>CYP2C9 activity is reduced in individuals with genetic polymorphisms that lead to reduced enzyme activity, such as those homozygous for the CYP2C9*2 and CYP2C9*3 polymorphisms. Limited data from 4 published reports that included a total of 8 subjects with the homozygous CYP2C9*3/*3 genotype showed celecoxib systemic levels that were 3- to 7-fold higher in these subjects compared to subjects with CYP2C9*1/*1 or *I/*3 genotypes. The pharmacokinetics of celecoxib have not been evaluated in subjects with other CYP2C9 polymorphisms, such as *2, *5, *6, *9 and *11. It is estimated that the frequency of the homozygous *3/*3 genotype is 0.3% to 1.0% in various ethnic groups. [see Dosage and Administration (2.6), Use in Specific Populations (8.8)].
761097, 11/08/2022	**Cemiplimab-rwlc (1)**	Oncology	ALK	Indications and Usage, Clinical Studies	**1 INDICATIONS AND USAGE**<br>**1.3 Non-Small Cell Lung Cancer**<br>LIBTAYO in combination with platinum-based chemotherapy is indicated for the first-line treatment of adult patients with non-small cell lung cancer (NSCLC) with no EGFR, ALK or ROS1 aberrations and is:<br>• locally advanced where patients are not candidates for surgical resection or definitive chemoradiation or<br>• metastatic.<br>LIBTAYO as a single agent is indicated for the first-line treatment of adult patients with NSCLC whose tumors have high PD-L1 expression [Tumor Proportion Score (TPS) ≥ 50%] as determined by an FDA-approved test [see Dosage and Administration (2.1)]), with no EGFR, ALK or ROS1 aberrations, and is:<br>• locally advanced where patients are not candidates for surgical resection or definitive chemoradiation or<br>• metastatic.<br><br>**14 CLINICAL STUDIES**<br>**14.3 Non-Small Cell Lung Cancer (NSCLC)**<br>*First-line treatment of NSCLC with LIBTAYO in combination with platinum-based chemotherapy*<br>Patients with EGFR, ALK or ROS1 genomic tumor aberrations; a medical condition that required systemic immunosuppression; autoimmune disease that required systemic therapy within 2 years of treatment; or who had never smoked were ineligible. Patients with a history of brain metastases were eligible if they had been adequately treated and had neurologically returned to baseline for at least 2 weeks prior to randomization. (…)<br>*First-line treatment of NSCLC with LIBTAYO as a single agent*

Table of Pharmacogenomic Biomarkers in Drug Labeling
Last Updated: 06/2024

NDA/ANDA/BLA Number, Label Version Date	Drug	Therapeutic Area*	Biomarker†	Labeling Sections	Labeling Text‡
					Patients with EGFR, ALK or ROS1 genomic tumor aberrations; a medical condition that required systemic immunosuppression; autoimmune disease that required systemic therapy within 2 years of treatment; or who had never smoked were ineligible. Patients with a history of brain metastases were eligible if they had been adequately treated and had neurologically returned to baseline for at least 2 weeks prior to randomization. (…)
761097, 11/08/2022	Cemiplimab-rwlc (2)	Oncology	CD274 (PD-L1)	Indications and Usage, Dosage and Administration, Clinical Studies	**1 INDICATIONS AND USAGE**<br>**1.3 Non-Small Cell Lung Cancer**<br>LIBTAYO is indicated for the first-line treatment of patients with non-small cell lung cancer (NSCLC) whose tumors have high PD-L1 expression [Tumor Proportion Score (TPS) ≥ 50%] as determined by an FDA-approved test [see Dosage and Administration (2.1)]), with no EGFR, ALK or ROS1 aberrations, and is:<br>• locally advanced where patients are not candidates for surgical resection or definitive chemoradiation or<br>• metastatic.<br><br>**2 DOSAGE AND ADMINISTRATION**<br>**2.1 Patient Selection for NSCLC**<br>Select patients with locally advanced or metastatic NSCLC for treatment with LIBTAYO based on PD-L1 expression on tumor cells [see Clinical Studies (14.3)]. Information on FDA-approved tests for the detection of PD-L1 expression is available at: http://www.fda.gov/CompanionDiagnostics.<br><br>**14 CLINICAL STUDIES**<br>**14.3 Non-Small Cell Lung Cancer (NSCLC)**<br>*First-line treatment of NSCLC with LIBTAYO in combination with platinum-based chemotherapy*The efficacy of LIBTAYO was evaluated in Study 1624 (NCT03088540), a randomized, multicenter, open-label, active-controlled trial in 710 patients with locally advanced NSCLC who were not candidates for surgical resection or definitive chemoradiation, or with metastatic NSCLC.<br>Only patients whose tumors had high PD-L1 expression [Tumor Proportion Score (TPS) ≥ 50%] as determined by an immunohistochemistry assay using the PD-L1 IHC 22C3 pharmDx kit and who had not received prior systemic treatment for metastatic NSCLC were eligible. (…)<br>*First-line treatment of NSCLC with LIBTAYO as a single agent*<br>The efficacy of LIBTAYO was evaluated in Study 1624 (NCT03088540), a randomized, multicenter, open-label, active-controlled trial in 710 patients with locally advanced NSCLC who were not candidates for surgical resection or definitive chemoradiation, or with metastatic NSCLC.<br>Only patients whose tumors had high PD-L1 expression [Tumor Proportion Score (TPS) ≥ 50%] as determined by an immunohistochemistry assay using the PD-L1 IHC 22C3 pharmDx kit and who had not received prior systemic treatment for metastatic NSCLC were eligible. (…)
761097, 11/08/2022	Cemiplimab-rwlc (2)	Oncology	EGFR	Indications and Usage, Clinical Studies	**1 INDICATIONS AND USAGE**<br>**1.3 Non-Small Cell Lung Cancer**<br>LIBTAYO in combination with platinum-based chemotherapy is indicated for the first-line treatment of adult patients with non-small cell lung cancer (NSCLC) with no EGFR, ALK or ROS1 aberrations and is:<br>• locally advanced where patients are not candidates for surgical resection or definitive chemoradiation or<br>• metastatic.<br>LIBTAYO as a single agent is indicated for the first-line treatment of adult patients with NSCLC whose tumors have high PD-L1 expression [Tumor Proportion Score (TPS) ≥ 50%] as determined by an FDA-approved test [see Dosage and Administration (2.1)]), with no EGFR, ALK or ROS1 aberrations, and is:<br>• locally advanced where patients are not candidates for surgical resection or definitive chemoradiation or<br>• metastatic.<br><br>**14 CLINICAL STUDIES**<br>**14.3 Non-Small Cell Lung Cancer (NSCLC)**<br>*First-line treatment of NSCLC with LIBTAYO in combination with platinum-based chemotherapy*<br>Patients with EGFR, ALK or ROS1 genomic tumor aberrations; a medical condition that required systemic immunosuppression; autoimmune disease that required systemic therapy within 2 years of treatment; or who had never smoked were ineligible. Patients with a history of brain metastases were eligible if they had been adequately treated and had neurologically returned to baseline for at least 2 weeks prior to randomization. (…)<br>*First-line treatment of NSCLC with LIBTAYO as a single agent*<br>Patients with EGFR, ALK or ROS1 genomic tumor aberrations; a medical condition that required systemic immunosuppression; autoimmune disease that required systemic therapy within 2 years of treatment; or who had never smoked were ineligible. Patients with a history of brain metastases were eligible if they had been adequately treated and had neurologically returned to baseline for at least 2 weeks prior to randomization. (…)
761097, 11/08/2022	Cemiplimab-rwlc (4)	Oncology	ROS1	Indications and Usage, Clinical Studies	**1 INDICATIONS AND USAGE**<br>**1.3 Non-Small Cell Lung Cancer**<br>LIBTAYO in combination with platinum-based chemotherapy is indicated for the first-line treatment of adult patients with non-small cell lung cancer (NSCLC) with no EGFR, ALK or ROS1 aberrations and is:<br>• locally advanced where patients are not candidates for surgical resection or definitive chemoradiation or<br>• metastatic.<br>LIBTAYO as a single agent is indicated for the first-line treatment of adult patients with NSCLC whose tumors have high PD-L1 expression [Tumor Proportion Score (TPS) ≥ 50%] as determined by an FDA-approved test [see Dosage and Administration (2.1)]), with no EGFR, ALK or ROS1 aberrations, and is:<br>• locally advanced where patients are not candidates for surgical resection or definitive chemoradiation or<br>• metastatic.

* Therapeutic areas do not necessarily reflect the CDER review division.
† Representative biomarkers are listed based on standard nomenclature as per the Human Genome Organization (HUGO) symbol and/or simplified descriptors using other common conventions. Listed biomarkers do not necessarily reflect the terminology used in labeling. The term "Nonspecific" is provided when labeling does not explicitly identify the specific biomarker(s) or when the biomarker is represented by a molecular phenotype or gene signature, and in some cases the biomarker was inferred based on the labeling language.
‡ Referenced figures and tables may be found in the labeling available at Drugs@FDA.
Blue text represents the most recent addtions and/or changes since last posted version.

NDA/ANDA/BLA Number, Label Version Date	Drug	Therapeutic Area*	Biomarker†	Labeling Sections	Labeling Text‡
					**14 CLINICAL STUDIES**<br>**14.3 Non-Small Cell Lung Cancer (NSCLC)**<br>*First-line treatment of NSCLC with LIBTAYO in combination with platinum-based chemotherapy*<br>Patients with EGFR, ALK or ROS1 genomic tumor aberrations; a medical condition that required systemic immunosuppression; autoimmune disease that required systemic therapy within 2 years of treatment; or who had never smoked were ineligible. Patients with a history of brain metastases were eligible if they had been adequately treated and had neurologically returned to baseline for at least 2 weeks prior to randomization. (…)<br>*First-line treatment of NSCLC with LIBTAYO as a single agent*<br>Patients with EGFR, ALK or ROS1 genomic tumor aberrations; a medical condition that required systemic immunosuppression; autoimmune disease that required systemic therapy within 2 years of treatment; or who had never smoked were ineligible. Patients with a history of brain metastases were eligible if they had been adequately treated and had neurologically returned to baseline for at least 2 weeks prior to randomization. (…)
050585, **07/12/2018**	**Ceftriaxone (1)**	Infectious Diseases	G6PD	Warnings	**WARNINGS**<br>**Methemoglobinemia**<br>Cases of methemoglobinemia have been reported in association with local anesthetic use (e.g. lidocaine). Although all patients are at risk for methemoglobinemia, patients with glucose-6-phosphate dehydrogenase deficiency, congenital or idiopathic methemoglobinemia, cardiac or pulmonary compromise, infants under 6 months of age, and concurrent exposure to oxidizing agents or their metabolites are more susceptible to developing clinical manifestations of the condition. If local anesthetics must be used in these patients, close monitoring for symptoms and signs of methemoglobinemia is recommended. (…)
050585, **07/12/2018**	**Ceftriaxone (2)**	Infectious Diseases	Nonspecific (Congenital Methemoglobinemia)	Warnings	**WARNINGS**<br>**Methemoglobinemia**<br>Cases of methemoglobinemia have been reported in association with local anesthetic use (e.g. lidocaine). Although all patients are at risk for methemoglobinemia, patients with glucose-6-phosphate dehydrogenase deficiency, congenital or idiopathic methemoglobinemia, cardiac or pulmonary compromise, infants under 6 months of age, and concurrent exposure to oxidizing agents or their metabolites are more susceptible to developing clinical manifestations of the condition. If local anesthetics must be used in these patients, close monitoring for symptoms and signs of methemoglobinemia is recommended. (…)
205755, **03/05/2019**	**Ceritinib**	Oncology	ALK	Indications and Usage, Dosage and Administration, Warnings and Precautions, Adverse Reactions, Clinical Studies	**1 INDICATIONS AND USAGE**<br>ZYKADIA® is indicated for the treatment of patients with metastatic non-small cell lung cancer (NSCLC) whose tumors are anaplastic lymphoma kinase (ALK)-positive as detected by an FDA-approved test.<br><br>**2 DOSAGE AND ADMINISTRATION**<br>**2.1 Patient Selection**<br>Select patients for treatment of metastatic NSCLC with ZYKADIA based on the presence of ALK positivity in tumor specimens [see Indications and Usage (1) and Clinical Studies (14.1)].<br>Information on FDA-approved tests for the detection of ALK rearrangements in NSCLC is available at: http://www.fda.gov/CompanionDiagnostics<br><br>**5 WARNINGS AND PRECAUTIONS**<br>Data in the Warnings and Precautions section reflect the safety of ZYKADIA 750 mg daily under fasted conditions in 925 patients with ALK-positive NSCLC across a pool of seven clinical studies at systemic exposures similar to the recommended dose of 450 mg with food. In a dose optimization study (ASCEND-8), there were no clinically meaningful differences observed in the incidence of toxicities described in Warnings and Precautions between patients receiving 750 mg daily under fasted conditions and 450 mg with food, except for a reduction in gastrointestinal adverse reactions as described [see Warnings and Precautions (5.1)]. (…)<br><br>**6 ADVERSE REACTIONS**<br>**6.1 Clinical Trials Experience**<br>Because clinical trials are conducted under widely varying conditions, adverse reaction rates observed in the clinical trials of a drug cannot be directly compared to rates in the clinical trials of another drug and may not reflect the rates observed in practice.<br>The data in the Warnings and Precautions section reflect exposure to ZYKADIA 750 mg once daily in 925 patients with ALK-positive NSCLC across seven clinical studies, including ASCEND-4 and ASCEND-1, described below, a randomized active-controlled study, two single arm studies, and two dose-escalation studies. (…)<br>In ASCEND-8, a dose optimization study, ZYKADIA 450 mg daily with food (N = 89) was compared to 750 mg daily under fasted conditions (N = 90) in both previously treated and untreated patients with ALK-positive NSCLC. (…)<br>*Previously Untreated ALK-Positive Metastatic NSCLC*<br>The safety evaluation of ZYKADIA is based on ASCEND-4, an open-label, randomized, active-controlled multicenter study of 376 previously untreated ALK-positive NSCLC patients. Patients received ZYKADIA 750 mg daily (N=189) or chemotherapy plus maintenance chemotherapy (N=187). (…)<br>*Previously Treated ALK-Positive Metastatic NSCLC*<br>The safety evaluation of ZYKADIA is based on 255 ALK-positive patients in ASCEND-1 (246 patients with NSCLC and 9 patients with other cancers who received ZYKADIA at a dose of 750 mg daily). (See Tables 5 and 6) (…)<br><br>**14 CLINICAL STUDIES**<br>**14.1 Previously Untreated ALK-Positive Metastatic NSCLC**

NDA/ANDA/BLA Number, Label Version Date	Drug	Therapeutic Area*	Biomarker†	Labeling Sections	Labeling Text‡
					The efficacy of ZYKADIA for the treatment of patients with ALK-positive NSCLC who had not received prior systemic therapy for metastatic disease was established in an open-label, randomized, active-controlled, multicenter study (ASCEND-4, NCT01828099). Patients were required to have WHO performance status 0-2 and ALK-positive NSCLC as identified by the VENTANA ALK (D5F3) CDx Assay. (…)<br>**14.2 Previously Treated ALK-Positive Metastatic NSCLC**<br>The efficacy of ZYKADIA was established in a multicenter, single-arm, open-label clinical trial (ASCEND-1, NCT01283516). A total of 163 patients with metastatic ALK-positive NSCLC who progressed while receiving or were intolerant to crizotinib were enrolled. All patients received ZYKADIA at a dose of 750 mg once daily. (…)<br>The study population characteristics were: median age 52 years, age less than 65 (87%), female (54%), Caucasian (66%), Asian (29%), never or former smoker (97%), ECOG PS 0 or 1 (87%), progression on previous crizotinib (91%), number of prior therapies 2 or more (84%), and adenocarcinoma histology (93%). Sites of extra-thoracic metastases included brain (60%), liver (42%), and bone (42%). ALK-positivity was verified retrospectively by review of local test results for 99% of patients. (…)
761052, **12/06/2019**	**Cerliponase Alfa**	Inborn Errors of Metabolism	TPP1	Indications and Usage, Use in Specific Populations, Clinical Studies	**1 INDICATIONS AND USAGE**<br>Brineura is indicated to slow the loss of ambulation in symptomatic pediatric patients 3 years of age and older with late infantile neuronal ceroid lipofuscinosis type 2 (CLN2), also known as tripeptidyl peptidase 1 (TPP1) deficiency.<br><br>**8 USE IN SPECIFIC POPULATIONS**<br>**8.4 Pediatric Use**<br>Safety and effectiveness of Brineura have been established in pediatric patients 3 years of age and older. Pediatric use of Brineura to slow the loss of ambulation in symptomatic pediatric patients 3 years of age and older with late infantile neuronal ceroid lipofuscinosis type 2 (CLN2), also known as tripeptidyl peptidase 1 (TPP1) deficiency, is supported by a non-randomized single-arm dose escalation clinical study with extension in patients with CLN2 disease and compared to untreated patients with CLN2 disease from an independent natural history cohort [see Clinical Studies (14)]. Safety and effectiveness in patients less than 3 years of age have not been established.<br><br>**14 CLINICAL STUDIES**<br>The efficacy of Brineura was assessed over 96 weeks in a non-randomized single-arm dose escalation clinical study with extension in symptomatic pediatric patients with late infantile neuronal ceroid lipofuscinosis type 2 (CLN2) disease, confirmed by TPP1 deficiency. (…)<br>(…) Motor scores of the 22 Brineura-treated patients in the clinical study with extension were compared to scores of the independent natural history cohort that included 42 untreated patients who satisfied inclusion criteria for the clinical study. The results of logistic modeling with covariates (screening age, screening motor score, genotype: 0 key mutations (yes/no)), demonstrated the odds of Brineura-treated patients not having a decline by 96 weeks were 13 times the odds of natural history cohort patients not having a decline (Odds Ratio (95% CI): 13.1 (1.2, 146.9)). (…)<br>*Descriptive non-randomized comparison*<br>(…) Given the non-randomized study design, a Cox Proportional Hazards Model adjusted for age, initial motor score, and genotype was used to evaluate time to unreversed 2-category decline or unreversed score of 0 in the Motor domain. This model showed a lesser decrease in motor function in the Brineura-treated patients when compared to the natural history cohort (see Figure 7). (…)<br>*Motor Domain Scores: Matched Patients Only*<br>(…) To further assess efficacy, the 22 patients from the Brineura clinical study with a baseline combined Motor plus Language CLN2 score less than 6 were matched to 42 patients in the natural history cohort. Patients were matched based on the following covariates: baseline age at time of screening within 3 months, genotype (0, 1, or 2 key mutations), and baseline Motor domain CLN2 score at time of screening. (see Table 3) (…)
125084, **09/24/2021**	**Cetuximab (1)**	Oncology	EGFR	Indications and Usage, Dosage and Administration, Adverse Reactions, Clinical Studies	**1 INDICATIONS AND USAGE**<br>**1.2 K-Ras Wild-type, EGFR-expressing Colorectal Cancer**<br>ERBITUX is indicated for the treatment of K-Ras wild-type, epidermal growth factor receptor (EGFR)-expressing, metastatic colorectal cancer (mCRC) as determined by an FDA-approved test [see Dosage and Administration (2.2)]:<br>• in combination with FOLFIRI (irinotecan, fluorouracil, leucovorin) for first-line treatment,<br>• in combination with irinotecan in patients who are refractory to irinotecan-based chemotherapy,<br>• as a single-agent in patients who have failed oxaliplatin- and irinotecan-based chemotherapy or who are intolerant to irinotecan.Limitation of Use: Erbitux is not indicated for treatment of Ras-mutant colorectal cancer or when he results of the Ras mutation tests are unknown [see Warnings and Precautions (5.7), Clinical Studies (14.2)].<br><br>**2 DOSAGE AND ADMINISTRATION**<br>**2.1 Patient Selection**<br>Select patients with metastatic colorectal cancer (CRC) for treatment with ERBITUX based on the presence of:<br>• Ras wild-type, EGFR-expressing CRC [see Clinical Studies (14.2)], or<br>• BRAF V600E mutation-positive metastatic CRC [see Clinical Studies (14.3)] Information on FDA-approved tests for the detection of K-Ras or BRAF V600E mutations in CRC in patients with metastatic CRC is available at: http://www.fda.gov/CompanionDiagnostics.<br>**6 ADVERSE REACTIONS**<br>**6.1 Clinical Trials Experience**<br>K-Ras Wild-type, EGFR-expressing, Metastatic Colorectal Cancer (mCRC)<br>*In Combination with FOLFIRI*

NDA/ANDA/BLA Number, Label Version Date	Drug	Therapeutic Area*	Biomarker†	Labeling Sections	Labeling Text‡
					(…) The safety of a cetuximab product in combination with FOLFIRI or FOLFIRI alone was evaluated in CRYSTAL. The data described below reflect exposure to a cetuximab product in 667 patients with K-Ras wild-type, EGFR-expressing, mCRC. (See Table 4) (…)<br>*As Monotherapy*<br>(…) The safety of ERBITUX with best supportive care (BSC) or BSC alone was evaluated in Study CA225-025. The data described below reflect exposure to ERBITUX in 242 patients with K-Ras wild-type, EGFR-expressing, metastatic colorectal cancer (mCRC) [see Warnings and Precautions (5.8)]. ERBITUX was administered intravenously at the recommended dosage (400 mg/m2 initial dose, followed by 250 mg/m2 weekly). Patients received a median of 17 infusions (range 1 to 51) [see Clinical Studies (14.2)]. (See Table 5) (…)<br>In Combination with Irinotecan<br>ERBITUX at the recommended dosage was administered in combination with irinotecan in 354 patients with EGFR expressing recurrent mCRC in Study CP02-9923 and BOND. (…)<br><br>**14 CLINICAL STUDIES**<br>**14.2 K-Ras Wild-type, EGFR-expressing, Metastatic Colorectal Cancer (CRC)**<br>*In Combination with FOLFIRI*<br>CRYSTAL (NCT00154102) was a randomized, open-label, multicenter, study of 1217 patients with EGFR-expressing, mCRC. Patients were randomized (1:1) to receive either a cetuximab product in combination with FOLFIRI or FOLFIRI alone as first-line treatment. Stratification factors were Eastern Cooperative Oncology Group (ECOG) performance status (0 and 1 versus 2) and region (Western Europe versus Eastern Europe versus other). (…)<br>*As Monotherapy*<br>Study CA225-025 (NCT00079066) was a multicenter, open-label, randomized, clinical trial conducted in 572 patients with EGFR-expressing, previously treated, recurrent mCRC. (See Table 9) (…)<br>*In Combination with Irinotecan*<br>BOND was a multicenter, clinical trial conducted in 329 patients with EGFR-expressing recurrent mCRC. Tumor specimens were not available for testing for K-Ras mutation status. (…)
125084, **09/24/2021**	Cetuximab (2)	Oncology	RAS	Indications and Usage, Dosage and Administration, Warnings and Precautions, Adverse Reactions, Clinical Studies	**1 INDICATIONS AND USAGE**<br>**1.2 K-Ras Wild-type, EGFR-expressing Colorectal Cancer**<br>ERBITUX is indicated for the treatment of K-Ras wild-type, epidermal growth factor receptor (EGFR)-expressing, metastatic colorectal cancer (mCRC) as determined by an FDA-approved test [see Dosage and Administration (2.2)]:<br>• in combination with FOLFIRI (irinotecan, fluorouracil, leucovorin) for first-line treatment,<br>• in combination with irinotecan in patients who are refractory to irinotecan-based chemotherapy,<br>• as a single-agent in patients who have failed oxaliplatin- and irinotecan-based chemotherapy or who are intolerant to irinotecan.Limitation of Use: Erbitux is not indicated for treatment of Ras-mutant colorectal cancer or when he results of the Ras mutation tests are unknown [see Warnings and Precautions (5.7), Clinical Studies (14.2)].<br><br>**2 DOSAGE AND ADMINISTRATION**<br>**2.1 Patient Selection**<br>Select patients with metastatic colorectal cancer (CRC) for treatment with ERBITUX based on the presence of:<br>• Ras wild-type, EGFR-expressing CRC [see Clinical Studies (14.2)], or<br>• BRAF V600E mutation-positive metastatic CRC [see Clinical Studies (14.3)] Information on FDA-approved tests for the detection of K-Ras or BRAF V600E mutations in CRC in patients with metastatic CRC is available at: http://www.fda.gov/CompanionDiagnostics.**5 WARNINGS AND PRECAUTIONS**<br>**5.7 Increased Tumor Progression, Increased Mortality, or Lack of Benefit in Patients with Ras-Mutant mCRC**<br>Erbitux is not indicated for the treatment of patients with colorectal cancer that harbor somatic mutations in exon 2 (codons 12 and 13), exon 3 (codons 59 and 61), and exon 4 (codons 117 and 146) of either K-Ras or N-Ras and hereafter is referred to as “Ras” or when the Ras status is unknown.<br>Retrospective subset analyses of Ras-mutant and wild-type populations across several randomized clinical trials including Study 4 were conducted to investigate the role of Ras mutations on the clinical effects of anti-EGFR-directed monoclonal antibodies. Use of cetuximab in patients with Ras mutations resulted in no clinical benefit with treatment related toxicity. [See Indications and Usage (1.2), Clinical Pharmacology (12.1), Clinical Studies (14.2).]<br><br>**6 ADVERSE REACTIONS**<br>**6.1 Clinical Trials Experience**<br>K-Ras Wild-type, EGFR-expressing, Metastatic Colorectal Cancer (mCRC)<br>*In Combination with FOLFIRI*<br>(…) The safety of a cetuximab product in combination with FOLFIRI or FOLFIRI alone was evaluated in CRYSTAL. The data described below reflect exposure to a cetuximab product in 667 patients with K-Ras wild-type, EGFR-expressing, mCRC. (See Table 4) (…)<br>*As Single-Agent*<br>The safety of ERBITUX with best supportive care (BSC) or BSC alone was evaluated in Study CA225-025. The data described below reflect exposure to ERBITUX in 242 patients with K-Ras wild-type, EGFR-expressing, metastatic colorectal cancer (mCRC) [see Warnings and Precautions (5.8)]. (See Table 5) (…)<br><br>**14 CLINICAL STUDIES**<br>**14.2 K-Ras Wild-type, EGFR-expressing, Metastatic Colorectal Cancer (CRC)**<br>*In Combination with FOLFIRI*

* Therapeutic areas do not necessarily reflect the CDER review division.
† Representative biomarkers are listed based on standard nomenclature as per the Human Genome Organization (HUGO) symbol and/or simplified descriptors using other common conventions. Listed biomarkers do not necessarily reflect the terminology used in labeling. The term “Nonspecific” is provided when labeling does not explicitly identify the specific biomarker(s) or when the biomarker is represented by a molecular phenotype or gene signature, and in some cases the biomarker was inferred based on the labeling language.
‡ Referenced figures and tables may be found in the labeling available at Drugs@FDA.
Blue text represents the most recent addtions and/or changes since last posted version.

NDA/ANDA/BLA Number, Label Version Date	Drug	Therapeutic Area*	Biomarker†	Labeling Sections	Labeling Text‡
					(…) K-Ras mutation status was available for 89% of the patients: 63% had K-Ras wild-type tumors and 37% had K-Ras mutant tumors where testing assessed for the following somatic mutations in codons 12 and 13 (exon 2): G12A, G12D, G12R, G12C, G12S, G12V, G13D. Baseline characteristics and demographics in the K-Ras wild-type subset were similar to that seen in the overall population. (…)<br>(…) Results of the planned PFS and ORR analysis in all randomized patients and post-hoc PFS and ORR analysis in subgroups of patients defined by K-Ras mutation status, and post-hoc analysis of updated OS based on additional followup (1000 events) in all randomized patients and in subgroups of patients defined by K-Ras mutation status are presented in Table 8 and Figure 2. The treatment effect in the all-randomized population for PFS was driven by treatment effects limited to patients who have K-Ras wild-type tumors. There is no evidence of effectiveness in the subgroup of patients with K-Ras mutant tumors. (See Table 8 and Figure 2) (…)<br>*As Single-Agent*<br>(…) K-Ras status was available for 79% of the patients: 54% had K-Ras wild-type tumors and 46% had K-Ras mutant tumors where testing assessed for the following somatic mutations in codons 12 and 13 (exon 2): G12A, G12D, G12R, G12C, G12S, G12V, G13D. (See Table 9 and Figure 3) (…)<br>*In Combination with Irinotecan*<br>BOND was a multicenter, clinical trial conducted in 329 patients with EGFR-expressing recurrent mCRC. Tumor specimens were not available for testing for K-Ras mutation status. (…)
**125084, 09/24/2021**	**Cetuximab (3)**	Oncology	BRAF	Indications and Usage, Dosage and Administration, Adverse Reactions, Use in Specific Populations, Clinical Studies	**1 INDICATIONS AND USAGE**<br>**1.3 BRAF V600E Mutation-Positive Metastatic Colorectal Cancer (CRC)**<br>ERBITUX is indicated, in combination with encorafenib, for the treatment of adult patients with metastatic colorectal cancer (CRC) with a BRAF V600E mutation, as detected by an FDA-approved test, after prior therapy [see Dosage and Administration (2.3)].<br><br>**2 DOSAGE AND ADMINISTRATION**<br>**2.1 Patient Selection**<br>Select patients with metastatic colorectal cancer (CRC) for treatment with ERBITUX based on the presence of:<br>• Ras wild-type, EGFR-expressing CRC [see Clinical Studies (14.2)], or<br>• BRAF V600E mutation-positive metastatic CRC [see Clinical Studies (14.3)] Information on FDA-approved tests for the detection of K-Ras or BRAF V600E mutations in CRC in patients with metastatic CRC is available at: http://www.fda.gov/CompanionDiagnostics.<br><br>**6 ADVERSE REACTIONS**<br>BRAF V600E Mutation-Positive Metastatic Colorectal Cancer (CRC) in Combination with Encorafenib<br>The safety of ERBITUX (400 mg/m 2 initial dose, followed by 250 mg/m 2 weekly) in combination with encorafenib (300 mg once daily) was evaluated in 216 patients with BRAF V600E mutation-positive metastatic CRC in a randomized, openlabel, active-controlled trial (BEACON CRC). (…)<br><br>**8 USE IN SPECIFIC POPULATIONS**<br>**8.5 Geriatric Use**<br>Of the 1662 patients with advanced colorectal cancer who received ERBITUX with irinotecan, with FOLFIRI or as singleagent in six studies (BOND, IMCL-CP02-9923, IMCL-CP02-0141, IMCL-CP02-0144, CA225-025 and CRYSTAL), 35% of patients were 65 years of age or older. No overall differences in safety or efficacy were observed between these patients and younger patients.<br>Clinical studies of ERBITUX conducted in patients with head and neck cancer did not include sufficient number of subjects aged 65 and over to determine whether they respond differently from younger subjects.<br>Of the 216 patients with BRAF V600E mutation positive metastatic CRC who received ERBITUX in combination with encorafenib 300 mg, once daily, 29% were 65 years of age to up to 75 years of age, while 20 (9%) were 75 years of age and over [see Clinical Studies (14.3)].<br>No overall differences in the safety or effectiveness of ERBITUX plus encorafenib were observed in elderly patients as compared to younger patients.<br><br>**14 CLINICAL STUDIES**<br>**14.3 BRAF V600E Mutation-Positive Metastatic Colorectal Cancer (CRC)**<br>ERBITUX in combination with encorafenib was evaluated in a randomized, active-controlled, open label, multicenter trial (BEACON CRC; NCT02928224). Eligible patients were required to have BRAF V600E mutation-positive metastatic CRC, as detected using the Qiagen therascreen BRAF V600E RGQ polymerase chain reaction (PCR) Kit, with disease progression after 1 or 2 prior regimens. (See Table 12 and Figure 4)
**020989, 12/08/2006**	**Cevimeline**	Dental	CYP2D6	Precautions	**PRECAUTIONS**<br>(…) Cevimeline should be used with caution in individuals known or suspected to be deficient in CYP2D6 activity, based on previous experience, as they may be at a higher risk of adverse events. In an in vitro study, cytochrome P450 isozymes 1A2, 2A6, 2C9, 2C19, 2D6, 2E1, and 3A4 were not inhibited by exposure to cevimeline. (…)
**006002, 10/24/2018**	**Chloroquine**	Infectious Diseases	G6PD	Precautions, Adverse Reactions	**PRECAUTIONS**<br>*Hematological Effects/Laboratory Tests*<br>Complete blood cell counts should be made periodically if patients are given prolonged therapy. If any severe blood disorder appears which is not attributable to the disease under treatment, discontinuance of the drug should be considered.<br>The drug should be administered with caution to patients having G-6-PD (glucose-6 phosphate dehydrogenase) deficiency.<br><br>**ADVERSE REACTIONS**

* Therapeutic areas do not necessarily reflect the CDER review division.
† Representative biomarkers are listed based on standard nomenclature as per the Human Genome Organization (HUGO) symbol and/or simplified descriptors using other common conventions. Listed biomarkers do not necessarily reflect the terminology used in labeling. The term “Nonspecific” is provided when labeling does not explicitly identify the specific biomarker(s) or when the biomarker is represented by a molecular phenotype or gene signature, and in some cases the biomarker was inferred based on the labeling language.
‡ Referenced figures and tables may be found in the labeling available at Drugs@FDA.
Blue text represents the most recent addtions and/or changes since last posted version.

NDA/ANDA/BLA Number, Label Version Date	Drug	Therapeutic Area*	Biomarker†	Labeling Sections	Labeling Text‡
					Blood and lymphatic system disorders: Pancytopenia, aplastic anemia, reversible agranulocytosis, thrombocytopenia and neutropenia. Hemolytic anemia in G6PD deficient patients (see PRECAUTIONS).
**011641, 02/01/2011**	**Chlorpropamide**	Endocrinology	G6PD	Precautions	**PRECAUTIONS**<br>*Hemolytic Anemia*<br>Treatment of patients with glucose 6-phosphate dehydrogenase (G6PD) deficiency with sulfonylurea agents can lead to hemolytic anemia. Because DIABINESE belongs to the class of sulfonylurea agents, caution should be used in patients with G6PD deficiency and a non-sulfonylurea alternative should be considered. In post marketing reports, hemolytic anemia has also been reported in patients who did not have known G6PD deficiency.
**009435, 11/02/2018**	**Chloroprocaine (1)**	Anesthesiology	G6PD	Warnings	**WARNINGS**<br>**Methemoglobinemia**<br>Cases of methemoglobinemia have been reported in association with local anesthetic use. Although all patients are at risk for methemoglobinemia, patients with glucose-6-phosphate dehydrogenase deficiency, congenital or idiopathic methemoglobinemia, cardiac or pulmonary compromise, infants under 6 months of age, and concurrent exposure to oxidizing agents or their metabolites are more susceptible to developing clinical manifestations of the condition. If local anesthetics must be used in these patients, close monitoring for symptoms and signs of methemoglobinemia is recommended. (…)
**009435, 11/02/2018**	**Chloroprocaine (2)**	Anesthesiology	Nonspecific (Congenital Methemoglobinemia)	Warnings	**WARNINGS**<br>**Methemoglobinemia**<br>Cases of methemoglobinemia have been reported in association with local anesthetic use. Although all patients are at risk for methemoglobinemia, patients with glucose-6-phosphate dehydrogenase deficiency, congenital or idiopathic methemoglobinemia, cardiac or pulmonary compromise, infants under 6 months of age, and concurrent exposure to oxidizing agents or their metabolites are more susceptible to developing clinical manifestations of the condition. If local anesthetics must be used in these patients, close monitoring for symptoms and signs of methemoglobinemia is recommended. (…)
**205750, 10/22/2020**	**Cholic Acid**	Inborn Errors of Metabolism	AMACR, AKR1D1, CYP7A1, CYP27A1, DHCR7, HSD3B2 (Bile Acid Synthesis Disorders)	Indications and Usage, Dosage and Administration, Warnings and Precautions, Adverse Reactions, Use in Specific Populations, Clinical Studies	**1 INDICATIONS AND USAGE**<br>**1.1 Bile Acid Synthesis Disorders Due to Single Enzyme Defects**<br>CHOLBAM is indicated for the treatment of bile acid synthesis disorders due to single enzyme defects (SEDs).<br>**1.3 Limitations of Use**<br>The safety and effectiveness of CHOLBAM on extrahepatic manifestations of bile acid synthesis disorders due to SEDs or PDs including Zellweger spectrum disorders have not been established.<br><br>**2 DOSAGE AND ADMINISTRATION**<br>**2.1 Dosage Regimen for Bile Acid Synthesis Disorders Due to SEDs and PDs Including Zellweger Spectrum Disorders**<br>The recommended dosage of CHOLBAM is 10 to 15 mg/kg administered orally once daily or in two divided doses, in pediatric patients and adults. (…)<br>**2.2 Treatment Monitoring**<br>(…) Assessment of serum or urinary bile acid levels using mass spectrometry is used in the diagnosis of bile acid synthesis disorders due to SEDs and PDs including Zellweger spectrum disorders. The utility of bile acid measurements in monitoring the clinical course of patients and in decisions regarding dose adjustment has not been demonstrated.<br><br>**5 WARNINGS AND PRECAUTIONS**<br>**5.1 Exacerbation of Liver Impairment**<br>In clinical trials, evidence of liver impairment was present before treatment with CHOLBAM in approximately 86% (44/51) of patients with bile acid synthesis disorders due to SEDs and in approximately 50% (14/28) of patients with PDs including Zellweger spectrum disorders. Five of the patients (3SED and 2 PD) with liver impairment at baseline experienced worsening serum transaminases, elevated bilirubin values, or worsening cholestasis on liver biopsy following treatment. Five additional patients (2 SED and 3 PD) who did not have baseline cholestasis experienced exacerbation of their liver disease while on treatment. In patients with cirrhosis, cases of severe hepatotoxicity have also been observed following postmarket use of CHOLBAM. Exacerbation of liver impairment by CHOLBAM in these patients cannot be ruled out.<br>Six patients with SEDs underwent liver transplant, including four patients diagnosed with AKR1D1 deficiency, one with 3β-HSD deficiency, and one with CYP7A1 deficiency. (…)<br><br>**6 ADVERSE REACTIONS**<br>**6.1 Clinical Trials Experience**<br>Clinical safety experience with CHOLBAM consists of:<br>• Trial 1: a non-randomized, open-label, single-arm trial of 50 patients with bile acid synthesis disorders due to SEDs and 29 patients with PDs including Zellweger spectrum disorders. Safety data are available over the 18 years of the trial.<br>• Trial 2: an extension trial of 12 new patients (10 SED and 2 PD) along with 31 (21 SED and 10 PD) patients who rolled over from Trial 1. Safety data are available for 3 years and 11 months of treatment. (…)<br><u>Deaths</u><br>In Trial 1, among the 50 patients with SEDs, 5 patients aged 1 year or less died, which included three patients originally diagnosed with AKR1D1 deficiency, one with 3β-HSD deficiency and one with CYP7A1 deficiency. The cause of death was attributed to progression of underlying liver disease in every patient.<br>Of 29 patients in Trial 1 with PDs including Zellweger spectrum disorders, 12 patients between the ages of 7 months and 2.5 years died. In the majority of these patients (8/12), the cause of death was attributed to progression of underlying liver disease or to a worsening of their primary illness.

* Therapeutic areas do not necessarily reflect the CDER review division.
† Representative biomarkers are listed based on standard nomenclature as per the Human Genome Organization (HUGO) symbol and/or simplified descriptors using other common conventions. Listed biomarkers do not necessarily reflect the terminology used in labeling. The term “Nonspecific” is provided when labeling does not explicitly identify the specific biomarker(s) or when the biomarker is represented by a molecular phenotype or gene signature, and in some cases the biomarker was inferred based on the labeling language.
‡ Referenced figures and tables may be found in the labeling available at Drugs@FDA.
Blue text represents the most recent addtions and/or changes since last posted version.

NDA/ANDA/BLA Number, Label Version Date	Drug	Therapeutic Area*	Biomarker†	Labeling Sections	Labeling Text‡
					Two additional patients in Trial 1 (1 SED and 1 PD) died who had been off study medication for more than one year with the cause of death most likely being a progression of underlying liver disease. Of the patients who died with disease progression, laboratory testing showed abnormal serum transaminases, bilirubin, or cholestasis on liver biopsy suggesting worsening of their underlying cholestasis.<br>In Trial 2, among the 31 patients with SED, two patients (1 new patient and 1 who rolled over from Trial 1) died. The cause of death in both cases was unrelated to their primary treatment or progression of their underlying liver disease.<br>Of the 12 patients with PD in Trial 2, four patients died between the ages of 4 and 8 years (1 new patient and 3 who rolled over from Trial 1). The cause of death in three of these patients was attributed to progression of underlying liver disease or to a worsening of their primary illness.<br>Worsening of Liver Impairment<br>Seven patients in Trial 1 (4 SED and 3 PD) and 3 patients in Trial 2 (1 SED and 2 PD) experienced worsening serum transaminases, elevated bilirubin values, or worsening cholestasis on liver biopsy during treatment [see Warnings and Precautions (5.1)].<br><br>**8 USE IN SPECIFIC POPULATIONS**<br>**8.4 Pediatric Use**<br>The safety and effectiveness of CHOLBAM have been established in pediatric patients 3 weeks of age and older for the treatment of bile acid synthesis disorders due to SEDs and for adjunctive treatment of patients with PDs including Zellweger spectrum disorders who exhibit manifestations of liver disease, steatorrhea, or complications from decreased fat-soluble vitamin absorption [see Clinical Studies (14)].<br><br>**14 CLINICAL STUDIES**<br>**14.1 Bile Acid Synthesis Disorders due to Single Enzyme Defects**<br>The effectiveness of CHOLBAM at dosages of 10 to 15 mg/kg per day in patients with SEDs was assessed in:<br>• Trial 1: a non-randomized, open-label, single-arm trial in 50 patients over an 18-year period<br>• Trial 2: an extension trial of 12 new patients along with 21 patients who rolled over from Trial 1 (n=33 total). Efficacy data are available for 21 months of treatment.<br>• A published case series of 15 patients with SEDs and 3 patients with PDs. (…)<br>Trials 1 and 2<br>On average, patients were 4 years of age at the start of cholic acid treatment (range three weeks to 36 years). The majority of patients were treated for an average of 310 weeks (6 years). Patient ages at the end of treatment ranged from 19 to 36 years.<br>Overall, 28 of 44 patients (64%) were responders. The breakdown by defect type is as follows: (See Table 4).<br>Among SED responsive patients, 45% of the responders met the two clinical criteria plus 1 to 3 laboratory criteria and 55% met the weight criteria.<br>Only six patients had pre- and post-treatment liver biopsies in Trial 1. Where biopsies were available, pre-treatment biopsies showed varying degrees of inflammation, bridging fibrosis, and giant cell formation. Post-treatment biopsies generally showed reduced or absent inflammation and reduced or absent giant cell formation. Fibrosis remained but did not progress.<br>It is difficult to evaluate long term survival in patients with SEDs since there is little natural history survival data for comparison. Overall, 41 of 62 (67%) patients with SEDs survived greater than 3 years from trial entry. Thirteen of these 41 patients (32%) survived for 10 to 24 years on treatment.<br>Four patients in Trial 1 underwent liver transplant, including two patients diagnosed with AKR1D1 deficiency, one with 3β-HSD deficiency, and one with CYP7A1 deficiency and two patients in Trial 2, both with AKR1D1.<br>CHOLBAM's effects on extrahepatic manifestations of SEDs, such as neurologic symptoms have not been established.<br>Case Series<br>A published report of a case series described 15 patients with SEDs; thirteen were diagnosed with 3β-HSD deficiency and two with AKR1D1 deficiency by mass spectrometry and gene sequencing. All patients were treated with cholic acid with a median duration of treatment of 12.4 years (range 5.6 to 15 years). (…)<br>Of the 8 patients who received ursodeoxycholic acid initially, the six with 3β-HSD deficiency demonstrated mild clinical improvement. Following treatment with cholic acid, all patients experienced resolution of their pre-existing jaundice and steatorrhea, and all but one experienced resolution of hepatosplenomegaly. Weight and height improved, and sexual maturation progressed normally in all patients. Liver biopsies were performed in 14 patients after at least 5 years of cholic acid treatment and all showed resolution of cholestasis. In one patient with 3βHSD deficiency, biliary bile acid analysis while on cholic acid therapy showed enrichment of the bile with cholic acid.
**018057, 02/22/2019**	**Cisplatin**	Oncology	TPMT	Adverse Reactions	**6 ADVERSE REACTIONS**<br>*Ototoxicity*<br>(…) Genetic factors (e.g., variants in the thiopurine S-methyltransferase [TPMT] gene) may contribute to cisplatin-induced ototoxicity; although this association has not been consistent across populations and study designs.
**020822, 01/11/2019**	**Citalopram (1)**	Psychiatry	CYP2C19	Dosage and Administration, Warnings, Clinical Pharmacology	**DOSAGE AND ADMINISTRATION**<br>*Special Populations*<br>20 mg/day is the maximum recommended dose for patients who are greater than 60 years of age, patients with hepatic impairment, and for CYP2C19 poor metabolizers or those patients taking cimetidine or another CYP2C19 inhibitor. (see WARNINGS)<br><br>**WARNINGS**<br>*QT-Prolongation and Torsade de Pointes*<br>The citalopram dose should be limited in certain populations. The maximum dose should be limited to 20 mg/day in patients who are CYP2C19 poor metabolizers or those patients who may be taking concomitant cimetidine or another CYP2C19 inhibitor, since higher citalopram exposures would be expected.

* Therapeutic areas do not necessarily reflect the CDER review division.
† Representative biomarkers are listed based on standard nomenclature as per the Human Genome Organization (HUGO) symbol and/or simplified descriptors using other common conventions. Listed biomarkers do not necessarily reflect the terminology used in labeling. The term "Nonspecific" is provided when labeling does not explicitly identify the specific biomarker(s) or when the biomarker is represented by a molecular phenotype or gene signature, and in some cases the biomarker was inferred based on the labeling language.
‡ Referenced figures and tables may be found in the labeling available at Drugs@FDA.
Blue text represents the most recent addtions and/or changes since last posted version.

Table of Pharmacogenomic Biomarkers in Drug Labeling
Last Updated: 06/2024

NDA/ANDA/BLA Number, Label Version Date	Drug	Therapeutic Area*	Biomarker†	Labeling Sections	Labeling Text‡
					**CLINICAL PHARMACOLOGY**<br>**Pharmacokinetics**<br>*Population Subgroups*<br>(…) CYP2C19 poor metabolizers – In CYP2C19 poor metabolizers, citalopram steady state Cmax and AUC was increased by 68% and 107%, respectively. Celexa 20 mg/day is the maximum recommended dose in CYP2C19 poor metabolizers due to the risk of QT prolongation (see WARNINGS and DOSAGE AND ADMINISTRATION).<br>CYP2D6 poor metabolizers - Citalopram steady state levels were not significantly different in poor metabolizers and extensive metabolizers of CYP2D6.
**020822, 01/11/2019**	Citalopram (2)	Psychiatry	CYP2D6	Clinical Pharmacology	**CLINICAL PHARMACOLOGY**<br>**Pharmacokinetics**<br>*Population Subgroups*<br>CYP2D6 poor metabolizers - Citalopram steady state levels were not significantly different in poor metabolizers and extensive metabolizers of CYP2D6.<br>*Drug-Drug Interactions*<br>Coadministration of a drug that inhibits CYP2D6 with Celexa is unlikely to have clinically significant effects on citalopram metabolism, based on the study results in CYP2D6 poor metabolizers.
**202067, 06/15/2018**	Clobazam	Neurology	CYP2C19	Dosage and Administration, Use in Specific Populations, Clinical Pharmacology	**2 DOSAGE AND ADMINISTRATION**<br>**2.5 Dosage Adjustments in CYP2C19 Poor Metabolizers**<br>In CYP2C19 poor metabolizers, levels of N-desmethylclobazam, clobazam's active metabolite, will be increased. Therefore, in patients known to be CYP2C19 poor metabolizers, the starting dose should be 5 mg/day and dose titration should proceed slowly according to weight, but to half the dose presented in Table 1, as tolerated. If necessary and based upon clinical response, an additional titration to the maximum dose (20 mg/day or 40 mg/day, depending on the weight group) may be started on day 21 [see Use in Specific Populations (8.6), Clinical Pharmacology (12.5)].<br><br>**8 USE IN SPECIFIC POPULATIONS**<br>**8.6 CYP2C19 Poor Metabolizers**<br>Concentrations of clobazam's active metabolite, N-desmethylclobazam, are higher in CYP2C19 poor metabolizers than in extensive metabolizers. For this reason, dosage modification is recommended [see Dosage and Administration (2.5), Clinical Pharmacology (12.3)].<br><br>**12 CLINICAL PHARMACOLOGY**<br>**12.3 Pharmacokinetics**<br>*Metabolism and Excretion*<br>(…) The polymorphic CYP2C19 is the major contributor to the metabolism of the pharmacologically active N-desmethylclobazam [see Clinical Pharmacology (12.5)]. In CYP2C19 poor metabolizers, levels of N-desmethylclobazam were 5-fold higher in plasma and 2- to 3-fold higher in the urine than in CYP2C19 extensive metabolizers.<br>**12.5 Pharmacogenomics**<br>The polymorphic CYP2C19 is the main enzyme that metabolizes the pharmacologically active N-desmethylclobazam. Compared to CYP2C19 extensive metabolizers, N-desmethylclobazam AUC and Cmax are approximately 3-5 times higher in poor metabolizers (e.g., subjects with *2/*2 genotype) and 2 times higher in intermediate metabolizers (e.g., subjects with *1/*2 genotype). The prevalence of CYP2C19 poor metabolism differs depending on racial/ethnic background. Dosage in patients who are known CYP2C19 poor metabolizers may need to be adjusted [see Dosage and Administration (2.5)].<br>The systemic exposure of clobazam is similar for both CYP2C19 poor and extensive metabolizers.
**019906, 05/10/2019**	Clomipramine	Psychiatry	CYP2D6	Precautions	**PRECAUTIONS**<br>*Drugs Metabolized by P450 2D6*<br>The biochemical activity of the drug metabolizing isozyme cytochrome P450 2D6 (debrisoquin hydroxylase) is reduced in a subset of the Caucasian population (about 7% to 10% of Caucasians are so-called "poor metabolizers"); reliable estimates of the prevalence of reduced P450 2D6 isozyme activity among Asian, African and other populations are not yet available. Poor metabolizers have higher than expected plasma concentrations of tricyclic antidepressants (TCAs) when given usual doses. Depending on the fraction of drug metabolized by P450 2D6, the increase in plasma concentration may be small, or quite large (8 fold increase in plasma AUC of the TCA). (…)
**020839, 05/17/2019**	Clopidogrel	Cardiology	CYP2C19	Boxed Warning, Warnings and Precautions, Clinical Pharmacology	**BOXED WARNING**<br>WARNING<br>*Diminished antiplatelet effect in patients with two loss-of-function alleles of the CYP2C19 gene*<br>The effectiveness of Plavix results from its antiplatelet activity, which is dependent on its conversion to an active metabolite by the cytochrome P450 (CYP) system, principally CYP2C19 [see Warnings and Precautions (5.1), Clinical Pharmacology (12.3)]. Plavix at recommended doses forms less of the active metabolite and so has a reduced effect on platelet activity in patients who are homozygous for nonfunctional alleles of the CYP2C19 gene, (termed "CYP2C19 poor metabolizers"). Tests are available to identify patients who are CYP2C19 poor metabolizers [see Clinical Pharmacology (12.5)]. Consider use of another platelet P2Y12 inhibitor in patients identified as CYP2C19 poor metabolizers.<br><br>**5 WARNINGS AND PRECAUTIONS**<br>**5.1 Diminished Antiplatelet Activity in Patients with Impaired CYP2C19 Function**<br>Clopidogrel is a prodrug. Inhibition of platelet aggregation by clopidogrel is achieved through an active metabolite. The metabolism of clopidogrel to its active metabolite can be impaired by genetic variations in CYP2C19 [see Boxed Warning].

* Therapeutic areas do not necessarily reflect the CDER review division.
† Representative biomarkers are listed based on standard nomenclature as per the Human Genome Organization (HUGO) symbol and/or simplified descriptors using other common conventions. Listed biomarkers do not necessarily reflect the terminology used in labeling. The term "Nonspecific" is provided when labeling does not explicitly identify the specific biomarker(s) or when the biomarker is represented by a molecular phenotype or gene signature, and in some cases the biomarker was inferred based on the labeling language.
‡ Referenced figures and tables may be found in the labeling available at Drugs@FDA.
Blue text represents the most recent addtions and/or changes since last posted version.

NDA/ANDA/BLA Number, Label Version Date	Drug	Therapeutic Area*	Biomarker†	Labeling Sections	Labeling Text‡
					**12 CLINICAL PHARMACOLOGY**<br>**12.5 Pharmacogenomics**<br>CYP2C19 is involved in the formation of both the active metabolite and the 2-oxo-clopidogrel intermediate metabolite. Clopidogrel active metabolite pharmacokinetics and antiplatelet effects, as measured by ex vivo platelet aggregation assays, differ according to CYP2C19 genotype. Patients who are homozygous for nonfunctional alleles of the CYP2C19 gene are termed "CYP2C19 poor metabolizers". Approximately 2% of White and 4% of Black patients are poor metabolizers; the prevalence of poor metabolism is higher in Asian patients (e.g., 14% of Chinese). Tests are available to identify patients who are CYP2C19 poor metabolizers.<br>A crossover study in 40 healthy subjects, 10 each in the four CYP2C19 metabolizer groups, evaluated pharmacokinetic and antiplatelet responses using 300 mg followed by 75 mg per day and 600 mg followed by 150 mg per day, each for a total of 5 days. Decreased active metabolite exposure and diminished inhibition of platelet aggregation were observed in the poor metabolizers as compared to the other groups. (See Table 3)
**019758, 02/23/2017**	**Clozapine**	Psychiatry	CYP2D6	Dosage and Administration, Use in Specific Populations, Clinical Pharmacology	**2 DOSAGE AND ADMINISTRATION**<br>**2.7 Renal or Hepatic Impairment or CYP2D6 Poor Metabolizers**<br>It may be necessary to reduce the CLOZARIL dose in patients with significant renal or hepatic impairment, or in CYP2D6 poor metabolizers [see Use in Specific Populations (8.6, 8.7)].<br><br>**8 USE IN SPECIFIC POPULATIONS**<br>**8.7 CYP2D6 Poor Metabolizers**<br>Dose reduction may be necessary in patients who are CYP2D6 poor metabolizers. Clozapine concentrations may be increased in these patients, because clozapine is almost completely metabolized and then excreted [see Dosage and Administration (2.7), Clinical Pharmacology (12.3)].<br><br>**12 CLINICAL PHARMACOLOGY**<br>**12.3 Pharmacokinetics**<br>*CYP2D6 Poor Metabolizers*<br>A subset (3%–10%) of the population has reduced activity of CYP2D6 (CYP2D6 poor metabolizers). These individuals may develop higher than expected plasma concentrations of clozapine when given usual doses.
206192, 10/28/2022	**Cobimetinib**	Oncology	BRAF	Indications and Usage, Dosage and Administration, Adverse Reactions, Clinical Studies	**1 INDICATIONS AND USAGE**<br>COTELLIC® is indicated for the treatment of patients with unresectable or metastatic melanoma with a BRAF V600E or V600K mutation, in combination with vemurafenib.<br><br>**2 DOSAGE AND ADMINISTRATION**<br>**2.1 Patient Selection for Treatment of Melanoma**<br>Confirm the presence of BRAF V600E or V600K mutation in tumor specimens prior to initiation of treatment with COTELLIC with vemurafenib. Information on FDA approved tests for the detection of BRAF V600 mutations in melanoma is available at: http://www.fda.gov/CompanionDiagnostics.<br><br>**6 ADVERSE REACTIONS**<br>**6.1 Clinical Trials Experience**<br>(…) The safety of COTELLIC was evaluated in Trial 1, a randomized (1:1), double blind, active-controlled trial in previously untreated patients with BRAF V600 mutation-positive, unresectable or metastatic melanoma [see Clinical Studies (14)]. (…)<br><br>**14 CLINICAL STUDIES**<br>**14.1 Unresectable or Metastatic Melanoma**<br>The safety and efficacy of cobimetinib was established in a multicenter, randomized (1:1), double-blinded, placebo-controlled trial conducted in 495 patients with previously untreated, BRAF V600 mutation-positive, unresectable or metastatic, melanoma. The presence of BRAF V600 mutation was detected using the cobas® 4800 BRAF V600 mutation test. (…)<br>(…) The effect on PFS was also supported by analysis of PFS based on the assessment by blinded independent review. A trend favoring the cobimetinib with vemurafenib arm was observed in exploratory subgroup analyses of PFS, OS, and ORR in both BRAF V600 mutation subtypes (V600E or V600K) in the 81% of patients in this trial where BRAF V600 mutation type was determined.<br>**14.2 Histiocytic Neoplasms**<br>A single-center, single-arm trial (Trial 2) was conducted to evaluate the efficacy, safety, and tolerability of COTELLIC as a single agent in adult patients with histologically confirmed histiocytic neoplasms of any mutational status. Patients with documented BRAF V600E mutations were enrolled if they were unable to access a BRAF inhibitor or discontinued a BRAF inhibitor due to toxicity. Enrolled patients had multi-system disease, recurrent or refractory disease, or single-system disease that is unlikely to benefit from conventional therapies, based on best available evidence.<br>The trial included 26 patients with histiocytic neoplasms including Langerhans Cell Histiocytosis (n=4), Rosai-Dorfman Disease (n=4), Erdheim-Chester Disease (n=13), Xanthogranuloma (n=2) and Mixed Histiocytosis (n=3). Patients with BRAF V600 mutant positive (n=6) and BRAF V600 Wild type (n=20) received COTELLIC. (…)
**022402, 09/18/2018**	**Codeine**	Anesthesiology	CYP2D6	Boxed Warning, Warnings and Precautions, Use in Specific	**BOXED WARNING**<br>WARNING: ADDICTION, ABUSE, AND MISUSE; LIFETHREATENING RESPIRATORY DEPRESSION; ACCIDENTAL INGESTION; NEONATAL OPIOID WITHDRAWAL SYNDROME; DEATH RELATED TO ULTRA-RAPID METABOLISM OF CODEINE TO MORPHINE; INTERACTIONS WITH DRUGS AFFECTING CYTOCHROME P450 ISOENZYMES; and RISKS FROM CONCOMITANT USE WITH BENZODIAZEPINES OR OTHER CNS DEPRESSANTS

NDA/ANDA/BLA Number, Label Version Date	Drug	Therapeutic Area*	Biomarker†	Labeling Sections	Labeling Text‡
				Populations, Patient Counseling Information	Ultra-Rapid Metabolism of Codeine and Other Risk Factors for Life-Threatening Respiratory Depression in Children<br>Life-threatening respiratory depression and death have occurred in children who received codeine. Most of the reported cases occurred following tonsillectomy and/or adenoidectomy, and many of the children had evidence of being an ultra-rapid metabolizer of codeine due to a CYP2D6 polymorphism [see Warnings and Precautions (5.4)]. Codeine Sulfate Tablets are contraindicated in children younger than 12 years of age and in children younger than 18 years of age following tonsillectomy and/or adenoidectomy [see Contraindications (4)]. Avoid the use of Codeine Sulfate Tablets in adolescents 12 to 18 years of age who have other risk factors that may increase their sensitivity to the respiratory depressant effects of codeine. (…)<br><br>**5 WARNINGS AND PRECAUTIONS**<br>**5.4 Ultra-Rapid Metabolism of Codeine and Other Risk Factors for Life-Threatening Respiratory Depression in Children**<br>Life-threatening respiratory depression and death have occurred in children who received codeine. Codeine is subject to variability in metabolism based upon CYP2D6 genotype (described below), which can lead to an increased exposure to the active metabolite morphine. Based upon post-marketing reports, children younger than 12 years old appear to be more susceptible to the respiratory depressant effects of codeine, particularly if there are risk factors for respiratory depression. For example, many reported cases of death occurred in the post-operative period following tonsillectomy and/or adenoidectomy, and many of the children had evidence of being ultra-rapid metabolizers of codeine. Furthermore, children with obstructive sleep apnea who are treated with codeine for post-tonsillectomy and/or adenoidectomy pain may be particularly sensitive to its respiratory depressant effect. (…)<br>*Nursing Mothers*<br>At least one death was reported in a nursing infant who was exposed to high levels of morphine in breast milk because the mother was an ultra-rapid metabolizer of codeine. Breastfeeding is not recommended during treatment with Codeine Sulfate Tablets [see Use in Specific Populations (8.2)].<br>*CYP2D6 Genetic Variability: Ultra-Rapid Metabolizers*<br>Some individuals may be ultra-rapid metabolizers because of a specific CYP2D6 genotype (e.g., gene duplications denoted as *1/*1xN or *1/*2xN). The prevalence of this CYP2D6 phenotype varies widely and has been estimated at 1 to 10% for Whites (European, North American), 3 to 4% for Blacks (African Americans), 1 to 2% for East Asians (Chinese, Japanese, Korean), and may be greater than 10% in certain racial/ethnic groups (i.e., Oceanian, Northern African, Middle Eastern, Ashkenazi Jews, Puerto Rican). These individuals convert codeine into its active metabolite, morphine, more rapidly and completely than other people. This rapid conversion results in higher than expected serum morphine levels. Even at labeled dosage regimens, individuals who are ultra-rapid metabolizers may have life-threatening or fatal respiratory depression or experience signs of overdose (such as extreme sleepiness, confusion, or shallow breathing) [see Overdosage (10)]. Therefore, individuals who are ultra-rapid metabolizers should not use Codeine Sulfate Tablets.<br><br>**8 USE IN SPECIFIC POPULATIONS**<br>**8.2 Lactation**<br>*Risk Summary*<br>Codeine is secreted into human milk. In women with normal codeine metabolism (normal CYP2D6 activity), the amount of codeine secreted into human milk is low and dose-dependent. However, some women are ultra-rapid metabolizers of codeine. These women achieve higher-than-expected serum levels of codeine's active metabolite, morphine, leading to higher-than-expected levels of morphine in breast milk and potentially dangerously high serum morphine levels in their breastfed infants. Therefore, maternal use of codeine can potentially lead to serious adverse reactions, including death, in nursing infants.<br>The developmental and health benefits of breastfeeding should be considered along with the mother's clinical need for Codeine Sulfate Tablets and any potential adverse effects on the breastfed infant from Codeine Sulfate Tablets or from the underlying maternal condition.<br>**8.4 Pediatric Use**<br>The safety and effectiveness of Codeine Sulfate Tablets in pediatric patients have not been established. Life-threatening respiratory depression and death have occurred in children who received codeine [see Warnings and Precautions (5.4)]. In most of the reported cases, these events followed tonsillectomy and/or adenoidectomy, and many of the children had evidence of being ultra-rapid metabolizers of codeine (i.e., multiple copies of the gene for cytochrome P450 isoenzyme 2D6 or high morphine concentrations). Children with sleep apnea may be particularly sensitive to the respiratory depressant effects of codeine. (…)<br><br>**17 PATIENT COUNSELING INFORMATION**<br>*Ultra-Rapid Codeine Metabolism of Codeine and Other Risk Factors for Life-Threatening Respiratory Depression in Children*<br>Advise caregivers that Codeine Sulfate Tablets are contraindicated in all children younger than 12 years of age and in children younger than 18 years of age following tonsillectomy and/or adenoidectomy. Advise caregivers of children 12 to 18 years of age receiving Codeine Sulfate Tablets to monitor for signs of respiratory depression [see Warnings and Precautions (5.4)].
**761128, 11/15/2019**	**Crizanlizumab-tmca**	Hematology	HBB	Adverse Reactions, Clinical Studies	**6 ADVERSE REACTIONS**<br>**6.1 Clinical Trials Experience**<br>Sickle Cell Disease<br>The safety of ADAKVEO was evaluated in the SUSTAIN trial [see Clinical Studies (14.1)]. Eligible patients were diagnosed with sickle cell disease (any genotype including HbSS, HbSC, HbS beta0 -thalassemia, HbSbeta+ thalassemia, and others). (…)<br><br>**14 CLINICAL STUDIES**<br>The efficacy of ADAKVEO was evaluated in patients with sickle cell disease in SUSTAIN [NCT01895361], a 52-week, randomized, multicenter, placebo-controlled, double-blind study. A total of 198 patients with sickle cell disease, any genotype (HbSS, HbSC, HbS/beta0 -thalassemia, HbS/beta+ -thalassemia, and others), and a history of 2-10 VOCs in the previous 12 months were eligible for inclusion. (See Table 2) (…)<br>(…) Patients with sickle cell disease who received ADAKVEO 5 mg/kg had a lower median annual rate of VOC compared to patients who received placebo (1.63 vs. 2.98) which was statistically significant (p = 0.010). Reductions in the frequency of VOCs were observed among patients regardless of sickle cell disease genotype and/or hydroxyurea use. (…)

* Therapeutic areas do not necessarily reflect the CDER review division.
† Representative biomarkers are listed based on standard nomenclature as per the Human Genome Organization (HUGO) symbol and/or simplified descriptors using other common conventions. Listed biomarkers do not necessarily reflect the terminology used in labeling. The term "Nonspecific" is provided when labeling does not explicitly identify the specific biomarker(s) or when the biomarker is represented by a molecular phenotype or gene signature, and in some cases the biomarker was inferred based on the labeling language.
‡ Referenced figures and tables may be found in the labeling available at Drugs@FDA.
Blue text represents the most recent addtions and/or changes since last posted version.

NDA/ANDA/BLA Number, Label Version Date	Drug	Therapeutic Area*	Biomarker†	Labeling Sections	Labeling Text‡
202570, 07/14/2022	Crizotinib (1)	Oncology	ALK	Indications and Usage, Dosage and Administration, Adverse Reactions, Use in Specific Populations, Clinical Pharmacology, Clinical Studies	**1 INDICATIONS AND USAGE**<br>**1.1 ALK- or ROS1-Positive Metastatic Non-Small Cell Lung Cancer**<br>XALKORI is indicated for the treatment of patients with metastatic non-small cell lung cancer (NSCLC) whose tumors are anaplastic lymphoma kinase (ALK) or ROS1-positive as detected by an FDA-approved test [see Dosage and Administration (2.1)].<br>**1.2 Relapsed or Refractory, Systemic ALK-Positive Anaplastic Large Cell Lymphoma**<br>XALKORI is indicated for the treatment of pediatric patients 1 year of age and older and young adults with relapsed or refractory, systemic anaplastic large cell lymphoma (ALCL) that is ALK-positive.<br>Limitations of Use: The safety and efficacy of XALKORI have not been established in older adults with relapsed or refractory, systemic ALK-positive ALCL.<br>**1.3 Unresectable, Recurrent, or Refractory ALK-Positive Inflammatory Myofibroblastic Tumor**<br>XALKORI is indicated for the treatment of adult and pediatric patients 1 year of age and older with unresectable, recurrent, or refractory inflammatory myofibroblastic tumor (IMT) that is ALK-positive.<br><br>**2 DOSAGE AND ADMINISTRATION**<br>**2.1 Patient Selection**<br>Select patients for the treatment of metastatic NSCLC with XALKORI based on the presence of ALK or ROS1 positivity in tumor specimens [see Clinical Studies (14.1, 14.2)].<br>Information on FDA-approved tests for the detection of ALK and ROS1 rearrangements in NSCLC is available at http://www.fda.gov/companiondiagnostics.<br>**2.2 Recommended Dosage for ALK- or ROS1-Positive Metastatic Non-Small Cell Lung Cancer**<br>**See Table 1**<br>**2.4 Dosage Modifications for Adverse Reactions**<br>Recommended Dosage Reductions<br>*Adult Patients with ALK- or ROS1-positive Metastatic NSCLC or Adult Patients with ALK-positive IMT*<br>The recommended dose reductions for adverse reactions are:<br>• First dose reduction: XALKORI 200 mg taken orally twice daily<br>• Second dose reduction: XALKORI 250 mg taken orally once daily<br>• Permanently discontinue if unable to tolerate XALKORI 250 mg taken orally once daily.<br>*Pediatric and Young Adult Patients with ALK-positive ALCL or Pediatric Patients with ALK-positive IMT*<br>The recommended dose reductions for adverse reactions are provided in Table 3.<br>**2.5 Dosage Modifications for Moderate and Severe Hepatic Impairment**<br>Adult Patients with ALK- or ROS1-positive Metastatic NSCLC or with ALK-positive IMT<br>The recommended dose of XALKORI in patients with moderate hepatic impairment [any aspartate aminotransferase (AST) and total bilirubin greater than 1.5 times the upper limit of normal (ULN) and less than or equal to 3 times ULN] is 200 mg orally twice daily.<br>The recommended dose of XALKORI in patients with severe hepatic impairment (any AST and total bilirubin greater than 3 times ULN) is 250 mg orally once daily [see Use in Specific Populations (8.6), Clinical Pharmacology (12.3)].<br>Pediatric and Young Adult Patients with ALK-positive ALCL or Pediatric Patients with ALK-positive IMT<br>The recommended dose of XALKORI in patients with moderate hepatic impairment [any aspartate aminotransferase (AST) and total bilirubin greater than 1.5 times the upper limit of normal (ULN) and less than or equal to 3 times ULN] is the first dose reduction based on BSA as shown in Table 3 [see Dosage and Administration (2.5), Use in Specific Populations (8.6), Clinical Pharmacology (12.3)].<br>The recommended dose of XALKORI in patients with severe hepatic impairment (any AST and total bilirubin greater than 3 times ULN) is the second dose reduction based on BSA as shown in Table 3 [see Dosage and Administration (2.5), Use in Specific Populations (8.6), Clinical Pharmacology (12.3)].<br>**2.6 Dosage Modification for Severe Renal Impairment**<br>Adult Patients with ALK- or ROS1-positive Metastatic NSCLC or with ALK-positive IMT<br>The recommended dosage of XALKORI in patients with severe renal impairment [creatinine clearance (CLcr) less than 30 mL/min, calculated using the modified Cockcroft-Gault equation] not requiring dialysis is 250 mg orally once daily [see Use in Specific Populations (8.7), Clinical Pharmacology (12.3)].<br>Pediatric and Young Adult Patients with ALK-positive ALCL or Pediatric Patients with ALK-positive IMT<br>The recommended dosage of XALKORI in patients with severe renal impairment (CLcr) less than 30 mL/min, calculated using the modified Cockcroft-Gault equation for adult patients and the Schwartz equation for pediatric patients not requiring dialysis is the second dose reduction based on BSA as shown in Table 3 [see Dosage and Administration (2.6), Use in Specific Populations (8.7), Clinical Pharmacology (12.3)].<br>**2.7 Dosage Modification for Concomitant Use of Strong CYP3A Inhibitors**<br>Adult Patients with ALK- or ROS1-positive metastatic NSCLC or with ALK-positive IMT<br>Avoid concomitant use of strong CYP3A inhibitors. If concomitant use of strong CYP3A inhibitors is unavoidable, reduce the dose of XALKORI to 250 mg orally once daily [see Drug Interactions (7.1)]. After discontinuation of a strong CYP3A inhibitor, resume the XALKORI dose used prior to initiating the strong CYP3A inhibitor.<br>Pediatric and Young Adult Patients with ALK-positive ALCL or Pediatric Patients with ALK-positive IMT<br>Avoid concomitant use of strong CYP3A inhibitors. If concomitant use of strong CYP3A inhibitors is unavoidable, reduce the dose of XALKORI to the second dose reduction based on BSA as shown in Table 3 [see Dosage and Administration (2.4), Drug Interactions (7.1)]. After discontinuation of a strong CYP3A inhibitor, resume the XALKORI dose used prior to initiating the strong CYP3A inhibitor..<br><br>**6 ADVERSE REACTIONS**<br>**6.1 Clinical Trials Experience**

* Therapeutic areas do not necessarily reflect the CDER review division.
† Representative biomarkers are listed based on standard nomenclature as per the Human Genome Organization (HUGO) symbol and/or simplified descriptors using other common conventions. Listed biomarkers do not necessarily reflect the terminology used in labeling. The term “Nonspecific” is provided when labeling does not explicitly identify the specific biomarker(s) or when the biomarker is represented by a molecular phenotype or gene signature, and in some cases the biomarker was inferred based on the labeling language.
‡ Referenced figures and tables may be found in the labeling available at Drugs@FDA.
Blue text represents the most recent addtions and/or changes since last posted version.

NDA/ANDA/BLA Number, Label Version Date	Drug	Therapeutic Area*	Biomarker†	Labeling Sections	Labeling Text‡
					(…)The data in the Warnings and Precautions reflect exposure to XALKORI in 1719 patients with NSCLC who received XALKORI 250 mg twice daily enrolled on Studies 1 (including an additional 109 patients who crossed over from the control arm), 2, 3, a single-arm trial (n=1063) of ALK-positive NSCLC, and an additional ALK-positive NSCLC expansion cohort of a dose finding study (n=154). The data also reflect exposure to XALKORI in 121 patients ages 1 to ≤21 years with relapsed or refractory tumors, including 26 patients with systemic ALCL, in a single-arm trial (Study ADVL0912).<br>ALK- or ROS1-Positive Metastatic NSCLC<br>The data described below is based primarily on 343 patients with ALK-positive metastatic NSCLC who received XALKORI 250 mg orally twice daily from 2 open-label, randomized, active-controlled trials (Studies 1 and 2). The safety of XALKORI was also evaluated in 50 patients with ROS1-positive metastatic NSCLC from a single-arm study (Study 3). (…)<br>*Previously Untreated ALK-Positive Metastatic NSCLC - Study 1 (PROFILE 1014)*<br>The data in Table 3 are derived from 340 patients with ALK-positive metastatic NSCLC who had not received previous systemic treatment for advanced disease who received treatment in a randomized, multicenter, open-label, active-controlled trial (Study 1). (…)<br>*Previously Treated ALK-Positive Metastatic NSCLC - Study 2 (PROFILE 1007)*<br>The data in Table 5 are derived from 343 patients with ALK-positive metastatic NSCLC enrolled in a randomized, multicenter, active-controlled, open-label trial (Study 2). (…)<br>*ROS1-Positive Metastatic NSCLC - Study 3 (PROFILE 1001)*<br>The safety profile of XALKORI from Study 3, which was evaluated in 50 patients with ROS1-positive metastatic NSCLC, was generally consistent with the safety profile of XALKORI evaluated in patients with ALK-positive metastatic NSCLC (n=1669). Vision disorders occurred in 92% of patients in Study 3; 90% were Grade 1 and 2% were Grade 2. The median duration of exposure to XALKORI was 34.4 months.<br>ROS1-Positive Metastatic NSCLC - Study 3 (PROFILE 1001)<br>The safety profile of XALKORI from Study 3, which was evaluated in 50 patients with ROS1-positive metastatic NSCLC, was generally consistent with the safety profile of XALKORI evaluated in patients with ALK-positive metastatic NSCLC (n=1669). Vision disorders occurred in 92% of patients in Study 3; 90% were Grade 1 and 2% were Grade 2. The median duration of exposure to XALKORI was 34.4 months.<br>*Renal toxicity*<br>The estimated glomerular filtration rate (eGFR) decreased from a baseline median of 96.42 mL/min/1.73 m2 (n=1681) to a median of 80.23 mL/min/1.73 m2 at 2 weeks (n=1499) in patients with ALK-positive advanced NSCLC who received XALKORI in clinical trials. (…)<br>Relapsed or Refractory, Systemic ALK-Positive ALCL - Study ADVL0912<br>The safety of XALKORI was evaluated in Study ADVL0912 [see Clinical Studies 14.2], which included 26 patients with relapsed or refractory, systemic ALCL after at least one systemic therapy. Eligible patients were 1 to ≤21 years of age and were required to have an absolute neutrophil count ≥1000/mm3 (750/mm3 if bone marrow was involved), platelet count ≥75,000/mm3 (25,000/mm3 if bone marrow was involved), creatinine clearance ≥70ml/min/1.73m2 , and QTc ≤480 msec. The study excluded patients with ALT >2.5 times upper limit of normal (ULN), bilirubin ≤1.5 times ULN, and central nervous system tumors.<br>Unresectable, Recurrent, or Refractory ALK-Positive IMT<br>*Study ADVL0912*<br>The safety of XALKORI was evaluated in Study ADVL0912 [see Clinical Studies (14.3)] that included 14 pediatric patients with unresectable, recurrent, or refractory IMT. (…)<br>*Study A8081013*<br>The safety of XALKORI for adult patients with ALK-positive IMT was evaluated in Study A8081013 [s ee Clinical Studies (14.3)] that included 7 patients with IMT with a median age of 38 years (range 23 to 73). The safety profile of this patient group was generally consistent with the safety profile of XALKORI evaluated in patients with ALK-positive or ROS1-positive NSCLC. The most frequent adverse reactions (≥20%) were vision disorders, nausea, and edema.<br><br>**8 USE IN SPECIFIC POPULATIONS**<br>**8.4 Pediatric Use**<br>The safety and effectiveness of XALKORI have been established in pediatric patients 12 months of age and older with relapsed or refractory, systemic ALK-positive ALCL [see Adverse Reactions (6.1), Clinical Studies (14.2)]. The safety and effectiveness have not been established in pediatric patients younger than 12 months of age with ALCL or in any pediatric patients with NSCLC.<br>In a study that evaluated XALKORI in combination with chemotherapy in pediatric patients with newly diagnosed ALCL (Study ANHL12P1; NCT01979536), 13 of 66 (20%) patients had a Grade 2 or higher thromboembolic event, including pulmonary embolism in 6%. The safety and effectiveness of XALKORI in combination with chemotherapy have not been established in patients with newly diagnosed ALCL.<br>**8.5 Geriatric Use**<br>Of the total number of patients with ALK-positive metastatic NSCLC in clinical studies of XALKORI (n=1669), 16% were 65 years or older and 3.8% were 75 years or older. No overall differences in safety or effectiveness were observed between these patients and younger patients. (…)<br><br>**12 CLINICAL PHARMACOLOGY**<br>**12.2 Pharmacodynamics**<br>*Cardiac electrophysiology*<br>In an ECG substudy conducted in 52 patients with ALK-positive NSCLC who received crizotinib 250 mg twice daily, the maximum mean QTcF (corrected QT by the Fridericia method) change from baseline was 12.3 ms (2-sided 90% upper CI: 19.5 ms). An exposure-QT analysis suggested a crizotinib plasma concentration dependent increase in QTcF [see Warnings and Precautions (5.3)].<br><br>**14 CLINICAL STUDIES**<br>**14.1 ALK- or ROS1-Positive Metastatic Non-Small Cell Lung Cancer**

NDA/ANDA/BLA Number, Label Version Date	Drug	Therapeutic Area*	Biomarker†	Labeling Sections	Labeling Text‡
					<u>Previously Untreated ALK-Positive Metastatic NSCLC - Study 1 (PROFILE 1014; NCT01154140)</u><br>The efficacy of XALKORI for the treatment of patients with ALK-positive metastatic NSCLC, who had not received previous systemic treatment for advanced disease, was demonstrated in a randomized, multicenter, open-label, active-controlled study (Study 1). Patients were required to have ALK-positive NSCLC as identified by the FDA-approved assay, Vysis ALK Break-Apart fluorescence in situ hybridization (FISH) Probe Kit, prior to randomization. The major efficacy outcome measure was progression-free survival (PFS) according to Response Evaluation Criteria in Solid Tumors (RECIST) version 1.1 as assessed by independent radiology review (IRR) committee. Additional efficacy outcome measures included objective response rate (ORR) as assessed by IRR, DOR, and overall survival (OS). Patient-reported lung cancer symptoms were assessed at baseline and periodically during treatment. (See Table 12) (…)<br><u>Previously Treated ALK-Positive Metastatic NSCLC - Study 2 (PROFILE 1007; NCT00932893)</u><br>The efficacy of XALKORI as monotherapy for the treatment of 347 patients with ALK-positive metastatic NSCLC, previously treated with 1 platinum-based chemotherapy regimen, were demonstrated in a randomized, multicenter, open-label, active-controlled study (Study 2). The major efficacy outcome was PFS according to RECIST version 1.1 as assessed by IRR. Additional efficacy outcomes included ORR as assessed by IRR, DOR, and OS.<br>Patients were randomized to receive XALKORI 250 mg orally twice daily (n=173) or chemotherapy (n=174). Chemotherapy consisted of pemetrexed 500 mg/m2 (if pemetrexed-naïve; n=99) or docetaxel 75 mg/m2 (n=72) intravenously (IV) every 21 days. Patients in both treatment arms continued treatment until documented disease progression, intolerance to therapy, or the investigator determined that the patient was no longer experiencing clinical benefit. Randomization was stratified by ECOG performance status (0-1, 2), brain metastases (present, absent), and prior EGFR tyrosine kinase inhibitor treatment (yes, no). Patients were required to have ALK-positive NSCLC as identified by the FDA-approved assay, Vysis ALK Break-Apart FISH Probe Kit, prior to randomization. (See Table 13) (…)<br>**14.2 Relapsed or Refractory, Systemic ALK-Positive Anaplastic Large Cell Lymphoma**<br>The efficacy of XALKORI was evaluated in Study ADVL0912 (NCT00939770), a multicenter, single arm, open-label study in patients 1 to ≤21 years of age that included 26 patients with relapsed or refractory, systemic ALK-positive ALCL after at least one systemic treatment. ALK positive status (confirmation of an ALK fusion) was determined locally by immunohistochemistry or fluorescence in situ hybridization. The study excluded patients with primary cutaneous ALCL or central nervous system involvement by lymphoma. (See Table 18) (…)<br>**14.3 Unresectable, Recurrent, or Refractory ALK-Positive Inflammatory Myofibroblastic Tumor**<br><u>Pediatric Patients with ALK-positive IMT</u><br>*Study ADVL0912*<br>The efficacy of XALKORI was evaluated in Study ADVL0912 (NCT00939770), a multicenter, single-arm, open-label study in patients 1 to ≤21 years of age that included 14 pediatric patients with unresectable, recurrent, or refractory ALK-positive IMT. Patients were required to have an ALK fusion determined locally by immunohistochemistry or fluorescence in situ hybridization. Patients (n=12) received XALKORI 280 mg/m2 twice daily until disease progression or unacceptable toxicity. Two patients received a lower dose. (see Table 19)<br><u>Adult Patients with ALK-positive IMT</u><br>Study A8081013 The efficacy of XALKORI was evaluated in Study A8081013 (NCT01121588), a multicenter, single-arm, open-label study that included 7 adult patients with unresectable, recurrent, or refractory ALK-positive IMT. ALK fusion was determined locally by immunohistochemistry or fluorescence in situ hybridization. Patients received XALKORI 250 mg twice daily.<br>The demographic characteristics were median age 38 years (range: 23 to 73); 57% male; 57% White, 43% Asian; and 86% ECOG performance status of 0 or 1. Two (29%) patients had at least one prior systemic treatment.<br>The major efficacy outcome was objective response rate according to RECIST version 1.1 per investigator assessment. For the 7 patients with ALK-positive IMT, 5 experienced a response including 1 complete response. The DOR was ≥6 months for all 5 patients and ≥12 months for 2 patients.
202570, 07/14/2022	Crizotinib (2)	Oncology	ROS1	Indications and Usage, Dosage and Administration, Adverse Reactions, Use in Specific Populations, Clinical Studies	**1 INDICATIONS AND USAGE**<br>**1.1 ALK- or ROS1-Positive Metastatic Non-Small Cell Lung Cancer**<br>XALKORI is indicated for the treatment of patients with metastatic non-small cell lung cancer (NSCLC) whose tumors are anaplastic lymphoma kinase (ALK) or ROS1-positive as detected by an FDA-approved test [see Dosage and Administration (2.1)].<br><br>**2 DOSAGE AND ADMINISTRATION**<br>**2.1 Patient Selection**<br>Select patients for the treatment of metastatic NSCLC with XALKORI based on the presence of ALK or ROS1 positivity in tumor specimens [see Clinical Studies (14.1, 14.2)].<br>Information on FDA-approved tests for the detection of ALK and ROS1 rearrangements in NSCLC is available at <u>http://www.fda.gov/companiondiagnostics</u>.<br>**2.2 Recommended Dosage for ALK- or ROS1-Positive Metastatic Non-Small Cell Lung Cancer**<br>See Table 1.<br>**2.4 Dosage Modifications for Adverse Reactions**<br><u>Recommended Dosage Reductions</u><br>*Adult Patients with ALK- or ROS1-positive Metastatic NSCLC or Adult Patients with ALK-positive IMT*<br>The recommended dose reductions for adverse reactions are:<br>• First dose reduction: XALKORI 200 mg taken orally twice daily<br>· Second dose reduction: XALKORI 250 mg taken orally once daily<br>• Permanently discontinue if unable to tolerate XALKORI 250 mg taken orally once daily.<br>*Pediatric and Young Adult Patients with ALK-positive ALCL or Pediatric Patients with ALK-positive IMT*<br>The recommended dose reductions for adverse reactions are provided in Table 3.<br>**2.5 Dosage Modifications for Moderate and Severe Hepatic Impairment**<br><u>Adult Patients with ALK- or ROS1-positive Metastatic NSCLC or with ALK-positive IMT</u>

* Therapeutic areas do not necessarily reflect the CDER review division.
† Representative biomarkers are listed based on standard nomenclature as per the Human Genome Organization (HUGO) symbol and/or simplified descriptors using other common conventions. Listed biomarkers do not necessarily reflect the terminology used in labeling. The term “Nonspecific” is provided when labeling does not explicitly identify the specific biomarker(s) or when the biomarker is represented by a molecular phenotype or gene signature, and in some cases the biomarker was inferred based on the labeling language.
‡ Referenced figures and tables may be found in the labeling available at Drugs@FDA.
Blue text represents the most recent addtions and/or changes since last posted version.

NDA/ANDA/BLA Number, Label Version Date	Drug	Therapeutic Area*	Biomarker†	Labeling Sections	Labeling Text‡
					The recommended dose of XALKORI in patients with moderate hepatic impairment [any aspartate aminotransferase (AST) and total bilirubin greater than 1.5 times the upper limit of normal (ULN) and less than or equal to 3 times ULN] is 200 mg orally twice daily.<br>The recommended dose of XALKORI in patients with severe hepatic impairment (any AST and total bilirubin greater than 3 times ULN) is 250 mg orally once daily [see Use in Specific Populations (8.6), Clinical Pharmacology (12.3)].<br><u>Pediatric and Young Adult Patients with ALK-positive ALCL or Pediatric Patients with ALK-positive IMT</u><br>The recommended dose of XALKORI in patients with moderate hepatic impairment [any aspartate aminotransferase (AST) and total bilirubin greater than 1.5 times the upper limit of normal (ULN) and less than or equal to 3 times ULN] is the first dose reduction based on BSA as shown in Table 3 [see Dosage and Administration (2.5), Use in Specific Populations (8.6), Clinical Pharmacology (12.3)].<br>The recommended dose of XALKORI in patients with severe hepatic impairment (any AST and total bilirubin greater than 3 times ULN) is the second dose reduction based on BSA as shown in Table 3 [see Dosage and Administration (2.5), Use in Specific Populations (8.6), Clinical Pharmacology (12.3)].<br>**2.6 Dosage Modification for Severe Renal Impairment**<br><u>Adult Patients with ALK- or ROS1-positive Metastatic NSCLC or with ALK-positive IMT</u><br>The recommended dosage of XALKORI in patients with severe renal impairment [creatinine clearance (CLcr) less than 30 mL/min, calculated using the modified Cockcroft-Gault equation] not requiring dialysis is 250 mg orally once daily [see Use in Specific Populations (8.7), Clinical Pharmacology (12.3)].<br><u>Pediatric and Young Adult Patients with ALK-positive ALCL or Pediatric Patients with ALK-positive IMT</u><br>The recommended dosage of XALKORI in patients with severe renal impairment (CLcr) less than 30 mL/min, calculated using the modified Cockcroft-Gault equation for adult patients and the Schwartz equation for pediatric patients not requiring dialysis is the second dose reduction based on BSA as shown in Table 3 [see Dosage and Administration (2.6), Use in Specific Populations (8.7), Clinical Pharmacology (12.3)].<br>**2.7 Dosage Modification for Concomitant Use of Strong CYP3A Inhibitors**<br><u>Adult Patients with ALK- or ROS1-positive metastatic NSCLC or with ALK-positive IMT</u><br>Avoid concomitant use of strong CYP3A inhibitors. If concomitant use of strong CYP3A inhibitors is unavoidable, reduce the dose of XALKORI to 250 mg orally once daily [see Drug Interactions (7.1)]. After discontinuation of a strong CYP3A inhibitor, resume the XALKORI dose used prior to initiating the strong CYP3A inhibitor.<br><u>Pediatric and Young Adult Patients with ALK-positive ALCL or Pediatric Patients with ALK-positive IMT</u><br>Avoid concomitant use of strong CYP3A inhibitors. If concomitant use of strong CYP3A inhibitors is unavoidable, reduce the dose of XALKORI to the second dose reduction based on BSA as shown in Table 3 [see Dosage and Administration (2.4), Drug Interactions (7.1)]. After discontinuation of a strong CYP3A inhibitor, resume the XALKORI dose used prior to initiating the strong CYP3A inhibitor.<br><br>**6 ADVERSE REACTIONS**<br>**6.1 Clinical Trials Experience**<br><u>ALK- or ROS1-Positive Metastatic NSCLC</u><br>The data described below is based primarily on 343 patients with ALK-positive metastatic NSCLC who received XALKORI 250 mg orally twice daily from 2 open-label, randomized, active-controlled trials (Studies 1 and 2). The safety of XALKORI was also evaluated in 50 patients with ROS1-positive metastatic NSCLC from a single-arm study (Study 3). (…)<br>*ROS1-Positive Metastatic NSCLC - Study 3 (PROFILE 1001)*<br>The safety profile of XALKORI from Study 3, which was evaluated in 50 patients with ROS1-positive metastatic NSCLC, was generally consistent with the safety profile of XALKORI evaluated in patients with ALK-positive metastatic NSCLC (n=1669). Vision disorders occurred in 92% of patients in Study 3; 90% were Grade 1 and 2% were Grade 2. The median duration of exposure to XALKORI was 34.4 months.<br>*Study A8081013*<br>The safety of XALKORI for adult patients with ALK-positive IMT was evaluated in Study A8081013 [s ee Clinical Studies (14.3)] that included 7 patients with IMT with a median age of 38 years (range 23 to 73). The safety profile of this patient group was generally consistent with the safety profile of XALKORI evaluated in patients with ALK-positive or ROS1-positive NSCLC. The most frequent adverse reactions (≥20%) were vision disorders, nausea, and edema.<br><br>**8 USE IN SPECIFIC POPULATIONS**<br>**8.5 Geriatric Use**<br>Of the total number of patients with ALK-positive metastatic NSCLC in clinical studies of XALKORI (n=1669), 16% were 65 years or older and 3.8% were 75 years or older. No overall differences in safety or effectiveness were observed between these patients and younger patients.<br>Clinical studies of XALKORI in patients with ROS1-positive metastatic NSCLC did not include sufficient numbers of patients age 65 years and older to determine whether they respond differently from younger patients.<br><br>**14 CLINICAL STUDIES**<br>**14.1 ALK- or ROS1-Positive Metastatic Non-Small Cell Lung Cancer**<br><u>Previously Untreated ALK-Positive Metastatic NSCLC - Study 1 (PROFILE 1014; NCT01154140)</u><br>The efficacy of XALKORI for the treatment of patients with ALK-positive metastatic NSCLC, who had not received previous systemic treatment for advanced disease, was demonstrated in a randomized, multicenter, open-label, active-controlled study (Study 1). Patients were required to have ALK-positive NSCLC as identified by the FDA-approved assay, Vysis ALK Break-Apart fluorescence in situ hybridization (FISH) Probe Kit, prior to randomization. The major efficacy outcome measure was progression-free survival (PFS) according to Response Evaluation Criteria in Solid Tumors (RECIST) version 1.1 as assessed by independent radiology review (IRR) committee. Additional efficacy outcome measures included objective response rate (ORR) as assessed by IRR, DOR, and overall survival (OS). Patient-reported lung cancer symptoms were assessed at baseline and periodically during treatment. (…)<br><u>ROS1-Positive Metastatic NSCLC - Study 3 (PROFILE 1001; NCT00585195)</u>

NDA/ANDA/BLA Number, Label Version Date	Drug	Therapeutic Area*	Biomarker†	Labeling Sections	Labeling Text‡
					The efficacy and safety of XALKORI was investigated in a multicenter, single-arm study (Study 3), in which patients with ROS1-positive metastatic NSCLC received XALKORI 250 mg orally twice daily. Patients were required to have histologically-confirmed advanced NSCLC with a ROS1 rearrangement, age 18 years or older, ECOG performance status of 0, 1, or 2, and measurable disease. The efficacy outcome measures were ORR and DOR according to RECIST version 1.0 as assessed by IRR and investigator, with imaging performed every 8 weeks for the first 60 weeks.<br>Baseline demographic and disease characteristics were female (56%), median age of 53 years, baseline ECOG performance status of 0 or 1 (98%), White (54%), Asian (42%), past smokers (22%), never smokers (78%), metastatic disease (92%), adenocarcinoma (96%), no prior systemic therapy for metastatic disease (14%), and prior platinum-based chemotherapy for metastatic disease (80%). The ROS1 status of NSCLC tissue samples was determined by laboratory-developed break-apart FISH (96%) or RT-PCR (4%) clinical trial assays. For assessment by FISH, ROS1 positivity required that ≥15% of a minimum of 50 evaluated nuclei contained a ROS1 gene rearrangement.<br>Efficacy results are summarized in Table 17.
202806, 05/26/2023	Dabrafenib (1)	Oncology	BRAF	Indications and Usage, Dosage and Administration, Warnings and Precautions, Adverse Reactions, Use in Specific Populations, Clinical Pharmacology, Clinical Studies	**1 INDICATIONS AND USAGE**<br>**1.1 BRAF V600E Mutation-Positive Unresectable or Metastatic Melanoma**<br>TAFINLAR® is indicated as a single agent for the treatment of patients with unresectable or metastatic melanoma with BRAF V600E mutation as detected by an FDA-approved test [see Dosage and Administration (2.1), (2.2)].<br>**1.2 BRAF V600E or V600K Mutation-Positive Unresectable or Metastatic Melanoma**<br>TAFINLAR is indicated, in combination with trametinib, for the treatment of patients with unresectable or metastatic melanoma with BRAF V600E or V600K mutations, as detected by an FDA-approved test [see Dosage and Administration (2.1), (2.2)].<br>**1.3 Adjuvant Treatment of BRAF V600E or V600K Mutation-Positive Melanoma**<br>TAFINLAR is indicated, in combination with trametinib, for the adjuvant treatment of patients with melanoma with BRAF V600E or V600K mutations, as detected by an FDA-approved test, and involvement of lymph node(s), following complete resection [see Dosage and Administration (2.1), (2.3)].<br>**1.4 BRAF V600E Mutation-Positive Metastatic NSCLC**<br>TAFINLAR is indicated, in combination with trametinib, for the treatment of patients with metastatic non-small cell lung cancer (NSCLC) with BRAF V600E mutation as detected by an FDA-approved test [see Dosage and Administration (2.1), (2.4)].<br>**1.5 BRAF V600E Mutation-Positive Locally Advanced or Metastatic Anaplastic Thyroid Cancer**<br>TAFINLAR is indicated, in combination with trametinib, for the treatment of patients with locally advanced or metastatic anaplastic thyroid cancer (ATC) with BRAF V600E mutation and with no satisfactory locoregional treatment options [see Dosage and Administration (2.1), (2.5)].<br>**1.6 BRAF V600E Mutation-Positive Unresectable or Metastatic Solid Tumors**<br>TAFINLAR is indicated, in combination with trametinib, for the treatment of adult and pediatric patients 6 years of age and older with unresectable or metastatic solid tumors with BRAF V600E mutation who have progressed following prior treatment and have no satisfactory alternative treatment options [see Dosage and Administration (2.1)]. This indication is approved under accelerated approval based on overall response rate and duration of response [see Clinical Studies (14.6)]. Continued approval for this indication may be contingent upon verification and description of clinical benefit in a confirmatory trial(s).<br>**1.7 BRAF V600E Mutation-Positive Low-Grade Glioma**<br>TAFINLAR is indicated, in combination with trametinib, for the treatment of pediatric patients 1 year of age and older with low-grade glioma (LGG) with a BRAF V600E mutation who require systemic therapy [see Dosage and Administration (2.1)].<br>**1.8 Limitations of Use**<br>• TAFINLAR is not indicated for treatment of patients with colorectal cancer because of known intrinsic resistance to BRAF inhibition [see Indications and Usage (1.6), Clinical Pharmacology (12.1)].<br>• TAFINLAR is not indicated for treatment of patients with wild-type BRAF solid tumors [see Warnings and Precautions (5.2)].<br><br>**2 DOSAGE AND ADMINISTRATION**<br>**2.1 Patient Selection**<br>Melanoma<br>• Confirm the presence of BRAF V600E mutation in tumor specimens prior to initiation of treatment with TAFINLAR as a single agent [see Warnings and Precautions (5.2) and Clinical Studies (14.1)].<br>• Confirm the presence of BRAF V600E or V600K mutation in tumor specimens prior to initiation of treatment with TAFINLAR and trametinib [see Warnings and Precautions (5.2), Clinical Studies (14.2), (14.3)].<br>• Information on FDA-approved tests for the detection of BRAF V600 mutations in melanoma is available at: http://www.fda.gov/CompanionDiagnostics.<br>NSCLC<br>• Confirm the presence of BRAF V600E mutation in tumor specimens prior to initiation of treatment with TAFINLAR and trametinib [see Clinical Studies (14.4)].<br>• Information on FDA-approved tests for the detection of BRAF V600E mutations in NSCLC is available at: http://www.fda.gov/CompanionDiagnostics.<br>ATC<br>• Confirm the presence of BRAF V600E mutation in tumor specimens prior to initiation of treatment with TAFINLAR and trametinib [see Clinical Studies (14.5)].<br>An FDA-approved test for the detection of BRAF V600E mutation in ATC is not currently available. (…)<br>Solid Tumors<br>• Confirm the presence of BRAF V600E mutation in tumor specimens prior to initiation of treatment with TAFINLAR and trametinib [see Clinical Studies (14.6)].<br>An FDA-approved test for the detection of BRAF V600E mutation in solid tumors other than melanoma and NSCLC is not currently available.<br>Low-Grade Glioma

* Therapeutic areas do not necessarily reflect the CDER review division.
† Representative biomarkers are listed based on standard nomenclature as per the Human Genome Organization (HUGO) symbol and/or simplified descriptors using other common conventions. Listed biomarkers do not necessarily reflect the terminology used in labeling. The term "Nonspecific" is provided when labeling does not explicitly identify the specific biomarker(s) or when the biomarker is represented by a molecular phenotype or gene signature, and in some cases the biomarker was inferred based on the labeling language.
‡ Referenced figures and tables may be found in the labeling available at Drugs@FDA.
Blue text represents the most recent addtions and/or changes since last posted version.

NDA/ANDA/BLA Number, Label Version Date	Drug	Therapeutic Area*	Biomarker†	Labeling Sections	Labeling Text‡
					• Confirm the presence of BRAF V600E mutation in tumor specimens prior to initiation of treatment with TAFINLAR and trametinib [see Clinical Studies (14.7)]. An FDA-approved test for the detection of BRAF V600E mutation in LGG is not currently available.<br><br>**5 WARNINGS AND PRECAUTIONS**<br>**5.2 Tumor Promotion in BRAF Wild-Type Melanoma**<br>In vitro experiments have demonstrated paradoxical activation of MAP-kinase signaling and increased cell proliferation in BRAF wild-type cells which are exposed to BRAF inhibitors. Confirm evidence of BRAF V600E or V600K mutation status prior to initiation of TAFINLAR as a single agent or in combination with trametinib [see Indications and Usage (1.6), Dosage and Administration (2.1)].<br><br>**6 ADVERSE REACTIONS**<br>**6.1 Clinical Trials Experience**<br>Because clinical trials are conducted under widely varying conditions, adverse reaction rates observed in the clinical trials of a drug cannot be directly compared to rates in the clinical trials of another drug and may not reflect the rates observed in practice.<br>The data described in the Warnings and Precautions section reflect exposure to TAFINLAR administered as a single agent in 586 patients with various solid tumors and exposure to TAFINLAR administered with trametinib in 559 patients with melanoma and 93 patients with NSCLC. The safety of TAFINLAR as a single agent was evaluated in 586 patients with BRAF V600 mutation-positive unresectable or metastatic melanoma, previously treated or untreated, who received TAFINLAR 150 mg orally twice daily until disease progression or unacceptable toxicity, including 181 patients treated for at least 6 months and 86 additional patients treated for more than 12 months. TAFINLAR was studied in open-label, single-arm trials and in an open-label, randomized, active-controlled trial. The median daily dose of TAFINLAR was 300 mg (range: 118 to 300 mg).<br>*Metastatic or Unresectable BRAF V600 Mutation Positive Melanoma*<br>*TAFINLAR as a Single Agent*<br>Table 3 and Table 4 present adverse drug reactions and laboratory abnormalities identified from analyses of the BREAK-3 study [see Clinical Studies (14.1)].This study, a multicenter, international, open-label, randomized (3:1), controlled trial allocated 250 patients with unresectable or metastatic BRAF V600E mutation-positive melanoma to receive TAFINLAR 150 mg orally twice daily (n = 187) or dacarbazine 1,000 mg/m2 intravenously every 3 weeks (n = 63). (…)<br>*TAFINLAR Administered with Trametinib*<br>The safety of TAFINLAR when administered with trametinib was evaluated in 559 patients with previously untreated, unresectable or metastatic, BRAF V600E or V600K mutation-positive melanoma who received TAFINLAR in two trials, Trial 2 (n = 209) a multicenter, double-blind, randomized (1:1), active controlled trial and Trial 3 (n = 350) a multicenter, open-label, randomized (1:1), active controlled trial. (…)<br>*Adjuvant Treatment of BRAF V600E or V600K Mutation-Positive Melanoma*<br>The safety of TAFINLAR when administered with trametinib was evaluated in 435 patients with Stage III melanoma with BRAF V600E or V600K mutations following complete resection who received at least one dose of study therapy in the COMBI-AD study [see Clinical Studies (14.3)]. (…)<br>*Metastatic, BRAF V600E-Mutation Positive, Non-Small Cell Lung Cancer (NSCLC)*<br>The safety of TAFINLAR when administered with trametinib was evaluated in 93 patients with previously untreated (n = 36) and previously treated (n = 57) metastatic BRAF V600E mutation-positive NSCLC in a multicenter, multi-cohort, non-randomized, open-label trial (Study BRF113928). (…)<br>*Locally Advanced or Metastatic, BRAF V600E-Mutation Positive, Anaplastic Thyroid Cancer (ATC)*<br>The safety of TAFINLAR when administered with trametinib was evaluated in a nine-cohort, multicenter, nonrandomized, open-label study in patients with rare cancers with the BRAF V600E mutation, including locally advanced or metastatic ATC (Study BRF117019). (…)<br>*Advanced BRAF V600E-Mutation Positive Tumors*<br>*Study BRF117019*<br>The safety of TAFINLAR when administered with trametinib was evaluated in a multi-cohort, multi-center, non-randomized, open-label study in adult patients with cancers with the BRAF V600E mutation (Study BRF117019). A total of 206 patients were enrolled in the trial, 36 of whom were enrolled in the ATC cohort, 105 were enrolled in specific solid tumor cohorts, and 65 in other malignancies [see Clinical Studies (14.5, 14.6)]. Patients received TAFINLAR 150 mg orally twice daily and trametinib 2 mg orally once daily until disease progression or unacceptable toxicity. (…)<br><br>**8 USE IN SPECIFIC POPULATIONS**<br>**8.4 Pediatric Use**<br>BRAF V600E Mutation-Positive Unresectable or Metastatic Solid Tumors and LGG<br>The safety and effectiveness of TAFINLAR in combination with trametinib have been established in pediatric patients 6 years of age and older with unresectable or metastatic solid tumors with BRAF V600E mutation who have progressed following prior treatment and have no satisfactory alternative treatment options; and patients 1 year of age and older with LGG with BRAF V600E mutation who require systemic therapy. Use of TAFINLAR in combination with trametinib for these indications is supported by evidence from studies X2101 and G2201 that enrolled 171 patients (1 to < 18 years) with BRAF V600 mutation-positive advanced solid tumors, of which 4 (2.3%) patients were 1 to < 2 years of age, 39 (23%) patients were 2 to < 6 years of age, 54 (32%) patients were 6 to < 12 years of age, and 74 (43%) patients were 12 to < 18 years of age [see Adverse Reactions (6.1), Clinical Pharmacology (12.3), Clinical Studies (14.6, 14.7)].<br>The safety and effectiveness of TAFINLAR in combination with trametinib have not been established in pediatric patients younger than 1 year old with LGG with BRAF V600E mutation, and in patients < 6 years old with unresectable or metastatic solid tumors with BRAF V600E mutation.<br><br>**12 CLINICAL PHARMACOLOGY**<br>**12.2 Pharmacodynamics**<br>*Cardiac Electrophysiology*

NDA/ANDA/BLA Number, Label Version Date	Drug	Therapeutic Area*	Biomarker†	Labeling Sections	Labeling Text‡
					The potential effect of TAFINLAR on QT prolongation was assessed in a dedicated multiple-dose study in 32 patients with BRAF V600 mutation-positive tumors. No large changes in the mean QT interval (i.e., >20 ms) were detected with dabrafenib 300 mg administered twice daily (two times the recommended dosage). (…)<br><br>**14 CLINICAL STUDIES**<br>**14.1 BRAF V600E Mutation-Positive Unresectable or Metastatic Melanoma – TAFINLAR Administered as a Single Agent**<br>BREAK-3<br>In the BREAK-3 study (NCT01227889), the safety and efficacy of TAFINLAR as a single agent were demonstrated in an international, multicenter, randomized (3:1), open-label, active-controlled trial conducted in 250 patients with previously untreated BRAF V600E mutation-positive, unresectable or metastatic melanoma. Patients with any prior use of BRAF inhibitors or MEK inhibitors were excluded. (…)<br>(…) All patients had tumor tissue with mutations in BRAF V600E as determined by a clinical trial assay at a centralized testing site. Tumor samples from 243 patients (97%) were tested retrospectively, using an FDAapproved companion diagnostic test, THxID™-BRAF assay.(…)<br>(…) In supportive analyses based on IRRC assessment and in an exploratory subgroup analysis of patients with retrospectively confirmed V600E mutation-positive melanoma with the THxID™-BRAF assay, the PFS results were consistent with those of the primary efficacy analysis.<br>BREAK-MB<br>Study The activity of TAFINLAR for the treatment of BRAF V600E mutation-positive melanoma, metastatic to the brain was evaluated in a single-arm, open-label, two-cohort multicenter trial (the BREAK-MB study; NCT01266967). (…)<br>**14.2 BRAF V600E or V600K Unresectable or Metastatic Melanoma – TAFINLAR Administered with Trametinib**<br>COMBI-d Study and COMBI-v Study<br>The safety and efficacy of TAFINLAR administered with trametinib were evaluated in two international, randomized, active-controlled trials: one double-blind trial (the COMBI-d study; NCT01584648) and one openlabel trial (the COMBI-v study; NCT01597908).<br>The COMBI-d study compared TAFINLAR and trametinib to TAFINLAR and placebo as first-line therapy for patients with unresectable (Stage IIIC) or metastatic (Stage IV) BRAF V600E or V600K mutation-positive cutaneous melanoma. Patients were randomized (1:1) to receive TAFINLAR 150 mg twice daily and trametinib 2 mg once daily or TAFINLAR 150 mg twice daily plus matching placebo. Randomization was stratified by lactate dehydrogenase (LDH) level (> the upper limit of normal (ULN) vs. ≤ ULN) and BRAF mutation subtype (V600E vs. V600K). The major efficacy outcome was investigator-assessed progression-free survival (PFS) per RECIST v1.1 with additional efficacy outcome measures of overall survival (OS) and confirmed overall response rate (ORR).<br>The COMBI-v study compared TAFINLAR and trametinib to vemurafenib as first-line treatment therapy for patients with unresectable (Stage IIIC) or metastatic (Stage IV) BRAF V600E or V600K mutation-positive cutaneous melanoma. Patients were randomized (1:1) to receive TAFINLAR 150 mg twice daily and trametinib 2 mg once daily or vemurafenib 960 mg twice daily. Randomization was stratified by lactate dehydrogenase (LDH) level (> the upper limit of normal (ULN) vs. ≤ ULN) and BRAF mutation subtype (V600E vs. V600K). The major efficacy outcome measure was overall survival. Additional efficacy outcome measures were PFS and ORR as assessed by investigator per RECIST v1.1. (…)<br>(…) All patients had tumor containing BRAF V600E or V600K mutations as determined by centralized testing, 85% with BRAF V600E mutations and 15% with BRAF V600K mutations. (…)<br>In the COMBI-v study, 704 patients were randomized to TAFINLAR plus trametinib (n = 352) or single-agent vemurafenib (n = 352). The median age was 55 years (range: 18 to 91 years), 96% were White, and 55% were male, 6% percent of patients had Stage IIIC, 61% had M1c disease, 67% had a normal LDH, 70% had ECOG performance status of 0, 89% had BRAF V600E mutation-positive melanoma, and one patient had a history of brain metastases. (See Table 12 and Figures 2, 3)<br>COMBI-MB Study<br>(…) The COMBI-MB study enrolled 121 patients with a BRAF V600E (85%) or V600K (15%) mutation. The median age was 54 years (range: 23 to 84 years), 58% were male, 100% were white, 8% were from the United States, 65% had a normal LDH value at baseline, and 97% had an ECOG performance status of 0 or 1. Intracranial metastases were asymptomatic in 87% and symptomatic in 13% of patients, 22% received prior local therapy for brain metastases, and 87% also had extracranial metastases. (…)<br>**14.3 Adjuvant Treatment of BRAF V600E or V600K Mutation-Positive Melanoma**<br>COMBI-AD (NCT 01682083) was an international, multi-center, randomized, double-blind, placebo-controlled trial that enrolled patients with Stage III melanoma with BRAF V600E or V600K mutations as detected by the THxID™-BRAF assay and pathologic involvement of regional lymph node(s). Patients were randomized (1:1) to receive TAFINLAR 150 mg twice daily and trametinib 2 mg once daily or two placebos for up to 1 year. Enrollment required complete resection of melanoma with complete lymphadenectomy within 12 weeks prior to randomization. The trial excluded patients with mucosal or ocular melanoma, unresectable in-transit metastases, distant metastatic disease, or prior systemic anticancer treatment, including radiotherapy. Randomization was stratified by BRAF mutation status (V600E or V600K) and American Joint Committee on Cancer (AJCC; 7th Edition) stage (IIIa, IIIb, or IIIc). (…)<br>(…) In COMBI-AD, a total of 870 patients were randomized: 438 to TAFINLAR in combination with trametinib and 432 to placebo. Median age was 51 years (range 18-89), 55% were male, 99% were White, and 91% had an ECOG performance status of 0. Disease characteristics were AJCC Stage IIIa (18%), Stage IIIb (41%), Stage IIIc (40%), stage unknown (1%); BRAF V600E mutation (91%), BRAF V600K mutation (9%); macroscopic lymph nodes (65%); and tumor ulceration (41%). The median duration of follow-up (time from randomization to last contact or death) was 2.8 years. (See Table 14) (…)<br>**14.4 BRAF V600E Mutation-Positive Metastatic Non-Small Cell Lung Cancer (NSCLC)**<br>In Study BRF113928 (NCT01336634), the safety and efficacy of TAFINLAR alone or administered with trametinib were evaluated in a multi-center, three-cohort, non-randomized, activity-estimating, open-label trial. Key eligibility criteria were locally confirmed BRAF V600E mutation-positive metastatic NSCLC, no prior exposure to BRAF or MEK-inhibitor, and absence of EGFR mutation or ALK rearrangement (unless patients had progression on prior tyrosine kinase inhibitor therapy). (…)<br>(…) In a subgroup analysis of patients with retrospectively, centrally confirmed BRAF V600E mutation-positive NSCLC with the Oncomine™ Dx Target Test, the ORR results were similar to those presented in Table 15. (See Table 15)

NDA/ANDA/BLA Number, Label Version Date	Drug	Therapeutic Area*	Biomarker†	Labeling Sections	Labeling Text‡
					**14.5 BRAF V600E Mutation-Positive Locally Advanced or Metastatic Anaplastic Thyroid Cancer (ATC)**<br>The safety and efficacy of TAFINLAR administered with trametinib was evaluated in Study BRF117019 (NCT02034110), an activity estimating, nine-cohort, multi-center, non-randomized, open-label trial in patients with rare cancers with the BRAF V600E mutation, including locally advanced, unresectable, or metastatic anaplastic thyroid cancer (ATC) with no standard locoregional treatment options. Trial BRF117019 excluded patients who could not swallow or retain the medication; who received prior treatment with BRAF or MEK inhibitors; with symptomatic or untreated CNS metastases; or who had airway obstruction. (…)<br>**14.6 BRAF V600E Mutation-Positive Unresectable or Metastatic Solid Tumors**<br>The safety and efficacy of TAFINLAR in combination with trametinib for the treatment of BRAF V600E mutation-positive unresectable or metastatic solid tumors were evaluated in Trials BRF117019, NCI-MATCH, and CTMT212X2101, and supported by results in COMBI-d, COMBI-v [see Clinical Studies (14.2)], and BRF113928 [see Clinical Studies (14.4)]. In adult studies, patients received TAFINLAR 150 mg twice daily and trametinib 2 mg once daily. The major efficacy outcome measures were ORR per RECIST v1.1, RANO [HGG] or modified RANO [LGG] criteria and duration of response (DoR).<br><u>BRF117019 Study and NCI-MATCH Study</u><br>Study BRF117019 (NCT02034110) [see Clinical Studies (14.5)] is a multi-cohort, multi-center, nonrandomized, open-label trial in adult patients with selected tumors with the BRAF V600E mutation, including high grade glioma (HGG) (n = 45), biliary tract cancer (BTC) (n = 43), low grade glioma (LGG) (n = 13), adenocarcinoma of small intestine (ASI) (n = 3), gastrointestinal stromal tumor (GIST) (n = 1), and anaplastic thyroid cancer [see Clinical Studies (14.5)]. Patients were enrolled based on local assessments of BRAF V600E mutation status; a central laboratory confirmed the BRAF mutation in 93 of 105 patients.<br>Arm H (EAY131-H) of the NCI-MATCH study (NCT02465060) is a single-arm, open-label study that enrolled patients with a BRAF V600E mutation. Patients with melanoma, thyroid cancer, or CRC were excluded. BRAF V600E mutation status for enrollment was determined either by central or local laboratory test. The study included adult patients with solid tumors including gastrointestinal tumors (n = 14), lung tumors (n = 7), gynecologic or peritoneal tumors (n = 6), CNS tumors (n = 4), and ameloblastoma of mandible (n = 1). (See Table 21)<br><u>CTMT212X2101 (X2101) Study</u><br>Study X2101 (NCT02124772) was a multi-center, open-label, multiple cohort study in pediatric patients with refractory or recurrent solid tumors. Part C was a dose escalation of TAFINLAR in combination with trametinib in patients with a BRAF V600E mutation. Part D was a cohort expansion phase of TAFINLAR in combination with trametinib in patients with LGG with a BRAF V600E mutation. The major efficacy outcome measure was ORR as assessed by independent review committee per RANO criteria.<br>The efficacy of TAFINLAR in combination with trametinib was evaluated in 48 pediatric patients, including 34 patients with LGG and 2 patients with HGG.<br>For patients with BRAF V600E mutant LGG and HGG in Parts C and D, the median age was 10 years (range: 1-17); 50% were male, 75% White, 8% Asian, 3% Black; and 58% had Karnofsky/Lansky performance status of 100. Prior anti-cancer treatments included surgery (83%), and external beam radiotherapy (2.8%), and systemic therapy (92%). The ORR was 25% (95% CI: 12%, 42%). For the 9 patients who responded, DoR was ≥6 months for 78% of patients and ≥24 months for 44% of patients.<br><u>CDRB436G2201 (G2201) Study – High-Grade Glioma Cohort</u><br>Study G2201 (NCT02684058) was a multi-center, randomized, open-label, Phase II study of dabrafenib and trametinib in chemotherapy naïve pediatric patients with BRAF V600E mutant low-grade glioma (LGG) and patients with relapsed or progressive BRAF V600E mutant HGG. Patients with HGG were enrolled in a singlearm cohort. The major efficacy outcome measure for the HGG cohort was ORR as assessed by independent review committee per RANO 2010 criteria.<br>**14.7 BRAF V600E Mutation-Positive Low-Grade Glioma**<br><u>CDRB436G2201 (G2201) Study – Low-Grade Glioma Cohort</u><br>The safety and efficacy of TAFINLAR in combination with trametinib for the treatment of BRAF V600E mutation-positive low-grade glioma (LGG) in pediatric patients aged 1 to < 18 years of age were evaluated in the multi-center, open-label trial (Study CDRB436G2201; NCT02684058). Patients with LGG (WHO grades 1 and 2) who required first systemic therapy were randomized in a 2:1 ratio to dabrafenib plus trametinib (D + T) or carboplatin plus vincristine (C + V). BRAF mutation status was identified prospectively via a local assessment or a central laboratory test. In addition, retrospective testing of available tumor samples by the central laboratory was performed to evaluate BRAF V600E mutation status. (…)
**202806, 05/26/2023**	Dabrafenib (2)	Oncology	G6PD	Warnings and Precautions, Adverse Reactions, Patient Counseling Information	**5 WARNINGS AND PRECAUTIONS**<br>**5.9 Glucose-6-Phosphate Dehydrogenase Deficiency**<br>TAFINLAR, which contains a sulfonamide moiety, confers a potential risk of hemolytic anemia in patients with glucose-6-phosphate dehydrogenase (G6PD) deficiency. Monitor patients with G6PD deficiency for signs of hemolytic anemia while taking TAFINLAR.<br><br>**6 ADVERSE REACTIONS**<br>**6.1 Clinical Trials Experience**<br><u>Metastatic or Unresectable BRAF V600E or V600K Mutation-Positive Melanoma</u><br>*TAFINLAR as a Single Agent*<br>Table 3 and Table 4 present adverse drug reactions identified from analyses of the BREAK-3 study [see Clinical Studies (14.1)]. This study, a multicenter, international, open-label, randomized (3:1), controlled trial allocated 250 patients with unresectable or metastatic BRAF V600E mutation-positive melanoma to receive TAFINLAR 150 mg orally twice daily (n = 187) or dacarbazine 1,000 mg/m2 intravenously every 3 weeks (n = 63). The trial excluded patients with abnormal left ventricular ejection fraction or cardiac valve morphology (≥ Grade 2), corrected QT interval greater than or equal to 480 milliseconds on electrocardiogram, or a known history of glucose-6-phosphate dehydrogenase deficiency. (…)<br><br>**17 PATIENT COUNSELING INFORMATION**<br>*Glucose-6-phosphate dehydrogenase (G6PD) deficiency*<br>TAFINLAR may cause hemolytic anemia in patients with G6PD deficiency. Advice patients with known G6PD deficiency to contact their healthcare provider to report signs or symptoms of anemia or hemolysis [see Warnings and Precautions (5.9)].

* Therapeutic areas do not necessarily reflect the CDER review division.
† Representative biomarkers are listed based on standard nomenclature as per the Human Genome Organization (HUGO) symbol and/or simplified descriptors using other common conventions. Listed biomarkers do not necessarily reflect the terminology used in labeling. The term "Nonspecific" is provided when labeling does not explicitly identify the specific biomarker(s) or when the biomarker is represented by a molecular phenotype or gene signature, and in some cases the biomarker was inferred based on the labeling language.
‡ Referenced figures and tables may be found in the labeling available at Drugs@FDA.
Blue text represents the most recent addtions and/or changes since last posted version.

Table of Pharmacogenomic Biomarkers in Drug Labeling
Last Updated: 06/2024

NDA/ANDA/BLA Number, Label Version Date	Drug	Therapeutic Area*	Biomarker†	Labeling Sections	Labeling Text‡
**202806, 05/26/2023**	Dabrafenib (3)	Oncology	RAS	Dosage and Administration, Warnings and Precautions	**2 DOSAGE AND ADMINISTRATION**<br>**2.3 Dosage Modifications for Adverse Reactions**<br>Permanently discontinue TAFINLAR in patients who develop RAS mutation-positive non-cutaneous malignancies. (See Table 2)<br><br>**5 WARNINGS AND PRECAUTIONS**<br>**5.1 New Primary Malignancies**<br>*Non-cutaneous Malignancies*<br>Based on its mechanism of action, TAFINLAR may promote the growth and development of malignancies with activation of RAS through mutation or other mechanisms [see Warnings and Precautions (5.2)]. Across clinical trials of TAFINLAR monotherapy and TAFINLAR administered with trametinib, non-cutaneous malignancies occurred in 1% of patients.<br>Monitor patients receiving TAFINLAR for signs or symptoms of non-cutaneous malignancies. Permanently discontinue TAFINLAR for RAS mutation-positive non-cutaneous malignancies [see Dosage and Administration (2.7)].
**206843, 11/09/2017**	Daclatasvir	Infectious Diseases	IFNL3 (IL28B)	Clinical Studies	**14 CLINICAL STUDIES**<br>**14.2 Clinical Trials in HCV Genotype 3 (ALLY-3)**<br>(…) The 152 treated subjects in ALLY-3 had a median age of 55 years (range, 24-73); 59% of the subjects were male; 90% were white, 5% were Asian, and 4% were black. Most subjects (76%) had baseline HCV RNA levels greater than or equal to 800,000 IU per mL; 21% of the subjects had compensated cirrhosis, and 40% had the IL28B rs12979860 CC genotype.<br>SVR12 and outcomes in subjects without SVR12 in ALLY-3 are shown by patient population in Table 13. SVR12 rates were comparable regardless of HCV treatment history, age, gender, IL28B allele status, or baseline HCV RNA level. For SVR outcomes related to baseline NS5A amino acid polymorphisms, see Microbiology (12.4). (…)<br>**14.3 Clinical Trials in HCV/HIV Coinfected Subjects (ALLY-2)**<br>(…) Most subjects (80%) had baseline HCV RNA levels greater than or equal to 800,000 IU per mL; 16% of the subjects had compensated cirrhosis, and 73% had IL28B rs12979860 non-CC genotype. (…)<br>(…) SVR12 rates were comparable regardless of antiretroviral therapy, HCV treatment history, age, race, gender, IL28B allele status, HCV genotype 1 subtype, or baseline HCV RNA level. For SVR outcomes related to baseline NS5A amino acid polymorphisms, see Microbiology (12.4). (…)<br>**14.4 Clinical Trials in Subjects with Child-Pugh A, B, or C Cirrhosis or with HCV Recurrence after Liver Transplantation (ALLY-1)**<br>(…) Fifty-eight percent of subjects had HCV genotype 1a, 19% had HCV genotype 1b, 4% had genotype 2, 15% had genotype 3, 4% had genotype 4, and 1% had genotype 6, 77% had IL28B rs12979860 non-CC genotype. (…)<br>(…) SVR12 rates were comparable regardless of age, gender, IL28B allele status, or baseline HCV RNA level. For SVR12 outcomes related to baseline NS5A amino acid polymorphisms, see Microbiology (12.4). (…)
**211288, 09/27/2018**	Dacomitinib	Oncology	EGFR	Indications and Usage, Dosage and Administration, Adverse Reactions, Use in Specific Populations, Clinical Studies	**1 INDICATIONS AND USAGE**<br>VIZIMPRO is indicated for the first-line treatment of patients with metastatic non-small cell lung cancer (NSCLC) with epidermal growth factor receptor (EGFR) exon 19 deletion or exon 21 L858R substitution mutations as detected by an FDA-approved test [see Dosage and Administration (2.1)].<br><br>**2 DOSAGE AND ADMINISTRATION**<br>**2.1 Patient Selection**<br>Select patients for the first-line treatment of metastatic NSCLC with VIZIMPRO based on the presence of an EGFR exon 19 deletion or exon 21 L858R substitution mutation in tumor specimens. Information on FDA-approved tests for the detection of EGFR mutations in NSCLC is available at: http://www.fda.gov/CompanionDiagnostics.<br><br>**6 ADVERSE REACTIONS**<br>**6.1 Clinical Trials Experience**<br>(…) The data in the Warnings and Precautions section reflect exposure to VIZIMPRO in 394 patients with first-line or previously treated NSCLC with EGFR exon 19 deletion or exon 21 L858R substitution mutations who received VIZIMPRO at the recommended dose of 45 mg once daily in 4 randomized, active-controlled trial [ARCHER 1050 (N=227), Study A7471009 (N=38), Study A7471011 (N=83), and Study A7471028 (N=16)]<br>and one single-arm trial [Study A7471017 (N=30)]. The median duration of exposure to VIZIMPRO was 10.8 months (range 0.07-68) [see Warnings and Precautions (5)].<br>The data described below reflect exposure to VIZIMPRO in 227 patients with EGFR mutation-positive, metastatic NSCLC enrolled in a randomized, active-controlled trial (ARCHER 1050); 224 patients received gefitinib 250 mg orally once daily in the active control arm [see Clinical Studies (14)].<br><br>**8 USE IN SPECIFIC POPULATIONS**<br>**8.5 Geriatric Use**<br>Of the total number of patients (N=394) in five clinical studies with EGFR mutation-positive NSCLC who received VIZIMPRO at a dose of 45 mg orally once daily [ARCHER 1050 (N=227), Study A7471009 (N=38), Study A7471011 (N=83), Study A7471028 (N=16), and Study A7471017 (N=30)] 40% were 65 years of age and older. (…)<br><br>**14 CLINICAL STUDIES**<br>The efficacy of VIZIMPRO was demonstrated in a randomized, multicenter, multinational, open-label study (ARCHER 1050; [NCT01774721]). Patients were required to have unresectable, metastatic NSCLC with no prior therapy for metastatic disease or recurrent disease with a minimum of 12 months disease-free after completion of systemic therapy; an Eastern Cooperative Oncology Group (ECOG) performance status of 0 or 1; EGFR exon 19 deletion or exon 21 L858R

* Therapeutic areas do not necessarily reflect the CDER review division.
† Representative biomarkers are listed based on standard nomenclature as per the Human Genome Organization (HUGO) symbol and/or simplified descriptors using other common conventions. Listed biomarkers do not necessarily reflect the terminology used in labeling. The term “Nonspecific” is provided when labeling does not explicitly identify the specific biomarker(s) or when the biomarker is represented by a molecular phenotype or gene signature, and in some cases the biomarker was inferred based on the labeling language.
‡ Referenced figures and tables may be found in the labeling available at Drugs@FDA.
Blue text represents the most recent addtions and/or changes since last posted version.

NDA/ANDA/BLA Number, Label Version Date	Drug	Therapeutic Area*	Biomarker†	Labeling Sections	Labeling Text‡
					substitution mutations. EGFR mutation status was prospectively determined by local laboratory or commercially available tests (e.g., therascreen® EGFR RGQ PCR and cobas® EGFR Mutation Test).<br>Patients were randomized (1:1) to receive VIZIMPRO 45 mg orally once daily or gefitinib 250 mg orally once daily until disease progression or unacceptable toxicity. Randomization was stratified by region (Japanese versus mainland Chinese versus other East Asian versus non-East Asian), and EGFR mutation status (exon 19 deletions versus exon 21 L858R substitution mutation). (…)<br>(…) Prognostic and tumor characteristics were ECOG performance status 0 (30%) or 1 (70%); 59% with exon 19 deletion and 41% with exon 21 L858R substitution; Stage IIIB (8%) and Stage IV (92%); 64% were never smokers; and 1% received prior adjuvant or neoadjuvant therapy. (…)
**021794, 05/18/2018**	Dapsone (1)	Dermatology	G6PD	Warnings and Precautions, Use in Specific Populations, Patient Counseling Information	**5 WARNINGS AND PRECAUTIONS**<br>**5.2 Hematologic Effects**<br>Oral dapsone treatment has produced dose-related hemolysis and hemolytic anemia. Individuals with glucose-6 phosphate dehydrogenase (G6PD) deficiency are more prone to hemolysis with the use of certain drugs. G6PD deficiency is most prevalent in populations of African, South Asian, Middle Eastern, and Mediterranean ancestry.<br>Some subjects with G6PD deficiency using ACZONE® Gel developed laboratory changes suggestive of hemolysis. There was no evidence of clinically relevant hemolysis or anemia in patients treated with ACZONE® Gel, 5%, including patients who were G6PD deficient.<br>Discontinue ACZONE® Gel, 5%, if signs and symptoms suggestive of hemolytic anemia occur. Avoid use of ACZONE® Gel, 5% in patients who are taking oral dapsone or antimalarial medications because of the potential for hemolytic reactions. Combination of ACZONE® Gel, 5%, with trimethoprim/sulfamethoxazole (TMP/SMX) may increase the likelihood of hemolysis in patients with G6PD deficiency.<br><br>**8 USE IN SPECIFIC POPULATIONS**<br>**8.2 Lactation Risk**<br>Summary There is no information regarding the presence of topical dapsone in breastmilk, the effects on the breastfed infant, or the effects on milk production. Orally administered dapsone appears in human milk and could result in hemolytic anemia and hyperbilirubinemia especially in infants with G6PD deficiency. Systemic absorption of dapsone following topical application is minimal relative to oral dapsone administration; however, it is known that dapsone is present in human milk following administration of oral dapsone.<br>**8.6 G6PD Deficiency**<br>ACZONE® Gel, 5% and vehicle were evaluated in a randomized, double-blind, cross-over design clinical study of 64 patients with G6PD deficiency and acne vulgaris. Subjects were Black (88%), Asian (6%), Hispanic (2%) or of other racial origin (5%). Blood samples were taken at Baseline, Week 2, and Week 12 during both vehicle and ACZONE® Gel, 5% treatment periods. There were 56 out of 64 subjects who had a Week 2 blood draw and applied at least 50% of treatment applications. Table 3 contains results from testing of relevant hematology parameters for these two treatment periods. ACZONE® Gel was associated with a 0.32 g/dL drop in hemoglobin after two weeks of treatment, but hemoglobin levels generally returned to baseline levels at Week 12. (See Table 3)<br>There were no changes from baseline in haptoglobin or lactate dehydrogenase during ACZONE® or vehicle treatment at either the 2-week or 12-week time point. The proportion of subjects who experienced decreases in hemoglobin ≥1 g/dL was similar between ACZONE® Gel, 5% and vehicle treatment (8 of 58 subjects had such decreases during ACZONE® treatment compared to 7 of 56 subjects during vehicle treatment among subjects with at least one on-treatment hemoglobin assessment). Subgroups based on gender, race, or G6PD enzyme activity did not display any differences in laboratory results from the overall study group. There was no evidence of clinically significant hemolytic anemia in this study. Some of these subjects developed laboratory changes suggestive of hemolysis.<br><br>**17 PATIENT COUNSELING INFORMATION**<br>Advise the patient to read the FDA-approved patient labeling (Patient Information).<br>*Hematological Effects*<br>• Inform patients that methemoglobinemia can occur with topical dapsone treatment. Advise patients to seek immediate medical attention if they develop cyanosis [see Warnings and Precautions (5.1)].<br>• Inform patients who have G6PD deficiency that hemolytic anemia may occur with topical dapsone treatment. Advise patients to seek medical attention if they develop signs and symptoms suggestive of hemolytic anemia [see Warnings and Precautions (5.2)].
**021794, 05/18/2018**	Dapsone (2)	Dermatology	Nonspecific (Congenital Methemoglobinemia)	Warnings and Precautions, Adverse Reactions, Patient Counseling Information	**5 WARNINGS AND PRECAUTIONS**<br>**5.1 Methemoglobinemia**<br>Cases of methemoglobinemia, with resultant hospitalization, have been reported postmarketing in association with ACZONE® Gel, 5% treatment. Patients with glucose-6-phosphate dehydrogenase deficiency or congenital or idiopathic methemoglobinemia are more susceptible to drug-induced methemoglobinemia. Avoid use of ACZONE® Gel, 5% in those patients with congenital or idiopathic methemoglobinemia.<br>Signs and symptoms of methemoglobinemia may be delayed some hours after exposure. Initial signs and symptoms of methemoglobinemia are characterized by a slate grey cyanosis seen in, e.g., buccal mucous membranes, lips and nail beds. Advise patients to discontinue ACZONE® Gel, 5% and seek immediate medical attention in the event of cyanosis.<br>Dapsone can cause elevated methemoglobin levels particularly in conjunction with methemoglobin-inducing agents.<br><br>**6 ADVERSE REACTIONS**<br>**6.3 Postmarketing Experience**<br>Because these reactions are reported voluntarily from a population of uncertain size, it is not always possible to reliably estimate their frequency or establish a causal relationship to drug exposure. The following adverse reactions have been identified during post-approval use of topical dapsone: methemoglobinemia, rash (including erythematous rash, application site rash) and swelling of face (including lip swelling, eye swelling).

* Therapeutic areas do not necessarily reflect the CDER review division.
† Representative biomarkers are listed based on standard nomenclature as per the Human Genome Organization (HUGO) symbol and/or simplified descriptors using other common conventions. Listed biomarkers do not necessarily reflect the terminology used in labeling. The term "Nonspecific" is provided when labeling does not explicitly identify the specific biomarker(s) or when the biomarker is represented by a molecular phenotype or gene signature, and in some cases the biomarker was inferred based on the labeling language.
‡ Referenced figures and tables may be found in the labeling available at Drugs@FDA.
Blue text represents the most recent addtions and/or changes since last posted version.

NDA/ANDA/BLA Number, Label Version Date	Drug	Therapeutic Area*	Biomarker†	Labeling Sections	Labeling Text‡
					**17 PATIENT COUNSELING INFORMATION**<br>Advise the patient to read the FDA-approved patient labeling (Patient Information).<br>*Hematological Effects*<br>• Inform patients that methemoglobinemia can occur with topical dapsone treatment. Advise patients to seek immediate medical attention if they develop cyanosis [see Warnings and Precautions (5.1)].<br>• Inform patients who have G6PD deficiency that hemolytic anemia may occur with topical dapsone treatment. Advise patients to seek medical attention if they develop signs and symptoms suggestive of hemolytic anemia [see Warnings and Precautions (5.2)].
**086841**	**Dapsone (3)**	Infectious Diseases	G6PD	Precautions, Adverse Reactions, Overdosage	Labeling not electronically available on Drugs@FDA
**021513, 03/15/2012**	**Darifenacin**	Urology	CYP2D6	Clinical Pharmacology	**12 CLINICAL PHARMACOLOGY**<br>**12.2 Pharmacodynamics**<br>*Electrophysiology*<br>The effect of six-day treatment of 15 mg and 75 mg Enablex on QT/QTc interval was evaluated in a multiple-dose, double-blind, randomized, placebo- and active-controlled (moxifloxacin 400 mg) parallel-arm design study in 179 healthy adults (44 percent male, 56 percent female) aged 18 to 65. Subjects included 18 percent poor metabolizer (PMs) and 82 percent extensive metabolizer (EMs). The QT interval was measured over a 24-hour period both predosing and at steady-state. The 75 mg Enablex dose was chosen because this achieves exposure similar to that observed in CYP2D6 poor metabolizers administered the highest recommended dose (15 mg) of darifenacin in the presence of a potent CYP3A4 inhibitor. At the doses studied, Enablex did not result in QT/QTc interval prolongation at any time during the steady-state, while moxifloxacin treatment resulted in a mean increase from baseline QTcF of about 7.0 msec when compared to placebo. In this study, darifenacin 15 mg and 75 mg doses demonstrated a mean heart rate change of 3.1 and 1.3 bpm, respectively, when compared to placebo. However, in the clinical efficacy and safety studies, the change in median HR following treatment with Enablex was no different from placebo.<br>**12.3 Pharmacokinetics**<br>*Absorption*<br>After oral administration of Enablex to healthy volunteers, peak plasma concentrations of darifenacin are reached approximately seven hours after multiple dosing and steady-state plasma concentrations are achieved by the sixth day of dosing. The mean (SD) steady-state time course of Enablex 7.5 mg and 15 mg extended-release tablets is depicted in Figure 1.<br>A summary of mean (standard deviation, SD) steady-state pharmacokinetic parameters of Enablex 7.5 mg and 15 mg extended-release tablets in EMs and PMs of CYP2D6 is provided in Table 3.<br>The mean oral bioavailability of Enablex in EMs at steady-state is estimated to be 15 percent and 19 percent for 7.5 mg and 15 mg tablets, respectively. (See Figure 1 and Table 3)<br>*Variability in Metabolism*<br>A subset of individuals (approximately 7 percent Caucasians and 2 percent African Americans) are poor metabolizers (PMs) of CYP2D6 metabolized drugs. Individuals with normal CYP2D6 activity are referred to as extensive metabolizers (EMs). The metabolism of darifenacin in PMs will be principally mediated via CYP3A4. The darifenacin ratios (PM versus EM) for Cmax and AUC following darifenacin 15 mg once daily at steadystate were 1.9 and 1.7, respectively.<br>*Excretion*<br>Following administration of an oral dose of 14C-darifenacin solution to healthy volunteers, approximately 60 percent of the radioactivity was recovered in the urine and 40 percent in the feces. Only a small percentage of the excreted dose was unchanged darifenacin (3 percent). Estimated darifenacin clearance is 40 L/h for EMs and 32 L/h for PMs. The elimination half-life of darifenacin following chronic dosing is approximately 13 to 19 hours.<br>*Drug-Drug Interactions*<br>*CYP3A4 Inhibitors*<br>In a drug interaction study, when a 7.5 mg once daily dose of Enablex was given to steadystate and co-administered with the potent CYP3A4 inhibitor ketoconazole 400 mg, mean darifenacin Cmax increased to 11.2 ng/mL for EMs (n = 10) and 55.4 ng/mL for one PM subject (n = 1). Mean AUC increased to 143 and 939 ng•h/mL for EMs and for one PM subject, respectively. When a 15 mg daily dose of Enablex was given with ketoconazole, mean darifenacin Cmax increased to 67.6 ng/mL and 58.9 ng/mL for EMs (n = 3) and one PM subject (n = 1), respectively. Mean AUC increased to 1110 and 931 ng•h/mL for EMs and for one PM subject, respectively [see Dosage and Administration (2) and Drug Interactions (7.1)].
**206619, 07/23/2018**	**Dasabuvir, Ombitasvir, Paritaprevir, and Ritonavir**	Infectious Diseases	IFNL3 (IL28B)	Clinical Studies	**14 CLINICAL STUDIES**<br>**14.2 Clinical Trial Results in Adults with Chronic HCV Genotype 1a and 1b Infection without Cirrhosis**<br>*Subjects with Chronic HCV GT1a Infection without Cirrhosis*<br>Subjects with HCV GT1a infection without cirrhosis treated with VIEKIRA PAK with RBV for 12 weeks in SAPPHIRE-I and -II and in PEARL-IV [see Clinical Studies (14.1)] had a median age of 53 years (range: 18 to 70); 63% of the subjects were male; 90% were White; 7% were Black/African American; 8% were Hispanic or Latino; 19% had a body mass index of at least 30 kg per m^2; 55% of patients were enrolled in US sites; 72% had IL28B (rs12979860) non-CC genotype; 85% had baseline HCV RNA levels of at least 800,000 IU per mL. (…)<br>*Subjects with Chronic HCV GT1b Infection without Cirrhosis*<br>Subjects with HCV GT1b infection without cirrhosis were treated with VIEKIRA PAK with or without RBV for 12 weeks in PEARL-II and -III [see Clinical Studies (14.1)]. Subjects had a median age of 52 years (range: 22 to 70); 47% of the subjects were male; 93% were White; 5% were Black/African American; 2% were

NDA/ANDA/BLA Number, Label Version Date	Drug	Therapeutic Area*	Biomarker†	Labeling Sections	Labeling Text‡
					Hispanic or Latino; 21% had a body mass index of at least 30 kg per m^2; 21% of patients were enrolled in US sites; 83% had IL28B (rs12979860) nonCC genotype; 77% had baseline HCV RNA levels of at least 800,000 IU per mL. (…)<br>**14.3 Clinical Trial Results in Adults with Chronic HCV Genotype 1a and 1b Infection and Compensated Cirrhosis**<br>(…) Treated subjects had a median age of 58 years (range: 21 to 71); 70% of the subjects were male; 95% were White; 3% were Black/African American; 12% were Hispanic or Latino; 28% had a body mass index of at least 30 kg per m^2; 43% of patients were enrolled in US sites; 82% had IL28B (rs12979860) non-CC genotype; 86% had baseline HCV RNA levels of at least 800,000 IU per mL; 69% had HCV GT1a infection, 31% had HCV GT1b infection; 42% were treatment-naïve, 36% were prior pegIFN/RBV null responders; 8% were prior pegIFN/RBV partial responders, 14% were prior pegIFN/RBV relapsers; 15% had platelet counts of less than 90 x 10^9 per L; 50% had albumin less than 4.0 mg per dL. (…)<br>(…) TURQUOISE-III was an open-label trial that enrolled 60 HCV GT1b-infected subjects with cirrhosis and mild hepatic impairment (Child-Pugh A) who were either treatment-naïve or did not achieve SVR with prior treatment with pegIFN/RBV. Subjects received VIEKIRA PAK without RBV for 12 weeks. Treated subjects had a median age of 61 years (range: 26 to 78); including 45% treatment-naïve and 55% pegIFN/RBV treatment-experienced; 25% were ≥65 years; 62% were male; 12% were Black; 5% were Hispanic or Latino; 28% had a body mass index of at least 30 kg per m^2; 40% of patients were enrolled in US sites; 22% had platelet counts of less than 90 x 10^9 per L; 17% had albumin less than 35 g/L; 92% had baseline HCV RNA levels of at least 800,000 IU per mL; 83% had IL28B (rs12979860) non-CC genotype. (…)<br>**14.6 Clinical Trial in Subjects with HCV/HIV-1 Co-infection (TURQUOISE-I)**<br>(…) Treated subjects had a median age of 51 years (range: 31 to 69); 24% of subjects were black; 81% of subjects had IL28B (rs12979860) non-CC genotype; 19% of subjects had compensated cirrhosis; 67% of subjects were HCV treatment-naïve; 33% of subjects had failed prior treatment with pegIFN/RBV; 89% of subjects had HCV genotype 1a infection. (…)
**021986, 12/21/2018**	Dasatinib	Oncology	BCR-ABL1 (Philadelphia chromosome)	Indications and Usage, Dosage and Administration, Warnings and Precautions, Adverse Reactions, Use in Specific Populations, Clinical Studies	**1 INDICATIONS AND USAGE**<br>SPRYCEL (dasatinib) is indicated for the treatment of adults with<br>• newly diagnosed Philadelphia chromosome-positive (Ph+) chronic myeloid leukemia (CML) in chronic phase.<br>• chronic, accelerated, or myeloid or lymphoid blast phase Ph+ CML with resistance or intolerance to prior therapy including imatinib.<br>• Philadelphia chromosome-positive acute lymphoblastic leukemia (Ph+ ALL) with resistance or intolerance to prior therapy.<br>SPRYCEL (dasatinib) is indicated for the treatment of pediatric patients 1 year of age and older with<br>• Ph+ CML in chronic phase.<br>• newly diagnosed Ph+ ALL in combination with chemotherapy.<br><br>**2 DOSAGE AND ADMINISTRATION**<br>**2.1 Dosage of SPRYCEL in Adult Patients**<br>The recommended starting dosage of SPRYCEL for chronic phase CML in adults is 100 mg administered orally once daily. The recommended starting dosage of SPRYCEL for accelerated phase CML, myeloid or lymphoid blast phase CML, or Ph+ ALL in adults is 140 mg administered orally once daily. Tablets should not be crushed, cut, or chewed; they should be swallowed whole. SPRYCEL can be taken with or without a meal, either in the morning or in the evening.<br>**2.2 Dosage of SPRYCEL in Pediatric Patients with CML or Ph+ ALL**<br>The recommended starting dosage for pediatrics is based on body weight as shown in Table 1. The recommended dose should be administered orally once daily with or without food. Recalculate the dose every 3 months based on changes in body weight, or more often if necessary. (See Table 1)<br>Refer to Section 2.4 for recommendations on dose escalation in adults with CML and Ph+ ALL, and pediatric patients with CML.<br>**2.4 Dose Escalation in Adults with CML and Ph+ ALL, and Pediatric Patients with CML**<br>For adult patients with CML and Ph+ ALL, consider dose escalation to 140 mg once daily (chronic phase CML) or 180 mg once daily (advanced phase CML and Ph+ ALL) in patients who do not achieve a hematologic or cytogenetic response at the recommended starting dosage. For pediatric patients with CML, consider dose escalation to 120 mg once daily (see Table 2 below). Dose escalation is not recommended for pediatric patients with Ph+ ALL, where SPRYCEL is administered in combination with chemotherapy. Escalate the SPRYCEL dose as shown in Table 2 in pediatric patients with chronic phase CML who do not achieve a hematologic or cytogenetic response at the recommended starting dosage. (See Tables 2, 3, and 4)<br>**2.5 Dose Adjustment for Adverse Reactions**<br>*Myelosuppression*<br>(…) For pediatric patients with Ph+ ALL, if neutropenia and/or thrombocytopenia result in a delay of the next block of treatment by more than 14 days, interrupt SPRYCEL and resume at the same dose level once the next block of treatment is started. (…)<br>Non-Hematologic Adverse Reactions<br>For adults with Ph+ CML and ALL, and pediatric patients with Ph+ CML, if a severe nonhematologic adverse reaction develops with SPRYCEL use, treatment must be withheld until the event has resolved or improved. Thereafter, treatment can be resumed as appropriate at a reduced dose depending on the severity and recurrence of the event [see Warnings and Precautions (5.1)]. For pediatric patients with Ph+ ALL, interrupt treatment for cases of grade > 3 non-hematologic adverse reactions with the exception of liver function test abnormalities, and resume at a reduced dose when resolved to grade <1. (…)<br>**2.6 Duration of Treatment**<br>In clinical studies, treatment with SPRYCEL in adults and in pediatric patients with chronic phase CML was continued until disease progression or until no longer tolerated by the patient. The effect of stopping treatment on long-term disease outcome after the achievement of a cytogenetic response (including complete cytogenetic response [CCyR]) or major molecular response (MMR and MR4.5) has not been established.<br>In clinical studies, treatment with SPRYCEL in pediatric patients with Ph+ ALL was administered for a maximum duration of 2 years [see Dosage and Administration (2.2) and Clinical Studies (14.4)]. SPRYCEL is an antineoplastic product.<br>Follow applicable special handling and disposal procedures.<br><br>**5 WARNINGS AND PRECAUTIONS**

Table of Pharmacogenomic Biomarkers in Drug Labeling

Last Updated: 06/2024

NDA/ANDA/BLA Number, Label Version Date	Drug	Therapeutic Area*	Biomarker†	Labeling Sections	Labeling Text‡
					**5.1 Myelosuppression**<br>Treatment with SPRYCEL is associated with severe (NCI CTC Grade 3 or 4) thrombocytopenia, neutropenia, and anemia, which occur earlier and more frequently in patients with advanced phase CML or Ph+ ALL than in patients with chronic phase CML.<br>In patients with chronic phase CML, perform complete blood counts (CBCs) every 2 weeks for 12 weeks, then every 3 months thereafter, or as clinically indicated. In patients with advanced phase CML or Ph+ ALL, perform CBCs weekly for the first 2 months and then monthly thereafter, or as clinically indicated.<br>(…)<br>In pediatric patients with Ph+ ALL treated with SPRYCEL in combination with chemotherapy, perform CBCs prior to the start of each block of chemotherapy and as clinically indicated. During the consolidation blocks of chemotherapy, perform CBCs every 2 days until recovery. (…)<br>**5.2 Bleeding-Related Events**<br>In addition to causing thrombocytopenia in human subjects, dasatinib caused platelet dysfunction in vitro. In all CML or Ph+ ALL clinical studies, ≥grade 3 central nervous system (CNS) hemorrhages, including fatalities, occurred in <1% of patients receiving SPRYCEL. Grade 3 or greater gastrointestinal hemorrhage, including fatalities, occurred in 4% of patients and generally required treatment interruptions and transfusions. Other cases of ≥grade 3 hemorrhage occurred in 2% of patients. Most bleeding events in clinical studies were associated with severe thrombocytopenia.<br>Concomitant medications that inhibit platelet function or anticoagulants may increase the risk of hemorrhage.<br>**5.3 Fluid Retention**<br>(…) In patients with advanced phase CML or Ph+ ALL treated with SPRYCEL at the recommended dose (n=304), grade 3 or 4 fluid retention was reported in 8% of patients, including grade 3 or 4 pleural effusion reported in 7% of patients. (…)<br><br>**6 ADVERSE REACTIONS**<br>**6.1 Clinical Trials Experience**<br>(…) The data described below reflect exposure to SPRYCEL at all doses tested in clinical studies including 324 patients with newly diagnosed chronic phase CML and in 2388 patients with imatinib-resistant or -intolerant chronic or advanced phase CML or Ph+ ALL. The median duration of therapy in 2712 SPRYCEL-treated patients was 19.2 months (range 0–93.2 months). In a randomized trial in patients with newly diagnosed chronic phase CML, the median duration of therapy was approximately 60 months. The median duration of therapy in 1618 patients with chronic phase CML was 29 months (range 0–92.9 months).<br>The median duration of therapy in 1094 patients with advanced phase CML or Ph+ ALL was 6.2 months (range 0–93.2 months). (…)<br>(…) In the randomized trial in patients with newly diagnosed chronic phase CML, drug was discontinued for adverse reactions in 16% of SPRYCEL-treated patients with a minimum of 60 months of follow-up. After a minimum of 60 months of follow-up, the cumulative discontinuation rate was 39%. Among the 1618 SPRYCEL-treated patients with chronic phase CML, drug-related adverse events leading to discontinuation were reported in 329 (20.3%) patients; among the 1094 SPRYCEL treated patients with advanced phase CML or Ph+ ALL, drug-related adverse events leading to discontinuation were reported in 191 (17.5%) patients. (…)<br>**Philadelphia Chromosome-Positive Acute Lymphoblastic Leukemia (Ph+ ALL) in Adults**<br>A total of 135 patients with Ph+ ALL were treated with SPRYCEL in clinical studies. The median duration of treatment was 3 months (range 0.03–31 months). The safety profile of patients with Ph+ ALL was similar to those with lymphoid blast phase CML. (…)<br>Philadelphia Chromosome-Positive Acute Lymphoblastic Leukemia (Ph+ ALL) in Pediatric Patients<br>The safety of SPRYCEL administered continuously in combination with multiagent chemotherapy was determined in a multicohort study of 81 pediatric patients with newly diagnosed Ph+ ALL. [see Clinical Studies (14.4)]. The median duration of therapy was 24 months (range 2 to 27 months). (See Tables 14 and 15)<br>(…)<br>**6.2 Additional Pooled Data From Clinical Trials**<br>The following additional adverse reactions were reported in patients in SPRYCEL CML and Ph+ ALL clinical studies at a frequency of ≥10%, 1%–<10%, 0.1%–<1%, or <0.1%. These events are included on the basis of clinical relevance.<br><br>**8 USE IN SPECIFIC POPULATIONS**<br>**8.4 Pediatric Use**<br>Ph+ CML in Chronic Phase<br>The safety and effectiveness of SPRYCEL monotherapy have been demonstrated in pediatric patients with newly diagnosed chronic phase CML [see Clinical Studies (14.3)]. There are no data in children under 1 year of age. Adverse reactions associated with bone growth and development were reported in 5 (5.2%) of patients [see Warnings and Precautions (5.10)].<br>Ph+ ALL<br>The safety and effectiveness of SPRYCEL in combination with chemotherapy have been demonstrated in pediatric patients one year and over with newly diagnosed Ph+ ALL. Use of SPRYCEL in pediatric patients is supported by evidence from one pediatric study. There are no data in children under 1 year of age. One case of grade 1 osteopenia was reported.<br>The safety profile of SPRYCEL in pediatric subjects was comparable to that reported in studies in adult subjects [see Adverse Reactions (6.1) and Clinical Studies (14.3, 14.4)].<br>Monitor bone growth and development in pediatric patients [see Warnings and Precautions (5.10)].<br>Pediatric Patients with Difficulty Swallowing Tablets<br>Five patients with Ph+ ALL 2 to 10 years of age received at least one dose of SPRYCEL tablet dispersed in juice on Study CA180372. (…)<br><br>**14 CLINICAL STUDIES**

* Therapeutic areas do not necessarily reflect the CDER review division.
† Representative biomarkers are listed based on standard nomenclature as per the Human Genome Organization (HUGO) symbol and/or simplified descriptors using other common conventions. Listed biomarkers do not necessarily reflect the terminology used in labeling. The term "Nonspecific" is provided when labeling does not explicitly identify the specific biomarker(s) or when the biomarker is represented by a molecular phenotype or gene signature, and in some cases the biomarker was inferred based on the labeling language.
‡ Referenced figures and tables may be found in the labeling available at Drugs@FDA.
Blue text represents the most recent addtions and/or changes since last posted version.

NDA/ANDA/BLA Number, Label Version Date	Drug	Therapeutic Area*	Biomarker†	Labeling Sections	Labeling Text‡
					(…) BCR-ABL sequencing was performed on blood samples from patients in the newly diagnosed trial who discontinued dasatinib or imatinib therapy. Among dasatinib-treated patients the mutations detected were T315I, F317I/L, and V299L. Dasatinib does not appear to be active against the T315I mutation, based on in vitro data.<br>**14.2 Imatinib-Resistant or -Intolerant CML or Ph+ ALL in Adults**<br>The efficacy and safety of SPRYCEL were investigated in adult patients with CML or Ph+ ALL whose disease was resistant to or who were intolerant to imatinib: 1158 patients had chronic phase CML, 858 patients had accelerated phase, myeloid blast phase, or lymphoid blast phase CML, and 130 patients had Ph+ ALL. In a clinical trial in chronic phase CML, resistance to imatinib was defined as failure to achieve a complete hematologic response (CHR; after 3 months), major cytogenetic response (MCyR; after 6 months), or complete cytogenetic response (CCyR; after 12 months); or loss of a previous molecular response (with concurrent ≥10% increase in Ph+ metaphases), cytogenetic response, or hematologic response. (…)<br>(…) The primary efficacy endpoint in chronic phase CML was MCyR, defined as elimination (CCyR) or substantial diminution (by at least 65%, partial cytogenetic response) of Ph+ hematopoietic cells. The primary efficacy endpoint in accelerated phase, myeloid blast phase, lymphoid blast phase CML, and Ph+ ALL was major hematologic response (MaHR), defined as either a CHR or no evidence of leukemia (NEL).<br>*Advanced Phase CML and Ph+ ALL*<br>Dose-Optimization Trial: One randomized open-label trial was conducted in patients with advanced phase CML (accelerated phase CML, myeloid blast phase CML, or lymphoid blast phase CML) to evaluate the efficacy and safety of SPRYCEL administered once daily compared with SPRYCEL administered twice daily. (See Table 19) (…)<br>(…) In patients with Ph+ ALL who were treated with SPRYCEL 140 mg once-daily, the median duration of MaHR was 4.6 months (min-max: 1.4-10.2). The medians of progression-free survival for patients with Ph+ ALL treated with SPRYCEL 140 mg once-daily and 70 mg twicedaily were 4.0 months (min-max: 0.4-11.1) and 3.1 months (min-max: 0.3-20.8), respectively.<br>**14.4 Ph+ ALL in Pediatric Patients**<br>The efficacy of SPRYCEL in combination with chemotherapy was evaluated in a single cohort (cohort 1) of Study CA180372 (NCT01460160), a multicenter, multiple-cohort study of pediatric patients with newly diagnosed B-cell precursor Ph+ ALL. The 78 patients in cohort 1 received SPRYCEL at a daily dose of 60 mg/m2 for up to 24 months, in combination with chemotherapy. The backbone chemotherapy regimen was the AIEOP-BFM ALL 2000 multi-agent chemotherapy protocol. (…)
**103767, 02/10/2020**	Denileukin Diftitox	Oncology	IL2RA (CD25 antigen)	Indications and Usage, Clinical Studies	**1 INDICATIONS AND USAGE**<br>Ontak is indicated for the treatment of patients with persistent or recurrent cutaneous T-cell lymphoma whose malignant cells express the CD25 component of the Il-2 receptor.<br><br>**14 CLINICAL STUDIES**<br>**14.1 Study 1: Placebo Controlled Study in CTCL (Stage Ia to III Patients)**<br>The safety and efficacy of Ontak were evaluated in a randomized, double-blind, placebo-controlled, 3-arm trial in patients with Stage Ia to III CD25(+) CTCL. Eligible patients were required to have expression of CD25 on ≥20% of biopsied malignant cells by immunohistochemistry [see Warnings and Precautions (5.4)] (…)<br>**14.2 Study 2: Dose Evaluation Study in CTCL (Stage IIb to IVa) Patients**<br>A randomized, double-blind study was conducted to evaluate doses of 9 or 18 mcg/kg/day in 71 patients with recurrent or persistent, Stage IIb to IVa CTCL. Entry to this study required demonstration of CD25 expression on at least 20% of the cells in any relevant tumor tissue sample (skin biopsy) or circulating cells. Tumor biopsies were not evaluated for expression of other IL-2 receptor subunit components (CDI22/CD132). (…)
**014399, 11/09/2018**	Desipramine	Psychiatry	CYP2D6	Precautions	**PRECAUTIONS**<br>*Drug Interactions*<br>*Drugs Metabolized by P450 2D6.*<br>The biochemical activity of the drug metabolizing isozyme cytochrome P450 2D6 (debrisoquin hydroxylase) is reduced in a subset of the Caucasian population (about 7% to 10% of Caucasians are so called “poor metabolizers”); reliable estimates of the prevalence of reduced P450 2D6 isozyme activity among Asian, African and other populations are not yet available. Poor metabolizers have higher than expected plasma concentrations of tricyclic antidepressants (TCAs) when given usual doses. Depending on the fraction of drug metabolized by P450 2D6, the increase in plasma concentration may be small, or quite large (8 fold increase in plasma AUC of the TCA). (…)
018938, 07/21/2022	Desmopressin	Hematology	F8	Indications and Usage, Dosage and Administration, Clinical Pharmacology	**1 INDICATIONS AND USAGE**<br>**1.2 Hemophilia A**<br>DDAVP Injection is indicated for patients with hemophilia A with factor VIII coagulant activity levels greater than 5% without factor VIII antibodies to:<br>• Maintain hemostasis during surgical procedures and postoperatively<br>• Reduce bleeding with episodes of spontaneous or traumatic injuries such as hemarthroses, intramuscular hematomas, or mucosal bleeding.<br>**1.3 von Willebrand’s Disease (Type I)**<br>DDAVP Injection is indicated for patients with mild to moderate von Willebrand’s disease (Type I) with factor VIII levels greater than 5% to:<br>• Maintain hemostasis during surgical procedures and postoperatively<br>• Reduce bleeding with episodes of spontaneous or traumatic injuries such as hemarthroses, intramuscular hematomas, or mucosal bleeding.<br>Limitations of Use<br>DDAVP is not indicated for the treatment of severe von Willebrand’s disease (Type I) and when there is evidence of an abnormal molecular form of factor VIII antigen [see Warnings and Precautions (5.2)].<br><br>**2 DOSAGE AND ADMINISTRATION**

* Therapeutic areas do not necessarily reflect the CDER review division.
† Representative biomarkers are listed based on standard nomenclature as per the Human Genome Organization (HUGO) symbol and/or simplified descriptors using other common conventions. Listed biomarkers do not necessarily reflect the terminology used in labeling. The term “Nonspecific” is provided when labeling does not explicitly identify the specific biomarker(s) or when the biomarker is represented by a molecular phenotype or gene signature, and in some cases the biomarker was inferred based on the labeling language.
‡ Referenced figures and tables may be found in the labeling available at Drugs@FDA.
Blue text represents the most recent addtions and/or changes since last posted version.

Table of Pharmacogenomic Biomarkers in Drug Labeling

Last Updated: 06/2024

NDA/ANDA/BLA Number, Label Version Date	Drug	Therapeutic Area*	Biomarker†	Labeling Sections	Labeling Text‡
					**2.1 Pretreatment Testing and On-Treatment Monitoring**<br>Hemophilia A<br>Prior to treatment with DDAVP Injection, verify that factor VIII coagulant activity levels are >5% and exclude the presence of factor VIII autoantibodies. Also assess serum sodium and aPTT prior to treatment. In certain clinical situations, it may be justified to try DDAVP in patients with factor VIII levels between 2% to 5%; however, these patients should be carefully monitored.<br>von Willebrand's Disease (Type I)<br>Prior to treatment with DDAVP Injection, verify that factor VIII coagulant activity levels are >5% and exclude severe von Willebrand's disease (Type I) and presence of abnormal molecular form of factor VIII antigen. During treatment with DDAVP Injection, assess serum sodium, bleeding time, factor VIII coagulant activity, ristocetin cofactor activity, and von Willebrand antigen to ensure that adequate levels are being achieved.<br>*For All Patients Receiving Repeated Doses:* Restrict free water intake and monitor for hyponatremia. Ensure that serum sodium is normal prior to initiating or resuming treatment with DDAVP Injection.<br>**2.2 Recommended Dosage**<br>Hemophilia A and von Willebrand's Disease (Type I):<br>The recommended dosage is 0.3 mcg/kg actual body weight (to a maximum of 20 mcg) administered by intravenous infusion over 15 minutes to 30 minutes. If used preoperatively, administer 30 minutes prior to the procedure. If used to reduce spontaneous or traumatic bleeding, doses may be repeated after 8 hours to 12 hours and once daily thereafter, if needed, based upon clinical condition and von Willebrand factor and factor VIII levels. The necessity for repeat administration of DDAVP or use of any blood products for hemostasis should be determined by laboratory response as well as the clinical condition of the patient. Tachyphylaxis (lessening of response) with repeated administration (i.e., given more frequently than every 48 hours) may occur. The initial response is reproducible if DDAVP is administered every 2 to 3 days.<br><br>**12 CLINICAL PHARMACOLOGY**<br>**12.2 Pharmacodynamics**<br>The response to DDAVP of factor VIII activity and plasminogen activator is dose-related, with maximal plasma levels of 300 to 400 percent change from baseline obtained after infusion of 0.4 mcg/kg. The increase of factor VIII is rapid and evident within 30 minutes, reaching a maximum at a point ranging from 90 minutes to two hours. The duration of the hemostatic effect depends on the half-life for VIII:C which is about 8-12 hours. The percentage increase of factor VIII levels in patients with mild hemophilia A and von Willebrand's disease was not significantly different from that observed in normal healthy individuals when treated with 0.3 mcg/kg of DDAVP infused over 10 minutes. The use of DDAVP Injection in patients with central diabetes insipidus reduces urinary output, increases urine osmolality, and decreases plasma osmolality.
020118,<br>11/01/2022	**Desflurane**	Anesthesiolgy	CACNA1S, RYR1 (Genetic Susceptibility to Malignant Hyperthermia)	Contraindications, Warnings and Precautions, Clinical Pharmacology	**4 CONTRAINDICATIONS**<br>The use of SUPRANE is contraindicated in the following conditions:<br>• Known or suspected genetic susceptibility to malignant hyperthermia. (…)<br><br>**5 WARNINGS AND PRECAUTIONS**<br>**5.1 Malignant Hyperthermia**<br>In susceptible individuals, volatile anesthetic agents, including desflurane, may trigger malignant hyperthermia, a skeletal muscle hypermetabolic state leading to high oxygen demand. Fatal outcomes of malignant hyperthermia have been reported. The risk of developing malignant hyperthermia increases with the concomitant administration of succinylcholine and volatile anesthetic agents. SUPRANE can induce malignant hyperthermia in patients with known or suspected susceptibility based on genetic factors or family history, including those with certain inherited ryanodine receptor (RYR1) or dihydropyridine receptor (CACNA1S) variants. [see Contraindications (4), Clinical Pharmacology (12.5)] (…)<br><br>**12. CLINICAL PHARMACOLOGY**<br>**12.5 Pharmacogenomics**<br>RYR1 and CACNA1S are polymorphic genes and multiple pathogenic variants have been associated with malignant hyperthermia susceptibility (MHS) in patients receiving volatile anesthetic agents, including SUPRANE. Case reports as well as exvivo studies have identified multiple variants in RYR1 and CACNA1S associated with MHS. Variant pathogenicity should be assessed based on prior clinical experience, functional studies, prevalence information, or other evidence [see Contraindications (4), Warnings and Precautions (5.1)].
**021992,<br>02/06/2018**	**Desvenlafaxine**	Psychiatry	CYP2D6	Clinical Pharmacology	**12 CLINICAL PHARMACOLOGY**<br>**12.3 Pharmacokinetics**<br>*Metabolism and elimination*<br>Desvenlafaxine is primarily metabolized by conjugation (mediated by UGT isoforms) and, to a minor extent, through oxidative metabolism. CYP3A4 is the cytochrome P450 isozyme mediating the oxidative metabolism (N-demethylation) of desvenlafaxine. The CYP2D6 metabolic pathway is not involved, and after administration of 100 mg, the pharmacokinetics of desvenlafaxine was similar in subjects with CYP2D6 poor and extensive metabolizer phenotype. (…)
**208082,<br>05/04/2022**	**Deutetrabenazine**	Neurology	CYP2D6	Dosage and Administration, Use in Specific Populations, Clinical Pharmacology	**2 DOSAGE AND ADMINISTRATION**<br>**2.4 Dosage Adjustment in Poor CYP2D6 Metabolizers**<br>In patients who are poor CYP2D6 metabolizers, the total daily dosage of AUSTEDO should not exceed 36 mg (maximum single dose of 18 mg) [see Use in Specific Populations (8.7)].<br><br>**8 USE IN SPECIFIC POPULATIONS**<br>**8.7 Poor CYP2D6 Metabolizers**

* Therapeutic areas do not necessarily reflect the CDER review division.
† Representative biomarkers are listed based on standard nomenclature as per the Human Genome Organization (HUGO) symbol and/or simplified descriptors using other common conventions. Listed biomarkers do not necessarily reflect the terminology used in labeling. The term "Nonspecific" is provided when labeling does not explicitly identify the specific biomarker(s) or when the biomarker is represented by a molecular phenotype or gene signature, and in some cases the biomarker was inferred based on the labeling language.
‡ Referenced figures and tables may be found in the labeling available at Drugs@FDA.
Blue text represents the most recent addtions and/or changes since last posted version.

NDA/ANDA/BLA Number, Label Version Date	Drug	Therapeutic Area*	Biomarker†	Labeling Sections	Labeling Text‡
					Although the pharmacokinetics of deutetrabenazine and its metabolites have not been systematically evaluated in patients who do not express the drug metabolizing enzyme, it is likely that the exposure to α-HTBZ and β-HTBZ would be increased similarly to taking a strong CYP2D6 inhibitor (approximately 3-fold). In patients who are CYP2D6 poor metabolizers, the daily dose of AUSTEDO should not exceed 36 mg (maximum single dose of 18 mg) [see Dosage and Administration (2.4) and Clinical Pharmacology (12.3)].<br><br>**12 CLINICAL PHARMACOLOGY**<br>**12.2 Pharmacodynamics**<br>**Cardiac Electrophysiology**<br>At the maximum recommended dose, AUSTEDO does not prolong the QT interval to any clinically relevant extent. An exposure-response analysis on QTc prolongation from a study in extensive or intermediate (EM) and poor CYP2D6 metabolizers (PM) showed that a clinicallyrelevant effect can be excluded at exposures following single doses of 24 and 48 mg of AUSTEDO.<br>**12.3 Pharmacokinetics**<br>*Poor CYP2D6 Metabolizers*<br>Although the pharmacokinetics of deutetrabenazine and its metabolites have not been systematically evaluated in patients who do not express the drug metabolizing enzyme CYP2D6, it is likely that the exposure to α-HTBZ and β-HTBZ would be increased similarly to taking strong CYP2D6 inhibitors (approximately 3-fold) [see Dosage and Administration (2.4), Drug Interactions (7.1)].
022287, 06/07/2018	Dexlansoprazole	Gastroenterology	CYP2C19	Drug Interactions, Clinical Pharmacology	**7 DRUG INTERACTIONS**<br>Potentially increased exposure of tacrolimus, especially in transplant patients who are intermediate or poor metabolizers of CYP2C19.<br><br>**12 CLINICAL PHARMACOLOGY**<br>**12.3 Pharmacokinetics**<br>*Metabolism*<br>(…) CYP2C19 is a polymorphic liver enzyme which exhibits three phenotypes in the metabolism of CYP2C19 substrates: extensive metabolizers (*1/*1), intermediate metabolizers (*1/mutant) and poor metabolizers (mutant/mutant). Dexlansoprazole is the major circulating component in plasma regardless of CYP2C19 metabolizer status. In CYP2C19 intermediate and extensive metabolizers, the major plasma metabolites are 5-hydroxy dexlansoprazole and its glucuronide conjugate, while in CYP2C19 poor metabolizers dexlansoprazole sulfone is the major plasma metabolite.<br>*Cytochrome P 450 Interactions*<br>(…) Although in vitro studies indicated that DEXILANT has the potential to inhibit CYP2C19 in vivo, an in vivo drug-drug interaction study in mainly CYP2C19 extensive and intermediate metabolizers has shown that DEXILANT does not affect the pharmacokinetics of diazepam (CYP2C19 substrate). (…)<br>*Clopidogrel*<br>Clopidogrel is metabolized to its active metabolite in part by CYP2C19. A study of healthy subjects who were CYP2C19 extensive metabolizers, receiving once daily administration of clopidogrel 75 mg alone or concomitantly with DEXILANT 60 mg capsules (n=40), for nine days was conducted. The mean AUC of the active metabolite of clopidogrel was reduced by approximately 9% (mean AUC ratio was 91%, with 90% CI of 86-97%) when DEXILANT was coadministered compared to administration of clopidogrel alone. Pharmacodynamic parameters were also measured and demonstrated that the change in inhibition of platelet aggregation (induced by 5 mcM ADP) was related to the change in the exposure to clopidogrel active metabolite. The effect on exposure to the active metabolite of clopidogrel and on clopidogrel-induced platelet inhibition is not considered clinically important.<br>**12.5 Pharmacogenomics**<br>*Effect of CYP2C19 Polymorphism on Systemic Exposure of Dexlansoprazole*<br>Systemic exposure of dexlansoprazole is generally higher in intermediate and poor metabolizers. In male Japanese subjects who received a single dose of DEXILANT 30 mg or 60 mg capsules (N=2 to 6 subjects/group), mean dexlansoprazole Cmax and AUC values were up to two times higher in intermediate compared to extensive metabolizers; in poor metabolizers, mean Cmax was up to four times higher and mean AUC was up to 12 times higher compared to extensive metabolizers. Though such study was not conducted in Caucasians and African Americans, it is expected dexlansoprazole exposure in these races will be affected by CYP2C19 phenotypes as well.
021879, 06/11/2019	Dextromethorphan and Quinidine	Neurology	CYP2D6	Warnings and Precautions, Clinical Pharmacology	**5 WARNINGS AND PRECAUTIONS**<br>**5.4 Concomitant use of CYP2D6 Substrates**<br>The quinidine in NUEDEXTA inhibits CYP2D6 in patients in whom CYP2D6 is not otherwise genetically absent or its activity otherwise pharmacologically inhibited [see Warnings and Precautions (5.8) and Clinical Pharmacology (12.3), (12.5)]. Because of this effect on CYP2D6, accumulation of parent drug and/or failure of active metabolite formation may decrease the safety and/or the efficacy of drugs used concomitantly with NUEDEXTA that are metabolized by CYP2D6 [see Drug Interactions (7.5)].<br>**5.8 CYP2D6 Poor Metabolizers**<br>The quinidine component of NUEDEXTA is intended to inhibit CYP2D6 so that higher exposure to dextromethorphan can be achieved compared to when dextromethorphan is given alone [see Warnings and Precautions (5.4) and Clinical Pharmacology (12.3), (12.5)]. Approximately 7-10% of Caucasians and 3-8% of African Americans lack the capacity to metabolize CYP2D6 substrates and are classified as poor metabolizers (PMs). The quinidine component of NUEDEXTA is not expected to contribute to the effectiveness of NUEDEXTA in PMs, but adverse events of the quinidine are still possible. In those patients who may be at risk of significant toxicity due to quinidine, genotyping to determine if they are PMs should be considered prior to making the decision to treat with NUEDEXTA.<br><br>**12 CLINICAL PHARMACOLOGY**<br>**12.2 Pharmacodynamics**

Table of Pharmacogenomic Biomarkers in Drug Labeling

Last Updated: 06/2024

NDA/ANDA/BLA Number, Label Version Date	Drug	Therapeutic Area*	Biomarker†	Labeling Sections	Labeling Text‡
					Cardiac Electrophysiology<br>The effect of dextromethorphan 30 mg/quinidine 10 mg (for 7 doses) on QTc prolongation was evaluate in a randomized, double-blind (except for moxifloxacin), placebo- and positive-controlled (400 mg moxifloxacin) crossover thorough QT study in 50 fasted normal healthy men and women with CYP2D6 extensive metabolizer (EM) genotype. Mean changes in QTcF were 6.8 ms for dextromethorphan 30 mg/quinidine 10 mg and 9.1 ms for the reference positive control (moxifloxacin). The maximum mean (95% upper confidence bound) difference from placebo after baseline correction was 10.2 (12.6) ms. This test dose is adequate to represent the steady state exposure in patients with CYP2D6 extensive metabolizer phenotype.<br>**12.3 Pharmacokinetics**<br>*Metabolism and Excretion*<br>NUEDEXTA is a combination product containing dextromethorphan and quinidine. Dextromethorphan is metabolized by CYP2D6 and quinidine is metabolized by CYP3A4. After dextromethorphan 30mg/quinidine 30mg administration in extensive metabolizers, the elimination half-life of dextromethorphan was approximately 13 hours and the elimination half-life of quinidine was approximately 7 hours. (…)<br>**12.5 Pharmacogenomics**<br>The quinidine component of NUEDEXTA is intended to inhibit CYP2D6 so that higher exposure to dextromethorphan can be achieved compared to when dextromethorphan is given alone. Approximately 7-10% of Caucasians and 3-8% of African Americans generally lack the capacity to metabolize CYP2D6 substrates and are classified as PMs. The quinidine component of NUEDEXTA is not expected to contribute to the effectiveness of NUEDEXTA in PMs, but adverse events of the quinidine are still possible. In those patients who may be at risk of significant toxicity due to quinidine, genotyping to determine if they are PMs should be considered prior to making the decision to treat with NUEDEXTA [see Warnings and Precautions (5.4),(5.8), and Clinical Pharmacology (12.3)].
**020648, 12/16/2016**	Diazepam	Neurology	CYP2C19	Clinical Pharmacology	**12 CLINICAL PHARMACOLOGY**<br>**12.3 Pharmacokinetics**<br>*Metabolism and Elimination*<br>(…) The marked inter-individual variability in the clearance of diazepam reported in the literature is probably attributable to variability of CYP2C19 (which is known to exhibit genetic polymorphism; about 3-5% of Caucasians have little or no activity and are “poor metabolizers”) and CYP3A4. (…)
**125516, 03/01/2017**	Dinutuximab	Oncology	MYCN	Clinical Studies	**14 CLINICAL STUDIES**<br>(…) Forty-six percent of patients had neuroblastoma that was not MYCN-amplified, 36% had tumors with known MYCN-amplification, and MYCN status was unknown or missing in 19% of patients. (…)
**022234, 10/11/2019**	Docetaxel	Oncology	ESR, PGR (Hormone Receptor)	Clinical Studies	**14 CLINICAL STUDIES**<br>**14.2 Adjuvant Treatment of Breast Cancer**<br>**(…)** Docetaxel was administered as a 1-hour infusion; all other drugs were given as intravenous bolus on day 1. In both arms, after the last cycle of chemotherapy, patients with positive estrogen and/or progesterone receptors received tamoxifen 20 mg daily for up to 5 years. Adjuvant radiation therapy was prescribed according to guidelines in place at participating institutions and was given to 69% of patients who received TAC and 72% of patients who received FAC. (See Table 14) (…)
**204790, 09/06/2018**	Dolutegravir	Infectious Diseases	UGT1A1	Clinical Pharmacology	**12 CLINICAL PHARMACOLOGY**<br>**12.3 Pharmacokinetics**<br>*Metabolism and Elimination*<br>Polymorphisms in Drug-Metabolizing Enzymes: In a meta-analysis of healthy subject trials, subjects with UGT1A1 (n = 7) genotypes conferring poor dolutegravir metabolism had a 32% lower clearance of dolutegravir and 46% higher AUC compared with subjects with genotypes associated with normal metabolism via UGT1A1 (n = 41).
**020690, 12/18/2018**	Donepezil	Neurology	CYP2D6	Clinical Pharmacology	**12 CLINICAL PHARMACOLOGY**<br>**12.3 Pharmacokinetics**<br>Donepezil is both excreted in the urine intact and extensively metabolized to four major metabolites, two of which are known to be active, and a number of minor metabolites, not all of which have been identified. Donepezil is metabolized by CYP 450 isoenzymes 2D6 and 3A4 and undergoes glucuronidation. Following administration of ^{14}C-labeled donepezil, plasma radioactivity, expressed as a percent of the administered dose, was present primarily as intact donepezil (53%) and as 6-O-desmethyl donepezil (11%), which has been reported to inhibit AChE to the same extent as donepezil in vitro and was found in plasma at concentrations equal to about 20% of donepezil. Approximately 57% and 15% of the total radioactivity was recovered in urine and feces, respectively, over a period of 10 days, while 28% remained unrecovered, with about 17% of the donepezil dose recovered in the urine as unchanged drug. Examination of the effect of CYP2D6 genotype in Alzheimer’s patients showed differences in clearance values among CYP2D6 genotype subgroups. When compared to the extensive metabolizers, poor metabolizers had a 31.5% slower clearance and ultra-rapid metabolizers had a 24% faster clearance.
**761174, 07/31/2023**	Dostarlimab-gxly	Oncology	Microsatellite Instability, Mismatch Repair	Indication and Usage, Dosage and Administration, Adverse Reactions, Clinical Studies	**1 INDICATIONS AND USAGE**<br>**1.1 Endometrial Cancer**<br>JEMPERLI, in combination with carboplatin and paclitaxel, followed by JEMPERLI as a single agent, is indicated for the treatment of adult patients with primary advanced or recurrent endometrial cancer (EC) that is mismatch repair deficient (dMMR), as determined by an FDAapproved test, or microsatellite instability-high (MSI-H) [see Dosage and Administration (2.1)].<br>JEMPERLI, as a single agent, is indicated for the treatment of adult patients with dMMR recurrent or advanced endometrial cancer, as determined by an FDA-approved test, that has progressed on or following prior treatment with a platinum-containing regimen in any setting and are not candidates for curative surgery or radiation [see Dosage and Administration (2.1)]. **1.2 Mismatch Repair Deficient Recurrent or Advanced Solid Tumors**<br>JEMPERLI, as a single agent, is indicated for the treatment of adult patients with dMMR recurrent or advanced solid tumors, as determined by an FDA-approved test, that have progressed on or following prior treatment and who have no satisfactory alternative treatment options [see Dosage and Administration (2.1)]. This

* Therapeutic areas do not necessarily reflect the CDER review division.

† Representative biomarkers are listed based on standard nomenclature as per the Human Genome Organization (HUGO) symbol and/or simplified descriptors using other common conventions. Listed biomarkers do not necessarily reflect the terminology used in labeling. The term “Nonspecific” is provided when labeling does not explicitly identify the specific biomarker(s) or when the biomarker is represented by a molecular phenotype or gene signature, and in some cases the biomarker was inferred based on the labeling language.

‡ Referenced figures and tables may be found in the labeling available at Drugs@FDA.

Blue text represents the most recent addtions and/or changes since last posted version.

NDA/ANDA/BLA Number, Label Version Date	Drug	Therapeutic Area*	Biomarker†	Labeling Sections	Labeling Text‡
					indication is approved under accelerated approval based on tumor response rate and durability of response [see Clinical Studies (14.2)]. Continued approval for this indication may be contingent upon verification and description of clinical benefit in a confirmatory trial(s).<br><br>**2 DOSAGE AND ADMINISTRATION**<br>**2.1 Patient Selection**<br>Combination Therapy<br>For use of JEMPERLI in combination with carboplatin and paclitaxel, select patients for treatment with JEMPERLI based on dMMR/MSI-H status in tumor specimens [see Clinical Studies (14.1)].<br>Single Agent<br>Select patients for treatment with JEMPERLI as a single agent based on the presence of dMMR in tumor specimens in:<br>• recurrent or advanced endometrial cancer [see Clinical Studies (14.1)].<br>• recurrent or advanced solid tumors [see Clinical Studies (14.2)]. Information on FDA-approved tests for the detection of dMMR status is available at https://www.fda.gov/companiondiagnostics.<br>• An FDA-approved test for the detection of MSI-H is not currently available [see Clinical Studies (14.1)].<br>Because the effect of prior chemotherapy on test results for dMMR in patients with high-grade gliomas is unclear, it is recommended to test for this marker in the primary tumor specimen obtained prior to initiation of temozolomide chemotherapy in patients with high-grade gliomas.<br>**2.2 Recommended Dosage**<br>The recommended dosage for JEMPERLI is presented in Table 1.<br><br>**6 ADVERSE REACTIONS**<br>Mismatch Repair Deficient (dMMR) or Microsatellite Instability-High (MSI-H) Primary Advanced or Recurrent EC: JEMPERLI In Combination with Carboplatin and Paclitaxel<br>The safety of JEMPERLI in patients with primary advanced or recurrent dMMR/MSI-H EC was evaluated in RUBY [see Clinical Studies (14.1)]. Patients received JEMPERLI 500 mg (n = 52) or placebo (n = 65) in combination with carboplatin and paclitaxel every 3 weeks for 6 doses followed by JEMPERLI 1,000 mg or placebo every 6 weeks until disease progression or unacceptable toxicity. Among the 52 patients, 56% were exposed for >1 year and 31% were exposed for >2 years.<br>Table 3 summarizes the adverse reactions that occurred in ≥10% of patients with primary advanced or recurrent dMMR/MSI-H EC receiving JEMPERLI in combination with carboplatin and paclitaxel in RUBY.<br>Clinically relevant adverse reactions in <10% of patients with primary advanced or recurrent dMMR/MSI-H EC who received JEMPERLI in combination with carboplatin and paclitaxel included:<br>Endocrine Disorders: Hyperthyroidism, thyroiditis.<br>Eye Disorders: Keratitis.<br>Gastrointestinal Disorders: Colitis, pancreatitis.<br>Metabolism and Nutrition Disorders: Type 1 diabetes mellitus.<br>Nervous System Disorders: Encephalopathy.<br>Table 4 summarizes the laboratory abnormalities in patients with primary advanced or recurrent dMMR/MSI-H EC receiving JEMPERLI in combination with carboplatin and paclitaxel in RUBY.<br>dMMR Recurrent or Advanced EC: JEMPERLI as a Single Agent<br>The safety of JEMPERLI was evaluated in GARNET in 150 patients with advanced or recurrent dMMR EC who received at least 1 dose of JEMPERLI [see Clinical Studies (14.1)]. (See Tables 5 and 6) (…)<br>dMMR Recurrent or Advanced Solid Tumors<br>The safety of JEMPERLI was investigated in 267 patients with recurrent or advanced dMMR solid tumors enrolled in GARNET [see Clinical Studies (14.2)]. (See Tables 7 and 8) (…)<br><br>**14 CLINICAL STUDIES**<br>**14.1 Endometrial Cancer**<br>In Combination with Carboplatin and Paclitaxel for the Treatment of dMMR or MSI-H Primary Advanced or Recurrent Endometrial Cancer<br>The efficacy of JEMPERLI in combination with carboplatin and paclitaxel, followed by JEMPERLI as a single agent, was evaluated in RUBY (NCT03981796), a randomized, multicenter, double-blind, placebo-controlled trial. Efficacy was assessed in a pre-specified subgroup of 122 patients with dMMR/MSI-H primary advanced or recurrent endometrial cancer.<br>The identification of dMMR/MSI-H tumor status was prospectively determined based on local testing assays (IHC, PCR or NGS), or central testing (IHC) using the VENTANA MMR RxDx Panel when no local result was available.<br>Randomization was stratified by MMR/MSI status, prior external pelvic radiotherapy, and disease status (recurrent, primary Stage III, or primary Stage IV). Treatment with JEMPERLI continued until disease progression, unacceptable toxicity, or a maximum of 3 years. Administration of JEMPERLI was permitted beyond RECIST-defined disease progression if the patient was clinically stable and considered to be deriving clinical benefit by the investigator.<br>Assessment of tumor status was performed every 6 weeks through Week 25, every 9 weeks through Week 52 and every 12 weeks thereafter. In the dMMR/MSI-H subgroup, the major efficacy outcome was investigator-assessed progression-free survival (PFS) using Response Evaluation Criteria in Solid Tumors (RECIST v 1.1). (See Table 9 and Figure 1)<br>As a Single Agent for the Treatment of dMMR Recurrent or Advanced Endometrial Cancer

* Therapeutic areas do not necessarily reflect the CDER review division.
† Representative biomarkers are listed based on standard nomenclature as per the Human Genome Organization (HUGO) symbol and/or simplified descriptors using other common conventions. Listed biomarkers do not necessarily reflect the terminology used in labeling. The term “Nonspecific” is provided when labeling does not explicitly identify the specific biomarker(s) or when the biomarker is represented by a molecular phenotype or gene signature, and in some cases the biomarker was inferred based on the labeling language.
‡ Referenced figures and tables may be found in the labeling available at Drugs@FDA.
Blue text represents the most recent addtions and/or changes since last posted version.

Table of Pharmacogenomic Biomarkers in Drug Labeling

Last Updated: 06/2024

NDA/ANDA/BLA Number, Label Version Date	Drug	Therapeutic Area*	Biomarker†	Labeling Sections	Labeling Text‡
					The efficacy of JEMPERLI as a single agent was evaluated in the GARNET trial (NCT02715284), a multicenter, multicohort, open-label trial conducted in patients with advanced solid tumors. The efficacy population consisted of a cohort of 141 patients with dMMR recurrent or advanced EC who had progressed on or after treatment with a platinum-containing regimen.<br>All patients with dMMR EC had received prior anticancer treatment, with 89% of patients receiving prior anticancer surgery and 71% receiving prior anticancer radiotherapy. Sixty-three percent of patients had one prior line of anticancer treatment and 37% had two or more prior lines. Forty-eight patients (34%) received treatment only in the neoadjuvant or adjuvant setting before participating in the study.<br>The dMMR tumor status was retrospectively confirmed using the VENTANA MMR RxDx Panel assay.<br>Efficacy results are presented in Table 10.<br>14.2 Mismatch Repair Deficient Recurrent or Advanced Solid Tumors<br>The efficacy of JEMPERLI as a single agent was evaluated in GARNET (NCT02715284), a non-randomized, multicenter, open-label, multicohort trial. The efficacy population consisted of a cohort of 209 patients with dMMR recurrent or advanced solid tumors who progressed following systemic therapy and had no satisfactory alternative treatment options. Patients with dMMR endometrial cancer must have progressed on or after treatment with a platinumcontaining regimen. Patients with dMMR colorectal cancer must have progressed after or been intolerant to a fluoropyrimidine, oxaliplatin, and irinotecan.<br>At time of trial entry, 97.2% of patients (103/106) with non-endometrial dMMR solid tumors had Stage IV disease, and 68.0% (70/103) of patients with dMMR endometrial tumors had FIGO Stage IV disease.<br>Approximately 43% of patients had received 1 prior line of systemic anticancer treatment, 36% had received 2 prior lines, and 21% had received 3 or more prior lines.<br>The dMMR tumor status was retrospectively confirmed using the VENTANA MMR RxDx Panel assay.<br>Efficacy results are presented in Tables 11 and 12.
**022036, 03/17/2010**	**Doxepin (1)**	Psychiatry	CYP2D6	Clinical Pharmacology	**12 CLINICAL PHARMACOLOGY**<br>**12.5. Special Population**<br>*Poor Metabolizers of CYPs*<br>Poor metabolizers of CYP2C19 and CYP2D6 may have higher doxepin plasma levels than normal subjects.
**022036, 03/17/2010**	**Doxepin (2)**	Psychiatry	CYP2C19	Clinical Pharmacology	**12 CLINICAL PHARMACOLOGY**<br>**12.5. Special Population**<br>*Poor Metabolizers of CYPs*<br>Poor metabolizers of CYP2C19 and CYP2D6 may have higher doxepin plasma levels than normal subjects.
**205525, 09/14/2018**	**Dronabinol**	Gastroenterology	CYP2C9	Use in Specific Populations, Clinical Pharmacology	**8 USE IN SPECIFIC POPULATIONS**<br>**8.6 Effect of CYP2C9 Polymorphism**<br>Published data suggest that systemic clearance of dronabinol may be reduced and concentrations may be increased in presence of CYP2C9 genetic polymorphism. Monitoring for increased adverse reactions is recommended in patients known to carry genetic variants associated with diminished CYP2C9 function [see Clinical Pharmacology (12.5)].<br><br>**12 CLINICAL PHARMACOLOGY**<br>**12.5 Pharmacogenomics**<br>Published data indicate a 2- to 3-fold higher dronabinol exposure in individuals carrying genetic variants associated with diminished CYP2C9 function.
**021676, 08/09/2017**	**Drospirenone and Ethinyl Estradiol**	Gynecology	CYP2C19	Clinical Pharmacology	**12 CLINICAL PHARMACOLOGY**<br>**12.3 Pharmacokinetics**<br>*Effects of Combined Oral Contraceptives on Other Drugs*<br>(…) In the study with 24 postmenopausal women [including 12 women with homozygous (wild type) CYP2C19 genotype and 12 women with heterozygous CYP2C19 genotype] the daily oral administration of 3 mg DRSP for 14 days did not affect the oral clearance of omeprazole (40 mg, single oral dose) and the CYP2C19 product 5-hydroxy omeprazole. (…)
**021427, 12/19/2017**	**Duloxetine**	Psychiatry	CYP2D6	Drug Interactions	**7 DRUG INTERACTIONS**<br>**7.3 Dual Inhibition of CYP1A2 and CYP2D6**<br>Concomitant administration of duloxetine 40 mg twice daily with fluvoxamine 100 mg, a potent CYP1A2 inhibitor, to CYP2D6 poor metabolizer subjects (n=14) resulted in a 6-fold increase in duloxetine AUC and Cmax.
**761069, 06/14/2024**	**Durvalumab (1)**	Oncology	ALK	Indications and Usage, Clinical Studies	**1 INDICATIONS AND USAGE**<br>**1.1 Non-Small Cell Lung Cancer**<br>• IMFINZI is indicated for the treatment of adult patients with unresectable Stage III non-small cell lung cancer (NSCLC) whose disease has not progressed following concurrent platinum-based chemotherapy and radiation therapy.<br>• IMFINZI, in combination with tremelimumab-actl and platinum-based chemotherapy, is indicated for the treatment of adult patients with metastatic NSCLC with no sensitizing epidermal growth factor receptor (EGFR) mutations or anaplastic lymphoma kinase (ALK) genomic tumor aberrations.<br><br>**14 CLINICAL STUDIES**<br>**14.1 Non-Small Cell Lung Cancer (NSCLC)**<br>*Metastatic NSCLC - POSEIDON*<br>The efficacy of IMFINZI in combination with tremelimumab-actl and platinum-based chemotherapy in previously untreated metastatic NSCLC patients with no sensitizing epidermal growth factor receptor (EGFR) mutation or anaplastic lymphoma kinase (ALK) genomic tumor aberrations was investigated in POSEIDON, a randomized, multicenter, active-controlled, open-label trial (NCT03164616). (…)

* Therapeutic areas do not necessarily reflect the CDER review division.

† Representative biomarkers are listed based on standard nomenclature as per the Human Genome Organization (HUGO) symbol and/or simplified descriptors using other common conventions. Listed biomarkers do not necessarily reflect the terminology used in labeling. The term “Nonspecific” is provided when labeling does not explicitly identify the specific biomarker(s) or when the biomarker is represented by a molecular phenotype or gene signature, and in some cases the biomarker was inferred based on the labeling language.

‡ Referenced figures and tables may be found in the labeling available at Drugs@FDA.

Blue text represents the most recent addtions and/or changes since last posted version.

Table of Pharmacogenomic Biomarkers in Drug Labeling
Last Updated: 06/2024

NDA/ANDA/BLA Number, Label Version Date	Drug	Therapeutic Area*	Biomarker†	Labeling Sections	Labeling Text‡
**761069, 06/14/2024**	**Durvalumab (2)**	Oncology	EGFR	Indications and Usage, Clinical Studies	**1 INDICATIONS AND USAGE**<br>**1.1 Non-Small Cell Lung Cancer**<br>• IMFINZI is indicated for the treatment of adult patients with unresectable Stage III non-small cell lung cancer (NSCLC) whose disease has not progressed following concurrent platinum-based chemotherapy and radiation therapy.<br>• IMFINZI, in combination with tremelimumab-actl and platinum-based chemotherapy, is indicated for the treatment of adult patients with metastatic NSCLC with no sensitizing epidermal growth factor receptor (EGFR) mutations or anaplastic lymphoma kinase (ALK) genomic tumor aberrations.<br><br>**14 CLINICAL STUDIES**<br>**14.1 Non-Small Cell Lung Cancer (NSCLC)**<br>*Metastatic NSCLC - POSEIDON*<br>The efficacy of IMFINZI in combination with tremelimumab-actl and platinum-based chemotherapy in previously untreated metastatic NSCLC patients with no sensitizing epidermal growth factor receptor (EGFR) mutation or anaplastic lymphoma kinase (ALK) genomic tumor aberrations was investigated in POSEIDON, a randomized, multicenter, active-controlled, open-label trial (NCT03164616). (…)
**761069, 06/14/2024**	**Durvalumab (3)**	Oncology	CD274 (PD-L1)	Clinical Pharmacology, Clinical Studies	**12 CLINICAL PHARMACOLOGY**<br>**12.3 Pharmacokinetics**<br>*Specific Populations*<br>There were no clinically significant differences in the pharmacokinetics of durvalumab based on body weight (31 to 175 kg), age (18 to 96 years), sex, race (White, Black, Asian, Native Hawaiian, Pacific Islander, or Native American), albumin levels (4 to 57 g/L), lactate dehydrogenase levels (18 to 15,800 U/L), soluble PD-L1 (67 to 3,470 pg/mL), tumor type (NSCLC, SCLC, BTC and HCC), mild or moderate renal impairment (CLcr 30 to 89 mL/min), and mild or moderate hepatic impairment (bilirubin ≤ 3x ULN and any AST). The effect of severe renal impairment (CLcr 15 to 29 mL/min) or severe hepatic impairment (bilirubin > 3x ULN and any AST) on the pharmacokinetics of durvalumab is unknown. (…)<br><br>**14 CLINICAL STUDIES**<br>**14.1 Non-Small Cell Lung Cancer (NSCLC)**<br>*Metastatic NSCLC - POSEIDON*<br>Randomization was stratified by tumor cells (TC) PD-L1 expression (TC ≥ 50% vs. TC < 50%), disease stage (Stage IVA vs. Stage IVB), and histology (non-squamous vs. squamous). (…)<br>A total of 675 patients were randomized to receive either IMFINZI with tremelimumab-actl and platinumbased-chemotherapy (n=338) or platinum-based chemotherapy (n=337). The median age was 63 years (range: 27 to 87), 46% of patients age ≥ 65 years, 77% male, 57% White, 34% Asian, 0.3% Native Hawaiian or Other Pacific Islander, 3% American Indian or Alaska Native, 2% Black or African American, 4% Other Race, 79% former or current smoker, 34% ECOG PS 0, and 66% ECOG PS 1. Thirty-six percent had squamous histology, 63% non-squamous histology, 29% PD-L1 expression TC ≥ 50%, 71% PD-L1 expression TC < 50%.<br>Efficacy results are summarized in Table 16 and Figure 2.
**761069, 06/14/2024**	**Durvalumab (4)**	Oncology	Mismatch Repair	Indications and Usage, Dosage and Administration, Adverse Reactions, Clinical Studies	**1 INDICATIONS AND USAGE**<br>**1.5 Endometrial Cancer**<br>IMFINZI in combination with carboplatin and paclitaxel followed by IMFINZI as a single agent is indicated for the treatment of adult patients with primary advanced or recurrent endometrial cancer that is mismatch repair deficient (dMMR).<br><br>**2 DOSAGE AND ADMINISTRATION**<br>**2.1 Patient Selection**<br>Advanced or Recurrent dMMR Endometrial Cancer<br>Select patients for treatment based on the presence of dMMR in tumor specimens [see Clinical Studies (14.5)].<br>An FDA-approved test for the detection of dMMR in tumor specimens from patients with primary advanced or recurrent endometrial cancer for treatment with IMFINZI is not available.<br><br>**6 ADVERSE REACTIONS**<br>The data described in this section reflect exposure to IMFINZI in patients with Stage III NSCLC enrolled in the PACIFIC study, in patients with metastatic NSCLC enrolled in the POSEIDON study, in patients with ES-SCLC enrolled in the CASPIAN study, in patients with BTC enrolled in the TOPAZ 1 study, in patients with uHCC included in the HIMALAYA study and in patients with dMMR endometrial cancer enrolled in the DUO-E study.<br>Endometrial Cancer<br>Advanced or Recurrent dMMR Endometrial Cancer – DUO-E<br>The safety of IMFINZI in combination with carboplatin and paclitaxel followed by IMFINZI as a single agent was evaluated in 44 patients with dMMR advanced or recurrent endometrial cancer in DUO-E, a randomized, double-blind, placebo-controlled trial [See Clinical Studies (14.5)]. Patients received IMFINZI 1,120 mg with carboplatin and paclitaxel every 3 weeks for up to six 21-day cycles followed by IMFINZI 1,500 mg every 4 weeks or carboplatin and paclitaxel every 3 weeks for up to six 21-day cycles alone. Treatment was continued until disease progression or unacceptable toxicity. The median duration of exposure to IMFINZI with carboplatin and paclitaxel was 14.8 months (range: 0.7 to 31.7)<br><br>**14 CLINICAL STUDIES**<br>**14.5 Endometrial cancer**<br>Advanced or Recurrent dMMR Endometrial Cancer - DUO-E

* Therapeutic areas do not necessarily reflect the CDER review division.
† Representative biomarkers are listed based on standard nomenclature as per the Human Genome Organization (HUGO) symbol and/or simplified descriptors using other common conventions. Listed biomarkers do not necessarily reflect the terminology used in labeling. The term "Nonspecific" is provided when labeling does not explicitly identify the specific biomarker(s) or when the biomarker is represented by a molecular phenotype or gene signature, and in some cases the biomarker was inferred based on the labeling language.
‡ Referenced figures and tables may be found in the labeling available at Drugs@FDA.
Blue text represents the most recent addtions and/or changes since last posted version.

NDA/ANDA/BLA Number, Label Version Date	Drug	Therapeutic Area*	Biomarker†	Labeling Sections	Labeling Text‡
					IMFINZI was evaluated in combination with carboplatin and paclitaxel in DUO-E (NCT04269200), a randomized, multicenter, double-blind, placebo-controlled trial in patients with advanced or recurrent endometrial cancer. The trial enrolled patients with newly diagnosed Stage III disease (with measurable disease per RECIST v1.1), or newly diagnosed Stage IV disease. The trial also enrolled patients with recurrent disease with a low potential for cure by radiation therapy or surgery. For patients with recurrent disease, prior chemotherapy was allowed only if it was administered in the adjuvant setting and at least 12 months had elapsed from the date of last dose of chemotherapy to the date of relapse. The trial included patients with epithelial endometrial carcinomas of all histologies, including carcinosarcomas. Patients with endometrial sarcoma were excluded, and patients who had active autoimmune disease or a medical condition that required immunosuppression were ineligible.<br>Randomization was stratified by tumor mismatch repair (MMR) status (proficient or deficient), disease status (recurrent or newly diagnosed), and geographic region (Asia or rest of the world). MMR status was assessed using an immunohistochemistry tumor tissue test.<br>Patients were randomized (1:1:1) to one of the following treatment arms:<br>• IMFINZI 1,120 mg in combination with carboplatin and paclitaxel every 3 weeks for a maximum of 6 cycles. Following completion of chemotherapy treatment, patients received IMFINZI 1,500 mg every 4 weeks as maintenance treatment until disease progression.<br>• Placebo in combination with carboplatin and paclitaxel every 3 weeks for a maximum of 6 cycles. Following completion of chemotherapy treatment, patients received placebo every 4 weeks as maintenance treatment until disease progression.<br>• An additional investigational combination regimen.<br>Treatment was continued until Response Evaluation Criteria in Solid Tumors (RECIST) v1.1-defined progression of disease or unacceptable toxicity. Assessment of tumor status was performed every 9 weeks for the first 18 weeks and every 12 weeks thereafter.<br>The major efficacy outcome measure was progression-free survival (PFS), determined by investigator assessment using RECIST 1.1. Additional efficacy outcome measures included overall response rate (ORR), duration of response (DOR) and overall survival (OS).<br>Among 95 patients with dMMR tumor, the baseline characteristics were median age of 63 years (range: 34 to 85); 47% age 65 or older; 62% White, 31% Asian, 2% Black or African American; 7% Hispanic or Latino, 1% American Indian or Alaska Native, and 4% other or not reported; ECOG PS of 0 (55%) or 1 (45%); 48% newly diagnosed (11% Stage III and 38% Stage IV) and 52% recurrent disease. The histologic subtypes were endometrioid (78%), mixed epithelial (6%), carcinosarcoma (5%), serous (4%), undifferentiated (1%), and other (5%).<br>While a statistically significant improvement in PFS was observed in the overall population for IMFINZI with carboplatin and paclitaxel compared to carboplatin and paclitaxel alone, based on an exploratory analysis by MMR status, the PFS improvement in the overall population was primarily attributed to patients with dMMR tumors.<br>Efficacy results for DUO-E are summarized in Table 22 and Figure 6 for patients with dMMR tumors. OS data in this subpopulation at the time of PFS analysis were immature with 26% of patients who died.
**211155, 09/26/2019**	**Duvelisib**	Oncology	Chromosome 17p	Clinical Studies	**14 CLINICAL STUDIES**<br>**14.1 Efficacy in Relapsed or Refractory CLL/SLL**<br>Study 1<br>(…) In this subset (95 randomized to COPIKTRA, 101 to ofatumumab), the median patient age was 69 years (range: 40 to 90 years), 59% were male, and 88% had an ECOG performance status of 0 or 1. Forty-six percent received 2 prior lines of therapy, and 54% received 3 or more prior lines. At baseline, 52% of patients had at least one tumor ≥ 5 cm, and 22% of patients had a documented 17p deletion. (…)
**125166, 06/27/2019**	**Eculizumab (1)**	Neurology	ACHR	Indications and Usage, Clinical Studies	**1 INDICATIONS AND USAGE**<br>**1.3 Generalized Myasthenia Gravis (gMG)**<br>Soliris is indicated for the treatment of generalized myasthenia gravis (gMG) in adult patients who are anti-acetylcholine receptor (AchR) antibody positive.<br><br>**14 CLINICAL STUDIES**<br>**14.3 Generalized Myasthenia Gravis (gMG)**<br>The efficacy of Soliris for the treatment of gMG was established in gMG Study 1 (NCT01997229), a 26-week randomized, double-blind, parallel-group, placebocontrolled, multi-center trial that enrolled patients who met the following criteria at screening:<br>1. Positive serologic test for anti-AChR antibodies,<br>2. Myasthenia Gravis Foundation of America (MGFA) Clinical Classification Class II to IV,<br>3. MG-Activities of Daily Living (MG-ADL) total score ≥6,<br>4. Failed treatment over 1 year or more with 2 or more immunosuppressive therapies (ISTs) either in combination or as monotherapy, or failed at least 1 IST and required chronic plasmapheresis or plasma exchange (PE) or intravenous immunoglobulin (IVIg). (…)
**125166, 06/27/2019**	**Eculizumab (2)**	Neurology	AQP4	Indications and Usage, Clinical Studies	**1 INDICATIONS AND USAGE**<br>**1.4 Neuromyelitis Optica Spectrum Disorder (NMOSD)**<br>Soliris is indicated for the treatment of neuromyelitis optica spectrum disorder (NMOSD) in adult patients who are anti-aquaporin-4 (AQP4) antibody positive.<br><br>**14 CLINICAL STUDIES**<br>**14.4 Neuromyelitis Optica Spectrum Disorder (NMOSD)**<br>The efficacy of Soliris for the treatment of NMOSD was established in NMOSD Study 1 (NCT01892345), a randomized, double-blind, placebo-controlled trial that enrolled 143 patients with NMOSD who were anti-AQP4 antibody positive and met the following criteria at screening:<br>1. History of at least 2 relapses in last 12 months or 3 relapses in the last 24 months, with at least 1 relapse in the 12 months prior to screening,<br>2. Expanded Disability Status Scale (EDSS) score ≤ 7 (consistent with the presence of at least limited ambulation with aid),<br>3. If on immunosuppressive therapy (IST), on a stable dose regimen,<br>4. The use of concurrent corticosteroids was limited to 20 mg per day or less,

Table of Pharmacogenomic Biomarkers in Drug Labeling
Last Updated: 06/2024

NDA/ANDA/BLA Number, Label Version Date	Drug	Therapeutic Area*	Biomarker†	Labeling Sections	Labeling Text‡
					5. Patients were excluded if they had been treated with rituximab or mitoxantrone within 3 months or with IVIg within 3 weeks prior to screening. (…)
**020972, 10/10/2017**	Efavirenz	Infectious Diseases	CYP2B6	Clinical Pharmacology	**12 CLINICAL PHARMACOLOGY**<br>**12.2 Pharmacodynamics**<br>*Cardiac Electrophysiology*<br>The effect of SUSTIVA on the QTc interval was evaluated in an open-label, positive and placebo controlled, fixed single sequence 3-period, 3-treatment crossover QT study in 58 healthy subjects enriched for CYP2B6 polymorphisms. The mean Cmax of efavirenz in subjects with CYP2B6 *6/*6 genotype following the administration of 600 mg daily dose for 14 days was 2.25-fold the mean Cmax observed in subjects with CYP2B6 *1/*1 genotype. A positive relationship between efavirenz concentration and QTc prolongation was observed. Based on the concentration-QTc relationship, the mean QTc prolongation and its upper bound 90% confidence interval are 8.7 ms and 11.3 ms in subjects with CYP2B6*6/*6 genotype following the administration of 600 mg daily dose for 14 days [see Warnings and Precautions (5.2)].
**761195, 12/17/2021**	Efgartigimod Alfa-fcab	Neurology	ACHR	Indications and Usage, Clinical Pharmacology, Clinical Studies	**1 INDICATIONS AND USAGE**<br>VYVGART is indicated for the treatment of generalized myasthenia gravis (gMG) in adult patients who are anti-acetylcholine receptor (AChR) antibody positive.<br><br>**12 CLINICAL PHARMACOLOGY**<br>**12.2 Pharmacodynamics**<br>In Study 1 [see Clinical Studies (14)], the pharmacological effect of efgartigimod alfa-fcab was assessed by measuring the decrease in serum IgG levels and AChR autoantibody levels. In patients testing positive for AChR antibodies and who were treated with VYVGART, there was a reduction in total IgG levels relative to baseline. Decrease in AChR autoantibody levels followed a similar pattern.<br><br>**14 CLINICAL STUDIES**<br>The efficacy of VYVGART for the treatment of generalized myasthenia gravis (gMG) in adults who are AChR antibody positive was established in a 26-week, multicenter, randomized, double-blind, placebo-controlled trial (Study 1; NCT03669588). (…)<br>The majority of patients (n=65 for VYVGART; n=64 for placebo) were positive for AChR antibodies.<br>The primary efficacy endpoint was the comparison of the percentage of MG-ADL responders during the first treatment cycle between treatment groups in the AChR-Ab positive population. A statistically significant difference favoring VYVGART was observed in the MG-ADL responder rate during the first treatment cycle [67.7% in the VYVGART-treated group vs 29.7% in the placebo-treated group (p <0.0001)]. (…)<br>The secondary endpoint was the comparison of the percentage of QMG responders during the first treatment cycle between both treatment groups in the AChR-Ab positive patients. A statistically significant difference favoring VYVGART was observed in the QMG responder rate during the first treatment cycle [63.1% in the VYVGART-treated group vs 14.1% in the placebo-treated group (p <0.0001)]. (See Table 2, Figures 1 and 2)
**215500, 12/13/2023**	Eflornithine	Oncology	MYCN	Adverse Reactions, Clinical Studies	**6 ADVERSE REACTIONS**<br>Study 3b<br>(…) The median age of patients who received IWILFIN was 4 years (range: 1 to 17); 59% male; 85% White, 7% Black, 1% Asian, 8% Hispanic or Latino; 87% had International Neuroblastoma Staging System Stage 4 disease; 47% had neuroblastoma with known MYCN-amplification. (…)<br><br>**14 CLINICAL STUDIES**<br>Study 3b<br>*Externally Controlled Trial*<br>(…) Patients who met the criteria for the comparison and had complete data for specified clinical covariates were matched (1:3) using propensity scores; the matched efficacy populations for the primary analysis included 90 patients treated with IWILFIN and 270 control patients from ANBL0032. The demographic characteristics of the primary analysis population (N=360) were 59% male; median age at diagnosis 3 years (range: 0.1 to 20.1); 88% White, 6% Black, 4% Asian, 7% Hispanic. The majority of patients had Stage 4 disease (86%) and MYCN amplification was observed in 44% of tumors. End of immunotherapy responses were complete response (CR; 87%), very good partial response (VGPR; 8%), or partial response (PR; 5%). (…)
217639, 01/27/2023	Elacestrant (1)	Oncology	ESR (Hormone Receptor)	Indications and Usage, Dosage and Administration, Adverse Reactions, Clinical Studies	**1 INDICATIONS AND USAGE**<br>ORSERDU is indicated for the treatment of postmenopausal women or adult men with estrogen receptor (ER)-positive, human epidermal growth factor receptor 2 (HER2)-negative, ESR1-mutated advanced or metastatic breast cancer with disease progression following at least one line of endocrine therapy.<br><br>**2 DOSAGE AND ADMINISTRATION**<br>**2.1 Patient Selection**<br>Select patients for treatment of ER-positive, HER2-negative advanced or metastatic breast cancer with ORSERDU based on the presence of ESR1 mutation(s) in plasma specimen using an FDA-approved test [see Indications and Usage (1) and Clinical Studies (14)].<br>Information on FDA-approved tests for detection of ESR1 mutations in breast cancer is available at: http://www.fda.gov/CompanionDiagnostics.<br><br>**6 ADVERSE REACTIONS**<br>**(…)** The safety of ORSERDU was evaluated in 467 patients with ER+/HER2- advanced breast cancer following CDK4/6 inhibitor therapy in EMERALD, a randomized, open-label, multicenter study [see Clinical Studies (14)]. Patients received ORSERDU 345 mg orally once daily (n=237) or standard of care (SOC) consisting of fulvestrant or an aromatase inhibitor (n=230). Among patients who received ORSERDU, 22% were exposed for 6 months or longer and 9% were exposed for greater than one year. (See Tables 3 and 4)<br><br>**14 CLINICAL STUDIES**

* Therapeutic areas do not necessarily reflect the CDER review division.
† Representative biomarkers are listed based on standard nomenclature as per the Human Genome Organization (HUGO) symbol and/or simplified descriptors using other common conventions. Listed biomarkers do not necessarily reflect the terminology used in labeling. The term “Nonspecific” is provided when labeling does not explicitly identify the specific biomarker(s) or when the biomarker is represented by a molecular phenotype or gene signature, and in some cases the biomarker was inferred based on the labeling language.
‡ Referenced figures and tables may be found in the labeling available at Drugs@FDA.
Blue text represents the most recent addtions and/or changes since last posted version.

Table of Pharmacogenomic Biomarkers in Drug Labeling
Last Updated: 06/2024

NDA/ANDA/BLA Number, Label Version Date	Drug	Therapeutic Area*	Biomarker†	Labeling Sections	Labeling Text‡
					The efficacy of ORSERDU was evaluated in EMERALD (NCT03778931), a randomized, open-label, active-controlled, multicenter trial that enrolled 478 postmenopausal women and men with ER+/HER2- advanced or metastatic breast cancer of which 228 patients had ESR1 mutations. Patients were required to have disease progression on one or two prior lines of endocrine therapy, including one line containing a CDK4/6 inhibitor. Eligible patients could have received up to one prior line of chemotherapy in the advanced or metastatic setting.<br>Patients were randomized (1:1) to receive ORSERDU 345 mg orally once daily (n=239), or investigator's choice of endocrine therapy (n=239), which included fulvestrant (n=166), or an aromatase inhibitor (n=73; anastrozole, letrozole or exemestane). Randomization was stratified by ESR1 mutation status (detected vs not detected), prior treatment with fulvestrant (yes vs no), and visceral metastasis (yes vs no). ESR1 mutational status was determined by blood circulating tumor deoxyribonucleic acid (ctDNA) using the Guardant360 CDx assay and was limited to ESR1 missense mutations in the ligand binding domain (between codons 310 to 547). Patients were treated until disease progression or unacceptable toxicity.<br>The major efficacy outcome was progression-free survival (PFS), assessed by a blinded imaging review committee (BIRC). An additional efficacy outcome measure was overall survival (OS).<br>A statistically significant difference in PFS was observed in the intention to treat (ITT) population and in the subgroup of patients with ESR1 mutations. An exploratory analysis of PFS in the 250 (52%) patients without ESR1 mutations showed a HR 0.86 (95% CI: 0.63, 1.19) indicating that the improvement in the ITT population was primarily attributed to the results seen in the ESR1 mutated population.<br>Among the patients with ESR1 mutations (n=228), the median age was 63 years (range: 28-89); 100% were female; 72% were White, 5.7% Asian, 3.5% Black, 0.4% Other, 18.4% unknown/not reported; 8.8% were Hispanic/Latino; and baseline ECOG performance status was 0 (57%) or 1 (43%). Most patients had visceral disease (71%); 62% had received 1 line of endocrine therapy and 39% had received 2 lines of endocrine therapy in the advanced or metastatic setting. All patients had received prior treatment with a CDK4/6 inhibitor, 24% had received prior fulvestrant, and 25% had received prior chemotherapy in the advanced or metastatic setting.<br>Efficacy results are presented in Table 7 and Figure 1 for patients with ESR1 mutations.
217639, 01/27/2023	Elacestrant (2)	Oncology	ERBB2 (HER2)	Indications and Usage, Dosage and Administration, Adverse Reactions, Clinical Studies	**1 INDICATIONS AND USAGE**<br>ORSERDU is indicated for the treatment of postmenopausal women or adult men with estrogen receptor (ER)-positive, human epidermal growth factor receptor 2 (HER2)-negative, ESR1-mutated advanced or metastatic breast cancer with disease progression following at least one line of endocrine therapy.<br><br>**2 DOSAGE AND ADMINISTRATION**<br>**2.1 Patient Selection**<br>Select patients for treatment of ER-positive, HER2-negative advanced or metastatic breast cancer with ORSERDU based on the presence of ESR1 mutation(s) in plasma specimen using an FDA-approved test [see Indications and Usage (1) and Clinical Studies (14)].<br>Information on FDA-approved tests for detection of ESR1 mutations in breast cancer is available at: http://www.fda.gov/CompanionDiagnostics.<br><br>**6 ADVERSE REACTIONS**<br>**(…)** The safety of ORSERDU was evaluated in 467 patients with ER+/HER2- advanced breast cancer following CDK4/6 inhibitor therapy in EMERALD, a randomized, open-label, multicenter study [see Clinical Studies (14)]. Patients received ORSERDU 345 mg orally once daily (n=237) or standard of care (SOC) consisting of fulvestrant or an aromatase inhibitor (n=230). Among patients who received ORSERDU, 22% were exposed for 6 months or longer and 9% were exposed for greater than one year. (See Tables 3 and 4)<br><br>**14 CLINICAL STUDIES**<br>The efficacy of ORSERDU was evaluated in EMERALD (NCT03778931), a randomized, open-label, active-controlled, multicenter trial that enrolled 478 postmenopausal women and men with ER+/HER2- advanced or metastatic breast cancer of which 228 patients had ESR1 mutations. Patients were required to have disease progression on one or two prior lines of endocrine therapy, including one line containing a CDK4/6 inhibitor. Eligible patients could have received up to one prior line of chemotherapy in the advanced or metastatic setting. (…)
**210450, 07/23/2018**	Elagolix	Gynecology	SLCO1B1	Clinical Pharmacology	**12 CLINICAL PHARMACOLOGY**<br>**12.5 Pharmacogenomics**<br>Disposition of elagolix involves the OATP 1B1 transporter protein. Higher plasma concentrations of elagolix have been observed in groups of patients who have two reduced function alleles of the gene that encodes OATP 1B1 (SLCO1B1 521T>C). The frequency of this SLCO1B1 521 C/C genotype is generally less than 5% in most racial/ethnic groups. Subjects with this genotype are expected to have a 78% mean increase in elagolix concentrations compared to subjects with normal transporter function (i.e., SLCO1B1 521T/T genotype).
**208261, 06/28/2018**	Elbasvir and Grazoprevir	Infectious Diseases	IFNL3 (IL28B)	Clinical Studies	**14 CLINICAL STUDIES**<br>**14.2 Clinical Trials in Treatment-Naïve Subjects with Genotype 1 HCV (C-EDGE TN and C-EDGE COINFECTION)**<br>(…) C-EDGE TN was a randomized, double-blind, placebo-controlled trial in treatment-naïve subjects with genotype 1 or 4 infection with or without cirrhosis. Subjects were randomized in a 3:1 ratio to: ZEPATIER for 12 weeks (immediate treatment group) or placebo for 12 weeks followed by open-label treatment with ZEPATIER for 12 weeks (deferred treatment group). Among subjects with genotype 1 infection randomized to the immediate treatment group, the median age was 55 years (range: 20 to 78); 56% of the subjects were male; 61% were White; 20% were Black or African American; 8% were Hispanic or Latino; mean body mass index was 26 kg/m^2 ; 72% had baseline HCV RNA levels greater than 800,000 IU per mL; 24% had cirrhosis; 67% had non-C/C IL28B alleles (CT or TT); and 55% had genotype 1a and 45% had genotype 1b chronic HCV infection.<br>C-EDGE COINFECTION was an open-label, single-arm trial in treatment-naïve HCV/HIV-1 coinfected subjects with genotype 1 or 4 infection with or without cirrhosis. Subjects received ZEPATIER for 12 weeks. Among subjects with genotype 1 infection, the median age was 50 years (range: 21 to 71); 85% of the subjects were male; 75% were White; 19% were Black or African American; 6% were Hispanic or Latino; mean body mass index was 25 kg per m^2 ; 59% had

* Therapeutic areas do not necessarily reflect the CDER review division.
† Representative biomarkers are listed based on standard nomenclature as per the Human Genome Organization (HUGO) symbol and/or simplified descriptors using other common conventions. Listed biomarkers do not necessarily reflect the terminology used in labeling. The term "Nonspecific" is provided when labeling does not explicitly identify the specific biomarker(s) or when the biomarker is represented by a molecular phenotype or gene signature, and in some cases the biomarker was inferred based on the labeling language.
‡ Referenced figures and tables may be found in the labeling available at Drugs@FDA.
Blue text represents the most recent addtions and/or changes since last posted version.

NDA/ANDA/BLA Number, Label Version Date	Drug	Therapeutic Area*	Biomarker†	Labeling Sections	Labeling Text‡
					baseline HCV RNA levels greater than 800,000 IU per mL; 16% had cirrhosis; 65% had non-C/C IL28B alleles (CT or TT); and 76% had genotype 1a, 23% had genotype 1b, and 1% had genotype 1-Other chronic HCV infection. (…)<br>**14.3 Clinical Trials in Treatment-Experienced Subjects with Genotype 1** HCV<br>*Treatment-Experienced Subjects who Failed Prior PegIFN with RBV Therapy (C-EDGE TE)*<br>(…) C-EDGE TE was a randomized, open-label comparative trial in subjects with genotype 1 or 4 infection, with or without cirrhosis, with or without HCV/HIV-1 co-infection, who had failed prior therapy with PegIFN + RBV therapy. Subjects were randomized in a 1:1:1:1 ratio to one of the following treatment groups: ZEPATIER for 12 weeks, ZEPATIER + RBV for 12 weeks, ZEPATIER for 16 weeks, or ZEPATIER + RBV for 16 weeks. Among subjects with genotype 1 infection, the median age was 57 years (range: 19 to 77); 64% of the subjects were male; 67% were White; 18% were Black or African American; 9% were Hispanic or Latino; mean body mass index was 28 kg/m^2 ; 78% had baseline HCV RNA levels greater than 800,000 IU/mL; 34% had cirrhosis; 79% had non-C/C IL28B alleles (CT or TT); and 60% had genotype 1a, 39% had genotype 1b, and 1% had genotype 1-Other chronic HCV infection. (…)<br>*Treatment-Experienced Subjects who Failed Prior PegIFN + RBV + HCV Protease Inhibitor Therapy (CSALVAGE)*<br>C-SALVAGE was an open-label single-arm trial in subjects with genotype 1 infection, with or without cirrhosis, who had failed prior treatment with boceprevir, simeprevir, or telaprevir in combination with PegIFN + RBV. Subjects received EBR 50 mg once daily + GZR 100 mg once daily + RBV for 12 weeks. Subjects had a median age of 55 years (range: 23 to 75); 58% of the subjects were male; 97% were White; 3% were Black or African American; 15% were Hispanic or Latino; mean body mass index was 28 kg/m^2; 63% had baseline HCV RNA levels greater than 800,000 IU/mL; 43% had cirrhosis; and 97% had non-C/C IL28B alleles (CT or TT); 46% had baseline NS3 resistance-associated substitutions.<br>Overall SVR was achieved in 96% (76/79) of subjects receiving EBR + GZR + RBV for 12 weeks. Four percent (3/79) of subjects did not achieve SVR due to relapse. Treatment outcomes were consistent in genotype 1a and genotype 1b subjects, in subjects with different response to previous HCV therapy, and in subjects with or without cirrhosis. Treatment outcomes were generally consistent in subjects with or without NS3 resistance-associated substitutions at baseline, although limited data are available for subjects with specific NS3 resistance-associated substitutions [see Microbiology (12.4)]. (…)<br>**14.4 Clinical Trial in Subjects with Genotype 1 HCV and Severe Renal Impairment including Subjects on Hemodialysis (C-SURFER)**<br>C-SURFER was a randomized, double-blind, placebo-controlled trial in subjects with genotype 1 infection, with or without cirrhosis, with chronic kidney disease (CKD) Stage 4 (eGFR 15-29 mL/min/1.73 m^2) or CKD Stage 5 (eGFR<15 mL/min/1.73m^2), including subjects on hemodialysis, who were treatment-naïve or who had failed prior therapy with IFN or PegIFN ± RBV therapy. Subjects were randomized in a 1:1 ratio to one of the following treatment groups: EBR 50 mg once daily + GZR 100 mg once daily for 12 weeks (immediate treatment group) or placebo for 12 weeks followed by open-label treatment with EBR + GZR for 12 weeks (deferred treatment group). In addition, 11 subjects received open-label EBR + GZR for 12 weeks (intensive pharmacokinetic [PK] group). Subjects randomized to the immediate treatment group and intensive PK group had a median age of 58 years (range: 31 to 76); 75% of the subjects were male; 50% were White; 45% were Black or African American; 11% were Hispanic or Latino; 57% had baseline HCV RNA levels greater than 800,000 IU/mL; 6% had cirrhosis; and 72% had non-C/C IL28B alleles (CT or TT). Treatment outcomes in subjects treated with ZEPATIER for 12 weeks in the pooled immediate treatment group and intensive PK group are presented in Table 15.
**212273, 10/21/2019**	**Elexacaftor, Ivacaftor, and Tezacaftor**	Pulmonary	CFTR	Indications and Usage, Use in Specific Populations, Clinical Pharmacology, Clinical Studies	**1 INDICATIONS AND USAGE**<br>TRIKAFTA is indicated for the treatment of cystic fibrosis (CF) in patients aged 12 years and older who have at least one F508del mutation in the cystic fibrosis transmembrane conductance regulator (CFTR) gene.<br>If the patient's genotype is unknown, an FDA-cleared CF mutation test should be used to confirm the presence of at least one F508del mutation.<br><br>**8 USE IN SPECIFIC POPULATIONS**<br>**8.4 Pediatric Use**<br>The safety and effectiveness of TRIKAFTA for the treatment of CF in pediatric patients 12 years and older who have at least one F508del mutation in the CFTR gene has been established. Use of TRIKAFTA for this indication was supported by evidence from two adequate and well-controlled studies in CF patients 12 years and older (Trial 1 and Trial 2) [see Clinical Studies (14)]. In these trials, a total of 72 adolescents (aged 12 to 17 years) received TRIKAFTA, including:<br>• In Trial 1, 56 adolescents who had an F508del mutation on one allele and a mutation on the second allele that results in either no CFTR protein or a CFTR protein that is not responsive to ivacaftor and tezacaftor/ivacaftor [see Adverse Reactions (6) and Clinical Studies (14)].<br>• In Trial 2, 16 adolescents who were homozygous for the F508del mutation [see Adverse Reactions (6) and Clinical Studies (14)].<br>The safety and effectiveness of TRIKAFTA in patients with CF younger than 12 years of age have not been established.<br><br>**12 CLINICAL PHARMACOLOGY**<br>**12.2 Pharmacodynamics**<br>Sweat Chloride Evaluation In Trial 1 (patients with an F508del mutation on one allele and a mutation on the second allele that results in either no CFTR protein or a CFTR protein that is not responsive ivacaftor and tezacaftor/ivacaftor), a reduction in sweat chloride was observed from baseline at Week 4 and sustained through the 24-week treatment period [see Clinical Studies (14.1)]. In Trial 2 (patients homozygous for the F508del mutation), a reduction in sweat chloride was observed from baseline at Week 4 [see Clinical Studies (14.2)].<br><br>**14 CLINICAL STUDIES**<br><u>Efficacy:</u><br>The efficacy of TRIKAFTA in patients with CF aged 12 years and older was evaluated in two Phase 3, double blind, controlled trials (Trials 1 and 2).<br>Trial 1 was a 24-week, randomized, double-blind, placebo-controlled study in patients who had an F508del mutation on one allele and a mutation on the second allele that results in either no CFTR protein or a CFTR protein that is not responsive to ivacaftor and tezacaftor/ivacaftor. An interim analysis was planned when at least 140 patients completed Week 4 and at least 100 patients completed Week 12.

* Therapeutic areas do not necessarily reflect the CDER review division.
† Representative biomarkers are listed based on standard nomenclature as per the Human Genome Organization (HUGO) symbol and/or simplified descriptors using other common conventions. Listed biomarkers do not necessarily reflect the terminology used in labeling. The term "Nonspecific" is provided when labeling does not explicitly identify the specific biomarker(s) or when the biomarker is represented by a molecular phenotype or gene signature, and in some cases the biomarker was inferred based on the labeling language.
‡ Referenced figures and tables may be found in the labeling available at Drugs@FDA.
Blue text represents the most recent addtions and/or changes since last posted version.

NDA/ANDA/BLA Number, Label Version Date	Drug	Therapeutic Area*	Biomarker†	Labeling Sections	Labeling Text‡
					Trial 2 was a 4-week, randomized, double-blind, active-controlled study in patients who are homozygous for the F508del mutation. Patients received tezacaftor 100 mg qd/ivacaftor 150 mg q12hr during a 4-week open-label run-in period and were then randomized and dosed to receive TRIKAFTA or tezacaftor 100 mg qd/ivacaftor 150 mg q12hr during a 4-week double-blind treatment period.<br>Patients in Trials 1 and 2 had a confirmed diagnosis of CF and at least one F508del mutation. Patients discontinued any previous CFTR modulator therapies, but continued on their other standard-of-care CF therapies (e.g., bronchodilators, inhaled antibiotics, dornase alfa, and hypertonic saline). Patients had a ppFEV1 at screening between 40-90%. Patients with a history of colonization with organisms associated with a more rapid decline in pulmonary status, including but not limited to Burkholderia cenocepacia, Burkholderia dolosa, or Mycobacterium abscessus, or who had an abnormal liver function test at screening (ALT, AST, ALP, or GGT ≥3 x ULN, or total bilirubin ≥2 x ULN), were excluded from the trials. Patients in Trials 1 and 2 were eligible to roll over into a 96-week open-label extension study.
**205494, 08/29/2018**	**Eliglustat**	Inborn Errors of Metabolism	CYP2D6	Indications and Usage, Dosage and Administration, Contraindications, Warnings and Precautions, Drug Interactions, Use in Specific Populations, Clinical Pharmacology, Clinical Studies	**1 INDICATIONS AND USAGE**<br>CERDELGA is indicated for the long-term treatment of adult patients with Gaucher disease type 1 (GD1) who are CYP2D6 extensive metabolizers (EMs), intermediate metabolizers (IMs), or poor metabolizers (PMs) as detected by an FDA-cleared test [see Dosage and Administration (2.1)].<br>Limitations of Use:<br>• Patients who are CYP2D6 ultra-rapid metabolizers (URMs) may not achieve adequate concentrations of CERDELGA to achieve a therapeutic effect [see Clinical Studies (14)].<br>• A specific dosage cannot be recommended for those patients whose CYP2D6 genotype cannot be determined (indeterminate metabolizers) [see Clinical Studies (14)].<br><br>**2 DOSAGE AND ADMINISTRATION**<br>**2.1 Patient Selection**<br>Select patients with Gaucher disease type 1 based on their CYP2D6 metabolizer status. It is recommended patient genotypes be established using an FDA-cleared test for determining CYP2D6 genotype [see Indications and Usage (1)].<br>**2.2 Recommended Adult Dosage**<br>The recommended dosage of CERDELGA in adults is based on the patient's CYP2D6 metabolizer status. (See Table 1)<br>**2.3 Dosage Adjustment in EMs and IMs With or Without Hepatic Impairment and Concomitant Use of CYP2D6 or CYP3A Inhibitors**<br>Reduce dosage frequency of CERDELGA 84 mg to once daily in CYP2D6 EMs and IMs with or without hepatic impairment taking CYP2D6 or CYP3A inhibitors, as shown in Table 2 [see Warnings and Precautions (5.1), Drug Interactions (7.1), Use in Specific Populations (8.7)]. (See Table 2)<br><br>**4 CONTRAINDICATIONS**<br>CERDELGA is contraindicated in the following patients based on CYP2D6 metabolizer status due to the risk of cardiac arrhythmias from prolongation of the PR, QTc, and/or QRS cardiac intervals.<br>EMs<br>• Taking a strong or moderate CYP2D6 inhibitor concomitantly with a strong or moderate CYP3A inhibitor [see Drug Interactions (7.1)]<br>• Moderate or severe hepatic impairment [see Use in Specific Populations (8.7)]<br>• Mild hepatic impairment and taking a strong or moderate CYP2D6 inhibitor [see Use in Specific Populations (8.7)]<br>IMs<br>• Taking a strong or moderate CYP2D6 inhibitor concomitantly with a strong or moderate CYP3A inhibitor [see Drug Interactions (7.1)]<br>• Taking a strong CYP3A inhibitor [see Drug Interactions (7.1)]<br>• Any degree of hepatic impairment [see Use in Specific Populations (8.7)]<br>PMs<br>• Taking a strong CYP3A inhibitor [see Drug Interactions (7.1)]<br>• Any degree of hepatic impairment [see Use in Specific Populations (8.7)]<br><br>**5 WARNINGS AND PRECAUTIONS**<br>**5.1 ECG Changes and Potential for Cardiac Arrhythmias**<br>CERDELGA is predicted to cause increases in ECG intervals (PR, QTc, and QRS) at substantially elevated eliglustat plasma concentrations and may increase the risk of cardiac arrhythmias.<br>• Use of CERDELGA is contraindicated, to be avoided, or requires dosage adjustment in patients taking CYP2D6 or CYP3A inhibitors, depending CYP2D6 metabolizer status, type of inhibitor, or degree of hepatic impairment [see Dosage and Administration (2.3), Contraindications (4), Drug Interactions (7.1)]. (…))].<br><br>**7 DRUG INTERACTIONS**<br>**7.1 Effect of Other Drugs on CERDELGA**<br>Coadministration of CERDELGA with:<br>• CYP2D6 or CYP3A inhibitors may increase eliglustat concentrations which may increase the risk of cardiac arrhythmias from prolongation of the PR, QTc, and/or QRS cardiac interval [see Warnings and Precautions (5.1), Clinical Pharmacology (12.3)].<br>• strong CYP3A inducers decreases eliglustat concentrations which may reduce CERDELGA efficacy [see Clinical Pharmacology (12.3)].<br>See Table 5 for prevention and management of interactions with drugs affecting CERDELGA. Use of CERDELGA is contraindicated, to be avoided, or may require dosage adjustment depending on the concomitant drug and CYP2D6 metabolizer status [see Dosage and Administration (2.2, 2.3), Contraindications (4), Drug Interactions (7.1)]. (See Tale 5)

* Therapeutic areas do not necessarily reflect the CDER review division.
† Representative biomarkers are listed based on standard nomenclature as per the Human Genome Organization (HUGO) symbol and/or simplified descriptors using other common conventions. Listed biomarkers do not necessarily reflect the terminology used in labeling. The term "Nonspecific" is provided when labeling does not explicitly identify the specific biomarker(s) or when the biomarker is represented by a molecular phenotype or gene signature, and in some cases the biomarker was inferred based on the labeling language.
‡ Referenced figures and tables may be found in the labeling available at Drugs@FDA.
Blue text represents the most recent addtions and/or changes since last posted version.

NDA/ANDA/BLA Number, Label Version Date	Drug	Therapeutic Area*	Biomarker†	Labeling Sections	Labeling Text‡
					**8 USE IN SPECIFIC POPULATIONS**<br>**8.6 Renal Impairment**<br>Use CERDELGA in patients with renal impairment based on the patient's CYP2D6 metabolizer status [see Clinical Pharmacology (12.3)].<br>EMs<br>• Avoid CERDELGA in patients with end-stage renal disease (ESRD) (estimated creatinine clearance (eCLcr) less than 15 mL/min not on dialysis or requiring dialysis).<br>• No dosage adjustment is recommended in patients with mild, moderate, or severe renal impairment (eCLcr at least 15 mL/min).<br>IMs and PMs<br>• Avoid CERDELGA in patients with any degree of renal impairment.<br>**8.7 Hepatic Impairment**<br>Use CERDELGA in patients with hepatic impairment based on CYP2D6 metabolizer status and concomitant use of CYP2D6 or CYP3A inhibitors [see Clinical Pharmacology (12.3)].<br>EMs<br>• CERDELGA is contraindicated in patients with [see Contraindications (4)]: o severe (Child-Pugh Class C) hepatic impairment o moderate (Child-Pugh Class B) hepatic impairment o mild (Child-Pugh Class A) hepatic impairment taking a strong or moderate CYP2D6 inhibitor<br>• Reduce dosage frequency of CERDELGA 84 mg to once daily [see Dosage and Administration (2.3)] in patients with mild hepatic impairment taking: o a weak CYP2D6 inhibitor o a strong, moderate, or weak CYP3A inhibitor<br>• No dosage adjustment is recommended in patients with mild hepatic impairment, unless otherwise specified above.<br>IMs and PMs<br>• CERDELGA is contraindicated in patients with any degree of hepatic impairment [see Contraindications (4)].<br><br>**12 CLINICAL PHARMACOLOGY**<br>**12.3 Pharmacokinetics**<br>Absorption<br>The oral bioavailability of eliglustat was less than 5% in CYP2D6 EMs following a single 84 mg dose of CERDELGA.<br>In CYP2D6 EMs, the eliglustat pharmacokinetics is time-dependent and the systemic exposure increases in a more than dose-proportional manner over the dose range of 42 to 294 mg (0.5 to 3.5 times the recommended dosage). In addition, after multiple oral doses of 84 mg twice daily in EMs, eliglustat systemic exposure (AUC0-12) increased up to about 2-fold at steady state compared to after the first dose (AUC0-∞). The pharmacokinetics of eliglustat in CYP2D6 PMs is expected to be linear and time-independent. Compared to EMs, the systemic exposure following 84 mg twice daily at steady state is 7-fold to 9-fold higher in PMs.<br>Dosing of CERDELGA 84 mg once daily has not been studied in PMs. The predicted Cmax and AUC0-24hr in PMs using a physiologically based pharmacokinetic (PBPK) model with 84 mg once daily were 75 ng/mL and 956 hr·ng/mL, respectively.<br>Table 7 describes the pharmacokinetic parameters for eliglustat in healthy subjects following multiple doses of 84 mg CERDELGA twice daily. (See Table 7)<br>Administration of CERDELGA with a high fat meal (approximately 1000 calories with 50% calories from fat) resulted in a 15% decrease in Cmax (not clinically significant) but no change in AUC.<br>Distribution<br>Following intravenous administration, the volume of distribution of eliglustat was 835 L in EMs. Plasma protein binding of eliglustat ranges from 76% to 83%.<br>Elimination<br>Eliglustat terminal elimination half-life was approximately 6.5 hours in CYP2D6 EMs, and 8.9 hours in PMs. Following intravenous administration of 42 mg (0.5 times the recommended oral dose) in healthy subjects, the mean (range) of eliglustat total body clearance was 88 L/h (80 to 105 L/h) in EMs.<br>Specific Populations<br>No clinically significant differences in the pharmacokinetics of eliglustat were observed based on age (18 to 71 years), sex, race (mostly were Caucasian, including those of Ashkenazi Jewish descent; however, it included the following populations: African American, American Indians, Hispanics, and Asians), or body weight (41 to 136 kg).<br>Patients with renal impairment<br>Eliglustat pharmacokinetics was similar in CYP2D6 EMs with severe renal impairment and healthy CYP2D6 EMs. Eliglustat pharmacokinetics in EMs with ESRD and in IMs or PMs with any degree of renal impairment is unknown [see Use in Specific Populations (8.6)].<br>Patients with hepatic impairment<br>Table 8 describes the effect of mild and moderate hepatic impairment on the pharmacokinetics of eliglustat in CYP2D6 EMs compared to EMs with normal hepatic function following a single 84 mg dose. The effect of hepatic impairment is highly variable with the coefficients of variation (CVs%) of 135% and 110% for Cmax and 171% and 121% for AUC in CYP2D6 EMs with mild and moderate hepatic impairment, respectively. (See Table 8)<br>Steady-state pharmacokinetics of eliglustat in CYP2D6 IMs and PMs with mild and moderate hepatic impairment is unknown. The effect of severe hepatic impairment in subjects with any CYP2D6 phenotype is unknown [see Use in Specific Populations (8.7)].<br>Drug Interaction Studies<br>Effect of other drugs on CERDELGA<br>Table 9 describes the effect of drug interactions on the pharmacokinetics of eliglustat [see Drug Interactions (7.1)]. (See Table 9)<br>No clinically significant pharmacokinetic changes were observed for eliglustat when coadministered with intravenous rifampin (an OATP inhibitor), or gastric pH modifying drugs (e.g., aluminum hydroxide, magnesium hydroxide, calcium carbonate, pantoprazole).<br>In vitro, eliglustat is a substrate of P-glycoprotein (P-gp). The effect of P-gp inhibitors on eliglustat pharmacokinetics is unknown.<br>Effect of CERDELGA on other drugs

NDA/ANDA/BLA Number, Label Version Date	Drug	Therapeutic Area*	Biomarker†	Labeling Sections	Labeling Text‡
					<u>CYP2D6 substrates</u><br>Following multiple doses of CERDELGA 127 mg twice daily (1.5 times the recommended dosage), metoprolol (a CYP2D6 substrate) mean Cmax and AUC increased by 1.7-fold and 2.3- fold in CYP2D6 EMs, respectively, and by 1.2-fold and 1.6-fold in IMs, respectively [see Drug Interactions (7.2)].<br><u>P-gp substrates</u><br>Following multiple doses of CERDELGA 127 mg twice daily (1.5 times the recommended dosage) in CYP2D6 EMs and IMs, or 84 mg twice daily in PMs, digoxin (a P-gp substrate) mean Cmax increased by 1.7-fold and AUC increased by 1.5-fold [see Drug Interactions (7.2)].<br><br>**14 CLINICAL STUDIES**<br>(…) The CERDELGA treatment group was comprised of IM (5%), EM (90%) and URM (5%) patients. (…)
**125460, 02/14/2014**	**Elosulfase**	Inborn Errors of Metabolism	GALNS	Indications and Usage, Warnings and Precautions, Use in Specific Populations, Clinical Pharmacology, Clinical Studies	**1 INDICATIONS AND USAGE**<br>Vimizim (elosulfase alfa) is indicated for patients with Mucopolysaccharidosis type IVA (MPS IVA; Morquio A syndrome).<br><br>**5 WARNINGS AND PRECAUTIONS**<br>**5.2 Risk of Acute Respiratory Complications**<br>Patients with acute febrile or respiratory illness at the time of Vimizim infusion may be at higher risk of life-threatening complications from hypersensitivity reactions. Careful consideration should be given to the patient's clinical status prior to administration of Vimizim and consider delaying the Vimizim infusion. Sleep apnea is common in MPS IVA patients. Evaluation of airway patency should be considered prior to initiation of treatment with Vimizim. Patients using supplemental oxygen or continuous positive airway pressure (CPAP) during sleep should have these treatments readily available during infusion in the event of an acute reaction, or extreme drowsiness/sleep induced by antihistamine use.<br>**5.3 Spinal or Cervical Cord Compression**<br>Spinal or cervical cord compression (SCC) is a known and serious complication of MPS IVA and may occur as part of the natural history of the disease. In clinical trials, SCC was observed both in patients receiving Vimizim and patients receiving placebo. Patients with MPS IVA should be monitored for signs and symptoms of SCC (including back pain, paralysis of limbs below the level of compression, urinary and fecal incontinence) and given appropriate clinical care.<br><br>**8 USE IN SPECIFIC POPULATIONS**<br>*Clinical Considerations*<br>*Disease-associated maternal and embryo/fetal risk*<br>Pregnancy can adversely affect the health of females affected with MPS IVA and lead to adverse pregnancy outcomes for both mother and fetus.<br>**8.3 Nursing Mothers**<br>It is not known if Vimizim is present in human milk. Elosulfase alfa is present in milk from treated rats [see Use in Specific Populations (8.1)]. The developmental and health benefits of breastfeeding should be considered along with the mother's clinical need for Vimizim and any potential adverse effects on the breastfed child from the drug or from MPS IVA. Exercise caution when administering Vimizim to a nursing mother. There is a Morquio A Registry that also collects data on breastfeeding women with MPS IVA who are treated with Vimizim.<br><br>**12 CLINICAL PHARMACOLOGY**<br>**12.3 Pharmacokinetics**<br>The pharmacokinetics of elosulfase alfa were evaluated in 23 patients with MPS IVA who received intravenous infusions of Vimizim 2 mg/kg once weekly, over approximately 4 hours, for 22 weeks. (…)<br><br>**14 CLINICAL STUDIES**<br>The safety and efficacy of Vimizim were assessed in a 24-week, randomized, double-blind, placebo-controlled clinical trial of 176 patients with MPS IVA. (…)
**761345, 08/14/2023**	**Elranatamab-bcmm (1)**	Oncology	Chromosome 17p	Clinical Studies	**14 CLINICAL STUDIES**<br>**14.1 Relapsed or Refractory Multiple Myeloma**<br>The 123 patients enrolled in pivotal Cohort A had received a median of 5 prior lines of therapy (range: 2 to 22). Ninety-seven patients who were not exposed to prior BCMA-directed therapy and received at least four prior lines of therapy comprised the efficacy population. Among the 97 patients in the efficacy population, the median age was 69 (range: 46 to 89) years with 18.6% of patients ≥75 years of age. Forty percent were female; 59.8% were White, 13.4% were Asian, 7.2% were Hispanic/Latino, 5.2% were Black or African American. Disease stage (R-ISS) at study entry was 20.6% in Stage I, 53.6% in Stage II, and 17.5% in Stage III. The median time since initial diagnosis of multiple myeloma to enrollment was 79.6 (range: 16 to 228) months. 96.9% were triple-class refractory, and 94.8% were refractory to their last line of therapy. 69.1% received prior autologous stem cell transplantation, and 7.2% received prior allogenic stem cell transplantation. High-risk cytogenetics [t(4;14), t(14;16), or del(17p)] were present in 22.7% of patients. 34.0% of patients had extramedullary disease at baseline by BICR. (…)
**761345, 08/14/2023**	**Elranatamab-bcmm (2)**	Oncology	Chromosome 4p;14q	Clinical Studies	**14 CLINICAL STUDIES**<br>**14.1 Relapsed or Refractory Multiple Myeloma**<br>The 123 patients enrolled in pivotal Cohort A had received a median of 5 prior lines of therapy (range: 2 to 22). Ninety-seven patients who were not exposed to prior BCMA-directed therapy and received at least four prior lines of therapy comprised the efficacy population. Among the 97 patients in the efficacy population, the median age was 69 (range: 46 to 89) years with 18.6% of patients ≥75 years of age. Forty percent were female; 59.8% were White, 13.4% were Asian, 7.2% were Hispanic/Latino, 5.2% were Black or African American. Disease stage (R-ISS) at study entry was 20.6% in Stage I, 53.6% in Stage II, and 17.5% in Stage III. The median time since initial diagnosis of multiple myeloma to enrollment was 79.6 (range: 16 to 228) months. 96.9% were triple-class refractory, and 94.8% were refractory to their last line of therapy. 69.1% received prior autologous stem cell transplantation, and 7.2% received prior allogenic stem cell transplantation. High-risk cytogenetics [t(4;14), t(14;16), or del(17p)] were present in 22.7% of patients. 34.0% of patients had extramedullary disease at baseline by BICR. (…)

Table of Pharmacogenomic Biomarkers in Drug Labeling

Last Updated: 06/2024

NDA/ANDA/BLA Number, Label Version Date	Drug	Therapeutic Area*	Biomarker†	Labeling Sections	Labeling Text‡
761345, 08/14/2023	Elranatamab-bcmm (3)	Oncology	Chromosome 14q;16q	Clinical Studies	**14 CLINICAL STUDIES**<br>**14.1 Relapsed or Refractory Multiple Myeloma**<br>The 123 patients enrolled in pivotal Cohort A had received a median of 5 prior lines of therapy (range: 2 to 22). Ninety-seven patients who were not exposed to prior BCMA-directed therapy and received at least four prior lines of therapy comprised the efficacy population. Among the 97 patients in the efficacy population, the median age was 69 (range: 46 to 89) years with 18.6% of patients ≥75 years of age. Forty percent were female; 59.8% were White, 13.4% were Asian, 7.2% were Hispanic/Latino, 5.2% were Black or African American. Disease stage (R-ISS) at study entry was 20.6% in Stage I, 53.6% in Stage II, and 17.5% in Stage III. The median time since initial diagnosis of multiple myeloma to enrollment was 79.6 (range: 16 to 228) months. 96.9% were triple-class refractory, and 94.8% were refractory to their last line of therapy. 69.1% received prior autologous stem cell transplantation, and 7.2% received prior allogenic stem cell transplantation. High-risk cytogenetics [t(4;14), t(14;16), or del(17p)] were present in 22.7% of patients. 34.0% of patients had extramedullary disease at baseline by BICR. (…)
022291, 04/29/2020	Eltrombopag (1)	Hematology	F5<br>(Factor V Leiden)	Warnings and Precautions	**5 WARNINGS AND PRECAUTIONS**<br>**5.3 Thrombotic/Thromboembolic Complications**<br>Thrombotic/thromboembolic complications may result from increases in platelet counts with PROMACTA. Reported thrombotic/thromboembolic complications included both venous and arterial events and were observed at low and at normal platelet counts.<br>Consider the potential for an increased risk of thromboembolism when administering PROMACTA to patients with known risk factors for thromboembolism (e.g., Factor V Leiden, ATIII deficiency, antiphospholipid syndrome, chronic liver disease). (…)
022291, 04/29/2020	Eltrombopag (2)	Hematology	SERPINC1<br>(Antithrombin III)	Warnings and Precautions	**5 WARNINGS AND PRECAUTIONS**<br>**5.3 Thrombotic/Thromboembolic Complications**<br>Thrombotic/thromboembolic complications may result from increases in platelet counts with PROMACTA. Reported thrombotic/thromboembolic complications included both venous and arterial events and were observed at low and at normal platelet counts.<br>Consider the potential for an increased risk of thromboembolism when administering PROMACTA to patients with known risk factors for thromboembolism (e.g., Factor V Leiden, ATIII deficiency, antiphospholipid syndrome, chronic liver disease). (…)
022291, 04/29/2020	Eltrombopag (3)	Hematology	Chromosome 7	Adverse Reactions	**6 ADVERSE REACTIONS**<br>**6.1 Clinical Trials Experience**<br>*Cytogenetic Abnormalities*<br>In this trial, patients had bone marrow aspirates evaluated for cytogenetic abnormalities. Seven patients in the PROMACTA D1-M6 cohort had a new cytogenetic abnormality reported of which 4 had the loss of chromosome 7; these 4 occurred within 6.1 months. Across all cohorts, clonal cytogenetic evolution occurred in 15 out of 153 (10%) patients. Of the 15 patients who experienced a cytogenetic abnormality: 7 patients had the loss of chromosome 7, 6 of which occurred within 6.1 months; 4 patients had chromosomal aberrations which were of unclear significance; 3 patients had a deletion of chromosome 13; and 1 patient had a follow-up bone marrow assessment at 5 years with features of dysplasia with hypercellularity concerning for potential development of MDS. It is unclear whether these findings occurred due to the underlying disease, the immunosuppressive therapy, and/or treatment with PROMACTA. (…)<br>(…) In this trial, patients had bone marrow aspirates evaluated for cytogenetic abnormalities. Eight patients had a new cytogenetic abnormality reported on therapy, including 5 patients who had complex changes in chromosome 7.
022291, 04/29/2020	Eltrombopag (4)	Hematology	Chromosome 13	Adverse Reactions	**6 ADVERSE REACTIONS**<br>**6.1 Clinical Trials Experience**<br>*Cytogenetic Abnormalities*<br>In this trial, patients had bone marrow aspirates evaluated for cytogenetic abnormalities. Seven patients in the PROMACTA D1-M6 cohort had a new cytogenetic abnormality reported of which 4 had the loss of chromosome 7; these 4 occurred within 6.1 months. Across all cohorts, clonal cytogenetic evolution occurred in 15 out of 153 (10%) patients. Of the 15 patients who experienced a cytogenetic abnormality: 7 patients had the loss of chromosome 7, 6 of which occurred within 6.1 months; 4 patients had chromosomal aberrations which were of unclear significance; 3 patients had a deletion of chromosome 13; and 1 patient had a follow-up bone marrow assessment at 5 years with features of dysplasia with hypercellularity concerning for potential development of MDS. It is unclear whether these findings occurred due to the underlying disease, the immunosuppressive therapy, and/or treatment with PROMACTA. (…)
761107, 11/20/2018	Emapalumab-lzsg	Hematology	PRF1, RAB27A, SH2D1A, STXBP2, STX11, UNC13D, XIAP<br>(Hemophagocytic Lymphohistiocytosis)	Clinical Studies	**14 CLINICAL STUDIES**<br>(…) A genetic mutation known to cause HLH was present in 82% of patients. The most frequent causative mutations were FHL3-UNC13D (MUNC 13-4) (26%), FHL2-PRF1 (19%), and Griscelli Syndrome type 2 (19%).<br>The HLH mutations in the population enrolled are described in Table 3. (See Table 3)
209606, 09/27/2019	Enasidenib	Oncology	IDH2	Indications and Usage, Dosage and Administration, Clinical Pharmacology, Clinical Studies	**1 INDICATIONS AND USAGE**<br>**1.1 Acute Myeloid Leukemia**<br>IDHIFA is indicated for the treatment of adult patients with relapsed or refractory acute myeloid leukemia (AML) with an isocitrate dehydrogenase-2 (IDH2) mutation as detected by an FDA-approved test.<br><br>**2 DOSAGE AND ADMINISTRATION**<br>**2.1 Patient Selection**<br>Select patients for the treatment of AML with IDHIFA based on the presence of IDH2 mutations in the blood or bone marrow [see Indications and Usage (1.1) and Clinical Studies (14.1)]. Information on FDA-approved tests for the detection of IDH2 mutations in AML is available at http://www.fda.gov/CompanionDiagnostics.<br><br>**12 CLINICAL PHARMACOLOGY**

* Therapeutic areas do not necessarily reflect the CDER review division.

† Representative biomarkers are listed based on standard nomenclature as per the Human Genome Organization (HUGO) symbol and/or simplified descriptors using other common conventions. Listed biomarkers do not necessarily reflect the terminology used in labeling. The term “Nonspecific” is provided when labeling does not explicitly identify the specific biomarker(s) or when the biomarker is represented by a molecular phenotype or gene signature, and in some cases the biomarker was inferred based on the labeling language.

‡ Referenced figures and tables may be found in the labeling available at Drugs@FDA.

Blue text represents the most recent addtions and/or changes since last posted version.

NDA/ANDA/BLA Number, Label Version Date	Drug	Therapeutic Area*	Biomarker†	Labeling Sections	Labeling Text‡
					**12.2 Pharmacodynamics**<br>*Cardiac Electrophysiology*<br>The potential for QTc prolongation with enasidenib was evaluated in an open-label study in patients with advanced hematologic malignancies with an IDH2 mutation. Based on the QTc data for a single dose of 30 mg to 650 mg and multiple doses of 100 mg daily in the fasted state, no large mean changes in the QTc interval (>20 ms) were observed following treatment with enasidenib.<br><br>**14 CLINICAL STUDIES**<br>**14.1 Acute Myeloid Leukemia**<br>The efficacy of IDHIFA was evaluated in an open-label, single-arm, multicenter, two-cohort clinical trial (Study AG221-C-001, NCT01915498) of 199 adult patients with relapsed or refractory AML and an IDH2 mutation, who were assigned to receive 100 mg daily dose. Cohort 1 included 101 patients and Cohort 2 included 98 patients. IDH2 mutations were identified by a local diagnostic test and retrospectively confirmed by the Abbott RealTime™ IDH2 assay, or prospectively identified by the Abbott RealTime™ IDH2 assay, which is the FDA-approved test for selection of patients with AML for treatment with IDHIFA. (See Table 4) (…)<br>(…) Efficacy was established on the basis of the rate of complete response (CR)/complete response with partial hematologic recovery (CRh), the duration of CR/CRh, and the rate of conversion from transfusion dependence to transfusion independence. The efficacy results are shown in Table 5 and were similar in both cohorts. The median follow-up was 6.6 months (range, 0.4 to 27.7 months). Similar CR/CRh rates were observed in patients with either R140 or R172 mutation. (See Table 5) (…)
**210496, 10/13/2023**	**Encorafenib (1)**	Oncology	BRAF	Indications and Usage, Dosage and Administration, Warnings and Precautions, Adverse Reactions, Use in Specific Populations, Clinical Pharmacology, Clinical Studies, Patient Counseling Information	**1 INDICATIONS AND USAGE**<br>**1.1 BRAF V600E or V600K Mutation-Positive Unresectable or Metastatic Melanoma**<br>BRAFTOVI® is indicated, in combination with binimetinib, for the treatment of patients with unresectable or metastatic melanoma with a BRAF V600E or V600K mutation, as detected by an FDA-approved test [see Dosage and Administration (2.1)].<br>**1.2 BRAF V600E Mutation-Positive Metastatic Colorectal Cancer (CRC)**<br>BRAFTOVI® is indicated, in combination with cetuximab, for the treatment of adult patients with metastatic colorectal cancer (CRC) with a BRAF V600E mutation, as detected by an FDA-approved test, after prior therapy [see Dosage and Administration (2.1)].<br>**1.3 BRAF V600E Mutation-Positive Metastatic Non-Small Cell Lung Cancer (NSCLC)**<br>BRAFTOVI is indicated, in combination with binimetinib, for the treatment of adult patients with metastatic non-small cell lung cancer (NSCLC) with a BRAF V600E mutation, as detected by an FDA-approved test [see Dosage and Administration (2.1)].<br>**1.4 Limitations of Use**<br>BRAFTOVI is not indicated for treatment of patients with wild-type BRAF melanoma, wild-type BRAF CRC, or wild-type BRAF NSCLC [see Warnings and Precautions (5.2)].<br><br>**2 DOSAGE AND ADMINISTRATION**<br>**2.1 Patient Selection**<br>BRAF V600E or V600K Mutation-Positive Unresectable or Metastatic Melanoma<br>Confirm the presence of a BRAF V600E or V600K mutation in tumor specimens prior to initiating BRAFTOVI [see Warnings and Precautions (5.2), Clinical Studies (14.1)]. Information on FDA-approved tests for the detection of BRAF V600E and V600K mutations in melanoma is available at: http://www.fda.gov/CompanionDiagnostics.<br>BRAF V600E Mutation-Positive Metastatic Colorectal Cancer (CRC)<br>Confirm the presence of a BRAF V600E mutation in tumor specimens prior to initiating BRAFTOVI [see Warnings and Precautions (5.2), Clinical Studies 14.2)]. Information on FDA-approved tests for the detection of BRAF V600E mutations in CRC is available at: http://www.fda.gov/CompanionDiagnostics.<br>BRAF V600E Mutation-Positive Metastatic Non-Small Cell Lung Cancer (NSCLC)<br>Confirm the presence of a BRAF V600E mutation in tumor or plasma specimens prior to initiating BRAFTOVI [see Warnings and Precautions (5.2), Clinical Studies (14.3)]. If no mutation is detected in a plasma specimen, test tumor tissue. Information on FDA-approved tests for the detection of BRAF V600E mutations in NSCLC is available at: http://www.fda.gov/CompanionDiagnostics.<br>2.2 Recommended Dosage for BRAF V600E or V600K Mutation-Positive Unresectable or Metastatic Melanoma and for BRAF V600E Mutation-Positive Metastatic Non-Small Cell Lung Cancer (NSCLC)<br>The recommended dosage of BRAFTOVI is 450 mg (six 75 mg capsules) orally once daily in combination with binimetinib until disease progression or unacceptable toxicity. Refer to the binimetinib prescribing information for recommended binimetinib dosing information.<br>**2.3 Recommended Dosage for BRAF V600E Mutation-Positive Metastatic Colorectal Cancer (CRC)**<br>The recommended dosage of BRAFTOVI is 300 mg (four 75 mg capsules) orally once daily in combination with cetuximab until disease progression or unacceptable toxicity. Refer to the cetuximab prescribing information for recommended cetuximab dosing information.<br>**2.5 Dosage Modifications for Adverse Reactions**<br>BRAF V600E or V600K Mutation-Positive Unresectable or Metastatic Melanoma or BRAF V600E Mutation-Positive Metastatic NSCLC<br>If binimetinib is withheld, reduce BRAFTOVI to a maximum dose of 300 mg (four 75 mg capsules) once daily until binimetinib is resumed [see Warnings and Precautions (5.9)]. Dose reductions for adverse reactions associated with BRAFTOVI are presented in Table 1.<br>BRAF V600E Mutation-Positive Metastatic Colorectal Cancer (CRC)<br>If cetuximab is discontinued, discontinue BRAFTOVI. Dose reductions for adverse reactions associated with BRAFTOVI are presented in Table 2.

NDA/ANDA/BLA Number, Label Version Date	Drug	Therapeutic Area*	Biomarker†	Labeling Sections	Labeling Text‡
					BRAF V600E or V600K Mutation-Positive Unresectable or Metastatic Melanoma, BRAF V600E Mutation-Positive Metastatic Colorectal Cancer (CRC), or BRAF V600E Mutation-Positive NSCL Dosage modifications for adverse reactions associated with BRAFTOVI are presented in Table 3.<br><br>**5 WARNINGS AND PRECAUTIONS**<br>**5.2 Tumor Promotion in BRAF Wild-Type Tumors**<br>In vitro experiments have demonstrated paradoxical activation of MAP-kinase signaling and increased cell proliferation in BRAF wild-type cells, which are exposed to BRAF inhibitors. Confirm evidence of BRAF V600E or V600K mutation prior to initiating BRAFTOVI [see Indications and Usage (1), Dosage and Administration (2.1)].<br><br>**6 ADVERSE REACTIONS**<br>**6.1 Clinical Trials Experience**<br>BRAF V600E or V600K Mutation-Positive Unresectable or Metastatic Melanoma<br>(…) The safety of BRAFTOVI in combination with binimetinib is described in 192 patients with BRAF V600 mutation-positive unresectable or metastatic melanoma who received BRAFTOVI (450 mg once daily) in combination with binimetinib (45 mg twice daily) in a randomized open-label, active-controlled trial (COLUMBUS). (…)<br>BRAF V600E Mutation-Positive Metastatic Colorectal Cancer (CRC)<br>The safety of BRAFTOVI 300 mg once daily in combination with cetuximab (400 mg/m2 initial dose, followed by 250 mg/m2 weekly) was evaluated in 216 patients with BRAF V600E mutation-positive metastatic CRC in a randomized, open-label, active-controlled trial (BEACON CRC). (…)<br>BRAF V600E Mutation-Positive Metastatic Non-Small Cell Lung Cancer (NSCLC)<br>The safety of BRAFTOVI in combination with binimetinib was evaluated in 98 patients with BRAF V600E mutation-positive metastatic NSCLC who received BRAFTOVI (450 mg once daily) in combination with binimetinib (45 mg twice daily) in an open-label, single-arm trial (PHAROS).<br><br>**8 USE IN SPECIFIC POPULATIONS**<br>**8.5 Geriatric Use**<br>Of the 690 patients with BRAF mutation-positive melanoma who received BRAFTOVI in combination with binimetinib across multiple clinical trials, 20% were aged 65 to 74 years and 8% were aged 75 years and older [see Clinical Studies (14.1)].<br>Of the 216 patients with BRAF V600E mutation-positive metastatic CRC who received BRAFTOVI in combination with cetuximab, 62 (29%) were 65 years of age to up to 75 years of age, while 20 (9%) were 75 years of age and over [see Clinical Studies (14.2)].<br>Of the 98 patients with BRAF V600E mutation-positive metastatic NSCLC who received BRAFTOVI with binimetinib, 62 (63%) were 65 years of age and over and 20 (20%) were 75 years and over [see Clinical Studies (14.3)].<br>No overall differences in the safety or effectiveness of BRAFTOVI plus binimetinib or BRAFTOVI plus cetuximab were observed in older patients as compared to younger patients.<br><br>**12 CLINICAL PHARMACOLOGY**<br>**12.3 Pharmacokinetics**<br>The pharmacokinetics of encorafenib were studied in healthy subjects and patients with solid tumors, including advanced and unresectable or metastatic cutaneous melanoma harboring a BRAF V600E or V600K mutation, BRAF V600E mutation-positive metastatic CRC. After a single dose, systemic exposure of encorafenib was dose proportional over the dose range of 50 mg to 700 mg (0.1 to 1.6 times the maximum recommended dose of 450 mg). After once-daily dosing, systemic exposure of encorafenib was less than dose proportional over the dose range of 50 mg to 800 mg (0.1 to 1.8 times the maximum recommended dose of 450 mg). Steady-state was reached within 15 days, with exposure being 50% lower compared to Day 1; intersubject variability (CV%) of AUC ranged from 12% to 69%.<br><br>**14 CLINICAL STUDIES**<br>**14.1 BRAF V600E or V600K Mutation-Positive Unresectable or Metastatic Melanoma**<br>BRAFTOVI in combination with binimetinib was evaluated in a randomized, active-controlled, open-label, multicenter trial (COLUMBUS; NCT01909453). Eligible patients were required to have BRAF V600E or V600K mutation-positive unresectable or metastatic melanoma, as detected using the bioMerieux THxID™BRAF assay. (…)<br>(…) Based on centralized testing, 100% of patients' tumors tested positive for BRAF mutations; BRAF V600E (88%), BRAF V600K (11%), or both (<1%). (…)<br>**14.2 BRAF V600E Mutation-Positive Metastatic Colorectal Cancer (CRC)**<br>BRAFTOVI in combination with cetuximab was evaluated in a randomized, active-controlled, open-label, multicenter trial (BEACON CRC; NCT02928224). Eligible patients were required to have BRAF V600E mutation-positive metastatic colorectal cancer (CRC), as detected using the Qiagen therascreen BRAF V600E RGQ polymerase chain reaction (PCR) Kit, with disease progression after 1 or 2 prior regimens. (…)<br>**14.3 BRAF V600E Mutation-Positive Metastatic Non-Small Cell Lung Cancer**<br>BRAFTOVI in combination with binimetinib was evaluated in an open-label, multicenter, single-arm study in patients with BRAF V600E mutation-positive metastatic non-small cell lung cancer (NSCLC) (PHAROS; NCT03915951). Eligible patients had a diagnosis of histologically-confirmed metastatic NSCLC with BRAF V600E mutation that was treatment-naïve or had been previously treated with 1 prior line of systemic therapy in the metastatic setting (platinum-based chemotherapy and/or anti-PD-1/PD-L1 therapies), age 18 years or older, Eastern Cooperative Oncology Group (ECOG) performance status (PS) of 0 or 1, and measurable disease as defined by Response Evaluation Criteria in Solid Tumors (RECIST) v1.1. Prior use of BRAF inhibitors or MEK inhibitors was not allowed. Patients received BRAFTOVI 450 mg once daily and binimetinib 45 mg orally twice daily until disease progression or unacceptable toxicity. The major efficacy outcome measures were objective response rate (ORR) per RECIST v1.1 and duration of response (DoR) as assessed by independent review committee (IRC).

* Therapeutic areas do not necessarily reflect the CDER review division.
† Representative biomarkers are listed based on standard nomenclature as per the Human Genome Organization (HUGO) symbol and/or simplified descriptors using other common conventions. Listed biomarkers do not necessarily reflect the terminology used in labeling. The term "Nonspecific" is provided when labeling does not explicitly identify the specific biomarker(s) or when the biomarker is represented by a molecular phenotype or gene signature, and in some cases the biomarker was inferred based on the labeling language.
‡ Referenced figures and tables may be found in the labeling available at Drugs@FDA.
Blue text represents the most recent addtions and/or changes since last posted version.

NDA/ANDA/BLA Number, Label Version Date	Drug	Therapeutic Area*	Biomarker†	Labeling Sections	Labeling Text‡
					In the efficacy population, BRAF V600E mutation status was determined by prospective local testing using tumor tissue (78%) or blood (22%) specimens. Of the 98 patients with BRAF V600E mutation, 6 patients were enrolled into the trial based on testing of their tumor tissue specimens with the FoundationOne CDx tissue test. Of the remaining 92 patients enrolled based on local testing, 68 patients had their tumor tissue specimens retrospectively confirmed as having BRAF V600E positive status by the FoundationOne CDx tissue test. The remaining patients had either BRAF V600E negative status (n=5) or had unevaluable results (n=19) by the FoundationOne CDx tissue test. In addition, plasma samples from 81 out of 98 patients were retrospectively tested using the FoundationOne Liquid CDx assay. Of the 81 patients, 48 were confirmed positive for BRAF V600E, while 33 patients were BRAF V600E mutation negative by FoundationOne Liquid CDx assay. The remaining 17 samples had unevaluable results with FoundationOne Liquid CDx assay.<br>Efficacy results for patients with BRAF V600E mutation-positive metastatic NSCLC are summarized in Table 13.<br><br>**17 PATIENT COUNSELING INFORMATION**<br>Tumor Promotion in BRAF Wild-Type Tumors Advise patients of the need to confirm BRAF V600E or V600K mutation prior to initiating BRAFTOVI [see Warnings and Precautions (5.2)].
**210496, 10/13/2023**	**Encorafenib (2)**	Oncology	RAS	Dosage and Administration, Warnings and Precautions, Clinical Studies	**2 DOSAGE AND ADMINISTRATION**<br>**2.5 Dosage Modifications for Adverse Reactions**<br>BRAF V600E or V600K Mutation-Positive Unresectable or Metastatic Melanoma, BRAF V600E Mutation-Positive Metastatic Colorectal Cancer (CRC), or BRAF V600E Mutation-Positive NSCLC Dosage modifications for adverse reactions associated with BRAFTOVI are presented in Table 3.<br><br>**5 WARNINGS AND PRECAUTIONS**<br>**5.1 New Primary Malignancies**<br>Non-Cutaneous Malignancies<br>Based on its mechanism of action, BRAFTOVI may promote malignancies associated with activation of RAS through mutation or other mechanisms [see Warnings and Precautions (5.2)]. Monitor patients receiving BRAFTOVI for signs and symptoms of non-cutaneous malignancies. Discontinue BRAFTOVI for RAS mutation-positive non-cutaneous malignancies [see Dosage and Administration (2.5)].<br><br>**14 CLINICAL STUDIES**<br>**14.2 BRAF V600E Mutation-Positive Metastatic Colorectal Cancer (CRC)**<br>BRAFTOVI in combination with cetuximab was evaluated in a randomized, active-controlled, open-label, multicenter trial (BEACON CRC; NCT02928224). Eligible patients were required to have BRAF V600E mutation-positive metastatic colorectal cancer (CRC), as detected using the Qiagen therascreen BRAF V600E RGQ polymerase chain reaction (PCR) Kit, with disease progression after 1 or 2 prior regimens. Other key eligibility criteria included absence of prior treatment with a RAF, MEK, or EGFR inhibitor, eligibility to receive cetuximab per local labeling with respect to tumor RAS status, and ECOG performance status (PS) 0-1. (…)
**212725, 10/20/2023**	**Entrectinib (1)**	Oncology	ROS1	Indications and Usage, Dosage and Administration, Adverse Reactions, Use in Specific Populations, Clinical Pharmacology, Clinical Studies	**1 INDICATIONS AND USAGE**<br>**1.1 ROS1-Positive Non-Small Cell Lung Cancer**<br>ROZLYTREK is indicated for the treatment of adult patients with metastatic non-small cell lung cancer (NSCLC) whose tumors are ROS1-positive.<br><br>**2 DOSAGE AND ADMINISTRATION**<br>**2.1 Patient Selection**<br>Select patients for the treatment of metastatic NSCLC with ROZLYTREK based on the presence of ROS1 rearrangement(s) in tumor specimens [see Clinical Studies (14.1)]. An FDA-approved test for detection of ROS1 rearrangement(s) in NSCLC for selecting patients for treatment with ROZLYTREK is not available.<br>**2.2 Recommended Dosage for ROS1-Positive Non-Small Cell Lung Cancer**<br>The recommended dosage of ROZLYTREK is 600 mg orally once daily with or without food until disease progression or unacceptable toxicity.<br><br>**6 ADVERSE REACTIONS**<br>**6.1 Clinical Trial Experience**<br>(…) ROS1 gene fusions were present in 42% and NTRK gene fusions were present in 20%. Most adults (75%) received ROZLYTREK 600 mg orally once daily. (…)<br><br>**8 USE IN SPECIFIC POPULATIONS**<br>**8.4 Pediatric Use**<br>(…) The safety and effectiveness of ROZLYTREK in pediatric patients with ROS1-positive NSCLC have not been established.<br><br>**12 CLINICAL PHARMACOLOGY**<br>**12.3 Pharmacokinetics**<br>The pharmacokinetics for entrectinib and its pharmacologically active major circulating metabolite M5 were characterized in adult patients with ROS1-positive NSCLC, NTRK gene fusion-positive solid tumors, and healthy subjects. (…)<br><br>**14 CLINICAL STUDIES**<br>**14.1 ROS1-Positive Non-Small Cell Lung Cancer**

* Therapeutic areas do not necessarily reflect the CDER review division.
† Representative biomarkers are listed based on standard nomenclature as per the Human Genome Organization (HUGO) symbol and/or simplified descriptors using other common conventions. Listed biomarkers do not necessarily reflect the terminology used in labeling. The term “Nonspecific” is provided when labeling does not explicitly identify the specific biomarker(s) or when the biomarker is represented by a molecular phenotype or gene signature, and in some cases the biomarker was inferred based on the labeling language.
‡ Referenced figures and tables may be found in the labeling available at Drugs@FDA.
Blue text represents the most recent addtions and/or changes since last posted version.

NDA/ANDA/BLA Number, Label Version Date	Drug	Therapeutic Area*	Biomarker†	Labeling Sections	Labeling Text‡
					The efficacy of ROZLYTREK was evaluated in a pooled subgroup of patients with ROS1-positive metastatic NSCLC who received ROZLYTREK at various doses and schedules (90% received ROZLYTREK 600 mg orally once daily) and were enrolled in one of three multicenter, single-arm, open-label clinical trials: ALKA, STARTRK-1 (NCT02097810) and STARTRK-2 (NCT02568267). To be included in this pooled subgroup, patients were required to have histologically confirmed, recurrent or metastatic, ROS1-positive NSCLC, ECOG performance status ≤ 2, measurable disease per RECIST v 1.1, ≥ 18 months of follow-up from first posttreatment tumor assessment, and no prior therapy with a ROS1 inhibitor. Identification of ROS1 gene fusion in tumor specimens was prospectively determined in local laboratories using either a fluorescence in situ hybridization (FISH), next-generation sequencing (NGS), or polymerase chain reaction (PCR) laboratorydeveloped tests. All patients were assessed for CNS lesions at baseline. The major efficacy outcome measures were overall response rate (ORR) and duration of response (DOR) according to RECIST v1.1 as assessed by Blinded Independent Central Review (BICR). Intracranial response according to Response Evaluation Criteria in Solid Tumors (RECIST v1.1) was assessed by BICR. Tumor assessments with imaging were performed every 8 weeks.<br>Efficacy was assessed in 92 patients with ROS1-positive NSCLC. The median age was 53 years (range: 27 to 86); female (65%); White (48%), Asian (45%), and Black (5%); and Hispanic or Latino (2.4%); never smoked (59%); and ECOG performance status 0 or 1 (88%). Ninety-nine percent of patients had metastatic disease, including 42% with CNS metastases; 96% had adenocarcinoma; 65% received prior platinum-based chemotherapy for metastatic or recurrent disease and no patient had progressed in less than 6 months following platinum-based adjuvant or neoadjuvant therapy. ROS1 positivity was determined by NGS in 79%, FISH in 16%, and PCR in 4%. Twenty-five percent had central laboratory confirmation of ROS1 positivity using an analytically validated NGS test.<br>Efficacy results are summarized in Table 12.
**212725, 10/20/2023**	**Entrectinib (2)**	Oncology	NTRK	Indications and Usage, Dosage and Administration, Adverse Reactions, Use in Specific Populations, Clinical Pharmacology, Clinical Studies	**1 INDICATIONS AND USAGE**<br>**1.3 NTRK Gene Fusion-Positive Solid Tumors**<br>ROZLYTREK is indicated for the treatment of adult and pediatric patients older than 1 month of age with solid tumors that:<br>• have a neurotrophic tyrosine receptor kinase (NTRK) gene fusion without a known acquired resistance mutation,<br>• are metastatic or where surgical resection is likely to result in severe morbidity, and<br>• have either progressed following treatment or have no satisfactory alternative therapy.<br>This indication is approved under accelerated approval based on tumor response rate and durability of response [see Clinical Studies (14.2)]. Continued approval for this indication may be contingent upon verification and description of clinical benefit in the confirmatory trials.<br><br>**2 DOSAGE AND ADMINISTRATION**<br>**2.1 Patient Selection**<br>• Select patients for the treatment of metastatic NSCLC with ROZLYTREK based on the presence of ROS1 rearrangement(s) in tumor or plasma specimens [see Clinical Studies (14.1)]. Testing using plasma specimens is only appropriate for patients for whom tumor tissue is not available for testing. Information on FDA-approved tests for the detection of ROS1 rearrangement(s) in NSCLC is available at http://www.fda.gov/CompanionDiagnostics.<br>• Select patients for treatment of locally advanced or metastatic solid tumors with ROZLYTREK based on the presence of a NTRK gene fusion in tumor or plasma specimens [see Clinical Studies (14.2)]. Testing using plasma specimens is only appropriate for patients for whom tumor tissue is not available for testing. Information on FDA-approved tests for the detection of NTRK gene fusion(s) in solid tumors is available at http://www.fda.gov/CompanionDiagnostics.<br>**2.3 Recommended Dosage for NTRK Gene Fusion-Positive Solid Tumors**<br><u>Adults</u><br>The recommended dosages of ROZLYTREK for the treatment of adult and pediatric patients with NTRK Gene Fusion-Positive Solid Tumors are provided in Table 1.<br>The recommended dosages of ROZLYTREK for the treatment of pediatric patients older than 6 months with NTRK Gene Fusion-Positive Solid Tumors is provided in Table 2.<br><br>**6 ADVERSE REACTIONS**<br>**6.1 Clinical Trial Experience**<br>(…) ROS1 gene fusions were present in 42% and NTRK gene fusions were present in 20%. Most adults (75%) received ROZLYTREK 600 mg orally once daily. (…)<br><u>Safety in Pediatric Patients</u><br>The safety of ROZLYTREK was evaluated was evaluated in pediatric patients with unresectable or metastatic solid tumors with a NTRK gene fusion enrolled in one of three multicenter, open-label clinical trials: STARTRK-NG (n=68), TAPISTRY (n=6) and STARTRK-2 (n=2). (…)<br><br>**8 USE IN SPECIFIC POPULATIONS**<br>**8.4 Pediatric Use**<br>The safety and effectiveness of ROZLYTREK in pediatric patients aged 12 years and older with solid tumors that have an NTRK gene fusion have been established. The effectiveness of ROZLYTREK in adolescent patients was established based on extrapolation of data from three open-label, single-arm clinical trials in adult patients with solid tumors harboring an NTRK gene fusion (ALKA, STARTRK-1, and STARTRK-2) and pharmacokinetic data in adolescents enrolled in STARTRK-NG. (…)<br>(…) The safety and effectiveness of ROZLYTREK in pediatric patients less than 12 years of age with solid tumors who have an NTRK gene fusion have not been established.<br><br>**12 CLINICAL PHARMACOLOGY**<br>**12.3 Pharmacokinetics**

NDA/ANDA/BLA Number, Label Version Date	Drug	Therapeutic Area*	Biomarker†	Labeling Sections	Labeling Text‡
					The pharmacokinetics for entrectinib and its pharmacologically active major circulating metabolite M5 were characterized in adult patients with ROS1-positive NSCLC, NTRK gene fusion-positive solid tumors, and healthy subjects. (…)<br><br>**14 CLINICAL STUDIES**<br>**14.2 NTRK Gene Fusion-Positive Solid Tumors**<br>Efficacy in Adult Patients<br>The efficacy of ROZLYTREK was evaluated in a pooled subgroup of adult patients with unresectable or metastatic solid tumors with a NTRK gene fusion enrolled in one of three multicenter, single-arm, open-label clinical trials: ALKA, STARTRK-1 (NCT02097810) and STARTRK-2 (NCT02568267). To be included in this pooled subgroup, patients were required to have progressed following systemic therapy for their disease, if available, or would have required surgery causing significant morbidity for locally advanced disease; measurable disease per RECIST v1.1; at least 2 years of follow-up from first post-treatment tumor assessment; and no prior therapy with a TRK inhibitor. Patients received ROZLYTREK at various doses and schedules (94% received ROZLYTREK 600 mg orally once daily) until unacceptable toxicity or disease progression. Identification of positive NTRK gene fusion status was prospectively determined in local laboratories or a central laboratory using various nucleic acid-based tests. The major efficacy outcome measures were ORR and DOR, as determined by a BICR according to RECIST v1.1. Intracranial response according to RECIST v1.1 as evaluated by BICR. Tumor assessments with imaging were performed every 8 weeks.<br>Efficacy was assessed in the first 54 adult patients with solid tumors with an NTRK gene fusion enrolled into these trials. The median age was 58 years (range: 21 to 83); female (59%); White (80%), Asian (13%) and Hispanic or Latino (7%); and ECOG performance status 0 (43%) or 1 (46%). Ninety-six percent of patients had metastatic disease, including 22% with CNS metastases, and 4% had locally advanced, unresectable disease. All patients had received prior treatment for their cancer including surgery (n = 43), radiotherapy (n = 36), or systemic therapy (n = 48). Forty patients (74%) received prior systemic therapy for metastatic disease with a median of 1 prior systemic regimen and 17% (n = 9) received 3 or more prior systemic regimens. The most common cancers were sarcoma (24%), lung cancer (19%), salivary gland tumors (13%), breast cancer (11%), thyroid cancer (9%), and colorectal cancer (7%). A total of 52 (96%) patients had an NTRK gene fusion detected by NGS and 2 (4%) had an NTRK gene fusion detected by other nucleic acid-based tests. Eighty-three percent of patients had central laboratory confirmation of NTRK gene fusion using an analytically validated NGS test.<br>Efficacy results are summarized in Tables 13, 14, and 15.<br>Efficacy in Pediatric Patients<br>The efficacy of ROZLYTREK was evaluated in pediatric patients with unresectable or metastatic solid tumors with a NTRK gene fusion enrolled in one of two multicenter, open-label clinical trials: STARTRK-NG (NCT02650401) and TAPISTRY (NCT04589845). To be included in the analysis, patients were required to have received at least 1 dose of ROZLYTREK; measurable or evaluable disease at baseline; at least 6 months of follow-up; and no prior therapy with a TRK inhibitor. Patients received ROZLYTREK 20 mg to 600 mg based on body surface area (BSA) orally or via enteral feeding tube once daily in 4-week cycles until unacceptable toxicity or disease progression. The major efficacy outcome measure was overall response rate (ORR) as assessed by BICR according to RECIST v1.1 for extracranial tumors and according to Response Assessment in Neuro-Oncology (RANO) for primary central nervous system (CNS) tumors. An additional efficacy outcome measure was DOR as evaluated by BICR.<br>Efficacy was assessed in 33 pediatric patients with NTRK fusion-positive solid tumors treated with ROZLYTREK. The median age was 4 years (range: 2 months to 15 years); male (52%); White (58%), Asian (30%), other races (9%), Black or African American (3.0%), and Hispanic or Latino (9%). Seventy-one percent of patients had locally advanced disease and 29% had metastatic disease. Eighty-five percent of patients had received prior treatment for their cancer including surgery (n=20), radiotherapy (n=7) and/or systemic therapy (n=22). The sites for metastatic disease included other (4 patients), brain (3 patients) and lung (2 patients).<br>Efficacy results are summarized in Tables 16 and 17.
**217388, 12/21/2023**	Eplontersen	Neurology	TTR	Adverse Reactions, Clinical Pharmacology, Clinical Studies	**6 ADVERSE REACTIONS**<br>Because clinical trials are conducted under widely varying conditions, adverse reaction rates observed in the clinical trials of WAINUA cannot be directly compared to rates in the clinical trials of another drug and may not reflect the rates observed in practice.<br>In Study 1 [see Clinical Studies (14)], a total of 144 patients with polyneuropathy caused by hereditary transthyretin-mediated amyloidosis (hATTR amyloidosis) were randomized to WAINUA and received at least one dose of WAINUA. Of these, 141 patients received at least 6 months of treatment and 107 patients received at least 12 months of treatment. The mean duration of treatment was 15 months (range: 1.9 to 19.4 months). The median patient age at baseline was 52 years and 69% of the patients were male. Seventy-eight percent of patients treated with WAINUA were White, 15% were Asian, 4% were Black, 2% were reported as other races, and <1% were multiple races. Fifty-nine percent of patients had the Val30Met variant in the transthyretin gene; the remaining patients had one of 19 other variants. At baseline, 80% of patients were in Stage 1 of the disease and 20% were in Stage 2 with a mean duration from polyneuropathy diagnosis of 47 months. The mean duration from onset of polyneuropathy symptoms was 68 months. (…)<br><br>**12 CLINICAL PHARMACOLOGY**<br>**12.2 Pharmacodynamics**<br>In Study 1 [see Clinical Studies (14)], following administration of the recommended WAINUA dosage every 4 weeks to patients with hATTR amyloidosis, a decrease in serum TTR levels was observed at the first assessment and the (least square) mean serum TTR at Week 35 was reduced by 81% from baseline. Similar TTR reductions were observed across subgroups including Val30Met variant status, body weight, sex, age, or race.<br>Eplontersen also reduced the mean steady state serum vitamin A by 71% by Week 37 [see Warnings and Precautions (5.1)].<br>Specific Populations<br>Population pharmacokinetic and pharmacodynamic analysis showed no clinically meaningful differences in the pharmacokinetics or pharmacodynamics of eplontersen based on age, body weight, sex, race, Val30Met variant status, mild and moderate renal impairment (eGFR≥30 to 1 x ULN, or total bilirubin >1.0 to 1.5 x ULN and any AST). Eplontersen has not been studied in patients with severe renal impairment, end-stage renal disease, or in patients with moderate to severe hepatic impairment, or in patients with prior liver transplant.

* Therapeutic areas do not necessarily reflect the CDER review division.
† Representative biomarkers are listed based on standard nomenclature as per the Human Genome Organization (HUGO) symbol and/or simplified descriptors using other common conventions. Listed biomarkers do not necessarily reflect the terminology used in labeling. The term “Nonspecific” is provided when labeling does not explicitly identify the specific biomarker(s) or when the biomarker is represented by a molecular phenotype or gene signature, and in some cases the biomarker was inferred based on the labeling language.
‡ Referenced figures and tables may be found in the labeling available at Drugs@FDA.
Blue text represents the most recent addtions and/or changes since last posted version.

NDA/ANDA/BLA Number, Label Version Date	Drug	Therapeutic Area*	Biomarker†	Labeling Sections	Labeling Text‡
					**14 CLINICAL STUDIES**<br>(…) Patients receiving WAINUA experienced similar improvements relative to those in the external placebo in mNIS+7, and Norfolk QoL-DN score across subgroups including age, sex, race, region, Val30Met variant status, and disease stage.
212018, 01/19/2024	Erdafitinib (1)	Oncology	FGFR3	Indications and Usage, Dosage and Administration, Adverse Reactions, Clinical Studies, Patient Counseling Information	**1 INDICATIONS AND USAGE**<br>BALVERSA is indicated for the treatment of adult patients with locally advanced or metastatic urothelial carcinoma (mUC) with susceptible FGFR3 genetic alterations whose disease has progressed on or after at least one line of prior systemic therapy.<br>Select patients for therapy based on an FDA-approved companion diagnostic for BALVERSA [see Dosage and Administration (2.1) and Clinical Studies (14.1)].<br>Limitations of Use<br>BALVERSA is not recommended for the treatment of patients who are eligible for and have not received prior PD-1 or PD-L1 inhibitor therapy [see Clinical Studies (14.1)].<br><br>**2 DOSAGE AND ADMINISTRATION**<br>**2.1 Patient Selection**<br>Select patients for the treatment of locally advanced or metastatic urothelial carcinoma with BALVERSA based on the presence of susceptible FGFR3 genetic alterations in tumor specimens as detected by an FDA-approved companion diagnostic [see Clinical Studies (14.1)]. Information on FDA-approved tests for the detection of FGFR3 genetic alterations in urothelial cancer is available at: http://www.fda.gov/CompanionDiagnostics.<br><br>**6 ADVERSE REACTIONS**<br>**6.1 Clinical Trials Experience**<br>Because clinical trials are conducted under widely varying conditions, adverse reaction rates observed in the clinical trials of a drug cannot be directly compared to rates in the clinical trials of another drug and may not reflect the rates observed in practice.<br>The pooled safety population described in the WARNINGS AND PRECAUTIONS reflects exposure to BALVERSA as a single agent at the recommended dose (8 to 9 mg orally daily) in 479 patients with advanced urothelial cancer and FGFR alterations in 42756493BLC3001 (NCT03390504), 42756493BLC2001 (NCT02365597), 42756493BLC2002 (NCT 03473743), and 42756493EDI1001 (NCT01703481). (…)<br>BLC3001<br>The safety of BALVERSA was evaluated in Cohort 1 of the BLC3001 study that included patients with locally advanced unresectable or metastatic urothelial carcinoma which had susceptible FGFR3 genetic alterations and were previously treated with a PD-1 or PD-L1 inhibitor [see Clinical Studies (14.1)]. Patients received either BALVERSA (8 mg orally once daily with individualized up-titration to 9 mg) (n=135) or chemotherapy (docetaxel 75 mg/m2 once every 3 weeks or vinflunine 320 mg/m2 once every 3 weeks) (n=112). Among patients who received BALVERSA, median duration of treatment was 4.8 months (range: 0.2 to 38 months).<br>BLC2001<br>The safety of BALVERSA was evaluated in the BLC2001 study that included 87 patients with locally advanced or metastatic urothelial carcinoma which had susceptible FGFR3 and other FGFR alterations, and which progressed during or following at least one line of prior chemotherapy including within 12 months of neoadjuvant or adjuvant chemotherapy [see Clinical Studies (14.1)]. Patients were treated with BALVERSA at 8 mg orally once daily; with a dose increase to 9 mg in patients with phosphate levels<br><br>**14 CLINICAL STUDIES**<br>**14.1 Urothelial Carcinoma with Susceptible FGFR3 Genetic Alterations**<br>The efficacy of BALVERSA was evaluated in Study BLC3001 (NCT03390504) Cohort 1, a randomized, open-label, multicenter study in which 266 patients with advanced urothelial cancer harboring selected FGFR3 alterations were randomized 1:1 to receive BALVERSA (8 mg with titration up to 9 mg) versus chemotherapy (docetaxel 75 mg/m2 once every 3 weeks or vinflunine 320 mg/m2 once every 3 weeks) until unacceptable toxicity or progression. Randomization was stratified by region (North America vs. Europe vs. rest of world), Eastern Cooperative Oncology Group (ECOG) performance status (0 or 1 vs. 2) and visceral or bone metastases (yes vs. no). All patients needed to have had disease progression after 1 or 2 prior treatments, at least 1 of which included a PD-1 or PD-L1 inhibitor. FGFR3 genetic alterations were identified from tumor tissue in a central laboratory by the QIAGEN therascreen® FGFR RGQ RT-Polymerase Chain Reaction (PCR) kit in 75% of patients while the remainder (25%) were identified by local next generation sequencing (NGS) assays.<br>The median age was 67 years (range: 32 to 86 years) and 71% were male; 54% were White, 29% Asian, 0.4% Black, 0.4% multiple races, 16% not reported; 2% were Hispanic/Latino; and baseline ECOG performance status was 0 (43%), 1 (48%), or 2 (9%). Eighty-one percent of patients had FGFR3 mutations, 17% had fusions, and 2% had both mutations and fusions. (…)<br>Study BLC3001<br>Cohort 2 Study BLC3001 (NCT03390504) Cohort 2 was a multicenter, open-label, randomized study in 351 patients with locally advanced or metastatic urothelial carcinoma with selected FGFR3 alterations who received 1 prior line of systemic therapy and no prior PD-1 or PD-L1 inhibitor. Patients were randomized 1:1 to receive BALVERSA (8 mg with titration up to 9 mg) or pembrolizumab 200 mg every 3 weeks. The study did not meet its major efficacy outcome measure for superiority of OS at the pre-specified final analysis. The OS hazard ratio (HR) was 1.18 (95% CI: 0.92, 1.51; p=0.18), median 10.9 (95% CI: 9.2, 12.6) months for BALVERSA versus 11.1 (95% CI: 9.7, 13.6) months for pembrolizumab [see Indications and Usage (1)].<br>Study BLC2001<br>Study BLC2001 (NCT02365597) was a multicenter, open-label, single-arm study to evaluate the efficacy and safety of BALVERSA in patients with locally advanced or metastatic urothelial carcinoma (mUC). FGFR mutation status for screening and enrollment of patients was determined by a clinical trial assay (CTA). The efficacy population consists of a cohort of eighty-seven patients who were enrolled in this study with disease that had progressed on or after at least

Table of Pharmacogenomic Biomarkers in Drug Labeling

Last Updated: 06/2024

NDA/ANDA/BLA Number, Label Version Date	Drug	Therapeutic Area*	Biomarker†	Labeling Sections	Labeling Text‡
					one prior chemotherapy and that had at least 1 of the following genetic alterations: FGFR3 gene mutations (R248C, S249C, G370C, Y373C) or FGFR gene fusions (FGFR3-TACC3, FGFR3-BAIAP2L1, FGFR2-BICC1, FGFR2-CASP7), as determined by the CTA performed at a central laboratory. Tumor samples from 69 patients were tested retrospectively by the QIAGEN therascreen® FGFR RGQ RT-PCR Kit, which is the FDA-approved test for selection of patients with mUC for BALVERSA. (See Table 11)<br><br>**17 PATIENT COUNSELING INFORMATION**<br>Advise the patient to read the FDA-approved patient labeling (Patient Information).<br>FGFR Genetic Alterations Advise patients that evidence of a susceptible FGFR3 mutation or gene fusion within the tumor specimen is necessary to identify patients for whom treatment is indicated [see Dosage and Administration (2.1)].
**212018, 01/19/2024**	Erdafitinib (2)	Oncology	CYP2C9	Use in Specific Populations, Clinical Pharmacology	**8 USE IN SPECIFIC POPULATIONS**<br>**8.6 CYP2C9 Poor Metabolizers**<br>CYP2C9*3/*3 Genotype: Erdafitinib plasma concentrations were predicted to be higher in patients with the CYP2C9*3/*3 genotype. Monitor for increased adverse reactions in patients who are known or suspected to have CYP2C9*3/*3 genotype [see Pharmacogenomics (12.5)].<br><br>**12 CLINICAL PHARMACOLOGY**<br>**12.5 Pharmacogenomics**<br>CYP2C9 activity is reduced in individuals with genetic variants, such as the CYP2C9*2 and CYP2C9*3 polymorphisms. Erdafitinib exposure was similar in subjects with CYP2C9*1/*2 and *1/*3 genotypes relative to subjects with CYP2C9*1/*1 genotype (wild type). No data are available in subjects characterized by other genotypes (e.g., *2/*2, *2/*3, *3/*3). Simulation suggested no clinically meaningful differences in erdafitinib exposure in subjects with CYP2C9*2/*2 and *2/*3 genotypes. The exposure of erdafitinib is predicted to be 50% higher in subjects with the CYP2C9*3/*3 genotype, estimated to be present in 0.4% to 3% of the population among various ethnic groups.
**201532, 10/19/2016**	Eribulin (1)	Oncology	ERBB2 (HER2)	Clinical Studies	**14 CLINICAL STUDIES**<br>**14.1 Metastatic Breast Cancer**<br>(…) Randomization was stratified by geographic region, HER2/neu status, and prior capecitabine exposure. HALAVEN was administered at a dose of 1.4 mg/m2 on Days 1 and 8 of a 21-day cycle. HALAVEN-treated patients received a median of 5 cycles (range: 1 to 23 cycles) of therapy. Control arm therapy consisted of 97% chemotherapy (26% vinorelbine, 18% gemcitabine, 18% capecitabine, 16% taxane, 9% anthracycline, 10% other chemotherapy), and 3% hormonal therapy. The main efficacy outcome was overall survival. (…)<br>**(…)** Tumor prognostic characteristics, including estrogen receptor status (positive: 67%, negative: 28%), progesterone receptor status (positive: 49%, negative: 39%), HER2/neu receptor status (positive: 16%, negative: 74%), triple negative status (ER , PR- , HER2/neu - : 19%), presence of visceral disease (82%, including 60% liver and 38% lung) and bone disease (61%), and number of sites of metastases (greater than two: 50%), were also similar in the HALAVEN and control arms. Patients received a median of four prior chemotherapy regimens in both arms. (See Table 5) (…)
**201532, 10/19/2016**	Eribulin (2)	Oncology	ESR, PGR (Hormone Receptor	Clinical Studies	**14 CLINICAL STUDIES**<br>**14.1 Metastatic Breast Cancer**<br>**(…)** Tumor prognostic characteristics, including estrogen receptor status (positive: 67%, negative: 28%), progesterone receptor status (positive: 49%, negative: 39%), HER2/neu receptor status (positive: 16%, negative: 74%), triple negative status (ER , PR- , HER2/neu - : 19%), presence of visceral disease (82%, including 60% liver and 38% lung) and bone disease (61%), and number of sites of metastases (greater than two: 50%), were also similar in the HALAVEN and control arms. Patients received a median of four prior chemotherapy regimens in both arms. (See Table 5) (…)
**021743, 10/18/2016**	Erlotinib	Oncology	EGFR	Indications and Usage, Dosage and Administration, Adverse Reactions, Clinical Studies	**1 INDICATIONS AND USAGE**<br>**1.1 Non-Small Cell Lung Cancer (NSCLC)**<br>TARCEVA® is indicated for:<br>• The treatment of patients with metastatic non-small cell lung cancer (NSCLC) whose tumors have epidermal growth factor receptor (EGFR) exon 19 deletions or exon 21 (L858R) substitution mutations as detected by an FDA-approved test receiving first-line, maintenance, or second or greater line treatment after progression following at least one prior chemotherapy regimen [see Clinical Studies (14.1, 14.3)].<br>Limitations of use:<br>• Safety and efficacy of TARCEVA have not been established in patients with NSCLC whose tumors have other EGFR mutations [see Clinical Studies (14.1, 14.2)].<br><br>**2 DOSAGE AND ADMINISTRATION**<br>**2.1 Selection of Patients with Metastatic NSCLC**<br>Select patients for the treatment of metastatic NSCLC with TARCEVA based on the presence of EGFR exon 19 deletions or exon 21 (L858R) substitution mutations in tumor or plasma specimens [See Clinical Studies (14.1, 14.2)]. If these mutations are not detected in a plasma specimen, test tumor tissue if available. Information on FDA-approved tests for the detection of EGFR mutations in NSCLC is available at: http://www.fda.gov/CompanionDiagnostics.<br><br>**6 ADVERSE REACTIONS**<br>**6.1 Clinical Trial Experience**<br>*Non-Small Cell Lung Cancer*<br>*First-Line Treatment of Patients with EGFR Mutations*<br>The most frequent (≥ 30%) adverse reactions in TARCEVA-treated patients were diarrhea, asthenia, rash, cough, dyspnea, and decreased appetite. In TARCEVA-treated patients the median time to onset of rash was 15 days and the median time to onset of diarrhea was 32 days. (…)

* Therapeutic areas do not necessarily reflect the CDER review division.

† Representative biomarkers are listed based on standard nomenclature as per the Human Genome Organization (HUGO) symbol and/or simplified descriptors using other common conventions. Listed biomarkers do not necessarily reflect the terminology used in labeling. The term “Nonspecific” is provided when labeling does not explicitly identify the specific biomarker(s) or when the biomarker is represented by a molecular phenotype or gene signature, and in some cases the biomarker was inferred based on the labeling language.

‡ Referenced figures and tables may be found in the labeling available at Drugs@FDA.

Blue text represents the most recent addtions and/or changes since last posted version.

Table of Pharmacogenomic Biomarkers in Drug Labeling
Last Updated: 06/2024

NDA/ANDA/BLA Number, Label Version Date	Drug	Therapeutic Area*	Biomarker†	Labeling Sections	Labeling Text‡
					**14 CLINICAL STUDIES**<br>**14.1 Non-Small Cell Lung Cancer (NSCLC) – First-Line Treatment of Patients with EGFR Mutations**<br>*Study 1*<br>The safety and efficacy of TARCEVA as monotherapy for the first-line treatment of patients with metastatic NSCLC containing EGFR exon 19 deletions or exon 21 (L858R) substitution mutations was demonstrated in Study 1, a randomized, open label, clinical trial conducted in Europe. One hundred seventy-four (174) White patients were randomized 1:1 to receive erlotinib 150 mg once daily until disease progression (n = 86) or four cycles of a standard platinum-based doublet chemotherapy (n = 88); standard chemotherapy regimens were cisplatin plus gemcitabine, cisplatin plus docetaxel, carboplatin plus gemcitabine, and carboplatin plus docetaxel. The main efficacy outcome measure was progression-free survival (PFS) as assessed by the investigator. Randomization was stratified by EGFR mutation (exon 19 deletion or exon 21 (L858R) substitution) and Eastern Cooperative Oncology Group Performance Status (ECOG PS) (0 vs. 1 vs. 2). EGFR mutation status for screening and enrollment of patients was determined by a clinical trials assay (CTA). Tumor samples from 134 patients (69 patients from the erlotinib arm and 65 patients from the chemotherapy arm) were tested retrospectively by the FDA-approved companion diagnostic, cobas® EGFR Mutation Test. (…)<br>(…) The disease characteristics were 93% Stage IV and 7% Stage IIIb with pleural effusion as classified by the American Joint Commission on Cancer (AJCC, 6th edition), 93% adenocarcinoma, 66% exon 19 mutation deletions and 34% exon 21 (L858R) point mutation by CTA. (…)<br>(…) In exploratory subgroup analyses based on EGFR mutation subtype, the hazard ratio (HR) for PFS was 0.27 (95% CI 0.17 to 0.43) in patients with exon 19 deletions and 0.52 (95% CI 0.29 to 0.95) in patients with exon 21 (L858R) substitution. The HR for OS was 0.94 (95% CI 0.57 to 1.54) in the exon 19 deletion subgroup and 0.99 (95% CI 0.56 to 1.76) in the exon 21 (L858R) substitution subgroup.<br>**14.2 NSCLC - Lack of Efficacy of TARCEVA in Maintenance Treatment of Patients without EGFR Mutations**<br>Lack of efficacy of TARCEVA for the maintenance treatment of patients with NSCLC without EGFR activating mutations was demonstrated in Study 2. Study 2 was a multicenter, placebo-controlled, randomized trial of 643 patients with advanced NSCLC without an EGFR exon 19 deletion or exon 21 L858R mutation who had not experienced disease progression after four cycles of platinum-based chemotherapy. (…)<br>**14.3 NSCLC – Maintenance Treatment or Second/Third Line Treatment**<br>Two randomized, double-blind, placebo-controlled trials, Studies 3 and 4, examined the efficacy and safety of TARCEVA administered to patients with metastatic NSCLC as maintenance therapy after initial treatment with chemotherapy (Study 3) or with disease progression following initial treatment with chemotherapy (Study 4). Determination of EGFR mutation status was not required for enrollment. (…)<br>(…) Disease characteristics were as follows: Stage IV (75%), Stage IIIb with effusion (25%) as classified by AJCC (6th edition) with histologic subtypes of adenocarcinoma including bronchioalveolar (45%), squamous (40%) and large cell (5%); and EGFR IHC positive (70%), negative (14%), indeterminate (4%), and missing (12%). (…)
**062759**	Erythromycin and Sulfisoxazole	Infectious Diseases	G6PD	Precautions	Labeling not electronically available on Drugs@FDA
**021323, 01/11/2019**	Escitalopram (1)	Psychiatry	CYP2D6	Drug Interactions	**7 DRUG INTERACTIONS**<br>**7.19 Drugs Metabolized by Cytochrome P4502D6**<br>In vitro studies did not reveal an inhibitory effect of escitalopram on CYP2D6. In addition, steady state levels of racemic citalopram were not significantly different in poor metabolizers and extensive CYP2D6 metabolizers after multiple-dose administration of citalopram, suggesting that coadministration, with escitalopram, of a drug that inhibits CYP2D6, is unlikely to have clinically significant effects on escitalopram metabolism. (…)
**021323, 01/11/2019**	Escitalopram (2)	Psychiatry	CYP2C19	Adverse Reactions	**6 ADVERSE REACTIONS**<br>**6.1 Clinical Trials Experience**<br>*ECG Changes*<br>(…) Based on the established exposure-response relationship, the predicted QTcF change from placebo arm (95% confidence interval) under the Cmax for the dose of 20 mg is 6.6 (7.9) msec. Escitalopram 30 mg given once daily resulted in mean Cmax of 1.7-fold higher than the mean Cmax for the maximum recommended therapeutic dose at steady state (20 mg). The exposure under supratherapeutic 30 mg dose is similar to the steady state concentrations expected in CYP2C19 poor metabolizers following a therapeutic dose of 20 mg.
**022101, 03/04/2022**	Esomeprazole	Gastroenterology	CYP2C19	Drug Interactions, Clinical Pharmacology	**7 DRUG INTERACTIONS**<br>(…) (See Table 3)<br><br>**12 CLINICAL PHARMACOLOGY**<br>**12.3 Pharmacokinetics**<br>(…) *Effect of Other Drugs on Esomeprazole/Omeprazole*<br>*St. John's Wort*<br>In a cross-over study in 12 healthy male subjects, St. John's Wort (300 mg three times daily for 14 days) significantly decreased the systemic exposure of omeprazole in CYP2C19 poor metabolizers (Cmax and AUC both decreased by 38%) and extensive metabolizers (Cmax and AUC decreased by 50% and 44%, respectively) [see Drug Interactions (7)].<br>**12.5 Pharmacogenomics**<br>CYP2C19, a polymorphic enzyme, is involved in the metabolism of esomeprazole. The CYP2C19*1 allele is fully functional while the CYP2C19*2 and *3 alleles are nonfunctional. There are other alleles associated with no or reduced enzymatic function. Patients carrying two fully functional alleles are extensive metabolizers and those carrying two lossof-function alleles are poor metabolizers. The systemic exposure to esomeprazole varies with a patient's metabolism status: poor metabolizers > intermediate metabolizers > extensive metabolizers. Approximately 3% of Caucasians and 15 to 20% of Asians are CYP2C19 poor metabolizers. Systemic esomeprazole exposures were modestly higher (approximately 17%) in CYP2C19 intermediate metabolizers (IM; n=6) compared to

* Therapeutic areas do not necessarily reflect the CDER review division.
† Representative biomarkers are listed based on standard nomenclature as per the Human Genome Organization (HUGO) symbol and/or simplified descriptors using other common conventions. Listed biomarkers do not necessarily reflect the terminology used in labeling. The term "Nonspecific" is provided when labeling does not explicitly identify the specific biomarker(s) or when the biomarker is represented by a molecular phenotype or gene signature, and in some cases the biomarker was inferred based on the labeling language.
‡ Referenced figures and tables may be found in the labeling available at Drugs@FDA.
Blue text represents the most recent addtions and/or changes since last posted version.

Table of Pharmacogenomic Biomarkers in Drug Labeling
Last Updated: 06/2024

NDA/ANDA/BLA Number, Label Version Date	Drug	Therapeutic Area*	Biomarker†	Labeling Sections	Labeling Text‡
					extensive metabolizers (EM; n=17) of CYP2C19. Similar pharmacokinetic differences were noted across these genotypes in a study of Chinese healthy subjects that included 7 EMs and 11 IMs. There is very limited pharmacokinetic information for poor metabolizers (PM) from these studies. At steady state following once daily administration of esomeprazole 40 mg, the ratio of AUC in poor metabolizers to AUC in the rest of the population (EMs) is approximately 1.5. This change in exposure is not considered clinically meaningful.
**009402, 02/16/2022**	**Estradiol Valerate**	Gynecology	ESR, PGR (Hormone Receptor)	Warnings	**WARNINGS**<br>**2. Malignant neoplasms**<br>b. Breast cancer<br>(…) Other prognostic factors such as histologic subtype, grade and hormone receptor status did not differ between the groups. (…)
**210132, 10/28/2018**	**Estradiol and Progesterone (1)**	Gynecology	PROC	Contraindications	**4 CONTRAINDICATIONS**<br>BIJUVA (estradiol and progesterone) capsules, 1 mg/100 mg, are contraindicated in women with any of the following conditions:<br>• Known protein C, protein S, or antithrombin deficiency, or other known thrombophilic disorders
**210132, 10/28/2018**	**Estradiol and Progesterone (2)**	Gynecology	PROS1	Contraindications	**4 CONTRAINDICATIONS**<br>BIJUVA (estradiol and progesterone) capsules, 1 mg/100 mg, are contraindicated in women with any of the following conditions:<br>• Known protein C, protein S, or antithrombin deficiency, or other known thrombophilic disorders
**210132, 10/28/2018**	**Estradiol and Progesterone (3)**	Gynecology	SERPINC1 (Antithrombin III)	Contraindications	**4 CONTRAINDICATIONS**<br>BIJUVA (estradiol and progesterone) capsules, 1 mg/100 mg, are contraindicated in women with any of the following conditions:<br>• Known protein C, protein S, or antithrombin deficiency, or other known thrombophilic disorders
**206488, 10/11/2018**	**Eteplirsen**	Neurology	DMD	Indications and Usage, Adverse Reactions, Use in Specific Populations, Clinical Studies	**1 INDICATIONS AND USAGE**<br>EXONDYS 51 is indicated for the treatment of Duchenne muscular dystrophy (DMD) in patients who have a confirmed mutation of the DMD gene that is amenable to exon 51 skipping. This indication is approved under accelerated approval based on an increase in dystrophin in skeletal muscle observed in some patients treated with EXONDYS 51 [see Clinical Studies (14)]. A clinical benefit of EXONDYS 51 has not been established. Continued approval for this indication may be contingent upon verification of a clinical benefit in confirmatory trials.<br><br>**6 ADVERSE REACTIONS**<br>**6.1 Clinical Trials Experience**<br>(…) In the EXONDYS 51 clinical development program, 107 patients received at least one intravenous dose of EXONDYS 51, ranging between 0.5 mg/kg (0.017 times the recommended dosage) and 50 mg/kg (1.7 times the recommended dosage). All patients were male and had genetically confirmed Duchenne muscular dystrophy. Age at study entry was 4 to 19 years. Most (89%) patients were Caucasian. (…)<br><br>**8 USE IN SPECIFIC POPULATIONS**<br>**8.4 Pediatric Use**<br>EXONDYS 51 is indicated for the treatment of Duchenne muscular dystrophy (DMD) in patients who have a confirmed mutation of the DMD gene that is amenable to exon 51 skipping, including pediatric patients [see Clinical Studies (14)]. (…)<br><br>**14 CLINICAL STUDIES**<br>EXONDYS 51 was evaluated in three clinical studies in patients who have a confirmed mutation of the DMD gene that is amenable to exon 51 skipping. (…)
**216956, 10/12/2023**	**Etrasimod**	Gastroenterology	CYP2C9	Drug Interactions, Use in Specific Populations, Clinical Pharmacology	**7 DRUG INTERACTIONS**<br>**CYP2C9 Poor Metabolizers Using Moderate to Strong Inhibitors of CYP2C8 or CYP3A4**<br>*Clinical Impact*- Increased exposure of etrasimod in patients who are CYP2C9 poor metabolizers is expected with concomitant use of moderate to strong inhibitors of CYP2C8 or CYP3A4 [see Clinical Pharmacology (12.3, 12.5)].<br>*Prevention or Management*- Concomitant use not recommended.<br><br>**8 USE IN SPECIFIC POPULATIONS**<br>**8.7 CYP2C9 Poor Metabolizers**<br>Increased exposure of etrasimod in patients who are CYP2C9 poor metabolizers is expected with concomitant use of moderate to strong inhibitors of CYP2C8 or CYP3A4. Concomitant use of VELSIPITY is not recommended in these patients [see Clinical Pharmacology (12.3, 12.5)].<br><br>**12 CLINICAL PHARMACOLOGY**<br>**12.5 Pharmacogenomics**<br>CYP2C9 activity is decreased in individuals with genetic variants such as CYP2C9*2 and CYP2C9*3 alleles. The impact of CYP2C9 genetic variants on the pharmacokinetics of etrasimod has not been directly evaluated. CYP2C9 poor metabolizers (e.g., *2/*3, *3/*3) may have decreased clearance of etrasimod when VELSIPITY is used concomitantly with moderate to strong inhibitors of CYP2C8 or CYP3A4 [see Drug Interactions (7) and Use in Specific Populations (8.7)].<br>CYP2C9 intermediate metabolizers (e.g., *1/*2, *1/*3, *2/*2) may have decreased clearance of etrasimod when VELSIPITY is used concomitantly with moderate to strong inhibitors of CYP2C8 or CYP3A4; however, the effect on VELSIPITY exposure in CYP2C9 intermediate metabolizers with concomitant CYP2C8 or CYP3A4 inhibitors is not known.<br>The prevalence of the CYP2C9 poor metabolizer phenotype is approximately 2 to 3% in White populations, 0.5 to 4% in Asian populations, and <1% in African-American populations. Other decreased or nonfunctional CYP2C9 alleles (e.g., *5, *6, *8, *11) are more prevalent in African-American populations.

* Therapeutic areas do not necessarily reflect the CDER review division.
† Representative biomarkers are listed based on standard nomenclature as per the Human Genome Organization (HUGO) symbol and/or simplified descriptors using other common conventions. Listed biomarkers do not necessarily reflect the terminology used in labeling. The term “Nonspecific” is provided when labeling does not explicitly identify the specific biomarker(s) or when the biomarker is represented by a molecular phenotype or gene signature, and in some cases the biomarker was inferred based on the labeling language.
‡ Referenced figures and tables may be found in the labeling available at Drugs@FDA.
Blue text represents the most recent addtions and/or changes since last posted version.

Table of Pharmacogenomic Biomarkers in Drug Labeling

Last Updated: 06/2024

NDA/ANDA/BLA Number, Label Version Date	Drug	Therapeutic Area*	Biomarker†	Labeling Sections	Labeling Text‡
**022334, 02/13/2020**	Everolimus (1)	Oncology	ERBB2 (HER2)	Indications and Usage, Dosage and Administration, Warnings and Precautions, Adverse Reactions, Use in Specific Populations, Clincal Pharmacology, Clinical Studies	**1 INDICATIONS AND USAGE**<br>**1.1 Hormone Receptor-Positive, HER2-Negative Breast Cancer**<br>AFINITOR® is indicated for the treatment of postmenopausal women with advanced hormone receptor-positive, HER2 negative breast cancer in combination with exemestane, after failure of treatment with letrozole or anastrozole.<br><br>**2 DOSAGE AND ADMINISTRATION**<br>**2.2 Recommended Dosage for Hormone Receptor-Positive, HER2-Negative Breast Cancer**<br>The recommended dosage of AFINITOR is 10 mg orally once daily until disease progression or unacceptable toxicity.<br><br>**5 WARNINGS AND PRECAUTIONS**<br>**5.7 Geriatric Patients**<br>In the randomized advanced hormone receptor-positive, HER2-negative breast cancer study, the incidence of deaths due to any cause within 28 days of the last AFINITOR dose was 6% in patients ≥ 65 years of age compared to 2% in patients < 65 years of age. Adverse reactions leading to permanent treatment discontinuation occurred in 33% of patients ≥ 65 years of age compared to 17% in patients < 65 years of age. Careful monitoring and appropriate dose adjustments for adverse reactions are recommended [see Dosage and Administration (2.2), Use in Specific Populations (8.5)]. (…)<br><br>**6 ADVERSE REACTIONS**<br>**6.1 Clinical Study Experience**<br><u>Hormone Receptor-Positive, HER2 Negative Breast Cancer</u><br>The safety of AFINITOR (10 mg orally once daily) in combination with exemestane (25 mg orally once daily) (n = 485) vs. placebo in combination with exemestane (n = 239) was evaluated in a randomized, controlled trial (BOLERO-2) in patients with advanced or metastatic hormone receptor-positive, HER2-negative breast cancer. The median age of patients was 61 years (28 to 93 years), and 75% were White. The median follow-up was approximately 13 months. (…)<br><u>Topical Prophylaxis for Stomatitis</u><br>In a single arm study (SWISH; N = 92) in postmenopausal women with hormone receptor-positive, HER2-negative breast cancer beginning AFINITOR (10 mg orally once daily) in combination with exemestane (25 mg orally once daily), patients started dexamethasone 0.5 mg/5mL alcohol-free mouthwash (10 mL swished for 2 minutes and spat, 4 times daily for 8 weeks) concurrently with AFINITOR and exemestane. (…)<br><br>**8 USE IN SPECIFIC POPULATIONS**<br><u>Other Indications</u><br>The safety and effectiveness of AFINITOR/AFINITOR DISPERZ in pediatric patients have not been established in:<br>· Hormone receptor-positive, HER2-negative breast cancer (…)<br><br>**12 CLINICAL PHARMACOLOGY**<br>**12.3 Pharmacokinetics**<br>(…) The coadministration of AFINITOR with exemestane increased exemestane Cmin by 45% and C2h by 64%; however, the corresponding estradiol levels at steady state (4 weeks) were not different between the 2 treatment arms. No increase in adverse reactions related to exemestane was observed in patients with hormone receptor-positive, HER2-negative advanced breast cancer receiving the combination. (…)<br><br>**14 CLINICAL STUDIES**<br>**14.1 Hormone Receptor-Positive, HER2-Negative Breast Cancer**<br>A randomized, double-blind, multicenter study of AFINITOR plus exemestane versus placebo plus exemestane was conducted in 724 postmenopausal women with estrogen receptor-positive, HER 2/neu-negative advanced breast cancer with recurrence or progression following prior therapy with letrozole or anastrozole. (See Table 20 and Figure 1) (…)
**022334, 02/13/2020**	Everolimus (2)	Oncology	ESR (Hormone Receptor)	Indications and Usage, Dosage and Administration, Warnings and Precautions, Adverse Reactions, Use in Specific Populations, Clinical Pharmacology, Clinical Studies	**1 INDICATIONS AND USAGE**<br>**1.1 Hormone Receptor-Positive, HER2-Negative Breast Cancer**<br>AFINITOR® is indicated for the treatment of postmenopausal women with advanced hormone receptor-positive, HER2- negative breast cancer (advanced HR+ BC) in combination with exemestane, after failure of treatment with letrozole or anastrozole.<br><br>**2 DOSAGE AND ADMINISTRATION**<br>2.2 Recommended Dosage for Hormone Receptor-Positive, HER2-Negative Breast Cancer<br>The recommended dosage of AFINITOR is 10 mg orally once daily until disease progression or unacceptable toxicity.<br><br>**5 WARNINGS AND PRECAUTIONS**<br>5.8 Geriatric Patients<br>In the randomized hormone receptor-positive, HER2-negative breast cancer study (BOLERO-2), the incidence of deaths due to any cause within 28 days of the last AFINITOR dose was 6% in patients ≥ 65 years of age compared to 2% in patients < 65 years of age. Adverse reactions leading to permanent treatment discontinuation occurred in 33% of patients ≥ 65 years of age compared to 17% in patients < 65 years of age. Careful monitoring and appropriate dose adjustments for adverse reactions are recommended [see Dosage and Administration (2.9), Use in Specific Populations (8.5)].

* Therapeutic areas do not necessarily reflect the CDER review division.
† Representative biomarkers are listed based on standard nomenclature as per the Human Genome Organization (HUGO) symbol and/or simplified descriptors using other common conventions. Listed biomarkers do not necessarily reflect the terminology used in labeling. The term "Nonspecific" is provided when labeling does not explicitly identify the specific biomarker(s) or when the biomarker is represented by a molecular phenotype or gene signature, and in some cases the biomarker was inferred based on the labeling language.
‡ Referenced figures and tables may be found in the labeling available at Drugs@FDA.
Blue text represents the most recent addtions and/or changes since last posted version.

NDA/ANDA/BLA Number, Label Version Date	Drug	Therapeutic Area*	Biomarker†	Labeling Sections	Labeling Text‡
					**6 ADVERSE REACTIONS**<br>**6.1 Clinical Study Experience**<br>Hormone Receptor-Positive, HER2 Negative Breast Cancer<br>The safety of AFINITOR (10 mg orally once daily) in combination with exemestane (25 mg orally once daily) (n = 485) vs. placebo in combination with exemestane (n = 239) was evaluated in a randomized, controlled trial (BOLERO-2) in patients with advanced or metastatic hormone receptor-positive, HER2-negative breast cancer. The median age of patients was 61 years (28 to 93 years), and 75% were White. The median follow-up was approximately 13 months. (See Tables 6 and 7) (…)<br>Topical Prophylaxis for Stomatitis<br>In a single arm study (SWISH; N = 92) in postmenopausal women with hormone receptor-positive, HER2-negative breast cancer beginning AFINITOR (10 mg orally once daily) in combination with exemestane (25 mg orally once daily), patients started dexamethasone 0.5 mg/5mL alcohol-free mouthwash (10 mL swished for 2 minutes and spat, 4 times daily for 8 weeks) concurrently with AFINITOR and exemestane. (…)<br><br>**8 USE IN SPECIFIC POPULATIONS**<br>Other Indications<br>The safety and effectiveness of AFINITOR/AFINITOR DISPERZ in pediatric patients have not been established in:<br>• Hormone receptor-positive, HER2-negative breast cancer (…)<br>**8.5 Geriatric Use**<br>In the randomized advanced hormone receptor positive, HER2-negative breast cancer study, 40% of AFINITOR-treated patients were ≥ 65 years of age, while 15% were 75 years and over. No overall differences in effectiveness were observed between elderly and younger patients. (…)<br><br>**12 CLINICAL PHARMACOLOGY**<br>**12.3 Pharmacokinetics**<br>(…) The coadministration of AFINITOR with exemestane increased exemestane Cmin by 45% and C2h by 64%; however, the corresponding estradiol levels at steady state (4 weeks) were not different between the 2 treatment arms. No increase in adverse reactions related to exemestane was observed in patients with hormone receptor-positive, HER2-negative advanced breast cancer receiving the combination. (…)<br><br>**14 CLINICAL STUDIES**<br>**14.1 Hormone Receptor-Positive, HER2-Negative Breast Cancer**<br>A randomized, double-blind, multicenter study of AFINITOR plus exemestane versus placebo plus exemestane was conducted in 724 postmenopausal women with estrogen receptor-positive, HER 2/neu-negative advanced breast cancer with recurrence or progression following prior therapy with letrozole or anastrozole. (See Table 20 and Figure 1) (…)
**02/11/2021, 761181**	Evinacumab-dgnb (1)	Endocrinology	LDLR	Clinical Studies	**14 CLINICAL STUDIES**<br>Study ELIPSE-HoFH (NCT03399786) was a multicenter, double-blind, randomized, placebocontrolled trial evaluating the efficacy and safety of EVKEEZA compared to placebo in 65 patients with HoFH. During the 24-week, double-blind treatment period, 43 patients were randomized to receive EVKEEZA 15 mg/kg IV every 4 weeks and 22 patients to receive placebo. After the double-blind treatment period, 64 of 65 patients entered a 24-week open-label extension period in which all patients received EVKEEZA 15 mg/kg IV every 4 weeks.<br>Patients were on a background of other lipid-lowering therapies, including maximally tolerated statins, ezetimibe, PCSK9 inhibitor antibodies, lomitapide, and lipoprotein apheresis. Enrolment was stratified by apheresis status and geographical region. The diagnosis of HoFH was determined by genetic testing or by the presence of the following clinical criteria: history of an untreated total cholesterol (TC) >500 mg/dL and either xanthoma before 10 years of age or evidence of TC >250 mg/dL in both parents. In this trial, 40% (26 of 65) patients had limited LDL receptor (LDLR) function, defined by either <15% receptor function by in vitro assays or by genetic variants likely to result in minimal to no LDLR function by mutation analysis. (See Table 2 and Figure 1)<br>At Week 24, the observed reduction in LDL-C with EVKEEZA was similar across predefined subgroups, including age, sex, limited LDLR activity, concomitant treatment with lipoprotein apheresis, and concomitant background lipid-lowering medications (statins, ezetimibe, PCSK9 inhibitor antibodies, and lomitapide).<br>Pediatric Patients with HoFH<br>In ELIPSE-HoFH, 1 pediatric patient received 15 mg/kg IV of EVKEEZA every 4 weeks, and 1 pediatric patient received placebo, as an adjunct to other lipid-lowering therapies (e.g., statins, ezetimibe, PCSK9 inhibitor antibodies and lipoprotein apheresis). Both patients had null/null variants in the LDLR. At Week 24, the percent change in LDL-C with EVKEEZA was −73% and with placebo was +60%.
**02/11/2021, 761181**	Evinacumab-dgnb (2)	Endocrinology	Nonspecific (Homozygous Familial Hypercholesterolemia)	Indication and Usage, Adverse Reactions, Use in Specific Populations, Clinical Pharmacology, Clinical Studies	**1 INDICATIONS AND USAGE**<br>EVKEEZA is indicated as an adjunct to other low-density lipoprotein-cholesterol (LDL-C) lowering therapies for the treatment of adult and pediatric patients, aged 12 years and older, with homozygous familial hypercholesterolemia (HoFH).<br>Limitations of Use:<br>• The safety and effectiveness of EVKEEZA have not been established in patients with other causes of hypercholesterolemia, including those with heterozygous familial hypercholesterolemia (HeFH).<br>• The effects of EVKEEZA on cardiovascular morbidity and mortality have not been determined.<br><br>**6 ADVERSE REACTIONS**<br>**6.1 Clinical Trials Experience**<br>Safety data are based on pooled results from two randomized, double-blind, placebo-controlled trials that included 81 patients treated with EVKEEZA. The mean age of EVKEEZA-treated patients was 48 years (range: 15 to 75 years), 52% were women, 5% were Hispanic, 82% were White, 7% Asian, 3% Black,

* Therapeutic areas do not necessarily reflect the CDER review division.
† Representative biomarkers are listed based on standard nomenclature as per the Human Genome Organization (HUGO) symbol and/or simplified descriptors using other common conventions. Listed biomarkers do not necessarily reflect the terminology used in labeling. The term “Nonspecific” is provided when labeling does not explicitly identify the specific biomarker(s) or when the biomarker is represented by a molecular phenotype or gene signature, and in some cases the biomarker was inferred based on the labeling language.
‡ Referenced figures and tables may be found in the labeling available at Drugs@FDA.
Blue text represents the most recent addtions and/or changes since last posted version.

NDA/ANDA/BLA Number, Label Version Date	Drug	Therapeutic Area*	Biomarker†	Labeling Sections	Labeling Text‡
					and 9% Other. Forty-four (54%) EVKEEZA-treated patients had HoFH. Patients received EVKEEZA as add-on therapy to other lipid-lowering therapies, including maximally tolerated statin, ezetimibe, PCSK9 inhibitors, lomitapide, and apheresis. (…)<br><br>**8 USE IN SPECIFIC POPULATIONS**<br>**8.4 Pediatric Use**<br>The safety and effectiveness of EVKEEZA as an adjunct to other LDL-C-lowering therapies for the treatment of HoFH have been established in pediatric patients aged 12 years and older. Use of EVKEEZA for this indication is supported by evidence from adequate and well-controlled trials in adults with additional efficacy and safety data in pediatric patients aged 12 years and older [see Adverse Reactions (6.1) and Clinical Studies (14)]. The safety and effectiveness of EVKEEZA have not been established in pediatric patients with HoFH who are younger than 12 years old.<br><br>**12 CLINICAL PHARMACOLOGY**<br>**12.2 Pharmacodynamics**<br>Administration of evinacumab-dgnb in HoFH patients resulted in reductions in LDL-C, total cholesterol (TC), HDL-C, apolipoprotein B and TG [see Clinical Studies (14)].<br>**12.3 Pharmacokinetics**<br>The pharmacokinetic parameters described in this section are presented following administration of evinacumab-dgnb 15 mg/kg intravenously every 4 weeks, unless otherwise specified. Steady-state is reached after 4 doses, and the accumulation ratio is 2. According to population pharmacokinetic modeling, the mean (standard deviation) steady-state trough concentration is 241 (96.5) mg/L, whereas the mean (standard deviation) Cmax at the end of infusion is 689 (157) mg/L. Due to non-linear clearance, a 4.3-fold increase in area under the concentration-time curve at steady-state (AUCtau.ss) for a 3-fold increase in evinacumab-dgnb dose up to 15 mg/kg IV every 4 weeks was predicted in patients with HoFH.<br><u>Specific Populations</u><br>A population PK analysis conducted on data from 183 healthy subjects and 95 patients with HoFH suggests that the following factors have no clinically significant effect on the exposure of evinacumab-dgnb: age (12 to 75 years), gender, body weight (42 to 152 kg), and race (White, Asian, Black, and Other).<br>*Pediatric Patients*<br>A 15-year-old patient with HoFH received evinacumab-dgnb at 15 mg/kg IV every 4 weeks. Steady-state trough and end-of-infusion concentrations were within the range observed in adult patients.<br><u>Drug Interaction</u><br>Studies Drug interaction studies have not been conducted with evinacumab-dgnb. In a clinical trial, the concentrations of statins (atorvastatin, rosuvastatin, simvastatin) were not meaningfully altered in patients taking statins prior to and post administration of evinacumab-dgnb. Concentrations of evinacumab-dgnb were comparable in patients with HoFH taking or not taking background lipidlowering therapy.<br><br>**14 CLINICAL STUDIES**<br>Study ELIPSE-HoFH (NCT03399786) was a multicenter, double-blind, randomized, placebocontrolled trial evaluating the efficacy and safety of EVKEEZA compared to placebo in 65 patients with HoFH. During the 24-week, double-blind treatment period, 43 patients were randomized to receive EVKEEZA 15 mg/kg IV every 4 weeks and 22 patients to receive placebo. After the double-blind treatment period, 64 of 65 patients entered a 24-week open-label extension period in which all patients received EVKEEZA 15 mg/kg IV every 4 weeks.<br>Patients were on a background of other lipid-lowering therapies, including maximally tolerated statins, ezetimibe, PCSK9 inhibitor antibodies, lomitapide, and lipoprotein apheresis. Enrolment was stratified by apheresis status and geographical region. The diagnosis of HoFH was determined by genetic testing or by the presence of the following clinical criteria: history of an untreated total cholesterol (TC) >500 mg/dL and either xanthoma before 10 years of age or evidence of TC >250 mg/dL in both parents. In this trial, 40% (26 of 65) patients had limited LDL receptor (LDLR) function, defined by either <15% receptor function by in vitro assays or by genetic variants likely to result in minimal to no LDLR function by mutation analysis. (See Table 2 and Figure 1)<br><u>Pediatric Patients with HoFH</u><br>In ELIPSE-HoFH, 1 pediatric patient received 15 mg/kg IV of EVKEEZA every 4 weeks, and 1 pediatric patient received placebo, as an adjunct to other lipid-lowering therapies (e.g., statins, ezetimibe, PCSK9 inhibitor antibodies and lipoprotein apheresis). Both patients had null/null variants in the LDLR. At Week 24, the percent change in LDL-C with EVKEEZA was −73% and with placebo was +60%. In an open-label extension study, 13 pediatric patients with HoFH (12 to 17 years of age) received 15 mg/kg IV of EVKEEZA every 4 weeks as an adjunct to other lipid-lowering therapies (e.g., statins, ezetimibe, PCSK9 inhibitor antibodies and lipoprotein apheresis) for a median treatment duration of 33 weeks. The mean percent change from baseline in LDL-C at Week 24 was −52% in the 9 patients who completed treatment and had a lipid assessment at Week 24. Overall, the effect of evinacumab-dgnb on lipid parameters in pediatric patients with HoFH was generally similar to that seen in adults with HoFH.
125522, 08/6/2022	Evolocumab (1)	Endocrinology	Nonspecific (Heterozygous Familial Hypercholesterolemia)	Indications and Usage, Dosage and Administration, Adverse Reactions, Use in Specific Populations, Clinical Pharmacology, Clinical Studies	**1 INDICATIONS AND USAGE**<br>REPATHA is indicated:<br>(…) • As an adjunct to diet, alone or in combination with other low-density lipoprotein cholesterol (LDL-C)-lowering therapies, in adults with primary hyperlipidemia, including heterozygous familial hypercholesterolemia (HeFH), to reduce LDL-C<br>• As an adjunct to diet and other LDL-C-lowering therapies in pediatric patients aged 10 years and older with HeFH, to reduce LDL-C<br><br>**2 DOSAGE AND ADMINISTRATION**<br>**2.1 Recommended Dosage**<br>• In pediatric patients aged 10 years and older with HeFH:<br>o The recommended dosage of REPATHA is either 140 mg every 2 weeks OR 420 mg once monthly administered subcutaneously [see Dosage and Administration (2.3)].

* Therapeutic areas do not necessarily reflect the CDER review division.
† Representative biomarkers are listed based on standard nomenclature as per the Human Genome Organization (HUGO) symbol and/or simplified descriptors using other common conventions. Listed biomarkers do not necessarily reflect the terminology used in labeling. The term “Nonspecific” is provided when labeling does not explicitly identify the specific biomarker(s) or when the biomarker is represented by a molecular phenotype or gene signature, and in some cases the biomarker was inferred based on the labeling language.
‡ Referenced figures and tables may be found in the labeling available at Drugs@FDA.
Blue text represents the most recent addtions and/or changes since last posted version.

NDA/ANDA/BLA Number, Label Version Date	Drug	Therapeutic Area*	Biomarker†	Labeling Sections	Labeling Text‡
					o If switching dosage regimens, administer the first dose of the new regimen on the next scheduled date of the prior regimen.<br><br>**6 ADVERSE REACTIONS**<br><u>Adverse Reactions in Pediatric Patients with HeFH</u><br>In a 24-week, randomized, placebo-controlled, double-blind trial of 157 pediatric patients with HeFH, 104 patients received 420 mg REPATHA subcutaneously once monthly [see Clinical Studies (14)]. The mean age was 13.7 years (range: 10 to 17 years), 56% were female, 85% White, 1% Black, 1% Asian, and 13% other; 8% identified as Hispanic ethnicity. Common adverse reactions (> 5% of patients treated with REPATHA and occurring more frequently than placebo) included:<br>• Nasopharyngitis (12% versus 11%)<br>• Headache (11% versus 2%)<br>• Oropharyngeal pain (7% versus 0%)<br>• Influenza (6% versus 4%)<br>• Upper respiratory tract infection (6% versus 2%)<br><br>**8 USE IN SPECIFIC POPULATIONS**<br>**8.4 Pediatric Use**<br>The safety and effectiveness of REPATHA as an adjunct to diet and other LDL-C-lowering therapies for the treatment of HeFH have been established in pediatric patients aged 10 years and older. Use of REPATHA for this indication is based on data from a 24-week, randomized, placebo-controlled, double-blind trial in pediatric patients with HeFH. In the trial, 104 patients received REPATHA 420 mg subcutaneously once monthly and 53 patients received placebo; 39 patients (25%) were 10 to 11 years of age [see Adverse Reactions (6.1) and Clinical Studies (14)].<br>The safety and effectiveness of REPATHA have not been established in pediatric patients with HeFH or HoFH who are younger than 10 years old or in pediatric patients with other types of hyperlipidemia.<br><br>**12 CLINICAL PHARMACOLOGY**<br>**12.3 Pharmacokinetics**<br><u>Specific Populations</u><br>Pediatric Patients<br>The pharmacokinetics of REPATHA were evaluated in 103 pediatric patients aged 10 to 17 years with HeFH (Study 6) [see Use in Specific Populations (8.4), Clinical Studies (14)]. Following subcutaneous administration of 420 mg REPATHA once monthly, mean trough serum concentrations were 22.4 mcg/mL and 25.8 mcg/mL over the Week 12 and Week 24 time points, respectively. The pharmacokinetics of REPATHA were evaluated in 12 pediatric patients aged 11 to 17 years with HoFH (Study 9) [see Use in Specific Populations (8.4), Clinical Studies (14)]. Following subcutaneous administration of 420 mg REPATHA once monthly, mean serum trough concentrations were 20.3 mcg/mL and 17.6 mcg/mL at Week 12 and Week 80, respectively.<br><br>**14 CLINICAL STUDIES**<br><u>Primary Hyperlipidemia</u><br>Study 5 (RUTHERFORD-2, NCT01763918) was a multicenter, double-blind, randomized, placebo-controlled, 12-week trial in 329 patients with HeFH on statins with or without other lipid-lowering therapies. Patients were randomized to receive subcutaneous injections of REPATHA 140 mg every two weeks, 420 mg once monthly, or placebo. HeFH was diagnosed by the Simon Broome criteria (1991). In Study 5, 38% of patients had clinical atherosclerotic cardiovascular disease. The mean age at baseline was 51 years (range: 19 to 79 years), 15% of the patients were ≥ 65 years old, 42% were women, 90% were White, 5% were Asian, and 1% were Black. The average LDL-C at baseline was 156 mg/dL with 76% of the patients on high-intensity statin therapy. (see Table 7 and Figure 5) (…)<br><u>Pediatric Patients with HeFH</u><br>Study 6 (HAUSER-RCT, NCT02392559) was a randomized, multicenter, placebo-controlled, double-blind, 24-week trial in 157 pediatric patients aged 10 to 17 years with HeFH [see Use in Specific Populations (8.4)]. HeFH was diagnosed by diagnostic criteria for HeFH [Simon Broome Register Group (1991), the Dutch Lipid Clinic Network (1999), MEDPED (1993)] or by genetic testing. Patients were required to be on a low-fat diet and optimized background lipid-lowering therapy. Patients were randomly assigned 2:1 to receive 24 weeks of subcutaneous once monthly 420 mg REPATHA or placebo; 104 patients received REPATHA and 53 patients received placebo. The mean age was 14 years (range: 10 to 17 years), 56% were female, 85% White, 1% Black, 1% Asian, 13% Other, and 8% Hispanic. The mean LDL-C at baseline was 184 mg/dL; 17% of patients were on high-intensity statin, 62% on moderateintensity statin, and 13% on ezetimibe. (see Figure 6 and Table 8)<br><u>Adults and Pediatric Patients with HoFH</u><br>Study 7 (TESLA, NCT01588496) was a multicenter, double-blind, randomized, placebo-controlled, 12-week trial in 49 patients (not on lipid-apheresis therapy) with HoFH. In this trial, 33 patients received subcutaneous injections of 420 mg of REPATHA once monthly and 16 patients received placebo as an adjunct to other lipid-lowering therapies (e.g., statins, ezetimibe). The mean age at baseline was 31 years, 49% were women, 90% White, 4% were Asian, and 6% other. The trial included 10 adolescents (ages 13 to 17 years), 7 of whom received REPATHA. The mean LDL-C at baseline was 349 mg/dL with all patients on statins (atorvastatin or rosuvastatin) and 92% on ezetimibe. The diagnosis of HoFH was made by genetic confirmation or a clinical diagnosis based on a history of an untreated LDL-C concentration > 500 mg/dL together with either xanthoma before 10 years of age or evidence of HeFH in both parents. (…)
125522, 08/6/2022	Evolocumab (2)	Endocrinology	Nonspecific (Homozygous Familial Hypercholesterolemia	Indications and Usage, Dosage and Administration, Adverse	**1 INDICATIONS AND USAGE**<br>REPATHA is indicated:<br>(…) • As an adjunct to other LDL-C-lowering therapies in adults and pediatric patients aged 10 years and older with homozygous familial hypercholesterolemia (HoFH), to reduce LDL-C

* Therapeutic areas do not necessarily reflect the CDER review division.
† Representative biomarkers are listed based on standard nomenclature as per the Human Genome Organization (HUGO) symbol and/or simplified descriptors using other common conventions. Listed biomarkers do not necessarily reflect the terminology used in labeling. The term “Nonspecific” is provided when labeling does not explicitly identify the specific biomarker(s) or when the biomarker is represented by a molecular phenotype or gene signature, and in some cases the biomarker was inferred based on the labeling language.
‡ Referenced figures and tables may be found in the labeling available at Drugs@FDA.
Blue text represents the most recent addtions and/or changes since last posted version.

NDA/ANDA/BLA Number, Label Version Date	Drug	Therapeutic Area*	Biomarker†	Labeling Sections	Labeling Text‡
				Reactions, Use in Specific Populations, Clinical Pharmacology, Clinical Studies	**2 DOSAGE AND ADMINISTRATION**<br>**2.1 Recommended Dosage**<br>• In adults and pediatric patients aged 10 years and older with HoFH:<br>o The initial recommended dosage of REPATHA is 420 mg once monthly administered subcutaneously [see Dosage and Administration (2.3)].<br>o The dosage can be increased to 420 mg every 2 weeks if a clinically meaningful response is not achieved in 12 weeks. o Patients on lipid apheresis may initiate treatment with 420 mg every 2 weeks to correspond with their apheresis schedule. Administer REPATHA after the apheresis session is complete.<br><br>**6 ADVERSE REACTIONS**<br><u>Adverse Reactions in Adults and Pediatric Patients with HoFH</u><br>In a 12-week, double-blind, randomized, placebo-controlled trial of 49 patients with HoFH, 33 patients received 420 mg of REPATHA subcutaneously once monthly [see Clinical Studies (14)]. The mean age was 31 years (range: 13 to 57 years), 49% were women, 90% White, 4% Asian, and 6% other. The adverse reactions that occurred in at least two (6.1%) REPATHA-treated patients, and more frequently than in placebo-treated patients, included:<br>• Upper respiratory tract infection (9.1% versus 6.3%)<br>• Influenza (9.1% versus 0%)<br>• Gastroenteritis (6.1% versus 0%)<br>• Nasopharyngitis (6.1% versus 0%)<br>In a multicenter, open-label 5-year extension study, 106 patients with HoFH, including 14 pediatric patients, received 420 mg of REPATHA subcutaneously once monthly or every 2 weeks [see Clinical Studies (14)]. The mean age was 34 years (range: 13 to 68 years), 51% were women, 80% White, 12% Asian, 1% Native American, and 7% other; 5% identified as Hispanic ethnicity. No new adverse reactions were observed during the open-label extension study.<br><br>**8 USE IN SPECIFIC POPULATIONS**<br>**8.4 Pediatric Use**<br>The safety and effectiveness of REPATHA in combination with diet and other LDL-C-lowering therapies for the treatment of HoFH have been established in pediatric patients aged 10 years and older. Use of REPATHA for this indication is supported by evidence from an adequate and well-controlled trial in adults and pediatric patients aged 13 years and older with HoFH (including 7 pediatric patients treated with REPATHA) and from open-label studies which included an additional 19 pediatric patients aged 11 years and older with HoFH not previously treated with REPATHA [see Adverse Reactions (6.1) and Clinical Studies (14)].<br>The safety and effectiveness of REPATHA have not been established in pediatric patients with HeFH or HoFH who are younger than 10 years old or in pediatric patients with other types of hyperlipidemia.<br><br>**12 CLINICAL PHARMACOLOGY**<br>**12.3 Pharmacokinetics**<br><u>Specific Populations</u><br>Pediatric Patients<br>The pharmacokinetics of REPATHA were evaluated in 103 pediatric patients aged 10 to 17 years with HeFH (Study 6) [see Use in Specific Populations (8.4), Clinical Studies (14)]. Following subcutaneous administration of 420 mg REPATHA once monthly, mean trough serum concentrations were 22.4 mcg/mL and 25.8 mcg/mL over the Week 12 and Week 24 time points, respectively. The pharmacokinetics of REPATHA were evaluated in 12 pediatric patients aged 11 to 17 years with HoFH (Study 9) [see Use in Specific Populations (8.4), Clinical Studies (14)]. Following subcutaneous administration of 420 mg REPATHA once monthly, mean serum trough concentrations were 20.3 mcg/mL and 17.6 mcg/mL at Week 12 and Week 80, respectively.<br><br>**14 CLINICAL STUDIES**<br><u>Adults and Pediatric Patients with HoFH</u><br>Study 7 (TESLA, NCT01588496) was a multicenter, double-blind, randomized, placebo-controlled, 12-week trial in 49 patients (not on lipid-apheresis therapy) with HoFH. In this trial, 33 patients received subcutaneous injections of 420 mg of REPATHA once monthly and 16 patients received placebo as an adjunct to other lipid-lowering therapies (e.g., statins, ezetimibe). The mean age at baseline was 31 years, 49% were women, 90% White, 4% were Asian, and 6% other. The trial included 10 adolescents (ages 13 to 17 years), 7 of whom received REPATHA. The mean LDL-C at baseline was 349 mg/dL with all patients on statins (atorvastatin or rosuvastatin) and 92% on ezetimibe. The diagnosis of HoFH was made by genetic confirmation or a clinical diagnosis based on a history of an untreated LDL-C concentration > 500 mg/dL together with either xanthoma before 10 years of age or evidence of HeFH in both parents. (see Table 9) (…)<br>Study 8 (TAUSSIG, NCT01624142) was a multicenter, open-label 5-year extension study with REPATHA in 106 patients with HoFH, who were treated with REPATHA as an adjunct to other lipid-lowering therapies. The study included 14 pediatric patients (ages 13 to 17 years). All patients in the study were initially treated with REPATHA 420 mg once monthly except for those receiving lipid apheresis at enrollment, who began with REPATHA 420 mg every 2 weeks. Dose frequency in non-apheresis patients could be titrated up to 420 mg once every 2 weeks based on LDL-C response and PCSK9 levels.<br>A total of 48 patients with HoFH received REPATHA 420 mg once monthly for at least 12 weeks in Study 8 followed by REPATHA 420 mg every 2 weeks for at least 12 weeks. Mean percent change from baseline in LDL-C were −20% at Week 12 of 420 mg once monthly treatment and −30% at Week 12 of 420 mg every 2 weeks treatment, based on available data.<br>Study 9 (HAUSER-OLE, NCT02624869) was an open-label, single-arm, multicenter, 80-week study to evaluate the safety, tolerability, and efficacy of REPATHA for LDL-C reduction in pediatric patients aged 10 to 17 years with HoFH [see Use in Specific Populations (8.4)]. Patients were on a low-fat diet and receiving background lipid-lowering therapy. Overall, 12 patients with HoFH received 420 mg REPATHA subcutaneously once monthly. The mean age was 12 years (range 11 to 17 years), 17% were female, 75% White, 17% Asian, and 8% Other. Median (Q1, Q3) LDL-C at baseline was 398 (343, 475) mg/dL, and all patients were on statins (atorvastatin or rosuvastatin) and ezetimibe. No patients were receiving lipid apheresis. The diagnosis of HoFH was made by genetic

* Therapeutic areas do not necessarily reflect the CDER review division.
† Representative biomarkers are listed based on standard nomenclature as per the Human Genome Organization (HUGO) symbol and/or simplified descriptors using other common conventions. Listed biomarkers do not necessarily reflect the terminology used in labeling. The term “Nonspecific” is provided when labeling does not explicitly identify the specific biomarker(s) or when the biomarker is represented by a molecular phenotype or gene signature, and in some cases the biomarker was inferred based on the labeling language.
‡ Referenced figures and tables may be found in the labeling available at Drugs@FDA.
Blue text represents the most recent addtions and/or changes since last posted version.

Table of Pharmacogenomic Biomarkers in Drug Labeling
Last Updated: 06/2024

NDA/ANDA/BLA Number, Label Version Date	Drug	Therapeutic Area*	Biomarker†	Labeling Sections	Labeling Text‡
					confirmation in all patients but enrollment by a clinical diagnosis was permitted. The median (Q1, Q3) percent change in LDL-C from baseline to Week 80 was −14% (−41, 4). Two of the 3 subjects with < 5% LDLR activity responded to evolocumab treatment.
020753, 05/18/2018	Exemestane	Oncology	ESR, PGR (Hormone Receptor)	Indications and Usage, Dosage and Administration, Clinical Studies	**1 INDICATIONS AND USAGE**<br>**1.1 Adjuvant Treatment of Postmenopausal Women**<br>AROMASIN is indicated for adjuvant treatment of postmenopausal women with estrogen-receptor positive early breast cancer who have received two to three years of tamoxifen and are switched to AROMASIN for completion of a total of five consecutive years of adjuvant hormonal therapy [see Clinical Studies (14.1)]. (…)<br><br>**2 DOSAGE AND ADMINISTRATION**<br>**2.1 Recommended Dose**<br>The recommended dose of AROMASIN in early and advanced breast cancer is one 25 mg tablet once daily after a meal.<br>• adjuvant treatment of postmenopausal women with estrogen-receptor positive early breast cancer who have received two to three years of tamoxifen and are switched to AROMASIN for completion of a total of five consecutive years of adjuvant hormonal therapy. (…)<br><br>**14 CLINICAL STUDIES**<br>**14.1 Adjuvant Treatment in Early Breast Cancer**<br>The Intergroup Exemestane Study 031 (IES) was a randomized, double-blind, multicenter, multinational study comparing exemestane (25 mg/day) vs. tamoxifen (20 or 30 mg/day) in postmenopausal women with early breast cancer. (See Table 5) (…)<br>(…) In the hormone receptor-positive subpopulation representing about 85% of the trial patients, disease-free survival was also statistically significantly improved (HR = 0.65, 95% CI: 0.53, 0.79, P = 0.00001) in the AROMASIN arm compared to the tamoxifen arm. Consistent results were observed in the subgroups of patients with node negative or positive disease, and patients who had or had not received prior chemotherapy. (See Table 9) (…)
761139, 04/05/2024	Fam-Trastuzumab Deruxtecan-nxki (1)	Oncology	ERBB2 (HER2)	Indications and Usage, Dosage and Administration, Warnings and Precautions, Adverse Reactions, Use in Specific Populations, Clinical Pharmacology, Clinical Studies	**1 INDICATIONS AND USAGE**<br>**1.1 HER2-Positive Metastatic Breast Cancer**<br>ENHERTU is indicated for the treatment of adult patients with unresectable or metastatic HER2-positive (IHC 3+ or ISH positive) breast cancer who have received a prior anti-HER2-based regimen either:<br>• in the metastatic setting, or<br>• in the neoadjuvant or adjuvant setting and have developed disease recurrence during or within six months of completing therapy.<br>**1.2 HER2-Low Metastatic Breast Cancer**<br>ENHERTU is indicated for the treatment of adult patients with unresectable or metastatic HER2-low (IHC 1+ or IHC 2+/ISH-) breast cancer, as determined by an FDA-approved test, who have received a prior chemotherapy in the metastatic setting or developed disease recurrence during or within 6 months of completing adjuvant chemotherapy [see Dosage and Administration (2.1)].<br>**1.3 HER2-Mutant Unresectable or Metastatic Non-Small Cell Lung Cancer**<br>ENHERTU is indicated for the treatment of adult patients with unresectable or metastatic non-small cell lung cancer (NSCLC) whose tumors have activating HER2 (ERBB2) mutations, as detected by an FDA-approved test, and who have received a prior systemic therapy.<br>This indication is approved under accelerated approval based on objective response rate and duration of response [see Clinical Studies (14.3)]. Continued approval for this indication may be contingent upon verification and description of clinical benefit in a confirmatory trial.<br>**1.4 HER2-Positive Locally Advanced or Metastatic Gastric Cancer**<br>ENHERTU is indicated for the treatment of adult patients with locally advanced or metastatic HER2-positive (IHC 3+ or IHC 2+/ISH positive) gastric or gastroesophageal junction (GEJ) adenocarcinoma who have received a prior trastuzumab-based regimen.<br>**1.5 HER2-Positive (IHC 3+) Unresectable or Metastatic Solid Tumors**<br>ENHERTU is indicated for the treatment of adult patients with unresectable or metastatic HER2-positive (IHC 3+) solid tumors who have received prior systemic treatment and have no satisfactory alternative treatment options.<br>This indication is approved under accelerated approval based on objective response rate and duration of response [see Clinical Studies (14.5)]. Continued approval for this indication may be contingent upon verification and description of clinical benefit in a confirmatory trial.<br><br>**2 DOSAGE AND ADMINISTRATION**<br>**2.1 Patient Selection**<br>HER2-Low Unresectable or Metastatic Breast Cancer<br>Select patients for treatment of unresectable or metastatic HER2-low breast cancer with ENHERTU based on HER2 expression (IHC 1+ or IHC 2+/ISH-) [see Clinical Studies (14.2)].<br>HER2-Mutant Unresectable or Metastatic NSCLC<br>Select patients for the treatment of unresectable or metastatic HER2-mutant NSCLC with ENHERTU based on the presence of activating HER2 (ERBB2) mutations in tumor or plasma specimens [see Clinical Studies (14.3)]. If no mutation is detected in a plasma specimen, test tumor tissue.<br>HER2-Positive Locally Advanced or Metastatic Gastric Cancer<br>Select patients with locally advanced or metastatic HER2-positive gastric cancer based on HER2 protein overexpression or HER2 gene amplification (IHC 3+ or IHC 2+/ISH positive). Reassess HER2 status if it is feasible to obtain a new tumor specimen after prior trastuzumab-based therapy and before treatment with ENHERTU.<br>HER2-Positive (IHC 3+) Unresectable or Metastatic Solid Tumors

* Therapeutic areas do not necessarily reflect the CDER review division.
† Representative biomarkers are listed based on standard nomenclature as per the Human Genome Organization (HUGO) symbol and/or simplified descriptors using other common conventions. Listed biomarkers do not necessarily reflect the terminology used in labeling. The term “Nonspecific” is provided when labeling does not explicitly identify the specific biomarker(s) or when the biomarker is represented by a molecular phenotype or gene signature, and in some cases the biomarker was inferred based on the labeling language.
‡ Referenced figures and tables may be found in the labeling available at Drugs@FDA.
Blue text represents the most recent addtions and/or changes since last posted version.

NDA/ANDA/BLA Number, Label Version Date	Drug	Therapeutic Area*	Biomarker†	Labeling Sections	Labeling Text‡
					Select patients for treatment of unresectable or metastatic solid tumors with ENHERTU based on HER2-positive (IHC 3+) specimens [see Clinical Studies (14.5)]. An FDA-approved test for the detection of HER2-positive (IHC 3+) solid tumors for treatment with ENHERTU is not currently available.<br>Additional Patient Selection Information<br>Information on FDA-approved tests for the detection of HER2 protein expression, HER2 gene amplification, and activating HER2 mutations is available at: http://www.fda.gov/CompanionDiagnostics.<br><br>**2.2 Recommended Dosage and Schedules**<br>Recommended Dosage for HER2-Positive or HER2-Low Metastatic Breast Cancer, HER2-Mutant Unresectable or Metastatic NSCLC, and HER2-Positive (IHC 3+) Unresectable or Metastatic Solid Tumors.<br>The recommended dosage of ENHERTU is 5.4 mg/kg given as an intravenous infusion once every 3 weeks (21-day cycle) until disease progression or unacceptable toxicity.<br>Recommended Dosage for HER2-Positive Locally Advanced or Metastatic Gastric Cancer<br>The recommended dosage of ENHERTU is 6.4 mg/kg given as an intravenous infusion once every 3 weeks (21-day cycle) until disease progression or unacceptable toxicity.<br><br>**5 WARNINGS AND PRECAUTIONS**<br>**5.1 Interstitial Lung Disease/Pneumonitis**<br>Severe, life-threatening, or fatal interstitial lung disease (ILD), including pneumonitis, can occur in patients treated with ENHERTU [see Adverse Reactions (6.1)]. A higher incidence of Grade 1 and 2 ILD/pneumonitis has been observed in patients with moderate renal impairment.<br>Advise patients to immediately report cough, dyspnea, fever, and/or any new or worsening respiratory symptoms. Monitor patients for signs and symptoms of ILD. Promptly investigate evidence of ILD. Evaluate patients with suspected ILD by radiographic imaging. Consider consultation with a pulmonologist. For asymptomatic (Grade 1) ILD, consider corticosteroid treatment (e.g., ≥0.5 mg/kg/day prednisolone or equivalent). Withhold ENHERTU until recovery [see Dosage and Administration (2.3)]. In cases of symptomatic ILD (Grade 2 or greater), promptly initiate systemic corticosteroid treatment (e.g., ≥1 mg/kg/day prednisolone or equivalent) and continue for at least 14 days followed by gradual taper for at least 4 weeks. Permanently discontinue ENHERTU in patients who are diagnosed with symptomatic (Grade 2 or greater) ILD [see Dosage and Administration (2.3)].<br>HER2-Positive or HER2-Low Metastatic Breast Cancer, HER2-Mutant NSCLC, and Solid Tumors (Including IHC 3+) (5.4 mg/kg)<br>In patients with metastatic breast cancer, HER2-mutant NSCLC, and other solid tumors treated with ENHERTU 5.4 mg/kg, ILD occurred in 12% of patients. Median time to first onset was 5.5 months (range: 0.9 to 31.5). Fatal outcomes due to ILD and/or pneumonitis occurred in 1.0% of patients treated with ENHERTU.<br>HER2-Positive Locally Advanced or Metastatic Gastric Cancer (6.4 mg/kg)<br>In patients with locally advanced or metastatic HER2-positive gastric or GEJ adenocarcinoma treated with ENHERTU 6.4 mg/kg, ILD occurred in 10% of patients. Median time to first onset was 2.8 months (range: 1.2 to 21).<br>**5.2 Neutropenia**<br>Severe neutropenia, including febrile neutropenia, can occur in patients treated with ENHERTU.<br>Monitor complete blood counts prior to initiation of ENHERTU and prior to each dose, and as clinically indicated. Based on the severity of neutropenia, ENHERTU may require dose interruption or reduction [see Dosage and Administration (2.3)].<br>HER2-Positive or HER2-Low Metastatic Breast Cancer, HER2-Mutant NSCLC, and Solid Tumors (Including IHC 3+) (5.4 mg/kg)<br>In patients with metastatic breast cancer, HER2-mutant NSCLC, and other solid tumors treated with ENHERTU 5.4 mg/kg, a decrease in neutrophil count was reported in 63% of patients. Seventeen percent had Grade 3 or 4 decreased neutrophil count. Median time to first onset of decreased neutrophil count was 22 days (range: 2 to 939). Febrile neutropenia was reported in 1% of patients.<br>HER2-Positive Locally Advanced or Metastatic Gastric Cancer (6.4 mg/kg)<br>In patients with locally advanced or metastatic HER2-positive gastric or GEJ adenocarcinoma treated with ENHERTU 6.4 mg/kg, a decrease in neutrophil count was reported in 72% of patients. Fifty-one percent had Grade 3 or 4 decreased neutrophil count. Median time to first onset of decreased neutrophil count was 16 days (range: 4 to 187). Febrile neutropenia was reported in 4.8% of patients.<br>**5.3 Left Ventricular Dysfunction**<br>Patients treated with ENHERTU may be at increased risk of developing left ventricular dysfunction. Left ventricular ejection fraction (LVEF) decrease has been observed with anti-HER2 therapies, including ENHERTU.<br>Assess LVEF prior to initiation of ENHERTU and at regular intervals during treatment as clinically indicated. Manage LVEF decrease through treatment interruption. Permanently discontinue ENHERTU if LVEF of less than 40% or absolute decrease from baseline of greater than 20% is confirmed. Permanently discontinue ENHERTU in patients with symptomatic congestive heart failure (CHF) [see Dosage and Administration (2.3)].<br>Treatment with ENHERTU has not been studied in patients with a history of clinically significant cardiac disease or LVEF less than 50% prior to initiation of treatment.<br>HER2-Positive or HER2-Low Metastatic Breast Cancer, HER2-Mutant NSCLC, and Solid Tumors (Including IHC 3+) (5.4 mg/kg)<br>In patients with metastatic breast cancer, HER2-mutant NSCLC, and other solid tumors treated with ENHERTU 5.4 mg/kg, LVEF decrease was reported in 3.8% of patients, of which 0.6% were Grade 3.<br>HER2-Positive Locally Advanced or Metastatic Gastric Cancer (6.4 mg/kg)<br>In patients with locally advanced or metastatic HER2-positive gastric or GEJ adenocarcinoma treated with ENHERTU 6.4 mg/kg, no clinical adverse events of heart failure were reported; however, on echocardiography, 8% were found to have asymptomatic Grade 2 decrease in LVEF.<br><br>**6 ADVERSE REACTIONS**

NDA/ANDA/BLA Number, Label Version Date	Drug	Therapeutic Area*	Biomarker†	Labeling Sections	Labeling Text‡
					**6.1 Clinical Trials Experience**<br>HER2-Positive and HER2-Low Metastatic Breast Cancer, HER2-Mutant NSCLC, and Solid Tumors (Including IHC 3+) (5.4 mg/kg)<br>The pooled safety population described in WARNINGS and PRECAUTIONS reflects exposure to ENHERTU 5.4 mg/kg intravenously every 3 weeks in 1799 patients in Study DS8201-A-J101 (NCT02564900), DESTINY-Breast01, DESTINY-Breast02, DESTINY-Breast03, DESTINY-Breast04, DESTINY-Lung01, DESTINY-Lung02, DESTINY-CRC02, and DESTINY-PanTumor02. Among these patients, 65% were exposed for greater than 6 months and 38% were exposed for greater than 12 months. In this pooled safety population, the most common (≥20%) adverse reactions (including laboratory abnormalities) were nausea (73%), decreased white blood cell count (70%), decreased hemoglobin (66%), decreased neutrophil count (63%), decreased lymphocyte count (58%), fatigue (56%), decreased platelet count (48%), increased aspartate aminotransferase (47%), increased alanine aminotransferase (43%), vomiting (40%), increased blood alkaline phosphatase (38%), alopecia (34%), constipation (33%), decreased appetite (32%), decreased blood potassium (31%), diarrhea (29%), musculoskeletal pain (24%), and abdominal pain (20%).<br>HER2-Positive Locally Advanced or Metastatic Gastric Cancer (6.4 mg/kg)<br>The data described in WARNINGS and PRECAUTIONS reflect exposure to ENHERTU 6.4 mg/kg intravenously every 3 weeks in 125 patients in DESTINY-Gastric01.<br>HER2-Positive Metastatic Breast Cancer<br>*DESTINY-Breast03*<br>The safety of ENHERTU was evaluated in 257 patients with unresectable or metastatic HER2-positive breast cancer who received at least one dose of ENHERTU 5.4 mg/kg in DESTINY-Breast03 [see Clinical Studies (14.1)]. ENHERTU was administered by intravenous infusion once every three weeks. The median duration of treatment was 14 months (range: 0.7 to 30) for patients who received ENHERTU and 7 months (range: 0.7 to 25) for patients who received ado-trastuzumab emtansine.<br>*DESTINY-Breast02*<br>The safety of ENHERTU was evaluated in 404 patients with unresectable or metastatic HER2-positive breast cancer who received at least one dose of ENHERTU 5.4 mg/kg in DESTINY-Breast02 [see Clinical Studies (14.1)]. ENHERTU was administered by intravenous infusion once every three weeks. The median duration of treatment was 11 months (range: 0.7 to 45) for patients who received ENHERTU.<br>*DESTINY-Breast01 and Study DS8201-A-J101*<br>The safety of ENHERTU was evaluated in a pooled analysis of 234 patients with unresectable or metastatic HER2-positive breast cancer who received at least one dose of ENHERTU 5.4 mg/kg in DESTINY-Breast01 and Study DS8201-A-J101 (NCT02564900) [see Clinical Studies (14.1)]. ENHERTU was administered by intravenous infusion once every three weeks. The median duration of treatment was 7 months (range: 0.7 to 31).<br>HER2-Low Metastatic Breast Cancer<br>The safety of ENHERTU was evaluated in 371 patients with unresectable or metastatic HER2-low (IHC 1+ or IHC 2+/ISH-) breast cancer who received ENHERTU 5.4 mg/kg in DESTINY-Breast04 [see Clinical Studies (14.2)]. ENHERTU was administered by intravenous infusion once every three weeks. The median duration of treatment was 8 months (range: 0.2 to 33) for patients who received ENHERTU.<br>HER2-Mutant Unresectable or Metastatic NSCLC<br>DESTINY-Lung02 evaluated two dose levels (5.4 mg/kg [n=101] and 6.4 mg/kg [n=50]); however, only the results for the recommended dose of 5.4 mg/kg intravenously every 3 weeks are described below due to increased toxicity observed with the higher dose in patients with NSCLC, including ILD/pneumonitis.<br>HER2-Positive Locally Advanced or Metastatic Gastric Cancer<br>The safety of ENHERTU was evaluated in 187 patients with locally advanced or metastatic HER2-positive gastric or GEJ adenocarcinoma in DESTINY-Gastric01 [see Clinical Studies (14.4)]. Patients intravenously received at least one dose of either ENHERTU (N=125) 6.4 mg/kg once every three weeks or either irinotecan (N=55) 150 mg/m2 biweekly or paclitaxel (N=7) 80 mg/m2 weekly for 3 weeks. The median duration of treatment was 4.6 months (range: 0.7 to 22.3) in the ENHERTU group and 2.8 months (range: 0.5 to 13.1) in the irinotecan/paclitaxel group.<br>HER2-Positive (IHC 3+) Unresectable or Metastatic Solid Tumors<br>The safety of ENHERTU was evaluated in 347 adult patients with unresectable or metastatic HER2-positive (IHC 3+) solid tumors who received ENHERTU 5.4 mg/kg in DESTINY-Breast01, DESTINY-PanTumor02, DESTINY-Lung01, and DESTINY-CRC02 [see Clinical Studies (14.1 and 14.5)]. ENHERTU was administered by intravenous infusion once every three weeks. The median duration of treatment was 8.3 months (range 0.7 to 30.2).<br><br>**8 USE IN SPECIFIC POPULATIONS**<br>**8.5 Geriatric Use**<br>Of the 883 patients with breast cancer treated with ENHERTU 5.4 mg/kg, 22% were 65 years or older and 3.6% were 75 years or older. No overall differences in efficacy within clinical studies were observed between patients ≥65 years of age compared to younger patients. There was a higher incidence of Grade 3-4 adverse reactions observed in patients aged 65 years or older (60%) as compared to younger patients (48%).<br>Of the 101 patients with unresectable or metastatic HER2-mutant NSCLC treated with ENHERTU 5.4 mg/kg, 40% were 65 years or older and 8% were 75 years or older. No overall differences in efficacy or safety were observed between patients ≥65 years of age compared to younger patients.<br>Of the 125 patients with locally advanced or metastatic HER2-positive gastric or GEJ adenocarcinoma treated with ENHERTU 6.4 mg/kg in DESTINY-Gastric01, 56% were 65 years or older and 14% were 75 years or older. No overall differences in efficacy or safety were observed between patients ≥65 years of age compared to younger patients.<br>Of the 192 patients with HER2-positive (IHC 3+) unresectable or metastatic solid tumors treated with ENHERTU 5.4 mg/kg in DESTINY-PanTumor02, DESTINY-Lung01 or DESTINY-CRC02, 39% were 65 years or older and 9% were 75 years or older. No overall differences in efficacy or safety were observed between patients ≥65 years of age compared to younger patients.<br><br>**12 CLINICAL PHARMACOLOGY**<br>**12.2 Pharmacodynamics**

Table of Pharmacogenomic Biomarkers in Drug Labeling
Last Updated: 06/2024

NDA/ANDA/BLA Number, Label Version Date	Drug	Therapeutic Area*	Biomarker†	Labeling Sections	Labeling Text‡
					Cardiac Electrophysiology<br>The administration of multiple doses of ENHERTU 6.4 mg/kg every 3 weeks did not show a large mean effect (i.e. >20 ms) on the QTc interval in an open-label, single-arm study in 51 patients with metastatic HER2-positive cancer.<br>**12.3 Pharmacokinetics**<br>The pharmacokinetics of fam-trastuzumab deruxtecan-nxki was evaluated in patients with cancer. Following a single dose, exposures (Cmax and AUC) of fam-trastuzumab deruxtecan-nxki and released topoisomerase inhibitor (DXd) increased proportionally over a dose range of 3.2 mg/kg to 8 mg/kg (approximately 0.6 to 1.5 times the recommended dose in breast cancer, NSCLC, and HER2-positive (IHC 3+) solid tumors and 0.5 to 1.25 times the recommended dose in gastric cancer).<br>At the recommended dosage of ENHERTU for patients with metastatic breast cancer, NSCLC, and HER2-positive (IHC 3+) solid tumors, the geometric mean (coefficient of variation [CV]%) Cmax of fam-trastuzumab deruxtecan-nxki and DXd were 132 µg/mL (20%) and 4.7 ng/mL (48%), respectively, and the AUC of fam-trastuzumab deruxtecan-nxki and DXd were 772 µg·day/mL (27%) and 29 ng·day/mL (48%), respectively. Accumulation of fam-trastuzumab deruxtecan-nxki was approximately 35% at steady-state (Cycle 3).<br>At the recommended dosage of ENHERTU for patients with HER2-positive gastric cancer, the geometric mean Cmax,ss of fam-trastuzumab deruxtecan-nxki and DXd were 126 µg/mL (18%) and 5.2 ng/mL (42%), respectively, and the AUCss of fam-trastuzumab deruxtecan-nxki and DXd were 743 µg·day/mL (26%) and 33 ng·day/mL (43%), respectively. Accumulation of fam-trastuzumab deruxtecan-nxki was approximately 39% at steady-state (Cycle 3).<br><br>**14 CLINICAL STUDIES**<br>**14.1 HER2-Positive Metastatic Breast Cancer**<br>DESTINY-Breast03<br>The efficacy of ENHERTU was evaluated in study DESTINY-Breast03 (NCT03529110), a multicenter, open-label, randomized trial that enrolled 524 patients with HER2-positive, unresectable and/or metastatic breast cancer who received prior trastuzumab and taxane therapy for metastatic disease or developed disease recurrence during or within 6 months of completing adjuvant therapy. HER2 expression was based on archival tissue tested at a central laboratory prior to enrollment with HER2 positivity defined as HER2 IHC 3+ or ISH positive. (…)<br>DESTINY-Breast02<br>The efficacy of ENHERTU was evaluated in study DESTINY-Breast02 (NCT03523585), a multicenter, open-label, randomized study that enrolled 608 patients with HER2-positive, unresectable and/or metastatic breast cancer who were previously treated with ado-trastuzumab emtansine. HER2 expression was based on archival tissue tested at a central laboratory prior to enrollment with HER2 positivity defined as HER2 IHC 3+ or ISH positive. (…)<br>DESTINY-Breast01<br>The efficacy of ENHERTU was evaluated in study DESTINY-Breast01 (NCT03248492), a multicenter, single-arm, trial that enrolled 184 female patients with HER2-positive, unresectable and/or metastatic breast cancer who had received two or more prior anti-HER2 therapies. Patients were excluded for a history of treated ILD or current ILD at screening. Patients were also excluded for history of clinically significant cardiac disease, active brain metastases, and ECOG performance status >1. HER2 expression was based on archival tissue tested at a central laboratory prior to enrollment with HER2 positivity defined as HER2 IHC 3+ or ISH positive. (…)<br>**14.2 HER2-Low Metastatic Breast Cancer**<br>The efficacy of ENHERTU was evaluated in study DESTINY-Breast04 (NCT03734029), a randomized, multicenter, open-label study that enrolled 557 adult patients with unresectable or metastatic HER2-low breast cancer. The study included 2 cohorts: 494 hormone receptor-positive (HR+) patients and 63 hormone receptor-negative (HR-) patients. HER2-low expression was defined as IHC 1+ or IHC 2+/ISH-, as determined at a central laboratory using Ventana's PATHWAY anti-HER-2/neu (4B5) Rabbit Monoclonal Primary Antibody assay. Patients must have received chemotherapy in the metastatic setting or have developed disease recurrence during or within 6 months of completing adjuvant chemotherapy. Patients who were HR+ must have received at least one endocrine therapy or be ineligible for endocrine therapy. Patients were randomized 2:1 to receive either ENHERTU 5.4 mg/kg (N=373) by intravenous infusion every 3 weeks or physician's choice of chemotherapy (N=184, eribulin, capecitabine, gemcitabine, nab paclitaxel, or paclitaxel). Randomization was stratified by HER2 IHC status of tumor samples (IHC 1+ or IHC 2+/ISH-), number of prior lines of chemotherapy in the metastatic setting (1 or 2), and HR status/prior CDK4/6i treatment (HR+ with prior CDK4/6 inhibitor treatment, HR+ without prior CDK4/6 inhibitor treatment, or HR-). (…)<br>**14.3 Unresectable or Metastatic HER2-Mutant Non-Small Cell Lung Cancer**<br>ENHERTU was evaluated in DESTINY-Lung01 (NCT03505710) and at two dose levels in DESTINY-Lung02 (NCT04644237). Patients were prospectively selected for treatment with ENHERTU based on the presence of activating HER2 (ERBB2) mutations by local testing using tissue. Samples from DESTINY-Lung01 were retrospectively tested using Oncomine™ Dx Target Test (Life Technologies Corporation, Tissue-test) and Guardant360® CDx test (Guardant Health Inc., Plasma test). Demographic and baseline disease characteristics were similar for patients in DESTINY-Lung01 and DESTINY-Lung02, except for race (34% Asian vs 79% Asian, respectively). Response rates were consistent across dose levels. Increased rates of ILD/pneumonitis were observed at the higher dose. The approved recommended dose of 5.4 mg/kg intravenously every 3 weeks in the DESTINY-Lung02 study is described below [see Adverse Reactions (6.1)].<br>The efficacy of ENHERTU was evaluated in DESTINY-Lung02, a multicenter, multicohort, randomized, blinded, dose-optimization trial. Eligible patients were required to have unresectable or metastatic HER2-mutant non-squamous NSCLC with disease progression after one prior systemic therapy. Patients with a history of steroid dependent ILD/pneumonitis, clinically significant cardiac disease, clinically active brain metastases, and ECOG performance status >1 were excluded. Patients received ENHERTU 5.4 mg/kg by intravenous infusion every 3 weeks until disease progression or unacceptable toxicity. Tumor imaging was obtained every 6 weeks and CT/MRI of the brain was mandatory for patients with stable brain metastases at baseline.<br>**14.4 HER2-Positive Locally Advanced or Metastatic Gastric Cancer**<br>The efficacy of ENHERTU was evaluated in study DESTINY-Gastric01 (NCT03329690), a multicenter, open-label, randomized trial conducted in Japan and South Korea that enrolled 188 adult patients with HER2-positive (IHC 3+ or IHC 2+/ISH positive), locally advanced or metastatic gastric or GEJ adenocarcinoma who had progressed on at least two prior regimens including trastuzumab, a fluoropyrimidine- and a platinum-containing chemotherapy. HER2 expression was

* Therapeutic areas do not necessarily reflect the CDER review division.
† Representative biomarkers are listed based on standard nomenclature as per the Human Genome Organization (HUGO) symbol and/or simplified descriptors using other common conventions. Listed biomarkers do not necessarily reflect the terminology used in labeling. The term “Nonspecific” is provided when labeling does not explicitly identify the specific biomarker(s) or when the biomarker is represented by a molecular phenotype or gene signature, and in some cases the biomarker was inferred based on the labeling language.
‡ Referenced figures and tables may be found in the labeling available at Drugs@FDA.
Blue text represents the most recent addtions and/or changes since last posted version.

Table of Pharmacogenomic Biomarkers in Drug Labeling

Last Updated: 06/2024

NDA/ANDA/BLA Number, Label Version Date	Drug	Therapeutic Area*	Biomarker†	Labeling Sections	Labeling Text‡
					determined by a central lab on tissue obtained either before or after prior trastuzumab treatment. Patients were excluded for a history of treated or current ILD, a history of clinically significant cardiac disease, active brain metastases, or ECOG performance status >1.<br>Patients were randomized 2:1 to receive ENHERTU (N=126) 6.4 mg/kg intravenously every 3 weeks or physician's choice of chemotherapy: irinotecan monotherapy (N=55) 150 mg/m2 intravenously every 2 weeks or paclitaxel monotherapy (N=7) 80 mg/m2 intravenously weekly. Randomization was stratified by HER2 status (IHC 3+ or IHC 2+/ISH+), ECOG performance status (0 or 1), and region (Japan or South Korea). Tumor imaging assessments were performed at screening and every 6 weeks from the first treatment dose. Treatment was administered until unacceptable toxicity or disease progression. The major efficacy outcomes were ORR assessed by ICR according to RECIST v1.1 and OS in the intent-to-treat population. Additional efficacy outcomes were PFS and DOR.<br>The median age was 66 years (range 28 to 82); 76% were male; and 100% were Asian. All patients received a trastuzumab product. Patients had an ECOG performance status of either 0 (49%) or 1 (51%); 87% had gastric adenocarcinoma and 13% had GEJ adenocarcinoma; 76% were IHC 3+ and 23% were IHC 2+/ISH+; 65% had inoperable advanced cancer; 35% had postoperative recurrent cancer; 54% had liver metastases; 29% had lung metastases; 45% had three or more prior regimens in the locally advanced or metastatic setting. A total of 30% of patients were identified as HER2-positive using tissue obtained following prior treatment with a trastuzumab product.<br>**14.5 HER2-Positive (IHC 3+) Unresectable or Metastatic Solid Tumors**<br>The efficacy of ENHERTU was evaluated in 192 adult patients with previously treated unresectable or metastatic HER2-positive (IHC 3+) solid tumors who were enrolled in one of three multicenter trials: DESTINY-PanTumor02, DESTINY-Lung01, and DESTINY-CRC02. All three studies excluded patients with a history of ILD/pneumonitis requiring treatment with steroids or ILD/pneumonitis at screening and clinically significant cardiac disease. Patients were also excluded for active brain metastases or ECOG performance status >1. Patients received ENHERTU 5.4 mg/kg by intravenous infusion every three weeks. Treatment was administered until disease progression, death, withdrawal of consent, or unacceptable toxicity. The major efficacy outcome measure in all three of the studies was confirmed objective response rate (ORR) and an additional efficacy outcome measure was duration of response (DOR). All outcomes were assessed by independent central review (ICR) based on RECIST v1.1.<br>*DESTINY-PanTumor02*<br>DESTINY-PanTumor02 (NCT04482309) was a multicenter, multicohort, open-label trial that included 111 adult patients with locally advanced, unresectable, or metastatic HER2-positive (IHC 3+ by either local or central assessment) solid tumors that progressed following at least one prior systemic regimen in the advanced/metastatic setting or that had no satisfactory alternative treatment option.<br>The median age was 64 years (range 23 to 85); 59% were female; 58% were White, 34% were Asian, and 4% were Black or African American; 3% of patients were of Hispanic/Latino ethnicity. Patients had an ECOG performance status of either 0 (49%) or 1 (51%) at baseline. The median number of prior regimens in any treatment setting was 2.<br>*DESTINY-Lung01*<br>DESTINY-Lung01 (NCT03505710) was a multicenter, open-label, 2-cohort trial that included 17 patients with previously treated, unresectable, or metastatic, centrally confirmed HER2-positive (IHC 3+) NSCLC. Patients must have relapsed from or be refractory to standard treatment or have no available standard treatment.<br>The median age was 59 years (range 31 to 74); 59% were male; 65% were White, 18% were Asian, and 12% were Black or African American. Patients had an ECOG performance status of either 0 (12%) or 1 (88%) at baseline. The median number of prior regimens in any treatment setting was 3.<br>*DESTINY-CRC02*<br>DESTINY-CRC02 (NCT04744831) was a multicenter, randomized, 2-arm trial that included 64 patients with previously treated, unresectable or metastatic centrally confirmed HER2-positive (IHC 3+) colorectal cancer (CRC). Unless contraindicated, patients must have received fluoropyrimidine, oxaliplatin and irinotecan. If clinically indicated, patients must have received anti-EGFR treatment, anti-VEGF treatment and anti-PDL1 therapy.
761139, 04/05/2024	Fam-Trastuzumab Deruxtecan-nxki (2)	Oncology	ESR (Hormone Receptor)	Clinical Studies	**14 CLINICAL STUDIES**<br>**14.2 HER2-Low Metastatic Breast Cancer**<br>The efficacy of ENHERTU was evaluated in study DESTINY-Breast04 (NCT03734029), a randomized, multicenter, open-label study that enrolled 557 adult patients with unresectable or metastatic HER2-low breast cancer. The study included 2 cohorts: 494 hormone receptor-positive (HR+) patients and 63 hormone receptor-negative (HR-) patients. HER2-low expression was defined as IHC 1+ or IHC 2+/ISH-, as determined at a central laboratory using Ventana's PATHWAY anti-HER-2/neu (4B5) Rabbit Monoclonal Primary Antibody assay. Patients must have received chemotherapy in the metastatic setting or have developed disease recurrence during or within 6 months of completing adjuvant chemotherapy. Patients who were HR+ must have received at least one endocrine therapy or be ineligible for endocrine therapy. Patients were randomized 2:1 to receive either ENHERTU 5.4 mg/kg (N=373) by intravenous infusion every 3 weeks or physician's choice of chemotherapy (N=184, eribulin, capecitabine, gemcitabine, nab paclitaxel, or paclitaxel). Randomization was stratified by HER2 IHC status of tumor samples (IHC 1+ or IHC 2+/ISH-), number of prior lines of chemotherapy in the metastatic setting (1 or 2), and HR status/prior CDK4/6i treatment (HR+ with prior CDK4/6 inhibitor treatment, HR+ without prior CDK4/6 inhibitor treatment, or HR-). (…)
**022030, 06/17/2021**	Fesoterodine	Urology	CYP2D6	Drug Interactions, Clinical Pharmacology	**7 DRUG INTERACTIONS**<br>**7.2 CYP3A4 Inhibitors**<br>Doses of Toviaz greater than 4 mg are not recommended in patients taking potent CYP3A4 inhibitors, such as ketoconazole, itraconazole, and clarithromycin. Coadministration of the potent CYP3A4 inhibitor ketoconazole with fesoterodine led to approximately a doubling of the maximum concentration (Cmax) and area under the concentration versus time curve (AUC) of 5-hydroxymethyl tolterodine (5-HMT), the active metabolite of fesoterodine. Compared with CYP2D6 extensive metabolizers not taking ketoconazole, further increases in the exposure to 5-HMT were observed in subjects who were CYP2D6 poor metabolizers taking ketoconazole [see Clinical Pharmacology (12.3), Warnings and Precautions (5.8), and Dosage and Administration (2)]. (…)<br>**7.4 CYP2D6 Inhibitors**<br>The interaction with CYP2D6 inhibitors was not tested clinically. In poor metabolizers for CYP2D6, representing a maximum CYP2D6 inhibition, Cmax and AUC of the active metabolite are increased 1.7- and 2-fold, respectively.

* Therapeutic areas do not necessarily reflect the CDER review division.

† Representative biomarkers are listed based on standard nomenclature as per the Human Genome Organization (HUGO) symbol and/or simplified descriptors using other common conventions. Listed biomarkers do not necessarily reflect the terminology used in labeling. The term "Nonspecific" is provided when labeling does not explicitly identify the specific biomarker(s) or when the biomarker is represented by a molecular phenotype or gene signature, and in some cases the biomarker was inferred based on the labeling language.

‡ Referenced figures and tables may be found in the labeling available at Drugs@FDA.

Blue text represents the most recent addtions and/or changes since last posted version.

NDA/ANDA/BLA Number, Label Version Date	Drug	Therapeutic Area*	Biomarker†	Labeling Sections	Labeling Text‡
					No dosing adjustments are recommended in the presence of CYP2D6 inhibitors.<br><br>**12 CLINICAL PHARMACOLOGY**<br>**12.2 Pharmacodynamics**<br>*Cardiac Electrophysiology*<br>(…) Electrocardiographic parameters were measured over a 24-hour period at pre-dose, after the first administration, and after the third administration of study medication. Fesoterodine 28 mg was chosen because this dose, when administered to CYP2D6 extensive metabolizers, results in an exposure to the active metabolite that is similar to the exposure in a CYP2D6 poor metabolizer receiving fesoterodine 8 mg together with CYP3A4 blockade. (…)<br>**12.3 Pharmacokinetics**<br>*Absorption*<br>(…) A summary of pharmacokinetic parameters for the active metabolite after a single dose of Toviaz 4 mg and 8 mg in extensive and poor metabolizers of CYP2D6 is provided in Table 2. (See Table 8) (…)<br>*Metabolism*<br>(…) Variability in CYP2D6 Metabolism: A subset of individuals (approximately 7% of Caucasians and approximately 2% of African Americans) are poor metabolizers for CYP2D6. Cmax and AUC of the active metabolite are increased 1.7- and 2-fold, respectively, in CYP2D6 poor metabolizers, as compared to extensive metabolizers.<br>*Pediatric Patients:* In pediatric patients, from 6 years to 17 years of age with NDO weighing 35 kg with CYP2D6 extensive metabolizer status receiving Toviaz tablets, the mean values of apparent oral clearance, volume of distribution and absorption rate constant of 5-HMT are estimated to be approximately 72 L/h, 68 L and 0.09 h-1, respectively. The Tmax and half-life of 5-HMT are estimated to be approximately 2.55 h and 7.73 h, respectively. Like adults, the 5-HMT exposures in CYP2D6 poor metabolizers was estimated to be approximately 2-fold higher compared with extensive metabolizers. The post-hoc estimates of steady-state exposures of 5-HMT in NDO patients weighing greater than 25 kg following Toviaz 4 mg and 8 mg tablets once daily are summarized in Table 9.<br>*Drug-Drug Interactions*<br>*CYP3A4 Inhibitors:* Following blockade of CYP3A4 by coadministration of the potent CYP3A4 inhibitor ketoconazole 200 mg twice a day for 5 days, Cmax and AUC of the active metabolite of fesoterodine increased 2.0- and 2.3-fold, respectively, after oral administration of Toviaz 8 mg to CYP2D6 extensive metabolizers. In CYP2D6 poor metabolizers, Cmax and AUC of the active metabolite of fesoterodine increased 2.1- and 2.5-fold, respectively, during coadministration of ketoconazole 200 mg twice a day for 5 days. Cmax and AUC were 4.5- and 5.7-fold higher, respectively, in subjects who were CYP2D6 poor metabolizers and taking ketoconazole compared to subjects who were CYP2D6 extensive metabolizers and not taking ketoconazole. In a separate study coadministering fesoterodine with ketoconazole 200 mg once a day for 5 days, the Cmax and AUC values of the active metabolite of fesoterodine were increased 2.2-fold in CYP2D6 extensive metabolizers and 1.5- and 1.9-fold, respectively, in CYP2D6 poor metabolizers. Cmax and AUC were 3.4- and 4.2 fold higher, respectively, in subjects who were CYP2D6 poor metabolizers and taking ketoconazole compared to subjects who were CYP2D6 extensive metabolizers and not taking ketoconazole. There is no clinically relevant effect of moderate CYP3A4 inhibitors on the pharmacokinetics of fesoterodine. (…)<br>*CYP2D6 Inhibitors:* The interaction with CYP2D6 inhibitors was not studied. In poor metabolizers for CYP2D6, representing a maximum CYP2D6 inhibition, Cmax and AUC of the active metabolite are increased 1.7- and 2-fold, respectively. [see Drug Interactions (7.4)].
**022526, 08/18/2015**	Flibanserin (1)	Gynecology	CYP2C9	Clinical Pharmacology	**12 CLINICAL PHARMACOLOGY**<br>**12.5 Pharmacogenomics**<br>Patients who are poor metabolizers of CYP2D6, CYP2C9 or CYP2C19 are deficient in CYP2D6, CYP2C9 or CYP2C19 enzyme activity, respectively. Extensive metabolizers have normal functioning CYP enzymes.<br>*CYP2C9 Poor Metabolizers*<br>A study comparing flibanserin exposure in CYP2C9 poor metabolizers to CYP2C9 extensive metabolizers was conducted in lieu of a drug interaction study with ADDYI and a strong CYP2C9 inhibitor. In 8 women who were poor metabolizers of CYP2C9, Cmax and AUC0-inf of flibanserin 100 mg once daily decreased 23% and 18%, compared to exposures among 8 extensive metabolizers of CYP2C9.
**022526, 08/18/2015**	Flibanserin (2)	Gynecology	CYP2C19	Adverse Reactions, Use in Specific Populations, Clinical Pharmacology	**6 ADVERSE REACTIONS**<br>**6.1 Clinical Trials Experience**<br>*Syncope in Poor CYP2C19 Metabolizers*<br>In a pharmacogenomic study of 100 mg ADDYI in subjects who were poor or extensive CYP2C19 metabolizers, syncope occurred in 1/9 (11%) subjects who were CYP2C19 poor metabolizers (this subject had a 3.2 fold higher flibanserin exposure compared to CYP2C19 extensive metabolizers) compared to no such adverse reactions in subjects who were CYP2C19 extensive metabolizers [see Drug Interactions (7), Use in Specific Populations (8.7) and Clinical Pharmacology (12.5)].<br><br>**8 USE IN SPECIFIC POPULATIONS**<br>**8.7 CYP2C19 Poor Metabolizers**<br>CYP2C19 poor metabolizers had increased flibanserin exposures compared to CYP2C19 extensive metabolizers. Additionally, syncope occurred in a subject who was a CYP2C19 poor metabolizer [see Adverse Reactions (6.1) and Clinical Pharmacology (12.5)]. Therefore, increase monitoring for adverse reactions (e.g., hypotension) in patients who are CYP2C19 poor metabolizers. The frequencies of poor CYP2C19 metabolizers are approximately 2–5% among Caucasians and Africans and approximately 2–15% among Asians.<br><br>**12 CLINICAL PHARMACOLOGY**<br>**12.5 Pharmacogenomics**

NDA/ANDA/BLA Number, Label Version Date	Drug	Therapeutic Area*	Biomarker†	Labeling Sections	Labeling Text‡
					Patients who are poor metabolizers of CYP2D6, CYP2C9 or CYP2C19 are deficient in CYP2D6, CYP2C9 or CYP2C19 enzyme activity, respectively. Extensive metabolizers have normal functioning CYP enzymes.<br>*CYP2C19 Poor Metabolizers*<br>A study comparing flibanserin exposure in CYP2C19 poor metabolizers to CYP2C19 extensive metabolizers was conducted in lieu of a drug interaction study with ADDYI and a strong CYP2C19 inhibitor. In 9 women who were poor metabolizers of CYP2C19, Cmax and AUC0-inf of flibanserin 100 mg once daily increased 1.5-fold (1.1-2.1) and 1.3-fold (0.9-2.1), compared to exposures among 8 extensive metabolizers of CYP2C19. Flibanserin half-life was increased from 11.1 hours in the extensive metabolizers of CYP2C19 to 13.5 hours in the poor metabolizers of CYP2C19 [see Adverse Reactions (6.1) and Use in Specific Populations (8.7)].<br>The frequencies of poor metabolizers of CYP2C19 are approximately 2–5% among Caucasians and Africans and approximately 2–15% among Asians.
**022526, 08/18/2015**	Flibanserin (3)	Gynecology	CYP2D6	Clinical Pharmacology	**12 CLINICAL PHARMACOLOGY**<br>**12.5 Pharmacogenomics**<br>Patients who are poor metabolizers of CYP2D6, CYP2C9 or CYP2C19 are deficient in CYP2D6, CYP2C9 or CYP2C19 enzyme activity, respectively. Extensive metabolizers have normal functioning CYP enzymes.<br>*CYP2D6 Poor Metabolizers*<br>A study comparing flibanserin exposure in CYP2D6 poor metabolizers to CYP2D6 extensive metabolizers was conducted in addition to a drug interaction study with paroxetine, a strong CYP2D6 inhibitor. In 12 poor metabolizers of CYP2D6, steady state Cmax and AUC of flibanserin 50 mg twice daily was decreased by 4% and increased by 18%, respectively, compared to exposures among 19 extensive metabolizers, intermediate metabolizers and ultra rapid metabolizers of CYP2D6.
020985, 05/26/2022	Fluorouracil (1)	Dermatology	DPYD	Contraindications, Warnings	**CONTRAINDICATIONS**<br>(…) Carac should not be used in patients with dihydropyrimidine dehydrogenase (DPD) enzyme deficiency. A large percentage of fluorouracil is catabolized by the enzyme dihydropyrimidine dehydrogenase (DPD). DPD enzyme deficiency can result in shunting of fluorouracil to the anabolic pathway, leading to cytotoxic activity and potential toxicities. (…)<br><br>**WARNINGS**<br>The potential for a delayed hypersensitivity reaction to fluorouracil exists. Patch testing to prove hypersensitivity may be inconclusive.<br>Patients should discontinue therapy with Carac if symptoms of DPD enzyme deficiency develop.<br>Rarely, unexpected, systemic toxicity (e.g. stomatitis, diarrhea, neutropenia, and neurotoxicity) associated with parental administration of fluorouracil has been attributed to deficiency of dihydropyrimidine dehydrogenase “DPD” activity. One case of life threatening systemic toxicity has been reported with the topical use of 5% fluorouracil in a patient with a complete absence of DPD enzyme activity. Symptoms included severe abdominal pain, bloody diarrhea, vomiting, fever, and chills. Physical examination revealed stomatitis, erythematous skin rash, neutropenia, thrombocytopenia, inflammation of the esophagus, stomach, and small bowel. Although this case was observed with 5% fluorouracil cream, it is unknown whether patients with profound DPD enzyme deficiency would develop systemic toxicity with lower concentrations of topically applied fluorouracil.<br>Applications to mucous membranes should be avoided due to the possibility of local inflammation and ulceration.
**202669, 03/21/2024**	Fluorouracil (2)	Oncology	DPYD	Warnings and Precautions, Clinical Pharmacology, Patient Counseling Information	**5 WARNINGS AND PRECAUTIONS**<br>**5.1 Serious Adverse Reactions from Dihydropyrimidine Dehydrogenase (DPD)-Deficiency**<br>Patients with certain homozygous or compound heterozygous variants in the DPYD gene known to result in complete or near complete absence of DPD activity (complete DPD deficiency) are at increased risk for acute early-onset toxicity and serious, including fatal, adverse reactions due to fluorouracil (e.g., mucositis, diarrhea, neutropenia, and neurotoxicity). Patients with partial DPD activity (partial DPD deficiency) may also have increased risk of serious, including fatal, adverse reactions.<br>Fluorouracil is not recommended for use in patients known to have certain homozygous or compound heterozygous DPYD variants that result in complete DPD deficiency.<br>Withhold or permanently discontinue fluorouracil based on clinical assessment of the onset, duration, and severity of the observed adverse events in patients with evidence of acute early-onset or unusually severe reactions, which may indicate complete DPD deficiency. No fluorouracil dose has been proven safe for patients with complete DPD deficiency. There are insufficient data to recommend a specific dose in patients with partial DPD deficiency.<br>Consider testing for genetic variants of DPYD prior to initiating fluorouracil to reduce the risk of serious adverse reactions if the patient's clinical status permits and based on clinical judgement [see Clinical Pharmacology (12.5)]. Serious adverse reactions may still occur even if no DPYD variants are identified.<br>An FDA-authorized test for the detection of genetic variants of DPYD to identify patients at risk of serious adverse reactions due to increased systemic exposure to fluorouracil is not currently available. Currently available tests used to identify DPYD variants may vary in accuracy and design (e.g., which DPYD variant(s) they identify).<br><br>**12 CLINICAL PHARMACOLOGY**<br>**12.5 Pharmacogenomics**<br>The DPYD gene encodes the enzyme DPD, which is responsible for the catabolism of >80% of fluorouracil. Approximately 3 to 5% of White populations have partial DPD deficiency and 0.2% of White populations have complete DPD deficiency, which may be due to certain genetic no function or decreased function variants in DPYD resulting in partial to complete or near complete absence of enzyme activity. DPD deficiency is estimated to be more prevalent in Black or African American populations compared to White populations. Insufficient information is available to estimate the prevalence of DPD deficiency in other populations.<br>Patients who are homozygous or compound heterozygous for no function DPYD variants (i.e., carry two no function DPYD variants) or are compound heterozygous for a no function DPYD variant plus a decreased function DPYD variant have complete DPD deficiency and are at increased risk for acute early-

* Therapeutic areas do not necessarily reflect the CDER review division.
† Representative biomarkers are listed based on standard nomenclature as per the Human Genome Organization (HUGO) symbol and/or simplified descriptors using other common conventions. Listed biomarkers do not necessarily reflect the terminology used in labeling. The term “Nonspecific” is provided when labeling does not explicitly identify the specific biomarker(s) or when the biomarker is represented by a molecular phenotype or gene signature, and in some cases the biomarker was inferred based on the labeling language.
‡ Referenced figures and tables may be found in the labeling available at Drugs@FDA.
Blue text represents the most recent addtions and/or changes since last posted version.

NDA/ANDA/BLA Number, Label Version Date	Drug	Therapeutic Area*	Biomarker†	Labeling Sections	Labeling Text‡
					onset of toxicity and serious life-threatening, or fatal adverse reactions due to increased systemic exposure to fluorouracil. Partial DPD deficiency can result from the presence of either two decreased function DPYD variants or one normal function plus either a decreased function or a no function DPYD variant. Patients with partial DPD deficiency may also be at an increased risk for toxicity from fluorouracil.<br>Four DPYD variants have been associated with impaired DPD activity in White populations, especially when present as homozygous or compound heterozygous variants: c.1905+1G>A (DPYD *2A), c.1679T>G (DPYD *13), c.2846A>T, and c.1129-5923C>G (Haplotype B3). DPYD*2A and DPYD*13 are no function variants, and c.2846A>T and c.1129-5923C>G are decreased function variants. The decreased function DPYD variant c.557A>G is observed in individuals of African ancestry. This is not a complete listing of all DPYD variants that may result in DPD deficiency [see Warnings and Precautions (5.1)].<br><br>**17 PATIENT COUNSELING INFORMATION**<br>Advise:<br>• Inform patients of the potential for serious and life-threatening adverse reactions due to DPD deficiency and discuss with your patient whether they should be tested for genetic variants of DPYD that are associated with an increased risk of serious adverse reactions from the use of fluorouracil. Advise patients to immediately contact their healthcare provider if symptoms of severe mucositis, diarrhea, neutropenia, and neurotoxicity occur [see Warnings and Precautions (5.1) and Clinical Pharmacology (12.5)].(…)
**018936, 10/06/2021**	Fluoxetine	Psychiatry	CYP2D6	Warnings and Precautions, Drug Interactions, Clinical Pharmacology	**5 WARNINGS AND PRECAUTIONS**<br>**5.11 QT Prolongation**<br>Post-marketing cases of QT interval prolongation and ventricular arrhythmia including Torsades de Pointes have been reported in patients treated with PROZAC. PROZAC should be used with caution in patients with congenital long QT syndrome; a previous history of QT prolongation; a family history of long QT syndrome or sudden cardiac death; and other conditions that predispose to QT prolongation and ventricular arrhythmia. Such conditions include concomitant use of drugs that prolong the QT interval; hypokalemia or hypomagnesemia; recent myocardial infarction, uncompensated heart failure, bradyarrhythmias, and other significant arrhythmias; and conditions that predispose to increased fluoxetine exposure (overdose, hepatic impairment, use of CYP2D6 inhibitors, CYP2D6 poor metabolizer status, or use of other highly protein-bound drugs). PROZAC is primarily metabolized by CYP2D6 [see Contraindications (4.2), Adverse Reactions (6.2), Drug Interactions (7.7, 7.8), Overdosage (10.1), and Clinical Pharmacology (12.3)]. (…)<br><br>**7 DRUG INTERACTIONS**<br>**7.7 Potential for PROZAC to affect Other Drugs**<br>*Drugs Metabolized by CYP2D6* — Fluoxetine inhibits the activity of CYP2D6, and may make individuals with normal CYP2D6 metabolic activity resemble a poor metabolizer. Coadministration of fluoxetine with other drugs that are metabolized by CYP2D6, including certain antidepressants (e.g., TCAs), antipsychotics (e.g., phenothiazines and most atypicals), and antiarrhythmics (e.g., propafenone, flecainide, and others) should be approached with caution. Therapy with medications that are predominantly metabolized by the CYP2D6 system and that have a relatively narrow therapeutic index (see list below) should be initiated at the low end of the dose range if a patient is receiving fluoxetine concurrently or has taken it in the previous 5 weeks.<br><br>**12 CLINICAL PHARMACOLOGY**<br>**12.3 Pharmacokinetics**<br>*Variability in Metabolism* — A subset (about 7%) of the population has reduced activity of the drug metabolizing enzyme cytochrome P450 2D6 (CYP2D6). Such individuals are referred to as “poor metabolizers” of drugs such as debrisoquin, dextromethorphan, and the TCAs. In a study involving labeled and unlabeled enantiomers administered as a racemate, these individuals metabolized S-fluoxetine at a slower rate and thus achieved higher concentrations of S-fluoxetine. Consequently, concentrations of S-norfluoxetine at steady state were lower. The metabolism of R-fluoxetine in these poor metabolizers appears normal. When compared with normal metabolizers, the total sum at steady state of the plasma concentrations of the 4 active enantiomers was not significantly greater among poor metabolizers. Thus, the net pharmacodynamic activities were essentially the same. Alternative, nonsaturable pathways (non-2D6) also contribute to the metabolism of fluoxetine. This explains how fluoxetine achieves a steady-state concentration rather than increasing without limit.
**018766, 05/09/2016**	Flurbiprofen	Rheumatology	CYP2C9	Clinical Pharmacology	**12 CLINICAL PHARMACOLOGY**<br>**12.3 Pharmacokinetics**<br>*Poor Metabolizers of CYP2C9 Substrates*<br>In patients who are known or suspected to be poor CYP2C9 metabolizers based on genotype or previous history/experience with other CYP2C9 substrates (such as warfarin and phenytoin), reduce the dose of flurbiprofen to avoid abnormally high plasma levels due to reduced metabolic clearance.
**018554, 07/23/2001**	Flutamide	Oncology	G6PD	Warnings	**WARNINGS**<br>**Aniline Toxicity:**<br>One metabolite of flutamide is 4-nitro-3-fluoromethylaniline. Several toxicities consistent with aniline exposure, including methemoglobinemia, hemolytic anemia and cholestatic jaundice have been observed in both animals and humans after flutamide administration. In patients susceptible to aniline toxicity (e.g., persons with glucose-6-phosphate dehydrogenase deficiency, hemoglobin M disease and smokers), monitoring of methemoglobin levels should be considered.
**022007, 05/29/2019**	Formoterol (1)	Pulmonary	CYP2D6	Clinical Pharmacology	**12 CLINICAL PHARMACOLOGY**<br>**12.3 Pharmacokinetics**<br>*Metabolism*<br>(…) Formoterol did not inhibit CYP450 enzymes at therapeutically relevant concentrations. Some patients may be deficient in CYP2D6 or 2C19 or both. Whether a deficiency in one or both of these isozymes results in elevated systemic exposure to formoterol or systemic adverse effects has not been adequately explored.

* Therapeutic areas do not necessarily reflect the CDER review division.
† Representative biomarkers are listed based on standard nomenclature as per the Human Genome Organization (HUGO) symbol and/or simplified descriptors using other common conventions. Listed biomarkers do not necessarily reflect the terminology used in labeling. The term “Nonspecific” is provided when labeling does not explicitly identify the specific biomarker(s) or when the biomarker is represented by a molecular phenotype or gene signature, and in some cases the biomarker was inferred based on the labeling language.
‡ Referenced figures and tables may be found in the labeling available at Drugs@FDA.
Blue text represents the most recent addtions and/or changes since last posted version.

NDA/ANDA/BLA Number, Label Version Date	Drug	Therapeutic Area*	Biomarker†	Labeling Sections	Labeling Text‡
022007, 05/29/2019	Formoterol (2)	Pulmonary	CYP2C19	Clinical Pharmacology	**12 CLINICAL PHARMACOLOGY**<br>**12.3 Pharmacokinetics**<br>*Metabolism*<br>(…) Formoterol did not inhibit CYP450 enzymes at therapeutically relevant concentrations. Some patients may be deficient in CYP2D6 or 2C19 or both. Whether a deficiency in one or both of these isozymes results in elevated systemic exposure to formoterol or systemic adverse effects has not been adequately explored.
02/26/2021, 214018	Fosdenopterin	Neurology	MOCS1	Indications and Usage, Dosage and Administration, Adverse Reactions, Use in Specific Populations, Clinical Pharmacology, Clinical Studies	**1 INDICATIONS AND USAGE**<br>NULIBRY is indicated to reduce the risk of mortality in patients with molybdenum cofactor deficiency (MoCD) Type A.<br><br>**2 DOSAGE AND ADMINISTRATION**<br>**2.1 Patient Selection**<br>Start NULIBRY if the patient has a diagnosis or presumptive diagnosis of MoCD Type A.<br>In patients with a presumptive diagnosis of MoCD Type A, confirm the diagnosis of MoCD Type A immediately after initiation of NULIBRY treatment. In such patients, discontinue NULIBRY if the MoCD Type A diagnosis is not confirmed by genetic testing.<br><br>**6 ADVERSE REACTIONS**<br>**6.1 Clinical Trials Experience**<br>Overview of Safety Evaluation<br>The safety of NULIBRY was assessed in 37 pediatric patients and healthy adults who received at least one intravenous infusion of NULIBRY or an E. coli derived non-salt, anhydrous form of cPMP (recombinant cPMP or rcPMP, which has the same active moiety and therefore the same biologic activity as NULIBRY). Of these 37 patients/healthy adults, 13 were pediatric patients with MoCD Type A in Studies 1, 2, and 3 [see Clinical Studies (14)], 6 were pediatric patients with presumptive MoCD Type A but who were later confirmed to not have MoCD Type A, and 18 were healthy adults (without MoCD Type A) in a Phase 1 study.<br>Adverse Reactions<br>Assessment of adverse reactions for NULIBRY is based on data from two open-label, single-arm studies, Study 1 (n=8) and Study 2 (n=1), in patients with a confirmed diagnosis of MoCD Type A (8 of the 9 patients were previously treated with rcPMP). In these studies, patients received a daily intravenous infusion of NULIBRY. The median exposure to NULIBRY was 4.3 years and ranged from 8 days to 5.6 years [see Clinical Studies (14)]. In these studies, 44% of patients were males and 56% were females, 67% were White and 33% were Asian. The mean age was 14 days and ranged from 1 day to 69 days at time of first infusion.<br>Table 2 presents the most common adverse reactions that occurred in NULIBRY-treated patients in Studies 1 and 2. (See Table 2)<br>Safety data are also available from 10 patients with MoCD Type A who received rcPMP in Study 3 (an observational study) [see Clinical Studies (14)]. The median time on rcPMP treatment was 1.5 years and ranged from 6 days to 4.4 years. In Study 3, the patient population was evenly distributed between males and females with a mean age of 18 days (range 1, 69) at time of first infusion, 70% were White, and 30% were Asian. (…)<br><br>**8 USE IN SPECIFIC POPULATIONS**<br>**8.4 Pediatric Use**<br>Safety and effectiveness of NULIBRY for the treatment of MoCD Type A have been established in pediatric patients starting from birth. Use of NULIBRY for this indication is supported by evidence from two open-label studies (Studies 1 and 2) and one observational study (Study 3), in which 13 pediatric patients aged birth to 6 years of age were treated with NULIBRY or rcPMP. Pediatric use information is discussed throughout the labeling.<br>**8.5 Geriatric Use**<br>MoCD Type A is largely a disease of pediatric patients. Clinical studies of NULIBRY did not include patients 65 years of age and older.<br>**8.6 Adult Use**<br>The safety and effectiveness of NULIBRY for the treatment of adults with MoCD Type A have been established. Use of NULIBRY in adults for this indication is based on an adequate and well- controlled clinical investigation in pediatric patients [see Clinical Studies (14)].<br><br>**12 CLINICAL PHARMACOLOGY**<br>**12.2 Pharmacodynamics**<br>In MoCD Type A, the lack of effective SOX leads to elevated levels of the neurotoxic sulfite, S-sulfocysteine (SSC). Treatment with NULIBRY resulted in a reduction in the level of urinary SSC normalized to creatinine and the reduction was sustained with long-term treatment with NULIBRY [see Clinical Studies (14)].<br>Specific Populations<br>Pediatric Patients Pharmacokinetic properties of fosdenopterin in pediatric MoCD Type A patients are similar to healthy adult subjects.<br><br>**14 CLINICAL STUDIES**<br>The efficacy of NULIBRY for the treatment of patients with MoCD Type A was established based on data from three clinical studies (Studies 1, 2, and 3) that were compared to data from a natural history study.<br>Study 1<br>Study 1 (NCT02047461) was a prospective, open-label, single-arm, dose escalation study in patients with MoCD Type A who were receiving treatment with rcPMP prior to treatment with NULIBRY. Study 1 included 8 patients, 6 of whom previously participated in Study 3. The initial NULIBRY dosage was matched to the patient's rcPMP dosage upon entering the study. The NULIBRY dosage was then titrated over a period of 5 months to a maximum dosage of 0.9 mg/kg administered once daily as an intravenous infusion.

* Therapeutic areas do not necessarily reflect the CDER review division.
† Representative biomarkers are listed based on standard nomenclature as per the Human Genome Organization (HUGO) symbol and/or simplified descriptors using other common conventions. Listed biomarkers do not necessarily reflect the terminology used in labeling. The term "Nonspecific" is provided when labeling does not explicitly identify the specific biomarker(s) or when the biomarker is represented by a molecular phenotype or gene signature, and in some cases the biomarker was inferred based on the labeling language.
‡ Referenced figures and tables may be found in the labeling available at Drugs@FDA.
Blue text represents the most recent addtions and/or changes since last posted version.

Table of Pharmacogenomic Biomarkers in Drug Labeling
Last Updated: 06/2024

NDA/ANDA/BLA Number, Label Version Date	Drug	Therapeutic Area*	Biomarker†	Labeling Sections	Labeling Text‡
					Study 2<br>Study 2 (NCT02629393) was a prospective, open-label, single-arm, dose escalation study in one patient with MoCD Type A who had not been previously treated with rcPMP. The initial dosage of NULIBRY in Study 2 was based on the gestational age of the patient (i.e., 36 weeks). The initial dosage was then incrementally escalated up to a maximum dosage of 0.98 mg/kg administered once daily as an intravenous infusion (1.1 times the maximum approved recommended dosage) [see Dosage and Administration (2.1)].<br>Study 3<br>Study 3 was a retrospective, observational study that included 10 patients with a confirmed diagnosis of MoCD Type A who received rcPMP. Six of these 10 patients were later enrolled in Study 1 to receive treatment with NULIBRY. Efficacy Results The efficacy of NULIBRY and rcPMP were assessed in a combined analysis of the 13 patients with genetically confirmed MoCD Type A from Study 1 (n=8), Study 2 (n=1), and Study 3 (n=4) who received substrate replacement therapy with NULIBRY or rcPMP. Of the 13 treated patients included in the combined analysis, 54% were male, 77% were White and 23% were Asian; the median gestational age was 39 weeks (range 35 to 41 weeks). Of these 13 treated patients, the age at first dose was ≤ 14 days for 10 patients (with 5 patients initiating treatment at 1 day of age) and ≥ 32 days and < 69 days for the remaining 3 patients. Overall Survival Efficacy was assessed by comparing overall survival in pediatric patients treated with NULIBRY or rcPMP (n=13) with an untreated natural history cohort of pediatric patients with genetically confirmed MoCD Type A who were genotype-matched to the treated patients (n=18). Patients treated with NULIBRY or rcPMP had an improvement in overall survival compared to the untreated, genotype-matched, historical control group (Table 4 and Figure 1). Results were similar when comparing treated patients with all patients in the untreated natural history cohort with genetically confirmed MoCD Type A (n=37, includes the 18 genotype-matched untreated patients as well as 19 additional untreated patients who were not genotype-matched). (See Table 4 and Figure 1)<br>*MoCD Biomarker Results*<br>Treatment with NULIBRY resulted in a reduction in urine concentrations of SSC in patients with MoCD Type A and the reduction was sustained with long-term treatment over 48 months. The baseline level of urinary SSC normalized to creatinine was characterized in one patient (Study 2) with a value of 89.8 μmol/mmol. Following treatment with NULIBRY in Studies 1 and 2 (n=9), the mean ± SD levels of urinary SSC normalized to creatinine ranged from 11 (±8.5) to 7 (±2.4) μmol/mmol from Month 3 to Month 48.
**020450, 02/16/2021**	Fosphenytoin (1)	Neurology	CYP2C9	Warnings and Precautions, Use in Specific Populations, Clinical Pharmacology	**5 WARNINGS AND PRECAUTIONS**<br>**5.4 Serious Dermatologic Reactions**<br>CEREBYX can cause severe cutaneous adverse reactions (SCARs), which may be fatal. Reported reactions in phenytoin (the active metabolite of CEREBYX)-treated patients have included toxic epidermal necrolysis (TEN), Stevens-Johnson syndrome (SJS), acute generalized exanthematous pustulosis (AGEP), and Drug Reaction with Eosinophilia and Systemic Symptoms (DRESS) [see Warnings and Precautions (5.5)]. The onset of symptoms is usually within 28 days, but can occur later. CEREBYX should be discontinued at the first sign of a rash, unless the rash is clearly not drug-related. If signs or symptoms suggest a severe cutaneous adverse reaction, use of this drug should not be resumed and alternative therapy should be considered. If a rash occurs, the patient should be evaluated for signs and symptoms of SCARs.<br>Studies in patients of Chinese ancestry have found a strong association between the risk of developing SJS/TEN and the presence of HLA-B*1502, an inherited allelic variant of the HLA B gene, in patients using carbamazepine. Limited evidence suggests that HLA-B*1502 may be a risk factor for the development of SJS/TEN in patients of Asian ancestry taking other antiepileptic drugs associated with SJS/TEN, including phenytoin. In addition, retrospective, case-control, genome-wide association studies in patients of southeast Asian ancestry have also identified an increased risk of SCARs in carriers of the decreased function CYP2C9*3 variant, which has also been associated with decreased clearance of phenytoin. Consider avoiding CEREBYX as an alternative to carbamazepine in patients who are positive for HLA-B*1502 or in CYP2C9*3 carriers.<br>Should CEREBYX be utilized for CYP2C9*3 carriers, consider starting at the lower end of the dosage range [see Use in Specific Populations (8.7)].<br>The use of HLA-B*1502 or CYP2C9 genotyping has important limitations and must never substitute for appropriate clinical vigilance and patient management. The role of other possible factors in the development of, and morbidity from, SJS/TEN, such as antiepileptic drug (AED) dose, compliance, concomitant medications, comorbidities, and the level of dermatologic monitoring have not been studied.<br><br>**8 USE IN SPECIFIC POPULATIONS**<br>**8.7 Use in Patients with Decreased CYP2C9 Function**<br>Patients who are intermediate or poor metabolizers of CYP2C9 substrates (e.g., *1/*3, *2/*2, *3/*3) may exhibit increased phenytoin serum concentrations compared to patients who are normal metabolizers (e.g., *1/*1). Thus, patients who are known to be intermediate or poor metabolizers may ultimately require lower doses to maintain similar steady-state concentrations compared to normal metabolizers. In patients who are known to be carriers of the decreased function CYP2C9*2 or *3 alleles (intermediate and poor metabolizers), consider starting at the low end of the dosage range and monitor serum concentrations to maintain total phenytoin concentrations of 10 to 20 mcg/mL. If early signs of dose-related central nervous system (CNS) toxicity develop, serum concentrations should be checked immediately [see Clinical Pharmacology (12.5)].<br><br>**12 CLINICAL PHARMACOLOGY**<br>**12.5 Pharmacogenomics**<br>CYP2C9 activity is decreased in individuals with genetic variants such as the CYP2C9*2 and CYP2C9*3 alleles. Carriers of variant alleles, resulting in intermediate (e.g., *1/*3, *2/*2) or poor metabolism (e.g., *2/*3, *3/*3) have decreased clearance of phenytoin. Other decreased or nonfunctional CYP2C9 alleles may also result in decreased clearance of phenytoin (e.g., *5, *6, *8, *11).<br>The prevalence of the CYP2C9 poor metabolizer phenotype is approximately 2-3% in the White population, 0.5-4% in the Asian population, and <1% in the African American population. The CYP2C9 intermediate phenotype prevalence is approximately 35% in the White population, 24% in the African American population, and 15-36% in the Asian population [see Warnings and Precautions (5.4) and Use in Specific Populations (8.7)].
**020450, 02/16/2021**	Fosphenytoin (2)	Neurology	HLA-B	Warnings and Precautions	**5 WARNINGS AND PRECAUTIONS**<br>**5.4 Serious Dermatologic Reactions**

* Therapeutic areas do not necessarily reflect the CDER review division.
† Representative biomarkers are listed based on standard nomenclature as per the Human Genome Organization (HUGO) symbol and/or simplified descriptors using other common conventions. Listed biomarkers do not necessarily reflect the terminology used in labeling. The term “Nonspecific” is provided when labeling does not explicitly identify the specific biomarker(s) or when the biomarker is represented by a molecular phenotype or gene signature, and in some cases the biomarker was inferred based on the labeling language.
‡ Referenced figures and tables may be found in the labeling available at Drugs@FDA.
Blue text represents the most recent addtions and/or changes since last posted version.

Table of Pharmacogenomic Biomarkers in Drug Labeling
Last Updated: 06/2024

NDA/ANDA/BLA Number, Label Version Date	Drug	Therapeutic Area*	Biomarker†	Labeling Sections	Labeling Text‡
					CEREBYX can cause severe cutaneous adverse reactions (SCARs), which may be fatal. Reported reactions in phenytoin (the active metabolite of CEREBYX)-treated patients have included toxic epidermal necrolysis (TEN), Stevens-Johnson syndrome (SJS), acute generalized exanthematous pustulosis (AGEP), and Drug Reaction with Eosinophilia and Systemic Symptoms (DRESS) [see Warnings and Precautions (5.5)]. The onset of symptoms is usually within 28 days, but can occur later. CEREBYX should be discontinued at the first sign of a rash, unless the rash is clearly not drug-related. If signs or symptoms suggest a severe cutaneous adverse reaction, use of this drug should not be resumed and alternative therapy should be considered. If a rash occurs, the patient should be evaluated for signs and symptoms of SCARs.<br>Studies in patients of Chinese ancestry have found a strong association between the risk of developing SJS/TEN and the presence of HLA-B*1502, an inherited allelic variant of the HLA B gene, in patients using carbamazepine. Limited evidence suggests that HLA-B*1502 may be a risk factor for the development of SJS/TEN in patients of Asian ancestry taking other antiepileptic drugs associated with SJS/TEN, including phenytoin. In addition, retrospective, case-control, genome-wide association studies in patients of southeast Asian ancestry have also identified an increased risk of SCARs in carriers of the decreased function CYP2C9*3 variant, which has also been associated with decreased clearance of phenytoin. Consider avoiding CEREBYX as an alternative to carbamazepine in patients who are positive for HLA-B*1502 or in CYP2C9*3 carriers.<br>Should CEREBYX be utilized for CYP2C9*3 carriers, consider starting at the lower end of the dosage range [see Use in Specific Populations (8.7)].<br>The use of HLA-B*1502 or CYP2C9 genotyping has important limitations and must never substitute for appropriate clinical vigilance and patient management. The role of other possible factors in the development of, and morbidity from, SJS/TEN, such as antiepileptic drug (AED) dose, compliance, concomitant medications, comorbidities, and the level of dermatologic monitoring have not been studied.
**022033, 01/04/2017**	Fluvoxamine	Psychiatry	CYP2D6	Drug Interactions	**7 DRUG INTERACTIONS**<br>**7.1 Potential Interactions with Drugs that Inhibit or are Metabolized by Cytochrome P450 Isoenzymes**<br>(…) Approximately 7% of the normal population has a genetic code that leads to reduced levels of activity of CYP2D6 enzyme. Such individuals have been referred to as "poor metabolizers" (PM) of drugs such as debrisoquin, dextromethorphan, and tricyclic antidepressants. While none of the drugs studied for drug interactions significantly affected the pharmacokinetics of fluvoxamine, an in vivo study of fluvoxamine single-dose pharmacokinetics in 13 PM subjects demonstrated altered pharmacokinetic properties compared to 16 "extensive metabolizers" (EM): mean Cmax, AUC, and half-life were increased by 52%, 200%, and 62%, respectively, in the PM compared to the EM group. This suggests that fluvoxamine is metabolized, at least in part, by CYP2D6. Caution is indicated in patient known to have reduced levels of cytochrome P450 2D6 activity and those receiving concomitant drugs known to inhibit this cytochrome P450 isoenzyme (e.g., quinidine). (…)
**217564, 11/08/2023**	Fruquintinib	Oncology	RAS	Indications and Usage, Clinical Studies	**1 INDICATIONS AND USAGE**<br>FRUZAQLA is indicated for the treatment of adult patients with metastatic colorectal cancer (mCRC) who have been previously treated with fluoropyrimidine-, oxaliplatin-, and irinotecan-based chemotherapy, an anti-VEGF therapy, and, if RAS wild-type and medically appropriate, an anti-EGFR therapy.<br><br>**14 CLINICAL STUDIES**<br>**14.1 Metastatic Colorectal Cancer**<br>FRESCO-2 Study<br>The efficacy of FRUZAQLA was evaluated in FRESCO-2 (NCT04322539), an international, multicenter, randomized, double-blind, placebo-controlled study that enrolled 691 patients with metastatic colorectal cancer who had disease progression during or after prior treatment with fluoropyrimidine-, oxaliplatin-, irinotecan-based chemotherapy, an anti-VEGF biological therapy, if RAS wild type, an anti-EGFR biological therapy, and trifluridine/tipiracil, regorafenib, or both. Patients with an ECOG PS ≥2, left ventricular fraction ≤50%, systolic blood pressure >140 mm Hg or diastolic blood pressure >90 mm Hg, urine protein ≥1 g/24h, or untreated brain metastases were ineligible. Randomization was stratified by prior use of trifluridine/tipiracil or regorafenib (trifluridine/tipiracil vs. regorafenib vs. trifluridine/tipiracil and regorafenib), RAS status (wild type vs. mutant), and duration of metastatic disease (≤18 months vs. 18 months).<br>The study population characteristics were median age of 64 years (range: 25 to 86), with 47% ≥65 years of age; 56% male; 81% White, 9% Asian, 2.9% Black or African American, and 0.7% Native Hawaiian/Pacific Islander; 43% had an ECOG PS of 0 and 57% had an ECOG PS of 1, and 63% had RAS-mutant tumors. Eighteen percent of the patients were enrolled in North America, 72% in Europe, and 10% in Asia Pacific (Japan and Australia) region. (…)<br>FRESCO Study<br>The efficacy of FRUZAQLA was evaluated in FRESCO (NCT02314819), a multicenter, randomized, double-blind, placebo-controlled study conducted in China that enrolled 416 patients with metastatic colorectal cancer who had disease progression during or after prior treatment with fluoropyrimidine-, oxaliplatin, or irinotecan-based chemotherapy. Patients older than 75 years of age, Eastern Cooperative Oncology Group (ECOG) performance status (PS) ≥2, left ventricular ejection fraction ≤50%, systolic blood pressure >140 mm Hg or diastolic blood pressure >90 mm Hg, urine protein ≥1 g/24h, or brain metastases were ineligible. Randomization was stratified by prior use of VEGF inhibitors (yes vs. no) and K-RAS status (wild type vs. mutant). (…)<br>The study population characteristics were median age of 56 years (range: 23 to 75), with 19% ≥65 years of age; 61% male; 100% Asian; 27% had an ECOG PS of 0 and 73% had an ECOG PS of 1 (73%), and 44% had K-RAS mutant tumors.
**021344, 05/13/2020**	Fulvestrant (1)	Oncology	ERBB2 (HER2)	Indications and Usage, Adverse Reactions, Clinical Studies	**1 INDICATIONS AND USAGE**<br>Monotherapy<br>FASLODEX is indicated for the treatment of:<br>• Hormone receptor (HR)-positive, human epidermal growth factor receptor 2 (HER2)-negative advanced breast cancer in postmenopausal women not previously treated with endocrine therapy, or<br>• HR-positive advanced breast cancer in postmenopausal women with disease progression following endocrine therapy.<br>Combination Therapy<br>FASLODEX is indicated for the treatment of:<br>• HR-positive, HER2-negative advanced or metastatic breast cancer in postmenopausal women in combination with ribociclib as initial endocrine based therapy or following disease progression on endocrine therapy.

* Therapeutic areas do not necessarily reflect the CDER review division.
† Representative biomarkers are listed based on standard nomenclature as per the Human Genome Organization (HUGO) symbol and/or simplified descriptors using other common conventions. Listed biomarkers do not necessarily reflect the terminology used in labeling. The term "Nonspecific" is provided when labeling does not explicitly identify the specific biomarker(s) or when the biomarker is represented by a molecular phenotype or gene signature, and in some cases the biomarker was inferred based on the labeling language.
‡ Referenced figures and tables may be found in the labeling available at Drugs@FDA.
Blue text represents the most recent addtions and/or changes since last posted version.

NDA/ANDA/BLA Number, Label Version Date	Drug	Therapeutic Area*	Biomarker†	Labeling Sections	Labeling Text‡
					• HR-positive, HER2-negative advanced or metastatic breast cancer in combination with palbociclib or abemaciclib in women with disease progression after endocrine therapy.<br><br>**6 ADVERSE REACTIONS**<br>**6.1 Clinical Trials Experience**<br>Combination Therapy<br>Combination Therapy with Palbociclib (PALOMA-3)<br>The safety of FASLODEX 500 mg plus palbociclib 125 mg/day versus FASLODEX plus placebo was evaluated in PALOMA-3. The data described below reflect exposure to FASLODEX plus palbociclib in 345 out of 517 patients with HR-positive, HER2-negative advanced or metastatic breast cancer who received at least 1 dose of treatment in PALOMA-3. The median duration of treatment for FASLODEX plus palbociclib was 10.8 months while the median duration of treatment for FASLODEX plus placebo arm was 4.8 months. (…)<br>Combination Therapy with Abemaciclib (MONARCH 2)<br>The safety of FASLODEX (500 mg) plus abemaciclib (150 mg twice daily) versus FASLODEX plus placebo was evaluated in MONARCH 2. The data described below reflect exposure to FASLODEX in 664 patients with HR-positive, HER2-negative advanced breast cancer who received at least one dose of FASLODEX plus abemaciclib or placebo in MONARCH 2. (…)<br>Combination Therapy with Ribociclib (MONALEESA-3)<br>The safety of FASLODEX 500 mg plus ribociclib 600 mg versus FASLODEX plus placebo was evaluated in MONALEESA-3. The data described below reflect exposure to FASLODEX plus ribociclib in 483 out of 724 postmenopausal patients with HR-positive, HER2-negative advanced or metastatic breast cancer for initial endocrine based therapy or after disease progression on endocrine therapy who received at least one dose of FASLODEX plus ribociclib or placebo in MONALEESA-3. Median duration of treatment was 15.8 months for FASLODEX plus ribociclib and 12 months for FASLODEX plus placebo. (…)<br><br>**14 CLINICAL STUDIES**<br>Comparison of FASLODEX 500 mg and Anastrozole 1 mg (FALCON)<br>A randomized, double-blind, double-dummy, multi-center study (FALCON, NCT01602380) of FASLODEX 500 mg versus anastrozole 1 mg was conducted in postmenopausal women with ER-positive and/or PgR-positive, HER2-negative locally advanced or metastatic breast cancer who had not previously been treated with any hormonal therapy. (…)<br>Combination Therapy<br>Patients with HR-positive, HER2-negative advanced or metastatic breast cancer who have had disease progression on or after prior adjuvant or metastatic endocrine therapy<br>FASLODEX 500 mg in Combination with Palbociclib 125 mg (PALOMA-3)<br>PALOMA-3 (NCT-1942135) was an international, randomized, double-blind, parallel group, multi-center study of FASLODEX plus palbociclib versus FASLODEX plus placebo conducted in women with HRpositive, HER2-negative advanced breast cancer, regardless of their menopausal status, whose disease progressed on or after prior endocrine therapy. (…)<br>FASLODEX 500 mg in Combination with Abemaciclib 150 mg (MONARCH 2)<br>MONARCH 2 (NCT02107703) was a randomized, placebo-controlled, multi-center study conducted in women with HR-positive, HER2-negative metastatic breast cancer with disease progression following endocrine therapy treated with FASLODEX plus abemaciclib versus FASLODEX plus placebo. (…)<br>Postmenopausal women with HR-positive, HER2-negative advanced or metastatic breast cancer for initial endocrine based therapy or after disease progression on endocrine therapy<br>FASLODEX 500 mg in Combination with Ribociclib 600 mg (MONALEESA-3)<br>MONALEESA-3 (NCT 02422615) was a randomized double-blind, placebo-controlled study of FASLODEX plus ribociclib versus FASLODEX plus placebo conducted in postmenopausal women with hormone receptor positive, HER2-negative, advanced breast cancer who have received no or only one line of prior endocrine treatment. (…)
**021344, 05/13/2020**	Fulvestrant (2)	Oncology	ESR, PGR (Hormone Receptor)	Indications and Usage, Adverse Reactions, Clinical Pharmacology, Clinical Studies	**1 INDICATIONS AND USAGE**<br>Monotherapy<br>FASLODEX is indicated for the treatment of:<br>• Hormone receptor (HR)-positive, human epidermal growth factor receptor 2 (HER2)-negative advanced breast cancer in postmenopausal women not previously treated with endocrine therapy, or<br>• HR-positive advanced breast cancer in postmenopausal women with disease progression following endocrine therapy.<br>Combination Therapy<br>FASLODEX is indicated for the treatment of:<br>• HR-positive, HER2-negative advanced or metastatic breast cancer in postmenopausal women in combination with ribociclib as initial endocrine based therapy or following disease progression on endocrine therapy.<br>• HR-positive, HER2-negative advanced or metastatic breast cancer in combination with palbociclib or abemaciclib in women with disease progression after endocrine therapy.<br><br>**6 ADVERSE REACTIONS**<br>**6.1 Clinical Trials Experience**<br>Comparison of FASLODEX 500 mg and Anastrozole 1 mg (FALCON)

* Therapeutic areas do not necessarily reflect the CDER review division.
† Representative biomarkers are listed based on standard nomenclature as per the Human Genome Organization (HUGO) symbol and/or simplified descriptors using other common conventions. Listed biomarkers do not necessarily reflect the terminology used in labeling. The term “Nonspecific” is provided when labeling does not explicitly identify the specific biomarker(s) or when the biomarker is represented by a molecular phenotype or gene signature, and in some cases the biomarker was inferred based on the labeling language.
‡ Referenced figures and tables may be found in the labeling available at Drugs@FDA.
Blue text represents the most recent addtions and/or changes since last posted version.

Table of Pharmacogenomic Biomarkers in Drug Labeling
Last Updated: 06/2024

NDA/ANDA/BLA Number, Label Version Date	Drug	Therapeutic Area*	Biomarker†	Labeling Sections	Labeling Text‡
					The safety of FASLODEX 500 mg versus anastrozole 1 mg was evaluated in FALCON. The data described below reflect exposure to FASLODEX in 228 out of 460 patients with HR-positive advanced breast cancer in postmenopausal women not previously treated with endocrine therapy who received at least one (1) dose of treatment in FALCON. (…)<br>Combination Therapy<br>Combination Therapy with Palbociclib (PALOMA-3)<br>The safety of FASLODEX 500 mg plus palbociclib 125 mg/day versus FASLODEX plus placebo was evaluated in PALOMA-3. The data described below reflect exposure to FASLODEX plus palbociclib in 345 out of 517 patients with HR-positive, HER2-negative advanced or metastatic breast cancer who received at least 1 dose of treatment in PALOMA-3. The median duration of treatment for FASLODEX plus palbociclib was 10.8 months while the median duration of treatment for FASLODEX plus placebo arm was 4.8 months. (…)<br>Combination Therapy with Abemaciclib (MONARCH 2)<br>The safety of FASLODEX (500 mg) plus abemaciclib (150 mg twice daily) versus FASLODEX plus placebo was evaluated in MONARCH 2. The data described below reflect exposure to FASLODEX in 664 patients with HR-positive, HER2-negative advanced breast cancer who received at least one dose of FASLODEX plus abemaciclib or placebo in MONARCH 2. (…)<br>Combination Therapy with Ribociclib (MONALEESA-3)<br>The safety of FASLODEX 500 mg plus ribociclib 600 mg versus FASLODEX plus placebo was evaluated in MONALEESA-3. The data described below reflect exposure to FASLODEX plus ribociclib in 483 out of 724 postmenopausal patients with HR-positive, HER2-negative advanced or metastatic breast cancer for initial endocrine based therapy or after disease progression on endocrine therapy who received at least one dose of FASLODEX plus ribociclib or placebo in MONALEESA-3. Median duration of treatment was 15.8 months for FASLODEX plus ribociclib and 12 months for FASLODEX plus placebo. (…)<br><br>**12 CLINICAL PHARMACOLOGY**<br>**12.2 Pharmacodynamics**<br>In a clinical study in postmenopausal women with primary breast cancer treated with single doses of FASLODEX 15-22 days prior to surgery, there was evidence of increasing down-regulation of ER with increasing dose. This was associated with a dose-related decrease in the expression of the progesterone receptor, an estrogen-regulated protein. These effects on the ER pathway were also associated with a decrease in Ki67 labeling index, a marker of cell proliferation.<br><br>**14 CLINICAL STUDIES**<br>Combination Therapy<br>Patients with HR-positive, HER2-negative advanced or metastatic breast cancer who have had disease progression on or after prior adjuvant or metastatic endocrine therapy<br>FASLODEX 500 mg in Combination with Palbociclib 125 mg (PALOMA-3)<br>PALOMA-3 (NCT-1942135) was an international, randomized, double-blind, parallel group, multi-center study of FASLODEX plus palbociclib versus FASLODEX plus placebo conducted in women with HRpositive, HER2-negative advanced breast cancer, regardless of their menopausal status, whose disease progressed on or after prior endocrine therapy. (…)<br>FASLODEX 500 mg in Combination with Abemaciclib 150 mg (MONARCH 2)<br>MONARCH 2 (NCT02107703) was a randomized, placebo-controlled, multi-center study conducted in women with HR-positive, HER2-negative metastatic breast cancer with disease progression following endocrine therapy treated with FASLODEX plus abemaciclib versus FASLODEX plus placebo. (…)<br>Postmenopausal women with HR-positive, HER2-negative advanced or metastatic breast cancer for initial endocrine based therapy or after disease progression on endocrine therapy<br>FASLODEX 500 mg in Combination with Ribociclib 600 mg (MONALEESA-3)<br>MONALEESA-3 (NCT 02422615) was a randomized double-blind, placebo-controlled study of FASLODEX plus ribociclib versus FASLODEX plus placebo conducted in postmenopausal women with hormone receptor positive, HER2-negative, advanced breast cancer who have received no or only one line of prior endocrine treatment. (…)
214801, 09/30/2022	**Futibatinib**	Oncology	FGFR2	Indications and Usage, Dosage and Administration, Adverse Reactions, Clinical Studies	**1 INDICATIONS AND USAGE**<br>LYTGOBI is indicated for the treatment of adult patients with previously treated, unresectable, locally advanced or metastatic intrahepatic cholangiocarcinoma harboring fibroblast growth factor receptor 2 (FGFR2) gene fusions or other rearrangements [see Dosage and Administration (2.1)].<br><br>**2 DOSAGE AND ADMINISTRATION**<br>**2.1 Patient Selection**<br>Select patients for the treatment of unresectable, locally advanced or metastatic intrahepatic cholangiocarcinoma with LYTGOBI based on the presence of an FGFR2 gene fusion or rearrangement [see Clinical Studies (14.1)]. An FDA-approved test for detection of FGFR2 gene fusions or other rearrangements in patients with unresectable, locally advanced, or metastatic intrahepatic cholangiocarcinoma for selecting patients for treatment with LYTGOBI is not available.<br><br>**6 ADVERSE REACTIONS**<br>Previously Treated, Unresectable Locally Advanced or Metastatic Intrahepatic Cholangiocarcinoma<br>The safety of LYTGOBI was evaluated in Study TAS-120-101, which included 103 patients with previously treated, unresectable locally advanced or metastatic intrahepatic cholangiocarcinoma harboring FGFR2 fusions or other gene rearrangements [see Clinical Studies (14.1)]. Patients were treated with LYTGOBI 20 mg orally once daily until disease progression or unacceptable toxicity. The median duration of treatment was 9 months (range: 0.5 - 25 months). (…)<br><br>**14 CLINICAL STUDIES**

* Therapeutic areas do not necessarily reflect the CDER review division.
† Representative biomarkers are listed based on standard nomenclature as per the Human Genome Organization (HUGO) symbol and/or simplified descriptors using other common conventions. Listed biomarkers do not necessarily reflect the terminology used in labeling. The term “Nonspecific” is provided when labeling does not explicitly identify the specific biomarker(s) or when the biomarker is represented by a molecular phenotype or gene signature, and in some cases the biomarker was inferred based on the labeling language.
‡ Referenced figures and tables may be found in the labeling available at Drugs@FDA.
Blue text represents the most recent addtions and/or changes since last posted version.

NDA/ANDA/BLA Number, Label Version Date	Drug	Therapeutic Area*	Biomarker†	Labeling Sections	Labeling Text‡
					**14.1 Cholangiocarcinoma**<br>TAS-120-101 (NCT02052778), a multicenter, open-label, single-arm trial, evaluated the efficacy of LYTGOBI in 103 patients with previously treated, unresectable, locally advanced or metastatic intrahepatic cholangiocarcinoma. The presence of FGFR2 fusions or other rearrangements was determined in 102 enrolled patients (99%) using next generation sequencing (NGS) testing. Qualifying in-frame fusions and other rearrangements were predicted to have a breakpoint within intron 17/exon 18 of the FGFR2 gene leaving the FGFR2 kinase domain intact.<br>Patients received LYTGOBI at a dosage of 20 mg orally once daily until disease progression or unacceptable toxicity. The major efficacy outcome measures were overall response rate (ORR) and duration of response (DoR) as determined by an independent review committee (IRC) according to Response Evaluation Criteria in Solid Tumors (RECIST) v1.1.<br>The trial population characteristics were: Median age was 58 years (range: 22 to 79 years) with 22% of patients ≥65 years, 56% were female, race was: 50% White, 29% Asian, 8% Black or African American, 1% Native Hawaiian or Other Pacific Islander, 13% unknown, baseline Eastern Cooperative Oncology Group (ECOG) performance status of 0 (47%) or 1 (53%). Seventy-eight percent (78%) of patients had in-frame FGFR2 gene fusions and the most commonly identified FGFR2 fusion partner was BICC1 (n=24, 23%). Twenty-two percent (22%) of patients had other FGFR2 rearrangements that may not be in-frame with the partner gene or the partner gene was not identifiable. (…)
**021169, 02/14/2017**	Galantamine	Neurology	CYP2D6	Clinical Pharmacology	**12 CLINICAL PHARMACOLOGY**<br>**12.3 Pharmacokinetics**<br>*Metabolism and Elimination*<br>Galantamine is metabolized by hepatic cytochrome P450 enzymes, glucuronidated, and excreted unchanged in the urine. In vitro studies indicate that cytochrome CYP2D6 and CYP3A4 were the major cytochrome P450 isoenzymes involved in the metabolism of galantamine, and inhibitors of both pathways increase oral bioavailability of galantamine modestly. O-demethylation, mediated by CYP2D6 was greater in extensive metabolizers of CYP2D6 than in poor metabolizers. In plasma from both poor and extensive metabolizers, however, unchanged galantamine and its glucuronide accounted for most of the sample radioactivity.<br>In studies of oral 3 H-galantamine, unchanged galantamine and its glucuronide, accounted for most plasma radioactivity in poor and extensive CYP2D6 metabolizers. Up to 8 hours post-dose, unchanged galantamine accounted for 39-77% of the total radioactivity in the plasma, and galantamine glucuronide for 14-24%. By 7 days, 93-99% of the radioactivity had been recovered, with about 95% in urine and about 5% in the feces. Total urinary recovery of unchanged galantamine accounted for, on average, 32% of the dose and that of galantamine glucuronide for another 12% on average. (…)<br>(…) RAZADYNE® ER 24 mg extended-release capsules administered once daily under fasting conditions are bioequivalent to RAZADYNE® tablets 12 mg twice daily with respect to AUC24h and Cmin. The Cmax and Tmax of the extended-release capsules were lower and occurred later, respectively, compared with the immediate-release tablets, with Cmax about 25% lower and median Tmax occurring about 4.5–5.0 hours after dosing. Dose-proportionality is observed for RAZADYNE® ER extended-release capsules over the dose range of 8 to 24 mg daily and steady state is achieved within a week. There was no effect of age on the pharmacokinetics of RAZADYNE® ER extended-release capsules. CYP2D6 poor metabolizers had drug exposures that were approximately 50% higher than for extensive metabolizers. (…)<br>*CYP2D6 Poor Metabolizers*<br>Approximately 7% of the normal population has a genetic variation that leads to reduced levels of activity of CYP2D6 isozyme. Such individuals have been referred to as poor metabolizers. After a single oral dose of 4 mg or 8 mg galantamine, CYP2D6 poor metabolizers demonstrated a similar Cmax and about 35% AUC∞ increase of unchanged galantamine compared to extensive metabolizers. A total of 356 patients with Alzheimer's disease enrolled in two Phase 3 studies were genotyped with respect to CYP2D6 (n=210 hetero-extensive metabolizers, 126 homo-extensive metabolizers, and 20 poor metabolizers). Population pharmacokinetic analysis indicated that there was a 25% decrease in median clearance in poor metabolizers compared to extensive metabolizers. Dosage adjustment is not necessary in patients identified as poor metabolizers as the dose of drug is individually titrated to tolerability.
**215904, 03/18/2022**	Ganaxolone	Neurology	CDKL5	Indications and Usage, Clinical Studies	**1 INDICATIONS AND USAGE**<br>ZTALMY is indicated for the treatment of seizures associated with cyclin-dependent kinase-like 5 (CDKL5) deficiency disorder (CDD) in patients 2 years of age and older.<br><br>**14 CLINICAL STUDIES**<br>The effectiveness of ZTALMY for the treatment of seizures associated with CDD in patients 2 years of age and older was established in a single, double-blind, randomized, placebo-controlled study in patients 2 to 19 years of age (Study 1, NCT03572933).<br>Patients enrolled in Study 1 (N=50 for ZTALMY; N=51 for placebo) had molecular confirmation of a pathogenic or likely pathogenic mutation in the CDKL5 gene, seizures inadequately controlled by at least 2 previous treatment regimens, and a minimum of 16 major motor seizures (i.e., bilateral tonic, generalized tonic-clonic, bilateral clonic, atonic, focal to bilateral tonic-clonic) per 28 days during a retrospective 2-month period prior to screening.
**062196**	Gentamicin	Infectious Diseases	MT-RNR1	Warnings	Labeling not electronically available on Drugs@FDA
**206995, 08/22/2018**	Gefitinib (1)	Oncology	EGFR	Indications and Usage, Dosage and Administration, Clinical Studies	**1 INDICATIONS AND USAGE**<br>IRESSA is indicated for the first-line treatment of patients with metastatic non-small cell lung cancer (NSCLC) whose tumors have epidermal growth factor receptor (EGFR) exon 19 deletions or exon 21 (L858R) substitution mutations as detected by an FDA-approved test [see Clinical Studies (14)].<br>*Limitation of Use:* Safety and efficacy of IRESSA have not been established in patients with metastatic NSCLC whose tumors have EGFR mutations other than exon 19 deletions or exon 21 (L858R) substitution mutations [see Clinical Studies (14)].<br><br>**2 DOSAGE AND ADMINISTRATION**<br>**2.1 Patient Selection**

* Therapeutic areas do not necessarily reflect the CDER review division.
† Representative biomarkers are listed based on standard nomenclature as per the Human Genome Organization (HUGO) symbol and/or simplified descriptors using other common conventions. Listed biomarkers do not necessarily reflect the terminology used in labeling. The term "Nonspecific" is provided when labeling does not explicitly identify the specific biomarker(s) or when the biomarker is represented by a molecular phenotype or gene signature, and in some cases the biomarker was inferred based on the labeling language.
‡ Referenced figures and tables may be found in the labeling available at Drugs@FDA.
Blue text represents the most recent addtions and/or changes since last posted version.

Table of Pharmacogenomic Biomarkers in Drug Labeling
Last Updated: 06/2024

NDA/ANDA/BLA Number, Label Version Date	Drug	Therapeutic Area*	Biomarker†	Labeling Sections	Labeling Text‡
					Select patients for the first-line treatment of metastatic NSCLC with IRESSA based on the presence of EGFR exon 19 deletion or exon 21 (L858R) substitution mutations in their tumor [see Indications and Usage (1), Clinical Studies (14)]. Information on FDA-approved tests for the detection of EGFR mutations in NSCLC is available at: http://www.fda.gov/CompanionDiagnostics.<br><br>**14 CLINICAL STUDIES**<br>*Non-Small Cell Lung Cancer (NSCLC)*<br>*Study 1*<br>The efficacy and safety of IRESSA for the first-line treatment of patients with metastatic NSCLC containing EGFR exon 19 deletions or L858R substitution mutations was demonstrated in a multicenter, single-arm, open-label clinical study (Study 1). A total of 106 treatment-naive patients with metastatic EGFR mutation positive NSCLC received IRESSA at a dose of 250 mg once daily until disease progression or intolerable toxicity. The major efficacy outcome measure was objective response rate (ORR) according to RECIST v1.1 as evaluated by both a Blinded Independent Central Review (BICR) and investigators. Duration of response (DOR) was an additional outcome measure. Eligible patients were required to have a deletion in EGFR exon 19 or L858R, L861Q, or G719X substitution mutation and no T790M or S768I mutation or exon 20 insertion in tumor specimens as prospectively determined by a clinical trial assay. Tumor samples from 87 patients were tested retrospectively using the therascreen® EGFR RGQ PCR Kit.<br>The study population characteristics were: median age 65 years, age 75 years or older (25%), age less than 65 years (49%), white (100%), female (71%), never smokers (64%), WHO PS 0 (45%), WHO PS 1 (48%), WHO PS 2 (7%), and adenocarcinoma histology (97%). Sixty patients had exon 19 deletions (65%), 29 patients had L858R substitution (31%), while two patients each had tumors harboring L861Q or G719X substitution mutation. The median duration of treatment was 8.0 months. (See Table 3)<br>The response rates were similar in patients whose tumors had EGFR exon 19 deletions and exon 21 L858R substitution mutations. Two partial responses were observed in both patients whose tumors had G719X substitution mutation with duration of response of at least 2.8 months and 5.6 months, respectively. One of two patients whose tumors had L861Q substitution mutation also achieved a partial response with duration of response of at least 2.8 months.<br>*Study 2*<br>The results of Study 1 were supported by an exploratory analysis of a subset of a randomized, multicenter, open-label trial (Study 2) conducted in patients with metastatic adenocarcinoma histology NSCLC receiving first-line treatment. Patients were randomized (1:1) to receive IRESSA 250 mg orally once daily or up to 6 cycles of carboplatin/paclitaxel. The efficacy outcomes included progression-free survival (PFS) and objective response rate (ORR) as assessed by BICR.<br>The subset population consisted of 186 of 1217 patients (15%) determined to be EGFR positive by the same clinical trial assay as used in Study 1 and had radiographic scans available for a retrospective assessment by BICR. In this subset, there were 88 IRESSA-treated patients and 98 carboplatin/paclitaxel-treated patients. (…)
**206995, 08/22/2018**	Gefitinib (2)	Oncology	CYP2D6	Clinical Pharmacology	**12 CLINICAL PHARMACOLOGY**<br>CYP2D6 Poor metabolizer:<br>CYP2D6 metabolizes gefitinib to O-desmethyl gefitinib in vitro. In healthy CYP2D6 poor metabolizers, O-desmethyl gefitinib concentration was not measurable and the mean exposure to gefitinib was 2-fold higher as compared to the extensive metabolizers. This increase in exposure in CYP2D6 poor metabolizers may be clinically important because some adverse drug reactions are related to higher exposure of gefitinib. No dose adjustment is recommended in patients with a known CYP2D6 poor metabolizer genotype, but these patients should be closely monitored for adverse reactions. The impact of CYP2D6 inhibiting drugs on gefitinib pharmacokinetics has not been evaluated. However, similar precautions should be used when administering CYP2D6 inhibitors with IRESSA because of the possibility of increased exposure in these patients.<br>An exploratory exposure response analysis showed an increase in the incidence of interstitial lung disease (ILD) with a greater than 2 fold increase in the gefitinib exposure [see Warnings and Precautions (5.1)].
**761060, 06/16/2020**	Gemtuzumab Ozogamicin	Oncology	CD33	Indications and Usage, Dosage and Administration, Adverse Reactions, Clinical Studies	**1 INDICATIONS AND USAGE**<br>**1.1 Newly-Diagnosed CD33-positive Acute Myeloid Leukemia (AML)**<br>MYLOTARG is indicated for the treatment of newly-diagnosed CD33-positive acute myeloid leukemia in adults and pediatric patients 1 month and older.<br>**1.2 Relapsed or Refractory CD33-positive AML**<br>MYLOTARG is indicated for the treatment of relapsed or refractory CD33-positive acute myeloid leukemia in adults and pediatric patients 2 years and older.<br><br>**2 DOSAGE AND ADMINISTRATION**<br>**2.2 Recommended Dosage**<br>Newly-Diagnosed De Novo CD33-positive AML (Combination Regimen)<br>*Adults*<br>The recommended dose of MYLOTARG in adults is 3 mg/m2. A treatment course including MYLOTARG in combination therapy for adults with newly-diagnosed de novo CD33-positive AML consists of 1 induction cycle and 2 consolidation cycles [see Clinical Studies (14.1)]. (...)<br>Newly-Diagnosed CD33-positive AML (Single-agent Regimen)<br>A treatment course of MYLOTARG as a single agent for adults with newly-diagnosed CD33-positive AML consists of 1 cycle of induction and up to 8 cycles of continuation therapy [see Clinical Studies (14.1)]. (…)<br>Relapsed or Refractory CD33-positive AML (Single-agent Regimen)<br>The recommended dose of MYLOTARG as a single agent for treatment for adults and pediatric patients 2 years and older with relapsed or refractory CD33-positive AML is 3 mg/m2 (up to one 4.5 mg vial) on Days 1, 4, and 7. Treatment in the relapsed or refractory setting consists of a single course of MYLOTARG [see Clinical Studies (14.2)]. (…)

* Therapeutic areas do not necessarily reflect the CDER review division.
† Representative biomarkers are listed based on standard nomenclature as per the Human Genome Organization (HUGO) symbol and/or simplified descriptors using other common conventions. Listed biomarkers do not necessarily reflect the terminology used in labeling. The term “Nonspecific” is provided when labeling does not explicitly identify the specific biomarker(s) or when the biomarker is represented by a molecular phenotype or gene signature, and in some cases the biomarker was inferred based on the labeling language.
‡ Referenced figures and tables may be found in the labeling available at Drugs@FDA.
Blue text represents the most recent addtions and/or changes since last posted version.

NDA/ANDA/BLA Number, Label Version Date	Drug	Therapeutic Area*	Biomarker†	Labeling Sections	Labeling Text‡
					**6 ADVERSE REACTIONS**<br>**6.1 Clinical Trials Experience**<br>Combination Therapy in Newly-Diagnosed De Novo CD33-positive AML<br>The safety of MYLOTARG in first-line combination therapy was evaluated in two prospective clinical trials, Study ALFA-0701 in adults and Study AAML0531 in pediatric patients. (…)<br>Monotherapy for Newly-Diagnosed CD33-positive AML<br>The safety evaluation of MYLOTARG (6 mg/m2 then 3 mg/m2 , with 7 days between the doses) as monotherapy is based on a randomized, open-label, Phase 3 trial of MYLOTARG (N=118) versus best supportive care (BSC) (N=119) in patients with previously untreated AML who were considered ineligible for intensive chemotherapy in Study AML-19 [see Clinical Studies (14.1)]. (…)<br>Monotherapy for Relapsed or Refractory CD33-positive AML<br>The adverse reactions described in this section reflect exposure to MYLOTARG 3 mg/m2 on Days 1, 4 and 7 as monotherapy in 57 patients with relapsed AML treated on MyloFrance-1 [see Clinical Studies (14.1)]. All 57 (100%) patients received the 3 planned doses of MYLOTARG. (…)<br><br>**14 CLINICAL STUDIES**<br>**14.1 Newly-Diagnosed CD33-positive AML**<br>Study ALFA-0701<br>(…) CD33 expression on AML blasts by flow cytometry harmonized from local laboratory results was determined in 194/271 (72%) patients overall. Few patients (14%) had low CD33 expression (less than 30% of blasts), and none had no expression of CD33. (…)<br>Study AML-19<br>(…) Patients were randomized 1:1 and stratified by age (61-75 vs 76-80 years vs ≥81 years), CD33 positivity of bone marrow blasts (less than 20 % vs 20-80% vs greater than 80% vs unknown), initial white blood cell count (less than 30 vs greater than or equal to 30 x 109 /L), WHO PS (0-1 vs 2 vs 3-4), and institution. (…)<br>(…) Fewer patients on the MYLOTARG arm had missing cytogenetics data (22% vs 35%). CD33 expression on AML blasts by flow cytometry at a centralized location was determined in 235/237 (99%) patients; 10% had CD33 expression less than 20%. (…)<br>**14.2 Relapsed or refractory CD33-positive AML**<br>Study MyloFrance-1<br>The efficacy of MYLOTARG as a single agent was evaluated in MyloFrance-1 a phase 2, single-arm, open-label study in adults with CD33-positive AML in first relapse. (…)
**211349, 05/29/2019**	Gilteritinib	Oncology	FLT3	Indications and Usage, Dosage and Administration, Clinical Studies	**1 INDICATIONS AND USAGE**<br>**1.1 Relapsed or Refractory Acute Myeloid Leukemia**<br>XOSPATA is indicated for the treatment of adult patients who have relapsed or refractory acute myeloid leukemia (AML) with a FMS-like tyrosine kinase 3 (FLT3) mutation as detected by an FDA-approved test.<br><br>**2 DOSAGE AND ADMINISTRATION**<br>**2.1 Patient Selection**<br>Select patients for the treatment of AML with XOSPATA based on the presence of FLT3 mutations in the blood or bone marrow [see Clinical Studies (14)]. Information on FDA-approved tests for the detection of a FLT3 mutation in AML is available at http://www.fda.gov/CompanionDiagnostics.<br><br>**14 CLINICAL STUDIES**<br>**14.1 Relapsed or Refractory Acute Myeloid Leukemia**<br>The efficacy of XOSPATA was assessed in the ADMIRAL trial (NCT02421939), which included 138 adult patients with relapsed or refractory AML having a FLT3 ITD, D835, or I836 mutation by the LeukoStrat CDx FLT3 Mutation Assay. (See Table 4) (…)<br>(…) For patients who achieved a CR/CRh, the median time to first response was 3.6 months (range, 0.9 to 9.6 months). The CR/CRh rate was 29 of 126 in patients with FLT3-ITD or FLT3-ITD/TKD and 0 of 12 in patients with FLT3-TKD only. (See Table 6) (…)<br>(…) In the final analysis, the CR/CRh rate in the gilteritinib arm was 22.6% (55/243) and the DOR was 7.4 months (range, <0.1 + to 23.1+). For patients who achieved a CR/CRh, the median time to first response was 2 months (range, 0.9 to 9.6 months). The CR/CRh rate was 49 of 215 in patients with FLT3-ITD only, 3 of 7 in patients with FLT3-ITD/TKD and 3 of 21 in patients with FLT3-TKD only. (…)
**212194, 11/20/2019**	Givosiran	Gastroenterology	CPOX, HMBS, PPOX (Acute Hepatic Porphyria)	Clinical Studies	**14 CLINICAL STUDIES**<br>The efficacy of GIVLAARI in patients with acute hepatic porphyria was evaluated in the ENVISION trial (NCT03338816), a randomized, double-blind, placebo-controlled, multinational study.<br>ENVISION enrolled 94 patients with acute hepatic porphyria (AHP) (89 patients with AIP, 2 patients with variegate porphyria [VP], 1 patient with hereditary coproporphyria [HCP], and 2 patients with no identified mutation). (…)
**020496, 12/21/2018**	Glimepiride	Endocrinology	G6PD	Warnings and Precautions,	**5 WARNINGS AND PRECAUTIONS**<br>**5.3 Hemolytic Anemia**

* Therapeutic areas do not necessarily reflect the CDER review division.
† Representative biomarkers are listed based on standard nomenclature as per the Human Genome Organization (HUGO) symbol and/or simplified descriptors using other common conventions. Listed biomarkers do not necessarily reflect the terminology used in labeling. The term “Nonspecific” is provided when labeling does not explicitly identify the specific biomarker(s) or when the biomarker is represented by a molecular phenotype or gene signature, and in some cases the biomarker was inferred based on the labeling language.
‡ Referenced figures and tables may be found in the labeling available at Drugs@FDA.
Blue text represents the most recent addtions and/or changes since last posted version.

NDA/ANDA/BLA Number, Label Version Date	Drug	Therapeutic Area*	Biomarker†	Labeling Sections	Labeling Text‡
				Adverse Reactions	Sulfonylureas can cause hemolytic anemia in patients with glucose 6-phosphate dehydrogenase (G6PD) deficiency. Because AMARYL is a sulfonylurea, use caution in patients with G6PD deficiency and consider the use of a non-sulfonylurea alternative. There are also postmarketing reports of hemolytic anemia in patients receiving AMARYL who did not have known G6PD deficiency [see Adverse Reactions (6.2)].<br><br>**6 ADVERSE REACTIONS**<br>**6.2 Postmarketing Experience**<br>The following adverse reactions have been identified during post-approval use of AMARYL. Because these reactions are reported voluntarily from a population of uncertain size, it is not always possible to reliably estimate their frequency or establish a causal relationship to drug exposure. (…)<br>• Hemolytic anemia in patients with and without G6PD deficiency [see Warnings and Precautions (5.3)] (…)
**017783, 08/18/2016**	Glipizide	Endocrinology	G6PD	Precautions	**PRECAUTIONS**<br>*Hemolytic Anemia*<br>Treatment of patients with glucose 6-phosphate dehydrogenase (G6PD) deficiency with sulfonylurea agents can lead to hemolytic anemia. Because GLUCOTROL belongs to the class of sulfonylurea agents, caution should be used in patients with G6PD deficiency and a non-sulfonylurea alternative should be considered. In post-marketing reports, hemolytic anemia has also been reported in patients who did not have known G6PD deficiency.
**020051, 08/22/2017**	Glyburide	Endocrinology	G6PD	Precautions	**PRECAUTIONS**<br>*Hemolytic Anemia*<br>Treatment of patients with glucose 6-phosphate dehydrogenase (G6PD) deficiency with sulfonylurea agents can lead to hemolytic anemia. Because GLYNASE PresTab belongs to the class of sulfonylurea agents, caution should be used in patients with G6PD deficiency and a non-sulfonylurea alternative should be considered. In post marketing reports, hemolytic anemia has also been reported in patients who did not have known G6PD deficiency.
**203284, 09/03/2021**	Glycerol phenylbutyrate (1)	Inborn Errors of Metabolism	ASS1, CPS1, OTC (Urea Cycle Disorders)	Indications and Usage, Adverse Reactions, Clinical Studies	**1 INDICATIONS AND USAGE**<br>RAVICTI is indicated for use as a nitrogen-binding agent for chronic management of patients with urea cycle disorders (UCDs) who cannot be managed by dietary protein restriction and/or amino acid supplementation alone. RAVICTI must be used with dietary protein restriction and, in some cases, dietary supplements (e.g., essential amino acids, arginine, citrulline, protein-free calorie supplements).<br>Limitations of Use:<br>• RAVICTI is not indicated for the treatment of acute hyperammonemia in patients with UCDs because more rapidly acting interventions are essential to reduce plasma ammonia levels.<br><br>**6 ADVERSE REACTIONS**<br>Assessment of adverse reactions was based on exposure of 45 adult patients (31 female and 14 male) with UCD subtype deficiencies of ornithine transcarbamylase (OTC, n=40), carbamoyl phosphate synthetase (CPS, n=2), and argininosuccinate synthetase (ASS, n=1) in a randomized, double-blind, active-controlled (RAVICTI vs sodium phenylbutyrate), crossover, 4-week study (Study 1) that enrolled patients 18 years of age and older [see Clinical Studies (14.1)]. One of the 45 patients received only sodium phenylbutyrate prior to withdrawing on day 1 of the study due to an adverse reaction.<br><br>**14 CLINICAL STUDIES**<br>**14.1 Clinical Studies in Adult Patients with UCDs**<br>Active-Controlled, 4-Week, Noninferiority Study (Study 1)<br>A randomized, double-blind, active-controlled, crossover, noninferiority study (Study 1) compared RAVICTI to sodium phenylbutyrate by evaluating ammonia levels in patients with UCDs who had been on sodium phenylbutyrate prior to enrollment for control of their UCD. Patients were required to have a confirmed diagnosis of UCD involving deficiencies of CPS, OTC, or ASS, confirmed via enzymatic, biochemical, or genetic testing. (…)<br>Demographic characteristics of the 45 patients enrolled in Study 1 were as follows: mean age at enrollment was 33 years (range: 18 to 75 years); 69% were female; 33% had adult-onset disease; 89% had OTC deficiency; 7% had ASS deficiency; 4% had CPS deficiency.<br>**14.2 Clinical Studies in Pediatric Patients 2 Years to 17 Years of Age with UCDs**<br>UCD subtypes included OTC (n=12), ASL (n=8), and ASS deficiency (n=2), and patients received a mean RAVICTI dose of 8 mL/m2 /day (8.8 g/m2 /day), with doses ranging from 1.4 to 13.1 mL/m2 /day (1.5 to 14.4 g/m2 /day). Doses in these patients were based on previous dosing of sodium phenylbutyrate.<br>**14.3 Clinical Studies in Pediatric Patients Less Than 2 Years of Age with UCDs**<br>*Pediatric Patients Less than 2 Months of Age*<br>A total of 16 pediatric patients less than 2 months of age participated in Study 6. Median age at enrollment was 0.5 months (range: 0.1 to 2 months). Eight patients had OTC deficiency, 7 patients had ASS deficiency, and 1 patient had ASL deficiency. (…)
**203284, 09/03/2021**	Glycerol phenylbutyrate (2)	Inborn Errors of Metabolism	NAGS	Indications and Usage	**1 INDICATIONS AND USAGE**<br>Limitations of Use:<br>• The safety and efficacy of RAVICTI for the treatment of N-acetylglutamate synthase (NAGS) deficiency has not been established.
**211970, 12/12/2019**	Golodirsen	Neurology	DMD	Indications and Usage, Use in Specific Populations, Clinical Pharmacology, Clinical Studies	**1 INDICATIONS AND USAGE**<br>VYONDYS 53 is indicated for the treatment of Duchenne muscular dystrophy (DMD) in patients who have a confirmed mutation of the DMD gene that is amenable to exon 53 skipping.<br>This indication is approved under accelerated approval based on an increase in dystrophin production in skeletal muscle observed in patients treated with VYONDYS 53 [see Clinical Studies (14)]. Continued approval for this indication may be contingent upon verification of a clinical benefit in confirmatory trials.<br><br>**8 USE IN SPECIFIC POPULATIONS**<br>**8.4 Pediatric Use**

* Therapeutic areas do not necessarily reflect the CDER review division.
† Representative biomarkers are listed based on standard nomenclature as per the Human Genome Organization (HUGO) symbol and/or simplified descriptors using other common conventions. Listed biomarkers do not necessarily reflect the terminology used in labeling. The term “Nonspecific” is provided when labeling does not explicitly identify the specific biomarker(s) or when the biomarker is represented by a molecular phenotype or gene signature, and in some cases the biomarker was inferred based on the labeling language.
‡ Referenced figures and tables may be found in the labeling available at Drugs@FDA.
Blue text represents the most recent addtions and/or changes since last posted version.

NDA/ANDA/BLA Number, Label Version Date	Drug	Therapeutic Area*	Biomarker†	Labeling Sections	Labeling Text‡
					VYONDYS 53 is indicated for the treatment of Duchenne muscular dystrophy (DMD) in patients who have a confirmed mutation of the DMD gene that is amenable to exon 53 skipping, including pediatric patients [see Clinical Studies (14)]. (…)<br><br>**12 CLINICAL PHARMACOLOGY**<br>**12.2 Pharmacodynamics**<br>After treatment with VYONDYS 53, all patients evaluated (n=25) in Study 1 Part 2 [see Clinical Studies (14)] had an increase in skipping of exon 53 demonstrated by reverse transcription polymerase chain reaction (RT-PCR), compared to baseline. (…)<br><br>**14 CLINICAL STUDIES**<br>The effect of VYONDYS 53 on dystrophin production was evaluated in one study in DMD patients with a confirmed mutation of the DMD gene that is amenable to exon 53 skipping (Study 1; NCT02310906). (…)<br>(…) Study 1 Part 2 was a 168-week, open-label study assessing the efficacy and safety of VYONDYS 53 at a dose of 30 mg/kg/week in the 12 patients enrolled in Part 1, plus 13 additional treatmentnaive patients with DMD amenable to exon 53 skipping. (…)
**019726, 02/12/2015**	**Goserelin**	Oncology	ESR, PGR (Hormone Receptor)	Indications and Usage, Clinical Studies	**1 INDICATIONS AND USAGE**<br>**1.5 Advanced Breast Cancer**<br>ZOLADEX is indicated for use in the palliative treatment of advanced breast cancer in pre- and perimenopausal women.<br>The estrogen and progesterone receptor values may help to predict whether ZOLADEX therapy is likely to be beneficial [see Dosage and Administration (2.6), Clinical Pharmacology (12.1), and Clinical Studies (14.5)].<br><br>**14 CLINICAL STUDIES**<br>**14.5 Breast Cancer**<br>The Southwest Oncology Group conducted a prospective, randomized clinical trial (SWOG-8692 [INT-0075]) in premenopausal women with advanced estrogen receptor positive or progesterone receptor positive breast cancer which compared ZOLADEX with oophorectomy. (…)<br>(…) Findings were similar in uncontrolled clinical trials involving patients with hormone receptor positive and negative breast cancer. Premenopausal women with estrogen receptor (ER) status of positive, negative, or unknown participated in the uncontrolled (Phase II and Trial 2302) clinical trials. Objective tumor responses were seen regardless of ER status, as shown in the following table. (See Table 8)
**020727, 03/12/2019**	**Hydralazine**	Cardiology	Nonspecific (NAT)	Clinical Pharmacology	**12 CLINICAL PHARMACOLOGY**<br>**12.3 Pharmacokinetics**<br>*Absorption*<br>(…) Hydralazine hydrochloride: About 2/3 of a 50-mg dose of 14C-hydralazine hydrochloride given in gelatin capsules was absorbed in hypertensive subjects. In patients with heart failure, mean absolute bioavailability of a single oral dose of hydralazine 75 mg varies from 10 to 26%, with the higher percentages in slow acetylators. Administration of doses escalating from 75 mg to 1000 mg three times daily to congestive heart failure patients resulted in an up to 9-fold increase in the dose normalized AUC, indicating non-linear kinetics of hydralazine, probably reflecting saturable first pass metabolism. (…)<br>*Metabolism*<br>Hydralazine is metabolized by acetylation, ring oxidation and conjugation with endogenous compounds including pyruvic acid. Acetylation occurs predominantly during the first-pass after oral administration which explains the dependence of the absolute bioavailability on the acetylator phenotype. About 50% of patients are fast acetylators and have lower exposure. (…)
**009768, 05/03/2021**	**Hydroxychloroquine**	Infectious Diseases	G6PD	Warnings and Precautions, Adverse Reactions	**5 WARNINGS AND PRECAUTIONS**<br>**5.6 Hemolytic Anemia Associated with G6PD Deficiency**<br>Hemolysis has been reported in patients with glucose-6-phosphate dehydrogenase (G6PD) deficiency. Monitor for hemolytic anemia as this can occur, particularly in association with other drugs that cause hemolysis.<br><br>**ADVERSE REACTIONS**<br>Hemolytic Anemia Associated with G6PD
205552, 08/24/2022	**Ibrutinib (1)**	Oncology	Chromosome 17p	Indications and Usage, Clinical Studies	**1 INDICATIONS AND USAGE**<br>**1.3 Chronic Lymphocytic Leukemia/Small Lymphocytic Lymphoma with 17p deletion**<br>IMBRUVICA is indicated for the treatment of patients with chronic lymphocytic leukemia (CLL)/small lymphocytic lymphoma (SLL) with 17p deletion [see Clinical Studies (14.2)].<br><br>**14 CLINICAL STUDIES**<br>**14.2 Chronic Lymphocytic Leukemia / Small Lymphocytic Lymphoma**<br>*RESONATE*<br>(…) Thirty-two percent of patients had 17p deletion. (…)<br>*CLL/SLL with 17p deletion (del 17p CLL/SLL) in RESONATE*<br>RESONATE included 127 patients with del 17p CLL/SLL. The median age was 67 years (range, 30 to 84 years), 62% were male, and 88% were Caucasian. All patients had a baseline ECOG performance status of 0 or 1. PFS and ORR were assessed by IRC. Efficacy results for del 17p CLL/SLL are shown in Table 22. (See Table 22)<br>63-Month Follow-Up

NDA/ANDA/BLA Number, Label Version Date	Drug	Therapeutic Area*	Biomarker†	Labeling Sections	Labeling Text‡
					With an overall follow-up of 63 months, the median investigator-assessed PFS in patients with del 17p per IWCLL criteria was 40.6 months [95% CI (25.4, 44.6)] in the IMBRUVICA arm and 6.2 months [95% CI (4.6, 8.1)] in the ofatumumab arm, respectively. Overall response rate as assessed by investigators in patients with del 17p was 88.9% in the IMBRUVICA arm versus 18.8% in the ofatumumab arm.<br>*iLLUMINATE*<br>The iLLUMINATE study (a multi-center study of ibrutinib in combination with obinutuzumab versus chlorambucil in combination with obinutuzumab) (NCT02264574) was conducted in patients with treatment naïve CLL or SLL. Patients were 65 years of age or older or < 65 years of age with coexisting medical conditions, reduced renal function as measured by creatinine clearance < 70 mL/min, or presence of del 17p/TP53 mutation. (…)<br>The trial enrolled 214 patients with CLL and 15 patients with SLL. At baseline, 65% of patients presented with CLL/SLL with high risk factors (del 17p/TP53 mutation [18%], del 11q [15%], or unmutated immunoglobulin heavy-chain variable region (unmutated IGHV) [54%]). The most common reasons for initiating CLL therapy included: lymphadenopathy (38%), night sweats (34%), progressive marrow failure (31%), fatigue (29%), splenomegaly (25%), and progressive lymphocytosis (21%). (…)<br>In the high risk CLL/SLL population (del 17p/TP53 mutation, del 11q, or unmutated IGHV), the PFS HR was 0.15 [95% CI (0.09, 0.27)].
205552, 08/24/2022	Ibrutinib (2)	Oncology	Chromosome 11q	Clinical Studies	**14 CLINICAL STUDIES**<br>**14.2 Chronic Lymphocytic Leukemia / Small Lymphocytic Lymphoma**<br>*RESONATE-2*<br>(…) The trial enrolled 249 patients with CLL and 20 patients with SLL. At baseline, 20% of patients had 11q deletion. The most common reasons for initiating CLL therapy include: progressive marrow failure demonstrated by anemia and/or thrombocytopenia (38%), progressive or symptomatic lymphadenopathy (37%), progressive or symptomatic splenomegaly (30%), fatigue (27%) and night sweats (25%). (…)<br>*HELIOS*<br>(…) The median age was 64 years (range, 31 to 86 years), 66% were male, and 91% were Caucasian. All patients had a baseline ECOG performance status of 0 or 1. The median time since diagnosis was 5.9 years and the median number of prior treatments was 2 (range, 1 to 11 treatments). At baseline, 56% of patients had at least one tumor > 5 cm and 26% presented with del11q. (…)<br>The median age was 71 years (range, 40 to 87 years), 64% were male, and 96% were Caucasian. All patients had a baseline ECOG performance status of 0 (48%) or 1-2 (52%). The trial enrolled 214 patients with CLL and 15 patients with SLL. At baseline, 65% of patients presented with CLL/SLL with high risk factors (del 17p/TP53 mutation [18%], del 11q [15%], or unmutated immunoglobulin heavy-chain variable region (unmutated IGHV) [54%]). The most common reasons for initiating CLL therapy included: lymphadenopathy (38%), night sweats (34%), progressive marrow failure (31%), fatigue (29%), splenomegaly (25%), and progressive lymphocytosis (21%). (…)<br>In the high risk CLL/SLL population (del 17p/TP53 mutation, del 11q, or unmutated IGHV), the PFS HR was 0.15 [95% CI (0.09, 0.27)].<br>E1912<br>(…) The median age was 58 years (range, 28 to 70 years), 67% were male, 90% were White and 98% had a ECOG performance status of 0-1. At baseline, 43% of patients were Rai stage 3 or 4 and 59% of patients presented with high risk factors (TP53 mutation [6%], del11q [22%], or unmutated IGHV [53%]). (…)
205552, 08/24/2022	Ibrutinib (3)	Oncology	MYD88	Clinical Studies	**14 CLINICAL STUDIES**<br>**14.3 Waldenström's Macroglobulinemia**<br>INNOVATE<br>(…) At baseline, the median serum IgM value was 3.2 g/dL (range, 0.6 to 8.3 g/dL), and MYD88 L265P mutations were present in 77% of patients, absent in 13% of patients, and 9% of patients were not evaluable for mutation status. (…)
**022192, 02/23/2017**	Iloperidone	Psychiatry	CYP2D6	Dosage and Administration, Warnings and Precautions, Drug Interactions, Clinical Pharmacology	**2 DOSAGE AND ADMINISTRATION**<br>**2.2 Dosage in Special Populations**<br>*Dosage adjustment for patients taking FANAPT who are poor metabolizers of CYP2D6*<br>FANAPT dose should be reduced by one-half for poor metabolizers of CYP2D6 [see Clinical Pharmacology (12.3)].<br><br>**5 WARNINGS AND PRECAUTIONS**<br>**5.3 QT Prolongation**<br>(…) Caution is warranted when prescribing FANAPT with drugs that inhibit FANAPT metabolism [see Drug Interactions (7.1)], and in patients with reduced activity of CYP2D6 [see Clinical Pharmacology (12.3)]. (…)<br><br>**7 DRUG INTERACTIONS**<br>**7.1 Potential for Other Drugs to Affect FANAPT**<br>*Fluoxetine:* Coadministration of fluoxetine (20 mg twice daily for 21 days), a potent inhibitor of CYP2D6, with a single 3 mg dose of iloperidone to 23 healthy volunteers, ages 29-44 years, who were classified as CYP2D6 extensive metabolizers, increased the AUC of iloperidone and its metabolite P88, by about 2- to 3-fold, and decreased the AUC of its metabolite P95 by one-half. (…)<br><br>**12 CLINICAL PHARMACOLOGY**<br>**12.3 Pharmacokinetics**<br>The observed mean elimination half-lives for iloperidone, P88 and P95 in CYP2D6 extensive metabolizers (EM) are 18, 26, and 23 hours, respectively, and in poor metabolizers (PM) are 33, 37 and 31 hours, respectively. Steady-state concentrations are attained within 3 -4 days of dosing. Iloperidone accumulation is predictable from single-dose pharmacokinetics. The pharmacokinetics of iloperidone is more than dose proportional. Elimination of iloperidone is mainly through hepatic metabolism involving 2 P450 isozymes, CYP2D6 and CYP3A4.<br>*Metabolism and Elimination*

* Therapeutic areas do not necessarily reflect the CDER review division.
† Representative biomarkers are listed based on standard nomenclature as per the Human Genome Organization (HUGO) symbol and/or simplified descriptors using other common conventions. Listed biomarkers do not necessarily reflect the terminology used in labeling. The term "Nonspecific" is provided when labeling does not explicitly identify the specific biomarker(s) or when the biomarker is represented by a molecular phenotype or gene signature, and in some cases the biomarker was inferred based on the labeling language.
‡ Referenced figures and tables may be found in the labeling available at Drugs@FDA.
Blue text represents the most recent addtions and/or changes since last posted version.

Table of Pharmacogenomic Biomarkers in Drug Labeling
Last Updated: 06/2024

NDA/ANDA/BLA Number, Label Version Date	Drug	Therapeutic Area*	Biomarker†	Labeling Sections	Labeling Text‡
					Iloperidone is metabolized primarily by 3 biotransformation pathways: carbonyl reduction, hydroxylation (mediated by CYP2D6) and O-demethylation (mediated by CYP3A4). There are 2 predominant iloperidone metabolites, P95 and P88. The iloperidone metabolite P95 represents 47.9% of the AUC of iloperidone and its metabolites in plasma at steady-state for extensive metabolizers (EM) and 25% for poor metabolizers (PM). The active metabolite P88 accounts for 19.5% and 34.0% of total plasma exposure in EM and PM, respectively.<br>Approximately 7% - 10% of Caucasians and 3% - 8% of black/African Americans lack the capacity to metabolize CYP2D6 substrates and are classified as poor metabolizers (PM), whereas the rest are intermediate, extensive or ultrarapid metabolizers. Coadministration of FANAPT with known strong inhibitors of CYP2D6 like fluoxetine results in a 2.3- fold increase in iloperidone plasma exposure, and therefore one-half of the FANAPT dose should be administered. Similarly, PMs of CYP2D6 have higher exposure to iloperidone compared with EMs and PMs should have their dose reduced by one-half. Laboratory tests are available to identify CYP2D6 PMs.<br>The bulk of the radioactive materials were recovered in the urine (mean 58.2% and 45.1% in EM and PM, respectively), with feces accounting for 19.9% (EM) to 22.1% (PM) of the dosed radioactivity.
021588, 08/21/2018	Imatinib (1)	Oncology	KIT	Indications and Usage, Dosage and Administration, Clinical Studies	**1 INDICATIONS AND USAGE**<br>**1.6 Aggressive Systemic Mastocytosis (ASM)**<br>Adult patients with aggressive systemic mastocytosis without the D816V c-Kit mutation as determined with an FDA-approved test [see Dosage and Administration (2.7)] or with c-Kit mutational status unknown.<br>**1.9 Kit+ Gastrointestinal Stromal Tumors (GIST)**<br>Patients with Kit (CD117) positive unresectable and/or metastatic malignant gastrointestinal stromal tumors.<br>**1.10 Adjuvant Treatment of GIST**<br>Adjuvant treatment of adult patients following complete gross resection of Kit (CD117) positive GIST.<br><br>**2 DOSAGE AND ADMINISTRATION**<br>**2.7 Adult Patients with ASM**<br>Determine D816V c-Kit mutation status prior to initiating treatment. Information on FDA-approved test for the detection of D816V c-Kit mutation is available at http://www.fda.gov/companiondiagnostics.<br>The recommended dose of Gleevec is 400 mg/day for adult patients with ASM without the D816V c-Kit mutation. If cKit mutational status is not known or unavailable, treatment with Gleevec 400 mg/day may be considered for patients with ASM not responding satisfactorily to other therapies. For patients with ASM associated with eosinophilia, a clonal hematological disease related to the fusion kinase FIP1L1-PDGFRα, a starting dose of 100 mg/day is recommended. Dose increase from 100 mg to 400 mg for these patients may be considered in the absence of adverse drug reactions if assessments demonstrate an insufficient response to therapy.<br><br>**14 CLINICAL STUDIES**<br>**14.5 Myelodysplastic/Myeloproliferative Diseases**<br>An open-label, multicenter, phase 2 clinical trial was conducted testing Gleevec in diverse populations of patients suffering from life-threatening diseases associated with Abl, Kit or PDGFR protein tyrosine kinases. (…)<br>**14.6 Aggressive Systemic Mastocytosis**<br>One open-label, multicenter, phase 2 study was conducted testing Gleevec in diverse populations of patients with life-threatening diseases associated with Abl, Kit or PDGFR protein tyrosine kinases. (…)<br>(…) Two patients had a Kit mutation in the juxtamembrane region (one Phe522Cys and one K509I) and four patients had a D816V c-Kit mutation (not considered sensitive to Gleevec), one with concomitant CML. (…)<br>(…) Patients that harbor the D816V mutation of c-Kit are not sensitive to Gleevec and should not receive Gleevec.<br>**14.7 Hypereosinophilic Syndrome/Chronic Eosinophilic Leukemia**<br>One open-label, multicenter, phase 2 study was conducted testing Gleevec in diverse populations of patients with life-threatening diseases associated with Abl, Kit or PDGFR protein tyrosine kinases. (…)<br>**14.8 Dermatofibrosarcoma Protuberans**<br>(…) An open-label, multicenter, phase 2 study was conducted testing Gleevec in a diverse population of patients with life-threatening diseases associated with Abl, Kit or PDGFR protein tyrosine kinases. (…)<br>**14.9 Gastrointestinal Stromal Tumors**<br>(…) One open-label, multinational Phase 2 study was conducted in patients with Kit (CD117) positive unresectable or metastatic malignant GIST. (…)<br>*Adjuvant Treatment of GIST*<br>In the adjuvant setting, Gleevec was investigated in a multicenter, double-blind, placebo-controlled, randomized trial involving 713 patients (Study 1). Patients were randomized one to one to Gleevec at 400 mg/day or matching placebo for 12 months. The ages of these patients ranged from 18 to 91 years. Patients were included who had a histologic diagnosis of primary GIST, expressing KIT protein by immunochemistry and a tumor size greater than or equal to 3 cm in maximum dimension with complete gross resection of primary GIST within 14 to 70 days prior to registration. (…)<br>(…) A second randomized, multicenter, open-label, phase 3 trial in the adjuvant setting (Study 2) compared 12 months of Gleevec treatment to 36 months of Gleevec treatment at 400 mg/day in adult patients with KIT (CD117) positive GIST after surgical resection with one of the following: tumor diameter greater than 5 cm and mitotic count greater than 5/50 high power fields (HPF), or tumor diameter greater than 10 cm and any mitotic count, or tumor of any size with mitotic count greater than 10/50 HPF, or tumors ruptured into the peritoneal cavity. (…)

* Therapeutic areas do not necessarily reflect the CDER review division.
† Representative biomarkers are listed based on standard nomenclature as per the Human Genome Organization (HUGO) symbol and/or simplified descriptors using other common conventions. Listed biomarkers do not necessarily reflect the terminology used in labeling. The term “Nonspecific” is provided when labeling does not explicitly identify the specific biomarker(s) or when the biomarker is represented by a molecular phenotype or gene signature, and in some cases the biomarker was inferred based on the labeling language.
‡ Referenced figures and tables may be found in the labeling available at Drugs@FDA.
Blue text represents the most recent addtions and/or changes since last posted version.

NDA/ANDA/BLA Number, Label Version Date	Drug	Therapeutic Area*	Biomarker†	Labeling Sections	Labeling Text‡
021588, 08/21/2018	Imatinib (2)	Oncology	BCR-ABL1 (Philadelphia chromosome)	Indications and Usage, Dosage and Administration, Warnings and Precautions, Adverse Reactions, Use in Specific Populations, Clinical Pharmacology, Clinical Studies	**1 INDICATIONS AND USAGE**<br>**1.1 Newly Diagnosed Philadelphia Positive Chronic Myeloid Leukemia (Ph+ CML)**<br>Newly diagnosed adult and pediatric patients with Philadelphia chromosome positive chronic myeloid leukemia in chronic phase.<br>**1.2 Ph+ CML in Blast Crisis (BC), Accelerated Phase (AP) or Chronic Phase (CP) After Interferon-alpha (IFN) Therapy**<br>Patients with Philadelphia chromosome positive chronic myeloid leukemia in blast crisis, accelerated phase, or in chronic phase after failure of interferon-alpha therapy.<br>**1.3 Adult patients with Ph+ Acute Lymphoblastic Leukemia (ALL)**<br>Adult patients with relapsed or refractory Philadelphia chromosome positive acute lymphoblastic leukemia.<br>**1.4 Pediatric patients with Ph+ Acute Lymphoblastic Leukemia (ALL)**<br>Pediatric patients with newly diagnosed Philadelphia chromosome positive acute lymphoblastic leukemia (Ph+ ALL) in combination with chemotherapy.<br><br>**2 DOSAGE AND ADMINISTRATION**<br>**2.2 Adult Patients with Ph+ CML CP, AP, or BC**<br>The recommended dose of Gleevec is 400 mg/day for adult patients in chronic phase CML and 600 mg/day for adult patients in accelerated phase or blast crisis. In CML, a dose increase from 400 mg to 600 mg in adult patients with chronic phase disease, or from 600 mg to 800 mg (given as 400 mg twice daily) in adult patients in accelerated phase or blast crisis may be considered in the absence of severe adverse drug reaction and severe non-leukemia related neutropenia or thrombocytopenia in the following circumstances: disease progression (at any time), failure to achieve a satisfactory hematologic response after at least 3 months of treatment, failure to achieve a cytogenetic response after 6–12 months of treatment, or loss of a previously achieved hematologic or cytogenetic response.<br>**2.3 Pediatric Patients with Ph+ CML CP**<br>The recommended dose of Gleevec for children with newly diagnosed Ph+ CML is 340 mg/m^2 /day (not to exceed 600 mg). Gleevec treatment can be given as a once daily dose or the daily dose may be split into two–one portion dosed in the morning and one portion in the evening. There is no experience with Gleevec treatment in children under 1 year of age.<br>**2.4 Adult Patients with Ph+ ALL**<br>The recommended dose of Gleevec is 600 mg/day for adult patients with relapsed/refractory Ph+ ALL.<br>**2.5 Pediatric Patients with Ph+ ALL**<br>The recommended dose of Gleevec to be given in combination with chemotherapy to children with newly diagnosed Ph+ ALL is 340 mg/m^2/day (not to exceed 600 mg). Gleevec treatment can be given as a once daily dose.<br>**2.14 Dose Adjustment for Hematologic Adverse Reactions**<br>Dose reduction or treatment interruptions for severe neutropenia and thrombocytopenia are recommended as indicated in Table 1. (See Table 1) (…)<br><br>**5 WARNINGS AND PRECAUTIONS**<br>**5.1 Fluid Retention and Edema**<br>(…) In a randomized trial in patients with newly diagnosed Ph+CML in chronic phase comparing Gleevec and nilotinib, severe (Grade 3 or 4) fluid retention occurred in 2.5% of patients receiving Gleevec and in 3.9% of patients receiving nilotinib 300 mg bid. (…)<br>**5.3 Congestive Heart Failure and Left Ventricular Dysfunction**<br>(…) In an international randomized phase 3 study in 1,106 patients with newly diagnosed Ph+ CML in chronic phase, severe cardiac failure and left ventricular dysfunction were observed in 0.7% of patients taking Gleevec compared to 0.9% of patients taking IFN + Ara-C. In another randomized trial with newly diagnosed Ph+ CML patients in chronic phase that compared Gleevec and nilotinib, cardiac failure was observed in 1.1% of patient in the Gleevec arm and 2.2% of patients in the nilotinib 300 mg bid arm and severe (Grade 3 or 4) cardiac failure occurred in 0.7% of patients in each group. (…)<br>**5.5 Hemorrhage**<br>(…) Gastrointestinal tumor sites may have been the source of GI hemorrhages. In a randomized trial in patients with newly diagnosed Ph+ CML in chronic phase comparing Gleevec and nilotinib, GI hemorrhage occurred in 1.4% of patients in the Gleevec arm, and in 2.9% of patients in the nilotinib 300 mg bid arm. None of these events were Grade 3 or 4 in the Gleevec arm; 0.7% were Grade 3 or 4 in the nilotinib 300 mg bid arm. In addition, gastric antral vascular ectasia has been reported in postmarketing experience.<br><br>**6 ADVERSE REACTIONS**<br>**6.1 Chronic Myeloid Leukemia**<br>The majority of Gleevec-treated patients experienced adverse reactions at some time. Gleevec was discontinued due to drug-related adverse reactions in 2.4% of patients receiving Gleevec in the randomized trial of newly diagnosed patients with Ph+ CML in chronic phase comparing Gleevec versus IFN+Ara-C, and in 12.5% of patients receiving Gleevec in the randomized trial of newly diagnosed patients with Ph+ CML in chronic phase comparing Gleevec and nilotinib. (Seee Table 3) (…)<br>**6.2 Adverse Reactions in Pediatric Population**<br>*In combination with multi-agent chemotherapy*<br>(…) Patients with Ph+ ALL (n=92) were assigned to receive Gleevec and treated in 5 successive cohorts. Gleevec exposure was systematically increased in successive cohorts by earlier introduction and more prolonged duration.<br>The safety of Gleevec given in combination with intensive chemotherapy was evaluated by comparing the incidence of grade 3 and 4 adverse events, neutropenia (less than 750/mcL) and thrombocytopenia (less than 75,000/mcL) in the 92 patients with Ph+ ALL compared to 65 patients with Ph- ALL enrolled on the trial who did not receive Gleevec. The safety was also evaluated comparing the incidence of adverse events in cycles of therapy administered with or without Gleevec. The protocol included up to 18 cycles of therapy. Patients were exposed to a cumulative total of 1425 cycles of therapy, 778 with Gleevec and

NDA/ANDA/BLA Number, Label Version Date	Drug	Therapeutic Area*	Biomarker†	Labeling Sections	Labeling Text‡
					647 without Gleevec. The adverse events that were reported with a 5% or greater incidence in patients with Ph+ ALL compared to Ph- ALL or with a 1% or greater incidence in cycles of therapy that included Gleevec are presented in Table 8. (See Table 8) (…)<br>**6.4 Acute Lymphoblastic Leukemia**<br>The adverse reactions were similar for Ph+ ALL as for Ph+ CML. The most frequently reported drug-related adverse reactions reported in the Ph+ ALL studies were mild nausea and vomiting, diarrhea, myalgia, muscle cramps and rash. Superficial edema was a common finding in all studies and were described primarily as periorbital or lower limb edemas. These edemas were reported as Grade 3/4 events in 6.3% of the patients and may be managed with diuretics, other supportive measures, or in some patients by reducing the dose of Gleevec.<br>**6.7 Hypereosinophilic Syndrome and Chronic Eosinophilic Leukemia**<br>The safety profile in the HES/CEL patient population does not appear to be different from the safety profile of Gleevec observed in other hematologic malignancy populations, such as Ph+ CML. All patients experienced at least one adverse reaction, the most common being gastrointestinal, cutaneous and musculoskeletal disorders. Hematological abnormalities were also frequent, with instances of CTC Grade 3 leukopenia, neutropenia, lymphopenia, and anemia.<br><br>**8 USE IN SPECIFIC POPULATIONS**<br>**8.4 Pediatric Use**<br>The safety and effectiveness of Gleevec have been demonstrated in pediatric patients with newly diagnosed Ph+ chronic phase CML and Ph+ ALL [see Clinical Studies (14.2, 14.4)]. There are no data in children under 1 year of age.<br><br>**12 CLINICAL PHARMACOLOGY**<br>**12.3 Pharmacokinetics**<br>*Pediatric Use*<br>(…) Based on pooled population pharmacokinetic analysis in pediatric patients with hematological disorders (CML, Ph+ ALL, or other hematological disorders treated with imatinib), clearance of imatinib increases with increasing body surface area (BSA). After correcting for the BSA effect, other demographics such as age, body weight and body mass index did not have clinically significant effects on the exposure of imatinib. The analysis confirmed that exposure of imatinib in pediatric patients receiving 260 mg/m2 once-daily (not exceeding 400 mg once-daily) or 340 mg/m2 once-daily (not exceeding 600 mg once-daily) were similar to those in adult patients who received imatinib 400 mg or 600 mg once-daily.<br><br>**14 CLINICAL STUDIES**<br>**14.1 Chronic Myeloid Leukemia**<br>*Chronic Phase, Newly Diagnosed*<br>An open-label, multicenter, international randomized Phase 3 study (Gleevec versus IFN+Ara-C) has been conducted in patients with newly diagnosed Philadelphia chromosome positive (Ph+) chronic myeloid leukemia (CML) in chronic phase. (See Table 18) (…)<br>(…) An open-label, multicenter, randomized trial (Gleevec versus nilotinib) was conducted to determine the efficacy of Gleevec versus nilotinib in adult patients with cytogenetically confirmed, newly diagnosed Ph+ CML-CP. Patients were within 6 months of diagnosis and were previously untreated for CML-CP, except for hydroxyurea and/or anagrelide. (See Table 19) (…)<br>(…) *Late Chronic Phase CML and Advanced Stage CML:* Three international, open-label, single-arm phase 2 studies were conducted to determine the safety and efficacy of Gleevec in patients with Ph+ CML: 1) in the chronic phase after failure of IFN therapy, 2) in accelerated phase disease, or 3) in myeloid blast crisis. About 45% of patients were women and 6% were black. In clinical studies, 38%–40% of patients were ≥60 years of age and 10%–12% of patients were ≥70 years of age.<br>(…) *Chronic Phase, Prior Interferon-Alpha Treatment:* Effectiveness was evaluated on the basis of the rate of hematologic response and by bone marrow exams to assess the rate of major cytogenetic response (up to 35% Ph+ metaphases) or complete cytogenetic response (0% Ph+ metaphases). (…)<br>**14.2 Pediatric CML**<br>One open-label, single-arm study enrolled 14 pediatric patients with Ph+ chronic phase CML recurrent after stem cell transplant or resistant to interferon-alpha therapy. (…)<br>(…) In a second study, 2 of 3 patients with Ph+ chronic phase CML resistant to interferon-alpha therapy achieved a complete cytogenetic response at doses of 242 and 257 $mg/m^2/day$.<br>**14.3 Acute Lymphoblastic Leukemia**<br>A total of 48 Philadelphia chromosome positive acute lymphoblastic leukemia (Ph+ ALL) patients with relapsed/refractory disease were studied, 43 of whom received the recommended Gleevec dose of 600 mg/day. In addition 2 patients with relapsed/refractory Ph+ ALL received Gleevec 600 mg/day in a phase 1 study.<br>Confirmed and unconfirmed hematologic and cytogenetic response rates for the 43 relapsed/refractory Ph+ALL phase 2 study patients and for the 2 phase 1 patients are shown in Table 21. The median duration of hematologic response was 3.4 months and the median duration of MCyR was 2.3 months. (See Table 21) (…)<br>**14.4 Pediatric ALL**<br>Pediatric and young adult patients with very high risk ALL, defined as those with an expected 5-year event-free survival (EFS) less than 45%, were enrolled after induction therapy on a multicenter, non-randomized cooperative group pilot protocol.<br>The safety and effectiveness of Gleevec (340 $mg/m^2/day$) in combination with intensive chemotherapy was evaluated in a subgroup of patients with Ph+ ALL. The protocol included intensive chemotherapy and hematopoietic stem cell transplant after 2 courses of chemotherapy for patients with an appropriate HLA-matched family donor. There were 92 eligible patients with Ph+ ALL enrolled. (…)<br>(…) There were 50 patients with Ph+ ALL assigned to cohort 5 all of whom received Gleevec plus chemotherapy; 30 were treated exclusively with chemotherapy and Gleevec and 20 received chemotherapy plus Gleevec and then underwent hematopoietic stem cell transplant, followed by further Gleevec treatment. (…)

* Therapeutic areas do not necessarily reflect the CDER review division.
† Representative biomarkers are listed based on standard nomenclature as per the Human Genome Organization (HUGO) symbol and/or simplified descriptors using other common conventions. Listed biomarkers do not necessarily reflect the terminology used in labeling. The term “Nonspecific” is provided when labeling does not explicitly identify the specific biomarker(s) or when the biomarker is represented by a molecular phenotype or gene signature, and in some cases the biomarker was inferred based on the labeling language.
‡ Referenced figures and tables may be found in the labeling available at Drugs@FDA.
Blue text represents the most recent addtions and/or changes since last posted version.

Table of Pharmacogenomic Biomarkers in Drug Labeling
Last Updated: 06/2024

NDA/ANDA/BLA Number, Label Version Date	Drug	Therapeutic Area*	Biomarker†	Labeling Sections	Labeling Text‡
**021588, 08/21/2018**	Imatinib (3)	Oncology	PDGFRB	Indications and Usage, Dosage and Administration, Clinical Studies	**1 INDICATIONS AND USAGE**<br>**1.5 Myelodysplastic/Myeloproliferative Diseases (MDS/MPD)**<br>Adult patients with myelodysplastic/myeloproliferative diseases associated with PDGFR (platelet-derived growth factor receptor) gene re-arrangements as determined with an FDA-approved test [see Dosage and Administration (2.6)].<br><br>**2 DOSAGE AND ADMINISTRATION**<br>**2.6 Adult Patients with MDS/MPD**<br>Determine PDGFRb gene rearrangements status prior to initiating treatment. Information on FDA-approved tests for the detection of PDGFRb rearrangements is available at http://www.fda.gov/companiondiagnostics.<br>The recommended dose of Gleevec is 400 mg/day for adult patients with MDS/MPD.<br><br>**14 CLINICAL STUDIES**<br>**14.5 Myelodysplastic/Myeloproliferative Diseases**<br>An open-label, multicenter, phase 2 clinical trial was conducted testing Gleevec in diverse populations of patients suffering from life-threatening diseases associated with Abl, Kit or PDGFR protein tyrosine kinases. (…)<br>(…) Sixteen patients had a translocation, involving chromosome 5q33 or 4q12, resulting in a PDGFR gene rearrangement. All of these patients responded hematologically (13 completely). Cytogenetic response was evaluated in 12 out of 14 patients, all of whom responded (10 patients completely). Only 1 (7%) out of the 14 patients without a translocation associated with PDGFR gene re-arrangement achieved a complete hematological response and none achieved a major cytogenetic response. A further patient with a PDGFR gene re-arrangement in molecular relapse after bone marrow transplant responded molecularly. Median duration of therapy was 12.9 months (0.8–26.7) in the 7 patients treated within the phase 2 study and ranged between 1 week and more than 18 months in responding patients in the published literature. Results are provided in Table 22. Response durations of phase 2 study patients ranged from 141+ days to 457+ days. (See Table 22)<br>**14.6 Aggressive Systemic Mastocytosis**<br>One open-label, multicenter, phase 2 study was conducted testing Gleevec in diverse populations of patients with life-threatening diseases associated with Abl, Kit or PDGFR protein tyrosine kinases. (…)<br>**14.7 Hypereosinophilic Syndrome/Chronic Eosinophilic Leukemia**<br>One open-label, multicenter, phase 2 study was conducted testing Gleevec in diverse populations of patients with life-threatening diseases associated with Abl, Kit or PDGFR protein tyrosine kinases. (…)<br>**14.8 Dermatofibrosarcoma Protuberans**<br>Dermatofibrosarcoma Protuberans (DFSP) is a cutaneous soft tissue sarcoma. It is characterized by a translocation of chromosomes 17 and 22 that results in the fusion of the collagen type 1 alpha 1 gene and the PDGF B gene. An open-label, multicenter, phase 2 study was conducted testing Gleevec in a diverse population of patients with life-threatening diseases associated with Abl, Kit or PDGFR protein tyrosine kinases. (…)<br>(…) Ten patients had the PDGF B gene rearrangement, 5 had no available cytogenetics and 3 had complex cytogenetic abnormalities. (See Table 25) (…)<br>(…) For the 10 study patients with the PDGF B gene rearrangement there were 4 complete and 6 partial responses. The median duration of response in the phase 2 study was 6.2 months, with a maximum duration of 24.3 months, while in the published literature it ranged between 4 weeks and more than 20 months.
**021588, 08/21/2018**	Imatinib (4)	Oncology	FIP1L1-PDGFRA	Indications and Usage, Dosage and Administration, Clinical Studies	**1 INDICATIONS AND USAGE**<br>**1.7 Hypereosinophilic Syndrome (HES) and/or Chronic Eosinophilic Leukemia (CEL)**<br>Adult patients with hypereosinophilic syndrome and/or chronic eosinophilic leukemia who have the FIP1L1-PDGFRα fusion kinase (mutational analysis or FISH demonstration of CHIC2 allele deletion) and for patients with HES and/or CEL who are FIP1L1-PDGFRα fusion kinase negative or unknown.<br><br>**2.7 Adult Patients with ASM**<br>Determine D816V c-Kit mutation status prior to initiating treatment. Information on FDA-approved test for the detection of D816V c-Kit mutation is available at http://www.fda.gov/companiondiagnostics.<br>The recommended dose of Gleevec is 400 mg/day for adult patients with ASM without the D816V c-Kit mutation. If cKit mutational status is not known or unavailable, treatment with Gleevec 400 mg/day may be considered for patients with ASM not responding satisfactorily to other therapies. For patients with ASM associated with eosinophilia, a clonal hematological disease related to the fusion kinase FIP1L1-PDGFRα, a starting dose of 100 mg/day is recommended. Dose increase from 100 mg to 400 mg for these patients may be considered in the absence of adverse drug reactions if assessments demonstrate an insufficient response to therapy.<br>**2.8 Adult Patients with HES/CEL**<br>The recommended dose of Gleevec is 400 mg/day for adult patients with HES/CEL. For HES/CEL patients with demonstrated FIP1L1-PDGFRα fusion kinase, a starting dose of 100 mg/day is recommended. Dose increase from 100 mg to 400 mg for these patients may be considered in the absence of adverse drug reactions if assessments demonstrate an insufficient response to therapy.<br>**2.14 Dose Adjustment for Hematologic Adverse Reactions**<br>Dose reduction or treatment interruptions for severe neutropenia and thrombocytopenia are recommended as indicated in Table 1. (See Table 1) (…)<br><br>**14 CLINICAL STUDIES**<br>**14.6 Aggressive Systemic Mastocytosis**<br>(…) Seven of these 20 patients had the FIP1L1-PDGFRα fusion kinase (or CHIC2 deletion). Patients with this cytogenetic abnormality were predominantly males and had eosinophilia associated with their systemic mast cell disease. Two patients had a Kit mutation in the juxtamembrane region (one Phe522Cys and one K509I) and four patients had a D816V c-Kit mutation (not considered sensitive to Gleevec), one with concomitant CML. (See Table 23) (…)

* Therapeutic areas do not necessarily reflect the CDER review division.
† Representative biomarkers are listed based on standard nomenclature as per the Human Genome Organization (HUGO) symbol and/or simplified descriptors using other common conventions. Listed biomarkers do not necessarily reflect the terminology used in labeling. The term “Nonspecific” is provided when labeling does not explicitly identify the specific biomarker(s) or when the biomarker is represented by a molecular phenotype or gene signature, and in some cases the biomarker was inferred based on the labeling language.
‡ Referenced figures and tables may be found in the labeling available at Drugs@FDA.
Blue text represents the most recent addtions and/or changes since last posted version.

NDA/ANDA/BLA Number, Label Version Date	Drug	Therapeutic Area*	Biomarker†	Labeling Sections	Labeling Text‡
					**14.7 Hypereosinophilic Syndrome/Chronic Eosinophilic Leukemia**<br>One open-label, multicenter, phase 2 study was conducted testing Gleevec in diverse populations of patients with life-threatening diseases associated with Abl, Kit or PDGFR protein tyrosine kinases. (See Table 24) (…)
017090, 07/28/2014	Imipramine	Psychiatry	CYP2D6	Precautions	**PRECAUTIONS**<br>*Drug Interactions*<br>*Drugs Metabolized by P450 2D6*<br>The biochemical activity of the drug metabolizing isozyme cytochrome P450 2D6 (debrisoquin hydroxylase) is reduced in a subset of the Caucasian population (about 7% to 10% of Caucasians are so-called “poor metabolizers”); reliable estimates of the prevalence of reduced P450 2D6 isozyme activity among Asian, African, and other populations are not yet available. Poor metabolizers have higher than expected plasma concentrations of tricyclic antidepressants (TCAs) when given usual doses. Depending on the fraction of drug metabolized by P450 2D6, the increase in plasma concentration may be small, or quite large (8-fold increase in plasma AUC of the TCA). (…)
214012, 12/22/2021	Inclisiran	Endocrinology	Nonspecific (Heterozygous Familial Hypercholesterolemia)	Indications and Usage, Clinical Studies	**1 INDICATIONS AND USAGE**<br>LEQVIO® is indicated as an adjunct to diet and maximally tolerated statin therapy for the treatment of adults with heterozygous familial hypercholesterolemia (HeFH) or clinical atherosclerotic cardiovascular disease (ASCVD), who require additional lowering of low-density lipoprotein cholesterol (LDL-C).<br>Limitations of Use<br>The effect of LEQVIO on cardiovascular morbidity and mortality has not been determined.<br><br>**14 CLINICAL STUDIES**<br>The efficacy of LEQVIO was investigated in three randomized, double-blind, placebo-controlled trials that enrolled 3457 adults with HeFH or clinical ASCVD, who were taking maximally tolerated statin therapy and who required additional LDL-C lowering. Demographics and baseline disease characteristics were balanced between the treatment arms in all trials. (…)<br>Heterozygous Familial Hypercholesterolemia (HeFH)<br>Study 3 (ORION-9, NCT03397121) was a multicenter, double-blind, randomized, placebo-controlled 18-month trial in which 482 patients with HeFH were randomized 1:1 to receive subcutaneous injections of either LEQVIO 284 mg (n = 242) or placebo (n = 240) on Day 1, Day 90, Day 270, and at Day 450. Patients with HeFH were taking a maximally tolerated dose of statin with or without other lipid modifying therapy, and required additional LDL-C reduction. The diagnosis of HeFH was made either by genotyping or clinical criteria using either the Simon Broome or WHO/Dutch Lipid Network criteria. Patients were stratified by country and by current use of statins or other lipid-modifying therapies. Patients taking PCSK9 inhibitors were excluded from the trial. (See Table 4 and Figure 3)
022383, 05/29/2019	Indacaterol	Pulmonary	UGT1A1	Clinical Pharmacology	**12 CLINICAL PHARMACOLOGY**<br>**12.4 Pharmacogenomics**<br>The pharmacokinetics of indacaterol were prospectively investigated in subjects with the UGT1A1 (TA)7/(TA)7 genotype (low UGT1A1 expression; also referred to as *28) and the (TA)6, (TA)6 genotype. Steady-state AUC and Cmax of indacaterol were 1.2-fold higher in the [(TA)7, (TA)7] genotype, suggesting no relevant effect of UGT1A1 genotype of indacaterol exposure.
761142, 06/11/2020	Inebilizumab-cdon	Neurology	AQP4	Indications and Usage, Clinical Studies	**1 INDICATIONS AND USAGE**<br>UPLIZNA is indicated for the treatment of neuromyelitis optica spectrum disorder (NMOSD) in adult patients who are anti-aquaporin-4 (AQP4) antibody positive.<br><br>**14 CLINICAL STUDIES**<br>The efficacy of UPLIZNA for the treatment of NMOSD was established in Study 1 (NCT02200770), a randomized (3:1), double-blind, placebo-controlled trial that enrolled 213 patients with NMOSD who were anti-AQP4 antibody positive and 17 who were anti-AQP4 antibody negative. (…)<br>Of the 213 enrolled anti-AQP4 antibody positive patients, a total of 161 were randomized to receive treatment with UPLIZNA, and 52 were randomized to receive placebo. (…)<br>The time to the first adjudicated relapse was significantly longer in patients treated with UPLIZNA compared to patients who received placebo (relative risk reduction 73%; hazard ratio: 0.272; $p < 0.0001$). In the anti-AQP4 antibody positive population there was a 77.3% relative reduction (hazard ratio: 0.227, $p < 0.0001$). There was no evidence of a benefit in patients who were anti-AQP4 antibody negative. (See Table 4 and Figure 1)<br>Compared to placebo-treated patients, patients treated with UPLIZNA who were anti-AQP4 antibody positive had reduced annualized rates of hospitalizations (0.11 for UPLIZNA versus 0.50 for placebo).
211172, 10/05/2018	Inotersen	Neurology	TTR	Adverse Reactions, Clinical Pharmacology	**6 ADVERSE REACTIONS**<br>**6.1 Clinical Trials Experience**<br>(…) Baseline disease characteristics were largely similar in TEGSEDI-treated patients and patients in the placebo control group. Sixty-seven percent of patients were in Stage 1 of the disease at baseline, and 33% in Stage 2. Fifty-two percent of patients had Val30Met mutations in the TTR gene, with the remaining 48% comprised of 26 different other point mutations. (…)<br><br>**12 CLINICAL PHARMACOLOGY**<br>**12.2 Pharmacodynamics**<br>The pharmacodynamic effects of TEGSEDI were evaluated in hATTR amyloidosis patients treated with 284 mg TEGSEDI via subcutaneous injection once weekly.<br>With repeat dosing, the mean percent decreases from baseline in serum TTR from Week 13 to Week 65 of treatment ranged from 68% to 74% (median range: 75% to 79%). Similar TTR reductions were observed regardless of TTR mutation, sex, age, or race. (…)

* Therapeutic areas do not necessarily reflect the CDER review division.
† Representative biomarkers are listed based on standard nomenclature as per the Human Genome Organization (HUGO) symbol and/or simplified descriptors using other common conventions. Listed biomarkers do not necessarily reflect the terminology used in labeling. The term “Nonspecific” is provided when labeling does not explicitly identify the specific biomarker(s) or when the biomarker is represented by a molecular phenotype or gene signature, and in some cases the biomarker was inferred based on the labeling language.
‡ Referenced figures and tables may be found in the labeling available at Drugs@FDA.
Blue text represents the most recent addtions and/or changes since last posted version.

Table of Pharmacogenomic Biomarkers in Drug Labeling
Last Updated: 06/2024

NDA/ANDA/BLA Number, Label Version Date	Drug	Therapeutic Area*	Biomarker†	Labeling Sections	Labeling Text‡
**761040, 03/06/2024**	Inotuzumab Ozogamicin	Oncology	BCR-ABL1 (Philadelphia chromosome)	Clinical Studies	**14 CLINICAL STUDIES**<br>**Patients With Relapsed or Refractory ALL – INO-VATE ALL**<br>Eligible patients were ≥ 18 years of age with Philadelphia chromosome-negative or Philadelphia chromosome-positive relapsed or refractory B-cell precursor ALL. All patients were required to have ≥ 5% bone marrow blasts and to have received 1 or 2 previous induction chemotherapy regimens for ALL. Patients with Philadelphia chromosome-positive B-cell precursor ALL were required to have disease that failed treatment with at least 1 tyrosine kinase inhibitor and standard chemotherapy. (…)<br>(…) The median age was 47 years (range: 18-79 years), 276 patients (85%) had Philadelphia chromosome-negative ALL, 206 patients (63%) had a duration of first remission < 12 months, and 55 patients (17%) had undergone a HSCT prior to receiving BESPONSA or Investigator's choice of chemotherapy. (…)
**125377, 11/13//2020**	Ipilimumab (1)	Oncology	HLA-A	Clinical Studies	**14 CLINICAL STUDIES**<br>**14.1 Unresectable or Metastatic Melanoma**<br>The safety and efficacy of YERVOY were investigated in a randomized (3:1:1), double-blind, double-dummy trial (MDX010-20, NCT00094653) that included 676 randomized patients with unresectable or metastatic melanoma previously treated with one or more of the following: aldesleukin, dacarbazine, temozolomide, fotemustine, or carboplatin. Of these 676 patients, 403 were randomized to receive YERVOY at 3 mg/kg in combination with an investigational peptide vaccine with incomplete Freund's adjuvant (gp100), 137 were randomized to receive YERVOY at 3 mg/kg, and 136 were randomized to receive gp100 as a single agent. The trial enrolled only patients with HLA-A2*0201 genotype; this HLA genotype facilitates the immune presentation of the investigational peptide vaccine. (…)
**125377, 11/13//2020**	Ipilimumab (2)	Oncology	Microsatellite Instability, Mismatch Repair	Indications and Usage, Dosage and Administration, Warnings and Precautions, Adverse Reactions, Use in Specific Populations, Clinical Studies	**1 INDICATIONS AND USAGE**<br>**1.4 Microsatellite Instability-High (MSI-H) or Mismatch Repair Deficient (dMMR) Metastatic Colorectal Cancer**<br>YERVOY, in combination with nivolumab, is indicated for the treatment of adult and pediatric patients 12 years of age and older with microsatellite instability-high (MSI-H) or mismatch repair deficient (dMMR) metastatic colorectal cancer (CRC) that has progressed following treatment with a fluoropyrimidine, oxaliplatin, and irinotecan [see Clinical Studies (14.4)]. This indication is approved under accelerated approval based on overall response rate and duration of response. Continued approval for this indication may be contingent upon verification and description of clinical benefit in confirmatory trials.<br><br>**2 DOSAGE AND ADMINISTRATION**<br>**2.2 Recommended Dosage**<br>The recommended dosages of YERVOY as a single agent are presented in Table 1. (See Table 2)<br><br>**5 WARNINGS AND PRECAUTIONS**<br>**5.5 Risks Associated When Administered in Combination with Nivolumab**<br>YERVOY is indicated for use in combination with nivolumab for patients with advanced RCC, MSI-H or dMMR mCRC, HCC, and NSCLC. Refer to the nivolumab Full Prescribing Information for additional risk information that applies to the combination use treatment.<br><br>**6 ADVERSE REACTIONS**<br>**6.1 Clinical Trials Experience**<br>The data described below reflect exposure to YERVOY 3 mg/kg as a single agent in MDX010-20, a randomized trial in patients with unresectable or metastatic melanoma; to YERVOY 10 mg/kg as a single agent in CA184-029, a randomized trial in patients with resected Stage IIIA (>1 mm nodal involvement), IIIB, and IIIC (with no in-transit metastases) cutaneous melanoma; and to YERVOY 1 mg/kg, administered in combination with nivolumab, in two trials: CHECKMATE214 (NCT02231749), a randomized trial in previously untreated patients with advanced renal cell carcinoma, and CHECKMATE-142 (NCT02060188), an open-label, multicenter, non-randomized multiple parallel cohort trial in patients with previously treated, MSI-H or dMMR metastatic colorectal cancer. (…)<br>**Previously Treated MSI-H or dMMR Metastatic Colorectal Cancer**<br>The safety of YERVOY was evaluated in CHECKMATE-142, an open-label, multicenter, nonrandomized, multiple parallel-cohort study. In CHECKMATE-142, 119 patients with previously treated MSI-H or dMMR mCRC received YERVOY, in combination with nivolumab, in a singlearm cohort. In another single-arm cohort under CHECKMATE-142, 74 patients with mCRC received nivolumab monotherapy. (See Tables 9 and 10) (…)<br><br>**8 USE IN SPECIFIC POPULATIONS**<br>**8.4 Pediatric Use**<br>The safety and effectiveness of YERVOY have been established in pediatric patients 12 years and older for the treatment of unresectable or metastatic melanoma or for the treatment of microsatellite instability-high (MSI-H) or mismatch repair deficient (dMMR) metastatic colorectal cancer that has progressed following treatment with a fluoropyrimidine, oxaliplatin, and irinotecan. Use of YERVOY in this age group is supported by evidence from adequate and well-controlled studies of YERVOY in adults and population pharmacokinetic data demonstrating that the exposure at doses of 3 mg/kg and 1 mg/kg in the pediatric and adult populations are comparable. In addition, the tumor biology and course of advanced melanoma and MSI-H or dMMR metastatic colorectal cancer are sufficiently similar in adults and pediatric patients 12 years and older to allow extrapolation of data from adults to pediatric patients. (…)<br><br>**14 CLINICAL STUDIES**<br>**14.4 Microsatellite Instability-High (MSI-H) or Mismatch Repair Deficient (dMMR) Metastatic Colorectal Cancer**<br>CHECKMATE-142 (NCT02060188) was a multicenter, non-randomized, multiple parallelcohort, open-label study conducted in patients with locally determined dMMR or MSI-H metastatic CRC (mCRC) who had disease progression during or after prior treatment with fluoropyrimidine-, oxaliplatin-, or irinotecan-based chemotherapy. Key eligibility criteria were at least one prior line of treatment for metastatic disease, ECOG PS 0 or 1, and absence of the following: active brain metastases, active autoimmune disease, or medical conditions requiring systemic immunosuppression.

* Therapeutic areas do not necessarily reflect the CDER review division.
† Representative biomarkers are listed based on standard nomenclature as per the Human Genome Organization (HUGO) symbol and/or simplified descriptors using other common conventions. Listed biomarkers do not necessarily reflect the terminology used in labeling. The term "Nonspecific" is provided when labeling does not explicitly identify the specific biomarker(s) or when the biomarker is represented by a molecular phenotype or gene signature, and in some cases the biomarker was inferred based on the labeling language.
‡ Referenced figures and tables may be found in the labeling available at Drugs@FDA.
Blue text represents the most recent addtions and/or changes since last posted version.

Table of Pharmacogenomic Biomarkers in Drug Labeling

Last Updated: 06/2024

NDA/ANDA/BLA Number, Label Version Date	Drug	Therapeutic Area*	Biomarker†	Labeling Sections	Labeling Text‡
					Patients enrolled in the YERVOY and nivolumab MSI-H mCRC cohort received YERVOY 1 mg/kg and nivolumab 3 mg/kg IV every 3 weeks for 4 doses, followed by nivolumab 3 mg/kg IV as a single agent every 2 weeks. Patients enrolled in the single-agent nivolumab MSI-H mCRC cohort received nivolumab 3 mg/kg by intravenous (IV) infusion every 2 weeks. Treatment in both cohorts continued until unacceptable toxicity or radiographic progression. (See Table 22) (…)
**125377, 11/13//2020**	Ipilimumab (3)	Oncology	CD274 (PD-L1)	Indications and Usage, Dosage and Administration, Use in Specific Populations, Clinical Studies	**1 INDICATIONS AND USAGE**<br>**1.6 Metastatic Non-Small Cell Lung Cancer**<br>YERVOY, in combination with nivolumab, is indicated for the first-line treatment of adult patients with metastatic non-small cell lung cancer (NSCLC) whose tumors express PD-L1 (≥1%) as determined by an FDA-approved test [see Dosage and Administration (2.1)], with no EGFR or ALK genomic tumor aberrations. YERVOY, in combination with nivolumab and 2 cycles of platinum-doublet chemotherapy, is indicated for the first-line treatment of adult patients with metastatic or recurrent NSCLC, with no EGFR or ALK genomic tumor aberrations.<br><br>**2 DOSAGE AND ADMINISTRATION**<br>**2.1 Patient Selection**<br>Select patients with metastatic NSCLC for treatment with YERVOY in combination with nivolumab based on PD-L1 expression [see Clinical Studies (14.6)]. Information on FDA-approved tests for the determination of PD-L1 expression in NSCLC is available at: http://www.fda.gov/CompanionDiagnostics.<br>**2.2 Recommended Dosage**<br>The recommended dosages of YERVOY as a single agent are presented in Table 1. (See Table 2)<br><br>**8 USE IN SPECIFIC POPULATIONS**<br>**8.5 Geriatric Use**<br>(…) Of the 576 patients randomized to YERVOY 1 mg/kg every 6 weeks with nivolumab 3 mg/kg every 2 weeks in CHECKMATE-227 (NSCLC), 48% were 65 years or older and 10% were 75 years or older. No overall difference in safety was reported between older patients and younger patients; however, there was a higher discontinuation rate due to adverse reactions in patients aged 75 years or older (29%) relative to all patients who received YERVOY with nivolumab (18%). Of the 396 patients in the primary efficacy population (PD-L1 ≥1%) randomized to YERVOY 1 mg/kg every 6 weeks with nivolumab 3 mg/kg every 2 weeks with in CHECKMATE-227, the hazard ratio for overall survival was 0.70 (95% CI: 0.55, 0.89) in the 199 patients younger than 65 years compared to 0.91 (95% CI: 0.72, 1.15) in the 197 patients 65 years or older [see Clinical Studies (14.6)]. (…)<br><br>**14 CLINICAL STUDIES**<br>**14.3 Previously Untreated Advanced Renal Cell Carcinoma**<br>CHECKMATE-214 (NCT02231749) was a randomized (1:1), open-label study in patients with previously untreated advanced RCC. Patients were included regardless of their PD-L1 status. CHECKMATE-214 excluded patients with any history of or concurrent brain metastases, active autoimmune disease, or medical conditions requiring systemic immunosuppression. Patients were stratified by International Metastatic RCC Database Consortium (IMDC) prognostic score and region. (…)<br>(…) Efficacy results from CHECKMATE-214 are presented in Table 21 and Figure 3. In intermediate/poor risk patients, the trial demonstrated statistically significant improvement in OS and ORR for patients randomized to YERVOY and nivolumab arm as compared with sunitinib arm. OS benefit was observed regardless of PD-L1 expression level. The trial did not demonstrate a statistically significant improvement in PFS. (…)<br>**14.6 Metastatic Non-Small Cell Lung Cancer**<br>First-line Treatment of Metastatic Non-Small Cell Lung Cancer (NSCLC) Expressing PD-L1 (≥1%): In Combination with Nivolumab<br>CHECKMATE-227 (NCT02477826) was a randomized, open-label, multi-part trial in patients with metastatic or recurrent NSCLC. The study included patients (18 years of age or older) with histologically confirmed Stage IV or recurrent NSCLC (per the 7th International Association for the Study of Lung Cancer [ASLC] classification), ECOG performance status 0 or 1, and no prior anticancer therapy. Patients were enrolled regardless of their tumor PD-L1 status. (…)<br>Primary efficacy results were based on Part 1a of the study, which was limited to patients with PD-L1 tumor expression t1%. Tumor specimens were evaluated prospectively using the PD-L1 IHC 28-8 pharmDx assay at a central laboratory. Randomization was stratified by tumor histology (non-squamous versus squamous). (…)<br>In Part 1a, a total of 793 patients were randomized to receive either YERVOY in combination with nivolumab (n=396) or platinum-doublet chemotherapy (n=397). The median age was 64 years (range: 26 to 87) with 49% of patients t65 years and 10% of patients t75 years, 76% White, and 65% male. Baseline ECOG performance status was 0 (34%) or 1 (65%), 50% with PD-L1 ≥50%, 29% with squamous and 71% with non-squamous histology, 10% had brain metastases, and 85% were former/current smokers.<br>The study demonstrated a statistically significant improvement in OS for PD-L1 ≥1% patients randomized to the YERVOY and nivolumab arm compared to platinum-doublet chemotherapy arm. The OS results are presented in Table 22 and Figure 4. (See Table 24 and Figure 4)<br>First-line Treatment of Metastatic or Recurrent NSCLC: In Combination with Nivolumab and Platinum-Doublet Chemotherapy<br>CHECKMATE-9LA (NCT03215706) was a randomized, open-label trial in patients with metastatic or recurrent NSCLC. The trial included patients (18 years of age or older) with histologically confirmed Stage IV or recurrent NSCLC (per the 7th International Association for the Study of Lung Cancer classification [IASLC]), ECOG performance status 0 or 1, and no prior anticancer therapy (including EGFR and ALK inhibitors) for metastatic disease. Patients were enrolled regardless of their tumor PD-L1 status. (…)<br>Platinum-doublet chemotherapy consisted of either carboplatin (AUC 5 or 6) and pemetrexed 500 mg/mg2, or cisplatin 75 mg/m2 and pemetrexed 500 mg/m2 for non-squamous NSCLC; or carboplatin (AUC 6) and paclitaxel 200 mg/m2 for squamous NSCLC. Patients with non-squamous NSCLC in the control arm could receive optional pemetrexed maintenance therapy. Stratification factors for randomization were tumor PD-L1 expression level (≥1% versus <1% or non-quantifiable), histology (squamous versus non-squamous), and sex (male versus female). Study treatment continued until disease progression, unacceptable toxicity, or for up to 2 years. (…)

* Therapeutic areas do not necessarily reflect the CDER review division.
† Representative biomarkers are listed based on standard nomenclature as per the Human Genome Organization (HUGO) symbol and/or simplified descriptors using other common conventions. Listed biomarkers do not necessarily reflect the terminology used in labeling. The term “Nonspecific” is provided when labeling does not explicitly identify the specific biomarker(s) or when the biomarker is represented by a molecular phenotype or gene signature, and in some cases the biomarker was inferred based on the labeling language.
‡ Referenced figures and tables may be found in the labeling available at Drugs@FDA.
Blue text represents the most recent addtions and/or changes since last posted version.

NDA/ANDA/BLA Number, Label Version Date	Drug	Therapeutic Area*	Biomarker†	Labeling Sections	Labeling Text‡
					A total of 719 patients were randomized to receive either YERVOY in combination with nivolumab and platinum-doublet chemotherapy (n=361) or platinum-doublet chemotherapy (n=358). The median age was 65 years (range: 26 to 86) with 51% of patients t65 years and 10% of patients t75 years. The majority of patients were White (89%) and male (70%). Baseline ECOG performance status was 0 (31%) or 1 (68%), 57% had tumors with PD-L1 expression t1% and 37% had tumors with PD-L1 expression that was <1%, 32% had tumors with squamous histology and 68% had tumors with non-squamous histology, 17% had CNS metastases, and 86% were former or current smokers. (…)<br>**14.7 Malignant Pleural Mesothelioma**<br>(…) A total of 605 patients were randomized to receive either YERVOY in combination with nivolumab (n=303) or chemotherapy (n=302). The median age was 69 years (range: 25 to 89), with 72% of patients ≥65 years and 26% ≥75 years; 85% were White, 11% were Asian, and 77% were male. Baseline ECOG performance status was 0 (40%) or 1 (60%), 35% had Stage III and 51% had Stage IV disease, 75% had epithelioid and 25% had non-epithelioid histology, 75% had tumors with PD-L1 expression ≥1%, and 22% had tumors with PD-L1 expression <1%.

Table of Pharmacogenomic Biomarkers in Drug Labeling
Last Updated: 06/2024

NDA/ANDA/BLA Number, Label Version Date	Drug	Therapeutic Area*	Biomarker†	Labeling Sections	Labeling Text‡
**125377, 11/13//2020**	Ipilimumab (4)	Oncology	ALK	Indications and Usage, Adverse Reactions, Clinical Studies	**1 INDICATIONS AND USAGE**<br>**1.6 Metastatic Non-Small Cell Lung Cancer**<br>YERVOY, in combination with nivolumab, is indicated for the first-line treatment of adult patients with metastatic non-small cell lung cancer (NSCLC) whose tumors express PD-L1 (≥1%) as determined by an FDA-approved test [see Dosage and Administration (2.1)], with no EGFR or ALK genomic tumor aberrations.<br>YERVOY, in combination with nivolumab and 2 cycles of platinum-doublet chemotherapy, is indicated for the first-line treatment of adult patients with metastatic or recurrent NSCLC, with no EGFR or ALK genomic tumor aberrations.<br><br>**6 ADVERSE REACTIONS**<br>The data described below reflect exposure to YERVOY 3 mg/kg as a single agent in MDX010-20, a randomized trial in patients with unresectable or metastatic melanoma; to YERVOY 10 mg/kg as a single agent in CA184-029, a randomized trial in patients with resected Stage IIIA (>1 mm nodal involvement), IIIB, and IIIC (with no in-transit metastases) cutaneous melanoma; to YERVOY 1 mg/kg, administered in combination with nivolumab, in three trials: CHECKMATE214, a randomized trial in previously untreated patients with advanced renal cell carcinoma, CHECKMATE-142, an open-label, multicenter, non-randomized multiple parallel cohort trial in patients with previously treated, MSI-H or dMMR metastatic colorectal cancer, and CHECKMATE-227, a randomized, multicenter, multi-cohort, open-label trial in patients with previously untreated metastatic or recurrent non-small cell lung cancer with no EGFR or ALK genomic tumor aberrations; and to YERVOY 3 mg/kg, administered in combination with nivolumab, in CHECKMATE-040, a multicenter, multiple cohort, open-label trial conducted in patients with hepatocellular carcinoma who progressed on or were intolerant to sorafenib; and to YERVOY 1 mg/kg, administered in combination with nivolumab and platinum-doublet chemotherapy in CHECKMATE-9LA, an open-label, multicenter, randomized trial in adult patients with previously untreated metastatic or recurrent non-small cell lung cancer with no EGFR or ALK genomic tumor aberrations. (…)<br>First-line Treatment of Metastatic NSCLC: In Combination with Nivolumab<br>The safety of YERVOY in combination with nivolumab was evaluated in CHECKMATE-227, a randomized, multicenter, multi-cohort, open-label trial in patients with previously untreated metastatic or recurrent NSCLC with no EGFR or ALK genomic tumor aberrations [see Clinical Studies (14.6)]. (…)<br><br>**14 CLINICAL STUDIES**<br>**14.6 Metastatic Non-Small Cell Lung Cancer**<br>First-line Treatment of Metastatic Non-Small Cell Lung Cancer (NSCLC) Expressing PD-L1 (≥1%): In Combination with Nivolumab<br>CHECKMATE-227 (NCT02477826) was a randomized, open-label, multi-part trial in patients with metastatic or recurrent NSCLC. The study included patients (18 years of age or older) with histologically confirmed Stage IV or recurrent NSCLC (per the 7th International Association for the Study of Lung Cancer [ASLC] classification), ECOG performance status 0 or 1, and no prior anticancer therapy. Patients were enrolled regardless of their tumor PD-L1 status. Patients with known EGFR mutations or ALK translocations sensitive to available targeted inhibitor therapy, untreated brain metastases, carcinomatous meningitis, active autoimmune disease, or medical conditions requiring systemic immunosuppression were excluded from the study. (…)<br>First-line Treatment of Metastatic or Recurrent NSCLC: In Combination with Nivolumab and Platinum-Doublet Chemotherapy<br>CHECKMATE-9LA (NCT03215706) was a randomized, open-label trial in patients with metastatic or recurrent NSCLC. The trial included patients (18 years of age or older) with histologically confirmed Stage IV or recurrent NSCLC (per the 7th International Association for the Study of Lung Cancer classification [IASLC]), ECOG performance status 0 or 1, and no prior anticancer therapy (including EGFR and ALK inhibitors) for metastatic disease. Patients were enrolled regardless of their tumor PD-L1 status. Patients with known EGFR mutations or ALK translocations sensitive to available targeted inhibitor therapy, untreated brain metastases, carcinomatous meningitis, active autoimmune disease, or medical conditions requiring systemic immunosuppression were excluded from the study. (…)

* Therapeutic areas do not necessarily reflect the CDER review division.
† Representative biomarkers are listed based on standard nomenclature as per the Human Genome Organization (HUGO) symbol and/or simplified descriptors using other common conventions. Listed biomarkers do not necessarily reflect the terminology used in labeling. The term “Nonspecific” is provided when labeling does not explicitly identify the specific biomarker(s) or when the biomarker is represented by a molecular phenotype or gene signature, and in some cases the biomarker was inferred based on the labeling language.
‡ Referenced figures and tables may be found in the labeling available at Drugs@FDA.
Blue text represents the most recent addtions and/or changes since last posted version.

Table of Pharmacogenomic Biomarkers in Drug Labeling
Last Updated: 06/2024

NDA/ANDA/BLA Number, Label Version Date	Drug	Therapeutic Area*	Biomarker†	Labeling Sections	Labeling Text‡
125377, 11/13//2020	Ipilimumab (5)	Oncology	EGFR	Indications and Usage, Adverse Reactions, Clinical Studies	**1 INDICATIONS AND USAGE**<br>**1.6 Metastatic Non-Small Cell Lung Cancer**<br>YERVOY, in combination with nivolumab, is indicated for the first-line treatment of adult patients with metastatic non-small cell lung cancer (NSCLC) whose tumors express PD-L1 (≥1%) as determined by an FDA-approved test [see Dosage and Administration (2.1)], with no EGFR or ALK genomic tumor aberrations.<br>YERVOY, in combination with nivolumab and 2 cycles of platinum-doublet chemotherapy, is indicated for the first-line treatment of adult patients with metastatic or recurrent NSCLC, with no EGFR or ALK genomic tumor aberrations.<br><br>**6 ADVERSE REACTIONS**<br>The data described below reflect exposure to YERVOY 3 mg/kg as a single agent in MDX010-20, a randomized trial in patients with unresectable or metastatic melanoma; to YERVOY 10 mg/kg as a single agent in CA184-029, a randomized trial in patients with resected Stage IIIA (>1 mm nodal involvement), IIIB, and IIIC (with no in-transit metastases) cutaneous melanoma; to YERVOY 1 mg/kg, administered in combination with nivolumab, in three trials: CHECKMATE214, a randomized trial in previously untreated patients with advanced renal cell carcinoma, CHECKMATE-142, an open-label, multicenter, non-randomized multiple parallel cohort trial in patients with previously treated, MSI-H or dMMR metastatic colorectal cancer, and CHECKMATE-227, a randomized, multicenter, multi-cohort, open-label trial in patients with previously untreated metastatic or recurrent non-small cell lung cancer with no EGFR or ALK genomic tumor aberrations; and to YERVOY 3 mg/kg, administered in combination with nivolumab, in CHECKMATE-040, a multicenter, multiple cohort, open-label trial conducted in patients with hepatocellular carcinoma who progressed on or were intolerant to sorafenib; and to YERVOY 1 mg/kg, administered in combination with nivolumab and platinum-doublet chemotherapy in CHECKMATE-9LA, an open-label, multicenter, randomized trial in adult patients with previously untreated metastatic or recurrent non-small cell lung cancer with no EGFR or ALK genomic tumor aberrations. (…)<br>First-line Treatment of Metastatic NSCLC: In Combination with Nivolumab<br>The safety of YERVOY in combination with nivolumab was evaluated in CHECKMATE-227, a randomized, multicenter, multi-cohort, open-label trial in patients with previously untreated metastatic or recurrent NSCLC with no EGFR or ALK genomic tumor aberrations [see Clinical Studies (14.6)]. (…)<br><br>**14 CLINICAL STUDIES**<br>**14.6 Metastatic Non-Small Cell Lung Cancer**<br>First-line Treatment of Metastatic Non-Small Cell Lung Cancer (NSCLC) Expressing PD-L1 (≥1%): In Combination with Nivolumab<br>CHECKMATE-227 (NCT02477826) was a randomized, open-label, multi-part trial in patients with metastatic or recurrent NSCLC. The study included patients (18 years of age or older) with histologically confirmed Stage IV or recurrent NSCLC (per the 7th International Association for the Study of Lung Cancer [ASLC] classification), ECOG performance status 0 or 1, and no prior anticancer therapy. Patients were enrolled regardless of their tumor PD-L1 status. Patients with known EGFR mutations or ALK translocations sensitive to available targeted inhibitor therapy, untreated brain metastases, carcinomatous meningitis, active autoimmune disease, or medical conditions requiring systemic immunosuppression were excluded from the study. (…)<br>First-line Treatment of Metastatic or Recurrent NSCLC: In Combination with Nivolumab and Platinum-Doublet Chemotherapy<br>CHECKMATE-9LA (NCT03215706) was a randomized, open-label trial in patients with metastatic or recurrent NSCLC. The trial included patients (18 years of age or older) with histologically confirmed Stage IV or recurrent NSCLC (per the 7th International Association for the Study of Lung Cancer classification [IASLC]), ECOG performance status 0 or 1, and no prior anticancer therapy (including EGFR and ALK inhibitors) for metastatic disease. Patients were enrolled regardless of their tumor PD-L1 status. Patients with known EGFR mutations or ALK translocations sensitive to available targeted inhibitor therapy, untreated brain metastases, carcinomatous meningitis, active autoimmune disease, or medical conditions requiring systemic immunosuppression were excluded from the study. (…)
020571, 02/13/2024	Irinotecan	Oncology	UGT1A1	Dosage and Administration, Clinical Pharmacology, Clinical Studies	**2 DOSAGE AND ADMINISTRATION**<br>**2.2 Recommended Dosage**<br>In combination with oxaliplatin, fluorouracil and leucovorin for the first-line treatment of patients with metastatic pancreatic adenocarcinoma<br>• Administer ONIVYDE prior to oxaliplatin, fluorouracil and leucovorin [see Clinical Studies (14)].<br>• The recommended dosage of ONIVYDE regardless of UGT1A1*28 allele genotype is 50 mg/m2 administered by intravenous infusion over 90 minutes every 2 weeks.<br>There is no recommended dosage of ONIVYDE for patients with serum bilirubin above the upper limit of normal [see Adverse Reactions (6.1) and Clinical Studies (14)].<br>In combination with fluorouracil and leucovorin for the treatment of patients with metastatic pancreatic adenocarcinoma after disease progression following gemcitabine-based therapy Administer ONIVYDE prior to fluorouracil and leucovorin [see Clinical Studies (14)].<br>• The recommended dosage of ONIVYDE is 70 mg/m2 administered by intravenous infusion over 90 minutes every 2 weeks.<br>• The recommended starting dose of ONIVYDE in patients known to be homozygous for the UGT1A1*28 allele is 50 mg/m2 administered by intravenous infusion over 90 minutes. Increase the dose of ONIVYDE to 70 mg/m2 as tolerated in subsequent cycles.<br>• There is no recommended dosage of ONIVYDE for patients with serum bilirubin above the upper limit of normal [see Adverse Reactions (6.1) and Clinical Studies (14)]. (See Table 2)<br><br>**12 CLINICAL PHARMACOLOGY**<br>**12.3 Pharmacokinetics**<br>Elimination<br>*Metabolism*<br>The metabolism of irinotecan liposome has not been evaluated. Irinotecan is subject to extensive metabolic conversion by various enzyme systems, including carboxylesterases to form the active metabolite SN-38, and UGT1A1 mediating glucuronidation of SN-38 to form the inactive glucuronide metabolite SN-38G. Irinotecan can also undergo CYP3A4-mediated oxidative metabolism to several inactive oxidation products, one of which can be hydrolyzed by carboxylesterase

* Therapeutic areas do not necessarily reflect the CDER review division.
† Representative biomarkers are listed based on standard nomenclature as per the Human Genome Organization (HUGO) symbol and/or simplified descriptors using other common conventions. Listed biomarkers do not necessarily reflect the terminology used in labeling. The term “Nonspecific” is provided when labeling does not explicitly identify the specific biomarker(s) or when the biomarker is represented by a molecular phenotype or gene signature, and in some cases the biomarker was inferred based on the labeling language.
‡ Referenced figures and tables may be found in the labeling available at Drugs@FDA.
Blue text represents the most recent addtions and/or changes since last posted version.

NDA/ANDA/BLA Number, Label Version Date	Drug	Therapeutic Area*	Biomarker†	Labeling Sections	Labeling Text‡
					to release SN-38. In the population PK analysis of irinotecan liposome, UGT1A1*28 7/7 homozygous status (10.6%) had no effect on SN-38 clearance compared with patients not homozygous for UGT1A1*28 7/7.<br>**12.5 Pharmacogenomics**<br>Individuals who are homozygous for the UGT1A1*28 allele are at increased risk for neutropenia from irinotecan HCl. In NAPOLI-1, patients homozygous for the UGT1A1*28 allele (N=7) initiated ONIVYDE at a reduced dose of 50 mg/m2 in combination with FU/LV. The frequency of Grade 3 or 4 neutropenia in these patients [2 of 7 (28.6%)] was similar to the frequency in patients not homozygous for the UGT1A1*28 allele who received a starting dose of ONIVYDE of 70 mg/m2 [30 of 110 (27.3%)]. In NAPOLI-3, patients homozygous for the UGT1A1*28 allele (N = 39) initiated ONIVYDE at the same starting dose of 50 mg/m2 as patients not homozygous for the UGT1A1*28 allele (N = 328). The frequency of Grade 3 or 4 neutropenia was 23% in patients homozygous for the UGT1A1*28 allele and 13% in patients not homozygous for the UGT1A1*28 allele. The frequency of dose reduction of ONIVYDE due to treatment-emergent adverse effects was 59% versus 51% in patients homozygous versus nonhomozygous for the UGT1A1*28 allele.<br><br>**14 CLINICAL STUDIES**<br><u>Pancreatic Adenocarcinoma</u><br>Patients homozygous for the UGT1A1*28 allele initiated ONIVYDE at the same dose (50 mg/m2 ONIVYDE). Treatment continued until RECIST v1.1 defined disease progression or unacceptable toxicity. Tumor status assessments were conducted at baseline and every 8 weeks thereafter as assessed by the investigator according to RECIST v1.1.<br><u>Previously treated metastatic pancreatic adenocarcinoma in combination with fluorouracil and leucovorin</u><br>(…) Patients homozygous for the UGT1A1*28 allele initiated ONIVYDE at a reduced dose (50 mg/m2 ONIVYDE, if given with FU/LV or 70 mg/m2 ONIVYDE as a single agent). When ONIVYDE was withheld or discontinued for adverse reactions, FU was also withheld or discontinued. When the dose of ONIVYDE was reduced for adverse reactions, the dose of FU was reduced by 25%. Treatment continued until disease progression or unacceptable toxicity.
**761113, 03/02/2020**	**Isatuximab- irfc (1)**	Oncology	Chromosome 17p	Clinical Studies	**14 CLINICAL STUDIES**<br>**14.1 Multiple Myeloma**<br><u>ICARIA-MM</u><br>(…) Overall, 20% of patients had high-risk chromosomal abnormalities at study entry; del(17p), t(4;14) and t(14;16) were present in 12%, 8% and 2% of patients, respectively. (…)
**761113, 03/02/2020**	**Isatuximab- irfc (2)**	Oncology	Chromosome 4p;14q	Clinical Studies	**14 CLINICAL STUDIES**<br>**14.1 Multiple Myeloma**<br><u>ICARIA-MM</u><br>(…) Overall, 20% of patients had high-risk chromosomal abnormalities at study entry; del(17p), t(4;14) and t(14;16) were present in 12%, 8% and 2% of patients, respectively. (…)
**761113, 03/02/2020**	**Isatuximab- irfc (3)**	Oncology	Chromosome 14q;16q	Clinical Studies	**14 CLINICAL STUDIES**<br>**14.1 Multiple Myeloma**<br><u>ICARIA-MM</u><br>(…) Overall, 20% of patients had high-risk chromosomal abnormalities at study entry; del(17p), t(4;14) and t(14;16) were present in 12%, 8% and 2% of patients, respectively. (…)
017624, 11/01/2022	**Isoflurane**	Anesthesiology	CACNA1S, RYR1 (Genetic Susceptibility to Malignant Hyperthermia)	Contraindications, Warnings, Clinical Pharmacology	**CONTRAINDICATIONS**<br>Known sensitivity to FORANE (isoflurane, USP) or to other halogenated agents. Known or suspected genetic susceptibility to malignant hyperthermia.<br><br>**WARNINGS**<br>**Malignant Hyperthermia:** In susceptible individuals, volatile anesthetic agents, including isoflurane, may trigger malignant hyperthermia, a skeletal muscle hypermetabolic state leading to high oxygen demand. Fatal outcomes of malignant hyperthermia have been reported. The risk of developing malignant hyperthermia increases with the concomitant administration of succinylcholine and volatile anesthetic agents. FORANE can induce malignant hyperthermia in patients with known or suspected susceptibility based on genetic factors or family history, including those with certain inherited ryanodine receptor (RYR1) or dihydropyridine receptor (CACNA1S) variants. [see CONTRAINDICATIONS, CLINICAL PHARMACOLOGY/Pharmacogenomics]

* Therapeutic areas do not necessarily reflect the CDER review division.
† Representative biomarkers are listed based on standard nomenclature as per the Human Genome Organization (HUGO) symbol and/or simplified descriptors using other common conventions. Listed biomarkers do not necessarily reflect the terminology used in labeling. The term “Nonspecific” is provided when labeling does not explicitly identify the specific biomarker(s) or when the biomarker is represented by a molecular phenotype or gene signature, and in some cases the biomarker was inferred based on the labeling language.
‡ Referenced figures and tables may be found in the labeling available at Drugs@FDA.
Blue text represents the most recent addtions and/or changes since last posted version.

NDA/ANDA/BLA Number, Label Version Date	Drug	Therapeutic Area*	Biomarker†	Labeling Sections	Labeling Text‡
					**CLINICAL PHARMACOLOGY**<br>**Pharmacogenomics:** RYR1 and CACNA1S are polymorphic genes, and multiple pathogenic variants have been associated with malignant hyperthermia susceptibility (MHS) in patients receiving volatile anesthetic agents, including FORANE. Case reports as well as ex-vivo studies have identified multiple variants in RYR1 and CACNA1S associated with MHS. Variant pathogenicity should be assessed based on prior clinical experience, functional studies, prevalence information, or other evidence (see CONTRAINDICATIONS, WARNINGS).
**050705, 02/28/2019**	**Isoniazid, Pyrazinamide, and Rifampin**	Infectious Diseases	Nonspecific (NAT)	Clinical Pharmacology	**CLINICAL PHARMACOLOGY**<br>(…) Isoniazid is metabolized in the liver mainly by acetylation and dehydrazination. The rate of acetylation is genetically determined. Approximately 50% of African Americans and Caucasians are "slow inactivators" and the rest are "rapid inactivators"; the majority of Eskimos and Asians are "rapid inactivators." The rate of acetylation does not significantly alter the effectiveness of isoniazid. However, slow acetylation may lead to higher blood levels of the drug, and thus, an increase in toxic reactions.
**019790, 10/24/2014**	**Isosorbide Dinitrate**	Cardiology	CYB5R	Overdosage	**OVERDOSAGE**<br>*Methemoglobinemia*<br>Nitrate ions liberated during metabolism of isosorbide dinitrate can oxidize hemoglobin into methemoglobin. Even in patients totally without cytochrome b5 reductase activity, however, and even assuming that the nitrate moieties of isosorbide dinitrate are quantitatively applied to oxidation of hemoglobin, about 1 mg/kg of isosorbide dinitrate should be required before any of these patients manifests clinically significant (≥ 10%) methemoglobinemia. In patients with normal reductase function, significant production of methemoglobin should require even larger doses of isosorbide dinitrate. In one study in which 36 patients received 2-4 weeks of continuous nitroglycerin therapy at 3.1 to 4.4 mg/hr (equivalent, in total administered dose of nitrate ions, to 4.8-6.9 mg of bioavailable isosorbide dinitrate per hour), the average methemoglobin level measured was 0.2%; this was comparable to that observed in parallel patients who received placebo.<br>Notwithstanding these observations, there are case reports of significant methemoglobinemia in association with moderate overdoses of organic nitrates. None of the affected patients had been thought to be unusually susceptible. Methemoglobin levels are available from most clinical laboratories. The diagnosis should be suspected in patients who exhibit signs of impaired oxygen delivery despite adequate cardiac output and adequate arterial pO2. Classically, methemoglobinemic blood is described as chocolate brown, without color change on exposure to air. When methemoglobinemia is diagnosed, the treatment of choice is methylene blue, 1-2 mg/kg intravenously.
**020215, 10/02/2014**	**Isosorbide Mononitrate**	Cardiology	CYB5R	Overdosage	**OVERDOSAGE**<br>*Methemoglobinemia*<br>Methemoglobinemia has been reported in patients receiving other organic nitrates, and it probably could also occur as a side effect of isosorbide mononitrate. Certainly nitrate ions liberated during metabolism of isosorbide mononitrate can oxidize hemoglobin into methemoglobin. Even in patients totally without cytochrome b5 reductase activity, however, and even assuming that the nitrate moiety of isosorbide mononitrate is quantitatively applied to oxidation of hemoglobin, about 2 mg/kg of isosorbide mononitrate should be required before any of these patients manifests clinically significant (≥10%) methemoglobinemia. In patients with normal reductase function, significant production of methemoglobin should require even larger doses of isosorbide mononitrate. In one study in which 36 patients received 2-4 weeks of continuous nitroglycerin therapy at 3.1 to 4.4 mg/hr (equivalent, in total administered dose of nitrate ions, to 7.8-11.1 mg of isosorbide mononitrate per hour), the average methemoglobin level measured was 0.2%; this was comparable to that observed in parallel patients who received placebo.<br>Notwithstanding these observations, there are case reports of significant methemoglobinemia in association with moderate overdoses of organic nitrates. None of the affected patients had been thought to be unusually susceptible.<br>Methemoglobin levels are available from most clinical laboratories. The diagnosis should be suspected in patients who exhibit signs of impaired oxygen delivery despite adequate cardiac output and adequate arterial p02. Classically, methemoglobinemic blood is described as chocolate brown, without color change on exposure to air.<br>When methemoglobinemia is diagnosed, the treatment of choice is methylene blue, 1-2 mg/kg intravenously.
**203188, 04/29/2019**	**Ivacaftor**	Pulmonary	CFTR	Indications and Usage, Adverse Reactions, Use in Specific Populations, Clinical Pharmacology, Clinical Studies	**1 INDICATIONS AND USAGE**<br>KALYDECO is a cystic fibrosis transmembrane conductance regulator (CFTR) potentiator indicated for the treatment of cystic fibrosis (CF) in patients age 6 months and older who have one mutation in the CFTR gene that is responsive to ivacaftor potentiation based on clinical and/or in vitro assay data [see Clinical Pharmacology (12.1) and Clinical Studies (14)].<br>If the patient's genotype is unknown, an FDA-cleared CF mutation test should be used to detect the presence of a CFTR mutation followed by verification with bi-directional sequencing when recommended by the mutation test instructions for use.<br><br>**6 ADVERSE REACTIONS**<br>**6.1 Clinical Trials Experience**<br>Because clinical trials are conducted under widely varying conditions, adverse reaction rates observed in the clinical trials of a drug cannot be directly compared to rates in the clinical trials of another drug and may not reflect the rates observed in clinical practice.<br>The overall safety profile of KALYDECO is based on pooled data from three placebo-controlled clinical trials conducted in 353 patients 6 years of age and older with CF who had a G551D mutation in the CFTR gene (Trials 1 and 2) or were homozygous for the F508del mutation (Trial 3). In addition, the following clinical trials have also been conducted [see Clinical Pharmacology (12) and Clinical Studies (14)]:<br>• An 8-week, crossover design trial (Trial 4) involving 39 patients between the ages of 6 and 57 years with a G1244E, G1349D, G178R, G551S, G970R, S1251N, S1255P, S549N, or S549R mutation in the CFTR gene.<br>• A 24-week, placebo-controlled trial (Trial 5) involving 69 patients between the ages of 6 and 68 years with an R117H mutation in the CFTR gene.

* Therapeutic areas do not necessarily reflect the CDER review division.
† Representative biomarkers are listed based on standard nomenclature as per the Human Genome Organization (HUGO) symbol and/or simplified descriptors using other common conventions. Listed biomarkers do not necessarily reflect the terminology used in labeling. The term "Nonspecific" is provided when labeling does not explicitly identify the specific biomarker(s) or when the biomarker is represented by a molecular phenotype or gene signature, and in some cases the biomarker was inferred based on the labeling language.
‡ Referenced figures and tables may be found in the labeling available at Drugs@FDA.
Blue text represents the most recent addtions and/or changes since last posted version.

NDA/ANDA/BLA Number, Label Version Date	Drug	Therapeutic Area*	Biomarker†	Labeling Sections	Labeling Text‡
					• A 24-week, open-label trial (Trial 6) in 34 patients 2 to less than 6 years of age. Patients eligible for Trial 6 were those with the G551D, G1244E, G1349D, G178R, G551S, G970R, S1251N, S1255P, S549N, or S549R mutation in the CFTR gene. Of 34 patients enrolled, 32 had the G551D mutation and 2 had the S549N mutation.<br>• An 8-week, crossover design trial (Trial 7) involving patients between the ages of 12 and 72 years who were heterozygous for the F508del mutation and a second CFTR mutation predicted to be responsive to ivacaftor. A total of 156 patients were randomized to and received KALYDECO.<br>• A cohort of 19 patients aged 12 months to less than 24 months, and a cohort of 11 patients aged 6 months to less than 12 months in a 24-week, open-label clinical trial in patients with CF aged less than 24 months (Trial 8).<br>Of the 353 patients included in the pooled analyses of patients with CF who had either a G551D mutation or were homozygous for the F508del mutation in the CFTR gene, 50% of patients were female and 97% were Caucasian; 221 received KALYDECO, and 132 received placebo from 16 to 48 weeks. (…)<br>The incidence of adverse reactions below is based upon two double-blind, placebo-controlled, 48-week clinical trials (Trials 1 and 2) in a total of 213 patients with CF ages 6 to 53 who have a G551D mutation in the CFTR gene and who were treated with KALYDECO 150 mg orally or placebo twice daily. Table 2 shows adverse reactions occurring in ≥8% of KALYDECO-treated patients with CF who have a G551D mutation in the CFTR gene that also occurred at a higher rate than in the placebo-treated patients in the two double-blind, placebo-controlled trials. (See Table 2) (…)<br><br>**8 USE IN SPECIFIC POPULATIONS**<br>**8.4 Pediatric Use**<br>KALYDECO is indicated for the treatment of CF in pediatric patients 6 months to 17 years of age who have one mutation in the CFTR gene that is responsive to ivacaftor potentiation based on clinical and/or in vitro assay data [see Clinical Pharmacology (12.1) and Clinical Studies (14)].<br>Placebo-controlled clinical trials established efficacy and safety in the following pediatric patients with CF:<br>• 6 to 17 years of age with a G551D, G1244E, G1349D, G178R, G551S, S1251N, S1255P, S549N, S549R, or R117H mutation in the CFTR gene [see Adverse Reactions (6) and Clinical Studies (14)].<br>• 12 to 17 years of age who are heterozygous for the F508del mutation and a second mutation predicted to be responsive to ivacaftor [see Adverse Reactions (6) and Clinical Studies (14)]. (…)<br><br>**12 CLINICAL PHARMACOLOGY**<br>**12.1 Mechanism of Action**<br>*CFTR Chloride Transport Assay in Fisher Rat Thyroid (FRT) cells expressing mutant CFTR*<br>In order to evaluate the response of mutant CFTR protein to ivacaftor, total chloride transport was determined in Ussing chamber electrophysiology studies using a panel of FRT cell lines transfected with individual CFTR mutations. Ivacaftor increased chloride transport in FRT cells expressing CFTR mutations that result in CFTR protein being delivered to the cell surface.<br>Data shown in Figure 1 are the mean (n=3-7) net change over baseline in CFTR mediated chloride transport following the addition of ivacaftor in FRT cells expressing mutant CFTR proteins. The in vitro CFTR chloride response threshold was designated as a net increase of at least 10% of normal over baseline (dotted line) because it is predictive or reasonably expected to predict clinical benefit. Mutations with an increase in chloride transport of 10% or greater are considered responsive. A patient must have at least one CFTR mutation responsive to ivacaftor to be indicated.<br>Mutations including F508del that are not responsive to ivacaftor potentiation, based on the in vitro CFTR chloride response threshold, are listed in Figure 1 below the dotted line. (see Figure 1)<br>Note that splice mutations cannot be studied in this FRT assay and are not included in Figure 1. Evidence of clinical efficacy exists for non-canonical splice mutations 2789+5G→A, 3272-26A→G, 3849+10kbC→T, 711+3A→G and E831X and these are listed in Table 3 below [see also Clinical Studies (14.4)]. The G970R mutation causes a splicing defect resulting in little-to-no CFTR protein at the cell surface that can be potentiated by ivacaftor [see Clinical Studies (14.2)].<br>Ivacaftor also increased chloride transport in cultured human bronchial epithelial (HBE) cells derived from CF patients who carried F508del on one CFTR allele and either G551D or R117H-5T on the second CFTR allele.<br>Table 3 lists mutations that are responsive to ivacaftor based on 1) a positive clinical response and/or 2) in vitro data in FRT cells indicating that ivacaftor increases chloride transport to at least 10% over baseline (% of normal). (see Table 3)<br>**12.2 Pharmacodynamics**<br>Sweat Chloride Evaluation<br>Changes in sweat chloride (a biomarker) response to KALYDECO were evaluated in seven clinical trials [see Clinical Studies (14)]. In a two-part, randomized, double-blind, placebo-controlled, crossover clinical trial in patients with CF who had a G1244E, G1349D, G178R, G551S, G970R, S1251N, S1255P, S549N, or S549R mutation in the CFTR gene (Trial 4), the treatment difference in mean change in sweat chloride from baseline through 8 weeks of treatment was -49 mmol/L (95% CI -57, -41). The mean changes in sweat chloride for the mutations for which KALYDECO is indicated ranged from -51 to -8, whereas the range for individual subjects with the G970R mutation was -1 to -11 mmol/L. In an open-label clinical trial in 34 patients ages 2 to less than 6 years administered either 50 mg or 75 mg of ivacaftor twice daily (Trial 6), the mean absolute change from baseline in sweat chloride through 24 weeks of treatment was -45 mmol/L (95% CI -53, -38) [see Use in Specific Populations (8.4)]. In a randomized, double-blind, placebo-controlled, 2-period, 3-treatment, 8-week crossover study in patients with CF age 12 years and older who were heterozygous for the F508del mutation and with a second CFTR mutation predicted to be responsive to ivacaftor (Trial 7), the treatment difference in mean change in sweat chloride from study baseline to the average of Week 4 and Week 8 of treatment for KALYDECO treated patients was -4.5 mmol/L (95% CI -6.7, -2.3). In a 24-week, open-label clinical trial in patients with CF aged less than 24 months administered either 25 mg, 50 mg or 75 mg of ivacaftor twice daily (Trial 8), the mean absolute change from baseline in sweat chloride for patients aged 12 months to less than 24 months (n=10) was -73.5 mmol/L (95% CI -86.0, -61.0) at Week 24, and the mean absolute change from baseline in sweat chloride for patients aged 6 months to less than 12 months (n=6) was -58.6 mmol/L (95% CI -75.9, -41.3) at Week 24. [see Use in Specific Populations (8.4)]. (…)<br><br>**14 CLINICAL STUDIES**

* Therapeutic areas do not necessarily reflect the CDER review division.
† Representative biomarkers are listed based on standard nomenclature as per the Human Genome Organization (HUGO) symbol and/or simplified descriptors using other common conventions. Listed biomarkers do not necessarily reflect the terminology used in labeling. The term “Nonspecific” is provided when labeling does not explicitly identify the specific biomarker(s) or when the biomarker is represented by a molecular phenotype or gene signature, and in some cases the biomarker was inferred based on the labeling language.
‡ Referenced figures and tables may be found in the labeling available at Drugs@FDA.
Blue text represents the most recent addtions and/or changes since last posted version.

NDA/ANDA/BLA Number, Label Version Date	Drug	Therapeutic Area*	Biomarker†	Labeling Sections	Labeling Text‡
					**14.1 Trials in Patients with CF who have a G551D Mutation in the CFTR Gene**<br>*Efficacy*<br>The efficacy of KALYDECO in patients with CF who have a G551D mutation in the CFTR gene was evaluated in two randomized, double-blind, placebo-controlled clinical trials in 213 clinically stable patients with CF (109 receiving KALYDECO 150 mg twice daily). All eligible patients from these trials were rolled over into an open-label extension study. (…)<br>**14.2 Trial in Patients with a G1244E, G1349D, G178R, G551S, G970R, S1251N, S1255P, S549N, or S549R Mutation in the CFTR Gene**<br>The efficacy and safety of KALYDECO in patients with CF who have a G1244E, G1349D, G178R, G551S, G970R, S1251N, S1255P, S549N, or S549R mutation in the CFTR gene were evaluated in a two-part, randomized, double-blind, placebo-controlled, crossover design clinical trial in 39 patients with CF (Trial 4). Patients who completed Part 1 of this trial continued into the 16-week open-label Part 2 of the study. The mutations studied were G178R, S549N, S549R, G551S, G970R, G1244E, S1251N, S1255P, and G1349D. See Clinical Studies (14.1) for efficacy in patients with a G551D mutation. (See Table 6) (…)<br>**14.3 Trial in Patients with CF who have an R117H Mutation in the CFTR Gene**<br>The efficacy and safety of KALYDECO in patients with CF who have an R117H mutation in the CFTR gene were evaluated in a randomized, double-blind, placebo-controlled, parallel-group clinical trial (Trial 5). (See Table 7) (…)<br>**14.5 Trial in Patients Homozygous for the F508del Mutation in the CFTR Gene**<br>Trial 3 was a 16-week, randomized, double-blind, placebo-controlled, parallel-group trial in 140 patients with CF age 12 years and older who were homozygous for the F508del mutation in the CFTR gene and who had FEV1 ≥40% predicted. (…)<br>The primary endpoint was improvement in lung function as determined by the mean absolute change from baseline through Week 16 in percent predicted FEV1. The treatment difference from placebo for the mean absolute change in percent predicted FEV1 through Week 16 in patients with CF homozygous for the F508del mutation in the CFTR gene was 1.72 percentage points (1.5% and -0.2% for patients in the KALYDECO and placebo-treated groups, respectively) and did not reach statistical significance (Table 9). (See Table 9) (…)
**206038, 08/15/2018**	Ivacaftor and Lumacaftor	Pulmonary	CFTR	Indications and Usage, Adverse Reactions, Use in Specific Populations, Clinical Studies	**1 INDICATIONS AND USAGE**<br>ORKAMBI is a combination of lumacaftor and ivacaftor indicated for the treatment of cystic fibrosis (CF) in patients age 2 years and older who are homozygous for the F508del mutation in the CFTR gene. If the patient's genotype is unknown, an FDA-cleared CF mutation test should be used to detect the presence of the F508del mutation on both alleles of the CFTR gene. *Limitations of Use*<br>The efficacy and safety of ORKAMBI have not been established in patients with CF other than those homozygous for the F508del mutation.<br><br>**6 ADVERSE REACTIONS**<br>**6.1 Clinical Trials Experience**<br>Because clinical trials are conducted under widely varying conditions, adverse reaction rates observed in the clinical trials of a drug cannot be directly compared to rates in the clinical trials of another drug and may not reflect the rates observed in practice.<br>The overall safety profile of ORKAMBI is based on the pooled data from 1108 patients with CF 12 years and older who are homozygous for the F508del mutation in the CFTR gene and who received at least one dose of study drug in 2 double-blind, placebo-controlled, Phase 3 clinical trials, each with 24 weeks of treatment (Trials 1 and 2).<br>In addition, the following clinical trials have been conducted:<br>• A 24-week open-label trial (Trial 3) in 58 patients with CF aged 6 through 11 years homozygous for the F508del-CFTR mutation.<br>• A 24-week, placebo-controlled trial (Trial 4) in 204 patients aged 6 through 11 years homozygous for the F508del-CFTR mutation.<br>• A 24-week, open label trial (Trial 5) in 46 patients aged 12 years and older homozygous for the F508del-CFTR mutation and with advanced lung disease (ppFEV1 <40).<br>• A 24-week, open-label trial (Trial 6) in 60 patients aged 2 through 5 years homozygous for the F508del-CFTR mutation. (…)<br>Table 3 shows adverse reactions occurring in ≥5% of patients with CF ages 12 years and older treated with ORKAMBI who are homozygous for the F508del mutation in the CFTR gene that also occurred at a higher rate than in patients who received placebo in the two double-blind, placebo-controlled trials. (See Table 3) (…)<br>The safety profile from two pediatric trials in CF patients aged 6 through 11 years who are homozygous for the F508del-CFTR mutation, a 24-week, open-label, multicenter Phase 3 safety trial in 58 patients (Trial 3) and a 24-week, placebo-controlled, Phase 3 clinical trial (Trial 4) in 204 patients (103 received lumacaftor 200 mg/ivacaftor 250 mg every 12 hours and 101 received placebo), was similar to that observed in Trials 1 and 2. Adverse reactions that are not listed in Table 3, and that occurred in ≥5% of lumacaftor/ivacaftor-treated patients with an incidence of ≥3% higher than placebo included: productive cough (17.5% vs 5.9%), nasal congestion (16.5% vs 7.9%), headache (12.6% vs 8.9%), abdominal pain upper (12.6% vs 6.9%), and sputum increased (10.7% vs 2.0%).<br>In a 24-week, open-label, multicenter Phase 3 study in 60 patients aged 2 through 5 years with CF who are homozygous for the F508del-CFTR mutation (Trial 6) the safety profile was similar to that observed in studies in patients aged 6 years and older.<br><br>**8 USE IN SPECIFIC POPULATIONS**<br>**8.4 Pediatric Use**<br>The efficacy of ORKAMBI in children ages 2 through 11 years is extrapolated from efficacy in patients ages 12 years and older homozygous for the F508del mutation in the CFTR gene with support from population pharmacokinetic analyses showing similar drug exposure levels in patients ages 12 years and older and in children ages 2 through 11 years [see Clinical Pharmacology (12.3)]. (…)<br><br>**14 CLINICAL STUDIES**<br>*Confirmatory*<br>The efficacy of ORKAMBI in patients with CF who are homozygous for the F508del mutation in the CFTR gene was evaluated in two randomized, double- blind, placebo-controlled, 24-week clinical trials (Trials 1 and 2) in 1108 clinically stable patients with CF of whom 369 patients received ORKAMBI twice daily. (…)

* Therapeutic areas do not necessarily reflect the CDER review division.
† Representative biomarkers are listed based on standard nomenclature as per the Human Genome Organization (HUGO) symbol and/or simplified descriptors using other common conventions. Listed biomarkers do not necessarily reflect the terminology used in labeling. The term "Nonspecific" is provided when labeling does not explicitly identify the specific biomarker(s) or when the biomarker is represented by a molecular phenotype or gene signature, and in some cases the biomarker was inferred based on the labeling language.
‡ Referenced figures and tables may be found in the labeling available at Drugs@FDA.
Blue text represents the most recent addtions and/or changes since last posted version.

NDA/ANDA/BLA Number, Label Version Date	Drug	Therapeutic Area*	Biomarker†	Labeling Sections	Labeling Text‡
210491, 02/12/2018	Ivacaftor and Tezacaftor	Pulmonary	CFTR	Indications and Usage, Adverse Reactions, Use in Specific Populations, Clinical Pharmacology, Clinical Studies	**1 INDICATIONS AND USAGE**<br>SYMDEKO is indicated for the treatment of patients with cystic fibrosis (CF) aged 12 years and older who are homozygous for the F508del mutation or who have at least one mutation in the cystic fibrosis transmembrane conductance regulator (CFTR) gene that is responsive to tezacaftor/ivacaftor based on in vitro data and/or clinical evidence [see Clinical Pharmacology (12.1) and Clinical Studies (14)].<br>If the patient's genotype is unknown, an FDA-cleared CF mutation test should be used to detect the presence of a CFTR mutation followed by verification with bi-directional sequencing when recommended by the mutation test instructions for use.<br><br>**6 ADVERSE REACTIONS**<br>(…) The safety profile for the CF patients enrolled in Trial 2 who were heterozygous for the F508del mutation and a second mutation predicted to be responsive to tezacaftor/ivacaftor was similar to that observed in Trials 1 and 3.<br><br>**8 USE IN SPECIFIC POPULATIONS**<br>**8.4 Pediatric Use**<br>SYMDEKO is indicated for the treatment of CF in pediatric patients ages 12-17 years who are homozygous for the F508del mutation or who have at least one mutation in the CFTR gene that is responsive to tezacaftor/ivacaftor based on in vitro data and/or clinical evidence [see Clinical Pharmacology (12.1) and Clinical Studies (14)]. Clinical trials included the following CF patients: • 12 to 17 years of age who are homozygous for the F508del mutation [see Adverse Reactions (6) and Clinical Studies (14)]. • 12 to 17 years of age who are heterozygous for the F508del mutation and a second mutation predicted to be responsive to tezacaftor/ivacaftor [see Adverse Reactions (6) and Clinical Studies (14)]. The safety and efficacy of SYMDEKO in patients with CF younger than 12 years of age have not been studied.<br><br>**12 CLINICAL PHARMACOLOGY**<br>**12.2 Pharmacodynamics**<br>Effects on Sweat Chloride<br>In Trial 1 (patients homozygous for the F508del mutation), the treatment difference between SYMDEKO and placebo in mean absolute change from baseline in sweat chloride through Week 24 was -10.1 mmol/L (95% CI: -11.4, -8.8). In Trial 2 (patients heterozygous for the F508del mutation and a second mutation predicted to be responsive to tezacaftor/ivacaftor), the treatment difference in mean absolute change from baseline in sweat chloride through Week 8 was -9.5 mmol/L (95% CI: -11.7, -7.3) between SYMDEKO and placebo, and -4.5 mmol/L (95% CI: -6.7, -2.3) between ivacaftor and placebo. (…)<br><br>**14 CLINICAL STUDIES**<br>Dose Ranging:<br>Dose selection for the clinical program primarily consisted of one double-blind, placebo-controlled, multiple-cohort trial which included 176 patients with CF (homozygous for the F508del mutation) 18 years of age and older with a screening ppFEV1≥40. In the study, 34 and 106 patients, respectively, received tezacaftor at once-daily doses of 10 mg, 30 mg, 100 mg, or 150 mg alone or in combination with ivacaftor 150 mg q12h, and 33 patients received placebo. During the 28-day treatment period, dose-dependent increases in mean ppFEV1 change from baseline were observed with tezacaftor in combination with ivacaftor. Tezacaftor/ivacaftor in general had a greater mean treatment effect than tezacaftor alone. No additional benefit was observed at tezacaftor doses greater than 100 mg daily.<br>Efficacy:<br>The efficacy of SYMDEKO in patients with CF aged 12 years and older was evaluated in three Phase 3, double-blind, placebo-controlled trials (Trials1, 2, and 3).<br>Trial 1 was a 24-week randomized, double-blind, placebo-controlled, two-arm study in CF patients who were homozygous for the F508del mutation in the CFTR gene.<br>Trial 2 was a randomized, double-blind, placebo-controlled, 2-period, 3-treatment, 8-week crossover study in CF patients who were heterozygous for the F508del mutation and a second mutation predicted to be responsive to tezacaftor/ivacaftor. Mutations predicted to be responsive were selected for the study based on the clinical phenotype (pancreatic sufficiency), biomarker data (sweat chloride), and in vitro responsiveness to tezacaftor/ivacaftor [see Clinical Studies (14.2)]. Patients were randomized to and received sequences of treatment that included SYMDEKO, ivacaftor, and placebo.<br>Trial 3 was a 12-week randomized, double-blind, placebo-controlled, two-arm study in CF patients who were heterozygous for the F508del mutation and a second CFTR mutation predicted to be unresponsive to tezacaftor/ivacaftor. Mutations predicted to be non-responsive were selected for the study based on biologic plausibility (mutation class), clinical phenotype (pancreatic insufficiency), biomarker data (sweat chloride), and in vitro testing to tezacaftor and/or ivacaftor. (…)<br>**14.1 Trial in Patients with CF Who Were Homozygous for the F508del Mutation in the CFTR Gene (Trial 1)**<br>Trial 1 evaluated 504 patients (248 SYMDEKO, 256 placebo) with CF aged 12 years and older (mean age 26.3 years). The mean ppFEV1 at baseline was 60.0% [range: 27.8% to 96.2%]. (see Table 8 and Figure 2) (…)<br>**14.2 Trial in Patients with CF Who Were Heterozygous for the F508del Mutation and a Second Mutation Predicted to be Responsive to Tezacaftor/Ivacaftor (Trial 2)**<br>Trial 2 evaluated 244 patients with CF aged 12 years and older (mean age 34.8 years). The mean ppFEV1 at baseline was 62.3% [range: 34.6 to 93.5]. Of the 244 patients included in the efficacy analysis, 146 patients had a splice mutation and 98 patients had a missense mutation as the second allele.<br>Statistically significant improvements compared to placebo were also observed in the subgroup of patients with splice mutations and missense mutations (see Table 9).<br>In an analysis of BMI at Week 8, an exploratory endpoint, patients treated with SYMDEKO had a mean improvement of 0.2 kg/m2 [95% CI (0.0, 0.3)], 0.1 kg/m2 [95% CI (-0.1, 0.3)], and 0.3 kg/m2 [95% CI (0.1, 0.5)] versus placebo for the overall, splice, and missense mutation populations of patients, respectively.

* Therapeutic areas do not necessarily reflect the CDER review division.
† Representative biomarkers are listed based on standard nomenclature as per the Human Genome Organization (HUGO) symbol and/or simplified descriptors using other common conventions. Listed biomarkers do not necessarily reflect the terminology used in labeling. The term "Nonspecific" is provided when labeling does not explicitly identify the specific biomarker(s) or when the biomarker is represented by a molecular phenotype or gene signature, and in some cases the biomarker was inferred based on the labeling language.
‡ Referenced figures and tables may be found in the labeling available at Drugs@FDA.
Blue text represents the most recent addtions and/or changes since last posted version.

NDA/ANDA/BLA Number, Label Version Date	Drug	Therapeutic Area*	Biomarker†	Labeling Sections	Labeling Text‡
					**14.3 Trial in Patients with CF Who Were Heterozygous for the F508del Mutation and a Second Mutation Not Predicted to be Responsive to Tezacaftor/Ivacaftor (Trial 3)**<br>Trial 3 evaluated 168 patients with CF (83 SYMDEKO and 85 placebo) aged 12 years and older (mean age 26.1 years) who were heterozygous for the F508del mutation and had a second CFTR mutation predicted to be unresponsive to tezacaftor/ivacaftor. CF patients with the F508del mutation and one of the following mutations in the CFTR gene were enrolled in the study (listed in decreasing frequency): W1282X, G542X, N1303K, 621+1G>T, 1717-1G>A, 1898+1G>A, CFTRdele2,3, 2183delAA>G, 2184insA, R1162X, R553X, 3659delC, 3905insT, G970R, I507del, R1066C, R347P, 1154insTC, 1811+1.6kbA>G, 2184delA, 405+1G>A, E60X, G85E, L1077P, Q39X, S466X, Y1092X, 1078delT, 1248+1G>A, 1677delTA, 1812-1G>A, 2869INSG, 3120+1G>A, 394delTT, 457TAT>G, 711+1G>T, 711+5G>A, 712-1G>T, G673x, L1065P, Q220X, Q493X, R709X, V520F. The mean ppFEV1 at baseline was 57.5% [range: 31.0 to 96.7]. The primary efficacy endpoint was change from baseline in absolute ppFEV1 through Week 12. The overall treatment difference between SYMDEKO and placebo for the mean absolute change in ppFEV1 from baseline through Week 12 was 1.2 percentage points (95% CI: -0.3, 2.6). This study was terminated following the planned interim analysis because the pre-specified futility criteria were met.
**211192, 10/24/2023**	**niidenib**	Oncology	IDH1	Indications and Usage, Dosage and Administration, Clinical Pharmacology, Clinical Studies	**1 INDICATIONS AND USAGE**<br>**1.1 Newly-Diagnosed Acute Myeloid Leukemia**<br>TIBSOVO is indicated in combination with azacitidine or as monotherapy for the treatment of newly diagnosed acute myeloid leukemia (AML) with a susceptible isocitrate dehydrogenase-1 (IDH1) mutation as detected by an FDA-approved test in adults 75 years or older, or who have comorbidities that preclude use of intensive induction chemotherapy [see Dosage and Administration (2.1), Clinical Pharmacology (12.1) and Clinical Studies (14.1)].<br>**1.2 Relapsed or Refractory Acute Myeloid Leukemia**<br>TIBSOVO is indicated for the treatment of adult patients with relapsed or refractory acute myeloid leukemia (AML) with a susceptible isocitrate dehydrogenase-1 (IDH1) mutation as detected by an FDA-approved test [see Dosage and Administration (2.1), Clinical Pharmacology (12.1) and Clinical Studies (14.2)].<br>**1.3 Relapsed or Refractory Myelodysplastic Syndromes**<br>TIBSOVO is indicated for the treatment of adult patients with relapsed or refractory myelodysplastic syndromes (MDS) with a susceptible isocitrate dehydrogenase-1 (IDH1) mutation as detected by an FDA-approved test [see Dosage and Administration (2.1), Clinical Pharmacology (12.1) and Clinical Studies (14.3)].<br>**1.4 Locally Advanced or Metastatic Cholangiocarcinoma**<br>TIBSOVO is indicated for the treatment of adult patients with previously treated, locally advanced or metastatic cholangiocarcinoma with an isocitrate dehydrogenase-1 (IDH1) mutation as detected by an FDA-approved test [see Dosage and Administration (2.1), Clinical Pharmacology (12.1), and Clinical Studies (14.3)].<br><br>**2 DOSAGE AND ADMINISTRATION**<br>**2.1 Patient Selection**<br>Select patients for treatment with TIBSOVO based on the presence of IDH1 mutations [see Clinical Studies (14.1, 14.2, 14.3, 14.4)]. Information on FDA-approved tests for the detection of IDH1 mutations in AML, MDS, and cholangiocarcinoma is available at http://www.fda.gov/CompanionDiagnostics.<br><br>**12 CLINICAL PHARMACOLOGY**<br>**12.2 Pharmacodynamics**<br>Multiple doses of ivosidenib 500 mg daily were observed to decrease plasma 2-HG concentrations in patients with hematological malignancies to levels similar to those observed at baseline in healthy subjects. In bone marrow, 2-HG concentrations were reduced by >90%.<br><u>Cardiac Electrophysiology</u><br>A concentration-dependent QTc interval prolongation of approximately 16.1 msec (90% CI: 13.3, 18.9) was observed at the steady-state Cmax following a 500 mg daily dose based on an analysis of 171 patients with an IDH1 mutation, including 136 patients with relapsed or refractory AML, who received TIBSOVO 500 mg daily [see Warnings and Precautions (5.1)]. Co-administration with moderate or strong CYP3A inhibitors is expected to further increase QTc interval prolongation from baseline.<br><br>**14 CLINICAL STUDIES**<br>**14.1 Newly-Diagnosed AML**<br>**Newly Diagnosed AML in Combination with Azacitidine**<br>The efficacy of TIBSOVO was evaluated in a randomized (1:1), multicenter, double-blind, placebo-controlled clinical trial (Study AG120-C-009, NCT03173248) of 146 adult patients with newly-diagnosed AML with an IDH1 mutation who were 75 years or older, or had comorbidities that precluded the use of intensive induction chemotherapy based on at least one of the following criteria: baseline Eastern Cooperative Oncology Group (ECOG) performance status of 2, severe cardiac or pulmonary disease, hepatic impairment with bilirubin > 1.5 times the upper limit of normal, creatinine clearance < 45 mL/min, or other comorbidity. IDH1 mutations were confirmed centrally using the Abbott RealTime™ IDH1 Assay. Local diagnostic tests were permitted for screening and randomization provided a bone marrow or peripheral blood sample was sent for central confirmation. Gene mutation analysis to document IDH1 mutated disease from a bone marrow or peripheral blood sample was conducted for all patients. (See Table 11) (…)<br>**Monotherapy in Newly Diagnosed AML**<br>The efficacy of TIBSOVO was evaluated in an open-label, single-arm, multicenter clinical trial (Study AG120-C-001, NCT02074839) that included 28 adult patients with newly diagnosed AML with an IDH1 mutation. IDH1 mutations were identified by a local or central diagnostic test and confirmed retrospectively using the Abbott RealTimeTM IDH1 Assay. (See Table 13) (…)<br>**14.2 Relapsed or Refractory AML**

* Therapeutic areas do not necessarily reflect the CDER review division.
† Representative biomarkers are listed based on standard nomenclature as per the Human Genome Organization (HUGO) symbol and/or simplified descriptors using other common conventions. Listed biomarkers do not necessarily reflect the terminology used in labeling. The term “Nonspecific” is provided when labeling does not explicitly identify the specific biomarker(s) or when the biomarker is represented by a molecular phenotype or gene signature, and in some cases the biomarker was inferred based on the labeling language.
‡ Referenced figures and tables may be found in the labeling available at Drugs@FDA.
Blue text represents the most recent addtions and/or changes since last posted version.

NDA/ANDA/BLA Number, Label Version Date	Drug	Therapeutic Area*	Biomarker†	Labeling Sections	Labeling Text‡
					The efficacy of TIBSOVO was evaluated in an open-label, single-arm, multicenter clinical trial (Study AG120-C-001, NCT02074839) of 174 adult patients with relapsed or refractory AML with an IDH1 mutation. IDH1 mutations were identified by a local or central diagnostic test and confirmed retrospectively using the Abbott RealTimeTM IDH1 Assay. (See Table 15) (…)<br>**14.3 Relapsed or Refractory MDS**<br>The efficacy of TIBSOVO was evaluated in an open-label, single-arm, multicenter study (study AG120-C-001, NCT02074839) of 18 adult patients with relapsed or refractory MDS with an IDH1 mutation. IDH1 mutations were detected in peripheral blood or bone marrow by a local or central diagnostic test and confirmed retrospectively using the Abbott RealTimeTM IDH1 Assay. TIBSOVO was given orally at a starting dose of 500 mg daily continuous for 28-day cycles until disease progression, development of unacceptable toxicity, or undergoing hematopoietic stem cell transplantation. One (6%) of the 18 patients went on to stem cell transplantation following TIBSOVO treatment.<br>The baseline demographic and disease characteristics are shown in Table 19.<br>**14.4 Locally Advanced or Metastatic Cholangiocarcinoma**<br>The efficacy of TIBSOVO was evaluated in a randomized (2:1), multicenter, double-blind, placebo-controlled clinical trial (Study AG120-C-005, NCT02989857) of 185 adult patients with locally advanced or metastatic cholangiocarcinoma with an IDH1 mutation whose disease had progressed following at least 1 but not more than 2 prior regimens, including at least one gemcitabine- or 5-FU-containing regimen. Patients were randomized to receive either TIBSOVO 500 mg orally once daily or matched placebo until disease progression or unacceptable toxicity. Randomization was stratified by number of prior therapies (1 or 2). Eligible patients who were randomized to placebo were allowed to cross over to receive TIBSOVO after documented radiographic disease progression. Patients with IDH1 mutations were selected using a central diagnostic next generation sequencing assay. Tumor imaging assessments were performed every 6 weeks for the first 8 assessments and every 8 weeks thereafter.<br>The median age was 62 years (range: 33 to 83); 63% were female; 57% were White, 12% Asian, 1.1% Black, 0.5% Native Hawaiian/Other Pacific Islander, 0.5% American Indian or Alaska Native, 28% race missing/not reported; and 37% had an ECOG performance status of 0 (37%) or 1 (62%). All patients received at least 1 prior line of systemic therapy and 47% received two prior lines. Most patients had intrahepatic cholangiocarcinoma (91%) at diagnosis and 92% had metastatic disease. Across both arms, 70% patients had an R132C mutation, 15% had an R132L mutation, 12% had an R132G mutation, 1.1% had an R132H mutation, and 1.6% had an R132S mutation.
**022065, 10/18/2011**	Ixabepilone (1)	Oncology	ERBB2 (HER2)	Clinical Studies	**14 CLINICAL STUDIES**<br>**Combination Therapy**<br>(…) Sixty-seven percent of patients were White, 23% were Asian, and 3% were Black. Both arms were evenly matched with regards to race, age (median 53 years), baseline performance status (Karnofsky 70-100%), and receipt of prior adjuvant or neo-adjuvant chemotherapy (75%). Tumors were ER-positive in 47% of patients, ER-negative in 43%, HER2-positive in 15%, HER2-negative in 61%, and ER-negative, PR-negative, HER2 negative in 25%. The baseline disease characteristics and previous therapies for all patients (n=752) are shown in Table 6. (…)<br>**Monotherapy**<br>HER2-positive patients must also have progressed during or after discontinuation of trastuzumab.<br>In this study, the median age was 51 years (range, 30-78), and 79% were White, 5% Black, and 2% Asian, Karnofsky performance status was 70-100%, 88% had received two or more prior chemotherapy regimens for metastatic disease, and 86% had liver and/or lung metastases. Tumors were ER-positive in 48% of patients, ER-negative in 44%, HER2-positive in 7%, HER2-negative in 72%, and ER-negative, PR-negative, HER2-negative in 33%. (…)
**022065, 10/18/2011**	Ixabepilone (2)	Oncology	ESR, PGR (Hormone Receptor)	Clinical Studies	**14 CLINICAL STUDIES**<br>**Combination Therapy**<br>(…) Sixty-seven percent of patients were White, 23% were Asian, and 3% were Black. Both arms were evenly matched with regards to race, age (median 53 years), baseline performance status (Karnofsky 70-100%), and receipt of prior adjuvant or neo-adjuvant chemotherapy (75%). Tumors were ER-positive in 47% of patients, ER-negative in 43%, HER2-positive in 15%, HER2-negative in 61%, and ER-negative, PR-negative, HER2 negative in 25%. The baseline disease characteristics and previous therapies for all patients (n=752) are shown in Table 6. (…)<br>**Monotherapy**<br>HER2-positive patients must also have progressed during or after discontinuation of trastuzumab.<br>In this study, the median age was 51 years (range, 30-78), and 79% were White, 5% Black, and 2% Asian, Karnofsky performance status was 70-100%, 88% had received two or more prior chemotherapy regimens for metastatic disease, and 86% had liver and/or lung metastases. Tumors were ER-positive in 48% of patients, ER-negative in 44%, HER2-positive in 7%, HER2-negative in 72%, and ER-negative, PR-negative, HER2-negative in 33%. (…)
**204839, 04/28/2016**	Lacosamide	Neurology	CYP2C19	Clinical Pharmacology	**12 CLINICAL PHARMACOLOGY**<br>**12.3 Pharmacokinetics**<br>*Special Populations*<br>*CYP2C19 Polymorphism*<br>There are no clinically relevant differences in the pharmacokinetics of lacosamide between CYP2C19 poor metabolizers and extensive metabolizers. Results from a trial in poor metabolizers (PM) (N=4) and extensive metabolizers (EM) (N=8) of cytochrome P450 (CYP) 2C19 showed that lacosamide plasma concentrations were similar in PMs and EMs, but plasma concentrations and the amount excreted into urine of the O-desmethyl metabolite were about 70% reduced in PMs compared to EMs.
**020406, 06/07/2018**	Lansoprazole	Gastroenterology	CYP2C19	Drug Interactions, Clinical Pharmacology	**7 DRUG INTERACTIONS**<br>**7.3 Tacrolimus**<br>Concomitant administration of lansoprazole and tacrolimus may increase whole blood levels of tacrolimus, especially in transplant patients who are intermediate or poor metabolizers of CYP2C19.<br><br>**12 CLINICAL PHARMACOLOGY**

NDA/ANDA/BLA Number, Label Version Date	Drug	Therapeutic Area*	Biomarker†	Labeling Sections	Labeling Text‡
					**12.3 Pharmacokinetics**<br>*Drug-Drug Interactions*<br>*Clopidogrel*<br>Clopidogrel is metabolized to its active metabolite in part by CYP2C19. A study of healthy subjects who were CYP2C19 extensive metabolizers, receiving once daily administration of clopidogrel 75 mg alone or concomitantly with PREVACID 30 mg (n=40), for nine days was conducted. (…)
**022059, 12/06/2018**	Lapatinib (1)	Oncology	ERBB2 (HER2)	Indications and Usage, Dosage and Administration, Adverse Reactions, Use in Specific Populations, Clinical Studies	**1 INDICATIONS AND USAGE**<br>TYKERB® is indicated in combination with:<br>• capecitabine for the treatment of patients with advanced or metastatic breast cancer whose tumors overexpress HER2 and who have received prior therapy including an anthracycline, a taxane, and trastuzumab.<br>*Limitation of Use:* Patients should have disease progression on trastuzumab prior to initiation of treatment with TYKERB in combination with capecitabine.<br>• letrozole for the treatment of postmenopausal women with hormone receptor-positive metastatic breast cancer that overexpresses the HER2 receptor for whom hormonal therapy is indicated.<br>TYKERB in combination with an aromatase inhibitor has not been compared to a trastuzumab-containing chemotherapy regimen for the treatment of metastatic breast cancer.<br><br>**2 DOSAGE AND ADMINISTRATION**<br>**2.1 Recommended Dosing**<br>*HER2-Positive Metastatic Breast Cancer*<br>The recommended dose of TYKERB is 1,250 mg given orally once daily on Days 1-21 continuously in combination with capecitabine 2,000 mg/m^2/day (administered orally in 2 doses approximately 12 hours apart) on Days 1-14 in a repeating 21-day cycle. TYKERB should be taken at least one hour before or one hour after a meal. The dose of TYKERB should be once daily (5 tablets administered all at once); dividing the daily dose is not recommended [see Clinical Pharmacology (12.3)]. Capecitabine should be taken with food or within 30 minutes after food. If a day's dose is missed, the patient should not double the dose the next day. Treatment should be continued until disease progression or unacceptable toxicity occurs.<br>*Hormone Receptor-Positive, HER2-Positive Metastatic Breast Cancer*<br>The recommended dose of TYKERB is 1,500 mg given orally once daily continuously in combination with letrozole. When coadministered with TYKERB, the recommended dose of letrozole is 2.5 mg once daily. TYKERB should be taken at least one hour before or one hour after a meal. The dose of TYKERB should be once daily (6 tablets administered all at once); dividing the daily dose is not recommended [see Clinical Pharmacology (12.3)].<br>**2.2 Dose Modification Guidelines**<br>*Hepatic Impairment*<br>Patients with severe hepatic impairment (Child-Pugh Class C) should have their dose of TYKERB reduced. A dose reduction from 1,250 mg/day to 750 mg/day (HER2-positive metastatic breast cancer indication) or from 1,500 mg/day to 1,000 mg/day (hormone receptor-positive, HER2-positive breast cancer indication) in patients with severe hepatic impairment is predicted to adjust the area under the curve (AUC) to the normal range and should be considered. However, there are no clinical data with this dose adjustment in patients with severe hepatic impairment.<br>*Concomitant Strong CYP3A4 Inducers*<br>The concomitant use of strong CYP3A4 inducers should be avoided (e.g., dexamethasone, phenytoin, carbamazepine, rifampin, rifabutin, rifapentin, phenobarbital, St. John's wort). If patients must be coadministered a strong CYP3A4 inducer, based on pharmacokinetic studies, the dose of lapatinib should be titrated gradually from 1,250 mg/day up to 4,500 mg/day (HER2-positive metastatic breast cancer indication) or from 1,500 mg/day up to 5,500 mg/day (hormone receptor-positive, HER2-positive breast cancer indication) based on tolerability. (…)<br><br>**6 ADVERSE REACTIONS**<br>**6.1 Clinical Trials Experience**<br>*HER2-Positive Metastatic Breast Cancer*<br>The safety of TYKERB has been evaluated in more than 12,000 patients in clinical trials. (…)<br>*Hormone Receptor-Positive, HER2+ Metastatic Breast Cancer:*<br>In another randomized, Phase 3 clinical trial of postmenopausal patients (N = 355) with hormone receptor positive (HR+), HER2-positive metastatic breast cancer (MBC) which had progressed after prior trastuzumab-containing chemotherapy and endocrine therapies patients received TYKERB with trastuzumab and an aromatase inhibitor (AI) (letrozole, exemestane, or anastrozole), TYKERB with an AI, or trastuzumab with an AI. (…)<br>*Decreases in Left Ventricular Ejection Fraction*<br>(…) Due to potential cardiac toxicity with HER2 (ErbB2) inhibitors, LVEF was monitored in clinical trials at approximately 8-week intervals. (…)<br><br>**8 USE IN SPECIFIC POPULATIONS**<br>**8.5 Geriatric Use**<br>Of the total number of metastatic breast cancer patients in clinical studies of TYKERB in combination with capecitabine (N = 198), 17% were 65 years of age and older, and 1% were 75 years of age and older. Of the total number of hormone receptor-positive, HER2-positive metastatic breast cancer patients in clinical studies of TYKERB in combination with letrozole (N = 642), 44% were 65 years of age and older, and 12% were 75 years of age and older. (…)<br><br>**14 CLINICAL STUDIES**<br>**14.1 HER2-Positive Metastatic Breast Cancer**

* Therapeutic areas do not necessarily reflect the CDER review division.
† Representative biomarkers are listed based on standard nomenclature as per the Human Genome Organization (HUGO) symbol and/or simplified descriptors using other common conventions. Listed biomarkers do not necessarily reflect the terminology used in labeling. The term "Nonspecific" is provided when labeling does not explicitly identify the specific biomarker(s) or when the biomarker is represented by a molecular phenotype or gene signature, and in some cases the biomarker was inferred based on the labeling language.
‡ Referenced figures and tables may be found in the labeling available at Drugs@FDA.
Blue text represents the most recent addtions and/or changes since last posted version.

NDA/ANDA/BLA Number, Label Version Date	Drug	Therapeutic Area*	Biomarker†	Labeling Sections	Labeling Text‡
					The efficacy and safety of TYKERB in combination with capecitabine in breast cancer were evaluated in a randomized, Phase 3 trial. Patients eligible for enrollment had HER2 (ErbB2) overexpressing (IHC 3+ or IHC 2+ confirmed by FISH), locally advanced or metastatic breast cancer, progressing after prior treatment that included anthracyclines, taxanes, and trastuzumab. (…)<br>(…) Ninety-seven percent (97%) had stage IV breast cancer, 48% were estrogen receptor+ (ER+) or progesterone receptor+ (PR+), and 95% were ErbB2 IHC 3+ or IHC 2+ with FISH confirmation. Approximately 95% of patients had prior treatment with anthracyclines, taxanes, and trastuzumab. (…)<br>(…) Clinical Studies Describing Limitation of Use: In two randomized trials, TYKERB based chemotherapy regimens have been shown to be less effective than trastuzumab-based chemotherapy regimens. The first randomized, open-label study compared the safety and efficacy of TYKERB in combination with capecitabine relative to trastuzumab in combination with capecitabine in women with HER2-positive metastatic breast cancer (N = 540). (…)<br>(…) The second randomized, open-label study compared the safety and efficacy of taxane-based chemotherapy plus TYKERB to taxane-based chemotherapy plus trastuzumab as first-line therapy in women with HER2-positive, metastatic breast cancer (N = 652). (…)<br>**14.2 Hormone Receptor-Positive, HER2-Positive Metastatic Breast Cancer**<br>The efficacy and safety of TYKERB in combination with letrozole were evaluated in a double-blind, placebo-controlled, multi-center study. A total of 1,286 postmenopausal women with hormone receptor-positive (ER positive and/or PgR positive) metastatic breast cancer, who had not received prior therapy for metastatic disease, were randomly assigned to receive either TYKERB (1,500 mg once daily) (n = 642) or letrozole (2.5 mg once daily) alone (n = 644). Of all patients randomized to treatment, 219 (17%) patients had tumors overexpressing the HER2 receptor, defined as fluorescence in situ hybridization (FISH) ≥2 or 3+ immunohistochemistry (IHC). There were 952 (74%) patients who were HER2- negative and 115 (9%) patients did not have their HER2 receptor status confirmed. The primary objective was to evaluate and compare progression-free survival (PFS) in the HER2-positive population. Progression-free survival was defined as the interval of time between date of randomization and the earlier date of first documented sign of disease progression or death due to any cause.<br>The baseline demographic and disease characteristics were balanced between the two treatment arms. The median age was 63 years and 45% were 65 years of age or older. Eighty-four percent (84%) of the patients were white. Approximately 50% of the HER2-positive population had prior adjuvant/neo-adjuvant chemotherapy and 56% had prior hormonal therapy. Only 2 patients had prior trastuzumab.<br>In the HER2-positive subgroup (n = 219), the addition of TYKERB to letrozole resulted in an improvement in PFS. In the HER2-negative subgroup, there was no improvement in PFS of the combination of TYKERB plus letrozole compared to the letrozole plus placebo. Overall response rate (ORR) was also improved with the combination of TYKERB plus letrozole. The overall survival (OS) data were not mature. Efficacy analyses for the hormone receptor-positive, HER2-positive and HER2-negative subgroups are presented in Table 8 and Figure 3. (See Table 8 and Figure 3)<br>The efficacy and safety of TYKERB, in combination with an aromatase inhibitor (AI), were confirmed in another randomized Phase 3 trial. Patients enrolled were post-menopausal women who had hormone receptorpositive (HR+)/HER2-positive, metastatic breast cancer, which had progressed after prior trastuzumabcontaining chemotherapy and endocrine therapies. (…)<br>The study was designed to evaluate a potential benefit in Progression Free Survival (PFS) when double versus single HER2 targeted therapy was administered in combination with an AI (letrozole, exemestane, or anastrozole). The major efficacy outcome measure was PFS based on local radiology/investigator's assessment comparing TYKERB + trastuzumab + AI versus trastuzumab + AI. (…)
**022059, 12/06/2018**	Lapatinib (2)	Oncology	ESR, PGR (Hormone Receptor)	Indications and Usage, Dosage and Administration, Adverse Reactions, Use in Specific Populations, Clinical Studies	**1 INDICATIONS AND USAGE**<br>TYKERB is indicated in combination with: (…)<br>• letrozole for the treatment of postmenopausal women with hormone receptor-positive metastatic breast cancer that overexpresses the HER2 receptor for whom hormonal therapy is indicated. (…)<br><br>**2 DOSAGE AND ADMINISTRATION**<br>**2.1 Recommended Dosing**<br>*Hormone Receptor-Positive, HER2-Positive Metastatic Breast Cancer*<br>The recommended dose of TYKERB is 1,500 mg given orally once daily continuously in combination with letrozole. When coadministered with TYKERB, the recommended dose of letrozole is 2.5 mg once daily. TYKERB should be taken at least one hour before or one hour after a meal. The dose of TYKERB should be once daily (6 tablets administered all at once); dividing the daily dose is not recommended [see Clinical Pharmacology (12.3)].<br>**2.2 Dose Modification Guidelines**<br>*Hepatic Impairment*<br>Patients with severe hepatic impairment (Child-Pugh Class C) should have their dose of TYKERB reduced. A dose reduction from 1,250 mg/day to 750 mg/day (HER2-positive metastatic breast cancer indication) or from 1,500 mg/day to 1,000 mg/day (hormone receptor-positive, HER2-positive breast cancer indication) in patients with severe hepatic impairment is predicted to adjust the area under the curve (AUC) to the normal range and should be considered. However, there are no clinical data with this dose adjustment in patients with severe hepatic impairment.<br>*Concomitant Strong CYP3A4 Inducers*<br>The concomitant use of strong CYP3A4 inducers should be avoided (e.g., dexamethasone, phenytoin, carbamazepine, rifampin, rifabutin, rifapentin, phenobarbital, St. John's wort). If patients must be coadministered a strong CYP3A4 inducer, based on pharmacokinetic studies, the dose of lapatinib should be titrated gradually from 1,250 mg/day up to 4,500 mg/day (HER2-positive metastatic breast cancer indication) or from 1,500 mg/day up to 5,500 mg/day (hormone receptor-positive, HER2-positive breast cancer indication) based on tolerability.<br><br>**6 ADVERSE REACTIONS**<br>**6.1 Clinical Trials Experience**<br>(…) Hormone Receptor-Positive, Metastatic Breast Cancer: In a randomized clinical trial of patients (N = 1,286) with hormone receptor-positive, metastatic breast cancer, who had not received chemotherapy for their metastatic disease, patients received letrozole with or without TYKERB. (…)

* Therapeutic areas do not necessarily reflect the CDER review division.
† Representative biomarkers are listed based on standard nomenclature as per the Human Genome Organization (HUGO) symbol and/or simplified descriptors using other common conventions. Listed biomarkers do not necessarily reflect the terminology used in labeling. The term "Nonspecific" is provided when labeling does not explicitly identify the specific biomarker(s) or when the biomarker is represented by a molecular phenotype or gene signature, and in some cases the biomarker was inferred based on the labeling language.
‡ Referenced figures and tables may be found in the labeling available at Drugs@FDA.
Blue text represents the most recent addtions and/or changes since last posted version.

NDA/ANDA/BLA Number, Label Version Date	Drug	Therapeutic Area*	Biomarker†	Labeling Sections	Labeling Text‡
					Hormone Receptor-Positive, HER2+ Metastatic Breast Cancer: In another randomized, Phase 3 clinical trial of postmenopausal patients (N = 355) with hormone receptor positive (HR+), HER2-positive metastatic breast cancer (MBC) which had progressed after prior trastuzumab-containing chemotherapy and endocrine therapies patients received TYKERB with trastuzumab and an aromatase inhibitor (AI) (letrozole, exemestane, or anastrozole), TYKERB with an AI, or trastuzumab with an AI. (…)<br><br>**8 USE IN SPECIFIC POPULATIONS**<br>**8.5 Geriatric Use**<br>Of the total number of metastatic breast cancer patients in clinical studies of TYKERB in combination with capecitabine (N = 198), 17% were 65 years of age and older, and 1% were 75 years of age and older. Of the total number of hormone receptor-positive, HER2-positive metastatic breast cancer patients in clinical studies of TYKERB in combination with letrozole (N = 642), 44% were 65 years of age and older, and 12% were 75 years of age and older. (…)<br><br>**14 CLINICAL STUDIES**<br>**14.1 HER2-Positive Metastatic Breast Cancer**<br>(…) Ninety-seven percent (97%) had stage IV breast cancer, 48% were estrogen receptor+ (ER+) or progesterone receptor+ (PR+), and 95% were ErbB2 IHC 3+ or IHC 2+ with FISH confirmation. (…)<br>**14.2 Hormone Receptor-Positive, HER2-Positive Metastatic Breast Cancer**<br>The efficacy and safety of TYKERB in combination with letrozole were evaluated in a double-blind, placebo-controlled, multi-center study. A total of 1,286 postmenopausal women with hormone receptor-positive (ER positive and/or PgR positive) metastatic breast cancer, who had not received prior therapy for metastatic disease, were randomly assigned to receive either TYKERB (1,500 mg once daily) plus letrozole (2.5 mg once daily) (n = 642) or letrozole (2.5 mg once daily) alone (n = 644). (…)<br>(…) In the HER2-positive subgroup (n = 219), the addition of TYKERB to letrozole resulted in an improvement in PFS. In the HER2-negative subgroup, there was no improvement in PFS of the combination of TYKERB plus letrozole compared to the letrozole plus placebo. Overall response rate (ORR) was also improved with the combination of TYKERB plus letrozole. The overall survival (OS) data were not mature. Efficacy analyses for the hormone receptor-positive, HER2-positive and HER2-negative subgroups are presented in Table 8 and Figure 3. (…)<br>The efficacy and safety of TYKERB, in combination with an aromatase inhibitor (AI), were confirmed in another randomized Phase 3 trial. Patients enrolled were post-menopausal women who had hormone receptorpositive (HR+)/HER2-positive, metastatic breast cancer, which had progressed after prior trastuzumabcontaining chemotherapy and endocrine therapies. (…)
**022059, 12/06/2018**	**Lapatinib (3)**	Oncology	HLA-DQA1	Clinical Pharmacology	**12 CLINICAL PHARMACOLOGY**<br>**12.5 Pharmacogenomics**<br>The HLA alleles DQA1*02:01 and DRB1*07:01 were associated with hepatotoxicity reactions in a genetic substudy of a monotherapy trial with TYKERB (n = 1,194). Severe liver injury (ALT >5 times the upper limit of normal, NCI CTCAE Grade 3) occurred in 2% of patients overall; the incidence of severe liver injury among DQA1*02:01 or DRBI*07:01 allele carriers was 8% versus 0.5% in non-carriers. These HLA alleles are present in approximately<br>15% to 25% of Caucasian, Asian, African, and Hispanic populations and 1% in Japanese populations. Liver function should be monitored in all patients receiving therapy with TYKERB regardless of genotype.
**022059, 12/06/2018**	**Lapatinib (4)**	Oncology	HLA-DRB1	Clinical Pharmacology	**12 CLINICAL PHARMACOLOGY**<br>**12.5 Pharmacogenomics**<br>The HLA alleles DQA1*02:01 and DRB1*07:01 were associated with hepatotoxicity reactions in a genetic substudy of a monotherapy trial with TYKERB (n = 1,194). Severe liver injury (ALT >5 times the upper limit of normal, NCI CTCAE Grade 3) occurred in 2% of patients overall; the incidence of severe liver injury among DQA1*02:01 or DRBI*07:01 allele carriers was 8% versus 0.5% in non-carriers. These HLA alleles are present in approximately<br>15% to 25% of Caucasian, Asian, African, and Hispanic populations and 1% in Japanese populations. Liver function should be monitored in all patients receiving therapy with TYKERB regardless of genotype.
210861, 11/23/2022	**Larotrectinib**	Oncology	NTRK	Indications and Usage, Dosage and Administration, Adverse Reactions, Clinical Studies	**1 INDICATIONS AND USAGE**<br>VITRAKVI is indicated for the treatment of adult and pediatric patients with solid tumors that:<br>• have a neurotrophic receptor tyrosine kinase (NTRK) gene fusion without a known acquired resistance mutation, (…)<br><br>**2 DOSAGE AND ADMINISTRATION**<br>**2.1 Patient Selection**<br>Select patients for treatment with VITRAKVI based on the presence of a NTRK gene fusion in tumor specimens [see Clinical Studies (14)]. Information on FDA-approved tests is available at http://www.fda.gov/companiondiagnostics.<br><br>**6 ADVERSE REACTIONS**<br>**6.1 Clinical Trial Experience**<br>(…) Across these 279 patients, the median age was 46 years (range: 0.1 days to 84 years); 33% were younger than 18 years; 48% were male; and 74% were White, 9% were Hispanic/Latino, 7% were Asian and, 6% were Black. The most common tumors (≥3%) in order of decreasing frequency were soft tissue sarcoma (17%), thyroid (13%), infantile fibrosarcoma (12%), primary central nervous system (CNS) (11%), salivary gland (9%), lung (9%), colon (6%), breast (4%), or melanoma (3%). NTRK gene fusions were confirmed or inferred in 75% of VITRAKVItreated patients. (…)<br><br>**14 CLINICAL STUDIES**

* Therapeutic areas do not necessarily reflect the CDER review division.
† Representative biomarkers are listed based on standard nomenclature as per the Human Genome Organization (HUGO) symbol and/or simplified descriptors using other common conventions. Listed biomarkers do not necessarily reflect the terminology used in labeling. The term “Nonspecific” is provided when labeling does not explicitly identify the specific biomarker(s) or when the biomarker is represented by a molecular phenotype or gene signature, and in some cases the biomarker was inferred based on the labeling language.
‡ Referenced figures and tables may be found in the labeling available at Drugs@FDA.
Blue text represents the most recent addtions and/or changes since last posted version.

Table of Pharmacogenomic Biomarkers in Drug Labeling
Last Updated: 06/2024

NDA/ANDA/BLA Number, Label Version Date	Drug	Therapeutic Area*	Biomarker†	Labeling Sections	Labeling Text‡
					The efficacy of VITRAKVI was evaluated in pediatric and adult patients with unresectable or metastatic solid tumors with a NTRK gene fusion enrolled in one of three multicenter, open-label, single-arm clinical trials: Study LOXO-TRK-14001 (NCT02122913), SCOUT (NCT02637687), and NAVIGATE (NCT02576431).<br>(…)<br>(…) Identification of positive NTRK gene fusion status was prospectively determined in local laboratories using next generation sequencing (NGS) or fluorescence in situ hybridization (FISH). NTRK gene fusions were inferred in three patients with infantile fibrosarcoma who had a documented ETV6 translocation identified by FISH. (…)<br>(…) The assessment of efficacy was based on the first 55 patients with solid tumors with an NTRK gene fusion enrolled across the three clinical trials. (…) The most common cancers were salivary gland tumors (22%), soft tissue sarcoma (20%), infantile fibrosarcoma (13%), and thyroid cancer (9%). A total of 50 patients had NTRK gene fusions detected by NGS and 5 patients had NTRK gene fusions detected by FISH.<br>Efficacy results are summarized in Tables 4, 5, and 6.
761269, 07/06/2023	Lecanemab-irmb	Neurology	APOE	Boxed Warning, Warnings and Precautions, Clinical Studies, Patient Counseling Information	**BOXED WARNING**<br>**ApoE ε4 Homozygotes**<br>Patients who are apolipoprotein E ε4 (ApoE ε4) homozygotes (approximately 15% of Alzheimer's disease patients) treated with this class of medications, including LEQEMBI, have a higher incidence of ARIA, including symptomatic, serious, and severe radiographic ARIA, compared to heterozygotes and noncarriers. Testing for ApoE ε4 status should be performed prior to initiation of treatment to inform the risk of developing ARIA. Prior to testing, prescribers should discuss with patients the risk of ARIA across genotypes and the implications of genetic testing results. Prescribers should inform patients that if genotype testing is not performed they can still be treated with LEQEMBI; however, it cannot be determined if they are ApoE ε4 homozygotes and at higher risk for ARIA [see Warnings and Precautions (5.1)].<br>Consider the benefit of LEQEMBI for the treatment of Alzheimer's disease and potential risk of serious adverse events associated with ARIA when deciding to initiate treatment with LEQEMBI [see Warnings and Precautions (5.1) and Clinical Studies (14)].<br><br>**5 WARNINGS AND PRECAUTIONS**<br>**5.1 Amyloid Related Imaging Abnormalities**<br>Monoclonal antibodies directed against aggregated forms of beta amyloid, including LEQEMBI, can cause amyloid related imaging abnormalities (ARIA), characterized as ARIA with edema (ARIA-E), which can be observed on MRI as brain edema or sulcal effusions, and ARIA with hemosiderin deposition (ARIA-H), which includes microhemorrhage and superficial siderosis. ARIA can occur spontaneously in patients with Alzheimer's disease. ARIA-H associated with monoclonal antibodies directed against aggregated forms of beta amyloid generally occurs in association with an occurrence of ARIA-E. ARIA-H of any cause and ARIA-E can occur together.<br>ARIA usually occurs early in treatment and is usually asymptomatic, although serious and life-threatening events, including seizure and status epilepticus, rarely can occur. When present, reported symptoms associated with ARIA may include headache, confusion, visual changes, dizziness, nausea, and gait difficulty. Focal neurologic deficits may also occur. Symptoms associated with ARIA usually resolve over time. The risk of ARIA, including symptomatic and serious ARIA, is increased in apolipoprotein E ε4 (ApoE ε4) homozygotes. In addition to ARIA, intracerebral hemorrhages greater than 1 cm in diameter have occurred in patients treated with LEQEMBI.<br>Consider the benefit of LEQEMBI for the treatment of Alzheimer's disease and potential risk of serious adverse events associated with ARIA when deciding to initiate treatment with LEQEMBI.<br>Incidence of ARIA<br>Symptomatic ARIA occurred in 3% (29/898) of patients treated with LEQEMBI in Study 2 [see Clinical Studies (14)]. Serious symptoms associated with ARIA were reported in 0.7% (6/898) of patients treated with LEQEMBI. Clinical symptoms associated with ARIA resolved in 79% (23/29) of patients during the period of observation. Similar findings were observed in Study 1.<br>Including asymptomatic radiographic events, ARIA was observed in 21% (191/898) of patients treated with LEQEMBI, compared to 9% (84/897) of patients on placebo in Study 2.<br>ARIA-E was observed in 13% (113/898) of patients treated with LEQEMBI compared with 2% (15/897) of patients on placebo. ARIA-H was observed in 17% (152/898) of patients treated with LEQEMBI compared with 9% (80/897) of patients on placebo. There was no increase in isolated ARIA-H (i.e., ARIA-H in patients who did not also experience ARIA-E) for LEQEMBI compared to placebo.<br>ApoE ε4 Carrier Status and Risk of ARIA<br>Approximately 15% of Alzheimer's disease patients are ApoE ε4 homozygotes. In Study 2, 16% (141/898) of patients in the LEQEMBI arm were ApoE ε4 homozygotes, 53% (479/898) were heterozygotes, and 31% (278/898) were noncarriers. The incidence of ARIA was higher in ApoE ε4 homozygotes (45% on LEQEMBI vs. 22% on placebo) than in heterozygotes (19% on LEQEMBI vs 9% on placebo) and noncarriers (13% on LEQEMBI vs 4% on placebo). Among patients treated with LEQEMBI, symptomatic ARIA-E occurred in 9% of ApoE ε4 homozygotes compared with 2% of heterozygotes and 1% noncarriers. Serious events of ARIA occurred in 3% of ApoE ε4 homozygotes, and approximately 1% of heterozygotes and noncarriers. The recommendations on management of ARIA do not differ between ApoE ε4 carriers and noncarriers [see Dosage and Administration (2.3)]. Testing for ApoE ε4 status should be performed prior to initiation of treatment to inform the risk of developing ARIA. Prior to testing, prescribers should discuss with patients the risk of ARIA across genotypes and the implications of genetic testing results. Prescribers should inform patients that if genotype testing is not performed they can still be treated with LEQEMBI; however, it cannot be determined if they are ApoE ε4 homozygotes and at higher risk for ARIA. An FDA-authorized test for the detection of ApoE ε4 alleles to identify patients at risk of ARIA if treated with LEQEMBI is not currently available. Currently available tests used to identify ApoE ε4 alleles may vary in accuracy and design. (See Table 3)<br>The majority of ARIA-E radiographic events occurred early in treatment (within the first 7 doses), although ARIA can occur at any time and patients can have more than 1 episode. The maximum radiographic severity of ARIA-E in patients treated with LEQEMBI was mild in 4% (37/898) of patients, moderate in 7% (66/898) of patients, and severe in 1% (9/898) of patients. Resolution on MRI occurred in 52% of ARIA-E patients by 12 weeks, 81% by 17 weeks, and 100% overall after detection. The maximum radiographic severity of ARIA-H microhemorrhage in patients treated with LEQEMBI was mild in 9% (79/898), moderate in

* Therapeutic areas do not necessarily reflect the CDER review division.
† Representative biomarkers are listed based on standard nomenclature as per the Human Genome Organization (HUGO) symbol and/or simplified descriptors using other common conventions. Listed biomarkers do not necessarily reflect the terminology used in labeling. The term "Nonspecific" is provided when labeling does not explicitly identify the specific biomarker(s) or when the biomarker is represented by a molecular phenotype or gene signature, and in some cases the biomarker was inferred based on the labeling language.
‡ Referenced figures and tables may be found in the labeling available at Drugs@FDA.
Blue text represents the most recent addtions and/or changes since last posted version.

NDA/ANDA/BLA Number, Label Version Date	Drug	Therapeutic Area*	Biomarker†	Labeling Sections	Labeling Text‡
					2% (19/898), and severe in 3% (28/898) of patients; superficial siderosis was mild in 4% (38/898), moderate in 1% (8/898), and severe in 0.4% (4/898). Among patients treated with LEQEMBI, the rate of severe radiographic ARIA-E was highest in ApoE ε4 homozygotes 5% (7/141), compared to heterozygotes 0.4% (2/479) or noncarriers 0% (0/278). Among patients treated with LEQEMBI, the rate of severe radiographic ARIA-H was highest in ApoE ε4 homozygotes 13.5% (19/141), compared to heterozygotes 2.1% (10/479) or noncarriers 1.1% (3/278).<br>Intracerebral Hemorrhage<br>Intracerebral hemorrhage greater than 1 cm in diameter was reported in 0.7% (6/898) of patients in Study 2 after treatment with LEQEMBI compared to 0.1% (1/897) on placebo. Fatal events of intracerebral hemorrhage in patients taking LEQEMBI have been observed. Concomitant Antithrombotic Medication In Study 2, baseline use of antithrombotic medication (aspirin, other antiplatelets, or anticoagulants) was allowed if the patient was on a stable dose. The majority of exposures to antithrombotic medications were to aspirin. Antithrombotic medications did not increase the risk of ARIA with LEQEMBI. The incidence of intracerebral hemorrhage was 0.9% (3/328 patients) in patients taking LEQEMBI with a concomitant antithrombotic medication at the time of the event compared to 0.6% (3/545 patients) in those who did not receive an antithrombotic. Patients taking LEQEMBI with an anticoagulant alone or combined with an antiplatelet medication or aspirin had an incidence of intracerebral hemorrhage of 2.5% (2/79 patients) compared to none in patients who received placebo.<br>Because intracerebral hemorrhages greater than 1 cm in diameter have been observed in patients taking LEQEMBI, additional caution should be exercised when considering the administration of anticoagulants or a thrombolytic agent (e.g., tissue plasminogen activator) to a patient already being treated with LEQEMBI.<br>*Other Risk Factors for Intracerebral Hemorrhage*<br>Patients were excluded from enrollment in Study 2 for findings on neuroimaging that indicated an increased risk for intracerebral hemorrhage. These included findings suggestive of cerebral amyloid angiopathy (prior cerebral hemorrhage greater than 1 cm in greatest diameter, more than 4 microhemorrhages, superficial siderosis, vasogenic edema) or other lesions (aneurysm, vascular malformation) that could potentially increase the risk of intracerebral hemorrhage. The presence of an ApoE ε4 allele is also associated with cerebral amyloid angiopathy, which has an increased risk for intracerebral hemorrhage.<br>Caution should be exercised when considering the use of LEQEMBI in patients with factors that indicate an increased risk for intracerebral hemorrhage and in particular for patients who need to be on anticoagulant therapy.<br><br>**14 CLINICAL STUDIES**<br>**(…)** In Study 1, 856 patients were randomized to receive one of 5 doses (161 of which were randomized to the recommended dosing regimen of 10 mg/kg every two weeks) of LEQEMBI or placebo (n=247). Of the total number of patients randomized, 71.4% were ApoE ε4 carriers and 28.6% were ApoE ε4 non-carriers. During the study the protocol was amended to no longer randomize ApoE ε4 carriers to the 10 mg/kg every two weeks dose arm. ApoE ε4 carriers who had been receiving LEQEMBI 10 mg/kg every two weeks for 6 months or less were discontinued from study drug. As a result, in the LEQEMBI 10 mg/kg every two weeks arm, 30.3% of patients were ApoE ε4 carriers and 69.7% were ApoE ε4 non-carriers. At baseline, the mean age of randomized patients was 71 years, with a range of 50 to 90 years. Fifty percent of patients were male and 90% were White. (…)<br>Study 2<br>In Study 2, 1795 patients were enrolled and randomized 1:1 to receive LEQEMBI 10 mg/kg or placebo once every 2 weeks. Of the total number of patients randomized, 69% were ApoE ε4 carriers and 31% were ApoE ε4 non-carriers. Overall median age of patients was 72 years, with a range of 50 to 90 years. Fifty-two percent were women, and 1381 (77%) were White, 303 (17%) were Asian, and 47 (3%) were Black. (…)<br>Both ApoE ε4 carriers and ApoE ε4 noncarriers showed statistically significant treatment differences for the primary endpoint and all secondary endpoints. In an exploratory subgroup analysis of ApoE ε4 homozygotes, which represented 15% of the trial population, a treatment effect was not observed with LEQEMBI treatment on the primary endpoint, CDR-SB, compared to placebo, although treatment effects that favored LEQEMBI were observed for the secondary clinical endpoints, ADAS-Cog 14 and ADCS MCI-ADL. Treatment effects on disease-relevant biomarkers (amyloid beta PET, plasma Aβ42/40 ratio, plasma p-tau 181) also favored LEQEMBI in the ApoE ε4 homozygous subgroup.<br><br>**17 PATIENT COUNSELING INFORMATION**<br>Amyloid Related Imaging Abnormalities<br>(…) Inform patients that although ARIA can occur in any patient treated with LEQEMBI, there is an increased risk in patients who are ApoE ε4 homozygotes and that testing for ApoE ε4 status should be performed prior to initiation of treatment to inform the risk of developing ARIA. Prior to testing, discuss with patients the risk of ARIA across genotypes and the implications of genetic testing results. Inform patients that if testing is not performed, it cannot be determined if they are ApoE ε4 homozygotes and at a higher risk for ARIA.
**205834, 11/09/2017**	**Ledipasvir and Sofosbuvir**	Infectious Diseases	IFNL3 (IL28B)	Clinical Studies	**14 CLINICAL STUDIES**<br>**14.2 Clinical Trials in Subjects with Genotype 1 HCV**<br>*Treatment-Naïve Adults without Cirrhosis — ION-3 (Study 0108)*<br>(…) Demographics and baseline characteristics were balanced across the treatment groups. Of the 647 treated subjects, the median age was 55 years (range: 20 to 75); 58% of the subjects were male; 78% were White; 19% were Black; 6% were Hispanic or Latino; mean body mass index was 28 kg/m^2 (range: 18 to 56 kg/m^2); 81% had baseline HCV RNA levels greater than or equal to 800,000 IU per mL; 80% had genotype 1a HCV infection; 73% had non-C/C IL28B alleles (CT or TT). (…)<br>*Treatment-Naïve Adults with or without Cirrhosis — ION-1 (Study 0102)*<br>(…) Demographics and baseline characteristics were balanced across the treatment groups. Of the 865 treated subjects, the median age was 54 years (range: 18 to 80); 59% of the subjects were male; 85% were White; 12% were Black; 12% were Hispanic or Latino; mean body mass index was 27 kg/m^2 (range: 18 to 48 kg/m^2); 79% had baseline HCV RNA levels greater than or equal to 800,000 IU per mL; 67% had genotype 1a HCV infection; 70% had non-C/C IL28B alleles (CT or TT); and 16% had cirrhosis. (…)<br>*Previously-Treated Adults with or without Cirrhosis — ION-2 (Study 0109)*

* Therapeutic areas do not necessarily reflect the CDER review division.
† Representative biomarkers are listed based on standard nomenclature as per the Human Genome Organization (HUGO) symbol and/or simplified descriptors using other common conventions. Listed biomarkers do not necessarily reflect the terminology used in labeling. The term “Nonspecific” is provided when labeling does not explicitly identify the specific biomarker(s) or when the biomarker is represented by a molecular phenotype or gene signature, and in some cases the biomarker was inferred based on the labeling language.
‡ Referenced figures and tables may be found in the labeling available at Drugs@FDA.
Blue text represents the most recent addtions and/or changes since last posted version.

NDA/ANDA/BLA Number, Label Version Date	Drug	Therapeutic Area*	Biomarker†	Labeling Sections	Labeling Text‡
					(…) Demographics and baseline characteristics were balanced across the treatment groups. Of the 440 treated subjects, the median age was 57 years (range: 24 to 75); 65% of the subjects were male; 81% were White; 18% were Black; 9% were Hispanic or Latino; mean body mass index was 28 kg/m^2 (range: 19 to 50 kg/m^2); 89% had baseline HCV RNA levels greater than or equal to 800,000 IU per mL; 79% had genotype 1a HCV infection; 88% had non-C/C IL28B alleles (CT or TT); and 20% had cirrhosis. Forty-seven percent (47%) of the subjects failed a prior therapy of pegylated interferon and ribavirin. Among these subjects, 49% were relapse/breakthrough and 51% were non-responder. Fifty-three percent (53%) of the subjects failed a prior therapy of pegylated interferon and ribavirin with an HCV protease inhibitor. Among these subjects, 62% were relapse/breakthrough and 38% were non-responder. (See Table 14) (…)<br>*Previously-Treated Adults with Cirrhosis ─ SIRIUS (Study 0121)*<br>(…) Demographics and baseline characteristics were balanced across the treatment groups. Of the 155 randomized subjects, the median age was 56 years (range: 23 to 77); 74% of the subjects were male; 97% were White; mean body mass index was 27 kg/m^2 (range: 19 to 47 kg/m^2); 63% had genotype 1a HCV infection; 94% had non-C/C IL28B alleles (CT or TT). One subject discontinued therapy while on placebo, and was not included in the efficacy analysis. (…)<br>**14.4 Clinical Trials in Subjects Coinfected with HCV and HIV-1**<br>(…) Of the 335 treated subjects, the median age was 52 years (range: 26 to 72); 82% of the subjects were male; 61% were White; 34% were Black; mean body mass index was 27 kg/m^2 (range: 18 to 66 kg/m^2); 75% had genotype 1a HCV infection; 2% had genotype 4 infection; 76% had non-C/C IL28B alleles (CT or TT); and 20% had compensated cirrhosis. Fifty-five percent (55%) of the subjects were treatment-experienced. (…)<br>(…) SVR12 rates were 94% (63/67) in subjects with cirrhosis and 98% (46/47) in subjects who were previously-treated and had cirrhosis. The relapse rate in the ION-4 trial in Black subjects was 9% (10/115), all of whom were IL28B non-CC genotype, and none in non-Black subjects (0/220). In the ION-1, ION-2, and ION-3 HCV mono-infection studies, relapse rates were 3% (10/305) in Black subjects and 2% (26/1637) in non-Black subjects. (…)
**021880, 05/28/2019**	**Lenalidomide**	Hematology	Chromosome 5q	Boxed Warning, Indications and Usage, Adverse Reactions, Use in Specific Populations, Clinical Studies	**BOXED WARNING**<br>WARNING: EMBRYO-FETAL TOXICITY, HEMATOLOGIC TOXICITY, and VENOUS and ARTERIAL THROMBOEMBOLISM<br>*Hematologic Toxicity (Neutropenia and Thrombocytopenia)*<br>REVLIMID can cause significant neutropenia and thrombocytopenia. Eighty percent of patients with del 5q myelodysplastic syndromes had to have a dose delay/reduction during the major study. Thirty-four percent of patients had to have a second dose delay/reduction. Grade 3 or 4 hematologic toxicity was seen in 80% of patients enrolled in the study. Patients on therapy for del 5q myelodysplastic syndromes should have their complete blood counts monitored weekly for the first 8 weeks of therapy and at least monthly thereafter. Patients may require dose interruption and/or reduction. Patients may require use of blood product support and/or growth factors [see Dosage and Administration (2.2)].<br><br>**1 INDICATIONS AND USAGE**<br>**1.2 Myelodysplastic Syndromes**<br>REVLIMID is indicated for the treatment of patients with transfusion-dependent anemia due to low- or intermediate-1-risk myelodysplastic syndromes (MDS) associated with a deletion 5q cytogenetic abnormality with or without additional cytogenetic abnormalities.<br><br>**6 ADVERSE REACTIONS**<br>**6.1 Clinical Trials Experience**<br>*Myelodysplastic Syndromes*<br>A total of 148 patients received at least 1 dose of 10 mg REVLIMID in the del 5q MDS clinical study. At least one adverse event was reported in all of the 148 patients who were treated with the 10 mg starting dose of REVLIMID. The most frequently reported adverse events were related to blood and lymphatic system disorders, skin and subcutaneous tissue disorders, gastrointestinal disorders, and general disorders and administrative site conditions.<br>Thrombocytopenia (61.5%; 91/148) and neutropenia (58.8%; 87/148) were the most frequently reported adverse events. The next most common adverse events observed were diarrhea (48.6%; 72/148), pruritus (41.9%; 62/148), rash (35.8%; 53/148) and fatigue (31.1%; 46/148). Table 8 summarizes the adverse events that were reported in ≥ 5% of the REVLIMID treated patients in the del 5q MDS clinical study. Table 9 summarizes the most frequently observed Grade 3 and Grade 4 adverse reactions regardless of relationship to treatment with REVLIMID. In the single-arm studies conducted, it is often not possible to distinguish adverse events that are drug-related and those that reflect the patient's underlying disease. (See Tables 9 and 10)<br><br>**8 USE IN SPECIFIC POPULATIONS**<br>**8.5 Geriatric Use**<br>(…) Of the 148 patients with del 5q MDS enrolled in the major study, 38% were age 65 and over, while 33% were age 75 and over. (…)<br><br>**14 CLINICAL STUDIES**<br>**14.2 Myelodysplastic Syndromes (MDS) with a Deletion 5q Cytogenetic Abnormality**<br>The efficacy and safety of REVLIMID were evaluated in patients with transfusion-dependent anemia in low- or intermediate-1- risk MDS with a 5q (q31-33) cytogenetic abnormality in isolation or with additional cytogenetic abnormalities, at a dose of 10 mg once daily or 10 mg once daily for 21 days every 28 days in an open-label, single-arm, multi-center study. (…)
217759, 03/24/2023	**Leniolisib (1)**	Pulmonary	PIK3CD	Clinical Studies	**14 CLINICAL STUDIES**<br>The efficacy of JOENJA was evaluated in the placebo-controlled portion of Study 2201 (NCT02435173), a 12-week blinded, randomized, placebo-controlled study in adult and pediatric patients 12 years of age and older with confirmed APDS-associated genetic PI3Kδ mutation with a documented variant in either PIK3CD or PIK3R1. Baseline patient demographics are shown in Table 2. (…)
217759, 03/24/2023	**Leniolisib (2)**	Pulmonary	PIK3R1	Clinical Studies	**14 CLINICAL STUDIES**

* Therapeutic areas do not necessarily reflect the CDER review division.
† Representative biomarkers are listed based on standard nomenclature as per the Human Genome Organization (HUGO) symbol and/or simplified descriptors using other common conventions. Listed biomarkers do not necessarily reflect the terminology used in labeling. The term "Nonspecific" is provided when labeling does not explicitly identify the specific biomarker(s) or when the biomarker is represented by a molecular phenotype or gene signature, and in some cases the biomarker was inferred based on the labeling language.
‡ Referenced figures and tables may be found in the labeling available at Drugs@FDA.
Blue text represents the most recent addtions and/or changes since last posted version.

Table of Pharmacogenomic Biomarkers in Drug Labeling
Last Updated: 06/2024

NDA/ANDA/BLA Number, Label Version Date	Drug	Therapeutic Area*	Biomarker†	Labeling Sections	Labeling Text‡
					The efficacy of JOENJA was evaluated in the placebo-controlled portion of Study 2201 (NCT02435173), a 12-week blinded, randomized, placebo-controlled study in adult and pediatric patients 12 years of age and older with confirmed APDS-associated genetic PI3Kδ mutation with a documented variant in either PIK3CD or PIK3R1. Baseline patient demographics are shown in Table 2. (…)
**206947, 12/19/2021**	Lenvatinib	Oncology	Microsatellite Instability, Mismatch Repair	Indications and Usage, Adverse Reactions, Clinical Studies	**1 INDICATIONS AND USAGE**<br>**1.4 Endometrial Carcinoma**<br>LENVIMA, in combination with pembrolizumab, is indicated for the treatment of patients with advanced endometrial carcinoma that is not microsatellite instability-high (MSI-H) or mismatch repair deficient (dMMR), who have disease progression following prior systemic therapy and are not candidates for curative surgery or radiation.<br>This indication is approved under accelerated approval based on tumor response rate and durability of response [see Clinical Studies (14.4)]. Continued approval for this indication may be contingent upon verification and description of clinical benefit in the confirmatory trial.<br><br>**6 ADVERSE REACTIONS**<br>**6.1 Clinical Trials Experience**<br>Endometrial Carcinoma<br>The safety of LENVIMA (20 mg orally once daily) in combination with pembrolizumab (200 mg intravenously every 3 weeks) was evaluated in Study 111, a single-arm, multicenter, open-label trial in 94 patients with endometrial carcinoma whose tumors had progressed following one line of systemic therapy and were not MSI-H or dMMR [see Clinical Studies (14.4)]. The median duration of study treatment was 7 months (range: 0.03 to 37.8 months). Pembrolizumab was continued for a maximum of 24 months; however, treatment with LENVIMA could be continued beyond 24 months.<br><br>**14 CLINICAL STUDIES**<br>**14.4 Endometrial Carcinoma (EC)**<br>(…) Among the 108 patients, 87% (n= 94) had tumors that were not MSI-H or dMMR; 10% (n=11) had tumors that were MSI-H or dMMR; and in 3% (n=3) the status was not known. Tumor MSI status was determined using a polymerase chain reaction (PCR) test. Tumor MMR status was determined using an immunohistochemistry (IHC) test. The baseline characteristics of the 94 patients with tumors that were not MSI-H or dMMR were: median age of 66 years with 62% 65 years or older; 86% White, 6% Black, 4% Asian, 3% other races; and ECOG PS of 0 (52%) or 1 (48%). All 94 of these patients received prior systemic therapy for endometrial carcinoma: 51% had one, 38% had two, and 11% had three or more prior systemic therapies. (See Table 18 and Figures 7 and 8)
**207988, 12/22/2015**	Lesinurad	Rheumatology	CYP2C9	Drug Interactions, Clinical Pharmacology	**7 DRUG INTERACTIONS**<br>**7.1 CYP2C9 Inhibitors, CYP2C9 Poor Metabolizers, and CYP2C9 inducers**<br>Lesinurad exposure is increased when ZURAMPIC is co-administered with inhibitors of CYP2C9, and in CYP2C9 poor metabolizers. ZURAMPIC should be used with caution in patients taking moderate inhibitors of CYP2C9 (eg, fluconazole, amiodarone), and in CYP2C9 poor metabolizers [see Clinical Pharmacology (12.3)]. (…)<br><br>**12 CLINICAL PHARMACOLOGY**<br>**12.3 Pharmacokinetics**<br>*Metabolism*<br>Patients who are CYP2C9 poor metabolizers are deficient in CYP2C9 enzyme activity. A cross-study pharmacogenomic analysis assessed the association between CYP2C9 polymorphism and lesinurad exposure in patients receiving single or multiple doses of lesinurad at 200 mg, 400 mg or 600 mg. At the 400 mg dose, ZURAMPIC exposure was approximately 1.8-fold higher in CYP2C9 poor metabolizers (i.e., subjects with CYP2C9 *2/*2 [N=1], and *3/*3 [N=1] genotype) compared to CYP2C9 extensive metabolizers (i.e., CYP2C9 *1/*1 [N=41] genotype). Use with caution in CYP2C9 poor metabolizers, and in patients taking moderate inhibitors of CYP2C9 [see Drug Interactions (7.1)].
**020726, 04/05/2018**	Letrozole	Oncology	ESR, PGR (Hormone Receptor)	Indications and Usage, Adverse Reactions, Clinical Studies	**1 INDICATIONS AND USAGE**<br>**1.1 Adjuvant Treatment of Early Breast Cancer**<br>Femara (letrozole) is indicated for the adjuvant treatment of postmenopausal women with hormone receptor positive early breast cancer.<br>**1.3 First and Second-Line Treatment of Advanced Breast Cancer**<br>Femara is indicated for first-line treatment of postmenopausal women with hormone receptor positive or unknown, locally advanced or metastatic breast cancer. Femara is also indicated for the treatment of advanced breast cancer in postmenopausal women with disease progression following antiestrogen therapy [see Clinical Studies (14.4, 14.5)].<br><br>**6 ADVERSE REACTIONS**<br>**6.1 Adjuvant Treatment of Early Breast Cancer**<br>(…) *Bone Study*<br>Results of a phase 3 safety trial in 262 postmenopausal women with resected receptor positive early breast cancer in the adjuvant setting comparing the effect on lumbar spine (L2-L4) bone mineral density (BMD) of adjuvant treatment with letrozole to that with tamoxifen showed at 24 months a median decrease in lumbar spine BMD of 4.1% in the letrozole arm compared to a median increase of 0.3% in the tamoxifen arm (difference = 4.4%) (P<0.0001). (…)<br>*Lipid Study*<br>In a phase 3 safety trial in 262 postmenopausal women with resected receptor positive early breast cancer at 24 months comparing the effects on lipid profiles of adjuvant letrozole to tamoxifen, 12% of patients on letrozole had at least one total cholesterol value of a higher CTCAE grade than at baseline compared with 4% of patients on tamoxifen. (…)

* Therapeutic areas do not necessarily reflect the CDER review division.
† Representative biomarkers are listed based on standard nomenclature as per the Human Genome Organization (HUGO) symbol and/or simplified descriptors using other common conventions. Listed biomarkers do not necessarily reflect the terminology used in labeling. The term “Nonspecific” is provided when labeling does not explicitly identify the specific biomarker(s) or when the biomarker is represented by a molecular phenotype or gene signature, and in some cases the biomarker was inferred based on the labeling language.
‡ Referenced figures and tables may be found in the labeling available at Drugs@FDA.
Blue text represents the most recent addtions and/or changes since last posted version.

Table of Pharmacogenomic Biomarkers in Drug Labeling

Last Updated: 06/2024

NDA/ANDA/BLA Number, Label Version Date	Drug	Therapeutic Area*	Biomarker†	Labeling Sections	Labeling Text‡
					**14 CLINICAL STUDIES**<br>**14.1 Updated Adjuvant Treatment of Early Breast Cancer**<br>In a multicenter study enrolling over 8,000 postmenopausal women with resected, receptor-positive early breast cancer, one of the following treatments was randomized in a double-blind manner (See Table 6) (…)<br>**14.2 Extended Adjuvant Treatment of Early Breast Cancer, Median Treatment Duration of 24 Months**<br>A double-blind, randomized, placebo-controlled trial of Femara was performed in over 5,100 postmenopausal women with receptor-positive or unknown primary breast cancer who were disease free after 5 years of adjuvant treatment with tamoxifen. (See Table 8) (…)<br>(…) Table 9 shows the study results. Disease-free survival was measured as the time from randomization to the earliest event of loco-regional or distant recurrence of the primary disease or development of contralateral breast cancer or death. DFS by hormone receptor status, nodal status and adjuvant chemotherapy were similar to the overall results. Data were premature for an analysis of survival. (See Table 9) (…)<br>**14.4 First-Line Treatment of Advanced Breast Cancer**<br>A randomized, double-blind, multinational trial compared Femara 2.5 mg with tamoxifen 20 mg in 916 postmenopausal patients with locally advanced (Stage IIIB or loco-regional recurrence not amenable to treatment with surgery or radiation) or metastatic breast cancer. Time to progression (TTP) was the primary endpoint of the trial. (See Table 11) (…)<br>(…) Table 13 shows results in the subgroup of women who had received prior antiestrogen adjuvant therapy, Table 14, results by disease site and Table 15, the results by receptor status. (…)<br>**14.5 Second-Line Treatment of Advanced Breast Cancer**<br>Femara was initially studied at doses of 0.1 mg to 5.0 mg daily in six non-comparative Phase I/II trials in 181 postmenopausal estrogen/progesterone receptor positive or unknown advanced breast cancer patients previously treated with at least antiestrogen therapy. (See Table 16) (…)
021402, 08/30/2022	Levothyroxine	Endocrinology	Nonspecific (Congenital Hypothyroidism)	Indications and Usage, Warnings and Precautions, Use in Specific Populations	**1 INDICATIONS AND USAGE**<br>**Hypothyroidism**<br>SYNTHROID is indicated in adult and pediatric patients, including neonates, as a replacement therapy in primary (thyroidal), secondary (pituitary), and tertiary (hypothalamic) congenital or acquired hypothyroidism.<br><br>**5 WARNINGS AND PRECAUTIONS**<br>**5.1 Serious Risks Related to Overtreatment or Undertreatment with SYNTHROID**<br>SYNTHROID has a narrow therapeutic index. Overtreatment or undertreatment with SYNTHROID may have negative effects on growth and development, cardiovascular function, bone metabolism, reproductive function, cognitive function, gastrointestinal function, and glucose and lipid metabolism in adult or pediatric patients.<br>In pediatric patients with congenital and acquired hypothyroidism, undertreatment may adversely affect cognitive development and linear growth, and overtreatment is associated with craniosynostosis and acceleration of bone age [see Use in Specific Populations (8.4)].<br>Titrate the dose of SYNTHROID carefully and monitor response to titration to avoid these effects [see Dosage and Administration (2.4)]. Consider the potential for food or drug interactions and adjust the administration or dosage of SYNTHROID as needed [see Dosage and Administration (2.1), Drug Interactions (7.1), and Clinical Pharmacology (12.3)].<br><br>**8 USE IN SPECIFIC POPULATIONS**<br>**8.4 Pediatric Use**<br>SYNTHROID is indicated in patients from birth to less than 17 years of age:<br>• As a replacement therapy in primary (thyroidal), secondary (pituitary), and tertiary (hypothalamic) congenital or acquired hypothyroidism.<br>• As an adjunct to surgery and radioiodine therapy in the management of thyrotropindependent well-differentiated thyroid cancer.<br>Rapid restoration of normal serum T4 concentrations is essential for preventing the adverse effects of congenital hypothyroidism on cognitive development as well as on overall physical growth and maturation. Therefore, initiate SYNTHROID therapy immediately upon diagnosis. Levothyroxine is generally continued for life in these patients [see Warnings and Precautions (5.1)].<br>Closely monitor infants during the first 2 weeks of SYNTHROID therapy for cardiac overload and arrhythmias.
**021451, 11/02/2018**	**Lidocaine and Prilocaine (1)**	Anesthesiology	Nonspecific (Congenital Methemoglobinemia)	Warnings and Precautions	**5 WARNINGS AND PRECAUTIONS**<br>**5.1 Methemoglobinemia**<br>Cases of methemoglobinemia have been reported in association with local anesthetic use. Although all patients are at risk for methemoglobinemia, patients with glucose-6-phosphate dehydrogenase deficiency, congenital or idiopathic methemoglobinemia, cardiac or pulmonary compromise, infants under 6 months of age, and concurrent exposure to oxidizing agents or their metabolites are more susceptible to developing clinical manifestations of the condition. If local anesthetics must be used in these patients, close monitoring for symptoms and signs of methemoglobinemia is recommended. (…)
**021451, 11/02/2018**	**Lidocaine and Prilocaine (2)**	Anesthesiology	G6PD	Warnings and Precautions, Clinical Pharmacology	**5 WARNINGS AND PRECAUTIONS**<br>**5.1 Methemoglobinemia**<br>Cases of methemoglobinemia have been reported in association with local anesthetic use. Although all patients are at risk for methemoglobinemia, patients with glucose-6-phosphate dehydrogenase deficiency, congenital or idiopathic methemoglobinemia, cardiac or pulmonary compromise, infants under 6 months of age, and concurrent exposure to oxidizing agents or their metabolites are more susceptible to developing clinical manifestations of the condition. If local anesthetics must be used in these patients, close monitoring for symptoms and signs of methemoglobinemia is recommended. (…)<br><br>**12 CLINICAL PHARMACOLOGY**<br>**12.3 Pharmacokinetics**

* Therapeutic areas do not necessarily reflect the CDER review division.
† Representative biomarkers are listed based on standard nomenclature as per the Human Genome Organization (HUGO) symbol and/or simplified descriptors using other common conventions. Listed biomarkers do not necessarily reflect the terminology used in labeling. The term “Nonspecific” is provided when labeling does not explicitly identify the specific biomarker(s) or when the biomarker is represented by a molecular phenotype or gene signature, and in some cases the biomarker was inferred based on the labeling language.
‡ Referenced figures and tables may be found in the labeling available at Drugs@FDA.
Blue text represents the most recent addtions and/or changes since last posted version.

NDA/ANDA/BLA Number, Label Version Date	Drug	Therapeutic Area*	Biomarker†	Labeling Sections	Labeling Text‡
					(…) Patients with glucose-6-phosphate dehydrogenase deficiencies and patients taking oxidizing drugs such as antimalarials and sulfonamides are more susceptible to drug-induced methemoglobinemia. [See Warnings and Precautions (5.1)] (…)
**021623, 11/02/2018**	**Lidocaine and Tetracaine (1)**	Anesthesiology	G6PD	Warnings and Precautions	**5 WARNINGS AND PRECAUTIONS**<br>**5.1 Methemoglobinemia**<br>Cases of methemoglobinemia have been reported in association with local anesthetic use. Although all patients are at risk for methemoglobinemia, patients with glucose-6-phosphate dehydrogenase deficiency, congenital or idiopathic methemoglobinemia, cardiac or pulmonary compromise, infants under 6 months of age, and concurrent exposure to oxidizing agents or their metabolites are more susceptible to developing clinical manifestations of the condition. If local anesthetics must be used in these patients, close monitoring for symptoms and signs of methemoglobinemia is recommended. (…)
**021623, 11/02/2018**	**Lidocaine and Tetracaine (2)**	Anesthesiology	Nonspecific (Congenital Methemoglobinemia)	Warnings and Precautions, Patient Counseling Information	**5 WARNINGS AND PRECAUTIONS**<br>**5.1 Methemoglobinemia**<br>Cases of methemoglobinemia have been reported in association with local anesthetic use. Although all patients are at risk for methemoglobinemia, patients with glucose-6-phosphate dehydrogenase deficiency, congenital or idiopathic methemoglobinemia, cardiac or pulmonary compromise, infants under 6 months of age, and concurrent exposure to oxidizing agents or their metabolites are more susceptible to developing clinical manifestations of the condition. If local anesthetics must be used in these patients, close monitoring for symptoms and signs of methemoglobinemia is recommended.<br>Signs of methemoglobinemia may occur immediately or may be delayed some hours after exposure, and are characterized by a cyanotic skin discoloration and/or abnormal coloration of the blood. Methemoglobin levels may continue to rise; therefore, immediate treatment is required to avert more serious central nervous system and cardiovascular adverse effects, including seizures, coma, arrhythmias, and death. Discontinue SYNERA and any other oxidizing agents. Depending on the severity of the signs and symptoms, patients may respond to supportive care, i.e., oxygen therapy, hydration. A more severe clinical presentation may require treatment with methylene blue, exchange transfusion, or hyperbaric oxygen.<br><br>**17 PATIENT COUNSELING INFORMATION**<br>• Advise patients not to use SYNERA if they have a history of methemoglobinemia.<br>• Inform patients that use of local anesthetics may cause methemoglobinemia, a serious condition that must be treated promptly. Advise patients or caregivers to stop use and seek immediate medical attention if they or someone in their care experience the following signs or symptoms: pale, gray, or blue colored skin (cyanosis); headache; rapid heart rate; shortness of breath; lightheadedness; or fatigue. (…)
**209229, 05/16/2018**	**Lofexidine**	Anesthesiology	CYP2D6	Use in Specific Populations	**8 USE IN SPECIFIC POPULATIONS**<br>**8.8 CYP2D6 Poor Metabolizers**<br>Although the pharmacokinetics of LUCEMYRA have not been systematically evaluated in patients who do not express the drug metabolizing enzyme CYP2D6, it is likely that the exposure to LUCEMYRA would be increased similarly to taking strong CYP2D6 inhibitors (approximately 28%). Monitor adverse events such as orthostatic hypotension and bradycardia in known CYP2D6 poor metabolizers. Approximately 8% of Caucasians and 3–8% of Black/African Americans cannot metabolize CYP2D6 substrates and are classified as poor metabolizers (PM) [see Clinical Pharmacology (12.3)].
**203858, 12/20/2019**	**Lomitapide**	Endocrinology	Nonspecific (Homozygous Familial Hypercholesterolemia)	Indications and Usage, Warnings and Precautions, Adverse Reactions, Clinical Studies	**1 INDICATIONS AND USAGE**<br>**2.1 Homozygous Familial Hypercholesterolemia**<br>JUXTAPID is indicated as an adjunct to a low-fat diet and other lipid-lowering treatments, including LDL apheresis where available, to reduce low-density lipoprotein cholesterol (LDLC), total cholesterol (TC), apolipoprotein B (apo B), and non-high-density lipoprotein cholesterol (non-HDL-C) in patients with homozygous familial hypercholesterolemia (HoFH).<br>Limitations of Use<br>• The safety and effectiveness of JUXTAPID have not been established in patients with hypercholesterolemia who do not have HoFH, including those with heterozygous familial hypercholesterolemia (HeFH).<br>• The effect of JUXTAPID on cardiovascular morbidity and mortality has not been determined.<br><br>**5 WARNINGS AND PRECAUTIONS**<br>**5.1 Risk of Hepatotoxicity**<br>JUXTAPID can cause elevations in transaminases and hepatic steatosis, as described below [see Warnings and Precautions (5.2)]. To what extent JUXTAPID-associated hepatic steatosis promotes the elevations in transaminases is unknown. Although cases of hepatic dysfunction (elevated transaminases with increase in bilirubin or INR) or hepatic failure have not been reported, there is concern that JUXTAPID could induce steatohepatitis, which can progress to cirrhosis over several years. The clinical studies supporting the safety and efficacy of JUXTAPID in HoFH would have been unlikely to detect this adverse outcome given their size and duration [see Clinical Studies (14)].<br><u>Elevation of Transaminases</u><br>Elevations in transaminases (alanine aminotransferase [ALT] and/or aspartate aminotransferase [AST]) are associated with JUXTAPID. In the clinical trial, 10 (34%) of the 29 patients with HoFH had at least one elevation in ALT or AST ≥3x ULN, and 4 (14%) of the patients had at least one elevation in ALT or AST ≥5x ULN. There were no concomitant or subsequent clinically meaningful elevations in bilirubin, INR, or alkaline phosphatase [see Adverse Reactions (6.1)].<br>During the 78-week HoFH clinical trial, no patients discontinued prematurely because of elevated transaminases. Among the 19 patients who subsequently enrolled in the HoFH extension study, one discontinued because of increased transaminases that persisted despite several dose reductions, and one temporarily discontinued because of markedly elevated transaminases (ALT 24x ULN, AST 13x ULN) that had several possible causes, including a drug-drug interaction between JUXTAPID and the strong CYP3A4 inhibitor clarithromycin [see

* Therapeutic areas do not necessarily reflect the CDER review division.
† Representative biomarkers are listed based on standard nomenclature as per the Human Genome Organization (HUGO) symbol and/or simplified descriptors using other common conventions. Listed biomarkers do not necessarily reflect the terminology used in labeling. The term “Nonspecific” is provided when labeling does not explicitly identify the specific biomarker(s) or when the biomarker is represented by a molecular phenotype or gene signature, and in some cases the biomarker was inferred based on the labeling language.
‡ Referenced figures and tables may be found in the labeling available at Drugs@FDA.
Blue text represents the most recent addtions and/or changes since last posted version.

NDA/ANDA/BLA Number, Label Version Date	Drug	Therapeutic Area*	Biomarker†	Labeling Sections	Labeling Text‡
					Drug Interactions (7.1)].<br>Hepatic Steatosis<br>JUXTAPID increases hepatic fat, with or without concomitant increases in transaminases. Hepatic steatosis is a risk factor for progressive liver disease, including steatohepatitis and cirrhosis. The long-term consequences of hepatic steatosis associated with JUXTAPID treatment are unknown. During the HoFH clinical trial, the median absolute increase in hepatic fat was 6% after both 26 weeks and 78 weeks of treatment, from 1% at baseline, measured by magnetic resonance spectroscopy (MRS) [see Adverse Reactions (6.1)]. Clinical data suggest that hepatic fat accumulation is reversible after stopping treatment with JUXTAPID, but whether histological sequelae remain is unknown, especially after long-term use; protocol liver biopsies were not performed in the HoFH clinical trial. (…)<br>**5.4 Reduced Absorption of Fat-Soluble Vitamins and Serum Fatty Acids**<br>Given its mechanism of action in the small intestine, JUXTAPID may reduce the absorption of fat-soluble nutrients. In the HoFH clinical trial, patients were provided daily dietary supplements of vitamin E, linoleic acid, alpha-linolenic acid (ALA), eicosapentaenoic acid (EPA), and docosahexaenoic acid (DHA). In this trial, the median levels of serum vitamin E, ALA, linoleic acid, EPA, DHA, and arachidonic acid decreased from baseline to Week 26 but remained above the lower limit of the reference range. Adverse clinical consequences of these reductions were not observed with JUXTAPID treatment of up to 78 weeks. Patients treated with JUXTAPID should take daily supplements that contain 400 international units vitamin E and at least 200 mg linoleic acid, 210 mg ALA, 110 mg EPA, and 80 mg DHA [see Dosage and Administration (2.1)]. Patients with chronic bowel or pancreatic diseases that predispose to malabsorption may be at increased risk for deficiencies in these nutrients with use of JUXTAPID.<br>5.5 Gastrointestinal Adverse Reactions<br>Gastrointestinal adverse reactions were reported by 27 (93%) of 29 patients in the HoFH clinical trial. Diarrhea occurred in 79% of patients, nausea in 65%, dyspepsia in 38%, and vomiting in 34%. Other reactions reported by at least 20% of patients include abdominal pain, abdominal discomfort, abdominal distension, constipation, and flatulence [see Adverse Reactions (6)]. Gastrointestinal adverse reactions of severe intensity were reported by 6 (21%) of 29 patients in the HoFH clinical trial, with the most common being diarrhea (4 patients, 14%); vomiting (3 patients, 10%); and abdominal pain, distension, and/or discomfort (2 patients, 7%). Gastrointestinal reactions contributed to the reasons for early discontinuation from the trial for 4 (14%) patients. (…)<br>**5.6 Concomitant Use of CYP3A4 Inhibitors**<br>CYP3A4 inhibitors increase the exposure of lomitapide, with strong inhibitors increasing exposure approximately 27-fold. Concomitant use of moderate or strong CYP3A4 inhibitors with JUXTAPID is contraindicated [see Drug Interactions (7.1)]. In the JUXTAPID clinical trials, one patient with HoFH developed markedly elevated transaminases (ALT 24x ULN, AST 13x ULN) within days of initiating the strong CYP3A4 inhibitor clarithromycin. If treatment with moderate or strong CYP3A4 inhibitors is unavoidable, JUXTAPID should be stopped during the course of treatment. (…)<br>**5.8 Risk of Supratherapeutic or Subtherapeutic Anticoagulation with Warfarin**<br>JUXTAPID increases the plasma concentrations of warfarin. Increases in the dose of JUXTAPID may lead to supratherapeutic anticoagulation, and decreases in the dose of JUXTAPID may lead to subtherapeutic anticoagulation. Difficulty controlling INR contributed to early discontinuation from the HoFH clinical trial for one of five patients taking concomitant warfarin. Patients taking warfarin should undergo regular monitoring of the INR, especially after any changes in JUXTAPID dosage. The dose of warfarin should be adjusted as clinically indicated [see Drug Interactions (7.3)].<br><br>**6 ADVERSE REACTIONS**<br>One single-arm, open-label, 78-week trial has been conducted in 29 patients with HoFH, 23 of whom completed at least one year of treatment. The initial dosage of JUXTAPID was 5 mg daily, with titration up to 60 mg daily during an 18-week period based on safety and tolerability. In this trial, the mean age was 30.7 years (range, 18 to 55 years), 16 (55%) patients were men, 25 (86%) patients were Caucasian, 2 (7%) were Asian, 1 (3%) was African American, and 1 (3%) was multi-racial [see Clinical Studies (14)].<br>Five (17%) of the 29 patients with HoFH that participated in the clinical trial discontinued treatment due to an adverse reaction. The adverse reactions that contributed to treatment discontinuations included diarrhea (2 patients; 7%) and abdominal pain, nausea, gastroenteritis, weight loss, headache, and difficulty controlling INR on warfarin (1 patient each; 3%).<br>The most common adverse reactions were gastrointestinal, reported by 27 (93%) of 29 patients. Adverse reactions reported by ≥8 (28%) patients in the HoFH clinical trial included diarrhea, nausea, vomiting, dyspepsia, and abdominal pain. Other common adverse reactions, reported by 5 to 7 (17-24%) patients, included weight loss, abdominal discomfort, abdominal distension, constipation, flatulence, increased ALT, chest pain, influenza, nasopharyngitis, and fatigue.<br>The adverse reactions reported in at least 10% of patients during the HoFH clinical trial are presented in Table 4.<br>Adverse reactions of severe intensity were reported by 8 (28%) of 29 patients, with the most common being diarrhea (4 patients, 14%), vomiting (3 patients, 10%), increased ALT or hepatotoxicity (3 patients, 10%), and abdominal pain, distension, and/or discomfort (2 patients, 7%).<br>Transaminase Elevations<br>During the HoFH clinical trial, 10 (34%) of 29 patients had at least one elevation in ALT and/or AST ≥3x ULN (see Table 5). No clinically meaningful elevations in total bilirubin or alkaline phosphatase were observed. Transaminases typically fell within one to four weeks of reducing the dose or withholding JUXTAPID. Among the 19 patients who enrolled in an extension study following the HoFH clinical trial, one discontinued because of increased transaminases that persisted despite several dose reductions, and one temporarily discontinued because of markedly elevated transaminases (ALT 24x ULN, AST 13x ULN) that had several possible causes, including a drug-drug interaction between JUXTAPID and the strong CYP3A4 inhibitor clarithromycin [see Drug Interactions (7.1)].<br>Hepatic Steatosis<br>Hepatic fat was prospectively measured using magnetic resonance spectroscopy (MRS) in all eligible patients during the HoFH clinical trial. After 26 weeks, the median absolute increase in hepatic fat from baseline was 6%, and the mean absolute increase was 8% (range, 0% to 30%). After 78 weeks, the median absolute increase in hepatic fat from baseline was 6%, and the mean absolute increase was 7% (range, 0% to 18%). Among the 23 patients with evaluable data, on at least one occasion during the trial, 18 (78%) exhibited an increase in hepatic fat >5% and 3 (13%) exhibited an increase >20%. Data from individuals who had repeat measurements after stopping JUXTAPID show that hepatic fat accumulation is reversible, but whether histological sequelae remain is unknown.

Table of Pharmacogenomic Biomarkers in Drug Labeling
Last Updated: 06/2024

NDA/ANDA/BLA Number, Label Version Date	Drug	Therapeutic Area*	Biomarker†	Labeling Sections	Labeling Text‡
					**14 CLINICAL STUDIES**<br>The safety and effectiveness of JUXTAPID as an adjunct to a low-fat diet and other lipidlowering treatments, including LDL apheresis where available, were evaluated in a multinational, single-arm, open-label, 78-week trial involving 29 adults with HoFH. A diagnosis of HoFH was defined by the presence of at least one of the following clinical criteria: (1) documented functional mutation(s) in both LDL receptor alleles or alleles known to affect LDL receptor functionality, or (2) skin fibroblast LDL receptor activity 500 mg/dL and TG 250 mg/dL. (…)
**213969, 11/20/2020**	Lonafarnib (1)	Inborn Errors of Metabolism	LMNA	Indications and Usage, Adverse Reactions, Use in Specific Populations, Clinical Studies	**1 INDICATIONS AND USAGE**<br>ZOKINVY is indicated in patients 12 months of age and older with a body surface area (BSA) of 0.39 m2 and above:<br>• To reduce the risk of mortality in Hutchinson-Gilford Progeria Syndrome (HGPS)<br>• For the treatment of processing-deficient Progeroid Laminopathies with either:<br>o Heterozygous LMNA mutation with progerin-like protein accumulation<br>o Homozygous or compound heterozygous ZMPSTE24 mutations<br>Limitations of Use<br>ZOKINVY is not indicated for other Progeroid Syndromes or processing-proficient Progeroid Laminopathies. Based upon its mechanism of action, ZOKINVY would not be expected to be effective in these populations.<br><br>**6 ADVERSE REACTIONS**<br>**6.1 Clinical Trial Experience**<br>The safety profile of ZOKINVY is based on 128 patient-years of treatment exposure (62 patients with HGPS and 1 patient with processing-deficient Progeroid Laminopathy with LMNA heterozygous mutation) and pooled results from two Phase 2 open-label, single-arm trials (n=63: 28 patients from Study 1 and 35 treatment naïve patients from Study 2). (…)<br>In these two studies, a total of 63 patients received ZOKINVY for a median duration of 2.2 years, with approximately 1.9 years at the recommended dose of 150 mg/m2 twice daily. The population was 2 to 17 years old, with a similar proportion of males (33 [52%] patients) and females (30 [48%] patients). Most patients had classic HGPS (60 [95%] patients) compared to non-classic HGPS (2 [3%] patients) and 1 (2%) patient had Progeroid Laminopathy with LMNA heterozygous mutation. (…)<br><br>**8 USE IN SPECIFIC POPULATIONS**<br>**8.4 Pediatric Use**<br>The safety and effectiveness of ZOKINVY for the treatment of HGPS and processing-deficient Progeroid Laminopathies (with either heterozygous LMNA mutation with progerin-like protein accumulation or homozygous or compound heterozygous ZMPSTE24 mutations) have been established in pediatric patients 12 months of age and older. Use of ZOKINVY for these indications is supported by adequate and well controlled studies in pediatric patients 2 years of age and older [see Clinical Studies (14)]. The safety and effectiveness of ZOKINVY in pediatric patients less than 12 months of age have not been established.<br>**8.6 Adult Use**<br>The safety and effectiveness of ZOKINVY for the treatment of HGPS and processing-deficient Progeroid Laminopathies (with either heterozygous LMNA mutation with progerin-like protein accumulation or homozygous or compound heterozygous ZMPSTE24 mutations) have been established in adults. Use of ZOKINVY in adults for these indications is based on adequate and well controlled studies in pediatric patients 2 years of age and older [see Clinical Studies (14)].<br><br>**14 CLINICAL STUDIES**<br>The efficacy of ZOKINVY is based on results from the Observational Cohort Survival Study, which retrospectively compared survival data from two Phase 2 studies in patients with HGPS to those from a natural history cohort. Study 1 (NCT00425607) was a Phase 2 open-label, single-arm trial that evaluated the efficacy of ZOKINVY in 28 patients (26 with classic HGPS, one with non-classic HGPS, and one with processing-deficient Progeroid Laminopathy with LMNA heterozygous mutation with progerinlike protein accumulation). (…)
**213969, 11/20/2020**	Lonafarnib (2)	Inborn Errors of Metabolism	ZMPSTE24	Indications and Usage, Use in Specific Populations	**1 INDICATIONS AND USAGE**<br>ZOKINVY is indicated in patients 12 months of age and older with a body surface area (BSA) of 0.39 m2 and above:<br>• To reduce the risk of mortality in Hutchinson-Gilford Progeria Syndrome (HGPS)<br>• For the treatment of processing-deficient Progeroid Laminopathies with either:<br>o Heterozygous LMNA mutation with progerin-like protein accumulation<br>o Homozygous or compound heterozygous ZMPSTE24 mutations<br>Limitations of Use<br>ZOKINVY is not indicated for other Progeroid Syndromes or processing-proficient Progeroid Laminopathies. Based upon its mechanism of action, ZOKINVY would not be expected to be effective in these populations.<br><br>**8 USE IN SPECIFIC POPULATIONS**<br>**8.4 Pediatric Use**<br>The safety and effectiveness of ZOKINVY for the treatment of HGPS and processing-deficient Progeroid Laminopathies (with either heterozygous LMNA mutation with progerin-like protein accumulation or homozygous or compound heterozygous ZMPSTE24 mutations) have been established in pediatric patients 12 months of age and older. Use of ZOKINVY for these indications is supported by adequate and well controlled studies in pediatric patients 2 years of age and older [see Clinical Studies (14)]. The safety and effectiveness of ZOKINVY in pediatric patients less than 12 months of age have not been established.<br>**8.6 Adult Use**

* Therapeutic areas do not necessarily reflect the CDER review division.
† Representative biomarkers are listed based on standard nomenclature as per the Human Genome Organization (HUGO) symbol and/or simplified descriptors using other common conventions. Listed biomarkers do not necessarily reflect the terminology used in labeling. The term “Nonspecific” is provided when labeling does not explicitly identify the specific biomarker(s) or when the biomarker is represented by a molecular phenotype or gene signature, and in some cases the biomarker was inferred based on the labeling language.
‡ Referenced figures and tables may be found in the labeling available at Drugs@FDA.
Blue text represents the most recent addtions and/or changes since last posted version.

NDA/ANDA/BLA Number, Label Version Date	Drug	Therapeutic Area*	Biomarker†	Labeling Sections	Labeling Text‡
					The safety and effectiveness of ZOKINVY for the treatment of HGPS and processing-deficient Progeroid Laminopathies (with either heterozygous LMNA mutation with progerin-like protein accumulation or homozygous or compound heterozygous ZMPSTE24 mutations) have been established in adults. Use of ZOKINVY in adults for these indications is based on adequate and well controlled studies in pediatric patients 2 years of age and older [see Clinical Studies (14)].
**210868, 03/03/2021**	Lorlatinib (1)	Oncology	ALK	Indications and Usage, Dosage and Administration, Adverse Reactions, Clinical Studies	**1 INDICATIONS AND USAGE**<br>LORBRENA® is indicated for the treatment of adult patients with metastatic non-small cell lung cancer (NSCLC) whose tumors are anaplastic lymphoma kinase (ALK)-positive as detected by an FDA-approved test.<br><br>**2 DOSAGE AND ADMINISTRATION**<br>**2.1 Patient Selection**<br>Select patients for the treatment of metastatic NSCLC with LORBRENA based on the presence of ALK positivity in tumor specimens [see Indications and Usage (1) and Clinical Studies (14)]. Information on FDA-approved tests for the detection of ALK rearrangements in NSCLC is available at http://www.fda.gov/CompanionDiagnostics.<br><br>**6 ADVERSE REACTIONS**<br>**6.1 Clinical Trials Experience**<br>Previously Untreated ALK-Positive Metastatic NSCLC (CROWN Study)<br>The safety of LORBRENA was evaluated in 149 patients with ALK-positive NSCLC in a randomized, openlabel, active-controlled trial for the treatment of patients with ALK-positive, locally advanced or metastatic, NSCLC who had not received previous systemic treatment for advanced disease [see Clinical Studies (14)]. The median duration of exposure to LORBRENA was 16.7 months (4 days to 34.3 months) and 76% received LORBRENA for at least 12 months. (…)<br>Previously Treated ALK-Positive Metastatic NSCLC<br>The data described below reflect exposure to LORBRENA in 295 patients with ALK-positive or ROS1-positive metastatic NSCLC who received LORBRENA 100 mg orally once daily in Study B7461001, a multi-cohort, non-comparative trial [see Clinical Studies (14)]. The median duration of exposure to LORBRENA was 12.5 months (1 day to 35 months) and 52% received LORBRENA for ≥12 months. Patient characteristics were: median age of 53 years (19 to 85 years), age ≥65 years (18%), female (58%), White (49%), Asian (37%), and ECOG performance status 0 or 1 (96%). (…)<br><br>**14 CLINICAL STUDIES**<br>Previously Untreated ALK-Positive Metastatic NSCLC (CROWN Study)<br>The efficacy of LORBRENA for the treatment of patients with ALK-positive NSCLC who had not received prior systemic therapy for metastatic disease was established in an open-label, randomized, active-controlled, multicenter study (Study B7461006; NCT03052608). Patients were required to have an ECOG performance status of 0-2 and ALK-positive NSCLC as identified by the VENTANA ALK (D5F3) CDx assay. Neurologically stable patients with treated or untreated asymptomatic CNS metastases, including leptomeningeal metastases, were eligible. (…)<br>ALK-Positive Metastatic NSCLC Previously Treated with an ALK Kinase Inhibitor<br>The efficacy of LORBRENA was demonstrated in a subgroup of patients with ALK-positive metastatic NSCLC previously treated with one or more ALK kinase inhibitors who were enrolled in a non-randomized, doseranging and activity-estimating, multi-cohort, multicenter study (Study B7461001; NCT01970865). Patients included in this subgroup were required to have metastatic disease with at least 1 measurable target lesion according to RECIST v1.1, ECOG performance status of 0 to 2, and documented ALK rearrangement in tumor tissue as determined by fluorescence in situ hybridization (FISH) assay or by Immunohistochemistry (IHC), and received LORBRENA 100 mg orally once daily. Patients with asymptomatic CNS metastases, including patients with stable or decreasing steroid use within 2 weeks prior to study entry, were eligible. Patients with severe, acute, or chronic psychiatric conditions including suicidal ideation or behavior were excluded. In addition, for patients with ALK-positive metastatic NSCLC, the extent and type of prior treatment was specified for each individual cohort (see Table 8).
**210868, 03/03/2021**	Lorlatinib (2)	Oncology	ROS1	Adverse Reactions	**6 ADVERSE REACTIONS**<br>**6.1 Clinical Trials Experience**<br>Previously Treated ALK-Positive Metastatic NSCLC<br>The data described below reflect exposure to LORBRENA in 295 patients with ALK-positive or ROS1-positive metastatic NSCLC who received LORBRENA 100 mg orally once daily in Study B7461001, a multi-cohort, non-comparative trial [see Clinical Studies (14)]. The median duration of exposure to LORBRENA was 12.5 months (1 day to 35 months) and 52% received LORBRENA for ≥12 months. Patient characteristics were: median age of 53 years (19 to 85 years), age ≥65 years (18%), female (58%), White (49%), Asian (37%), and ECOG performance status 0 or 1 (96%). (…)
**214103, 11/23/2020**	Lumasiran	Urology	AGXT	Indications and Usage, Adverse Reactions, Use in Specific Populations, Clinical Pharmacology, Clinical Studies	**1 INDICATIONS AND USAGE**<br>OXLUMO is indicated for the treatment of primary hyperoxaluria type 1 (PH1) to lower urinary oxalate levels in pediatric and adult patients [see Clinical Pharmacology (12.1), Clinical Studies (14.1, 14.2)].<br><br>**6 ADVERSE REACTIONS**<br>**6.1 Clinical Trials Experience**<br>The data reflect placebo-controlled and open-label clinical studies in 77 patients with PH1 (including 56 pediatric patients). Patients ranged in age from 4 months to 61 years at first dose. The median duration of exposure was 9.1 months (range 1.9 to 21.7 months). Overall, 58 patients were treated for at least 6 months, and 18 patients for at least 12 months.

* Therapeutic areas do not necessarily reflect the CDER review division.
† Representative biomarkers are listed based on standard nomenclature as per the Human Genome Organization (HUGO) symbol and/or simplified descriptors using other common conventions. Listed biomarkers do not necessarily reflect the terminology used in labeling. The term “Nonspecific” is provided when labeling does not explicitly identify the specific biomarker(s) or when the biomarker is represented by a molecular phenotype or gene signature, and in some cases the biomarker was inferred based on the labeling language.
‡ Referenced figures and tables may be found in the labeling available at Drugs@FDA.
Blue text represents the most recent addtions and/or changes since last posted version.

NDA/ANDA/BLA Number, Label Version Date	Drug	Therapeutic Area*	Biomarker†	Labeling Sections	Labeling Text‡
					In the randomized, placebo-controlled, double-blind study ILLUMINATE-A in pediatric and adult patients with PH1 aged 6 to 61 years, 26 patients received OXLUMO and 13 patients received placebo. Of these, 25 patients received ≥5 months of treatment. (…)<br>In the single-arm study (ILLUMINATE-B) in patients with PH1 who are <6 years of age, the safety profile observed was similar to that seen in ILLUMINATE-A [see Clinical Studies (14)].<br><br>**8 USE IN SPECIFIC POPULATIONS**<br>**8.4 Pediatric Use**<br>The safety and effectiveness of OXLUMO have been established in pediatric patients aged birth and older. Use of OXLUMO in these age groups is supported by evidence from an adequate and well controlled study of OXLUMO in children 6 years or older and adults with PH1 (ILLUMINATE-A), and a single-arm clinical study in children less than 6 years of age with PH1 (ILLUMINATE-B) [see Adverse Reactions (6.1), Clinical Studies (14)].<br><br>**12 CLINICAL PHARMACOLOGY**<br>**12.2 Pharmacodynamics**<br>The pharmacodynamic effects of OXLUMO have been evaluated in adult and pediatric patients with PH1 across a range of doses and dosing frequency. Dose-dependent reductions in urinary oxalate levels were observed, resulting in the selection of the recommended body weight-based loading and maintenance dosing regimens. With the recommended dosing regimens, onset of effect was observed within two weeks after the first dose and maximal reductions in urinary oxalate were observed by Month 2 and persisted with continued use of OXLUMO maintenance dosage [see Figures 1 and 2 in Clinical Studies (14.1, 14.2)].<br>**12.3 Pharmacokinetics**<br>The pharmacokinetic (PK) properties of OXLUMO were evaluated following administration of single and multiple dosages in patients with PH1 as summarized in Table 3. (See Table 3)<br><br>**14 CLINICAL STUDIES**<br>**14.1 ILLUMINATE-A**<br>ILLUMINATE-A was a randomized, double-blind trial comparing lumasiran and placebo in 39 patients 6 years of age and older with PH1 and an eGFR ≥30 mL/min/1.73 m2 (ILLUMINATE-A; NCT03681184). Patients received 3 loading doses of 3 mg/kg OXLUMO (N=26) or placebo (N=13) administered once monthly, followed by quarterly maintenance doses of 3 mg/kg OXLUMO or placebo [see Dosage and Administration (2.1)]. (…)<br>**14.2 ILLUMINATE-B**<br>ILLUMINATE-B was a single-arm study in 18 patients >45 mL/min/1.73 m2 for patients ≥12 months of age or a normal serum creatinine for patients <12 months of age (ILLUMINATE-B; NCT03905694). Efficacy analyses included the first 16 patients who received 6 months of treatment with OXLUMO. Dosing was based on body weight [see Dosage and Administration (2.1)]. (…)
761136, 11/08/2019	Luspatercept–aamt	Hematology	HBB	Clinical Studies	**14 CLINICAL STUDIES**<br>**14.1 Beta Thalassemia**<br>(…) The BELIEVE trial excluded patients with hemoglobin S/β-thalassemia or alpha-thalassemia or who had major organ damage (liver disease, heart disease, lung disease, renal insufficiency). Patients with recent deep vein thrombosis or stroke or recent use of ESA, immunosuppressant, or hydroxyurea therapy were also excluded. The median age was 30 years (range: 18-66). The trial was comprised of patients who were 42% male, 54.2% white, 34.8% Asian, and 0.3% Black or African American. The percent of patients reporting their race as "other" was 7.7%, and race was not collected or reported for 3% of patients. Table 4 summarizes the baseline disease-related characteristics in the BELIEVE study. (See Table 4) (…)
210923, 07/31/2018	Lusutrombopag (1)	Hematology	F2 (Prothrombin)	Warnings and Precautions	**5 WARNINGS AND PRECAUTIONS**<br>**5.1 Thrombotic/Thromboembolic Complications**<br>(…) Consider the potential increased thrombotic risk when administering MULPLETA to patients with known risk factors for thromboembolism, including genetic pro-thrombotic conditions (Factor V Leiden, Prothrombin 20210A, Antithrombin deficiency, or Protein C or S deficiency). In patients with ongoing or prior thrombosis or absence of hepatopetal blood flow, MULPLETA should only be used if the potential benefit to the patient justifies the potential risk. MULPLETA should not be administered to patients with chronic liver disease in an attempt to normalize platelet counts.
210923, 07/31/2018	Lusutrombopag (2)	Hematology	F5 (Factor V Leiden)	Warnings and Precautions	**5 WARNINGS AND PRECAUTIONS**<br>**5.1 Thrombotic/Thromboembolic Complications**<br>(…) Consider the potential increased thrombotic risk when administering MULPLETA to patients with known risk factors for thromboembolism, including genetic pro-thrombotic conditions (Factor V Leiden, Prothrombin 20210A, Antithrombin deficiency, or Protein C or S deficiency). In patients with ongoing or prior thrombosis or absence of hepatopetal blood flow, MULPLETA should only be used if the potential benefit to the patient justifies the potential risk. MULPLETA should not be administered to patients with chronic liver disease in an attempt to normalize platelet counts.
210923, 07/31/2018	Lusutrombopag (3)	Hematology	PROC	Warnings and Precautions	**5 WARNINGS AND PRECAUTIONS**<br>**5.1 Thrombotic/Thromboembolic Complications**<br>(…) Consider the potential increased thrombotic risk when administering MULPLETA to patients with known risk factors for thromboembolism, including genetic pro-thrombotic conditions (Factor V Leiden, Prothrombin 20210A, Antithrombin deficiency, or Protein C or S deficiency). In patients with ongoing or prior thrombosis or absence of hepatopetal blood flow, MULPLETA should only be used if the potential benefit to the patient justifies the potential risk. MULPLETA should not be administered to patients with chronic liver disease in an attempt to normalize platelet counts.
210923, 07/31/2018	Lusutrombopag (4)	Hematology	PROS1	Warnings and Precautions	**5 WARNINGS AND PRECAUTIONS**<br>**5.1 Thrombotic/Thromboembolic Complications**

NDA/ANDA/BLA Number, Label Version Date	Drug	Therapeutic Area*	Biomarker†	Labeling Sections	Labeling Text‡
					(…) Consider the potential increased thrombotic risk when administering MULPLETA to patients with known risk factors for thromboembolism, including genetic pro-thrombotic conditions (Factor V Leiden, Prothrombin 20210A, Antithrombin deficiency, or Protein C or S deficiency). In patients with ongoing or prior thrombosis or absence of hepatopetal blood flow, MULPLETA should only be used if the potential benefit to the patient justifies the potential risk. MULPLETA should not be administered to patients with chronic liver disease in an attempt to normalize platelet counts.
**210923, 07/31/2018**	Lusutrombopag (5)	Hematology	SERPINC1 (Antithrombin III)	Warnings and Precautions	**5 WARNINGS AND PRECAUTIONS**<br>**5.1 Thrombotic/Thromboembolic Complications**<br>(…) Consider the potential increased thrombotic risk when administering MULPLETA to patients with known risk factors for thromboembolism, including genetic pro-thrombotic conditions (Factor V Leiden, Prothrombin 20210A, Antithrombin deficiency, or Protein C or S deficiency). In patients with ongoing or prior thrombosis or absence of hepatopetal blood flow, MULPLETA should only be used if the potential benefit to the patient justifies the potential risk. MULPLETA should not be administered to patients with chronic liver disease in an attempt to normalize platelet counts.
**208700, 04/23/2024**	**Lutetium Lu 177 Dotatate**	Oncology	SSTR	Indications and Usage, Adverse Reactions, Use in Specific Populations, Clinical Pharmacology, Clinical Studies	**1 INDICATIONS AND USAGE**<br>LUTATHERA is indicated for the treatment of somatostatin receptor-positive gastroenteropancreatic neuroendocrine tumors (GEP-NETs), including foregut, midgut, and hindgut neuroendocrine tumors in adults.<br><br>**6 ADVERSE REACTIONS**<br>**6.1 Clinical Trials Experience**<br>The data in Warnings and Precautions reflect exposure to LUTATHERA in 111 patients with advanced, progressive midgut neuroendocrine tumors (NETTER-1). Safety data in Warnings and Precautions were also obtained in an additional 22 patients in a non-randomized pharmacokinetic substudy of NETTER-1 and in a subset of patients (811 of 1214) with advanced somatostatin receptor-positive tumors enrolled in ERASMUS [see Warnings and Precautions (5)]. (…)<br>NETTER-1<br>The safety data of LUTATHERA with octreotide was evaluated in NETTER-1 [see Clinical Studies (14.1)] Patients with progressive, somatostatin receptor-positive midgut carcinoid tumors to receive LUTATHERA 7.4 GBq (200 mCi) administered every 8 to 16 weeks concurrently with the recommended amino acid solution and with long-acting octreotide (30 mg administered by intramuscular injection within 24 hours of each LUTATHERA dose) (n = 111), or high-dose octreotide (defined as long-acting octreotide 60 mg by intramuscular injection every 4 weeks) (n = 112) [see Clinical Studies (14.1)]. (…)<br>ERASMUS<br>Safety data are available from 1214 patients in ERASMUS, an international, single-institution, single-arm, open-label trial of patients with somatostatin receptor-positive tumors (neuroendocrine and other primaries). Patients received LUTATHERA 7.4 GBq (200 mCi) administered every 6 to 13 weeks with or without octreotide. (…)<br><br>**8 USE IN SPECIFIC POPULATIONS**<br>**8.4 Pediatric Use**<br>Somatostatin Receptor-Positive Gastroenteropancreatic Neuroendocrine Tumors<br>The safety and effectiveness of LUTATHERA have been established in pediatric patients 12 years and older with somatostatin receptor-positive gastroenteropancreatic neuroendocrines (GEP-NETs). Use of LUTATHERA for this indication is supported by evidence from an adequate and well-controlled study of LUTATHERA in adults with additional safety, pharmacokinetic, and dosimetry data in pediatric patients aged 12 years and older with somatostatin receptor-positive tumors, including 4 pediatric patients with GEP-NETs [see Adverse Reactions (6.1), Clinical Pharmacology (12.3), Clinical Studies (14)].<br>The risks of radiation exposure associated with LUTATHERA are greater in pediatric patients than in adult patients due to longer life expectancy. Continued follow-up is recommended for evaluation of long-term effects.<br>There was no clinically relevant difference in lutetium Lu 177 dotatate exposure in pediatric patients aged 13 to 16 years versus adult patients [see Clinical Pharmacology (12.3)].<br>The pharmacokinetic profile and safety of LUTATHERA in pediatric patients 12 years and older with baseline renal impairment have not been studied.<br>The safety and effectiveness of LUTATHERA have not been established in pediatric patients younger than 12 years old with somatostatin receptor-positive GEP-NETs.<br><br>**12 CLINICAL PHARMACOLOG**<br>**12.2 Pharmacodynamics**<br>Lutetium Lu 177 exposure-response relationships and the time course of pharmacodynamics response are unknown.<br>Cardiac Electrophysiology<br>The ability of LUTATHERA to prolong the QTc interval at the therapeutic dose was assessed in an open label study in 20 patients with somatostatin receptor-positive midgut carcinoid tumors. No large changes in the mean QTc interval (i.e., >20 ms) were detected.<br>**12.3 Pharmacokinetics**<br>The pharmacokinetics (PK) of lutetium Lu 177 dotatate have been characterized in patients with progressive, somatostatin receptor-positive neuroendocrine tumors. The mean blood exposure (AUC) of lutetium Lu 177 dotatate at the recommended dose is 41 ng.h/mL [coefficient of variation (CV) 36 %]. The mean maximum blood concentration (Cmax) for lutetium Lu 177 dotatate is 10 ng/mL (CV 50%), which generally occurred at the end of the LUTATHERA infusion.<br><br>**14 CLINICAL STUDIES**<br>**14.1 Progressive, Well-differentiated Advanced or Metastatic Somatostatin ReceptorPositive Midgut Carcinoid Tumors**<br>The efficacy of LUTATHERA in patients with progressive, well-differentiated, locally advanced/inoperable or metastatic somatostatin receptor-positive midgut carcinoid tumors was established in NETTER-1 (NCT01578239), a randomized, multicenter, open-label, activecontrolled trial. Key eligibility criteria included Ki67

Table of Pharmacogenomic Biomarkers in Drug Labeling
Last Updated: 06/2024

NDA/ANDA/BLA Number, Label Version Date	Drug	Therapeutic Area*	Biomarker†	Labeling Sections	Labeling Text‡
					index ≤ 20%, Karnofsky performance status ≥ 60, confirmed presence of somatostatin receptors on all lesions (OctreoScan uptake ≥ normal liver), creatinine clearance ≥ 50 mL/min, no prior treatment with peptide receptor radionuclide therapy (PRRT), and no prior external radiation therapy to more than 25% of the bone marrow. (…)<br>**14.2 Somatostatin Receptor-Positive Gastroenteropancreatic Neuroendocrine Tumors**<br>The efficacy of LUTATHERA in patients with foregut, midgut, and hindgut gastroenteropancreatic neuroendocrine tumors (GEP-NETs) was assessed in 360 patients in the ERASMUS study. In ERASMUS, LUTATHERA was initially provided as expanded access under a general peptide receptor radionuclide therapy protocol at a single site in the Netherlands. (…)
215833, 03/23/2022	Lutetium Lu 177 Vipivotide Tetraxetan	Oncology	FOLH1 (PSMA)	Indications and Usage, Dosage and Administration, Adverse Reactions, Clinical Studies	**1 INDICATIONS AND USAGE**<br>PLUVICTO is indicated for the treatment of adult patients with prostate-specific membrane antigen (PSMA)-positive metastatic castration-resistant prostate cancer (mCRPC) who have been treated with androgen receptor (AR) pathway inhibition and taxane-based chemotherapy.<br><br>**2 DOSAGE AND ADMINISTRATION**<br>**2.2 Patient Selection**<br>Select patients with previously treated mCRPC for treatment with PLUVICTO using LOCAMETZ or another approved PSMA-11 imaging agent based on PSMA expression in tumors. Additional selection criteria were used in the VISION study [see Clinical Studies (14)].<br>Refer to the prescribing information for the PSMA imaging agent.<br><br>**6 ADVERSE REACTIONS**<br>**6.1 Clinical Trials Experience**<br>The safety of PLUVICTO was evaluated in the VISION study in patients with progressive, PSMA-positive mCRPC [see Clinical Studies (14)]. (See Tables 3 and 4)<br><br>**14 CLINICAL STUDIES**<br>The efficacy of PLUVICTO was evaluated in VISION (NCT03511664), a randomized (2:1), multicenter, open-label trial that evaluated PLUVICTO plus BSoC (N = 551) or BSoC alone (N = 280) in men with progressive, PSMA-positive mCRPC.
019832, 06/05/1998	Mafenide	Infectious Diseases	G6PD	Warnings, Adverse Reactions	**WARNINGS**<br>Fatal hemolytic anemia with disseminated intravascular coagulation, presumably related to a glucose-6-phosphate dehydrogenase deficiency, has been reported following therapy with mafenide acetate.<br><br>**ADVERSE REACTIONS**<br>(…) Fatal hemolytic anemia with disseminated intravascular coagulation, presumably related to a glucose-6-phosphate dehydrogenase deficiency, has been reported following therapy with mafenide acetate. (…)
214662, 03/13/2024	Maralixibat (1)	Gastroenterology	JAG1	Clinical Studies	**14 CLINICAL STUDIES**<br>The efficacy of LIVMARLI was assessed in Trial 1 (NCT02160782), which consisted of an 18-week open-label treatment period; a 4-week randomized, double-blind, placebo-controlled drug-withdrawal period; a subsequent 26-week open-label treatment period; and a long-term open-label extension period.<br>Thirty-one pediatric ALGS patients with cholestasis and pruritus were enrolled, with 90.3% of patients receiving at least one medication to treat pruritus at study entry. All patients had JAGGED1 mutation. (…)
214662, 03/13/2024	Maralixibat (2)	Gastroenterology	ABCB4	Clinical Pharmacology, Clinical Studies	**12 CLINICAL PHARMACOLOGY**<br>**12.5. Pharmacogenomics**<br>PFIC is a heterogenous disease caused by homozygous or compound heterozygous variants, with different PFIC subtypes occurring in the general population. PFIC1 is caused by variants in the aminophospholipid flippase (ATP8B1) gene, which encodes the Familial Intrahepatic Cholestasis 1 (FIC1) protein, while PFIC2 results from variants in the ABCB11 gene, which encodes the Bile Salt Export Pump (BSEP) protein. PFIC2 is further categorized into BSEP subgroups based on specific variants. The BSEP-1 subgroup includes patients with at least one p.D482G (c.1445A>G) or p.E297G (c.890A>G) variant, BSEP-2 includes patients with at least one missense variant other than p.D482G or p.E297G (non BSEP-1), and BSEP-3 includes patients with variants that are predicted to encode a non-functional protein. PFIC3 is caused by variants in the ABCB4 gene, which encodes multidrug resistance protein 3 (MDR3). PFIC4 is caused by variants in the tight junction protein 2 gene (TJP2), which encodes TJP2. PFIC6 is caused by variants in myosin 5B (MYO5B), which encodes MYO5B. Patients can be clinically diagnosed with PFIC without a known pathogenic variant.<br>PFIC2 is the most common subtype accounting for 37-90% of patients with PFIC. The prevalence of BSEP-1, BSEP-2, and BSEP-3 subgroups are approximately 27.3%, 51.5%, and 21.2%, respectively, based on data from a global consortium characterizing the natural history of severe BSEP deficiency.<br><br>**14 CLINICAL STUDIES**<br>**14.2 Progressive Familial Intrahepatic Cholestasis**<br>The efficacy of LIVMARLI was assessed in Trial 2 (NCT03905330), a 26-week randomized, placebo-controlled trial.<br>Efficacy was evaluated in 64 patients with documented molecular diagnosis of PFIC with presence of biallelic known pathogenic variants; this included 31 non-truncated PFIC2 patients (i.e., excluding patients with BSEP3) and 33 patients with PFIC1 (n=13), PFIC3 (n=9), PFIC4 (n=7), or PFIC6 (n=4) [see Clinical Pharmacology (12.5)]. Additional patients with BSEP3 (n=9), prior surgical diversion (n=8), heterozygous subjects (n=2), or no variants associated with cholestasis (n=8) were included for evaluation in a supplemental cohort [see Adverse Reactions (6.1)]. (See Table 6 and Figure 1)
214662, 03/13/2024	Maralixibat (3)	Gastroenterology	ABCB11	Indications and Usage, Clinical	**1 INDICATIONS AND USAGE**<br>**1.2 Treatment of Cholestatic Pruritus in Patients with Progressive Familial Intrahepatic Cholestasis**

* Therapeutic areas do not necessarily reflect the CDER review division.
† Representative biomarkers are listed based on standard nomenclature as per the Human Genome Organization (HUGO) symbol and/or simplified descriptors using other common conventions. Listed biomarkers do not necessarily reflect the terminology used in labeling. The term "Nonspecific" is provided when labeling does not explicitly identify the specific biomarker(s) or when the biomarker is represented by a molecular phenotype or gene signature, and in some cases the biomarker was inferred based on the labeling language.
‡ Referenced figures and tables may be found in the labeling available at Drugs@FDA.
Blue text represents the most recent addtions and/or changes since last posted version.

Table of Pharmacogenomic Biomarkers in Drug Labeling
Last Updated: 06/2024

NDA/ANDA/BLA Number, Label Version Date	Drug	Therapeutic Area*	Biomarker†	Labeling Sections	Labeling Text‡
				Pharmacology, Clinical Studies	LIVMARLI is indicated for the treatment of cholestatic pruritus in patients 5 years of age and older with progressive familial intrahepatic cholestasis (PFIC).<br>Limitations of Use:<br>LIVMARLI is not recommended in a subgroup of PFIC type 2 patients with specific ABCB11 variants resulting in non-functional or complete absence of bile salt export pump (BSEP) protein [see Clinical Studies (14.2)].<br><br>**12 CLINICAL PHARMACOLOGY**<br>**12.5. Pharmacogenomics**<br>PFIC is a heterogenous disease caused by homozygous or compound heterozygous variants, with different PFIC subtypes occurring in the general population. PFIC1 is caused by variants in the aminophospholipid flippase (ATP8B1) gene, which encodes the Familial Intrahepatic Cholestasis 1 (FIC1) protein, while PFIC2 results from variants in the ABCB11 gene, which encodes the Bile Salt Export Pump (BSEP) protein. PFIC2 is further categorized into BSEP subgroups based on specific variants. The BSEP-1 subgroup includes patients with at least one p.D482G (c.1445A>G) or p.E297G (c.890A>G) variant, BSEP-2 includes patients with at least one missense variant other than p.D482G or p.E297G (non BSEP-1), and BSEP-3 includes patients with variants that are predicted to encode a non-functional protein. PFIC3 is caused by variants in the ABCB4 gene, which encodes multidrug resistance protein 3 (MDR3). PFIC4 is caused by variants in the tight junction protein 2 gene (TJP2), which encodes TJP2. PFIC6 is caused by variants in myosin 5B (MYO5B), which encodes MYO5B. Patients can be clinically diagnosed with PFIC without a known pathogenic variant.<br>PFIC2 is the most common subtype accounting for 37-90% of patients with PFIC. The prevalence of BSEP-1, BSEP-2, and BSEP-3 subgroups are approximately 27.3%, 51.5%, and 21.2%, respectively, based on data from a global consortium characterizing the natural history of severe BSEP deficiency.<br><br>**14 CLINICAL STUDIES**<br>**14.2 Progressive Familial Intrahepatic Cholestasis**<br>The efficacy of LIVMARLI was assessed in Trial 2 (NCT03905330), a 26-week randomized, placebo-controlled trial.<br>Efficacy was evaluated in 64 patients with documented molecular diagnosis of PFIC with presence of biallelic known pathogenic variants; this included 31 non-truncated PFIC2 patients (i.e., excluding patients with BSEP3) and 33 patients with PFIC1 (n=13), PFIC3 (n=9), PFIC4 (n=7), or PFIC6 (n=4) [see Clinical Pharmacology (12.5)]. Additional patients with BSEP3 (n=9), prior surgical diversion (n=8), heterozygous subjects (n=2), or no variants associated with cholestasis (n=8) were included for evaluation in a supplemental cohort [see Adverse Reactions (6.1)]. (See Table 6 and Figure 1)
**214662, 03/13/2024**	**Maralixibat (4)**	Gastroenterology	ATP8B1	Clinical Pharmacology, Clinical Studies	**12 CLINICAL PHARMACOLOGY**<br>**12.5. Pharmacogenomics**<br>PFIC is a heterogenous disease caused by homozygous or compound heterozygous variants, with different PFIC subtypes occurring in the general population. PFIC1 is caused by variants in the aminophospholipid flippase (ATP8B1) gene, which encodes the Familial Intrahepatic Cholestasis 1 (FIC1) protein, while PFIC2 results from variants in the ABCB11 gene, which encodes the Bile Salt Export Pump (BSEP) protein. PFIC2 is further categorized into BSEP subgroups based on specific variants. The BSEP-1 subgroup includes patients with at least one p.D482G (c.1445A>G) or p.E297G (c.890A>G) variant, BSEP-2 includes patients with at least one missense variant other than p.D482G or p.E297G (non BSEP-1), and BSEP-3 includes patients with variants that are predicted to encode a non-functional protein. PFIC3 is caused by variants in the ABCB4 gene, which encodes multidrug resistance protein 3 (MDR3). PFIC4 is caused by variants in the tight junction protein 2 gene (TJP2), which encodes TJP2. PFIC6 is caused by variants in myosin 5B (MYO5B), which encodes MYO5B. Patients can be clinically diagnosed with PFIC without a known pathogenic variant.<br>PFIC2 is the most common subtype accounting for 37-90% of patients with PFIC. The prevalence of BSEP-1, BSEP-2, and BSEP-3 subgroups are approximately 27.3%, 51.5%, and 21.2%, respectively, based on data from a global consortium characterizing the natural history of severe BSEP deficiency.<br><br>**14 CLINICAL STUDIES**<br>**14.2 Progressive Familial Intrahepatic Cholestasis**<br>The efficacy of LIVMARLI was assessed in Trial 2 (NCT03905330), a 26-week randomized, placebo-controlled trial.<br>Efficacy was evaluated in 64 patients with documented molecular diagnosis of PFIC with presence of biallelic known pathogenic variants; this included 31 non-truncated PFIC2 patients (i.e., excluding patients with BSEP3) and 33 patients with PFIC1 (n=13), PFIC3 (n=9), PFIC4 (n=7), or PFIC6 (n=4) [see Clinical Pharmacology (12.5)]. Additional patients with BSEP3 (n=9), prior surgical diversion (n=8), heterozygous subjects (n=2), or no variants associated with cholestasis (n=8) were included for evaluation in a supplemental cohort [see Adverse Reactions (6.1)]. (See Table 6 and Figure 1)
**214662, 03/13/2024**	**Maralixibat (5)**	Gastroenterology	MYO5B	Clinical Pharmacology, Clinical Studies	**12 CLINICAL PHARMACOLOGY**<br>**12.5. Pharmacogenomics**<br>PFIC is a heterogenous disease caused by homozygous or compound heterozygous variants, with different PFIC subtypes occurring in the general population. PFIC1 is caused by variants in the aminophospholipid flippase (ATP8B1) gene, which encodes the Familial Intrahepatic Cholestasis 1 (FIC1) protein, while PFIC2 results from variants in the ABCB11 gene, which encodes the Bile Salt Export Pump (BSEP) protein. PFIC2 is further categorized into BSEP subgroups based on specific variants. The BSEP-1 subgroup includes patients with at least one p.D482G (c.1445A>G) or p.E297G (c.890A>G) variant, BSEP-2 includes patients with at least one missense variant other than p.D482G or p.E297G (non BSEP-1), and BSEP-3 includes patients with variants that are predicted to encode a non-functional protein. PFIC3 is caused by variants in the ABCB4 gene, which encodes multidrug resistance protein 3 (MDR3). PFIC4 is caused by variants in the tight junction protein 2 gene (TJP2), which encodes TJP2. PFIC6 is caused by variants in myosin 5B (MYO5B), which encodes MYO5B. Patients can be clinically diagnosed with PFIC without a known pathogenic variant.<br>PFIC2 is the most common subtype accounting for 37-90% of patients with PFIC. The prevalence of BSEP-1, BSEP-2, and BSEP-3 subgroups are approximately 27.3%, 51.5%, and 21.2%, respectively, based on data from a global consortium characterizing the natural history of severe BSEP deficiency.<br><br>**14 CLINICAL STUDIES**<br>**14.2 Progressive Familial Intrahepatic Cholestasis**

* Therapeutic areas do not necessarily reflect the CDER review division.
† Representative biomarkers are listed based on standard nomenclature as per the Human Genome Organization (HUGO) symbol and/or simplified descriptors using other common conventions. Listed biomarkers do not necessarily reflect the terminology used in labeling. The term “Nonspecific” is provided when labeling does not explicitly identify the specific biomarker(s) or when the biomarker is represented by a molecular phenotype or gene signature, and in some cases the biomarker was inferred based on the labeling language.
‡ Referenced figures and tables may be found in the labeling available at Drugs@FDA.
Blue text represents the most recent addtions and/or changes since last posted version.

NDA/ANDA/BLA Number, Label Version Date	Drug	Therapeutic Area*	Biomarker†	Labeling Sections	Labeling Text‡
					The efficacy of LIVMARLI was assessed in Trial 2 (NCT03905330), a 26-week randomized, placebo-controlled trial.<br>Efficacy was evaluated in 64 patients with documented molecular diagnosis of PFIC with presence of biallelic known pathogenic variants; this included 31 non-truncated PFIC2 patients (i.e., excluding patients with BSEP3) and 33 patients with PFIC1 (n=13), PFIC3 (n=9), PFIC4 (n=7), or PFIC6 (n=4) [see Clinical Pharmacology (12.5)]. Additional patients with BSEP3 (n=9), prior surgical diversion (n=8), heterozygous subjects (n=2), or no variants associated with cholestasis (n=8) were included for evaluation in a supplemental cohort [see Adverse Reactions (6.1)]. (See Table 6 and Figure 1)
**214662, 03/13/2024**	**Maralixibat (6)**	Gastroenterology	TJP2	Clinical Pharmacology, Clinical Studies	**12 CLINICAL PHARMACOLOGY**<br>**12.5. Pharmacogenomics**<br>PFIC is a heterogenous disease caused by homozygous or compound heterozygous variants, with different PFIC subtypes occurring in the general population. PFIC1 is caused by variants in the aminophospholipid flippase (ATP8B1) gene, which encodes the Familial Intrahepatic Cholestasis 1 (FIC1) protein, while PFIC2 results from variants in the ABCB11 gene, which encodes the Bile Salt Export Pump (BSEP) protein. PFIC2 is further categorized into BSEP subgroups based on specific variants. The BSEP-1 subgroup includes patients with at least one p.D482G (c.1445A>G) or p.E297G (c.890A>G) variant, BSEP-2 includes patients with at least one missense variant other than p.D482G or p.E297G (non BSEP-1), and BSEP-3 includes patients with variants that are predicted to encode a non-functional protein. PFIC3 is caused by variants in the ABCB4 gene, which encodes multidrug resistance protein 3 (MDR3). PFIC4 is caused by variants in the tight junction protein 2 gene (TJP2), which encodes TJP2. PFIC6 is caused by variants in myosin 5B (MYO5B), which encodes MYO5B. Patients can be clinically diagnosed with PFIC without a known pathogenic variant.<br>PFIC2 is the most common subtype accounting for 37-90% of patients with PFIC. The prevalence of BSEP-1, BSEP-2, and BSEP-3 subgroups are approximately 27.3%, 51.5%, and 21.2%, respectively, based on data from a global consortium characterizing the natural history of severe BSEP deficiency.<br><br>**14 CLINICAL STUDIES**<br>**14.2 Progressive Familial Intrahepatic Cholestasis**<br>The efficacy of LIVMARLI was assessed in Trial 2 (NCT03905330), a 26-week randomized, placebo-controlled trial.<br>Efficacy was evaluated in 64 patients with documented molecular diagnosis of PFIC with presence of biallelic known pathogenic variants; this included 31 non-truncated PFIC2 patients (i.e., excluding patients with BSEP3) and 33 patients with PFIC1 (n=13), PFIC3 (n=9), PFIC4 (n=7), or PFIC6 (n=4) [see Clinical Pharmacology (12.5)]. Additional patients with BSEP3 (n=9), prior surgical diversion (n=8), heterozygous subjects (n=2), or no variants associated with cholestasis (n=8) were included for evaluation in a supplemental cohort [see Adverse Reactions (6.1)]. (See Table 6 and Figure 1)
**761150, 12/16/2020**	**Margetuximab-cmkb (1)**	Oncology	ERBB2 (HER2)	Indications and Usage, Adverse Reactions, Clinical Pharmacology, Clinical Studies	**1 INDICATIONS AND USAGE**<br>MARGENZA is indicated, in combination with chemotherapy, for the treatment of adult patients with metastatic HER2-positive breast cancer who have received two or more prior anti-HER2 regimens, at least one of which was for metastatic disease [see Dosage and Administration (2.1) and Clinical Studies (14.1)].<br><br>**6 ADVERSE REACTIONS**<br>**6.1 Clinical Trials Experience**<br>The safety of MARGENZA was evaluated in HER2-positive breast cancer patients who received two or more prior anti-HER2 regimens in SOPHIA [see Clinical Studies (14.1)]. (See Tables 1 and 2) (…)<br><br>**12 CLINICAL PHARMACOLOGY**<br>**12.3 Pharmacokinetics**<br>Following the approved recommended dosage, the steady-state geometric mean (% CV) Cmax of margetuximab-cmkb is 466 (20%) µg/mL and AUC0-21d is 4120 (21%) µg.day/mL in patients with HER2-positive relapsed or refractory advanced breast cancer. (…)<br>Specific Populations<br>No clinically significant differences in margetuximab-cmkb PK were observed based on age (29 to 83 years), sex, race (Caucasian, Black, Asian), mild to moderate (CLcr 30 to 89 mL/min estimated using the Cockcroft-Gault equation) renal impairment, mild hepatic impairment (total bilirubin ≤ ULN and AST > ULN, or total bilirubin 1 to 1.5 ULN and any AST), HER2 expression level (0 to 3 by IHC), tumor burden (2 – 317 mm), ECOG score (0 to 2), albumin (24 to 50 g/L), FCGR3A (CD16A), FCGR2A (CD32A) and FCGR2B (CD32B) genotype, number of metastatic sites (≤ 2 or > 2), number of prior therapy lines (≤ 2 or > 2) or concurrent chemotherapies (capecitabine, gemcitabine, eribulin and vinorelbine). The effect of severe renal impairment (CLcr 15 to 29 mL/min), end-stage renal disease with or without hemodialysis, and moderate (total bilirubin > 1.5 to ≤ 3 ULN and any AST) or severe hepatic impairment (total bilirubin >3 ULN and any AST) on margetuximab-cmkb PK is unknown.<br><br>**14 CLINICAL STUDIES**<br>**14.1 Metastatic Breast Cancer**<br>The efficacy of MARGENZA plus chemotherapy was evaluated in SOPHIA (NCT02492711), a randomized, multicenter, open-label trial of 536 patients with IHC 3+ or ISH-amplified HER2+ metastatic breast cancer who had received prior treatment with other anti-HER2 therapies. Patients were randomized (1:1) to MARGENZA plus chemotherapy or trastuzumab plus chemotherapy.
**761150, 12/16/2020**	**Margetuximab-cmkb (2)**	Oncology	FCGR2A (CD32A)	Clinical Pharmacology	**12 CLINICAL PHARMACOLOGY**<br>**12.3 Pharmacokinetics**<br>Specific Populations<br>No clinically significant differences in margetuximab-cmkb PK were observed based on age (29 to 83 years), sex, race (Caucasian, Black, Asian), mild to moderate (CLcr 30 to 89 mL/min estimated using the Cockcroft-Gault equation) renal impairment, mild hepatic impairment (total bilirubin ≤ ULN and AST > ULN, or total bilirubin 1 to 1.5 ULN and any AST), HER2 expression level (0 to 3 by IHC), tumor burden (2 – 317 mm), ECOG score (0 to 2), albumin (24 to 50 g/L), FCGR3A (CD16A), FCGR2A (CD32A) and FCGR2B (CD32B) genotype, number of metastatic sites (≤ 2 or > 2), number of prior therapy lines (≤ 2 or > 2) or concurrent chemotherapies (capecitabine, gemcitabine, eribulin and vinorelbine). The effect of severe renal impairment (CLcr 15 to 29 mL/min), end-stage renal

NDA/ANDA/BLA Number, Label Version Date	Drug	Therapeutic Area*	Biomarker†	Labeling Sections	Labeling Text‡
					disease with or without hemodialysis, and moderate (total bilirubin > 1.5 to ≤ 3 ULN and any AST) or severe hepatic impairment (total bilirubin >3 ULN and any AST) on margetuximab-cmkb PK is unknown.
**761150, 12/16/2020**	**Margetuximab-cmkb (3)**	Oncology	FCGR2B (CD32B)	Clinical Pharmacology	**12 CLINICAL PHARMACOLOGY**<br>**12.3 Pharmacokinetics**<br>Specific Populations<br>No clinically significant differences in margetuximab-cmkb PK were observed based on age (29 to 83 years), sex, race (Caucasian, Black, Asian), mild to moderate (CLcr 30 to 89 mL/min estimated using the Cockcroft-Gault equation) renal impairment, mild hepatic impairment (total bilirubin ≤ ULN and AST > ULN, or total bilirubin 1 to 1.5 ULN and any AST), HER2 expression level (0 to 3 by IHC), tumor burden (2 – 317 mm), ECOG score (0 to 2), albumin (24 to 50 g/L), FCGR3A (CD16A), FCGR2A (CD32A) and FCGR2B (CD32B) genotype, number of metastatic sites (≤ 2 or > 2), number of prior therapy lines (≤ 2 or > 2) or concurrent chemotherapies (capecitabine, gemcitabine, eribulin and vinorelbine). The effect of severe renal impairment (CLcr 15 to 29 mL/min), end-stage renal disease with or without hemodialysis, and moderate (total bilirubin > 1.5 to ≤ 3 ULN and any AST) or severe hepatic impairment (total bilirubin >3 ULN and any AST) on margetuximab-cmkb PK is unknown.
**761150, 12/16/2020**	**Margetuximab-cmkb (4)**	Oncology	FCGR3A (CD16A)	Clinical Pharmacology	**12 CLINICAL PHARMACOLOGY**<br>**12.3 Pharmacokinetics**<br>Specific Populations<br>No clinically significant differences in margetuximab-cmkb PK were observed based on age (29 to 83 years), sex, race (Caucasian, Black, Asian), mild to moderate (CLcr 30 to 89 mL/min estimated using the Cockcroft-Gault equation) renal impairment, mild hepatic impairment (total bilirubin ≤ ULN and AST > ULN, or total bilirubin 1 to 1.5 ULN and any AST), HER2 expression level (0 to 3 by IHC), tumor burden (2 – 317 mm), ECOG score (0 to 2), albumin (24 to 50 g/L), FCGR3A (CD16A), FCGR2A (CD32A) and FCGR2B (CD32B) genotype, number of metastatic sites (≤ 2 or > 2), number of prior therapy lines (≤ 2 or > 2) or concurrent chemotherapies (capecitabine, gemcitabine, eribulin and vinorelbine). The effect of severe renal impairment (CLcr 15 to 29 mL/min), end-stage renal disease with or without hemodialysis, and moderate (total bilirubin > 1.5 to ≤ 3 ULN and any AST) or severe hepatic impairment (total bilirubin >3 ULN and any AST) on margetuximab-cmkb PK is unknown.
**214998, 04/28/2022**	**Mavacamten**	Cardiology	CYP2C19	Dosage and Administration, Clinical Pharmacology	**2. DOSAGE AND ADMINISTRATION**<br>**2.1. Initiation, Maintenance, and Interruption of Treatment**<br>Confirm absence of pregnancy and usage of effective contraception in females of reproductive potential [see Warnings and Precautions (5.4)].<br>Initiation or up-titration of CAMZYOS in patients with LVEF <55% is not recommended.<br>The recommended starting dose is 5 mg once daily without regard to food; allowable subsequent doses with titration are 2.5, 5, 10, or 15 mg once daily. Patients may develop heart failure while taking CAMZYOS. Regular LVEF and Valsalva left ventricular outflow tract (LVOT) gradient assessment is required for careful titration to achieve an appropriate target Valsalva LVOT gradient, while maintaining LVEF ≥50% and avoiding heart failure symptoms (see Figure 1 and Figure 2).<br>Daily dosing takes weeks to reach steady-state drug levels and therapeutic effects, and genetic variation in metabolism and drug interactions can cause large differences in exposure [see Boxed Warning, Contraindications (4), Warnings and Precautions (5.2), Drug Interactions (7.1) and Clinical Pharmacology (12.3)].<br>When initiating or titrating CAMZYOS, first consider LVEF then consider the Valsalva LVOT gradient and patient clinical status to guide appropriate CAMZYOS dosing. Follow the algorithms for Initiation (Figure 1) and Maintenance (Figure 2) for appropriate CAMZYOS dosing and monitoring schedules.<br>If LVEF <50% while taking CAMZYOS, interrupt treatment. Follow the algorithm for Interruption (Figure 3) for guidance on interrupting, restarting, or discontinuing CAMZYOS. If interrupted at 2.5 mg, either restart at 2.5 mg or discontinue permanently. (See Figures 1-3)<br>Delay dose increases when there is intercurrent illness (e.g., serious infection) or arrhythmia (e.g., atrial fibrillation or other uncontrolled tachyarrhythmia) that may impair systolic function. Consider interruption of CAMZYOS in patients with intercurrent illness [see Warnings and Precautions (5.1)].<br>Missed or delayed doses<br>If a dose is missed, it should be taken as soon as possible, and the next scheduled dose should be taken at the usual time the following day. Exact timing of dosing during the day is not essential, but two doses should not be taken on the same day. Swallow capsules whole. Do not break, open, or chew the capsules.<br><br>**12 CLINICAL PHARMACOLOGY**<br>**12.3 Pharmacokinetics**<br>Elimination<br>Mavacamten has a variable terminal t1/2 that depends on CYP2C19 metabolic status. Mavacamten terminal half-life is 6-9 days in CYP2C19 normal metabolizers (NMs), which is prolonged in CYP2C19 poor metabolizers (PMs) to 23 days. Drug accumulation occurs with an accumulation ratio of about 2-fold for Cmax and about 7-fold for AUC in CYP2C19 NMs. The accumulation depends on the metabolism status for CYP2C19 with the largest accumulation observed in CYP2C19 PMs. At steady-state, the peak-to-trough plasma concentration ratio with once daily dosing is approximately 1.5.<br>Drug Interactions<br>*Clinical Studies and Model-Informed Approaches*<br>*Weak CYP2C19 Inhibitors:* Concomitant use of mavacamten (15 mg) with omeprazole (20 mg) once daily increased mavacamten AUCinf by 48% with no effect on Cmax in healthy CYP2C19 NMs and rapid metabolizers (RMs; e.g., *1/*17).<br>*Moderate CYP3A4 Inhibitors:* Concomitant use of mavacamten (25 mg) with verapamil sustained release (240 mg) increased mavacamten AUCinf by 15% and Cmax by 52% in intermediate metabolizers (IMs; e.g., *1/*2, *1/*3, *2/*17, *3/*17) and NMs of CYP2C19. Concomitant use of mavacamten with diltiazem in CYP2C19 PMs is predicted to increase mavacamten AUC0-24h and Cmax up to 55% and 42%, respectively.<br>*Strong CYP3A4 Inhibitors:* Concomitant use of mavacamten (15 mg) with ketoconazole 400 mg once daily is predicted to increase mavacamten AUC0-24 and Cmax up to 130% and 90%, respectively.

* Therapeutic areas do not necessarily reflect the CDER review division.
† Representative biomarkers are listed based on standard nomenclature as per the Human Genome Organization (HUGO) symbol and/or simplified descriptors using other common conventions. Listed biomarkers do not necessarily reflect the terminology used in labeling. The term “Nonspecific” is provided when labeling does not explicitly identify the specific biomarker(s) or when the biomarker is represented by a molecular phenotype or gene signature, and in some cases the biomarker was inferred based on the labeling language.
‡ Referenced figures and tables may be found in the labeling available at Drugs@FDA.
Blue text represents the most recent addtions and/or changes since last posted version.

Table of Pharmacogenomic Biomarkers in Drug Labeling
Last Updated: 06/2024

NDA/ANDA/BLA Number, Label Version Date	Drug	Therapeutic Area*	Biomarker†	Labeling Sections	Labeling Text‡
					Strong CYP2C19 and CYP3A4 Inducers: Concomitant use of mavacamten (a single 15 mg dose) with a strong CYP2C19 and CYP3A4 inducer (rifampin 600 mg daily dose) is predicted to decrease mavacamten AUC0-inf and Cmax by 87% and 22%, respectively, in CYP2C19 NMs, and by 69% and 4%, respectively, in CYP2C19 PMs.<br>*CYP3A4 Substrates:* Concomitant use of a 16-day course of mavacamten (25 mg on days 1 and 2, followed by 15 mg for 14 days) resulted in a 13% and 7% decrease in midazolam AUCinf and Cmax, respectively, in healthy CYP2C19 NMs. Following coadministration of mavacamten once daily in HCM patients, midazolam AUCinf and Cmax are predicted to decrease by 21 to 64% and 13 to 48%, respectively, depending on the dose of mavacamten and CYP2C19 phenotype. *CYP2C8 Substrates:* Concomitant use of mavacamten once daily in HCM patients is predicted to decrease AUC and Cmax of repaglinide, a CYP2C8 and CYP3A substrate, by 12 to 39%, depending on the dose of mavacamten and CYP2C19 phenotype.<br>*CYP2C9 Substrates:* Concomitant use of mavacamten once daily in HCM patients is predicted to decrease AUC and Cmax of tolbutamide, a CYP2C9 substrate, by 33 to 65%, depending on the dose of mavacamten and CYP2C19 phenotype.<br>*CYP2C19 Substrates:* Concomitant use of mavacamten once daily in HCM patients is predicted to decrease AUC and Cmax of omeprazole, a CYP2C19 substrate, by 48 to 67%, depending on the dose of mavacamten and CYP2C19 phenotype.<br>**12.5 Pharmacogenomics**<br>Mavacamten AUCinf increased by 241% and Cmax increased by 47% in CYP2C19 poor metabolizers (PMs) compared to normal metabolizers (NMs) following a single dose of 15 mg mavacamten. Mean half-life is prolonged in CYP2C19 PMs compared to NMs (23 days vs. 6 to 9 days, respectively).<br>Polymorphic CYP2C19 is the main enzyme involved in the metabolism of CAMZYOS. An individual carrying two normal function alleles is a NM (e.g., *1/*1). An individual carrying two no function alleles is a PM (e.g., *2/*2, *2/*3, *3/*3).<br>The prevalence of CYP2C19 poor metabolizers differs depending on ancestry. Approximately 2% of individuals of European ancestry and 4% of individuals of African ancestry are PMs; the prevalence of PMs is higher in Asian populations (e.g., approximately 13% of East Asians).
**218709, 04/26/2024**	**Mavorixafor**	Hematology	CXCR4	Clinical Studies	**14 CLINICAL STUDIES**<br>The efficacy of XOLREMDI in patients aged 12 years and older with WHIM syndrome was demonstrated in the 52-week, randomized, double-blind, placebo-controlled portion of Study 1 [NCT03995108]. Enrolled patients had a genotype-confirmed variant of CXCR4 consistent with WHIM syndrome, and a confirmed absolute neutrophil count (ANC) ≤400 cells/μL. Patients were permitted to continue (but not initiate) immunoglobulin therapy at the same dose. Use of other CXCR4 antagonists was not permitted. (…)
**021839, 03/15/2024**	**Mecasermin**	Endocrinology	Nonspecific (GH)	Indications and Usage, Clinical Studies	**1 INDICATIONS AND USAGE**<br>Severe Primary IGF-1 Deficiency (Primary IGFD)<br>INCRELEX is indicated for the treatment of growth failure in pediatric patients 2 years of age and older with:<br>1 severe primary IGF-1 deficiency or<br>2 growth hormone (GH) gene deletion who have developed neutralizing antibodies to GH.<br>Severe Primary IGF-1 deficiency (IGFD) is defined by:<br>• height standard deviation score ≤ –3.0 and<br>• basal IGF-1 standard deviation score ≤ –3.0 and<br>• normal or elevated growth hormone (GH).<br>Limitations of use:<br>INCRELEX is not a substitute to GH for approved GH indications.<br>INCRELEX is not indicated for use in patients with secondary forms of IGF-1 deficiency, such as GH deficiency, malnutrition, hypothyroidism, or chronic treatment with pharmacologic doses of anti-inflammatory corticosteroids.<br><br>**14 CLINICAL STUDIES**<br>**14.1 Effects of INCRELEX Treatment in Children with Severe Primary Insulin-like Growth Factor-1 Deficiency (Severe Primary IGFD)**<br>Five clinical studies (four open-label and one double-blind, placebo-controlled), with subcutaneous doses of INCRELEX generally ranging from 0.06 to 0.12 mg/kg (60 to 120 micrograms/kg) administered twice daily, were conducted in 71 pediatric subjects with severe Primary IGFD. Patients were enrolled in the trials on the basis of extreme short stature, slow growth rates, low IGF-1 serum concentrations, and normal growth hormone secretion. Data from these 5 clinical studies were pooled for a global efficacy and safety analysis. Baseline characteristics for the patients evaluated in the primary and secondary efficacy analyses were (mean, SD): chronological age (years): 6.7 ± 3.8; height (cm): 84.8 ± 15.3 cm; height standard deviation score (SDS): -6.7 ± 1.8; height velocity (cm/yr): 2.8 ± 1.8; height velocity SDS: -3.3 ± 1.7; IGF-1 (ng/mL): 21.6 ± 20.6; IGF-1 SDS: -4.3 ± 1.6; and bone age (years): 4.2 ± 2.8. Sixty-one subjects had at least one year of treatment. Fifty-three (87%) had Laron Syndrome; 7 (11%) had GH gene deletion, and 1 (2%) had neutralizing antibodies to GH. Thirty-seven (61%) of the subjects were male; forty-eight (79%) were Caucasian. Fifty-six (92%) of the subjects were pre-pubertal at baseline.
**010721, 06/28/2019**	**Meclizine**	Neurology	CYP2D6	Warnings and Precautions	**8 USE IN SPECIFIC POPULATIONS**<br>**8.8 Genetic CYP2D6 Polymorphism**<br>The genetic polymorphism of CYP2D6 that results in poor-, intermediate-, extensive-, and ultrarapid metabolizer phenotypes could contribute to large inter-individual variability in meclizine exposure. Therefore, when ANTIVERT® is administered to patients with CYP2D6 polymorphism, monitor for adverse reactions and clinical effect accordingly.
**011839, 02/15/2024**	**Medroxyprogesterone**	Gynecology	ESR (Hormone Receptor)	Warnings	**WARNINGS**<br>**2. Malignant Neoplasms**<br>**a. Breast Cancer**<br>The WHI substudy of daily CE (0.625 mg)-alone provided information about breast cancer in estrogen-alone users. In the WHI estrogen-alone substudy, after an average follow-up of 7.1 years, daily CE-alone was not associated with an increased risk of invasive breast cancer [relative risk (RR) 0.80] compared to placebo (see CLINICAL STUDIES.)

* Therapeutic areas do not necessarily reflect the CDER review division.
† Representative biomarkers are listed based on standard nomenclature as per the Human Genome Organization (HUGO) symbol and/or simplified descriptors using other common conventions. Listed biomarkers do not necessarily reflect the terminology used in labeling. The term "Nonspecific" is provided when labeling does not explicitly identify the specific biomarker(s) or when the biomarker is represented by a molecular phenotype or gene signature, and in some cases the biomarker was inferred based on the labeling language.
‡ Referenced figures and tables may be found in the labeling available at Drugs@FDA.
Blue text represents the most recent addtions and/or changes since last posted version.

Table of Pharmacogenomic Biomarkers in Drug Labeling
Last Updated: 06/2024

NDA/ANDA/BLA Number, Label Version Date	Drug	Therapeutic Area*	Biomarker†	Labeling Sections	Labeling Text‡
					After a mean follow-up of 5.6 years, the estrogen plus progestin substudy reported an increased risk of invasive breast cancer in women who took daily CE plus MPA. In this substudy, prior use of estrogen alone or estrogen plus progestin therapy was reported by 26 percent of the women. The relative risk of invasive breast cancer was 1.24, and the absolute risk was 41 versus 33 cases per 10,000 women-years, for CE plus MPA compared with placebo. Among women who reported prior use of hormone therapy, the relative risk of invasive breast cancer was 1.86, and the absolute risk was 46 versus 25 cases per 10,000 women-years, for CE plus MPA compared with placebo. Among women who reported no prior use of hormone therapy, the relative risk of invasive breast cancer was 1.09, and the absolute risk was 40 versus 36 cases per 10,000 women-years, for CE plus MPA compared with placebo. In the same substudy, invasive breast cancers were larger , were more likely to be node positive, and were diagnosed at a more advanced stage in the CE(0.625 mg) plus MPA (2.5 mg) group compared with the placebo group. Metastatic disease was rare with no apparent difference between the two groups. Other prognostic factors such as histologic subtype, grade, and hormone receptor status did not differ between the groups. (See CLINICAL STUDIES.) (…)
**211210, 10/19/2019**	**Meloxicam**	Anesthesiology	CYP2C9	Use in Specific Populations, Clinical Pharmacology	**8 USE IN SPECIFIC POPULATIONS**<br>**8.8 Poor Metabolizers of CYP2C9 Substrates**<br>In patients who are known or suspected to be poor CYP2C9 metabolizers based on genotype or previous history/experience with other CYP2C9 substrates (such as warfarin or phenytoin), consider dose reduction, as these patients may have abnormally high plasma levels of meloxicam due to reduced metabolic clearance. Monitor these patients for adverse effects.<br><br>**12 CLINICAL PHARMACOLOGY**<br>**12.5 Pharmacogenomics**<br>CYP2C9 activity is reduced in individuals with genetic variants such as CYP2C9*2 and CYP2C9*3 polymorphisms. Limited data from three published reports showed that meloxicam AUC was substantially higher in individuals with reduced CYP2C9 activity, particularly in poor metabolizers (e.g., *3/*3), compared to normal metabolizers (*1/*1). The frequency of CYP2C9 poor metabolizer genotypes varies based on racial/ethnic background but is generally present in <5% of the population.
**012250, 11/02/2018**	**Mepivacaine (1)**	Anesthesiology	G6PD	Warnings	**WARNINGS**<br>**Methemoglobinemia**<br>Cases of methemoglobinemia have been reported in association with local anesthetic use. Although all patients are at risk for methemoglobinemia, patients with glucose-6-phosphate dehydrogenase deficiency, congenital or idiopathic methemoglobinemia, cardiac or pulmonary compromise, infants under 6 months of age, and concurrent exposure to oxidizing agents or their metabolites are more susceptible to developing clinical manifestations of the condition. If local anesthetics must be used in these patients, close monitoring for symptoms and signs of methemoglobinemia is recommended. (…)
**012250, 11/02/2018**	**Mepivacaine (2)**	Anesthesiology	Nonspecific (Congenital Methemoglobinemia)	Warnings	**WARNINGS**<br>**Methemoglobinemia**<br>Cases of methemoglobinemia have been reported in association with local anesthetic use. Although all patients are at risk for methemoglobinemia, patients with glucose-6-phosphate dehydrogenase deficiency, congenital or idiopathic methemoglobinemia, cardiac or pulmonary compromise, infants under 6 months of age, and concurrent exposure to oxidizing agents or their metabolites are more susceptible to developing clinical manifestations of the condition. If local anesthetics must be used in these patients, close monitoring for symptoms and signs of methemoglobinemia is recommended. (…)
**125526, 03/08/2023**	**Mepolizumab**	Oncology	FIP1L1-PDGFRA	Adverse Reactions, Clinical Studies	**6 ADVERSE REACTIONS**<br>**6.4 Clinical Trials Experience in Hypereosinophilic Syndrome**<br>A total of 108 adult and adolescent patients aged 12 years and older with HES were evaluated in a randomized, placebo-controlled, multicenter, 32-week treatment trial. Patients with non-hematologic secondary HES or FIP1L1-PDGFRα kinase-positive HES were excluded from the trial. (…)<br><br>**14 CLINICAL STUDIES**<br>**14.4 Hypereosinophilic Syndrome**<br>A total of 108 adult and adolescent patients aged 12 years and older with HES for at least 6 months were evaluated in a randomized, double-blind, placebo-controlled, multicenter, 32-week trial (NCT02836496). Patients with non-hematologic secondary HES (e.g., drug hypersensitivity, parasitic helminth infection, HIV infection, non-hematologic malignancy) or FIP1L1-PDGFRα kinase-positive HES were excluded from the trial. (…)
**205919, 04/29/2020**	**Mercaptopurine (1)**	Oncology	TPMT	Dosage and Administration, Warnings and Precautions, Clinical Pharmacology	**2 DOSAGE AND ADMINISTRATION**<br>**2.1 Maintenance Therapy**<br>The recommended starting dose of PURIXAN in multi-agent combination chemotherapy maintenance regimens is 1.5 to 2.5 mg/kg (50 to 75 mg/m^2) as a single daily dose. After initiating PURIXAN, monitor complete blood counts (CBCs), transaminases, and bilirubin. Maintain ANC at a desirable level by reducing the dose in patients with excessive hematological toxicity. Evaluate the bone marrow in patients with prolonged or repeated marrow suppression to assess leukemia status and marrow cellularity. Evaluate thiopurine Smethyltransferase (TPMT) and nucleotide diphosphatase (NUDT15) status in patients with clinical or laboratory evidence of severe bone marrow toxicity, or repeated episodes of myelosuppression.<br>**2.2 Dosage in Patients with TPMT and/or NUDT15 Deficiency**<br>Consider testing for TPMT and NUDT15 deficiency in patients who experience severe bone marrow toxicities or repeated episodes of myelosuppression [see Warnings and Precautions (5.1) and Clinical Pharmacology (12.5)].<br>*Homozygous deficiency in either TPMT or NUDT15*<br>Patients with homozygous deficiency of either enzyme typically require 10% or less of the standard PURIXAN dosage. Reduce initial dosage in patients who are known to have homozygous TPMT or NUDT15 deficiency.<br>*Heterozygous deficiency in TPMT and/or NUDT15*

* Therapeutic areas do not necessarily reflect the CDER review division.
† Representative biomarkers are listed based on standard nomenclature as per the Human Genome Organization (HUGO) symbol and/or simplified descriptors using other common conventions. Listed biomarkers do not necessarily reflect the terminology used in labeling. The term “Nonspecific” is provided when labeling does not explicitly identify the specific biomarker(s) or when the biomarker is represented by a molecular phenotype or gene signature, and in some cases the biomarker was inferred based on the labeling language.
‡ Referenced figures and tables may be found in the labeling available at Drugs@FDA.
Blue text represents the most recent addtions and/or changes since last posted version.

NDA/ANDA/BLA Number, Label Version Date	Drug	Therapeutic Area*	Biomarker†	Labeling Sections	Labeling Text‡
					Reduce the PURIXAN dosage based on tolerability. Most patients with heterozygous TPMT or NUDT15 deficiency tolerate recommended mercaptopurine doses, but some require dose reduction based on toxicities. Patients who are heterozygous for both TPMT and NUDT15 may require more substantial dosage reductions.<br><br>**5 WARNINGS AND PRECAUTIONS**<br>**5.1 Myelosuppression**<br>The most consistent, dose-related toxicity of PURIXAN is bone marrow suppression, manifested by anemia, leukopenia, thrombocytopenia, or any combination of these. Monitor CBC and adjust the dose of PURIXAN for severe neutropenia and thrombocytopenia.<br>Evaluate patients with repeated severe myelosuppression for thiopurine S-methyltransferase (TPMT) or nucleotide diphosphatase (NUDT15) deficiency. TPMT genotyping or phenotyping (red blood cell TPMT activity) and NUDT15 genotyping can identify patients who have reduced activity of these enzymes. Patients with homozygous TPMT or NUDT15 deficiency require substantial dosage reductions of PURIXAN [see Dosage and Administration (2.2) and Clinical Pharmacology (12.5)].<br>Avoid the concurrent use of allopurinol and PURIXAN. Concomitant allopurinol and PURIXAN can result in a significant increase in bone marrow toxicity. Myelosuppression can be exacerbated by coadministration with drugs that inhibit TPMT (e.g., olsalazine, mesalamine, or sulfasalazine) or drugs whose primary or secondary toxicity is myelosuppression [see Drug Interactions (7.1, 7.3 and 7.4)].<br><br>**12 CLINICAL PHARMACOLOGY**<br>**12.5 Pharmacogenomics**<br>Several published studies indicate that patients with reduced TPMT or NUDT15 activity receiving usual doses of mercaptopurine, accumulate excessive cellular concentrations of active 6-TGNs, and are at higher risk for severe myelosuppression [see Warnings and Precautions (5.1)]. In a study of 1028 children with ALL, the approximate tolerated mercaptopurine dosage range for patients with TPMT and/or NUDT15 deficiency on mercaptopurine maintenance therapy (as a percentage of the planned dosage) was as follows: heterozygous for either TPMT or NUDT15, 50-90%; heterozygous for both TPMT and NUDT15, 30-50%; homozygous for either TPMT or NUDT15, 5-10%. Approximately 0.3% (1:300) of patients of European or African ancestry have two loss-of-function alleles of the TPMT gene and have little or no TMPT activity (homozygous deficient or poor metabolizers), and approximately 10% of patients have one loss-of-function TPMT allele leading to intermediate TPMT activity (heterozygous deficient or intermediate metabolizers). The TPMT*2, TPMT*3A, and TPMT*3C alleles account for about 95% of individuals with reduced levels of TPMT activity. NUDT15 deficiency is detected in <1% of patients of European or African ancestry. Among patients of East Asian ancestry (i.e., Chinese, Japanese, Vietnamese), 2% have two loss-of-function alleles of the NUDT15 gene, and approximately 21% have one loss-of-function allele. The p.R139C variant of NUDT15 (present on the *2 and *3 alleles) is the most commonly observed, but other less common loss-of-function NUDT15 alleles have been observed.<br>Consider all clinical information when interpreting results from phenotypic testing used to determine the level of thiopurine nucleotides or TPMT activity in erythrocytes, since some coadministered drugs can influence measurement of TPMT activity in blood, and blood from recent transfusions will misrepresent a patient's actual TPMT activity [see Dosage and Administration (2.2) and Warnings and Precautions (5.1)].
**205919, 04/29/2020**	Mercaptopurine (2)	Oncology	NUDT15	Dosage and Administration, Warnings and Precautions, Clinical Pharmacology	**2 DOSAGE AND ADMINISTRATION**<br>**2.1 Maintenance Therapy**<br>The recommended starting dose of PURIXAN in multi-agent combination chemotherapy maintenance regimens is 1.5 to 2.5 mg/kg (50 to 75 mg/m^2) as a single daily dose. After initiating PURIXAN, monitor complete blood counts (CBCs), transaminases, and bilirubin. Maintain ANC at a desirable level by reducing the dose in patients with excessive hematological toxicity. Evaluate the bone marrow in patients with prolonged or repeated marrow suppression to assess leukemia status and marrow cellularity. Evaluate thiopurine Smethyltransferase (TPMT) and nucleotide diphosphatase (NUDT15) status in patients with clinical or laboratory evidence of severe bone marrow toxicity, or repeated episodes of myelosuppression.<br>**2.2 Dosage in Patients with TPMT and/or NUDT15 Deficiency**<br>Consider testing for TPMT and NUDT15 deficiency in patients who experience severe bone marrow toxicities or repeated episodes of myelosuppression [see Warnings and Precautions (5.1) and Clinical Pharmacology (12.5)].<br>*Homozygous deficiency in either TPMT or NUDT15*<br>Patients with homozygous deficiency of either enzyme typically require 10% or less of the standard PURIXAN dosage. Reduce initial dosage in patients who are known to have homozygous TPMT or NUDT15 deficiency.<br>*Heterozygous deficiency in TPMT and/or NUDT15*<br>Reduce the PURIXAN dosage based on tolerability. Most patients with heterozygous TPMT or NUDT15 deficiency tolerate recommended mercaptopurine doses, but some require dose reduction based on toxicities. Patients who are heterozygous for both TPMT and NUDT15 may require more substantial dosage reductions.<br><br>**5 WARNINGS AND PRECAUTIONS**<br>**5.1 Myelosuppression**<br>The most consistent, dose-related toxicity of PURIXAN is bone marrow suppression, manifested by anemia, leukopenia, thrombocytopenia, or any combination of these. Monitor CBC and adjust the dose of PURIXAN for severe neutropenia and thrombocytopenia.<br>Evaluate patients with repeated severe myelosuppression for thiopurine S-methyltransferase (TPMT) or nucleotide diphosphatase (NUDT15) deficiency. TPMT genotyping or phenotyping (red blood cell TPMT activity) and NUDT15 genotyping can identify patients who have reduced activity of these enzymes. Patients with homozygous TPMT or NUDT15 deficiency require substantial dosage reductions of PURIXAN [see Dosage and Administration (2.2) and Clinical Pharmacology (12.5)].

* Therapeutic areas do not necessarily reflect the CDER review division.
† Representative biomarkers are listed based on standard nomenclature as per the Human Genome Organization (HUGO) symbol and/or simplified descriptors using other common conventions. Listed biomarkers do not necessarily reflect the terminology used in labeling. The term "Nonspecific" is provided when labeling does not explicitly identify the specific biomarker(s) or when the biomarker is represented by a molecular phenotype or gene signature, and in some cases the biomarker was inferred based on the labeling language.
‡ Referenced figures and tables may be found in the labeling available at Drugs@FDA.
Blue text represents the most recent addtions and/or changes since last posted version.

Table of Pharmacogenomic Biomarkers in Drug Labeling
Last Updated: 06/2024

NDA/ANDA/BLA Number, Label Version Date	Drug	Therapeutic Area*	Biomarker†	Labeling Sections	Labeling Text‡
					Avoid the concurrent use of allopurinol and PURIXAN. Concomitant allopurinol and PURIXAN can result in a significant increase in bone marrow toxicity. Myelosuppression can be exacerbated by coadministration with drugs that inhibit TPMT (e.g., olsalazine, mesalamine, or sulfasalazine) or drugs whose primary or secondary toxicity is myelosuppression [see Drug Interactions (7.1, 7.3 and 7.4)].<br><br>**12 CLINICAL PHARMACOLOGY**<br>**12.5 Pharmacogenomics**<br>Several published studies indicate that patients with reduced TPMT or NUDT15 activity receiving usual doses of mercaptopurine, accumulate excessive cellular concentrations of active 6-TGNs, and are at higher risk for severe myelosuppression [see Warnings and Precautions (5.1)]. In a study of 1028 children with ALL, the approximate tolerated mercaptopurine dosage range for patients with TPMT and/or NUDT15 deficiency on mercaptopurine maintenance therapy (as a percentage of the planned dosage) was as follows: heterozygous for either TPMT or NUDT15, 50-90%; heterozygous for both TPMT and NUDT15, 30-50%; homozygous for either TPMT or NUDT15, 5-10%. Approximately 0.3% (1:300) of patients of European or African ancestry have two loss-of-function alleles of the TPMT gene and have little or no TMPT activity (homozygous deficient or poor metabolizers), and approximately 10% of patients have one loss-of-function TPMT allele leading to intermediate TPMT activity (heterozygous deficient or intermediate metabolizers). The TPMT*2, TPMT*3A, and TPMT*3C alleles account for about 95% of individuals with reduced levels of TPMT activity. NUDT15 deficiency is detected in <1% of patients of European or African ancestry. Among patients of East Asian ancestry (i.e., Chinese, Japanese, Vietnamese), 2% have two loss-of-function alleles of the NUDT15 gene, and approximately 21% have one loss-of-function allele. The p.R139C variant of NUDT15 (present on the *2 and *3 alleles) is the most commonly observed, but other less common loss-of-function NUDT15 alleles have been observed.<br>Consider all clinical information when interpreting results from phenotypic testing used to determine the level of thiopurine nucleotides or TPMT activity in erythrocytes, since some coadministered drugs can influence measurement of TPMT activity in blood, and blood from recent transfusions will misrepresent a patient's actual TPMT activity [see Dosage and Administration (2.2) and Warnings and Precautions (5.1)].
**204630, 05/21/2018**	**Methylene Blue**	Hematology	G6PD	Contraindications, Warnings and Precautions	**4 CONTRAINDICATIONS**<br>PROVAYBLUE™ is contraindicated in the following conditions:<br>• Severe hypersensitivity reactions to methylene blue or any other thiazine dye [see Warnings and Precautions (5.2)].<br>• Patients with glucose-6-phosphate dehydrogenase deficiency (G6PD) due to the risk of hemolytic anemia [see Warnings and Precautions (5.3, 5.4)]<br><br>**5 WARNINGS AND PRECAUTIONS**<br>**5.3 Lack of Effectiveness**<br>Methemoglobinemia may not resolve or may rebound after response to treatment with PROVAYBLUE™ in patients with methemoglobinemia due to aryl amines such as aniline or sulfa drugs such as dapsone. Monitor response to therapy with PROVAYBLUE™ through resolution of methemoglobinemia. If methemoglobinemia does not respond to 2 doses of PROVAYBLUE™ or if methemoglobinemia rebounds after a response, consider additional treatment options [see Dosage and Administration (2.2)].<br>Patients with glucose-6-phosphate dehydrogenase deficiency may not reduce PROVAYBLUE™ to its active form in vivo. PROVAYBLUE™ may not be effective in patients with glucose-6-phosphate dehydrogenase (G6PD) deficiency.<br>**5.4 Hemolytic Anemia**<br>Hemolysis can occur during treatment of methemoglobinemia with PROVAYBLUE™. Laboratory testing may show Heinz bodies, elevated indirect bilirubin and low haptoglobin, but the Coombs test is negative. The onset of anemia may be delayed 1 or more days after treatment with PROVAYBLUE™. The anemia may require red blood cell transfusions. [see Adverse Reactions (6.1)]. Use the lowest effective number of doses of PROVAYBLUE™ to treat methemoglobinemia. Discontinue PROVAYBLUE™ and consider alternative treatments of methemoglobinemia if severe hemolysis occurs.<br>Treatment of patients with glucose-6-phosphate dehydrogenase (G6PD) deficiency with PROVAYBLUE™ may result in severe hemolysis and severe anemia. PROVAYBLUE™ is contraindicated for use in patients with glucose-6-phosphate dehydrogenase (G6PD) deficiency [see Contraindications (4)].
**017854, 08/29/2017**	**Metoclopramide (1)**	Gastroenterology	CYB5R	Use in Specific Populations	**8 USE IN SPECIFIC POPULATIONS**<br>**8.8 NADH-Cytochrome b5 Reductase Deficiency**<br>Metoclopramide-treated patients with NADH-cytochrome b5 reductase deficiency are at an increased risk of developing methemoglobinemia and/or sulfhemoglobinemia. For patients with glucose-6-phosphate dehydrogenase (G6PD) deficiency with metoclopramide-induced methemoglobinemia, methylene blue treatment is not recommended. Methylene blue may cause hemolytic anemia in patients with G6PD deficiency, which may be fatal [see Overdosage (10)].
**017854, 08/29/2017**	**Metoclopramide (2)**	Gastroenterology	G6PD	Use in Specific Populations, Overdosage	**8 USE IN SPECIFIC POPULATIONS**<br>**8.8 NADH-Cytochrome b5 Reductase Deficiency**<br>Metoclopramide-treated patients with NADH-cytochrome b5 reductase deficiency are at an increased risk of developing methemoglobinemia and/or sulfhemoglobinemia. For patients with glucose-6-phosphate dehydrogenase (G6PD) deficiency with metoclopramide-induced methemoglobinemia, methylene blue treatment is not recommended. Methylene blue may cause hemolytic anemia in patients with G6PD deficiency, which may be fatal [see Overdosage (10)].<br><br>**10 OVERDOSAGE**<br>(…) Methemoglobinemia can be reversed by the intravenous administration of methylene blue. However, methylene blue may cause hemolytic anemia in patients with glucose-6-phosphate dehydrogenase (G6PD) deficiency, which may be fatal. (…)
**017854, 08/29/2017**	**Metoclopramide (3)**	Gastroenterology	CYP2D6	Dosage and Administration, Use in Specific Populations,	**2 DOSAGE AND ADMINISTRATION**<br>**2.2 Dosage for Gastroesophageal Reflux**<br>Reglan tablets may be administered continuously or intermittently in patients with symptomatic gastroesophageal reflux who fail to respond to conventional therapy:

* Therapeutic areas do not necessarily reflect the CDER review division.
† Representative biomarkers are listed based on standard nomenclature as per the Human Genome Organization (HUGO) symbol and/or simplified descriptors using other common conventions. Listed biomarkers do not necessarily reflect the terminology used in labeling. The term "Nonspecific" is provided when labeling does not explicitly identify the specific biomarker(s) or when the biomarker is represented by a molecular phenotype or gene signature, and in some cases the biomarker was inferred based on the labeling language.
‡ Referenced figures and tables may be found in the labeling available at Drugs@FDA.
Blue text represents the most recent addtions and/or changes since last posted version.

Table of Pharmacogenomic Biomarkers in Drug Labeling
Last Updated: 06/2024

NDA/ANDA/BLA Number, Label Version Date	Drug	Therapeutic Area*	Biomarker†	Labeling Sections	Labeling Text‡
				Clinical Pharmacology	<u>Continuous Dosing</u><br>The recommended adult dosage of Reglan is 10 to 15 mg four times daily for 4 to 12 weeks. The treatment duration is determined by endoscopic response. Administer the dosage thirty minutes before each meal and at bedtime. The maximum recommended daily dosage is 60 mg.<br>Table 1 displays the recommended daily dosage and maximum daily dosage for adults and dosage adjustments for patients with moderate or severe hepatic impairment (Child-Pugh B or C), in patients with creatinine clearance less than 60 mL/minute, in cytochrome P450 2D6 (CYP2D6) poor metabolizers, and with concomitant use with strong CYP2D6 inhibitors. (See Table 1)<br>**2.3 Dosage for Acute and Recurrent Diabetic Gastroparesis**<br>The recommended adult dosage for the treatment of acute and recurrent diabetic gastroparesis is 10 mg four times daily for 2 to 8 weeks, depending on symptomatic response. Avoid Reglan treatment for greater than 12 weeks [see Warnings and Precautions (5.1)]. Administer the dosage thirty minutes before each meal and at bedtime. The maximum recommended daily dosage is 40 mg.<br>Table 2 displays the recommended daily dosage and maximum daily dosage for adults and dosage adjustments for patients with moderate or severe hepatic impairment (Child-Pugh B or C), in patients with creatinine clearance less than 60 mL/minute, in cytochrome P450 2D6 (CYP2D6) poor metabolizers, and with concomitant use with strong CYP2D6 inhibitors. (See Table 2)<br><br>**8 USE IN SPECIFIC POPULATIONS**<br>**8.9 CYP2D6 Poor Metabolizers**<br>Metoclopramide is a substrate of CYP2D6. The elimination of metoclopramide may be slowed in patients who are CYP2D6 poor metabolizers (compared to patients who are CYP2D6 intermediate, extensive, or ultra-rapid metabolizers); possibly increasing the risk of dystonic and other adverse reactions to Reglan [see Clinical Pharmacology (12.3)]. Reduce the Reglan dosage in patients who are poor CYP2D6 metabolizers [see Dosage and Administration (2.2, 2.3)].<br><br>**12 CLINICAL PHARMACOLOGY**<br>**12.3 Pharmacokinetics**<br><u>Elimination</u><br>Metabolism: Metoclopramide undergoes enzymatic metabolism via oxidation as well as glucuronide and sulfate conjugation reactions in the liver. Monodeethylmetoclopramide, a major oxidative metabolite, is formed primarily by CYP2D6, an enzyme subject to genetic variability [see Dosage and Administration (2.2, 2.3), Use in Specific Populations (8.9)].
**019962, 06/01/2022**	**Metoprolol**	Cardiology	CYP2D6	Clinical Pharmacology	**12 CLINICAL PHARMACOLOGY**<br>**12.3 Pharmacokinetics**<br>*Drug Interactions CYP2D6*<br>Metoprolol is metabolized predominantly by CYP2D6. In healthy subjects with CYP2D6 extensive metabolizer phenotype, coadministration of quinidine 100 mg, a potent CYP2D6 inhibitor, and immediate-release metoprolol 200 mg tripled the concentration of S-metoprolol and doubled the metoprolol elimination half-life. In four patients with cardiovascular disease, coadministration of propafenone 150 mg t.i.d. with immediaterelease metoprolol 50 mg t.i.d. resulted in steady-state concentration of metoprolol 2- to 5-fold what is seen with metoprolol alone. Extensive metabolizers who concomitantly use CYP2D6 inhibiting drugs will have increased (several-fold) metoprolol blood levels, decreasing metoprolol's cardioselectivity [see Drug Interactions (7.2)].<br>**12.5 Pharmacogenomics**<br>CYP2D6 is absent in about 8% of Caucasians (poor metabolizers) and about 2% of most other populations. CYP2D6 can be inhibited by several drugs. Poor metabolizers of CYP2D6 will have increased (several-fold) metoprolol blood levels, decreasing metoprolol's cardioselectivity.
**125390, 02/28/2022**	**Metreleptin**	Endocrinology	LEP	Contraindications	**4 CONTRAINDICATIONS**<br>**4.1 General Obesity**<br>MYALEPT is contraindicated in patients with general obesity not associated with congenital leptin deficiency. MYALEPT has not been shown to be effective in treating general obesity, and the development of anti-metreleptin antibodies with neutralizing activity has been reported in obese patients treated with MYALEPT [see Warnings and Precautions (5.1)].
**207997, 03/04/2020**	**Midostaurin (1)**	Oncology	FLT3	Indications and Usage, Dosage and Administration, Adverse Reactions, Clinical Studies	**1 INDICATIONS AND USAGE**<br>**1.1 Acute Myeloid Leukemia**<br>RYDAPT is indicated, in combination with standard cytarabine and daunorubicin induction and cytarabine consolidation chemotherapy, for the treatment of adult patients with newly diagnosed acute myeloid leukemia (AML) who are FLT3 mutation-positive, as detected by a FDA approved test [see Dosage and Administration (2.1), Clinical Studies (14.1)]. (…)<br><br>**2 DOSAGE AND ADMINISTRATION**<br>**2.1 Patient Selection**<br>Select patients for the treatment of AML with RYDAPT based on the presence of FLT3 mutation positivity [see Clinical Studies (14)]. Information on FDA-approved tests for the detection of FLT3 mutation in AML is available at: <u>http://www.fda.gov/CompanionDiagnostics</u>.<br><br>**6 ADVERSE REACTIONS**<br>**6.1 Clinical Trials Experience**<br>*Acute Myeloid Leukemia*<br>The safety evaluation of RYDAPT (50 mg twice daily with food) in patients with newly diagnosed FLT3 mutated AML is based on a randomized, double-blind, trial of RYDAPT (n=345) or placebo (n=335) with chemotherapy [see Clinical Studies (14.1)] (…)

* Therapeutic areas do not necessarily reflect the CDER review division.
† Representative biomarkers are listed based on standard nomenclature as per the Human Genome Organization (HUGO) symbol and/or simplified descriptors using other common conventions. Listed biomarkers do not necessarily reflect the terminology used in labeling. The term “Nonspecific” is provided when labeling does not explicitly identify the specific biomarker(s) or when the biomarker is represented by a molecular phenotype or gene signature, and in some cases the biomarker was inferred based on the labeling language.
‡ Referenced figures and tables may be found in the labeling available at Drugs@FDA.
Blue text represents the most recent addtions and/or changes since last posted version.

NDA/ANDA/BLA Number, Label Version Date	Drug	Therapeutic Area*	Biomarker†	Labeling Sections	Labeling Text‡
					(…) Table 2 presents the frequency category of adverse reactions reported in the randomized trial in patients with newly diagnosed FLT3 mutated AML. Adverse reactions are listed according to body system. Within each body system, the adverse reactions are ranked by frequency, with the most frequent reactions first. Table 3 presents the key laboratory abnormalities from the same randomized trial in patients with newly diagnosed FLT3 mutated AML. (See Table 2) (…)<br><br>**14 CLINICAL STUDIES**<br>**14.1 Acute Myeloid Leukemia**<br>*Study 1*<br>RYDAPT in combination with chemotherapy was investigated in a randomized, double-blind placebo-controlled trial of 717 patients with newly-diagnosed FLT3-mutated AML. In this study, FLT3 mutation status was determined prospectively with a clinical trial assay and verified retrospectively using the companion diagnostic LeukoStrat® CDx FLT3 Mutation Assay, which is an FDA-approved test for selection of patients with AML for RYDAPT treatment.<br>Patients were stratified by FLT3 mutation status: TKD, ITD with allelic ratio less than 0.7, and ITD with allelic ratio greater than or equal to 0.7. (…)<br>(…) The randomized patients had a median age of 47 years (range, 18-60 years), 44% were male, and 88% had a performance status of 0-1. AML was de novo onset in 95%. The percentage of patients with FLT3-ITD allelic ratio < 0.7, FLT3-ITD allelic ratio ≥ 0.7, and FLT3-TKD mutations were identical (per randomized FLT3 stratum) on both arms (48%, 30%, and 23%, respectively). (…).
**207997, 03/04/2020**	**Midostaurin (2)**	Oncology	NPM1	Clinical Studies	**14 CLINICAL STUDIES**<br>**14.1 Acute Myeloid Leukemia**<br>*Study 1*<br>(…) Of the 563 patients with NPM1 testing, 58% had an NPM1 mutation. The two treatment groups were generally balanced with respect to the baseline demographics and disease characteristics, except that the placebo arm had a higher percentage of females (59%) than in the midostaurin arm (52%). NPM1 mutations were identified in 55% of patients tested on the midostaurin arm and 60% of patients tested on the placebo arm. (…)
**207997, 03/04/2020**	**Midostaurin (3)**	Oncology	KIT	Clinical Studies	**14 CLINICAL STUDIES**<br>**14.2 Systemic Mastocytosis**<br>*Study 2*<br>(…) Of the 116 patients treated, a study steering committee identified 89 patients who had measurable C-findings and were evaluable for response. The median age in this group was 64 years (range: 25 to 82), 64% of patients were male, and nearly all patients (97%) were Caucasian. Among these patients, 36% had prior therapy for SM, and 82% had the KIT D816V mutation detected at baseline. Their median duration of treatment was 11 months (range: < 1 to 68 months), with treatment ongoing in 17%.<br>Efficacy was established on the basis of confirmed complete remission (CR) plus incomplete remission (ICR) by 6 cycles of RYDAPT by modified Valent criteria for ASM and SM-AHN (Table 7). Table 7 shows responses to RYDAPT according to modified Valent criteria. Confirmed major or partial responses occurred in 46 of 73 patients with a documented KIT D816V mutation, 7 of 16 with wild-type or unknown status with respect to KIT D816V mutation, and 21 of 32 having prior therapy for SM. (…)
**208623, 08/10/2018**	**Migalastat**	Inborn Errors of Metabolism	GLA	Indications and Usage, Dosage and Administration, Clinical Pharmacology, Clinical Studies	**1 INDICATIONS AND USAGE**<br>GALAFOLD™ is indicated for the treatment of adults with a confirmed diagnosis of Fabry disease and an amenable galactosidase alpha gene (GLA) variant based on in vitro assay data [see Clinical Pharmacology (12.1)]. (…)<br><br>**2 DOSAGE AND ADMINISTRATION**<br>• Select adults with confirmed Fabry disease who have an amenable GLA variant for treatment with GALAFOLD [see Table 2 in Clinical Pharmacology (12.1)].<br>• Treatment is indicated for patients with an amenable GLA variant that is interpreted by a clinical genetics professional as causing Fabry disease (pathogenic, likely pathogenic) in the clinical context of the patient. Consultation with a clinical genetics professional is strongly recommended in cases where the amenable GLA variant is of uncertain clinical significance (VUS, variant of uncertain significance) or may be benign (not causing Fabry disease). (…)<br><br>**12 CLINICAL PHARMACOLOGY**<br>**12.1 Mechanism of Action**<br><u>In Vitro Amenability Assay</u><br>In an in vitro assay (HEK-293 assay), Human Embryonic Kidney (HEK-293) cell lines were transfected with specific GLA variants (mutations) which produced mutant alpha-Gal A proteins. In the transfected cells, amenability of the GLA variants was assessed after a 5-day incubation with 10 micromol/L migalastat. A GLA variant was categorized as amenable if the resultant mutant alpha-Gal A activity (measured in the cell lysates) met two criteria: 1) it showed a relative increase of at least 20% compared to the pre-treatment alpha-Gal A activity, and 2) it showed an absolute increase of at least 3% of the wild-type (normal) alpha-Gal A activity.<br>The in vitro assay did not evaluate trafficking of the mutant alpha-Gal A proteins into the lysosome or the dissociation of migalastat from the mutant alpha-Gal A proteins within the lysosome. Also, the in vitro assay did not test whether a GLA variant causes Fabry disease or not.<br>The GLA variants which are amenable to treatment with GALAFOLD, based on the in vitro assay data, are shown in Table 2. Inclusion of GLA variants in this table does not reflect interpretation of their clinical significance in Fabry disease. Whether a certain amenable GLA variant in a patient with Fabry disease is disease-causing or not should be determined by the prescribing physician (in consultation with a clinical genetics professional, if needed) prior to treatment initiation. Consultation with a clinical genetics professional is strongly recommended in cases where the amenable GLA variant is of uncertain clinical significance (VUS, variant of uncertain significance) or may be benign (not causing Fabry disease). (See Table 2)<br>If a GLA variant does not appear in Table 2, it is either non-amenable (if tested) or has not been tested for in vitro amenability. For further information, please contact Amicus Medical Information at 1-877-4AMICUS or <u>medinfousa@amicusrx.com</u>.<br>**12.2 Pharmacodynamics**

* Therapeutic areas do not necessarily reflect the CDER review division.
† Representative biomarkers are listed based on standard nomenclature as per the Human Genome Organization (HUGO) symbol and/or simplified descriptors using other common conventions. Listed biomarkers do not necessarily reflect the terminology used in labeling. The term “Nonspecific” is provided when labeling does not explicitly identify the specific biomarker(s) or when the biomarker is represented by a molecular phenotype or gene signature, and in some cases the biomarker was inferred based on the labeling language.
‡ Referenced figures and tables may be found in the labeling available at Drugs@FDA.
Blue text represents the most recent addtions and/or changes since last posted version.

NDA/ANDA/BLA Number, Label Version Date	Drug	Therapeutic Area*	Biomarker†	Labeling Sections	Labeling Text‡
					In Study 1, 31 of 50 patients with amenable GLA variants (18 on GALAFOLD, 13 on placebo) had lyso-Gb3 assessments available after 6 months of treatment. (…)<br>(…) In Study 2, 46 of 56 patients with amenable GLA variants (31 on GALAFOLD, 15 on enzyme replacement therapy (ERT)) had lyso-Gb3 assessments available after 18 months of treatment. The median change from baseline to month 18 in plasma lyso-Gb3 (nmol/L) was 0.53 (range -2.27, 28.3) in patients on GALAFOLD and -0.03 (range -11.9, 2.57) in patients on ERT.<br><br>**14 CLINICAL STUDIES**<br>(…) Of the 67 enrolled patients, 50 patients (32 females, 18 males) had amenable GLA variants based on the in vitro amenability assay [see Clinical Pharmacology (12.1)]. (See Table 3)<br>In Study 1, patients with non-amenable GLA variants (n = 17) had no change from baseline in the average number of GL-3 inclusions per KIC after 6 months of treatment.
**202611, 04/27/2018**	**Mirabegron**	Urology	CYP2D6	Clinical Pharmacology	**12 CLINICAL PHARMACOLOGY**<br>**12.3 Pharmacokinetics**<br>*Metabolism*<br>Mirabegron is metabolized via multiple pathways involving dealkylation, oxidation, (direct) glucuronidation, and amide hydrolysis. Mirabegron is the major circulating component following a single dose of 14C-mirabegron. Two major metabolites were observed in human plasma and are phase 2 glucuronides representing 16% and 11% of total exposure, respectively. These metabolites are not pharmacologically active toward beta-3 adrenergic receptor. Although in vitro studies suggest a role for CYP2D6 and CYP3A4 in the oxidative metabolism of mirabegron, in vivo results indicate that these isozymes play a limited role in the overall elimination. In healthy subjects who are genotypically poor metabolizers of CYP2D6, mean Cmax and AUCtau were approximately 16% and 17% higher than in extensive metabolizers of CYP2D6, respectively. In vitro and ex vivo studies have shown the involvement of butylcholinesterase, uridine diphospho-glucuronosyltransferases (UGT) and possibly alcohol dehydrogenase in the metabolism of mirabegron, in addition to CYP3A4 and CYP2D6.
**761310, 03/22/2024**	**Mirvetuximab Soravtansine-gynx**	Oncology	FOLR1	Indications and Usage, Dosage and Administration, Clinical Studies	**1 INDICATIONS AND USAGE**<br>ELAHERE® is indicated for the treatment of adult patients with folate receptor-alpha (FRα) positive, platinum-resistant epithelial ovarian, fallopian tube, or primary peritoneal cancer, who have received one to three prior systemic treatment regimens. Select patients for therapy based on an FDA-approved test [see Dosage and Administration (2.1)].<br><br>**2 DOSAGE AND ADMINISTRATION**<br>**2.1 Patient Selection**<br>Select patients for the treatment of platinum-resistant epithelial ovarian, fallopian tube, or primary peritoneal cancer with ELAHERE based on the presence of FRα tumor expression [see Indications & Usage (1) and Clinical Studies (14)] using an FDA-approved test.<br>Information on FDA-approved tests for the measurement of FRα tumor expression is available at http://www.fda.gov/CompanionDiagnostics..<br><br>**14 CLINICAL STUDIES**<br><u>Study 0416</u><br>The efficacy of ELAHERE was evaluated in Study 0416 (MIRASOL, NCT04209855), a multicenter, open-label, active-controlled, randomized, two-arm, trial in patients (n=453) with FRα positive, platinum-resistant epithelial ovarian, fallopian tube, or primary peritoneal cancer. Patients were permitted to receive up to three prior lines of systemic therapy. The trial enrolled patients whose tumors were positive for FRα expression as determined by the VENTANA FOLR1 (FOLR1-2.1) RxDx Assay. Patients were excluded if they had corneal disorders, ocular conditions requiring ongoing treatment, Grade >1 peripheral neuropathy, or noninfectious interstitial lung disease.<br><u>Study 0417</u><br>The efficacy of ELAHERE was evaluated in Study 0417 (SORAYA, NCT04296890), a single-arm trial of patients with FRα positive, platinum-resistant epithelial ovarian, fallopian tube, or primary peritoneal cancer (n=106). Patients were permitted to receive up to three prior lines of systemic therapy. All patients were required to have received prior bevacizumab. The trial enrolled patients whose tumors were positive for FRα expression as determined by the VENTANA FOLR1 (FOLR1-2.1) RxDx Assay. Patients were excluded if they had corneal disorders, ocular conditions requiring ongoing treatment, Grade >1 peripheral neuropathy, or noninfectious interstitial lung disease.
**216196, 02/17/2022**	**Mitapivat**	Hematology	PKLR	Clinical Studies	**14 CLINICAL STUDIES**<br>**Patients with PK Deficiency**<br><u>Patients Not Regularly Transfused</u><br>The efficacy of PYRUKYND was evaluated in ACTIVATE, a multinational, randomized, double-blind, placebo-controlled clinical study (NCT03548220) of 80 adults with PK deficiency who were not regularly transfused, defined as having had no more than 4 transfusions in the 52-week period prior to treatment and no transfusions in the 3-month period prior to treatment. Patients were included if they had documented presence of at least 2 variant alleles in the pyruvate kinase liver and red blood cell (PKLR) gene, of which at least 1 was a missense variant, and Hb less than or equal to 10 g/dL. Patients who were homozygous for the c.1436G>A (p.R479H) variant or had 2 non-missense variants (without the presence of another missense variant) in the PKLR gene were excluded because these patients did not achieve Hb response (change from baseline in Hb ≥1.5 g/dL at >50% assessments) in the dose-ranging study. Randomization was stratified by average screening Hb (<8.5 vs ≥8.5 g/dL) and PKLR gene variant category (missense/missense vs. missense/non-missense). (…)<br>The median duration of treatment with PYRUKYND was 24.1 weeks (range 23.6 to 27.4 weeks). Overall, 30 (75%) patients were exposed to PYRUKYND for >24 weeks and <28 weeks. Among the 80 randomized patients, the median age was 33 years (range 18 to 78) and 40% were male; race was reported in 88% of patients: 75% were White, 10% Asian, 1.3% Native Hawaiian/Other Pacific Islander and 1.3% were other races. The median baseline hemoglobin was 8.5 g/dL

NDA/ANDA/BLA Number, Label Version Date	Drug	Therapeutic Area*	Biomarker†	Labeling Sections	Labeling Text‡
					(range: 6.4 to 10.2 g/dL). There were 55 patients (69%) with the missense/missense PKLR gene variant category, and 25 patients (31%) with the missense/non-missense PKLR gene variant category. There were 58 patients (73%) who had a history of splenectomy. (See Table 6) (…)<br>Patients Who Were Regularly Transfused<br>The efficacy of PYRUKYND in patients with PK deficiency who were regularly transfused was evaluated in ACTIVATE-T, a multinational single-arm clinical trial (NCT03559699) of 27 adults with PK deficiency who had a minimum of 6 transfusion episodes in the 52-week period prior to informed consent. Patients were included if they had documented presence of at least 2 variant alleles in the PKLR gene, of which at least 1 was a missense variant. Patients who were homozygous for the c.1436G>A (p.R479H) variant or had 2 non-missense variants (without the presence of another missense variant) in the PKLR gene were excluded. Following a period of dose titration up to 50 mg twice daily, patients continued on a fixed dose of PYRUKYND for 24 weeks.<br>The median duration of treatment with PYRUKYND was 40.3 weeks (range 16.3 to 46.3 weeks). Overall, 20 (74%) patients were exposed to PYRUKYND for >40 weeks and <47 weeks. The median age was 36 years (range 18 to 68) and 26% were male; race was reported in 85% of patients: 74% were White and 11% Asian. The median baseline hemoglobin was 9.1 g/dL (range: 7.4 to 10.9 g/dL). Patients had a median of 9 transfusion episodes (range: 6 to 17 episodes) in the 52 weeks before the first dose of study treatment and a median of 7 red blood cell units transfused (range: 3 to 20 units) standardized to 24 weeks. There were 20 patients (74%) with the missense/missense PKLR gene variant category, and 7 patients (26%) with the missense/non-missense PKLR gene variant category. (…)
**020098, 07/26/2018**	**Mivacurium**	Anesthesiology	BCHE	Warnings, Precautions, Clinical Pharmacology	**WARNINGS**<br>**Administration**<br>MIVACRON is metabolized by plasma cholinesterase and should be used with great caution, if at all, in patients known to be or suspected of being homozygous for the atypical plasma cholinesterase gene.<br><br>**PRECAUTIONS**<br>**Reduced Plasma Cholinesterase Activity**<br>The possibility of prolonged neuromuscular block following administration of MIVACRON must be considered in patients with reduced plasma cholinesterase (pseudocholinesterase) activity.<br>Plasma cholinesterase activity may be diminished in the presence of genetic abnormalities of plasma cholinesterase (e.g., patients heterozygous or homozygous for the atypical plasma cholinesterase gene), pregnancy, liver or kidney disease, malignant tumors, infections, burns, anemia, decompensated heart disease, peptic ulcer, or myxedema. Plasma cholinesterase activity may also be diminished by chronic administration of oral contraceptives, glucocorticoids, or certain monoamine oxidase inhibitors and by irreversible inhibitors of plasma cholinesterase (e.g., organophosphate insecticides, echothiophate, and certain antineoplastic drugs).<br>MIVACRON has been used safely in patients heterozygous for the atypical plasma cholinesterase gene. At doses of 0.1 to 0.2 mg/kg MIVACRON, the clinically effective duration of action was 8 minutes to 11 minutes longer in patients heterozygous for the atypical gene than in genotypically normal patients.<br>As with succinylcholine, patients homozygous for the atypical plasma cholinesterase gene (one in 2500 patients) are extremely sensitive to the neuromuscular blocking effect of MIVACRON. In three such adult patients, a small dose of 0.03 mg/kg (approximately the ED10-20 in genotypically normal patients) produced complete neuromuscular block for 26 to 128 minutes. Once spontaneous recovery had begun, neuromuscular block in these patients was antagonized with conventional doses of neostigmine. One adult patient, who was homozygous for the atypical plasma cholinesterase gene, received a dose of 0.18 mg/kg MIVACRON and exhibited complete neuromuscular block for about 4 hours. Response to post-tetanic stimulation was present after 4 hours, all four responses to train-of-four stimulation were present after 6 hours, and the patient was extubated after 8 hours. Reversal was not attempted in this patient.<br><br>**CLINICAL PHARMACOLOGY**<br>**Pharmacodynamics**<br>Administration of MIVACRON over 30 to 60 seconds does not alter the time to maximum neuromuscular block or the duration of action. The duration of action of MIVACRON may be prolonged in patients with reduced plasma cholinesterase (pseudocholinesterase) activity (see PRECAUTIONS - Reduced Plasma Cholinesterase Activity and CLINICAL PHARMACOLOGY - Individualization of Dosages subsection).<br>**Individualization of Dosages**<br>**Reduced Plasma Cholinesterase Activity**<br>The possibility of prolonged neuromuscular block following administration of MIVACRON must be considered in patients with reduced plasma cholinesterase (pseudocholinesterase) activity. MIVACRON should be used with great caution, if at all, in patients known or suspected of being homozygous for the atypical plasma cholinesterase gene (see WARNINGS). Doses of 0.03 mg/kg produced complete neuromuscular block for 26 to 128 minutes in three such patients; thus initial doses greater than 0.03 mg/kg are not recommended in homozygous patients. Infusions of MIVACRON are not recommended in homozygous patients.<br>MIVACRON has been used safely in patients heterozygous for the atypical plasma cholinesterase gene and in genotypically normal patients with reduced plasma cholinesterase activity. After an initial dose of 0.15 mg/kg MIVACRON, the clinically effective duration of block in heterozygous patients may be approximately 10 minutes longer than in patients with normal genotype and normal plasma cholinesterase activity. Lower infusion rates of MIVACRON are recommended in these patients (see PRECAUTIONS - Reduced Plasma Cholinesterase Activity).
**215310, 09/15/2021**	**Mobocertinib**	Oncology	EGFR	Indications and Usage, Dosage and Administration, Adverse Reactions, Clinical Studies	**1 INDICATIONS AND USAGE**<br>EXKIVITY is indicated for the treatment of adult patients with locally advanced or metastatic nonsmall cell lung cancer (NSCLC) with epidermal growth factor receptor (EGFR) exon 20 insertion mutations, as detected by an FDA-approved test [see Dosage and Administration (2.1)], whose disease has progressed on or after platinum-based chemotherapy.<br><br>**2 DOSAGE AND ADMINISTRATION**<br>**2.1 Patient Selection**

* Therapeutic areas do not necessarily reflect the CDER review division.
† Representative biomarkers are listed based on standard nomenclature as per the Human Genome Organization (HUGO) symbol and/or simplified descriptors using other common conventions. Listed biomarkers do not necessarily reflect the terminology used in labeling. The term “Nonspecific” is provided when labeling does not explicitly identify the specific biomarker(s) or when the biomarker is represented by a molecular phenotype or gene signature, and in some cases the biomarker was inferred based on the labeling language.
‡ Referenced figures and tables may be found in the labeling available at Drugs@FDA.
Blue text represents the most recent addtions and/or changes since last posted version.

NDA/ANDA/BLA Number, Label Version Date	Drug	Therapeutic Area*	Biomarker†	Labeling Sections	Labeling Text‡
					Select patients with locally advanced or metastatic NSCLC for treatment with EXKIVITY based on the presence of EGFR exon 20 insertion mutations [see Clinical Studies (14)]. Information on FDA-approved tests is available at: http://www.fda.gov/CompanionDiagnostics.<br><br>**6 ADVERSE REACTIONS**<br>**6.1 Clinical Trials Experience**<br>The pooled safety population described in WARNINGS AND PRECAUTIONS reflects exposure to EXKIVITY as a single agent at a dose of 160 mg orally once daily in 256 patients, including 114 patients with EGFR exon 20 insertion mutation-positive locally advanced or metastatic NSCLC from Study AP32788-15-101, and patients with other solid tumors. Forty-eight percent (48%) were exposed for 6 months or longer and 12% were exposed for greater than one year. The most common (>20%) adverse reactions were diarrhea, rash, nausea, stomatitis, vomiting, decreased appetite, paronychia, fatigue, dry skin, and musculoskeletal pain. The most common (≥2%) Grade 3 or 4 laboratory abnormalities were decreased lymphocytes, increased amylase, increased lipase, decreased potassium, decreased hemoglobin, increased creatinine, and decreased magnesium.<br>EGFR Exon 20 Insertion Mutation-Positive Locally Advanced or Metastatic NSCLC Previously Treated with Platinum-Based Chemotherapy<br>The safety of EXKIVITY was evaluated in a subset of patients in Study AP32788-15-101 with EGFR exon 20 insertion mutation-positive locally advanced or metastatic NSCLC who received prior platinum-based chemotherapy [see Clinical Studies (14)]. (See Tables 3 and 4) (…)<br><br>**14 CLINICAL STUDIES**<br>The efficacy of EXKIVITY was evaluated in a pooled subset of patients with EGFR exon 20 insertion mutation-positive metastatic or locally advanced NSCLC whose disease had progressed on or after platinum-based chemotherapy enrolled in an international, open-label, multicohort clinical trial (AP32788-15-101, NCT02716116). Patients had histologically or cytologically confirmed locally advanced or metastatic disease (Stage IIIB or IV) and a documented EGFR exon 20 insertion mutation based on local testing. Patients received EXKIVITY at a dose of 160 mg once daily until disease progression or intolerable toxicity. In the efficacy population, EGFR exon 20 insertion mutation status was determined by prospective local testing using samples from tumor tissue (87%), plasma (5%), or other specimens such as pleural fluid (8%). Of the 114 patients with EGFR exon 20 insertion mutations, 70% of patient tissue samples were tested retrospectively using Life Technologies Corporation Oncomine Dx™ Target Test. While 75% of patients were positive for EGFR exon 20 insertion mutation, 14% did not have an EGFR exon 20 insertion mutation identified, and 11% did not generate reportable results. (See Table 5)
**020717, 01/15/2015**	Modafinil	Psychiatry	CYP2D6	Clinical Pharmacology	**12 CLINICAL PHARMACOLOGY**<br>**12.3 Pharmacokinetics**<br>*Interactions with CNS Active Drugs*<br>CYP2C19 also provides an ancillary pathway for the metabolism of certain tricyclic antidepressants (e.g., clomipramine and desipramine) and selective serotonin reuptake inhibitors that are primarily metabolized by CYP2D6. In tricyclic-treated patients deficient in CYP2D6 (i.e., those who are poor metabolizers of debrisoquine; 7-10% of the Caucasian population; similar or lower in other populations), the amount of metabolism by CYP2C19 may be substantially increased. PROVIGIL may cause elevation of the levels of the tricyclics in this subset of patients [see Drug Interactions (7)]. (…)
**050791, 10/27/2015**	Mycophenolic Acid	Transplantation	HPRT1	Warnings and Precautions	**5 WARNINGS AND PRECAUTIONS**<br>**5.10 Rare Hereditary Deficiencies**<br>Myfortic is an inosine monophosphate dehydrogenase inhibitor (IMPDH Inhibitor). Myfortic should be avoided in patients with rare hereditary deficiency of hypoxanthine-guanine phosphoribosyl-transferase (HGPRT) such as Lesch-Nyhan and Kelley-Seegmiller syndromes because it may cause an exacerbation of disease symptoms characterized by the overproduction and accumulation of uric acid leading to symptoms associated with gout such as acute arthritis, tophi, nephrolithiasis or urolithiasis and renal disease including renal failure.
**014214, 11/28/2012**	Nalidixic Acid	Infectious Diseases	G6PD	Precautions, Adverse Reactions	**PRECAUTIONS**<br>(…) Caution should be observed in patients with glucose-6-phosphate dehydrogenase deficiency. (See ADVERSE REACTIONS) (…)<br><br>**ADVERSE REACTIONS**<br>(…) Tendon disorders including tendon rupture, cholestasis, paresthesia, metabolic acidosis, thrombocytopenia, leukopenia, or hemolytic anemia, sometimes associated with glucose 6- phosphate dehydrogenase deficiency and peripheral neuropathy. (See WARNINGS) (…)
**021204, 10/28/2021**	Nateglinide	Endocrinology	CYP2C9	Drug Interactions	**7 DRUG INTERACTIONS**<br>**Drugs That May Increase the Blood-Glucose-Lowering Effect of STARLIX and Susceptibility to Hypoglycemia**<br>*Drugs:*<br>Nonsteroidal anti-inflammatory drugs (NSAIDs), salicylates, monoamine oxidase inhibitors, non-selective beta-adrenergic-blocking agents, anabolic hormones (e.g., methandrostenolone), guanethidine, gymnema sylvestre, glucomannan, thioctic acid, and inhibitors of CYP2C9 (e.g., amiodarone, fluconazole, voriconazole, sulfinpyrazone) or in patients known to be poor metabolizers of CYP2C9 substrates, alcohol.<br>*Intervention:*<br>Dose reductions and increased frequency of glucose monitoring may be required when STARLIX is coadministered with these drugs.
**021742, 11/30/2017**	Nebivolol	Cardiology	CYP2D6	Dosage and Administration, Clinical Pharmacology	**2 DOSAGE AND ADMINISTRATION**<br>**2.2 Subpopulations**<br>*CYP2D6 Polymorphism*<br>No dose adjustments are necessary for patients who are CYP2D6 poor metabolizers. The clinical effect and safety profile observed in poor metabolizers were similar to those of extensive metabolizers [see Clinical Pharmacology (12.3)].<br><br>**12 CLINICAL PHARMACOLOGY**<br>**12.3 Pharmacokinetics**

Table of Pharmacogenomic Biomarkers in Drug Labeling
Last Updated: 06/2024

NDA/ANDA/BLA Number, Label Version Date	Drug	Therapeutic Area*	Biomarker†	Labeling Sections	Labeling Text‡
					Nebivolol is metabolized by a number of routes, including glucuronidation and hydroxylation by CYP2D6. The active isomer (d-nebivolol) has an effective half-life of about 12 hours in CYP2D6 extensive metabolizers (most people), and 19 hours in poor metabolizers and exposure to d-nebivolol is substantially increased in poor metabolizers. This has less importance than usual, however, because the metabolites, including the hydroxyl metabolite and glucuronides (the predominant circulating metabolites), contribute to β-blocking activity.<br>Plasma levels of d–nebivolol increase in proportion to dose in EMs and PMs for doses up to 20mg. Exposure to l-nebivolol is higher than to d-nebivolol but l-nebivolol contributes little to the drug's activity as d-nebivolol's beta receptor affinity is > 1000-fold higher than l-nebivolol. For the same dose, PMs attain a 5-fold higher Cmax and 10-fold higher AUC of d-nebivolol than do EMs. d-Nebivolol accumulates about 1.5-fold with repeated once-daily dosing in EMs.
**215842, 09/29/2023**	Nedosiran	Nephrology	AGXT	Indications and Usage, Adverse Reactions, Use in Specific Populations, Clinical Pharmacology, Clinical Studies	**1 INDICATIONS AND USAGE**<br>RIVFLOZA is indicated to lower urinary oxalate levels in children 9 years of age and older and adults with primary hyperoxaluria type 1 (PH1) and relatively preserved kidney function, e.g., eGFR ≥ 30 mL/min/1.73 m2 [see Clinical Pharmacology (12.3)], Clinical Studies (14.1)].<br><br>**6 ADVERSE REACTIONS**<br>**6.1 Clinical Trials Experience**<br>Because clinical trials are conducted under widely varying conditions, adverse reaction rates observed in the clinical trials of a drug cannot be directly compared to rates in the clinical trials of another drug and may not reflect the rates observed in practice.<br>The safety of RIVFLOZA has been evaluated in one placebo-controlled clinical trial (PHYOX2) and one open-label extension study (PHYOX3). Across these studies, 29 adults and 12 children with PH1 have been treated with RIVFLOZA. Patients with PH1 in these studies ranged in age from 9 to 46 years at first dose. The median duration of exposure was approximately 15 months (range 1-29 months). Overall, 38 patients with PH1 were treated for at least 6 months, 24 patients for at least 12 months, and 16 patients for at least 18 months.<br>In the randomized, placebo-controlled, double-blind PHYOX2 trial in pediatric and adult patients 9 to 46 years of age, 18 patients with PH1 received RIVFLOZA and 11 patients received placebo. Of the 18 patients treated with RIVFLOZA, 17 patients received ≥ 5 months of active treatment. The most common adverse reaction was injection site reactions, which were reported in 7 patients with PH1 (39%) on RIVFLOZA as compared to no patients on placebo.<br>Injection site reactions included erythema, pain, bruising, and rash and were generally mild and did not lead to discontinuation of treatment. In the single-arm extension study (PHYOX3) that included 40 patients with PH1, additional injection site reactions included atrophy in 1 patient (3%).<br><br>**8 USE IN SPECIFIC POPULATIONS**<br>**8.7 Renal Impairment**<br>No dose adjustment is recommended in patients with an estimated glomerular filtration rate (eGFR) of ≥ 30 mL/min/1.73 m2 [see Clinical Pharmacology (12.3)]. RIVFLOZA has not been studied in PH1 patients with severe renal impairment (eGFR < 30 mL/min/1.73 m2).<br><br>**12 CLINICAL PHARMACOLOGY**<br>**12.2 Pharmacodynamics**<br>The pharmacodynamic effects of RIVFLOZA were evaluated after single-dose and monthly-dose administration in patients with PH1. Dose-dependent reductions in urinary oxalate were observed in the single-dose range of 1.5 mg/kg to 6.0 mg/kg. With the recommended monthly dose regimen of RIVFLOZA, onset of effect was observed at the first measurement (30 days after the first dose) and the effect persisted with continued monthly dosing [see Clinical Studies (14.1)]. (…)<br>**12.3 Pharmacokinetics**<br>The pharmacokinetic (PK) properties of RIVFLOZA were evaluated following administration of single and multiple dosages in patients with PH1 or PH2 as summarized in Table 2.<br>**12.6 Immunogenicity**<br>Across all clinical studies in the nedosiran development program, including patients with PH1 dosed with RIVFLOZA, RIVFLOZA did not induce or boost anti-drug antibodies (ADA). Among 59 patients tested with the ADA assay, none developed treatment-emergent ADA.<br><br>**14 CLINICAL STUDIES**<br>**14.1 PHYOX2**<br>PHYOX2 was a randomized, double-blind trial comparing RIVFLOZA and placebo in patients aged 6 years or older with PH1 or PH2 and an eGFR ≥ 30 mL/min/1.73 m2 (NCT03847909). Too few PH2 patients were enrolled to evaluate efficacy in the PH2 population. Therefore, RIVFLOZA is only indicated for patients with PH1 [see Indications and Usage (1)]. Unless otherwise noted, data are presented for the complete study population (PH1 and PH2).<br>Patients received monthly doses of RIVFLOZA (N=23) or placebo (N=12). The RIVFLOZA dose for patients at least 12 years of age weighing at least 50 kg was 160 mg, for patients at least 12 years of age weighing less than 50 kg was 128 mg, and for children 6 to 11 years of age was 3.3 mg/kg (to a maximum of 128 mg).<br>The median age was 20 years (range 9 - 46 years), 51% were female, 71% were White, 17% were Asian, 83% had PH1, and 17% had PH2. At baseline, mean 24-hour urinary oxalate excretion, normalized by 1.73 m2 BSA in patients less than 18 years of age, was 1547 µmol/24-hour. Mean plasma oxalate was 8.2 µmol/L, 43% of patients had an eGFR ≥ 90 mL/min/1.73 m2, 34% had an eGFR 60 to < 90 mL/min/1.73 m2 , 23% had an eGFR 30 to < 60 mL/min/1.73 m2 , and 60% were taking pyridoxine.<br>The primary efficacy endpoint was the area under the curve, from Days 90 to 180, of the percent change from baseline in 24-hour urinary oxalate excretion (AUC24-hour Uox). The least-squares (LS) mean AUC24-hour Uox was -3486 (95% CI: -5025, -1947) in the RIVFLOZA group compared to 1490 (95% CI: 781, 3761) in the placebo group, for a between group difference of 4976 (95% CI: 2803, 7149; p<0.0001).<br>The LS mean percent change from baseline in 24-hour urinary oxalate excretion (corrected for

* Therapeutic areas do not necessarily reflect the CDER review division.
† Representative biomarkers are listed based on standard nomenclature as per the Human Genome Organization (HUGO) symbol and/or simplified descriptors using other common conventions. Listed biomarkers do not necessarily reflect the terminology used in labeling. The term "Nonspecific" is provided when labeling does not explicitly identify the specific biomarker(s) or when the biomarker is represented by a molecular phenotype or gene signature, and in some cases the biomarker was inferred based on the labeling language.
‡ Referenced figures and tables may be found in the labeling available at Drugs@FDA.
Blue text represents the most recent addtions and/or changes since last posted version.

NDA/ANDA/BLA Number, Label Version Date	Drug	Therapeutic Area*	Biomarker†	Labeling Sections	Labeling Text‡
					BSA in patients <18 years of age) averaged over Days 90, 120, 150 and 180, was -37% (95% CI: -53%, -21%) in the RIVFLOZA group and 12% (95% CI: -12%, 36%) in the placebo group, for a between group difference of 49% (95% CI: 26%, 72%) [Figure 1]. Among patients with PH1, the between group difference was 56% (95% CI: 33%, 80%). (see Figure 1).<br>After 6 months of treatment in PHYOX2, patients could enroll in an ongoing single-arm extension study, PHYOX3 (NCT04042402), in which all patients were treated with RIVFLOZA. The reduction in urinary oxalate was maintained in the 13 patients with PH1 who received an additional 6 months of treatment in PHYOX3.
**076037, 07/17/2014**	**Nefazodone**	Psychiatry	CYP2D6	Precautions	**PRECAUTIONS**<br>*Cardiovascular-Active Drugs*<br>*Digoxin*<br>When nefazodone (200 mg BID) and digoxin (0.2 mg QD) were coadministered for 9 days to healthy male volunteers (n = 18) who were phenotyped as CYP2D6 extensive metabolizers, Cmax, Cmin, and AUC of digoxin were increased by 29%, 27%, and 15%, respectively. Digoxin had no effects on the pharmacokinetics of nefazodone and its active metabolites. Because of the narrow therapeutic index of digoxin, caution should be exercised when nefazodone and digoxin are coadministered; plasma level monitoring for digoxin is recommended.<br>*Propranolol*<br>The coadministration of nefazodone (200 mg BID) and propranolol (40 mg BID) for 5.5 days to healthy male volunteers (n = 18), including 3 poor and 15 extensive CYP2D6 metabolizers, resulted in 30% and 14% reductions in Cmax and AUC of propranolol, respectively, and a 14% reduction in Cmax for the metabolite, 4-hydroxypropranolol. The kinetics of nefazodone, hydroxynefazodone, and triazole-dione were not affected by coadministration of propranolol. However, Cmax, Cmin, and AUC of m-chlorophenylpiperazine were increased by 23%, 54%, and 28%, respectively. No change in initial dose of either drug is necessary and dose adjustments should be made on the basis of clinical response.<br>*CYP2D6 Isozyme*<br>A subset (3% to 10%) of the population has reduced activity of the drug-metabolizing enzyme CYP2D6. Such individuals are referred to commonly as “poor metabolizers” of drugs such as debrisoquin, dextromethorphan, and the tricyclic antidepressants. The pharmacokinetics of nefazodone and its major metabolites are not altered in these “poor metabolizers.” Plasma concentrations of one minor metabolite (mCPP) are increased in this population; the adjustment of nefazodone dosage is not required when administered to “poor metabolizers.” Nefazodone and its metabolites have been shown in vitro to be extremely weak inhibitors of CYP2D6. Thus, it is not likely that nefazodone will decrease the metabolic clearance of drugs metabolized by this isozyme.
**061579**	**Neomycin**	Infectious Diseases	MT-RNR1	Warnings	Labeling not electronically available on Drugs@FDA.
**208051, 02/25/2020**	**Neratinib (1)**	Oncology	ERBB2 (HER2)	Indications and Usage, Adverse Reactions, Clinical Studies	**1 INDICATIONS AND USAGE**<br>**1.1 Extended Adjuvant Treatment of Early-Stage Breast Cancer**<br>NERLYNX as a single agent is indicated for the extended adjuvant treatment of adult patients with early-stage human epidermal growth factor receptor 2 (HER2)-positive breast cancer, to follow adjuvant trastuzumab based therapy [see Clinical Studies (14.1)].<br>**1.2 Advanced or Metastatic Breast Cancer**<br>NERLYNX in combination with capecitabine is indicated for the treatment of adult patients with advanced or metastatic HER2-positive breast cancer who have received two or more prior anti-HER2 based regimens in the metastatic setting [see Clinical Studies (14.2)].<br><br>**6 ADVERSE REACTIONS**<br>**6.1 Clinical Trials Experience***Extended Adjuvant Treatment of Early Stage Breast Cancer*<br>ExteNET<br>The data described below reflect exposure of NERLYNX as a single agent in ExteNET, a multicenter, randomized, double-blind, placebo-controlled study of NERLYNX within 2 years after completion of adjuvant treatment with trastuzumab-based therapy in women with HER2- positive early-stage breast cancer. (…)<br>*Advanced or Metastatic Breast Cancer*<br>NALA<br>The data described below reflect the safety data of NERLYNX plus capecitabine in NALA, a randomized, multicenter, multinational, open-label, active-controlled study of HER2+ metastatic breast cancer in patients, with or without brain metastases, who have received two or more prior anti HER2-based regimens in the metastatic setting. (…)<br>CONTROL<br>The CONTROL (NCT02400476) study was a multicenter, open-label, multi-cohort trial evaluating patients with early stage HER2-positive breast cancer treated with neratinib 240 mg daily for up to one year receiving loperamide prophylaxis with and without an additional anti-diarrheal treatment. (…)

* Therapeutic areas do not necessarily reflect the CDER review division.
† Representative biomarkers are listed based on standard nomenclature as per the Human Genome Organization (HUGO) symbol and/or simplified descriptors using other common conventions. Listed biomarkers do not necessarily reflect the terminology used in labeling. The term “Nonspecific” is provided when labeling does not explicitly identify the specific biomarker(s) or when the biomarker is represented by a molecular phenotype or gene signature, and in some cases the biomarker was inferred based on the labeling language.
‡ Referenced figures and tables may be found in the labeling available at Drugs@FDA.
Blue text represents the most recent addtions and/or changes since last posted version.

NDA/ANDA/BLA Number, Label Version Date	Drug	Therapeutic Area*	Biomarker†	Labeling Sections	Labeling Text‡
					**14 CLINICAL STUDIES**<br>**14.1 Extended Adjuvant Treatment of Early Stage Breast Cancer**<br>The safety and efficacy of NERLYNX were investigated in the ExteNET trial (NCT00878709), a multicenter, randomized, double-blind, placebo-controlled study of NERLYNX after adjuvant treatment with trastuzumab in women with HER2-positive breast cancer.<br>A total of 2840 patients with early-stage HER2-positive breast cancer within two years of completing treatment with adjuvant trastuzumab was randomized to receive either NERLYNX (n=1420) or placebo (n=1420). (…)<br>**14.2 Advanced or Metastatic Breast Cancer**<br>The safety and efficacy of NERLYNX in combination with capecitabine was studied in NALA (NCT01808573), a randomized, multicenter, open-label clinical trial in patients (N=621) with metastatic HER2 positive breast cancer who had received 2 or more prior anti-HER2 based regimens in the metastatic setting.<br>HER2 expression was based on archival tissue tested at a central laboratory prior to enrollment. HER2 positivity was defined as a HER2 immunohistochemistry (IHC) score of 3+ or IHC 2+ with confirmatory in situ hybridization (ISH) positive. Fifty-nine percent of these patients were hormone receptor positive (HR+) and 41% were hormone receptor negative (HR-); 69% had received two prior anti-HER2 based regimens, 31% had received three or more prior anti-HER2 based regimens, 81% had visceral disease, and 19% had non-visceralonly disease. Patients with asymptomatic or stable brain metastases were included in NALA trial (16%). (See Table 14) (…)
**208051, 02/25/2020**	Neratinib (2)	Oncology	ESR, PGR (Hormone Receptor)	Clinical Studies	**14 CLINICAL STUDIES**<br>**14.1 Extended Adjuvant Treatment of Early Stage Breast Cancer** (…) A total of 2840 patients with early-stage HER2-positive breast cancer within two years of completing treatment with adjuvant trastuzumab was randomized to receive either NERLYNX (n=1420) or placebo (n=1420). Randomization was stratified by the following factors: hormone receptor status, nodal status (0, 1-3 vs 4 or more positive nodes) and whether trastuzumab was given sequentially versus concurrently with chemotherapy. (…)<br>(…) Fifty-seven percent (57%) had hormone receptor positive disease (defined as ER-positive and/or PgR-positive), 24% were node negative, 47% had one to three positive nodes and 30% had four or more positive nodes. (See Table 12) (…)<br>HER2 expression was based on archival tissue tested at a central laboratory prior to enrollment. HER2 positivity was defined as a HER2 immunohistochemistry (IHC) score of 3+ or IHC 2+ with confirmatory in situ hybridization (ISH) positive. Fifty-nine percent of these patients were hormone receptor positive (HR+) and 41% were hormone receptor negative (HR-); 69% had received two prior anti-HER2 based regimens, 31% had received three or more prior anti-HER2 based regimens, 81% had visceral disease, and 19% had non-visceralonly disease. Patients with asymptomatic or stable brain metastases were included in NALA trial (16%). (See Table 14) (…)
**022068, 09/25/2019**	Nilotinib (1)	Oncology	BCR-ABL1 (Philadelphia chromosome)	Indications and Usage, Dosage and Administration, Warnings and Precautions, Adverse Reactions, Use in Specific Populations, Clinical Pharmacology, Clinical Studies	**1 INDICATIONS AND USAGE**<br>**1.1 Adult and Pediatric Patients with Newly Diagnosed Ph+ CML-CP**<br>Tasigna (nilotinib) is indicated for the treatment of adult and pediatric patients greater than or equal to 1 year of age with newly diagnosed Philadelphia chromosome positive chronic myeloid leukemia (Ph+ CML) in chronic phase.<br>**1.2 Adult Patients with Resistant or Intolerant Ph+ CML-CP and CML-AP**<br>Tasigna is indicated for the treatment of adult patients with chronic phase and accelerated phase Philadelphia chromosome positive chronic myelogenous leukemia (Ph+ CML) resistant or intolerant to prior therapy that included imatinib.<br>**1.3 Pediatric Patients with Resistant or Intolerant Ph+ CML-CP**<br>Tasigna is indicated for the treatment of pediatric patients greater than or equal to 1 year of age with chronic phase Philadelphia chromosome positive chronic myeloid leukemia (Ph+ CML) with resistance or intolerance to prior tyrosine-kinase inhibitor (TKI) therapy.<br><br>**2 DOSAGE AND ADMINISTRATION**<br>**2.1 Recommended Dosing**<br>*Dosage in Adult Patients with Newly Diagnosed Ph+ CML-CP*<br>The recommended dose of Tasigna is 300 mg orally twice daily.<br>*Dosage in Adult Patients with Resistant or Intolerant Ph+ CML-CP and CML-AP*<br>The recommended dose of Tasigna is 400 mg orally twice daily.<br>*Dosage in Pediatric Patients with Newly Diagnosed Ph+ CML-CP or Resistant or Intolerant Ph+ CML-CP*<br>The recommended dose of Tasigna for pediatric patients is 230 mg/m2 orally twice daily, rounded to the nearest 50 mg dose (to a maximum single dose of 400 mg) (see Table 1). If needed, attain the desired dose by combining different strengths of Tasigna capsules. Continue treatment as long as clinical benefit is observed or until unacceptable toxicity occurs.<br>**2.2 Discontinuation of treatment after a sustained molecular response (MR4.5) on Tasigna**<br>*Patient Selection*<br>*Eligibility for Discontinuation of Treatment*<br>Ph+ CML-CP patients with typical BCR-ABL transcripts who have been taking Tasigna for a minimum of 3 years and have achieved a sustained molecular response (MR4.5, corresponding to = BCR-ABL/ABL ≤ 0.0032% IS) may be eligible for treatment discontinuation [see Clinical Studies (14.3, 14.4)]. Information on FDA authorized tests for the detection and quantitation of BCR-ABL transcripts to determine eligibility for treatment discontinuation is available at http://www.fda.gov/CompanionDiagnostics.<br>Patients with typical BCR-ABL transcripts (i.e., 13a2/b2a2 or e14a2/b3a2) who achieve the sustained MR4.5 criteria are eligible for discontinuation of Tasigna treatment. Patients must continue to be monitored for possible loss of molecular remission after treatment discontinuation. Use the same FDA authorized test to consistently monitor molecular response levels while on and off treatment.<br>Consider discontinuation of treatment in patients with newly diagnosed Ph+ CML-CP who have:<br>• been treated with Tasigna for at least 3 years

NDA/ANDA/BLA Number, Label Version Date	Drug	Therapeutic Area*	Biomarker†	Labeling Sections	Labeling Text‡
					• maintained a molecular response of at least MR4.0 (corresponding to = BCR-ABL/ABL ≤ 0.01% IS) for one year prior to discontinuation of therapy<br>• achieved an MR4.5 for the last assessment taken immediately prior to discontinuation of therapy<br>• been confirmed to express the typical BCR-ABL transcripts (e13a2/b2a2 or e14a2/b3a2)<br>• no history of accelerated phase or blast crisis<br>• no history of prior attempts of treatment-free remission discontinuation that resulted in relapse.<br>Consider discontinuation of treatment in patients with Ph+ CML-CP that are resistant or intolerant to treatment with imatinib who have achieved a sustained molecular response (MR4.5) on Tasigna who have:<br>• been treated with Tasigna for a minimum of 3 years<br>• been treated with imatinib only prior to treatment with Tasigna<br>· achieved a molecular response of MR4.5 (corresponding to = BCR-ABL/ABL ≤ 0.0032% IS)<br>• sustained an MR4.5 for a minimum of one year immediately prior to discontinuation of therapy<br>• been confirmed to express the typical BCR-ABL transcripts (e13a2/b2a2 or e14a2/b3a2)<br>• no history of accelerated phase or blast crisis<br>• no history of prior attempts of treatment-free remission discontinuation that resulted in relapse.<br>Monitor BCR-ABL transcript levels and complete blood count with differential in patients who have discontinued Tasigna therapy monthly for one year, then every 6 weeks for the second year, and every 12 weeks thereafter [see Warnings and Precautions (5.16)].<br>Upon the loss of MR4.0 (corresponding to = BCR-ABL/ABL ≤ 0.01%IS) during the treatment-free phase, monitor BCR-ABL transcript levels every 2 weeks until BCR-ABL levels remain lower than major molecular response (MMR, corresponding to MR3.0 or = BCR-ABL/ABL ≤ 0.1%IS) for 4 consecutive measurements. The patient can then proceed to the original monitoring schedule.<br>**2.3 Reinitiation of treatment in patients who lose molecular response after discontinuation of therapy with Tasigna.**<br>• Newly diagnosed patients who lose MMR must reinitiate treatment within 4 weeks at the dose level prior to discontinuation of therapy [see Warnings and Precautions (5.16)]. Patients who reinitiate Tasigna therapy should have their BCR-ABL transcript levels monitored monthly until major molecular response is re-established and every 12 weeks thereafter.<br>• Patients resistant or intolerant to prior treatment that included imatinib with confirmed loss of MR4.0 (2 consecutive measures separated by at least 4 weeks showing loss of MR4.0) or loss of MMR must reinitiate treatment within 4 weeks at the dose level prior to discontinuation of therapy [see Warnings and Precautions (5.16)]. Patients who reinitiate Tasigna therapy should have their BCR-ABL transcript levels monitored monthly until previous major molecular response or MR4.0 is re-established and every 12 weeks thereafter.<br>**2.4 Dosage Modification for QT Interval Prolongation**<br>See Table 2 for dose adjustments for QT interval prolongation [see Clinical Pharmacology (12.2)]. (See Table 2) (…)<br>**2.7 Dosage Modification for Hepatic Impairment**<br>If possible, consider alternative therapies. If Tasigna must be administered to patients with hepatic impairment, consider the following dose reduction: (See Table 6) (…)<br>**2.8 Dosage Modification with Concomitant Strong CYP3A4 Inhibitors**<br>Avoid the concomitant use of strong CYP3A4 inhibitors. Should treatment with any of these agents be required, interrupt therapy with Tasigna. If patients must be coadministered a strong CYP3A4 inhibitor, reduce dosage to 300 mg once daily in patients with resistant or intolerant Ph+ CML or to 200 mg once daily in patients with newly diagnosed Ph+ CML-CP. (…)<br><br>**5 WARNINGS AND PRECAUTIONS**<br>**5.12 Hemorrhage**<br>In a randomized trial in patients with newly diagnosed Ph+ CML in chronic phase comparing Tasigna and imatinib, Grade 3 or 4 hemorrhage occurred in 1.1% of patients in the Tasigna 300 mg bid arm, in 1.8% patients in the Tasigna 400 mg bid arm, and 0.4% of patients in the imatinib arm. GI hemorrhage occurred in 2.9% and 5.1% of patients in the Tasigna 300 mg bid and 400 mg bid arms and in 1.4% of patients in the imatinib arm, respectively. Grade 3 or 4 events occurred in 0.7% and 1.4% of patients in the Tasigna 300 mg bid and 400 mg bid arms, respectively, and in no patients in the imatinib arm.<br>**5.17 Fluid Retention**<br>In the randomized trial in patients with newly diagnosed Ph+ CML in chronic phase, severe (Grade 3 or 4) fluid retention occurred in 3.9% and 2.9% of patients receiving Tasigna 300 mg bid and 400 mg bid, respectively, and in 2.5% of patients receiving imatinib. (…)<br>**5.14 Effects on Growth and Development in Pediatric Patients**<br>Growth retardation has been reported in pediatric patients with Ph+ CML in chronic phase treated with Tasigna. In a pediatric trial with 58 patients with Ph+ CML in chronic phase after a median follow-up of 33 months, 12% (n = 7) of patients experienced a decrease of two main height percentile lines (percentile lines: 5th, 10th, 25th, 50th, 75th, 90th, and 95th). Adverse reactions associated with growth retardation were reported in 3 patients (5%). Monitor growth and development in pediatric patients receiving Tasigna treatment.<br><br>**6 ADVERSE REACTIONS**<br>**6.1 Clinical Trials Experience**<br>*In Adult Patients with Newly Diagnosed Ph+ CML-CP*<br>The data below reflect exposure to Tasigna from a randomized trial in patients with newly diagnosed Ph+ CML in chronic phase treated at the recommended dose of 300 mg twice daily (n=279). The median time on treatment in the Tasigna 300 mg twice daily group was 61 months (range 0.1 to 71 months). The median actual dose intensity was 593 mg/day in the Tasigna 300 mg twice daily group. (…) (…)<br>*In Adult Patients with Resistant or Intolerant Ph+ CML-CP and CML-AP*

* Therapeutic areas do not necessarily reflect the CDER review division.
† Representative biomarkers are listed based on standard nomenclature as per the Human Genome Organization (HUGO) symbol and/or simplified descriptors using other common conventions. Listed biomarkers do not necessarily reflect the terminology used in labeling. The term "Nonspecific" is provided when labeling does not explicitly identify the specific biomarker(s) or when the biomarker is represented by a molecular phenotype or gene signature, and in some cases the biomarker was inferred based on the labeling language.
‡ Referenced figures and tables may be found in the labeling available at Drugs@FDA.
Blue text represents the most recent addtions and/or changes since last posted version.

NDA/ANDA/BLA Number, Label Version Date	Drug	Therapeutic Area*	Biomarker†	Labeling Sections	Labeling Text‡
					In the single open-label multicenter clinical trial, a total of 458 patients with Ph+ CML-CP and CML-AP resistant to or intolerant to at least one prior therapy including imatinib were treated (CML-CP=321; CMLAP=137) at the recommended dose of 400 mg twice daily. (…)<br>*Most Frequently Reported Adverse Reactions*<br>Tables 7 and 8 show the percentage of adult patients experiencing non-hematologic adverse reactions (excluding laboratory abnormalities) regardless of relationship to study drug. Adverse reactions reported in greater than 10% of adult patients who received at least 1 dose of Tasigna are listed. (See Tables 7 and 8) (…)<br>*Laboratory Abnormalities*<br>Table 9 shows the percentage of adult patients experiencing treatment-emergent Grade 3/4 laboratory abnormalities in patients who received at least one dose of Tasigna. (See Table 9) (…)<br>*Treatment discontinuation in Ph+ CML-CP patients who have achieved a sustained molecular response (MR4.5)*<br>The rate of musculoskeletal symptoms decreased in patients who entered the Tasigna treatment reinitiation (NTRI) phase, at 11/88 (12.5%) in the newly diagnosed population and 14/56 (25%) in the population previously treated with imatinib. Other adverse reactions observed in the Tasigna re-treatment phase were similar to those observed Tasigna use in patients with newly diagnosed Ph+ CML-CP and resistant or intolerant Ph+ CML-CP and CML-AP. (See Table 10) (…)<br>**Additional Data from Clinical Trials**<br>The following adverse drug reactions were reported in adult patients in the Tasigna clinical studies at the recommended doses. These adverse drug reactions are ranked under a heading of frequency, the most frequent first using the following convention: common (greater than or equal to 1% and less than 10%), uncommon (greater than or equal to 0.1% and less than 1%), and unknown frequency (single events). For laboratory abnormalities, very common events (greater than or equal to 10%), which were not included in Tables 7 and 8, are also reported. These adverse reactions are included based on clinical relevance and ranked in order of decreasing seriousness within each category, obtained from 2 clinical studies:<br>1. Adult patients with newly diagnosed Ph+ CML-CP 60 month analysis and,<br>2. Adult patients with resistant or intolerant Ph+ CML-CP and CMP-AP 24 months' analysis. (…)<br>*In Pediatric Patients with Newly Diagnosed Ph+ CML-CP or Resistant or Intolerant Ph+ CML-CP*<br>The data below reflect exposure to Tasigna from two studies in pediatric patients from 2 to less than 18 years of age with either newly diagnosed Ph+ CML-CP or imatinib/dasatinib resistant or intolerant Ph+ CML-CP treated at the recommended dose of 230 mg/m2 twice daily (n=69) [see Clinical Studies (14.5)]. The median time on treatment with Tasigna was 13.8 months (range: 0.7 to 30.9 months). The median actual dose intensity was 435.5 mg/m2 /day (range: 149 to 517 mg/m2 /day), and the median relative dose intensity was 94.7% (range: 32 to 112%). Forty patients (58.0%) had relative dose intensity superior to 90%.<br>In pediatric patients with Ph+ CML-CP, the most common (greater than 20%) non-hematologic adverse drug reactions were headache, rash, hyperbilirubinemia, alanine aminotransferase increased, pyrexia, nausea, upper respiratory tract infection, aspartate aminotransferase increased, and vomiting. The most common (greater than 5%) Grade 3/4 non-hematologic adverse drug reactions were alanine aminotransferase increased and hyperbilirubinemia. (…)<br>*Growth Retardation in Pediatric Population*<br>In a multicenter, open-label, single-arm study of 58 pediatric patients with newly diagnosed or resistant Ph+ CML-CP treated with Tasigna, with a median exposure of 33 months in each cohort, adverse reactions associated with growth and deceleration of growth in regard to height were reported in 3 patients (5%). The adverse reactions include growth retardation in 2 adolescent patients and growth hormone deficiency with body height below normal in the remaining patient (age category: child). Of the 58 pediatric patients, 12% (n = 7) experienced a decrease of two main height percentiles compared with baseline (percentile lines: 5th, 10th, 25th, 50th, 75th, 90th, and 95th). Close monitoring of growth in pediatric patients under Tasigna treatment is recommended [see Warnings and Precautions (5.14)].<br><br>**8 USE IN SPECIFIC POPULATIONS**<br>**8.4 Pediatric Use**<br>The safety and effectiveness of Tasigna have been established in pediatric patients greater than or equal to 1 year of age with newly diagnosed and resistant or intolerant Ph+ CML in chronic phase [see Clinical Studies (14.5)]. There are no data for pediatric patients under 2 years of age. Use of Tasigna in pediatric patients 1 to less than 2 years of age is supported by efficacy in pediatric patients 2 to 6 years of age.<br>Use of Tasigna in pediatric patients 1 to less than 18 years of age is supported by evidence from two clinical trials [see Clinical Studies (14.5)]. The 25 patients with newly diagnosed Ph+ CML-CP were in the following age groups: 6 children (age 2 to less than 12 years) and 19 adolescents (age 12 to less than 18 years). The 44 patients with resistant or intolerant Ph+ CML-CP included 18 children (age 2 to less than 12 years) and 26 adolescents (age 12 to less than 18 years). (…)<br>**8.5 Geriatric Use**<br>In the clinical trials of Tasigna (patients with newly diagnosed Ph+ CML-CP and resistant or intolerant Ph+ CML-CP and CML-AP), approximately 12% and 30% of patients were 65 years or over respectively.<br>• Patients with newly diagnosed Ph+ CML-CP: There was no difference in major molecular response between patients aged less than 65 years and those greater than or equal to 65 years. (…)<br><br>**12 CLINICAL PHARMACOLOGY**<br>**12.3 Pharmacokinetics**<br>Steady-state nilotinib exposure was dose-dependent with less than dose-proportional increases in systemic exposure at dose levels higher than 400 mg given as once or twice daily dosing. In adult patients with resistant or intolerant Ph+ CML given Tasigna 400 mg twice daily, the steady-state mean (%CV) Cmax and AUC0-12h were 2260 ng/ml (35%) and 18000 ng·h/ml (33%), respectively. In adult patients with newly diagnosed Ph+ CML given Tasigna 300 mg twice daily, the steady-state mean (%CV) Cmax and AUC0-12h were 1540 ng/ml (48%) and 13337 ng·h/ml (46%), respectively. (…)<br>12.3 Pharmacokinetics

* Therapeutic areas do not necessarily reflect the CDER review division.
† Representative biomarkers are listed based on standard nomenclature as per the Human Genome Organization (HUGO) symbol and/or simplified descriptors using other common conventions. Listed biomarkers do not necessarily reflect the terminology used in labeling. The term "Nonspecific" is provided when labeling does not explicitly identify the specific biomarker(s) or when the biomarker is represented by a molecular phenotype or gene signature, and in some cases the biomarker was inferred based on the labeling language.
‡ Referenced figures and tables may be found in the labeling available at Drugs@FDA.
Blue text represents the most recent addtions and/or changes since last posted version.

NDA/ANDA/BLA Number, Label Version Date	Drug	Therapeutic Area*	Biomarker†	Labeling Sections	Labeling Text‡
					Specific Populations<br>Age, sex, race/ethnicity, or body weight did not significantly affect the pharmacokinetics of nilotinib. The effect of renal impairment on nilotinib pharmacokinetics is unknown.<br>*Pediatric Patients*<br>Following administration of the approved recommend pediatric dosage of nilotinib, steady-state exposure of nilotinib were within 2-fold to adult patients treated with 400 mg twice daily. Steady-state Cmin was comparable across all age groups (pediatric patients from ages 2 to less than 18 years), diseases (patients with newly diagnosed and resistant or intolerant Ph+ CML) and studies.<br>Body surface area correlated with nilotinib clearance and was the primary factor responsible for the PK differences between pediatrics and adults.<br><br>**14 CLINICAL STUDIES**<br>**14.1 Adult Newly Diagnosed Ph+ CML-CP**<br>The ENESTnd (Evaluating Nilotinib Efficacy and Safety in clinical Trials-Newly Diagnosed patients) study (NCT00471497) was an open-label, multicenter, randomized trial conducted to determine the efficacy of Tasigna versus imatinib tablets in adult patients with cytogenetically confirmed newly diagnosed Ph+ CML-CP. Patients were within 6 months of diagnosis and were previously untreated for CML-CP, except for hydroxyurea and/or anagrelide. Efficacy was based on a total of 846 patients: 283 patients in the imatinib 400 mg once daily group, 282 patients in the Tasigna 300 mg twice daily group, 281 patients in the Tasigna 400 mg twice daily group. (…)<br>The primary efficacy endpoint was major molecular response (MMR) at 12 months after the start of study medication. MMR was defined as less than or equal to 0.1% BCR-ABL/ABL % by international scale measured by RQ-PCR, which corresponds to a greater than or equal to 3 log reduction of BCR-ABL transcript from standardized baseline. Efficacy endpoints are summarized in Table 12. (See Table 12) (…)<br>**14.2 Adult Patients with Resistant or Intolerant Ph+ CML-CP and CML-AP**<br>Study CAMN107A2101 (referred to as Study A2101) (NCT00109707) was a single-arm, open-label, multicenter study conducted to evaluate the efficacy and safety of Tasigna (400 mg twice daily) in patients with imatinib-resistant or -intolerant CML with separate cohorts for chronic and accelerated phase disease. The definition of imatinib resistance included failure to achieve a complete hematologic response (by 3 months), cytogenetic response (by 6 months) or major cytogenetic response (by 12 months) or progression of disease after a previous cytogenetic or hematologic response. Imatinib intolerance was defined as discontinuation of treatment due to toxicity and lack of a major cytogenetic response at time of study entry. At the time of data cutoff, 321 patients with CML-CP and 137 patients with CML-AP with a minimum follow-up of 24 months were enrolled. In this study, about 50% of CML-CP and CML-AP patients were males, over 90% (CML-CP) and 80% (CML-AP) were Caucasian, and approximately 30% were age 65 years or older.<br>**14.3 Treatment discontinuation in newly diagnosed Ph+ CML-CP patients who have achieved a sustained molecular response (MR4.5)**<br>The ENESTfreedom (Evaluating Nilotinib Efficacy and Safety in clinical Trials-freedom) study (NCT01784068) is an open-label, multicenter, single-arm study, where 215 adult patients with Ph+ CML-CP treated with Tasigna in first-line for ≥ 2 years who achieved MR4.5 as measured with the MolecularMD MRDx™ BCR-ABL Test were enrolled to continue Tasigna treatment for an additional 52 weeks (Tasigna consolidation phase).<br>Of the 215 patients, 190 patients (88.4%) entered the “Treatment-free Remission” (TFR) phase after achieving a sustained molecular response (MR4.5) during the consolidation phase, defined by the following criteria:<br>• The 4 last quarterly assessments (taken every 12 weeks) were at least MR4 (BCR-ABL/ABL ≤ 0.01% IS), and maintained for 1 year<br>• The last assessment being MR4.5 (BCR-ABL/ABL ≤ 0.0032% IS)<br>• No more than two assessments falling between MR4 and MR4.5 (0.0032% IS < BCR-ABL/ABL ≤ 0.01% IS).<br>The median age of patients who entered the TFR phase was 55 years, 49.5% were females, and 21.1% of the patients were ≥ 65 years of age. BCR-ABL levels were monitored every 4 weeks during the first 48 weeks of the TFR phase. Monitoring frequency was intensified to every 2 weeks upon the loss of MR4.0. Biweekly monitoring ended at one of the following time points:<br>• Loss of MMR requiring patient to reinitiate Tasigna treatment<br>• When the BCR-ABL levels returned to a range between MR4.0 and MR4.5<br>• When the BCR-ABL levels remained lower than MMR for 4 consecutive measurements (8 weeks from initial loss of MR4.0).<br>**14.4 Treatment discontinuation in Ph+ CML-CP patients who have achieved a sustained molecular response (MR4.5) on Tasigna following prior imatinib therapy**<br>The ENESTop (Evaluating Nilotinib Efficacy and Safety in clinical Trials-STop) study (NCT01698905) is an open-label, multicenter, single-arm study, where 163 adult patients with Ph+ CML-CP taking tyrosine kinase inhibitors (TKIs) for ≥ 3 years (imatinib as initial TKI therapy for more than 4 weeks without documented MR4.5 on imatinib at the time of switch to Tasigna, then switched to Tasigna for at least 2 years), and who achieved MR4.5 on Tasigna treatment as measured with the MolecularMD MRDx ™ BCR-ABL Test were enrolled to continue Tasigna treatment for an additional 52 weeks (Tasigna consolidation phase). Of the 163 patients, 126 patients (77.3%) entered the TFR phase after achieving a sustained molecular response (MR4.5) during the consolidation phase, defined by the following criterion:<br>• The 4 last quarterly assessments (taken every 12 weeks) showed no confirmed loss of MR4.5 (BCR-ABL/ABL ≤ 0.0032% IS) during 1 year.<br>(…) Patients who entered the TFR phase but experienced two consecutive measurements of BCR-ABL/ABL > 0.01% IS were considered having a confirmed loss of MR4.0, triggering reinitiation of Tasigna treatment. Patients with loss of MMR in the TFR phase immediately restarted Tasigna treatment without confirmation. All patients who restarted Tasigna therapy had BCR-ABL transcript levels monitored every 4 weeks for the first 24 weeks, then once every 12 weeks. (…)<br>**14.5 Pediatric Patients with Newly Diagnosed Ph+ CML-CP or Resistant or Intolerant Ph+ CML-CP**<br>The safety and efficacy of Tasigna in pediatric patients with Ph+ CML-CP have been investigated in two studies: Study CAMN107A2120 (NCT01077544), an open-label, single-arm, multi-center study that evaluated the pharmacokinetics, safety, and preliminary efficacy of Tasigna in pediatric patients with Ph+ CML resistant or intolerant to imatinib or dasatinib (n=11), and Study CAMN107A2203 (NCT01844765), an open-label, single-arm, multi-center study evaluating the efficacy and safety of Tasigna in pediatric patients with Ph+ CML-CP resistant or intolerant to imatinib or dasatinib (n=33) and newly diagnosed Ph+ CML-CP

* Therapeutic areas do not necessarily reflect the CDER review division.
† Representative biomarkers are listed based on standard nomenclature as per the Human Genome Organization (HUGO) symbol and/or simplified descriptors using other common conventions. Listed biomarkers do not necessarily reflect the terminology used in labeling. The term “Nonspecific” is provided when labeling does not explicitly identify the specific biomarker(s) or when the biomarker is represented by a molecular phenotype or gene signature, and in some cases the biomarker was inferred based on the labeling language.
‡ Referenced figures and tables may be found in the labeling available at Drugs@FDA.
Blue text represents the most recent addtions and/or changes since last posted version.

NDA/ANDA/BLA Number, Label Version Date	Drug	Therapeutic Area*	Biomarker†	Labeling Sections	Labeling Text‡
					(n=25). In both studies, patients received Tasigna treatment at a dose of 230 mg/m2 twice daily, rounded to the nearest 50 mg dose (to a maximum single dose of 400 mg). Data was pooled from a total of 69 pediatric patients (from 2 to less than 18 years of age) with either newly diagnosed Ph+ CML-CP (n=25; 6 children from 2 to less than 12 years and 19 adolescents from 12 to less than 18 years) or imatinib/dasatinib resistant or intolerant Ph+ CMLCP (n=44; 18 children from 2 to less than 12 years and 26 adolescents from 12 to less than 18 years).<br>The median time on treatment with Tasigna was 13.80 months (range: 0.7 to 30.9 months).<br>In patients with resistant or intolerant CML, the major molecular response (MMR; BCR-ABL/ABL ≤0.1% IS) rate was 40.9% (18/44, 95% CI: 26.3%, 56.8%) at 12 cycles (28 days per cycle). In patients with newly diagnosed CML, the MMR rate was 60.0% (15/25, 95% CI: 38.7%, 78.9%) at 12 cycles. In patients with resistant or intolerant CML, the cumulative MMR rate was 47.7% (21/44) by cycle 12. In patients with newly diagnosed CML, the cumulative MMR rate was 64.0% (16/25) by cycle 12.<br>Among the 21 patients with resistant or intolerant CML who were in MMR at any time on treatment, the median time to first MMR was 2.8 months (range: 0.0 to 11.3). For the 17 patients with newly diagnosed CML who achieved MMR, the median time to first MMR was 5.6 months (range: 2.7 to 16.6).<br>Among patients with resistant or intolerant CML, 4.5% of patients achieved BCR-ABL/ABL ≤0.0032% IS (MR4.5) by the cut-off date. Among patients with newly diagnosed CML, the percentage of patients who achieved MR4.5 was 28.0%. (See Table 13) (…)
**022068, 09/25/2019**	Nilotinib (2)	Oncology	UGT1A1	Clinical Pharmacology	**12 CLINICAL PHARMACOLOGY**<br>**12.5 Pharmacogenomics**<br>Tasigna can increase bilirubin levels. A pharmacogenetic analysis of 97 patients evaluated the polymorphisms of UGT1A1 and its potential association with hyperbilirubinemia during Tasigna treatment. In this study, the (TA)7/(TA)7 genotype was associated with a statistically significant increase in the risk of hyperbilirubinemia relative to the (TA)6/(TA)6 and (TA)6/(TA)7 genotypes. However, the largest increases in bilirubin were observed in the (TA)7/(TA)7 genotype (UGT1A1*28) patients [see Warnings and Precautions (5.6)].
**208447, 04/26/2023**	Niraparib	Oncology	BRCA (Homologous Recombination Deficiency)	Indications and Usage, Dosage and Administration, Adverse Reactions, Clinical Studies	**1 INDICATIONS AND USAGE**<br>**1.2 Maintenance Treatment of Recurrent Germline BRCA-mutated Ovarian Cancer**<br>ZEJULA is indicated for the maintenance treatment of adult patients with deleterious or suspected deleterious germline BRCA-mutated (gBRCAmut) recurrent epithelial ovarian, fallopian tube, or primary peritoneal cancer who are in a complete or partial response to platinum-based chemotherapy. Select patients for therapy based on an FDA-approved companion diagnostic for ZEJULA [see Dosage and Administration (2.1)].<br><br>**2 DOSAGE AND ADMINISTRATION**<br>**2.1 Patient Selection**<br>Maintenance Treatment of Recurrent Germline BRCA-mutated Ovarian Cancer<br>Select patients for the maintenance treatment of recurrent ovarian cancer with ZEJULA based on the presence of deleterious or suspected deleterious germline BRCA mutations [see Clinical Studies (14.2)].<br>Information on FDA-approved tests for the detection of deleterious or suspected deleterious germline BRCA mutations for this indication is available at https://www.fda.gov/companiondiagnostics.<br>**2.2 Recommended Dosage**<br>Maintenance Treatment of Recurrent Germline BRCA-mutated Ovarian Cancer<br>The recommended dosage of ZEJULA is 300 mg (three 100-mg capsules) taken orally once daily. For the maintenance treatment of recurrent ovarian cancer, patients should start treatment with ZEJULA no later than 8 weeks after their most recent platinum-containing regimen.<br><br>**6 ADVERSE REACTIONS**<br>Maintenance Treatment of Recurrent Germline BRCA-mutated Ovarian Cancer<br>The safety of monotherapy with ZEJULA 300 mg once daily has been studied in 136 patients with platinum-sensitive recurrent gBRCAmut ovarian, fallopian tube, and primary peritoneal cancer in the NOVA trial.<br>Table 8 and Table 9 summarize the common adverse reactions and abnormal laboratory findings, respectively, observed in patients treated with ZEJULA in the gBRCAmut cohort in NOVA.<br>The following adverse reactions have been identified in ≥1 to <10% of the 136 patients receiving ZEJULA in the gBRCAmut cohort of the NOVA trial and not included in the table: palpitations (9%), mucositis/stomatitis (9%), MDS/AML (7%), tachycardia (7%), and bronchitis (4%).<br><br>**14 CLINICAL STUDIES**<br>**14.1 First-Line Maintenance Treatment of Advanced Ovarian Cancer**<br>(…) Patients were randomized post completion of first-line platinum-based chemotherapy plus surgery. Randomization was stratified by best response during the front-line platinum regimen (complete response vs partial response), neoadjuvant chemotherapy (NACT) (yes vs no), and HRD status (positive vs negative or not determined). HRD status was determined using the FDA-approved Myriad myChoice CDx assay. HRD positive status included either tumor BRCA mutant (tBRCAm) or a genomic instability score (GIS) ≥ 42.<br>The major efficacy outcome measure, progression-free survival (PFS), was determined by blinded independent central review (BICR) per Response Evaluation Criteria in Solid Tumors (RECIST) version 1.1. In some cases, criteria other than RECIST, such as clinical signs and symptoms and increasing CA-125, were also applied. Overall survival was an additional efficacy outcome measure. PFS testing was performed hierarchically: first in the homologous recombination (HR)-deficient (HRD positive) population, then in the overall population. The median age of 62 ranged from 32 to 85 years among patients randomized with ZEJULA and 33 to 88 years among patients randomized with placebo.<br>PRIMA demonstrated a statistically significant improvement in PFS for patients randomized to ZEJULA as compared with placebo in the HR-deficient and overall population (Table 10, Figure 1, and Figure 2).

Table of Pharmacogenomic Biomarkers in Drug Labeling
Last Updated: 06/2024

NDA/ANDA/BLA Number, Label Version Date	Drug	Therapeutic Area*	Biomarker†	Labeling Sections	Labeling Text‡
					In exploratory subgroup analyses of patients who were administered a starting dose of ZEJULA or matched placebo based on baseline weight or platelet count, the hazard ratio for PFS was 0.39 (95% CI: 0.22, 0.72) in the HR-deficient subgroup (n = 130) and 0.68 (95% CI: 0.48, 0.97) in the overall population (n = 258). (…)<br>**14.2 Maintenance Treatment of Recurrent Germline BRCA-mutated Ovarian Cancer**<br>Randomization was stratified by time to progression after the penultimate platinum therapy (6 to <12 months and ≥12 months); use of bevacizumab in conjunction with the penultimate or last platinum regimen (yes/no); and best response during the most recent platinum regimen (complete response and partial response). Eligible patients were assigned to one of two cohorts based on the results of the BRACAnalysis CDx. Patients with deleterious or suspected deleterious germline BRCA mutations (gBRCAm) were assigned to the germline BRCA mutated (gBRCAmut) cohort (n=203), and those without germline BRCA mutations were assigned to the non-gBRCAmut cohort (n=350). (…)<br>The trial demonstrated a statistically significant improvement in PFS for patients randomized to ZEJULA as compared with placebo in the gBRCAmut cohort and the non-gBRCAmut cohort (Table 11 and Figure 3).<br>A final OS analysis was conducted after 154 events were observed. Exploratory OS results showed a HR of 0.85 (95% CI: 0.61, 1.20) in the gBRCAmut cohort with a median OS of 40.9 months (95% CI: 34.9, 52.9) for patients treated with ZEJULA and 38.1 months (95% CI: 27.6, 47.3) for patients on placebo.
**217677, 11/27/2023**	Nirogacestat (1)	Oncology	APC	Clinical Studies	**14 CLINICAL STUDIES**<br>**14.1 Desmoid Tumor**<br>(…) A total of 142 patients were randomized. The median age was 34 years (range: 18 to 76); 65% were female; race was 83% White, 6% Black, 3% Asian, and other or not reported in 8%; and 73% had an ECOG performance status (PS) of 0, 27% had an ECOG PS of 1, and 0.7% had an ECOG PS of 2. Twenty-three percent of patients had intra-abdominal disease or both intraand extra-abdominal disease, and 77% had only extra-abdominal disease. Forty-one percent of patients had multifocal disease and 59% had single focal disease. Of 105 patients with known tumor mutation status, 81% had a CTNNB1 mutation and 21% had an APC mutation. Seventeen percent of patients had a family history of familial adenomatous polyposis (FAP). (…)
**217677, 11/27/2023**	Nirogacestat (2)	Oncology	CTNNB1	Clinical Studies	**14 CLINICAL STUDIES**<br>**14.1 Desmoid Tumor**<br>(…) A total of 142 patients were randomized. The median age was 34 years (range: 18 to 76); 65% were female; race was 83% White, 6% Black, 3% Asian, and other or not reported in 8%; and 73% had an ECOG performance status (PS) of 0, 27% had an ECOG PS of 1, and 0.7% had an ECOG PS of 2. Twenty-three percent of patients had intra-abdominal disease or both intraand extra-abdominal disease, and 77% had only extra-abdominal disease. Forty-one percent of patients had multifocal disease and 59% had single focal disease. Of 105 patients with known tumor mutation status, 81% had a CTNNB1 mutation and 21% had an APC mutation. Seventeen percent of patients had a family history of familial adenomatous polyposis (FAP). (…)
**009175, 11/04/2013**	Nitrofurantoin	Infectious Diseases	G6PD	Warnings, Adverse Reactions	**WARNINGS**<br>*Hemolytic anemia*<br>Cases of hemolytic anemia of the primaquine-sensitivity type have been induced by nitrofurantoin. Hemolysis appears to be linked to a glucose-6-phosphatedehydrogenase deficiency in the red blood cells of the affected patients. This deficiency is found in 10 percent of Blacks and a small percentage of ethnic groups of Mediterranean and Near-Eastern origin. Hemolysis is an indication for discontinuing Furadantin; hemolysis ceases when the drug is withdrawn.<br><br>**ADVERSE REACTIONS**<br>*Laboratory Adverse Events*<br>The following laboratory adverse events have been reported with the use of nitrofurantoin; increased AST (SGOT), increased ALT (SGPT), decreased hemoglobin, increased serum phosphorus, eosinophilia, glucose-6-phosphate dehydrogenase deficiency anemia (see Warnings), agranulocytosis, leukopenia, granulocytopenia, hemolytic anemia, thrombocytopenia, megaloblastic anemia. In most cases, these hematologic abnormalities resolved following cessation of therapy. Aplastic anemia has been reported rarely.

* Therapeutic areas do not necessarily reflect the CDER review division.
† Representative biomarkers are listed based on standard nomenclature as per the Human Genome Organization (HUGO) symbol and/or simplified descriptors using other common conventions. Listed biomarkers do not necessarily reflect the terminology used in labeling. The term “Nonspecific” is provided when labeling does not explicitly identify the specific biomarker(s) or when the biomarker is represented by a molecular phenotype or gene signature, and in some cases the biomarker was inferred based on the labeling language.
‡ Referenced figures and tables may be found in the labeling available at Drugs@FDA.
Blue text represents the most recent addtions and/or changes since last posted version.

Table of Pharmacogenomic Biomarkers in Drug Labeling
Last Updated: 06/2024

NDA/ANDA/BLA Number, Label Version Date	Drug	Therapeutic Area*	Biomarker†	Labeling Sections	Labeling Text‡
**125554, 10/13/2023**	Nivolumab (1)	Oncology	BRAF	Adverse Reactions, Clinical Studies	**6 ADVERSE REACTIONS**<br>**6.1 Clinical Trials Experience**<br>*Unresectable or Metastatic Melanoma*<br>*Previously Treated Metastatic Melanoma*<br>(…) In CHECKMATE-037, patients had documented disease progression following treatment with ipilimumab and, if BRAF V600 mutation positive, a BRAF inhibitor. (...)<br>*Previously Untreated Metastatic Melanoma*<br>CHECKMATE-066<br>The safety of OPDIVO was also evaluated in Trial 4, a randomized, double-blind, active-controlled trial in which 411 previously untreated patients with BRAF V600 wild-type unresectable or metastatic melanoma received OPDIVO 3 mg/kg every 2 weeks (n=206) or dacarbazine 1000 mg/m2 every 3 weeks (n=205) [see Clinical Studies (14.1)]. The median duration of exposure was 6.5 months (range: 1 day to 16.6 months) in OPDIVO-treated patients. (…)<br><br>**14 CLINICAL STUDIES**<br>**14.1 Unresectable or Metastatic Melanoma**<br>*Previously Treated Metastatic Melanoma*<br>(…) Patients were required to have progression of disease on or following ipilimumab treatment and, if BRAF V600 mutation positive, a BRAF inhibitor. (…)<br>(…) Disease characteristics were M1c disease (76%), BRAF V600 mutation positive (22%), elevated LDH (56%), history of brain metastases (18%), and two or more prior systemic therapies for metastatic disease (68%). (…)<br>(…) There were objective responses in patients with and without BRAF V600 mutation-positive melanoma.<br>*Previously Untreated Metastatic Melanoma*<br>*CHECKMATE-066*<br>CHECKMATE-066 was a multicenter, double-blind, randomized (1:1) trial conducted in patients with BRAF V600 wild-type unresectable or metastatic melanoma. (…)<br>*CHECKMATE-067*<br>(…) Randomization was stratified by PD-L1 expression (≥5% vs. <5% tumor cell membrane expression) as determined by a clinical trial assay, BRAF V600 mutation status, and M stage per the American Joint Committee on Cancer (AJCC) staging system (M0, M1a, M1b vs. M1c). (…) (…) Disease characteristics were: AJCC Stage IV disease (93%); M1c disease (58%); elevated LDH (36%); history of brain metastases (4%); BRAF V600 mutation-positive melanoma (32%); PD-L1 ≥5% tumor cell membrane expression as determined by the clinical trials assay (46%); and prior adjuvant therapy (22%). (…)<br>**14.2 Adjuvant Treatment of Melanoma**<br>(…) Disease characteristics were AJCC Stage IIIB (34%), Stage IIIC (47%), Stage IV (19%), M1a-b (14%), BRAF V600 mutation positive (42%), BRAF wild-type (45%), elevated LDH (8%), PD-L1 ≥ 5% tumor cell membrane expression determined by clinical trial assay (34%), macroscopic lymph nodes (48%), and tumor ulceration (32%). (…)
**125554, 10/13/2023**	Nivolumab (2)	Oncology	CD274 (PD-L1)	Indications and Usage, Dosage and Administration, Use in Specific Populations, Clinical Pharmacology, Clinical Studies	**1 INDICATIONS AND USAGE**<br>**1.4 Metastatic Non-Small Cell Lung Cancer**<br>• OPDIVO, in combination with ipilimumab, is indicated for the first-line treatment of adult patients with metastatic non-small cell lung cancer (NSCLC) whose tumors express PD-L1 (≥1%) as determined by an FDA-approved test [see Dosage and Administration (2.1)], with no EGFR or ALK genomic tumor aberrations.<br><br>**2 DOSAGE AND ADMINISTRATION**<br>**2.1 Patient Selection**<br>Select patients with metastatic NSCLC for treatment with OPDIVO in combination with ipilimumab based on PD-L1 expression [see Clinical Studies (14.3)]. Information on FDA-approved tests for the determination of PD-L1 expression in NSCLC is available at: http://www.fda.gov/CompanionDiagnostics.<br>**2.2 Recommended Dosage**<br>The recommended dosages of OPDIVO as a single agent are presented in Table 1. (See Table 2)<br><br>**8 USE IN SPECIFIC POPULATIONS**<br>**8.5 Geriatric Use**<br>(…) Of the 576 patients randomized to OPDIVO 3 mg/kg every 2 weeks with ipilimumab 1 mg/kg every 6 weeks in CHECKMATE-227 (NSCLC), 48% were 65 years or older and 10% were 75 years or older. No overall difference in safety was reported between older patients and younger patients; however, there was a higher discontinuation rate due to adverse reactions in patients aged 75 years or older (29%) relative to all patients who received OPDIVO with ipilimumab (18%). Of the 396 patients in the primary efficacy population (PD-L1 ≥1%) randomized to OPDIVO 3 mg/kg every 2 weeks with ipilimumab 1 mg/kg every 6 weeks in CHECKMATE-227, the hazard ratio for overall survival was 0.70 (95% CI: 0.55, 0.89) in the 199 patients younger than 65 years compared to 0.91 (95% CI: 0.72, 1.15) in the 197 patients 65 years or older [see Clinical Studies (14.3)]. (…)<br><br>**12.3 Pharmacokinetics**<br>Specific Populations<br>The following factors had no clinically important effect on the clearance of nivolumab: age (29 to 87 years), weight (35 to 160 kg), sex, race, baseline LDH, PD-L1 expression, solid tumor type, tumor size, renal impairment (eGFR ≥ 15 mL/min/1.73 m2), and mild (total bilirubin [TB] less than or equal to the ULN and AST greater than ULN or TB greater than 1 to 1.5 times ULN and any AST) or moderate hepatic impairment (TB greater than 1.5 to 3 times ULN and any AST). Nivolumab has not been studied in patients with severe hepatic impairment (TB greater than 3 times ULN and any AST).

* Therapeutic areas do not necessarily reflect the CDER review division.
† Representative biomarkers are listed based on standard nomenclature as per the Human Genome Organization (HUGO) symbol and/or simplified descriptors using other common conventions. Listed biomarkers do not necessarily reflect the terminology used in labeling. The term "Nonspecific" is provided when labeling does not explicitly identify the specific biomarker(s) or when the biomarker is represented by a molecular phenotype or gene signature, and in some cases the biomarker was inferred based on the labeling language.
‡ Referenced figures and tables may be found in the labeling available at Drugs@FDA.
Blue text represents the most recent addtions and/or changes since last posted version.

NDA/ANDA/BLA Number, Label Version Date	Drug	Therapeutic Area*	Biomarker†	Labeling Sections	Labeling Text‡
					**14 CLINICAL STUDIES**<br>**14.1 Unresectable or Metastatic Melanoma**<br>*Previously Untreated Metastatic Melanoma*<br>*CHECKMATE-066*<br>(…) Randomization was stratified by PD-L1 status (greater than or equal to 5% of tumor cell membrane staining by immunohistochemistry vs. less than 5% or indeterminate result) and M stage (M0/M1a/M1b versus M1c). (…)<br>(…) Disease characteristics were M1c stage disease (61%), cutaneous melanoma (74%), mucosal melanoma 44 Reference ID: 4198384 (11%), elevated LDH level (37%), PD-L1 greater than or equal to 5% tumor cell membrane expression (35%), and history of brain metastasis (4%). (…)<br>*CHECKMATE-067*<br>(…) Randomization was stratified by PD-L1 expression (≥5% vs. <5% tumor cell membrane expression) as determined by a clinical trial assay, BRAF V600 mutation status, and M stage per the American Joint Committee on Cancer (AJCC) staging system (M0, M1a, M1b vs. M1c). (…)<br>The trial population characteristics were: median age 61 years (range: 18 to 90); 65% male; 97% White; ECOG performance score 0 (73%) or 1 (27%). Disease characteristics were: AJCC Stage IV disease (93%); M1c disease (58%); elevated LDH (36%); history of brain metastases (4%); BRAF V600 mutation-positive melanoma (32%); PD-L1 ≥5% tumor cell membrane expression as determined by the clinical trials assay (46%); and prior adjuvant therapy (22%). (…)<br>**14.2 Adjuvant Treatment of Melanoma**<br>*CHECKMATE-238*<br>*(…)* Randomization was stratified by PD-L1 status (positive [based on 5% level] vs negative/indeterminate) and American Joint Committee on Cancer (AJCC) stage (Stage IIIB/C vs Stage IV M1a-M1b vs Stage IV M1c). (…) (…) Disease characteristics were AJCC Stage IIIB (34%), Stage IIIC (47%), Stage IV (19%), M1a-b (14%), BRAF V600 mutation positive (42%), BRAF wild-type (45%), elevated LDH (8%), PD-L1 ≥ 5% tumor cell membrane expression determined by clinical trial assay (34%), macroscopic lymph nodes (48%), and tumor ulceration (32%). (…)<br>**14.3 Metastatic Non-Small Cell Lung Cancer (NSCLC)**<br>*First-line Treatment of Metastatic Non-Small Cell Lung Cancer (NSCLC) Expressing PD-L1 (≥1%): In Combination with Ipilimumab*<br>CHECKMATE-227 (NCT02477826) was a randomized, open-label, multi-part trial in patients with metastatic or recurrent NSCLC. The study included patients (18 years of age or older) with histologically confirmed Stage IV or recurrent NSCLC (per the 7th International Association for the Study of Lung Cancer [ASLC] classification), ECOG performance status 0 or 1, and no prior anticancer therapy. Patients were enrolled regardless of their tumor PD-L1 status. (…)<br>Primary efficacy results were based on Part 1a of the study, which was limited to patients with PD-L1 tumor expression ≥1%. Tumor specimens were evaluated prospectively using the PD-L1 IHC 28-8 pharmDx assay at a central laboratory. Randomization was stratified by tumor histology (non-squamous versus squamous). (…)<br>In Part 1a, a total of 793 patients were randomized to receive either OPDIVO in combination with ipilimumab (n=396) or platinum-doublet chemotherapy (n=397). The median age was 64 years (range: 26 to 87) with 49% of patients ≥65 years and 10% of patients ≥75 years, 76% White, and 65% male. Baseline ECOG performance status was 0 (34%) or 1 (65%), 50% with PD-L1 ≥50%, 29% with squamous and 71% with non-squamous histology, 10% had brain metastases, and 85% were former/current smokers.<br>The study demonstrated a statistically significant improvement in OS for PD-L1 ≥1% patients randomized to the OPDIVO and ipilimumab arm compared with the platinum-doublet chemotherapy arm. The OS results are presented in Table 35 and Figure 5. (…)<br><u>First-line Treatment of Metastatic or Recurrent NSCLC: In Combination with Ipilimumab and Platinum-Doublet Chemotherapy</u><br>CHECKMATE-9LA (NCT03215706) was a randomized, open-label trial in patients with metastatic or recurrent NSCLC. The trial included patients (18 years of age or older) with histologically confirmed Stage IV or recurrent NSCLC (per the 7th International Association for the Study of Lung Cancer classification [IASLC]), ECOG performance status 0 or 1, and no prior anticancer therapy (including EGFR and ALK inhibitors) for metastatic disease. Patients were enrolled regardless of their tumor PD-L1 status. Patients with known EGFR mutations or ALK translocations sensitive to available targeted inhibitor therapy, untreated brain metastases, carcinomatous meningitis, active autoimmune disease, or medical conditions requiring systemic immunosuppression were excluded from the study. Patients with stable brain metastases were eligible for enrollment. (…)<br>Platinum-doublet chemotherapy consisted of either carboplatin (AUC 5 or 6) and pemetrexed 500 mg/mg2, or cisplatin 75 mg/m2 and pemetrexed 500 mg/m2 for non-squamous NSCLC; or carboplatin (AUC 6) and paclitaxel 200 mg/m2 for squamous NSCLC. Patients with non-squamous NSCLC in the control arm could receive optional pemetrexed maintenance therapy. Stratification factors for randomization were tumor PD-L1 expression level (≥1% versus <1% or nonquantifiable), histology (squamous versus non-squamous), and sex (male versus female). (…)<br>A total of 719 patients were randomized to receive either OPDIVO in combination with ipilimumab and platinum-doublet chemotherapy (n=361) or platinum-doublet chemotherapy (n=358). The median age was 65 years (range: 26 to 86) with 51% of patients ≥65 years and 10% of patients ≥75 years. The majority of patients were White (89%) and male (70%). Baseline ECOG performance status was 0 (31%) or 1 (68%), 57% had tumors with PD-L1 expression ≥1% and 37% had tumors with PD-L1 expression that was <1%, 32% had tumors with squamous histology and 68% had tumors with non-squamous histology, 17% had CNS metastases, and 86% were former or current smokers. (…)<br>*Second-line Treatment of Metastatic Squamous NSCLC*<br>*CHECKMATE-017*<br>(…) This study included patients regardless of their PD-L1 status. (…)<br>(…) Archival tumor specimens were retrospectively evaluated for PD-L1 expression. Across the study population, 17% (47/272) of patients had non-quantifiable results. Among the 225 patients with quantifiable results, 47% (106/225) had PD-L1 negative squamous NSCLC, defined as <1% of tumor cells expressing PD-L1, and 53% (119/225) had PD-L1 positive squamous NSCLC, defined as ≥1% of tumor cells expressing PD-L1. In pre-specified exploratory subgroup analyses, the hazard ratios for survival were 0.58 (95% CI: 0.37, 0.92) in the PD-L1 negative subgroup and 0.69 (95% CI: 0.45, 1.05) in the PD-L1 positive NSCLC subgroup. (…)<br>*Second-line Treatment of Metastatic Non-Squamous NSCLC*

* Therapeutic areas do not necessarily reflect the CDER review division.
† Representative biomarkers are listed based on standard nomenclature as per the Human Genome Organization (HUGO) symbol and/or simplified descriptors using other common conventions. Listed biomarkers do not necessarily reflect the terminology used in labeling. The term “Nonspecific” is provided when labeling does not explicitly identify the specific biomarker(s) or when the biomarker is represented by a molecular phenotype or gene signature, and in some cases the biomarker was inferred based on the labeling language.
‡ Referenced figures and tables may be found in the labeling available at Drugs@FDA.
Blue text represents the most recent addtions and/or changes since last posted version.

NDA/ANDA/BLA Number, Label Version Date	Drug	Therapeutic Area*	Biomarker†	Labeling Sections	Labeling Text‡
					CHECKMATE-057<br>(…) The major efficacy outcome measure was OS. Additional efficacy outcome measures were investigator-assessed ORR and PFS. In addition, prespecified analyses were conducted in subgroups defined by PD-L1 expression. (…)<br>(…) Archival tumor specimens were evaluated for PD-L1 expression following completion of the trial. Across the study population, 22% (127/582) of patients had non-quantifiable results. Of the remaining 455 patients, the proportion of patients in retrospectively determined subgroups based on PD-L1 testing using the PD-L1 IHC 28-8 pharmDx assay were: 46% (209/455) PD-L1 negative, defined as <1% of tumor cells expressing PD-L1 and 54% (246/455) had PD-L1 expression, defined as ≥1% of tumor cells expressing PD-L1. Among the 246 patients with tumors expressing PD-L1, 26% (65/246) had ≥1%, but <5% tumor cells with positive staining, 7% (16/246) had ≥5% but <10% tumor cells with positive staining, and 67% (165/246) had greater than or equal to 10% tumor cells with positive staining. Figure 9 summarizes the results of prespecified analyses of survival in subgroups determined by percentage of tumor cells expressing PD-L1. Figure 10 summarizes the results of prespecified analyses of progression-free survival in subgroups determined by percentage of tumor cells expressing PD-L1. (see Figures 9 and 10) (…)<br>**14.4 Small Cell Lung Cancer**<br>CHECKMATE-032 (NCT01928394) was a multicenter, open-label, multi-cohort, ongoing trial evaluating nivolumab as a single agent or in combination with ipilimumab in patients with advanced or metastatic solid tumors. Several cohorts enrolled patients with metastatic small cell lung cancer (SCLC), regardless of PD-L1 tumor status, with disease progression after platinumbased chemotherapy to receive treatment with OPDIVO 3 mg/kg by intravenous infusion over 60 minutes every 2 weeks. (…)<br>**14.5 Advanced Renal Cell Carcinoma**<br>*Previously Treated Renal Cell Carcinoma*<br>CHECKMATE-025 (NCT01668784) was a randomized (1:1), open-label study in patients with advanced RCC who had experienced disease progression during or after one or two prior antiangiogenic therapy regimens. Patients had to have a Karnofsky Performance Score (KPS) ≥70% and patients were included regardless of their PD-L1 status. (…)<br>(…) OS benefit was observed regardless of PD-L1 expression level. (See Table 40) (…)<br>*Previously Untreated Renal Cell Carcinoma*<br>CHECKMATE-214 (NCT02231749) was a randomized (1:1), open-label study in patients with previously untreated advanced RCC. Patients were included regardless of their PD-L1 status. (…)<br>(…) OS benefit was observed regardless of PD-L1 expression level. (See Table 41) (…)<br>Two studies evaluated the efficacy of OPDIVO as a single agent in adult patients with cHL after failure of autologous HSCT.<br>*CHECKMATE-9ER*<br>CHECKMATE-9ER (NCT03141177) was a randomized, open-label study of OPDIVO combined with cabozantinib versus sunitinib in patients with previously untreated advanced RCC. CHECKMATE-9ER excluded patients with autoimmune disease or other medical conditions requiring systemic immunosuppression. Patients were stratified by IMDC prognostic score (favorable vs. intermediate vs. poor), PD-L1 tumor expression (≥1% vs. <1% or indeterminate), and region (US/Canada/Western Europe/Northern Europe vs. Rest of World). (…)<br>The major efficacy outcome measure was PFS (BICR assessed). Additional efficacy outcome measures were OS and ORR (BICR assessed). The trial demonstrated a statistically significant improvement in PFS, OS, and ORR for patients randomized to OPDIVO and cabozantinib compared with sunitinib. Consistent results for PFS were observed across pre-specified subgroups of IMDC risk categories and PD-L1 tumor expression status. Efficacy results are shown in Table 46 and Figures 13 and 14.<br>Previously Treated Renal Cell Carcinoma<br>*CHECKMATE-025*<br>CHECKMATE-025 (NCT01668784) was a randomized (1:1), open-label trial in patients with advanced RCC who had experienced disease progression during or after one or two prior antiangiogenic therapy regimens. Patients had to have a Karnofsky Performance Score (KPS) ≥70% and patients were included regardless of their PD-L1 status. (…)<br>The trial demonstrated a statistically significant improvement in OS for patients randomized to OPDIVO as compared with everolimus at the prespecified interim analysis when 398 events were observed (70% of the planned number of events for final analysis). OS benefit was observed regardless of PD-L1 expression level. Efficacy results are shown in Table 47 and Figure 15. (…)<br>**14.6 Classical Hodgkin Lymphoma**<br>CHECKMATE-205 (NCT02181738) was a single-arm, open-label, multicenter, multicohort study in cHL. CHECKMATE-039 (NCT01592370) was an open-label, multicenter, dose escalation study that included cHL. Both studies included patients regardless of their tumor PD-L1 status and excluded patients with ECOG performance status of 2 or greater, autoimmune disease, symptomatic interstitial lung disease, hepatic transaminases more than 3 times ULN, creatinine clearance less than 40 mL/min, prior allogeneic HSCT, or chest irradiation within 24 weeks. (…)<br>Two studies evaluated the efficacy of OPDIVO as a single agent in adult patients with cHL after failure of autologous HSCT.<br>CHECKMATE-205 (NCT02181738) was a single-arm, open-label, multicenter, multicohort trial in cHL. CHECKMATE-039 (NCT01592370) was an open-label, multicenter, dose escalation trial that included cHL. Both studies included patients regardless of their tumor PD-L1 status and excluded patients with ECOG performance status of 2 or greater, autoimmune disease, symptomatic interstitial lung disease, hepatic transaminases more than 3 times ULN, creatinine clearance <40 mL/min, prior allogeneic HSCT, or chest irradiation within 24 weeks. In addition, both studies required an adjusted diffusion capacity of the lungs for carbon monoxide (DLCO) of over 60% in patients with prior pulmonary toxicity. (…)<br>**14.7 Recurrent or Metastatic Squamous Cell Carcinoma of the Head and Neck**<br>*CHECKMATE-141*<br>(…) Archival tumor specimens were retrospectively evaluated for PD-L1 expression using the PD-L1 IHC 28-8 pharmDx assay. Across the study population, 28% (101/361) of patients had nonquantifiable results. Among the 260 patients with quantifiable results, 43% (111/260) had PD-L1 negative SCCHN, defined as <1% of tumor cells expressing PD-L1, and 57% (149/260) had PDL1 positive SCCHN, defined as ≥1% of tumor cells expressing PD-L1. In pre-specified

NDA/ANDA/BLA Number, Label Version Date	Drug	Therapeutic Area*	Biomarker†	Labeling Sections	Labeling Text‡
					exploratory subgroup analyses, the hazard ratio for survival was 0.89 (95% CI: 0.54, 1.45) with median survivals of 5.7 and 5.8 months for the nivolumab and chemotherapy arms, respectively, in the PD-L1 negative subgroup. The HR for survival was 0.55 (95% CI: 0.36, 0.83) with median survivals of 8.7 and 4.6 months for the nivolumab and chemotherapy arms, respectively, in the PD-L1 positive SCCHN subgroup.<br>**14.8 Urothelial Carcinoma**<br>*CHECKMATE-275*<br>(…) Patients were included regardless of their PD-L1 status.<br>Tumor specimens were evaluated prospectively using the PD-L1 IHC 28-8 pharmDx assay at a central laboratory and the results were used to define subgroups for pre-specified analyses. Of the 270 patients, 46% were defined as having PD-L1 expression of ≥1% (defined as ≥1% of tumor cells expressing PD-L1). The remaining 54% of patients, were classified as having PD-L1 expression of <1% (defined as <1% of tumor cells expressing PD-L1). Confirmed ORR in all patients and the two PD-L1 subgroups are summarized in Table 27. Median time to response was 1.9 months (range: 1.6-7.2). In 77 patients who received prior systemic therapy only in the neoadjuvant or adjuvant setting, the ORR was 23.4% (95% CI: 14.5%, 34.4%). (see Table 45)<br>**14.11 Esophageal Squamous Cell Cancer**<br>ATTRACTION-3 (NCT02569242) was a multicenter, randomized (1:1), active-controlled, open-label trial in patients with unresectable advanced, recurrent, or metastatic ESCC, who were refractory or intolerant to at least one fluoropyrimidine- and platinum-based regimen. The trial enrolled patients regardless of PD-L1 status, but tumor specimens were evaluated prospectively using the PD-L1 IHC 28-8 pharmDx assay at a central laboratory. (…)<br>Randomization was stratified by region (Japan vs. Rest of World), number of organs with metastases (≤1 vs. ≥2), and PD-L1 status (≥1% vs. <1% or indetrmine). (…)<br>ATTRACTION-3 demonstrated a statistically significant improvement in OS for patients randomized to OPDIVO as compared with investigator's choice of taxane chemotherapy. OS benefit was observed regardless of PD-L1 expression level. The minimum follow-up was 17.6 months. Efficacy results are shown in Table 48 and Figure 14.<br>Of the 419 patients, 48% had PD-L1 positive ESCC, defined as ≥1% of tumor cells expressing PD-L1. The remaining 52% had PD-L1 negative ESCC defined as <1% of tumor cells expressing PD-L1.<br>In a pre-specified exploratory analysis by PD-L1 status, the hazard ratio (HR) for OS was 0.69 (95% CI: 0.51, 0.94) with median survivals of 10.9 and 8.1 months for the OPDIVO and investigator's choice arms, respectively, in the PD-L1 positive subgroup. In the PD-L1 negative subgroup, the HR for OS was 0.84 (95% CI: 0.62, 1.14) with median survivals of 10.9 and 9.3 months for the OPDIVO and investigator's choice arms, respectively.<br>**14.12 Gastric Cancer, Gastroesophageal Junction Cancer, and Esophageal Adenocarcinoma**<br>CHECKMATE-649 (NCT02872116) was a randomized, multicenter, open-label trial in patients (n=1581) with previously untreated advanced or metastatic gastric cancer, gastroesophageal junction cancer, and esophageal adenocarcinoma. The trial enrolled patients regardless of PDL1 status, and tumor specimens were evaluated prospectively using the PD-L1 IHC 28-8 pharmDx assay at a central laboratory. (…)<br>Randomization was stratified by tumor cell PD-L1 status (≥1% vs. <1% or indeterminate), region (Asia vs. US vs. Rest of World), ECOG performance status (0 vs. 1), and chemotherapy regimen (mFOLFOX6 vs. CapeOX). The major efficacy outcome measures, assessed in patients with PD-L1 CPS ≥5, were PFS assessed by BICR and OS. Additional efficacy outcome measures included OS and PFS in patients with PD-L1 CPS ≥1 and in all randomized patients, and ORR and DOR as assessed by BICR in patients with PD-L1 CPS ≥1 and ≥5, and in all randomized patients. Tumor assessments were conducted per RECIST v1.1 every 6 weeks up to and including week 48, then every 12 weeks thereafter. (…)<br>CHECKMATE-649 demonstrated a statistically significant improvement in OS and PFS for patients with PD-L1 CPS ≥5. Statistically significant improvement in OS was also demonstrated for all randomized patients. The minimum follow-up was 12.1 months. Efficacy results are shown in Table 60 and Figures 19, 20, and 21.<br>In an exploratory analysis in patients with PD-L1 CPS<1 (n=265), the median OS was 13.1 months (95% CI: 9.8, 16.7) for the OPDIVO and chemotherapy arm and 12.5 months (95% CI: 10.1, 13.8) for the chemotherapy arm, with a stratified HR of 0.85 (95% CI: 0.63, 1.15). In an exploratory analysis in patients with PD-L1 CPS<5 (n=606), the median OS was 12.4 months (95% CI: 10.6, 14.3) for the OPDIVO and chemotherapy arm and 12.3 months (95% CI: 11.0, 13.2) for the chemotherapy arm, with a stratified HR of 0.94 (95% CI: 0.78, 1.14). (…)
125554, 10/13/2023	Nivolumab (3)	Oncology	Microsatellite Instability, Mismatch Repair	Indications and Usage, Dosage and Administration, Adverse Reactions, Use in Specific Populations, Clinical Studies	**1 INDICATIONS AND USAGE**<br>**1.9 Microsatellite Instability-High (MSI-H) or Mismatch Repair Deficient (dMMR) Metastatic Colorectal Cancer**<br>OPDIVO, as a single agent or in combination with ipilimumab, is indicated for the treatment of adult and pediatric patients 12 years and older with microsatellite instability-high (MSI-H) or mismatch repair deficient (dMMR) metastatic colorectal cancer (CRC) that has progressed following treatment with a fluoropyrimidine, oxaliplatin, and irinotecan.<br>This indication is approved under accelerated approval based on overall response rate and duration of response [see Clinical Studies (14.9)]. Continued approval for these indications may be contingent upon verification and description of clinical benefit in confirmatory trials.<br><br>**2 DOSAGE AND ADMINISTRATION**<br>**2.1 Recommended Dosage**<br>The recommended dosages of OPDIVO as a single agent are presented in Table 1. (See Tables 1 and 2) (…)<br><br>**6 ADVERSE REACTIONS**<br>**MSI-H or dMMR Metastatic Colorectal Cancer**<br>The safety of OPDIVO administered as a single agent or in combination with ipilimumab was evaluated in CHECKMATE-142, a multicenter, non-randomized, multiple parallel-cohort, openlabel study. (See Tables 26 and 27) (…)<br><br>**8 USE IN SPECIFIC POPULATIONS**<br>**8.4 Pediatric Use**

* Therapeutic areas do not necessarily reflect the CDER review division.
† Representative biomarkers are listed based on standard nomenclature as per the Human Genome Organization (HUGO) symbol and/or simplified descriptors using other common conventions. Listed biomarkers do not necessarily reflect the terminology used in labeling. The term “Nonspecific” is provided when labeling does not explicitly identify the specific biomarker(s) or when the biomarker is represented by a molecular phenotype or gene signature, and in some cases the biomarker was inferred based on the labeling language.
‡ Referenced figures and tables may be found in the labeling available at Drugs@FDA.
Blue text represents the most recent addtions and/or changes since last posted version.

Table of Pharmacogenomic Biomarkers in Drug Labeling
Last Updated: 06/2024

NDA/ANDA/BLA Number, Label Version Date	Drug	Therapeutic Area*	Biomarker†	Labeling Sections	Labeling Text‡
					The safety and effectiveness of OPDIVO have been established in pediatric patients age 12 years and older with microsatellite instability-high (MSI-H) or mismatch repair deficient (dMMR) metastatic colorectal cancer (mCRC) that has progressed following treatment with a fluoropyrimidine, oxaliplatin, and irinotecan. Use of OPDIVO for this indication is supported by evidence from adequate and well-controlled studies of OPDIVO in adults with MSI-H or dMMR mCRC with additional population pharmacokinetic data demonstrating that age and body weight had no clinically meaningful effect on the steady state exposure of nivolumab, that drug exposure is generally similar between adults and pediatric patients age 12 years and older for monoclonal antibodies, and that the course of MSI-H or dMMR mCRC is sufficiently similar in adults and pediatric patients to allow extrapolation of data in adults to pediatric patients. The recommended dose in pediatric patients 12 years of age or greater for this indication is the same as that in adults [see Dosage and Administration (2.8), Clinical Pharmacology (12.3), and Clinical Studies (14)].<br>The safety and effectiveness of OPDIVO have not been established (1) in pediatric patients less than 12 years old with MSI-H or dMMR mCRC or (2) in pediatric patients less than 18 years old for the other approved indications.<br><br>**14 CLINICAL STUDIES**<br>**14.9 Microsatellite Instability-High (MSI-H) or Mismatch Repair Deficient (dMMR) Metastatic Colorectal Cancer**<br>CHECKMATE-142 (NCT02060188) was a multicenter, open-label, single arm study conducted in patients with locally determined dMMR or MSI-H metastatic CRC who had disease progression during, after, or were intolerant to, prior treatment with fluoropyrimidine-, oxaliplatin-, or irinotecan-based chemotherapy. (…)<br>(…) Patients enrolled in the single agent OPDIVO MSI-H mCRC cohort received OPDIVO 3 mg/kg by intravenous infusion (IV) every 2 weeks. Patients enrolled in the OPDIVO plus ipilimumab MSI-H mCRC cohort received OPDIVO 3 mg/kg and ipilimumab 1 mg/kg IV every 3 weeks for 4 doses, followed by OPDIVO 3 mg/kg IV as a single agent every 2 weeks. Treatment in both cohorts continued until unacceptable toxicity or radiographic progression. (…)<br>(…) A total of 74 patients were enrolled in the single-agent MSI-H mCRC OPDIVO cohort. The median age was 53 years (range: 26 to 79) with 23% ≥65 years of age and 5% ≥75 years of age, 59% were male and 88% were White. (…)<br>(…) A total of 119 patients were enrolled in the OPDIVO plus ipilimumab MSI-H mCRC cohort. (See Table 56) (…)
**125554, 10/13/2023**	**Nivolumab (4)**	Oncology	EGFR	Indications and Usage, Adverse Reactions, Clinical Studies	**1 INDICATIONS AND USAGE**<br>**1.4 Metastatic Non-Small Cell Lung Cancer**<br>• OPDIVO, in combination with ipilimumab, is indicated for the first-line treatment of adult patients with metastatic non-small cell lung cancer (NSCLC) whose tumors express PD-L1 (≥1%) as determined by an FDA-approved test [see Dosage and Administration (2.1)], with no EGFR or ALK genomic tumor aberrations.<br>• OPDIVO, in combination with ipilimumab and 2 cycles of platinum-doublet chemotherapy, is indicated for the first-line treatment of adult patients with metastatic or recurrent non-small cell lung cancer (NSCLC), with no EGFR or ALK genomic tumor aberrations.<br>• OPDIVO is indicated for the treatment of patients with metastatic NSCLC with progression on or after platinum-based chemotherapy. Patients with EGFR or ALK genomic tumor aberrations should have disease progression on FDA-approved therapy for these aberrations prior to receiving OPDIVO.<br><br>**6 ADVERSE REACTIONS**<br>Metastatic Non-Small Cell Lung Cancer<br>*First-line Treatment of Metastatic NSCLC: In Combination with Ipilimumab*<br>The safety of OPDIVO in combination with ipilimumab was evaluated in CHECKMATE-227, a randomized, multicenter, multi-cohort, open-label trial in patients with previously untreated metastatic or recurrent NSCLC with no EGFR or ALK genomic tumor aberrations [see Clinical Studies (14.3)]. (…)<br><br>**14 CLINICAL STUDIES**<br>**14.3 Metastatic Non-Small Cell Lung Cancer**<br>First-line Treatment of Metastatic Non-Small Cell Lung Cancer (NSCLC) Expressing PD-L1 (≥1%): In Combination with Ipilimumab<br>CHECKMATE-227 (NCT02477826) was a randomized, open-label, multi-part trial in patients with metastatic or recurrent NSCLC. The study included patients (18 years of age or older) with histologically confirmed Stage IV or recurrent NSCLC (per the 7th International Association for the Study of Lung Cancer [ASLC] classification), ECOG performance status 0 or 1, and no prior anticancer therapy. Patients were enrolled regardless of their tumor PD-L1 status. Patients with known EGFR mutations or ALK translocations sensitive to available targeted inhibitor therapy, untreated brain metastases, carcinomatous meningitis, active autoimmune disease, or medical conditions requiring systemic immunosuppression were excluded from the study. (…)<br>First-line Treatment of Metastatic or Recurrent NSCLC: In Combination with Ipilimumab and Platinum-Doublet Chemotherapy<br>CHECKMATE-9LA (NCT03215706) was a randomized, open-label trial in patients with metastatic or recurrent NSCLC. The trial included patients (18 years of age or older) with histologically confirmed Stage IV or recurrent NSCLC (per the 7th International Association for the Study of Lung Cancer classification [IASLC]), ECOG performance status 0 or 1, and no prior anticancer therapy (including EGFR and ALK inhibitors) for metastatic disease. Patients were enrolled regardless of their tumor PD-L1 status. Patients with known EGFR mutations or ALK translocations sensitive to available targeted inhibitor therapy, untreated brain metastases, carcinomatous meningitis, active autoimmune disease, or medical conditions requiring systemic immunosuppression were excluded from the study. Patients with stable brain metastases were eligible for enrollment. (…)<br>Second-line Treatment of Metastatic Non-Squamous NSCLC<br>**(…)** CHECKMATE-057 (NCT01673867) was a randomized (1:1), open-label trial in 582 patients with metastatic non-squamous NSCLC who had experienced disease progression during or after one prior platinum doublet-based chemotherapy regimen. Appropriate prior targeted therapy in patients with known sensitizing EGFR mutation or ALK translocation was allowed. (…)<br>The trial population characteristics: median age was 62 years (range: 21 to 85) with 42% of patients ≥65 years and 7% of patients ≥75 years. The majority of patients were White (92%) and male (55%); the majority of patients were enrolled in Europe (46%) followed by the US/Canada (37%) and the rest of the world (17%). Baseline ECOG performance status was 0 (31%) or 1 (69%), 79% were former/current smokers, 3.6% had NSCLC with ALK rearrangement, 14% had NSCLC with EGFR mutation, and 12% had previously treated brain metastases. (…)

* Therapeutic areas do not necessarily reflect the CDER review division.
† Representative biomarkers are listed based on standard nomenclature as per the Human Genome Organization (HUGO) symbol and/or simplified descriptors using other common conventions. Listed biomarkers do not necessarily reflect the terminology used in labeling. The term “Nonspecific” is provided when labeling does not explicitly identify the specific biomarker(s) or when the biomarker is represented by a molecular phenotype or gene signature, and in some cases the biomarker was inferred based on the labeling language.
‡ Referenced figures and tables may be found in the labeling available at Drugs@FDA.
Blue text represents the most recent addtions and/or changes since last posted version.

Table of Pharmacogenomic Biomarkers in Drug Labeling
Last Updated: 06/2024

NDA/ANDA/BLA Number, Label Version Date	Drug	Therapeutic Area*	Biomarker†	Labeling Sections	Labeling Text‡
**125554, 10/13/2023**	**Nivolumab (5)**	Oncology	ALK	Indications and Usage, Adverse Reactions, Clinical Studies	**1 INDICATIONS AND USAGE**<br>**1.4 Metastatic Non-Small Cell Lung Cancer**<br>• OPDIVO, in combination with ipilimumab, is indicated for the first-line treatment of adult patients with metastatic non-small cell lung cancer (NSCLC) whose tumors express PD-L1 (≥1%) as determined by an FDA-approved test [see Dosage and Administration (2.1)], with no EGFR or ALK genomic tumor aberrations.<br>• OPDIVO, in combination with ipilimumab and 2 cycles of platinum-doublet chemotherapy, is indicated for the first-line treatment of adult patients with metastatic or recurrent non-small cell lung cancer (NSCLC), with no EGFR or ALK genomic tumor aberrations.<br>• OPDIVO is indicated for the treatment of patients with metastatic NSCLC with progression on or after platinum-based chemotherapy. Patients with EGFR or ALK genomic tumor aberrations should have disease progression on FDA-approved therapy for these aberrations prior to receiving OPDIVO.<br><br>**6 ADVERSE REACTIONS**<br>Metastatic Non-Small Cell Lung Cancer<br>*First-line Treatment of Metastatic NSCLC: In Combination with Ipilimumab*<br>The safety of OPDIVO in combination with ipilimumab was evaluated in CHECKMATE-227, a randomized, multicenter, multi-cohort, open-label trial in patients with previously untreated metastatic or recurrent NSCLC with no EGFR or ALK genomic tumor aberrations [see Clinical Studies (14.3)]. (…)<br><br>**14 CLINICAL STUDIES**<br>**14.3 Metastatic Non-Small Cell Lung Cancer**<br>First-line Treatment of Metastatic Non-Small Cell Lung Cancer (NSCLC) Expressing PD-L1 (≥1%): In Combination with Ipilimumab<br>CHECKMATE-227 (NCT02477826) was a randomized, open-label, multi-part trial in patients with metastatic or recurrent NSCLC. The study included patients (18 years of age or older) with histologically confirmed Stage IV or recurrent NSCLC (per the 7th International Association for the Study of Lung Cancer [ASLC] classification), ECOG performance status 0 or 1, and no prior anticancer therapy. Patients were enrolled regardless of their tumor PD-L1 status. Patients with known EGFR mutations or ALK translocations sensitive to available targeted inhibitor therapy, untreated brain metastases, carcinomatous meningitis, active autoimmune disease, or medical conditions requiring systemic immunosuppression were excluded from the study. (…)<br>First-line Treatment of Metastatic or Recurrent NSCLC: In Combination with Ipilimumab and Platinum-Doublet Chemotherapy<br>CHECKMATE-9LA (NCT03215706) was a randomized, open-label trial in patients with metastatic or recurrent NSCLC. The trial included patients (18 years of age or older) with histologically confirmed Stage IV or recurrent NSCLC (per the 7th International Association for the Study of Lung Cancer classification [IASLC]), ECOG performance status 0 or 1, and no prior anticancer therapy (including EGFR and ALK inhibitors) for metastatic disease. Patients were enrolled regardless of their tumor PD-L1 status. Patients with known EGFR mutations or ALK translocations sensitive to available targeted inhibitor therapy, untreated brain metastases, carcinomatous meningitis, active autoimmune disease, or medical conditions requiring systemic immunosuppression were excluded from the study. Patients with stable brain metastases were eligible for enrollment. (…)<br>Second-line Treatment of Metastatic Non-Squamous NSCLC<br>**(…)** CHECKMATE-057 (NCT01673867) was a randomized (1:1), open-label trial in 582 patients with metastatic non-squamous NSCLC who had experienced disease progression during or after one prior platinum doublet-based chemotherapy regimen. Appropriate prior targeted therapy in patients with known sensitizing EGFR mutation or ALK translocation was allowed. (…)<br>The trial population characteristics: median age was 62 years (range: 21 to 85) with 42% of patients ≥65 years and 7% of patients ≥75 years. The majority of patients were White (92%) and male (55%); the majority of patients were enrolled in Europe (46%) followed by the US/Canada (37%) and the rest of the world (17%). Baseline ECOG performance status was 0 (31%) or 1 (69%), 79% were former/current smokers, 3.6% had NSCLC with ALK rearrangement, 14% had NSCLC with EGFR mutation, and 12% had previously treated brain metastases. (…)
**125554, 10/13/2023**	**Nivolumab (6)**	Oncology	ERBB2 (HER2)	Adverse Reactions, Clinical Studies	**6 ADVERSE REACTIONS**<br>Gastric Cancer, Gastroesophageal Junction Cancer, and Esophageal Adenocarcinoma<br>The safety of OPDIVO in combination with chemotherapy was evaluated in CHECKMATE649, a randomized, multicenter, open-label trial in patients with previously untreated advanced or metastatic gastric cancer, gastroesophageal junction cancer, and esophageal adenocarcinoma [see Clinical Studies (14.12)]. The trial excluded patients who were known human epidermal growth factor receptor 2 (HER2) positive, or had untreated CNS metastases. Patients were randomized to receive OPDIVO in combination with chemotherapy or chemotherapy. Patients received one of the following treatments:<br><br>**14 CLINICAL STUDIES**<br>**14.12 Gastric Cancer, Gastroesophageal Junction Cancer, and Esophageal Adenocarcinoma** CHECKMATE-649 (NCT02872116) was a randomized, multicenter, open-label trial in patients (n=1581) with previously untreated advanced or metastatic gastric cancer, gastroesophageal junction cancer, and esophageal adenocarcinoma. The trial enrolled patients regardless of PDL1 status, and tumor specimens were evaluated prospectively using the PD-L1 IHC 28-8 pharmDx assay at a central laboratory. The trial excluded patients who were known human epidermal growth factor receptor 2 (HER2) positive, or had untreated CNS metastases.
**761234, 03/18/2022**	**Nivolumab and Relatlimab-rmbw** (1)	Oncology	BRAF	Clinical Studies	**14 CLINICAL STUDIES**<br>The efficacy of OPDUALAG was investigated in RELATIVITY-047 (NCT03470922), a randomized (1:1), double-blinded trial in 714 patients with previously untreated metastatic or unresectable Stage III or IV melanoma. (…) Randomization was stratified by tumor PD-L1 expression (≥1% vs. <1%) using PD-L1 IH 28-8 pharmDx test, LAG-3 expression (≥1% vs. <1%) using a clinical trial assay, BRAF V600 mutation status (V600 mutation positive vs. wild type), and M stage per the American Joint Committee on Cancer (AJCC) version 8 staging system (M0/M1any [0] vs. M1any[1]).<br>The trial population characteristics were: median age 63 years (range: 20 to 94); 58% male; 97% White 0.7% African American, and American Indian/Alaskan Native 0.1%; Hispanic 7%; and ECOG performance score was 0 (67%) or 1 (33%). Disease characteristics were: PD-L1 expression ≥1% (41%), LAG-

* Therapeutic areas do not necessarily reflect the CDER review division.
† Representative biomarkers are listed based on standard nomenclature as per the Human Genome Organization (HUGO) symbol and/or simplified descriptors using other common conventions. Listed biomarkers do not necessarily reflect the terminology used in labeling. The term “Nonspecific” is provided when labeling does not explicitly identify the specific biomarker(s) or when the biomarker is represented by a molecular phenotype or gene signature, and in some cases the biomarker was inferred based on the labeling language.
‡ Referenced figures and tables may be found in the labeling available at Drugs@FDA.
Blue text represents the most recent addtions and/or changes since last posted version.

NDA/ANDA/BLA Number, Label Version Date	Drug	Therapeutic Area*	Biomarker†	Labeling Sections	Labeling Text‡
					expression ≥1% (75%), AJCC Stage IV disease (92%), M1c disease (39%); M1d disease (2.4%), elevated LDH (36%), and BRAF V600 mutation-positive melanoma (39%).
**761234, 03/18/2022**	**Nivolumab and Relatlimab-rmbw** (2)	Oncology	CD274 (PD-L1)	Clinical Studies	**14 CLINICAL STUDIES**<br>The efficacy of OPDUALAG was investigated in RELATIVITY-047 (NCT03470922), a randomized (1:1), double-blinded trial in 714 patients with previously untreated metastatic or unresectable Stage III or IV melanoma. (…) Randomization was stratified by tumor PD-L1 expression (≥1% vs. <1%) using PD-L1 IH 28-8 pharmDx test, LAG-3 expression (≥1% vs. <1%) using a clinical trial assay, BRAF V600 mutation status (V600 mutation positive vs. wild type), and M stage per the American Joint Committee on Cancer (AJCC) version 8 staging system (M0/M1any [0] vs. M1any [1]).<br>The trial population characteristics were: median age 63 years (range: 20 to 94); 58% male; 97% White 0.7% African American, and American Indian/Alaskan Native 0.1%; Hispanic 7%; and ECOG performance score was 0 (67%) or 1 (33%). Disease characteristics were: PD-L1 expression ≥1% (41%), LAG-expression ≥1% (75%), AJCC Stage IV disease (92%), M1c disease (39%); M1d disease (2.4%), elevated LDH (36%), and BRAF V600 mutation-positive melanoma (39%).
**761234, 03/18/2022**	**Nivolumab and Relatlimab-rmbw** (3)	Oncology	LAG3	Clinical Studies	**14 CLINICAL STUDIES**<br>The efficacy of OPDUALAG was investigated in RELATIVITY-047 (NCT03470922), a randomized (1:1), double-blinded trial in 714 patients with previously untreated metastatic or unresectable Stage III or IV melanoma. (…) Randomization was stratified by tumor PD-L1 expression (≥1% vs. <1%) using PD-L1 IH 28-8 pharmDx test, LAG-3 expression (≥1% vs. <1%) using a clinical trial assay, BRAF V600 mutation status (V600 mutation positive vs. wild type), and M stage per the American Joint Committee on Cancer (AJCC) version 8 staging system (M0/M1any [0] vs. M1any [1]).<br>The trial population characteristics were: median age 63 years (range: 20 to 94); 58% male; 97% White 0.7% African American, and American Indian/Alaskan Native 0.1%; Hispanic 7%; and ECOG performance score was 0 (67%) or 1 (33%). Disease characteristics were: PD-L1 expression ≥1% (41%), LAG-expression ≥1% (75%), AJCC Stage IV disease (92%), M1c disease (39%); M1d disease (2.4%), elevated LDH (36%), and BRAF V600 mutation-positive melanoma (39%).
**018013, 04/09/2019**	**Nortriptyline**	Psychiatry	CYP2D6	Precautions	**PRECAUTIONS**<br>*Drugs Metabolized by P450 2D6*<br>The biochemical activity of the drug metabolizing isozyme cytochrome P450 2D6 (debrisoquin hydroxylase) is reduced in a subset of the Caucasian population (about 7% to 10% of Caucasians are so called “poor metabolizers”); reliable estimates of the prevalence of reduced P450 2D6 isozyme activity among Asian, African and other populations are not yet available. Poor metabolizers have higher than expected plasma concentrations of tricyclic antidepressants (TCAs) when given usual doses. Depending on the fraction of drug metabolized by P450 2D6, the increase in plasma concentration may be small, or quite large (8 fold increase in plasma AUC of the TCA). (…)
**209531, 10/10/2018**	**Nusinersen**	Neurology	SMN2	Clinical Pharmacology, Clinical Studies	**12 CLINICAL PHARMACOLOGY**<br>**12.2 Pharmacodynamics**<br>Autopsy samples from patients (n=3) had higher levels of SMN2 messenger ribonucleic acid (mRNA) containing exon 7 in the thoracic spinal cord compared to untreated SMA infants.<br><br>**14 CLINICAL STUDIES**<br>**14.1 Infantile-Onset SMA**<br>(…) Baseline demographics were balanced between the SPINRAZA and control groups with the exception of age at first treatment (median age 175 vs. 206 days, respectively). The SPINRAZA and control groups were balanced with respect to gestational age, birth weight, disease duration, and SMN2 copy number. (…)<br>**14.3 Presymptomatic SMA**<br>(…) Some patients receiving SPINRAZA before the onset of SMA symptoms survived without requiring permanent ventilation beyond what would be expected based on their SMN2 copy number, and some patients also achieved age-appropriate growth and developmental motor milestones such as the ability to sit unassisted, stand, or walk.
**125486, 03/27/2020**	**Obinutuzumab**	Oncology	MS4A1 (CD20 antigen)	Clinical Studies	**14 CLINICAL STUDIES**<br>**14.1 Chronic Lymphocytic Leukemia**<br>GAZYVA was evaluated in a three-arm, open-label, active-controlled, randomized, multicenter trial (Study 1) in 781 patients with previously untreated CD20+ chronic lymphocytic leukemia requiring treatment who had coexisting medical conditions or reduced renal function as measured by creatinine clearance (CrCl) < 70 mL/min. (…)
**215498, 06/13/2023**	**Odevixibat (1)**	Gastroenterology	ABCB11	Indications and Usage, Clinical Pharmacology, Clinical Studies	**1 INDICATIONS AND USAGE**<br>BYLVAY is indicated for the treatment of pruritus in patients 3 months of age and older with progressive familial intrahepatic cholestasis (PFIC).<br>Limitations of Use<br>• BYLVAY may not be effective in PFIC type 2 patients with ABCB11 variants resulting in nonfunctional or complete absence of bile salt export pump protein (BSEP-3).<br><br>**12 CLINICAL PHARMACOLOGY**<br>**12.5 Pharmacogenomics**<br>PFIC is a heterogenous disease caused by homozygous or compound heterozygous variants, with different PFIC subtypes occurring in the general population. PFIC2 is the most common subtype accounting for 37-90% of PFIC patients. PFIC1 is caused by variants in the ATP8B1 gene, which encodes the Familial Intrahepatic Cholestasis 1 (FIC1) protein, while PFIC2 results from variants in the ABCB11 gene, which encodes the Bile Salt Export Pump (BSEP) protein. PFIC2 patients are further categorized into BSEP subgroups based on their specific variants. The BSEP-1 subgroup includes patients with at least one p.D482G

Table of Pharmacogenomic Biomarkers in Drug Labeling
Last Updated: 06/2024

NDA/ANDA/BLA Number, Label Version Date	Drug	Therapeutic Area*	Biomarker†	Labeling Sections	Labeling Text‡
					(c.1445A>G) or p.E297G (c.890A>G) variant, BSEP-2 includes patients with at least one missense variant other than p.D482G or p.E297G (non BSEP-1), and BSEP-3 includes patients with variants that are predicted to encode a non-functional protein. No BSEP-3 patients were studied in clinical Trial 1 (NCT03566238). The frequencies of BSEP-1, BSEP2, and BSEP-3 subgroups within PFIC2 patients are approximately 27.3%, 51.5%, and 21.2%, respectively, based on data from a global consortium characterizing the natural history of severe BSEP deficiency.<br><br>**14 CLINICAL STUDIES**<br>The efficacy of BYLVAY was evaluated in Trial 1 (NCT03566238), a 24-week, randomized, doubleblind, placebo-controlled trial. Trial 1 was conducted in 62 pediatric patients, aged 6 months to 17 years, with a confirmed molecular diagnosis of PFIC type 1 or type 2, and presence of pruritus at baseline. Patients with variants in the ABCB11 gene that predict non-function or complete absence of the bile salt export pump (BSEP) protein, who had experienced prior hepatic decompensation events, who had other concomitant liver disease, whose INR was greater than 1.4, whose ALT or total bilirubin was greater than 10-times the upper limit of normal (ULN), or who had received a liver transplant were excluded in Trial 1.
**215498, 06/13/2023**	Odevixibat (2)	Gastroenterology	ATP8B1	Indications and Usage, Clinical Pharmacology, Clinical Studies	**1 INDICATIONS AND USAGE**<br>BYLVAY is indicated for the treatment of pruritus in patients 3 months of age and older with progressive familial intrahepatic cholestasis (PFIC).<br>Limitations of Use<br>• BYLVAY may not be effective in PFIC type 2 patients with ABCB11 variants resulting in nonfunctional or complete absence of bile salt export pump protein (BSEP-3).<br><br>**12 CLINICAL PHARMACOLOGY**<br>**12.5 Pharmacogenomics**<br>PFIC is a heterogenous disease caused by homozygous or compound heterozygous variants, with different PFIC subtypes occurring in the general population. PFIC2 is the most common subtype accounting for 37-90% of PFIC patients. PFIC1 is caused by variants in the ATP8B1 gene, which encodes the Familial Intrahepatic Cholestasis 1 (FIC1) protein, while PFIC2 results from variants in the ABCB11 gene, which encodes the Bile Salt Export Pump (BSEP) protein. PFIC2 patients are further categorized into BSEP subgroups based on their specific variants. The BSEP-1 subgroup includes patients with at least one p.D482G (c.1445A>G) or p.E297G (c.890A>G) variant, BSEP-2 includes patients with at least one missense variant other than p.D482G or p.E297G (non BSEP-1), and BSEP-3 includes patients with variants that are predicted to encode a non-functional protein. No BSEP-3 patients were studied in clinical Trial 1 (NCT03566238). The frequencies of BSEP-1, BSEP2, and BSEP-3 subgroups within PFIC2 patients are approximately 27.3%, 51.5%, and 21.2%, respectively, based on data from a global consortium characterizing the natural history of severe BSEP deficiency.<br><br>**14 CLINICAL STUDIES**<br>The efficacy of BYLVAY was evaluated in Trial 1 (NCT03566238), a 24-week, randomized, doubleblind, placebo-controlled trial. Trial 1 was conducted in 62 pediatric patients, aged 6 months to 17 years, with a confirmed molecular diagnosis of PFIC type 1 or type 2, and presence of pruritus at baseline. Patients with variants in the ABCB11 gene that predict non-function or complete absence of the bile salt export pump (BSEP) protein, who had experienced prior hepatic decompensation events, who had other concomitant liver disease, whose INR was greater than 1.4, whose ALT or total bilirubin was greater than 10-times the upper limit of normal (ULN), or who had received a liver transplant were excluded in Trial 1.
**215498, 06/13/2023**	Odevixibat (3)	Gastroenterology	JAG1	Clinical Studies	**14 CLINICAL STUDIES**<br>**14.2 ALGS**<br>The efficacy of BYLVAY was evaluated in Trial 3 (NCT04674761), a 24-week, randomized, doubleblind, placebo-controlled trial. Trial 3 was conducted in 52 pediatric patients, aged 6 months to 15 years, with a confirmed diagnosis of ALGS and presence of pruritus at baseline. Patients who had decompensated liver disease, who had other concomitant liver disease, whose INR was greater than 1.4, whose ALT was greater than 10-times the upper limit of normal (ULN) at screening, whose total bilirubin was greater than 15-times the ULN at screening, or who had received a liver transplant were excluded from Trial 3..<br>Patients were randomized to placebo (n=17) or 120 mcg/kg (n=35). Study drug was administered once daily with a meal in the morning. In patients weighing less than 19.5 kg or patients who could not swallow the whole capsule, study drug was sprinkled on soft food and then administered orally.<br>Median age (range) of the patients in Trial 3 was 6.1 (1.7 to 15.5) years in the BYLVAY group and 4.2 (0.5 to 14.3) years in the placebo group; 5 patients were older than 12 years of age. Of the 52 patients, 52% were male and 83% were white; 92% of patients had the JAG1 mutation and 8% had the NOTCH2 mutation. The mean (standard deviation [SD]) scratching score in the 2 weeks prior to baseline was 2.9 (0.6). Baseline mean (SD) eGFR was 159 (51.4) mL/min/1.73 m2. Baseline median (range) ALT, AST, and total bilirubin were 152 (39-403) U/L, 135 (57-427) U/L, and 2.0 (0.4-11.4) mg/dL, respectively.
**215498, 06/13/2023**	Odevixibat (4)	Gastroenterology	NOTCH2	Clinical Studies	**14 CLINICAL STUDIES**<br>**14.2 ALGS**<br>The efficacy of BYLVAY was evaluated in Trial 3 (NCT04674761), a 24-week, randomized, doubleblind, placebo-controlled trial. Trial 3 was conducted in 52 pediatric patients, aged 6 months to 15 years, with a confirmed diagnosis of ALGS and presence of pruritus at baseline. Patients who had decompensated liver disease, who had other concomitant liver disease, whose INR was greater than 1.4, whose ALT was greater than 10-times the upper limit of normal (ULN) at screening, whose total bilirubin was greater than 15-times the ULN at screening, or who had received a liver transplant were excluded from Trial 3..<br>Patients were randomized to placebo (n=17) or 120 mcg/kg (n=35). Study drug was administered once daily with a meal in the morning. In patients weighing less than 19.5 kg or patients who could not swallow the whole capsule, study drug was sprinkled on soft food and then administered orally.<br>Median age (range) of the patients in Trial 3 was 6.1 (1.7 to 15.5) years in the BYLVAY group and 4.2 (0.5 to 14.3) years in the placebo group; 5 patients were older than 12 years of age. Of the 52 patients, 52% were male and 83% were white; 92% of patients had the JAG1 mutation and 8% had the NOTCH2 mutation. The mean (standard deviation [SD]) scratching score in the 2 weeks prior to baseline was 2.9 (0.6). Baseline mean (SD) eGFR was 159 (51.4) mL/min/1.73 m2. Baseline median (range) ALT, AST, and total bilirubin were 152 (39-403) U/L, 135 (57-427) U/L, and 2.0 (0.4-11.4) mg/dL, respectively.

* Therapeutic areas do not necessarily reflect the CDER review division.
† Representative biomarkers are listed based on standard nomenclature as per the Human Genome Organization (HUGO) symbol and/or simplified descriptors using other common conventions. Listed biomarkers do not necessarily reflect the terminology used in labeling. The term "Nonspecific" is provided when labeling does not explicitly identify the specific biomarker(s) or when the biomarker is represented by a molecular phenotype or gene signature, and in some cases the biomarker was inferred based on the labeling language.
‡ Referenced figures and tables may be found in the labeling available at Drugs@FDA.
Blue text represents the most recent addtions and/or changes since last posted version.

Table of Pharmacogenomic Biomarkers in Drug Labeling
Last Updated: 06/2024

NDA/ANDA/BLA Number, Label Version Date	Drug	Therapeutic Area*	Biomarker†	Labeling Sections	Labeling Text‡
208558, 05/31//2023	Olaparib (1)	Oncology	BRCA	Indications and Usage, Dosage and Administration, Warnings and Precautions, Adverse Reactions, Clinical Studies	**1 INDICATIONS AND USAGE**<br>**1.1 First-Line Maintenance Treatment of BRCA-mutated Advanced Ovarian Cancer**<br>Lynparza is indicated for the maintenance treatment of adult patients with deleterious or suspected deleterious germline or somatic BRCA-mutated (gBRCAm or sBRCAm) advanced epithelial ovarian, fallopian tube or primary peritoneal cancer who are in complete or partial response to first-line platinumbased chemotherapy. Select patients with gBRCAm advanced epithelial ovarian, fallopian tube or primary peritoneal cancer for therapy based on an FDA-approved companion diagnostic for Lynparza [see Dosage and Administration (2.1)].<br>**1.2 First-line Maintenance Treatment of HRD-positive Advanced Ovarian Cancer in Combination with Bevacizumab**<br>Lynparza is indicated in combination with bevacizumab for the maintenance treatment of adult patients with advanced epithelial ovarian, fallopian tube or primary peritoneal cancer who are in complete or partial response to first-line platinum-based chemotherapy and whose cancer is associated with homologous recombination deficiency (HRD)-positive status defined by either:<br>• a deleterious or suspected deleterious BRCA mutation, and/or<br>• genomic instability.<br>Select patients for therapy based on an FDA-approved companion diagnostic for Lynparza [see Dosage and Administration (2.1)].<br>**1.4 Advanced Germline BRCA-mutated Ovarian Cancer After 3 or More Lines of Chemotherapy**<br>Lynparza is indicated for the treatment of adult patients with deleterious or suspected deleterious germline BRCA-mutated (gBRCAm) advanced ovarian cancer who have been treated with three or more prior lines of chemotherapy. Select patients for therapy based on an FDA-approved companion diagnostic for Lynparza [see Dosage and Administration (2.1)].<br>**1.5 Adjuvant Treatment of Germline BRCA-mutated HER2-negative High Risk Early Breast Cancer**<br>Lynparza is indicated for the adjuvant treatment of adult patients with deleterious or suspected deleterious gBRCAm human epidermal growth factor receptor 2 (HER2)-negative high risk early breast cancer who have been treated with neoadjuvant or adjuvant chemotherapy. Select patients for therapy based on an FDA-approved companion diagnostic for Lynparza [see Dosage and Administration (2.1)]<br>**1.6 Germline BRCA-mutated HER2-negative Metastatic Breast Cancer**<br>Lynparza is indicated for the treatment of adult patients with deleterious or suspected deleterious gBRCAm, HER2-negative metastatic breast cancer, who have been treated with chemotherapy in the neoadjuvant, adjuvant, or metastatic setting. Patients with hormone receptor (HR)-positive breast cancer should have been treated with a prior endocrine therapy or be considered inappropriate for endocrine therapy. Select patients for therapy based on an FDA-approved companion diagnostic for Lynparza [see Dosage and Administration (2.1)].<br>**1.7 First-Line Maintenance Treatment of Germline BRCA-mutated Metastatic Pancreatic Adenocarcinoma**<br>Lynparza is indicated for the maintenance treatment of adult patients with deleterious or suspected deleterious gBRCAm metastatic pancreatic adenocarcinoma whose disease has not progressed on at least 16 weeks of a first-line platinum-based chemotherapy regimen. Select patients for therapy based on an FDA-approved companion diagnostic for Lynparza [see Dosage and Administration (2.1)].<br>**1.8 Treatment of BRCA-mutated Metastatic Castration-Resistant Prostate Cancer in Combination with Abiraterone and Prednisone or Prednisolone**<br>Lynparza is indicated in combination with abiraterone and prednisone or prednisolone for the treatment of adult patients with deleterious or suspected deleterious BRCA-mutated (BRCAm) metastatic castrationresistant prostate cancer (mCRPC). Select patients for therapy based on an FDA-approved companion diagnostic for Lynparza [see Dosage and Administration (2.1)].<br><br>**2 DOSAGE AND ADMINISTRATION**<br>2.1 Patient Selection<br>Information on FDA-approved tests for the detection of genetic mutations is available at http://www.fda.gov/companiondiagnostics. Select patients for treatment with Lynparza based on the presence of deleterious or suspected deleterious HRR gene mutations, including BRCA mutations, or genomic instability based on the indication, biomarker, and sample type (Table 1).<br>**2.2 Recommended Dosing**<br><u>First-Line Maintenance Treatment of BRCA-mutated Advanced Ovarian Cancer</u><br>Continue treatment until disease progression, unacceptable toxicity, or completion of 2 years of treatment. Patients with a complete response (no radiological evidence of disease) at 2 years should stop treatment. Patients with evidence of disease at 2 years, who in the opinion of the treating healthcare provider can derive further benefit from continuous treatment, can be treated beyond 2 years.<br><u>Recurrent Ovarian Cancer, Germline BRCAm Advanced Ovarian Cancer, HER2-negative Metastatic Breast Cancer, Metastatic Pancreatic Adenocarcinoma, and HRR Gene-mutated Metastatic CastrationResistant Prostate Cancer</u><br>Continue treatment until disease progression or unacceptable toxicity for:<br>• Maintenance treatment of recurrent ovarian cancer<br>• Advanced germline BRCA-mutated ovarian cancer<br>• Germline BRCA-mutated HER-2 negative metastatic breast cancer<br>• First-line maintenance treatment of germline BRCA-mutated metastatic pancreatic adenocarcinoma. (…)<br><br>**5 WARNINGS AND PRECAUTIONS**<br>**5.1 Myelodysplastic Syndrome/Acute Myeloid Leukemia**<br>In clinical studies enrolling 2351 patients with various cancers who received Lynparza as a single agent [see Adverse Reactions (6.1)], the incidence of Myelodysplastic Syndrome/Acute Myeloid Leukemia (MDS/AML) was <1.5% (28/2351) and the majority of events had a fatal outcome. Of these, 25/28 patients had a documented BRCA mutation, 2 patients had gBRCA wildtype and in 1 patient the BRCA mutation status was unknown. (…)

* Therapeutic areas do not necessarily reflect the CDER review division.
† Representative biomarkers are listed based on standard nomenclature as per the Human Genome Organization (HUGO) symbol and/or simplified descriptors using other common conventions. Listed biomarkers do not necessarily reflect the terminology used in labeling. The term “Nonspecific” is provided when labeling does not explicitly identify the specific biomarker(s) or when the biomarker is represented by a molecular phenotype or gene signature, and in some cases the biomarker was inferred based on the labeling language.
‡ Referenced figures and tables may be found in the labeling available at Drugs@FDA.
Blue text represents the most recent addtions and/or changes since last posted version.

NDA/ANDA/BLA Number, Label Version Date	Drug	Therapeutic Area*	Biomarker†	Labeling Sections	Labeling Text‡
					**6 ADVERSE REACTIONS**<br>**First-Line Maintenance Treatment of BRCA-mutated Advanced Ovarian Cancer**<br>*SOLO-1*<br>The safety of Lynparza for the maintenance treatment of patients with BRCA-mutated advanced ovarian cancer following first-line treatment with platinum-based chemotherapy was investigated in SOLO-1, a placebo-controlled, double-blind study in which 390 patients received either Lynparza 300 mg BID (n=260) or placebo tablets (n=130) until disease progression or unacceptable toxicity. The median duration of study treatment was 25 months for patients who received Lynparza and 14 months for patients who received placebo. (…)<br>**Maintenance Treatment of Recurrent Ovarian Cancer**<br>*SOLO-2*<br>The safety of Lynparza for the maintenance treatment of patients with platinum sensitive gBRCAm ovarian cancer was investigated in SOLO-2. This study was a placebo-controlled, double-blind study in which 294 patients received either Lynparza 300 mg (2 x 150 mg tablets) twice daily (n=195) or placebo tablets twice daily (n=99) until disease progression or unacceptable toxicity. (…)<br>**Advanced Germline BRCA-mutated Ovarian Cancer After 3 or More Lines of Chemotherapy**<br>*Pooled data*<br>The safety of Lynparza was investigated in 223 patients (pooled from 6 studies) with gBRCAm advanced ovarian cancer who had received 3 or more prior lines of chemotherapy [see Clinical Studies (14.4)]. Patients received Lynparza capsules 400 mg orally twice daily until disease progression or unacceptable tolerability. The median exposure to Lynparza in these patients was 5.2 months. (…)<br>**Germline BRCA-mutated HER2-negative Metastatic Breast Cancer**<br>*OlympiAD*<br>The safety of Lynparza was evaluated in gBRCAm patients with HER2-negative metastatic breast cancer who had previously received up to two lines of chemotherapy for the treatment of metastatic disease in OlympiAD [see Clinical Studies (14.5)]. (…)<br>**First-line Maintenance Treatment of Germline BRCA-mutated Metastatic Pancreatic Adenocarcinoma**<br>*POLO*<br>The safety of Lynparza as maintenance treatment of germline BRCA-mutated metastatic pancreatic adenocarcinoma following first-line treatment with platinum-based chemotherapy was evaluated in POLO [see Clinical Studies (14.6)]. (…)<br>**HRR Gene-mutated Metastatic Castration-Resistant Prostate Cancer**<br>*PROfound*<br>The safety of Lynparza as monotherapy was evaluated in patients with mCRPC and HRR gene mutations who have progressed following prior treatment with enzalutamide or abiraterone in PROfound [see Clinical Studies (14.7)]. This study was a randomized, open-label, multi-center study in which 386 patients received either Lynparza tablets 300 mg orally twice daily (n=256) or investigator's choice of enzalutamide or abiraterone acetate (n=130) until disease progression or unacceptable toxicity. Among patients receiving Lynparza, 62% were exposed for 6 months or longer and 20% were exposed for greater than one year. (…)<br>**Treatment of BRCA-mutated Metastatic Castration-Resistant Prostate Cancer in Combination with Abiraterone and Prednisone or Prednisolone**<br>*PROpel*<br>The safety of Lynparza in combination with abiraterone and prednisone or prednisolone for the treatment of patients in the first-line mCRPC setting was investigated in PROpel [see Clinical Studies (14.8)]. Patients were randomized to receive either Lynparza tablets 300 mg orally twice daily plus abiraterone tablets 1000 mg once daily (Lynparza/abiraterone) (n=398), or placebo plus abiraterone 1000 mg once daily (placebo/abiraterone) (n=396) until disease progression or unacceptable toxicity. Patients in both arms also received either prednisone or prednisolone 5 mg twice daily. (…)<br><br>**14 CLINICAL STUDIES**<br>**14.1 First-Line Maintenance Treatment of BRCA-mutated Advanced Ovarian Cancer**<br>The efficacy of Lynparza was evaluated in SOLO-1 (NCT01844986), a randomized (2:1), double-blind, placebo-controlled, multi-center trial in patients with BRCA-mutated advanced ovarian, fallopian tube, or primary peritoneal cancer following first-line platinum-based chemotherapy. (…)<br>A total of 391 patients were randomized, 260 to Lynparza and 131 to placebo. The median age of patients treated with Lynparza was 53 years (range: 29 to 82) and 53 years (range: 31 to 84) among patients on placebo. The ECOG performance status (PS) was 0 in 77% of patients receiving Lynparza and 80% of patients receiving placebo. Of all patients, 82% were White, 36% were enrolled in the U.S. or Canada, and 82% were in complete response to their most recent platinum-based regimen. The majority of patients (n=389) had germline BRCA mutation (gBRCAm), and 2 patients had somatic BRCAm (sBRCAm).<br>Of the 391 patients randomized in SOLO-1, 386 were retrospectively or prospectively tested with a Myriad BRACAnalysis test and 383 patients were confirmed to have deleterious or suspected deleterious gBRCAm status; 253 were randomized to the Lynparza arm and 130 to the placebo arm. Two out of 391 patients randomized in SOLO-1 were confirmed to have sBRCAm based on an investigational Foundation Medicine tissue test. (…)<br>**14.2 First-line Maintenance Treatment of HRD-positive Advanced Ovarian Cancer in Combination with Bevacizumab**<br>*PAOLA-1*<br>PAOLA-1 (NCT02477644) was a randomized, double-blind, placebo-controlled, multi-center trial that compared the efficacy of Lynparza in combination with bevacizumab versus placebo/bevacizumab for the maintenance treatment of advanced high-grade epithelial ovarian cancer, fallopian tube or primary peritoneal cancer following first-line platinum-based chemotherapy and bevacizumab. Randomization was stratified by first-line treatment outcome (timing and outcome of cytoreductive surgery and response to platinum-based chemotherapy) and tBRCAm status, determined by prospective local testing. All available clinical samples were retrospectively tested with Myriad myChoice® CDx. (…)<br>**14.3 Maintenance Treatment of Recurrent Ovarian Cancer**<br>The efficacy of Lynparza was investigated in two randomized, placebo-controlled, double-blind, multicenter studies in patients with recurrent ovarian cancers who were in response to platinum-based therapy.

* Therapeutic areas do not necessarily reflect the CDER review division.
† Representative biomarkers are listed based on standard nomenclature as per the Human Genome Organization (HUGO) symbol and/or simplified descriptors using other common conventions. Listed biomarkers do not necessarily reflect the terminology used in labeling. The term “Nonspecific” is provided when labeling does not explicitly identify the specific biomarker(s) or when the biomarker is represented by a molecular phenotype or gene signature, and in some cases the biomarker was inferred based on the labeling language.
‡ Referenced figures and tables may be found in the labeling available at Drugs@FDA.
Blue text represents the most recent addtions and/or changes since last posted version.

NDA/ANDA/BLA Number, Label Version Date	Drug	Therapeutic Area*	Biomarker†	Labeling Sections	Labeling Text‡
					SOLO-2<br>The efficacy of Lynparza was evaluated in SOLO-2 (NCT01874353), a randomized (2:1) double-blind, placebo-controlled trial in patients with gBRCAm ovarian, fallopian tube, or primary peritoneal cancer. Patients were randomized to Lynparza tablets 300 mg orally twice daily or placebo until unacceptable toxicity or progressive disease. (…)<br>All patients had a deleterious or suspected deleterious germline BRCA mutation as detected either by a local test (n=236) or central Myriad CLIA test (n=59), subsequently confirmed by BRACAnalysis CDx® (n=286). (…)<br>*Study 19*<br>A retrospective analysis for germline BRCA mutation status, some performed using the Myriad test, indicated that 36% (n=96) of patients from the ITT population had deleterious gBRCA mutation, including 39% (n=53) of patients on Lynparza and 33% (n=43) of patients on placebo. (…)<br>**14.4 Advanced Germline BRCA-mutated Ovarian Cancer Treated with 3 or More Prior Lines of Chemotherapy**<br>The efficacy of Lynparza was investigated in a single-arm study of patients with deleterious or suspected deleterious gBRCAm advanced cancers. A total of 137 patients with measurable, advanced gBRCAm ovarian cancer treated with three or more prior lines of chemotherapy were enrolled. All patients received Lynparza capsules 400 mg orally twice daily until disease progression or intolerable toxicity. The efficacy outcome measures were objective response rate (ORR) and duration of response (DOR) as assessed by the investigator according to RECIST, version 1.0.<br>The median age of the patients was 58 years, the majority were White (94%) and 93% had an ECOG PS of 0 or 1. Deleterious or suspected deleterious gBRCAm status was verified retrospectively in 97% (59/61) of the patients for whom blood samples were available by the BRACAnalysis CDxTM . (See Table 22) (…)<br>**14.5 Adjuvant Treatment of Germline BRCA-mutated HER2-negative High Risk Early Breast Cancer**<br>The efficacy of Lynparza was evaluated in OlympiA (NCT02032823), a randomized (1:1), double-blind, placebo-controlled, international study in patients with gBRCAm HER2-negative high risk early breast cancer who had completed definitive local treatment and neoadjuvant or adjuvant chemotherapy. (See Table 25)<br>3 patients who received prior adjuvant chemotherapy: patients with TNBC must have had node positive disease or node negative disease with a ≥2cm primary tumor; patients with hormone receptor positive, HER2-negative breast cancer must have had ≥4 pathologically confirmed positive lymph nodes. (…)<br>Patients enrolled based on local gBRCA test results provided a sample for retrospective confirmatory central testing with BRACAnalysis®. Out of 1836 patients enrolled into OlympiA, 1623 were confirmed as gBRCAm by Myriad BRACAnalysis®, either prospectively or retrospectively. (…)<br>**14.6 Treatment of Germline BRCA-mutated HER2-negative Metastatic Breast Cancer**<br>The efficacy of Lynparza was evaluated in OlympiAD (NCT02000622), an open-label randomized (2:1) study in patients with gBRCAm HER2-negative metastatic breast cancer. Patients were required to have received treatment with an anthracycline (unless contraindicated) and a taxane, in the neoadjuvant, adjuvant or metastatic setting. (…)<br>Of the 302 patients randomized onto OlympiAD, 299 were tested with the BRACAnalysis CDx® and 297 were confirmed to have deleterious or suspected deleterious gBRCAm status; 202 were randomized to the Lynparza arm and 95 to the healthcare provider's choice of chemotherapy arm. (…)<br>**14.7 Treatment of Germline BRCA-mutated HER2-negative Metastatic Breast Cancer**<br>The efficacy of Lynparza was evaluated in OlympiAD (NCT02000622), an open-label randomized (2:1) study in patients with gBRCAm HER2-negative metastatic breast cancer. Patients were required to have received treatment with an anthracycline (unless contraindicated) and a taxane, in the neoadjuvant, adjuvant or metastatic setting. (…)<br>Of the 302 patients randomized onto OlympiAD, 299 were tested with the BRACAnalysis CDx® and 297 were confirmed to have deleterious or suspected deleterious gBRCAm status; 202 were randomized to the Lynparza arm and 95 to the healthcare provider's choice of chemotherapy arm. (…)<br>**14.7 First-Line Maintenance Treatment of Germline BRCA-mutated Metastatic Pancreatic Adenocarcinoma**<br>The efficacy of Lynparza was evaluated in POLO (NCT02184195), a randomized (3:2), double-blind placebo-controlled, multi-center trial. Patients were required to have metastatic pancreatic adenocarcinoma with a deleterious or suspected deleterious germline BRCA mutation (gBRCAm) and absence of disease progression after receipt of first-line platinum-based chemotherapy for at least 16 weeks. (…)<br>All patients had a deleterious or suspected deleterious germline BRCA-mutation as detected by the Myriad BRACAnalysis® or BRACAnalysis CDx® at a central laboratory only (n=106), local BRCA test only (n=4), or both local and central testing (n=44). Among the 150 patients with central test results, 30% had a mutation in BRCA1; 69% had a mutation in BRCA2; and 1 patient (1%) had mutations in both BRCA1 and BRCA2.<br>Efficacy results of POLO are provided in Table 24 and Figure 6.<br>**14.8 HRR Gene-mutated Metastatic Castration-Resistant Prostate Cancer**<br>(…) Patients were divided into two cohorts based on HRR gene mutation status. Patients with mutations in either BRCA1, BRCA2, or ATM were randomized in Cohort A; patients with mutations among 12 other genes involved in the HRR pathway (BARD1, BRIP1, CDK12, CHEK1, CHEK2, FANCL, PALB2, PPP2R2A, RAD51B, RAD51C, RAD51D, or RAD54L) were randomized in Cohort B; patients with comutations (BRCA1, BRCA2, or ATM plus a Cohort B gene) were assigned to Cohort A. (…)Determination of deleterious or suspected deleterious somatic or germline HRR mutation status in line with the FDA approved mutation classification and testing criteria for the Foundation Medicine F1CDx tissue-based assay and assessment of the germline-BRCA status using the Myriad BRACAnalysis CDx blood-based assay was performed retrospectively. Representation of individual gene mutations by cohort is provided in Table 25.<br>No patients were enrolled who had mutations in two of the 15 pre-specified HRR genes: FANCL and RAD51C. (See Table 25)<br>Consistent results were observed in exploratory analyses of rPFS for patients who received or did not receive prior taxane therapy and for those with germline-BRCA mutations identified using the Myriad BRACAnalysis CDx assay compared with those with BRCA mutations identified using the Foundation Medicine F1CDx assay. (See Table 27)<br>**14.8 Treatment of BRCA-mutated Metastatic Castration-Resistant Prostate Cancer in Combination with Abiraterone and Prednisone or Prednisolone**<br>The efficacy of Lynparza in the treatment of patients with mCRPC was investigated in PROpel (NCT03732820), a randomized, double-blind, placebo-controlled, multi-center study that compared the efficacy of Lynparza in combination with abiraterone with placebo plus abiraterone for patients with mCRPC. (…)

* Therapeutic areas do not necessarily reflect the CDER review division.
† Representative biomarkers are listed based on standard nomenclature as per the Human Genome Organization (HUGO) symbol and/or simplified descriptors using other common conventions. Listed biomarkers do not necessarily reflect the terminology used in labeling. The term "Nonspecific" is provided when labeling does not explicitly identify the specific biomarker(s) or when the biomarker is represented by a molecular phenotype or gene signature, and in some cases the biomarker was inferred based on the labeling language.
‡ Referenced figures and tables may be found in the labeling available at Drugs@FDA.
Blue text represents the most recent addtions and/or changes since last posted version.

Table of Pharmacogenomic Biomarkers in Drug Labeling
Last Updated: 06/2024

NDA/ANDA/BLA Number, Label Version Date	Drug	Therapeutic Area*	Biomarker†	Labeling Sections	Labeling Text‡
					BRCA gene mutation (BRCAm) status was assessed after randomization and before primary analysis by both NGS-based tumor tissue and ctDNA tests. BRCAm classification criteria in line with the FDA approved assays were used to determine the deleterious and suspected deleterious somatic or germline mutation status of patients. (…)<br>Of the 796 patients tested, 85 (11%) had BRCAm determined by either a positive ctDNA test (9%) or a tumor tissue test (6%). Among these 85 patients, the median age was 68 years (range 43 to 85), and 67% were 65 years or older; 72% were White, 22% Asian, and 2% Black or African American; 66% had ECOG performance status (PS) 0 and 34% had ECOG PS 1; 25% had prior docetaxel treatment for mHSPC; 53% had bone-only metastases, 15% had visceral metastases, and 32% had other metastases.<br>A statistically significant improvement in rPFS for Lynparza/abiraterone compared to placebo/abiraterone was observed in the intention to treat (ITT) population. In an exploratory analysis in the subgroup of 711 patients without an identified BRCAm, the rPFS hazard ratio was 0.77 (95% CI: 0.63, 0.96) and the OS hazard ratio was 0.92 (95% CI: 0.74, 1.14), indicating that the improvement in the ITT population was primarily attributed to the results seen in the subgroup of patients with BRCAm.<br>Results of an exploratory analysis in the subgroup of 85 patients on PROpel with BRCAm are summarized in Table 31 and Figure 12.<br>Results from the BICR assessment were consistent with the investigator-assessed rPFS results.
**208558, 05/31//2023**	**Olaparib (2)**	Oncology	ERBB2 (HER2)	Indications and Usage, Dosage and Administration, Adverse Reactions, Clinical Studies	**1 INDICATIONS AND USAGE**<br>**1.5 Adjuvant Treatment of Germline BRCA-mutated HER2-negative High Risk Early Breast Cancer**<br>Lynparza is indicated for the adjuvant treatment of adult patients with deleterious or suspected deleterious gBRCAm human epidermal growth factor receptor 2 (HER2)-negative high risk early breast cancer who have been treated with neoadjuvant or adjuvant chemotherapy. Select patients for therapy based on an FDA-approved companion diagnostic for Lynparza [see Dosage and Administration (2.1)]<br>**1.6 Germline BRCA-mutated HER2-negative Metastatic Breast Cancer**<br>Lynparza is indicated for the treatment of adult patients with deleterious or suspected deleterious gBRCAm, HER2-negative metastatic breast cancer, who have been treated with chemotherapy in the neoadjuvant, adjuvant, or metastatic setting. Patients with hormone receptor (HR)-positive breast cancer should have been treated with a prior endocrine therapy or be considered inappropriate for endocrine therapy. Select patients for therapy based on an FDA-approved companion diagnostic for Lynparza [see Dosage and Administration (2.1)].<br><br>**2 DOSAGE AND ADMINISTRATION**<br>**2.1 Patient Selection**<br>Information on FDA-approved tests for the detection of genetic mutations is available at http://www.fda.gov/companiondiagnostics. Select patients for treatment with Lynparza based on the presence of deleterious or suspected deleterious HRR gene mutations, including BRCA mutations, or genomic instability based on the indication, biomarker, and sample type (Table 1). (Table 1).<br>**2.2 Recommended Dosing**<br>Adjuvant Treatment of Germline BRCA-mutated HER2-negative High Risk Early Breast Cancer<br>Continue treatment for a total of 1 year, or until disease recurrence, or unacceptable toxicity, whichever occurs first. Patients receiving Lynparza for hormone receptor positive HER2-negative breast cancer should continue concurrent treatment with endocrine therapy as per current clinical practice guidelines.<br>Recurrent Ovarian Cancer, Germline BRCAm Advanced Ovarian Cancer, HER2-negative Metastatic Breast Cancer, Metastatic Pancreatic Adenocarcinoma, and HRR Gene-mutated Metastatic Castration-Resistant Prostate Cancer<br>Continue treatment until disease progression or unacceptable toxicity for:<br>• Maintenance treatment of recurrent ovarian cancer<br>• Advanced germline BRCA-mutated ovarian cancer<br>• Germline BRCA-mutated HER-2 negative metastatic breast cancer<br>• First-line maintenance treatment of germline BRCA-mutated metastatic pancreatic adenocarcinoma.<br>• HRR gene-mutated metastatic castration-resistant prostate cancer Patients receiving<br>Lynparza for mCRPC should also receive a gonadotropin-releasing hormone (GnRH) analog concurrently or should have had bilateral orchiectomy.<br><br>**6 ADVERSE REACTIONS**<br>Treatment of gBRCAm HER2-negative Metastatic Breast Cancer<br>OlympiAD<br>The safety of Lynparza tablets as monotherapy was also evaluated in gBRCAm patients with HER2- negative metastatic breast cancer who had previously received up to two lines of chemotherapy for the treatment of metastatic disease in OlympiAD. (…)<br><br>**14 CLINICAL STUDIES**<br>**14.5 Adjuvant Treatment of Germline BRCA-mutated HER2-negative High Risk Early Breast Cancer**<br>The efficacy of Lynparza was evaluated in OlympiA (NCT02032823), a randomized (1:1), double-blind, placebo-controlled, international study in patients with gBRCAm HER2-negative high risk early breast cancer who had completed definitive local treatment and neoadjuvant or adjuvant chemotherapy. (See Table 25)

* Therapeutic areas do not necessarily reflect the CDER review division.
† Representative biomarkers are listed based on standard nomenclature as per the Human Genome Organization (HUGO) symbol and/or simplified descriptors using other common conventions. Listed biomarkers do not necessarily reflect the terminology used in labeling. The term “Nonspecific” is provided when labeling does not explicitly identify the specific biomarker(s) or when the biomarker is represented by a molecular phenotype or gene signature, and in some cases the biomarker was inferred based on the labeling language.
‡ Referenced figures and tables may be found in the labeling available at Drugs@FDA.
Blue text represents the most recent addtions and/or changes since last posted version.

NDA/ANDA/BLA Number, Label Version Date	Drug	Therapeutic Area*	Biomarker†	Labeling Sections	Labeling Text‡
					4 patients who received prior adjuvant chemotherapy: patients with TNBC must have had node positive disease or node negative disease with a ≥2cm primary tumor; patients with hormone receptor positive, HER2-negative breast cancer must have had ≥4 pathologically confirmed positive lymph nodes. (…)<br>Patients enrolled based on local gBRCA test results provided a sample for retrospective confirmatory central testing with BRACAnalysis®. Out of 1836 patients enrolled into OlympiA, 1623 were confirmed as gBRCAm by Myriad BRACAnalysis®, either prospectively or retrospectively. (…)<br>**14.6 Treatment of Germline BRCA-mutated HER2-negative Metastatic Breast Cancer**<br>The efficacy of Lynparza was evaluated in OlympiAD (NCT02000622), an open-label randomized (2:1) study in patients with gBRCAm HER2-negative metastatic breast cancer. Patients were required to have received treatment with an anthracycline (unless contraindicated) and a taxane, in the neoadjuvant, adjuvant or metastatic setting. (…)
208558, 05/31//2023	Olaparib (3)	Oncology	ESR, PGR (Hormone Receptor)	Indications and Usage, Clinical Studies	**1 INDICATIONS AND USAGE**<br>**1.5 Germline BRCA-mutated HER2-negative Metastatic Breast Cancer**<br>Lynparza is indicated for the treatment of adult patients with deleterious or suspected deleterious gBRCAm, HER2-negative metastatic breast cancer, who have been treated with chemotherapy in the neoadjuvant, adjuvant, or metastatic setting. Patients with hormone receptor (HR)-positive breast cancer should have been treated with a prior endocrine therapy or be considered inappropriate for endocrine therapy. Select patients for therapy based on an FDA-approved companion diagnostic for Lynparza [see Dosage and Administration (2.1)].<br><br>**14 CLINICAL STUDIES**<br>**14.5 Adjuvant Treatment of Germline BRCA-mutated HER2-negative High Risk Early Breast Cancer**<br>The efficacy of Lynparza was evaluated in OlympiA (NCT02032823), a randomized (1:1), double-blind, placebo-controlled, international study in patients with gBRCAm HER2-negative high risk early breast cancer who had completed definitive local treatment and neoadjuvant or adjuvant chemotherapy. (See Table 25)<br>5 patients who received prior adjuvant chemotherapy: patients with TNBC must have had node positive disease or node negative disease with a ≥2cm primary tumor; patients with hormone receptor positive, HER2-negative breast cancer must have had ≥4 pathologically confirmed positive lymph nodes. (…)<br>Patients enrolled based on local gBRCA test results provided a sample for retrospective confirmatory central testing with BRACAnalysis®. Out of 1836 patients enrolled into OlympiA, 1623 were confirmed as gBRCAm by Myriad BRACAnalysis®, either prospectively or retrospectively. (…)<br>**14.6 Treatment of Germline BRCA-mutated HER2-negative Metastatic Breast Cancer**<br>The efficacy of Lynparza was evaluated in OlympiAD (NCT02000622), an open-label randomized (2:1) study in patients with gBRCAm HER2-negative metastatic breast cancer. Patients were required to have received treatment with an anthracycline (unless contraindicated) and a taxane, in the neoadjuvant, adjuvant or metastatic setting. Patients with hormone receptor-positive disease must have progressed on at least 1 endocrine therapy (adjuvant or metastatic), or have disease that the treating healthcare provider believed to be inappropriate for endocrine therapy. (See Table 23) (…)<br>Randomization was stratified by prior use of chemotherapy for metastatic disease (yes vs no), hormone receptor status (hormone receptor positive vs triple negative), and previous use of platinum-based chemotherapy (yes vs no). The major efficacy outcome measure was PFS assessed by blinded independent central review (BICR) using RECIST version 1.1. (…)<br>A total of 302 patients were randomized, 205 to Lynparza and 97 to chemotherapy. Among the 205 patients treated with Lynparza, the median age was 44 years (range: 22 to 76), 65% were White, 4% were males and all the patients had an ECOG PS of 0 or 1. Approximately 50% of patients had triple-negative tumors and 50% had estrogen receptor and/or progesterone receptor positive tumors and the proportions were balanced across treatment arms. Patients in each treatment arm had received a median of 1 prior chemotherapy regimen for metastatic disease; approximately 30% had not received a prior chemotherapy regimen for metastatic breast cancer. Twenty-one percent of patients in the Lynparza arm and 14% in the chemotherapy arm had received platinum therapy for metastatic disease. Seven percent of patients in each treatment arm had received platinum therapy for localized disease.
208558, 05/31//2023	Olaparib (4)	Oncology	BRCA, Genomic Instability (Homologous Recombination Deficiency)	Indications and Usage, Dosage and Administration, Adverse Reactions, Clinical Studies	**1 INDICATIONS AND USAGE**<br>**1.2 First-line Maintenance Treatment of HRD-positive Advanced Ovarian Cancer in Combination with Bevacizumab**<br>Lynparza is indicated in combination with bevacizumab for the maintenance treatment of adult patients with advanced epithelial ovarian, fallopian tube or primary peritoneal cancer who are in complete or partial response to first-line platinum-based chemotherapy and whose cancer is associated with homologous recombination deficiency (HRD)-positive status defined by either:<br>• a deleterious or suspected deleterious BRCA mutation, and/or<br>• genomic instability.<br>Select patients for therapy based on an FDA-approved companion diagnostic for Lynparza [see Dosage and Administration (2.1)].<br><br>**2 DOSAGE AND ADMINISTRATION**<br>**2.1 Patient Selection**<br>Information on FDA-approved tests for the detection of genetic mutations is available at http://www.fda.gov/companiondiagnostics.<br>Select patients for treatment with Lynparza based on the presence of deleterious or suspected deleterious HRR gene mutations, including BRCA mutations, or genomic instability based on the indication, biomarker, and sample type (Table 1).<br>**2.2 Recommended Dosage**<br>First-Line Maintenance Treatment of HRD-positive Advanced Ovarian Cancer in Combination with Bevacizumab<br>Continue Lynparza treatment until disease progression, unacceptable toxicity, or completion of 2 years of treatment. Patients with a complete response (no radiological evidence of disease) at 2 years should stop treatment. Patients with evidence of disease at 2 years, who in the opinion of the treating healthcare

* Therapeutic areas do not necessarily reflect the CDER review division.
† Representative biomarkers are listed based on standard nomenclature as per the Human Genome Organization (HUGO) symbol and/or simplified descriptors using other common conventions. Listed biomarkers do not necessarily reflect the terminology used in labeling. The term “Nonspecific” is provided when labeling does not explicitly identify the specific biomarker(s) or when the biomarker is represented by a molecular phenotype or gene signature, and in some cases the biomarker was inferred based on the labeling language.
‡ Referenced figures and tables may be found in the labeling available at Drugs@FDA.
Blue text represents the most recent addtions and/or changes since last posted version.

NDA/ANDA/BLA Number, Label Version Date	Drug	Therapeutic Area*	Biomarker†	Labeling Sections	Labeling Text‡
					provider can derive further benefit from continuous Lynparza treatment, can be treated beyond 2 years. When used with Lynparza, the recommended dose of bevacizumab is 15 mg/kg every three weeks. Bevacizumab should be given for a total of 15 months including the period given with chemotherapy and given as maintenance. Refer to the Prescribing Information for bevacizumab when used in combination with Lynparza for more information.<br>Recurrent Ovarian Cancer, Germline BRCAm Advanced Ovarian Cancer, HER2-negative Metastatic Breast Cancer, Metastatic Pancreatic Adenocarcinoma, and HRR Gene-mutated Metastatic CastrationResistant Prostate Cancer<br>Continue treatment until disease progression or unacceptable toxicity for:<br>• Maintenance treatment of recurrent ovarian cancer<br>• Advanced germline BRCA-mutated ovarian cancer<br>• Germline BRCA-mutated HER-2 negative metastatic breast cancer<br>• First-line maintenance treatment of germline BRCA-mutated metastatic pancreatic adenocarcinoma.<br>• HRR gene-mutated metastatic castration-resistant prostate cancer<br>Patients receiving Lynparza for mCRPC should also receive a gonadotropin-releasing hormone (GnRH) analog concurrently or should have had bilateral orchiectomy.<br><br>**6 ADVERSE REACTIONS**<br>**6.1 Clinical Trial Experience**<br>First-line Maintenance Treatment of HRD-positive Advanced Ovarian Cancer in Combination with Bevacizumab<br>PAOLA-1<br>The safety of Lynparza in combination with bevacizumab for the maintenance treatment of patients with advanced ovarian cancer following first-line treatment containing platinum-based chemotherapy and bevacizumab was investigated in PAOLA-1 [see Clinical Studies (14.2)]. (…)<br><br>**14 CLINICAL STUDIES**<br>**14.2 First-line Maintenance Treatment of HRD-positive Advanced Ovarian Cancer in Combination with Bevacizumab**<br>*PAOLA-1*<br>PAOLA-1 (NCT02477644) was a randomized, double-blind, placebo-controlled, multi-center trial that compared the efficacy of Lynparza in combination with bevacizumab versus placebo/bevacizumab for the maintenance treatment of advanced high-grade epithelial ovarian cancer, fallopian tube or primary peritoneal cancer following first-line platinum-based chemotherapy and bevacizumab. Randomization was stratified by first-line treatment outcome (timing and outcome of cytoreductive surgery and response to platinum-based chemotherapy) and tBRCAm status, determined by prospective local testing. All available clinical samples were retrospectively tested with Myriad myChoice® CDx. (…)<br>(…) Demographics and baseline disease characteristics were balanced and comparable between the study and placebo arms in the Intention to Treat (ITT) population and also in the HRD-positive subgroup.<br>Efficacy results from a biomarker subgroup analysis of 387 patients with HRD-positive tumors, identified post-randomization using the Myriad myChoice® HRD Plus tumor test, who received Lynparza/bevacizumab (n=255) or placebo/bevacizumab (n=132), are summarized in Table 19 and Figure 2. Results from a blinded independent review of PFS were consistent. Overall survival data in this subpopulation were immature with 16% deaths. (See Table 19 and Figure 2)<br>(…)
**208558, 05/31//2023**	Olaparib (5)	Oncology	Homologous Recombination Repair	Indications and Usage, Dosage and Administration, Adverse Reactions, Clinical Studies	**1 INDICATIONS AND USAGE**<br>**1.7 HRR Gene-mutated Metastatic Castration-Resistant Prostate Cancer**<br>Lynparza is indicated for the treatment of adult patients with deleterious or suspected deleterious germline or somatic homologous recombination repair (HRR) gene-mutated metastatic castration-resistant prostate cancer (mCRPC) who have progressed following prior treatment with enzalutamide or abiraterone. Select patients for therapy based on an FDA-approved companion diagnostic for Lynparza [see Dosage and Administration (2.1)].<br><br>**2 DOSAGE AND ADMINISTRATION**<br>**2.1 Patient Selection**<br>Information on FDA-approved tests for the detection of genetic mutations is available at http://www.fda.gov/companiondiagnostics.<br>Select patients for treatment with Lynparza based on the presence of deleterious or suspected deleterious HRR gene mutations, including BRCA mutations, or genomic instability based on the indication, biomarker, and sample type (Table 1).<br>**2.2 Recommended Dosage**<br>Recurrent Ovarian Cancer, Germline BRCAm Advanced Ovarian Cancer, HER2-negative Metastatic Breast Cancer, Metastatic Pancreatic Adenocarcinoma, and HRR Gene-mutated Metastatic Castration-Resistant Prostate Cancer<br>Continue treatment until disease progression or unacceptable toxicity for:<br>• Maintenance treatment of recurrent ovarian cancer<br>• Advanced germline BRCA-mutated ovarian cancer<br>• Germline BRCA-mutated HER-2 negative metastatic breast cancer<br>• First-line maintenance treatment of germline BRCA-mutated metastatic pancreatic adenocarcinoma.<br>• HRR gene-mutated metastatic castration-resistant prostate cancer Patients receiving<br>Lynparza for mCRPC should also receive a gonadotropin-releasing hormone (GnRH) analog concurrently or should have had bilateral orchiectomy.<br><br>**6 ADVERSE REACTIONS**<br>**6.1 Clinical Trial Experience**

NDA/ANDA/BLA Number, Label Version Date	Drug	Therapeutic Area*	Biomarker†	Labeling Sections	Labeling Text‡
					**HRR Gene-mutated Metastatic Castration-Resistant Prostate Cancer**<br>*PROfound*<br>The safety of Lynparza as monotherapy was evaluated in patients with mCRPC and HRR gene mutations who have progressed following prior treatment with enzalutamide or abiraterone in PROfound [see Clinical Studies (14.7)].<br><br>**14 CLINICAL STUDIES**<br>**14.7 HRR Gene-mutated Metastatic Castration-Resistant Prostate Cancer**<br>The efficacy of Lynparza was evaluated in PROfound (NCT02987543), randomized, open-label, multicenter trial that evaluated the efficacy of Lynparza 300 mg twice daily versus a comparator arm of investigator's choice of enzalutamide or abiraterone acetate in men with metastatic castration-resistant prostate cancer (mCRPC). All patients received a GnRH analog or had prior bilateral orchiectomy. Patients needed to have progressed on prior enzalutamide or abiraterone for the treatment of metastatic prostate cancer and/or CRPC and have a tumor mutation in one of 15 genes involved in the homologous recombination repair (HRR) pathway.<br>Patients were divided into two cohorts based on HRR gene mutation status. Patients with mutations in either BRCA1, BRCA2, or ATM were randomized in Cohort A; patients with mutations among 12 other genes involved in the HRR pathway (BARD1, BRIP1, CDK12, CHEK1, CHEK2, FANCL, PALB2, PPP2R2A, RAD51B, RAD51C, RAD51D, or RAD54L) were randomized in Cohort B; patients with comutations (BRCA1, BRCA2, or ATM plus a Cohort B gene) were assigned to Cohort A. Although patients with PPP2R2A gene mutations were enrolled in the trial, Lynparza is not indicated for the treatment of patients with this gene mutation due to unfavorable risk-benefit. (…)<br>Patients with HRR gene mutations were identified by tissue-based testing using the Foundation Medicine FoundationOne® clinical trial HRR assay performed at a central laboratory.<br>Determination of deleterious or suspected deleterious somatic or germline HRR mutation status in line with the FDA approved mutation classification and testing criteria for the Foundation Medicine F1CDx tissue-based assay and assessment of the germline-BRCA status using the Myriad BRACAnalysis CDx blood-based assay was performed retrospectively. Representation of individual gene mutations by cohort is provided in Table 25. No patients were enrolled who had mutations in two of the 15 pre-specified HRR genes: FANCL and RAD51C. (See Table 25) (…)<br>Response data by HRR mutations for patients in the Lynparza arm are presented in Table 27. In the comparator arm of Cohorts A and B, a total of three patients achieved a partial response, including one patient with an ATM mutation alone and 2 patients with co-occurring mutations (one with PALB2+PPP2R2A and one with CDK12+PALB2). (See Table 27) (…)
**208558, 05/31//2023**	Olaparib (6)	Oncology	PPP2R2A	Clinical Studies	**14 CLINICAL STUDIES**<br>**14.7 HRR Gene-mutated Metastatic Castration-Resistant Prostate Cancer**<br>(…) Patients were divided into two cohorts based on HRR gene mutation status. Patients with mutations in either BRCA1, BRCA2, or ATM were randomized in Cohort A; patients with mutations among 12 other genes involved in the HRR pathway (BARD1, BRIP1, CDK12, CHEK1, CHEK2, FANCL, PALB2, PPP2R2A, RAD51B, RAD51C, RAD51D, or RAD54L) were randomized in Cohort B; patients with comutations (BRCA1, BRCA2, or ATM plus a Cohort B gene) were assigned to Cohort A. Although patients with PPP2R2A gene mutations were enrolled in the trial, Lynparza is not indicated for the treatment of patients with this gene mutation due to unfavorable risk-benefit. (See Tables 25 and 26) (…)<br>Response data by HRR mutations for patients in the Lynparza arm are presented in Table 27. In the comparator arm of Cohorts A and B, a total of three patients achieved a partial response, including one patient with an ATM mutation alone and 2 patients with co-occurring mutations (one with PALB2+PPP2R2A and one with CDK12+PALB2). (See Table 27) (…)
**761038, 10/19/2016**	Olaratumab	Oncology	PDGFRA	Clinical Studies	**14 CLINICAL STUDIES**<br>The efficacy of LARTRUVO was demonstrated in Trial 1, an open-label, randomized, active-controlled study. Eligible patients were required to have soft tissue sarcoma not amenable to curative treatment with surgery or radiotherapy, a histologic type of sarcoma for which an anthracycline-containing regimen was appropriate but had not been administered, ECOG PS of 0-2, and tumor specimen available for assessment of PDGFR-α expression by an investigational use assay. Patients were randomized (1:1) to receive LARTRUVO in combination with doxorubicin or doxorubicin as a single agent. PDGFR-α expression (positive versus negative), number of previous lines of treatment (0 versus 1 or more), histological tumor type (leiomyosarcoma versus synovial sarcoma versus all others), and ECOG PS (0 or 1 versus 2) were used to allocate patients in the randomization. (…)
**210730, 08/07/2020**	Oliceridine	Anesthesiology	CYP2D6	Warnings and Precautions, Drug Interactions, Use in Specific Populations, Clinical Pharmacology	**5 WARNINGS AND PRECAUTIONS**<br>**5.6 Risk of Use in Patients with Decreased Cytochrome P450 2D6 Function or Concomitant Use or Discontinuation with Cytochrome P450 3A4 Inhibitors and Inducers**<br>Risk of Increased Oliceridine Plasma Concentrations<br>Increased plasma concentrations of oliceridine, which may result in prolonged opioid adverse reactions and exacerbated respiratory depression, may occur when OLINVYK is used under the following conditions:<br>In patients with decreased Cytochrome P450 (CYP) 2D6 function (poor metabolizers of CYP2D6 or normal metabolizers taking moderate or strong CYP2D6 inhibitors) [See Drug Interactions (7) and Use in Specific Populations (8.8)].<br>• In patients taking a moderate or strong CYP3A4 Inhibitor<br>• In patients with decreased CYP2D6 function who are also receiving a moderate or strong CYP3A4 inhibitor<br>• Discontinuation of a CYP3A4 inducer (…)<br><br>**7 DRUG INTERACTIONS**<br>(…) Patients who are CYP2D6 normal metabolizers taking a CYP2D6 inhibitor, and a strong CYP3A4 inhibitor (or discontinuation of CYP3A4 inducers) may require less frequent dosing. Patients who are known CYP2D6 poor metabolizers and taking a CYP3A4 inhibitor (or discontinuation of CYP3A4 inducers) may

NDA/ANDA/BLA Number, Label Version Date	Drug	Therapeutic Area*	Biomarker†	Labeling Sections	Labeling Text‡
					require less frequent dosing. These patients should be closely monitored for respiratory depression and sedation at frequent intervals, and subsequent doses should be based on the patient's severity of pain and response to treatment. (…)<br><br>**8 USE IN SPECIFIC POPULATIONS**<br>**8.8 Poor Metabolizers of CYP2D6 Substrates**<br>In patients who are known or suspected to be poor CYP2D6 metabolizers, based on genotype or previous history/experience with other CYP2D6 substrates, less frequent dosing of OLINVYK may be required. These patients should be closely monitored, and subsequent doses should be based on the patient's severity of pain and response to treatment. [See Warnings and Precautions (5.6), Clinical Pharmacology (12.5)].<br><br>**12 CLINICAL PHARMACOLOGY**<br>**12.3 Pharmacokinetics**<br>Drug Interaction Studies<br>In vitro studies suggest that oliceridine is metabolized primarily by the CYP3A4 and CYP2D6 P450 hepatic enzymes, with minor contributions from CYP2C9 and CYP2C19. Inhibition studies using selective inhibitors of all the major CYP enzymes show that only the inhibition of CYP3A4 and CYP2D6 significantly affects the metabolism of oliceridine in these assays, suggesting that the contribution of CYP2C9 and CYP2C19 to the metabolism of oliceridine is minor.<br>The effect of concomitant administration of a CYP2D6 inhibitor on the pharmacokinetics of OLINVYK, although not studied, may be similar to that noted in subjects who are CYP2D6 poor metabolizers. The plasma clearance of oliceridine in CYP2D6 poor metabolizers is approximately 50% of plasma clearance in subjects who are nonpoor CYP2D6 metabolizers [See Pharmacogenomics (12.5)].<br>In healthy subjects CYP2D6 poor metabolizers (n=4) given a single 0.25 mg dose of OLINVYK after 5 days of itraconazole 200 mg QD (a strong CYP3A4 inhibitor), the total exposure (AUC) of OLINVYK was increased by approximately 80%; however, the peak concentration was not significantly affected [See Pharmacogenomics 12.5]. The mean clearance of oliceridine was reduced to approximately 30% of that observed in nonpoor metabolizers of CYP2D6 [see Drug Interactions (7)].<br>**12.5 Pharmacogenomics**<br>Oliceridine is metabolized by polymorphic enzyme CYP2D6. CYP2D6 poor metabolizers have little to no enzyme activity. Approximately 3 to 10% of Whites, 2 to 7% of African Americans, and <2% of Asians, generally lack the capacity to metabolize CYP2D6 substrates and are classified as poor metabolizers.<br>In healthy subjects who are CYP2D6 poor metabolizers, the AUC0-inf of oliceridine was approximately 2-fold higher than in subjects who are nonpoor CYP2D6 metabolizers. [see Warnings and Precautions (5.6), Use in Specific Populations (8.8)].
215814, 12/01/2022	Olutasidenib	Oncology	IDH1	Indications and Usage, Dosage and Administration, Adverse Reactions, Use in Specific Populations, Clinical Pharmacology, Clinical Studies	**1 INDICATIONS AND USAGE**<br>**Relapsed or Refractory Acute Myeloid Leukemia**<br>REZLIDHIA is indicated for the treatment of adult patients with relapsed or refractory acute myeloid leukemia (AML) with a susceptible isocitrate dehydrogenase-1 (IDH1) mutation as detected by an FDA-approved test [see Dosage and Administration (2.1), Clinical Pharmacology (12.1), and Clinical Studies (14.1)].<br><br>**2 DOSAGE AND ADMINISTRATION**<br>**2.1 Patient Selection**<br>Select patients for the treatment of relapsed or refractory AML with REZLIDHIA based on the presence of IDH1 mutations in blood or bone marrow [see Clinical Trials (14.1)]. Information on FDAapproved tests for the detection of IDH1 mutations in AML is available at http://www.fda.gov/CompanionDiagnostics.<br><br>**6 ADVERSE REACTIONS**<br>**Relapsed or Refractory AML**<br>The safety of REZLIDHIA 150 mg administered twice daily was evaluated in 153 adults with relapsed or refractory AML with an IDH1 mutation [see Clinical Studies (14.1). Among the 153 patients who received REZLIDHIA, 35% were exposed for at least 6 months and 21% were exposed for at least 1 year. The median duration of exposure to REZLIDHIA was 4.7 months (range: 0.1 to 34 months). (…)<br><br>**8 USE IN SPECIFIC POPULATIONS**<br>**8.5 Geriatric Use**<br>Among the 153 patients with relapsed or refractory AML with an IDH1 mutation treated with REZLIDHIA, 116 (76%) were 65 years of age or older and 48 (31%) were 75 years or older. No overall differences in effectiveness were observed between patients 65 years and older and younger patients. Compared to patients younger than 65 years of age, an increase in incidence of hepatotoxicity and hypertension was observed in patients □65 years of age.<br><br>**12 CLINICAL PHARMACOLOGY**<br>**12.2 Pharmacodynamics**<br>The mean [% coefficient of variation (%CV)] reduction in 2-HG plasma concentration was 59.1% (122%) by pre-dose Cycle 2 and was sustained throughout the treatment period in patients with AML and IDH1 mutations following the approved recommended olutasidenib dosage.<br><br>**14 CLINICAL STUDIES**<br>**14.1 Acute Myeloid Leukemia**<br>The efficacy of REZLIDHIA was evaluated in an open-label, single-arm, multicenter clinical trial (Study 2102-HEM-101, NCT02719574) in 147 adult patients with relapsed or refractory AML with an IDH1 mutation. Reference ID: 5086793 IDH1 mutations in blood or bone marrow were confirmed retrospectively using the Abbott RealTimeTM IDH1 Assay. REZLIDHIA was given orally at a dose of 150 mg twice daily until disease progression, development of unacceptable toxicity, or hematopoietic stem cell transplantation. Sixteen of the 147 patients (11%) underwent stem cell transplantation following REZLIDHIA treatment.

* Therapeutic areas do not necessarily reflect the CDER review division.
† Representative biomarkers are listed based on standard nomenclature as per the Human Genome Organization (HUGO) symbol and/or simplified descriptors using other common conventions. Listed biomarkers do not necessarily reflect the terminology used in labeling. The term "Nonspecific" is provided when labeling does not explicitly identify the specific biomarker(s) or when the biomarker is represented by a molecular phenotype or gene signature, and in some cases the biomarker was inferred based on the labeling language.
‡ Referenced figures and tables may be found in the labeling available at Drugs@FDA.
Blue text represents the most recent addtions and/or changes since last posted version.

NDA/ANDA/BLA Number, Label Version Date	Drug	Therapeutic Area*	Biomarker†	Labeling Sections	Labeling Text‡
					The baseline demographic and disease characteristics are shown in Table 4. (see Tables 4 and 5)
**203585, 11/26/2019**	**Omacetaxine**	Oncology	BCR-ABL1 (Philadelphia chromosome)	Clinical Studies	**14 CLINICAL STUDIES**<br>The efficacy of SYNRIBO was evaluated using a combined cohort of adult patients with CML from two trials. The combined cohort consisted of patients who had received 2 or more approved TKIs and had, at a minimum, documented evidence of resistance or intolerance to dasatinib and/or nilotinib. Resistance was defined as one of the following: no complete hematologic response (CHR) by 12 weeks (whether lost or never achieved); or no cytogenetic response by 24 weeks (i.e., 100% Ph positive [Ph+]) (whether lost or never achieved); or no major cytogenetic response (MCyR) by 52 weeks (i.e., ≥35% Ph+) (whether lost or never achieved); or progressive leukocytosis. (See Table 5)
**207931, 07/23/2018**	**Ombitasvir, Paritaprevir, and Ritonavir**	Infectious Diseases	IFNL3 (IL28B)	Clinical Studies	**14 CLINICAL STUDIES**<br>**14.2 Clinical Trial Results in Adults with Chronic GT4 HCV Infection without Cirrhosis**<br>(…) HCV GT4-infected subjects had a median age of 51 years (range: 19 to 70); 64% were treatment-naïve, 17% were prior pegIFN/RBV null responders; 7% were prior pegIFN/RBV partial responders, 13% were prior pegIFN/RBV relapsers; 65% were male; 9% were Black; 14% had a body mass index at least 30 kg/m2 ; 70% had baseline HCV RNA levels at least 800,000 IU/mL; 79% had IL28B (rs12979860) non-CC genotype; 7% had bridging fibrosis (F3). (…)<br>**14.3 Clinical Trial Results in Adults with Chronic GT4 HCV Infection with Compensated Cirrhosis**<br>**(…)** Of the 59 subjects in the 12 week arm, median age was 56 years (range: 43 to 81); 51% were treatment-naïve, 29% were prior pegIFN/RBV null responders; 8% were prior pegIFN/RBV partial responders, 12% were prior pegIFN/RBV relapsers; 76% were male; 17% were Black; 29% had a body mass index of at least 30 kg/m2 ; 76% had baseline HCV RNA levels of at least 800,000 IU per mL; 86% had IL28B (rs12979860) non-CC genotype; 12% had platelet counts of less than 90 x 109 per L; and 5% had albumin less than 3.5 mg per dL. (…)
**022056, 08/21/2018**	**Omeprazole**	Gastroenterology	CYP2C19	Drug Interactions, Clinical Pharmacology	**7 DRUG INTERACTIONS**<br>*Tacrolimus*<br>Potential for increased exposure of tacrolimus, especially in transplant patients who are intermediate or poor metabolizers of CYP2C19. (See Table 3)<br><br>**12 CLINICAL PHARMACOLOGY**<br>**12.5 Pharmacogenomics**<br>CYP2C19, a polymorphic enzyme, is involved in the metabolism of omeprazole. The CYP2C19*1 allele is fully functional while the CYP2C19*2 and *3 alleles are nonfunctional. There are other alleles associated with no or reduced enzymatic function. Patients carrying two fully functional alleles are extensive metabolizers and those carrying two loss-of-function alleles are poor metabolizers. In extensive metabolizers, omeprazole is primarily metabolized by CYP2C19. The systemic exposure to omeprazole varies with a patient's metabolism status: poor metabolizers > intermediate metabolizers > extensive metabolizers. Approximately 3% of Caucasians and 15 to 20% of Asians are CYP2C19 poor metabolizers.<br>In a pharmacokinetic study of single 20 mg omeprazole dose, the AUC of omeprazole in Asian subjects was approximately four-fold of that in Caucasians [see Dosage and Administration (2.1), Use in Specific Populations (8.7)].
**020007, 03/08/2017**	**Ondansetron**	Gastroenterology	CYP2D6	Clinical Pharmacology	**12 CLINICAL PHARMACOLOGY**<br>**12.3 Pharmacokinetics**<br>*Metabolism*<br>(…) The pharmacokinetics of intravenous ondansetron did not differ between subjects who were poor metabolisers of CYP2D6 and those who were extensive metabolisers of CYP2D6, further supporting the limited role of CYP2D6 in ondansetron disposition in vivo. (…)

* Therapeutic areas do not necessarily reflect the CDER review division.
† Representative biomarkers are listed based on standard nomenclature as per the Human Genome Organization (HUGO) symbol and/or simplified descriptors using other common conventions. Listed biomarkers do not necessarily reflect the terminology used in labeling. The term "Nonspecific" is provided when labeling does not explicitly identify the specific biomarker(s) or when the biomarker is represented by a molecular phenotype or gene signature, and in some cases the biomarker was inferred based on the labeling language.
‡ Referenced figures and tables may be found in the labeling available at Drugs@FDA.
Blue text represents the most recent addtions and/or changes since last posted version.

Table of Pharmacogenomic Biomarkers in Drug Labeling
Last Updated: 06/2024

NDA/ANDA/BLA Number, Label Version Date	Drug	Therapeutic Area*	Biomarker†	Labeling Sections	Labeling Text‡
**208065, 04/29/2024**	**Osimertinib**	Oncology	EGFR	Indications and Usage, Dosage and Administration, Adverse Reactions, Use in Specific Populations, Clinical Studies	**1 INDICATIONS AND USAGE**<br>**1.1 Adjuvant Treatment of EGFR Mutation-Positive Non-Small Cell Lung Cancer (NSCLC)**<br>TAGRISSO is indicated as adjuvant therapy after tumor resection in adult patients with non-small cell lung cancer (NSCLC) whose tumors have epidermal growth factor receptor (EGFR) exon 19 deletions or exon 21 L858R mutations, as detected by an FDA-approved test [see Dosage and Administration (2.2)].<br>**1.2 First-line Treatment of EGFR Mutation-Positive Metastatic NSCLC**<br>TAGRISSO is indicated for the first-line treatment of adult patients with metastatic NSCLC whose tumors have EGFR exon 19 deletions or exon 21 L858R mutations, as detected by an FDA-approved test [see Dosage and Administration (2.2)].<br>**1.3 First-line Treatment of EGFR Mutation-Positive Locally Advanced or Metastatic NSCLC**<br>TAGRISSO in combination with pemetrexed and platinum-based chemotherapy is indicated for the first-line treatment of adult patients with locally advanced or metastatic NSCLC whose tumors have EGFR exon 19 deletions or exon 21 L858R mutations, as detected by an FDA-approved test [see Dosage and Administration (2.2)].<br>**1.4 Previously Treated EGFR T790M Mutation-Positive Metastatic NSCLC**<br>TAGRISSO is indicated for the treatment of adult patients with metastatic EGFR T790M mutation-positive NSCLC, as detected by an FDA-approved test, whose disease has progressed on or after EGFR tyrosine kinase inhibitor (TKI) therapy [see Dosage and Administration (2.2)].<br><br>**2 DOSAGE AND ADMINISTRATION**<br>**2.2 Patient Selection**<br>Adjuvant Treatment of EGFR Mutation-Positive NSCLC<br>Select patients with resectable tumors for the adjuvant treatment of NSCLC with TAGRISSO based on the presence of EGFR exon 19 deletions or exon 21 L858R mutations in tumor specimens [see Clinical Studies (14.1)].<br>First-line Treatment of EGFR Mutation-Positive Metastatic NSCLC<br>Select patients for the first-line treatment of locally advanced or metastatic EGFR-positive NSCLC with TAGRISSO based on the presence of EGFR exon 19 deletions or exon 21 L858R mutations in tumor or plasma specimens [see Clinical Studies (14.2)]. If these mutations are not detected in a plasma specimen, test tumor tissue if feasible.<br>First-line Treatment of EGFR Mutation-Positive Locally Advanced or Metastatic NSCLC<br>Select patients for the first-line treatment of locally advanced or metastatic EGFR-positive NSCLC with TAGRISSO in combination with pemetrexed and platinum-based chemotherapy, based on the presence of EGFR exon 19 deletions or exon 21 L858R mutations in tumor or plasma specimens [see Clinical Studies (14.3)]. If these mutations are not detected in a plasma specimen, test tumor tissue if feasible.<br>Previously Treated EGFR T790M Mutation-Positive Metastatic NSCLC<br>Select patients for the treatment of metastatic EGFR T790M mutation-positive NSCLC with TAGRISSO following progression on or after EGFR TKI therapy based on the presence of an EGFR T790M mutation in tumor or plasma specimens [see Clinical Studies (14.4)]. Testing for the presence of the T790M mutation in plasma specimens is recommended only in patients for whom a tumor biopsy cannot be obtained. If this mutation is not detected in a plasma specimen, re-evaluate the feasibility of biopsy for tumor tissue testing.<br>Information on FDA-approved tests for the detection of EGFR mutations is available at http://www.fda.gov/companiondiagnostics.<br>**2.3 Recommended Dosage and Administration**<br>See Table 1<br><br>**6 ADVERSE REACTIONS**<br>**6.1 Clinical Trials Experience**<br>(…)The data in the WARNINGS AND PRECAUTIONS section reflect exposure to TAGRISSO in 1813 patients with EGFR mutation-positive NSCLC who received TAGRISSO monotherapy at the recommended dose of 80 mg once daily in four randomized, controlled trials [ADAURA (n=337), FLAURA (n=338), FLAURA2 (monotherapy arm; n=275), and AURA3 (n=279)], two single arm trials [AURA Extension (n=201) and AURA2 (n=210)], and one dose-finding study, AURA1 (n=173) [see WARNINGS AND PRECAUTIONS (5)]. Among 1813 patients who received TAGRISSO monotherapy, 82% were exposed for 6 months or longer and 67% were exposed for greater than one year. In this pooled safety population, the most common adverse reactions in ≥20% of 1813 patients who received TAGRISSO monotherapy were diarrhea (47%), rash (46%), musculoskeletal pain (38%), nail toxicity (34%), dry skin (32%), stomatitis (24%), and fatigue (21%). The most common laboratory abnormalities in ≥20% of 1813 patients who received TAGRISSO monotherapy were leukopenia (65%), lymphopenia (64%), thrombocytopenia (53%), anemia (52%), and neutropenia (36%).<br>The data described below reflect exposure to TAGRISSO (80 mg daily) in 337 patients with EGFR mutation-positive resectable NSCLC, and 833 patients with EGFR mutation-positive locally advanced or metastatic NSCLC in four randomized, controlled trials [ADAURA (n=337), FLAURA (n=279), FLAURA2 (monotherapy arm; n=275), and AURA3 (n=279)]. The data also reflect exposure to TAGRISSO at the recommended dose of 80 mg daily given in combination with pemetrexed and platinum-based chemotherapy in 276 patients with EGFR mutation-positive locally advanced or metastatic NSCLC in one randomized controlled trial [FLAURA2 (n=276)]. Patients with a history of interstitial lung disease, drug induced interstitial disease or radiation pneumonitis that required steroid treatment, serious arrhythmia or baseline QTc interval greater than 470 msec on electrocardiogram were excluded from enrollment in these studies.<br>Adjuvant Treatment of EGFR Mutation-Positive NSCLC - Monotherapy<br>The safety of TAGRISSO was evaluated in ADAURA, a randomized, double-blind, placebo-controlled trial for the adjuvant treatment of patients with EGFR exon 19 deletions or exon 21 L858R mutation-positive NSCLC who had complete tumor resection, with or without prior adjuvant chemotherapy. At time of DFS analysis, the median duration of exposure to TAGRISSO was 22.5 months. (…)<br>Previously Untreated EGFR Mutation-Positive Metastatic Non-Small Cell Lung Cancer - Monotherapy

* Therapeutic areas do not necessarily reflect the CDER review division.
† Representative biomarkers are listed based on standard nomenclature as per the Human Genome Organization (HUGO) symbol and/or simplified descriptors using other common conventions. Listed biomarkers do not necessarily reflect the terminology used in labeling. The term "Nonspecific" is provided when labeling does not explicitly identify the specific biomarker(s) or when the biomarker is represented by a molecular phenotype or gene signature, and in some cases the biomarker was inferred based on the labeling language.
‡ Referenced figures and tables may be found in the labeling available at Drugs@FDA.
Blue text represents the most recent addtions and/or changes since last posted version.

NDA/ANDA/BLA Number, Label Version Date	Drug	Therapeutic Area*	Biomarker†	Labeling Sections	Labeling Text‡
					The safety of TAGRISSO was evaluated in FLAURA, a multicenter international double-blind randomized (1:1) active-controlled trial conducted in 556 patients with EGFR exon 19 deletion or exon 21 L858R mutation-positive, unresectable or metastatic NSCLC who had not received previous systemic treatment for advanced disease. The median duration of exposure to TAGRISSO was 16.2 months.<br>Previously Untreated EGFR Mutation-Positive Locally Advanced or Metastatic Non-Small Cell Lung Cancer – TAGRISSO in Combination with Pemetrexed and Platinum-based Chemotherapy<br>The safety of TAGRISSO in combination with pemetrexed and platinum-based chemotherapy was evaluated in FLAURA2, a multicenter international open-label, randomized (1:1), active-controlled trial conducted in 557 patients with EGFR exon 19 deletion or exon 21 L858R mutation-positive, locally advanced or metastatic NSCLC who had not received previous systemic treatment for advanced disease. The median duration of exposure to TAGRISSO in combination with pemetrexed and platinum-based chemotherapy was 22.3 months and the median duration of exposure to TAGRISSO monotherapy was 19.3 months.<br>Previously Treated EGFR T790M Mutation-Positive Metastatic Non-Small Cell Lung Cancer – Monotherapy<br>The safety of TAGRISSO was evaluated in AURA3, a multicenter international open label randomized (2:1) controlled trial conducted in 419 patients with unresectable or metastatic EGFR T790M mutation-positive NSCLC who had progressive disease following first line EGFR TKI treatment. A total of 279 patients received TAGRISSO 80 mg orally once daily until intolerance to therapy, disease progression, or investigator determination that the patient was no longer benefiting from treatment.<br><br>**8 USE IN SPECIFIC POPULATIONS**<br>**8.5 Geriatric Use**<br>Monotherapy<br>Of the 1813 patients with EGFR exon 19 deletion or exon 21 L858R mutation-positive NSCLC who were treated with TAGRISSO monotherapy, 770 patients were ≥65 years and 207 patients were ≥75 years of age [see Adverse Reactions (6.1)]. Exploratory analysis suggests a higher incidence of Grade 3 or higher adverse reactions (43% vs 33%) and more frequent dosage modifications for adverse reactions (34% vs 23%) in patients 65 years or older as compared to those younger than 65 years. No overall differences in safety or effectiveness were observed between patients 65 years or older and younger patients.<br>TAGRISSO in Combination with Pemetrexed and Platinum-based Chemotherapy<br>Of the 276 patients with EGFR exon 19 deletion or exon 21 L858R mutation-positive, locally advanced or metastatic NSCLC treated with TAGRISSO in combination with pemetrexed and platinum-based chemotherapy, 104 patients were ≥65 years and 23 patients were ≥75 years of age [see Adverse Reactions (6.1)]. Exploratory analysis suggests a higher incidence of Grade 3 or higher adverse reactions (68% vs 61%) and more frequent dosage modifications for adverse reactions (55% vs 43%) in patients 65 years or older as compared to those younger than 65 years. Clinical studies of TAGRISSO in combination with pemetrexed and platinum-based chemotherapy did not include sufficient numbers of patients age 65 and over to determine whether they respond differently from younger patients.<br><br>**14 CLINICAL STUDIES**<br>**14.1 Adjuvant Treatment of Early-Stage EGFR Mutation-Positive Non-Small Cell Lung Cancer (NSCLC)**<br>14.1 Adjuvant Treatment of Early-Stage EGFR Mutation-Positive Non-Small Cell Lung Cancer (NSCLC)<br>The efficacy of TAGRISSO was demonstrated in a randomized, double-blind, placebo-controlled trial (ADAURA [NCT02511106]) for the adjuvant treatment of patients with EGFR exon 19 deletions or exon 21 L858R mutation-positive NSCLC who had complete tumor resection, with or without prior adjuvant chemotherapy. Eligible patients with resectable tumors (stage IB – IIIA according to American Joint Commission on Cancer [AJCC] 7th edition) were required to have predominantly non-squamous histology and EGFR exon 19 deletions or exon 21 L858R mutations identified prospectively from tumor tissue in a central laboratory by the cobas® EGFR Mutation Test. Patients with clinically significant uncontrolled cardiac disease, prior history of ILD/pneumonitis, or who received treatment with any EGFR kinase inhibitor were not eligible for the study.<br>Patients were randomized (1:1) to receive TAGRISSO 80 mg orally once daily or placebo following recovery from surgery and standard adjuvant chemotherapy if given. Patients who did not receive adjuvant chemotherapy were randomized within 10 weeks and patients who received adjuvant chemotherapy were randomized within 26 weeks following surgery. Randomization was stratified by mutation type (exon 19 deletions or exon 21 L858R mutations), race (Asian or non-Asian) and pTNM staging (IB or II or IIIA) according to AJCC 7th edition. Treatment was given for 3 years or until disease recurrence, or unacceptable toxicity.<br>The major efficacy outcome measure was disease-free survival (DFS, defined as reduction in the risk of disease recurrence or death) in patients with stage II – IIIA NSCLC determined by investigator assessment. Additional efficacy outcome measures included DFS in the overall population (patients with stage IB – IIIA NSCLC), and overall survival (OS) in patients with stage II – IIIA NSCLC and in the overall population.<br>A total of 682 patients were randomized to TAGRISSO (n=339) or placebo (n=343). The median age was 63 years (range 30-86 years); 70% were female; 64% were Asian and 72% were never smokers. Baseline World Health Organization (WHO) performance status was 0 (64%) or 1 (36%); 31% had stage IB, 35% II, and 34% IIIA. With regard to EGFR mutation status, 55% were exon 19 deletions and 45% were exon 21 L858R mutations. The majority (60%) of patients received adjuvant chemotherapy prior to randomization (27% IB; 70% II, 79% IIIA) (…)<br>**14.2 Previously Untreated EGFR Mutation-Positive Metastatic NSCLC**<br>FLAURA – TAGRISSO Monotherapy<br>The efficacy of TAGRISSO was demonstrated in a randomized, multicenter, double-blind, active-controlled trial (FLAURA [NCT02296125]) in patients with EGFR exon 19 deletions or exon 21 L858R mutation-positive, metastatic NSCLC, who had not received previous systemic treatment for metastatic disease. Patients were required to have measurable disease per RECIST v1.1, a WHO performance status of 0-1, and EGFR exon 19 deletions or exon 21 L858R mutation in tumor prospectively identified by the cobas® EGFR Mutation Test in a central laboratory or by an investigational assay at a CLIA-certified or accredited laboratory. Patients with CNS metastases not requiring steroids and with stable neurologic status for at least two weeks after completion of definitive surgery or radiotherapy were eligible. Patients were assessed at the investigator’s discretion for CNS metastases if they had a history of, or suspected, CNS metastases at study entry.

NDA/ANDA/BLA Number, Label Version Date	Drug	Therapeutic Area*	Biomarker†	Labeling Sections	Labeling Text‡
					Patients were randomized (1:1) to receive TAGRISSO 80 mg orally once daily or to receive gefitinib 250 mg orally once daily or erlotinib 150 mg orally once daily until disease progression or unacceptable toxicity. Randomization was stratified by EGFR mutation type (exon 19 deletions or exon 21 L858R mutation) and ethnicity (Asian or non-Asian). Patients randomized to the control arm were offered TAGRISSO at the time of disease progression if tumor samples tested positive for the EGFR T790M mutation. The major efficacy outcome measure was progression-free survival (PFS), as assessed by investigator. Additional efficacy outcome measures included OS and overall response rate (ORR).<br>A total of 556 patients were randomized to TAGRISSO (n=279) or to control (gefitinib n=183; erlotinib n=94). The median age was 64 years (range 26-93 years); 54% were <65 years of age; 63% were female; 62% were Asian and 64% were never smokers. Baseline WHO performance status was 0 (41%) or 1 (59%); 5% had Stage IIIb and 95% had Stage IV; and 7% received prior systemic cytotoxic chemotherapy as neoadjuvant or adjuvant therapy. With regard to EGFR tumor testing, 63% were exon 19 deletions and 37% were exon 21 L858R; 5 patients (<1%) also had a concomitant de novo T790M mutation. EGFR mutation status was confirmed centrally using the cobas® EGFR Mutation Test in 90% of patients. At the time of the final data cut-off, of those randomized to TAGRISSO and to investigator's choice erlotinib or gefitinib arm, 133 (48%) and 180 (65%) patients had received at least one subsequent treatment, respectively. Out of the 180 patients randomized to erlotinib or gefitinib who received subsequent treatment, 85 (47%) patients received TAGRISSO as first subsequent therapy. (…)<br>**14.3 Previously Untreated EGFR Mutation-Positive Locally Advanced or Metastatic NSCLC**<br>FLAURA2 – TAGRISSO in Combination with Pemetrexed and Platinum-based Chemotherapy<br>The efficacy of TAGRISSO in combination with pemetrexed and platinum-based chemotherapy was demonstrated in a randomized, multicenter, open-label trial (FLAURA2 [NCT04035486]) in patients with EGFR exon 19 deletion or exon 21 L858R mutation-positive locally advanced or metastatic NSCLC, who had not received previous systemic treatment for advanced disease. Patients were required to have measurable disease per RECIST v1.1, a WHO performance status of 0-1, and EGFR exon 19 deletions or exon 21 L858R mutations as identified by the cobas® EGFR Mutation Test v2 performed prospectively in tissue samples in a central laboratory or by a local test performed in a CLIA-certified or accredited laboratory.<br>Patients were randomized (1:1) to one of the following treatment arms:<br>• TAGRISSO (80 mg) orally once daily with pemetrexed (500 mg/m2) and investigator's choice of cisplatin (75 mg/m2) or carboplatin (AUC5) administered intravenously on Day 1 of 21-day cycles for 4 cycles, followed by TAGRISSO (80 mg) orally once daily and pemetrexed (500 mg/m2) administered intravenously every 3 weeks<br>• TAGRISSO (80 mg) orally once daily<br>Randomization was stratified by race (Chinese/Asian, non-Chinese/Asian or non-Asian), WHO performance status (0 or 1), and method for tissue testing (central or local). Patients received study therapy until intolerance to therapy, or the investigator determined that the patient was no longer experiencing clinical benefit. Progression-free survival, as assessed by investigator per RECIST 1.1 was the primary efficacy outcome measure. Overall survival was a key secondary outcome measure. Additional efficacy outcome measures included ORR and DoR.<br>A total of 557 patients were randomized to either TAGRISSO in combination with pemetrexed and platinum-based chemotherapy (n=279) or TAGRISSO monotherapy (n=278). The median age was 61 years (range 26-85 years); 39% were ≥65 years and 8% were ≥75 years of age; 61% were female; 64% were Asian and 66% were never smokers. Baseline WHO PS was 0 (37%) or 1 (63%); 4% had locally advanced and 96% had metastatic NSCLC; and 1.8% received prior systemic cytotoxic chemotherapy as neoadjuvant or adjuvant therapy. With regard to EGFR tumor testing, 61% of tumors had exon 19 deletions and 38% had exon 21 L858R mutations; 0.7% of patients had tumors with both exon 19 deletions and exon 21 L858R. EGFR mutation status was centrally confirmed using the cobas® EGFR Mutation Test v2 in 96% of patients. (…)<br>**14.4 Previously Treated EGFR T790M Mutation-Positive Metastatic NSCLC**<br>The efficacy of TAGRISSO was demonstrated in a randomized, multicenter open-label, active-controlled trial in patients with metastatic EGFR T790M mutation-positive NSCLC who had progressed on prior systemic therapy, including an EGFR TKI (AURA3). All patients were required to have EGFR T790M mutation-positive NSCLC identified by the cobas® EGFR Mutation Test performed in a central laboratory prior to randomization. (…)
**203505, 01/25/2019**	**Ospemifene (1)**	Gynecology	CYP2C9	Clinical Pharmacology	**12 CLINICAL PHARMACOLOGY**<br>**12.3 Pharmacokinetics**<br>**Drug Interactions**<br>*Effect of Ospemifene on the Pharmacokinetics of the Co-Administered Drug*<br>Warfarin<br>Ospemifene 60 mg was given after a light breakfast (two slices of bread with ham and cheese and juice) once daily for 12 days in sixteen postmenopausal women who were determined to be rapid metabolizers of CYP2C9 (CYP2C9*1/*1 or CYP2C9*1/*2). On Day 8, a single dose of warfarin 10 mg and vitamin K 10 mg were administered one hour after a light breakfast. The geometric mean ratio (90% CI) for S-warfarin with and without ospemifene for Cmax and AUC0-inf were 0.97 (0.92-1.02) and 0.96 (0.91-1.02), respectively. Multiple doses of ospemifene did not significantly affect the pharmacokinetics of a single dose of warfarin. No study was conducted with multiple doses of warfarin.
**203505, 01/25/2019**	**Ospemifene (2)**	Gynecology	CYP2B6	Clinical Pharmacology	**12 CLINICAL PHARMACOLOGY**<br>**12.3 Pharmacokinetics**<br>**Drug Interactions**<br>*Effect of Ospemifene on the Pharmacokinetics of the Co-Administered Drug*<br>Bupropion<br>Ospemifene 60 mg was administered once daily for seven consecutive days after the evening meal in sixteen postmenopausal women (not homozygous for CYP2B6*6). On the Day 8 after overnight fast, a single 150 mg dose of sustained release bupropion was administered in morning under fasted condition. The geometric mean ratio (90% CI) for bupropion with and without ospemifene for Cmax and AUC0-inf were 0.82 (0.75-0.91) and 0.81 (0.77-0.86), respectively. The geometric mean ratio (90% CI) for hydroxybupropion, an active metabolite formed via CYP2B6, with and without ospemifene for Cmax and AUC0-inf were 1.16 (1.09-1.24) and 0.98 (0.92-1.04), respectively.

* Therapeutic areas do not necessarily reflect the CDER review division.
† Representative biomarkers are listed based on standard nomenclature as per the Human Genome Organization (HUGO) symbol and/or simplified descriptors using other common conventions. Listed biomarkers do not necessarily reflect the terminology used in labeling. The term "Nonspecific" is provided when labeling does not explicitly identify the specific biomarker(s) or when the biomarker is represented by a molecular phenotype or gene signature, and in some cases the biomarker was inferred based on the labeling language.
‡ Referenced figures and tables may be found in the labeling available at Drugs@FDA.
Blue text represents the most recent addtions and/or changes since last posted version.

Table of Pharmacogenomic Biomarkers in Drug Labeling
Last Updated: 06/2024

NDA/ANDA/BLA Number, Label Version Date	Drug	Therapeutic Area*	Biomarker†	Labeling Sections	Labeling Text‡
**202810, 12/13/2018**	Oxcarbazepine	Neurology	HLA-B	Warnings and Precautions	**5 WARNINGS AND PRECAUTIONS**<br>**5.4 Serious Dermatological Reactions**<br>*Association with HLA-B*1502*<br>Patients carrying the HLA-B*1502 allele may be at increased risk for SJS/TEN with Oxtellar XR treatment.<br>Human Leukocyte Antigen (HLA) allele B*1502 increases the risk for developing SJS/TEN in patients treated with carbamazepine. The chemical structures of immediate release oxcarbazepine and Oxtellar XR are similar to that of carbamazepine. Available clinical evidence, and data from nonclinical studies showing a direct interaction between immediate release oxcarbazepine and HLA-B*1502 protein, suggest that the HLAB*1502 allele may also increase the risk for SJS/TEN with Oxtellar XR. The frequency of HLA-B*1502 allele ranges from 2 to 12% in Han Chinese populations, is about 8% in Thai populations, and above 15% in the Philippines and in some Malaysian populations. Allele frequencies up to about 2% and 6% have been reported in Korea and India, respectively. The frequency of the HLA-B*1502 allele is negligible in people from European descent, several African populations, indigenous peoples of the Americas, Hispanic populations, and in Japanese (<1%).<br>Testing for the presence of the HLA-B*1502 allele should be considered in patients with ancestry in genetically at-risk populations, prior to initiating treatment with Oxtellar XR. The use of Oxtellar XR should be avoided in patients positive for HLA-B*1502 unless the benefits clearly outweigh the risks. Consideration should also be given to avoid the use of other drugs associated with SJS/TEN in HLA-B*1502 positive patients, when alternative therapies are otherwise equally acceptable. Screening is not generally recommended in patients from populations in which the prevalence of HLA-B*1502 is low, or in current Oxtellar XR users, as the risk of SJS/TEN is largely confined to the first few months of therapy, regardless of HLA-B*1502 status.<br>The use of HLA-B*1502 genotyping has important limitations and must never substitute for appropriate clinical vigilance and patient management. The role of other possible factors in the development of, and morbidity from, SJS/TEN, such as antiepileptic drug (AED) dose, compliance, concomitant medications, comorbidities, and the level of dermatologic monitoring have not been well characterized.
**208032, 11/02/2018**	Oxymetazoline and Tetracaine (1)	Anesthesiology	G6PD	Warnings and Precautions	**5 WARNINGS AND PRECAUTIONS**<br>**5.1 Methemoglobinemia**<br>Cases of methemoglobinemia have been reported in association with local anesthetic use. Although all patients are at risk for methemoglobinemia, patients with glucose-6-phosphate dehydrogenase deficiency, congenital or idiopathic methemoglobinemia, cardiac or pulmonary compromise, infants under 6 months of age, and concurrent exposure to oxidizing agents or their metabolites are more susceptible to developing clinical manifestations of the condition. If local anesthetics must be used in these patients, close monitoring for symptoms and signs of methemoglobinemia is recommended. (…)
**208032, 11/02/2018**	Oxymetazoline and Tetracaine (2)	Anesthesiology	Nonspecific (Congenital Methemoglobinemia)	Warnings and Precautions	**5 WARNINGS AND PRECAUTIONS**<br>**5.1 Methemoglobinemia**<br>Cases of methemoglobinemia have been reported in association with local anesthetic use. Although all patients are at risk for methemoglobinemia, patients with glucose-6-phosphate dehydrogenase deficiency, congenital or idiopathic methemoglobinemia, cardiac or pulmonary compromise, infants under 6 months of age, and concurrent exposure to oxidizing agents or their metabolites are more susceptible to developing clinical manifestations of the condition. If local anesthetics must be used in these patients, close monitoring for symptoms and signs of methemoglobinemia is recommended.<br>Signs of methemoglobinemia may occur immediately or may be delayed some hours after exposure,and are characterized by a cyanotic skin discoloration and/or abnormal coloration of the blood. Methemoglobin levels may continue to rise; therefore, immediate treatment is required to avert more serious central nervous system and cardiovascular adverse effects, including seizures, coma, arrhythmias, and death. Discontinue KOVANAZE and any other oxidizing agents. Depending on the severity of the signs and symptoms, patients may respond to supportive care, i.e., oxygen therapy, hydration. A more severe clinical presentation may require treatment with methylene blue, exchange transfusion, or hyperbaric oxygen.
**207103, 09/09/2019**	Palbociclib (1)	Oncology	ESR (Hormone Receptor)	Indications and Usage, Adverse Reactions, Clinical Studies	**1 INDICATIONS AND USAGE**<br>IBRANCE is indicated for the treatment of adult patients with hormone receptor (HR)-positive, human epidermal growth factor receptor 2 (HER2)-negative advanced or metastatic breast cancer in combination with:<br>• an aromatase inhibitor as initial endocrine-based therapy in postmenopausal women or in men; or<br>• fulvestrant in patients with disease progression following endocrine therapy.<br><br>**6 ADVERSE REACTIONS**<br>**6.1 Clinical Studies Experience**<br>**Study 1: IBRANCE plus Letrozole**<br>**Patients with estrogen receptor (ER)-positive, HER2-negative advanced or metastatic breast cancer for initial endocrine based therapy**<br>The safety of IBRANCE (125 mg/day) plus letrozole (2.5 mg/day) versus placebo plus letrozole was evaluated in Study 1 (PALOMA-2). The data described below reflect exposure to IBRANCE in 444 out of 666 patients with ER-positive, HER2-negative advanced breast cancer who received at least 1 dose of IBRANCE plus letrozole in Study 1. The median duration of treatment for IBRANCE plus letrozole was 19.8 months while the median duration of treatment for placebo plus letrozole arm was 13.8 months. (…)<br>**Study 2: IBRANCE plus Fulvestrant**<br>**Patients with HR-positive, HER2-negative advanced or metastatic breast cancer who have had disease progression on or after prior adjuvant or metastatic endocrine therapy**<br>The safety of IBRANCE (125 mg/day) plus fulvestrant (500 mg) versus placebo plus fulvestrant was evaluated in Study 2 (PALOMA-3). The data described below reflect exposure to IBRANCE in 345 out of 517 patients with HR-positive, HER2-negative advanced or metastatic breast cancer who received at least 1 dose of IBRANCE plus fulvestrant in Study 2. The median duration of treatment for IBRANCE plus fulvestrant was 10.8 months while the median duration of treatment for placebo plus fulvestrant arm was 4.8 months. (…)<br>**Male patients with HR-positive, HER2-negative advanced or metastatic breast cancer**

* Therapeutic areas do not necessarily reflect the CDER review division.
† Representative biomarkers are listed based on standard nomenclature as per the Human Genome Organization (HUGO) symbol and/or simplified descriptors using other common conventions. Listed biomarkers do not necessarily reflect the terminology used in labeling. The term “Nonspecific” is provided when labeling does not explicitly identify the specific biomarker(s) or when the biomarker is represented by a molecular phenotype or gene signature, and in some cases the biomarker was inferred based on the labeling language.
‡ Referenced figures and tables may be found in the labeling available at Drugs@FDA.
Blue text represents the most recent addtions and/or changes since last posted version.

NDA/ANDA/BLA Number, Label Version Date	Drug	Therapeutic Area*	Biomarker†	Labeling Sections	Labeling Text‡
					Based on limited data from postmarketing reports and electronic health records, the safety profile for men treated with IBRANCE is consistent with the safety profile in women treated with IBRANCE.<br><br>**14 CLINICAL STUDIES**<br>**Study 1: IBRANCE plus Letrozole**<br>**Patients with ER-positive, HER2-negative advanced or metastatic breast cancer for initial endocrine based therapy**<br>Study 1 (PALOMA-2) was an international, randomized, double-blind, parallel-group, multicenter study of IBRANCE plus letrozole versus placebo plus letrozole conducted in postmenopausal women with ER-positive, HER2-negative advanced breast cancer who had not received previous systemic treatment for their advanced disease. (…)<br>**Study 2: IBRANCE plus Fulvestrant**<br>**Patients with HR-positive, HER2-negative advanced or metastatic breast cancer who have had disease progression on or after prior adjuvant or metastatic endocrine therapy**<br>Study 2 (PALOMA-3) was an international, randomized, double-blind, parallel group, multicenter study of IBRANCE plus fulvestrant versus placebo plus fulvestrant conducted in women with HR-positive, HER2-negative advanced breast cancer, regardless of their menopausal status, whose disease progressed on or after prior endocrine therapy. (…)
**207103, 09/09/2019**	Palbociclib (2)	Oncology	ERBB2 (HER2)	Indications and Usage, Adverse Reactions, Clinical Studies	**1 INDICATIONS AND USAGE**<br>IBRANCE is indicated for the treatment of adult patients with hormone receptor (HR)-positive, human epidermal growth factor receptor 2 (HER2)-negative advanced or metastatic breast cancer in combination with:<br>• an aromatase inhibitor as initial endocrine-based therapy in postmenopausal women or in men; or<br>• fulvestrant in patients with disease progression following endocrine therapy.<br><br>**6 ADVERSE REACTIONS**<br>**6.1 Clinical Studies Experience**<br>**Study 1: IBRANCE plus Letrozole**<br>**Patients with estrogen receptor (ER)-positive, HER2-negative advanced or metastatic breast cancer for initial endocrine based therapy**<br>The safety of IBRANCE (125 mg/day) plus letrozole (2.5 mg/day) versus placebo plus letrozole was evaluated in Study 1 (PALOMA-2). The data described below reflect exposure to IBRANCE in 444 out of 666 patients with ER-positive, HER2-negative advanced breast cancer who received at least 1 dose of IBRANCE plus letrozole in Study 1. The median duration of treatment for IBRANCE plus letrozole was 19.8 months while the median duration of treatment for placebo plus letrozole arm was 13.8 months. (…)<br>**Study 2: IBRANCE plus Fulvestrant**<br>**Patients with HR-positive, HER2-negative advanced or metastatic breast cancer who have had disease progression on or after prior adjuvant or metastatic endocrine therapy**<br>The safety of IBRANCE (125 mg/day) plus fulvestrant (500 mg) versus placebo plus fulvestrant was evaluated in Study 2 (PALOMA-3). The data described below reflect exposure to IBRANCE in 345 out of 517 patients with HR-positive, HER2-negative advanced or metastatic breast cancer who received at least 1 dose of IBRANCE plus fulvestrant in Study 2. The median duration of treatment for IBRANCE plus fulvestrant was 10.8 months while the median duration of treatment for placebo plus fulvestrant arm was 4.8 months. (…)<br>**Male patients with HR-positive, HER2-negative advanced or metastatic breast cancer**<br>Based on limited data from postmarketing reports and electronic health records, the safety profile for men treated with IBRANCE is consistent with the safety profile in women treated with IBRANCE.<br><br>**14 CLINICAL STUDIES**<br>**Study 1: IBRANCE plus Letrozole**<br>**Patients with ER-positive, HER2-negative advanced or metastatic breast cancer for initial endocrine based therapy**<br>Study 1 (PALOMA-2) was an international, randomized, double-blind, parallel-group, multicenter study of IBRANCE plus letrozole versus placebo plus letrozole conducted in postmenopausal women with ER-positive, HER2-negative advanced breast cancer who had not received previous systemic treatment for their advanced disease. (…)<br>**Study 2: IBRANCE plus Fulvestrant**<br>**Patients with HR-positive, HER2-negative advanced or metastatic breast cancer who have had disease progression on or after prior adjuvant or metastatic endocrine therapy**<br>Study 2 (PALOMA-3) was an international, randomized, double-blind, parallel group, multicenter study of IBRANCE plus fulvestrant versus placebo plus fulvestrant conducted in women with HR-positive, HER2-negative advanced breast cancer, regardless of their menopausal status, whose disease progressed on or after prior endocrine therapy. (…)
**021999, 01/25/2019**	Paliperidone	Psychiatry	CYP2D6	Clinical Pharmacology	**12 CLINICAL PHARMACOLOGY**<br>**12.3 Pharmacokinetics**<br>*Metabolism and Elimination*<br>(…) Population pharmacokinetics analyses indicated no discernible difference on the apparent clearance of paliperidone after administration of oral paliperidone between extensive metabolizers and poor metabolizers of CYP2D6 substrates. (…)
**021372, 09/18/2014**	Palonosetron	Gastroenterology	CYP2D6	Clinical Pharmacology	**12 CLINICAL PHARMACOLOGY**<br>**12.3 Pharmacokinetics**

NDA/ANDA/BLA Number, Label Version Date	Drug	Therapeutic Area*	Biomarker†	Labeling Sections	Labeling Text‡
					Metabolism<br>Palonosetron is eliminated by multiple routes with approximately 50% metabolized to form two primary metabolites: N-oxide-palonosetron and 6-Shydroxy-palonosetron. These metabolites each have less than 1% of the 5- HT3 receptor antagonist activity of palonosetron. In vitro metabolism studies have suggested that CYP2D6 and to a lesser extent, CYP3A4 and CYP1A2 are involved in the metabolism of palonosetron. However, clinical pharmacokinetic parameters are not significantly different between poor and extensive metabolizers of CYP2D6 substrates.
125147, 06/29/2017	Panitumumab (1)	Oncology	EGFR	Adverse Reactions, Clinical Pharmacology, Clinical Studies	**6 ADVERSE REACTIONS**<br>**6.1 Clinical Trials Experience**<br>(…) Safety data are presented from two clinical trials in which patients received Vectibix: Study 20020408, an open-label, multinational, randomized, controlled, monotherapy clinical trial (N = 463) evaluating Vectibix with best supportive care (BSC) versus BSC alone in patients with EGFR-expressing mCRC and Study 20050203, a randomized, controlled trial (N = 1183) in patients with mCRC that evaluated Vectibix in combination with FOLFOX chemotherapy versus FOLFOX chemotherapy alone. Safety data for Study 20050203 are limited to 656 patients with wild-type KRAS mCRC. The safety profile of Vectibix in patients with wild-type RAS mCRC is similar with that seen in patients with wild-type KRAS mCRC.<br><br>**12 CLINICAL PHARMACOLOGY**<br>**12.3 Pharmacokinetics**<br>(…) A population pharmacokinetic analysis was performed to explore the potential effects of selected covariates on panitumumab pharmacokinetics. Results suggest that age (21-88 years), gender, race (15% nonwhite), mild-tomoderate renal dysfunction, mild-to-moderate hepatic dysfunction, and EGFR membrane-staining intensity (1+, 2+, and 3+) in tumor cells had no apparent impact on the pharmacokinetics of panitumumab.<br>No formal pharmacokinetic studies of panitumumab have been conducted in patients with renal or hepatic impairment.<br><br>**14 CLINICAL STUDIES**<br>**14.1 Recurrent or Refractory mCRC**<br>The safety and efficacy of Vectibix was demonstrated in Study 20020408, an open-label, multinational, randomized, controlled trial of 463 patients with EGFR-expressing, metastatic carcinoma of the colon or rectum, in Study 20080763, an open-label, multicenter, multinational, randomized trial of 1010 patients with wild-type KRAS mCRC, and in Study 20100007, an open-label, multicenter, multinational, randomized trial of 377 patients with wild-type KRAS mCRC. (…)
125147, 06/29/2017	Panitumumab (2)	Oncology	RAS	Indications and Usage, Dosage and Administration, Warnings and Precautions, Adverse Reactions, Clinical Studies	**1 INDICATIONS AND USAGE**<br>**1.1 Metastatic Colorectal Cancer**<br>Vectibix is indicated for the treatment of patients with wild-type RAS (defined as wild-type in both KRAS and NRAS as determined by an FDA-approved test for this use) metastatic colorectal cancer (mCRC) [see Dosage and Administration (2.1)]:<br>• As first-line therapy in combination with FOLFOX [see Clinical Studies (14.2)].<br>• As monotherapy following disease progression after prior treatment with fluoropyrimidine-, oxaliplatin-, and irinotecan-containing chemotherapy [see Clinical Studies (14.1)].<br>Limitation of Use: Vectibix is not indicated for the treatment of patients with RAS-mutant mCRC or for whom RAS mutation status is unknown [see Dosage and Administration (2.1), Warnings and Precautions (5.2), and Clinical Pharmacology (12.1)].<br><br>**2 DOSAGE AND ADMINISTRATION**<br>**2.1 Patient Selection**<br>Prior to initiation of treatment with Vectibix, assess RAS mutational status in colorectal tumors and confirm the absence of a RAS mutation in exon 2 (codons 12 and 13), exon 3 (codons 59 and 61), and exon 4 (codons 117 and 146) of both KRAS and NRAS. Information on FDA-approved tests for the detection of RAS mutations in patients with metastatic colorectal cancer is available at: http://www.fda.gov/CompanionDiagnostics.<br><br>**5 WARNINGS AND PRECAUTIONS**<br>**5.2 Increased Tumor Progression, Increased Mortality, or Lack of Benefit in Patients with RAS-Mutant mCRC**<br>Vectibix is not indicated for the treatment of patients with colorectal cancer that harbor somatic mutations in exon 2 (codons 12 and 13), exon 3 (codons 59 and 61), and exon 4 (codons 117 and 146) of either KRAS or NRAS and hereafter is referred to as "RAS" [see Indications and Usage (1.1), Dosage and Administration (2.1), Clinical Pharmacology (12.1) and Clinical Studies (14)].<br>Retrospective subset analyses across several randomized clinical trials were conducted to investigate the role of RAS mutations on the clinical effects of anti-EGFR-directed monoclonal antibodies (panitumumab or cetuximab). Anti-EGFR antibodies in patients with tumors containing RAS mutations resulted in exposing those patients to anti-EGFR related adverse reactions without clinical benefit from these agents [see Indications and Usage (1.1), and Clinical Pharmacology (12.1)].<br>Additionally, in Study 20050203, 272 patients with RAS-mutant mCRC tumors received Vectibix in combination with FOLFOX and 276 patients received FOLFOX alone. In an exploratory subgroup analysis, OS was shorter (HR = 1.21, 95% CI: 1.01-1.45) in patients with RAS-mutant mCRC who received Vectibix and FOLFOX versus FOLFOX alone [see Indications and Usage (1.1)].<br><br>**6 ADVERSE REACTIONS**<br>The following adverse reactions are discussed in greater detail in other sections of the label:<br>• (…) Increased Tumor Progression, Increased Mortality, or Lack of Benefit in RAS-Mutant mCRC [see Indications and Usage (1.1) and Warnings and Precautions (5.2)] (…)<br>**6.1 Clinical Trials Experience**

* Therapeutic areas do not necessarily reflect the CDER review division.
† Representative biomarkers are listed based on standard nomenclature as per the Human Genome Organization (HUGO) symbol and/or simplified descriptors using other common conventions. Listed biomarkers do not necessarily reflect the terminology used in labeling. The term "Nonspecific" is provided when labeling does not explicitly identify the specific biomarker(s) or when the biomarker is represented by a molecular phenotype or gene signature, and in some cases the biomarker was inferred based on the labeling language.
‡ Referenced figures and tables may be found in the labeling available at Drugs@FDA.
Blue text represents the most recent addtions and/or changes since last posted version.

NDA/ANDA/BLA Number, Label Version Date	Drug	Therapeutic Area*	Biomarker†	Labeling Sections	Labeling Text‡
					(…) Safety data are presented from two clinical trials in which patients received Vectibix: Study 20020408, an open-label, multinational, randomized, controlled, monotherapy clinical trial (N = 463) evaluating Vectibix with best supportive care (BSC) versus BSC alone in patients with EGFR-expressing mCRC and Study 20050203, a randomized, controlled trial (N = 1183) in patients with mCRC that evaluated Vectibix in combination with FOLFOX chemotherapy versus FOLFOX chemotherapy alone. Safety data for Study 20050203 are limited to 656 patients with wild-type KRAS mCRC. The safety profile of Vectibix in patients with wild-type RAS mCRC is similar with that seen in patients with wild-type KRAS mCRC.<br>(…)<br>*Vectibix in Combination with FOLFOX Chemotherapy*<br>The most commonly reported adverse reactions (≥ 20%) in patients with wild-type KRAS mCRC receiving Vectibix (6 mg/kg every 2 weeks) and FOLFOX therapy (N = 322) in Study 20050203 were diarrhea, stomatitis, mucosal inflammation, asthenia, paronychia, anorexia, hypomagnesemia, hypokalemia, rash, acneiform dermatitis, pruritus, and dry skin (Table 2). Serious adverse reactions (≥ 2% difference between treatment arms) in Vectibix-treated patients with wildtype KRAS mCRC were diarrhea and dehydration. The commonly reported adverse reactions (≥ 1%) leading to discontinuation in patients with wild-type KRAS mCRC receiving Vectibix were rash, paresthesia, fatigue, diarrhea, acneiform dermatitis, and hypersensitivity. One grade 5 adverse reaction, hypokalemia, occurred in a patient who received Vectibix. (See Table 2) (…)<br><br>**14 CLINICAL STUDIES**<br>**14.1 Recurrent or Refractory mCRC**<br>The safety and efficacy of Vectibix was demonstrated in Study 20020408, an open-label, multinational, randomized, controlled trial of 463 patients with EGFR-expressing, metastatic carcinoma of the colon or rectum, and in Study 20080763, an open-label, multicenter, multinational, randomized trial of 1010 patients with wild-type KRAS mCRC, and in Study 20100007, an open-label, multicenter, multinational, randomized trial of 377 patients with wild-type KRAS mCRC. (…)<br>*Study 20020408 (NCT00113763)*<br>(…) The study results were analyzed in the wild-type KRAS subgroup where KRAS status was retrospectively determined using archived paraffin-embedded tumor tissue. KRAS mutation status was determined in 427 patients (92%); of these, 243 (57%) had no detectable KRAS mutations in either codons 12 or 13. The hazard ratio for PFS in patients with wild-type KRAS mCRC was 0.45 (95% CI: 0.34-0.59) favoring the panitumumab arm. The response rate was 17% for the panitumumab arm and 0% for BSC. There were no differences in OS; 77% of patients in the BSC arm received panitumumab at the time of disease progression.<br>*Study 20080763 (NCT01001377)*<br>Study 20080763was an open-label, multicenter, multinational, randomized (1:1) clinical trial, stratified by region (North America, Western Europe, and Australia versus rest of the world) and ECOG PS (0 and 1 vs 2) in patients with wild-type KRAS mCRC. (See Table 3 and Figure 1) (…)<br>*Study 20100007 (NCT01412957)*<br>*Study 20100007 was an open-label, multicenter, randomized (1:1) clinical study stratified by ECOG performance status (0 or 1 vs 2) and region (sites in Europe versus Asia versus rest of world) in patients with wild-type KRAS mCRC. Eligible patients were required to have received prior therapy with irinotecan, oxaliplatin, and a thymidylate synthase inhibitor, and have wild-type KRAS exon 2 mCRC as determined by a clinical trial assay. An assessment for RAS status (defined as KRAS exons 2, 3, and 4 and NRAS exons 2, 3, and 4) using Sanger sequencing was conducted in patients for whom tumor tissue was available. Patients were randomized to receive Vectibix (6 mg/kg intravenously every 14 days) plus BSC or BSC alone. Patients received Vectibix and BSC or BSC until disease progression, withdrawal of consent, unacceptable toxicity, or death. Patients randomized to BSC were not offered Vectibix at the time of disease progression. The major efficacy outcome measure was OS in patients with wild-type KRAS mCRC. Secondary efficacy outcome measures included OS in the subgroup of patients with wild-type RAS mCRC; PFS and ORR in patients with wild-type KRAS; and PFS and ORR in the subgroup of patients with wild-type RAS mCRC.* (…)<br>(…) KRAS tumor mutation status was available for all patients and RAS tumor mutation status was available for 86% of the 377 patients. Among the 377 patients, 270 (72%) patients had wild-type RAS tumors, 54 (14%) had mutant RAS tumors, and 54 (14%) had unknown RAS tumor status. (See Table 4 and Figure 2).<br>**14.2 First-line in Combination with FOLFOX Chemotherapy**<br>(…) The prespecified major efficacy measure was PFS in patients (n = 656) with wild-type KRAS mCRC as assessed by a blinded independent central review of imaging. Other key efficacy measures included OS and ORR.<br>In Study 20050203, in the wild-type KRAS subgroup (n = 656), 64% of patients were men, 92% White, 2% Black, and 4% Hispanic or Latino. Sixty-six percent of patients had colon cancer and 34% had rectal cancer. ECOG performance was 0 in 56% of patients, 1 in 38% of patients, and 2 in 6% of patients. Median age was 61.5 years.<br>The efficacy results in Study 20050203 in patients with wild-type KRAS mCRC are presented in Table 5 below. (See Table 5) (…) *(…)*<br>*Exploratory Analysis of OS*<br>An exploratory analysis of OS with updated information based on events in 82% of patients with wild-type KRAS mCRC estimated the treatment effect of Vectibix plus FOLFOX compared with FOLFOX alone on OS (Figure 3). Median OS among 325 patients with wild-type KRAS mCRC who received Vectibix plus FOLFOX was 23.8 months (95% CI: 20.0, 27.7) vs 19.4 months (95% CI: 17.4, 22.6) among 331 patients who received FOLFOX alone (HR = 0.83, 95% CI: 0.70, 0.98). (See Figure 3)<br>*Retrospective exploratory analyses in the RAS wild-type subgroup*<br>Among the 656 patients with wild-type KRAS exon 2 mCRC, RAS mutation status was assessed for 620 patients using Sanger bidirectional sequencing and Surveyor®/WAVE® analysis. Of these 620 patients, approximately 17% of patients (n = 104) tumors harbored mutations in KRAS exons 3 or 4 or in NRAS exons 2, 3, and 4.<br>Retrospective subset analyses were then conducted among the subset of patients without RAS mutations (n = 512) as described above.

* Therapeutic areas do not necessarily reflect the CDER review division.
† Representative biomarkers are listed based on standard nomenclature as per the Human Genome Organization (HUGO) symbol and/or simplified descriptors using other common conventions. Listed biomarkers do not necessarily reflect the terminology used in labeling. The term “Nonspecific” is provided when labeling does not explicitly identify the specific biomarker(s) or when the biomarker is represented by a molecular phenotype or gene signature, and in some cases the biomarker was inferred based on the labeling language.
‡ Referenced figures and tables may be found in the labeling available at Drugs@FDA.
Blue text represents the most recent addtions and/or changes since last posted version.

NDA/ANDA/BLA Number, Label Version Date	Drug	Therapeutic Area*	Biomarker†	Labeling Sections	Labeling Text‡
					In the wild-type RAS subgroup, 65% of patients were men and 91% were White, 2% Black, and 5% Hispanic or Latino. Sixty-five percent of patients had colon cancer and 35% had rectal cancer. ECOG performance was 0 in 57% of patients, 1 in 37% of patients, and 2 in 6% of patients. Median age was 61 years. (See Table 6 and Figure 4)<br>**14.3 RAS-Mutant mCRC**<br>Vectibix is not effective for the treatment of patients with RAS-mutant mCRC, defined as a RAS mutation in exon 2 (codons 12 and 13), exon 3 (codons 59 and 61), or exon 4 (codons 117 and 146) of KRAS and NRAS.<br>In Study 20050203, among patients with RAS-mutant tumors, the median PFS was 7.3 months (95% CI: 6.3, 7.9) among 272 patients receiving Vectibix plus FOLFOX and 8.7 months (95% CI: 7.6, 9.4) among patients who received FOLFOX alone (HR = 1.31, 95% CI: 1.07, 1.60). The median OS was 15.6 months (95% CI: 13.4, 17.9) among patients receiving Vectibix plus FOLFOX and 19.2 months (95% CI: 16.7, 21.8) among patients who received FOLFOX alone (HR = 1.25, 95% CI: 1.02, 1.55).<br>In Study 20100007, among patients with RAS-mutant tumors, no differences in OS or PFS were observed between the treatment arms [n = 54; OS HR = 0.99 (95% CI: 0.49, 2.00); PFS HR = 1.03 (95% CI: 0.56, 1.90)].
**020987, 04/25/2019**	**Pantoprazole**	Gastroenterology	CYP2C19	Clinical Pharmacology	**12 CLINICAL PHARMACOLOGY**<br>**12.3 Pharmacokinetics**<br>*Patients with Hepatic Impairment*<br>In patients with mild to severe hepatic impairment (Child-Pugh A to C cirrhosis), maximum pantoprazole concentrations increased only slightly (1.5-fold) relative to healthy subjects. Although serum half-life values increased to 7-9 hours and AUC values increased by 5- to 7-fold in hepatic-impaired patients, these increases were no greater than those observed in CYP2C19 poor metabolizers, where no dosage adjustment is warranted. These pharmacokinetic changes in hepatic-impaired patients result in minimal drug accumulation following once-daily, multiple-dose administration. Doses higher than 40 mg/day have not been studied in hepatically impaired patients.<br>**12.5 Pharmacogenomics**<br>CYP2C19 displays a known genetic polymorphism due to its deficiency in some subpopulations (e.g., approximately 3% of Caucasians and African-Americans and 17% to 23% of Asians are poor metabolizers). Although these subpopulations of pantoprazole poor metabolizers have elimination half-life values of 3.5 to 10 hours in adults, they still have minimal accumulation (23% or less) with once-daily dosing. For adult patients who are CYP2C19 poor metabolizers, no dosage adjustment is needed.<br>Similar to adults, pediatric patients who have the poor metabolizer genotype of CYP2C19 (CYP2C19 *2/*2) exhibited greater than a 6-fold increase in AUC compared to pediatric extensive (CYP2C19 *1/*1) and intermediate (CYP2C19 *1/*x) metabolizers. Poor metabolizers exhibited approximately 10 fold lower apparent oral clearance compared to extensive metabolizers.<br>For known pediatric poor metabolizers, a dose reduction should be considered.
**125511, 12/17/2018**	**Parathyroid Hormone**	Inborn Errors of Metabolism	CASR	Indications and Usage, Clinical Studies	**1 INDICATIONS AND USAGE**<br>NATPARA is a parathyroid hormone indicated as an adjunct to calcium and vitamin D to control hypocalcemia in patients with hypoparathyroidism.<br>*Limitations of Use:*<br>• Because of the potential risk of osteosarcoma, NATPARA is recommended only for patients who cannot be well-controlled on calcium supplements and active forms of vitamin D alone [see Warnings and Precautions (5.1)].<br>• NATPARA was not studied in patients with hypoparathyroidism caused by calcium-sensing receptor mutations.<br>• NATPARA was not studied in patients with acute post-surgical hypoparathyroidism.<br><br>**14 CLINICAL STUDIES**<br>*Study in Patients with Established Hypoparathyroidism*<br>(…) Patients with hypoparathyroidism due to calcium-sensing receptor mutations were excluded from the trial. (…)
**020031, 01/04/2017**	**Paroxetine**	Psychiatry	CYP2D6	Drug Interactions, Clinical Pharmacology	**DRUG INTERACTIONS**<br>**Drugs Metabolized by CYP2D6:**<br>(…) In healthy volunteers who were extensive metabolizers of CYP2D6, paroxetine 20 mg daily was given in combination with 20 mg atomoxetine every 12 hours. This resulted in increases in steady state atomoxetine AUC values that were 6- to 8-fold greater and in atomoxetine Cmax values that were 3- to 4-fold greater than when atomoxetine was given alone. Dosage adjustment of atomoxetine may be necessary and it is recommended that atomoxetine be initiated at a reduced dose when it is given with paroxetine. (…)<br><br>**CLINICAL PHARMACOLOGY**<br>**Pharmacokinetics:** Paroxetine hydrochloride is completely absorbed after oral dosing of a solution of the hydrochloride salt. The mean elimination half-life is approximately 21 hours (CV 32%) after oral dosing of 30 mg tablets of PAXIL daily for 30 days. Paroxetine is extensively metabolized and the metabolites are considered to be inactive. Nonlinearity in pharmacokinetics is observed with increasing doses. Paroxetine metabolism is mediated in part by CYP2D6, and the metabolites are primarily excreted in the urine and to some extent in the feces. Pharmacokinetic behavior of paroxetine has not been evaluated in subjects who are deficient in CYP2D6 (poor metabolizers).
**210922, 08/10/2018**	**Patisiran**	Neurology	TTR	Adverse Reactions, Clinical Pharmacology, Clinical Studies	**6 ADVERSE REACTIONS**<br>(…) At baseline, 46% of patients were in Stage 1 of the disease and 53% were in Stage 2. Forty-three percent of patients had Val30Met mutations in the transthyretin gene; the remaining patients had 38 other point mutations. Sixty-two percent of ONPATTRO-treated patients had non-Val30Met mutations, compared to 48% of the placebo-treated patients. (…)<br><br>**12 CLINICAL PHARMACOLOGY**

* Therapeutic areas do not necessarily reflect the CDER review division.
† Representative biomarkers are listed based on standard nomenclature as per the Human Genome Organization (HUGO) symbol and/or simplified descriptors using other common conventions. Listed biomarkers do not necessarily reflect the terminology used in labeling. The term “Nonspecific” is provided when labeling does not explicitly identify the specific biomarker(s) or when the biomarker is represented by a molecular phenotype or gene signature, and in some cases the biomarker was inferred based on the labeling language.
‡ Referenced figures and tables may be found in the labeling available at Drugs@FDA.
Blue text represents the most recent addtions and/or changes since last posted version.

NDA/ANDA/BLA Number, Label Version Date	Drug	Therapeutic Area*	Biomarker†	Labeling Sections	Labeling Text‡
					**12.2 Pharmacodynamics**<br>(…) Similar TTR reductions were observed regardless of TTR mutation, sex, age, or race. (…)<br><br>**14 CLINICAL STUDIES**<br>(…) Patients receiving ONPATTRO experienced similar improvements relative to placebo in mNIS+7 and Norfolk QoL-DN score across all subgroups including age, sex, race, region, NIS score, Val30Met mutation status, and disease stage.
**022465, 06/02/2020**	**Pazopanib (1)**	Oncology	UGT1A1	Clinical Pharmacology	**12 CLINICAL PHARMACOLOGY**<br>**12.5 Pharmacogenomics**<br>Pazopanib can increase serum total bilirubin levels [see Warnings and Precautions (5.1)]. In vitro studies showed that pazopanib inhibits UGT1A1, which glucuronidates bilirubin for elimination. A pooled pharmacogenetic analysis of 236 Caucasian patients evaluated the TA-repeat polymorphism of UGT1A1 and its potential association with hyperbilirubinemia during pazopanib treatment. In this analysis, the (TA)7/(TA)7 genotype (UGT1A1*28/*28) (underlying genetic susceptibility to Gilbert's syndrome) was associated with a statistically significant increase in the incidence of hyperbilirubinemia relative to the (TA)6/(TA)6 and (TA)6/(TA)7 genotypes.
**022465, 06/02/2020**	**Pazopanib (2)**	Oncology	HLA-B	Clinical Pharmacology	**12 CLINICAL PHARMACOLOGY**<br>**12.5 Pharmacogenomics**<br>(…) In a pooled pharmacogenetic analysis of data from 31 clinical studies of pazopanib administered as either monotherapy or in combination with other agents, ALT > 3 X ULN (NCI CTC Grade 2) occurred in 32% (42/133) of HLA-B*57:01 allele carriers and in 19% (397/2101) of non-carriers and ALT > 5 X ULN (NCI CTC Grade 3) occurred in 19% (25/133) of HLA-B*57:01 allele carriers and in 10% (213/2101) of non-carriers. In this dataset, 6% (133/2234) of the patients carried the HLA-B*57:01 allele. Liver function should be monitored in all subjects receiving pazopanib, regardless of genotype [see Warnings and Precautions (5.1)].
**103949, 01/08/2019**	**Peginterferon Alfa-2b**	Infectious Diseases	IFNL3 (IL28B)	Clinical Pharmacology	**12 CLINICAL PHARMACOLOGY**<br>**12.5 Pharmacogenomics**<br>A retrospective genome-wide association analysis1,2 of 1671 subjects (1604 subjects from Study 4 [see Clinical Studies (14.1)] and 67 subjects from another clinical trial) was performed to identify human genetic contributions to anti-HCV treatment response in previously untreated HCV genotype 1 subjects. A single nucleotide polymorphism near the gene encoding interferon-lambda-3 (IL28B rs12979860) was associated with variable SVR rates. The rs12979860 genotype was categorized as CC, CT and TT. In the pooled analysis of Caucasian, African-American, and Hispanic subjects from these trials (n=1587), SVR rates by rs12979860 genotype were as follows: CC 66% vs. CT 30% vs. TT 22%. The genotype frequencies differed depending on racial/ethnic background, but the relationship of SVR to IL28B genotype was consistent across various racial/ethnic groups (see Table 14). Other variants near the IL28B gene (e.g., rs8099917 and rs8103142) have been identified; however, they have not been shown to independently influence SVR rates during treatment with pegylated interferon alpha therapies combined with ribavirin. (See Table 13)
125293, 07/07/2022	**Pegloticase**	Rheumatology	G6PD	Boxed Warning, Contraindications, Warnings and Precautions, Adverse Reactions, Patient Counseling Information	**BOXED WARNING**<br>WARNING: ANAPHYLAXIS and INFUSION REACTIONS; G6PD DEFICIENCY ASSOCIATED HEMOLYSIS and METHEMOGLOBINEMIA<br>(…) Screen patients at risk for G6PD deficiency prior to starting KRYSTEXXA. Hemolysis and methemoglobinemia have been reported with KRYSTEXXA in patients with G6PD deficiency. Do not administer KRYSTEXXA to patients with G6PD deficiency (4, 5.3).<br><br>**4 CONTRAINDICATIONS**<br>Glucose-6-phosphate dehydrogenase (G6PD) deficiency [See Warnings and Precautions (5.3)]<br><br>**5 WARNINGS AND PRECAUTIONS**<br>**5.3 G6PD Deficiency Associated Hemolysis and Methemoglobinemia**<br>Life threatening hemolytic reactions and methemoglobinemia have been reported with KRYSTEXXA in patients with glucose-6-phosphate dehydrogenase (G6PD) deficiency. Because of the risk of hemolysis and methemoglobinemia, do not administer KRYSTEXXA to patients with G6PD deficiency. [see Contraindications (4)] Screen patients at risk for G6PD deficiency prior to starting KRYSTEXXA. For example, patients of African, Mediterranean (including Southern European and Middle Eastern), and Southern Asian ancestry are at increased risk for G6PD deficiency.<br><br>**6 ADVERSE REACTIONS**<br>**Glucose-6-phosphate dehydrogenase (G6PD) Deficiency**<br>Inform patients not to take KRYSTEXXA if they have a condition known as G6PD deficiency. Explain to patients that G6PD deficiency is more frequently found in individuals of African, Mediterranean, or Southern Asian ancestry and that they may be tested to determine if they have G6PD deficiency, unless already known [see Warnings and Precautions (5.3), Contraindications (4)].<br><br>**17 PATIENT COUNSELING INFORMATION**<br>*Glucose-6-phosphate dehydrogenase (G6PD) Deficiency*<br>Inform patients not to take KRYSTEXXA if they have a condition known as G6PD deficiency. Explain to patients that G6PD deficiency is more frequently found in individuals of African, Mediterranean, or Southern Asian ancestry and that they may be tested to determine if they have G6PD deficiency, unless already known. [See Warnings and Precautions (5.3), Contraindications (4)]
**125514, 06/17/2024**	**Pembrolizumab (1)**	Oncology	BRAF	Adverse Reactions, Clinical Studies	**6 ADVERSE REACTIONS**<br>**6.1 Clinical Trials Experience**<br>*Ipilimumab-Refractory Melanoma*

* Therapeutic areas do not necessarily reflect the CDER review division.
† Representative biomarkers are listed based on standard nomenclature as per the Human Genome Organization (HUGO) symbol and/or simplified descriptors using other common conventions. Listed biomarkers do not necessarily reflect the terminology used in labeling. The term "Nonspecific" is provided when labeling does not explicitly identify the specific biomarker(s) or when the biomarker is represented by a molecular phenotype or gene signature, and in some cases the biomarker was inferred based on the labeling language.
‡ Referenced figures and tables may be found in the labeling available at Drugs@FDA.
Blue text represents the most recent addtions and/or changes since last posted version.

NDA/ANDA/BLA Number, Label Version Date	Drug	Therapeutic Area*	Biomarker†	Labeling Sections	Labeling Text‡
					The safety of KEYTRUDA in patients with unresectable or metastatic melanoma with disease progression following ipilimumab and, if BRAF V600 mutation positive, a BRAF inhibitor, was evaluated in Study KEYNOTE-002. (…)<br><br>**14 CLINICAL STUDIES**<br>**14.1 Melanoma**<br>*Ipilimumab-Naive Melanoma*<br>(…) Patients with BRAF V600E mutation-positive melanoma were not required to have received prior BRAF inhibitor therapy. (…)<br>(…) A total of 834 patients were randomized: 277 patients to the KEYTRUDA 10 mg/kg every 3 weeks arm, 279 to the KEYTRUDA 10 mg/kg every 2 weeks arm, and 278 to the ipilimumab arm. The study population characteristics were: median age of 62 years (range: 18 to 89 years), 60% male, 98% White, 66% had no prior systemic therapy for metastatic disease, 69% ECOG PS of 0, 80% had PD-L1 positive melanoma, 18% had PD-L1 negative melanoma, and 2% had unknown PD-L1 status using the IUO assay, 65% had M1c stage disease, 68% with normal LDH, 36% with reported BRAF mutation-positive melanoma, and 9% with a history of brain metastases. Among patients with BRAF mutation-positive melanoma, 139 (46%) were previously treated with a BRAF inhibitor. (…)<br>*Ipilimumab-Refractory Melanoma*<br>(…) Randomization was stratified by ECOG performance status (0 vs. 1), LDH levels (normal vs. elevated [≥110% ULN]) and BRAF V600 mutation status (wild-type [WT] or V600E). The trial included patients with unresectable or metastatic melanoma with progression of disease; refractory to two or more doses of ipilimumab (3 mg/kg or higher) and, if BRAF V600 mutation-positive, a BRAF or MEK inhibitor; and disease progression within 24 weeks following the last dose of ipilimumab. (…)<br>(…) Twenty-three percent of patients were BRAF V600 mutation positive, 40% had elevated LDH at baseline, 82% had M1c disease, and 73% had two or more prior therapies for advanced or metastatic disease. (…)<br>*Melanoma*<br>*Ipilimumab-Refractory Melanoma*<br>(…) Randomization was stratified by ECOG performance status (0 vs. 1), LDH levels (normal vs. elevated [≥110% ULN]) and BRAF V600 mutation status (wild-type [WT] or V600E). The trial included patients with unresectable or metastatic melanoma with progression of disease; refractory to two or more doses of ipilimumab (3 mg/kg or higher) and, if BRAF V600 mutation-positive, a BRAF or MEK inhibitor; and disease progression within 24 weeks following the last dose of ipilimumab. The trial excluded patients with uveal melanoma and active brain metastasis. (…)<br>(…) The treatment arms consisted of KEYTRUDA 2 mg/kg (n=180) or 10 mg/kg (n=181) every 3 weeks or investigator's choice chemotherapy (n=179). Among the 540 randomized patients, the median age was 62 years (range: 15 to 89 years), with 43% age 65 or older; 61% male; 98% White; and ECOG performance score was 0 (55%) and 1 (45%). Twenty-three percent of patients were BRAF V600 mutation positive, 40% had elevated LDH at baseline, 82% had M1c disease, and 73% had two or more prior therapies for advanced or metastatic disease. (…)<br>*Adjuvant Treatment of Resected Melanoma*<br>*(…)* The study population characteristics were: median age of 54 years (range: 19 to 88), 25% age 65 or older; 62% male; and 94% ECOG PS of 0 and 6% ECOG PS of 1. Sixteen percent had stage IIIA, 46% had stage IIIB, 18% had stage IIIC (1-3 positive lymph nodes), and 20% had stage IIIC (≥4 positive lymph nodes); 50% were BRAF V600 mutation positive and 44% were BRAF wild-type; and 84% had PD-L1 positive melanoma with TPS ≥1% according to an IUO assay. (…)
**125514, 06/17/2024**	**Pembrolizumab (2)**	Oncology	CD274 (PD-L1)	Indications and Usage, Dosage and Administration, Adverse Reactions, Use in Specific Populations, Clinical Studies	**1 INDICATIONS AND USAGE**<br>**1.2 Non-Small Cell Lung Cancer**<br>KEYTRUDA, in combination with pemetrexed and platinum chemotherapy, is indicated for the first-line treatment of patients with metastatic nonsquamous non-small cell lung cancer (NSCLC), with no EGFR or ALK genomic tumor aberrations.<br>KEYTRUDA, in combination with carboplatin and either paclitaxel or paclitaxel protein-bound, is indicated for the first-line treatment of patients with metastatic squamous NSCLC.<br>KEYTRUDA, as a single agent, is indicated for the first-line treatment of patients with NSCLC expressing PD-L1 [Tumor Proportion Score (TPS) ≥1%] as determined by an FDA-approved test [see Dosage and Administration (2.1)], with no EGFR or ALK genomic tumor aberrations, and is:<br>· stage III where patients are not candidates for surgical resection or definitive chemoradiation, or<br>· metastatic.<br>KEYTRUDA, as a single agent, is indicated for the treatment of patients with metastatic NSCLC whose tumors express PD-L1 (TPS ≥1%) as determined by an FDA-approved test [see Dosage and Administration (2.1)], with disease progression on or after platinum-containing chemotherapy. Patients with EGFR or ALK genomic tumor aberrations should have disease progression on FDA-approved therapy for these aberrations prior to receiving KEYTRUDA.<br>**1.3 Head and Neck Squamous Cell Cancer**<br>KEYTRUDA, in combination with platinum and fluorouracil (FU), is indicated for the first-line treatment of patients with metastatic or with unresectable, recurrent head and neck squamous cell carcinoma (HNSCC).<br>KEYTRUDA, as a single agent, is indicated for the first line treatment of patients with metastatic or with unresectable, recurrent HNSCC whose tumors express PD-L1 [Combined Positive Score (CPS) ≥1] as determined by an FDA-approved test [see Dosage and Administration (2.1)].<br>KEYTRUDA, as a single agent, is indicated for the treatment of patients with recurrent or metastatic HNSCC with disease progression on or after platinum-containing chemotherapy.<br>**1.9 Gastric Cancer**<br>KEYTRUDA, in combination with trastuzumab, fluoropyrimidine- and platinum-containing chemotherapy, is indicated for the first-line treatment of adults with locally advanced unresectable or metastatic HER2-positive gastric or gastroesophageal junction (GEJ) adenocarcinoma whose tumors express PD-L1 (CPS ≥1) as determined by an FDA-approved test [see Dosage and Administration (2.1)].<br>This indication is approved under accelerated approval based on tumor response rate and durability of response [see Clinical Studies (14.9)]. Continued approval of this indication may be contingent upon verification and description of clinical benefit in the confirmatory trials.

* Therapeutic areas do not necessarily reflect the CDER review division.
† Representative biomarkers are listed based on standard nomenclature as per the Human Genome Organization (HUGO) symbol and/or simplified descriptors using other common conventions. Listed biomarkers do not necessarily reflect the terminology used in labeling. The term "Nonspecific" is provided when labeling does not explicitly identify the specific biomarker(s) or when the biomarker is represented by a molecular phenotype or gene signature, and in some cases the biomarker was inferred based on the labeling language.
‡ Referenced figures and tables may be found in the labeling available at Drugs@FDA.
Blue text represents the most recent addtions and/or changes since last posted version.

NDA/ANDA/BLA Number, Label Version Date	Drug	Therapeutic Area*	Biomarker†	Labeling Sections	Labeling Text‡
					KEYTRUDA, in combination with fluoropyrimidine- and platinum-containing chemotherapy, is indicated for the first-line treatment of adults with locally advanced unresectable or metastatic HER2-negative gastric or gastroesophageal junction (GEJ) adenocarcinoma.**1.10 Esophageal Cancer**<br>KEYTRUDA is indicated for the treatment of patients with recurrent locally advanced or metastatic squamous cell carcinoma of the esophagus whose tumors express PD-L1 (CPS ≥10) as determined by an FDA-approved test [see Dosage and Administration (2.1)], with disease progression after one or more prior lines of systemic therapy.<br>**1.10 Esophageal Cancer**<br>KEYTRUDA is indicated for the treatment of patients with locally advanced or metastatic esophageal or gastroesophageal junction (GEJ) (tumors with epicenter 1 to 5 centimeters above the GEJ) carcinoma that is not amenable to surgical resection or definitive chemoradiation either:<br>• in combination with platinum- and fluoropyrimidine-based chemotherapy, or<br>• as a single agent after one or more prior lines of systemic therapy for patients with tumors of squamous cell histology that express PD-L1 (CPS ≥10) as determined by an FDA-approved test [see Dosage and Administration (2.1)].<br>**1.11 Cervical Cancer**<br>KEYTRUDA, in combination with chemoradiotherapy (CRT), is indicated for the treatment of patients with FIGO 2014 Stage III-IVA cervical cancer.<br>KEYTRUDA, in combination with chemotherapy, with or without bevacizumab, is indicated for the treatment of patients with persistent, recurrent, or metastatic cervical cancer whose tumors express PD-L1 (CPS ≥1) as determined by an FDA-approved test *[see Dosage and Administration (2.1)]*.<br>KEYTRUDA, as a single agent, is indicated for the treatment of patients with recurrent or metastatic cervical cancer with disease progression on or after chemotherapy whose tumors express PD-L1 (CPS ≥1) as determined by an FDA-approved test *[see Dosage and Administration (2.1)]*.<br>**1.12 Hepatocellular Carcinoma**<br>KEYTRUDA is indicated for the treatment of patients with hepatocellular carcinoma (HCC) secondary to hepatitis B who have received prior systemic therapy other than a PD-1/PD-L1-containing regimen.<br>**1.19 Triple-Negative Breast Cancer**<br>KEYTRUDA is indicated for the treatment of patients with high-risk early-stage triple-negative breast cancer (TNBC) in combination with chemotherapy as neoadjuvant treatment, and then continued as a single agent as adjuvant treatment after surgery.<br>KEYTRUDA, in combination with chemotherapy, is indicated for the treatment of patients with locally recurrent unresectable or metastatic TNBC whose tumors express PD-L1 (CPS ≥10) as determined by an FDA-approved test *[see Dosage and Administration (2.1)]*.<br><br>**1 DOSAGE AND ADMINISTRATION**<br>**2.1 Patient Selection**<br>Information on FDA-approved tests for patient selection is available at:<br>**http://www.fda.gov/CompanionDiagnostics** .<br>Patient Selection for Single-Agent Treatment<br>Select patients for treatment with KEYTRUDA as a single agent based on the presence of positive PD-L1 expression in:<br>• Stage III NSCLC who are not candidates for surgical resection or definitive chemoradiation *[see Clinical Studies (14.2)]*.<br>• metastatic NSCLC *[see Clinical Studies (14.2)]*.<br>• first-line treatment of metastatic or unresectable, recurrent HNSCC *[see Clinical Studies (14.3)]*.<br>• previously treated recurrent locally advanced or metastatic esophageal cancer *[see Clinical Studies (14.10)]*.<br>• recurrent or metastatic cervical cancer with disease progression on or after chemotherapy *[see Clinical Studies (14.11)]*.<br>For the MSI-H/dMMR indications, select patients for treatment with KEYTRUDA as a single agent based on MSI-H/dMMR status in tumor specimens *[see Clinical Studies (14.7, 14.8)]*.<br>For the TMB-H indication, select patients for treatment with KEYTRUDA as a single agent based on TMB-H status in tumor specimens *[see Clinical Studies (14.17)]*.<br>Because subclonal dMMR mutations and microsatellite instability may arise in high-grade gliomas during temozolomide therapy, it is recommended to test for TMB-H, MSI-H, and dMMR in the primary tumor specimens obtained prior to initiation of temozolomide chemotherapy in patients with high-grade gliomas.<br>*Additional Patient Selection Information for MSI-H or dMMR in Patients with non-CRC Solid Tumors*<br>Due to discordance between local tests and FDA-approved tests, confirmation of MSI-H or dMMR status is recommended by an FDA-approved test in patients with MSI-H or dMMR solid tumors, if feasible. If unable to perform confirmatory MSI-H/dMMR testing, the presence of TMB ≥10 mut/Mb, as determined by an FDA-approved test, may be used to select patients for treatment *[see Clinical Studies (14.7)]*.<br>Patient Selection for Combination Therapy<br>For use of KEYTRUDA in combination with chemotherapy and trastuzumab, select patients based on the presence of positive PD-L1 expression (CPS ≥1) in locally advanced unresectable or metastatic HER2-positive gastric or gastroesophageal junction (GEJ) adenocarcinoma *[see Clinical Studies (14.9)]*.<br>For use of KEYTRUDA in combination with chemotherapy, with or without bevacizumab, select patients based on the presence of positive PD-L1 expression in persistent, recurrent, or metastatic cervical cancer *[see Clinical Studies (14.11)]*.<br>For the pMMR/not MSI-H advanced endometrial carcinoma indication, select patients for treatment with KEYTRUDA in combination with lenvatinib based on MSI or MMR status in tumor specimens *[see Clinical Studies (14.16)]*.<br>For use of KEYTRUDA in combination with chemotherapy, select patients based on the presence of positive PD-L1 expression in locally recurrent unresectable or metastatic TNBC *[see Clinical Studies (14.19)]*.<br>Additional Patient Selection Information

* Therapeutic areas do not necessarily reflect the CDER review division.
† Representative biomarkers are listed based on standard nomenclature as per the Human Genome Organization (HUGO) symbol and/or simplified descriptors using other common conventions. Listed biomarkers do not necessarily reflect the terminology used in labeling. The term “Nonspecific” is provided when labeling does not explicitly identify the specific biomarker(s) or when the biomarker is represented by a molecular phenotype or gene signature, and in some cases the biomarker was inferred based on the labeling language.
‡ Referenced figures and tables may be found in the labeling available at Drugs@FDA.
Blue text represents the most recent addtions and/or changes since last posted version.

NDA/ANDA/BLA Number, Label Version Date	Drug	Therapeutic Area*	Biomarker†	Labeling Sections	Labeling Text‡
					• An FDA-approved test for the detection of not MSI-H is currently unavailable for the selection of patients with not MSI-H endometrial carcinoma for treatment with KEYTRUDA in combination with lenvatinib *[see Clinical Studies (14.16)]*.<br><br>**6 ADVERSE REACTIONS**<br>*Previously Untreated NSCLC*<br>The safety of KEYTRUDA was investigated in KEYNOTE-042, a multicenter, open-label, randomized (1:1), active-controlled trial in 1251 patients with PD-L1 expressing, previously untreated stage III NSCLC who were not candidates for surgical resection or definitive chemoradiation or metastatic NSCLC [see Clinical Studies (14.2)]. (…)<br>*Persistent, Recurrent, or Metastatic Cervical Cancer*<br>The safety of KEYTRUDA in combination with paclitaxel and cisplatin or paclitaxel and carboplatin, with or without bevacizumab, was investigated in KEYNOTE-826, a multicenter, double-blind, randomized (1:1), placebo-controlled trial in patients with persistent, recurrent, or first-line metastatic cervical cancer who had not been treated with chemotherapy except when used concurrently as a radio-sensitizing agent [see Clinical Studies (14.11)]. A total of 616 patients, regardless of tumor PD-L1 expression, received KEYTRUDA 200 mg and chemotherapy with or without bevacizumab (n=307) every 3 weeks or placebo and chemotherapy with or without bevacizumab (n=309) every 3 weeks.<br><br>**8 USE IN SPECIFIC POPULATIONS**<br>**8.4 Pediatric Use**<br>The safety and effectiveness of KEYTRUDA as a single agent have been established in pediatric patients with cHL, PMBCL, MCC, MSI-H cancer, and TMB-H cancer. Use of KEYTRUDA in pediatric patients for these indications is supported by evidence from adequate and well-controlled studies in adults with additional pharmacokinetic and safety data in pediatric patients [see Adverse Reactions (6.1), Clinical Pharmacology (12.3), Clinical Studies (14.5, 14.6, 14.8, 14.14, 14.17)].<br>In KEYNOTE-051, 161 pediatric patients (62 pediatric patients aged 6 months to younger than 12 years and 99 pediatric patients aged 12 to 17 years) with advanced melanoma, lymphoma, or PD-L1 positive solid tumors received KEYTRUDA 2 mg/kg every 3 weeks. The median duration of exposure was 2.1 months (range: 1 day to 24 months). Adverse reactions that occurred at a ≥10% higher rate in pediatric patients when compared to adults included pyrexia (33%), vomiting (30%), upper respiratory tract infection (29%), and headache (25%). Laboratory abnormalities that occurred at a ≥10% higher rate in pediatric patients when compared to adults were leukopenia (30%), neutropenia (26%), and Grade 3 anemia (17%).<br>The safety and effectiveness of KEYTRUDA in pediatric patients have not been established in the other approved indications [see Indications and Usage (1)].<br><br>**14 CLINICAL STUDIES**<br>**14.1 Melanoma**<br>*Ipilimumab-Naive Melanoma*<br>(…) Randomization was stratified by line of therapy (0 vs. 1), ECOG PS (0 vs. 1), and PD-L1 expression (≥1% of tumor cells [positive] vs. <1% of tumor cells [negative]) according to an investigational use only (IUO) assay. (…)<br>The study population characteristics were: median age of 62 years (range: 18 to 89); 60% male; 98% White; 66% had no prior systemic therapy for metastatic disease; 69% ECOG PS of 0; 80% had PD-L1 positive melanoma, 18% had PD-L1 negative melanoma, and 2% had unknown PD-L1 status using the IUO assay; 65% had M1c stage disease; 68% with normal LDH; 36% with reported BRAF mutationpositive melanoma; and 9% with a history of brain metastases. Among patients with BRAF mutationpositive melanoma, 139 (46%) were previously treated with a BRAF inhibitor. (…)<br>*Adjuvant Treatment of Resected Melanoma*<br>(…) The major efficacy outcome measure was investigator-assessed recurrence-free survival (RFS) in the whole population and in the population with PD-L1 positive tumors where RFS was defined as the time between the date of randomization and the date of first recurrence (local, regional, or distant metastasis) or death, whichever occurs first. Patients underwent imaging every 12 weeks after the first dose of KEYTRUDA for the first two years, then every 6 months from year 3 to 5, and then annually.<br>The study population characteristics were: median age of 54 years (range: 19 to 88), 25% age 65 or older; 62% male; and 94% ECOG PS of 0 and 6% ECOG PS of 1. Sixteen percent had stage IIIA, 46% had stage IIIB, 18% had stage IIIC (1-3 positive lymph nodes), and 20% had stage IIIC (≥4 positive lymph nodes); 50% were BRAF V600 mutation positive and 44% were BRAF wild-type; and 84% had PD-L1 positive melanoma with TPS ≥1% according to an IUO assay. (…)<br>For patients with PD-L1 positive tumors, the HR was 0.54 (95% CI: 0.42, 0.69); p<0.001. The RFS benefit for KEYTRUDA compared to placebo was observed regardless of tumor PD-L1 expression.<br>**14.2 Non-Small Cell Lung Cancer**<br>*First-line treatment of metastatic nonsquamous NSCLC with pemetrexed and platinum chemotherapy*<br>The efficacy of KEYTRUDA in combination with pemetrexed and platinum chemotherapy was investigated in KEYNOTE-189 (NCT02578680), a randomized, multicenter, double-blind, activecontrolled trial conducted in 616 patients with metastatic nonsquamous NSCLC, regardless of PD-L1 tumor expression status, who had not previously received systemic therapy for metastatic disease and in whom there were no EGFR or ALK genomic tumor aberrations. Patients with autoimmune disease that required systemic therapy within 2 years of treatment; a medical condition that required immunosuppression; or who had received more than 30 Gy of thoracic radiation within the prior 26 weeks were ineligible. Randomization was stratified by smoking status (never vs. former/current), choice of platinum (cisplatin vs. carboplatin), and tumor PD-L1 status (TPS <1% [negative] vs. TPS ≥1%). (…)<br>(…) The study population characteristics were: median age of 64 years (range: 34 to 84); 49% age 65 or older; 59% male; 94% White and 3% Asian; 56% ECOG performance status of 1; and 18% with history of brain metastases. Thirty-one percent had tumor PD-L1 expression TPS <1% [negative]. (See Table 21) (…)<br>*First-line treatment of metastatic squamous NSCLC with carboplatin and either paclitaxel or nabpaclitaxel chemotherapy*

* Therapeutic areas do not necessarily reflect the CDER review division.
† Representative biomarkers are listed based on standard nomenclature as per the Human Genome Organization (HUGO) symbol and/or simplified descriptors using other common conventions. Listed biomarkers do not necessarily reflect the terminology used in labeling. The term "Nonspecific" is provided when labeling does not explicitly identify the specific biomarker(s) or when the biomarker is represented by a molecular phenotype or gene signature, and in some cases the biomarker was inferred based on the labeling language.
‡ Referenced figures and tables may be found in the labeling available at Drugs@FDA.
Blue text represents the most recent addtions and/or changes since last posted version.

NDA/ANDA/BLA Number, Label Version Date	Drug	Therapeutic Area*	Biomarker†	Labeling Sections	Labeling Text‡
					The efficacy of KEYTRUDA in combination with carboplatin and investigator's choice of either paclitaxel or nab-paclitaxel was investigated in KEYNOTE-407 (NCT02775435), a randomized, multi-center, double-blind, placebo-controlled trial conducted in 559 patients with metastatic squamous NSCLC, regardless of PD-L1 tumor expression status, who had not previously received systemic therapy for metastatic disease. Patients with autoimmune disease that required systemic therapy within 2 years of treatment; a medical condition that required immunosuppression; or who had received more than 30 Gy of thoracic radiation within the prior 26 weeks were ineligible. Randomization was stratified by tumor PDL1 status (TPS<1% [negative] vs. TPS ≥1%), choice of paclitaxel or nab-paclitaxel, and geographic region (East Asia vs. non-East Asia). (…)<br>(…) The study population characteristics were: median age of 64 years (range: 34 to 84), 49% age 65 or older; 59% male; 94% White and 3% Asian; 56% ECOG PS of 1; and 18% with history of brain metastases. Thirty-one percent had tumor PD-L1 expression TPS <1% [negative]. Seventy-two percent received carboplatin and 12% were never smokers. A total of 85 patients in the placebo and chemotherapy arm received an anti-PD-1/PD-L1 monoclonal antibody at the time of disease progression. (See Table 35) (…)<br>*First-line treatment of metastatic squamous NSCLC with carboplatin and either paclitaxel or paclitaxel protein-bound chemotherapy*<br>The efficacy of KEYTRUDA in combination with carboplatin and investigator's choice of either paclitaxel or paclitaxel protein-bound was investigated in KEYNOTE-407 (NCT02775435), a randomized, multicenter, double-blind, placebo-controlled trial conducted in 559 patients with metastatic squamous NSCLC, regardless of PD-L1 tumor expression status, who had not previously received systemic therapy for metastatic disease. Patients with autoimmune disease that required systemic therapy within 2 years of treatment; a medical condition that required immunosuppression; or who had received more than 30 Gy of thoracic radiation within the prior 26 weeks were ineligible. Randomization was stratified by tumor PDL1 status (TPS <1% [negative] vs. TPS ≥1%), choice of paclitaxel or paclitaxel protein-bound, and geographic region (East Asia vs. non-East Asia). (…)<br>The study population characteristics were: median age of 65 years (range: 29 to 88), 55% age 65 or older; 81% male; 77% White; 71% ECOG PS of 1; and 8% with a history of brain metastases. Thirty-five percent had tumor PD-L1 expression TPS <1%; 19% were from the East Asian region; and 60% received paclitaxel.<br>*First-line treatment of metastatic NSCLC as a single agent*<br>*KEYNOTE-042*<br>The efficacy of KEYTRUDA was investigated in KEYNOTE-042 (NCT02220894), a randomized, multicenter, open-label, active-controlled trial conducted in 1274 patients with stage III NSCLC who were not candidates for surgical resection or definitive chemoradiation, or patients with metastatic NSCLC. Only patients whose tumors expressed PD-L1 (TPS ≥1%) by an immunohistochemistry assay using the PD-L1 IHC 22C3 pharmDx kit and who had not received prior systemic treatment for metastatic NSCLC were eligible. Patients with EGFR or ALK genomic tumor aberrations; autoimmune disease that required systemic therapy within 2 years of treatment; a medical condition that required immunosuppression; or who had received more than 30 Gy of radiation in the thoracic region within the prior 26 weeks of initiation of study were ineligible. Randomization was stratified by ECOG PS (0 vs. 1), histology (squamous vs. nonsquamous), geographic region (East Asia vs. non-East Asia), and PD-L1 expression (TPS ≥50% vs. TPS 1 to 49%). (…)<br>The trial demonstrated a statistically significant improvement in OS for patients (PD-L1 TPS ≥50%, TPS ≥20%, TPS ≥1%) randomized to KEYTRUDA as compared with chemotherapy. Table 37 and Figure 6 summarize the efficacy results in the subgroup of patients with TPS ≥50% and in all randomized patients with TPS ≥1%. (See Table 37) (…)<br>The results of all efficacy outcome measures in the subgroup of patients with PD-L1 TPS ≥20% NSCLC were intermediate between the results of those with PD-L1 TPS ≥1% and those with PD-L1 TPS ≥50%. In a pre-specified exploratory subgroup analysis for patients with TPS 1-49% NSCLC, the median OS was 13.4 months (95% CI: 10.7, 18.2) for the pembrolizumab group and 12.1 months (95% CI: 11.0, 14.0) in the chemotherapy group, with an HR of 0.92 (95% CI: 0.77, 1.11).<br>*KEYNOTE-024*<br>The efficacy of KEYTRUDA was also investigated in KEYNOTE-024 (NCT02142738), a randomized, multicenter, open-label, active-controlled trial in 305 previously untreated patients with metastatic NSCLC. The study design was similar to that of KEYNOTE-042, except that only patients whose tumors had high PD-L1 expression (TPS of 50% or greater) by an immunohistochemistry assay using the PD-L1 IHC 22C3 pharmDx kit were eligible. (…)<br>*Previously treated NSCLC*<br>The efficacy of KEYTRUDA was investigated in KEYNOTE-010 (NCT01905657), a randomized, multicenter, open-label, active-controlled trial conducted in 1033 patients with metastatic NSCLC that had progressed following platinum-containing chemotherapy, and if appropriate, targeted therapy for EGFR or ALK genomic tumor aberrations. Eligible patients had PD-L1 expression TPS of 1% or greater by an immunohistochemistry assay using the PD-L1 IHC 22C3 pharmDx kit. Patients with autoimmune disease; a medical condition that required immunosuppression; or who had received more than 30 Gy of thoracic radiation within the prior 26 weeks were ineligible. Randomization was stratified by tumor PD-L1 expression (PD-L1 expression TPS ≥50% vs. PD-L1 expression TPS=1-49%), ECOG PS (0 vs. 1), and geographic region (East Asia vs. non-East Asia). (…)<br>The study population characteristics were: median age of 63 years (range: 20 to 88), 42% age 65 or older; 61% male; 72% White and 21% Asian; 66% ECOG PS of 1; 43% with high PD-L1 tumor expression; 21% with squamous, 70% with nonsquamous, and 8% with mixed, other or unknown histology; 91% metastatic (M1) disease; 15% with history of brain metastases; and 8% and 1% with EGFR and ALK genomic aberrations, respectively. (…)<br>**14.4 Head and Neck Squamous Cell Cancer**<br>*First-line treatment of metastatic or unresectable, recurrent HNSCC*<br>The efficacy of KEYTRUDA was investigated in KEYNOTE-048 (NCT02358031), a randomized, multicenter, open-label, active-controlled trial conducted in 882 patients with metastatic HNSCC who had not previously received systemic therapy for metastatic disease or with recurrent disease who were considered incurable by local therapies. Patients with active autoimmune disease that required systemic therapy within two years of treatment or a medical condition that required immunosuppression were ineligible. Randomization was stratified by tumor PD-L1 expression (TPS ≥50% or <50%) according to the PD-L1 IHC 22C3 pharmDx kit, HPV status according to p16 IHC (positive or negative), and ECOG PS (0 vs. 1). (…)<br>(…) A retrospective re-classification of patients' tumor PD-L1 status according to CPS using the PD-L1 IHC 22C3 pharmDx kit was conducted using the tumor specimens used for randomization. (…)

* Therapeutic areas do not necessarily reflect the CDER review division.
† Representative biomarkers are listed based on standard nomenclature as per the Human Genome Organization (HUGO) symbol and/or simplified descriptors using other common conventions. Listed biomarkers do not necessarily reflect the terminology used in labeling. The term "Nonspecific" is provided when labeling does not explicitly identify the specific biomarker(s) or when the biomarker is represented by a molecular phenotype or gene signature, and in some cases the biomarker was inferred based on the labeling language.
‡ Referenced figures and tables may be found in the labeling available at Drugs@FDA.
Blue text represents the most recent addtions and/or changes since last posted version.

NDA/ANDA/BLA Number, Label Version Date	Drug	Therapeutic Area*	Biomarker†	Labeling Sections	Labeling Text‡
					The study population characteristics were: median age of 61 years (range: 20 to 94), 36% age 65 or older; 83% male; 73% White, 20% Asian and 2.4% Black; 61% had ECOG PS of 1; and 79% were former/current smokers. Twenty-two percent of patients' tumors were HPV-positive, 23% had PD-L1 TPS ≥50%, and 95% had Stage IV disease (Stage IVA 19%, Stage IVB 6%, and Stage IVC 70%). Eighty-five percent of patients' tumors had PD-L1 expression of CPS ≥1 and 43% had CPS ≥20. (…)<br>The trial also demonstrated a statistically significant improvement in OS for the subgroup of patients with PD-L1 CPS ≥1 randomized to KEYTRUDA as a single agent compared to those randomized to cetuximab in combination with chemotherapy. (…)<br>In KEYNOTE-048, OS HRs for patients randomized to KEYTRUDA in combination with chemotherapy, compared with cetuximab in combination with chemotherapy, were similar for all populations regardless of PD-L1 expression in a pre-specified interim analysis: ITT (HR 0.77, 95% CI: 0.63, 0.93), CPS ≥1 (HR 0.71, 95% CI: 0.57, 0.88), CPS ≥20 (HR 0.69, 95% CI:0.51, 0.94).<br>**14.7 Urothelial Carcinoma**<br>*Cisplatin Ineligible Patients with Urothelial Carcinoma*<br>Among the 370 patients, 30% (n = 110) had tumors that expressed PD-L1 with a CPS ≥10. PD-L1 status was determined using the PD-L1 IHC 22C3 pharmDx kit. The study population characteristics of these 110 patients were: median age of 73 years; 68% male; and 87% White. Eighty-two percent had M1 disease, and 18% had M0 disease. (See Table 46) (…)<br>*Previously Untreated Urothelial Carcinoma*<br>(…) The independent Data Monitoring Committee (iDMC) for the study conducted a review of early data and found that in patients classified as having low PD-L1 expression (CPS <10), those treated with KEYTRUDA monotherapy had decreased survival compared to those who received platinum-based chemotherapy. The iDMC recommended to stop further accrual of patients with low PD-L1 expression in the monotherapy arm, however, no other changes were recommended, including any change of therapy for patients who had already been randomized to and were receiving treatment in the monotherapy arm.<br>**14.8 Microsatellite Instability-High Cancer**<br>(See Table 49)<br>**14.9 Gastric Cancer**<br>Among the 259 patients, 55% (n = 143) had tumors that expressed PD-L1 with a CPS ≥1 and microsatellite stable (MSS) tumor status or undetermined MSI or MMR status. PD-L1 status was determined using the PD-L1 IHC 22C3 pharmDx kit. (…)<br>**14.10 Esophageal Cancer**<br>*KEYNOTE-181*<br>The efficacy of KEYTRUDA was investigated in KEYNOTE-181 (NCT02564263), a multicenter, randomized, open-label, active-controlled trial that enrolled 628 patients with recurrent locally advanced or metastatic esophageal cancer who progressed on or after one prior line of systemic treatment for advanced disease. Patients with HER2/neu positive esophageal cancer were required to have received treatment with approved HER2/neu targeted therapy. All patients were required to have tumor specimens for PD-L1 testing at a central laboratory; PD-L1 status was determined using the PD-L1 IHC 22C3 pharmDx kit. (…)<br>The major efficacy outcome measure was OS evaluated in the following co-primary populations: patients with ESCC, patients with tumors expressing PD-L1 CPS ≥10, and all randomized patients. (…)<br>A total of 628 patients were enrolled and randomized to KEYTRUDA (n=314) or investigator's treatment of choice (n=314). Of these 628 patients, 167 (27%) had ESCC that expressed PD-L1 with a CPS ≥10. (…)<br>The observed OS hazard ratio was 0.77 (95% CI: 0.63, 0.96) in patients with ESCC, 0.70 (95% CI: 0.52, 0.94) in patients with tumors expressing PD-L1 CPS ≥10, and 0.89 (95% CI: 0.75, 1.05) in all randomized patients. On further examination in patients whose ESCC tumors expressed PD-L1 (CPS ≥10), an improvement in OS was observed among patients randomized to KEYTRUDA as compared with chemotherapy. Table 52 and Figure 12 summarize the key efficacy measures for KEYNOTE-181 for patients with ESCC CPS ≥10.<br>KEYNOTE-180<br>Among the 121 patients enrolled, 29% (n=35) had ESCC that expressed PD-L1 CPS ≥10. The baseline characteristics of these 35 patients were: median age of 65 years (range: 47 to 81), 51% age 65 or older; 71% male; 26% White and 69% Asian; 40% had an ECOG PS of 0 and 60% had an ECOG PS of 1. One hundred percent had M1 disease.<br>The ORR in the 35 patients with ESCC expressing PD-L1 was 20% (95% CI: 8, 37). Among the 7 responding patients, the DoR ranged from 4.2 to 25.1+ months, with 5 patients (71%) having responses of 6 months or longer and 3 patients (57%) having responses of 12 months or longer.<br>**14.11 Cervical Cancer**<br>Among the 98 patients in Cohort E, 77 (79%) had tumors that expressed PD-L1 with a CPS ≥ 1 and received at least one line of chemotherapy in the metastatic setting. PD-L1 status was determined using the IHC 22C3 pharmDx kit. The baseline characteristics of these 77 patients were: median age of 45 years (range: 27 to 75); 81% White, 14% Asian, and 3% Black; 32% ECOG PS of 0 and 68% ECOG PS of 1; 92% had squamous cell carcinoma, 6% adenocarcinoma, and 1% adenosquamous histology; 95% had M1 disease and 5% had recurrent disease; and 35% had one and 65% had two or more prior lines of therapy in the recurrent or metastatic setting.<br>No responses were observed in patients whose tumors did not have PD-L1 expression (CPS <1). Efficacy results are summarized in Table 53 for patients with PD-L1 expression (CPS ≥1). (…)<br>**14.14 Renal Cell Carcinoma**<br>The efficacy of KEYTRUDA in combination with axitinib was investigated in KEYNOTE-426 (NCT02853331), a randomized, multicenter, open-label trial conducted in 861 patients who had not received systemic therapy for advanced RCC. Patients were enrolled regardless of PD-L1 tumor expression status. Patients with active autoimmune disease requiring systemic immunosuppression within the last 2 years were ineligible. (…)<br>Consistent results were observed across pre-specified subgroups, IMDC risk categories and PD-L1 tumor expression status. (…)<br>**14.19 Triple-Negative Breast Cancer**<br>The efficacy of KEYTRUDA in combination with paclitaxel, paclitaxel protein-bound, or gemcitabine and carboplatin was investigated in KEYNOTE-355 (NCT02819518), a multicenter, double-blind, randomized, placebo-controlled trial conducted in 847 patients with locally recurrent unresectable or metastatic

Table of Pharmacogenomic Biomarkers in Drug Labeling

Last Updated: 06/2024

NDA/ANDA/BLA Number, Label Version Date	Drug	Therapeutic Area*	Biomarker†	Labeling Sections	Labeling Text‡
					TNBC, regardless of tumor PD-L1 expression, who had not been previously treated with chemotherapy in the metastatic setting. Patients with active autoimmune disease that required systemic therapy within 2 years of treatment or a medical condition that required immunosuppression were ineligible. Randomization was stratified by chemotherapy treatment (paclitaxel or paclitaxel protein-bound vs. gemcitabine and carboplatin), tumor PD-L1 expression (CPS ≥1 vs. CPS <1) according to the PD-L1 IHC 22C3 pharmDx kit, and prior treatment with the same class of chemotherapy in the neoadjuvant setting (yes vs. no).<br>(…)<br>The study population characteristics for patients were: median age of 53 years (range: 22 to 85), 21% age 65 or older; 100% female; 68% White, 21% Asian, and 4% Black; 60% ECOG PS of 0 and 40% ECOG PS of 1; and 68% were post-menopausal status. Seventy-five percent of patients had tumor PD-L1 expression CPS ≥1 and 38% had tumor PD-L1 expression CPS ≥10.<br>Table 68 and Figure 16 summarize the efficacy results for KEYNOTE-355.
**125514, 06/17/2024**	**Pembrolizumab (3)**	Oncology	Microsatellite Instability, Mismatch Repair	Indications and Usage, Dosage and Administration, Adverse Reactions, Use in Specific Populations, Clinical Studies	**1 INDICATIONS AND USAGE**<br>**1.7 Microsatellite Instability-High or Mismatch Repair Deficient Cancer**<br>KEYTRUDA is indicated for the treatment of adult and pediatric patients with unresectable or metastatic microsatellite instability-high (MSI-H) or mismatch repair deficient (dMMR) solid tumors, as determined by an FDA-approved test, that have progressed following prior treatment and who have no satisfactory alternative treatment options [see Dosage and Administration (2.1)].<br>**1.8 Microsatellite Instability-High or Mismatch Repair Deficient Colorectal Cancer**<br>KEYTRUDA is indicated for the treatment of patients with unresectable or metastatic MSI-H or dMMR colorectal cancer (CRC) as determined by an FDA-approved test [see Dosage and Administration (2.1)].<br>**1.15 Endometrial Carcinoma**<br>KEYTRUDA, in combination with lenvatinib, is indicated for the treatment of patients with advanced endometrial carcinoma that is mismatch repair proficient (pMMR) as determined by an FDA-approved test or not MSI-H, who have disease progression following prior systemic therapy in any setting and are not candidates for curative surgery or radiation [see Dosage and Administration (2.1)]. KEYTRUDA, as a single agent, is indicated for the treatment of patients with advanced endometrial carcinoma that is MSI-H or dMMR, as determined by an FDA-approved test, who have disease progression following prior systemic therapy in any setting and are not candidates for curative surgery or radiation [see Dosage and Administration (2.1)].<br><br>**2 DOSAGE AND ADMINISTRATION**<br>2.1 Patient Selection<br>Information on FDA-approved tests for patient selection is available at: http://www.fda.gov/CompanionDiagnostics .<br>Patient Selection for Single-Agent Treatment<br>Select patients for treatment with KEYTRUDA as a single agent based on the presence of positive PD-L1 expression in:<br>• Stage III NSCLC who are not candidates for surgical resection or definitive chemoradiation [see Clinical Studies (14.2)].<br>• metastatic NSCLC [see Clinical Studies (14.2)].<br>• first-line treatment of metastatic or unresectable, recurrent HNSCC [see Clinical Studies (14.3)].<br>• previously treated recurrent locally advanced or metastatic esophageal cancer [see Clinical Studies (14.10)].<br>• recurrent or metastatic cervical cancer with disease progression on or after chemotherapy [see Clinical Studies (14.11)].<br>For the MSI-H/dMMR indications, select patients for treatment with KEYTRUDA as a single agent based on MSI-H/dMMR status in tumor specimens [see Clinical Studies (14.7, 14.8)].<br>For the TMB-H indication, select patients for treatment with KEYTRUDA as a single agent based on TMB-H status in tumor specimens [see Clinical Studies (14.17)].<br>Because subclonal dMMR mutations and microsatellite instability may arise in high-grade gliomas during temozolomide therapy, it is recommended to test for TMB-H, MSI-H, and dMMR in the primary tumor specimens obtained prior to initiation of temozolomide chemotherapy in patients with high-grade gliomas.<br>Additional Patient Selection Information for MSI-H or dMMR in Patients with non-CRC Solid Tumors<br>Due to discordance between local tests and FDA-approved tests, confirmation of MSI-H or dMMR status is recommended by an FDA-approved test in patients with MSI-H or dMMR solid tumors, if feasible. If unable to perform confirmatory MSI-H/dMMR testing, the presence of TMB ≥10 mut/Mb, as determined by an FDA-approved test, may be used to select patients for treatment [see Clinical Studies (14.7)].<br>Patient Selection for Combination Therapy<br>For use of KEYTRUDA in combination with chemotherapy and trastuzumab, select patients based on the presence of positive PD-L1 expression (CPS ≥1) in locally advanced unresectable or metastatic HER2-positive gastric or gastroesophageal junction (GEJ) adenocarcinoma [see Clinical Studies (14.9)].<br>For use of KEYTRUDA in combination with chemotherapy, with or without bevacizumab, select patients based on the presence of positive PD-L1 expression in persistent, recurrent, or metastatic cervical cancer [see Clinical Studies (14.11)].<br>For the pMMR/not MSI-H advanced endometrial carcinoma indication, select patients for treatment with KEYTRUDA in combination with lenvatinib based on MSI or MMR status in tumor specimens [see Clinical Studies (14.16)].<br>For use of KEYTRUDA in combination with chemotherapy, select patients based on the presence of positive PD-L1 expression in locally recurrent unresectable or metastatic TNBC [see Clinical Studies (14.19)].<br>Additional Patient Selection Information<br>♣ An FDA-approved test for the detection of not MSI-H is currently unavailable for the selection of patients with not MSI-H endometrial carcinoma for treatment with KEYTRUDA in combination with lenvatinib [see Clinical Studies (14.16)].<br>**2.2 Recommended Dosage**<br>See Table 1<br><br>**6 ADVERSE REACTIONS**

* Therapeutic areas do not necessarily reflect the CDER review division.

† Representative biomarkers are listed based on standard nomenclature as per the Human Genome Organization (HUGO) symbol and/or simplified descriptors using other common conventions. Listed biomarkers do not necessarily reflect the terminology used in labeling. The term "Nonspecific" is provided when labeling does not explicitly identify the specific biomarker(s) or when the biomarker is represented by a molecular phenotype or gene signature, and in some cases the biomarker was inferred based on the labeling language.

‡ Referenced figures and tables may be found in the labeling available at Drugs@FDA.

Blue text represents the most recent addtions and/or changes since last posted version.

NDA/ANDA/BLA Number, Label Version Date	Drug	Therapeutic Area*	Biomarker†	Labeling Sections	Labeling Text‡
					Microsatellite Instability-High or Mismatch Repair Deficient Cancer<br>The safety of KEYTRUDA was investigated in 504 patients with MSI-H or dMMR cancer enrolled in KEYNOTE-158, KEYNOTE-164, and KEYNOTE-051 [see Clinical Studies (14.7)]. The median duration of exposure to KEYTRUDA was 6.2 months (range: 1 day to 53.5 months). Adverse reactions occurring in patients with MSI-H or dMMR cancer were similar to those occurring in patients with other solid tumors who received KEYTRUDA as a single agent.<br>Microsatellite Instability-High or Mismatch Repair Deficient Colorectal Cancer<br>Among the 153 patients with MSI-H or dMMR CRC enrolled in KEYNOTE-177 [see Clinical Studies (14.9)] treated with KEYTRUDA, the median duration of exposure to KEYTRUDA was 11.1 months (range: 1 day to 30.6 months). Patients with autoimmune disease or a medical condition that required immunosuppression were ineligible. Adverse reactions occurring in patients with MSI-H or dMMR CRC were similar to those occurring in 2799 patients with melanoma or NSCLC treated with KEYTRUDA as a single agent.<br>Endometrial Carcinoma<br>The safety of KEYTRUDA in combination with lenvatinib was investigated in KEYNOTE-775, a multicenter, open-label, randomized (1:1), active-controlled trial in patients with advanced endometrial carcinoma previously treated with at least one prior platinum-based chemotherapy regimen in any setting, including in the neoadjuvant and adjuvant settings [see Clinical Studies (14.15)]. Patients with endometrial carcinoma that is not MSI-H or dMMR received KEYTRUDA 200 mg every 3 weeks in combination with lenvatinib 20mg orally once daily (n=342) or received doxorubicin or paclitaxel (n=325).<br>For patients with not MSI-H or dMMR tumor status, the median duration of study treatment was 7.2 months (range 1 day to 26.8 months) and the median duration of exposure to KEYTRUDA was 6.8 months (range: 1 day to 25.8 months). (See Tables 35 and 36) (…)<br>*As a Single Agent for the Treatment of Advanced MSI-H or dMMR Endometrial Carcinoma*<br>Among the 90 patients with MSI-H or dMMR endometrial carcinoma enrolled in KEYNOTE-158 [see Clinical Studies (14.16)] treated with KEYTRUDA as a single agent, the median duration of exposure to KEYTRUDA was 8.3 months (range: 1 day to 26.9 months). Adverse reactions occurring in patients with endometrial carcinoma were similar to those occurring in 2799 patients with melanoma or NSCLC treated with KEYTRUDA as a single agent.<br><br>**8 USE IN SPECIFIC POPULATIONS**<br>**8.4 Pediatric Use**<br>The safety and effectiveness of KEYTRUDA as a single agent have been established in pediatric patients with cHL, PMBCL, MCC, MSI-H cancer, and TMB-H cancer. Use of KEYTRUDA in pediatric patients for these indications is supported by evidence from adequate and well-controlled studies in adults with additional pharmacokinetic and safety data in pediatric patients [see Adverse Reactions (6.1), Clinical Pharmacology (12.3), Clinical Studies (14.4, 14.5, 14.7, 14.13, 14.16)]. (…)<br><br>**14 CLINICAL STUDIES**<br>**14.7 Microsatellite Instability-High or Mismatch Repair Deficient Cancer**<br>The efficacy of KEYTRUDA was investigated in patients with MSI-H or mismatch repair deficient (dMMR), solid tumors enrolled in one of five uncontrolled, open-label, multi-cohort, multi-center, single-arm trials. Patients with active autoimmune disease or a medical condition that required immunosuppression were ineligible across the five trials. (See Table 56) (…)<br>A total of 149 patients with MSI-H or dMMR cancers were identified across the five trials. Among these 149 patients, the baseline characteristics were: median age of 55 years, 36% age 65 or older; 56% male; 77% White, 19% Asian, and 2% Black; and 36% ECOG PS of 0 and 64% ECOG PS of 1. Ninety-eight percent of patients had metastatic disease and 2% had locally advanced, unresectable disease. The median number of prior therapies for metastatic or unresectable disease was two. Eighty-four percent of patients with metastatic CRC and 53% of patients with other solid tumors received two or more prior lines of therapy.<br>The identification of MSI-H or dMMR tumor status for the majority of patients (135/149) was prospectively determined using local laboratory-developed, polymerase chain reaction (PCR) tests for MSI-H status or immunohistochemistry (IHC) tests for dMMR. Fourteen of the 149 patients were retrospectively identified as MSI-H by testing tumor samples from a total of 415 patients using a central laboratory developed PCR test. Forty-seven patients had dMMR cancer identified by IHC, 60 had MSI-H identified by PCR, and 42 were identified using both tests.<br>Efficacy results are summarized in Tables 57 and 58.<br>**14.8 Microsatellite Instability-High or Mismatch Repair Deficient Colorectal Cancer**<br>The efficacy of KEYTRUDA was investigated in KEYNOTE-177 (NCT02563002), a multicenter, randomized, open-label, active-controlled trial that enrolled 307 patients with previously untreated unresectable or metastatic MSI-H or dMMR CRC. MSI or MMR tumor status was determined locally using polymerase chain reaction (PCR) or immunohistochemistry (IHC), respectively. Patients with autoimmune disease or a medical condition that required immunosuppression were ineligible. (See Table 59) (…)<br>**14.9 Gastric Cancer**<br>First-line Treatment of Locally Advanced Unresectable or Metastatic HER2-Positive Gastric or Gastroesophageal Junction (GEJ) Adenocarcinoma<br>(…)At the time of the interim analysis, ORR and DoR were assessed in the first 264 patients randomized. Among the 264 patients, the population characteristics were: median age of 62 years (range: 19 to 84), 41% age 65 or older; 82% male; 63% White, 31% Asian, and 0.8% Black; 47% ECOG PS of 0 and 53% ECOG PS of 1. Ninety-seven percent of patients had metastatic disease (stage IV) and 3% had locally advanced unresectable disease. Eighty-seven percent had tumors that expressed PD-L1 with a CPS ≥1. Ninety-one percent (n=240) had tumors that were not MSI-H, 1% (n=2) had tumors that were MSI-H, and in 8% (n=22) the status was not known. Eighty-seven percent of patients received CAPOX.<br>Previously Treated Gastric or Gastroesophageal Junction (GEJ) Adenocarcinoma<br>Among the 259 patients, 55% (n = 143) had tumors that expressed PD-L1 with a CPS ≥1 and microsatellite stable (MSS) tumor status or undetermined MSI or MMR status. PD-L1 status was determined using the PD-L1 IHC 22C3 pharmDx kit. The baseline characteristics of these 143 patients were: median age of 64 years, 47% age 65 or older; 77% male; 82% White and 11% Asian; and 43% ECOG PS of 0 and 57% ECOG PS of 1. Eighty-five percent had M1 disease and 7% had M0 disease. Fifty-one percent had two and 49% had three or more prior lines of therapy in the recurrent or metastatic setting. (…)<br>**14.15 Endometrial Carcinoma**

* Therapeutic areas do not necessarily reflect the CDER review division.
† Representative biomarkers are listed based on standard nomenclature as per the Human Genome Organization (HUGO) symbol and/or simplified descriptors using other common conventions. Listed biomarkers do not necessarily reflect the terminology used in labeling. The term “Nonspecific” is provided when labeling does not explicitly identify the specific biomarker(s) or when the biomarker is represented by a molecular phenotype or gene signature, and in some cases the biomarker was inferred based on the labeling language.
‡ Referenced figures and tables may be found in the labeling available at Drugs@FDA.
Blue text represents the most recent addtions and/or changes since last posted version.

NDA/ANDA/BLA Number, Label Version Date	Drug	Therapeutic Area*	Biomarker†	Labeling Sections	Labeling Text‡
					Patients with endometrial carcinoma that were not MSI-H or dMMR were stratified by ECOG performance status, geographic region, and history of pelvic radiation. (…)<br>Efficacy results for the not MSI-H or dMMR patients are summarized in Table 67 and Figures 17 and 18. (See Table 67 and Figures 17 and 18) (…)
**125514, 06/17/2024**	Pembrolizumab (4)	Oncology	EGFR	Indications and Usage, Adverse Reactions, Clinical Studies	**1 INDICATIONS AND USAGE**<br>**1.2 Non-Small Cell Lung Cancer**<br>KEYTRUDA, in combination with pemetrexed and platinum chemotherapy, is indicated for the first-line treatment of patients with metastatic nonsquamous non-small cell lung cancer (NSCLC), with no EGFR or ALK genomic tumor aberrations.<br>KEYTRUDA, in combination with carboplatin and either paclitaxel or paclitaxel protein-bound, is indicated for the first-line treatment of patients with metastatic squamous NSCLC.<br>KEYTRUDA, as a single agent, is indicated for the first-line treatment of patients with NSCLC expressing PD-L1 [Tumor Proportion Score (TPS) ≥1%] as determined by an FDA-approved test [see Dosage and Administration (2.1)], with no EGFR or ALK genomic tumor aberrations, and is:<br>• stage III where patients are not candidates for surgical resection or definitive chemoradiation, or<br>• metastatic.<br>KEYTRUDA, as a single agent, is indicated for the treatment of patients with metastatic NSCLC whose tumors express PD-L1 (TPS ≥1%) as determined by an FDA-approved test [see Dosage and Administration (2.1)], with disease progression on or after platinum-containing chemotherapy. Patients with EGFR or ALK genomic tumor aberrations should have disease progression on FDA-approved therapy for these aberrations prior to receiving KEYTRUDA.<br><br>**6 ADVERSE REACTIONS**<br>**6.1 Clinical Trials Experience**<br>*NSCLC*<br>*First-line treatment of metastatic nonsquamous NSCLC with pemetrexed and platinum chemotherapy*<br>The safety of KEYTRUDA in combination with pemetrexed and investigator's choice of platinum (either carboplatin or cisplatin) was investigated in KEYNOTE-189, a multicenter, double-blind, randomized (2:1), active-controlled trial in patients with previously untreated, metastatic nonsquamous NSCLC with no EGFR or ALK genomic tumor aberrations [see Clinical Studies (14.2)]. (…)<br>*Previously Untreated NSCLC*<br>The safety of KEYTRUDA was investigated in KEYNOTE-042, a multicenter, open-label, randomized (1:1), active-controlled trial in 1251 patients with PD-L1 expressing, previously untreated stage III NSCLC who were not candidates for surgical resection or definitive chemoradiation or metastatic NSCLC [see Clinical Studies (14.2)]. Patients received KEYTRUDA 200 mg every 3 weeks (n=636) or investigator's choice of chemotherapy (n=615), consisting of pemetrexed and carboplatin followed by optional pemetrexed (n=312) or paclitaxel and carboplatin followed by optional pemetrexed (n=303) every 3 weeks. Patients with EGFR or ALK genomic tumor aberrations; autoimmune disease that required systemic therapy within 2 years of treatment; a medical condition that required immunosuppression; or who had received more than 30 Gy of thoracic radiation within the prior 26 weeks were ineligible. (…)<br>*Previously Treated NSCLC*<br>The safety of KEYTRUDA was investigated in KEYNOTE-010, a multicenter, open-label, randomized (1:1:1), active-controlled trial, in patients with advanced NSCLC who had documented disease progression following treatment with platinum-based chemotherapy and, if positive for EGFR or ALK genetic aberrations, appropriate therapy for these aberrations [see Clinical Studies (14.2)].<br><br>**14 CLINICAL STUDIES**<br>**14.2 Non-Small Cell Lung Cancer**<br>First-line treatment of metastatic nonsquamous NSCLC with pemetrexed and platinum chemotherapy<br>The efficacy of KEYTRUDA in combination with pemetrexed and platinum chemotherapy was investigated in KEYNOTE-189 (NCT02578680), a randomized, multicenter, double-blind, activecontrolled trial conducted in 616 patients with metastatic nonsquamous NSCLC, regardless of PD-L1 tumor expression status, who had not previously received systemic therapy for metastatic disease and in whom there were no EGFR or ALK genomic tumor aberrations. (…)<br>First-line treatment of metastatic NSCLC as a single agent<br>*KEYNOTE-042*<br>The efficacy of KEYTRUDA was investigated in KEYNOTE-042 (NCT02220894), a randomized, multicenter, open-label, active-controlled trial conducted in 1274 patients with stage III NSCLC who were not candidates for surgical resection or definitive chemoradiation, or patients with metastatic NSCLC. Only patients whose tumors expressed PD-L1 (TPS ≥1%) by an immunohistochemistry assay using the PD-L1 IHC 22C3 pharmDx kit and who had not received prior systemic treatment for metastatic NSCLC were eligible. Patients with EGFR or ALK genomic tumor aberrations; autoimmune disease that required systemic therapy within 2 years of treatment; a medical condition that required immunosuppression; or who had received more than 30 Gy of radiation in the thoracic region within the prior 26 weeks of initiation of study were ineligible. (…)<br>Previously treated NSCLC<br>The efficacy of KEYTRUDA was investigated in KEYNOTE-010 (NCT01905657), a randomized, multicenter, open-label, active-controlled trial conducted in 1033 patients with metastatic NSCLC that had progressed following platinum-containing chemotherapy, and if appropriate, targeted therapy for EGFR or ALK genomic tumor aberrations. (…)<br>(…) The study population characteristics were: median age of 63 years (range: 20 to 88), 42% age 65 or older; 61% male; 72% White and 21% Asian; 66% ECOG PS of 1; 43% with high PD-L1 tumor expression; 21% with squamous, 70% with nonsquamous, and 8% with mixed, other or unknown histology; 91% metastatic (M1) disease; 15% with history of brain metastases; and 8% and 1% with EGFR and ALK genomic aberrations, respectively. All patients had received prior therapy with a platinumdoublet regimen, 29% received two or more prior therapies for their metastatic disease. (…)

* Therapeutic areas do not necessarily reflect the CDER review division.
† Representative biomarkers are listed based on standard nomenclature as per the Human Genome Organization (HUGO) symbol and/or simplified descriptors using other common conventions. Listed biomarkers do not necessarily reflect the terminology used in labeling. The term "Nonspecific" is provided when labeling does not explicitly identify the specific biomarker(s) or when the biomarker is represented by a molecular phenotype or gene signature, and in some cases the biomarker was inferred based on the labeling language.
‡ Referenced figures and tables may be found in the labeling available at Drugs@FDA.
Blue text represents the most recent addtions and/or changes since last posted version.

NDA/ANDA/BLA Number, Label Version Date	Drug	Therapeutic Area*	Biomarker†	Labeling Sections	Labeling Text‡
125514, 06/17/2024	Pembrolizumab (5)	Oncology	ALK	Indications and Usage, Adverse Reactions, Clinical Studies	**1 INDICATIONS AND USAGE**<br>**1.2 Non-Small Cell Lung Cancer**<br>KEYTRUDA, in combination with pemetrexed and platinum chemotherapy, is indicated for the first-line treatment of patients with metastatic nonsquamous non-small cell lung cancer (NSCLC), with no EGFR or ALK genomic tumor aberrations.<br>KEYTRUDA, in combination with carboplatin and either paclitaxel or paclitaxel protein-bound, is indicated for the first-line treatment of patients with metastatic squamous NSCLC.<br>KEYTRUDA, as a single agent, is indicated for the first-line treatment of patients with NSCLC expressing PD-L1 [Tumor Proportion Score (TPS) ≥1%] as determined by an FDA-approved test [see Dosage and Administration (2.1)], with no EGFR or ALK genomic tumor aberrations, and is:<br>• stage III where patients are not candidates for surgical resection or definitive chemoradiation, or<br>• metastatic.<br>KEYTRUDA, as a single agent, is indicated for the treatment of patients with metastatic NSCLC whose tumors express PD-L1 (TPS ≥1%) as determined by an FDA-approved test [see Dosage and Administration (2.1)], with disease progression on or after platinum-containing chemotherapy. Patients with EGFR or ALK genomic tumor aberrations should have disease progression on FDA-approved therapy for these aberrations prior to receiving KEYTRUDA.<br><br>**6 ADVERSE REACTIONS**<br>**6.1 Clinical Trials Experience**<br>*NSCLC*<br>*First-line treatment of metastatic nonsquamous NSCLC with pemetrexed and platinum chemotherapy*<br>The safety of KEYTRUDA in combination with pemetrexed and investigator's choice of platinum (either carboplatin or cisplatin) was investigated in KEYNOTE-189, a multicenter, double-blind, randomized (2:1), active-controlled trial in patients with previously untreated, metastatic nonsquamous NSCLC with no EGFR or ALK genomic tumor aberrations [see Clinical Studies (14.2)]. (…)<br>Previously Untreated NSCLC<br>The safety of KEYTRUDA was investigated in KEYNOTE-042, a multicenter, open-label, randomized (1:1), active-controlled trial in 1251 patients with PD-L1 expressing, previously untreated stage III NSCLC who were not candidates for surgical resection or definitive chemoradiation or metastatic NSCLC [see Clinical Studies (14.2)]. Patients received KEYTRUDA 200 mg every 3 weeks (n=636) or investigator's choice of chemotherapy (n=615), consisting of pemetrexed and carboplatin followed by optional pemetrexed (n=312) or paclitaxel and carboplatin followed by optional pemetrexed (n=303) every 3 weeks. Patients with EGFR or ALK genomic tumor aberrations; autoimmune disease that required systemic therapy within 2 years of treatment; a medical condition that required immunosuppression; or who had received more than 30 Gy of thoracic radiation within the prior 26 weeks were ineligible. (…)<br>*Previously Treated NSCLC*<br>The safety of KEYTRUDA was investigated in KEYNOTE-010, a multicenter, open-label, randomized (1:1:1), active-controlled trial, in patients with advanced NSCLC who had documented disease progression following treatment with platinum-based chemotherapy and, if positive for EGFR or ALK genetic aberrations, appropriate therapy for these aberrations [see Clinical Studies (14.2)].<br><br>**14 CLINICAL STUDIES**<br>**14.2 Non-Small Cell Lung Cancer**<br><u>First-line treatment of metastatic nonsquamous NSCLC with pemetrexed and platinum chemotherapy</u><br>The efficacy of KEYTRUDA in combination with pemetrexed and platinum chemotherapy was investigated in KEYNOTE-189 (NCT02578680), a randomized, multicenter, double-blind, activecontrolled trial conducted in 616 patients with metastatic nonsquamous NSCLC, regardless of PD-L1 tumor expression status, who had not previously received systemic therapy for metastatic disease and in whom there were no EGFR or ALK genomic tumor aberrations. (…)<br><u>First-line treatment of metastatic NSCLC as a single agent</u><br>*KEYNOTE-042*<br>The efficacy of KEYTRUDA was investigated in KEYNOTE-042 (NCT02220894), a randomized, multicenter, open-label, active-controlled trial conducted in 1274 patients with stage III NSCLC who were not candidates for surgical resection or definitive chemoradiation, or patients with metastatic NSCLC. Only patients whose tumors expressed PD-L1 (TPS ≥1%) by an immunohistochemistry assay using the PD-L1 IHC 22C3 pharmDx kit and who had not received prior systemic treatment for metastatic NSCLC were eligible. Patients with EGFR or ALK genomic tumor aberrations; autoimmune disease that required systemic therapy within 2 years of treatment; a medical condition that required immunosuppression; or who had received more than 30 Gy of radiation in the thoracic region within the prior 26 weeks of initiation of study were ineligible. (…)<br><u>Previously treated NSCLC</u><br>The efficacy of KEYTRUDA was investigated in KEYNOTE-010 (NCT01905657), a randomized, multicenter, open-label, active-controlled trial conducted in 1033 patients with metastatic NSCLC that had progressed following platinum-containing chemotherapy, and if appropriate, targeted therapy for EGFR or ALK genomic tumor aberrations. (…)<br>(…) The study population characteristics were: median age of 63 years (range: 20 to 88), 42% age 65 or older; 61% male; 72% White and 21% Asian; 66% ECOG PS of 1; 43% with high PD-L1 tumor expression; 21% with squamous, 70% with nonsquamous, and 8% with mixed, other or unknown histology; 91% metastatic (M1) disease; 15% with history of brain metastases; and 8% and 1% with EGFR and ALK genomic aberrations, respectively. All patients had received prior therapy with a platinumdoublet regimen, 29% received two or more prior therapies for their metastatic disease. (…)
125514, 06/17/2024	Pembrolizumab (6)	Oncology	Tumor Mutational Burden	Indications and Usage, Dosage and Administration, Use in Specific	**1 INDICATIONS AND USAGE**<br>**1.17 Tumor Mutational Burden-High Cancer**<br>KEYTRUDA is indicated for the treatment of adult and pediatric patients with unresectable or metastatic tumor mutational burden-high (TMB-H) [≥10 mutations/megabase (mut/Mb)] solid tumors, as determined by an FDA-approved test [see Dosage and Administration (2.1)], that have progressed following prior treatment and who have no satisfactory alternative treatment options.

* Therapeutic areas do not necessarily reflect the CDER review division.
† Representative biomarkers are listed based on standard nomenclature as per the Human Genome Organization (HUGO) symbol and/or simplified descriptors using other common conventions. Listed biomarkers do not necessarily reflect the terminology used in labeling. The term "Nonspecific" is provided when labeling does not explicitly identify the specific biomarker(s) or when the biomarker is represented by a molecular phenotype or gene signature, and in some cases the biomarker was inferred based on the labeling language.
‡ Referenced figures and tables may be found in the labeling available at Drugs@FDA.
Blue text represents the most recent addtions and/or changes since last posted version.

NDA/ANDA/BLA Number, Label Version Date	Drug	Therapeutic Area*	Biomarker†	Labeling Sections	Labeling Text‡
				Populations, Clinical Studies	This indication is approved under accelerated approval based on tumor response rate and durability of response [see Clinical Studies (14.16)]. Continued approval for this indication may be contingent upon verification and description of clinical benefit in the confirmatory trials.<br>Limitations of Use: The safety and effectiveness of KEYTRUDA in pediatric patients with TMB-H central nervous system cancers have not been established.<br><br>**2 DOSAGE AND ADMINISTRATION**<br>**2.1 Patient Selection for NSCLC, HNSCC, Urothelial Carcinoma, Gastric Cancer, Esophageal Cancer, Cervical Cancer, MSI-H Cancer, or TMB-H Cancer**<br>(…) For the TMB-H indication, select patients for treatment with KEYTRUDA as a single agent based on TMB-H status in tumor specimens [see Clinical Studies (14.16)].<br>For the MSI-H/dMMR indication, select patients for treatment with KEYTRUDA as a single agent based on MSI-H/dMMR status in tumor specimens [see Clinical Studies (14.8)].<br>Because the effect of prior chemotherapy on test results for tumor mutation burden (TMB-H), MSI-H, or dMMR in patients with high-grade gliomas is unclear, it is recommended to test for these markers in the primary tumor specimens obtained prior to initiation of temozolomide chemotherapy in patients with high-grade gliomas.<br>Information on FDA-approved tests for the detection of PD-L1 expression and TMB status is available at: http://www.fda.gov/CompanionDiagnostics. An FDA-approved test for the detection of MSI-H or dMMR is not currently available. (See Table 2)<br>Because subclonal dMMR mutations and microsatellite instability may arise in high-grade gliomas during temozolomide therapy, it is recommended to test for TMB-H, MSI-H, and dMMR in the primary tumor specimens obtained prior to initiation of temozolomide chemotherapy in patients with high-grade gliomas.<br>Additional Patient Selection Information for MSI-H or dMMR in Patients with non-CRC Solid Tumors<br>Due to discordance between local tests and FDA-approved tests, confirmation of MSI-H or dMMR status is recommended by an FDA-approved test in patients with MSI-H or dMMR solid tumors, if feasible. If unable to perform confirmatory MSI-H/dMMR testing, the presence of TMB ≥10 mut/Mb, as determined by an FDA-approved test, may be used to select patients for treatment [see Clinical Studies (14.7)].<br><br>**8 USE IN SPECIFIC POPULATIONS**<br>8.4 Pediatric Use The safety and effectiveness of KEYTRUDA as a single agent have been established in pediatric patients with melanoma, cHL, PMBCL, MCC, MSI-H or dMMR cancer, and TMB-H cancer. Use of KEYTRUDA in pediatric patients for these indications is supported by evidence from adequate and well-controlled studies in adults with additional pharmacokinetic and safety data in pediatric patients [see Adverse Reactions (6.1), Clinical Pharmacology (12.3), Clinical Studies (14.1, 14.4, 14.5, 14.7, 14.14, 14.17)].<br><br>**14 CLINICAL STUDIES**<br>**14.17 Tumor Mutational Burden-High Cancer**<br>The efficacy of KEYTRUDA was investigated in a prospectively-planned retrospective analysis of 10 cohorts (A through J) of patients with various previously treated unresectable or metastatic solid tumors with high tumor mutation burden (TMB-H) who were enrolled in a multicenter, non-randomized, open-label trial, KEYNOTE-158 (NCT02628067). (…)<br>The statistical analysis plan pre-specified ≥10 and ≥13 mutations per megabase using the FoundationOne CDx assay as cutpoints to assess TMB. Testing of TMB was blinded with respect to clinical outcomes. The major efficacy outcome measures were ORR and DoR in patients who received at least one dose of KEYTRUDA as assessed by BICR according to RECIST v1.1, modified to follow a maximum of 10 target lesions and a maximum of 5 target lesions per organ.<br>In KEYNOTE-158, 1050 patients were included in the efficacy analysis population. TMB was analysed in the subset of 790 patients with sufficient tissue for testing based on protocol-specified testing requirements. Of the 790 patients, 102 (13%) had tumors identified as TMB-H, defined as TMB ≥10 mutations per megabase. Among the 102 patients with TMB-H advanced solid tumors, the study population characteristics were: median age of 61 years (range: 27 to 80), 34% age 65 or older; 34% male; 81% White; and 41% ECOG PS of 0 and 58% ECOG PS of 1. Fifty-six percent of patients had at least two prior lines of therapy. (See Tables 58 and 59)<br>In an exploratory analysis in 32 patients enrolled in KEYNOTE-158 whose cancer had TMB ≥10 mut/Mb and <13 mut/Mb, the ORR was 13% (95% CI: 4%, 29%), including two complete responses and two partial responses.
**125514, 06/17/2024**	**Pembrolizumab (7)**	Oncology	ERBB2 (HER2)	Indications and Usage, Adverse Reactions, Clinical Studies	**1 INDICATIONS AND USAGE**<br>**1.9 Gastric Cancer**<br>KEYTRUDA, in combination with trastuzumab, fluoropyrimidine- and platinum-containing chemotherapy, is indicated for the first-line treatment of adults with locally advanced unresectable or metastatic HER2-positive gastric or gastroesophageal junction (GEJ) adenocarcinoma whose tumors express PD-L1 (CPS ≥1) as determined by an FDA-approved test [see Dosage and Administration (2.1)].<br>This indication is approved under accelerated approval based on tumor response rate and durability of response [see Clinical Studies (14.9)]. Continued approval of this indication may be contingent upon verification and description of clinical benefit in the confirmatory trials.<br>KEYTRUDA, in combination with fluoropyrimidine- and platinum-containing chemotherapy, is indicated for the first-line treatment of adults with locally advanced unresectable or metastatic HER2-negative gastric or gastroesophageal junction (GEJ) adenocarcinoma.<br>Patient Selection for Combination Therapy<br>For use of KEYTRUDA in combination with chemotherapy and trastuzumab, select patients based on the presence of positive PD-L1 expression (CPS ≥1) in locally advanced unresectable or metastatic HER2-positive gastric or gastroesophageal junction (GEJ) adenocarcinoma [see Clinical Studies (14.9)].<br><br>**6 ADVERSE REACTIONS**<br>**6.1 Clinical Trials Experience**<br>Gastric Cancer

* Therapeutic areas do not necessarily reflect the CDER review division.
† Representative biomarkers are listed based on standard nomenclature as per the Human Genome Organization (HUGO) symbol and/or simplified descriptors using other common conventions. Listed biomarkers do not necessarily reflect the terminology used in labeling. The term “Nonspecific” is provided when labeling does not explicitly identify the specific biomarker(s) or when the biomarker is represented by a molecular phenotype or gene signature, and in some cases the biomarker was inferred based on the labeling language.
‡ Referenced figures and tables may be found in the labeling available at Drugs@FDA.
Blue text represents the most recent addtions and/or changes since last posted version.

NDA/ANDA/BLA Number, Label Version Date	Drug	Therapeutic Area*	Biomarker†	Labeling Sections	Labeling Text‡
					First-line Treatment of Locally Advanced Unresectable or Metastatic HER2-Positive Gastric or Gastroesophageal Junction Adenocarcinoma<br>The safety of KEYTRUDA was evaluated in 433 patients with HER2-positive gastric or GEJ cancer enrolled in KEYNOTE-811, which included 217 patients treated with KEYTRUDA 200 mg, trastuzumab, and CAPOX (n=189) or FP (n=28) every 3 weeks, compared to 216 patients treated with placebo, trastuzumab, and CAPOX (n=187) or FP (n=29) every 3 weeks [see Clinical Studies (14.9)]. (…)<br>*First-line Treatment of Locally Advanced Unresectable or Metastatic HER2-Negative Gastric or Gastroesophageal Junction Adenocarcinoma*<br>The safety of KEYTRUDA was evaluated in 1572 patients with HER2-negative gastric or GEJ cancer enrolled in KEYNOTE-859, which included 785 patients treated with KEYTRUDA 200 mg and FP (n=106) or CAPOX (n=674) every 3 weeks, compared to 787 patients who received placebo and FP (n=107) or CAPOX (n=679) every 3 weeks [see Clinical Studies (14.9)]. (…)<br><br>**14 CLINICAL STUDIES**<br>**14.9 Gastric Cancer**<br>First-line Treatment of Locally Advanced Unresectable or Metastatic HER2-Positive Gastric or Gastroesophageal Junction (GEJ) Adenocarcinoma<br>The efficacy of KEYTRUDA in combination with trastuzumab plus fluoropyrimidine and platinum chemotherapy was investigated in KEYNOTE-811 (NCT03615326), a multicenter, randomized, double-blind, placebo-controlled trial that was designed to enroll 692 patients with HER2-positive advanced gastric or gastroesophageal junction (GEJ) adenocarcinoma who had not previously received systemic therapy for metastatic disease. Patients with an autoimmune disease that required systemic therapy within 2 years of treatment or a medical condition that required immunosuppression were ineligible. Randomization was stratified by PD-L1 expression (CPS ≥1 or CPS <1), chemotherapy regimen (5-FU plus cisplatin [FP] or capecitabine plus oxaliplatin [CAPOX]), and geographic region (Europe/Israel/North America/Australia, Asia, or Rest of the World). (…)<br>First-line Treatment of Locally Unresectable or Metastatic HER2-Negative Gastric or Gastroesophageal Junction Adenocarcinoma<br>The efficacy of KEYTRUDA in combination with fluoropyrimidine- and platinum-containing chemotherapy was investigated in KEYNOTE-859 (NCT03675737), a multicenter, randomized, double-blind, placebo-controlled trial that enrolled 1579 patients with HER2-negative advanced gastric or GEJ adenocarcinoma who had not previously received systemic therapy for metastatic disease. (…)<br>**14.10 Esophageal Cancer**<br>Previously Treated Recurrent Locally Advanced or Metastatic Esophageal Cancer<br>*KEYNOTE-181*<br>The efficacy of KEYTRUDA was investigated in KEYNOTE-181 (NCT02564263), a multicenter, randomized, open-label, active-controlled trial that enrolled 628 patients with recurrent locally advanced or metastatic esophageal cancer who progressed on or after one prior line of systemic treatment for advanced disease. Patients with HER2/neu positive esophageal cancer were required to have received treatment with approved HER2/neu targeted therapy. All patients were required to have tumor specimens for PD-L1 testing at a central laboratory; PD-L1 status was determined using the PD-L1 IHC 22C3 pharmDx kit. Patients with a history of non-infectious pneumonitis that required steroids or current pneumonitis, active autoimmune disease, or a medical condition that required immunosuppression were ineligible. (…)
021462, 08/31/2022	Pemetrexed (1)	Onclology	ALK	Indications and Usage, Adverse Reactions, Clinical Studies	**1 INDICATIONS AND USAGE**<br>**1.1 Non-Squamous Non-Small Cell Lung Cancer (NSCLC)**<br>ALIMTA® is indicated:<br>• in combination with pembrolizumab and platinum chemotherapy, for the initial treatment of patients with metastatic non-squamous non-small cell lung cancer (NSCLC), with no EGFR or ALK genomic tumor aberrations. (…)<br>Limitations of Use: ALIMTA is not indicated for the treatment of patients with squamous cell, non-small cell lung cancer [see Clinical Studies (14.1)].<br><br>**6 ADVERSE REACTIONS**<br>Non-Squamous NSCLC<br>First-line Treatment of Metastatic Non-squamous NSCLC with Pembrolizumab and Platinum Chemotherapy The safety of ALIMTA, in combination with pembrolizumab and investigator's choice of platinum (either carboplatin or cisplatin), was investigated in Study KEYNOTE-189, a multicenter, double-blind, randomized (2:1), active-controlled trial in patients with previously untreated, metastatic non-squamous NSCLC with no EGFR or ALK genomic tumor aberrations. A total of 607 patients received ALIMTA, pembrolizumab, and platinum every 3 weeks for 4 cycles followed by ALIMTA and pembrolizumab (n=405), or placebo, ALIMTA, and platinum every 3 weeks for 4 cycles followed by placebo and ALIMTA (n=202). Patients with autoimmune disease that required systemic therapy within 2 years of treatment; a medical condition that required immunosuppression; or who had received more than 30 Gy of thoracic radiation within the prior 26 weeks were ineligible [see Clinical Studies (14.1)].<br><br>**14 CLINICAL STUDIES**<br>**14.1 Non-Squamous NSCLC**<br>Initial Treatment in Combination with Pembrolizumab and Platinum The efficacy of ALIMTA in combination with pembrolizumab and platinum chemotherapy was investigated in Study KEYNOTE-189 (NCT02578680), a randomized, multicenter, double-blind, active-controlled trial conducted in patients with metastatic non-squamous NSCLC, regardless of PD-L1 tumor expression status, who had not previously received systemic therapy for metastatic disease and in whom there were no EGFR or ALK genomic tumor aberrations. Patients with autoimmune disease that required systemic therapy within 2 years of treatment; a medical condition that required immunosuppression; or who had received more than 30 Gy of thoracic radiation within the prior 26 weeks were ineligible. Randomization was stratified by smoking status (never versus former/current), choice of platinum (cisplatin versus carboplatin), and tumor PD-L1 status (TPS <1% [negative] versus TPS ≥1%). Patients were randomized (2:1) to one of<br>the following treatment arms: (…)
021462, 08/31/2022	Pemetrexed (2)	Onclology	EGFR	Indications and Usage, Adverse	**1 INDICATIONS AND USAGE**<br>**1.1 Non-Squamous Non-Small Cell Lung Cancer (NSCLC)**

NDA/ANDA/BLA Number, Label Version Date	Drug	Therapeutic Area*	Biomarker†	Labeling Sections	Labeling Text‡
				Reactions, Clinical Studies	ALIMTA® is indicated:<br>• in combination with pembrolizumab and platinum chemotherapy, for the initial treatment of patients with metastatic non-squamous non-small cell lung cancer (NSCLC), with no EGFR or ALK genomic tumor aberrations. (…)<br>Limitations of Use: ALIMTA is not indicated for the treatment of patients with squamous cell, non-small cell lung cancer [see Clinical Studies (14.1)].<br><br>**6 ADVERSE REACTIONS**<br>Non-Squamous NSCLC<br>First-line Treatment of Metastatic Non-squamous NSCLC with Pembrolizumab and Platinum Chemotherapy The safety of ALIMTA, in combination with pembrolizumab and investigator's choice of platinum (either carboplatin or cisplatin), was investigated in Study KEYNOTE-189, a multicenter, double-blind, randomized (2:1), active-controlled trial in patients with previously untreated, metastatic non-squamous NSCLC with no EGFR or ALK genomic tumor aberrations. A total of 607 patients received ALIMTA, pembrolizumab, and platinum every 3 weeks for 4 cycles followed by ALIMTA and pembrolizumab (n=405), or placebo, ALIMTA, and platinum every 3 weeks for 4 cycles followed by placebo and ALIMTA (n=202). Patients with autoimmune disease that required systemic therapy within 2 years of treatment; a medical condition that required immunosuppression; or who had received more than 30 Gy of thoracic radiation within the prior 26 weeks were ineligible [see Clinical Studies (14.1)].<br><br>**14 CLINICAL STUDIES**<br>**14.1 Non-Squamous NSCLC**<br>Initial Treatment in Combination with Pembrolizumab and Platinum The efficacy of ALIMTA in combination with pembrolizumab and platinum chemotherapy was investigated in Study KEYNOTE-189 (NCT02578680), a randomized, multicenter, double-blind, active-controlled trial conducted in patients with metastatic non-squamous NSCLC, regardless of PD-L1 tumor expression status, who had not previously received systemic therapy for metastatic disease and in whom there were no EGFR or ALK genomic tumor aberrations. Patients with autoimmune disease that required systemic therapy within 2 years of treatment; a medical condition that required immunosuppression; or who had received more than 30 Gy of thoracic radiation within the prior 26 weeks were ineligible. Randomization was stratified by smoking status (never versus former/current), choice of platinum (cisplatin versus carboplatin), and tumor PD-L1 status (TPS <1% [negative] versus TPS ≥1%). Patients were randomized (2:1) to one of<br>the following treatment arms: (…)
021462, 08/31/2022	Pemetrexed (3)	Onclology	CD274 (PD-L1)	Clinical Studies	**14 CLINICAL STUDIES**<br>**14.1 Non-Squamous NSCLC**<br>Initial Treatment in Combination with Pembrolizumab and Platinum The efficacy of ALIMTA in combination with pembrolizumab and platinum chemotherapy was investigated in Study KEYNOTE-189 (NCT02578680), a randomized, multicenter, double-blind, active-controlled trial conducted in patients with metastatic non-squamous NSCLC, regardless of PD-L1 tumor expression status, who had not previously received systemic therapy for metastatic disease and in whom there were no EGFR or ALK genomic tumor aberrations. Patients with autoimmune disease that required systemic therapy within 2 years of treatment; a medical condition that required immunosuppression; or who had received more than 30 Gy of thoracic radiation within the prior 26 weeks were ineligible. Randomization was stratified by smoking status (never versus former/current), choice of platinum (cisplatin versus carboplatin), and tumor PD-L1 status (TPS <1% [negative] versus TPS ≥1%). Patients were randomized (2:1) to one of<br>the following treatment arms: (…)<br>A total of 616 patients were randomized: 410 patients to the ALIMTA, pembrolizumab, and platinum chemotherapy arm and 206 to the placebo, ALIMTA, and platinum chemotherapy arm. The study population characteristics were: median age of 64 years (range: 34 to 84); 49% age 65 or older; 59% male; 94% White and 3% Asian; 56% ECOG performance status of 1; and 18% with history of brain metastases. Thirty-one percent had tumor PD-L1 expression TPS<1%. Seventy-two percent received carboplatin and 12% were never smokers. A total of 85 patients in the placebo, ALIMTA, and chemotherapy arm received an anti-PD-1/PD-L1 monoclonal antibody at the time of disease progression. (see Table 10) (…)
213736, 08/26/2022	Pemigatinib (1)	Oncology	FGFR1	Indications and Usage, Dosage and Administration, Adverse Reactions, Use in Specific Populations, Clinical Studies	**1 INDICATIONS AND USAGE**<br>**1.2 Myeloid/Lymphoid Neoplasms with FGFR1 Rearrangement**<br>PEMAZYRE is indicated for the treatment of adults with relapsed or refractory myeloid/lymphoid neoplasms (MLNs) with fibroblast growth factor receptor 1 (FGFR1) rearrangement.<br><br>**2 DOSAGE AND ADMINISTRATION**<br>**2.1 Patient Selection**<br>Select patients for the treatment of relapsed or refractory myeloid/lymphoid neoplasms with FGFR1 rearrangement with PEMAZYRE based on the presence of an FGFR1 rearrangement [see Clinical Studies (14.2)]. An FDA-approved test for detection of FGFR1 rearrangement in patients with relapsed or refractory myeloid/lymphoid neoplasm for selecting patients for treatment with PEMAZYRE is not available.<br>**2.2 Recommended Dosage**<br>Myeloid/Lymphoid Neoplasms with FGFR1 Rearrangement<br>The recommended dosage of PEMAZYRE is 13.5 mg orally once daily on a continuous basis.<br>Continue treatment until disease progression or unacceptable toxicity occurs.<br>**2.3 Dosage Modification for Adverse Reactions**<br>The recommended dose reductions for adverse reactions are provided in Table 1.<br><br>**6 ADVERSE REACTIONS**<br>**6.1 Clinical Trials Experience**

NDA/ANDA/BLA Number, Label Version Date	Drug	Therapeutic Area*	Biomarker†	Labeling Sections	Labeling Text‡
					Because clinical trials are conducted under widely varying conditions, adverse reaction rates observed in the clinical trials of a drug cannot be directly compared to rates in the clinical trials of another drug and may not reflect the rates observed in practice.<br>The pooled safety population described in the WARNINGS AND PRECAUTIONS section reflects exposure to PEMAZYRE at a starting dose of 13.5 mg orally once daily (intermittent or continuous administration) in 635 patients with advanced malignancies. Among the 635 patients, 31% were exposed for 6 months or longer and 11% were exposed greater than one year, including patients with previously treated, advanced, or metastatic cholangiocarcinoma in FIGHT-202 and patients with MLNs with FGFR1 rearrangement in FIGHT-203.<br>Myeloid/Lymphoid Neoplasms with FGFR1 Rearrangement<br>*FIGHT-203*<br>The safety of PEMAZYRE was evaluated in FIGHT-203, which included 34 patients who were treated for MLN with FGFR1 rearrangement [see Clinical Studies (14.2)]. Patients were treated with PEMAZYRE 13.5 mg once daily on a continuous schedule (the approved recommended starting dosage) or for 14 days on followed by 7 days off therapy (an unapproved dosage regimen in MLN with FGFR1 rearrangement) until disease progression, unacceptable toxicity, or they were able to receive allogeneic stem cell transplant. The median duration of treatment was 205 days (range: 30-1347 days). (…)<br><br>**8 USE IN SPECIFIC POPULATIONS**<br>**8.5 Geriatric Use**<br>In FIGHT-202 in patients with cholangiocarcinoma, 32% of patients were 65 years and older, and 8% of patients were 75 years and older. In FIGHT-203 in patients with MLN with FGFR1 rearrangement, 44% of patients were 65 years and older, and 2.9% of patients were 75 years and older.<br>No overall differences in safety or effectiveness were observed between these patients and younger patients.<br><br>**14 CLINICAL STUDIES**<br>**14.2 Myeloid/Lymphoid Neoplasms with FGFR1 Rearrangement**<br>FIGHT-203 (NCT03011372), a multicenter open-label, single-arm trial, evaluated the efficacy of PEMAZYRE in 28 patients with MLNs with FGFR1 rearrangement. Inclusion criteria included documented myeloid/lymphoid neoplasms with 8p11 rearrangement shown to be an FGFR1 activating mutation, based on cytogenetic evaluation.<br>Patients could have relapsed after allogeneic hematopoietic stem cell transplantation (allo-HSCT) or after a disease modifying therapy, or were not a candidate for allo-HSCT or other disease modifying therapies. Patients received PEMAYZRE 13.5 mg once daily in 21-day cycles, either on a continuous schedule (the approved recommended starting dosage) or as an intermittent schedule (14 days on, 7 days off, an unapproved dosage regimen in MLN with FGFR1 rearrangement). PEMAZYRE was administered until disease progression or unacceptable toxicity or until patients were able to receive allo-HSCT. The median age was 65 years (range: 39-78), 64% were female, 68% were White, 3.6% were Black or African American, 11% were Asian, 3.6% were American Indian/Alaska Native 3.6% were other race, and race was unknown or not collected for 11% of patients; 3.6% were Hispanic, 68% were not Hispanic, 11% were other ethnicity, and ethnicity was not reported in 18%, and 88% had an ECOG performance status of 0 or 1. (…)
213736, 08/26/2022	Pemigatinib (2)	Oncology	FGFR2	Indications and Usage, Dosage and Administration, Clinical Studies	**1 INDICATIONS AND USAGE**<br>**1.1 Cholangiocarcinoma**<br>PEMAZYRE is indicated for the treatment of adults with previously treated, unresectable locally advanced or metastatic cholangiocarcinoma with a fibroblast growth factor receptor 2 (FGFR2) fusion or other rearrangement as detected by an FDA-approved test [see Dosage and Administration (2.1)].<br>This indication is approved under accelerated approval based on overall response rate and duration of response [see Clinical Studies (14.1)]. Continued approval for this indication may be contingent upon verification and description of clinical benefit in a confirmatory trial(s).<br><br>**2 DOSAGE AND ADMINISTRATION**<br>**2.1 Patient Selection**<br>Select patients for the treatment of locally advanced or metastatic cholangiocarcinoma with PEMAZYRE based on the presence of an FGFR2 fusion or rearrangement as detected by an FDA-approved test [see Clinical Studies (14.1)].<br>Information on FDA-approved test(s) for the detection of an FGFR2 fusion or rearrangement in cholangiocarcinoma is available at http://www.fda.gov/CompanionDiagnostics.<br>**2.3 Dosage Modification for Adverse Reactions**<br>The recommended dose reductions for adverse reactions are provided in Table 1.<br><br>**14 CLINICAL STUDIES**<br>**14.1 Cholangiocarcinoma**<br>FIGHT-202 (NCT02924376), a multicenter open-label single-arm trial, evaluated the efficacy of PEMAZYRE in 107 patients with locally advanced unresectable or metastatic cholangiocarcinoma whose disease had progressed on or after at least 1 prior therapy and who had an FGFR2 gene fusion or non-fusion rearrangement, as determined by a clinical trial assay performed at a central laboratory. Qualifying in-frame fusions and other rearrangements were predicted to have a breakpoint within intron 17/exon 18 of the FGFR2 gene leaving the FGFR2 kinase domain intact. (…)<br>(…) The median age was 56 years (range: 26 to 77 years), 61% were female, 74% were White, and 95% had a baseline Eastern Cooperative Oncology Group (ECOG) performance status of 0 (42%) or 1 (53%). Ninety-eight percent of patients had intrahepatic cholangiocarcinoma. Eighty-six percent of patients had in-frame FGFR2 gene fusions and the most commonly identified FGFR2 fusion was FGFR2-BICC1 (34%). Fourteen percent of patients had other FGFR2 rearrangements that could not be confidently predicted to be in-frame fusions, including rearrangements without an identifiable partner gene. All patients had received at least 1 prior line of systemic therapy, 27% had 2 prior lines of therapy, and 12% had 3 or more prior lines of therapy. Ninety-six percent of patients had received prior platinum-based therapy including 76% with prior gemcitabine/cisplatin.

* Therapeutic areas do not necessarily reflect the CDER review division.
† Representative biomarkers are listed based on standard nomenclature as per the Human Genome Organization (HUGO) symbol and/or simplified descriptors using other common conventions. Listed biomarkers do not necessarily reflect the terminology used in labeling. The term “Nonspecific” is provided when labeling does not explicitly identify the specific biomarker(s) or when the biomarker is represented by a molecular phenotype or gene signature, and in some cases the biomarker was inferred based on the labeling language.
‡ Referenced figures and tables may be found in the labeling available at Drugs@FDA.
Blue text represents the most recent addtions and/or changes since last posted version.

NDA/ANDA/BLA Number, Label Version Date	Drug	Therapeutic Area*	Biomarker†	Labeling Sections	Labeling Text‡
**010775, 05/10/2002**	**Perphenazine**	Psychiatry	CYP2D6	Precautions, Clinical Pharmacology	**PRECAUTIONS**<br>*Drug Interactions*<br>Metabolism of a number of medications, including antipsychotics, antidepressants, b- blockers, and antiarrhythmics, occurs through the cytochrome P450 2D6 isoenzyme (debrisoquine hydroxylase). Approximately 10% of the Caucasian population has reduced activity of this enzyme, so-called “poor” metabolizers. Among other populations the prevalence is not known. Poor metabolizers demonstrate higher plasma concentrations of antipsychotic drugs at usual doses, which may correlate with emergence of side effects. In one study of 45 elderly patients suffering from dementia treated with perphenazine, the 5 patients who were prospectively identified as poor P450 2D6 metabolizers had reported significantly greater side effects during the first 10 days of treatment than the 40 extensive metabolizers, following which the groups tended to converge. Prospective phenotyping of elderly patients prior to antipsychotic treatment may identify those at risk for adverse events. (…)<br><br>**12 CLINICAL PHARMACOLOGY**<br>**12.3 Pharmacokinetics**<br>(…) The pharmacokinetics of perphenazine covary with the hydroxylation of debrisoquine which is mediated by cytochrome P450 2D6 (CYP 2D6) and thus is subject to genetic polymorphism- ie, 7%-10% of Caucasians and a low percentage of Asians have little or no activity and are called “poor metabolizers.” Poor metabolizers of CYP 2D6 will metabolize perphenazine more slowly and will experience higher concentrations compared with normal or “extensive” metabolizers. (…)
**125409, 01/16/2020**	**Pertuzumab (1)**	Oncology	ERBB2 (HER2)	Indications and Usage, Dosage and Administration, Warnings and Precautions, Adverse Reactions, Clinical Pharmacology, Clinical Studies	**1 INDICATIONS AND USAGE**<br>**1.1 Metastatic Breast Cancer (MBC)**<br>PERJETA is indicated for use in combination with trastuzumab and docetaxel for the treatment of patients with HER2-positive metastatic breast cancer who have not received prior anti-HER2 therapy or chemotherapy for metastatic disease.<br>**1.2 Early Breast Cancer (EBC)**<br>PERJETA is indicated for use in combination with trastuzumab and chemotherapy for<br>• the neoadjuvant treatment of patients with HER2-positive, locally advanced, inflammatory, or early stage breast cancer (either greater than 2 cm in diameter or node positive) as part of a complete treatment regimen for early breast cancer [see Dosage and Administration (2.2) and Clinical Studies (14.2)].<br>• the adjuvant treatment of patients with HER2-positive early breast cancer at high risk of recurrence [see Dosage and Administration (2.2) and Clinical Studies (14.3)].<br><br>**2 DOSAGE AND ADMINISTRATION**<br>**2.1 Patient Selection**<br>Select patients based on HER2 protein overexpression or HER2 gene amplification in tumor specimens [see Indications and Usage (1) and Clinical Studies (14)]. Assessment of HER2 protein overexpression and HER2 gene amplification should be performed using FDA-approved tests specific for breast cancer by laboratories with demonstrated proficiency. Information on the FDA-approved tests for the detection of HER2 protein overexpression and HER2 gene amplification is available at: http://www.fda.gov/CompanionDiagnostics.<br>Improper assay performance, including use of suboptimally fixed tissue, failure to utilize specified reagents, deviation from specific assay instructions, and failure to include appropriate controls for assay validation, can lead to unreliable results.<br><br>**5 WARNINGS AND PRECAUTIONS**<br>**5.1 Left Ventricular Dysfunction**<br>Decreases in LVEF have been reported with drugs that block HER2 activity, including PERJETA. Assess LVEF prior to initiation of PERJETA and at regular intervals during treatment to ensure that LVEF is within normal limits. If the LVEF declines and has not improved, or has declined further at the subsequent assessment, discontinuation of PERJETA and trastuzumab should be strongly considered [Dosage and Administration (2.3)]. (…)<br><br>**6 ADVERSE REACTIONS**<br>**6.1 Clinical Trials Experience**<br>*Metastatic Breast Cancer (MBC)*<br>(…) The adverse reactions described in Table 1 were identified in 804 patients with HER2-positive metastatic breast cancer treated in Study 1. (…)<br>*Adjuvant Treatment of Breast Cancer (APHINITY)*<br>The adverse reactions described in Table 6 were identified in 4769 patients with HER2-positive early breast cancer treated in APHINITY. (…)<br><br>**12 CLINICAL PHARMACOLOGY**<br>**12.6 Cardiac Electrophysiology**<br>The effect of pertuzumab with an initial dose of 840 mg followed by a maintenance dose of 420 mg every three weeks on QTc interval was evaluated in a subgroup of 20 patients with HER2-positive breast cancer in CLEOPATRA. No large changes in the mean QT interval (i.e., greater than 20 ms) from placebo based on Fridericia correction method were detected in the trial. A small increase in the mean QTc interval (i.e., less than 10 ms) cannot be excluded because of the limitations of the trial design.<br><br>**14 CLINICAL STUDIES**<br>**14.1 Metastatic Breast Cancer**<br>CLEOPATRA (NCT00567190) was a multicenter, double-blind, placebo-controlled trial of 808 patients with HER2- positive metastatic breast cancer. HER2 overexpression was defined as a score of 3+ IHC or FISH amplification ratio of 2.0 or greater as determined by a central laboratory. Patients were randomly

NDA/ANDA/BLA Number, Label Version Date	Drug	Therapeutic Area*	Biomarker†	Labeling Sections	Labeling Text‡
					allocated 1:1 to receive placebo plus trastuzumab and docetaxel or PERJETA plus trastuzumab and docetaxel. Randomization was stratified by prior treatment (prior or no prior adjuvant/neoadjuvant anti-HER2 therapy or chemotherapy) and geographic region (Europe, North America, South America, and Asia). Patients with prior adjuvant or neoadjuvant therapy were required to have a disease-free interval of greater than 12 months before trial enrollment. (…)<br>(…) Approximately half of the patients received prior adjuvant or neoadjuvant anti-HER2 therapy or chemotherapy (placebo 47%, PERJETA 46%). Among patients with hormone receptor positive tumors, 45% received prior adjuvant hormonal therapy and 11% received hormonal therapy for metastatic disease. Eleven percent of patients received prior adjuvant or neoadjuvant trastuzumab. (…)<br>**14.2 Neoadjuvant Treatment of Breast Cancer**<br>*NeoSphere*<br>NeoSphere (NCT00545688) was a multicenter, randomized trial conducted in 417 patients with operable, locally advanced, or inflammatory HER2-positive breast cancer (T2-4d) who were scheduled for neoadjuvant therapy. HER2 overexpression was defined as a score of 3+ IHC or FISH amplification ratio of 2.0 or greater as determined by a central laboratory. Patients were randomly allocated to receive 1 of 4 neoadjuvant regimens prior to surgery as follows: trastuzumab plus docetaxel, PERJETA plus trastuzumab and docetaxel, PERJETA plus trastuzumab, or PERJETA plus docetaxel. Randomization was stratified by breast cancer type (operable, locally advanced, or inflammatory) and estrogen receptor (ER) or progesterone receptor (PgR) positivity.<br>*TRYPHAENA*<br>An additional neoadjuvant study (TRYPHAENA, NCT00976989) was conducted in 225 patients with HER2-positive locally advanced, operable, or inflammatory (T2-4d) breast cancer designed primarily to assess cardiac safety in which all arms included PERJETA. HER2 overexpression was defined as a score of 3+ IHC or FISH amplification ratio of 2.0 or greater as determined by a central laboratory. (…)<br>*BERENICE*<br>A two-arm non-randomized study (BERENICE, NCT02132949) was conducted in 401 patients with HER2-positive locally advanced, inflammatory, or early-stage HER2-positive breast cancer. HER2 overexpression was defined as a score of 3+ IHC or ISH amplification ratio of 2.0 or greater as determined by a central laboratory. (…)<br>**14.3 Adjuvant Treatment of Breast Cancer**<br>APHINITY (NCT01358877) was a multicenter, randomized, double-blind, placebo-controlled study conducted in 4804 patients with HER2-positive early breast cancer who had their primary tumor excised prior to randomization. (…)
125409, 01/16/2020	Pertuzumab (2)	Oncology	ESR, PGR (Hormone Receptor)	Clinical Studies	**14 CLINICAL STUDIES**<br>**14.1 Metastatic Breast Cancer**<br>(…) Patient demographic and baseline characteristics were balanced between the treatment arms. The median age was 54 (range 22 to 89 years), 59% were White, 32% were Asian, and 4% were Black. All were women with the exception of 2 patients. Seventeen percent of patients were enrolled in North America, 14% in South America, 38% in Europe, and 31% in Asia. Tumor prognostic characteristics, including hormone receptor status (positive 48%, negative 50%), presence of visceral disease (78%) and non-visceral disease only (22%) were similar in the study arms. Approximately half of the patients received prior adjuvant or neoadjuvant anti-HER2 therapy or chemotherapy (placebo 47%, PERJETA 46%). Among patients with hormone receptor positive tumors, 45% received prior adjuvant hormonal therapy and 11% received hormonal therapy for metastatic disease. Eleven percent of patients received prior adjuvant or neoadjuvant trastuzumab. (…)<br>(…) Consistent results were observed across several patient subgroups including age (< 65 or 547 ≥ 65 years), race, geographic region, prior adjuvant/neoadjuvant anti-HER2 therapy or chemotherapy (yes or no), and prior adjuvant/neoadjuvant trastuzumab (yes or no). In the subgroup of patients with hormone receptor-negative disease (n=408), the hazard ratio was 0.55 (95% CI: 0.42, 0.72). In the subgroup of patients with hormone receptor-positive disease (n=388), the hazard ratio was 0.72 (95% CI: 0.55, 0.95). In the subgroup of patients with disease limited to non-visceral metastasis (n=178), the hazard ratio was 0.96 (95% CI: 0.61, 1.52). (…)<br>**14.2 Neoadjuvant Treatment of Breast Cancer**<br>*NeoSphere*<br>(…) Randomization was stratified by breast cancer type (operable, locally advanced, or inflammatory) and estrogen receptor (ER) or progesterone receptor (PgR) positivity. (…)<br>(…) Approximately half the patients in each treatment group had hormone receptor-positive disease (defined as ER-positive and/or PgR positive). (…)<br>(…) The pCR rates and magnitude of improvement with PERJETA were lower in the subgroup of patients with hormone receptor-positive tumors compared to patients with hormone receptor-negative tumors. (See Table 8) (…)<br>*TRYPHAENA*<br>(…) The pCR (ypT0/is ypN0) rates were 56.2% (95% CI: 44.1%, 67.8%), 54.7% (95% CI: 42.7%, 66.2%), and 63.6% (95% CI: 51.9%, 74.3%) for patients treated with PERJETA plus trastuzumab and FEC followed by PERJETA plus trastuzumab and docetaxel, PERJETA plus trastuzumab and docetaxel following FEC, or PERJETA plus TCH, respectively. The pCR rates were lower in the subgroups of patients with hormone receptor-positive tumors: 41.0% (95% CI: 25.6%, 57.9%), 45.7% (95% CI: 28.8%, 63.4%), and 47.5% (95% CI: 31.5%, 63.9%) than with hormone receptor-negative tumors: 73.5% (95% CI: 55.6%, 87.1%), 62.5% (95% CI: 45.8%, 77.3%), and 81.1% (95% CI: 64.8%, 92.0%), respectively. (…)<br>*BERENICE*<br>(…) The pCR (ypT0/is ypN0) rates were 61.8% (95% CI: 54.7, 68.6) and 60.7% (95% CI: 53.6, 67.5) for patients treated with ddAC followed by PERJETA plus trastuzumab and paclitaxel, or FEC followed by PERJETA plus trastuzumab and docetaxel, respectively. The pCR rates were lower in the subgroups of patients with hormone receptor-positive tumors: 51.6% (95% CI: 42.6, 60.5%) and 57.3% (95% CI: 48.1, 66.1%) than with hormone receptor-negative tumors: 81.5% (95% CI: 70.0, 90.1%) and 68.0% (95% CI: 56.2, 78.3%), respectively.<br>**14.3 Adjuvant Treatment of Breast Cancer**<br>APHINITY (NCT01358877) was a multicenter, randomized, double-blind, placebo-controlled study conducted in 4804 patients with HER2-positive early breast cancer who had their primary tumor excised prior to randomization. Patients were then randomized to receive PERJETA or placebo, in combination with

NDA/ANDA/BLA Number, Label Version Date	Drug	Therapeutic Area*	Biomarker†	Labeling Sections	Labeling Text‡
					adjuvant trastuzumab and chemotherapy. Randomization was stratified by the following factors: region, nodal status, protocol version, central hormone receptor status, and adjuvant chemotherapy regimen. (…)<br>(…) Demographics were generally balanced between the two treatment arms. The median age was 51 years (range 18-86), 13% of patients were 65 or older, and over 99% of patients were female. Sixty-three percent of patients had node-positive disease, 64% had hormone receptor-positive disease, and 71% were Caucasian. All patients had an ECOG performance status of 0 or 1. Seventy-eight percent received an anthracycline containing regimen. (See Tables 9 and 10)<br>(…)
**008762, 02/16/2021**	Phenytoin (1)	Neurology	CYP2C9	Warnings and Precautions, Use in Specific Populations, Clinical Pharmacology	**5 WARNINGS AND PRECAUTIONS**<br>**5.3 Serious Dermatologic Reactions**<br>DILANTIN can cause severe cutaneous adverse reactions (SCARs), which may be fatal. Reported reactions in phenytoin-treated patients have included toxic epidermal necrolysis (TEN), Stevens-Johnson syndrome (SJS), acute generalized exanthematous pustulosis (AGEP), and Drug Reaction with Eosinophilia and Systemic Symptoms (DRESS) [see Warnings and Precautions (5.4)]. The onset of symptoms is usually within 28 days, but can occur later. DILANTIN should be discontinued at the first sign of a rash, unless the rash is clearly not drug-related. If signs or symptoms suggest a severe cutaneous adverse reaction, use of this drug should not be resumed and alternative therapy should be considered. If a rash occurs, the patient should be evaluated for signs and symptoms of SCARs. Studies in patients of Chinese ancestry have found a strong association between the risk of developing SJS/TEN and the presence of HLA-B*1502, an inherited allelic variant of the HLA B gene, in patients using carbamazepine. Limited evidence suggests that HLA-B*1502 may be a risk factor for the development of SJS/TEN in patients of Asian ancestry taking other antiepileptic drugs associated with SJS/TEN, including phenytoin. In addition, retrospective, case-control, genome-wide association studies in patients of southeast Asian ancestry have also identified an increased risk of SCARs in carriers of the decreased function CYP2C9*3 variant, which has also been associated with decreased clearance of phenytoin. Consider avoiding DILANTIN as an alternative to carbamazepine in patients who are positive for HLA-B*1502 or in CYP2C9*3 carriers [see Use in Specific Populations (8.7) and Clinical Pharmacology (12.5)].<br>The use of HLA-B*1502 or CYP2C9 genotyping has important limitations and must never substitute for appropriate clinical vigilance and patient management. The role of other possible factors in the development of, and morbidity from, SJS/TEN, such as antiepileptic drug (AED) dose, compliance, concomitant medications, comorbidities, and the level of dermatologic monitoring have not been studied.<br><br>**8 USE IN SPECIFIC POPULATIONS**<br>**8.7 Use in Patients with Decreased CYP2C9 Function**<br>Patients who are intermediate or poor metabolizers of CYP2C9 substrates (e.g., *1/*3, *2/*2, *3/*3) may exhibit increased phenytoin serum concentrations compared to patients who are normal metabolizers (e.g., *1/*1). Thus, patients who are known to be intermediate or poor metabolizers may ultimately require lower doses of phenytoin to maintain similar steady-state concentrations compared to normal metabolizers. If early signs of dose-related central nervous system (CNS) toxicity develop, serum concentrations should be checked immediately [see Clinical Pharmacology (12.5)].<br><br>**12 CLINICAL PHARMACOLOGY**<br>**12.3 Pharmacokinetics**<br><u>Metabolism</u><br>Phenytoin is primarily metabolized by the hepatic cytochrome P450 enzyme CYP2C9 and to a lesser extent by CYP2C19. Because phenytoin is hydroxylated in the liver by an enzyme system which is saturable at high serum levels, small incremental doses may increase the half-life and produce very substantial increases in serum levels, when these are in the upper range. The steady-state level may be disproportionately increased, with resultant intoxication, from an increase in dosage of 10% or more.<br>In most patients maintained at a steady dosage, stable phenytoin serum levels are achieved. There may be wide interpatient variability in phenytoin serum levels with equivalent dosages. Patients with unusually low levels may be noncompliant or hypermetabolizers of phenytoin. Unusually high levels result from liver disease, variant CYP2C9 and CYP2C19 alleles, or drug interactions which result in metabolic interference. The patient with large variations in phenytoin serum levels, despite standard doses, presents a difficult clinical problem. Serum level determinations in such patients may be particularly helpful. As phenytoin is highly protein bound, free phenytoin levels may be altered in patients whose protein binding characteristics differ from normal.<br>**12.5 Pharmacogenomics**<br>CYP2C9 activity is decreased in individuals with genetic variants such as the CYP2C9*2 and CYP2C9*3 alleles. Carriers of variant alleles, resulting in intermediate (e.g., *1/*3, *2/*2) or poor metabolism (e.g., *2/*3, *3/*3) have decreased clearance of phenytoin. Other decreased or nonfunctional CYP2C9 alleles may also result in decreased clearance of phenytoin (e.g., *5, *6, *8, *11). The prevalence of the CYP2C9 poor metabolizer phenotype is approximately 2-3% in the White population, 0.5-4% in the Asian population, and <1% in the African American population. The CYP2C9 intermediate phenotype prevalence is approximately 35% in the White population, 24% in the African American population, and 15-36% in the Asian population [see Warnings and Precautions (5.3) and Use in Specific Populations (8.7)].
**008762, 02/16/2021**	Phenytoin (2)	Neurology	CYP2C19	Clinical Pharmacology	**12 CLINICAL PHARMACOLOGY**<br>**12.3 Pharmacokinetics**<br><u>Metabolism</u><br>Phenytoin is primarily metabolized by the hepatic cytochrome P450 enzyme CYP2C9 and to a lesser extent by CYP2C19. Because phenytoin is hydroxylated in the liver by an enzyme system which is saturable at high serum levels, small incremental doses may increase the half-life and produce very substantial increases in serum levels, when these are in the upper range. The steady-state level may be disproportionately increased, with resultant intoxication, from an increase in dosage of 10% or more.<br>In most patients maintained at a steady dosage, stable phenytoin serum levels are achieved. There may be wide interpatient variability in phenytoin serum levels with equivalent dosages. Patients with unusually low levels may be noncompliant or hypermetabolizers of phenytoin. Unusually high levels result from liver disease, variant CYP2C9 and CYP2C19 alleles, or drug interactions which result in metabolic interference. The patient with large variations in phenytoin serum

NDA/ANDA/BLA Number, Label Version Date	Drug	Therapeutic Area*	Biomarker†	Labeling Sections	Labeling Text‡
					levels, despite standard doses, presents a difficult clinical problem. Serum level determinations in such patients may be particularly helpful. As phenytoin is highly protein bound, free phenytoin levels may be altered in patients whose protein binding characteristics differ from normal.
**008762, 02/16/2021**	Phenytoin (3)	Neurology	HLA-B	Warnings and Precautions	**5 WARNINGS AND PRECAUTIONS**<br>**5.3 Serious Dermatologic Reactions**<br>DILANTIN can cause severe cutaneous adverse reactions (SCARs), which may be fatal. Reported reactions in phenytoin-treated patients have included toxic epidermal necrolysis (TEN), Stevens-Johnson syndrome (SJS), acute generalized exanthematous pustulosis (AGEP), and Drug Reaction with Eosinophilia and Systemic Symptoms (DRESS) [see Warnings and Precautions (5.4)]. The onset of symptoms is usually within 28 days, but can occur later. DILANTIN should be discontinued at the first sign of a rash, unless the rash is clearly not drug-related. If signs or symptoms suggest a severe cutaneous adverse reaction, use of this drug should not be resumed and alternative therapy should be considered. If a rash occurs, the patient should be evaluated for signs and symptoms of SCARs.<br>Studies in patients of Chinese ancestry have found a strong association between the risk of developing SJS/TEN and the presence of HLA-B*1502, an inherited allelic variant of the HLA B gene, in patients using carbamazepine. Limited evidence suggests that HLA-B*1502 may be a risk factor for the development of SJS/TEN in patients of Asian ancestry taking other antiepileptic drugs associated with SJS/TEN, including phenytoin. In addition, retrospective, case-control, genome-wide association studies in patients of southeast Asian ancestry have also identified an increased risk of SCARs in carriers of the decreased function CYP2C9*3 variant, which has also been associated with decreased clearance of phenytoin. Consider avoiding DILANTIN as an alternative to carbamazepine in patients who are positive for HLA-B*1502 or in CYP2C9*3 carriers [see Use in Specific Populations (8.7) and Clinical Pharmacology (12.5)].<br>The use of HLA-B*1502 or CYP2C9 genotyping has important limitations and must never substitute for appropriate clinical vigilance and patient management. The role of other possible factors in the development of, and morbidity from, SJS/TEN, such as antiepileptic drug (AED) dose, compliance, concomitant medications, comorbidities, and the level of dermatologic monitoring have not been studied.
**017473, 09/27/2011**	Pimozide	Psychiatry	CYP2D6	Dosage and Administration, Precautions	**DOSAGE AND ADMINISTRATION**<br>*Children*<br>Reliable dose response data for the effects of ORAP (pimozide) on tic manifestation in Tourette's Disorder patients below the age of twelve are not available. Treatment should be initiated at a dose of 0.05 mg/kg preferably taken once at bedtime. The dose may be increased every third day to a maximum of 0.2 mg/kg not to exceed 10 mg/day.<br>At doses above 0.05 mg/kg/day, CYP 2D6 genotyping should be performed. In poor CYP 2D6 metabolizers, ORAP doses should not exceed 0.05 mg/kg/day, and doses should not be increased earlier than 14 days (see Precautions – Pharmacogenomics).<br>*Adults*<br>In general, treatment with ORAP should be initiated with a dose of 1 to 2 mg a day in divided doses. The dose may be increased thereafter every other day. Most patients are maintained at less than 0.2 mg/kg/day, or 10 mg/day, whichever is less. Doses greater than 0.2 mg/kg/day or 10 mg/day are not recommended.<br>At doses above 4 mg/day, CYP 2D6 genotyping should be performed. In poor CYP 2D6 metabolizers, ORAP doses should not exceed 4 mg/day, and doses should not be increased earlier than 14 days (see Precautions – Pharmacogenomics).<br><br>**PRECAUTIONS**<br>*Pharmacogenomics*<br>Individuals with genetic variations resulting in poor CYP 2D6 metabolism (approximately 5 to 10% of the population) exhibit higher pimozide concentrations than extensive CYP 2D6 metabolizers. The concentrations observed in poor CYP 2D6 metabolizers are similar to those seen with strong CYP 2D6 inhibitors such as paroxetine. The time to achieve steady state pimozide concentrations is expected to be longer (approximately 2 weeks) in poor CYP 2D6 metabolizers because of the prolonged half-life. Alternative dosing strategies are recommended in patients who are genetically poor CYP 2D6 metabolizers (see Dosage and Administration).
**018147, 05/03/2019**	Piroxicam	Rheumatology	CYP2C9	Clinical Pharmacology	**12 CLINICAL PHARMACOLOGY**<br>**12.3 Pharmacokinetics**<br>*Metabolism*<br>(…) Higher systemic exposure of piroxicam has been noted in subjects with CYP2C9 polymorphisms compared to normal metabolizer type subjects [see Clinical Pharmacology (12.5)].<br>**12.5 Pharmacogenomics**<br>CYP2C9 activity is reduced in individuals with genetic polymorphisms, such as the CYP2C9*2 and CYP2C9*3 polymorphisms. Limited data from two published reports showed that subjects with heterozygous CYP2C9*1/*2 (n=9), heterozygous CYP2C9*1/*3 (n=9), and homozygous CYP2C9*3/*3 (n=1) genotypes showed 1.7-, 1.7-, and 5.3-fold higher piroxicam systemic levels, respectively, than the subjects with CYP2C9*1/*1 (n=17, normal metabolizer genotype) following administration of a single oral dose. The mean elimination half-life values of piroxicam for subjects with CYP2C9*1/*3 (n=9) and CYP2C9*3/*3 (n=1) genotypes were 1.7- and 8.8-fold higher than subjects with CYP2C9*1/*1 (n=17). It is estimated that the frequency of the homozygous*3/*3 genotype is 0% to 1% in the population at large; however, frequencies as high as 5.7% have been reported in certain ethnic groups.<br>*Poor Metabolizers of CYP2C9 Substrates*<br>In patients who are known or suspected to be poor CYP2C9 metabolizers based on genotype or previous history/experience with other CYP2C9 substrates (such as warfarin and phenytoin) consider dose reduction as they may have abnormally high plasma levels due to reduced metabolic clearance.
**216059, 12/01/2023**	Pirtobrutinib (1)	Oncology	BTK	Clinical Studies	**14 CLINICAL STUDIES**<br>**14.1 Mantle Cell Lymphoma**<br>The efficacy of JAYPIRCA in patients with MCL was evaluated in BRUIN [NCT03740529], an open-label, international, multicohort, single-arm study of JAYPIRCA as monotherapy. Efficacy was based on 120 patients with MCL treated with JAYPIRCA who were previously treated with a BTK inhibitor. JAYPIRCA was given orally at a dose of 200 mg once daily and was continued until disease progression or unacceptable toxicity. Patients with active central nervous

* Therapeutic areas do not necessarily reflect the CDER review division.
† Representative biomarkers are listed based on standard nomenclature as per the Human Genome Organization (HUGO) symbol and/or simplified descriptors using other common conventions. Listed biomarkers do not necessarily reflect the terminology used in labeling. The term "Nonspecific" is provided when labeling does not explicitly identify the specific biomarker(s) or when the biomarker is represented by a molecular phenotype or gene signature, and in some cases the biomarker was inferred based on the labeling language.
‡ Referenced figures and tables may be found in the labeling available at Drugs@FDA.
Blue text represents the most recent addtions and/or changes since last posted version.

NDA/ANDA/BLA Number, Label Version Date	Drug	Therapeutic Area*	Biomarker†	Labeling Sections	Labeling Text‡
					system lymphoma or allogeneic hematopoietic stem cell transplantation (HSCT) or CAR-T cell therapy within 60 days were excluded. The median age was 71 years (range: 46 to 88 years); 79% were male; 78% were White, 14% Asian, 1.7% Black or African American. Seventy-eight percent of patients had the classic/leukemic variant of MCL, 12% had pleomorphic MCL, and 11% had blastoid MCL. The simplified Mantle Cell Lymphoma International Prognostic Index (sMIPI) score was low in 15%, intermediate in 59%, and high in 26% of patients. (…)<br>**14.2 Chronic Lymphocytic Leukemia/Small Lymphocytic Lymphoma**<br>(…) The median age was 68 years (range: 41 to 88 years); 69% were male; 89% were White, 4.6% Black or African American, 1.9% Asian and 1.9% were Hispanic or Latino. Baseline ECOG performance status was 0 or 1 in 91% of patients and 48% of patients had Rai stage III or IV disease. Among those patients with central testing available, 42% (37 of 88 patients) had a C481 BTK mutation, 54% (43 of 79 patients) had 17p deletion and/or TP53 mutation, 93% (77 of 83 patients) had unmutated IGHV, and 22% (16 of 72 patients) had 11q deletion. Patients received a median number of 5 prior lines of therapy (range: 2 to 11). The most common prior BTK inhibitors received were ibrutinib (97%), acalabrutinib (9%), and zanubrutinib (0.9%). Seventy-seven percent of patients discontinued the last BTK inhibitor for refractory or progressive disease, 13% discontinued for toxicity, and 10% discontinued for other reasons.
**216059, 12/01/2023**	Pirtobrutinib (2)	Oncology	Chromosome 11q	Clinical Studies	**14 CLINICAL STUDIES**<br>**14.2 Chronic Lymphocytic Leukemia/Small Lymphocytic Lymphoma**<br>(…) The median age was 68 years (range: 41 to 88 years); 69% were male; 89% were White, 4.6% Black or African American, 1.9% Asian and 1.9% were Hispanic or Latino. Baseline ECOG performance status was 0 or 1 in 91% of patients and 48% of patients had Rai stage III or IV disease. Among those patients with central testing available, 42% (37 of 88 patients) had a C481 BTK mutation, 54% (43 of 79 patients) had 17p deletion and/or TP53 mutation, 93% (77 of 83 patients) had unmutated IGHV, and 22% (16 of 72 patients) had 11q deletion. Patients received a median number of 5 prior lines of therapy (range: 2 to 11). The most common prior BTK inhibitors received were ibrutinib (97%), acalabrutinib (9%), and zanubrutinib (0.9%). Seventy-seven percent of patients discontinued the last BTK inhibitor for refractory or progressive disease, 13% discontinued for toxicity, and 10% discontinued for other reasons.
**216059, 12/01/2023**	Pirtobrutinib (3)	Oncology	Chromosome 17p	Clinical Studies	**14 CLINICAL STUDIES**<br>**14.2 Chronic Lymphocytic Leukemia/Small Lymphocytic Lymphoma**<br>(…) The median age was 68 years (range: 41 to 88 years); 69% were male; 89% were White, 4.6% Black or African American, 1.9% Asian and 1.9% were Hispanic or Latino. Baseline ECOG performance status was 0 or 1 in 91% of patients and 48% of patients had Rai stage III or IV disease. Among those patients with central testing available, 42% (37 of 88 patients) had a C481 BTK mutation, 54% (43 of 79 patients) had 17p deletion and/or TP53 mutation, 93% (77 of 83 patients) had unmutated IGHV, and 22% (16 of 72 patients) had 11q deletion. Patients received a median number of 5 prior lines of therapy (range: 2 to 11). The most common prior BTK inhibitors received were ibrutinib (97%), acalabrutinib (9%), and zanubrutinib (0.9%). Seventy-seven percent of patients discontinued the last BTK inhibitor for refractory or progressive disease, 13% discontinued for toxicity, and 10% discontinued for other reasons.
**216059, 12/01/2023**	Pirtobrutinib (4)	Oncology	IGHV	Clinical Studies	**14 CLINICAL STUDIES**<br>**14.2 Chronic Lymphocytic Leukemia/Small Lymphocytic Lymphoma**<br>(…) The median age was 68 years (range: 41 to 88 years); 69% were male; 89% were White, 4.6% Black or African American, 1.9% Asian and 1.9% were Hispanic or Latino. Baseline ECOG performance status was 0 or 1 in 91% of patients and 48% of patients had Rai stage III or IV disease. Among those patients with central testing available, 42% (37 of 88 patients) had a C481 BTK mutation, 54% (43 of 79 patients) had 17p deletion and/or TP53 mutation, 93% (77 of 83 patients) had unmutated IGHV, and 22% (16 of 72 patients) had 11q deletion. Patients received a median number of 5 prior lines of therapy (range: 2 to 11). The most common prior BTK inhibitors received were ibrutinib (97%), acalabrutinib (9%), and zanubrutinib (0.9%). Seventy-seven percent of patients discontinued the last BTK inhibitor for refractory or progressive disease, 13% discontinued for toxicity, and 10% discontinued for other reasons.
**216059, 12/01/2023**	Pirtobrutinib (5)	Oncology	TP53	Clinical Studies	**14 CLINICAL STUDIES**<br>**14.2 Chronic Lymphocytic Leukemia/Small Lymphocytic Lymphoma**<br>(…) The median age was 68 years (range: 41 to 88 years); 69% were male; 89% were White, 4.6% Black or African American, 1.9% Asian and 1.9% were Hispanic or Latino. Baseline ECOG performance status was 0 or 1 in 91% of patients and 48% of patients had Rai stage III or IV disease. Among those patients with central testing available, 42% (37 of 88 patients) had a C481 BTK mutation, 54% (43 of 79 patients) had 17p deletion and/or TP53 mutation, 93% (77 of 83 patients) had unmutated IGHV, and 22% (16 of 72 patients) had 11q deletion. Patients received a median number of 5 prior lines of therapy (range: 2 to 11). The most common prior BTK inhibitors received were ibrutinib (97%), acalabrutinib (9%), and zanubrutinib (0.9%). Seventy-seven percent of patients discontinued the last BTK inhibitor for refractory or progressive disease, 13% discontinued for toxicity, and 10% discontinued for other reasons.
**022363, 03/14/2024**	**Pitavastatin**	Endocrinology	Nonspecific (Heterozygous Familial Hypercholesterolemia)	Indications and Usage, Adverse Reactions, Use in Specific Populations, Clinical Studies	**1 INDICATIONS AND USAGE**<br>Pitavastatin tablets are indicated as an adjunct to diet to reduce low-density lipoprotein cholesterol (LDL-C) in:<br>• Adults with primary hyperlipidemia.<br>• Adults and pediatric patients aged 8 years and older with heterozygous familial hypercholesterolemia (HeFH).<br><br>**6 ADVERSE REACTIONS**<br>Adverse Reactions in Pediatric Patients Aged 8 Years and Older with HeFH<br>In a 12-week, double-blind, placebo-controlled trial of pitavastatin tablets 1 mg, 2 mg, and 4 mg once daily in 82 pediatric patients 8 years to 16 years of age with HeFH and a 52-week open-label trial in 85 pediatric patients with HeFH, the safety profile was similar to that observed in the adult population.<br><br>**8 USE IN SPECIFIC POPULATIONS**<br>**8.4 Pediatric Use**<br>The safety and effectiveness of pitavastatin tablets as an adjunctive therapy to diet to reduce elevated LDL-C in pediatric patients aged 8 years and older with HeFH have been established. Use of pitavastatin tablets for this indication is supported by a 12-week, double-blind, placebo-controlled trial in 82 pediatric patients 8 to 16 years of age with HeFH [see Clinical Studies (14)] and a 52-week open-label trial in 85 pediatric patients with HeFH.<br>The safety and effectiveness of pitavastatin tablets have not been established in pediatric patients younger than 8 years of age with HeFH or in pediatric patients with other types of hyperlipidemia (other than HeFH).

* Therapeutic areas do not necessarily reflect the CDER review division.
† Representative biomarkers are listed based on standard nomenclature as per the Human Genome Organization (HUGO) symbol and/or simplified descriptors using other common conventions. Listed biomarkers do not necessarily reflect the terminology used in labeling. The term “Nonspecific” is provided when labeling does not explicitly identify the specific biomarker(s) or when the biomarker is represented by a molecular phenotype or gene signature, and in some cases the biomarker was inferred based on the labeling language.
‡ Referenced figures and tables may be found in the labeling available at Drugs@FDA.
Blue text represents the most recent addtions and/or changes since last posted version.

NDA/ANDA/BLA Number, Label Version Date	Drug	Therapeutic Area*	Biomarker†	Labeling Sections	Labeling Text‡
					**14 CLINICAL STUDIES**<br>**HeFH in Pediatric Patients**: In a double-blind, placebo-controlled, 12-week trial, 82 pediatric patients (36 boys and 46 girls), 8 to 16 years of age with genetically confirmed HeFH, fasting low-density lipoprotein cholesterol (LDL-C) ≥ 190 mg/dL or LDL-C ≥ 160 mg/dL with an additional cardiovascular risk factor (male gender, a family history of premature CV disease, presence of low HDL (< 45 mg/dL) or high TG (> 150 mg/dL), presence of high lipoprotein (a) (> 75 nmol/L), presence of type 2 diabetes mellitus or presence of hypertension) were randomized to pitavastatin tablets 1 mg, 2 mg, and 4 mg. Mean LDL-C at baseline was 235 mg/dL (range 160.5 mg/dL to 441 mg/dL). Approximately 39% of patients were Tanner Stage 1 at baseline.<br>Pitavastatin tablets significantly reduced plasma LDL-C, non-HDL-C, TC, and Apo-B compared to placebo. The reductions in LDL-C, Apo-B, TC, and non-HDL-C were dose dependent. There was no statistically significant improvement in HDL-C or TG at any pitavastatin tablets dose. See the lipid results in Table 10.
**211150, 08/14/2019**	**Pitolisant**	Psychiatry	CYP2D6	Dosage and Administration, Use in Specific Populations, Clinical Pharmacology	**2 DOSAGE AND ADMINISTRATION**<br>**2.5 Use in Patients Who Are Known CYP2D6 Poor Metabolizers (PMs)**<br>In patients known to be poor CYP2D6 metabolizers, initiate WAKIX at 8.9 mg once daily and titrate to a maximum dose of 17.8 mg once daily after 7 days [see Use in Specific Populations (8.8), Clinical Pharmacology (12.5)].<br><br>**8 USE IN SPECIFIC POPULATIONS**<br>**8.8 CYP2D6 Poor Metabolizers**<br>Dosage reduction is recommended in patients known to be poor CYP2D6 metabolizers because these patients have higher pitolisant concentrations than normal CYP2D6 metabolizers [see Dosage and Administration (2.5), Clinical Pharmacology (12.3, 12.5)].<br><br>**12 CLINICAL PHARMACOLOGY**<br>**12.3 Pharmacokinetics**<br>*CYP2D6 Poor Metabolizers*<br>The pharmacokinetics of pitolisant were evaluated in 3 subjects who were CYP2D6 poor metabolizers (PMs) and 5 subjects who were CYP2D6 extensive metabolizers (EMs). All subjects received WAKIX 17.8 mg daily for 7 days. Exposure of pitolisant in CYP2D6 PMs are summarized in Figure 3. (See Figure 3)<br>**12.5 Pharmacogenomics**<br>Approximately 3 to 10% of Caucasians and 2 to 7% of African Americans generally lack the capacity to metabolize CYP2D6 substrates and are classified as poor metabolizers. The AUC of pitolisant was approximately 2.4 times higher in CYP2D6 poor metabolizers than in normal metabolizers and is similar to the exposure of pitolisant when WAKIX is administered concomitantly with a CYP2D6 inhibitor [see Dosage and Administration (2.5), Drug Interactions (7.1)].<br>In CYP2D6 poor metabolizers, the Cmax of pitolisant is 153 (151 to 157) ng/mL and the AUC is 1920 (1854 to 2000) ng*hr/mL after steady state dosing with 35.6 mg once daily.
**210303, 02/10/2023**	**Plazomicin**	Infectious Diseases	MT-RNR1	Warnings and Precautions	**5 WARNINGS AND PRECAUTIONS**<br>**5.2 Ototoxicity**<br>Ototoxicity with use of ZEMDRI Ototoxicity, manifested as hearing loss, tinnitus, and/or vertigo, has been reported with ZEMDRI. Symptoms of aminoglycoside-associated ototoxicity may be irreversible and may not become evident until after completion of therapy.<br>Regarding the incidence of adverse reactions associated with cochlear or vestibular function, in Trial 1, there was one case of reversible hypoacusis (1/303;0.3%) in ZEMDRI-treated patients and one case of tinnitus (1/301;0.3%) in meropenem-treated patients [see Adverse Reactions (6.1)]. In Trial 2, one case each of irreversible tinnitus and reversible vertigo was reported in ZEMDRI-treated patients, and one case of an abnormal audiogram occurred in a levofloxacintreated patient [see Adverse Reactions (6.1)].<br>Aminoglycoside-associated ototoxicity has been observed primarily in patients with a family history of hearing loss (excluding age-related hearing loss), patients with renal impairment, and in patients receiving higher doses and/or for longer periods than recommended. In Trial 1 and Trial 2, patients with a history of hearing loss, with the exception of age-related hearing loss, were excluded. The benefit-risk of ZEMDRI therapy should be considered in these patients.<br>*Risk of Ototoxicity Due to Mitochondrial DNA Variants*<br>Cases of ototoxicity with aminoglycosides have been observed in patients with certain variants in the mitochondrially encoded 12S rRNA gene (MT-RNR1), particularly the m.1555A>G variant. Ototoxicity occurred in some patients even when their aminoglycoside serum levels were within the recommended range. Mitochondrial DNA variants are present in less than 1% of the general US population, and the proportion of the variant carriers who may develop ototoxicity as well as the severity of ototoxicity is unknown. In case of known maternal history of ototoxicity due to aminoglycoside use or a known mitochondrial DNA variant in the patient, consider alternative treatments other than aminoglycosides unless the increased risk of permanent hearing loss is outweighed by the severity of infection and lack of safe and effective alternative therapies.
**761121, 04/19/2023**	**Polatuzumab Vedotin-piiq (1)**	Oncology	BCL2	Clinical Studies	**14 CLINICAL STUDIES**<br>**14.1 Previously Untreated DLBCL, NOS or HGBL**<br>*GO39942 (POLARIX)*<br>(…) Of the 879 patients randomized (440 to POLIVY plus R-CHP, 439 to R-CHOP), the median age was 65 years (range 19 to 80 years), 54% were male, 54% were White, 19% were Asian, 1.8% were Black or African American, and 6% were Hispanic or Latino. In total, 38% had an IPI score of 2, 62% had an IPI score of 3–5, 89% had Stage 3 or 4 disease, and 44% had bulky disease. The majority of patients had DLBCL, NOS (84%; n = 740), 11% (n = 93) had HGBL with MYC and BCL2 and/or BCL6 rearrangements or HGBL, NOS, and 5% had other large B-cell lymphomas. (…)
**761121, 04/19/2023**	**Polatuzumab Vedotin-piiq (2)**	Oncology	BCL6	Clinical Studies	**14 CLINICAL STUDIES**<br>**14.1 Previously Untreated DLBCL, NOS or HGBL**<br>*GO39942 (POLARIX)*

* Therapeutic areas do not necessarily reflect the CDER review division.
† Representative biomarkers are listed based on standard nomenclature as per the Human Genome Organization (HUGO) symbol and/or simplified descriptors using other common conventions. Listed biomarkers do not necessarily reflect the terminology used in labeling. The term “Nonspecific” is provided when labeling does not explicitly identify the specific biomarker(s) or when the biomarker is represented by a molecular phenotype or gene signature, and in some cases the biomarker was inferred based on the labeling language.
‡ Referenced figures and tables may be found in the labeling available at Drugs@FDA.
Blue text represents the most recent addtions and/or changes since last posted version.

NDA/ANDA/BLA Number, Label Version Date	Drug	Therapeutic Area*	Biomarker†	Labeling Sections	Labeling Text‡
					(…) Of the 879 patients randomized (440 to POLIVY plus R-CHP, 439 to R-CHOP), the median age was 65 years (range 19 to 80 years), 54% were male, 54% were White, 19% were Asian, 1.8% were Black or African American, and 6% were Hispanic or Latino. In total, 38% had an IPI score of 2, 62% had an IPI score of 3–5, 89% had Stage 3 or 4 disease, and 44% had bulky disease. The majority of patients had DLBCL, NOS (84%; n = 740), 11% (n = 93) had HGBL with MYC and BCL2 and/or BCL6 rearrangements or HGBL, NOS, and 5% had other large B-cell lymphomas. (…)
**761121, 04/19/2023**	**Polatuzumab Vedotin-piiq (3)**	Oncology	MYC	Clinical Studies	**14 CLINICAL STUDIES**<br>**14.1 Previously Untreated DLBCL, NOS or HGBL**<br>*GO39942 (POLARIX)*<br>(…) Of the 879 patients randomized (440 to POLIVY plus R-CHP, 439 to R-CHOP), the median age was 65 years (range 19 to 80 years), 54% were male, 54% were White, 19% were Asian, 1.8% were Black or African American, and 6% were Hispanic or Latino. In total, 38% had an IPI score of 2, 62% had an IPI score of 3–5, 89% had Stage 3 or 4 disease, and 44% had bulky disease. The majority of patients had DLBCL, NOS (84%; n = 740), 11% (n = 93) had HGBL with MYC and BCL2 and/or BCL6 rearrangements or HGBL, NOS, and 5% had other large B-cell lymphomas. (…)
**203469, 01/10/2020**	**Ponatinib**	Oncology	BCR-ABL1 (Philadelphia chromosome)	Indications and Usage, Dosage and Administration, Warnings and Precautions, Adverse Reactions, Use in Specific Populations, Clinical Studies	**1 INDICATIONS AND USAGE**<br>ICLUSIG is indicated for the treatment of adult patients with:<br>Philadelphia Chromosome-Positive Acute Lymphoblastic Leukemia (Ph+ ALL)<br>• Newly diagnosed Ph+ ALL in combination with chemotherapy.<br>This indication is approved under accelerated approval based on minimal residual disease (MRD)-negative complete remission (CR) at the end of induction [see Clinical Studies (14)]. Continued approval for this indication may be contingent upon verification of clinical benefit in a confirmatory trial(s).<br>• As monotherapy in Ph+ ALL for whom no other kinase inhibitors are indicated or T315I-positive Ph+ ALL.<br>Chronic Myeloid Leukemia (CML)<br>• Chronic phase (CP) CML with resistance or intolerance to at least two prior kinase inhibitors.<br>• Accelerated phase (AP) or blast phase (BP) CML for whom no other kinase inhibitors are indicated.<br>• T315I-positive CML (chronic phase, accelerated phase, or blast phase).<br>Limitations of Use: ICLUSIG is not indicated and is not recommended for the treatment of patients with newly diagnosed CP-CML [see Warnings and Precautions (5.7)].<br><br>**2 DOSAGE AND ADMINISTRATION**<br>**2.1 Recommended Dosage**<br>Newly Diagnosed Ph+ ALL<br>The recommended starting dosage of ICLUSIG in combination with chemotherapy is 30 mg orally once daily with a reduction to 15 mg orally once daily upon achievement of MRD-negative (≤0.01% BCR::ABL1/ABL1) CR at the end of induction. Continue ICLUSIG in combination with chemotherapy for up to 20 cycles until loss of response or unacceptable toxicity [see Clinical Studies (14)].<br>For a description of dosing of agents administered in combination with ICLUSIG, [see Clinical Studies (14)].<br>Monotherapy for Ph+ ALL for Whom No Other Kinase Inhibitors Are Indicated or T315I-positive Ph+ ALL<br>The optimal dose of ICLUSIG has not been identified.<br>The recommended starting dosage of ICLUSIG is 45 mg orally once daily. Continue ICLUSIG until loss of response or unacceptable toxicity.<br>Consider discontinuing ICLUSIG if response has not occurred by 3 months.<br>CP-CML<br>The recommended starting dosage of ICLUSIG is 45 mg orally once daily with a reduction to 15 mg orally once daily upon achievement of ≤1% BCR::ABL1IS. Patients with loss of response can re-escalate the dose of ICLUSIG to a previously tolerated dosage of 30 mg or 45 mg orally once daily. Continue ICLUSIG until loss of response at the re-escalated dose or unacceptable toxicity.<br>Consider discontinuing ICLUSIG if hematologic response has not occurred by 3 months.<br>**2.4 Dosage for Patients with Hepatic Impairment**<br>For patients with CP-CML, AP-CML, BP-CML, and Ph+ ALL receiving monotherapy, reduce the starting dose of ICLUSIG from 45 mg orally once daily to 30 mg orally once daily in patients with pre-existing hepatic impairment (Child-Pugh A, B, or C).<br>For patients with newly diagnosed Ph+ ALL, no dosage adjustment is recommended when administering ICLUSIG to patients with mild hepatic impairment (Child-Pugh A). Closely monitor patients with moderate or severe hepatic impairment (Child-Pugh B or C) and modify the ICLUSIG dosage in the event of adverse reactions [see Dosage and Administration (2.2), Use in Specific Populations (8.6)].<br><br>**5 WARNINGS AND PRECAUTIONS**<br>**5.2 Venous Thromboembolic Events**<br>Serious or severe VTEs have occurred in patients who received ICLUSIG.<br>In PhALLCON, VTEs occurred in 12% of 163 patients, including serious or severe (Grade 3 or 4) in 3.1%. VTEs included deep vein thrombosis (6%), superficial vein thrombosis (2.5%), embolism (1.8%), pulmonary embolism and thrombosis (1.2% each), and jugular vein thrombosis and retinal vein occlusion (0.6% each). The median time to onset of the first VTE event was 2.5 months (range: 6 days to 1.8 years).<br>In OPTIC, of the 94 patients who received a starting dose of 45 mg, 1 patient experienced a VTE (Grade 1 retinal vein occlusion).<br>In PACE, VTEs occurred in 6% of 449 patients, including serious or severe (Grade 3 or 4) in 5.8%. VTEs included deep venous thrombosis (2.2%), pulmonary embolism (1.8%), superficial thrombophlebitis (0.7%), retinal vein occlusion (0.7%), and retinal vein thrombosis (0.4%) with vision loss. VTEs occurred in 10% of the 62 patients with BP-CML, 9% of the 32 patients with Ph+ ALL, 6% of the 270 patients with CP-CML, and 3.5% of the 85 patients with AP-CML.

* Therapeutic areas do not necessarily reflect the CDER review division.
† Representative biomarkers are listed based on standard nomenclature as per the Human Genome Organization (HUGO) symbol and/or simplified descriptors using other common conventions. Listed biomarkers do not necessarily reflect the terminology used in labeling. The term "Nonspecific" is provided when labeling does not explicitly identify the specific biomarker(s) or when the biomarker is represented by a molecular phenotype or gene signature, and in some cases the biomarker was inferred based on the labeling language.
‡ Referenced figures and tables may be found in the labeling available at Drugs@FDA.
Blue text represents the most recent addtions and/or changes since last posted version.

NDA/ANDA/BLA Number, Label Version Date	Drug	Therapeutic Area*	Biomarker†	Labeling Sections	Labeling Text‡
					Monitor for evidence of VTEs. Interrupt, then resume at the same or decreased dose or discontinue ICLUSIG based on recurrence/severity [see Dosage and Administration (2.2)].<br>**5.4 Hepatotoxicity**<br>Iclusig can cause hepatotoxicity, including liver failure and death. Fulminant hepatic failure leading to death occurred in an Iclusig-treated patient within one week of starting Iclusig. Two additional fatal cases of acute liver failure also occurred. The fatal cases occurred in patients with blast phase (BP) CML or Ph+ ALL. Severe (grade 3 or 4) hepatotoxicity occurred in all disease cohorts. (…)<br>**5.10 Hemorrhage**<br>Serious hemorrhage events including fatalities, occurred in 6% (28/449) of patients treated with Iclusig in the phase 2 trial, with 48 months follow-up. Hemorrhage occurred in 28% (124/449) of patients. The incidence of serious bleeding events was higher in patients with AP-CML, BP-CML, and Ph+ ALL. Gastrointestinal hemorrhage and subdural hematoma were the most commonly reported serious bleeding events occurring in 1% (4/449 and 4/449, respectively). Most hemorrhagic events, but not all, occurred in patients with grade 4 thrombocytopenia [see Warnings and Precautions (5.13)]. Interrupt Iclusig for serious or severe hemorrhage and evaluate [see Dosage and Administration (2.3)].<br>**5.13 Myelosuppression**<br>Myelosuppression was reported as an adverse reaction in 59% (266/449) of patients, and severe (grade 3 or 4) myelosuppression occurred in 50% (226/449) of patients treated with Iclusig. With 48 months of follow-up, the incidence of these events was greater in patients with AP-CML, BP-CML, and Ph+ ALL than in patients with CP-CML. (…)<br><br>**6 ADVERSE REACTIONS**<br>**6.1 Clinical Trial Experience**<br>*Previously Treated CML or Ph+ ALL*<br>The adverse reactions described in this section were identified in a single-arm, open-label, international, multicenter trial in 449 patients with CML or Ph+ ALL whose disease was considered to be resistant or intolerant to prior tyrosine kinase inhibitor (TKI) therapy including those with the BCR-ABL T315I mutation. (…)<br>(…) At the time of analysis (48 months of follow-up), 133 patients (30%) were ongoing (110 CP-CML; 20 AP-CML; 3 BPCML; 0 Ph+ ALL), and the median duration of treatment with Iclusig was 32.2 months in patients with CP-CML, 19.4 months in patients with AP-CML, 2.9 months in patients with BP-CML, and 2.7 months in patients with Ph+ ALL. (…)<br>(…) The rates of treatment-emergent adverse reactions resulting in discontinuation were 19% in CP-CML, 12% in AP-CML, 15% in BP-CML, and 9% in Ph+ ALL. The most common adverse reactions that led to treatment discontinuation was thrombocytopenia (4%). (See Table 5) (…)<br>*Laboratory Abnormalities*<br>(…) Myelosuppression was commonly reported in all patient populations. The frequency of grade 3 or 4 thrombocytopenia, neutropenia, and anemia was higher in patients with AP-CML, BP-CML, and Ph+ ALL than in patients with CP-CML. (See Table 7) (…)<br><br>**8 USE IN SPECIFIC POPULATIONS**<br>**8.5 Geriatric Use**<br>Of the 163 patients with Ph+ALL who received ICLUSIG in PhALLCON, 21% were 65 years and older and 7% were 75 years and older. Overall, no differences in efficacy of ICLUSIG were observed between patients 65 years of age or older compared to younger patients. AOEs occurred in 21% (7/34) of patients 65 years and older and 2.3% (3/129) of patients less than 65 years of age.<br>Of the 94 patients with CP-CML who received ICLUSIG at a starting dose of 45 mg in OPTIC, 17% were 65 years and older and 2.1% were 75 years and older. Patients aged 65 years and older had a lower ≤1% BCR::ABL1IS rate at 12 months (27%) as compared with patients less than 65 years of age (47%). AOEs occurred in 38% (6/16) of patients 65 years and older and 9% (7/78) of patients less than 65 years of age [see Warnings and Precautions (5.1)].<br>Of the 449 patients who received ICLUSIG in PACE, 35% were 65 years and older and 8% were 75 years and older. In patients with CP-CML, patients aged 65 years and older had a lower major cytogenetic response rate (40%) as compared with patients less than 65 years of age (65%). In patients with AP-CML, BP-CML, and Ph+ ALL, patients aged 65 years and older had a similar hematologic response rate (45%) as compared with patients less than 65 years of age (44%). AOEs occurred in 35% (54/155) of patients 65 years and older and in 21% (61/294) of patients less than 65 years of age [see Warnings and Precautions (5.1)].<br>Patients aged 65 years or older are more likely to experience adverse reactions including vascular occlusion, decreased platelet count, peripheral edema, increased lipase, dyspnea, asthenia, muscle spasms, and decreased appetite. In general, dose selection for an elderly patient should be cautious, reflecting the greater frequency of decreased hepatic, renal, or cardiac function, and of concomitant disease or other drug therapy.<br>**8.6 Hepatic Impairment**<br>Patients with hepatic impairment are more likely to experience adverse reactions compared to patients with normal hepatic function. For patients with CP-CML, AP-CML, BP-CML, and Ph+ ALL receiving monotherapy, reduce the starting dose of ICLUSIG for patients with pre-existing hepatic impairment (Child-Pugh A, B, or C). For patients with newly diagnosed Ph+ ALL, dosage adjustment is not recommended when administering ICLUSIG to patients with mild hepatic impairment (Child-Pugh A). Clinical data in patients with newly diagnosed Ph+ ALL with pre-existing moderate or severe hepatic impairment (Child-Pugh B or C) is not available and patients should be closely monitored for potential increased incidence of adverse reactions. Modify the ICLUSIG dosage in the event of adverse reactions [see Dosage and Administration (2.2, 2.4), Clinical Pharmacology (12.3)]. The safety of multiple doses, or doses higher than 30 mg, has not been studied in patients with hepatic impairment.<br><br>**14 CLINICAL STUDIES**<br>Newly Diagnosed Ph+ ALL<br>The efficacy of ICLUSIG in combination with chemotherapy was evaluated in PhALLCON (NCT03589326), a randomized, active-controlled, multicenter, open-label trial of 245 patients with newly diagnosed Ph+ ALL. Randomization was stratified by age at the time of induction therapy (18 to <45 years; ≥45 to <60

* Therapeutic areas do not necessarily reflect the CDER review division.
† Representative biomarkers are listed based on standard nomenclature as per the Human Genome Organization (HUGO) symbol and/or simplified descriptors using other common conventions. Listed biomarkers do not necessarily reflect the terminology used in labeling. The term “Nonspecific” is provided when labeling does not explicitly identify the specific biomarker(s) or when the biomarker is represented by a molecular phenotype or gene signature, and in some cases the biomarker was inferred based on the labeling language.
‡ Referenced figures and tables may be found in the labeling available at Drugs@FDA.
Blue text represents the most recent addtions and/or changes since last posted version.

Table of Pharmacogenomic Biomarkers in Drug Labeling
Last Updated: 06/2024

NDA/ANDA/BLA Number, Label Version Date	Drug	Therapeutic Area*	Biomarker†	Labeling Sections	Labeling Text‡
					years; and ≥60 years). Patients were randomized (2:1) to receive either ICLUSIG 30 mg orally once daily (n=164) or imatinib 600 mg orally once daily (n=81) in combination with chemotherapy (imatinib in combination with chemotherapy is an unapproved regimen in adult patients). The ICLUSIG dose was reduced to 15 mg once daily after completion of the induction phase and achievement of MRD-negative complete remission (CR). If a patient lost MRD negativity at any time after dose reduction to 15 mg, re-escalation to 30 mg once daily was allowed. Only patients who achieved CR or CR with incomplete hematologic recovery (CRi) with MRD-negativity at the end of induction could continue study treatment at the investigator's discretion.<br>Per protocol, patients were allowed to receive one cycle of optional prephase therapy excluding TKI prior to randomization to manage the acute disease during the screening period.<br>Patients were randomized to receive either ICLUSIG or imatinib in combination with 20 cycles of chemotherapy, followed by ICLUSIG or imatinib as single-agent therapy (ICLUSIG or imatinib as single-agent after chemotherapy for newly diagnosed Ph+ ALL is not an approved regimen). Each cycle lasted 28 days.<br>Chronic Phase (CP), Accelerated Phase (AP), Blast Phase (BP) CML and Philadelphia Chromosome-Positive Acute Lymphoblastic Leukemia (Ph+ ALL)<br>The efficacy of ICLUSIG was evaluated in PACE (NCT01207440), a single-arm, open-label, international, multicenter trial. Eligible patients had CML and Ph+ ALL whose disease was considered to be resistant or intolerant to a prior kinase inhibitor. Patients were assigned to one of six cohorts based on disease phase (CP-CML, AP-CML, or BP-CML/Ph+ ALL), resistance or intolerance (R/I) to prior kinase inhibitors, and the presence of the T315I mutation. T315I mutation testing was performed on peripheral blood by Sanger Sequencing of the p190 or p210 BCR::ABL region.<br>Resistance in CP-CML while on a prior kinase inhibitor was defined as failure to achieve either a complete hematologic response (by 3 months), a minor cytogenetic response (by 6 months), or a major cytogenetic response (by 12 months). Patients with CP-CML who experienced a loss of response or development of a kinase domain mutation in the absence of a complete cytogenetic response or progression to AP-CML or BP-CML at any time on a prior kinase inhibitor were also considered resistant.<br>Resistance in AP-CML, BP-CML, and Ph+ ALL was defined as failure to achieve either a major hematologic response (by 3 months in AP-CML, and by 1 month in BP-CML and Ph+ ALL), loss of major hematologic response (at any time), or development of a kinase domain mutation in the absence of a complete major hematologic response while on a prior kinase inhibitor. Intolerance was defined as the discontinuation of a prior kinase inhibitor due to toxicities despite optimal management in the absence of a complete cytogenetic response in patients with CP-CML or major hematologic response for patients with AP-CML, BP-CML, or Ph+ ALL.<br>Patients were administered a starting dose of ICLUSIG 45 mg orally once daily.<br>The major efficacy outcome measure for patients with CP-CML was major cytogenetic response (MCyR), which included complete and partial cytogenetic responses (CCyR and PCyR). The major efficacy outcome measure for patients with AP-CML, BP-CML, and Ph+ ALL was major hematologic response (MaHR), defined as either a complete hematologic response (CHR) or no evidence of leukemia (NEL).<br>The trial enrolled 449 patients, of which 444 were eligible for efficacy analysis: 267 patients with CP-CML (R/I Cohort: N=203, T315I: N=64), 83 patients with AP-CML, 62 patients with BP-CML, and 32 patients with Ph+ ALL. Five patients were not eligible for efficacy analysis due to lack of confirmation of T315I mutation status, and these patients had not received prior dasatinib or nilotinib.<br>At study completion, the median duration of follow-up for the trial (all cohorts) was 40.5 months (range: 0.1 months to 79.5 months). The median duration of treatment was 35 months for patients with CP-CML, 21.1 months for patients with AP-CML, 3.2 months for patients with BP-CML and 2.9 months for patients with Ph+ ALL. Baseline demographic characteristics are described in Table 15.<br>The median time to MaHR in patients with AP-CML, BP-CML, and Ph+ ALL was 0.8 months (range: 0.4 to 6.3 months), 1.0 month (range: 0.4 to 4 months), and 0.7 months (range: 0.4 to 6 months), respectively. The median duration of MaHR for patients with AP-CML, BP-CML, and Ph+ ALL was 14 months (range: 1.3 to 74.3 months), 6.5 months (range: 1.9 to 64.7 months), and 3.5 months (range: 1.9 to 13.7 months), respectively.
**214701, 12/01/2020**	Pralsetinib	Oncology	CCDC6-RET, KIF5B-RET, RET	Indications and Usage, Dosage and Administration, Adverse Reactions, Use in Specific Populations, Clinical Pharmacology, Clinical Studies	**1 INDICATIONS AND USAGE**<br>**1.1 Metastatic RET Fusion-Positive Non-Small Cell Lung Cancer**<br>GAVRETO is indicated for the treatment of adult patients with metastatic RET fusion-positive non-small cell lung cancer (NSCLC) as detected by an FDA approved test.<br>This indication is approved under accelerated approval based on overall response rate and duration of response [see Clinical Studies (14)]. Continued approval for this indication may be contingent upon verification and description of clinical benefit in confirmatory trial(s).<br>**1.2 RET-Mutant Medullary Thyroid Cancer**<br>GAVRETO is indicated for the treatment of adult and pediatric patients 12 years of age and older with advanced or metastatic RET-mutant medullary thyroid cancer (MTC) who require systemic therapy.<br>This indication is approved under accelerated approval based on overall response rate and duration of response [see Clinical Studies (14.2)]. Continued approval for this indication may be contingent upon verification and description of clinical benefit in confirmatory trial(s).<br>**1.3 RET Fusion-Positive Thyroid Cancer**<br>GAVRETO is indicated for the treatment of adult and pediatric patients 12 years of age and older with advanced or metastatic RET fusion-positive thyroid cancer who require systemic therapy and who are radioactive iodine-refractory (if radioactive iodine is appropriate).<br>This indication is approved under accelerated approval based on overall response rate and duration of response [see Clinical Studies (14.3)]. Continued approval for this indication may be contingent upon verification and description of clinical benefit in confirmatory trial(s).<br><br>**2 DOSAGE AND ADMINISTRATION**<br>**2.1 Patient Selection**<br>Select patients for treatment with GAVRETO based on the presence of a RET gene fusion (NSCLC or thyroid cancer) or RET gene mutation (MTC) [see Clinical Studies (14)].<br>Information on FDA-approved tests for RET gene fusion (NSCLC) is available at http://www.fda.gov/CompanionDiagnostics.<br>An FDA-approved test for the detection of RET gene fusion (thyroid cancer) and RET gene mutations is not currently available.

* Therapeutic areas do not necessarily reflect the CDER review division.
† Representative biomarkers are listed based on standard nomenclature as per the Human Genome Organization (HUGO) symbol and/or simplified descriptors using other common conventions. Listed biomarkers do not necessarily reflect the terminology used in labeling. The term "Nonspecific" is provided when labeling does not explicitly identify the specific biomarker(s) or when the biomarker is represented by a molecular phenotype or gene signature, and in some cases the biomarker was inferred based on the labeling language.
‡ Referenced figures and tables may be found in the labeling available at Drugs@FDA.
Blue text represents the most recent addtions and/or changes since last posted version.

NDA/ANDA/BLA Number, Label Version Date	Drug	Therapeutic Area*	Biomarker†	Labeling Sections	Labeling Text‡
					**6 ADVERSE REACTIONS**<br>**6.1 Clinical Trials Experience**<br>The pooled safety population in the WARNINGS AND PRECAUTIONS reflect exposure to GAVRETO as a single agent at 400 mg orally once daily in 438 patients with RET altered solid tumors in ARROW [see Clinical Studies (14)]. Among 438 patients who received GAVRETO, 47% were exposed for 6 months or longer and 23% were exposed for greater than one year.<br>RET Fusion-Positive Non-Small Cell Lung Cancer<br>The safety of GAVRETO was evaluated as a single agent at 400 mg orally once daily in 220 patients with metastatic rearranged during transfection (RET fusion-positive) non-small cell lung cancer (NSCLC) in ARROW [see Clinical Studies (14)]. (…)Table 4 summarizes the adverse reactions in RET Fusion-Positive NSCLC Patients in ARROW.<br>RET-altered Thyroid Cancer<br>The safety of GAVRETO was evaluated as a single agent at 400 mg orally once daily in 138 patients with RET-altered Thyroid Cancer in ARROW [see Clinical Studies (14.2, 14.3)]. Among the 138 patients who received GAVRETO, 68% were exposed for 6 months or longer, and 40% were exposed for greater than one year. (…) Table 6 summarizes the adverse reactions occurring in RET-altered Thyroid Cancer Patients in ARROW.<br>Table 7 summarizes the laboratory abnormalities occurring in RET-altered Thyroid Cancer Patients in ARROW.<br><br>**8 USE IN SPECIFIC POPULATIONS**<br>**8.4 Pediatric Use**<br>The safety and effectiveness of GAVRETO have been established in pediatric patients aged 12 years and older for RET-mutant MTC and RET-fusion thyroid cancer. Use of GAVRETO in this age group is supported by evidence from an adequate and well-controlled study of GAVRETO in adults with additional population pharmacokinetic data demonstrating that age and body weight had no clinically meaningful effect on the pharmacokinetics of pralsetinib, that the exposure of pralsetinib is expected to be similar between adults and pediatric patients aged 12 years and older, and that the course of RET-mutant MTC and RET-fusion thyroid cancer is sufficiently similar in adults and pediatric patients to allow extrapolation of data in adults to pediatric patients[see Adverse Reactions (6.1), Clinical Pharmacology (12.3), and Clinical Studies (14.2)].<br>The safety and effectiveness of GAVRETO have not been established in pediatric patients with RET fusion-positive NSCLC or in pediatric patients younger than 12 years old with RET-mutant MTC or RET-fusion thyroid cancer.<br><br>**12 CLINICAL PHARMACOLOGY**<br>**12.2 Pharmacodynamics**<br>Pralsetinib exposure-response relationships and the time course of pharmacodynamics response have not been fully characterized.<br>Cardiac Electrophysiology<br>The QT interval prolongation potential of GAVRETO was assessed in 34 patients with RET fusion-positive solid tumors administered at the recommended dosage. No large mean increase in QTc (> 20 ms) was detected in the study.<br><br>**14 CLINICAL STUDIES**<br>**14.1 Metastatic RET Fusion-Positive Non-Small Cell Lung Cancer**<br>The efficacy of GAVRETO was evaluated in patients with RET fusion-positive metastatic NSCLC in a multicenter, non-randomized, open-label, multi-cohort clinical trial (ARROW, NCT03037385). The study enrolled, in separate cohorts, patients with metastatic RET fusionpositive NSCLC who had progressed on platinum-based chemotherapy and treatment-naïve patients with metastatic NSCLC. Identification of a RET gene fusion was determined by local laboratories using next generation sequencing (NGS), fluorescence in situ hybridization (FISH), and other tests. Among the 114 patients in the efficacy population(s) described in this section, samples from 59% of patients were retrospectively tested with the Life Technologies Corporation Oncomine Dx Target Test (ODxTT). Patients with asymptomatic central nervous system (CNS) metastases, including patients with stable or decreasing steroid use within 2 weeks prior to study entry, were enrolled. Patients received GAVRETO 400mg orally once daily until disease progression or unacceptable toxicity.<br>Metastatic RET Fusion-Positive NSCLC Previously Treated with Platinum Chemotherapy<br>Efficacy was evaluated in 87 patients with RET fusion-positive NSCLC with measurable disease who were previously treated with platinum chemotherapy enrolled into a cohort of ARROW. The median age was 60 years (range: 28 to 85); 49% were female, 53% were White, 35% were Asian, 6% were Hispanic/Latino. ECOG performance status was 0-1 (94%) or 2 (6%), 99% of patients had metastatic disease, and 43% had either a history of or current CNS metastasis. Patients received a median of 2 prior systemic therapies (range 1–6); 45% had prior anti-PD1/PD-L1 therapy and 25% had prior kinase inhibitors. A total of 52% of the patients received prior radiation therapy. RET fusions were detected in 77% of patients using NGS (45% tumor samples; 26% blood or plasma samples, 6% unknown), 21% using FISH, and 2% using other methods. The most common RET fusion partners were KIF5B (75%) and CCDC6 (17%).<br>Efficacy results for RET fusion-positive NSCLC patients who received prior platinum-based chemotherapy are summarized in Table 8.<br>For the 39 patients who received an anti-PD-1 or anti-PD-L1 therapy, either sequentially or concurrently with platinum-based chemotherapy, an exploratory subgroup analysis of ORR was 59% (95% CI: 42, 74) and the median DOR was not reached (95% CI: 11.3, NE).<br>Among the 87 patients with RET-fusion positive NSCLC, 8 had measurable CNS metastases at baseline as assessed by BICR. No patients received radiation therapy (RT) to the brain within 2 months prior to study entry. Responses in intracranial lesions were observed in 4 of these 8 patients including 2 patients with a CNS complete response; 75% of responders had a DOR of ≥ 6 months.<br>Treatment-naïve RET Fusion-Positive NSCLC<br>Efficacy was evaluated in 27 patients with treatment-naïve RET fusion-positive NSCLC with measurable disease enrolled into ARROW. The median age was 65 years (range 30 to 87); 52% were female, 59% were White, 33% were Asian, and 4% were Hispanic or Latino. ECOG performance status was 0-1 for 96% of the patients and all patients (100%) had metastatic disease 37% had either history of or current CNS metastasis. RET fusions were detected in 67% of patients

* Therapeutic areas do not necessarily reflect the CDER review division.
† Representative biomarkers are listed based on standard nomenclature as per the Human Genome Organization (HUGO) symbol and/or simplified descriptors using other common conventions. Listed biomarkers do not necessarily reflect the terminology used in labeling. The term “Nonspecific” is provided when labeling does not explicitly identify the specific biomarker(s) or when the biomarker is represented by a molecular phenotype or gene signature, and in some cases the biomarker was inferred based on the labeling language.
‡ Referenced figures and tables may be found in the labeling available at Drugs@FDA.
Blue text represents the most recent addtions and/or changes since last posted version.

NDA/ANDA/BLA Number, Label Version Date	Drug	Therapeutic Area*	Biomarker†	Labeling Sections	Labeling Text‡
					using NGS (41% tumor samples; 22% blood or plasma; 4% unknown) and 33% using FISH. The most common RET fusion partners were KIF5B (70%) and CCDC6 (11%). Efficacy results for treatment-naïve RET fusion-positive NSCLC are summarized in Table 9.<br>**14.2 RET-Mutant Medullary Thyroid Cancer**<br>The efficacy of GAVRETO was evaluated in patients with RET-mutant MTC in a multicenter, open-label, multi-cohort clinical trial (ARROW; NCT03037385).<br>RET-Mutant MTC Previously Treated with Cabozantinib or Vandetanib<br>Efficacy was evaluated in 55 patients with RET-mutant metastatic MTC previously treated with cabozantinib or vandetanib (or both). The median age was 59 years (range: 25 to 83); 69% were male, 78% were White, 5% were Asian, 5% were Hispanic/Latino. ECOG performance status was 0-1 (95%) or 2 (5%), and 7% had a history of CNS metastases. Patients had received a median of 2 prior therapies (range 1-7). RET mutation status was detected in 73% using NGS [55% tumor sample, 18% plasma], 26% using PCR sequencing, and 2% other. The primary mutations in RET-mutant MTC previously treated with cabozantinib or vandetanib are described in Table 10.<br>Efficacy results for RET-mutant MTC are summarized in Table 11.<br>Cabozantinib and Vandetanib-naïve RET-mutant MTC<br>Efficacy was evaluated in 29 patients with RET-mutant advanced MTC who were cabozantinib and vandetanib treatment-naïve. The median age was 61 years (range: 19 to 81); 72% were male, 76% were White, 17% were Asian, 3.4% were Hispanic/Latino. ECOG performance status was 0-1 (100%), 97% had metastatic disease, and 14% had a history of CNS metastases. Twenty-eight percent (28%) had received up to 3 lines of prior systemic therapy (including 10% PD-1/PD-L1 inhibitors, 10% radioactive iodine, 3.4% kinase inhibitors). RET mutation status was detected in 90% using NGS [52% tumor sample, 35% plasma, 3.4% blood] and 10% using PCR sequencing. The primary mutations used to identify and enroll patients are described in Table 10. Efficacy results for cabozantinib and vandetanib-naïve RET-mutant MTC are summarized in Table 12.<br>**14.3 RET Fusion-Positive Thyroid Cancer**<br>The efficacy of GAVRETO was evaluated in RET fusion-positive metastatic thyroid cancer patients in a multicenter, open-label, multi-cohort clinical trial (ARROW, NCT03037385). All patients with RET fusion-positive thyroid cancer were required to have disease progression<br>following standard therapy, measurable disease by RECIST version 1.1, and have RET fusion status as detected by local testing (89% NGS tumor samples and 11% using FISH). The median age was 61 years (range: 46 to 74); 67% were male, 78% were White, 22% were Asian, 11% were Hispanic/Latino. All patients (100%) had papillary thyroid cancer. ECOG performance status was 0-1 (100%), all patients (100%) had metastatic disease, and 56% had a history of CNS metastases. Patients had received a median of 2 prior therapies (range 1-8). Prior systemic treatments included prior radioactive iodine (100%) and prior sorafenib and/or lenvatinib (56%). Efficacy results are summarized in Table 13.
**022307, 03/28/2019**	Prasugrel (1)	Cardiology	CYP2C19	Use in Specific Populations, Clinical Pharmacology, Clinical Studies	**8 USE IN SPECIFIC POPULATIONS**<br>**8.9 Metabolic Status**<br>In healthy subjects, patients with stable atherosclerosis, and patients with ACS receiving prasugrel, there was no relevant effect of genetic variation in CYP2B6, CYP2C9, CYP2C19, or CYP3A5 on the pharmacokinetics of prasugrel's active metabolite or its inhibition of platelet aggregation.<br><br>**12 CLINICAL PHARMACOLOGY**<br>**12.5 Pharmacogenomics**<br>There is no relevant effect of genetic variation in CYP2B6, CYP2C9, CYP2C19, or CYP3A5 on the pharmacokinetics of prasugrel's active metabolite or its inhibition of platelet aggregation.<br><br>**14 CLINICAL STUDIES**<br>(…) There is, however, an alternative explanation: both prasugrel and clopidogrel are pro-drugs that must be metabolized to their active moieties. Whereas the pharmacokinetics of prasugrel's active metabolite are not known to be affected by genetic variations in CYP2B6, CYP2C9, CYP2C19, or CYP3A5, the pharmacokinetics of clopidogrel's active metabolite are affected by CYP2C19 genotype, and approximately 30% of Caucasians are reduced-metabolizers. (…)
**022307, 03/28/2019**	Prasugrel (2)	Cardiology	CYP2C9	Use in Specific Populations, Clinical Pharmacology, Clinical Studies	**8 USE IN SPECIFIC POPULATIONS**<br>**8.9 Metabolic Status**<br>In healthy subjects, patients with stable atherosclerosis, and patients with ACS receiving prasugrel, there was no relevant effect of genetic variation in CYP2B6, CYP2C9, CYP2C19, or CYP3A5 on the pharmacokinetics of prasugrel's active metabolite or its inhibition of platelet aggregation.<br><br>**12 CLINICAL PHARMACOLOGY**<br>**12.5 Pharmacogenomics**<br>There is no relevant effect of genetic variation in CYP2B6, CYP2C9, CYP2C19, or CYP3A5 on the pharmacokinetics of prasugrel's active metabolite or its inhibition of platelet aggregation.<br><br>**14 CLINICAL STUDIES**<br>(…) There is, however, an alternative explanation: both prasugrel and clopidogrel are pro-drugs that must be metabolized to their active moieties. Whereas the pharmacokinetics of prasugrel's active metabolite are not known to be affected by genetic variations in CYP2B6, CYP2C9, CYP2C19, or CYP3A5, the pharmacokinetics of clopidogrel's active metabolite are affected by CYP2C19 genotype, and approximately 30% of Caucasians are reduced-metabolizers. (…)
**022307, 03/28/2019**	Prasugrel (3)	Cardiology	CYP3A5	Use in Specific Populations, Clinical Pharmacology, Clinical Studies	**8 USE IN SPECIFIC POPULATIONS**<br>**8.9 Metabolic Status**<br>In healthy subjects, patients with stable atherosclerosis, and patients with ACS receiving prasugrel, there was no relevant effect of genetic variation in CYP2B6, CYP2C9, CYP2C19, or CYP3A5 on the pharmacokinetics of prasugrel's active metabolite or its inhibition of platelet aggregation.

* Therapeutic areas do not necessarily reflect the CDER review division.
† Representative biomarkers are listed based on standard nomenclature as per the Human Genome Organization (HUGO) symbol and/or simplified descriptors using other common conventions. Listed biomarkers do not necessarily reflect the terminology used in labeling. The term "Nonspecific" is provided when labeling does not explicitly identify the specific biomarker(s) or when the biomarker is represented by a molecular phenotype or gene signature, and in some cases the biomarker was inferred based on the labeling language.
‡ Referenced figures and tables may be found in the labeling available at Drugs@FDA.
Blue text represents the most recent addtions and/or changes since last posted version.

Table of Pharmacogenomic Biomarkers in Drug Labeling
Last Updated: 06/2024

NDA/ANDA/BLA Number, Label Version Date	Drug	Therapeutic Area*	Biomarker†	Labeling Sections	Labeling Text‡
					**12 CLINICAL PHARMACOLOGY**<br>**12.5 Pharmacogenomics**<br>There is no relevant effect of genetic variation in CYP2B6, CYP2C9, CYP2C19, or CYP3A5 on the pharmacokinetics of prasugrel's active metabolite or its inhibition of platelet aggregation.<br><br>**14 CLINICAL STUDIES**<br>(…) There is, however, an alternative explanation: both prasugrel and clopidogrel are pro-drugs that must be metabolized to their active moieties. Whereas the pharmacokinetics of prasugrel's active metabolite are not known to be affected by genetic variations in CYP2B6, CYP2C9, CYP2C19, or CYP3A5, the pharmacokinetics of clopidogrel's active metabolite are affected by CYP2C19 genotype, and approximately 30% of Caucasians are reduced-metabolizers. (…)
**022307, 03/28/2019**	**Prasugrel (4)**	Cardiology	CYP2B6	Use in Specific Populations, Clinical Pharmacology, Clinical Studies	**8 USE IN SPECIFIC POPULATIONS**<br>**8.9 Metabolic Status**<br>In healthy subjects, patients with stable atherosclerosis, and patients with ACS receiving prasugrel, there was no relevant effect of genetic variation in CYP2B6, CYP2C9, CYP2C19, or CYP3A5 on the pharmacokinetics of prasugrel's active metabolite or its inhibition of platelet aggregation.<br><br>**12 CLINICAL PHARMACOLOGY**<br>**12.5 Pharmacogenomics**<br>There is no relevant effect of genetic variation in CYP2B6, CYP2C9, CYP2C19, or CYP3A5 on the pharmacokinetics of prasugrel's active metabolite or its inhibition of platelet aggregation.<br><br>**14 CLINICAL STUDIES**<br>(…) There is, however, an alternative explanation: both prasugrel and clopidogrel are pro-drugs that must be metabolized to their active moieties. Whereas the pharmacokinetics of prasugrel's active metabolite are not known to be affected by genetic variations in CYP2B6, CYP2C9, CYP2C19, or CYP3A5, the pharmacokinetics of clopidogrel's active metabolite are affected by CYP2C19 genotype, and approximately 30% of Caucasians are reduced-metabolizers. (…)
**008316, 06/22/2017**	**Primaquine (1)**	Infectious Diseases	G6PD	Contraindications, Warnings, Precautions, Adverse Reactions, Overdosage	**CONTRAINDICATIONS**<br>Severe glucose-6-phosphate dehydrogenase (G6PD) deficiency (see Warnings).<br><br>**WARNINGS**<br>*Hemolytic anemia and G6PD deficiency*<br>Due to the risk of hemolytic anemia in patients with G6PD deficiency, G6PD testing has to be performed before using primaquine. Due to the limitations of G6PD tests, physicians need to be aware of residual risk of hemolysis and adequate medical support and follow-up to manage hemolytic risk should be available. Primaquine should not be prescribed for patients with severe G6PD deficiency (see Contraindications).<br>In case of mild to moderate G6PD deficiency, a decision to prescribe primaquine must be based on an assessment of the risks and benefits of using primaquine. If primaquine administration is considered, baseline hematocrit and hemoglobin must be checked before treatment and close hematological monitoring (e.g. at day 3 and 8) is required. Adequate medical support to manage hemolytic risk should be available.<br>When the G6PD status is unknown and G6PD testing is not available, a decision to prescribe primaquine must be based on an assessment of the risks and benefits of using primaquine. Risk factors for G6PD deficiency or favism must be assessed. Baseline hematocrit and hemoglobin must be checked before treatment and close hematological monitoring (e.g. at day 3 and 8) is required. Adequate medical support to manage hemolytic risk should be available.<br>Discontinue the use of primaquine phosphate promptly if signs suggestive of hemolytic anemia occur (darkening of the urine, marked fall of hemoglobin or erythrocytic count).<br>Hemolytic reactions (moderate to severe) may occur in individuals with G6PD deficiency and in individuals with a family or personal history of favism. Areas of high prevalence of G6PD deficiency are Africa, Southern Europe, Mediterranean region, Middle East, South-East Asia, and Oceania. People from these regions have a greater tendency to develop hemolytic anemia (due to a congenital deficiency of erythrocytic G6PD) while receiving primaquine and related drugs.<br>*Usage in Pregnancy*<br>Safe usage of this preparation in pregnancy has not been established. Primaquine is contraindicated in pregnant women. Even if a pregnant woman is G6PD normal, the fetus may not be (see Contraindications).<br><br>**PRECAUTIONS**<br>*Blood Monitoring*<br>Since anemia, methemoglobinemia, and leukopenia have been observed following administration of large doses of primaquine, the adult dosage of 1 tablet (= 15 mg base) daily for fourteen days should not be exceeded. In G6PD normal patients it is also advisable to perform routine blood examinations (particularly blood cell counts and hemoglobin determinations) during therapy.<br><br>**ADVERSE REACTIONS**<br>*Hematologic*<br>Leukopenia, hemolytic anemia in G6PD deficient individuals, and methemoglobinemia in nicotinamide adenine dinucleotide (NADH) methemoglobin reductase deficient individuals.<br><br>**OVERDOSAGE**

* Therapeutic areas do not necessarily reflect the CDER review division.
† Representative biomarkers are listed based on standard nomenclature as per the Human Genome Organization (HUGO) symbol and/or simplified descriptors using other common conventions. Listed biomarkers do not necessarily reflect the terminology used in labeling. The term "Nonspecific" is provided when labeling does not explicitly identify the specific biomarker(s) or when the biomarker is represented by a molecular phenotype or gene signature, and in some cases the biomarker was inferred based on the labeling language.
‡ Referenced figures and tables may be found in the labeling available at Drugs@FDA.
Blue text represents the most recent addtions and/or changes since last posted version.

Table of Pharmacogenomic Biomarkers in Drug Labeling
Last Updated: 06/2024

NDA/ANDA/BLA Number, Label Version Date	Drug	Therapeutic Area*	Biomarker†	Labeling Sections	Labeling Text‡
					Symptoms of overdosage of primaquine phosphate include abdominal cramps, vomiting, burning epigastric distress, central nervous system and cardiovascular disturbances, including cardiac arrhythmia and QT interval prolongation, cyanosis, methemoglobinemia, moderate leukocytosis or leukopenia, and anemia. The most striking symptoms are granulocytopenia and acute hemolytic anemia in G6PD deficient patients. Acute hemolysis occurs, but patients recover completely if the dosage is discontinued.
**008316, 06/22/2017**	**Primaquine (2)**	Infectious Diseases	CYB5R	Precautions, Adverse Reactions	**PRECAUTIONS**<br>*Blood Monitoring*<br>(…) If primaquine phosphate is prescribed for an individual who has shown a previous idiosyncratic reaction to primaquine phosphate as manifested by hemolytic anemia, methemoglobinemia, or leukopenia; an individual with a family or personal history of hemolytic anemia or nicotinamide adenine dinucleotide (NADH) methemoglobin reductase deficiency, the person should be observed closely. In all patients, the drug should be discontinued immediately if marked darkening of the urine or sudden decrease in hemoglobin concentration or leukocyte count occurs.<br><br>**ADVERSE REACTIONS**<br>*Hematologic*<br>Leukopenia, hemolytic anemia in G6PD deficient individuals, and methemoglobinemia in nicotinamide adenine dinucleotide (NADH) methemoglobin reductase deficient individuals.
**007898**	**Probenecid**	Rheumatology	G6PD	Adverse Reactions	Labeling not electronically available on Drugs@FDA
**020545**	**Procainamide**	Cardiology	Nonspecific (NAT)	Adverse Reactions, Clinical Pharmacology	Labeling not electronically available on Drugs@FDA
**021416, 11/02/2018**	**Propafenone**	Cardiology	CYP2D6	Dosage and Administration, Warnings and Precautions, Drug Interactions, Clinical Pharmacology	**2 DOSAGE AND ADMINISTRATION**<br>(…) The combination of CYP3A4 inhibition and either CYP2D6 deficiency or CYP2D6 inhibition with the simultaneous administration of propafenone may significantly increase the concentration of propafenone and thereby increase the risk of proarrhythmia and other adverse events. Therefore, avoid simultaneous use of RYTHMOL SR with both a CYP2D6 inhibitor and a CYP3A4 inhibitor [see Warnings and Precautions (5.4) and Drug Interactions (7.1)].<br><br>**5 WARNINGS AND PRECAUTIONS**<br>**5.4 Drug Interactions: Simultaneous Use with Inhibitors of Cytochrome P450 Isoenzymes 2D6 and 3A4**<br>Propafenone is metabolized by CYP2D6, CYP3A4, and CYP1A2 isoenzymes. Approximately 6% of Caucasians in the U.S. population are naturally deficient in CYP2D6 activity and to a somewhat lesser extent in other demographic groups. Drugs that inhibit these CYP pathways (such as desipramine, paroxetine, ritonavir, sertraline for CYP2D6; ketoconazole, erythromycin, saquinavir, and grapefruit juice for CYP3A4; and amiodarone and tobacco smoke for CYP1A2) can be expected to cause increased plasma levels of propafenone.<br>Increased exposure to propafenone may lead to cardiac arrhythmias and exaggerated beta-adrenergic blocking activity. Because of its metabolism, the combination of CYP3A4 inhibition and either CYP2D6 deficiency or CYP2D6 inhibition in users of propafenone is potentially hazardous. Therefore, avoid simultaneous use of RYTHMOL SR with both a CYP2D6 inhibitor and a CYP3A4 inhibitor.<br><br>**7 DRUG INTERACTIONS**<br>**7.1 CYP2D6 and CYP3A4 Inhibitors**<br>Drugs that inhibit CYP2D6 (such as desipramine, paroxetine, ritonavir, sertraline) and CYP3A4 (such as ketoconazole, ritonavir, saquinavir, erythromycin, and grapefruit juice) can be expected to cause increased plasma levels of propafenone. The combination of CYP3A4 inhibition and either CYP2D6 deficiency or CYP2D6 inhibition with administration of propafenone may increase the risk of adverse reactions, including proarrhythmia. Therefore, simultaneous use of RYTHMOL SR with both a CYP2D6 inhibitor and a CYP3A4 inhibitor should be avoided [see Warnings and Precautions (5.4) and Dosage and Administration (2)].<br><br>**12 CLINICAL PHARMACOLOGY**<br>**12.3 Pharmacokinetic**<br>*Metabolism*<br>There are two genetically determined patterns of propafenone metabolism. In over 90% of patients, the drug is rapidly and extensively metabolized with an elimination half-life from 2-10 hours. These patients metabolize propafenone into two active metabolites: 5- hydroxypropafenone which is formed by CYP2D6 and N-depropylpropafenone (norpropafenone) which is formed by both CYP3A4 and CYP1A2. In less than 10% of patients, metabolism of propafenone is slower because the 5-hydroxy metabolite is not formed or is minimally formed. In these patients, the estimated propafenone elimination half-life ranges from 10 to 32 hours. Decreased ability to form the 5-hydroxy metabolite of propafenone is associated with a diminished ability to metabolize debrisoquine and a variety of other drugs such as encainide, metoprolol, and dextromethorphan whose metabolism is mediated by the CYP2D6 isozyme. In these patients, the N-depropylpropafenone metabolite occurs in quantities comparable to the levels occurring in extensive metabolizers.<br>As a consequence of the observed differences in metabolism, administration of RYTHMOL SR to slow and extensive metabolizers results in significant differences in plasma concentrations of propafenone, with slow metabolizers achieving concentrations about twice those of the extensive metabolizers at daily doses of 850 mg/day. At low doses the differences are greater, with slow metabolizers attaining concentrations about 3 to 4 times higher than extensive metabolizers. In extensive metabolizers, saturation of the hydroxylation pathway<br>(CYP2D6) results in greater-than-linear increases in plasma levels following administration of RYTHMOL SR capsules. In slow metabolizers, propafenone pharmacokinetics is linear. Because the difference decreases at high doses and is mitigated by the lack of the active 5-hydroxymetabolite in the slow

* Therapeutic areas do not necessarily reflect the CDER review division.
† Representative biomarkers are listed based on standard nomenclature as per the Human Genome Organization (HUGO) symbol and/or simplified descriptors using other common conventions. Listed biomarkers do not necessarily reflect the terminology used in labeling. The term “Nonspecific” is provided when labeling does not explicitly identify the specific biomarker(s) or when the biomarker is represented by a molecular phenotype or gene signature, and in some cases the biomarker was inferred based on the labeling language.
‡ Referenced figures and tables may be found in the labeling available at Drugs@FDA.
Blue text represents the most recent addtions and/or changes since last posted version.

Table of Pharmacogenomic Biomarkers in Drug Labeling
Last Updated: 06/2024

NDA/ANDA/BLA Number, Label Version Date	Drug	Therapeutic Area*	Biomarker†	Labeling Sections	Labeling Text‡
					metabolizers, and because steady-state conditions are achieved after 4 to 5 days of dosing in all patients, the recommended dosing regimen of RYTHMOL SR is the same for all patients. The larger inter-subject variability in blood levels require that the dose of the drug be titrated carefully in patients with close attention paid to clinical and ECG evidence of toxicity [see Dosage and Administration (2)].<br>*Inter-Subject Variability*<br>With propafenone, there is a considerable degree of inter- subject variability in pharmacokinetics which is due in large part to the first pass hepatic effect and non-linear pharmacokinetics in extensive metabolizers. A higher degree of inter-subject variability in pharmacokinetic parameters of propafenone was observed following both single and multiple dose administration of RYTHMOL SR capsules. Inter-subject variability appears to be substantially less in the poor metabolizer group than in the extensive metabolizer group, suggesting that a large portion of the variability is intrinsic to CYP2D6 polymorphism rather than to the formulation.
**021438, 11/19/2013**	**Propranolol**	Cardiology	CYP2D6	Clinical Pharmacology	**12 CLINICAL PHARMACOLOGY**<br>**12.3 Pharmacokinetics**<br>*Metabolism and Elimination*<br>In healthy subjects, no difference was observed between CYP2D6 extensive metabolizers (EMs) and poor metabolizers (PMs) with respect to oral clearance or elimination half-life. Partial clearance to 4-hydroxy propranolol was significantly higher and to naphthyloxylactic acid was significantly lower in EMs than PMs.
**073644, 07/17/2014**	**Protriptyline**	Psychiatry	CYP2D6	Precautions	**PRECAUTIONS**<br>*Drugs Metabolized by Cytochrome P450 2D6*<br>The biochemical activity of the drug metabolizing isozyme cytochrome P450 2D6 (debrisoquine hydroxylase) is reduced in a subset of the Caucasian population (about 7% to 10% of Caucasians are so called "poor metabolizers"); reliable estimates of the prevalence of reduced P450 2D6 isozyme activity among Asian, African, and other populations are not yet available. Poor metabolizers have higher than expected plasma concentrations of tricyclic antidepressants (TCAs) when given usual doses. Depending on the fraction of drug metabolized by P450 2D6, the increase in plasma concentration may be small or quite large (8 fold increase in plasma AUC of the TCA). (…)
**089338, 02/02/2010**	**Quinidine**	Cardiology	CYP2D6	Precautions	**PRECAUTIONS**<br>(…) Constitutional deficiency of cytochrome P450IID6 is found in less than 1% of Orientals, in about 2% of American blacks, and in about 8% of American whites. Testing with debrisoquine is sometimes used to distinguish the P450IID6-deficient "poor metabolizers" from the majority-phenotype "extensive metabolizers". When drugs whose metabolism is P450IID6-dependent are given to poor metabolizers, the serum levels achieved are higher, sometimes much higher, than the serum levels achieved when identical doses are given to extensive metabolizers. To obtain similar clinical benefit without toxicity, doses given to poor metabolizers may need to be greatly reduced. In the case of prodrugs whose actions are actually mediated by P450IID6-produced metabolites (for example, codeine and hydrocodone, whose analgesic and antitussive effects appear to be mediated by morphine and hydromorphone, respectively), it may not be possible to achieve the desired clinical benefits in poor metabolizers. Quinidine is not metabolized by cytochrome P450IID6, but therapeutic serum levels of quinidine inhibit the action of cytochrome P450IID6, effectively converting extensive metabolizers into poor metabolizers. Caution must be exercised whenever quinidine is prescribed together with drugs metabolized by cytochrome P450IID6. (…)
**021799, 06/19/2019**	**Quinine Sulfate (1)**	Infectious Diseases	G6PD	Warnings and Precautions	**5 WARNINGS AND PRECAUTIONS**<br>**5.3 Hemolytic Anemia**<br>Acute hemolytic anemia has been reported in patients receiving quinine for treatment of malaria, including patients with glucose-6-phosphate dehydrogenase (G6PD) deficiency. The cause for the acute hemolytic anemia in quinine-treated patients with malaria and its potential relationship with G6PD deficiency has not been determined. Closely monitor hemoglobin and hematocrit during quinine treatment. Quinine should be discontinued if patients develop acute hemolytic anemia.
**021799, 06/19/2019**	**Quinine Sulfate (2)**	Infectious Diseases	CYP2D6	Clinical Pharmacology	**12 CLINICAL PHARMACOLOGY**<br>**12.3 Pharmacokinetics**<br>*Effects of quinine on other drugs*<br>*Desipramine (CYP2D6 substrate)*<br>Quinine (750 mg/day for 2 days) decreased the metabolism of desipramine in patients who were extensive CYP2D6 metabolizers, but had no effect in patients who were poor CYP2D6 metabolizers. (…)
**216993, 07/20/2023**	**Quizartinib**	Oncology	FLT3	Indications and Usage, Dosage and Administration, Warnings and Precautions, Adverse Reactions, Clinical Studies	**1 INDICATIONS AND USAGE**<br>VANFLYTA is indicated in combination with standard cytarabine and anthracycline induction and cytarabine consolidation, and as maintenance monotherapy following consolidation chemotherapy, for the treatment of adult patients with newly diagnosed acute myeloid leukemia (AML) that is FLT3 internal tandem duplication (ITD)-positive as detected by an FDA-approved test [see Dosage and Administration (2.1) and Clinical Studies (14)].<br><br>**2 DOSAGE AND ADMINISTRATION**<br>**2.1 Patient Selection**<br>Select patients for the treatment of AML with VANFLYTA based on the presence of FLT3-ITD mutation positivity [see Clinical Studies (14)]. Information on FDA-approved tests for the detection of FLT3-ITD mutation in AML is available at: http://www.fda.gov/CompanionDiagnostics.<br><br>**5 WARNINGS AND PRECAUTIONS**<br>**5.1 QT Prolongation, Torsades de Pointes, and Cardiac Arrest**<br>**(…)** Of the 265 patients with newly diagnosed FLT3-ITD-positive AML treated with VANFLYTA in combination with chemotherapy in the clinical trial, 2.3% were found to have a QTcF greater than 500 ms and 10% of patients had an increase from baseline QTcF greater than 60 ms. (…)

* Therapeutic areas do not necessarily reflect the CDER review division.
† Representative biomarkers are listed based on standard nomenclature as per the Human Genome Organization (HUGO) symbol and/or simplified descriptors using other common conventions. Listed biomarkers do not necessarily reflect the terminology used in labeling. The term "Nonspecific" is provided when labeling does not explicitly identify the specific biomarker(s) or when the biomarker is represented by a molecular phenotype or gene signature, and in some cases the biomarker was inferred based on the labeling language.
‡ Referenced figures and tables may be found in the labeling available at Drugs@FDA.
Blue text represents the most recent addtions and/or changes since last posted version.

NDA/ANDA/BLA Number, Label Version Date	Drug	Therapeutic Area*	Biomarker†	Labeling Sections	Labeling Text‡
					**6 ADVERSE REACTIONS**<br>Newly Diagnosed FLT3-ITD positive AML<br>The safety of VANFLYTA (35.4 mg orally once daily with chemotherapy, 26.5 mg to 53 mg orally once daily as maintenance) in adult patients with newly diagnosed FLT3-ITD positive AML is based on QuANTUM-First, a randomized, double-blind clinical trial of VANFLYTA (n=265) or placebo (n=268) with chemotherapy [see Clinical Studies (14)]. (See Tables 5 and 6) (…)<br>Other Clinical Trials<br>Clinically relevant adverse reactions in <10% of patients who received quizartinib for relapsed or refractory FLT3-ITD positive AML, an indication for which VANFLYTA is not approved, included differentiation syndrome (5%) and acute febrile neutrophilic dermatosis (3%).<br><br>**14 CLINICAL STUDIES**<br>The efficacy of VANFLYTA in combination with chemotherapy was evaluated in QuANTUM-First (NCT02668653), a randomized, double-blind, placebo-controlled study of 539 patients with newly diagnosed FLT3-ITD positive AML. FLT3-ITD status was determined prospectively with a clinical trial assay and verified retrospectively using the companion diagnostic LeukoStrat® CDx FLT3 Mutation Assay, which is an FDA-approved test for selection of patients with AML for VANFLYTA treatment.<br>(…)<br>The two treatment groups were generally balanced with respect to baseline demographics and disease characteristics. Of the 539 randomized patients, the median age was 56 years (range 20-75 years); 46% were male; 60% were White, 29% were Asian, 1% were Black or African American, and 10% were other races. Eighty-four percent had an Eastern Cooperative Oncology Group (ECOG) baseline performance status of 0 or 1. The majority of the patients (72%) had intermediate risk cytogenetics at baseline. FLT3-ITD variant allelic frequency (VAF) was 3-25% in 36% of patients, >25-50% in 52% of patients, and >50% in 12% of patients. NPM1 mutations were identified in 52% of patients.
**020973, 06/07/2018**	Rabeprazole	Gastroenterology	CYP2C19	Drug Interactions, Clinical Pharmacology	**7 DRUG INTERACTIONS**<br>*Tacrolimus*<br>Potential for increased exposure of tacrolimus, especially in transplant patients who are intermediate or poor metabolizers of CYP2C19. (See Table 3)<br><br>**12 CLINICAL PHARMACOLOGY**<br>**12.3 Pharmacokinetics**<br>*Metabolism*<br>(…) CYP2C19 exhibits a known genetic polymorphism due to its deficiency in some sub-populations (e.g., 3 to 5% of Caucasians and 17 to 20% of Asians). Rabeprazole metabolism is slow in these sub-populations, therefore, they are referred to as poor metabolizers of the drug.<br>*Drug Interaction Studies*<br>*Combined Administration with Antimicrobials*<br>Sixteen healthy subjects genotyped as extensive metabolizers with respect to CYP2C19 were given 20 mg ACIPHEX delayedrelease tablets, 1000 mg amoxicillin, 500 mg clarithromycin, or all 3 drugs in a four-way crossover study. (…)<br>*Clopidogrel*<br>Clopidogrel is metabolized to its active metabolite in part by CYP2C19. A study of healthy subjects including CYP2C19 extensive and intermediate metabolizers receiving once daily administration of clopidogrel 75 mg concomitantly with placebo or with 20 mg ACIPHEX delayed-release tablets (n=36), for 7 days was conducted. The mean AUC of the active metabolite of clopidogrel was reduced by approximately 12% (mean AUC ratio was 88 %, with 90% CI of 81.7 to 95.5%) when ACIPHEX delayed-release tablets were coadministered compared to administration of clopidogrel with placebo [see Drug Interactions (7)].<br>**12.5 Pharmacogenomics**<br>In a clinical study in evaluating ACIPHEX delayed-release tablets in Japanese adult patients categorized by CYP2C19 genotype (n=6 per genotype category), gastric acid suppression was higher in poor metabolizers as compared to extensive metabolizers. This could be due to higher rabeprazole plasma levels in poor metabolizers. The clinical relevance of this is not known. Whether or not interactions of rabeprazole sodium with other drugs metabolized by CYP2C19 would be different between extensive metabolizers and poor metabolizers has not been studied.
**020815, 06/27/2018**	Raloxifene	Oncology	ESR (Hormone Receptor)	Clinical Studies	**14 CLINICAL STUDIES**<br>**14.3 Reduction in Risk of Invasive Breast Cancer in Postmenopausal Women with Osteoporosis**<br>MORE Trial<br>The effect of EVISTA on the incidence of breast cancer was assessed as a secondary safety endpoint in a randomized, placebo-controlled, double-blind, multinational osteoporosis treatment trial in postmenopausal women [see Clinical Studies (14.1)]. After 4 years, EVISTA, 60 mg administered once daily, reduced the incidence of all breast cancers by 62%, compared with placebo (HR 0.38, 95% CI 0.22-0.67). EVISTA reduced the incidence of invasive breast cancer by 71%, compared with placebo (ARR 3.1 per 1000 women-years); this was primarily due to an 80% reduction in the incidence of ER-positive invasive breast cancer in the EVISTA group compared with placebo. (See Table 7)<br>CORE Trial<br>The effect of EVISTA on the incidence of invasive breast cancer was evaluated for 4 additional years in a follow-up study conducted in a subset of postmenopausal women originally enrolled in the MORE osteoporosis treatment trial. Women were not re-randomized; the treatment assignment from the osteoporosis treatment trial was carried forward to this study. EVISTA, 60 mg administered once daily, reduced the incidence of invasive breast cancer by 56%, compared with placebo (ARR 3.0 per 1000 women-years); this was primarily due to a 63% reduction in the incidence of ER-positive invasive breast cancer in the EVISTA group compared with placebo. There was no reduction in the incidence of ERnegative breast cancer. In the osteoporosis treatment trial and the follow-up study, there was no difference in incidence of noninvasive breast cancer between the EVISTA and placebo groups.

* Therapeutic areas do not necessarily reflect the CDER review division.
† Representative biomarkers are listed based on standard nomenclature as per the Human Genome Organization (HUGO) symbol and/or simplified descriptors using other common conventions. Listed biomarkers do not necessarily reflect the terminology used in labeling. The term “Nonspecific” is provided when labeling does not explicitly identify the specific biomarker(s) or when the biomarker is represented by a molecular phenotype or gene signature, and in some cases the biomarker was inferred based on the labeling language.
‡ Referenced figures and tables may be found in the labeling available at Drugs@FDA.
Blue text represents the most recent addtions and/or changes since last posted version.

Table of Pharmacogenomic Biomarkers in Drug Labeling
Last Updated: 06/2024

NDA/ANDA/BLA Number, Label Version Date	Drug	Therapeutic Area*	Biomarker†	Labeling Sections	Labeling Text‡
					In a subset of postmenopausal women followed for up to 8 years from randomization in MORE to the end of CORE, EVISTA, 60 mg administered once daily, reduced the incidence of invasive breast cancer by 60% in women assigned EVISTA (N=1355) compared with placebo (N=1286) (HR 0.40, 95% CI 0.21, 0.77; ARR 1.95 per 1000 womenyears); this was primarily due to a 65% reduction in the incidence of ER-positive invasive breast cancer in the EVISTA group compared with placebo. (See Table 7)<br>RUTH Trial<br>EVISTA, 60 mg administered once daily, reduced the incidence of invasive breast cancer by 44% compared with placebo [absolute risk reduction (ARR) 1.2 per 1000 women-years]; this was primarily due to a 55% reduction in estrogen receptor (ER)-positive invasive breast cancer in the EVISTA group compared with placebo (ARR 1.2 per 1000 womenyears). There was no reduction in ER-negative invasive breast cancer. Table 8 presents efficacy and selected safety outcomes. (See Table 8) (…)
**022145, 03/05/2018**	Raltegravir	Infectious Diseases	UGT1A1	Clinical Pharmacology	**12 CLINICAL PHARMACOLOGY**<br>**12.5 Pharmacogenomics**<br>*UGT1A1 Polymorphism*<br>There is no evidence that common UGT1A1 polymorphisms alter raltegravir pharmacokinetics to a clinically meaningful extent. In a comparison of 30 adult subjects with *28/*28 genotype (associated with reduced activity of UGT1A1) to 27 adult subjects with wild-type genotype, the geometric mean ratio (90% CI) of AUC was 1.41 (0.96, 2.09). In the neonatal study IMPAACT P1110, there was no association between apparent clearance (CL/F) of raltegravir and UGT 1A1 genotype polymorphisms.
**125477, 05/29/2020**	Ramucirumab (1)	Oncology	EGFR	Indications and Usage, Dosage and Administration, Adverse Reactions, Clinical Studies	**1 INDICATIONS AND USAGE**<br>**1.2 Non-Small Cell Lung Cancer**<br>CYRAMZA, in combination with erlotinib, is indicated for the first-line treatment of patients with metastatic non-small cell lung cancer (NSCLC) whose tumors have epidermal growth factor receptor (EGFR) exon 19 deletions or exon 21 (L858R) substitution mutations. CYRAMZA, in combination with docetaxel, is indicated for the treatment of patients with metastatic non-small cell lung cancer (NSCLC) with disease progression on or after platinum-based chemotherapy. Patients with epidermal growth factor receptor (EGFR) or anaplastic lymphoma kinase (ALK) genomic tumor aberrations should have disease progression on FDA-approved therapy for these aberrations prior to receiving CYRAMZA.<br><br>**2 DOSAGE AND ADMINISTRATION**<br>**2.3 Recommended Dosage for Non-Small Cell Lung Cancer**<br>EGFR Exon 19 Deletions or Exon 21 (L858R) Substitution Mutations – CYRAMZA in Combination with Erlotinib<br>• The recommended dosage of CYRAMZA is 10 mg/kg every 2 weeks administered by intravenous infusion over 60 minutes. If the first infusion is tolerated, all subsequent CYRAMZA infusions may be administered over 30 minutes. Continue CYRAMZA until disease progression or unacceptable toxicity.<br>• Refer to the prescribing information for erlotinib for dosage information.<br><br>**6 ADVERSE REACTIONS**<br>**6.1 Clinical Trials Experience**<br>Non-Small Cell Lung Cancer<br>*CYRAMZA Administered in Combination with Erlotinib (RELAY)*<br>The safety of CYRAMZA was evaluated in RELAY [see Clinical Studies (14.2)]. Patients had previously untreated EGFR exon 19 deletion or exon 21 (L858R) substitution mutation-positive metastatic NSCLC. (…)<br><br>**14 CLINICAL STUDIES**<br>**14.2 Non-Small Cell Lung Cancer**<br>RELAY<br>The efficacy of CYRAMZA in combination with erlotinib was evaluated in RELAY (NCT02411448), a multinational, randomized, double-blind, placebo-controlled, multicenter study in patients with previously untreated metastatic non-small cell lung cancer (NSCLC) whose tumors have epidermal growth factor receptor (EGFR) exon 19 deletion or exon 21 (L858R) substitution mutations. Patients in RELAY were required to have measurable disease, ECOG PS of 0 or 1, no central nervous system (CNS) metastases, and no known EGFR T790M mutations at baseline. Patients were randomized (1:1) to receive either CYRAMZA 10 mg/kg or placebo every 2 weeks as an intravenous infusion, in combination with erlotinib 150 mg orally once daily until disease progression or unacceptable toxicity. Randomization was stratified by geographic region (East Asia versus other), gender, EGFR mutation (exon 19 deletion versus exon 21 [L858R] substitution mutation), and local EGFR testing method (therascreen® and cobas® versus other polymerase chain reaction [PCR] and sequencing-based methods).<br>A total of 449 patients were randomized, 224 to the CYRAMZA-treatment group and 225 to the placebo-treatment group. Baseline demographics and disease characteristics were similar between treatment arms. The median age was 65 years (range 23-89); 63% of patients were female; 77% were Asian and 22% were White; 52% had ECOG PS 0; 61% were never smokers; 54% had exon 19 mutation deletions, and 45% had exon 21 (L858R) substitution mutations. (…)<br>REVEL

* Therapeutic areas do not necessarily reflect the CDER review division.
† Representative biomarkers are listed based on standard nomenclature as per the Human Genome Organization (HUGO) symbol and/or simplified descriptors using other common conventions. Listed biomarkers do not necessarily reflect the terminology used in labeling. The term “Nonspecific” is provided when labeling does not explicitly identify the specific biomarker(s) or when the biomarker is represented by a molecular phenotype or gene signature, and in some cases the biomarker was inferred based on the labeling language.
‡ Referenced figures and tables may be found in the labeling available at Drugs@FDA.
Blue text represents the most recent addtions and/or changes since last posted version.

Table of Pharmacogenomic Biomarkers in Drug Labeling

Last Updated: 06/2024

NDA/ANDA/BLA Number, Label Version Date	Drug	Therapeutic Area*	Biomarker†	Labeling Sections	Labeling Text‡
					(…) Tumor EGFR status was unknown for the majority of patients (65%). Where tumor EGFR status was known (n=445), 7.4% were positive for EGFR mutation (n=33). No data were collected regarding tumor ALK rearrangement status. (…)
**125477, 05/29/2020**	Ramucirumab (2)	Oncology	RAS	Clinical Studies	**14 CLINICAL STUDIES**<br>**14.3 Colorectal Cancer**<br>(…) Randomization was stratified by geographic region, tumor KRAS status, and time to disease progression after beginning first-line treatment (<6 months versus ≥6 months).<br>Demographic and baseline characteristics were similar between treatment arms. Median age was 62 years; 57% of patients were men; 76% were White and 20% Asian; 49% had ECOG PS 0; 49% of patients had KRAS mutant tumors; and 24% of patients had <6 months from time to disease progression after beginning first-line treatment. (…)
**103946, 12/12/2019**	Rasburicase (1)	Oncology	G6PD	Boxed Warning, Contraindications, Warnings and Precautions	**BOXED WARNING**<br>WARNING: HYPERSENSITIVITY REACTIONS, HEMOLYSIS, METHEMOGLOBINEMIA, AND INTERFERENCE WITH URIC ACID MEASUREMENTS<br>*Hemolysis*<br>Do not administer Elitek to patients with glucose-6- phosphate dehydrogenase (G6PD) deficiency. Immediately and permanently discontinue Elitek if hemolysis occurs. Screen patients at higher risk for G6PD deficiency (e.g., patients of African or Mediterranean ancestry) prior to starting Elitek therapy (4, 5.2).<br><br>**4 CONTRAINDICATIONS**<br>Elitek is contraindicated in individuals deficient in glucose-6-phosphate dehydrogenase (G6PD) [see Boxed Warning, Warnings and Precautions (5.2)].<br><br>**5 WARNINGS AND PRECAUTIONS**<br>**5.2 Hemolysis**<br>Elitek is contraindicated in patients with G6PD deficiency because hydrogen peroxide is one of the major by-products of the conversion of uric acid to allantoin. In clinical studies, hemolysis occurs in <1% patients receiving Elitek; severe hemolytic reactions occurred within 2-4 days of the start of Elitek. Immediately and permanently discontinue Elitek administration in any patient developing hemolysis. Institute appropriate patient monitoring and support measures (e.g., transfusion support). Screen patients at higher risk for G6PD deficiency (e.g., patients of African or Mediterranean ancestry) prior to starting Elitek [see Boxed Warning, Contraindications (4)].
**103946, 12/12/2097**	Rasburicase (2)	Oncology	CYB5R	Boxed Warning, Contraindications, Warnings and Precautions	**BOXED WARNING**<br>WARNING: HYPERSENSITIVITY REACTIONS, HEMOLYSIS, METHEMOGLOBINEMIA, AND INTERFERENCE WITH URIC ACID MEASUREMENTS<br>*Methemoglobinemia*<br>Elitek can result in methemoglobinemia in some patients. Immediately and permanently discontinue Elitek if methemoglobinemia occurs (4, 5.3).<br><br>**4 CONTRAINDICATIONS**<br>Elitek is contraindicated in patients with a history of anaphylaxis or severe hypersensitivity to rasburicase or in patients with development of hemolytic reactions or methemoglobinemia with rasburicase [see Boxed Warning, Warnings and Precautions (5)].<br><br>**5 WARNINGS AND PRECAUTIONS**<br>**5.3 Methemoglobinemia**<br>In clinical studies, methemoglobinemia occurred in <1% patients receiving Elitek. These included cases of serious hypoxemia requiring intervention with medical support measures. It is not known whether patients with deficiency of cytochrome b5 reductase (formerly known as methemoglobin reductase) or of other enzymes with antioxidant activity are at increased risk for methemoglobinemia or hemolytic anemia. Immediately and permanently discontinue Elitek administration in any patient identified as having developed methemoglobinemia. Institute appropriate monitoring and support measures (e.g., transfusion support, methylene-blue administration) [see Boxed Warning, Contraindications (4)].
**761108, 06/28/2024**	Ravulizumab-cwvz (1)	Neurology	ACHR	Indications and Usage, Clinical Studies	**1 INDICATIONS AND USAGE**<br>**1.3 Generalized Myasthenia Gravis**<br>ULTOMIRIS is indicated for the treatment of adult patients with generalized myasthenia gravis (gMG) who are anti-acetylcholine receptor (AChR) antibody-positive.<br><br>**14 CLINICAL STUDIES**<br>**14.3 Generalized Myasthenia Gravis (gMG)**<br>The efficacy of ULTOMIRIS for the treatment of gMG was demonstrated in a randomized, double-blind, placebo-controlled, multicenter study (ALXN1210-MG-306; NCT03920293). Patients were randomized 1:1 to either receive ULTOMIRIS (n=86) or placebo (n=89) for 26 weeks. ULTOMIRIS was administered intravenously according to the weight-based recommended dosage [see Dosage and Administration (2.2)].<br>Patients with gMG with a positive serologic test for anti-AChR antibodies, Myasthenia Gravis Foundation of America (MGFA) clinical classification class II to IV, and Myasthenia Gravis-Activities of Daily Living (MG-ADL) total score ≥6 were enrolled. (…)
**761108, 06/28/2024**	**Ravulizumab-cwvz (2)**	Neurology	AQP4	Indications and Usage, Clinical Studies	**1 INDICATIONS AND USAGE**<br>**1.4 Neuromyelitis Optica Spectrum Disorder**<br>ULTOMIRIS is indicated for the treatment of adult patients with neuromyelitis optica spectrum disorder (NMOSD) who are anti-aquaporin-4 (AQP4) antibody positive.<br><br>**14 CLINICAL STUDIES**

* Therapeutic areas do not necessarily reflect the CDER review division.
† Representative biomarkers are listed based on standard nomenclature as per the Human Genome Organization (HUGO) symbol and/or simplified descriptors using other common conventions. Listed biomarkers do not necessarily reflect the terminology used in labeling. The term “Nonspecific” is provided when labeling does not explicitly identify the specific biomarker(s) or when the biomarker is represented by a molecular phenotype or gene signature, and in some cases the biomarker was inferred based on the labeling language.
‡ Referenced figures and tables may be found in the labeling available at Drugs@FDA.
Blue text represents the most recent addtions and/or changes since last posted version.

NDA/ANDA/BLA Number, Label Version Date	Drug	Therapeutic Area*	Biomarker†	Labeling Sections	Labeling Text‡
					**14.4 Neuromyelitis Optica Spectrum Disorder (NMOSD)**<br>The efficacy and safety of ULTOMIRIS in adult patients with anti-AQP4 antibody positive NMOSD was assessed in an open-label multicenter study, Study ALXN1210-NMO-307 (NCT04291262). Patients participating in Study ALXN1210-NMO-307 received ULTOMIRIS intravenously in the Primary Treatment Period that ended when the last enrolled patient completed (or discontinued prior to) 50 weeks on study, representing a median study duration of 73.5 weeks (minimum 13.7, maximum 117.7). Efficacy assessments were based on a comparison of patients in Study ALXN1210-NMO-307 with an external placebo control group from another study (Study ECU-NMO-301, NCT01892345) composed of a comparable population of adult patients with anti-AQP4 antibody positive NMOSD. Study ALXN1210-NMO-307 enrolled 58 adult patients with NMOSD who had a positive serologic test for anti-AQP4 antibodies, at least 1 relapse in the last 12 months prior to the Screening Period, and an Expanded Disability Status Scale (EDSS) score ≤ 7. In the external placebo control group, eligibility criteria were similar except patients were required to have at least 2 relapses in last 12 months or 3 relapses in the last 24 months with at least 1 relapse in the 12 months prior to screening. Prior treatment with immunosuppressant therapies (ISTs) was not required for enrollment. However, patients on selected ISTs (i.e., corticosteroids, azathioprine, mycophenolate mofetil, methotrexate, and tacrolimus) were permitted to continue on therapy, with a requirement for stable dosing until they reached Week 106 in the Study. Similar IST use was permitted in the external placebo control group. (…)
**203085, 02/13/2020**	Regorafenib	Oncology	RAS	Indications and Usage, Clinical Studies	**1 INDICATIONS AND USAGE**<br>**1.1 Colorectal Cancer**<br>STIVARGA is indicated for the treatment of patients with metastatic colorectal cancer (CRC) who have been previously treated with fluoropyrimidine-, oxaliplatin- and irinotecan-based chemotherapy, an anti-VEGF therapy, and, if RAS wildtype, an anti-EGFR therapy.<br><br>**14 CLINICAL STUDIES**<br>**14.1 Colorectal Cancer**<br>(…) Baseline demographics were: median age 61 years, 61% men, 78% White, and all patients had an ECOG performance status of 0 or 1. The primary sites of disease were colon (65%), rectum (29%), or both (6%). History of KRAS evaluation was reported for 729 (96%) patients; 430 (59%) of these patients were reported to have KRAS mutation. The median number of prior lines of therapy for metastatic disease was 3. All patients received prior treatment with fluoropyrimidine-, oxaliplatin-, and irinotecan-based chemotherapy, and with bevacizumab. All but one patient with KRAS mutationnegative tumors received panitumumab or cetuximab. (…)
**218213, 06/13/2024**	**Repotrectinib (1)**	Oncology	ROS1	Indications and Usage, Dosage and Administration, Adverse Reactions, Use in Specific Populations, Clinical Studies	**1 INDICATIONS AND USAGE**<br>AUGTYRO is indicated for the treatment of adult patients with locally advanced or metastatic ROS1-positive non-small cell lung cancer (NSCLC) [see Dosage and Administration (2.1)].<br><br>**2 DOSAGE AND ADMINISTRATION**<br>**2.1 Patient Selection**<br>Select patients for the treatment of locally advanced or metastatic NSCLC with AUGTYRO based on the presence of ROS1 rearrangement(s) in tumor specimens [see Clinical Studies (14.1)]. An FDA-approved test to detect ROS1 rearrangements for selecting patients for treatment with AUGTYRO is not currently available.<br><br>**6 ADVERSE REACTIONS**<br>The pooled safety population described in WARNINGS AND PRECAUTIONS reflects exposure to AUGTYRO as a single agent dosed at 160 mg orally once daily for 14 days, then increased to 160 mg twice daily until disease progression or unacceptable toxicity in 351 patients with ROS1- positive NSCLC and other solid tumors in the TRIDENT-1 trial. (…)<br>TRIDENT-1<br>The safety of AUGTYRO was evaluated in 264 patients with ROS1-positive NSCLC in TRIDENT-1 [see Clinical Studies (14.1)]. Eligible patients had an ECOG status of ≤1. (See Tables 3 and 4) (…)<br><br>**8 USE IN SPECIFIC POPULATIONS**<br>**8.4 Pediatric Use**<br>The safety and effectiveness of AUGTYRO in pediatric patients with ROS1-positive NSCLC has not been established.<br>**8.5 Geriatric Use**<br>Of the 426 patients who received AUGTYRO, in the TRIDENT-1 study for ROS1-positive non-small cell lung cancer or NTRK gene fusion-positive solid tumors, 19% were 65 to 75 years old, and 6% were 75 years of age or older. There were no clinically meaningful differences in safety and efficacy between patients younger than 65 years of age and patients 65 years of age or older.<br><br>**14 CLINICAL STUDIES**<br>**14.1 Locally Advanced or Metastatic ROS1-Positive NSCLC**<br>The efficacy of AUGTYRO was evaluated in TRIDENT-1, a multicenter, single-arm, open-label, multi-cohort clinical trial (NCT03093116). Patients were required to have ROS1-positive locally advanced or metastatic NSCLC, ECOG performance status ≤1, measurable disease per RECIST v 1.1, and ≥8 months from first dose. All patients were assessed for CNS lesions at baseline, and patients with symptomatic brain metastases were excluded from the trial. Patients received AUGTYRO 160 mg orally once daily for 14 days, then increased to 160 mg twice daily until disease progression or unacceptable toxicity. Tumor assessments were performed at least every 8 weeks. Identification of ROS1 gene fusions in tumor specimens was prospectively determined in local laboratories using next-generation sequencing (NGS), polymerase chain reaction (PCR) or fluorescence in situ hybridization (FISH) tests. All ROS1-positive patients by local FISH testing required central laboratory confirmation of ROS1 fusion using an analytically validated NGS test. ROS1 fusions were identified by NGS in 51%, FISH in 26%, and PCR in 23%. The major efficacy outcome measures were overall response rate (ORR) and duration of response (DOR) according to RECIST v1.1 as

* Therapeutic areas do not necessarily reflect the CDER review division.
† Representative biomarkers are listed based on standard nomenclature as per the Human Genome Organization (HUGO) symbol and/or simplified descriptors using other common conventions. Listed biomarkers do not necessarily reflect the terminology used in labeling. The term “Nonspecific” is provided when labeling does not explicitly identify the specific biomarker(s) or when the biomarker is represented by a molecular phenotype or gene signature, and in some cases the biomarker was inferred based on the labeling language.
‡ Referenced figures and tables may be found in the labeling available at Drugs@FDA.
Blue text represents the most recent addtions and/or changes since last posted version.

NDA/ANDA/BLA Number, Label Version Date	Drug	Therapeutic Area*	Biomarker†	Labeling Sections	Labeling Text‡
					assessed by blinded independent central review (BICR). Intracranial response according to modified RECIST v1.1 was assessed by BICR. Tumor assessments with imaging were performed every 8 weeks. The efficacy populations included 71 ROS1 TKI-naïve patients who received up to 1 prior line of platinum-based chemotherapy and/or immunotherapy and 56 patients who received 1 prior ROS1 TKI with no prior platinum-based chemotherapy or immunotherapy.<br>Among the 71 ROS1 TKI-naïve patients, the median age was 57 years (range: 28 to 80); female (60.6%); Asian (67.6%), White (25.4%), Hispanic or Latino (4.2%), Black or African American (1.4%); never smoked (63.4%); and ECOG performance status of 1 at baseline (66.2%). At baseline, 94.4% of patients had metastatic disease, 25.4% of patients had CNS metastases by BICR; 97.2% had adenocarcinoma; and 28.2% patients had prior chemotherapy consisting of platinum-based chemotherapy and/or immunotherapy for locally advanced or metastatic disease.<br>Among the 56 patients who had received 1 prior ROS1 TKI (including crizotinib [82%] and entrectinib [16%]) with no prior platinum-based chemotherapy or immunotherapy, the median age was 57 years (range: 33 – 78); female (67.9%); Asian (48.2%), White (44.6%), Black or African American and Hispanic or Latino (1.8% each); never smoked (64.3%); and ECOG performance status of 1 at baseline (67.9%). At baseline, 98.2% patients had metastatic disease, 42.9% with CNS metastases by BICR, and 94.6% had adenocarcinoma.<br>Efficacy results are summarized in Table 5.<br>Among TKI-naïve patients, 8 had measurable CNS metastases at baseline as assessed by BICR; responses in intracranial lesions were observed in 7 of these 8 patients. Among the TKI pretreated patients with no prior platinum-based chemotherapy, 12 had measurable CNS metastases at baseline as assessed by BICR; responses in intracranial lesions were observed in 5 of these 12 patients.<br>Among the 56 ROS1 inhibitor-pretreated patients, 8 had resistance mutations following TKI therapy. Responses were observed in 6 of these 8 patients; responders included patients with solvent front (ROS1G2032R), gatekeeper (ROS1L2026M), and other mutations (ROS1S1986F/Y).
**218213, 06/13/2024**	**Repotrectinib (2)**	Oncology	NTRK	Indications and Usage, Dosage and Administration, Adverse Reactions, Use in Specific Populations, Clinical Studies	**1 INDICATIONS AND USAGE**<br>**1.2 NTRK Gene Fusion-Positive Solid Tumors**<br>AUGTYRO is indicated for the treatment of adult and pediatric patients 12 years of age and older with solid tumors that:<br>• have a neurotrophic tyrosine receptor kinase (NTRK) gene fusion [see Dosage and Administration (2.1)],<br>• are locally advanced or metastatic or where surgical resection is likely to result in severe morbidity, and<br>• have progressed following treatment or have no satisfactory alternative therapy.<br>This indication is approved under accelerated approval based on overall response rate and duration of response [seeClinical Studies (14.2)]. Continued approval for this indication may be contingent upon verification and description of clinical benefit in a confirmatory trial(s).<br><br>**2 DOSAGE AND ADMINISTRATION**<br>**2.1 Patient Selection**<br><u>Solid Tumors</u><br>Select patients for the treatment of solid tumors with AUGTYRO based on the presence of NTRK1/2/3 rearrangements in tumor specimens [see Clinical Studies (14.2)]. An FDA-approved test to detect NTRK1/2/3 rearrangements for selecting patients for treatment with AUGTYRO is not currently available.<br>• In patients with secretory breast cancer or mammary analogue secretory cancer, consider treatment without confirmation of NTRK rearrangements in tumor specimens.<br><br>**6 ADVERSE REACTIONS**<br>**6.1 Clinical Trials Experience**<br>Because clinical trials are conducted under widely varying conditions, adverse reaction rates reported in the clinical trials of a drug cannot be directly compared to rates in the clinical trials of another drug and may not reflect the rates observed in practice.<br>The pooled safety population described in WARNINGS AND PRECAUTIONS and below reflects exposure to AUGTYRO in 426 patients with ROS1-positive NSCLC (n=320), NTRK1/2/3-positive solid tumors (n=104), or other solid tumors (n=2) in TRIDENT-1. Patients received AUGTYRO at a dose of 160 mg orally once daily for the first 14 days, then increased to 160 mg orally twice daily until disease progression or unacceptable toxicity [see Clinical Studies (14.1), (14.2)]. Eligible patients had an ECOG status of ≤1. Patients with a history of ILD, drug-related pneumonitis, significant, uncontrolled, active cardiovascular disease, or prolonged QTc interval were excluded from enrollment in this trial. Forty-eight percent of patients were exposed to AUGTYRO for at least 6 months, and 28% were exposed for greater than 1 year.<br><br>**8 USE IN SPECIFIC POPULATIONS**<br>**8.4 Pediatric Use**<br>The safety and effectiveness of AUGTYRO in pediatric patients with ROS1-positive NSCLC have not been established.<br>The safety and effectiveness of AUGTYRO have not been established in pediatric patients younger than 12 years of age with solid tumors who have an NTRK gene fusion.<br>The safety and effectiveness of AUGTYRO for the treatment of locally advanced or metastatic NTRK-positive solid tumors have been established in pediatric patients 12 years of age or older. Use of AUGTYRO in this age group is supported by evidence from an adequate and well-controlled study in adult patients with additional pharmacokinetic and safety data in pediatric patients aged 12 years and older. This includes data demonstrating that the exposure of repotrectinib in pediatric patients 12 years of age and older is expected to result in similar safety and efficacy to that of adults, and that the course of locally advanced or metastatic NTRK-positive solid tumors is sufficiently similar in adults and pediatric patients 12 years of age or older to allow extrapolation of data in adult to pediatric patients 12 years of age or older [see Dosage and Administration (2.4), Warnings and Precautions (5.7), Adverse Reactions (6.1) and Clinical Pharmacology (12.3)].<br>**8.5 Geriatric Use**

NDA/ANDA/BLA Number, Label Version Date	Drug	Therapeutic Area*	Biomarker†	Labeling Sections	Labeling Text‡
					Of the 426 patients who received AUGTYRO, in the TRIDENT-1 study for ROS1-positive non-small cell lung cancer or NTRK gene fusion-positive solid tumors, 19% were 65 to 75 years old, and 6% were 75 years of age or older. There were no clinically meaningful differences in safety and efficacy between patients younger than 65 years of age and patients 65 years of age or older.<br><br>**14 CLINICAL STUDIES**<br>**14.2 Locally Advanced or Metastatic NTRK Gene Fusion-Positive Solid Tumors**<br>The efficacy of AUGTYRO was evaluated in TRIDENT-1 (NCT03093116), a multi-center, single-arm, open-label, multi-cohort clinical trial in 88 adult patients with locally advanced or metastatic NTRK gene fusion-positive (NTRK1/2/3) solid tumors who had either received a prior TKI treatment or were TKI-naïve. All patients were assessed for CNS lesions at baseline, and patients with symptomatic brain metastases were excluded from the trial. Patients received AUGTYRO 160 mg orally once daily for 14 days, then increased to 160 mg twice daily until disease progression or unacceptable toxicity. Tumor assessments were performed every 8 weeks. NTRK gene fusions were identified prospectively using NGS in 94%, FISH in 5%, and PCR in 1%. NTRK gene fusion-positive tumors identified by local FISH testing required central laboratory confirmation using an analytically validated NGS test. The major efficacy outcome measures were ORR and DOR according to RECIST v1.1 as assessed by BICR. Intracranial response according to modified RECIST v1.1 was assessed by BICR.<br>Among the 40 TRK TKI-naïve patients, the median age was 61 years (range: 25 to 84); 60% were female patients; race was Asian 53%, White 25%, Black or African American 5%, and other or not reported 18%; ethnicity was Hispanic or Latino 5%, not Hispanic or Latino 87%, and not reported 8%; and ECOG performance status of 1 at baseline was 55%. At baseline, 98% of patients had metastatic disease and 23% of patients had CNS metastases by BICR. Seventy percent (n=28) of patients received prior systemic therapy with a median of one prior systemic regimen, and 7.5% (n=3) received three or more prior systemic regimens.<br>Among the 48 TRK TKI-pretreated patients, the median age was 58 years (range: 20 to 81); 48% were female patients; race was White 65%, Asian 25%, Black or African American 2%, and not reported 8%; ethnicity was not Hispanic or Latino 92%, and missing 8%; and ECOG performance status of 1 at baseline was 60%. At baseline, 96% of patients had metastatic disease and 25% of patients had CNS metastases by BICR. Seventy-seven percent (n=37) of patients received 2 or more prior systemic regimens, and 46% (n=22) received three or more prior systemic regimens, and 7 patients (15%) received 2 prior TKI therapies.<br>Efficacy results are summarized in Table 6.<br>Among the 88 patients, 5 had measurable CNS metastases at baseline as assessed by BICR. Responses were seen in 2 (100%) TKI-naïve patients and 3 (100%) TKI-pretreated patients. One out of 2 TKI-naïve and 2 out of 3 TKI-pretreated patients received prior radiotherapy to the brain, all more than 2 months prior to study entry.<br>Twenty-six of the TRK TKI-pretreated patients had a resistance mutation at baseline, including 24 with solvent front mutations (NTRK1G595R and NTRK3G623L/R/E/V mutations), one with both a solvent front mutation and a gatekeeper mutation (NTRK1F589L), and one with another mutation (NTRK1G667C). In the 25 TKI-pretreated patients with solvent front mutations at baseline, ORR was 60% (95% CI: 39, 79).<br>ORR and DOR by tumor type in adult patients with NTRK gene fusion-positive solid tumors are presented in Tables 7 and 8 below.<br>ORR and DOR in adult patients are presented by NTRK gene fusion partner Tables 9 and 10 below.
217785, 03/14/2024	**Resmetirom**	Gastroenterology	ABCG2	Clinical Pharmacology	**12 CLINICAL PHARMACOLOGY**<br>**12.3 Pharmacokinetics**<br>Specific Populations<br>No clinically significant differences in the pharmacokinetics of resmetirom were observed based on age (18 to 83 years), sex, race (White, Black, or Asian), or ABCG2 genotype (BCRP p.Gln141Lys, p.Val12Met).
**209092, 01/21/2020**	**Ribociclib (1)**	Oncology	ESR, PGR (Hormone Receptor)	Indications and Usage, Adverse Reactions, Clinical Studies	**1 INDICATIONS AND USAGE**<br>KISQALI is indicated in combination with:<br>• an aromatase inhibitor for the treatment of pre/perimenopausal or postmenopausal women, with hormone receptor (HR)-positive, human epidermal growth factor receptor 2 (HER2)-negative advanced or metastatic breast cancer, as initial endocrine-based therapy; or<br>• fulvestrant for the treatment of postmenopausal women with HR-positive, HER2-negative advanced or metastatic breast cancer, as initial endocrine based therapy or following disease progression on endocrine therapy.<br><br>**6 ADVERSE REACTIONS**<br>**MONALEESA-2: KISQALI in combination with Letrozole**<br>*Postmenopausal women with HR-positive, HER2-negative advanced or metastatic breast cancer for initial endocrine based therapy*<br>The safety data reported below are based on MONALEESA-2, a clinical study of 668 postmenopausal women receiving KISQALI plus letrozole or placebo plus letrozole. The median duration of exposure to KISQALI plus letrozole was 13 months with 58% of patients exposed for ≥12 months. (…)<br>**MONALEESA-7: KISQALI in combination with an Aromatase Inhibitor**<br>*Pre/perimenopausal patients with HR-positive, HER2-negative advanced or metastatic breast cancer for initial endocrine based therapy*<br>MONALEESA-7 was conducted in 672 pre/perimenopausal patients with HR-positive, HER2-negative advanced or metastatic breast cancer receiving either KISQALI plus a non-steroidal aromatase inhibitors (NSAI) or tamoxifen plus goserelin or placebo plus NSAI or tamoxifen plus goserelin. (…)<br>**MONALEESA-3: KISQALI in combination with Fulvestrant**<br>*Postmenopausal patients with HR-positive, HER2-negative advanced or metastatic breast cancer for initial endocrine based therapy or after disease progression on endocrine therapy*<br>The safety data reported below are based on MONALEESA-3, a clinical study of 724 postmenopausal women receiving KISQALI plus fulvestrant or placebo plus fulvestrant. The median duration of exposure to KISQALI plus fulvestrant was 15.8 months with 58% of patients exposed for ≥ 12 months. (…)<br><br>**14 CLINICAL STUDIES**

* Therapeutic areas do not necessarily reflect the CDER review division.
† Representative biomarkers are listed based on standard nomenclature as per the Human Genome Organization (HUGO) symbol and/or simplified descriptors using other common conventions. Listed biomarkers do not necessarily reflect the terminology used in labeling. The term “Nonspecific” is provided when labeling does not explicitly identify the specific biomarker(s) or when the biomarker is represented by a molecular phenotype or gene signature, and in some cases the biomarker was inferred based on the labeling language.
‡ Referenced figures and tables may be found in the labeling available at Drugs@FDA.
Blue text represents the most recent addtions and/or changes since last posted version.

NDA/ANDA/BLA Number, Label Version Date	Drug	Therapeutic Area*	Biomarker†	Labeling Sections	Labeling Text‡
					**MONALEESA-2: KISQALI in Combination with Letrozole**<br>*Postmenopausal women with HR-positive, HER2-negative advanced or metastatic breast cancer for initial endocrine based therapy*<br>MONALEESA-2 was a randomized, double-blind, placebo-controlled, multicenter clinical study of KISQALI plus letrozole versus placebo plus letrozole conducted in postmenopausal women with HR-positive, HER2-negative, advanced breast cancer who received no prior therapy for advanced disease. (…)<br>**MONALEESA-7: KISQALI in Combination with an Aromatase Inhibitor**<br>*Pre/perimenopausal patients with HR-positive, HER2-negative advanced or metastatic breast cancer for initial endocrine based therapy*<br>MONALEESA-7 was a randomized, double-blind, placebo-controlled study of KISQALI plus either a non-steroidal aromatase inhibitor (NSAI) or tamoxifen and goserelin versus placebo plus either a NSAI or tamoxifen and goserelin conducted in pre/perimenopausal women with HR-positive, HER2-negative, advanced breast cancer who received no prior endocrine therapy for advanced disease.(…)<br>**MONALEESA-3: KISQALI in Combination with Fulvestrant**<br>*Postmenopausal women with HR-positive, HER2-negative advanced or metastatic breast cancer for initial endocrine based therapy or after disease progression on endocrine therapy*<br>MONALEESA-3 was a randomized double-blind, placebo-controlled study of ribociclib in combination with fulvestrant for the treatment of postmenopausal women with hormone receptor positive, HER2-negative, advanced breast cancer who have received no or only one line of prior endocrine treatment. (…)
**209092, 01/21/2020**	Ribociclib (2)	Oncology	ERBB2 (HER2)	Indications and Usage, Adverse Reactions, Clincal Studies	**1 INDICATIONS AND USAGE**<br>KISQALI is indicated in combination with:<br>• an aromatase inhibitor for the treatment of pre/perimenopausal or postmenopausal women, with hormone receptor (HR)-positive, human epidermal growth factor receptor 2 (HER2)-negative advanced or metastatic breast cancer, as initial endocrine-based therapy; or<br>• fulvestrant for the treatment of postmenopausal women with HR-positive, HER2-negative advanced or metastatic breast cancer, as initial endocrine based therapy or following disease progression on endocrine therapy.<br><br>**6 ADVERSE REACTIONS**<br>**MONALEESA-2: KISQALI in combination with Letrozole**<br>*Postmenopausal women with HR-positive, HER2-negative advanced or metastatic breast cancer for initial endocrine based therapy*<br>The safety data reported below are based on MONALEESA-2, a clinical study of 668 postmenopausal women receiving KISQALI plus letrozole or placebo plus letrozole. The median duration of exposure to KISQALI plus letrozole was 13 months with 58% of patients exposed for ≥12 months. (…)<br>**MONALEESA-7: KISQALI in combination with an Aromatase Inhibitor**<br>*Pre/perimenopausal patients with HR-positive, HER2-negative advanced or metastatic breast cancer for initial endocrine based therapy*<br>MONALEESA-7 was conducted in 672 pre/perimenopausal patients with HR-positive, HER2-negative advanced or metastatic breast cancer receiving either KISQALI plus a non-steroidal aromatase inhibitors (NSAI) or tamoxifen plus goserelin or placebo plus NSAI or tamoxifen plus goserelin. (…)<br>**MONALEESA-3: KISQALI in combination with Fulvestrant**<br>*Postmenopausal patients with HR-positive, HER2-negative advanced or metastatic breast cancer for initial endocrine based therapy or after disease progression on endocrine therapy*<br>The safety data reported below are based on MONALEESA-3, a clinical study of 724 postmenopausal women receiving KISQALI plus fulvestrant or placebo plus fulvestrant. The median duration of exposure to KISQALI plus fulvestrant was 15.8 months with 58% of patients exposed for ≥ 12 months. (…)<br><br>**14 CLINICAL STUDIES**<br>**MONALEESA-2: KISQALI in Combination with Letrozole**<br>*Postmenopausal women with HR-positive, HER2-negative advanced or metastatic breast cancer for initial endocrine based therapy*<br>MONALEESA-2 was a randomized, double-blind, placebo-controlled, multicenter clinical study of KISQALI plus letrozole versus placebo plus letrozole conducted in postmenopausal women with HR-positive, HER2-negative, advanced breast cancer who received no prior therapy for advanced disease. (…)<br>**MONALEESA-7: KISQALI in Combination with an Aromatase Inhibitor**<br>*Pre/perimenopausal patients with HR-positive, HER2-negative advanced or metastatic breast cancer for initial endocrine based therapy*<br>MONALEESA-7 was a randomized, double-blind, placebo-controlled study of KISQALI plus either a non-steroidal aromatase inhibitor (NSAI) or tamoxifen and goserelin versus placebo plus either a NSAI or tamoxifen and goserelin conducted in pre/perimenopausal women with HR-positive, HER2-negative, advanced breast cancer who received no prior endocrine therapy for advanced disease.(…)<br>**MONALEESA-3: KISQALI in Combination with Fulvestrant**<br>*Postmenopausal women with HR-positive, HER2-negative advanced or metastatic breast cancer for initial endocrine based therapy or after disease progression on endocrine therapy*<br>MONALEESA-3 was a randomized double-blind, placebo-controlled study of ribociclib in combination with fulvestrant for the treatment of postmenopausal women with hormone receptor positive, HER2-negative, advanced breast cancer who have received no or only one line of prior endocrine treatment. (…)
**212728, 02/27/2020**	Rimegepant	Anesthesiology	CYP2C9	Clinical Pharmacology	**12 CLINICAL PHARMACOLOGY**<br>**12.3 Pharmacokinetics**<br>*Other Specific Populations*<br>No clinically significant differences in the pharmacokinetics of rimegepant were observed based on age, sex, race/ethnicity, body weight, or CYP2C9 genotype [see Clinical Pharmacology (12.5)].<br>**12.5 Pharmacogenomics**<br>CYP2C9 activity is reduced in individuals with genetic variants such as the CYP2C9*2 and CYP2C9*3 alleles. Rimegepant Cmax and AUC0-inf were similar in CYP2C9 intermediate metabolizers (i.e., *1/*2, *2/*2, *1/*3, n=43) as compared to normal metabolizers (i.e., *1/*1, N=72). Adequate PK data are not available

* Therapeutic areas do not necessarily reflect the CDER review division.
† Representative biomarkers are listed based on standard nomenclature as per the Human Genome Organization (HUGO) symbol and/or simplified descriptors using other common conventions. Listed biomarkers do not necessarily reflect the terminology used in labeling. The term “Nonspecific” is provided when labeling does not explicitly identify the specific biomarker(s) or when the biomarker is represented by a molecular phenotype or gene signature, and in some cases the biomarker was inferred based on the labeling language.
‡ Referenced figures and tables may be found in the labeling available at Drugs@FDA.
Blue text represents the most recent addtions and/or changes since last posted version.

NDA/ANDA/BLA Number, Label Version Date	Drug	Therapeutic Area*	Biomarker†	Labeling Sections	Labeling Text‡
					from CYP2C9 poor metabolizers (i.e., *2/*3). Since the contribution of CYP2C9 to rimegepant metabolism is considered minor, CYP2C9 polymorphism is not expected to significantly affect its exposure.
**213535, 08/07/2020**	Risdiplam	Neurology	SMN1, SMN2	Clinical Studies	**14 CLINICAL STUDIES**<br>(…) The median age of onset of clinical signs and symptoms of Type 1 SMA in patients enrolled in Part 1 of Study 1 was 2.0 months (range: 0.9 to 3.0); 71% of patients were female, 81% were Caucasian, and 19% were Asian. The median age at enrollment was 6.7 months (range: 3.3 to 6.9), and the median time between onset of symptoms and first dose was 4.0 months (range: 2.0 to 5.8). All patients had genetic confirmation of homozygous deletion or compound heterozygosity predictive of loss of function of the SMN1 gene, and two SMN2 gene copies. (…)
**020272, 01/25/2019**	Risperidone	Psychiatry	CYP2D6	Clinical Pharmacology	**12 CLINICAL PHARMACOLOGY**<br>**12.3 Pharmacokinetics**<br>*Absorption*<br>Risperidone is well absorbed. The absolute oral bioavailability of risperidone is 70% (CV=25%). The relative oral bioavailability of risperidone from a tablet is 94% (CV=10%) when compared to a solution.<br>Pharmacokinetic studies showed that RISPERDAL M-TAB Orally Disintegrating Tablets and RISPERDAL Oral Solution are bioequivalent to RISPERDAL Tablets.<br>Plasma concentrations of risperidone, its major metabolite, 9-hydroxyrisperidone, and risperidone plus 9-hydroxyrisperidone are dose proportional over the dosing range of 1 to 16 mg daily (0.5 to 8 mg twice daily). Following oral administration of solution or tablet, mean peak plasma concentrations of risperidone occurred at about 1 hour. Peak concentrations of 9-hydroxyrisperidone occurred at about 3 hours in extensive metabolizers, and 17 hours in poor metabolizers. Steady-state concentrations of risperidone are reached in 1 day in extensive metabolizers and would be expected to reach steady-state in about 5 days in poor metabolizers. Steady-state concentrations of 9-hydroxyrisperidone are reached in 5-6 days (measured in extensive metabolizers).<br>*Metabolism*<br>(…) CYP 2D6, also called debrisoquin hydroxylase, is the enzyme responsible for metabolism of many neuroleptics, antidepressants, antiarrhythmics, and other drugs. CYP 2D6 is subject to genetic polymorphism (about 6%-8% of Caucasians, and a very low percentage of Asians, have little or no activity and are "poor metabolizers") and to inhibition by a variety of substrates and some non-substrates, notably quinidine. Extensive CYP 2D6 metabolizers convert risperidone rapidly into 9-hydroxyrisperidone, whereas poor CYP 2D6 metabolizers convert it much more slowly. Although extensive metabolizers have lower risperidone and higher 9-hydroxyrisperidone concentrations than poor metabolizers, the pharmacokinetics of risperidone and 9-hydroxyrisperidone combined, after single and multiple doses, are similar in extensive and poor metabolizers.<br>Risperidone could be subject to two kinds of drug-drug interactions. First, inhibitors of CYP 2D6 interfere with conversion of risperidone to 9-hydroxyrisperidone [see Drug Interactions (7)]. This occurs with quinidine, giving essentially all recipients a risperidone pharmacokinetic profile typical of poor metabolizers. The therapeutic benefits and adverse effects of risperidone in patients receiving quinidine have not been evaluated, but observations in a modest number (n≅70) of poor metabolizers given RISPERDAL do not suggest important differences between poor and extensive metabolizers. Second, co-administration of known enzyme inducers (e.g., carbamazepine, phenytoin, rifampin, and phenobarbital) with RISPERDAL may cause a decrease in the combined plasma concentrations of risperidone and 9-hydroxyrisperidone [see Drug Interactions (7)]. It would also be possible for risperidone to interfere with metabolism of other drugs metabolized by CYP 2D6. Relatively weak binding of risperidone to the enzyme suggests this is unlikely [see Drug Interactions (7)]. (…)<br>*Excretion*<br>Risperidone and its metabolites are eliminated via the urine and, to a much lesser extent, via the feces. As illustrated by a mass balance study of a single 1 mg oral dose of 14C-risperidone administered as solution to three healthy male volunteers, total recovery of radioactivity at 1 week was 84%, including 70% in the urine and 14% in the feces.<br>The apparent half-life of risperidone was 3 hours (CV=30%) in extensive metabolizers and 20 hours (CV=40%) in poor metabolizers. The apparent half-life of 9-hydroxyrisperidone was about 21 hours (CV=20%) in extensive metabolizers and 30 hours (CV=25%) in poor metabolizers. The pharmacokinetics of risperidone and 9-hydroxyrisperidone combined, after single and multiple doses, were similar in extensive and poor metabolizers, with an overall mean elimination half-life of about 20 hours.
**103705, 12/17/2021**	Rituximab	Oncology	MS4A1 (CD20 antigen)	Indications and Usage, Dosage and Administration, Adverse Reactions, Use in Specific Populations, Clinical Studies	**1 INDICATIONS AND USAGE**<br>**1.1 Non–Hodgkin's Lymphoma (NHL)**<br>Rituxan (rituximab) is indicated for the treatment of patients with:<br>• Relapsed or refractory, low-grade or follicular, CD20-positive, B-cell NHL as a single agent.<br>• Previously untreated follicular, CD20-positive, B-cell NHL in combination with first line chemotherapy and, in patients achieving a complete or partial response to Rituxan in combination with chemotherapy, as single-agent maintenance therapy.<br>• Non-progressing (including stable disease), low-grade, CD20-positive, B-cell NHL as a single agent after first-line CVP chemotherapy.<br>• Previously untreated diffuse large B-cell, CD20-positive NHL in combination with CHOP or other anthracycline-based chemotherapy regimens.<br>RITUXAN is indicated for the treatment of pediatric patients aged 6 months and older with:<br>• Previously untreated, advanced stage, CD20-positive diffuse large B-cell lymphoma (DLBCL), Burkitt lymphoma (BL), Burkitt-like lymphoma (BLL) or mature B-cell acute leukemia (B-AL) in combination with chemotherapy.<br>**1.2 Chronic Lymphocytic Leukemia (CLL)**<br>Rituxan (rituximab) is indicated, in combination with fludarabine and cyclophosphamide (FC), for the treatment of patients with previously untreated and previously treated CD20-positive CLL.<br><br>**2 DOSAGE AND ADMINISTRATION**<br>**2.2 Recommended Dos e for Non-Hodgkin's Lymphoma (NHL)**<br>The recommended dose is 375 mg/m^2 as an intravenous infusion according to the following schedules:

* Therapeutic areas do not necessarily reflect the CDER review division.
† Representative biomarkers are listed based on standard nomenclature as per the Human Genome Organization (HUGO) symbol and/or simplified descriptors using other common conventions. Listed biomarkers do not necessarily reflect the terminology used in labeling. The term "Nonspecific" is provided when labeling does not explicitly identify the specific biomarker(s) or when the biomarker is represented by a molecular phenotype or gene signature, and in some cases the biomarker was inferred based on the labeling language.
‡ Referenced figures and tables may be found in the labeling available at Drugs@FDA.
Blue text represents the most recent addtions and/or changes since last posted version.

NDA/ANDA/BLA Number, Label Version Date	Drug	Therapeutic Area*	Biomarker†	Labeling Sections	Labeling Text‡
					• *Relapsed or Refractory, Low-Grade or Follicular, CD20-Positive, B-Cell NHL*<br>Administer once weekly for 4 or 8 doses.<br>• *Retreatment for Relapsed or Refractory, Low-Grade or Follicular, CD20-Positive, B-Cell NHL*<br>Administer once weekly for 4 doses.<br>• *Previously Untreated, Follicular, CD20-Positive, B-Cell NHL*<br>Administer on Day 1 of each cycle of chemotherapy, for up to 8 doses. In patients with complete or partial response, initiate Rituxan maintenance eight weeks following completion of Rituxan in combination with chemotherapy. Administer Rituxan as a single-agent every 8 weeks for 12 doses.<br>• *Non-progressing, Low-Grade, CD20-Positive, B-cell NHL, after first-line CVP chemotherapy*<br>Following completion of 6–8 cycles of CVP chemotherapy, administer once weekly for 4 doses at 6-month intervals to a maximum of 16 doses.<br>• *Diffuse Large B-Cell NHL*<br>Administer on Day 1 of each cycle of chemotherapy for up to 8 infusions.<br><br>**6 ADVERSE REACTIONS**<br>**6.1 Clinical Trials Experience in Lymphoid Malignancies**<br>*Cytopenias and hypogammaglobulinemia*<br>(…) Adverse reactions in Table 1 occurred in 356 patients with relapsed or refractory, low-grade or follicular, CD20-positive, B-cell NHL treated in single-arm studies of Rituxan administered as a single agent [See Clinical Studies (14.1)]. Most patients received Rituxan 375 mg/m^2 weekly for 4 doses. (…)<br><br>**8 USE IN SPECIFIC POPULATIONS**<br>**8.4 Pediatric Use**<br>Mature B-Cell NHL/B-AL<br>The safety and effectiveness of RITUXAN in combination with chemotherapy for the treatment of previously untreated, advanced stage, CD20-positive DLBCL/BL/BLL/B-AL have been established in pediatric patients aged 6 months and older. (…)<br>The safety and effectiveness of RITUXAN in combination with chemotherapy for previously untreated, advanced stage, CD20-positive DLBCL/BL/BLL/B-AL have not been established in pediatric patients less than 6 months of age.<br>**8.5 Geriatric Use**<br>*Low-Grade or Follicular Non-Hodgkin's Lymphoma*<br>Patients with previously untreated follicular NHL evaluated in Study 5 were randomized to Rituxan as single-agent maintenance therapy (n=505) or observation (n=513) after achieving a response to Rituxan in combination with chemotherapy. Of these, 123 (24%) patients in the Rituxan arm were age 65 or older. No overall differences in safety or effectiveness were observed between these patients and younger patients. Other clinical studies of Rituxan in low-grade or follicular, CD20-positive, B-cell NHL did not include sufficient numbers of patients aged 65 and over to determine whether they respond differently from younger subjects.<br><br>**14 CLINICAL STUDIES**<br>**14.1 Relaps ed or Refractory, Low-Grade or Follicular, CD20-Positive, B-Cell NHL**<br>The safety and effectiveness of Rituxan in relapsed, refractory CD20+ NHL were demonstrated in 3 single-arm studies enrolling 296 patients. (…)<br>**14.2 Previously Untreated, Low-Grade or Follicular, CD20-Positive, B-Cell NHL**<br>The safety and effectiveness of Rituxan in previously untreated, low-grade or follicular, CD20+ NHL were demonstrated in 3 randomized, controlled trials enrolling 1,662 patients. (…)<br>*NHL Study 11*<br>RITUXAN in combination with chemotherapy was evaluated in Inter-B-NHL Ritux 2010 (NCT01516580), a multicenter, open-label, randomized trial of patients with previously untreated, advanced stage, CD20-positive DLBCL/BL/BLL/B-AL aged 6 months and older. Advanced stage is defined as Stage III with elevated lactose dehydrogenase (LDH) level [LDH greater than twice the institutional upper limit of the adult normal values] or stage IV B-cell NHL or B-AL. LMB therapy was administered based on the clinical group classification of group B (stage III with high LDH and non-central nervous system (CNS) (Stage IV), group C1 (B-AL, CNS positive and cerebrospinal fluid (CSF) negative) and C3 (CSF positive).
**022406, 01/15/2019**	Rivaroxaban	Cardiology	F5<br>(Factor V Leiden)	Clinical Studies	**14 CLINICAL STUDIES**<br>**14.3 Reduction in the Risk of Recurrence of DVT and/or PE**<br>EINSTEIN CHOICE Study<br>(…) A total of 2275 patients were randomized and followed on study treatment for a mean of 290 days for the XARELTO and aspirin treatment groups. The mean age was approximately 59 years. The population was 56% male, 70% Caucasian, 14% Asian and 3% Black. In the EINSTEIN CHOICE study, 51% of patients had DVT only, 33% had PE only, and 16% had PE and DVT combined. Other risk factors included idiopathic VTE (43%), previous episode of DVT/PE (17%), recent surgery or trauma (12%), prolonged immobilization (10%), use of estrogen containing drugs (5%), known thrombophilic conditions (6%), Factor V Leiden gene mutation (4%), or active cancer (3%). (…)
**761166, 11/12/2021**	Ropeginterferon Alfa-2b-njft	Hematology	JAK2	Clinical Pharmacology, Clinical Studies	**12 CLINICAL PHARMACOLOGY**<br>**12.3 Pharmacokinetics**<br>Specific Populations<br>No clinically significant differences in the pharmacokinetics of BESREMi were observed based on age, sex, body surface area, and JAK2V617F mutation.<br><br>**14 CLINICAL STUDIES**

* Therapeutic areas do not necessarily reflect the CDER review division.
† Representative biomarkers are listed based on standard nomenclature as per the Human Genome Organization (HUGO) symbol and/or simplified descriptors using other common conventions. Listed biomarkers do not necessarily reflect the terminology used in labeling. The term "Nonspecific" is provided when labeling does not explicitly identify the specific biomarker(s) or when the biomarker is represented by a molecular phenotype or gene signature, and in some cases the biomarker was inferred based on the labeling language.
‡ Referenced figures and tables may be found in the labeling available at Drugs@FDA.
Blue text represents the most recent addtions and/or changes since last posted version.

NDA/ANDA/BLA Number, Label Version Date	Drug	Therapeutic Area*	Biomarker†	Labeling Sections	Labeling Text‡
					The efficacy and safety of BESREMi were evaluated in the PEGINVERA study, a prospective, multicenter, singlearm trial of 7.5 years duration. The study included 51 adults with polycythemia vera. The mean age at baseline was 56 years (range 35-82 years) with 20 (39%) women and 31 (61%) men. All patients had the JAK2V617F mutation with 16% of subjects being newly diagnosed; 84% had known disease with a median duration of 2.2 years. One-third (33%) of patients were undergoing treatment with hydroxyurea (HU) upon study entry. (…)
**020533, 11/02/2018**	Ropivacaine (1)	Anesthesiology	G6PD	Warnings	**WARNINGS**<br>**Methemoglobinemia**<br>Cases of methemoglobinemia have been reported in association with local anesthetic use. Although all patients are at risk for methemoglobinemia, patients with glucose-6- phosphate dehydrogenase deficiency, congenital or idiopathic methemoglobinemia, cardiac or pulmonary compromise, infants under 6 months of age, and concurrent exposure to oxidizing agents or their metabolites are more susceptible to developing clinical manifestations of the condition. If local anesthetics must be used in these patients, close monitoring for symptoms and signs of methemoglobinemia is recommended. (…)
**020533, 11/02/2018**	Ropivacaine (2)	Anesthesiology	Nonspecific (Congenital Methemoglobinemia)	Warnings	**WARNINGS**<br>**Methemoglobinemia**<br>Cases of methemoglobinemia have been reported in association with local anesthetic use. Although all patients are at risk for methemoglobinemia, patients with glucose-6- phosphate dehydrogenase deficiency, congenital or idiopathic methemoglobinemia, cardiac or pulmonary compromise, infants under 6 months of age, and concurrent exposure to oxidizing agents or their metabolites are more susceptible to developing clinical manifestations of the condition. If local anesthetics must be used in these patients, close monitoring for symptoms and signs of methemoglobinemia is recommended. (…)
**021366, 11/09/2018**	Rosuvastatin	Endocrinology	SLCO1B1	Clinical Pharmacology	**12 CLINICAL PHARMACOLOGY**<br>**12.5 Pharmacogenomics**<br>Disposition of HMG-CoA reductase inhibitors, including rosuvastatin, involves OATP1B1 and other transporter proteins. Higher plasma concentrations of rosuvastatin have been reported in very small groups of patients (n=3 to 5) who have two reduced function alleles of the gene that encodes OATP1B1 (SLCO1B1 521T > C). The frequency of this genotype (i.e., SLCO1B1 521 C/C) is generally lower than 5% in most racial/ethnic groups. The impact of this polymorphism on efficacy and/or safety of rosuvastatin has not been clearly established.
761286, 06/26/2023	Rozanolixizumab-noli (1)	Neurology	ACHR	Indications and Usage, Clinical Pharmacology, Clinical Studies	**1 INDICATIONS AND USAGE**<br>RYSTIGGO is indicated for the treatment of generalized myasthenia gravis (gMG) in adult patients who are anti-acetylcholine receptor (AChR) or anti-muscle-specific tyrosine kinase (MuSK) antibody positive.<br><br>**12 CLINICAL PHARMACOLOGY**<br>**12.2 Pharmacodynamics**<br>In Study 1 [see Clinical Studies (14)], the pharmacological effect of rozanolixizumab-noli was assessed by measuring the decrease in serum IgG levels and AChR and MuSK autoantibody levels. In patients testing positive for AChR and MuSK autoantibodies who were treated with RYSTIGGO, there was a reduction in total IgG levels relative to baseline. Decreases in AChR autoantibody and MuSK autoantibody levels followed a similar pattern.<br><br>**14 CLINICAL STUDIES**<br>The efficacy of RYSTIGGO for the treatment of generalized myasthenia gravis (gMG) in adults who are anti-AChR antibody positive or anti-MuSK antibody positive was established in a multicenter, randomized, double-blind, placebo-controlled study (Study 1; NCT03971422). The study included a 4-week screening period and a 6-week treatment period followed by 8 weeks of observation. During the treatment period, RYSTIGGO or placebo were administered subcutaneously once a week for six weeks.<br>Study 1 enrolled patients who met the following criteria:<br>• Presence of autoantibodies against AChR or MuSK (…)<br>(…) In Study 1, a total of 200 patients were randomized 1:1:1 to receive weight-tiered doses of RYSTIGGO (n=133), equivalent to ≈7 mg/kg (n=66) or ≈10 mg/kg (n=67), or placebo (n=67). Baseline characteristics were similar between treatment groups. Patients had a median age of 52 years at baseline (range: 18 to 89 years) and a median time since diagnosis of 6 years. Sixty-one percent of patients were female, 68% were White, 11% were Asian, 3% were Black or African American, 1% were American Indian or Alaska Native, and 7% were of Hispanic or Latino ethnicity. Median MG-ADL total score was 8, and the median Quantitative Myasthenia Gravis (QMG) total score was 15. The majority of patients, 89.5% (n=179) were positive for AChR antibodies and 10.5% (n=21) were positive for MuSK antibodies. (See Table 3)
761286, 06/26/2023	Rozanolixizumab-noli (2)	Neurology	MUSK	Indications and Usage, Clinical Pharmacology, Clinical Studies	**1 INDICATIONS AND USAGE**<br>RYSTIGGO is indicated for the treatment of generalized myasthenia gravis (gMG) in adult patients who are anti-acetylcholine receptor (AChR) or anti-muscle-specific tyrosine kinase (MuSK) antibody positive.<br><br>**12 CLINICAL PHARMACOLOGY**<br>**12.2 Pharmacodynamics**<br>In Study 1 [see Clinical Studies (14)], the pharmacological effect of rozanolixizumab-noli was assessed by measuring the decrease in serum IgG levels and AChR and MuSK autoantibody levels. In patients testing positive for AChR and MuSK autoantibodies who were treated with RYSTIGGO, there was a reduction in total IgG levels relative to baseline. Decreases in AChR autoantibody and MuSK autoantibody levels followed a similar pattern.<br><br>**14 CLINICAL STUDIES**<br>The efficacy of RYSTIGGO for the treatment of generalized myasthenia gravis (gMG) in adults who are anti-AChR antibody positive or anti-MuSK antibody positive was established in a multicenter, randomized, double-blind, placebo-controlled study (Study 1; NCT03971422). The study included a 4-week screening

NDA/ANDA/BLA Number, Label Version Date	Drug	Therapeutic Area*	Biomarker†	Labeling Sections	Labeling Text‡
					period and a 6-week treatment period followed by 8 weeks of observation. During the treatment period, RYSTIGGO or placebo were administered subcutaneously once a week for six weeks.<br>Study 1 enrolled patients who met the following criteria:<br>• Presence of autoantibodies against AChR or MuSK (…)<br>(…) In Study 1, a total of 200 patients were randomized 1:1:1 to receive weight-tiered doses of RYSTIGGO (n=133), equivalent to ≈7 mg/kg (n=66) or ≈10 mg/kg (n=67), or placebo (n=67). Baseline characteristics were similar between treatment groups. Patients had a median age of 52 years at baseline (range: 18 to 89 years) and a median time since diagnosis of 6 years. Sixty-one percent of patients were female, 68% were White, 11% were Asian, 3% were Black or African American, 1% were American Indian or Alaska Native, and 7% were of Hispanic or Latino ethnicity. Median MG-ADL total score was 8, and the median Quantitative Myasthenia Gravis (QMG) total score was 15. The majority of patients, 89.5% (n=179) were positive for AChR antibodies and 10.5% (n=21) were positive for MuSK antibodies. (See Table 3)
**209115, 06/10/2022**	**Rucaparib (1)**	Oncology	BRCA	Indications and Usage, Dosage and Administration, Adverse Reactions, Clinical Studies	**1 INDICATIONS AND USAGE**<br>**1.2 Metastatic Castration-Resistant Prostate Cancer with BRCA Mutations**<br>Rubraca is indicated for the treatment of adult patients with a deleterious BRCA mutation (germline and/or somatic) associated metastatic castration-resistant prostate cancer (mCRPC) who have been treated with androgen receptordirected therapy and a taxane-based chemotherapy. Select patients for therapy based on an FDA-approved companion diagnostic for Rubraca [see Dosage and Administration (2.1)].<br>This indication is approved under accelerated approval based on objective response rate and duration of response [see Clinical Studies (14.2)]. Continued approval for this indication may be contingent upon verification and description of clinical benefit in confirmatory trials.<br><br>**2 DOSAGE AND ADMINISTRATION**<br>**2.1 Patient Selection**<br>Treatment of BRCA-mutated mCRPC after Androgen Receptor-directed Therapy and Chemotherapy<br>Select patients for the treatment of mCRPC with Rubraca based on the presence of a deleterious BRCA mutation (germline and/or somatic) in plasma specimens [see Clinical Studies (14.2)]. A negative result from a plasma specimen does not mean that the patient's tumor is negative for BRCA mutations. Should the plasma specimen have a negative result, consider performing further genomic testing using tumor specimens as clinically indicated.<br>Information on the FDA-approved tests for the detection of a BRCA mutation in patients with ovarian cancer or with prostate cancer is available at: http://www.fda.gov/CompanionDiagnostics.<br><br>**6 ADVERSE REACTIONS**<br>**6.1 Clinical Trials Experience**<br>Treatment of BRCA-mutated mCRPC after Androgen Receptor-directed Therapy and Chemotherapy<br>The safety of Rubraca 600 mg twice daily was evaluated in a single arm trial (TRITON2) [see Clinical Studies (14.2)]. TRITON2 enrolled 209 patients with HRD-positive mCRPC, including 115 with BRCA-mutated mCRPC. Among the patients with BRCA-mutated mCRPC, the median duration of Rubraca treatment was 6.5 months (range 0.5 to 26.7). (…)<br>Tables 4 and 5 summarize the adverse reactions and laboratory abnormalities, respectively, in patients with BRCA-mutated mCRPC in TRITON2. (See Tables 4 and 5) (…)<br><br>**14 CLINICAL STUDIES**<br>**14.1 Ovarian Cancer**<br>Maintenance Treatment of Recurrent Ovarian Cancer<br>(…) Tumor tissue samples were tested using a clinical trial assay (CTA) (N=564), and the FoundationFocus™ CDx BRCA LOH test (n=518). Of the samples evaluated with both tests, homologous recombination deficiency (HRD) positive status (as defined by the presence of a deleterious BRCA mutation or high genomic loss of heterozygosity) was confirmed by the FoundationFocus™ CDx BRCA LOH test for 94% (313/332) of HRD-positive patients determined by the CTA; and of these, tumor BRCA (tBRCA) mutant status was confirmed by the FoundationFocus™ CDx BRCA LOH test for 99% (177/178) of tBRCA-positive patients determined by the CTA. Blood samples for 94% (186/196) of the tBRCA patients were evaluated using a central blood germline BRCA test. Based on these results, 70% (130/186) of the tBRCA patients had a germline BRCA mutation and 30% (56/186) had a somatic BRCA mutation.<br>ARIEL3 demonstrated a statistically significant improvement in PFS for patients randomized to Rubraca as compared with placebo in all patients, and in the HRD and tBRCA subgroups. Results from a blinded independent radiology review were consistent. At the time of the analysis of PFS, overall survival (OS) data were not mature (with 22% of events). (See Table 6, Figures 1, 2, and 3)<br>**14.2 Metastatic Castration-Resistant Prostate Cancer with BRCA mutations**<br>The efficacy of Rubraca was investigated in TRITON2 (NCT02952534), an ongoing multi-center, single arm clinical trial in patients with BRCA-mutated mCRPC who had been treated with androgen receptor-directed therapy and taxane-based chemotherapy. There were 115 patients with either germline or somatic BRCA mutations enrolled in TRITON2, of whom 62 patients had measurable disease at baseline by independent radiology review (IRR). (…)<br>All 62 patients had a deleterious somatic or germline BRCA mutation detected from either central plasma (26%), central tissue (32%), or local (42%) testing. Of the 62 patients, 66% had a somatic BRCA mutation, 34% had a germline BRCA mutation, 85% had a BRCA2 mutation, and 15% had a BRCA1 mutation.<br>The major efficacy outcomes of the study were confirmed ORR by IRR using modified RECIST v1.1/PCWG3 criteria and DOR. Efficacy results of TRITON2 are provided in Table 7. The ORR by IRR was similar in patients with germline versus somatic BRCA mutation. (See Table 7)
**209115, 10/08/2020**	**Rucaparib (2)**	Oncology	CYP2D6	Clinical Pharmacology	**12 CLINICAL PHARMACOLOGY**<br>**12.3 Pharmacokinetics**<br>*Specific Populations*

* Therapeutic areas do not necessarily reflect the CDER review division.
† Representative biomarkers are listed based on standard nomenclature as per the Human Genome Organization (HUGO) symbol and/or simplified descriptors using other common conventions. Listed biomarkers do not necessarily reflect the terminology used in labeling. The term "Nonspecific" is provided when labeling does not explicitly identify the specific biomarker(s) or when the biomarker is represented by a molecular phenotype or gene signature, and in some cases the biomarker was inferred based on the labeling language.
‡ Referenced figures and tables may be found in the labeling available at Drugs@FDA.
Blue text represents the most recent addtions and/or changes since last posted version.

NDA/ANDA/BLA Number, Label Version Date	Drug	Therapeutic Area*	Biomarker†	Labeling Sections	Labeling Text‡
					CYP Enzyme Polymorphism<br>Based on population pharmacokinetic analyses, steady-state concentrations following rucaparib 600 mg twice daily did not differ significantly across CYP2D6 or CYP1A2 genotype subgroups.
**209115, 05/15/2020**	**Rucaparib (3)**	Oncology	CYP1A2	Clinical Pharmacology	**12 CLINICAL PHARMACOLOGY**<br>**12.3 Pharmacokinetics**<br>*Specific Populations*<br>*CYP Enzyme Polymorphism*<br>Based on population pharmacokinetic analyses, steady-state concentrations following rucaparib 600 mg twice daily did not differ significantly across CYP2D6 or CYP1A2 genotype subgroups.
**209115, 05/15/2020**	**Rucaparib (4)**	Oncology	BRCA, Loss of Heterozygosity (Homologous Recombination Deficiency)	Warnings and Precautions, Adverse Reactions, Clinical Studies	**5 WARNINGS AND PRECAUTIONS**<br>**5.1 Myelodysplastic Syndrome/Acute Myeloid Leukemia**<br>Myelodysplastic Syndrome (MDS)/Acute Myeloid Leukemia (AML) occur in patients treated with Rubraca, and are potentially fatal adverse reactions. In 1146 treated patients [see Adverse Reactions (6.1)], MDS/AML occurred in 20 patients (1.7%), including those in long term follow-up. Of these, 8 occurred during treatment or during the 28 day safety follow-up (0.7%). The duration of Rubraca treatment prior to the diagnosis of MDS/AML ranged from 1 month to approximately 53 months. The cases were typical of secondary MDS/cancer therapy-related AML; in all cases, patients had received previous platinum-containing chemotherapy regimens and/or other DNA damaging agents.<br>In TRITON2, MDS/AML was not observed in patients with mCRPC (n=209) regardless of homologous recombination deficiency (HRD) mutation [see Adverse Reactions (6.1)].<br><br>**6 ADVERSE REACTIONS**<br>**6.1 Clinical Trials Experience**<br><u>Treatment of BRCA-mutated mCRPC after Androgen Receptor-directed Therapy and Chemotherapy</u><br>The safety of Rubraca 600 mg twice daily was evaluated in a single arm trial (TRITON2) [see Clinical Studies (14.2)]. TRITON2 enrolled 209 patients with HRD-positive mCRPC, including 115 with BRCA-mutated mCRPC. Among the patients with BRCA-mutated mCRPC, the median duration of Rubraca treatment was 6.5 months (range 0.5 to 26.7). (…)<br><br>**14 CLINICAL STUDIES**<br>**14.1 Maintenance Treatment of Recurrent Ovarian Cancer**<br>(…) Tumor tissue samples were tested using a clinical trial assay (CTA) (N=564), and the FoundationFocus™ CDx BRCA LOH test (n=518). Of the samples evaluated with both tests, homologous recombination deficiency (HRD) positive status (as defined by the presence of a deleterious BRCA mutation or high genomic loss of heterozygosity) was confirmed by the FoundationFocus™ CDx BRCA LOH test for 94% (313/332) of HRD-positive patients determined by the CTA; and of these, tumor BRCA (tBRCA) mutant status was confirmed by the FoundationFocus™ CDx BRCA LOH test for 99% (177/178) of tBRCA-positive patients determined by the CTA. Blood samples for 94% (186/196) of the tBRCA patients were evaluated using a central blood germline BRCA test. Based on these results, 70% (130/186) of the tBRCA patients had a germline BRCA mutation and 30% (56/186) had a somatic BRCA mutation.<br>ARIEL3 demonstrated a statistically significant improvement in PFS for patients randomized to Rubraca as compared with placebo in all patients, and in the HRD and tBRCA subgroups. Results from a blinded independent radiology review were consistent. At the time of the analysis of PFS, overall survival (OS) data were not mature (with 22% of events). (See Table 8 and Figure 2).
**761115, 02/03/2023**	**Sacituzumab Govitecan-hziy (1)**	Oncology	UGT1A1	Warnings and Precautions, Clinical Pharmacology	**5 WARNINGS AND PRECAUTIONS**<br>**5.5 Increased Risk of Adverse Reactions in Patients with Reduced UGT1A1 Activity**<br>Patients homozygous for the uridine diphosphate-glucuronosyl transferase 1A1 (UGT1A1)*28 allele are at increased risk for neutropenia, febrile neutropenia, and anemia; and may be at increased risk for other adverse reactions when treated with TRODELVY. The incidence of neutropenia and anemia was analyzed in 948 patients who received TRODELVY and had UGT1A1 genotype results. In patients homozygous for the UGT1A1 *28 allele (n=112), the incidence of Grade 3-4 neutropenia was 58%. In patients heterozygous for the UGT1A1*28 allele (n=420), the incidence of Grade 3-4 neutropenia was 49%. In patients homozygous for the wild-type allele (n=416), the incidence of Grade 3-4 neutropenia was 43% [see Clinical Pharmacology (12.5)]. In patients homozygous for the UGT1A1 *28 allele, the incidence of Grade 3-4 anemia was 21%. In patients heterozygous for the UGT1A1*28 allele, the incidence of Grade 3-4 anemia was 10%. In patients homozygous for the wild-type allele, the incidence of Grade 3-4 anemia was 9%.<br>The median time to first neutropenia including febrile neutropenia was 9 days in patients homozygous for the UGT1A1*28 allele, 15 days in patients heterozygous for the UGT1A1*28 allele, and 20 days in patients homozygous for the wild-type allele.<br>The median time to first anemia was 21 days in patients homozygous for the UGT1A1*28 allele, 25 days in patients heterozygous for the UGT1A1*28 allele, and 28 days in patients homozygous for the wild-type allele. Closely monitor patients with known reduced UGT1A1 activity for adverse reactions. Withhold or permanently discontinue TRODELVY based on clinical assessment of the onset, duration and severity of the observed adverse reactions in patients with evidence of acute early-onset or unusually severe adverse reactions, which may indicate reduced UGT1A1 enzyme activity [see Dosage and Administration (2.3)].<br><br>**12 CLINICAL PHARMACOLOGY**<br>**12.5 Pharmacogenomics**<br>SN-38 is metabolized via UGT1A1 [see Clinical Pharmacology (12.3)]. Genetic variants of the UGT1A1 gene such as the UGT1A1*28 allele lead to reduced UGT1A1 enzyme activity. Individuals who are homozygous or heterozygous for the UGT1A1*28 allele are at increased risk for neutropenia, febrile neutropenia, and anemia from TRODELVY compared to individuals who are wildtype (*1/*1) [see Warnings and Precautions (5.5)]. Approximately 20% of the Black or African

NDA/ANDA/BLA Number, Label Version Date	Drug	Therapeutic Area*	Biomarker†	Labeling Sections	Labeling Text‡
					American population, 10% of the White population, and 2% of the East Asian population are homozygous for the UGT1A1*28 allele (*28/*28). Approximately 40% of the Black or African American population, 50% of the White population, and 25% of the East Asian population are heterozygous for the UGT1A1*28 allele (*1/*28). Decreased function alleles other than UGT1A1*28 may be present in certain populations.
**761115, 02/03/2023**	Sacituzumab Govitecan-hziy (2)	Oncology	BRCA	Clinical Studies	**14 CLINICAL STUDIES**<br>**14.1 Locally Advanced or Metastatic Triple-Negative Breast Cancer**<br>*ASCENT*<br>Efficacy was evaluated in a multicenter, open-label, randomized study (ASCENT; NCT02574455) conducted in 529 patients with unresectable locally advanced or metastatic triple-negative breast cancer (mTNBC) who had relapsed after at least two prior chemotherapies for breast cancer (one of which could be in the neoadjuvant or adjuvant setting provided progression occurred within a 12 month period). All patients received previous taxane treatment in either the adjuvant, neoadjuvant, or advanced stage unless there was a contraindication or intolerance to taxanes during or at the end of the first taxane cycle. Magnetic resonance imaging (MRI) to determine brain metastases was required prior to enrollment for patients with known or suspected brain metastases. Patients with brain metastases were allowed to enroll up to a predefined maximum of 15% of patients in the ASCENT study. Patients with known Gilbert's disease or bone-only disease were excluded. (…)<br>(…) The median age of patients in the full population (n = 529) was 54 years (range: 27 to 82 years); 99.6% were female; 79% were White, 12% were Black/African American; and 81% of patients were < 65 years of age. All patients had an ECOG performance status of 0 (43%) or 1 (57%). Forty-two percent of patients had hepatic metastases, 9% were BRCA1/BRCA2 mutational status positive, and 70% were TNBC at diagnosis. Twelve percent had baseline brain metastases previously treated and stable (n=61; 32 on TRODELVY arm and 29 on single agent chemotherapy arm). (…)
**761115, 02/03/2023**	Sacituzumab Govitecan-hziy (3)	Oncology	ESR (Hormone Receptor)	Indications and Usage, Adverse Reactions, Use in Specific Populations, Clinical Studies	**1 INDICATIONS AND USAGE**<br>**1.1 Locally Advanced or Metastatic Breast Cancer**<br>• TRODELVY is indicated for the treatment of adult patients with unresectable locally advanced or metastatic triple-negative breast cancer (mTNBC) who have received two or more prior systemic therapies, at least one of them for metastatic disease.<br>• TRODELVY is indicated for the treatment of adult patients with unresectable locally advanced or metastatic hormone receptor (HR)-positive, human epidermal growth factor receptor 2 (HER2)-negative (IHC 0, IHC 1+ or IHC 2+/ISH–) breast cancer who have received endocrine-based therapy and at least two additional systemic therapies in the metastatic setting.<br><br>**6 ADVERSE REACTIONS**<br>**6.1 Clinical Trials Experience**<br>Because clinical trials are conducted under widely varying conditions, adverse reaction rates observed in the clinical trials of a drug cannot be directly compared to rates in the clinical trials of another drug and may not reflect the rates observed in clinical practice. The pooled safety population described in the Warnings and Precautions section reflect exposure to TRODELVY in 1063 patients from four studies, IMMU-132-01, ASCENT, TROPiCS-02, and TROPHY which included 366 patients with mTNBC, 322 patients with hormone receptor-positive (HR+)/human epidermal growth factor receptor 2-negative (HER2-) breast cancer, and 180 patients with mUC. TRODELVY was administered as an intravenous infusion once weekly on Days 1 and 8 of 21-day treatment cycles at doses of 10 mg/kg until disease progression or unacceptable toxicity. (…)<br>Locally Advanced or Metastatic HR-Positive, HER2-Negative Breast Cancer<br>*TROPiCS-02 Study*<br>The safety of TRODELVY was evaluated in a randomized, active-controlled, open-label, study (TROPiCS-02) in patients with unresectable locally advanced or metastatic HR-positive, HER2-negative breast cancer whose disease has progressed after the following in any setting: a CDK 4/6 inhibitor, endocrine therapy, and a taxane; patients received at least two prior chemotherapies in the metastatic setting (one of which could be in the neoadjuvant or adjuvant setting if progression occurred within 12 months). (See Tables 6 and 7) (…)<br><br>**8 USE IN SPECIFIC POPULATIONS**<br>**8.5 Geriatric Use**<br>Of the 366 patients with TNBC who were treated with TRODELVY, 19% of patients were 65 years and 3% were 75 years and older. No overall differences in safety and effectiveness were observed between patients ≥ 65 years of age and younger patients.<br>Of the 322 patients with HR+/HER2- breast cancer who were treated with TRODELVY, 26% of patients were 65 years and older and 6% were 75 years and older. No overall differences in effectiveness were observed between patients ≥ 65 years of age and younger patients. There was a higher discontinuation rate due to adverse reactions in patients aged 65 years or older (14%) compared with younger patients (3%).<br>Of the 180 patients with UC who were treated with TRODELVY, 59% of patients were 65 years and older and 27% were 75 years and older. No overall differences in effectiveness were observed between patients ≥ 65 years of age and younger patients. There was a higher discontinuation rate due to adverse reactions in patients aged 65 years or older (14%) compared with younger patients (8%).<br><br>**14 CLINICAL STUDIES**<br>**14.2 Locally Advanced or Metastatic HR-Positive, HER2-Negative Breast Cancer**<br>*TROPiCS-02 Study*<br>The efficacy of TRODELVY was evaluated in a multicenter, open label, randomized study (TROPiCS-02; NCT03901339) conducted in 543 patients with unresectable locally advanced or metastatic HR-positive, HER2-negative (IHC 0, IHC 1+ or IHC 2+/ISH–) breast cancer whose disease has progressed after the following in any setting: a CDK 4/6 inhibitor, endocrine therapy, and a taxane; patients received at least two prior chemotherapies in the metastatic setting (one of which could be in the neoadjuvant or adjuvant setting if recurrence occurred within 12 months). (…)
**761115, 02/03/2023**	Sacituzumab Govitecan-hziy (4)	Oncology	ERBB2 (HER2)	Indications and Usage, Adverse	**1 INDICATIONS AND USAGE**<br>**1.2 Locally Advanced or Metastatic Breast Cancer**

* Therapeutic areas do not necessarily reflect the CDER review division.
† Representative biomarkers are listed based on standard nomenclature as per the Human Genome Organization (HUGO) symbol and/or simplified descriptors using other common conventions. Listed biomarkers do not necessarily reflect the terminology used in labeling. The term "Nonspecific" is provided when labeling does not explicitly identify the specific biomarker(s) or when the biomarker is represented by a molecular phenotype or gene signature, and in some cases the biomarker was inferred based on the labeling language.
‡ Referenced figures and tables may be found in the labeling available at Drugs@FDA.
Blue text represents the most recent addtions and/or changes since last posted version.

NDA/ANDA/BLA Number, Label Version Date	Drug	Therapeutic Area*	Biomarker†	Labeling Sections	Labeling Text‡
				Reactions, Use in Specific Populations, Clinical Studies	• TRODELVY is indicated for the treatment of adult patients with unresectable locally advanced or metastatic triple-negative breast cancer (mTNBC) who have received two or more prior systemic therapies, at least one of them for metastatic disease.<br>• TRODELVY is indicated for the treatment of adult patients with unresectable locally advanced or metastatic hormone receptor (HR)-positive, human epidermal growth factor receptor 2 (HER2)-negative (IHC 0, IHC 1+ or IHC 2+/ISH–) breast cancer who have received endocrine-based therapy and at least two additional systemic therapies in the metastatic setting.<br><br>**6 ADVERSE REACTIONS**<br>**6.1 Clinical Trials Experience**<br>Because clinical trials are conducted under widely varying conditions, adverse reaction rates observed in the clinical trials of a drug cannot be directly compared to rates in the clinical trials of another drug and may not reflect the rates observed in clinical practice. The pooled safety population described in the Warnings and Precautions section reflect exposure to TRODELVY in 1063 patients from four studies, IMMU-132-01, ASCENT, TROPiCS-02, and TROPHY which included 366 patients with mTNBC, 322 patients with hormone receptor-positive (HR+)/human epidermal growth factor receptor 2-negative (HER2-) breast cancer, and 180 patients with mUC. TRODELVY was administered as an intravenous infusion once weekly on Days 1 and 8 of 21-day treatment cycles at doses of 10 mg/kg until disease progression or unacceptable toxicity. (…)<br>Locally Advanced or Metastatic HR-Positive, HER2-Negative Breast Cancer<br>*TROPiCS-02 Study*<br>The safety of TRODELVY was evaluated in a randomized, active-controlled, open-label, study (TROPiCS-02) in patients with unresectable locally advanced or metastatic HR-positive, HER2-negative breast cancer whose disease has progressed after the following in any setting: a CDK 4/6 inhibitor, endocrine therapy, and a taxane; patients received at least two prior chemotherapies in the metastatic setting (one of which could be in the neoadjuvant or adjuvant setting if progression occurred within 12 months). (See Tables 6 and 7) (…)<br><br>**8 USE IN SPECIFIC POPULATIONS**<br>**8.5 Geriatric Use**<br>Of the 366 patients with TNBC who were treated with TRODELVY, 19% of patients were 65 years and 3% were 75 years and older. No overall differences in safety and effectiveness were observed between patients ≥ 65 years of age and younger patients.<br>Of the 322 patients with HR+/HER2- breast cancer who were treated with TRODELVY, 26% of patients were 65 years and older and 6% were 75 years and older. No overall differences in effectiveness were observed between patients ≥ 65 years of age and younger patients. There was a higher discontinuation rate due to adverse reactions in patients aged 65 years or older (14%) compared with younger patients (3%).<br>Of the 180 patients with UC who were treated with TRODELVY, 59% of patients were 65 years and older and 27% were 75 years and older. No overall differences in effectiveness were observed between patients ≥ 65 years of age and younger patients. There was a higher discontinuation rate due to adverse reactions in patients aged 65 years or older (14%) compared with younger patients (8%).<br><br>**14 CLINICAL STUDIES**<br>**14.2 Locally Advanced or Metastatic HR-Positive, HER2-Negative Breast Cancer**<br>*TROPiCS-02 Study*<br>The efficacy of TRODELVY was evaluated in a multicenter, open label, randomized study (TROPiCS-02; NCT03901339) conducted in 543 patients with unresectable locally advanced or metastatic HR-positive, HER2-negative (IHC 0, IHC 1+ or IHC 2+/ISH–) breast cancer whose disease has progressed after the following in any setting: a CDK 4/6 inhibitor, endocrine therapy, and a taxane; patients received at least two prior chemotherapies in the metastatic setting (one of which could be in the neoadjuvant or adjuvant setting if recurrence occurred within 12 months). (…)
**020772, 05/25/2022**	Sacrosidase	Inborn Errors of Metabolism	Nonspecific (Congenital Sucrase-Isomaltase Deficiency)	Indications and Usage, Adverse Reactions, Clinical Pharmacology	**INDICATIONS AND USAGE**<br>Sucraid® (sacrosidase) Oral Solution is indicated as oral replacement therapy of the genetically determined sucrase deficiency, which is part of congenital sucrase-isomaltase deficiency (CSID).<br><br>**ADVERSE REACTIONS**<br>(…) Note: diarrhea and abdominal pain can be a part of the clinical presentation of the genetically determined sucrase deficiency, which is part of congenital sucrase-isomaltase deficiency (CSID). (…)<br><br>**CLINICAL PHARMACOLOGY**<br>Congenital sucrase-isomaltase deficiency (CSID) is a chronic, autosomal recessive, inherited, phenotypically heterogeneous disease with very variable enzyme activity. CSID is usually characterized by a complete or almost complete lack of endogenous sucrase activity, a very marked reduction in isomaltase activity, a moderate decrease in maltase activity, and normal lactase levels. (…)<br>GENERAL<br>Although Sucraid provides replacement therapy for the deficient sucrase, it does not provide specific replacement therapy for the deficient isomaltase. Therefore, restricting starch in the diet may still be necessary to reduce symptoms as much as possible. The need for dietary starch restriction for patients using Sucraid should be evaluated in each patient. (…)
**761149, 08/14/2020**	Satralizumab-mwge	Neurology	AQP4	Indications and Usage, Adverse Reactions, Clinical Studies	**1 INDICATIONS AND USAGE**<br>ENSPRYNG is indicated for the treatment of neuromyelitis optica spectrum disorder (NMOSD) in adult patients who are anti-aquaporin-4 (AQP4) antibody positive.<br><br>**6 ADVERSE REACTIONS**

NDA/ANDA/BLA Number, Label Version Date	Drug	Therapeutic Area*	Biomarker†	Labeling Sections	Labeling Text‡
					**6.1 Clinical Trials Experience**<br>The safety of ENSPRYNG was evaluated in two randomized, placebo-controlled clinical trials [Study 1 evaluated ENSPRYNG without concurrent immunosuppressive therapy (IST) and Study 2 evaluated ENSPRYNG with concurrent IST], which included 41 anti-AQP4 seropositive patients treated with ENSPRYNG in Study 1 and 26 anti-AQP4 seropositive patients treated with ENSPRYNG in Study 2 [see Clinical Studies (14)]. In the double-blind, controlled period, the median exposure time on ENSPRYNG treatment was approximately 2 years in Study 1 and approximately 3 years in Study 2. The median exposure time on placebo treatment was approximately 1 year in both Study 1 and Study 2. (…)<br><br>**14 CLINICAL STUDIES**<br>The efficacy of ENSPRYNG for the treatment of NMOSD in adult patients was established in two studies. Study 1 was a randomized (2:1), placebo-controlled trial in 95 patients without concurrent IST (Study 1, NCT02073279) in which 64 patients were anti-AQP4 antibody positive and 31 patients were anti-AQP4 antibody negative. Study 2 was a randomized (1:1), placebo-controlled trial in 76 adult patients with concurrent IST (Study 2, NCT02028884). Of these, 52 adult patients were anti-AQP4 antibody positive and 24 adult patients were anti-AQP4 antibody negative. (…)<br>In Study 1, 41 anti-AQP4 antibody positive adult patients were randomized to and received ENSPRYNG and 23 received placebo. Females accounted for 76% of the ENSPRYNG group and 96% of the placebo group. The remaining baseline demographic characteristics were balanced between the treatment groups. The mean age was 44 years. Fifty percent were White, 22% were Black or African-American, and 20% were Asian. The mean EDSS score was 3.8.<br>In Study 2, 26 anti-AQP4 antibody positive adult patients were randomized to and received ENSPRYNG and 26 received placebo. All patients were receiving either concurrent azathioprine (42%), oral corticosteroids (52%), or mycophenolate mofetil (6%) during the trial. The baseline demographic and disease characteristics were balanced between the treatment groups. Females accounted for 100% of the study population. Forty-six percent of patients were White and 52% were Asian. The mean age was 46 years. The mean EDSS score was 4.0.<br>All potential relapses were adjudicated by a blinded Clinical Endpoint Committee (CEC). The primary efficacy endpoint for both studies was the time to the first CEC-confirmed relapse.<br>In Study 1, the time to the first CEC-confirmed relapse was significantly longer in ENSPRYNGtreated patients compared to patients who received placebo (risk reduction 55%; hazard ratio 0.45; p = 0.0184). In the anti-AQP4 antibody positive population, there was a 74% risk reduction; hazard ratio 0.26; p = 0.0014 (Table 5; Figure 1). There was no evidence of a benefit in the anti-AQP4 antibody negative patients.<br>In Study 2, the time to the first CEC-confirmed relapse was significantly longer in patients treated with ENSPRYNG compared to patients who received placebo (risk reduction 62%; hazard ratio 0.38; p = 0.0184). In the anti-AQP4 antibody positive population, there was a 78% risk reduction; hazard ratio 0.22; p = 0.0143 (Table 5; Figure 2). There was no evidence of a benefit in the anti-AQP4 antibody negative patients. (See Table 5 and Figures 1 and 2)
**213246, 05/29/2024**	**Selpercatinib**	Oncology	RET	Indications and Usage, Dosage and Administration, Adverse Reactions, Use in Specific Populations, Clinical Studies	**1 INDICATIONS AND USAGE**<br>**1.1 RET Fusion-Positive Non-Small Cell Lung Cancer**<br>RETEVMO® is indicated for the treatment of adult patients with locally advanced or metastatic non-small cell lung cancer (NSCLC) with a rearranged during transfection (RET) gene fusion, as detected by an FDA-approved test.<br>**1.2 RET-Mutant Medullary Thyroid Cancer**<br>RETEVMO is indicated for the treatment of adult and pediatric patients 2 years of age and older with advanced or metastatic medullary thyroid cancer (MTC) with a RET mutation, as detected by an FDA-approved test, who require systemic therapy.<br>This indication is approved under accelerated approval based on overall response rate and duration of response [see Clinical Studies (14.2)]. Continued approval for this indication may be contingent upon verification and description of clinical benefit in confirmatory trial(s)<br>**1.3 RET Fusion-Positive Thyroid Cancer**<br>RETEVMO is indicated for the treatment of adult and pediatric patients 2 years of age and older with advanced or metastatic thyroid cancer with a RET gene fusion, as detected by an FDA-approved test, who require systemic therapy and who are radioactive iodine-refractory (if radioactive iodine is appropriate).<br>This indication is approved under accelerated approval based on overall response rate and duration of response [see Clinical Studies (14.3)]. Continued approval for this indication may be contingent upon verification and description of clinical benefit in confirmatory trial(s).<br>**1.4 Other RET Fusion-Positive Solid Tumors**<br>RETEVMO is indicated for the treatment of adult and pediatric patients 2 years of age and older with locally advanced or metastatic solid tumors with a RET gene fusion, as detected by an FDA-approved test, that have progressed on or following prior systemic treatment or who have no satisfactory alternative treatment options.<br>This indication is approved under accelerated approval based on overall response rate and duration of response [see Clinical Studies (14.4)]. Continued approval for this indication may be contingent upon verification and description of clinical benefit in confirmatory trial(s).<br><br>**2 DOSAGE AND ADMINISTRATION**<br>**2.1 Patient Selection**<br>Select patients for treatment with RETEVMO based on the presence of a RET gene fusion (NSCLC, thyroid cancer, or other solid tumors) or specific RET gene mutation (MTC) in tumor specimens [see Clinical Studies (14)]. Information on FDA-approved test(s) for the detection of RET gene fusions and RET gene mutations is available at: http://www.fda.gov/CompanionDiagnostics. An FDA-approved companion diagnostic test for the detection of RET gene fusions and RET gene mutations in plasma is not available.<br><br>**6 ADVERSE REACTIONS**<br>LIBRETTO-121<br>The safety population described below reflects exposure to RETEVMO as a single agent at 92 mg/m2 orally twice daily evaluated in 27 patients with advanced solid tumors harboring an activating RET alteration in LIBRETTO-121 [see Clinical Studies (14)]. Among the 27 pediatric and adolescent patients who received RETEVMO, 81% were exposed for 6 months or longer and 59% were exposed for greater than one year. (…)

* Therapeutic areas do not necessarily reflect the CDER review division.
† Representative biomarkers are listed based on standard nomenclature as per the Human Genome Organization (HUGO) symbol and/or simplified descriptors using other common conventions. Listed biomarkers do not necessarily reflect the terminology used in labeling. The term “Nonspecific” is provided when labeling does not explicitly identify the specific biomarker(s) or when the biomarker is represented by a molecular phenotype or gene signature, and in some cases the biomarker was inferred based on the labeling language.
‡ Referenced figures and tables may be found in the labeling available at Drugs@FDA.
Blue text represents the most recent addtions and/or changes since last posted version.

NDA/ANDA/BLA Number, Label Version Date	Drug	Therapeutic Area*	Biomarker†	Labeling Sections	Labeling Text‡
					**8 USE IN SPECIFIC POPULATIONS**<br>**8.4 Pediatric Use**<br>The safety and effectiveness of RETEVMO have been established in pediatric patients 2 years of age and older for the treatment of:<br>• advanced or metastatic medullary thyroid cancer (MTC) with a RET mutation who require systemic therapy<br>advanced or metastatic thyroid cancer with a RET gene fusion who require systemic therapy and are radioactive iodine-refractory (if radioactive iodine is appropriate)<br>locally advanced or metastatic solid tumors with a RET gene fusion that have progressed on or following prior systemic treatment or who have no satisfactory alternative treatment options<br><br>**14 CLINICAL STUDIES**<br>**14.1 RET Fusion-Positive Non-Small Cell Lung Cancer**<br>The efficacy of RETEVMO was evaluated in patients with advanced RET fusion-positive NSCLC enrolled in a multicenter, open-label, multi-cohort clinical trial (LIBRETTO-001, NCT03157128). The study enrolled patients with advanced or metastatic RET fusion-positive NSCLC who had progressed on platinum-based chemotherapy and patients with locally advanced (stage III who were not candidates for surgical resection or definitive chemoradiation) or metastatic NSCLC without prior systemic therapy in separate cohorts. Identification of a RET gene alteration was prospectively determined in local laboratories using next generation sequencing (NGS), polymerase chain reaction (PCR), fluorescence in situ hybridization (FISH) or other local testing methods. Adult patients received RETEVMO 160 mg orally twice daily until unacceptable toxicity or disease progression; patients enrolled in the dose escalation phase were permitted to adjust their dose to 160 mg twice daily. The major efficacy outcome measures were confirmed overall response rate (ORR) and duration of response (DOR), as determined by a blinded independent review committee (BIRC) according to RECIST v1.1<br>RET Fusion-Positive NSCLC Previously Treated with Platinum Chemotherapy.<br>Efficacy was evaluated in 247 patients with RET fusion-positive NSCLC previously treated with platinum chemotherapy enrolled into a cohort of LIBRETTO-001. The median age was 61 years (range: 23 to 81); 57% were female; 44% were White, 48% were Asian, 4.9% were Black or African American, and 2.8% were Hispanic/Latino. ECOG performance status was 0-1 (97%) or 2 (3%) and 97% of patients had metastatic disease. Patients received a median of 2 prior systemic therapies (range 1–15); 58% had prior anti-PD-1/PD-L1 therapy. RET fusions were detected in 94% of patients using NGS (84.6% tumor samples; 9.3% blood or plasma samples), 4.0% using FISH, 1.6% using PCR and 0.4% by other local testing methods.<br>Efficacy results for previously treated RET fusion-positive NSCLC are summarized in Table 11**.**<br>For the 144 patients who received an anti-PD-1 or anti-PD-L1 therapy, either sequentially or concurrently with platinum-based chemotherapy, an exploratory subgroup analysis of ORR was 63% (95% CI: 54%, 70%) and the median DOR was 28.6 months (95% CI: 14.8, NE).<br>Among the 247 patients with previously treated RET fusion-positive NSCLC, 16 had measurable CNS metastases at baseline as assessed by BIRC. One patient received radiation therapy (RT) to the brain within 2 months prior to study entry. Responses in intracranial lesions were observed in 14 of these 16 patients; 39% of responders had an intracranial DOR of ≥ 12 months.<br>Treatment-naïve RET Fusion-Positive NSCLC<br>Efficacy was evaluated in 69 patients with treatment-naïve RET fusion-positive NSCLC enrolled into a cohort of LIBRETTO-001. The median age was 63 years (range 23 to 92); 62% were female; 70% were White, 19% were Asian, and 6% were Black or African American. ECOG performance status was 0-1 (94%) or 2 (6%) and 99% of patients had metastatic disease. RET fusions were detected in 91% of patients using NGS (60.9% tumor samples; 30.4% in blood), 7.2% using FISH and 1.4% using PCR.<br>Efficacy results for treatment naïve RET fusion-positive NSCLC are summarized in Table 12**.**<br>Among the 69 patients with treatment-naïve RET fusion-positive NSCLC, 5 had measurable CNS metastases at baseline as assessed by BIRC. Two patients received RT to the brain within 2 months prior to study entry. Responses in intracranial lesions were observed in 4 of these 5 patients; 38% of responders had an intracranial DOR of ≥ 12 months.<br>**14.2 RET-Mutant Medullary Thyroid Cancer**<br>LIBRETTO-001<br>The efficacy of RETEVMO was evaluated in patients with RET-mutant MTC enrolled in a multicenter, open-label, multi-cohort clinical trial (LIBRETTO-001, NCT03157128). The study enrolled patients with advanced or metastatic RET-mutant MTC who had been previously treated with cabozantinib or vandetanib (or both) and patients with advanced or metastatic RET-mutant MTC who were naïve to cabozantinib and vandetanib in separate cohorts.<br>RET-Mutant MTC Previously Treated with Cabozantinib or Vandetanib<br>Efficacy was evaluated in 55 patients with RET-mutant advanced MTC who had previously treated with cabozantinib or vandetanib enrolled into a cohort of LIBRETTO-001.<br>The median age was 57 years (range: 17 to 84); 66% were male; 89% were White, 1.8% were Black or African American; and 7% were Hispanic/Latino. ECOG performance status was 0-1 (95%) or 2 (5%) and 98% of patients had metastatic disease. Patients received a median of 2 prior systemic therapies (range 1 – 8). RET mutation status was detected in 82% of patients using NGS (78% tumor samples; 4% blood or plasma), 16% using PCR, and 2% using an unknown test. The protocol excluded patients with synonymous, frameshift or nonsense RET mutations; the specific mutations used to identify and enroll patients are described in Table 13. **14.3 RET Fusion-Positive Thyroid Cancer**<br>The efficacy of RETEVMO was evaluated in patients with advanced RET fusion-positive thyroid cancer enrolled in a multicenter, open-label, multi-cohort clinical trial (LIBRETTO-001, NCT03157128). Efficacy was evaluated in 27 patients with RET fusion-positive thyroid cancer who were radioactive iodine (RAI)-refractory (if RAI was an appropriate treatment option) and were systemic therapy naïve and patients with RET fusion-positive thyroid cancer who were RAI-refractory and had received sorafenib, lenvatinib, or both, in separate cohorts.

* Therapeutic areas do not necessarily reflect the CDER review division.
† Representative biomarkers are listed based on standard nomenclature as per the Human Genome Organization (HUGO) symbol and/or simplified descriptors using other common conventions. Listed biomarkers do not necessarily reflect the terminology used in labeling. The term “Nonspecific” is provided when labeling does not explicitly identify the specific biomarker(s) or when the biomarker is represented by a molecular phenotype or gene signature, and in some cases the biomarker was inferred based on the labeling language.
‡ Referenced figures and tables may be found in the labeling available at Drugs@FDA.
Blue text represents the most recent addtions and/or changes since last posted version.

NDA/ANDA/BLA Number, Label Version Date	Drug	Therapeutic Area*	Biomarker†	Labeling Sections	Labeling Text‡
					The median age was 54 years (range 20 to 88); 52% were male; 74% were White, 11% were Hispanic/Latino, 7.4% were Asian, and 3.7% were Black. ECOG performance status was 0-1 (89%) or 2 (11%). All (100%) patients had metastatic disease with primary tumor histologies including papillary thyroid cancer (78%), poorly differentiated thyroid cancer (11%), anaplastic thyroid cancer (7%) and Hurthle cell thyroid cancer (4%). Patients had received a median of 3 prior therapies (range 1–7). RET fusion-positive status was detected in 93% of patients using NGS tumor samples and in 7% using blood samples.<br>Efficacy results for RET fusion-positive thyroid cancer are summarized in Table 13. (See Table 13)<br>Cabozantinib and Vandetanib-naïve RET-Mutant MTC<br>Efficacy was evaluated in 88 patients with RET-mutant MTC who were cabozantinib and vandetanib treatment-naïve enrolled into a cohort of LIBRETTO-001. The median age was 58 years (range: 15 to 82) with two patients (2.3%) aged 12 to 16 years; 66% were male; and 86% were White, 4.5% were Asian, and 2.3% were Hispanic/Latino. ECOG performance status was 0-1 (97%) or 2 (3.4%). All patients (100%) had metastatic disease and 18% had received 1 or 2 prior systemic therapies (including 8% kinase inhibitors, 4.5% chemotherapy, 2.3% anti-PD1/PD-L1 therapy, and 1.1% radioactive iodine). RET mutation status was detected in 77.3% of patients using NGS (75.0% tumor samples; 2.3% blood samples), 18.2% using PCR, and 4.5% using an unknown test. The mutations used to identify and enroll patients are described in Table 10.<br>Efficacy results for cabozantinib and vandetanib-naïve RET-mutant MTC are summarized in Table 15.<br>LIBRETTO-121<br>The efficacy of RETEVMO was evaluated in pediatric and young adult patients with advanced RET-activated solid tumors enrolled in a multicenter, open-label, multi-cohort clinical trial (LIBRETTO-121, NCT03899792). Patients received selpercatinib 92 mg/m2 orally twice daily until disease progression, unacceptable toxicity, or other reason for treatment discontinuation. Tumor assessments were performed every 8 weeks for one year, then every 12 weeks; responses were assessed according to RECIST 1.1 per BIRC.<br>Efficacy was evaluated in 14 patients with RET-mutant MTC who were non-responsive to available therapies or had no standard systemic curative therapy available. The median age was 14 years (range 2 to 20); 64% were male; 71% were White, 14% were Black or African American; and 14% were Hispanic/Latino. Patients had metastatic (71%) or locally advanced (29%) disease; 43% had measurable disease at baseline; 21% had received prior systemic therapy. RET-mutant status was detected in 79% of patients using NGS tumor samples and in 21% using PCR.<br>Efficacy results for RET-mutant MTC in pediatric and young adult patients are summarized in Table 16.<br>**14.3 RET Fusion-Positive Thyroid Cancer**<br>LIBRETTO-001<br>The efficacy of RETEVMO was evaluated in patients with advanced RET fusion-positive thyroid cancer enrolled in a multicenter, open-label, multi-cohort clinical trial (LIBRETTO-001, NCT03157128). Efficacy was evaluated in 27 patients with RET fusion-positive thyroid cancer who were radioactive iodine (RAI)-refractory (if RAI was an appropriate treatment option) and were systemic therapy naïve and patients with RET fusion-positive thyroid cancer who were RAI-refractory and had received sorafenib, lenvatinib, or both, in separate cohorts.<br>The median age was 54 years (range 20 to 88); 52% were male; 74% were White, 7.4% were Asian, 3.7% were Black or African American; and 11% were Hispanic/Latino. ECOG performance status was 0-1 (89%) or 2 (11%). All (100%) patients had metastatic disease with primary tumor histologies including papillary thyroid cancer (78%), poorly differentiated thyroid cancer (11%), anaplastic thyroid cancer (7%) and Hurthle cell thyroid cancer (4%). Patients had received a median of 3 prior therapies (range 1–7). RET fusion-positive status was detected in 93% of patients using NGS tumor samples and in 7% using blood samples.<br>Efficacy results for RET fusion-positive thyroid cancer are summarized in Table 17.<br>LIBRETTO-121<br>The efficacy of RETEVMO was evaluated in pediatric and young adult patients with advanced RET-activated solid tumors enrolled in a multicenter, open-label, multi-cohort clinical trial (LIBRETTO-121, NCT03899792) [see Clinical Studies (14.2)].<br>Efficacy was evaluated in 10 patients with RET fusion-positive thyroid cancer who were non-responsive to available therapies or had no standard systemic curative therapy available. The median age was 13.5 years (range 12 to 20); 60% were male; 40% were White, 50% were Asian; and 30% were Hispanic/Latino. All (100%) patients had metastatic disease and papillary thyroid cancer histology; 40% had measurable disease at baseline; 30% had received prior systemic therapy. RET fusion-positive status was detected in 90% of patients using NGS tumor samples and in 10% using FISH. Efficacy results for RET fusion-positive thyroid cancer in pediatric and young adult patients are summarized in Table 18.<br>**14.4 Other RET Fusion-Positive Solid Tumors**<br>LIBRETTO-001<br>The efficacy of RETEVMO was evaluated in patients with locally advanced or metastatic RET fusion-positive solid tumors enrolled in a multicenter, open-label, multi-cohort clinical trial (LIBRETTO-001, NCT03157128). Efficacy was evaluated in 41 patients with RET fusion-positive tumors other than NSCLC and thyroid cancer with disease progression on or following prior systemic treatment or who had no satisfactory alternative treatment options.<br>The median age was 50 years (range 21 to 85), 54% were female, 68% were White, 24% were Asian, and 4.9% were Black; and 7% were Hispanic/Latino. ECOG performance status was 0-1 (95%) or 2 (5%) and 95% of patients had metastatic disease. Thirty-seven patients (90%) received prior systemic therapy (median 2 [range 0 – 9]; 32% received 3 or more). The most common cancers were pancreatic adenocarcinoma (27%), colorectal (24%), salivary (10%) and unknown primary (7%). RET fusion-positive status was detected in 97.6% of patients using NGS and 2.4% using FISH.<br>Efficacy results for RET fusion-positive solid tumors other than NSCLC and thyroid cancer are summarized in Table 19 and Table 20.<br>LIBRETTO-121<br>The efficacy of RETEVMO was evaluated in pediatric and young adult patients with advanced RET-activated solid tumors enrolled in a multicenter, open-label, multi-cohort clinical trial (LIBRETTO-121, NCT03899792) [see Clinical Studies (14.2)].<br>Efficacy was evaluated in one patient with locally advanced refractory RET-fusion positive malignant peripheral nerve sheath tumor who did not respond. Responses were observed in patients with RET fusion-positive thyroid cancer [see Clinical Studies (14.3)].

* Therapeutic areas do not necessarily reflect the CDER review division.
† Representative biomarkers are listed based on standard nomenclature as per the Human Genome Organization (HUGO) symbol and/or simplified descriptors using other common conventions. Listed biomarkers do not necessarily reflect the terminology used in labeling. The term “Nonspecific” is provided when labeling does not explicitly identify the specific biomarker(s) or when the biomarker is represented by a molecular phenotype or gene signature, and in some cases the biomarker was inferred based on the labeling language.
‡ Referenced figures and tables may be found in the labeling available at Drugs@FDA.
Blue text represents the most recent addtions and/or changes since last posted version.

Table of Pharmacogenomic Biomarkers in Drug Labeling
Last Updated: 06/2024

NDA/ANDA/BLA Number, Label Version Date	Drug	Therapeutic Area*	Biomarker†	Labeling Sections	Labeling Text‡
020478, 11/01/2022	Sevoflurane	Anesthesiology	CACNA1S, RYR1 (Genetic Susceptibility to Malignant Hyperthermia)	Contraindications, Warnings, Clinical Pharmacology	**CONTRAINDICATIONS**<br>• Known or suspected genetic susceptibility to malignant hyperthermia (see WARNINGS - Malignant Hyperthermia, CLINICAL PHARMACOLOGY - Pharmacogenomics).<br><br>**WARNINGS**<br>Malignant Hyperthermia<br>In susceptible individuals, volatile anesthetic agents, including sevoflurane, may trigger malignant hyperthermia, a skeletal muscle hypermetabolic state leading to high oxygen demand. Fatal outcomes of malignant hyperthermia have been reported. In clinical studies of ULTANE, 1 case of malignant hyperthermia was reported.<br>The risk of developing malignant hyperthermia increases with the concomitant administration of succinylcholine and volatile anesthetic agents. ULTANE can induce malignant hyperthermia in patients with known or suspected susceptibility based on genetic factors or family history, including those with certain inherited ryanodine receptor (RYR1) or dihydropyridine receptor (CACNA1S) variants (see CONTRAINDICATIONS, CLINICAL PHARMACOLOGY - Pharmacogenomics).<br>(…)<br><br>**CLINICAL PHARMACOLOGY**<br>**Pharmacogenomics**<br>RYR1 and CACNA1S are polymorphic genes, and multiple pathogenic variants have been associated with malignant hyperthermia susceptibility (MHS) in patients receiving volatile anesthetic agents, including sevoflurane. Case reports as well as ex-vivo studies have identified multiple variants in RYR1 and CACNA1S associated with MHS. Variant pathogenicity should be assessed based on prior clinical experience, functional studies, prevalence information, or other evidence (see CONTRAINDICATIONS, WARNINGS - Malignant Hyperthermia).
**213793, 06/16/2022**	Setmelanotide (1)	Endocrinology	LEPR	Indications and Usage, Dosage and Administration, Adverse Reactions, Use in Specific Populations, Clinical Studies	**1 INDICATIONS AND USAGE**<br>IMCIVREE is indicated for chronic weight management in adult and pediatric patients 6 years of age and older with monogenic or syndromic obesity due to:<br>• Pro-opiomelanocortin (POMC), proprotein convertase subtilisin/kexin type 1 (PCSK1), or leptin receptor (LEPR) deficiency as determined by an FDA-approved test demonstrating variants in POMC, PCSK1, or LEPR genes that are interpreted as pathogenic, likely pathogenic, or of uncertain significance (VUS) [see Dosage and Administration (2.1)]<br>• Bardet-Biedl syndrome (BBS) [see Dosage and Administration (2.2)]. <u>Limitations of Use:</u><br>IMCIVREE is not indicated for the treatment of patients with the following conditions as IMCIVREE would not be expected to be effective:<br>• Obesity due to suspected POMC, PCSK1, or LEPR deficiency with POMC, PCSK1, or LEPR variants classified as benign or likely benign<br>• Other types of obesity not related to POMC, PCSK1 or LEPR deficiency, including obesity associated with other genetic syndromes and general (polygenic) obesity<br><br>**2 DOSAGE AND ADMINISTRATION**<br>2.1 Patient Selection - POMC, PCSK1, or LEPR Deficiency<br>• Select patients for treatment with IMCIVREE who have genetically determined or suspected deficiency of POMC, PCSK1, or LEPR [see Clinical Studies (14)].<br>• Treat patients with variants in POMC, PCSK1, or LEPR genes that are interpreted as pathogenic, likely pathogenic, or of uncertain significance (VUS) in the clinical context of the patient [see Clinical Studies (14)].<br>• Information on an FDA-approved test for the detection of variants in the POMC, PCSK1, or LEPR is available at <u>http://www.fda.gov/CompanionDiagnostics</u>.<br><br>**6 ADVERSE REACTIONS**<br>**6.1 Clinical Trials Experience**<br>The safety of IMCIVREE was evaluated in two 52-week, open-label clinical studies of 27 patients with obesity due to POMC, PCSK1, or LEPR deficiency with POMC, PCSK1, or LEPR genes that are interpreted as pathogenic, likely pathogenic, or of uncertain significance [see Clinical Studies (14)]. (…)<br>**6.2 Immunogenicity**<br>(…) Approximately 61% of adult and pediatric patients with POMC- or LEPR-deficiency who received IMCIVREE (N=28) screened positive for antibodies to IMCIVREE, and 39% screened negative. The 61% of patients who screened positive for antibodies to IMCIVREE were inconclusive for antibodies to IMCIVREE in the confirmatory assay. There was no observation of a rapid decline in IMCIVREE concentrations to suggest the presence of anti-drug antibodies. (…)<br>Approximately 13% of adult and pediatric patients with LEPR-deficiency (3 patients) confirmed positive for antibodies to alpha-MSH that were classified as low-titer and non-persistent. Of these 3 patients (13%), 2 tested positive post- IMCIVREE treatment and 1 was positive pretreatment. None of the patients with POMC-deficiency were confirmed to have antibodies to alpha-MSH. (…)<br><br>**8 USE IN SPECIFIC POPULATIONS**<br>**8.4 Pediatric Use**<br>The safety and effectiveness of IMCIVREE have been established for chronic weight management in pediatric patients aged 6 years and older with obesity due to:<br>• POMC, PCSK1, or LEPR deficiency with variants in POMC, PCSK1, or LEPR genes that are interpreted as pathogenic, likely pathogenic, or of uncertain significance (VUS) [see Clinical Studies (14.1)]<br>• BBS [see Clinical Studies (14.2)]<br><br>**14 CLINICAL STUDIES**

* Therapeutic areas do not necessarily reflect the CDER review division.
† Representative biomarkers are listed based on standard nomenclature as per the Human Genome Organization (HUGO) symbol and/or simplified descriptors using other common conventions. Listed biomarkers do not necessarily reflect the terminology used in labeling. The term “Nonspecific” is provided when labeling does not explicitly identify the specific biomarker(s) or when the biomarker is represented by a molecular phenotype or gene signature, and in some cases the biomarker was inferred based on the labeling language.
‡ Referenced figures and tables may be found in the labeling available at Drugs@FDA.
Blue text represents the most recent addtions and/or changes since last posted version.

NDA/ANDA/BLA Number, Label Version Date	Drug	Therapeutic Area*	Biomarker†	Labeling Sections	Labeling Text‡
					The safety and efficacy of IMCIVREE for chronic weight management in patients with obesity due to POMC, PCSK1, and LEPR deficiency were assessed in 2 identically designed, 1-year, open-label studies, each with an 8-week, double-blind withdrawal period. Study 1 (NCT02896192) enrolled patients aged 6 years and above with obesity and genetically confirmed or suspected POMC or PCSK1 deficiency, and Study 2 (NCT03287960) enrolled patients aged 6 years and above with obesity and genetically confirmed or suspected LEPR deficiency. In both studies, the local genetic testing results were centrally confirmed using Sanger sequencing. The studies enrolled patients with homozygous or presumed compound heterozygous pathogenic, likely pathogenic variants, or VUS for either the POMC or PCSK1 genes (Study 1) or the LEPR gene (Study 2). Patients with double heterozygous variants in 2 different genes were not eligible for treatment with IMCIVREE. In both studies, adult patients had a body mass index (BMI) of ≥30 kg/m2 . Weight in pediatric patients was ≥95th percentile using growth chart assessments.<br>Effect of IMCIVREE on Body Weight<br>In Study 1, 80% of patients with obesity due to POMC or PCSK1 deficiency met the primary endpoint, achieving a ≥10% weight loss after 1 year of treatment with IMCIVREE. In Study 2, 46% of patients with obesity due to LEPR deficiency achieved a ≥10% weight loss after 1 year of treatment with IMCIVREE (Table 2). (See Tables 2,3, and 4)
**213793, 06/16/2022**	**Setmelanotide (2)**	Endocrinology	PCSK1	Indications and Usage, Dosage and Administration, Adverse Reactions, Use in Specific Populations, Clinical Studies	**1 INDICATIONS AND USAGE**<br>IMCIVREE is indicated for chronic weight management in adult and pediatric patients 6 years of age and older with monogenic or syndromic obesity due to:<br>• Pro-opiomelanocortin (POMC), proprotein convertase subtilisin/kexin type 1 (PCSK1), or leptin receptor (LEPR) deficiency as determined by an FDA-approved test demonstrating variants in POMC, PCSK1, or LEPR genes that are interpreted as pathogenic, likely pathogenic, or of uncertain significance (VUS) [see Dosage and Administration (2.1)]<br>• Bardet-Biedl syndrome (BBS) [see Dosage and Administration (2.2)]. Limitations of Use:<br>IMCIVREE is not indicated for the treatment of patients with the following conditions as IMCIVREE would not be expected to be effective:<br>• Obesity due to suspected POMC, PCSK1, or LEPR deficiency with POMC, PCSK1, or LEPR variants classified as benign or likely benign<br>• Other types of obesity not related to POMC, PCSK1 or LEPR deficiency, including obesity associated with other genetic syndromes and general (polygenic) obesity<br><br>**2 DOSAGE AND ADMINISTRATION**<br>2.1 Patient Selection - POMC, PCSK1, or LEPR Deficiency<br>• Select patients for treatment with IMCIVREE who have genetically determined or suspected deficiency of POMC, PCSK1, or LEPR [see Clinical Studies (14)].<br>• Treat patients with variants in POMC, PCSK1, or LEPR genes that are interpreted as pathogenic, likely pathogenic, or of uncertain significance (VUS) in the clinical context of the patient [see Clinical Studies (14)].<br>• Information on an FDA-approved test for the detection of variants in the POMC, PCSK1, or LEPR is available at http://www.fda.gov/CompanionDiagnostics.<br><br>**6 ADVERSE REACTIONS**<br>**6.1 Clinical Trials Experience**<br>The safety of IMCIVREE was evaluated in two 52-week, open-label clinical studies of 27 patients with obesity due to POMC, PCSK1, or LEPR deficiency with POMC, PCSK1, or LEPR genes that are interpreted as pathogenic, likely pathogenic, or of uncertain significance [see Clinical Studies (14)]. (…)<br><br>**8 USE IN SPECIFIC POPULATIONS**<br>**8.4 Pediatric Use**<br>The safety and effectiveness of IMCIVREE have been established for chronic weight management in pediatric patients aged 6 years and older with obesity due to:<br>• POMC, PCSK1, or LEPR deficiency with variants in POMC, PCSK1, or LEPR genes that are interpreted as pathogenic, likely pathogenic, or of uncertain significance (VUS) [see Clinical Studies (14.1)]<br>• BBS [see Clinical Studies (14.2)].<br><br>**14 CLINICAL STUDIES**<br>The safety and efficacy of IMCIVREE for chronic weight management in patients with obesity due to POMC, PCSK1, and LEPR deficiency were assessed in 2 identically designed, 1-year, open-label studies, each with an 8-week, double-blind withdrawal period. Study 1 (NCT02896192) enrolled patients aged 6 years and above with obesity and genetically confirmed or suspected POMC or PCSK1 deficiency, and Study 2 (NCT03287960) enrolled patients aged 6 years and above with obesity and genetically confirmed or suspected LEPR deficiency. In both studies, the local genetic testing results were centrally confirmed using Sanger sequencing. The studies enrolled patients with homozygous or presumed compound heterozygous pathogenic, likely pathogenic variants, or VUS for either the POMC or PCSK1 genes (Study 1) or the LEPR gene (Study 2). Patients with double heterozygous variants in 2 different genes were not eligible for treatment with IMCIVREE. In both studies, adult patients had a body mass index (BMI) of ≥30 kg/m2 . Weight in pediatric patients was ≥95th percentile using growth chart assessments.<br>Effect of IMCIVREE on Body Weight<br>In Study 1, 80% of patients with obesity due to POMC or PCSK1 deficiency met the primary endpoint, achieving a ≥10% weight loss after 1 year of treatment with IMCIVREE. In Study 2, 46% of patients with obesity due to LEPR deficiency achieved a ≥10% weight loss after 1 year of treatment with IMCIVREE (Table 2). (See Tables 2,3, and 4)
**213793, 06/16/2022**	**Setmelanotide (3)**	Endocrinology	POMC	Indications and Usage, Dosage and	**1 INDICATIONS AND USAGE**<br>IMCIVREE is indicated for chronic weight management in adult and pediatric patients 6 years of age and older with monogenic or syndromic obesity due to:

* Therapeutic areas do not necessarily reflect the CDER review division.
† Representative biomarkers are listed based on standard nomenclature as per the Human Genome Organization (HUGO) symbol and/or simplified descriptors using other common conventions. Listed biomarkers do not necessarily reflect the terminology used in labeling. The term “Nonspecific” is provided when labeling does not explicitly identify the specific biomarker(s) or when the biomarker is represented by a molecular phenotype or gene signature, and in some cases the biomarker was inferred based on the labeling language.
‡ Referenced figures and tables may be found in the labeling available at Drugs@FDA.
Blue text represents the most recent addtions and/or changes since last posted version.

NDA/ANDA/BLA Number, Label Version Date	Drug	Therapeutic Area*	Biomarker†	Labeling Sections	Labeling Text‡
				Administration, Adverse Reactions, Use in Specific Populations, Clinical Studies	• Pro-opiomelanocortin (POMC), proprotein convertase subtilisin/kexin type 1 (PCSK1), or leptin receptor (LEPR) deficiency as determined by an FDA-approved test demonstrating variants in POMC, PCSK1, or LEPR genes that are interpreted as pathogenic, likely pathogenic, or of uncertain significance (VUS) [see Dosage and Administration (2.1)]<br>• Bardet-Biedl syndrome (BBS) [see Dosage and Administration (2.2)]. Limitations of Use:<br>IMCIVREE is not indicated for the treatment of patients with the following conditions as IMCIVREE would not be expected to be effective:<br>• Obesity due to suspected POMC, PCSK1, or LEPR deficiency with POMC, PCSK1, or LEPR variants classified as benign or likely benign<br>• Other types of obesity not related to POMC, PCSK1 or LEPR deficiency, including obesity associated with other genetic syndromes and general (polygenic) obesity<br><br>**2 DOSAGE AND ADMINISTRATION**<br>2.1 Patient Selection - POMC, PCSK1, or LEPR Deficiency<br>• Select patients for treatment with IMCIVREE who have genetically determined or suspected deficiency of POMC, PCSK1, or LEPR [see Clinical Studies (14)].<br>• Treat patients with variants in POMC, PCSK1, or LEPR genes that are interpreted as pathogenic, likely pathogenic, or of uncertain significance (VUS) in the clinical context of the patient [see Clinical Studies (14)].<br>• Information on an FDA-approved test for the detection of variants in the POMC, PCSK1, or LEPR is available at http://www.fda.gov/CompanionDiagnostics.<br><br>**6 ADVERSE REACTIONS**<br>**6.1 Clinical Trials Experience**<br>The safety of IMCIVREE was evaluated in two 52-week, open-label clinical studies of 27 patients with obesity due to POMC, PCSK1, or LEPR deficiency with POMC, PCSK1, or LEPR genes that are interpreted as pathogenic, likely pathogenic, or of uncertain significance [see Clinical Studies (14)]. (…)<br>**6.2 Immunogenicity**<br>(…) Approximately 61% of adult and pediatric patients with POMC- or LEPR-deficiency who received IMCIVREE (N=28) screened positive for antibodies to IMCIVREE, and 39% screened negative. The 61% of patients who screened positive for antibodies to IMCIVREE were inconclusive for antibodies to IMCIVREE in the confirmatory assay. There was no observation of a rapid decline in IMCIVREE concentrations to suggest the presence of anti-drug antibodies. (…)<br>Approximately 13% of adult and pediatric patients with LEPR-deficiency (3 patients) confirmed positive for antibodies to alpha-MSH that were classified as low-titer and non-persistent. Of these 3 patients (13%), 2 tested positive post- IMCIVREE treatment and 1 was positive pretreatment. None of the patients with POMC-deficiency were confirmed to have antibodies to alpha-MSH. (…)<br><br>**8 USE IN SPECIFIC POPULATIONS**<br>**8.4 Pediatric Use**<br>The safety and effectiveness of IMCIVREE have been established for chronic weight management in pediatric patients aged 6 years and older with obesity due to:<br>• POMC, PCSK1, or LEPR deficiency with variants in POMC, PCSK1, or LEPR genes that are interpreted as pathogenic, likely pathogenic, or of uncertain significance (VUS) [see Clinical Studies (14.1)]<br>• BBS [see Clinical Studies (14.2)]<br><br>**14 CLINICAL STUDIES**<br>The safety and efficacy of IMCIVREE for chronic weight management in patients with obesity due to POMC, PCSK1, and LEPR deficiency were assessed in 2 identically designed, 1-year, open-label studies, each with an 8-week, double-blind withdrawal period. Study 1 (NCT02896192) enrolled patients aged 6 years and above with obesity and genetically confirmed or suspected POMC or PCSK1 deficiency, and Study 2 (NCT03287960) enrolled patients aged 6 years and above with obesity and genetically confirmed or suspected LEPR deficiency. In both studies, the local genetic testing results were centrally confirmed using Sanger sequencing. The studies enrolled patients with homozygous or presumed compound heterozygous pathogenic, likely pathogenic variants, or VUS for either the POMC or PCSK1 genes (Study 1) or the LEPR gene (Study 2). Patients with double heterozygous variants in 2 different genes were not eligible for treatment with IMCIVREE. In both studies, adult patients had a body mass index (BMI) of ≥30 kg/m2 . Weight in pediatric patients was ≥95th percentile using growth chart assessments.<br>Effect of IMCIVREE on Body Weight<br>In Study 1, 80% of patients with obesity due to POMC or PCSK1 deficiency met the primary endpoint, achieving a ≥10% weight loss after 1 year of treatment with IMCIVREE. In Study 2, 46% of patients with obesity due to LEPR deficiency achieved a ≥10% weight loss after 1 year of treatment with IMCIVREE (Table 2). (See Tables 2,3, and 4)
**205123, 11/09/2017**	Simeprevir	Infectious Diseases	IFNL3 (IL28B)	Clinical Pharmacology, Clinical Studies	**12 CLINICAL PHARMACOLOGY**<br>**12.5 Pharmacogenomics**<br>A genetic variant near the gene encoding interferon-lambda-3 (IL28B rs12979860, a C [cytosine] to T [thymine] substitution) is a strong predictor of response to Peg-IFN-alfa and RBV (PR). In the Phase 3 trials, IL28B genotype was a stratification factor. Overall, SVR rates were lower in subjects with the CT and TT genotypes compared to those with the CC genotype (Tables 12 and 13). Among both treatment-naïve subjects and those who experienced previous treatment failures, subjects of all IL28B genotypes had the highest SVR rates with OLYSIO-containing regimens. (See Table 12 and 13)<br><br>**14 CLINICAL STUDIES**<br>**14.2 OLYSIO in Combination with Sofosbuvir**<br>*Adult Subjects with HCV Genotype 1 Infection*

* Therapeutic areas do not necessarily reflect the CDER review division.
† Representative biomarkers are listed based on standard nomenclature as per the Human Genome Organization (HUGO) symbol and/or simplified descriptors using other common conventions. Listed biomarkers do not necessarily reflect the terminology used in labeling. The term “Nonspecific” is provided when labeling does not explicitly identify the specific biomarker(s) or when the biomarker is represented by a molecular phenotype or gene signature, and in some cases the biomarker was inferred based on the labeling language.
‡ Referenced figures and tables may be found in the labeling available at Drugs@FDA.
Blue text represents the most recent addtions and/or changes since last posted version.

NDA/ANDA/BLA Number, Label Version Date	Drug	Therapeutic Area*	Biomarker†	Labeling Sections	Labeling Text‡
					(…) These 59 subjects had a median age of 57 years (range 27 to 68 years; with 2% above 65 years); 53% were male; 76% were White, and 24% Black or African American; 46% had a BMI greater than or equal to 30 kg/m2 ; the median baseline HCV RNA level was 6.75 log10 IU/mL; 19%, 31% and 22% had METAVIR fibrosis scores F0-F1, F2 and F3, respectively, and 29% had METAVIR fibrosis score F4 (cirrhosis); 75% had HCV genotype 1a of which 41% carried Q80K at baseline, and 25% had HCV genotype 1b; 14% had IL28B CC genotype, 64% IL28B CT genotype, and 22% IL28B TT genotype; 75% were prior null responders to Peg-IFN-alfa and RBV, and 25% were treatment-naïve.<br>OPTIMIST-1 was an open-label, randomized Phase 3 trial in HCV genotype 1-infected subjects without cirrhosis who were treatment-naïve or treatment-experienced (including prior relapsers, non-responders and IFN-intolerant subjects). Subjects were randomized to treatment arms of different durations. One hundred fifty-five subjects received 12 weeks of OLYSIO with sofosbuvir. The 155 subjects without cirrhosis receiving 12 weeks of OLYSIO with sofosbuvir had a median age of 56 years (range 19 to 70 years; with 7% above 65 years); 53% were male; 78% were White, 20% Black or African American, and 16% Hispanic; 37% had a BMI ≥ 30 kg/m2 ; the median baseline HCV RNA level was 6.83 log10 IU/mL; 75% had HCV genotype 1a of which 40% had Q80K polymorphism at baseline, and 25% had HCV genotype 1b; 28% had IL28B CC genotype, 55% IL28B CT genotype, and 17% IL28B TT genotype; 74% were treatment-naïve and 26% were treatment-experienced. (…)<br>(…) Among subjects without cirrhosis in OPTIMIST-1 who received 12 weeks of OLYSIO in combination with sofosbuvir, similar SVR12 rates were observed among subgroups, including: treatment-naïve and treatment-experienced subjects (112/115 [97%] and 38/40 [95%] respectively), subjects with HCV genotype 1a with and without NS3 Q80K polymorphism (44/46 [96%] and 68/70 [97%], respectively), genotype 1b (38/39 [97%]), and subjects with IL28B CC and non-CC genotypes (43/43 [100%] and 107/112 [96%], respectively).<br>**14.3 OLYSIO in Combination with Peg-IFN-alfa and RBV**<br>*Treatment-Naïve Adult Subjects with HCV Genotype 1 Infection*<br>(…) In the pooled analysis for QUEST 1 and QUEST 2, demographics and baseline characteristics were balanced between both trials and between the OLYSIO and placebo treatment groups. In the pooled analysis of trials (QUEST 1 and QUEST 2), the 785 enrolled subjects had a median age of 47 years (range: 18 to 73 years; with 2% above 65 years); 56% were male; 91% were White, 7% Black or African American, 1% Asian, and 17% Hispanic; 23% had a body mass index (BMI) greater than or equal to 30 kg/m2 ; 78% had baseline HCV RNA levels greater than 800000 IU/mL; 74% had METAVIR fibrosis score F0, F1 or F2, 16% METAVIR fibrosis score F3, and 10% METAVIR fibrosis score F4 (cirrhosis); 48% had HCV genotype 1a, and 51% HCV genotype 1b; 29% had IL28B CC genotype, 56% IL28B CT genotype, and 15% IL28B TT genotype; 17% of the overall population and 34% of the subjects with genotype 1a virus had the NS3 Q80K polymorphism at baseline. In QUEST 1, all subjects received Peg-IFN-alfa-2a; in QUEST 2, 69% of the subjects received Peg-IFN-alfa-2a and 31% received Peg-IFN-alfa-2b.<br>Table 17 shows the response rates in treatment-naïve adult subjects with HCV genotype 1 infection. In the OLYSIO treatment group, SVR12 rates were lower in subjects with genotype 1a virus with the NS3 Q80K polymorphism at baseline compared to subjects infected with genotype 1a virus without the Q80K polymorphism. (See Table 17) (…)<br>*Treatment-Naïve East Asian Subjects with HCV Genotype 1 Infection*<br>(…) These 304 subjects had a median age of 45 years (range: 18 to 68 years; with 2% above 65 years); 49% were male; all were East Asians (81% were enrolled in China, and 19% in South Korea); 3% had a body mass index (BMI) greater or equal to 30 kg/m2 ; 84% had baseline HCV RNA levels greater than 800000 IU/mL; 82% had METAVIR fibrosis score F0, F1 or F2, 12% METAVIR fibrosis score F3, and 6% METAVIR fibrosis score F4 (cirrhosis); 1% had HCV genotype 1a, and 99% HCV genotype 1b; less than 1% of the overall population had Q80K polymorphism at baseline; 79% had IL28B CC genotype, 20% IL28B CT genotype, and 1% IL28B TT genotype. Demographics and baseline characteristics were balanced across the OLYSIO 150 mg and placebo treatment groups. (…)<br>*Adult Subjects with HCV Genotype 1 Infection who Failed Prior Peg-IFN-alfa and RBV Therapy*<br>(…) Demographics and baseline characteristics were balanced between the OLYSIO and placebo treatment groups. The 393 subjects enrolled in the PROMISE trial had a median age of 52 years (range: 20 to 71 years; with 3% above 65 years); 66% were male; 94% were White, 3% Black or African American, 2% Asian, and 7% Hispanic; 26% had a BMI greater than or equal to 30 kg/m2 ; 84% had baseline HCV RNA levels greater than 800000 IU/mL; 69% had METAVIR fibrosis score F0, F1 or F2, 15% METAVIR fibrosis score F3, and 15% METAVIR fibrosis score F4 (cirrhosis); 42% had HCV genotype 1a, and 58% HCV genotype 1b; 24% had IL28B CC genotype, 64% IL28B CT genotype, and 12% IL28B TT genotype; 13% of the overall population and 31% of the subjects with genotype 1a virus had the NS3 Q80K polymorphism at baseline. The prior IFN-based HCV therapy was Peg-IFN-alfa-2a/RBV (68%) or Peg-IFN-alfa-2b/RBV (27%). (…)<br>(…) SVR12 rates were higher for the OLYSIO treatment group compared to the placebo treatment group by sex, age, race, BMI, HCV genotype/subtype, baseline HCV RNA load (less than or equal to 800000 IU/mL, greater than 800000 IU/mL), prior HCV therapy, METAVIR fibrosis score, and IL28B genotype. Table 20 shows the SVR rates by METAVIR fibrosis score. (…)<br>(…) In this trial, 66 subjects received 12 weeks of 150 mg OLYSIO in combination with Peg-IFN-alfa-2a and RBV for 48 weeks, and 66 subjects received placebo in combination with Peg-IFN-alfa-2a and RBV for 48 weeks. These 132 subjects had a median age of 49 years (range: 20 to 66 years; with 1% above 65 years); 66% were male; 93% were White, 3% Black or African American, and 2% Asian; 27% had a BMI greater than or equal to 30 kg/m2 ; 85% had baseline HCV RNA levels greater than 800000 IU/mL; 64% had METAVIR fibrosis score F0, F1, or F2, 18% METAVIR fibrosis score F3, and 18% METAVIR fibrosis score F4 (cirrhosis); 43% had HCV genotype 1a, and 57% HCV genotype 1b; 17% had IL28B CC genotype, 67% IL28B CT genotype, and 16% IL28B TT genotype (information available for 93 subjects); 27% of the overall population and 23% of the subjects with genotype 1a virus had the NS3 Q80K polymorphism at baseline. Forty percent (40%) of subjects were prior relapsers, 35% prior partial responders, and 25% prior null responders following prior therapy with Peg-IFN-alfa and RBV. Demographics and baseline characteristics were balanced between the 12 weeks 150 mg OLYSIO and placebo treatment groups. (See Table 21)<br>SVR24 rates were higher in the OLYSIO-treated subjects compared to subjects receiving placebo in combination with Peg-IFN-alfa and RBV, regardless of HCV geno/subtype, METAVIR fibrosis score, and IL28B genotype.<br>*Subjects with HCV/HIV-1 Co-Infection*

NDA/ANDA/BLA Number, Label Version Date	Drug	Therapeutic Area*	Biomarker†	Labeling Sections	Labeling Text‡
					(…) The 106 enrolled subjects in the C212 trial had a median age of 48 years (range: 27 to 67 years; with 2% above 65 years); 85% were male; 82% were White, 14% Black or African American, 1% Asian, and 6% Hispanic; 12% had a BMI greater than or equal to 30 kg/m2 ; 86% had baseline HCV RNA levels greater than 800,000 IU/mL; 68% had METAVIR fibrosis score F0, F1 or F2, 19% METAVIR fibrosis score F3, and 13% METAVIR fibrosis score F4; 82% had HCV genotype 1a, and 17% HCV genotype 1b; 28% of the overall population and 34% of the subjects with genotype 1a had Q80K polymorphism at baseline; 27% had IL28B CC genotype, 56% IL28B CT genotype, and 17% IL28B TT genotype; 50% (n=53) were HCV treatment-naïve subjects, 14% (n=15) prior relapsers, 9% (n=10) prior partial responders, and 26% (n=28) prior null responders. (…)<br>*Adult Subjects with HCV Genotype 4 Infection*<br>(…) The 107 enrolled subjects in the RESTORE trial with HCV genotype 4 had a median age of 49 years (range: 27 to 69 years; with 5% above 65 years); 79% were male; 72% were White, 28% Black or African American, and 7% Hispanic; 14% had a BMI greater than or equal to 30 kg/m2 ; 60% had baseline HCV RNA levels greater than 800,000 IU/mL; 57% had METAVIR fibrosis score F0, F1 or F2, 14% METAVIR fibrosis score F3, and 29% METAVIR fibrosis score F4; 42% had HCV genotype 4a, and 24% had HCV genotype 4d; 8% had IL28B CC genotype, 58% IL28B CT genotype, and 35% IL28B TT genotype; 33% (n=35) were treatment-naïve HCV subjects, 21% (n=22) prior relapsers, 9% (n=10) prior partial responders, and 37% (n=40) prior null responders. (…)
**209884, 06/21/2022**	**Siponimod**	Neurology	CYP2C9	Dosage and Administration, Contraindications, Drug Interactions, Use in Specific Populations, Clinical Pharmacology	**2 DOSAGE AND ADMINISTRATION**<br>**2.1 Assessments Prior to First Dose of MAYZENT**<br>Before initiation of treatment with MAYZENT, assess the following:<br>CYP2C9 Genotype Determination<br>Test patients for CYP2C9 variants to determine CYP2C9 genotype [see Dosage and Administration (2.2, 2.3), Contraindications (4), and Use in Specific Populations (8.6)]. An FDA-cleared or -approved test for the detection of CYP2C9 variants to direct the use of siponimod is not currently available.<br>**2.2 Recommended Dosage in Patients With CYP2C9 Genotypes *1/*1, *1/*2, or *2/*2**<br>Maintenance Dosage<br>After treatment titration (see Treatment Initiation), the recommended maintenance dosage of MAYZENT is 2 mg taken orally once daily starting on Day 6. Dosage adjustment is required in patients with a CYP2C9 *1/*3 or *2/*3 genotype [see Dosage and Administration (2.3)].<br>Treatment Initiation<br>Initiate MAYZENT with a 5-day titration, as shown in Table 1 [see Warnings and Precautions (5.3)]. A 12-tablet starter pack should be used for patients who will be titrated to the 2-mg maintenance dosage [see How Supplied/Storage and Handling (16.1, 16.2)]. (See Table 1)<br>If one titration dose is missed for more than 24 hours, treatment needs to be reinitiated with Day 1 of the titration regimen.<br>**2.3 Recommended Dosage in Patients With CYP2C9 Genotypes *1/*3 or *2/*3**<br>Maintenance Dosage<br>In patients with a CYP2C9 *1/*3 or *2/*3 genotype, after treatment titration (see Treatment Initiation), the recommended maintenance dosage of MAYZENT is 1 mg taken orally once daily starting on Day 5.<br>Treatment Initiation<br>Initiate MAYZENT with a 4-day titration, as shown in Table 2 [see Warnings and Precautions (5.3) and Use in Specific Populations (8.6)]. A 7-tablet starter pack should be used for patients who will be titrated to the 1-mg maintenance dosage [see How Supplied/Storage and Handling (16.1, 16.2)]. (See Table 2)<br>If one titration dose is missed for more than 24 hours, treatment needs to be reinitiated with Day 1 of the titration regimen.<br><br>**4 CONTRAINDICATIONS**<br>MAYZENT is contraindicated in patients who have:<br>• A CYP2C9*3/*3 genotype [see Use in Specific Populations (8.6) and Clinical Pharmacology (12.5)] (…)<br><br>**7 DRUG INTERACTIONS**<br>**7.6 CYP2C9 and CYP3A4 Inducers**<br>Because of a significant decrease in siponimod exposure, concomitant use of MAYZENT and drugs that cause moderate CYP2C9 and strong CYP3A4 induction is not recommended for all patients. This concomitant drug regimen can consist of moderate CYP2C9/strong CYP3A4 dual inducer (e.g., rifampin or carbamazepine) or a moderate CYP2C9 inducer in combination with a separate strong CYP3A4 inducer.<br>Caution should be exercised for concomitant use of MAYZENT with moderate CYP2C9 inducers.<br>Concomitant use of MAYZENT and moderate (e.g., modafinil, efavirenz) or strong CYP3A4 inducers is not recommended for patients with CYP2C9*1/*3 or *2/*3 genotype [see Clinical Pharmacology (12.3)].<br><br>**8 USE IN SPECIFIC POPULATIONS**<br>**8.6 CYP2C9 Genotype**<br>Before initiation of treatment with MAYZENT, test patients to determine CYP2C9 genotype.<br>MAYZENT is contraindicated in patients homozygous for CYP2C9*3 (i.e., CYP2C9*3/*3 genotype) because of substantially elevated siponimod plasma levels. The *3/*3 genotype is present in approximately 0.5% of white patients and 1% of Asian patients, and is less prevalent in other racial/ethnic groups.<br>MAYZENT dosage adjustment is recommended in patients with CYP2C9*1/*3 or *2/*3 genotype because of an increase in exposure to siponimod [see Dosage and Administration (2.3) and Clinical Pharmacology (12.5)]. The *1/*3 or *2/*3 genotypes are present in 2% to 20% of the population depending on ancestry.<br>There are other less frequently occurring polymorphisms in CYP2C9. Some polymorphisms, such as *5, *6, *8, and *11, are associated with decreased or loss of enzyme function. The impact of variants other than *2 and *3 on the pharmacokinetics of siponimod has not been evaluated. It is anticipated that variants that result in loss of CYP2C9 function (e.g., *6) will have similar effects on siponimod pharmacokinetics as the *3 variant [see Dosage and Administration (2.3) and Clinical Pharmacology (12.5)].<br>**12 CLINICAL PHARMACOLOGY**

* Therapeutic areas do not necessarily reflect the CDER review division.
† Representative biomarkers are listed based on standard nomenclature as per the Human Genome Organization (HUGO) symbol and/or simplified descriptors using other common conventions. Listed biomarkers do not necessarily reflect the terminology used in labeling. The term "Nonspecific" is provided when labeling does not explicitly identify the specific biomarker(s) or when the biomarker is represented by a molecular phenotype or gene signature, and in some cases the biomarker was inferred based on the labeling language.
‡ Referenced figures and tables may be found in the labeling available at Drugs@FDA.
Blue text represents the most recent addtions and/or changes since last posted version.

NDA/ANDA/BLA Number, Label Version Date	Drug	Therapeutic Area*	Biomarker†	Labeling Sections	Labeling Text‡
					**12.2 Pharmacodynamics**<br>Immune System<br>MAYZENT induces a dose-dependent reduction of the peripheral blood lymphocyte count within 6 hours of the first dose, caused by the reversible sequestration of lymphocytes in lymphoid tissues.<br>With continued daily dosing, the lymphocyte count continues to decrease, reaching a nadir median (90% CI) lymphocyte count of approximately 0.560 (0.271-1.08) cells/nL in a typical CYP2C9*1/*1 or *1/*2, non-Japanese patient, corresponding to 20% to 30% of baseline. Low lymphocyte counts are maintained with chronic daily dosing [see Warnings and Precautions (5.1)].<br>Lymphocyte counts returned to the normal range in 90% of patients within 10 days of stopping therapy. After stopping MAYZENT treatment, residual lowering effects on peripheral lymphocyte count may persist for up to 3-4 weeks after the last dose [see Warnings and Precautions (5.1)].<br>**12.3 Pharmacokinetics**<br>Drug Interaction Studies<br>*Siponimod as an Object of Interaction*<br>CYP2C9 is polymorphic and the genotype influences the fractional contributions of the two oxidative metabolism pathways to overall elimination. Physiologically based PK modeling indicates a differential CYP2C9 genotype-dependent inhibition and induction of CYP3A4 pathways. With decreased CYP2C9 metabolic activity in the respective genotypes, a larger effect of the CYP3A4 perpetrators on siponimod exposure is anticipated.<br>*Coadministration of Siponimod with CYP2C9 and CYP3A4 Inhibitors*<br>The coadministration of fluconazole (moderate CYP2C9 and CYP3A4 dual inhibitor) 200 mg daily at steady-state and a single dose of siponimod 4 mg in CYP2C9*1/*1 healthy volunteers led to a 2-fold increase in the AUC of siponimod. Mean siponimod terminal half-life was increased by 50%. Fluconazole led to a 2-to 4-fold increase in the AUCtau,ss of siponimod across different CYP2C9 genotypes, according to in silico evaluation [see Drug Interactions (7.5)].<br>*Coadministration of Siponimod with CYP2C9 and CYP3A4 Inducers*<br>The coadministration of siponimod 2 mg daily in the presence of 600 mg daily doses of rifampin (strong CYP3A4 and moderate CYP2C9 dual inducer) decreased siponimod AUCtau,ss and Cmax,ss by 57% and 45%, respectively in CY2C9*1/*1 subjects. Rifampin and efavirenz (moderate CYP3A4 inducer) reduced the AUCtau,ss of siponimod by up to 78% and up to 52%, respectively, across CYP2C9 genotypes, according to in silico evaluation [see Drug Interactions (7.6)].<br>*Oral Contraceptives*<br>The effects of coadministration of siponimod 2 mg and 4 mg (twice the recommended dosage) once daily with a monophasic oral contraceptive (OC) containing 30 mcg ethinyl estradiol and 150 mcg levonorgestrel were assessed in 24 healthy female subjects (18 to 40 years of age; CYP2C9*1/*1 genotype). There were no clinically relevant effects on the PK or PD of the OC. No interaction studies have been performed with OCs containing other progestagens; however, an effect of siponimod on their exposure is not expected.<br>**12.5 Pharmacogenomics**<br>The CYP2C9 genotype has a significant impact on siponimod metabolism. After a single dose of 0.25 mg siponimod, AUCinf and AUClast were approximately 2- and 4-fold higher in subjects with the CYP2C9*2/*3 and CYP2C9*3/*3 genotypes, respectively, while there was only a minor increase of Cmax by 21% and 16%, respectively, compared to extensive metabolizers (CYP2C9*1/*1). Mean half-life is prolonged in CYP2C9*2/*3 and CYP2C9*3/*3 carriers (51 hours and 126 hours, respectively).<br>An apparent systemic clearance (CL/F) of about 3.11 L/h was estimated in CYP2C9 extensive metabolizer (CYP2C9*1/*1 and CYP2C9*1/*2) MS patients after multiple oral administrations of siponimod. Cl/F is 2.5, 1.9, 1.6, and 0.9 L/h in subjects with the CYP2C9*2/*2, CYP2C9*1/*3, CYP2C9*2/*3, and CYP2C9*3/*3 genotypes, respectively. The resultant increase in siponimod AUC was approximately 25%, 61%, 91%, and 285% higher in CYP2C9*2/*2, CYP2C9*1/*3, CYP2C9*2/*3, and CYP2C9*3/*3 subjects, respectively, as compared to CYP2C9*1/*1 subjects [see Dosage and Administration (2.1, 2.3) and Contraindications (4)]. As the apparent clearance estimated for CYP2C9*1/*2 subjects is comparable to that of CYP2C9*1/*1 subjects, similar siponimod exposure is expected for both genotypes.<br>Variants other than *2 and *3 may also lead to decreased or loss of CYP2C9 function (e.g., *5, *6, *8, *11) and may have substrate-specific effects. The frequency of certain CYP2C9 variants differs based on ancestry. The *2 and *3 variants are more prevalent in patients of European or Asian ancestry, while *5, *6, *8, and *11 are more prevalent in individuals of African ancestry [see Use in Specific Populations (8.6)].
**203922, 10/19/2017**	Sodium Nitrite (1)	Toxicology	G6PD	Warnings and Precautions	**5 WARNINGS AND PRECAUTIONS**<br>**5.6 G6PD Deficiency**<br>Because patients with G6PD deficiency are at increased risk of a hemolytic crisis with sodium nitrite administration, alternative therapeutic approaches should be considered in these patients. Patients with known or suspected G6PD deficiency should be monitored for an acute drop in hematocrit. Exchange transfusion may be needed for patients with G6PD deficiency who receive sodium nitrite.
**203922, 10/19/2017**	Sodium Nitrite (2)	Toxicology	Nonspecific (Congenital Methemoglobinemia)	Boxed Warning, Warnings and Precautions	**BOXED WARNING**<br>**WARNING: LIFE THREATENING HYPOTENSION AND METHEMOGLOBIN FORMATION**<br>Sodium nitrite can cause serious adverse reactions and death in humans, even at doses less than twice the recommended therapeutic dose. Sodium nitrite causes hypotension and methemoglobin formation, which diminishes oxygen carrying capacity. Hypotension and methemoglobin formation can occur concurrently or separately. Because of these risks, sodium nitrite should be used to treat acute life-threatening cyanide poisoning and be used with caution in patients where the diagnosis of cyanide poisoning is uncertain. Patients should be closely monitored to ensure adequate perfusion and oxygenation during treatment with sodium nitrite. Alternative therapeutic approaches should be considered in patients known to have diminished oxygen or cardiovascular reserve (e.g., smoke inhalation victims, pre-existing anemia, cardiac or respiratory compromise), and those at higher risk of developing methemoglobinemia (e.g., congenital methemoglobin reductase deficiency) as they are at greater risk for potentially life-threatening adverse events related to the use of sodium nitrite. [see Warnings and Precautions (5.1 and 5.2)]

* Therapeutic areas do not necessarily reflect the CDER review division.
† Representative biomarkers are listed based on standard nomenclature as per the Human Genome Organization (HUGO) symbol and/or simplified descriptors using other common conventions. Listed biomarkers do not necessarily reflect the terminology used in labeling. The term "Nonspecific" is provided when labeling does not explicitly identify the specific biomarker(s) or when the biomarker is represented by a molecular phenotype or gene signature, and in some cases the biomarker was inferred based on the labeling language.
‡ Referenced figures and tables may be found in the labeling available at Drugs@FDA.
Blue text represents the most recent addtions and/or changes since last posted version.

Table of Pharmacogenomic Biomarkers in Drug Labeling
Last Updated: 06/2024

NDA/ANDA/BLA Number, Label Version Date	Drug	Therapeutic Area*	Biomarker†	Labeling Sections	Labeling Text‡
					**5 WARNINGS AND PRECAUTIONS**<br>**5.1 Hypotension**<br>Sodium nitrite has been associated with severe hypotension, methemoglobinemia, and death at doses less than twice recommended therapeutic doses. Hypotension may occur concurrently or separately. Sodium nitrite should be used to treat life-threatening cyanide poisoning. When the diagnosis of cyanide poisoning is uncertain and/or the patient is not in extremis, special consideration should be given to administration of sodium nitrite if the patient is known or suspected to have diminished oxygen or cardiovascular reserve (e.g., smoke inhalation victims, pre-existing anemia, substantial blood loss, cardiac or respiratory compromise) or to be at higher risk of developing methemoglobinemia (e.g., congenital methemoglobin reductase deficiency).<br>**5.2 Methemoglobinemia**<br>Supportive care alone may be sufficient treatment without administration of antidotes for many cases of cyanide intoxication, particularly in conscious patients without signs of severe toxicity. Monitor patients closely to ensure adequate perfusion and oxygenation during treatment with sodium nitrite.<br>Monitor methemoglobin levels and administer oxygen during treatment with sodium nitrite whenever possible. When sodium nitrite is administered to humans a wide range of methemoglobin concentrations occur. (…)
**021196, 09/25/2020**	Sodium Oxybate	Neurology	ALDH5A1 (Succinic Semialdehyde Dehydrogenase Deficiency)	Contraindications	**4 CONTRAINDICATIONS**<br>Xyrem is contraindicated for use in:<br>• combination with sedative hypnotics [see Warnings and Precautions (5.1)].<br>• combination with alcohol [see Warnings and Precautions (5.1)].<br>• patients with succinic semialdehyde dehydrogenase deficiency [see Clinical Pharmacology (12.3)].
020572, 07/29/2022	Sodium Phenylbutyrate	Inborn Errors of Metabolism	ASS1, CPS1, OTC (Urea Cycle Disorders)	Indications and Usage, Dosage and Administration, Adverse Reactions, Clinical Pharmacology	**INDICATIONS AND USAGE**<br>BUPHENYL® is indicated as adjunctive therapy in the chronic management of patients with urea cycle disorders involving deficiencies of carbamylphosphate synthetase (CPS), ornithine transcarbamylase (OTC), or argininosuccinic acid synthetase (AS). It is indicated in all patients with neonatal-onset deficiency (complete enzymatic deficiency, presenting within the first 28 days of life). It is also indicated in patients with late-onset disease (partial enzymatic deficiency, presenting after the first month of life) who have a history of hyperammonemic encephalopathy. It is important that the diagnosis be made early and treatment initiated immediately to improve survival. Any episode of acute hyperammonemia should be treated as a lifethreatening emergency. (…)<br>(…) Those who had IQ tests administered had an incidence of mental retardation as follows: ornithine transcarbamylase deficiency, 100% (14/14 patients tested); argininosuccinic acid synthetase deficiency, 88% (15/17 patients tested); and carbamylphosphate synthetase deficiency, 57% (4/7 patients tested). (…)<br>(…) In late-onset deficiency patients, including females heterozygous for ornithine transcarbamylase deficiency, who recover from hyperammonemic encephalopathy and are then treated chronically with sodium phenylbutyrate and dietary protein restriction, the survival rate is 98%. (…)<br><br>**DOSAGE AND ADMINISTRATION**<br>For oral use only.<br>The use of BUPHENYL® Tablets is indicated for children weighing more than 20 kg and for adults.<br>The usual total daily dose of BUPHENYL Tablets and Powder for patients with urea cycle disorders is 450– 600 mg/kg/day in patients weighing less than 20 kg, or 9.9–13.0 g/m2 /day in larger patients. The tablets and powder are to be taken in equally divided amounts with each meal or feeding (i.e., three to six times per day). (…)<br>**NUTRITIONAL MANAGEMENT**<br>(…) At the recommended dose of sodium phenylbutyrate, it is suggested that infants with neonatal-onset CPS and OTC deficiencies initially receive a daily dietary protein intake limited to approximately 1.6 g/kg/day for the first 4 months of life. If tolerated, the daily protein intake may be increased to 1.9 g/kg/day during this period.<br>Protein tolerance will decrease as the growth rate decreases, requiring a reduction in dietary nitrogen intake. From 4 months to 1 year of age, it is recommended that the infant receive at least 1.4 g/kg/day, but 1.7 g/kg/day is advisable. From 1 to 3 years of age, the protein intake should not be less than 1.2 g/kg/day; 1.4 g/kg/day is advisable during this period. For neonatal-onset patients with carbamylphosphate synthetase deficiency or ornithine transcarbamylase deficiency who are at least 6 months of age, it is recommended that the daily protein intake be equally divided between natural protein and supplemental essential amino acids.<br>Patients with argininosuccinic acid synthetase deficiency and those with late-onset disease (partial deficiencies, including females heterozygous for ornithine transcarbamylase), initially may receive a diet containing the age-determined minimal daily natural protein allowance. The protein intake may be increased as tolerated and determined by plasma glutamine and other amino acid levels. However, many patients with partial deficiencies avoid dietary protein.<br>Citrulline supplementation is required and recommended for patients diagnosed with neonatal-onset deficiency of carbamylphosphate synthetase or ornithine transcarbamylase; citrulline daily intake is recommended at 0.17 g/kg/day or 3.8 g/m2 /day.<br>The free-base form of arginine may be used instead of citrulline in patients with milder forms of carbamylphosphate synthetase and ornithine transcarbamylase deficiency (daily intake is recommended at 0.17 g/kg/day or 3.8 g/m2 /day).<br>Arginine supplementation is needed for patients diagnosed with deficiency of argininosuccinic acid synthetase; arginine (free base) daily intake is recommended at 0.4–0.7 g/kg/day or 8.8–15.4 g/m2 /day.<br><br>**ADVERSE REACTIONS**<br>**Laboratory Adverse Events:**<br>In patients with urea cycle disorders, the frequency of laboratory adverse events by body system were:<br>**Metabolic**: acidosis (14%), alkalosis and hyperchloremia (each 7%), hypophosphatemia (6%), hyperuricemia and hyperphosphatemia (each 2%), and hypernatremia and hypokalemia (each 1%).<br>**Nutritional:** hypoalbuminemia (11%) and decreased total protein (3%).

* Therapeutic areas do not necessarily reflect the CDER review division.
† Representative biomarkers are listed based on standard nomenclature as per the Human Genome Organization (HUGO) symbol and/or simplified descriptors using other common conventions. Listed biomarkers do not necessarily reflect the terminology used in labeling. The term “Nonspecific” is provided when labeling does not explicitly identify the specific biomarker(s) or when the biomarker is represented by a molecular phenotype or gene signature, and in some cases the biomarker was inferred based on the labeling language.
‡ Referenced figures and tables may be found in the labeling available at Drugs@FDA.
Blue text represents the most recent addtions and/or changes since last posted version.

NDA/ANDA/BLA Number, Label Version Date	Drug	Therapeutic Area*	Biomarker†	Labeling Sections	Labeling Text‡
					**Hepatic**: increased alkaline phosphatase (6%), increased liver transaminases (4%), and hyperbilirubinemia (1%).<br>**Hematologic:** anemia (9%), leukopenia and leukocytosis (each 4%), thrombocytopenia (3%), and thrombocytosis (1%).<br>The clinician is advised to routinely perform urinalysis, blood chemistry profiles, and hematologic tests.<br><br>**CLINICAL PHARMACOLOGY**<br>**Pharmacodynamics:**<br>In patients with urea cycle disorders, BUPHENYL decreased elevated plasma ammonia glutamine levels. It increases waste nitrogen excretion in the form of phenylacetylglutamine.<br>**Hepatic insufficiency:**<br>In patients who did not have urea cycle disorders but had impaired hepatic function, the metabolism and excretion of sodium phenylbutyrate were not affected. However, this information was obtained from unvalidated, uncontrolled case studies.
**204671, 10/23/2018**	**Sofosbuvir**	Infectious Diseases	IFNL3 (IL28B)	Clinical Studies	**14 CLINICAL STUDIES**<br>**14.2 Clinical Trials in Subjects with Genotype 1 or 4 HCV**<br>*Treatment-Naïve Adults – NEUTRINO (Study 110)*<br>(…) SVR12 rates were 99% (89/90) in subjects with genotype 1 or 4 HCV and baseline IL28B C/C allele and 87% (200/230) in subjects with genotype 1 or 4 HCV and baseline IL28B non-C/C alleles. It is estimated that the SVR12 in patients who previously failed pegylated interferon and ribavirin therapy will approximate the observed SVR12 in NEUTRINO subjects with multiple baseline factors traditionally associated with a lower response to interferonbased treatment (Table 9). The SVR12 rate in the NEUTRINO trial in genotype 1 subjects with IL28B non-C/C alleles, HCV RNA greater than 800,000 IU/mL and Metavir F3/F4 fibrosis was 71% (37/52). (See Table 9)<br>**14.4 Clinical Trials in Subjects Coinfected with HCV and HIV-1**<br>(…) In subjects with HCV genotype 1 infection, the SVR12 rate was 82% (74/90) in subjects with genotype 1a infection and 54% (13/24) in subjects with genotype 1b infection, with relapse accounting for the majority of treatment failures. SVR12 rates in subjects with HCV genotype 1 infection were 80% (24/30) in subjects with baseline IL28B C/C allele and 75% (62/83) in subjects with baseline IL28B non-C/C alleles. (…)<br>**14.5 Clinical Trial in Pediatrics**<br>The efficacy of SOVALDI in HCV-infected pediatric subjects 12 years of age and older was evaluated in 50 subjects with HCV genotype 2 (N = 13) or genotype 3 (N = 37) in a Phase 2, open label clinical trial. Subjects with HCV genotype 2 or 3 infection in the trial were treated with SOVALDI and weight-based ribavirin for 12 or 24 weeks, respectively [see Dosage and Administration (2.3)].<br>Of the 50 treated subjects, the median age was 15 years (range: 12 to 17); 42% of the subjects were female; 90% were White, 4% were Black, and 2% were Asian; 4% were Hispanic/Latino; mean weight was 61 kg (range: 30 to 101 kg); 18% were treatment experienced; 66% had baseline HCV RNA levels greater than or equal to 800,000 IU/mL; 74% of subjects had non-CC IL28B alleles (CT or TT); and no subjects had known cirrhosis. The majority of subjects (69%) had been infected through vertical transmission.<br>The SVR12 rate was 100% (13/13) in genotype 2 subjects and 97% (36/37) in genotype 3 subjects. No subject experienced on-treatment virologic failure or relapse.
**208341, 11/09/2017**	**Sofosbuvir and Velpatasvir**	Infectious Diseases	IFNL3 (IL28B)	Clinical Studies	**14 CLINICAL STUDIES**<br>**14.2 Clinical Trials in Subjects without Cirrhosis and Subjects with Compensated Cirrhosis**<br>*Genotype 1, 2, 4, 5, and 6 HCV Infected Adults (ASTRAL-1)*<br>(…) Demographics and baseline characteristics were balanced between the EPCLUSA and placebo group. Of the 740 treated subjects, the median age was 56 years (range: 18 to 82); 60% of the subjects were male; 79% were White, 9% were Black; 21% had a baseline body mass index at least 30 kg/m2 ; the proportions of subjects with genotype 1, 2, 4, 5, or 6 HCV infection were 53%, 17%, 19%, 5%, and 7%, respectively; 69% had non-CC IL28B alleles (CT or TT); 74% had baseline HCV RNA levels at least 800,000 IU/mL; 19% had compensated cirrhosis; and 32% were treatment-experienced. (…)<br>*Genotype 2 HCV Infected Adults (ASTRAL-2)*<br>(…) Demographics and baseline characteristics were balanced across the two treatment groups. Of the 266 treated subjects, the median age was 58 years (range: 23 to 81); 59% of the subjects were male; 88% were White; 7% were Black; 33% had a baseline body mass index at least 30 kg/m2 ; 62% had non-CC IL28B alleles (CT or TT); 80% had baseline HCV RNA levels at least 800,000 IU/mL; 14% had compensated cirrhosis; and 15% were treatment-experienced. (…)<br>*Genotype 3 HCV Infected Adults (ASTRAL-3)*<br>(…) Demographics and baseline characteristics were balanced across the treatment groups. Of the 552 treated subjects, the median age was 52 years (range: 19 to 76); 62% of the subjects were male; 89% were White; 9% were Asian; 20% had a baseline body mass index at least 30 kg/m2 ; 61% had non-CC IL28B alleles (CT or TT); 70% had baseline HCV RNA levels at least 800,000 IU/mL; 30% had compensated cirrhosis; and 26% were treatment-experienced. (…)<br>**14.3 Clinical Trial in Subjects Coinfected with HCV and HIV-1**<br>(…)Of the 106 treated subjects, the median age was 57 years (range: 25 to 72); 86% of the subjects were male; 51% were White; 45% were Black; 22% had a baseline body mass index at least 30 kg/m2 ; the proportions of patients with genotype 1, 2, 3, or 4 HCV infection were 74%; 10%; 11%, and 5% respectively; no subjects with genotype 5 or 6 HCV were treated with EPCLUSA; 77% had non- CC IL28B alleles (CT or TT); 74% had baseline HCV RNA levels of at least 800,000 IU/mL; 18% had compensated cirrhosis; and 29% were treatment experienced. The overall mean CD4+ count was 598 cells/μL (range: 183−1513 cells/μL) and 57% of subjects had CD4+ counts > 500 cells/μL. (…)<br>**14.4 Clinical Trials in Subjects with Decompensated Cirrhosis**<br>(…) Demographics and baseline characteristics were balanced across the treatment groups. Of the 267 treated subjects, the median age was 59 years (range: 40 to 73); 70% of the subjects were male; 90% were White, 6% were Black; 42% had a baseline body mass index at least 30 kg/m2 . The proportions of subjects with genotype 1, 2, 3, 4, or 6 HCV were 78%, 4%, 15%, 3%, and less than 1% (1 subject), respectively. No subjects with genotype 5 HCV infection were

NDA/ANDA/BLA Number, Label Version Date	Drug	Therapeutic Area*	Biomarker†	Labeling Sections	Labeling Text‡
					enrolled. 76% had non-CC IL28B alleles (CT or TT); 56% had baseline HCV RNA levels at least 800,000 IU/mL; 55% were treatmentexperienced; and 95% of subjects had Model for End Stage Liver Disease (MELD) score less than or equal to 15 at baseline. Although all subjects had Child-Pugh B cirrhosis at screening, 6% and 4% of subjects were assessed to have Child-Pugh A and Child-Pugh C cirrhosis, respectively, on the first day of treatment. (…)
**209195, 11/09/2017**	**Sofosbuvir, Velpatasvir, and Voxilaprevir**	Infectious Diseases	IFNL3 (IL28B)	Clinical Studies	**CLINICAL STUDIES**<br>**14.2 Clinical Trials in HCV DAA-Experienced Subjects**<br>*NS5A Inhibitor-Experienced Adults Without Cirrhosis or With Compensated Cirrhosis (POLARIS-1)*<br>(…) Demographics and baseline characteristics were generally balanced across treatment groups. Of the 415 treated subjects, the median age was 59 years (range: 27 to 84); 77% of the subjects were male; 81% were White; 14% were Black; 6% were Hispanic or Latino; 33% had a baseline body mass index at least 30 kg/m^2; the majority of subjects had genotype 1 (72%) or genotype 3 (19%) HCV infection; 82% had a non-CC IL28B genotype (CT or TT); 74% had baseline HCV RNA levels at least 800,000 IU/mL; and 41% had compensated cirrhosis. (…)<br>*DAA-Experienced Adults Without Cirrhosis or With Compensated Cirrhosis Who Had Not Received An NS5A Inhibitor (POLARIS-4)*<br>(…) Demographics and baseline characteristics were generally balanced across treatment groups. Of the 333 treated subjects, the median age was 58 years (range: 24 to 85); 77% of the subjects were male; 87% were White, 9% were Black; 8% were Hispanic or Latino; 35% had a baseline body mass index at least 30 kg/m^2; 81% had non-CC IL28B genotypes (CT or TT); 75% had baseline HCV RNA levels at least 800,000 IU/mL; and 46% had compensated cirrhosis. (…)
**05/28/2021, 214665**	**Sotorasib**	Oncology	KRAS	Indication and Usage, Dosage and Administration, Adverse Reactions, Clinical Pharmacology, Clinical Studies	**1 INDICATIONS AND USAGE**<br>LUMAKRAS is indicated for the treatment of adult patients with KRAS G12C-mutated locally advanced or metastatic non-small cell lung cancer (NSCLC), as determined by an FDA-approved test [see Dosage and Administration (2.1)], who have received at least one prior systemic therapy.<br>This indication is approved under accelerated approval based on overall response rate (ORR) and duration of response (DOR) [see Clinical Studies (14)]. Continued approval for this indication may be contingent upon verification and description of clinical benefit in a confirmatory trial(s).<br><br>**2 DOSAGE AND ADMINISTRATION**<br>**2.1 Patient Selection**<br>Select patients for treatment of locally advanced or metastatic NSCLC with LUMAKRAS based on the presence of KRAS G12C mutation in tumor or plasma specimens [see Clinical Studies (14)]. If no mutation is detected in a plasma specimen, test tumor tissue.<br>Information on FDA-approved tests for the detection of KRAS G12C mutations is available at: http://www.fda.gov/CompanionDiagnostics.<br><br>**6 ADVERSE REACTIONS**<br>**6.1 Clinical Trials Experience**<br>The pooled safety population described in the WARNINGS AND PRECAUTIONS reflect exposure to LUMAKRAS as a single agent at 960 mg orally once daily in 357 patients in with NSCLC and other solid tumors with KRAS G12C mutation enrolled in CodeBreaK 100, 28% were exposed for 6 months or longer and 3% were exposed for greater than one year.<br>Non-Small Cell Lung Cancer<br>The safety of LUMAKRAS was evaluated in a subset of patients with KRAS G12C-mutated locally advanced or metastatic NSCLC in CodeBreaK 100 [see Clinical Studies (14)]. Patients received LUMAKRAS 960 mg orally once daily until disease progression or unacceptable toxicity (n = 204). Among patients who received LUMAKRAS, 39% were exposed for 6 months or longer and 3% were exposed for greater than one year. (See Tables 3 and 4) (…)<br><br>**12 CLINICAL PHARMACOLOGY**<br>**12.3 Pharmacokinetics**<br>The pharmacokinetics of sotorasib have been characterized in healthy subjects and in patients with KRAS G12C-mutated solid tumors, including NSCLC. Sotorasib exhibited non-linear, time-dependent, pharmacokinetics over the dose range of 180 mg to 960 mg (0.19 to 1 time the approved recommended dosage) once daily with similar systemic exposure (i.e., AUC0-24h and Cmax) across doses at steady-state. Sotorasib systemic exposure was comparable between film-coated tablets and film-coated tablets predispersed in water administered under fasted conditions. Sotorasib plasma concentrations reached steady state within 22 days. No accumulation was observed after repeat LUMAKRAS dosages with a mean accumulation ratio of 0.56 (coefficient of variation (CV): 59%).<br><br>**14 CLINICAL STUDIES**<br>The efficacy of LUMAKRAS was demonstrated in a subset of patients enrolled in a single-arm, open-label, multicenter trial (CodeBreaK 100 [NCT03600883]). Eligible patients were required to have locally advanced or metastatic KRAS G12C-mutated NSCLC with disease progression after receiving an immune checkpoint inhibitor and/or platinum-based chemotherapy, an Eastern Cooperative Oncology Group Performance Status (ECOG PS) of 0 or 1, and at least one measurable lesion as defined by Response Evaluation Criteria in Solid Tumors (RECIST v1.1).<br>All patients were required to have prospectively identified KRAS G12C-mutated NSCLC in tumor tissue samples by using the QIAGEN therascreen® KRAS RGQ PCR Kit performed in a central laboratory. Of 126 total enrolled subjects, 2 (2%) were unevaluable for efficacy analysis due to the absence of radiographically measurable lesions at baseline. Of the 124 patients with KRAS G12C mutations confirmed in tumor tissue, plasma samples from 112 patients were tested retrospectively using the Guardant360® CDx. 78/112 patients (70%) had KRAS G12C mutation identified in plasma specimen, 31/112 patients (28%) did not have KRAS G12C mutation identified in plasma specimen and 3/112 (2%) were unevaluable due to Guardant360® CDx test failure. (See Table 5)
**060076**	**Streptomycin**	Infectious Diseases	MT-RNR1	Warnings	Labeling not electronically available on Drugs@FDA
**019998, 10/02/2018**	**Succimer**	Hematology	G6PD	Clinical Pharmacology	**CLINICAL PHARMACOLOGY**

* Therapeutic areas do not necessarily reflect the CDER review division.
† Representative biomarkers are listed based on standard nomenclature as per the Human Genome Organization (HUGO) symbol and/or simplified descriptors using other common conventions. Listed biomarkers do not necessarily reflect the terminology used in labeling. The term “Nonspecific” is provided when labeling does not explicitly identify the specific biomarker(s) or when the biomarker is represented by a molecular phenotype or gene signature, and in some cases the biomarker was inferred based on the labeling language.
‡ Referenced figures and tables may be found in the labeling available at Drugs@FDA.
Blue text represents the most recent addtions and/or changes since last posted version.

NDA/ANDA/BLA Number, Label Version Date	Drug	Therapeutic Area*	Biomarker†	Labeling Sections	Labeling Text‡
					(…) In addition to the controlled studies, approximately 250 patients with lead poisoning have been treated with succimer either orally or parenterally in open U.S. and foreign studies with similar results reported. Succimer has been used for the treatment of lead poisoning in one patient with sickle cell anemia and in five patients with glucose-6-phosphodehydrogenase (G6PD) deficiency without adverse reactions. (…)
008453, 11/01/2022	Succinylcholine (1)	Anesthesiology	BCHE	Warnings, Precautions	**WARNINGS**<br>(…) Succinylcholine is metabolized by plasma cholinesterase and should be used with caution, if at all, in patients known to be or suspected of being homozygous for the atypical plasma cholinesterase gene.<br><br>**PRECAUTIONS**<br>*Reduced Plasma Cholinesterase Activity*<br>Succinylcholine should be used carefully in patients with reduced plasma cholinesterase (pseudocholinesterase) activity. The likelihood of prolonged neuromuscular block following administration of succinylcholine must be considered in such patients (see DOSAGE AND ADMINISTRATION).<br>Plasma cholinesterase activity may be diminished in the presence of genetic abnormalities of plasma cholinesterase (e.g., patients heterozygous or homozygous for atypical plasma cholinesterase gene), pregnancy, severe liver or kidney disease, malignant tumors, infections, burns, anemia, decompensated heart disease, peptic ulcer, or myxedema. (…)<br>(…) Patients homozygous for atypical plasma cholinesterase gene (1 in 2500 patients) are extremely sensitive to the neuromuscular blocking effect of succinylcholine. In these patients, a 5- to 10-mg test dose of succinylcholine may be administered to evaluate sensitivity to succinylcholine, or neuromuscular blockade may be produced by the cautious administration of a 1-mg/mL solution of succinylcholine by slow IV infusion. Apnea or prolonged muscle paralysis should be treated with controlled respiration.
008453, 11/01/2022	Succinylcholine (2)	Anesthesiology	CACNA1S, RYR1 (Genetic Susceptibility to Malignant Hyperthermia)	Boxed Warning, Contraindications, Warnings, Precautions, Adverse Reactions	**WARNING**<br>**RISK OF CARDIAC ARREST FROM HYPERKALEMIC RHABDOMYOLYSIS**<br>There have been rare reports of acute rhabdomyolysis with hyperkalemia followed by ventricular dysrhythmias, cardiac arrest, and death after the administration of succinylcholine to apparently healthy pediatric patients who were subsequently found to have undiagnosed skeletal muscle myopathy, most frequently Duchenne's muscular dystrophy. This syndrome often presents as peaked T-waves and sudden cardiac arrest within minutes after the administration of the drug in healthy appearing pediatric patients (usually, but not exclusively, males, and most frequently 8 years of age or younger). There have also been reports in adolescents. Therefore, when a healthy appearing infant or child develops cardiac arrest soon after administration of succinylcholine not felt to be due to inadequate ventilation, oxygenation, or anesthetic overdose, immediate treatment for hyperkalemia should be instituted. This should include administration of intravenous calcium, bicarbonate, and glucose with insulin, with hyperventilation. Due to the abrupt onset of this syndrome, routine resuscitative measures are likely to be unsuccessful. However, extraordinary and prolonged resuscitative efforts have resulted in successful resuscitation in some reported cases. In addition, in the presence of signs of malignant hyperthermia, appropriate treatment should be instituted concurrently. Since there may be no signs or symptoms to alert the practitioner to which patients are at risk, it is recommended that the use of succinylcholine in pediatric patients should be reserved for emergency intubation or instances where immediate securing of the airway is necessary, e.g. laryngospasm, difficult airway, full stomach, or for intramuscular use when a suitable vein is inaccessible (see PRECAUTIONS: Pediatric Use and DOSAGE AND ADMINISTRATION).<br><br>**CONTRAINDICATIONS**<br>Succinylcholine is contraindicated in persons with personal or familial history of malignant hyperthermia, skeletal muscle myopathies, and known hypersensitivity to the drug. It is also contraindicated in patients after the acute phase of injury following major burns, multiple trauma, extensive denervation of skeletal muscle, or upper motor neuron injury, because succinylcholine administered to such individuals may result in severe hyperkalemia which may result in cardiac arrest (see WARNINGS). The risk of hyperkalemia in these patients increases over time and usually peaks at 7 to 10 days after the injury. The risk is dependent on the extent and location of the injury. The precise time of onset and the duration of the risk period are not known.<br><br>**WARNINGS**<br>**Malignant Hyperthermia**<br>In susceptible individuals, succinylcholine may trigger malignant hyperthermia, a skeletal muscle hypermetabolic state leading to high oxygen demand. Fatal outcomes of malignant hyperthermia have been reported.<br>The risk of developing malignant hyperthermia increases with the concomitant administration of succinylcholine and volatile anesthetic agents. Anectine can induce malignant hyperthermia in patients with known or suspected susceptibility based on genetic factors or family history, including those with certain inherited ryanodine receptor (RYR1) or dihydropyridine receptor (CACNA1S) variants. (see CONTRAINDICATIONS, CLINICAL PHARMACOLOGY; Pharmacogenomics)<br>(…)<br><br>**PRECAUTIONS**<br>**Pediatric Use**<br>(…) In addition, in the presence of signs of malignant hyperthermia, appropriate treatment should be initiated concurrently (see WARNINGS). Since it is difficult to identify which patients are at risk, it is recommended that the use of succinylcholine in pediatric patients should be reserved for emergency intubation or instances where immediate securing of the airway is necessary, e.g., laryngospasm, difficult airway, full stomach, or for intramuscular use when a suitable vein is inaccessible.<br><br>**ADVERSE REACTIONS**<br>Adverse reactions to succinylcholine consist primarily of an extension of its pharmacological actions. Succinylcholine causes profound muscle relaxation resulting in respiratory depression to the point of apnea; this effect may be prolonged. Hypersensitivity reactions, including anaphylaxis, may occur in rare

* Therapeutic areas do not necessarily reflect the CDER review division.
† Representative biomarkers are listed based on standard nomenclature as per the Human Genome Organization (HUGO) symbol and/or simplified descriptors using other common conventions. Listed biomarkers do not necessarily reflect the terminology used in labeling. The term "Nonspecific" is provided when labeling does not explicitly identify the specific biomarker(s) or when the biomarker is represented by a molecular phenotype or gene signature, and in some cases the biomarker was inferred based on the labeling language.
‡ Referenced figures and tables may be found in the labeling available at Drugs@FDA.
Blue text represents the most recent addtions and/or changes since last posted version.

NDA/ANDA/BLA Number, Label Version Date	Drug	Therapeutic Area*	Biomarker†	Labeling Sections	Labeling Text‡
					instances. The following additional adverse reactions have been reported: cardiac arrest, malignant hyperthermia, arrhythmias, bradycardia, tachycardia, hypertension, hypotension, hyperkalemia, prolonged respiratory depression or apnea, increased intraocular pressure, muscle fasciculation, jaw rigidity, postoperative muscle pain, rhabdomyolysis with possible myoglobinuric acute renal failure, excessive salivation, and rash.<br><br>**CLINICAL PHARMACOLOGY**<br>**Pharmacogenomics**<br>RYR1 and CACNA1S are polymorphic genes, and multiple pathogenic variants have been associated with malignant hyperthermia susceptibility (MHS) in patients receiving succinylcholine, including Anectine. Case reports as well as ex-vivo studies have identified multiple variants in RYR1 and CACNA1S associated with MHS. Variant pathogenicity should be assessed based on prior clinical experience, functional studies, prevalence information, or other evidence (see CONTRAINDICATIONS, WARNINGS).
**017381, 08/01/2016**	**Sulfadiazine**	Infectious Diseases	G6PD	Warnings	**WARNINGS**<br>(…) The use of SILVADENE Cream 1% (silver sulfadiazine) in some cases of glucose-6-phosphate dehydrogenase-deficient individuals may be hazardous, as hemolysis may occur.
**017377, 04/30/2021**	**Sulfamethoxazole and Trimethoprim (1)**	Infectious Diseases	G6PD	Precautions	**PRECAUTIONS**<br>Hemolysis In glucose-6-phosphate dehydrogenase deficient individuals, hemolysis may occur. This reaction is frequently dose-related (see Clinical Pharmacology and Dosage and Administration).
**017377, 04/30/2021**	**Sulfamethoxazole and Trimethoprim (2)**	Infectious Diseases	Nonspecific (NAT)	Precautions	**PRECAUTIONS**<br>*Electrolyte Abnormalities*<br>(…) During treatment, adequate fluid intake and urinary output should be ensured to prevent crystalluria. Patients who are "slow acetylators" may be more prone to idiosyncratic reactions to sulfonamides. (…)
**007073, 03/04/2014**	**Sulfasalazine (1)**	Gastroenterology	G6PD	Precautions	**PRECAUTIONS**<br>*General*<br>AZULFIDINE EN-tabs Tablets should be given with caution to patients with severe allergy or bronchial asthma. Adequate fluid intake must be maintained in order to prevent crystalluria and stone formation. Patients with glucose-6-phosphate dehydrogenase deficiency should be observed closely for signs of hemolytic anemia. This reaction is frequently dose related. If toxic or hypersensitivity reactions occur, AZULFIDINE EN-tabs should be discontinued immediately.
**007073, 03/04/2014**	**Sulfasalazine (2)**	Gastroenterology	Nonspecific (NAT)	Clincal Pharmacology	**CLINICAL PHARMACOLOGY**<br>**Pharmacokinetics**<br>*Metabolism*: As mentioned above, SSZ is metabolized by intestinal bacteria to SP and 5- ASA. Approximately 15% of a dose of SSZ is absorbed as parent and is metabolized to some extent in the liver to the same two species. The observed plasma half-life for intravenous sulfasalazine is 7.6 ± 3.4 hours. The primary route of metabolism of SP is via acetylation to form AcSP. The rate of metabolism of SP to AcSP is dependent upon acetylator phenotype. In fast acetylators, the mean plasma half-life of SP is 10.4 hours while in slow acetylators, it is 14.8 hours. SP can also be metabolized to 5-hydroxysulfapyridine (SPOH) and N-acetyl-5-hydroxy-sulfapyridine. 5-ASA is primarily metabolized in both the liver and intestine to N-acetyl-5-aminosalicylic acid via a nonacetylation phenotype dependent route. Due to low plasma levels produced by 5-ASA after oral administration, reliable estimates of plasma half-life are not possible.<br>**Special Populations**<br>*Acetylator Status*: The metabolism of SP to AcSP is mediated by polymorphic enzymes such that two distinct populations of slow and fast metabolizers exist. Approximately 60% of the Caucasian population can be classified as belonging to the slow acetylator phenotype. These subjects will display a prolonged plasma half-life for SP (14.8 hours vs 10.4 hours) and an accumulation of higher plasma levels of SP than fast acetylators. The clinical implication of this is unclear; however, in a small pharmacokinetic trial where acetylator status was determined, subjects who were slow acetylators of SP showed a higher incidence of adverse events.
**020992, 11/30/2020**	**Synthetic Conjugated Estrogens, A (1)**	Gynecology	PROC	Contraindications	**4 CONTRAINDICATIONS**<br>CENESTIN is contraindicated in women with any of the following conditions:<br>• Undiagnosed abnormal genital bleeding [see Warnings and Precautions (5.2)].<br>• Breast cancer or a history of breast cancer [see Warnings and Precautions (5.2)].<br>• Estrogen-dependent neoplasia [see Warnings and Precautions (5.2).<br>• Active DVT, PE, or a history of these conditions [see Warnings and Precautions (5.1)].<br>• Active arterial thromboembolic disease (for example, stroke and MI), or a history of these conditions {see Warnings and Precautions (5.1)].<br>• Known anaphylactic reaction or angioedema or hypersensitivity to CENESTIN.<br>• Hepatic impairment or disease.<br>• Protein C, protein S, or antithrombin deficiency, or other known thrombophilic disorders.
**020992, 11/30/2020**	**Synthetic Conjugated Estrogens, A (2)**	Gynecology	PROS1	Contraindications	**4 CONTRAINDICATIONS**<br>CENESTIN is contraindicated in women with any of the following conditions:<br>• Undiagnosed abnormal genital bleeding [see Warnings and Precautions (5.2)].<br>• Breast cancer or a history of breast cancer [see Warnings and Precautions (5.2)].<br>• Estrogen-dependent neoplasia [see Warnings and Precautions (5.2).<br>• Active DVT, PE, or a history of these conditions [see Warnings and Precautions (5.1)].<br>• Active arterial thromboembolic disease (for example, stroke and MI), or a history of these conditions {see Warnings and Precautions (5.1)].<br>• Known anaphylactic reaction or angioedema or hypersensitivity to CENESTIN.<br>• Hepatic impairment or disease.

* Therapeutic areas do not necessarily reflect the CDER review division.
† Representative biomarkers are listed based on standard nomenclature as per the Human Genome Organization (HUGO) symbol and/or simplified descriptors using other common conventions. Listed biomarkers do not necessarily reflect the terminology used in labeling. The term "Nonspecific" is provided when labeling does not explicitly identify the specific biomarker(s) or when the biomarker is represented by a molecular phenotype or gene signature, and in some cases the biomarker was inferred based on the labeling language.
‡ Referenced figures and tables may be found in the labeling available at Drugs@FDA.
Blue text represents the most recent addtions and/or changes since last posted version.

NDA/ANDA/BLA Number, Label Version Date	Drug	Therapeutic Area*	Biomarker†	Labeling Sections	Labeling Text‡
					• Protein C, protein S, or antithrombin deficiency, or other known thrombophilic disorders.
**020992, 11/30/2020**	**Synthetic Conjugated Estrogens, A (3)**	Gynecology	SERPINC1 (Antithrombin III)	Contraindications	**4 CONTRAINDICATIONS**<br>CENESTIN is contraindicated in women with any of the following conditions:<br>• Undiagnosed abnormal genital bleeding [see Warnings and Precautions (5.2)].<br>• Breast cancer or a history of breast cancer [see Warnings and Precautions (5.2)].<br>• Estrogen-dependent neoplasia [see Warnings and Precautions (5.2).<br>• Active DVT, PE, or a history of these conditions [see Warnings and Precautions (5.1)].<br>• Active arterial thromboembolic disease (for example, stroke and MI), or a history of these conditions {see Warnings and Precautions (5.1)].<br>• Known anaphylactic reaction or angioedema or hypersensitivity to CENESTIN.<br>• Hepatic impairment or disease.<br>• Protein C, protein S, or antithrombin deficiency, or other known thrombophilic disorders.
**211996, 05/03/2019**	**Tafamidis**	Cardiology	TTR	Clinical Pharmacology, Clinical Studies	**12. CLINICAL PHARMACOLOGY**<br>**12.2 Pharmacodynamics**<br>A proprietary TTR stabilization assay was utilized as a pharmacodynamic marker and assessed the stability of the TTR tetramer ex vivo. The TTR stabilization assay quantifies immunoturbidimetric measurement of the stable TTR tetramer in plasma pre- and post-treatment with 2-day in vitro denaturation with urea. Using this proprietary assay, a dose-dependent trend for greater TTR tetramer stabilization is observed for VYNDAQEL 80-mg compared to VYNDAQEL 20-mg. However, the clinical relevance of a higher TTR tetramer stabilization towards cardiovascular outcomes is not known.<br>VYNDAQEL stabilized both the wild type TTR tetramer and the tetramers of 14 TTR variants tested clinically after once-daily dosing. Tafamidis also stabilized the TTR tetramer for 25 variants tested ex vivo. (…)<br><br>**14 CLINICAL STUDIES**<br>Efficacy was demonstrated in a multicenter, international, randomized, double-blind, placebo-controlled study in 441 patients with wild type or hereditary ATTR-CM (NCT01994889).<br>Patients were randomized in a 1:2:2 ratio to receive VYNDAQEL 20 mg (n=88), VYNDAQEL 80 mg (administered as four 20-mg VYNDAQEL capsules) (n=176), or matching placebo (n=177) once daily for 30 months, in addition to standard of care (e.g., diuretics). Treatment assignment was stratified by the presence or absence of a variant TTR genotype as well as baseline disease severity (NYHA Class). Transplant patients were excluded from this study. Table 1 describes the patient demographics and baseline characteristics. (See Tables 1 and 3, Figures 1 and 4)
**210607, 08/08/2018**	**Tafenoquine**	Infectious Diseases	G6PD	Dosage and Administration, Contraindications, Warnings and Precautions, Use in Specific Populations, Patient Counseling Information	**2 DOSAGE AND ADMINISTRATION**<br>**2.1 Tests to be Performed Prior to ARAKODA Dose Initiation**<br>All patients must be tested for glucose-6-phosphate dehydrogenase (G6PD) deficiency prior to prescribing ARAKODA [see Contraindications (4), Warnings and Precautions (5.1)].<br><br>**4 CONTRAINDICATIONS**<br>ARAKODA is contraindicated in:<br>• patients with G6PD deficiency or unknown G6PD status due to the risk of hemolytic anemia [see Warnings and Precautions (5.2)].<br>• breastfeeding by a lactating woman when the infant is found to be G6PD deficient or if the G6PD status of the infant is unknown [see Warnings and Precautions (5.3), Use in Specific Populations (8.2)].<br><br>**5 WARNINGS AND PRECAUTIONS**<br>**5.1. Hemolytic Anemia**<br>Due to the risk of hemolytic anemia in patients with G6PD deficiency, G6PD testing must be performed before prescribing ARAKODA [see Contraindications (4)]. Due to the limitations with G6PD tests, physicians need to be aware of residual risk of hemolysis and adequate medical support and follow-up to manage hemolytic risk should be available. Treatment with ARAKODA is contraindicated in patients with G6PD deficiency or unknown G6PD status [see Contraindications (4)]. In clinical trials, declines in hemoglobin levels were reported in some G6PD-normal patients [see Adverse Reactions (6.1)]. Monitor patients for clinical signs or symptoms of hemolysis [see Warnings and Precautions (5.6)]. Advise patients to discontinue ARAKODA and seek medical attention if signs of hemolysis occur.<br>**5.2 G6PD Deficiency in Pregnancy and Lactation**<br>**Potential Harm to the Fetus**<br>The use of ARAKODA during pregnancy may cause hemolytic anemia in a G6PD-deficient fetus. Even if a pregnant woman has normal levels of G6PD, the fetus could be G6PD deficient. Advise females of reproductive potential that treatment with ARAKODA during pregnancy is not recommended and to avoid pregnancy or use effective contraception during treatment and for 3 months after the last dose of ARAKODA. If a pregnancy is detected during ARAKODA use, discontinue ARAKODA as soon as possible and switch to an alternative prophylactic drug for malaria during pregnancy [see Use in Specific Populations (8.1 and 8.3)].<br>**Potential Harm to the Breastfeeding Infant**<br>A G6PD-deficient infant may be at risk for hemolytic anemia from exposure to ARAKODA through breast milk. Infant G6PD status should be checked before breastfeeding begins. ARAKODA is contraindicated in breastfeeding women when the infant is found to be G6PD deficient or the G6PD status of the infant is unknown [see Contraindications (4)]. Advise the woman with a G6PD-deficient infant or if the G6PD status of the infant is unknown not to breastfeed during treatment with ARAKODA and for 3 months after the final dose [see Use in Specific Populations (8.2)].

NDA/ANDA/BLA Number, Label Version Date	Drug	Therapeutic Area*	Biomarker†	Labeling Sections	Labeling Text‡
					**8 USE IN SPECIFIC POPULATIONS**<br>**8.1 Pregnancy Risk Summary**<br>The use of ARAKODA during pregnancy may cause hemolytic anemia in a fetus who is G6PDdeficient. Treatment with ARAKODA during pregnancy is not recommended. If a pregnancy is detected during ARAKODA use, discontinue ARAKODA as soon as possible and switch to an alternative prophylactic drug for malaria during pregnancy [see Warnings and Precautions (5.2)]. (…)<br>**8.2 Lactation Risk Summary**<br>A breastfed infant with G6PD deficiency is at risk for hemolytic anemia from exposure to ARAKODA. Infant G6PD status should be checked before breastfeeding begins. ARAKODA is contraindicated in breastfeeding women when the infant is found to be G6PD deficient or the G6PD status of the infant is unknown [see Contraindications (4) and Clinical Considerations].<br>There is no information regarding the presence of ARAKODA in human milk, the effects of the drug on the breastfed infant, or the effects of the drug on milk production. In a breastfed infant with normal G6PD, the developmental and health benefits of breastfeeding should be considered along with the mother's clinical need for ARAKODA and any potential effects on the breastfed infant from ARAKODA or from the underlying maternal condition.<br>**Clinical Considerations**<br>Check the infant's G6PD status before maternal breastfeeding commences. If an infant is G6PDdeficient, exposure to ARAKODA during breastfeeding may result in hemolytic anemia in the infant; therefore, advise the woman with an infant who has G6PD deficiency or whose G6PD status is unknown, not to breastfeed during treatment with ARAKODA and for 3 months after the final dose of ARAKODA.<br>**8.3 Females and Males of Reproductive Potential**<br>**Contraception**<br>ARAKODA may cause hemolytic anemia in a G6PD-deficient fetus [see Warnings and Precautions (5.2), Use in Specific Populations (8.1)]. Advise females of reproductive potential that treatment with ARAKODA during pregnancy is not recommended and to avoid pregnancy or use effective contraception for 3 months after the final dose of ARAKODA.<br><br>**17 PATIENT COUNSELING INFORMATION**<br>**G6PD Testing and Hemolytic Anemia**<br>Inform patients of the need for testing for G6PD deficiency before starting ARAKODA. Advise patients on the symptoms of hemolytic anemia and instruct them to seek medical advice promptly if such symptoms occur. Patients should contact their health care provider if they have darker lips or urine as these may be signs of hemolysis or methemoglobinemia [see Warnings and Precautions (5.1)].<br>**Lactation**<br>Advise women with a G6PD-deficient infant, or if they do not know the G6PD status of their infant, not to breastfeed during treatment with ARAKODA and for 3 months after the final dose [see Contraindication (4), Warnings and Precautions (5.2), Use in Specific Populations (8.2)].
**211651, 06/20/2023**	Talazoparib (1)	Oncology	BRCA	Indications and Usage, Dosage and Administration, Adverse Reactions, Clinical Studies	**1 INDICATIONS AND USAGE**<br>TALZENNA is indicated for the treatment of adult patients with deleterious or suspected deleterious germline breast cancer susceptibility gene (BRCA)-mutated (gBRCAm) human epidermal growth factor receptor 2 (HER2)-negative locally advanced or metastatic breast cancer. Select patients for therapy based on an FDA-approved companion diagnostic for TALZENNA [see Dosage and Administration (2.1)].<br><br>**2 DOSAGE AND ADMINISTRATION**<br>**2.1 Patient Selection**<br>Information on the FDA-approved tests for the detection of genetic mutations is available at http://www.fda.gov/companiondiagnostics.<br>gBRCAm HER2-negative Locally Advanced or Metastatic Breast Cancer<br>Select patients for the treatment of advanced breast cancer with TALZENNA based on the presence of germline BRCA mutations [see Indications and Usage (1.1), Clinical Studies (14.1)].<br>**2.2 Recommended Dosage for gBRCAm HER2-negative Locally Advanced or Metastatic Breast Cancer**<br>The recommended dosage of TALZENNA is 1 mg taken orally once daily, until disease progression or unacceptable toxicity.<br>**2.5 Dosage Modifications for Adverse Reactions**<br>gBRCAm HER2-negative Locally Advanced or Metastatic Breast Cancer<br>See Tables 1 and 3<br>**2.6 Recommended Dosage in Patients with Renal Impairment**<br>gBRCAm HER2-negative Locally Advanced or Metastatic Breast Cancer<br>The recommended dosage of TALZENNA for patients with moderate renal impairment (CLcr 30 - 59 mL/min) is 0.75 mg taken orally once daily [see Use in Specific Populations (8.7)].<br>The recommended dosage of TALZENNA for patients with severe renal impairment (CLcr 15 - 29 mL/min) is 0.5 mg taken orally once daily [see Use in Specific Populations (8.7)].<br>**2.7 Dosage Modifications for P-glycoprotein Inhibitors**<br>gBRCAm HER2-negative Locally Advanced or Metastatic Breast Cancer<br>Avoid coadministration of TALZENNA with the following P-glycoprotein (P-gp) inhibitors: itraconazole, amiodarone, carvedilol, clarithromycin, itraconazole, and verapamil. If coadministration of TALZENNA with these P-gp inhibitors cannot be avoided, reduce the dose of TALZENNA to 0.75 mg taken orally once daily. When the P-gp inhibitor is discontinued, increase the dose of TALZENNA (after 3 – 5 half-lives of the P-gp inhibitor) to the dose of TALZENNA that was used before starting the P-gp inhibitor [see Drug Interactions (7.1)].<br>Monitor for increased adverse reactions and modify the dosage as recommended for adverse reactions when TALZENNA is coadministered with other P-gp inhibitors [see Dosage and Administration (2.5)].

* Therapeutic areas do not necessarily reflect the CDER review division.
† Representative biomarkers are listed based on standard nomenclature as per the Human Genome Organization (HUGO) symbol and/or simplified descriptors using other common conventions. Listed biomarkers do not necessarily reflect the terminology used in labeling. The term "Nonspecific" is provided when labeling does not explicitly identify the specific biomarker(s) or when the biomarker is represented by a molecular phenotype or gene signature, and in some cases the biomarker was inferred based on the labeling language.
‡ Referenced figures and tables may be found in the labeling available at Drugs@FDA.
Blue text represents the most recent addtions and/or changes since last posted version.

NDA/ANDA/BLA Number, Label Version Date	Drug	Therapeutic Area*	Biomarker†	Labeling Sections	Labeling Text‡
					**6 ADVERSE REACTIONS**<br>**6.1 Clinical Trials Experience**<br>Treatment of gBRCAm HER2-negative Locally Advanced or Metastatic Breast Cancer<br>*EMBRACA*<br>The safety of TALZENNA as monotherapy was evaluated in gBRCAm patients with HER2-negative locally advanced or metastatic breast cancer who had previously received no more than 3 lines of chemotherapy for the treatment of locally advanced/metastatic disease. EMBRACA was a randomized, open-label, multi-center study in which 412 patients received either TALZENNA 1 mg once daily (n=286) or a chemotherapy agent (capecitabine, eribulin, gemcitabine, or vinorelbine) of the healthcare provider's choice (n=126) until disease progression or unacceptable toxicity. (…)<br><br>**14 CLINICAL STUDIES**<br>**14.1 Deleterious or Suspected Deleterious Germline BRCA-mutated HER2-negative Locally Advanced or Metastatic Breast Cancer**<br>EMBRACA (NCT01945775) was an open-label study in which patients (N=431) with gBRCAm HER2-negative locally advanced or metastatic breast cancer were randomized 2:1 to receive TALZENNA 1 mg or healthcare provider's choice of chemotherapy (capecitabine, eribulin, gemcitabine, or vinorelbine) until disease progression or unacceptable toxicity. Randomization was stratified by prior use of chemotherapy for metastatic disease (0 versus 1, 2, or 3), by triple-negative disease status (triple-negative breast cancer [TNBC] versus non-TNBC), and history of central nervous system (CNS) metastasis (yes versus no). (…)<br>(…) No prior treatment with a PARP inhibitor was permitted. Of the 431 patients randomized in the EMBRACA study, 408 (95%) were centrally confirmed to have a deleterious or suspected deleterious gBRCAm using a clinical trial assay; out of which 354 (82%) were confirmed using the BRACAnalysis CDx®. BRCA mutation status (breast cancer susceptibility gene 1 [BRCA1] positive or breast cancer susceptibility gene 2 [BRCA2] positive) was similar across both treatment arms. (…)
**211651, 06/20/2023**	Talazoparib (2)	Oncology	ERBB2 (HER2)	Indications and Usage, Dosage and Administration, Adverse Reactions, Clinical Studies	**1 INDICATIONS AND USAGE**<br>TALZENNA is indicated for the treatment of adult patients with deleterious or suspected deleterious germline breast cancer susceptibility gene (BRCA)-mutated (gBRCAm) human epidermal growth factor receptor 2 (HER2)-negative locally advanced or metastatic breast cancer. Select patients for therapy based on an FDA-approved companion diagnostic for TALZENNA [see Dosage and Administration (2.1)].<br><br>**2 DOSAGE AND ADMINISTRATION**<br>**2.1 Patient Selection**<br>Information on the FDA-approved tests for the detection of genetic mutations is available at http://www.fda.gov/companiondiagnostics.<br>gBRCAm HER2-negative Locally Advanced or Metastatic Breast Cancer<br>Select patients for the treatment of advanced breast cancer with TALZENNA based on the presence of germline BRCA mutations [see Indications and Usage (1.1), Clinical Studies (14.1)].<br>**2.2 Recommended Dosage for gBRCAm HER2-negative Locally Advanced or Metastatic Breast Cancer**<br>The recommended dosage of TALZENNA is 1 mg taken orally once daily, until disease progression or unacceptable toxicity.<br>**2.5 Dosage Modifications for Adverse Reactions**<br>gBRCAm HER2-negative Locally Advanced or Metastatic Breast Cancer<br>See Tables 1 and 3<br>**2.6 Recommended Dosage in Patients with Renal Impairment**<br>gBRCAm HER2-negative Locally Advanced or Metastatic Breast Cancer<br>The recommended dosage of TALZENNA for patients with moderate renal impairment (CLcr 30 - 59 mL/min) is 0.75 mg taken orally once daily [see Use in Specific Populations (8.7)].<br>The recommended dosage of TALZENNA for patients with severe renal impairment (CLcr 15 - 29 mL/min) is 0.5 mg taken orally once daily [see Use in Specific Populations (8.7)].<br>**2.7 Dosage Modifications for P-glycoprotein Inhibitors**<br>gBRCAm HER2-negative Locally Advanced or Metastatic Breast Cancer<br>Avoid coadministration of TALZENNA with the following P-glycoprotein (P-gp) inhibitors: itraconazole, amiodarone, carvedilol, clarithromycin, itraconazole, and verapamil. If coadministration of TALZENNA with these P-gp inhibitors cannot be avoided, reduce the dose of TALZENNA to 0.75 mg taken orally once daily. When the P-gp inhibitor is discontinued, increase the dose of TALZENNA (after 3 – 5 half-lives of the P-gp inhibitor) to the dose of TALZENNA that was used before starting the P-gp inhibitor [see Drug Interactions (7.1)].<br>Monitor for increased adverse reactions and modify the dosage as recommended for adverse reactions when TALZENNA is coadministered with other P-gp inhibitors [see Dosage and Administration (2.5)].<br><br>**6 ADVERSE REACTIONS**<br>**6.1 Clinical Trials Experience**<br>Treatment of gBRCAm HER2-negative Locally Advanced or Metastatic Breast Cancer<br>*EMBRACA*<br>The safety of TALZENNA as monotherapy was evaluated in gBRCAm patients with HER2-negative locally advanced or metastatic breast cancer who had previously received no more than 3 lines of chemotherapy for the treatment of locally advanced/metastatic disease. EMBRACA was a randomized, open-label, multi-center study in which 412 patients received either TALZENNA 1 mg once daily (n=286) or a chemotherapy agent (capecitabine, eribulin, gemcitabine, or vinorelbine) of the healthcare provider's choice (n=126) until disease progression or unacceptable toxicity. (…)

* Therapeutic areas do not necessarily reflect the CDER review division.
† Representative biomarkers are listed based on standard nomenclature as per the Human Genome Organization (HUGO) symbol and/or simplified descriptors using other common conventions. Listed biomarkers do not necessarily reflect the terminology used in labeling. The term "Nonspecific" is provided when labeling does not explicitly identify the specific biomarker(s) or when the biomarker is represented by a molecular phenotype or gene signature, and in some cases the biomarker was inferred based on the labeling language.
‡ Referenced figures and tables may be found in the labeling available at Drugs@FDA.
Blue text represents the most recent addtions and/or changes since last posted version.

NDA/ANDA/BLA Number, Label Version Date	Drug	Therapeutic Area*	Biomarker†	Labeling Sections	Labeling Text‡
					**14 CLINICAL STUDIES**<br>**14.1 Deleterious or Suspected Deleterious Germline BRCA-mutated HER2-negative Locally Advanced or Metastatic Breast Cancer**<br>EMBRACA (NCT01945775) was an open-label study in which patients (N=431) with gBRCAm HER2-negative locally advanced or metastatic breast cancer were randomized 2:1 to receive TALZENNA 1 mg or healthcare provider's choice of chemotherapy (capecitabine, eribulin, gemcitabine, or vinorelbine) until disease progression or unacceptable toxicity. Randomization was stratified by prior use of chemotherapy for metastatic disease (0 versus 1, 2, or 3), by triple-negative disease status (triple-negative breast cancer [TNBC] versus non-TNBC), and history of central nervous system (CNS) metastasis (yes versus no). (…)
**211651, 06/20/2023**	Talazoparib (3)	Oncology	Homologous Recombination Repair	Indications and Usage, Dosage and Administrations, Adverse Reactions, Drug Interactions, Clinical Studies	**1 INDICATIONS AND USAGE**<br>**1.3 HRR Gene-mutated mCRPC**<br>TALZENNA is indicated in combination with enzalutamide for the treatment of adult patients with homologous recombination repair (HRR) gene-mutated metastatic castration-resistant prostate cancer (mCRPC) [see Dosage and Administration (2.3)].<br><br>**2 DOSAGE AND ADMINISTRATION**<br>**2.1 Patient Selection**<br>Information on the FDA-approved tests for the detection of genetic mutations is available at http://www.fda.gov/companiondiagnostics.<br>HRR Gene-mutated Metastatic Castration-Resistant Prostate Cancer<br>Select patients for the treatment of HRR gene-mutated mCRPC with TALZENNA based on the presence of HRR gene mutations (ATM, ATR, BRCA1, BRCA2, CDK12, CHEK2, FANCA, MLH1, MRE11A, NBN, PALB2, or RAD51C) [see Indications and Usage (1.2), Clinical Studies (14.2)].<br>An FDA-approved test for the detection of HRR gene mutations for use with TALZENNA is not currently available.<br>**2.3 Recommended Dosage for HRR Gene-mutated mCRPC**<br>The recommended dosage of TALZENNA is 0.5 mg taken orally once daily in combination with enzalutamide until disease progression or unacceptable toxicity. Refer to the enzalutamide prescribing information for recommended enzalutamide dosing information. Patients receiving TALZENNA and enzalutamide should also receive a gonadotropin-releasing hormone (GnRH) analog concurrently or should have had bilateral orchiectomy.<br>**2.5 Dosage Modifications for Adverse Reactions**<br>HRR Gene-mutated mCRPC<br>See Tables 2 and 3<br>**2.6 Recommended Dosage in Patients with Renal Impairment**<br>HRR Gene-mutated mCRPC<br>The recommended dosage of TALZENNA for patients with moderate renal impairment (CLcr 30 - 59 mL/min) is 0.35 mg taken orally once daily in combination with enzalutamide [see Use in Specific Populations (8.7)].<br>The recommended dosage of TALZENNA for patients with severe renal impairment (CLcr 15 - 29 mL/min) is 0.25 mg taken orally once daily in combination with enzalutamide [see Use in Specific Populations (8.7)].<br><br>**6 ADVERSE REACTIONS**<br>**6.1 Clinical Trials Experience**<br>HRR Gene-mutated mCRPC<br>The safety of TALZENNA in combination with enzalutamide was evaluated in patients with HRR gene-mutated mCRPC enrolled in TALAPRO-2 [see Clinical Studies (14.2)]. Patients were randomized to receive either TALZENNA 0.5 mg in combination with enzalutamide 160 mg once daily (n=197), or placebo in enzalutamide 160 mg once daily (n=199) until disease progression or unacceptable toxicity. Among patients receiving TALZENNA, 86% were exposed for 6 months or longer, 60% were exposed for greater than one year, and 18% were exposed for greater than two years. (…)<br><br>**7 DRUG INTERACTIONS**<br>**7.1 Effect of Other Drugs on TALZENNA**<br>*Effect of P-gp Inhibitors*<br>HRR Gene-mutated mCRPC<br>The effect of coadministration of P-gp inhibitors on talazoparib exposure when TALZENNA is taken in combination with enzalutamide has not been studied. Monitor patients for increased adverse reactions and modify the dosage as recommended for adverse reactions when TALZENNA is coadministered with a P-gp inhibitor [see Dosage and Administration (2.5)].<br><br>**14 CLINICAL STUDIES**<br>**14.2 HRR Gene-mutated mCRPC**<br>The efficacy of TALZENNA in combination with enzalutamide was evaluated in TALAPRO-2 (NCT03395197), a randomized, double-blind, placebo-controlled, multi-cohort trial in which 399 patients with HRR gene-mutated (HRRm) mCRPC were randomized 1:1 to receive enzalutamide 160 mg daily plus either TALZENNA 0.5 mg or placebo daily until unacceptable toxicity or progression. All patients received a GnRH analog or had prior bilateral orchiectomy and needed to have progressed on prior androgen deprivation therapy. Prior treatment with a CYP17 inhibitor or docetaxel for metastatic castration-sensitive prostate cancer (mCSPC) was permitted. Mutation status of HRR genes was determined prospectively using solid tumor tissue or circulating tumor DNA (ctDNA)-based next generation sequencing assays. Patients were required to have a mutation in at least one of 12 genes involved in the HRR pathway (ATM, ATR, BRCA1, BRCA2, CDK12, CHEK2, FANCA, MLH1, MRE11A, NBN, PALB2, or RAD51C).<br>Randomization was stratified by previous treatment with a CYP17 inhibitor or docetaxel (yes/no).

* Therapeutic areas do not necessarily reflect the CDER review division.
† Representative biomarkers are listed based on standard nomenclature as per the Human Genome Organization (HUGO) symbol and/or simplified descriptors using other common conventions. Listed biomarkers do not necessarily reflect the terminology used in labeling. The term "Nonspecific" is provided when labeling does not explicitly identify the specific biomarker(s) or when the biomarker is represented by a molecular phenotype or gene signature, and in some cases the biomarker was inferred based on the labeling language.
‡ Referenced figures and tables may be found in the labeling available at Drugs@FDA.
Blue text represents the most recent addtions and/or changes since last posted version.

NDA/ANDA/BLA Number, Label Version Date	Drug	Therapeutic Area*	Biomarker†	Labeling Sections	Labeling Text‡
					The median age was 70 years (range: 41 to 90); 100% were male; 68% were White, 21% Asian, 2.8% Black, 0.8% Other, 7% unknown/not reported; 12% were Hispanic/Latino; and baseline ECOG performance status was 0 (62%) or 1 (38%). Thirty-nine percent of patients had bone-only disease; 15% had visceral disease. In the mCSPC setting, 29% percent of patients had received docetaxel and 9% had received a prior CYP17 inhibitor. The most commonly mutated HRR genes (>5%), including co-occurring mutations, were: BRCA2 (34%), ATM (22%), CDK12 (19%), CHEK2 (18%), and BRCA1 (6%). (See Tables 10 and 11, Figure 3)
**021807, 04/08/2019**	Tamoxifen (1)	Oncology	ESR, PGR (Hormone Receptor)	Indications and Usage, Adverse Reactions, Clinical Pharmacology, Clinical Studies	**1 INDICATIONS AND USAGE**<br>**1.1 Metastatic Breast Cancer**<br>SOLTAMOX is indicated for the treatment of adult patients with estrogen receptor-positive metastatic breast cancer.<br>**1.2 Adjuvant Treatment of Breast Cancer**<br>SOLTAMOX is indicated:<br>• for the adjuvant treatment of adult patients with early stage estrogen receptor-positive breast cancer<br>• to reduce the occurrence of contralateral breast cancer in adult patients when used as adjuvant therapy for the treatment of breast cancer.<br><br>**6 ADVERSE REACTIONS**<br>**6.1 Clinical Trials Experience**<br>*Anastrozole Adjuvant Trial (ATAC: Arimidex, Tamoxifen, Alone or in Combination) – Study of Anastrozole Compared to Tamoxifen for Adjuvant Treatment of Early Breast Cancer*<br>At a median follow-up of 33 months, the combination of anastrozole and tamoxifen did not demonstrate an efficacy benefit when compared to tamoxifen monotherapy in all patients as well as in the hormone receptorpositive subpopulation. The combination treatment arm was discontinued from the trial. The median duration of adjuvant treatment for safety evaluation was 59.8 months and 59.6 months for patients receiving anastrozole 1 mg and tamoxifen 20 mg monotherapy, respectively. (…)<br><br>**12 CLINICAL PHARMACOLOGY**<br>**12.5 Pharmacogenomics**<br>The impact of CYP2D6 polymorphisms on the efficacy of tamoxifen is not well established. CYP2D6 poor metabolizers carrying two non-functional alleles exhibit significantly lower endoxifen plasma concentrations compared to patients carrying one or more fully functional alleles of CYP2D6. In patients with estrogen receptor-positive breast cancer who were participating in the WHEL (Women's Health Eating and Living) Study (NCT00003787), the mean (SD) serum concentration of endoxifen was 22.8 (11.3), 15.9 (9.2), 8.1 (4.9) and 5.6 (3.8) ng/mL in 27 ultrarapid, 1,097 normal, 164 intermediate and 82 poor metabolizers (p<0.0001), respectively. This finding is consistent with other published studies that report lower endoxifen concentrations in poor metabolizers compared to normal metabolizers.<br><br>**14 CLINICAL STUDIES**<br>**14.2 Adjuvant Treatment of Breast Cancer**<br><u>Pooled Studies of Adjuvant Treatment of Breast Cancer</u><br>The Early Breast Cancer Trialists' Collaborative Group (EBCTCG) conducted worldwide overviews of systemic adjuvant therapy for early breast cancer in 1985, 1990, 1995, 1998 and 2011. The 10-year outcome data were reported in 1998 for 36,689 women in 55 randomized trials of another formulation of adjuvant tamoxifen using doses of 20 to 40 mg per day for 1 to 5+ years. Twenty-five percent of patients received 1 year or less of trial treatment, 52% received 2 years, and 23% received about 5 years. Fortyeight percent of tumors were estrogen receptor (ER)- positive (>10 fmol/mg), 21% were ER-poor (<10 fmol/mg), and 31% were ER-unknown. Among 29,441 patients with ER-positive or ER-unknown breast cancer, 58% were entered into trials comparing tamoxifen to no adjuvant therapy and 42% were entered into trials comparing tamoxifen in combination with chemotherapy vs. the same chemotherapy alone. Among these patients, 54% had node-positive disease and 46% had node-negative disease.<br>*In women with ER-positive or ER-unknown breast cancer:*<br>• With positive nodes who received about 5 years of treatment, overall survival at 10 years was 61.4% for tamoxifen vs. 50.5% for control (log-rank 2p <0.00001). The recurrence-free rate at 10 years was 59.7% for tamoxifen vs. 44.5% for control (log-rank 2p <0.00001).<br>• With negative nodes who received about 5 years of treatment, overall survival at 10 years was 78.9% for tamoxifen vs. 73.3% for control (log-rank 2p <0.00001). The recurrence-free rate at 10 years was 79.2% for tamoxifen vs. 64.3% for control (log-rank 2p <0.00001).<br>• Who received 1 year or less, 2 years, or about 5 years of tamoxifen, the proportional reductions in mortality were 12%, 17%, and 26%, respectively (2p <0.003). The corresponding reductions in breast cancer recurrence were 21%, 29%, and 47% (2p <0.00001).<br>Results in patients with ER-poor breast cancer<br>• Benefit is less clear for women with ER-poor breast cancer in whom the proportional reduction in recurrence was 10% (2p = 0.007) for all durations taken together, or 9% (2p = 0.02) if contralateral breast cancers are excluded. The corresponding reduction in mortality was 6% (not significant).<br><u>Node-positive: Individual Studies</u><br>(…) In the Hubay study, patients with a positive (more than 3 fmol) estrogen receptor were more likely to benefit. In the NSABP B-09 study in women age 50 to 59 years, only women with both estrogen and progesterone receptor levels 10 fmol or greater clearly benefited, while survival results were poorer in women with both estrogen and progesterone receptor levels less than 10 fmol. In women age 60 to 70 years, there was an improvement in disease-free survival with tamoxifen without any clear relationship to estrogen or progesterone receptor status. (…)<br><u>Node–negative: Individual Studies</u><br>NSABP B-14, a prospective, double-blind, randomized study, compared another formulation of tamoxifen to placebo as adjuvant therapy in women with axillary node-negative, estrogen-receptor positive (≥10 fmol/mg cytosol protein) breast cancer (following total mastectomy and axillary dissection, or segmental

* Therapeutic areas do not necessarily reflect the CDER review division.
† Representative biomarkers are listed based on standard nomenclature as per the Human Genome Organization (HUGO) symbol and/or simplified descriptors using other common conventions. Listed biomarkers do not necessarily reflect the terminology used in labeling. The term "Nonspecific" is provided when labeling does not explicitly identify the specific biomarker(s) or when the biomarker is represented by a molecular phenotype or gene signature, and in some cases the biomarker was inferred based on the labeling language.
‡ Referenced figures and tables may be found in the labeling available at Drugs@FDA.
Blue text represents the most recent addtions and/or changes since last posted version.

NDA/ANDA/BLA Number, Label Version Date	Drug	Therapeutic Area*	Biomarker†	Labeling Sections	Labeling Text‡
					resection, axillary dissection, and breast radiation). After five years of treatment, there was a significant improvement in disease-free survival in women receiving tamoxifen. This benefit was apparent both in women under age 50 and in women at or beyond age 50.<br>One additional randomized study (NATO) demonstrated improved disease-free survival for another formulation of tamoxifen compared to no adjuvant therapy following total mastectomy and axillary dissection in postmenopausal women with axillary node-negative breast cancer. In this study, the benefits of tamoxifen appeared to be independent of estrogen receptor status.<br>Anastrozole Adjuvant Trial (ATAC: Arimidex, Tamoxifen, Alone or in Combination) – Study of Anastrozole Compared to Tamoxifen for Adjuvant Treatment of Early Breast Cancer<br>A trial was conducted in 9,366 postmenopausal women with operable breast cancer who were randomized to receive adjuvant treatment with either anastrozole 1 mg daily, another formulation of tamoxifen 20 mg daily, or a combination of these two treatments for 5 years or until recurrence of the disease. At a median follow-up of 33 months, the combination of anastrozole and tamoxifen did not demonstrate any efficacy benefit when compared to tamoxifen alone in all patients, as well as in the hormone receptor-positive subpopulation. The combination treatment arm was discontinued from the trial [see Drug Interactions (7.1) and Clinical Pharmacology (12.3)]. Refer to the full prescribing information for anastrozole tablets for additional information on this trial. (…)<br>**14.4 Reduction in Breast Cancer Incidence in Women at High Risk**<br>Breast Cancer Prevention Trial (NSABP P-1)<br>(…) Table 9 describes the characteristics of the breast cancers in the NSABP P-1 trial in women at high risk for breast cancer. Tamoxifen decreased the incidence of small estrogen receptor-positive tumors, but did not alter the incidence of estrogen receptor-negative tumors or larger tumors. (See Table 9) (…)
**021807, 04/08/2019**	Tamoxifen (2)	Oncology	F5<br>(Factor V Leiden)	Warnings and Precautions	**5 WARNINGS AND PRECAUTIONS**<br>**5.2 Thromboembolic Events**<br>(…) In a small substudy (N = 81) of the NSABP-1 trial, there appeared to be no benefit to screening women for Factor V Leiden and Prothrombin mutations G20210A as a means to identify those who may not be appropriate candidates for tamoxifen therapy. (…)
**021807, 04/08/2019**	Tamoxifen (3)	Oncology	F2<br>(Prothrombin)	Warnings and Precautions	**5 WARNINGS AND PRECAUTIONS**<br>**5.2 Thromboembolic Events**<br>(…) In a small substudy (N = 81) of the NSABP-1 trial, there appeared to be no benefit to screening women for Factor V Leiden and Prothrombin mutations G20210A as a means to identify those who may not be appropriate candidates for tamoxifen therapy. (…)
**021807, 04/08/2019**	Tamoxifen (4)	Oncology	CYP2D6	Clinical Pharmacology	**12 CLINICAL PHARMACOLOGY**<br>**12.3 Pharmacokinetics**<br>Metabolism<br>Tamoxifen is extensively metabolized by CYP450 enzymes, including CYP3A, CYP2D6, CYP2C9, CYP2C19, and CYP2B6. N-desmethyltamoxifen, formed predominantly by CYP3A, is the major metabolite found in plasma. The pharmacological activity of N-desmethyltamoxifen is similar to that of tamoxifen. Endoxifen and 4-hydroxytamoxifen, identified as minor metabolites, have 100-fold greater affinity for the estrogen receptor and 30 to 100-fold greater potency in suppressing estrogen-dependent cell proliferation than tamoxifen. The polymorphic enzyme CYP2D6 is involved in the formation of endoxifen and 4-hydroxytamoxifen, and it is the key enzyme that catalyzes the formation of endoxifen from N-desmethyltamoxifen. Endoxifen concentrations may differ among patients because of various CYP2D6 genotypes [see Clinical Pharmacology (12.5)]. Phase 2 enzymes, such as SULT1A1, UGT2B7, and UGT1A4, are associated with tamoxifen clearance from plasma.<br>Drug-Drug Interactions<br>*CYP2D6 Inhibitors*<br>CYP2D6 Inhibitors Although concomitant administration of CYP2D6 inhibitors reduces the plasma concentration of endoxifen, a potent metabolite, the clinical significance is not well established [see Drug Interactions (7.4)]. The mean steady-state endoxifen plasma concentration in patients taking CYP2D6 inhibitors was significantly reduced compared to those not taking concomitant CYP2D6 inhibitors (14.8 ± 10.6 versus 26.7 ± 15.4 ng/mL). The mean steady-state plasma concentration of endoxifen in CYP2D6 normal metabolizers who were not receiving CYP2D6 inhibitors was 31.4 ± 14.7 ng/mL compared to 8.8 ± 3.5 ng/mL in CYP2D6 normal metabolizers receiving potent CYP2D6 inhibitors (e.g., paroxetine, fluoxetine) with tamoxifen. The plasma levels of endoxifen in CYP2D6 normal metabolizers taking potent CYP2D6 inhibitors were similar to the levels observed in CYP2D6 poor metabolizers taking no CYP2D6 inhibitors (8.8 versus 7.2 ng/mL).<br>**12.5 Pharmacogenomics**<br>The impact of CYP2D6 polymorphisms on the efficacy of tamoxifen is not well established.<br>CYP2D6 poor metabolizers carrying two non-functional alleles exhibit significantly lower endoxifen plasma concentrations compared to patients carrying one or more fully functional alleles of CYP2D6.<br>In patients with estrogen receptor-positive breast cancer who were participating in the WHEL (Women's Health Eating and Living) Study (NCT00003787), the mean (SD) serum concentration of endoxifen was 22.8 (11.3), 15.9 (9.2), 8.1 (4.9) and 5.6 (3.8) ng/mL in 27 ultrarapid, 1,097 normal, 164 intermediate and 82 poor metabolizers (p<0.0001), respectively. This finding is consistent with other published studies that report lower endoxifen concentrations in poor metabolizers compared to normal metabolizers.
**020579, 01/23/2019**	Tamsulosin	Urology	CYP2D6	Warnings and Precautions, Adverse Interactions, Clinical Pharmacology	**5 WARNINGS AND PRECAUTIONS**<br>**5.2 Drug Interactions**<br>Tamsulosin is extensively metabolized, mainly by CYP3A4 and CYP2D6. FLOMAX capsules 0.4 mg should not be used in combination with strong inhibitors of CYP3A4 (e.g., ketoconazole) [see Drug Interactions (7.1) and Clinical Pharmacology (12.3)]. FLOMAX capsules should be used with caution in combination with moderate inhibitors of CYP3A4 (e.g., erythromycin), in combination with strong (e.g., paroxetine) or moderate (e.g., terbinafine) inhibitors of CYP2D6, in patients known to be CYP2D6 poor metabolizers particularly at a dose higher than 0.4 mg (e.g., 0.8 mg) [see Drug Interactions (7.1) and Clinical Pharmacology (12.3)].<br>(…)

* Therapeutic areas do not necessarily reflect the CDER review division.
† Representative biomarkers are listed based on standard nomenclature as per the Human Genome Organization (HUGO) symbol and/or simplified descriptors using other common conventions. Listed biomarkers do not necessarily reflect the terminology used in labeling. The term "Nonspecific" is provided when labeling does not explicitly identify the specific biomarker(s) or when the biomarker is represented by a molecular phenotype or gene signature, and in some cases the biomarker was inferred based on the labeling language.
‡ Referenced figures and tables may be found in the labeling available at Drugs@FDA.
Blue text represents the most recent addtions and/or changes since last posted version.

NDA/ANDA/BLA Number, Label Version Date	Drug	Therapeutic Area*	Biomarker†	Labeling Sections	Labeling Text‡
					**7 DRUG INTERACTIONS**<br>**7.1 Cytochrome P450 Inhibition Strong and Moderate Inhibitors of CYP3A4 or CYP2D6**<br>(…) Concomitant treatment with paroxetine (a strong inhibitor of CYP2D6) resulted in an increase in the Cmax and AUC of tamsulosin by a factor of 1.3 and 1.6, respectively [see Warnings and Precautions (5.2) and Clinical Pharmacology (12.3)]. A similar increase in exposure is expected in CYP2D6 poor metabolizers (PM) as compared to extensive metabolizers (EM). Since CYP2D6 PMs cannot be readily identified and the potential for significant increase in tamsulosin exposure exists when FLOMAX 0.4 mg is co-administered with strong CYP3A4 inhibitors in CYP2D6 PMs, FLOMAX 0.4 mg capsules should not be used in combination with strong inhibitors of CYP3A4 (e.g., ketoconazole) [see Warnings and Precautions (5.2) and Clinical Pharmacology (12.3)]. (…)<br><br>**12 CLINICAL PHARMACOLOGY**<br>**12.3 Pharmacokinetics**<br>**Drug Interactions**<br>**Cytochrome P450 Inhibition**<br>**Strong and Moderate Inhibitors of CYP3A4 or CYP2D6**<br>(…) The effects of paroxetine (a strong inhibitor of CYP2D6) at 20 mg once daily for 9 days on the pharmacokinetics of a single FLOMAX capsule 0.4 mg dose was investigated in 24 healthy volunteers (age range 23 to 47 years). Concomitant treatment with paroxetine resulted in an increase in the Cmax and AUC of tamsulosin by a factor of 1.3 and 1.6, respectively [see Warnings and Precautions (5.2) and Drug Interactions (7.1)]. A similar increase in exposure is expected in CYP2D6 poor metabolizers (PM) as compared to extensive metabolizers (EM). A fraction of the population (about 7% of Caucasians and 2% of African Americans) are CYP2D6 PMs. Since CYP2D6 PMs cannot be readily identified and the potential for significant increase in tamsulosin exposure exists when FLOMAX 0.4 mg is co-administered with strong CYP3A4 inhibitors in CYP2D6 PMs, FLOMAX 0.4 mg capsules should not be used in combination with strong inhibitors of CYP3A4 (e.g., ketoconazole) [see Warnings and Precautions (5.2) and Drug Interactions (7.1)]. (…)
**761228, 01/25/2022**	Tebentafusp-tebn	Oncology	HLA-A	Indications and Usage, Dosage and Administration, Clinical Studies	**1 INDICATIONS AND USAGE**<br>KIMMTRAK is indicated for the treatment of HLA-A*02:01-positive adult patients with unresectable or metastatic uveal melanoma.<br><br>**2 DOSAGE AND ADMINISTRATION**<br>**2.1 Patient Selection**<br>Select patients for treatment of unresectable or metastatic uveal melanoma with KIMMTRAK based on a positive HLA-A*02:01 genotyping test [see Clinical Studies (14)]. An FDA-approved test for the detection of HLA-A*02:01 genotyping is not currently available.<br><br>**14 CLINICAL STUDIES**<br>Study IMCgp100-202: First line metastatic uveal melanoma<br>KIMMTRAK was evaluated in IMCgp100-202, a randomized, open-label, multicenter trial (NCT03070392) that enrolled patients with metastatic uveal melanoma (N=378). Patients were required to be HLA-A*02:01 genotype positive identified by a central assay. (…)
761291, 10/25/2022	**Teclistamab-cqyv (1)**	Oncology	Chromosome 17p	Clinical Studies	**14 CLINICAL STUDIES**<br>(…) The efficacy population included 110 patients. The median age was 66 (range: 33 to 82) years with 16% of patients 75 years of age or older; 56% were male; 91% were White, 5% were Black or African American, 3% were Asian. The International Staging System (ISS) at study entry was Stage I in 50%, Stage II in 38%, and Stage III in 12% of patients. High-risk cytogenetics (presence of del(17p), t(4;14) and t(14;16)) were present in 25% of patients. Seventeen percent of patients had extramedullary plasmacytomas. Patients with prior BCMA-targeted therapy were not included in the efficacy population. (…)
761291, 10/25/2022	**Teclistamab-cqyv (2)**	Oncology	Chromosome 4p;14q	Clinical Studies	**14 CLINICAL STUDIES**<br>(…) The efficacy population included 110 patients. The median age was 66 (range: 33 to 82) years with 16% of patients 75 years of age or older; 56% were male; 91% were White, 5% were Black or African American, 3% were Asian. The International Staging System (ISS) at study entry was Stage I in 50%, Stage II in 38%, and Stage III in 12% of patients. High-risk cytogenetics (presence of del(17p), t(4;14) and t(14;16)) were present in 25% of patients. Seventeen percent of patients had extramedullary plasmacytomas. Patients with prior BCMA-targeted therapy were not included in the efficacy population. (…)
761291, 10/25/2022	**Teclistamab-cqyv (3)**	Oncology	Chromosome 14q;16q	Clinical Studies	**14 CLINICAL STUDIES**<br>(…) The efficacy population included 110 patients. The median age was 66 (range: 33 to 82) years with 16% of patients 75 years of age or older; 56% were male; 91% were White, 5% were Black or African American, 3% were Asian. The International Staging System (ISS) at study entry was Stage I in 50%, Stage II in 38%, and Stage III in 12% of patients. High-risk cytogenetics (presence of del(17p), t(4;14) and t(14;16)) were present in 25% of patients. Seventeen percent of patients had extramedullary plasmacytomas. Patients with prior BCMA-targeted therapy were not included in the efficacy population. (…)
**201917, 10/28/2013**	Telaprevir	Infectious Diseases	IFNL3 (IL28B)	Clinical Pharmacology, Clinical Studies	**12 CLINICAL PHARMACOLOGY**<br>**12.5 Pharmacogenomics**<br>A genetic variant near the gene encoding interferon-lambda-3 (IL28B rs12979860, a C to T change) is a strong predictor of response to peginterferon alfa and ribavirin (PR). rs12979860 was genotyped in 454 of 1088 subjects in Trial 108 (treatment-naïve) and 527 of 662 subjects in Trial C216 (previously treated) [see Clinical Studies (14.2 and 14.3) for trial descriptions]. SVR rates tended to be lower in subjects with the CT and TT genotypes compared to those with the CC genotype, particularly among treatment-naïve subjects receiving PR48 (Table 9). Among both treatment-naïve and previous treatment failures, subjects of all IL28B genotypes appeared to have higher SVR rates with regimens containing INCIVEK. The results of this retrospective subgroup analysis should be viewed with caution because of the small sample size and potential differences in demographic or clinical characteristics of the subtrial population relative to the overall trial population. In Trial C211, all subjects were prospectively tested for IL28B variants; there were no clinically relevant differences in SVR12 responses between q8h and twice-daily dosing within the genetic subgroups. (See Table 9)<br><br>**14 CLINICAL STUDIES**

* Therapeutic areas do not necessarily reflect the CDER review division.
† Representative biomarkers are listed based on standard nomenclature as per the Human Genome Organization (HUGO) symbol and/or simplified descriptors using other common conventions. Listed biomarkers do not necessarily reflect the terminology used in labeling. The term "Nonspecific" is provided when labeling does not explicitly identify the specific biomarker(s) or when the biomarker is represented by a molecular phenotype or gene signature, and in some cases the biomarker was inferred based on the labeling language.
‡ Referenced figures and tables may be found in the labeling available at Drugs@FDA.
Blue text represents the most recent addtions and/or changes since last posted version.

NDA/ANDA/BLA Number, Label Version Date	Drug	Therapeutic Area*	Biomarker†	Labeling Sections	Labeling Text‡
					**14.2 Treatment-Naïve Adults**<br>*Trial C211 (OPTIMIZE)*<br>(…) SVR rates were similar for the T12 (twice daily)/PR and T12 (q8h)/PR groups across subgroups determined by sex, age, race, ethnicity, body mass index, HCV genotype subtype, IL28B genotype, baseline HCV RNA (less than 800,000, greater than or equal to 800,000 IU per mL), and extent of liver fibrosis. However, there were small numbers of subjects enrolled in some key subgroups. (…)
**214096, 02/15/2024**	**Tepotinib (1)**	Oncology	ALK	Clinical Studies	**14 CLINICAL STUDIES**<br>The efficacy of TEPMETKO was evaluated in a single-arm, open-label, multicenter, non-randomized, multicohort study (VISION, NCT02864992). Eligible patients were required to have advanced or metastatic NSCLC harboring METex14 skipping alterations, epidermal growth factor receptor (EGFR) wild-type and anaplastic lymphoma kinase (ALK) negative status, at least one measurable lesion as defined by Response Evaluation Criteria in Solid Tumors (RECIST) version 1.1, and Eastern Cooperative Oncology Group (ECOG) Performance Status of 0 to 1. Patients with symptomatic CNS metastases, clinically significant uncontrolled cardiac disease, or who received treatment with any MET or hepatocyte growth factor (HGF) inhibitor were not eligible for the study. (…)
**214096, 02/15/2024**	**Tepotinib (2)**	Oncology	EGFR	Clinical Studies	**14 CLINICAL STUDIES**<br>The efficacy of TEPMETKO was evaluated in a single-arm, open-label, multicenter, non-randomized, multicohort study (VISION, NCT02864992). Eligible patients were required to have advanced or metastatic NSCLC harboring METex14 skipping alterations, epidermal growth factor receptor (EGFR) wild-type and anaplastic lymphoma kinase (ALK) negative status, at least one measurable lesion as defined by Response Evaluation Criteria in Solid Tumors (RECIST) version 1.1, and Eastern Cooperative Oncology Group (ECOG) Performance Status of 0 to 1. Patients with symptomatic CNS metastases, clinically significant uncontrolled cardiac disease, or who received treatment with any MET or hepatocyte growth factor (HGF) inhibitor were not eligible for the study. (…)
**214096, 02/15/2024**	**Tepotinib (3)**	Oncology	MET	Indications and Usage, Dosage and Administration, Adverse Reactions, Use in Specific Populations, Clinical Studies	**1 INDICATIONS AND USAGE**<br>TEPMETKO is indicated for the treatment of adult patients with metastatic non-small cell lung cancer (NSCLC) harboring mesenchymal-epithelial transition (MET) exon 14 skipping alterations.<br><br>**2 DOSAGE AND ADMINISTRATION**<br>**2.1 Patient Selection for METex14 Skipping Alterations**<br>Select patients for treatment with TEPMETKO based on the presence of MET exon 14 skipping alterations in plasma or tumor specimens. Testing for the presence of MET exon 14 skipping alterations in plasma specimens is recommended only in patients for whom a tumor biopsy cannot be obtained. If an alteration is not detected in a plasma specimen, re-evaluate the feasibility of biopsy for tumor tissue testing. An FDA-approved test for detection of MET exon 14 skipping alterations in NSCLC for selecting patients for treatment with TEPMETKO is not available.<br><br>**6 ADVERSE REACTIONS**<br>**6.1 Clinical Trials Experience**<br>The pooled safety population described in the WARNINGS AND PRECAUTIONS reflect exposure to TEPMETKO in 506 patients with solid tumors enrolled in five open-label, single-arm studies receiving TEPMETKO as single agent at a dose of 450 mg once daily. This included 313 patients with NSCLC positive for METex14 skipping alterations, who received TEPMETKO in VISION. Among 506 patients who received TEPMETKO, 44% were exposed for 6 months or longer, and 22% were exposed for more than one year.<br>The data described below reflect exposure to TEPMETKO 450 mg once daily in 313 patients with metastatic non-small cell lung cancer (NSCLC) with METex14 skipping alterations in VISION [see Clinical Studies (14)]. (…)<br><br>**8 USE IN SPECIFIC POPULATIONS**<br>**8.5 Geriatric Use**<br>Of 313 patients with NSCLC positive for METex14 skipping alterations in VISION who received 450 mg TEPMETKO once daily, 79% were 65 years or older, and 41% were 75 years or older. No clinically important differences in safety or efficacy were observed between patients aged 65 years or older and younger patients.<br><br>**14 CLINICAL STUDIES**<br>The efficacy of TEPMETKO was evaluated in a single-arm, open-label, multicenter, non-randomized, multicohort study (VISION, NCT02864992). Eligible patients were required to have advanced or metastatic NSCLC harboring METex14 skipping alterations, epidermal growth factor receptor (EGFR) wild-type and anaplastic lymphoma kinase (ALK) negative status, at least one measurable lesion as defined by Response Evaluation Criteria in Solid Tumors (RECIST) version 1.1, and Eastern Cooperative Oncology Group (ECOG) Performance Status of 0 to 1. Patients with symptomatic CNS metastases, clinically significant uncontrolled cardiac disease, or who received treatment with any MET or hepatocyte growth factor (HGF) inhibitor were not eligible for the study.<br>Identification of METex14 skipping alterations was prospectively determined using central laboratories employing either a PCR-based or next-generation sequencing-based clinical trial assay using tissue (66%) and/or plasma (57%) samples. (…)
**021894, 09/13/2017**	**Tetrabenazine**	Neurology	CYP2D6	Dosage and Administration, Warnings and Precautions, Use in Specific Populations, Clinical Pharmacology	**2 DOSAGE AND ADMINISTRATION**<br>**2.2 Individualization of Dose**<br>Dosing Recommendations Above 50 mg per day Patients who require doses of XENAZINE greater than 50 mg per day should be first tested and genotyped to determine if they are poor metabolizers (PMs) or extensive metabolizers (EMs) by their ability to express the drug metabolizing enzyme, CYP2D6. The dose of XENAZINE should then be individualized accordingly to their status as PMs or EMs [see Warnings and Precautions (5.3), Use in Specific Populations (8.7), Clinical Pharmacology (12.3)].<br>*Extensive and Intermediate CYP2D6 Metabolizers*

* Therapeutic areas do not necessarily reflect the CDER review division.
† Representative biomarkers are listed based on standard nomenclature as per the Human Genome Organization (HUGO) symbol and/or simplified descriptors using other common conventions. Listed biomarkers do not necessarily reflect the terminology used in labeling. The term “Nonspecific” is provided when labeling does not explicitly identify the specific biomarker(s) or when the biomarker is represented by a molecular phenotype or gene signature, and in some cases the biomarker was inferred based on the labeling language.
‡ Referenced figures and tables may be found in the labeling available at Drugs@FDA.
Blue text represents the most recent addtions and/or changes since last posted version.

NDA/ANDA/BLA Number, Label Version Date	Drug	Therapeutic Area*	Biomarker†	Labeling Sections	Labeling Text‡
					Genotyped patients who are identified as extensive (EMs) or intermediate metabolizers (IMs) of CYP2D6, who need doses of XENAZINE above 50 mg per day, should be titrated up slowly at weekly intervals by 12.5 mg daily, to allow the identification of a tolerated dose that reduces chorea. Doses above 50 mg per day should be given in a three times a day regimen. The maximum recommended daily dose is 100 mg and the maximum recommended single dose is 37.5 mg. If adverse reactions such as akathisia, parkinsonism, depression, insomnia, anxiety or sedation occur, titration should be stopped and the dose should be reduced. If the adverse reaction does not resolve, consideration should be given to withdrawing XENAZINE treatment or initiating other specific treatment (e.g., antidepressants) [see Warnings and Precautions (5.3), Use in Specific Populations (8.7), Clinical Pharmacology (12.3)].<br>*Poor CYP2D6 Metabolizers*<br>In PMs, the initial dose and titration is similar to EMs except that the recommended maximum single dose is 25 mg, and the recommended daily dose should not exceed a maximum of 50 mg [see Use in Specific Populations (8.7), Clinical Pharmacology (12.3)].<br><br>**5 WARNINGS AND PRECAUTIONS**<br>**5.3 Laboratory Tests**<br>Before prescribing a daily dose of XENAZINE that is greater than 50 mg per day, patients should be genotyped to determine if they express the drug metabolizing enzyme, CYP2D6. CYP2D6 testing is necessary to determine whether patients are poor metabolizers (PMs), extensive (EMs) or intermediate metabolizers (IMs) of XENAZINE.<br>Patients who are PMs of XENAZINE will have substantially higher levels of the primary drug metabolites (about 3-fold for α-HTBZ and 9-fold for β-HTBZ) than patients who are EMs. The dosage should be adjusted according to a patient's CYP2D6 metabolizer status. In patients who are identified as CYP2D6 PMs, the maximum recommended total daily dose is 50 mg and the maximum recommended single dose is 25 mg [see Dosage and Administration (2.2), Use in Specific Populations (8.7), Clinical Pharmacology (12.3)].<br><br>**8 USE IN SPECIFIC POPULATIONS**<br>**8.7 Poor or Extensive CYP2D6 Metabolizers**<br>Patients who require doses of XENAZINE greater than 50 mg per day, should be first tested and genotyped to determine if they are poor (PMs) or extensive metabolizers (EMs) by their ability to express the drug metabolizing enzyme, CYP2D6. The dose of XENAZINE should then be individualized accordingly to their status as either poor (PMs) or extensive metabolizers (EMs) [see Dosage and Administration (2.2), Warnings and Precautions (5.3), Clinical Pharmacology (12.3)].<br>*Poor Metabolizers*<br>Poor CYP2D6 metabolizers (PM) will have substantially higher levels of exposure to the primary metabolites (about 3-fold for α-HTBZ and 9-fold for β-HTBZ) compared to EMs. The dosage should, therefore, be adjusted according to a patient's CYP2D6 metabolizer status by limiting a single dose to a maximum of 25 mg and the recommended daily dose to not exceed a maximum of 50 mg/day in patients who are CYP2D6 PMs [see Dosage and Administration (2.2), Warnings and Precautions (5.3), Clinical Pharmacology (12.3)].<br>*Extensive / Intermediate Metabolizers*<br>In extensive (EMs) or intermediate metabolizers (IMs), the dosage of XENAZINE can be titrated to a maximum single dose of 37.5 mg and a recommended maximum daily dose of 100 mg [see Dosage and Administration (2.2), Drug Interactions (7.1), Clinical Pharmacology (12.3)].<br><br>**12 CLINICAL PHARMACOLOGY**<br>**12.3 Pharmacokinetics**<br>*Specific Populations*<br>*Poor CYP2D6 Metabolizers*<br>Although the pharmacokinetics of XENAZINE and its metabolites in patients who do not express the drug metabolizing enzyme, CYP2D6, poor metabolizers, (PMs), have not been systematically evaluated, it is likely that the exposure to α-HTBZ and β-HTBZ would be increased similar to that observed in patients taking strong CYP2D6 inhibitors (3- and 9-fold, respectively) [see Dosage and Administration (2.3), Warnings and Precautions (5.3), Use in Specific Populations (8.7)].
**012429, 05/23/2018**	Thioguanine (1)	Oncology	TPMT	Dosage and Administration, Warnings, Precautions, Clinical Pharmacology	**DOSAGE AND ADMINISTRATION**<br>(…) Patients with homozygous deficiency of either TPMT or NUDT15 enzyme typically require 10% or less of the standard thioguanine dosage. Reduce initial dosage in patients who are known to have homozygous TPMT or NUDT15 deficiency. Most patients with heterozygous TPMT or NUDT15 deficiency tolerate recommended thioguanine doses, but some require dose reduction based on toxicities. Patients who are heterozygous for both TPMT and NUDT15 may require more substantial dosage reductions. Reduce the dosage based on tolerability.<br>**WARNINGS**<br>**(…)** Evaluate patients with repeated severe myelosuppression for thiopurine S-methyltransferase (TPMT) or nucleotide diphosphatase (NUDT15) deficiency. TPMT genotyping or phenotyping (red blood cell TPMT activity) and NUDT15 genotyping can identify patients who have reduced activity of these enzymes. Patients with homozygous TPMT or NUDT15 deficiency require substantial dosage reductions. Bone marrow suppression could be exacerbated by coadministration with drugs that inhibit TPMT, such as olsalazine, mesalazine, or sulphasalazine.<br><br>**PRECAUTIONS**<br>*Laboratory Tests*<br>Consider testing for TPMT and NUDT15 deficiency in patients who experience severe bone marrow toxicities or repeated episodes of myelosuppression. (see WARNINGS).<br><br>**CLINICAL PHARMACOLOGY**

* Therapeutic areas do not necessarily reflect the CDER review division.
† Representative biomarkers are listed based on standard nomenclature as per the Human Genome Organization (HUGO) symbol and/or simplified descriptors using other common conventions. Listed biomarkers do not necessarily reflect the terminology used in labeling. The term "Nonspecific" is provided when labeling does not explicitly identify the specific biomarker(s) or when the biomarker is represented by a molecular phenotype or gene signature, and in some cases the biomarker was inferred based on the labeling language.
‡ Referenced figures and tables may be found in the labeling available at Drugs@FDA.
Blue text represents the most recent addtions and/or changes since last posted version.

NDA/ANDA/BLA Number, Label Version Date	Drug	Therapeutic Area*	Biomarker†	Labeling Sections	Labeling Text‡
					Metabolism and Genetic Polymorphism<br>Several published studies indicate that patients with reduced TPMT or NUDT15 activity receiving usual doses of mercaptopurine, accumulate excessive cellular concentrations of active 6-TGNs, and are at higher risk for severe myelosuppression. In a study of 1028 children with ALL, the approximate tolerated mercaptopurine dosage range for patients with TPMT and/or NUDT15 deficiency on mercaptopurine maintenance therapy (as a percentage of the planned dosage) was as follows: heterozygous for either TPMT or NUDT15, 50-90%; heterozygous for both TPMT and NUDT15, 30-50%; homozygous for either TPMT or NUDT15, 5-10%.<br>Approximately 0.3% (1:300) of patients of European or African ancestry have two loss-offunction alleles of the TPMT gene and have little or no TMPT activity (homozygous deficient or poor metabolizers), and approximately 10% of patients have one loss-of-function TPMT allele leading to intermediate TPMT activity (heterozygous deficient or intermediate metabolizers). The TPMT*2, TPMT*3A, and TPMT*3C alleles account for about 95% of individuals with reduced levels of TPMT activity. NUDT15 deficiency is detected in <1% of patients of European or African ancestry. Among patients of East Asian ancestry (i.e., Chinese, Japanese, Vietnamese), 2% have two loss-of-function alleles of the NUDT15 gene, and approximately 21% have one loss-of-function allele. The p.R139C variant of NUDT15 (present on the *2 and *3 alleles) is the most commonly observed, but other less common lossof-function NUDT15 alleles have been observed.<br>Consider all clinical information when interpreting results from phenotypic testing used to determine the level of thiopurine nucleotides or TPMT activity in erythrocytes, since some coadministered drugs can influence measurement of TPMT activity in blood, and blood from recent transfusions will misrepresent a patient's actual TPMT activity.
**012429, 05/23/2018**	Thioguanine (2)	Oncology	NUDT15	Dosage and Administration, Warnings, Precautions, Clinical Pharmacology	**DOSAGE AND ADMINISTRATION**<br>(…) Patients with homozygous deficiency of either TPMT or NUDT15 enzyme typically require 10% or less of the standard thioguanine dosage. Reduce initial dosage in patients who are known to have homozygous TPMT or NUDT15 deficiency. Most patients with heterozygous TPMT or NUDT15 deficiency tolerate recommended thioguanine doses, but some require dose reduction based on toxicities. Patients who are heterozygous for both TPMT and NUDT15 may require more substantial dosage reductions. Reduce the dosage based on tolerability.<br><br>**WARNINGS**<br>**(…)** Evaluate patients with repeated severe myelosuppression for thiopurine S-methyltransferase (TPMT) or nucleotide diphosphatase (NUDT15) deficiency. TPMT genotyping or phenotyping (red blood cell TPMT activity) and NUDT15 genotyping can identify patients who have reduced activity of these enzymes. Patients with homozygous TPMT or NUDT15 deficiency require substantial dosage reductions. Bone marrow suppression could be exacerbated by coadministration with drugs that inhibit TPMT, such as olsalazine, mesalazine, or sulphasalazine.<br><br>**PRECAUTIONS**<br>*Laboratory Tests*<br>Consider testing for TPMT and NUDT15 deficiency in patients who experience severe bone marrow toxicities or repeated episodes of myelosuppression. (see WARNINGS).<br><br>**CLINICAL PHARMACOLOGY**<br>*Metabolism and Genetic Polymorphism*<br>Several published studies indicate that patients with reduced TPMT or NUDT15 activity receiving usual doses of mercaptopurine, accumulate excessive cellular concentrations of active 6-TGNs, and are at higher risk for severe myelosuppression. In a study of 1028 children with ALL, the approximate tolerated mercaptopurine dosage range for patients with TPMT and/or NUDT15 deficiency on mercaptopurine maintenance therapy (as a percentage of the planned dosage) was as follows: heterozygous for either TPMT or NUDT15, 50-90%; heterozygous for both TPMT and NUDT15, 30-50%; homozygous for either TPMT or NUDT15, 5-10%.<br>Approximately 0.3% (1:300) of patients of European or African ancestry have two loss-offunction alleles of the TPMT gene and have little or no TMPT activity (homozygous deficient or poor metabolizers), and approximately 10% of patients have one loss-of-function TPMT allele leading to intermediate TPMT activity (heterozygous deficient or intermediate metabolizers). The TPMT*2, TPMT*3A, and TPMT*3C alleles account for about 95% of individuals with reduced levels of TPMT activity. NUDT15 deficiency is detected in <1% of patients of European or African ancestry. Among patients of East Asian ancestry (i.e., Chinese, Japanese, Vietnamese), 2% have two loss-of-function alleles of the NUDT15 gene, and approximately 21% have one loss-of-function allele. The p.R139C variant of NUDT15 (present on the *2 and *3 alleles) is the most commonly observed, but other less common lossof-function NUDT15 alleles have been observed.<br>Consider all clinical information when interpreting results from phenotypic testing used to determine the level of thiopurine nucleotides or TPMT activity in erythrocytes, since some coadministered drugs can influence measurement of TPMT activity in blood, and blood from recent transfusions will misrepresent a patient's actual TPMT activity.
**011808**	Thioridazine	Psychiatry	CYP2D6	Contraindications, Warnings, Precautions	Labeling not electronically available on Drugs@FDA
**022433, 04/03/2019**	Ticagrelor	Cardiology	CYP2C19	Clinical Pharmacology	**12 CLINICAL PHARMACOLOGY**<br>**12.5 Pharmacogenetics**<br>In a genetic substudy cohort of PLATO, the rate of thrombotic CV events in the BRILINTA arm did not depend on CYP2C19 loss of function status.
**207981, 08/02/2023**	Tipiracil and Trifluridine (1)	Oncology	ERBB2 (HER2)	Indications and Usage, Adverse	**1 INDICATIONS AND USAGE**<br>**1.2 Metastatic Gastric Cancer**

* Therapeutic areas do not necessarily reflect the CDER review division.
† Representative biomarkers are listed based on standard nomenclature as per the Human Genome Organization (HUGO) symbol and/or simplified descriptors using other common conventions. Listed biomarkers do not necessarily reflect the terminology used in labeling. The term "Nonspecific" is provided when labeling does not explicitly identify the specific biomarker(s) or when the biomarker is represented by a molecular phenotype or gene signature, and in some cases the biomarker was inferred based on the labeling language.
‡ Referenced figures and tables may be found in the labeling available at Drugs@FDA.
Blue text represents the most recent addtions and/or changes since last posted version.

Table of Pharmacogenomic Biomarkers in Drug Labeling
Last Updated: 06/2024

NDA/ANDA/BLA Number, Label Version Date	Drug	Therapeutic Area*	Biomarker†	Labeling Sections	Labeling Text‡
				Reactions, Clinical Studies	LONSURF is indicated for the treatment of adult patients with metastatic gastric or gastroesophageal junction adenocarcinoma previously treated with at least two prior lines of chemotherapy that included a fluoropyrimidine, a platinum, either a taxane or irinotecan, and if appropriate, HER2/neu-targeted therapy.<br><br>**6 ADVERSE REACTIONS**<br>**6.1 Clinical Trials Experience**<br>Metastatic Gastric Cancer<br>(…) The safety of LONSURF was evaluated in TAGS, an international, randomized (2:1), doubleblind, placebo-controlled trial in patients with metastatic gastric or gastroesophageal junction (GEJ) adenocarcinoma who were previously treated with at least 2 prior chemotherapy regimens for advanced disease [see Clinical Studies (14.2)]. Previous treatments must have included a fluoropyrimidine, a platinum, and either a taxane or irinotecan. Patients with HER2/neu-positive tumors must have received prior HER2/neu-targeted therapy, if available. (…)<br><br>**14 CLINICAL STUDIES**<br>**14.2 Metastatic Gastric Cancer**<br>(…) Patients with HER2/neu-positive tumors must have received prior HER2/neu-targeted therapy, if available. Adjuvant chemotherapy could be counted as one prior regimen in patients who had recurrence during or within 6 months of completion of the adjuvant chemotherapy. (…)<br>(…) The HER2 status was negative in 62%, positive in 19%, and unknown in 20% of patients. Among the 94 patients with HER2 positive tumors, 89% received prior anti-HER2 therapy. Efficacy results are summarized in Table 11 and Figure 3.<br>(…)
207981, 08/02/2023	Tipiracil and Trifluridine (2)	Oncology	RAS	Indications and Usage, Clinical Studies	**1 INDICATIONS AND USAGE**<br>**1.1 Metastatic Colorectal Cancer**<br>LONSURF, as a single agent or in combination with bevacizumab, is indicated for the treatment of adult patients with metastatic colorectal cancer previously treated with fluoropyrimidine-, oxaliplatin- and irinotecan-based chemotherapy, an anti-VEGF biological therapy, and if RAS wild-type, an anti-EGFR therapy.<br><br>**14 CLINICAL STUDIES**<br>**14.1 Metastatic Colorectal Cancer**<br>Previously treated metastatic colorectal cancer (single agent LONSURF) RECOURSE The efficacy of LONSURF was evaluated in RECOURSE (NCT01607957), an international, randomized, double-blind, placebo-controlled study conducted in patients with previously treated metastatic colorectal cancer (mCRC). Key eligibility criteria included prior treatment with at least 2 lines of standard chemotherapy for metastatic CRC, ECOG performance status (PS) 0-1, absence of brain metastasis, and absence of ascites requiring drainage in the past four weeks. Patients were randomized 2:1 to receive LONSURF 35 mg/m2 or matching placebo orally twice daily after meals on Days 1-5 and 8-12 of each 28-day cycle until disease progression or unacceptable toxicity. Randomization was stratified by KRAS status (wild-type vs. mutant), time since diagnosis of first metastasis (<18 months vs. ≥ 18 months), and region (Japan vs. US, Europe and Australia). The major efficacy outcome measure was overall survival (OS) and an additional efficacy outcome measure was progression-free survival (PFS). A total of 800 patients were randomized to LONSURF (N=534) with best supportive care (BSC) or matching placebo (N=266) plus BSC. The median age was 63 years, 61% were male, 58% and 35% were White and Asian respectively, and all patients had baseline ECOG PS of 0 or 1. The primary site of disease was colon (62%) or rectum (38%). KRAS status was wild-type (49%) or mutant (51%) at study entry. All patients received prior treatment with fluoropyrimidine-, oxaliplatin-, and irinotecan-based chemotherapy. All but one patient received bevacizumab and all but two patients with KRAS wild-type tumors received panitumumab or cetuximab.<br>Efficacy results are summarized in Table 9 and Figure 1.<br>Previously treated metastatic colorectal cancer (LONSURF in combination with bevacizumab)<br>SUNLIGHT<br>The efficacy of LONSURF in combination with bevacizumab was evaluated in SUNLIGHT (NCT 04737187), an international, randomized (1:1), open label study in patients with previously treated metastatic colorectal cancer. Patients were required to have received no more than 2 prior treatments for advanced disease, including a fluoropyrimidine, irinotecan, oxaliplatin, an anti-VEGF monoclonal antibody (optional) and an anti-EGFR monoclonal antibody for patients with RAS wild-type. Other key eligibility criteria included ECOG performance status (PS) 0-1, absence of symptomatic brain metastases, absence of ascites requiring drainage in the past 4 weeks, absence of uncontrolled hypertension, absence of non-healing wound, and absence of deep venous thromboembolic event in the past 4 weeks. Patients were randomized to receive LONSURF 35 mg/m2 administered orally twice daily on Days 1 to 5 and 8 to 12 of each 28-day cycle with or without bevacizumab 5 mg/kg administered intravenously every 2 weeks (on Day 1 and Day 15) of each 4-week cycle until disease progression or unacceptable toxicity. Randomization was stratified by geographic region (North America, European Union, Rest of the World), time since diagnosis of metastatic disease (<18 months, ≥18 months) and RAS status (wild-type, mutant). The major efficacy outcome was overall survival (OS), and an additional efficacy outcome measure was progression-free survival (PFS).<br>A total of 492 patients were randomized to receive LONSURF in combination with bevacizumab (N=246) or LONSURF as a single agent (N=246). The trial population characteristics were as follows: median age 63 years, 52% male, 88% White, 1.4% Black, 0.2% Asian, 0.2% American Indian or Alaska Native, and 9.6% were unknown, 46% had ECOG PS 0 and 54% had ECOG PS 1. The primary site of disease was colon (73%) or rectum (27%). Seventy-one percent of patients had a RAS mutant status. A total of 92% of patients received 2 prior anticancer treatment regimens for advanced CRC; all patients received prior fluoropyrimidine; 99.8% of patients received prior irinotecan; 98% of patients received prior oxaliplatin. Among all 492 treated patients, 76% received prior anti-VEGF treatment, and 72% received an anti-VEGF monoclonal antibody. Among the 142 patients with RAS wild-type mCRC, 94% received prior anti-EGFR monoclonal antibody.<br>Efficacy results are summarized in Table 10 and Figure 2.

* Therapeutic areas do not necessarily reflect the CDER review division.
† Representative biomarkers are listed based on standard nomenclature as per the Human Genome Organization (HUGO) symbol and/or simplified descriptors using other common conventions. Listed biomarkers do not necessarily reflect the terminology used in labeling. The term “Nonspecific” is provided when labeling does not explicitly identify the specific biomarker(s) or when the biomarker is represented by a molecular phenotype or gene signature, and in some cases the biomarker was inferred based on the labeling language.
‡ Referenced figures and tables may be found in the labeling available at Drugs@FDA.
Blue text represents the most recent addtions and/or changes since last posted version.

NDA/ANDA/BLA Number, Label Version Date	Drug	Therapeutic Area*	Biomarker†	Labeling Sections	Labeling Text‡
761232, 03/13/2024	Tislelizumab-jsgr	Oncology	CD274 (PD-L1)	Clinical Studies	**14 CLINICAL STUDIES**<br>**14.1 Esophageal Squamous Cell Cancer**<br>RATIONALE-302 (NCT03430843) was a multicenter, randomized (1:1), open-label trial in 512 adult patients with unresectable advanced or metastatic ESCC who progressed on or after prior systemic chemotherapy.<br>Patients were enrolled regardless of their tumor PD-L1 expression level. PD-L1 expression was evaluated at a central laboratory using the Ventana PD-L1 (SP263) assay that identified PD-L1 staining on both tumor and tumorassociated immune cells. The trial excluded patients who received prior immune checkpoint inhibitor, had brain or leptomeningeal metastases that were symptomatic or required treatment, active autoimmune disease, a medical condition requiring systemic corticosteroids or immunosuppressants, or apparent tumor invasion of organs adjacent to the esophageal tumor.<br>A total of 512 patients were enrolled and randomized to TEVIMBRA (n=256) or ICC (n=256) [irinotecan (46%), paclitaxel (33%), or docetaxel (21%)]. Of the 512 patients, 142 (28%) had PD-L1 ≥ 10%, 222 (43%) had PD-L1 < 10%, and 148 (29%) had unknown baseline PD-L1 status. (…)
201820, 02/10/2023	Tobramycin	Infectious Diseases	MT-RNR1	Warnings and Precautions	**5 WARNINGS AND PRECAUTIONS**<br>**5.1 Ototoxicity**<br>Ototoxicity with use of BETHKIS<br>Caution should be exercised when prescribing BETHKIS to patients with known or suspected auditory or vestibular dysfunction.<br>Findings related to ototoxicity as measured by audiometric evaluations and auditory adverse event reports were similar between BETHKIS and placebo in controlled clinical trials. Hearing loss was reported in two (1.1%) BETHKIS-treated patients and in one (0.9%) placebo-treated patient during clinical studies. Additionally, dizziness and vertigo, both of which may be manifestations of vestibular forms of ototoxicity, were observed in similar numbers of BETHKIS- and placebo-treated patients. Dizziness occurred in two (1.1%) BETHKIS-treated patients and one (0.9%) placebo-treated patient and vertigo occurred in two (1.1%) BETHKIS-treated patients versus no placebo patients in clinical studies. None of the BETHKIS patients discontinued their therapy due to hearing loss, dizziness or vertigo.<br>Tinnitus may be a sentinel symptom of ototoxicity. No reports of tinnitus occurred in patients during clinical studies with BETHKIS, but because it has been observed with inhaled tobramycin solutions [see Adverse Reactions (6.2)], onset of this symptom warrants caution. Ototoxicity, manifested as both auditory and vestibular toxicity, has been reported with parenteral aminoglycosides. Vestibular toxicity may be manifested by vertigo, ataxia or dizziness. Patients with known or suspected auditory or vestibular dysfunction should be closely monitored when taking BETHKIS. Monitoring may include obtaining audiometric evaluations and serum tobramycin levels. If ototoxicity is noted, the patient should be managed as medically appropriate, including potentially discontinuing BETHKIS.<br>Risk of Ototoxicity Due to Mitochondrial DNA Variants<br>Cases of ototoxicity with aminoglycosides have been observed in patients with certain variants in the mitochondrially encoded 12S rRNA gene (MT-RNR1), particularly the m.1555A>G variant. Ototoxicity occurred in some patients even when their aminoglycoside serum levels were within the recommended range. Mitochondrial DNA variants are present in less than 1% of the general US population, and the proportion of the variant carriers who may develop ototoxicity as well as the severity of ototoxicity is unknown. In case of known maternal history of ototoxicity due to aminoglycoside use or a known mitochondrial DNA variant in the patient, consider alternative treatments other than aminoglycosides unless the increased risk of permanent hearing loss is outweighed by the severity of infection and lack of safe and effective alternative therapies.
215887, 04/25/2023	Tofersen	Oncology	SOD1	Indications and Usage, Use in Specific Populations, Clinical Studies	**1 INDICATIONS AND USAGE**<br>QALSODY is indicated for the treatment of amyotrophic lateral sclerosis (ALS) in adults who have a mutation in the superoxide dismutase 1 (SOD1) gene. This indication is approved under accelerated approval based on reduction in plasma neurofilament light chain (NfL) observed in patients treated with QALSODY [see Clinical Studies (14)]. Continued approval for this indication may be contingent upon verification of clinical benefit in confirmatory trial(s).<br><br>**8 USE IN SPECIFIC POPULATIONS**<br>**8.5 Geriatric Use**<br>A total of 13.5% (22/162) patients were 65 years of age and older and 1.2% (2/162) patients were 75 years of age and older at initiation of treatment in clinical studies for ALS in patients who have a mutation in the superoxide dismutase 1 (SOD1) gene [see Clinical Studies (14)]. No overall differences in safety or effectiveness were observed between these patients and younger patients, but a greater sensitivity of some older individuals cannot be ruled out. There is no evidence for special dosage considerations based on age when QALSODY is administered.<br><br>**14 CLINICAL STUDIES**<br>The efficacy of QALSODY was assessed in a 28-week randomized, double-blind, placebocontrolled clinical study in patients 23 to 78 years of age with weakness attributable to ALS and a SOD1 mutation confirmed by a central laboratory (Study 1 Part C, NCT02623699). One hundred eight (108) patients were randomized 2:1 to receive treatment with either QALSODY 100 mg (n = 72) or placebo (n = 36) for 24 weeks (3 loading doses followed by 5 maintenance doses). Concomitant riluzole and/or edaravone use was permitted for patients.<br>The prespecified primary analysis population (n = 60, modified intent to treat [mITT]) had a slow vital capacity (SVC) ≥ 65% of predicted value and met prognostic enrichment criteria for rapid disease progression, defined based on their pre-randomization ALS Functional Rating Scale–Revised (ALSFRS-R) decline slope and SOD1 mutation type.
018894	Tolazamide	Endocrinology	G6PD	Precautions	Labeling not electronically available on Drugs@FDA
010670	Tolbutamide	Endocrinology	G6PD	Precautions	Labeling not electronically available on Drugs@FDA
021228, 07/13/2018	Tolterodine	Urology	CYP2D6	Warnings and Precautions, Drug Interactions,	**5 WARNINGS AND PRECAUTIONS**<br>**5.9 Use in Patients with Congenital or Acquired QT Prolongation**

NDA/ANDA/BLA Number, Label Version Date	Drug	Therapeutic Area*	Biomarker†	Labeling Sections	Labeling Text‡
				Clinical Pharmacology	In a study of the effect of tolterodine immediate release tablets on the QT interval [see CLINICAL PHARMACOLOGY (12.2)], the effect on the QT interval appeared greater for 8 mg/day (two times the therapeutic dose) compared to 4 mg/day and was more pronounced in CYP2D6 poor metabolizers (PM) than extensive metabolizers (EMs). The effect of tolterodine 8 mg/day was not as large as that observed after four days of therapeutic dosing with the active control moxifloxacin. However, the confidence intervals overlapped. (…)<br><br>**7 DRUG INTERACTIONS**<br>**7.1 Potent CYP2D6 Inhibitors**<br>Fluoxetine, a potent inhibitor of CYP2D6 activity, significantly inhibited the metabolism of tolterodine immediate release in CYP2D6 extensive metabolizers, resulting in a 4.8-fold increase in tolterodine AUC. There was a 52% decrease in Cmax and a 20% decrease in AUC of 5-hydroxymethyl tolterodine (5-HMT), the pharmacologically active metabolite of tolterodine [see CLINICAL PHARMACOLOGY (12.1)]. The sums of unbound serum concentrations of tolterodine and 5-HMT are only 25% higher during the interaction. No dose adjustment is required when tolterodine and fluoxetine are co-administered [see CLINICAL PHARMACOLOGY (12.3)].<br>**7.2 Potent CYP3A4 Inhibitors**<br>Ketoconazole (200 mg daily), a potent CYP3A4 inhibitor, increased the mean Cmax and AUC of tolterodine by 2- and 2.5-fold, respectively, in CYP2D6 poor metabolizers.<br>For patients receiving ketoconazole or other potent CYP3A4 inhibitors such as itraconazole, clarithromycin, or ritonavir, the recommended dose of DETROL LA is 2 mg once daily [see DOSAGE AND ADMINISTRATION(2.2) and CLINICAL PHARMACOLOGY (12.3)].<br><br>**12 CLINICAL PHARMACOLOGY**<br>**12.2 Pharmacodynamics**<br>*Cardiac Electrophysiology*<br>The effect of 2 mg BID and 4 mg BID of DETROL immediate release (tolterodine IR) tablets on the QT interval was evaluated in a 4-way crossover, double-blind, placebo- and active-controlled (moxifloxacin 400 mg QD) study in healthy male (N=25) and female (N=23) volunteers aged 18–55 years. Study subjects [approximately equal representation of CYP2D6 extensive metabolizers (EMs) and poor metabolizers (PMs)] completed sequential 4-day periods of dosing with moxifloxacin 400 mg QD, tolterodine 2 mg BID, tolterodine 4 mg BID, and placebo. The 4 mg BID dose of tolterodine IR (two times the highest recommended dose) was chosen because this dose results in tolterodine exposure similar to that observed upon coadministration of tolterodine 2 mg BID with potent CYP3A4 inhibitors in patients who are CYP2D6 poor metabolizers [see DRUG INTERACTIONS (7.2)]. QT interval was measured over a 12-hour period following dosing, including the time of peak plasma concentration (Tmax) of tolterodine and at steady state (Day 4 of dosing). (…)<br>(…) Tolterodine's effect on QT interval was found to correlate with plasma concentration of tolterodine. There appeared to be a greater QTc interval increase in CYP2D6 poor metabolizers than in CYP2D6 extensive metabolizers after tolterodine treatment in this study. (…)<br>**12.3 Pharmacokinetics**<br>*Variability in Metabolism:* A subset of individuals (approximately 7% of Caucasians and approximately 2% of African Americans) are poor metabolizers for CYP2D6, the enzyme responsible for the formation of 5-HMT from tolterodine. The identified pathway of metabolism for these individuals ("poor metabolizers") is dealkylation via cytochrome P450 3A4 (CYP3A4) to N-dealkylated tolterodine. The remainder of the population is referred to as "extensive metabolizers." Pharmacokinetic studies revealed that tolterodine is metabolized at a slower rate in poor metabolizers than in extensive metabolizers; this results in significantly higher serum concentrations of tolterodine and in negligible concentrations of 5-HMT.<br>*Excretion:* Following administration of a 5 mg oral dose of 14C-tolterodine solution to healthy volunteers, 77% of radioactivity was recovered in urine and 17% was recovered in feces in 7 days. Less than 1% (< 2.5% in poor metabolizers) of the dose was recovered as intact tolterodine, and 5% to 14% (<1% in poor metabolizers) was recovered as 5-HMT.<br>A summary of mean (± standard deviation) pharmacokinetic parameters of tolterodine extended release and 5 HMT in extensive (EM) and poor (PM) metabolizers is provided in Table 3. These data were obtained following single and multiple doses of tolterodine extended release administered daily to 17 healthy male volunteers (13 EM, 4 PM). (See Table 3) (…)<br>*Drug Interactions:*<br>Potent CYP2D6 inhibitors: Fluoxetine is a selective serotonin reuptake inhibitor and a potent inhibitor of CYP2D6 activity. In a study to assess the effect of fluoxetine on the pharmacokinetics of tolterodine immediate release and its metabolites, it was observed that fluoxetine significantly inhibited the metabolism of tolterodine immediate release in extensive metabolizers, resulting in a 4.8-fold increase in tolterodine AUC. There was a 52% decrease in Cmax and a 20% decrease in AUC of 5-hydroxymethyl tolterodine (5-HMT, the pharmacologically active metabolite of tolterodine). Fluoxetine thus alters the pharmacokinetics in patients who would otherwise be CYP2D6 extensive metabolizers of tolterodine immediate release to resemble the pharmacokinetic profile in poor metabolizers. The sums of unbound serum concentrations of tolterodine immediate release and 5-HMT are only 25% higher during the interaction. No dose adjustment is required when tolterodine and fluoxetine are co-administered.<br>Potent CYP3A4 inhibitors: The effect of a 200 mg daily dose of ketoconazole on the pharmacokinetics of tolterodine immediate release was studied in 8 healthy volunteers, all of whom were CYP2D6 poor metabolizers. In the presence of ketoconazole, the mean Cmax and AUC of tolterodine increased by 2- and 2.5 fold, respectively. Based on these findings, other potent CYP3A4 inhibitors may also lead to increases of tolterodine plasma concentrations. (…)
**020497, 05/12/2017**	Toremifene	Oncology	ESR (Hormone Receptor)	Indications and Usage, Clinical Studies	**1 INDICATIONS AND USAGE**<br>FARESTON® is an estrogen agonist/antagonist indicated for the treatment of metastatic breast cancer in postmenopausal women with estrogen-receptor positive or unknown tumors.<br><br>**14 CLINICAL STUDIES**

* Therapeutic areas do not necessarily reflect the CDER review division.
† Representative biomarkers are listed based on standard nomenclature as per the Human Genome Organization (HUGO) symbol and/or simplified descriptors using other common conventions. Listed biomarkers do not necessarily reflect the terminology used in labeling. The term "Nonspecific" is provided when labeling does not explicitly identify the specific biomarker(s) or when the biomarker is represented by a molecular phenotype or gene signature, and in some cases the biomarker was inferred based on the labeling language.
‡ Referenced figures and tables may be found in the labeling available at Drugs@FDA.
Blue text represents the most recent addtions and/or changes since last posted version.

Table of Pharmacogenomic Biomarkers in Drug Labeling
Last Updated: 06/2024

NDA/ANDA/BLA Number, Label Version Date	Drug	Therapeutic Area*	Biomarker†	Labeling Sections	Labeling Text‡
					Three prospective, randomized, controlled clinical studies (North American, Eastern European, and Nordic) were conducted to evaluate the efficacy of FARESTON for the treatment of breast cancer in postmenopausal women. The patients were randomized to parallel groups receiving FARESTON 60 mg (FAR60) or tamoxifen 20 mg (TAM20) in the North American Study or tamoxifen 40 mg (TAM40) in the Eastern European and Nordic studies. The North American and Eastern European studies also included high-dose toremifene arms of 200 and 240 mg daily, respectively. The studies included postmenopausal patients with estrogen-receptor (ER) positive or estrogenreceptor (ER) unknown metastatic breast cancer. (…)
**217700, 06/26/2024**	**Tovorafenib**	Oncology	BRAF	Indications and Usage, Dosage and Administration, Warnings and Precautions, Adverse Reactions, Use in Specific Populations, Clinical Studies	**1 INDICATIONS AND USAGE**<br>OJEMDA is indicated for the treatment of patients 6 months of age and older with relapsed or refractory pediatric low-grade glioma (LGG) harboring a BRAF fusion or rearrangement, or BRAF V600 mutation.<br>This indication is approved under accelerated approval based on response rate and duration of response [see Clinical Studies (14)]. Continued approval for this indication may be contingent upon verification and description of clinical benefit in a confirmatory trial(s).<br><br>**2 DOSAGE AND ADMINISTRATION**<br>**2.1 Patient Selection**<br>Confirm the presence of BRAF fusion or rearrangement, or BRAF V600 mutation prior to initiation of treatment with OJEMDA [see Warnings and Precautions (5.6), Clinical Studies (14)]. An FDA approved test for the detection of BRAF fusion or rearrangement, or BRAF V600 mutation in relapsed or refractory pediatric LGG is not currently available.<br><br>**5 WARNINGS AND PRECAUTIONS**<br>**5.6 NF1 Associated Tumors**<br>Based on nonclinical data in NF1 models without BRAF alterations, tovorafenib may promote tumor growth in patients with NF1 tumors [see Nonclinical Toxicology (13.2)]. Confirm evidence of a BRAF alteration prior to initiation of treatment with OJEMDA.<br><br>**6 ADVERSE REACTIONS**<br>**6.1 Clinical Trials Experience**<br>Pediatric Low-grade Glioma<br>The safety of OJEMDA was evaluated in 137 patients with relapsed or refractory pediatric LGG harboring a BRAF alteration in FIREFLY-1 (Arms 1 and 2) [see Clinical Studies (14)]. Patients received OJEMDA at a dose based on body surface area [see Dosage and Administration (2.3] orally once weekly until disease progression or intolerable toxicity.<br><br>**8 USE IN SPECIFIC POPULATIONS**<br>**8.4 Pediatric Use**<br>The safety and effectiveness of OJEMDA in pediatric patients 6 months of age and older with relapsed or refractory pediatric LGG harboring a BRAF fusion or rearrangement, or BRAF V600 mutation have been established based on data from a multicenter, open-label, single-arm clinical trial [see Clinical Studies (14)]. (…)<br><br>**14 CLINICAL STUDIES**<br>The efficacy of OJEMDA was evaluated in a multicenter, open-label, single-arm clinical trial (FIREFLY-1; NCT04775485). Eligible patients (N=76) were required to have a relapsed or refractory pediatric low-grade glioma (LGG) harboring an activating BRAF alteration based on local laboratory testing. Patients were also required to have at least one measurable lesion as defined by RANO 2010 criteria. All patients had received at least one line of prior systemic therapy and had documented evidence of radiographic progression. Patients with tumors harboring additional activating molecular alteration(s) (e.g., IDH1/2 mutations, FGFR mutations, etc.) or patients with known or suspected diagnosis of neurofibromatosis type 1 (NF1) were excluded.<br>Patients received OJEMDA approximately 420 mg/m2 orally once weekly (range: 290 to 476 mg/m2 , 0.76-1.25 times the approved recommended dosage) according to body surface area with a maximum dose of 600 mg until disease progression or unacceptable toxicity. Although the OJEMDA dosages administered in FIREFLY-1 were between 290 mg/m2 to 476 mg/m2 , the recommended OJEMDA dosage is 380 mg/m2 orally once weekly because this dosage was determined to be safe and effective for the treatment of patients 6 months of age and older with relapsed or refractory pediatric LGG harboring a BRAF fusion or rearrangement, or BRAF V600 mutation [see Dosage and Administration (2.3)].<br>Tumor assessments were performed every 12 weeks. (…)<br>The efficacy population included 76 patients who had measurable disease at baseline and who received OJEMDA. The median age was 8.5 years (range 2 to 21 years); 53% were male; 53% White, 7% Asian, 2.6% Black or African American, 3.9% multiple races, 8% other race, 26% where race was not reported; 3.9% were Hispanic or Latino, and 93% had Karnofsky/Lansky performance status of 80 to 100. Patients received a median of 3 prior systemic regimens (range: 1 to 9). Forty-five patients (59%) received prior treatment with a MAP kinase pathway inhibitor. The most common tumor locations were the optic pathway (51%), deep midline structures (12%), brain stem (8%), cerebral hemisphere and cerebellum (7% each). Fifty-six patients (74%) had a KIAA1549:BRAF fusion, twelve patients (16%) had a V600E mutation, and eight patients (11%) had a BRAF alteration classified as "other" including BRAF duplication or BRAF rearrangement. Efficacy results are shown in Table 10.<br>Among responders, the median time to response was 5.3 months (range 1.6, 11.2). In exploratory analyses of BRAF alteration status, the ORR was 52% among patients with BRAF fusion or rearrangement (n=64), and 50% among patients with BRAF V600E mutation (n=12), respectively. In exploratory analyses of prior therapies, the ORR was 49% among patients who had received prior MAPK-targeted therapy (n=45), and 55% among patients who had not received prior MAPK-targeted therapy (n=31).<br>Based on RANO-LGG (2011) criteria (n=76), the ORR was 53% [95% CI: (41, 64)], including 20 patients each with PR and MR, respectively.

* Therapeutic areas do not necessarily reflect the CDER review division.
† Representative biomarkers are listed based on standard nomenclature as per the Human Genome Organization (HUGO) symbol and/or simplified descriptors using other common conventions. Listed biomarkers do not necessarily reflect the terminology used in labeling. The term "Nonspecific" is provided when labeling does not explicitly identify the specific biomarker(s) or when the biomarker is represented by a molecular phenotype or gene signature, and in some cases the biomarker was inferred based on the labeling language.
‡ Referenced figures and tables may be found in the labeling available at Drugs@FDA.
Blue text represents the most recent addtions and/or changes since last posted version.

Table of Pharmacogenomic Biomarkers in Drug Labeling
Last Updated: 06/2024

NDA/ANDA/BLA Number, Label Version Date	Drug	Therapeutic Area*	Biomarker†	Labeling Sections	Labeling Text‡
020281, 04/08/2019	Tramadol	Anesthesiology	CYP2D6	Boxed Warning, Warnings and Precautions, Use in Specific Populations, Clinical Pharmacology, Patient Counseling Information	**BOXED WARNING**<br>**ULTRA-RAPID METABOLISM OF TRAMADOL AND OTHER RISK FACTORS FOR LIFE-THREATENING RESPIRATORY DEPRESSION IN CHILDREN**<br>Life-threatening respiratory depression and death have occurred in children who received tramadol. Some of the reported cases followed tonsillectomy and/or adenoidectomy; in a t l e a s t one case, the child had evidence of being an ultra-rapid metabolizer of tramadol due to a CYP2D6 polymorphism (see WARNINGS). ULTRAM is contraindicated in children younger than 12 years of age and in children younger than 18 years of age following tonsillectomy and/or adenoidectomy (see CONTRAINDICATIONS). Avoid the use of ULTRAM in adolescents 12 to 18 years of age who have other risk factors that may increase their sensitivity to the respiratory depressant effects of tramadol (see WARNINGS).<br><br>**5 WARNINGS AND PRECAUTIONS**<br>**5.4 Ultra-Rapid Metabolism of Tramadol and Other Risk Factors for Lifethreatening Respiratory Depression in Children**<br>Life-threatening respiratory depression and death have occurred in children who received tramadol. Tramadol and codeine are subject to variability in metabolism based upon CYP2D6 genotype (described below), which can lead to increased exposure to an active metabolite. Based upon post-marketing reports with tramadol or with codeine, children younger than 12 years of age may be more susceptible to the respiratory depressant effects of tramadol. Furthermore, children with obstructive sleep apnea who are treated with opioids for post-tonsillectomy and/or adenoidectomy pain may be particularly sensitive to their respiratory depressant effect. Because of the risk of life-threatening respiratory depression and death:<br>• ULTRAM is contraindicated for all children younger than 12 years of age [see Contraindications (4)].<br>• ULTRAM is contraindicated for post-operative management in pediatric patients younger than 18 years of age following tonsillectomy and/or adenoidectomy [see Contraindications (4)].<br>• Avoid the use of ULTRAM in adolescents 12 to 18 years of age who have other risk factors that may increase their sensitivity to the respiratory depressant effects of tramadol unless the benefits outweigh the risks. Risk factors include conditions associated with hypoventilation such as postoperative status, obstructive sleep apnea, obesity, severe pulmonary disease, neuromuscular disease, and concomitant use of other medications that cause respiratory depression.<br>• As with adults, when prescribing opioids for adolescents, healthcare providers should choose the lowest effective dose for the shortest period of time and inform patients and caregivers about these risks and the signs of opioid overdose [see Use in Specific Populations (8.4), Overdosage (10)].<br>*Nursing Mothers*<br>Tramadol is subject to the same polymorphic metabolism as codeine, with ultra-rapid metabolizers of CYP2D6 substrates being potentially exposed to life-threatening levels of the active metabolite O-desmethyltramadol (M1). At least one death was reported in a nursing infant who was exposed to high levels of morphine in breast milk because the mother was an ultrarapid metabolizer of codeine. A baby nursing from an ultra-rapid metabolizer mother taking ULTRAM could potentially be exposed to high levels of M1, and experience life-threatening respiratory depression. For this reason, breastfeeding is not recommended during treatment with ULTRAM [see Use in Specific Populations (8.2)].<br>*CYP2D6 Genetic Variability: Ultra-rapid metabolizer*<br>Some individuals may be ultra-rapid metabolizers because of a specific CYP2D6 genotype (e.g., gene duplications denoted as *1/*1xN or *1/*2xN). The prevalence of this CYP2D6 phenotype varies widely and has been estimated at 1 to 10% for Whites (European, North American), 3 to 4% for Blacks (African Americans), 1 to 2% for East Asians (Chinese, Japanese, Korean), and may be greater than 10% in certain racial/ethnic groups (i.e., Oceanian, Northern African, Middle Eastern, Ashkenazi Jews, Puerto Rican). These individuals convert tramadol into its active metabolite, O-desmethyltramadol (M1), more rapidly and completely than other people. This rapid conversion results in higher than expected serum M1 levels. Even at labeled dosage regimens, individuals who are ultra- rapid metabolizers may have life-threatening or fatal respiratory depression or experience signs of overdose (such as extreme sleepiness, confusion, or shallow breathing) [see Overdosage (10)]. Therefore, individuals who are ultra-rapid metabolizers should not use ULTRAM.<br><br>**8 USE IN SPECIFIC POPULATIONS**<br>**8.2 Lactation**<br>**Risk Summary**<br>ULTRAM is not recommended for obstetrical preoperative medication or for post-delivery analgesia in nursing mothers because its safety in infants and newborns has not been studied.<br>Tramadol and its metabolite, O-desmethyltramadol (M1), are present in human milk. There is no information on the effects of the drug on the breastfed infant or the effects of the drug on milk production. The M1 metabolite is more potent than tramadol in mu opioid receptor binding [see Clinical Pharmacology (12)]. Published studies have reported tramadol and M1 in colostrum with administration of tramadol to nursing mothers in the early post-partum period. Women who are ultra-rapid metabolizers of tramadol may have higher than expected serum levels of M1, potentially leading to higher levels of M1 in breast milk that can be dangerous in their breastfed infants. In women with normal tramadol metabolism, the amount of tramadol secreted into human milk is low and dose-dependent. Because of the potential for serious adverse reactions, including excess sedation and respiratory depression in a breastfed infant, advise patients that breastfeeding is not recommended during treatment with ULTRAM [see Warnings and Precautions (5.4)]. (…)<br>**8.4 Pediatric Use**<br>The safety and effectiveness of ULTRAM in pediatric patients have not been established.<br>Life-threatening respiratory depression and death have occurred in children who received tramadol [see Warnings and Precautions (5.4)]. In some of the reported cases, these events followed tonsillectomy and/or adenoidectomy, and one of the children had evidence of being an ultra-rapid metabolizer of tramadol (i.e., multiple copies of the gene for cytochrome P450 isoenzyme 2D6). Children with sleep apnea may be particularly sensitive to the respiratory depressant effects of tramadol. Because of the risk of life-threatening respiratory depression and death:<br>• ULTRAM is contraindicated for all children younger than 12 years of age [see Contraindications (4)].<br>• ULTRAM is contraindicated for post-operative management in pediatric patients younger than 18 years of age following tonsillectomy and/or adenoidectomy [see Contraindications (4)].

* Therapeutic areas do not necessarily reflect the CDER review division.
† Representative biomarkers are listed based on standard nomenclature as per the Human Genome Organization (HUGO) symbol and/or simplified descriptors using other common conventions. Listed biomarkers do not necessarily reflect the terminology used in labeling. The term "Nonspecific" is provided when labeling does not explicitly identify the specific biomarker(s) or when the biomarker is represented by a molecular phenotype or gene signature, and in some cases the biomarker was inferred based on the labeling language.
‡ Referenced figures and tables may be found in the labeling available at Drugs@FDA.
Blue text represents the most recent addtions and/or changes since last posted version.

NDA/ANDA/BLA Number, Label Version Date	Drug	Therapeutic Area*	Biomarker†	Labeling Sections	Labeling Text‡
					Avoid the use of ULTRAM in adolescents 12 to 18 years of age who have other risk factors that may increase their sensitivity to the respiratory depressant effects of tramadol unless the benefits outweigh the risks. Risk factors include conditions associated with hypoventilation such as postoperative status, obstructive sleep apnea, obesity, severe pulmonary disease, neuromuscular disease, and concomitant use of other medications that cause respiratory depression.<br><br>**12 CLINICAL PHARMACOLOGY**<br>**12.3 Pharmacokinetics**<br>*Metabolism*<br>(…) Approximately 7% of the population has reduced activity of the CYP2D6 isoenzyme of cytochrome P-450. These individuals are “poor metabolizers” of debrisoquine, dextromethorphan, tricyclic antidepressants, among other drugs. Based on a population PK analysis of Phase I studies in healthy subjects, concentrations of tramadol were approximately 20% higher in “poor metabolizers” versus “extensive metabolizers”, while M1 concentrations were 40% lower.<br>(…)<br>*Poor / Extensive Metabolizers, CYP2D6*<br>The formation of the active metabolite, M1, is mediated by CYP2D6, a polymorphic enzyme. Approximately 7% of the population has reduced activity of the CYP2D6 isoenzyme of cytochrome P450 metabolizing enzyme system. These individuals are “poor metabolizers” of debrisoquine, dextromethorphan and tricyclic antidepressants, among other drugs. Based on a population PK analysis of Phase 1 studies with IR tablets in healthy subjects, concentrations of tramadol were approximately 20% higher in “poor metabolizers” versus “extensive metabolizers,” while M1 concentrations were 40% lower.<br><br>**17 PATIENT COUNSELING INFORMATION**<br>Ultra-Rapid Metabolism of Tramadol and Other Risk Factors for Life-threatening Respiratory Depression in Children<br>Advise caregivers that ULTRAM is contraindicated in children younger than 12 years of age and in children younger than 18 years of age following tonsillectomy and/or adenoidectomy. Advise caregivers of children ages 12 to 18 years of age receiving ULTRAM to monitor for signs of respiratory depression [see Warnings and Precautions (5.4)].
**204114, 06/22/2022**	Trametinib (1)	Oncology	BRAF	Indications and Usage, Dosage and Administration, Adverse Reactions, Use in Specific Populations, Clinical Pharmacology, Clinical Studies	**1 INDICATIONS AND USAGE**<br>**1.1 BRAF V600E or V600K Mutation-Positive Unresectable or Metastatic Melanoma**<br>MEKINIST® is indicated, as a single agent in BRAF-inhibitor treatment-naïve patients or in combination with dabrafenib, for the treatment of patients with unresectable or metastatic melanoma with BRAF V600E or V600K mutations, as detected by an FDA-approved test [see Dosage and Administration (2.1)]. **1.2 Adjuvant Treatment of BRAF V600E or V600K Mutation-Positive Melanoma**<br>MEKINIST is indicated, in combination with dabrafenib, for the adjuvant treatment of patients with melanoma with BRAF V600E or V600K mutations as detected by an FDA-approved test, and involvement of lymph node(s), following complete resection [see Dosage and Administration (2.1)].<br>**1.3 BRAF V600E Mutation-Positive Metastatic NSCLC**<br>MEKINIST is indicated, in combination with dabrafenib, for the treatment of patients with metastatic non-small cell lung cancer (NSCLC) with BRAF V600E mutation as detected by an FDA-approved test [see Dosage and Administration (2.1)].<br>**1.4 BRAF V600E Mutation-Positive Locally Advanced or Metastatic Anaplastic Thyroid Cancer**<br>MEKINIST is indicated, in combination with dabrafenib, for the treatment of patients with locally advanced or metastatic anaplastic thyroid cancer (ATC) with BRAF V600E mutation and with no satisfactory locoregional treatment options [see Dosage and Administration (2.1), (2.5)].<br>1.5 BRAF V600E Mutation-Positive Unresectable or Metastatic Solid Tumors<br>MEKINIST is indicated, in combination with dabrafenib, for the treatment of adult and pediatric patients 6 years of age and older with unresectable or metastatic solid tumors with BRAF V600E mutation who have progressed following prior treatment and have no satisfactory alternative treatment options [see Dosage and Administration (2.1)]. This indication is approved under accelerated approval based on overall response rate and duration of response [see Clinical Studies (14.6)]. Continued approval for this indication may be contingent upon verification and description of clinical benefit in a confirmatory trial(s).<br><br>**2 DOSAGE AND ADMINISTRATION**<br>**2.1 Patient Selection**<br>Melanoma<br>• Confirm the presence of BRAF V600E or V600K mutation in tumor specimens prior to initiation of treatment with MEKINIST as a single agent or in combination with dabrafenib [see Clinical Studies (14.1), (14.2)].<br>• Information on FDA-approved tests for the detection of BRAF V600 mutations in melanoma is available at: http://www.fda.gov/CompanionDiagnostics.<br>NSCLC<br>• Confirm the presence of BRAF V600E mutation in tumor specimens prior to initiation of treatment with MEKINIST and dabrafenib [see Clinical Studies (14.3)].<br>• Information on FDA-approved tests for the detection of BRAF V600E mutations in NSCLC is available at: http://www.fda.gov/CompanionDiagnostics.<br>ATC<br>• Confirm the presence of BRAF V600E mutation in tumor specimens prior to initiation of treatment with MEKINIST and dabrafenib [see Clinical Studies (14.4)].<br>An FDA-approved test for the detection of BRAF V600E mutation in ATC is not currently available.<br>Solid Tumors<br>• Confirm the presence of BRAF V600E mutation in tumor specimens prior to initiation of treatment with MEKINIST and dabrafenib [see Clinical Studies (14.6)].<br>An FDA-approved test for the detection of BRAF V600E mutation in solid tumors other than melanoma and NSCLC is not currently available.

* Therapeutic areas do not necessarily reflect the CDER review division.
† Representative biomarkers are listed based on standard nomenclature as per the Human Genome Organization (HUGO) symbol and/or simplified descriptors using other common conventions. Listed biomarkers do not necessarily reflect the terminology used in labeling. The term “Nonspecific” is provided when labeling does not explicitly identify the specific biomarker(s) or when the biomarker is represented by a molecular phenotype or gene signature, and in some cases the biomarker was inferred based on the labeling language.
‡ Referenced figures and tables may be found in the labeling available at Drugs@FDA.
Blue text represents the most recent addtions and/or changes since last posted version.

NDA/ANDA/BLA Number, Label Version Date	Drug	Therapeutic Area*	Biomarker†	Labeling Sections	Labeling Text‡
					**6 ADVERSE REACTIONS**<br>**6.1 Clinical Trials Experience**<br>*Unresectable or Metastatic BRAF V600E Mutation Positive Melanoma*<br>*MEKINIST Administered as a Single Agent*<br>Table 3 presents adverse reactions identified from analyses of the METRIC study, a randomized, open-label trial of patients with BRAF V600E or V600K mutation-positive melanoma receiving MEKINIST (N = 211) 2 mg orally once daily or chemotherapy (N = 99) (either dacarbazine 1,000 mg/m2 every 3 weeks or paclitaxel 175 mg/m2 every 3 weeks) [see Clinical Studies (14.1)]. (…)<br>*Adjuvant Treatment of BRAF V600E or V600K Mutation-Positive Melanoma*<br>The safety of MEKINIST when administered with dabrafenib was evaluated in 435 patients with Stage III melanoma with BRAF V600E or V600K mutations following complete resection who received at least one dose of study therapy in the COMBI-AD study [see Clinical Studies (14.2)]. (…)<br>*Metastatic, BRAF V600E Mutation-Positive NSCLC*<br>The safety of MEKINIST when administered with dabrafenib was evaluated in 93 patients with previously untreated (n = 36) and previously treated (n = 57) metastatic BRAF V600E mutation-positive NSCLC in a multicenter, multi-cohort, non-randomized, open-label trial (Study BRF113928). (…)<br>*Locally Advanced or Metastatic, BRAF V600E-Mutation Positive, Anaplastic Thyroid Cancer (ATC)*<br>The safety of MEKINIST when administered with dabrafenib was evaluated in a nine-cohort, multicenter, nonrandomized, open-label study in patients with rare cancers with the BRAF V600E mutation, including locally advanced or metastatic ATC (Study BRF117019). (…)<br>*Advanced BRAF V600E-Mutation Positive Tumors*<br>*Study BRF117019*<br>The safety of MEKINIST when administered with dabrafenib was evaluated in a multi-cohort, multi-center, non-randomized, open-label study in adult patients with cancers with the BRAF V600E mutation (Study BRF117019). A total of 206 patients were enrolled in the trial, 36 of whom were enrolled in the ATC cohort, 105 were enrolled in specific solid tumor cohorts, and 65 in other malignancies [see Clinical Studies (14.4, 14.6)]. Patients received MEKINIST 2 mg orally once daily and dabrafenib 150 mg orally twice daily until disease progression or unacceptable toxicity.<br><br>**8 USE IN SPECIFIC POPULATIONS**<br>**8.4 Pediatric Use**<br>BRAF V600E Mutation-Positive Unresectable or Metastatic Solid Tumors<br>The safety and effectiveness of MEKINIST in combination with dabrafenib in pediatric patients 6 years of age and older that weigh at least 26 kg was established based on data in adults and data from a pediatric study X2101. In study X2101, Parts C and D enrolled a total of 48 patients (ages 1 to 17) with the following tumor types: LGG (n = 34), HGG (n = 2), LCH (n = 11), and juvenile xanthogranulomatosis (n = 1) [see Adverse Reactions (6.1), Clinical Pharmacology (12.3), and Clinical Studies (14.6)].<br>The safety and effectiveness of MEKINIST in combination with dabrafenib in pediatric patients younger than 6 years old have not been established. The safety and effectiveness of MEKINIST as a single agent in pediatric patients have not been established.<br><br>**12 CLINICAL PHARMACOLOGY**<br>**12.2 Pharmacodynamics**<br>Administration of 1 mg and 2 mg MEKINIST to patients with BRAF V600 mutation-positive melanoma resulted in dose-dependent changes in tumor biomarkers including inhibition of phosphorylated ERK, inhibition of Ki67 (a marker of cell proliferation), and increases in p27 (a marker of apoptosis).<br>**12.3 Pharmacokinetics**<br>The pharmacokinetics (PK) of trametinib were characterized following single- and repeat-oral administration in patients with solid tumors and BRAF V600 mutation-positive metastatic melanoma. (…)<br><br>**14 CLINICAL STUDIES**<br>**14.1 BRAF V600E or V600K Mutation-Positive Unresectable or Metastatic Melanoma**<br>*Mekinist as a Single Agent*<br>The safety and efficacy of MEKINIST were evaluated in an international, multicenter, randomized (2:1), open-label, active-controlled trial (Trial 1) in 322 patients with BRAF V600E or V600K mutation-positive, unresectable or metastatic melanoma. (…)<br>(…) Tumor tissue was evaluated for BRAF mutations at a central testing site using a clinical trial assay. Tumor samples from 289 patients (196 patients treated with MEKINIST and 93 chemotherapy-treated patients) were also tested retrospectively using an FDA-approved companion diagnostic test, THxID™-BRAF assay. (…)<br>(…) The distribution of BRAF V600 mutations was BRAF V600E (87%), V600K (12%), or both (less than 1%). The median durations of follow-up prior to initiation of alternative treatment were 4.9 months for patients treated with MEKINIST and 3.1 months for patients treated with chemotherapy. Fifty-one (47%) patients crossed over from the chemotherapy arm at the time of disease progression to receive MEKINIST. (…)<br>*Mekinist with Dabrafenib*<br>*COMBI-d Study*<br>The safety and efficacy of MEKINIST administered with dabrafenib were evaluated in an international, randomized, double-blind, active-controlled trial (the COMBI-d study; NCT01584648). The COMBI-d study compared dabrafenib plus MEKINIST to dabrafenib plus placebo as first-line treatment for patients with unresectable (Stage IIIc) or metastatic (Stage IV) BRAF V600E or V600K mutation-positive cutaneous melanoma. Patients were randomized (1:1) to receive MEKINIST 2 mg once daily plus dabrafenib 150 mg twice daily or dabrafenib 150 mg twice daily plus matching placebo. Randomization was stratified by lactate dehydrogenase (LDH) level (greater than the upper limit of normal (ULN) vs. ≤ ULN) and BRAF mutation subtype (V600E vs. V600K). The major efficacy

* Therapeutic areas do not necessarily reflect the CDER review division.
† Representative biomarkers are listed based on standard nomenclature as per the Human Genome Organization (HUGO) symbol and/or simplified descriptors using other common conventions. Listed biomarkers do not necessarily reflect the terminology used in labeling. The term “Nonspecific” is provided when labeling does not explicitly identify the specific biomarker(s) or when the biomarker is represented by a molecular phenotype or gene signature, and in some cases the biomarker was inferred based on the labeling language.
‡ Referenced figures and tables may be found in the labeling available at Drugs@FDA.
Blue text represents the most recent addtions and/or changes since last posted version.

NDA/ANDA/BLA Number, Label Version Date	Drug	Therapeutic Area*	Biomarker†	Labeling Sections	Labeling Text‡
					outcome was investigator-assessed progression-free survival (PFS) per RECIST v1.1 with additional efficacy outcome measures of overall survival (OS) and confirmed overall response rate (ORR).<br>In the COMBI-d study, 423 patients were randomized to MEKINIST plus dabrafenib (n = 211) or dabrafenib plus placebo (n = 212). The median age was 56 years (range: 22 to 89 years), 53% were male, >99% were White, 72% had ECOG performance status of 0, 4% had Stage IIIc, 66% had M1c disease, 65% had a normal LDH, and 2 patients had a history of brain metastases. All patients had tumor containing BRAF V600E or V600K mutations as determined by centralized testing with the FDA-approved companion diagnostic test; 85% had BRAF V600E mutation-positive melanoma and 15% had BRAF V600K mutation-positive melanoma.<br>*COMBI-MB Study*<br>The activity of MEKINIST with dabrafenib for the treatment of BRAF V600E or V600K mutation-positive melanoma, metastatic to the brain, was evaluated in a non-randomized, open-label, multicenter, multi-cohort trial (the COMBI-MB study; NCT02039947). (…)<br>The COMBI-MB study enrolled 121 patients with a BRAF V600E (85%) or V600K (15%) mutation. The median age was 54 years (range: 23 to 84 years), 58% were male, 100% were white, 8% were from the United States, 65% had a normal LDH value at baseline, and 97% had an ECOG performance status of 0 or 1. Intracranial metastases were asymptomatic in 87% and symptomatic in 13% of patients, 22% received prior local therapy for brain metastases, and 87% also had extracranial metastases. (…)<br>**14.2 Adjuvant Treatment of BRAF V600E or V600K Mutation-Positive Melanoma**<br>COMBI-AD (NCT01682083) was an international, multi-center, randomized, double-blind, placebo-controlled trial that enrolled patients with Stage III melanoma with BRAF V600E or V600K mutations as detected by the THxID™-BRAF assay and pathologic involvement of regional lymph node(s). Patients were randomized (1:1) to receive MEKINIST 2 mg once daily in combination with dabrafenib 150 mg twice daily or two placebos for up to 1 year. Enrollment required complete resection of melanoma with complete lymphadenectomy within 12 weeks prior to randomization. The trial excluded patients with mucosal or ocular melanoma, unresectable intransit metastases, distant metastatic disease, or prior systemic anticancer treatment, including radiotherapy. Randomization was stratified by BRAF mutation status (V600E or V600K) and American Joint Committee on Cancer (AJCC; 7th Edition) stage (IIIa, IIIb, or IIIc). (…)<br>In COMBI-AD, a total of 870 patients were randomized: 438 to the MEKINIST in combination with dabrafenib and 432 to placebo. Median age was 51 years (range: 18 to 89), 55% were male, 99% were White, and 91% had an ECOG performance status of 0. Disease characteristics were AJCC Stage IIIa (18%), Stage IIIb (41%), Stage IIIc (40%), stage unknown (1%); BRAF V600E mutation (91%), BRAF V600K mutation (9%); macroscopic lymph nodes (65%); and tumor ulceration (41%). The median duration of follow-up (time from randomization to last contact or death) was 2.8 years. (See Table 13) (…)<br>**14.3 BRAF V600E Mutation-Positive Metastatic Non-Small Cell Lung Cancer (NSCLC)**<br>In Study BRF113928 (NCT01336634), the safety and efficacy of dabrafenib alone or administered with MEKINIST were evaluated in a multicenter, three-cohort, non-randomized, activity-estimating, open-label trial. Key eligibility criteria were locally confirmed BRAF V600E mutation-positive metastatic NSCLC, no prior exposure to BRAF or MEK inhibitor, and absence of EGFR mutation or ALK rearrangement (unless patients had progression on prior tyrosine kinase inhibitor therapy). (…)<br>In a subgroup analysis of patients with retrospectively centrally confirmed BRAF V600E mutation-positive NSCLC with the Oncomine™ Dx Target Test, the ORR results were similar to those presented in Table 14.<br>**14.4 BRAF V600E Mutation-Positive Locally Advanced or Metastatic Anaplastic Thyroid Cancer**<br>The safety and efficacy of MEKINIST administered with dabrafenib was evaluated in Study BRF117019 (NCT02034110), an activity-estimating, nine-cohort, multi-center, non-randomized, open-label trial in patients with rare cancers with the BRAF V600E mutation, including locally advanced, unresectable, or metastatic anaplastic thyroid cancer (ATC) with no standard locoregional treatment options. (…)<br>**14.5 Lack of Clinical Activity in Metastatic Melanoma Following BRAF-Inhibitor Therapy**<br>The clinical activity of MEKINIST as a single agent was evaluated in a single-arm, multicenter, international trial in 40 patients with BRAF V600E or V600K mutation-positive, unresectable or metastatic melanoma who had received prior treatment with a BRAF inhibitor. All patients received MEKINIST at a dose of 2 mg orally once daily until disease progression or unacceptable toxicity.<br>The median age was 58 years, 63% were male, all were White, 98% had baseline ECOG PS of 0 or 1, and the distribution of BRAF V600 mutations was V600E (83%), V600K (10%), and the remaining patients had multiple V600 mutations (5%), or unknown mutational status (2%). No patient achieved a confirmed partial or complete response as determined by the clinical investigators.<br>**14.6 BRAF V600E Mutation-Positive Unresectable or Metastatic Solid Tumors**<br>The safety and efficacy of MEKINIST in combination with dabrafenib for the treatment of BRAF V600E mutation-positive unresectable or metastatic solid tumors were evaluated in Trials BRF117019, NCI-MATCH, and CTMT212X2101, and supported by results in COMBI-d, COMBI-v [see Clinical Studies (14.2)], and BRF113928 [see Clinical Studies (14.4)]. In adult studies, patients received MEKINIST 2 mg once daily and dabrafenib 150 mg twice daily. The major efficacy outcome measures were ORR per RECIST v1.1, RANO [HGG] or modified RANO [LGG] criteria and duration of response (DoR).<br>*BRF117019 Study and NCI-MATCH Study*<br>Study BRF117019 (NCT02034110) [see Clinical Studies (14.5)] is a multi-cohort, multi-center, nonrandomized, open-label trial in adult patients with selected tumors with the BRAF V600E mutation, including high grade glioma (HGG) (n = 45), biliary tract cancer (BTC) (n = 43), low grade glioma (LGG) (n = 13), adenocarcinoma of small intestine (ASI) (n = 3), gastrointestinal stromal tumor (GIST) (n = 1), and anaplastic thyroid cancer [see Clinical Studies (14.5)]. Patients were enrolled based on local assessments of BRAF V600E mutation status; a central laboratory confirmed the BRAF V600E mutation in 93 of 105 patients.<br>Arm H (EAY131-H) of the NCI-MATCH study (NCT02465060) is a single-arm, open-label study that enrolled patients with a BRAF V600E mutation. Patients with melanoma, thyroid cancer, or CRC were excluded. BRAF mutation status for enrollment was determined either by central or local laboratory test. The study included adult patients with solid tumors including gastrointestinal tumors (n = 14), lung tumors (n = 7), gynecologic or peritoneal tumors (n = 6), CNS tumors (n = 4), and ameloblastoma of mandible (n = 1). (…)<br>Study X2101 (NCT02124772) was a multi-center, open-label, multiple cohort study in pediatric patients with refractory or recurrent solid tumors. Part C was a dose escalation of MEKINIST in combination with dabrafenib in patients with a BRAF V600E mutation. Part D was a cohort expansion phase of MEKINIST in

NDA/ANDA/BLA Number, Label Version Date	Drug	Therapeutic Area*	Biomarker†	Labeling Sections	Labeling Text‡
					combination with dabrafenib in patients with LGG with a BRAF V600E mutation. The major efficacy outcome measure was ORR as assessed by independent review committee per RANO criteria.<br>The efficacy of MEKINIST in combination with dabrafenib was evaluated in 48 pediatric patients, including 34 patients with LGG and 2 patients with HGG.<br>For patients with BRAF V600E mutant LGG and HGG in Parts C and D, the median age was 10 years (range: 1-17); 50% were male, 75% White, 8% Asian, 3% Black; and 58% had Karnofsky/Lansky performance status of 100. Prior anti-cancer treatments included surgery (83%), and external beam radiotherapy (2.8%), and systemic therapy (92%). The ORR was 25% (95% CI: 12%, 42%). For the 9 patients who responded, DoR was ≥6 months for 78% of patients and ≥24 months for 44% of patients.
**204114, 06/22/2022**	Trametinib (2)	Oncology	G6PD	Adverse Reactions	**6 ADVERSE REACTIONS**<br>**6.1 Clinical Trials Experience**<br>*MEKINIST with Dabrafenib*<br>(…) The trials excluded patients with abnormal left ventricular ejection fraction, history of acute coronary syndrome within 6 months, history of Class II or greater congestive heart failure (New York Heart Association), history of RVO or RPED, QTcB interval ≥480 msec, uncontrolled hypertension, uncontrolled arrhythmias, active brain metastases, or known history of G6PD deficiency. (…)
**204114, 06/22/2022**	Trametinib (3)	Oncology	RAS	Warnings and Precautions	**5 WARNINGS AND PRECAUTIONS**<br>**5.1 New Primary Malignancies**<br>*Non-Cutaneous Malignancies*<br>Based on its mechanism of action, dabrafenib may promote growth and development of malignancies with activation of RAS through mutation or other mechanisms; refer to the Prescribing Information for dabrafenib. Across clinical trials of MEKINIST administered with dabrafenib, non-cutaneous malignancies occurred in 1% of patients.
**103792, 11/29/2018**	Trastuzumab (1)	Oncology	ERBB2 (HER2)	Indications and Usage, Dosage and Administration, Clinical Pharmacology, Clinical Studies	**1 INDICATIONS AND USAGE**<br>**1.1 Adjuvant Breast Cancer**<br>Herceptin is indicated for adjuvant treatment of HER2 overexpressing node positive or node negative (ER/PR negative or with one high risk feature [see Clinical Studies (14.1)]) breast cancer<br>• as part of a treatment regimen consisting of doxorubicin, cyclophosphamide, and either paclitaxel or docetaxel<br>• with docetaxel and carboplatin<br>• as a single agent following multi-modality anthracycline based therapy.<br>**1.2 Metastatic Breast Cancer**<br>Herceptin is indicated:<br>• In combination with paclitaxel for first-line treatment of HER2-overexpressing metastatic breast cancer<br>• As a single agent for treatment of HER2-overexpressing breast cancer in patients who have received one or more chemotherapy regimens for metastatic disease.<br>**1.3 Metastatic Gastric Cancer**<br>Herceptin is indicated, in combination with cisplatin and capecitabine or 5-fluorouracil, for the treatment of patients with HER2 overexpressing metastatic gastric or gastroesophageal junction adenocarcinoma who have not received prior treatment for metastatic disease.<br><br>**2 DOSAGE AND ADMINISTRATION**<br>**2.1 Patient Selection**<br>Select patients based on HER2 protein overexpression or HER2 gene amplification in tumor specimens [see Indications and Usage (1) and Clinical Studies (14)]. Assessment of HER2 protein overexpression and HER2 gene amplification should be performed using FDA-approved tests specific for breast or gastric cancers by laboratories with demonstrated proficiency. Information on the FDA-approved tests for the detection of HER2 protein overexpression and HER2 gene amplification is available at: http://www.fda.gov/CompanionDiagnostics.<br>Assessment of HER2 protein overexpression and HER2 gene amplification in metastatic gastric cancer should be performed using FDA-approved tests specifically for gastric cancers due to differences in gastric vs. breast histopathology, including incomplete membrane staining and more frequent heterogeneous expression of HER2 seen in gastric cancers.<br>Improper assay performance, including use of suboptimally fixed tissue, failure to utilize specified reagents, deviation from specific assay instructions, and failure to include appropriate controls for assay validation, can lead to unreliable results.<br><br>**12 CLINICAL PHARMACOLOGY**<br>**12.2 Pharmacodynamics**<br>*Cardiac Electrophysiology*<br>The effects of trastuzumab on electrocardiographic (ECG) endpoints, including QTc interval duration, were evaluated in patients with HER2 positive solid tumors. Trastuzumab had no clinically relevant effect on the QTc interval duration and there was no apparent relationship between serum trastuzumab concentrations and change in QTcF interval duration in patients with HER2 positive solid tumors.<br><br>**14 CLINICAL STUDIES**<br>**14.1 Adjuvant Breast Cancer**<br>The safety and efficacy of Herceptin in women receiving adjuvant chemotherapy for HER2 overexpressing breast cancer were evaluated in an integrated analysis of two randomized, open-label, clinical trials (Studies 1 and 2) with a total of 4063 women at the protocol-specified final overall survival analysis, a third

* Therapeutic areas do not necessarily reflect the CDER review division.
† Representative biomarkers are listed based on standard nomenclature as per the Human Genome Organization (HUGO) symbol and/or simplified descriptors using other common conventions. Listed biomarkers do not necessarily reflect the terminology used in labeling. The term "Nonspecific" is provided when labeling does not explicitly identify the specific biomarker(s) or when the biomarker is represented by a molecular phenotype or gene signature, and in some cases the biomarker was inferred based on the labeling language.
‡ Referenced figures and tables may be found in the labeling available at Drugs@FDA.
Blue text represents the most recent addtions and/or changes since last posted version.

NDA/ANDA/BLA Number, Label Version Date	Drug	Therapeutic Area*	Biomarker†	Labeling Sections	Labeling Text‡
					randomized, open-label, clinical trial (Study 3) with a total of 3386 women at definitive Disease-Free Survival analysis for one-year Herceptin treatment versus observation, and a fourth randomized, open-label clinical trial with a total of 3222 patients (Study 4).<br>*Studies 1 and 2*<br>In Studies 1 and 2, breast tumor specimens were required to show HER2 overexpression (3+ by IHC) or gene amplification (by FISH). HER2 testing was verified by a central laboratory prior to randomization (Study 2) or was required to be performed at a reference laboratory (Study 1). (…)<br>*Study 3*<br>In Study 3, breast tumor specimens were required to show HER2 overexpression (3+ by IHC) or gene amplification (by FISH) as determined at a central laboratory. (…)<br>(…) Study 3 was designed to compare one and two years of three-weekly Herceptin treatment versus observation in patients with HER2 positive EBC following surgery, established chemotherapy and radiotherapy (if applicable). (…)<br>*Study 4*<br>In Study 4, breast tumor specimens were required to show HER2 gene amplification (FISH+ only) as determined at a central laboratory. (…)<br>(…) Exploratory analyses of DFS as a function of HER2 overexpression or gene amplification were conducted for patients in Studies 2 and 3, where central laboratory testing data were available.<br>The results are shown in Table 10. The number of events in Study 2 was small with the exception of the IHC 3+/FISH+ subgroup, which constituted 81% of those with data. Definitive conclusions cannot be drawn regarding efficacy within other subgroups due to the small number of events. The number of events in Study 3 was adequate to demonstrate significant effects on DFS in the IHC 3+/FISH unknown and the FISH +/IHC unknown subgroups. (See Table 10) (…)<br>**14.2 Metastatic Breast Cancer**<br>The safety and efficacy of Herceptin in treatment of women with metastatic breast cancer were studied in a randomized, controlled clinical trial in combination with chemotherapy (Study 5, n = 469 patients) and an open-label single agent clinical trial (Study 6, n = 222 patients). Both trials studied patients with metastatic breast cancer whose tumors overexpress the HER2 protein. Patients were eligible if they had 2 or 3 levels of overexpression (based on a 0 to 3 scale) by immunohistochemical assessment of tumor tissue performed by a central testing lab.<br>*Previously Untreated Metastatic Breast Cancer (Study 5)*<br>Study 5 was a multicenter, randomized, open-label clinical trial conducted in 469 women with metastatic breast cancer who had not been previously treated with chemotherapy for metastatic disease. Tumor specimens were tested by IHC (Clinical Trial Assay, CTA) and scored as 0, 1+, 2+, or 3+, with 3+ indicating the strongest positivity. Only patients with 2+ or 3+ positive tumors were eligible (about 33% of those screened). (…)<br>(…) Data from Study 5 suggest that the beneficial treatment effects were largely limited to patients with the highest level of HER2 protein overexpression (3+) (See Table 12). (…)<br>*Previously Treated Metastatic Breast Cancer (Study 6)*<br>Herceptin was studied as a single agent in a multicenter, open-label, single-arm clinical trial (Study 6) in patients with HER2 overexpressing metastatic breast cancer who had relapsed following one or two prior chemotherapy regimens for metastatic disease. (…)<br>**14.3 Metastatic Gastric Cancer**<br>The safety and efficacy of Herceptin in combination with cisplatin and a fluoropyrimidine (capecitabine or 5-fluorouracil) were studied in patients previously untreated for metastatic gastric or gastroesophageal junction adenocarcinoma (Study 7). In this open-label, multi-center trial, 594 patients were randomized 1:1 to Herceptin in combination with cisplatin and a fluoropyrimidine (FC+H) or chemotherapy alone (FC). Randomization was stratified by extent of disease (metastatic vs. locally advanced), primary site (gastric vs. gastroesophageal junction), tumor measurability (yes vs. no), ECOG performance status (0,1 vs. 2), and fluoropyrimidine (capecitabine vs. 5-fluorouracil). All patients were either HER2 gene amplified (FISH+) or HER2 overexpressing (IHC 3+). Patients were also required to have adequate cardiac function (e.g., LVEF > 50%). (…)<br>(…) An exploratory analysis of OS in patients based on HER2 gene amplification (FISH) and protein overexpression (IHC) testing is summarized in Table 14. (See Table 14)
**103792, 11/29/2018**	Trastuzumab (2)	Oncology	ESR, PGR (Hormone Receptor)	Clinical Studies	**14 CLINICAL STUDIES**<br>**14.1 Adjuvant Breast Cancer**<br>*Study 4*<br>(…) The final OS analysis results from Studies 1 and 2 indicate that OS benefit by age, hormone receptor status, number of positive lymph nodes, tumor size and grade, and surgery/radiation therapy was consistent with the treatment effect in the overall population. In patients ≤ 50 years of age (n = 2197), the OS hazard ratio was 0.65 (95% CI: 0.52, 0.81) and in patients > 50 years of age (n = 1866), the OS hazard ratio was 0.63 (95% CI: 0.51, 0.78). In the subgroup of patients with hormone receptor-positive disease (ER-positive and/or PR-positive) (n = 2223), the hazard ratio for OS was 0.63 (95% CI: 0.51, 0.78). In the subgroup of patients with hormone receptor-negative disease (ER-negative and PR-negative) (n = 1830), the hazard ratio for OS was 0.64 (95% CI: 0.52, 0.80). In the subgroup of patients with tumor size ≤ 2 cm (n = 1604), the hazard ratio for OS was 0.52 (95% CI: 0.39, 0.71). In the subgroup of patients with tumor size > 2 cm (n = 2448), the hazard ratio for OS was 0.67 (95% CI: 0.56, 0.80). (See Table 9) (…)
761270, 11/01/2022	Tremelimumab-actl (1)	Oncology	ALK	Indications and Usage, Clinical Studies	**1 INDICATIONS AND USAGE**<br>**1.2 Non-Small Cell Lung Cancer (NSCLC)**<br>IMJUDO, in combination with durvalumab and platinum-based chemotherapy, is indicated for the treatment of adult patients with metastatic NSCLC with no sensitizing epidermal growth factor receptor (EGFR) mutations or anaplastic lymphoma kinase (ALK) genomic tumor aberrations.<br><br>**14 CLINICAL STUDIES**<br>**14.2 Metastatic NSCLC**<br>*Metastatic NSCLC - POSEIDON*

* Therapeutic areas do not necessarily reflect the CDER review division.
† Representative biomarkers are listed based on standard nomenclature as per the Human Genome Organization (HUGO) symbol and/or simplified descriptors using other common conventions. Listed biomarkers do not necessarily reflect the terminology used in labeling. The term "Nonspecific" is provided when labeling does not explicitly identify the specific biomarker(s) or when the biomarker is represented by a molecular phenotype or gene signature, and in some cases the biomarker was inferred based on the labeling language.
‡ Referenced figures and tables may be found in the labeling available at Drugs@FDA.
Blue text represents the most recent addtions and/or changes since last posted version.

NDA/ANDA/BLA Number, Label Version Date	Drug	Therapeutic Area*	Biomarker†	Labeling Sections	Labeling Text‡
					The efficacy of IMJUDO in combination with durvalumab and platinum-based chemotherapy in previously untreated metastatic NSCLC patients with no sensitizing epidermal growth factor receptor (EGFR) mutation or anaplastic lymphoma kinase (ALK) genomic tumor aberrations was investigated in POSEIDON, a randomized, multicenter, active-controlled, open-label trial (NCT03164616). (…)
761270, 11/01/2022	Tremelimumab-actl (2)	Oncology	EGFR	Indications and Usage, Clinical Studies	**1 INDICATIONS AND USAGE**<br>**1.2 Non-Small Cell Lung Cancer (NSCLC)**<br>IMJUDO, in combination with durvalumab and platinum-based chemotherapy, is indicated for the treatment of adult patients with metastatic NSCLC with no sensitizing epidermal growth factor receptor (EGFR) mutations or anaplastic lymphoma kinase (ALK) genomic tumor aberrations.<br><br>**14 CLINICAL STUDIES**<br>**14.2 Metastatic NSCLC**<br>*Metastatic NSCLC - POSEIDON*<br>The efficacy of IMJUDO in combination with durvalumab and platinum-based chemotherapy in previously untreated metastatic NSCLC patients with no sensitizing epidermal growth factor receptor (EGFR) mutation or anaplastic lymphoma kinase (ALK) genomic tumor aberrations was investigated in POSEIDON, a randomized, multicenter, active-controlled, open-label trial (NCT03164616). (…)
761270, 11/01/2022	Tremelimumab-actl (3)	Oncology	CD274 (PD-L1)	Clinical Studies	**14 CLINICAL STUDIES**<br>**14.2 Metastatic NSCLC**<br>*Metastatic NSCLC - POSEIDON*<br>The efficacy of IMJUDO in combination with durvalumab and platinum-based chemotherapy in previously untreated metastatic NSCLC patients with no sensitizing epidermal growth factor receptor (EGFR) mutation or anaplastic lymphoma kinase (ALK) genomic tumor aberrations was investigated in POSEIDON, a randomized, multicenter, active-controlled, open-label trial (NCT03164616). (…)<br>Randomization was stratified by tumor cells (TC) PD-L1 expression (TC ≥ 50% vs. TC < 50%), disease stage (Stage IVA vs. Stage IVB), and histology (non-squamous vs. squamous). (…)<br>A total of 675 patients were randomized to receive either IMJUDO with durvalumab and platinum-based chemotherapy (n=338) or platinum-based chemotherapy (n=337). The median age was 63 years (range: 27 to 87), 46% of patients age ≥ 65 years, 77% male, 57% White, 34% Asian, 0.3% Native Hawaiian or Other Pacific Islander, 3% American Indian or Alaska Native, 2% Black or African American, 4% Other Race, 79% former or current smoker, 34% ECOG PS 0, and 66% ECOG PS 1. Thirty-six percent had squamous histology, 63% non-squamous histology, 29% PD-L1 expression TC ≥ 50%, 71% PD-L1 expression TC < 50%. (see Table 9)
**020438, 02/21/2023**	Tretinoin	Oncology	PML-RARA	Indications and Usage, Dosage and Administration, Warnings and Precautions, Patient Counseling Information	**1 INDICATIONS AND USAGE**<br>VESANOID is indicated for the induction of remission in adults and pediatric patients 1 year of age and older with acute promyelocytic leukemia (APL) characterized by the presence of the t(15;17) translocation or PML/RARα gene expression, and who are refractory to or who have relapsed from anthracycline chemotherapy or for whom anthracycline-based chemotherapy is contraindicated.<br><br>**2 DOSAGE AND ADMINISTRATION**<br>**2.2 Recommended Dosage**<br>The recommended dosage of VESANOID is 22.5 mg/m2 orally twice daily until complete remission is documented. Discontinue VESANOID 30 days after achievement of complete remission or after 90 days of treatment, whichever occurs first.<br>Discontinue VESANOID if the t(15;17) translocation or PML/RARα fusion has not been identified [see Warnings and Precautions (5.3)]. (…)<br><br>**5 WARNINGS AND PRECAUTIONS**<br>**5.3 Patients Without t(15;17) Translocation or PML/RARα Fusion**<br>VESANOID may be initiated based on the morphological diagnosis of acute promyelocytic leukemia (APL). Confirm the diagnosis of APL by detection of the t(15;17) translocation using cytogenetic studies or PML/RARα fusion using molecular diagnostic techniques. VESANOID is not recommended for use in patients without these genetic markers [see Indications and Usage (1)].<br><br>**17 PATIENT COUNSELING INFORMATION**<br>Patients Without t(15;17) Translocation or PML/RARα Fusion Advise patients that VESANOID is not recommended for use in patients without t(15;17) translocation or PML/RARα fusion [see Warnings and Precautions (5.3)].
**213687, 06/30/2020**	Triheptanoin	Inborn Errors of Metabolism	ACADVL, CPT2, HADHA, HADHB (Long-Chain Fatty Acid Oxidation Disorders)	Indications and Usage, Clinical Studies	**1 INDICATIONS AND USAGE**<br>DOJOLVI is indicated as a source of calories and fatty acids for the treatment of pediatric and adult patients with molecularly confirmed long-chain fatty acid oxidation disorders (LC-FAOD).<br><br>**14 CLINICAL STUDIES**<br>The efficacy of triheptanoin as a source of calories and fatty acids was evaluated in Study 3, a 4 month double-blind randomized controlled study comparing triheptanoin (7-carbon chain fatty acid) with trioctanoin (8-carbon chain fatty acid). The study enrolled 32 adult and pediatric patients with a confirmed diagnosis of LC-FAOD and evidence of at least one significant episode of rhabdomyolysis and at least two of the following diagnostic criteria: disease specific elevation of acylcarnitines on a new born blood spot or in plasma, low enzyme activity in cultured fibroblasts, or one or more known pathogenic mutations in CPT2, ACADVL, HADHA, or HADHB. (…)
**016792,**	Trimipramine	Psychiatry	CYP2D6	Precautions	**PRECAUTIONS**

* Therapeutic areas do not necessarily reflect the CDER review division.
† Representative biomarkers are listed based on standard nomenclature as per the Human Genome Organization (HUGO) symbol and/or simplified descriptors using other common conventions. Listed biomarkers do not necessarily reflect the terminology used in labeling. The term “Nonspecific” is provided when labeling does not explicitly identify the specific biomarker(s) or when the biomarker is represented by a molecular phenotype or gene signature, and in some cases the biomarker was inferred based on the labeling language.
‡ Referenced figures and tables may be found in the labeling available at Drugs@FDA.
Blue text represents the most recent addtions and/or changes since last posted version.

NDA/ANDA/BLA Number, Label Version Date	Drug	Therapeutic Area*	Biomarker†	Labeling Sections	Labeling Text‡
**07/17/2014**					*Drugs Metabolized by P450 2D6*<br>The biochemical activity of the drug metabolizing isozyme cytochrome P450 2D6 (debrisoquin hydroxylase) is reduced in a subset of the Caucasian population (about 7-10% of Caucasians are so called “poor metabolizers”); reliable estimates of the prevalence of reduced P450 2D6 isozyme activity among Asian, African, and other populations are not yet available. Poor metabolizers have higher than expected plasma concentrations of tricyclic antidepressants (TCAs) when given usual doses. Depending on the fraction of drug metabolized by P450 2D6, the increase in plasma concentration may be small, or quite large (8 fold increase in plasma AUC of the TCA). In addition, certain drugs inhibit the activity of the isozyme and make normal metabolizers resemble poor metabolizers. An individual who is stable on a given dose of TCA may become abruptly toxic when given one of these inhibiting drugs as concomitant therapy. (…)
217026, 03/10/2023	Trofinetide	Neurology	MECP2	Clinical Studies	**14 CLINICAL STUDIES**<br>The efficacy of DAYBUE for the treatment of Rett syndrome was established in a 12-week randomized, double-blind, placebo-controlled study in patients with Rett syndrome 5 to 20 years of age (Study 1; NCT04181723).<br>Patients (N=187) had a diagnosis of typical Rett syndrome according to the Rett Syndrome Diagnostic Criteria with a documented disease-causing mutation in the MECP2 gene. Patients were randomized to receive DAYBUE (N=93) or matching placebo (N=94) for 12 weeks. The DAYBUE dosage was based on patient weight to achieve similar exposure in all patients [see Dosage and Administration (2.1)].
**213411, 01/19/2023**	Tucatinib (1)	Oncology	ERBB2 (HER2)	Indications and Usage, Dosage and Administration, Adverse Reactions, Clinical Studies	**1 INDICATIONS AND USAGE**<br>**1.1 Metastatic Breast Cancer**<br>TUKYSA is indicated in combination with trastuzumab and capecitabine for treatment of adult patients with advanced unresectable or metastatic HER2-positive breast cancer, including patients with brain metastases, who have received one or more prior anti-HER2-based regimens in the metastatic setting.<br>**1.2 Unresectable or Metastatic Colorectal Cancer**<br>TUKYSA is indicated in combination with trastuzumab for the treatment of adult patients with RAS wild-type, HER2-positive unresectable or metastatic colorectal cancer that has progressed following treatment with fluoropyrimidine-, oxaliplatin-, and irinotecan-based chemotherapy.<br>This indication is approved under accelerated approval based on tumor response rate and durability of response [see Clinical Studies (14.2)]. Continued approval for this indication may be contingent upon verification and description of clinical benefit in confirmatory trials.<br><br>**2 DOSAGE AND ADMINISTRATION**<br>**2.1 Patient Selection**<br>Select patients for treatment of unresectable or metastatic colorectal cancer with TUKYSA based on the presence of:<br>• HER2 overexpression or gene amplification [see Clinical Studies (14.2)]. FDA-approved tests for the detection of HER2 overexpression and gene amplification in patients with unresectable or metastatic colorectal cancer are not currently available, and<br>• RAS wild-type [see Clinical Studies (14.2)]. Information on FDA-approved tests for the detection of RAS mutations in patients with unresectable or metastatic colorectal cancer is available at http://www.fda.gov/CompanionDiagnostics.<br><br>**6 ADVERSE REACTIONS**<br>HER2-Positive Metastatic Breast Cancer<br>(…) The safety of TUKYSA in combination with trastuzumab and capecitabine was evaluated in HER2CLIMB [see Clinical Studies (14)]. Patients received either TUKYSA 300 mg twice daily plus trastuzumab and capecitabine (n=404) or placebo plus trastuzumab and capecitabine (n=197). The median duration of treatment was 5.8 months (range: 3 days, 2.9 years) for the TUKYSA arm. (…)<br>RAS Wild-Type, HER2-Positive Unresectable or Metastatic Colorectal Cancer<br>The safety of TUKYSA in combination with trastuzumab or a non-US approved trastuzumab product was evaluated in 86 patients enrolled in MOUNTAINEER with unresectable or metastatic colorectal cancer [see Clinical Studies (14.2)]. The median duration of exposure to TUKYSA was 6.9 months (range 0.7, 49.3).<br><br>**14 CLINICAL STUDIES**<br>**14.1 HER2-Positive Metastatic Breast Cancer**<br>The efficacy of TUKYSA in combination with trastuzumab and capecitabine was evaluated in 612 patients in HER2CLIMB (NCT02614794), a randomized (2:1), double-blind, placebo-controlled trial. Patients were required to have HER2-positive, unresectable locally advanced or metastatic breast cancer, with or without brain metastases, and prior treatment with trastuzumab, pertuzumab, and ado-trastuzumab emtansine (T-DM1) separately or in combination, in the neoadjuvant, adjuvant or metastatic setting. HER2 positivity was based on archival or fresh tissue tested with an FDA-approved test at a central laboratory prior to enrollment with HER2 positivity defined as HER2 IHC 3+ or ISH positive. (…)<br>**14.2 HER2-Positive Metastatic Colorectal Cancer**<br>The efficacy of TUKYSA in combination with trastuzumab was evaluated in 84 patients in MOUNTAINEER (NCT03043313), an open-label, multicenter trial. Patients were required to have HER2-positive, RAS wildtype, unresectable or metastatic colorectal cancer and prior treatment with fluoropyrimidines, oxaliplatin, irinotecan, and anti-vascular endothelial growth factor (VEGF) monoclonal antibody (mAb). Patients whose disease had deficient mismatch repair (dMMR) proteins or microsatellite instability-high (MSI-H) must have also received an anti-programmed cell death protein-1 (PD-1) mAb. Patients who received prior anti-HER2 targeting therapy were excluded. HER2 positivity as defined by HER2 overexpression or gene amplification was prospectively determined in local laboratories using immunohistochemistry (IHC), in situ hybridization (ISH), and/or next generation sequencing (NGS) on tumor tissue. RAS status was performed as standard of care prior to study entry based on expanded RAS testing including KRAS exons 2, 3, and 4 and NRAS exons 2, 3, and 4. (…)
**213411, 01/19/2023**	Tucatinib (2)	Oncology	Microsatellite Instability, Mismatch Repair	Indications and Usage, Dosage and Administration,	**1 INDICATIONS AND USAGE**<br>**1.2 Unresectable or Metastatic Colorectal Cancer**<br>TUKYSA is indicated in combination with trastuzumab for the treatment of adult patients with RAS wild-type, HER2-positive unresectable or metastatic colorectal cancer that has progressed following treatment with fluoropyrimidine-, oxaliplatin-, and irinotecan-based chemotherapy.

NDA/ANDA/BLA Number, Label Version Date	Drug	Therapeutic Area*	Biomarker†	Labeling Sections	Labeling Text‡
				Adverse Reactions, Clinical Studies	This indication is approved under accelerated approval based on tumor response rate and durability of response [see Clinical Studies (14.2)]. Continued approval for this indication may be contingent upon verification and description of clinical benefit in confirmatory trials.<br><br>**2 DOSAGE AND ADMINISTRATION**<br>**2.1 Patient Selection**<br>Select patients for treatment of unresectable or metastatic colorectal cancer with TUKYSA based on the presence of:<br>• RAS wild-type [see Clinical Studies (14.2)]. Information on FDA-approved tests for the detection of RAS mutations in patients with unresectable or metastatic colorectal cancer is available at http://www.fda.gov/CompanionDiagnostics.<br><br>**6 ADVERSE REACTIONS**<br>RAS Wild-Type, HER2-Positive Unresectable or Metastatic Colorectal Cancer<br>The safety of TUKYSA in combination with trastuzumab or a non-US approved trastuzumab product was evaluated in 86 patients enrolled in MOUNTAINEER with unresectable or metastatic colorectal cancer [see Clinical Studies (14.2)]. The median duration of exposure to TUKYSA was 6.9 months (range 0.7, 49.3).<br><br>**14 CLINICAL STUDIES**<br>**14.2 HER2-Positive Metastatic Colorectal Cancer**<br>The efficacy of TUKYSA in combination with trastuzumab was evaluated in 84 patients in MOUNTAINEER (NCT03043313), an open-label, multicenter trial. Patients were required to have HER2-positive, RAS wildtype, unresectable or metastatic colorectal cancer and prior treatment with fluoropyrimidines, oxaliplatin, irinotecan, and anti-vascular endothelial growth factor (VEGF) monoclonal antibody (mAb). Patients whose disease had deficient mismatch repair (dMMR) proteins or microsatellite instability-high (MSI-H) must have also received an anti-programmed cell death protein-1 (PD-1) mAb. Patients who received prior anti-HER2 targeting therapy were excluded. HER2 positivity as defined by HER2 overexpression or gene amplification was prospectively determined in local laboratories using immunohistochemistry (IHC), in situ hybridization (ISH), and/or next generation sequencing (NGS) on tumor tissue. RAS status was performed as standard of care prior to study entry based on expanded RAS testing including KRAS exons 2, 3, and 4 and NRAS exons 2, 3, and 4. (…)
**213411, 01/19/2023**	Tucatinib (3)	Oncology	RAS	Clinical Studies	**14 CLINICAL STUDIES**<br>**14.2 HER2-Positive Metastatic Colorectal Cancer**<br>The efficacy of TUKYSA in combination with trastuzumab was evaluated in 84 patients in MOUNTAINEER (NCT03043313), an open-label, multicenter trial. Patients were required to have HER2-positive, RAS wildtype, unresectable or metastatic colorectal cancer and prior treatment with fluoropyrimidines, oxaliplatin, irinotecan, and anti-vascular endothelial growth factor (VEGF) monoclonal antibody (mAb). Patients whose disease had deficient mismatch repair (dMMR) proteins or microsatellite instability-high (MSI-H) must have also received an anti-programmed cell death protein-1 (PD-1) mAb. Patients who received prior anti-HER2 targeting therapy were excluded. HER2 positivity as defined by HER2 overexpression or gene amplification was prospectively determined in local laboratories using immunohistochemistry (IHC), in situ hybridization (ISH), and/or next generation sequencing (NGS) on tumor tissue. RAS status was performed as standard of care prior to study entry based on expanded RAS testing including KRAS exons 2, 3, and 4 and NRAS exons 2, 3, and 4. (…)
**205382, 05/11/2022**	Umeclidinium	Pulmonary	CYP2D6	Clinical Pharmacology	**12 CLINICAL PHARMACOLOGY**<br>**12.3 Pharmacokinetics**<br>***Umeclidinium and Cytochrome P450 2D6:***<br>In vitro metabolism of umeclidinium is mediated primarily by CYP2D6. However, no clinically meaningful difference in systemic exposure to umeclidinium (500 mcg) (8 times the approved dose) was observed following repeat daily inhaled dosing to normal (ultrarapid, extensive, and intermediate metabolizers) and CYP2D6 poor metabolizer subjects (Figure 1).
**211675, 08/16/2019**	Upadacitinib	Rheumatology	CYP2D6	Clinical Pharmacology	**12 CLINICAL PHARMACOLOGY**<br>**12.3 Pharmacokinetics**<br>Drug Interaction Studies<br>*Potential for Other Drugs to Influence the Pharmacokinetics of Upadacitinib*<br>Upadacitinib is metabolized in vitro by CYP3A4 with a minor contribution from CYP2D6. The effect of co-administered drugs on upadacitinib plasma exposures is provided in Table 3 [see Drug Interactions (7)].<br>pH modifying medications (e.g., antacids or proton pump inhibitors) are not expected to affect upadacitinib plasma exposures based on in vitro assessments and population pharmacokinetic analyses. CYP2D6 metabolic phenotype had no effect on upadacitinib pharmacokinetics (based on population pharmacokinetic analyses), indicating that inhibitors of CYP2D6 have no clinically relevant effect on upadacitinib exposures.
**761044, 09/23/2016**	Ustekinumab	Dermatology and Gastroenterology	IL12A, IL12B, IL23A	Warnings and Precautions	**5 WARNINGS AND PRECAUTIONS**<br>**5.2 Theoretical Risk for Vulnerability to Particular Infections**<br>Individuals genetically deficient in IL-12/IL-23 are particularly vulnerable to disseminated infections from mycobacteria (including nontuberculous, environmental mycobacteria), salmonella (including nontyphi strains), and Bacillus Calmette-Guerin (BCG) vaccinations. Serious infections and fatal outcomes have been reported in such patients.<br>It is not known whether patients with pharmacologic blockade of IL-12/IL-23 from treatment with STELARA® may be susceptible to these types of infections. Appropriate diagnostic testing should be considered, e.g., tissue culture, stool culture, as dictated by clinical circumstances.
**209241, 08/18/2023**	Valbenazine	Neurology	CYP2D6	Dosage and Administration, Warnings and Precautions, Use in Specific	**2 DOSAGE AND ADMINISTRATION**<br>**2.3 Dosage Recommendations for Known CYP2D6 Poor Metabolizers**<br>The recommended dosage for known CYP2D6 poor metabolizers is INGREZZA 40 mg once daily [see Use in Specific Populations (8.6), Clinical Pharmacology (12.3, 12.5)].

* Therapeutic areas do not necessarily reflect the CDER review division.
† Representative biomarkers are listed based on standard nomenclature as per the Human Genome Organization (HUGO) symbol and/or simplified descriptors using other common conventions. Listed biomarkers do not necessarily reflect the terminology used in labeling. The term “Nonspecific” is provided when labeling does not explicitly identify the specific biomarker(s) or when the biomarker is represented by a molecular phenotype or gene signature, and in some cases the biomarker was inferred based on the labeling language.
‡ Referenced figures and tables may be found in the labeling available at Drugs@FDA.
Blue text represents the most recent addtions and/or changes since last posted version.

NDA/ANDA/BLA Number, Label Version Date	Drug	Therapeutic Area*	Biomarker†	Labeling Sections	Labeling Text‡
				Populations, Clinical Pharmacology	**5 WARNINGS AND PRECAUTIONS**<br>**5.4 QT Prolongation**<br>INGREZZA may prolong the QT interval, although the degree of QT prolongation is not clinically significant at concentrations expected with recommended dosing. In patients taking a strong CYP2D6 or CYP3A4 inhibitor, or who are CYP2D6 poor metabolizers, INGREZZA concentrations may be higher and QT prolongation clinically significant [see Clinical Pharmacology (12.2)]. For patients who are CYP2D6 poor metabolizers or are taking a strong CYP2D6 inhibitor, dose reduction may be necessary. For patients taking a strong CYP3A4 inhibitor, reduce the dose of INGREZZA to 40 mg once daily [see Dosage and Administration (2.3, 2.4)]. INGREZZA should be avoided in patients with congenital long QT syndrome or with arrhythmias associated with a prolonged QT interval. For patients at increased risk of a prolonged QT interval, assess the QT interval before increasing the dosage.<br><br>**8 USE IN SPECIFIC POPULATIONS**<br>**8.6 CYP2D6 Poor Metabolizers**<br>Dosage reduction of INGREZZA is recommended for known CYP2D6 poor metabolizers [see Dosage and Administration (2.3)]. Increased exposure (Cmax and AUC) to valbenazine's active metabolite was observed in CYP2D6 poor metabolizers. Increased exposure of active metabolite may increase the risk of exposure-related adverse reactions [see Clinical Pharmacology (12.3, 12.5)].<br><br>**12 CLINICAL PHARMACOLOGY**<br>**12.2 Pharmacodynamic**<br>*Cardiac Electrophysiology*<br>(…) INGREZZA may cause an increase in the corrected QT interval in patients who are CYP2D6 poor metabolizers or who are taking a strong CYP2D6 or CYP3A4 inhibitor. An exposure-response analysis of clinical data from two healthy volunteer studies revealed increased QTc interval with higher plasma concentrations of the active metabolite. Based on this model, patients taking an INGREZZA 60 mg or 80 mg dose with increased exposure to the metabolite (e.g., being a CYP2D6 poor metabolizer) may have a mean (upper bound of double-sided 90% CI) QT prolongation of 9.6 (12.0) msec or 11.7 (14.7) msec, respectively as compared to otherwise healthy volunteers given INGREZZA, who had a respective mean (upper bound of double-sided 90% CI) QT prolongation of 5.3 (6.7) msec or 6.7 (8.4) msec [see Warnings and Precautions (5.4)]<br>**12.5 Pharmacogenomics**<br>CYP2D6 metabolizes the active metabolite of valbenazine ([+]-α-HTBZ). The gene encoding CYP2D6 has polymorphisms that impact protein function. CYP2D6 poor metabolizers are individuals with two nonfunctioning alleles, resulting in no enzyme activity.<br>Pharmacokinetic data from CYP2D6 poor metabolizers (n=25) treated with valbenazine demonstrate an approximate 2-fold higher AUCinf and a 1.8-fold higher Cmax, of ([+]-α-HTBZ) compared to normal metabolizers. Dosage reduction is recommended in CYP2D6 poor metabolizers [see Dosage and Administration (2.3), Warnings and Precautions (5.3), and Use in Specific Populations (8.6)].<br>In a clinical study, AUC of [+]-α-HTBZ was 22% higher and Cmax was 9% lower in intermediate metabolizers (n=7) as compared to normal metabolizers (n=11), which is not considered clinically relevant. The effects of ultrarapid metabolizer status on the pharmacokinetics of [+]-α-HTBZ have not been studied.<br>Approximately 7% of White populations, 2% of Asian populations, and 2% of African-American populations are poor metabolizers.
**018081, 02/21/2019**	Valproic Acid (1)	Neurology	POLG	Boxed Warning, Contraindications, Warnings and Precautions	**BOXED WARNING**<br>WARNING: LIFE THREATENING ADVERSE REACTIONS<br>*Patients with Mitochondrial Disease*<br>There is an increased risk of valproate-induced acute liver failure and resultant deaths in patients with hereditary neurometabolic syndromes caused by DNA mutations of the mitochondrial DNA Polymerase γ (POLG) gene (e.g., Alpers Huttenlocher Syndrome). Depakene is contraindicated in patients known to have mitochondrial disorders caused by POLG mutations and children under two years of age who are clinically suspected of having a mitochondrial disorder [see Contraindications (4)]. In patients over two years of age who are clinically suspected of having a hereditary mitochondrial disease, Depakene should only be used after other anticonvulsants have failed. This older group of patients should be closely monitored during treatment with Depakene for the development of acute liver injury with regular clinical assessments and serum liver testing. POLG mutation screening should be performed in accordance with current clinical practice [see Warnings and Precautions (5.1)].<br><br>**4 CONTRAINDICATIONS**<br>(…) Depakene is contraindicated in patients known to have mitochondrial disorders caused by mutations in mitochondrial DNA polymerase γ (POLG; e.g., Alpers-Huttenlocher Syndrome) and children under two years of age who are suspected of having a POLG-related disorder [see Warnings and Precautions (5.1)]. (…)<br><br>**5 WARNINGS AND PRECAUTIONS**<br>**5.1 Hepatotoxicity**<br>*Patients with Known or Suspected Mitochondrial Disease*<br>Depakene is contraindicated in patients known to have mitochondrial disorders caused by POLG mutations and children under two years of age who are clinically suspected of having a mitochondrial disorder [see Contraindications (4)]. Valproate-induced acute liver failure and liver-related deaths have been reported in patients with hereditary neurometabolic syndromes caused by mutations in the gene for mitochondrial DNA polymerase γ (POLG) (e.g., Alpers-Huttenlocher Syndrome) at a higher rate than those without these syndromes. Most of the reported cases of liver failure in patients with these syndromes have been identified in children and adolescents.<br>POLG-related disorders should be suspected in patients with a family history or suggestive symptoms of a POLG-related disorder, including but not limited to unexplained encephalopathy, refractory epilepsy (focal, myoclonic), status epilepticus at presentation, developmental delays, psychomotor regression, axonal sensorimotor neuropathy, myopathy cerebellar ataxia, ophthalmoplegia, or complicated migraine with occipital aura. POLG mutation testing should be performed

NDA/ANDA/BLA Number, Label Version Date	Drug	Therapeutic Area*	Biomarker†	Labeling Sections	Labeling Text‡
					in accordance with current clinical practice for the diagnostic evaluation of such disorders. The A467T and W748S mutations are present in approximately 2/3 of patients with autosomal recessive POLG-related disorders.<br>In patients over two years of age who are clinically suspected of having a hereditary mitochondrial disease, Depakene should only be used after other anticonvulsants have failed. This older group of patients should be closely monitored during treatment with Depakene for the development of acute liver injury with regular clinical assessments and serum liver test monitoring.<br>The drug should be discontinued immediately in the presence of significant hepatic dysfunction, suspected or apparent. In some cases, hepatic dysfunction has progressed in spite of discontinuation of drug [see Boxed Warning and Contraindications (4)].
**018081, 02/21/2019**	Valproic Acid (2)	Neurology	Nonspecific (Urea Cycle Disorders)	Contraindications, Warnings and Precautions	**4 CONTRAINDICATIONS**<br>(…) Depakene is contraindicated in patients with known urea cycle disorders [see Warnings and Precautions (5.6)].<br><br>**5 WARNINGS AND PRECAUTIONS**<br>**5.6 Urea Cycle Disorders (UCD)**<br>Valproic acid is contraindicated in patients with known urea cycle disorders. Hyperammonemic encephalopathy, sometimes fatal, has been reported following initiation of valproate therapy in patients with urea cycle disorders, a group of uncommon genetic abnormalities, particularly ornithine transcarbamylase deficiency. Prior to the initiation of valproate therapy, evaluation for UCD should be considered in the following patients: 1) those with a history of unexplained encephalopathy or coma, encephalopathy associated with a protein load, pregnancy-related or postpartum encephalopathy, unexplained mental retardation, or history of elevated plasma ammonia or glutamine; 2) those with cyclical vomiting and lethargy, episodic extreme irritability, ataxia, low BUN, or protein avoidance; 3) those with a family history of UCD or a family history of unexplained infant deaths (particularly males); 4) those with other signs or symptoms of UCD. Patients who develop symptoms of unexplained hyperammonemic encephalopathy while receiving valproate therapy should receive prompt treatment (including discontinuation of valproate therapy) and be evaluated for underlying urea cycle disorders [see Contraindications (4) and Warnings and Precautions (5.10)].<br>**5.9 Hyperammonemia**<br>Hyperammonemia has been reported in association with valproate therapy and may be present despite normal liver function tests. In patients who develop unexplained lethargy and vomiting or changes in mental status, hyperammonemic encephalopathy should be considered and an ammonia level should be measured. Hyperammonemia should also be considered in patients who present with hypothermia [see Warnings and Precautions (5.11)]. If ammonia is increased, valproate therapy should be discontinued. Appropriate interventions for treatment of hyperammonemia should be initiated, and such patients should undergo investigation for underlying urea cycle disorders [see Contraindications (4) and Warnings and Precautions (5.6, 5.10)].<br>Asymptomatic elevations of ammonia are more common and when present, require close monitoring of plasma ammonia levels. If the elevation persists, discontinuation of valproate therapy should be considered.
**202429, 05/18/2020**	Vemurafenib (1)	Oncology	BRAF	Indications and Usage, Dosage and Administration, Warnings and Precautions, Adverse Reasctions, Use in Specific Populations, Clinical Pharmacology, Clinical Studies, Patient Counseling Information	**1 INDICATIONS AND USAGE**<br>**1.1 Unresectable or Metastatic Melanoma**<br>ZELBORAF® is indicated for the treatment of patients with unresectable or metastatic melanoma with BRAF V600E mutation as detected by an FDA-approved test.<br>Limitation of Use: ZELBORAF is not indicated for treatment of patients with wild-type BRAF melanoma [see Warnings and Precautions (5.2)].<br>**1.2 Erdheim-Chester Disease**<br>ZELBORAF® is indicated for the treatment of patients with Erdheim-Chester Disease (ECD) with BRAF V600 mutation.<br><br>**2 DOSAGE AND ADMINISTRATION**<br>**2.1 Patient Selection**<br>Confirm the presence of BRAF V600E mutation in melanoma tumor specimens prior to initiation of treatment with ZELBORAF [see Warnings and Precautions (5.2)]. Information on FDA-approved tests for the detection of BRAF V600 mutations in melanoma is available at http://www.fda.gov/CompanionDiagnostics.<br><br>**5 WARNINGS AND PRECAUTIONS**<br>**5.2 Tumor Promotion in BRAF Wild-Type Melanoma**<br>In vitro experiments have demonstrated paradoxical activation of MAP-kinase signaling and increased cell proliferation in BRAF wild-type cells that are exposed to BRAF inhibitors. Confirm evidence of BRAF V600E mutation in tumor specimens prior to initiation of ZELBORAF [see Indications and Usage (1) and Dosage and Administration (2.1)].<br>**5.5 QT Prolongation**<br>Concentration-dependent QT prolongation occurred in an uncontrolled, open-label QT sub-study in previously treated patients with BRAF V600E mutation-positive metastatic melanoma [see Clinical Pharmacology (12.2)]. (…)<br><br>**6 ADVERSE REACTIONS**<br>**6.1 Clinical Trials Experience**<br>(…) *Unresectable or Metastatic Melanoma with BRAF V600E Mutation*<br>This section describes adverse drug reactions (ADRs) identified from analyses of Trial 1 and Trial 2 [see Clinical Studies (14)]. (…)<br>*Erdheim-Chester Disease (ECD)*<br>This section describes adverse reactions identified from analyses of Trial 4 [see Clinical Studies (14)]. In Trial 4, 22 patients with BRAF V600 mutation-positive ECD received ZELBORAF 960 mg twice daily.

* Therapeutic areas do not necessarily reflect the CDER review division.
† Representative biomarkers are listed based on standard nomenclature as per the Human Genome Organization (HUGO) symbol and/or simplified descriptors using other common conventions. Listed biomarkers do not necessarily reflect the terminology used in labeling. The term “Nonspecific” is provided when labeling does not explicitly identify the specific biomarker(s) or when the biomarker is represented by a molecular phenotype or gene signature, and in some cases the biomarker was inferred based on the labeling language.
‡ Referenced figures and tables may be found in the labeling available at Drugs@FDA.
Blue text represents the most recent addtions and/or changes since last posted version.

NDA/ANDA/BLA Number, Label Version Date	Drug	Therapeutic Area*	Biomarker†	Labeling Sections	Labeling Text‡
					The median treatment duration for ECD patients in this study was 14.2 months. Table 3 presents adverse reactions reported in at least 20% of BRAF V600 mutation-positive ECD patients treated with ZELBORAF.<br>In Trial 4, the most commonly reported adverse reactions (> 50%) in patients with BRAF V600 mutationpositive ECD treated with ZELBORAF were arthralgia, rash maculo-papular, alopecia, fatigue, electrocardiogram QT interval prolonged, and skin papilloma. The most common (≥ 10%) Grade □3 adverse reactions were squamous cell carcinoma of the skin, hypertension, rash maculo-papular, and arthralgia. (…)<br><br>**8 USE IN SPECIFIC POPULATIONS**<br>**8.4 Pediatric Use**<br>The safety and effectiveness of ZELBORAF in pediatric patients have not been established. Vemurafenib was studied in 6 adolescent patients 15 to 17 years of age with unresectable or metastatic melanoma with BRAF V600 mutation. A maximum tolerated dose was not reached with doses up to vemurafenib 960 mg twice daily. No new safety signals were observed. Vemurafenib steady-state exposure in these 6 adolescent patients was generally similar to that in adults.<br><br>**12 CLINICAL PHARMACOLOGY**<br>**12.2 Pharmacodynamics**<br>*Cardiac Electrophysiology*<br>In a multi-center, open-label, single-arm study in 132 patients with BRAF V600E mutation-positive metastatic melanoma, patients administered vemurafenib 960 mg orally twice daily did not experience large changes in mean QTc interval (i.e., > 20 ms) from baseline. (…)<br>**12.3 Pharmacokinetics**<br>The pharmacokinetics of vemurafenib were determined in patients with BRAF mutation-positive metastatic melanoma following 15 days of 960 mg twice daily with dosing approximately 12 hours apart. The population pharmacokinetic analysis pooled data from 458 patients. At steady-state, vemurafenib exhibits linear pharmacokinetics within the 240 mg to 960 mg dose range.<br><br>**14 CLINICAL STUDIES**<br>*Treatment-Naïve Patients with BRAF V600E Mutation-Positive Unresectable or Metastatic Melanoma*<br>Trial 1, an international, open-label, randomized controlled trial, equally allocated 675 patients with treatment-naive, BRAF V600E mutation-positive unresectable or metastatic melanoma, as detected by the cobas® 4800 BRAF V600 Mutation Test, to receive ZELBORAF 960 mg by mouth twice daily (n=337) or dacarbazine 1000 mg/m^2 intravenously on Day 1 every 3 weeks (n=338). (See Table 5) (…)<br>*Patients with BRAF V600E Mutation-Positive Metastatic Melanoma Who Received Prior Systemic Therapy* (…) In a single-arm, multicenter, multinational trial (Trial 2), 132 patients with BRAF V600E mutation-positive metastatic melanoma, as detected by the cobas® 4800 BRAF V600 Mutation Test, who had received at least one prior systemic therapy, received ZELBORAF 960 mg by mouth twice daily.. (…)<br>*Patients with BRAF V600E Mutation-Positive Melanoma with Brain Metastases*<br>The activity of ZELBORAF for the treatment of BRAF V600E mutation-positive melanoma, metastatic to the brain was evaluated in an open-label, multicenter, single-arm, two cohort trial (Trial 3). (See Table 6)(…)<br>*Patients with Wild-Type BRAF Melanoma*<br>ZELBORAF has not been studied in patients with wild-type BRAF melanoma [see Warnings and Precautions (5.2)].<br>*Patients with Erdheim-Chester Disease (ECD)*<br>An open-label, multicenter, single-arm, multiple cohort study of ZELBORAF (Trial 4) was conducted in patients ≥ 16 years of age with non-melanoma BRAF V600 mutation–positive diseases. (…)<br><br>**17 PATIENT COUNSELING INFORMATION**<br>Healthcare providers should advise patients of the potential benefits and risks of ZELBORAF and instruct their patients to read the Medication Guide before starting ZELBORAF therapy. Inform patients of the following:<br>• Evidence of BRAF V600E mutation in the tumor specimen with an FDA approved test is necessary to identify patients for whom treatment with ZELBORAF is indicated [see Dosage and Administration (2.1)]. (…)
**202429, 05/18/2020**	Vemurafenib (2)	Oncology	RAS	Warnings and Precautions, Adverse Reactions	**5 WARNINGS AND PRECAUTIONS**<br>*Other Malignancies*<br>Based on mechanism of action, ZELBORAF may promote malignancies associated with activation of RAS through mutation or other mechanisms [see Warnings and Precautions (5.2)]. Monitor patients receiving ZELBORAF closely for signs or symptoms of other malignancies.<br><br>**6 ADVERSE REACTIONS**<br>**6.2 Postmarketing Experience**<br>The following adverse reactions have been identified during post approval use of ZELBORAF. Because these reactions are reported voluntarily from a population of uncertain size, it is not always possible to reliably estimate their frequency or establish a causal relationship to drug exposure.<br>*Neoplasms benign, malignant and unspecified (incl. cysts and polyps)*<br>Progression of pre-existing chronic myelomonocytic leukemia with NRAS mutation [see Warnings and Precautions (5.1)]. (…)
**020699, 12/19/2017**	Venlafaxine	Psychiatry	CYP2D6	Drug Interactions, Use in Specific Populations,	**7 DRUG INTERACTIONS**<br>**7.5 Weight Loss Agents**<br>The safety and efficacy of venlafaxine therapy in combination with weight loss agents, including phentermine, have not been established. Coadministration of Effexor XR and weight loss agents is not recommended. Effexor XR is not indicated for weight loss alone or in combination with other products. (See Figure 1)

* Therapeutic areas do not necessarily reflect the CDER review division.
† Representative biomarkers are listed based on standard nomenclature as per the Human Genome Organization (HUGO) symbol and/or simplified descriptors using other common conventions. Listed biomarkers do not necessarily reflect the terminology used in labeling. The term "Nonspecific" is provided when labeling does not explicitly identify the specific biomarker(s) or when the biomarker is represented by a molecular phenotype or gene signature, and in some cases the biomarker was inferred based on the labeling language.
‡ Referenced figures and tables may be found in the labeling available at Drugs@FDA.
Blue text represents the most recent addtions and/or changes since last posted version.

NDA/ANDA/BLA Number, Label Version Date	Drug	Therapeutic Area*	Biomarker†	Labeling Sections	Labeling Text‡
				Clinical Pharmacology	**8 USE IN SPECIFIC POPULATIONS**<br>**8.6 Age and Gender**<br>A population pharmacokinetic analysis of 404 Effexor-treated patients from two studies involving both twice daily and three times daily regimens showed that dose-normalized trough plasma levels of either venlafaxine or ODV were unaltered by age or gender differences. Dosage adjustment based on the age or gender of a patient is generally not necessary [see Dosage and Administration (2.6)] (see Table 15). (See Figure 3)<br><br>**12 CLINICAL PHARMACOLOGY**<br>**12.3 Pharmacokinetics**<br>**Metabolism and elimination**<br>Following absorption, venlafaxine undergoes extensive presystemic metabolism in the liver, primarily to ODV, but also to N-desmethylvenlafaxine, N,O-didesmethylvenlafaxine, and other minor metabolites. In vitro studies indicate that the formation of ODV is catalyzed by CYP2D6; this has been confirmed in a clinical study showing that patients with low CYP2D6 levels (poor metabolizers) had increased levels of venlafaxine and reduced levels of ODV compared to people with normal CYP2D6 levels (extensive metabolizers) [see Use in Specific Populations 8.7].
**208573, 05/29/2020**	Venetoclax (1)	Oncology	Chromosome 17p	Clinical Studies	**14 CLINICAL STUDIES**<br>**14.1 Chronic Lymphocytic Leukemia/Small Lymphocytic Lymphoma**<br>Combination Therapy<br>CLL14<br>(…) A total of 432 patients were randomized, 216 to each study arm. Baseline demographic and disease characteristics were similar between the study arms. The median age was 72 years (range: 41 to 89 years), 89% were white, 67% were male; 36% and 43% were Binet stage B and C, respectively, and 88% had Eastern Cooperative Oncology Group (ECOG) performance status <2. The median CIRS score was 8.0 (range: 0 to 28) and 58% of patients had CLcr <70mL/min. A 17p deletion was detected in 8% of patients, TP53 mutations in 10%, 11q deletion in 19%, and unmutated IgVH in 57%. (…)<br>MURANO<br>(…) Prior therapies included alkylating agents (94%), anti-CD20 antibodies (77%), B-cell receptor pathway inhibitors (2%), and prior purine analogs (81%, including fludarabine/cyclophosphamide/rituximab in 55%). A 17p deletion was detected in 24% of patients, TP53 mutations in 25%, 11q deletion in 32%, and unmutated IgVH in 63%. (See Table 21) (…)<br>Monotherapy<br>The efficacy of VENCLEXTA monotherapy in previously-treated CLL or SLL is based on three single-arm studies.<br>Study M13-982<br>The efficacy of VENCLEXTA was established in study M13-982 (NCT01889186), an openlabel, single-arm, multicenter clinical trial of 106 patients with CLL with 17p deletion who had received at least one prior therapy. In the study, 17p deletion was confirmed in peripheral blood specimens from patients using Vysis CLL FISH Probe Kit, which is FDA approved for selection of patients for VENCLEXTA treatment. (See Table 23) (…)<br>Study M12-175<br>Study M12-175 (NCT01328626) was a multicenter, open-label trial that enrolled previously treated patients with CLL or SLL, including those with 17p deletion. Efficacy was evaluated in 67 patients (59 with CLL, 8 with SLL) who had received a 400 mg daily dose of VENCLEXTA. Patients continued this dose until disease progression or unacceptable toxicity. The median duration of treatment at the time of evaluation was 22.1 months (range: 0.5 to 50.1 months).<br>The median age was 66 years (range: 42 to 84 years), 78% were male and 87% were white. The median number of prior treatments was 3 (range: 1 to 11). At baseline, 67% of patients had one or more nodes ≥5 cm, 30% of patients had ALC ≥25 x 109 /L, 33% had documented unmutated IgVH, and 21% had documented 17p deletion. (…)<br>Study M14-032<br>Of the 127 patients treated (91 with prior ibrutinib, 36 with prior idelalisib), the median age was 66 years (range: 28 to 85 years), 70% were male and 92% were white. The median number of prior treatments was 4 (range: 1 to 15). At baseline, 41% of patients had one or more nodes ≥5 cm, 31% had an absolute lymphocyte count ≥25 x 109 /L, 57% had documented unmutated IgVH, and 39% had documented 17p deletion. (…)
**208573, 05/29/2020**	Venetoclax (2)	Oncology	Chromosome 11q	Clinical Studies	**14 CLINICAL STUDIES**<br>**14.1 Chronic Lymphocytic Leukemia/Small Lymphocytic Lymphoma**<br>Combination Therapy<br>CCL14<br>(…) Prior therapies included alkylating agents (94%), anti-CD20 antibodies (77%), B-cell receptor pathway inhibitors (2%), and prior purine analogs (81%, including fludarabine/cyclophosphamide/rituximab in 55%). A 17p deletion was detected in 24% of patients, TP53 mutations in 25%, 11q deletion in 32%, and unmutated IgVH in 63%. (…)<br>MURANO<br>(…) Prior therapies included alkylating agents (94%), anti-CD20 antibodies (77%), B-cell receptor pathway inhibitors (2%), and prior purine analogs (81%, including fludarabine/cyclophosphamide/rituximab in 55%). A 17p deletion was detected in 24% of patients, TP53 mutations in 25%, 11q deletion in 32%, and unmutated IgVH in 63%. (…)
**208573, 05/29/2020**	Venetoclax (3)	Oncology	TP53	Clinical Studies	**14 CLINICAL STUDIES**<br>**14.1 Chronic Lymphocytic Leukemia/Small Lymphocytic Lymphoma**<br>Combination Therapy<br>CCL14

NDA/ANDA/BLA Number, Label Version Date	Drug	Therapeutic Area*	Biomarker†	Labeling Sections	Labeling Text‡
					(…) The median CIRS score was 8.0 (range: 0 to 28) and 58% of patients had CLcr<70 mL/min. A 17p deletion was detected in 8% of patients, TP53 mutations in 7%, 11q deletion in 19%, and unmutated IgVH in 57%. (…)<br>MURANO<br>(…) Prior therapies included alkylating agents (94%), anti-CD20 antibodies (77%), B-cell receptor pathway inhibitors (2%), and prior purine analogs (81%, including fludarabine/cyclophosphamide/rituximab in 55%). A 17p deletion was detected in 24% of patients, TP53 mutations in 25%, 11q deletion in 32%, and unmutated IgVH in 63%. (See Table 17) (…)<br>**14.2 Acute Myeloid Leukemia**<br>Study M14-358<br>VENCLEXTA was studied in a non-randomized, open-label clinical trial (NCT02203773) of VENCLEXTA in combination with azacitidine (N=84) or decitabine (N=31) in patients with newly-diagnosed AML. Of those patients, 67 who received azacitidine combination and 13 who received decitabine combination were age 75 or older or had comorbidities that precluded the use of intensive induction chemotherapy. (See Table 24) (…)<br>Study M14-387<br>(…) Patients initiated VENCLEXTA via daily ramp-up to a final 600 mg once daily dose [see Dosage and Administration (2.1)]. During the ramp-up, patients received TLS prophylaxis and were hospitalized for monitoring. Cytarabine at a dose of 20 mg/m2 was administered subcutaneously once daily on Days 1-10 of each 28-day cycle beginning on Cycle 1 Day 1. Patients continued to receive treatment cycles until disease progression or unacceptable toxicity. Dose reduction for low-dose cytarabine was not implemented in the clinical trial. (See Table 26) (…)
**208573, 05/29/2020**	Venetoclax (4)	Oncology	IDH1	Clinical Studies	**14 CLINICAL STUDIES**<br>**14.2 Acute Myeloid Leukemia**<br>Study M14-358<br>VENCLEXTA was studied in a non-randomized, open-label clinical trial (NCT02203773) of VENCLEXTA in combination with azacitidine (N=84) or decitabine (N=31) in patients with newly-diagnosed AML. Of those patients, 67 who received azacitidine combination and 13 who received decitabine combination were age 75 or older or had comorbidities that precluded the use of intensive induction chemotherapy. (See Table 24) (…)<br>Study M14-387<br>(…) Patients initiated VENCLEXTA via daily ramp-up to a final 600 mg once daily dose [see Dosage and Administration (2.1)]. During the ramp-up, patients received TLS prophylaxis and were hospitalized for monitoring. Cytarabine at a dose of 20 mg/m2 was administered subcutaneously once daily on Days 1-10 of each 28-day cycle beginning on Cycle 1 Day 1. Patients continued to receive treatment cycles until disease progression or unacceptable toxicity. Dose reduction for low-dose cytarabine was not implemented in the clinical trial. (See Table 26) (…)
**208573, 05/29/2020**	Venetoclax (5)	Oncology	IDH2	Clinical Studies	**14 CLINICAL STUDIES**<br>**14.2 Acute Myeloid Leukemia**<br>Study M14-358<br>VENCLEXTA was studied in a non-randomized, open-label clinical trial (NCT02203773) of VENCLEXTA in combination with azacitidine (N=84) or decitabine (N=31) in patients with newly-diagnosed AML. Of those patients, 67 who received azacitidine combination and 13 who received decitabine combination were age 75 or older or had comorbidities that precluded the use of intensive induction chemotherapy. (See Table 24) (…)<br>Study M14-387<br>(…) Patients initiated VENCLEXTA via daily ramp-up to a final 600 mg once daily dose [see Dosage and Administration (2.1)]. During the ramp-up, patients received TLS prophylaxis and were hospitalized for monitoring. Cytarabine at a dose of 20 mg/m2 was administered subcutaneously once daily on Days 1-10 of each 28-day cycle beginning on Cycle 1 Day 1. Patients continued to receive treatment cycles until disease progression or unacceptable toxicity. Dose reduction for low-dose cytarabine was not implemented in the clinical trial. (See Table 26) (…)
**208573, 05/29/2020**	Venetoclax (6)	Oncology	IGHV	Clinical Studies	**14 CLINICAL STUDIES**<br>**14.1 Chronic Lymphocytic Leukemia/Small Lymphocytic Lymphoma**<br>Combination Therapy<br>CCL14<br>(…) The median CIRS score was 8.0 (range: 0 to 28) and 58% of patients had CLcr<70mL/min. A 17p deletion was detected in 8% of patients, TP53 mutations in 7%, 11q deletion in 19%, and unmutated IgVH in 57%. (…)<br>MURANO<br>(…) Prior therapies included alkylating agents (94%), anti-CD20 antibodies (77%), B-cell receptor pathway inhibitors (2%), and prior purine analogs (81%, including fludarabine/cyclophosphamide/rituximab in 55%). A 17p deletion was detected in 24% of patients, TP53 mutations in 25%, 11q deletion in 32%, and unmutated IgVH in 63%. (…)<br>Study M12-175<br>(…) The median age was 66 years (range: 42 to 84 years), 78% were male and 87% were white. The median number of prior treatments was 3 (range: 1 to 11). At baseline, 67% of patients had one or more nodes ≥5 cm, 30% of patients had ALC ≥25 x 109 /L, 33% had documented unmutated IgVH, and 21% had documented 17p deletion. (…)<br>Study M14-032<br>(…) Of the 127 patients treated (91 with prior ibrutinib, 36 with prior idelalisib), the median age was 66 years (range: 28 to 85 years), 70% were male and 92% were white. The median number of prior treatments was 4 (range: 1 to 15). At baseline, 41% of patients had one or more nodes ≥5 cm, 31% had an absolute lymphocyte count ≥25 x 109 /L, 57% had documented unmutated IgVH, and 39% had documented 17p deletion. (…)
**208573, 05/29/2020**	Venetoclax (7)	Oncology	NPM1	Clinical Studies	**14 CLINICAL STUDIES**<br>**14.2 Acute Myeloid Leukemia**<br>Study M14-358

* Therapeutic areas do not necessarily reflect the CDER review division.
† Representative biomarkers are listed based on standard nomenclature as per the Human Genome Organization (HUGO) symbol and/or simplified descriptors using other common conventions. Listed biomarkers do not necessarily reflect the terminology used in labeling. The term "Nonspecific" is provided when labeling does not explicitly identify the specific biomarker(s) or when the biomarker is represented by a molecular phenotype or gene signature, and in some cases the biomarker was inferred based on the labeling language.
‡ Referenced figures and tables may be found in the labeling available at Drugs@FDA.
Blue text represents the most recent addtions and/or changes since last posted version.

NDA/ANDA/BLA Number, Label Version Date	Drug	Therapeutic Area*	Biomarker†	Labeling Sections	Labeling Text‡
					VENCLEXTA was studied in a non-randomized, open-label clinical trial (NCT02203773) of VENCLEXTA in combination with azacitidine (N=84) or decitabine (N=31) in patients with newly-diagnosed AML. Of those patients, 67 who received azacitidine combination and 13 who received decitabine combination were age 75 or older or had comorbidities that precluded the use of intensive induction chemotherapy. (See Table 24) (…)<br>Study M14-387<br>(…) Patients initiated VENCLEXTA via daily ramp-up to a final 600 mg once daily dose [see Dosage and Administration (2.1)]. During the ramp-up, patients received TLS prophylaxis and were hospitalized for monitoring. Cytarabine at a dose of 20 mg/m2 was administered subcutaneously once daily on Days 1-10 of each 28-day cycle beginning on Cycle 1 Day 1. Patients continued to receive treatment cycles until disease progression or unacceptable toxicity. Dose reduction for low-dose cytarabine was not implemented in the clinical trial. (See Table 26) (…)
**208573, 05/29/2020**	Venetoclax (8)	Oncology	FLT3	Clinical Studies	**14 CLINICAL STUDIES**<br>**14.2 Acute Myeloid Leukemia**<br>Study M14-358<br>VENCLEXTA was studied in a non-randomized, open-label clinical trial (NCT02203773) of VENCLEXTA in combination with azacitidine (N=84) or decitabine (N=31) in patients with newly-diagnosed AML. Of those patients, 67 who received azacitidine combination and 13 who received decitabine combination were age 75 or older or had comorbidities that precluded the use of intensive induction chemotherapy. (See Table 24) (…)<br>Study M14-387<br>(…) Patients initiated VENCLEXTA via daily ramp-up to a final 600 mg once daily dose [see Dosage and Administration (2.1)]. During the ramp-up, patients received TLS prophylaxis and were hospitalized for monitoring. Cytarabine at a dose of 20 mg/m2 was administered subcutaneously once daily on Days 1-10 of each 28-day cycle beginning on Cycle 1 Day 1. Patients continued to receive treatment cycles until disease progression or unacceptable toxicity. Dose reduction for low-dose cytarabine was not implemented in the clinical trial. (See Table 26) (…)
**04/02/2021, 211964**	Viloxazine (1)	Psychiatry	CYP2D6	Clinical Pharmacology	**12 CLINICAL PHARMACOLOGY**<br>**12.3 Pharmacokinetics**<br>*CYP2D6 Metabolism*<br>A multiple-dose study was conducted with Qelbree 900 mg once-daily in healthy volunteers to compare the effect of CYP2D6 poor metabolizers (PMs) and extensive metabolizers (EMs) on the PK of viloxazine. At steady state, viloxazine geometric means for Cmax and AUC0-24 were 21% and 26%, respectively, higher in CYP2D6 PMs compared to EMs.
**04/02/2021, 211964**	Viloxazine (2)	Psychiatry	SLCO1B1	Clinical Pharmacology	**12 CLINICAL PHARMACOLOGY**<br>**12.3 Pharmacokinetics**<br>*In Vitro Studies*<br>Based on in vitro data, drugs that inhibit CYP isozymes 1A1, 1A2, 2B6, 2D6, 2C8, 2C9, 2C19, and 2E1 are not expected to have significant impact on the pharmacokinetic profile of viloxazine.<br>Viloxazine does not inhibit CYP2C8, 2C9 or 2C19 activities. Viloxazine is a reversible inhibitor of P450-1A2, 2B6, 2D6 and 3A4/5. Viloxazine is a potential inducer of CYP1A2 and CYP2B6. Viloxazine is not a inhibitor of P-gp, BCRP, MATE2-K, OATP1B1*1a, and OATP1B3 transporters. Viloxazine appears to be a weak inhibitor of the MATE1. Viloxazine is not a substrate of either OATP1B1*1a or OATP1B3 transporters.
**212154, 08/12/2020**	Viltolarsen	Neurology	DMD	Indications and Usage, Adverse Reactions, Use in Specific Populations, Clinical Pharmacology, Clinical Studies	**1 INDICATIONS AND USAGE**<br>VILTEPSO is indicated for the treatment of Duchenne muscular dystrophy (DMD) in patients who have a confirmed mutation of the DMD gene that is amenable to exon 53 skipping. This indication is approved under accelerated approval based on an increase in dystrophin production in skeletal muscle observed in patients treated with VILTEPSO [see Clinical Studies (14)]. Continued approval for this indication may be contingent upon verification and description of clinical benefit in a confirmatory trial.<br><br>**6 ADVERSE REACTIONS**<br>**6.1 Clinical Trials Experience**<br>In clinical trials with VILTEPSO, 32 patients have been exposed to VILTEPSO once weekly, ranging between 40 mg/kg (0.5 times the recommended dosage) and 80 mg/kg (the recommended dosage), including 16 patients treated for greater than 12 months and 8 patients treated for greater than 24 months as part of an ongoing open-label extension study. All patients were male and had genetically confirmed DMD. (…)<br><br>**8 USE IN SPECIFIC POPULATIONS**<br>**8.4 Pediatric Use**<br>VILTEPSO is indicated for the treatment of DMD in patients who have a confirmed mutation of the DMD gene that is amenable to exon 53 skipping, including pediatric patients [see Clinical Studies (14)].<br><br>**12 CLINICAL PHARMACOLOGY**<br>**12.3 Pharmacokinetics**<br>The pharmacokinetics of viltolarsen was evaluated in DMD patients following administration of intravenous (IV) doses ranging from 1.25 mg/kg/week (0.016 times the recommended dosage) to 80 mg/kg/week (the recommended dosage). Viltolarsen exposure increased proportionally with dose, with minimal accumulation with once-weekly dosing. Inter-subject variability (as %CV) for Cmax and AUC ranged from 16% to 27% respectively.<br>VILTEPSO is administered as an IV infusion over 60 minutes. Bioavailability is assumed to be 100%, and median Tmax was around 1 hour (end of infusion).<br>Specific Populations<br>*Age, Sex & Race*

NDA/ANDA/BLA Number, Label Version Date	Drug	Therapeutic Area*	Biomarker†	Labeling Sections	Labeling Text‡
					The pharmacokinetics of viltolarsen have been evaluated only in male pediatric DMD patients.There is no experience with VILTEPSO in patients 65 years of age or older. No marked differences in any PK parameters were observed between White and Asian patients.<br>*Patients with Renal or Hepatic Impairment*<br>VILTEPSO has not been studied in patients with renal or hepatic impairment. Viltolarsen was found to be metabolically stable, and hepatic metabolism does not contribute to the elimination of viltolarsen. In addition, viltolarsen was mainly excreted unchanged in the urine. Viltolarsen is eliminated renally, and renal impairment is expected to result in increasing exposure of viltolarsen. However, because of the effect of reduced skeletal muscle mass on creatinine measurements in DMD patients, no specific dosage adjustment can be recommended for DMD patients with renal impairment based on glomerular filtration rate estimated by serum creatinine [see Use in Specific Populations (8.6)].<br><br>**14 CLINICAL STUDIES**<br>The effect of VILTEPSO on dystrophin production was evaluated in one study in DMD patients with a confirmed mutation of the DMD gene that is amenable to exon 53 skipping (Study 1; NCT02740972). (…)
**202497, 03/15/2022**	**Vincristine**	Oncology	BCR-ABL1 (Philadelphia chromosome)	Indications and Usage, Adverse Reactions, Clinical Studies	**1 INDICATIONS AND USAGE**<br>**1.1 Adult ALL in Second or Greater Relapse**<br>Marqibo® is indicated for the treatment of adult patients with Philadelphia chromosome-negative (Ph-) acute lymphoblastic leukemia (ALL) in second or greater relapse or whose disease has progressed following two or more anti-leukemia therapies. This indication is based on overall response rate. Clinical benefit such as improvement in overall survival has not been verified.<br><br>**6 ADVERSE REACTIONS**<br>Acute Lymphoblastic Leukemia Relapsed or Refractory Philadelphia Chromosome Negative ALL<br>The safety of Marqibo was evaluated in a total of 83 adults in two trials: study 1 and study 2. Patients received Marqibo 2.25 mg/m2 once every seven days. Adverse reactions were observed in 100% of patients. The most common adverse reactions (>30%) were constipation (57%), nausea (52%), pyrexia (43%), fatigue (41%), peripheral neuropathy (39%), febrile neutropenia (38%), diarrhea (37%), anemia (34%), decreased appetite (33%), and insomnia (32%). (…)<br><br>**14 CLINICAL STUDIES**<br>**14.1 Acute Lymphoblastic Leukemia**<br>Marqibo was studied in an international, open-label, multi-center, single-arm trial (Study 1). Eligible patients were 18 years of age or older with Philadelphia chromosome negative ALL in second or greater relapse or whose disease progressed after two or greater treatment lines of anti-leukemia therapy. Patients had to have achieved a complete remission (CR) to at least one prior anti-leukemia chemotherapy, defined by a leukemia-free interval of equal or more than 90 days. Patients were not eligible for immediate hematopoietic stem cell transplantation (HSCT) at the time of screening and enrollment. (…)
**021266, 04/30/2019**	**Voriconazole**	Infectious Diseases	CYP2C19	Clinical Pharmacology	**12 CLINICAL PHARMACOLOGY**<br>**12.3 Pharmacokinetics**<br>*Metabolism*<br>In vitro studies showed that voriconazole is metabolized by the human hepatic cytochrome P450 enzymes, CYP2C19, CYP2C9 and CYP3A4 [see Drug Interactions (7)].<br>In vivo studies indicated that CYP2C19 is significantly involved in the metabolism of voriconazole. This enzyme exhibits genetic polymorphism. For example, 15-20% of Asian populations may be expected to be poor metabolizers. For Caucasians and Blacks, the prevalence of poor metabolizers is 3-5%. Studies conducted in Caucasian and Japanese healthy subjects have shown that poor metabolizers have, on average, 4-fold higher voriconazole exposure (AUC_τ) than their homozygous extensive metabolizer counterparts. Subjects who are heterozygous extensive metabolizers have, on average, 2-fold higher voriconazole exposure than their homozygous extensive metabolizer counterparts. (…)<br>**12.5 Pharmacogenomics**<br>CYP2C19, significantly involved in the metabolism of voriconazole, exhibits genetic polymorphism. Approximately 15-20% of Asian populations may be expected to be poor metabolizers. For Caucasians and Blacks, the prevalence of poor metabolizers is 3-5%. Studies conducted in Caucasian and Japanese healthy subjects have shown that poor metabolizers have, on average, 4-fold higher voriconazole exposure (AUC_τ) than their homozygous extensive metabolizer counterparts. Subjects who are heterozygous extensive metabolizers have, on average, 2-fold higher voriconazole exposure than their homozygous extensive metabolizer counterparts [see Clinical Pharmacology (12.3)].
**204447, 10/19/2018**	**Vortioxetine**	Psychiatry	CYP2D6	Dosage and Administration, Clinical Pharmacology	**2 DOSAGE AND ADMINISTRATION**<br>**2.6 Use of TRINTELLIX in Known CYP2D6 Poor Metabolizers or in Patients Taking Strong CYP2D6 Inhibitors**<br>The maximum recommended dose of TRINTELLIX is 10 mg/day in known CYP2D6 poor metabolizers. Reduce the dose of TRINTELLIX by one-half when patients are receiving a CYP2D6 strong inhibitor (e.g., bupropion, fluoxetine, paroxetine, or quinidine) concomitantly. The dose should be increased to the original level when the CYP2D6 inhibitor is discontinued [see Drug Interactions (7.3)].<br><br>**12 CLINICAL PHARMACOLOGY**<br>**12.3 Pharmacokinetics**<br>*Metabolism and Elimination*<br>Vortioxetine is extensively metabolized primarily through oxidation via cytochrome P450 isozymes CYP2D6, CYP3A4/5, CYP2C19, CYP2C9, CYP2A6, CYP2C8 and CYP2B6 and subsequent glucuronic acid conjugation. CYP2D6 is the primary enzyme catalyzing the metabolism of vortioxetine to its major, pharmacologically inactive, carboxylic acid metabolite, and poor metabolizers of CYP2D6 have approximately twice the vortioxetine plasma concentration of extensive metabolizers. (…)

* Therapeutic areas do not necessarily reflect the CDER review division.
† Representative biomarkers are listed based on standard nomenclature as per the Human Genome Organization (HUGO) symbol and/or simplified descriptors using other common conventions. Listed biomarkers do not necessarily reflect the terminology used in labeling. The term “Nonspecific” is provided when labeling does not explicitly identify the specific biomarker(s) or when the biomarker is represented by a molecular phenotype or gene signature, and in some cases the biomarker was inferred based on the labeling language.
‡ Referenced figures and tables may be found in the labeling available at Drugs@FDA.
Blue text represents the most recent addtions and/or changes since last posted version.

NDA/ANDA/BLA Number, Label Version Date	Drug	Therapeutic Area*	Biomarker†	Labeling Sections	Labeling Text‡
**213137, 11/25/2019**	Voxelotor	Hematology	HBB	Clinical Pharmacology, Clinical Studies	**12 CLINICAL PHARMACOLOGY**<br>**12.3 Pharmacokinetics**<br>*Patients with HbSC Genotype*<br>Voxelotor steady state whole blood AUC and Cmax were 50% and 45% higher in HbSC genotype patients (n=11) compared to HbSS genotype (n=220) patients and voxelotor steady state plasma AUC and Cmax were 23% and 15% higher in HbSC genotype patients compared to HbSS genotype patients.<br><br>**14 CLINICAL STUDIES**<br>(…) The majority of patients had HbSS or HbS/beta0 -thalassemia genotype (90%) and were receiving background hydroxyurea therapy (65%). The median age was 24 years (range: 12 to 64 years); 46 (17%) patients were 12 to < 17 years of age. Median baseline Hb was 8.5 g/dL (5.9 to 10.8 g/dL). One hundred and fifteen (42%) had 1 VOC event and 159 (58%) had 2 to 10 events within 12 months prior to enrollment. (…)
**215515, 06/13/2022**	Vutrisiran	Neurology	TTR	Adverse Reactions, Clinical Pharmacology, Clinical Studies	**6 ADVERSE REACTIONS**<br>**6.1 Clinical Trials Experience**<br>In Study 1 [see Clinical Studies (14)], a total of 122 patients with polyneuropathy caused by hereditary transthyretin-mediated amyloidosis (hATTR amyloidosis) received AMVUTTRA. Of these, 118 patients received at least 9 months of treatment and 34 patients received at least 15 months of treatment. The mean duration of treatment was 12.9 months (range: 1.7 to 19.3 months). The median patient age at baseline was 60 years and 65% of the patients were male. Seventy percent of AMVUTTRA-treated patients were Caucasian, 17% were Asian, 3% were Black, and 9% were reported as Other. Forty-four percent of patients had the Val30Met mutation in the transthyretin gene; the remaining patients had one of 21 other mutations. (…)<br><br>**12 CLINICAL PHARMACOLOGY**<br>**12.2 Pharmacodynamics**<br>In Study 1 [see Clinical Studies (14)], following administration of the recommended AMVUTTRA dosage every 3 months to patients with hATTR amyloidosis, vutrisiran reduced mean serum TTR at steady state by 83%. Similar TTR reductions were observed regardless of Val30Met genotype status, weight, sex, age, or race. Vutrisiran also reduced the mean steady state serum vitamin A by 62% over 9 months [see Warnings and Precautions (5.1)].<br><br>**14 CLINICAL STUDIES**<br>(…) Patients receiving AMVUTTRA in Study 1 experienced similar improvements relative to those in the external placebo group in mNIS+7 and Norfolk QoL-DN total score across all subgroups including age, sex, race, region, NIS score, Val30Met genotype status, and disease stage.
**009218, 08/14/2017**	Warfarin (1)	Hematology	CYP2C9	Dosage and Administration, Drug Interactions, Clinical Pharmacology	**2 DOSAGE AND ADMINISTRATION**<br>**2.3 Initial and Maintenance Dosing**<br>The appropriate initial dosing of COUMADIN varies widely for different patients. Not all factors responsible for warfarin dose variability are known, and the initial dose is influenced by:<br>• Clinical factors including age, race, body weight, sex, concomitant medications, and comorbidities<br>• Genetic factors (CYP2C9 and VKORC1 genotypes) [see Clinical Pharmacology (12.5)] (…)<br>*Dosing Recommendations without Consideration of Genotype*<br>If the patient's CYP2C9 and VKORC1 genotypes are not known, the initial dose of COUMADIN is usually 2 to 5 mg once daily. Determine each patient's dosing needs by close monitoring of the INR response and consideration of the indication being treated. Typical maintenance doses are 2 to 10 mg once daily.<br>*Dosing Recommendations with Consideration of Genotype*<br>Table 1 displays three ranges of expected maintenance COUMADIN doses observed in subgroups of patients having different combinations of CYP2C9 and VKORC1 gene variants [see Clinical Pharmacology (12.5)]. If the patient's CYP2C9 and/or VKORC1 genotype are known, consider these ranges in choosing the initial dose. Patients with CYP2C9 *1/*3, *2/*2, *2/*3, and *3/*3 may require more prolonged time (>2 to 4 weeks) to achieve maximum INR effect for a given dosage regimen than patients without these CYP variants. (See Table 1)<br><br>**12 CLINICAL PHARMACOLOGY**<br>**12.3 Pharmacokinetics**<br>*Metabolism*<br>The elimination of warfarin is almost entirely by metabolism. Warfarin is stereoselectively metabolized by hepatic cytochrome P-450 (CYP450) microsomal enzymes to inactive hydroxylated metabolites (predominant route) and by reductases to reduced metabolites (warfarin alcohols) with minimal anticoagulant activity. Identified metabolites of warfarin include dehydrowarfarin, two diastereoisomer alcohols, and 4'-, 6-, 7-, 8-, and 10- hydroxywarfarin. The CYP450 isozymes involved in the metabolism of warfarin include CYP2C9, 2C19, 2C8, 2C18, 1A2, and 3A4. CYP2C9, a polymorphic enzyme, is likely to be the principal form of human liver CYP450 that modulates the in vivo anticoagulant activity of warfarin. Patients with one or more variant CYP2C9 alleles have decreased S-warfarin clearance [see Clinical Pharmacology (12.5)].<br>**12.5 Pharmacogenomics**<br>CYP2C9 and VKORC1 Polymorphisms The S-enantiomer of warfarin is mainly metabolized to 7-hydroxywarfarin by CYP2C9, a polymorphic enzyme. The variant alleles, CYP2C9*2 and CYP2C9*3, result in decreased in vitro CYP2C9 enzymatic 7-hydroxylation of S-warfarin. The frequencies of these alleles in Caucasians are approximately 11% and 7% for CYP2C9*2 and CYP2C9*3, respectively. Other CYP2C9 alleles associated with reduced enzymatic activity occur at lower frequencies, including *5, *6, and *11 alleles in populations of African ancestry and *5, *9, and *11 alleles in Caucasians.<br>Warfarin reduces the regeneration of vitamin K from vitamin K epoxide in the vitamin K cycle through inhibition of VKOR, a multiprotein enzyme complex. Certain single nucleotide polymorphisms in the VKORC1 gene (e.g., –1639G>A) have been associated with variable warfarin dose requirements. VKORC1 and CYP2C9 gene variants generally explain the largest proportion of known variability in warfarin dose requirements.<br>CYP2C9 and VKORC1 genotype information, when available, can assist in selection of the initial dose of warfarin [see Dosage and Administration (2.3)].

* Therapeutic areas do not necessarily reflect the CDER review division.
† Representative biomarkers are listed based on standard nomenclature as per the Human Genome Organization (HUGO) symbol and/or simplified descriptors using other common conventions. Listed biomarkers do not necessarily reflect the terminology used in labeling. The term "Nonspecific" is provided when labeling does not explicitly identify the specific biomarker(s) or when the biomarker is represented by a molecular phenotype or gene signature, and in some cases the biomarker was inferred based on the labeling language.
‡ Referenced figures and tables may be found in the labeling available at Drugs@FDA.
Blue text represents the most recent addtions and/or changes since last posted version.

Table of Pharmacogenomic Biomarkers in Drug Labeling
Last Updated: 06/2024

NDA/ANDA/BLA Number, Label Version Date	Drug	Therapeutic Area*	Biomarker†	Labeling Sections	Labeling Text‡
**009218, 08/14/2017**	Warfarin (2)	Hematology	VKORC1	Dosage and Administration, Clinical Pharmacology	**2 DOSAGE AND ADMINISTRATION**<br>**2.3 Initial and Maintenance Dosing**<br>The appropriate initial dosing of COUMADIN varies widely for different patients. Not all factors responsible for warfarin dose variability are known, and the initial dose is influenced by:<br>• Clinical factors including age, race, body weight, sex, concomitant medications, and comorbidities<br>• Genetic factors (CYP2C9 and VKORC1 genotypes) [see Clinical Pharmacology (12.5)] (…)<br>*Dosing Recommendations without Consideration of Genotype*<br>If the patient's CYP2C9 and VKORC1 genotypes are not known, the initial dose of COUMADIN is usually 2 to 5 mg once daily. Determine each patient's dosing needs by close monitoring of the INR response and consideration of the indication being treated. Typical maintenance doses are 2 to 10 mg once daily.<br>*Dosing Recommendations with Consideration of Genotype*<br>Table 1 displays three ranges of expected maintenance COUMADIN doses observed in subgroups of patients having different combinations of CYP2C9 and VKORC1 gene variants [see Clinical Pharmacology (12.5)]. If the patient's CYP2C9 and/or VKORC1 genotype are known, consider these ranges in choosing the initial dose. Patients with CYP2C9 *1/*3, *2/*2, *2/*3, and *3/*3 may require more prolonged time (>2 to 4 weeks) to achieve maximum INR effect for a given dosage regimen than patients without these CYP variants. (See Table 1)<br><br>**12 CLINICAL PHARMACOLOGY**<br>**12.5 Pharmacogenomics**<br>CYP2C9 and VKORC1 Polymorphisms The S-enantiomer of warfarin is mainly metabolized to 7-hydroxywarfarin by CYP2C9, a polymorphic enzyme. The variant alleles, CYP2C9*2 and CYP2C9*3, result in decreased in vitro CYP2C9 enzymatic 7-hydroxylation of S-warfarin. The frequencies of these alleles in Caucasians are approximately 11% and 7% for CYP2C9*2 and CYP2C9*3, respectively. Other CYP2C9 alleles associated with reduced enzymatic activity occur at lower frequencies, including *5, *6, and *11 alleles in populations of African ancestry and *5, *9, and *11 alleles in Caucasians.<br>Warfarin reduces the regeneration of vitamin K from vitamin K epoxide in the vitamin K cycle through inhibition of VKOR, a multiprotein enzyme complex. Certain single nucleotide polymorphisms in the VKORC1 gene (e.g., –1639G>A) have been associated with variable warfarin dose requirements. VKORC1 and CYP2C9 gene variants generally explain the largest proportion of known variability in warfarin dose requirements.<br>CYP2C9 and VKORC1 genotype information, when available, can assist in selection of the initial dose of warfarin [see Dosage and Administration (2.3)].
**009218, 08/14/2017**	Warfarin (3)	Hematology	PROS1	Warnings and Precautions	**5 WARNINGS AND PRECAUTIONS**<br>**5.8 Other Clinical Settings with Increased Risks**<br>In the following clinical settings, the risks of COUMADIN therapy may be increased:<br>(…) Deficiency in protein C-mediated anticoagulant response: COUMADIN reduces the synthesis of the naturally occurring anticoagulants, protein C and protein S. Hereditary or acquired deficiencies of protein C or its cofactor, protein S, have been associated with tissue necrosis following warfarin administration. Concomitant anticoagulation therapy with heparin for 5 to 7 days during initiation of therapy with COUMADIN may minimize the incidence of tissue necrosis in these patients. (…)<br><br>**12 CLINICAL PHARMACOLOGY**<br>**12.2 Pharmacodynamics**<br>An anticoagulation effect generally occurs within 24 hours after warfarin administration. However, peak anticoagulant effect may be delayed 72 to 96 hours. The duration of action of a single dose of racemic warfarin is 2 to 5 days. The effects of COUMADIN may become more pronounced as effects of daily maintenance doses overlap. This is consistent with the half-lives of the affected vitamin K-dependent clotting factors and anticoagulation proteins: Factor II - 60 hours, VII - 4 to 6 hours, IX - 24 hours, X - 48 to 72 hours, and proteins C and S are approximately 8 hours and 30 hours, respectively.
**009218, 08/14/2017**	Warfarin (4)	Hematology	PROC	Warnings and Precautions	**5 WARNINGS AND PRECAUTIONS**<br>**5.8 Other Clinical Settings with Increased Risks**<br>In the following clinical settings, the risks of COUMADIN therapy may be increased:<br>(…) Deficiency in protein C-mediated anticoagulant response: COUMADIN reduces the synthesis of the naturally occurring anticoagulants, protein C and protein S. Hereditary or acquired deficiencies of protein C or its cofactor, protein S, have been associated with tissue necrosis following warfarin administration. Concomitant anticoagulation therapy with heparin for 5 to 7 days during initiation of therapy with COUMADIN may minimize the incidence of tissue necrosis in these patients. (…)<br><br>**12 CLINICAL PHARMACOLOGY**<br>**12.2 Pharmacodynamics**<br>An anticoagulation effect generally occurs within 24 hours after warfarin administration. However, peak anticoagulant effect may be delayed 72 to 96 hours. The duration of action of a single dose of racemic warfarin is 2 to 5 days. The effects of COUMADIN may become more pronounced as effects of daily maintenance doses overlap. This is consistent with the half-lives of the affected vitamin K-dependent clotting factors and anticoagulation proteins: Factor II - 60 hours, VII - 4 to 6 hours, IX - 24 hours, X - 48 to 72 hours, and proteins C and S are approximately 8 hours and 30 hours, respectively.
**213217, 04/21/2023**	Zanubrutinib (1)	Oncology	MYD88	Adverse Reactions, Clinical Studies	**6 ADVERSE REACTIONS**<br>Waldenström's Macroglobulinemia (WM)<br>The safety of BRUKINSA was investigated in two cohorts of Study BGB-3111-302 (ASPEN). Cohort 1 included 199 patients with MYD88 mutation (MYD88MUT) WM, randomized to and treated with either BRUKINSA (101 patients) or ibrutinib (98 patients). The trial also included a non-randomized arm, Cohort 2, with 26 wild type MYD88 (MYD88WT) WM patients and 2 patients with unknown MYD88 status [see Clinical Studies (14.2)]. (…)

* Therapeutic areas do not necessarily reflect the CDER review division.
† Representative biomarkers are listed based on standard nomenclature as per the Human Genome Organization (HUGO) symbol and/or simplified descriptors using other common conventions. Listed biomarkers do not necessarily reflect the terminology used in labeling. The term "Nonspecific" is provided when labeling does not explicitly identify the specific biomarker(s) or when the biomarker is represented by a molecular phenotype or gene signature, and in some cases the biomarker was inferred based on the labeling language.
‡ Referenced figures and tables may be found in the labeling available at Drugs@FDA.
Blue text represents the most recent addtions and/or changes since last posted version.

NDA/ANDA/BLA Number, Label Version Date	Drug	Therapeutic Area*	Biomarker†	Labeling Sections	Labeling Text‡
					**14 CLINICAL STUDIES**<br>**14.2 Waldenström's Macroglobulinemia**<br>The efficacy of BRUKINSA was evaluated in ASPEN [NCT03053440], a randomized, active control, open-label trial, comparing BRUKINSA and ibrutinib in patients with MYD88 L265P mutation (MYD88MUT) WM. Patients in Cohort 1 (n=201) were randomized 1:1 to receive BRUKINSA 160 mg twice daily or ibrutinib 420 mg once daily until disease progression or unacceptable toxicity. Randomization was stratified by number of prior therapies (0 versus 1-3 versus > 3) and CXCR4 status (presence or absence of a WHIM-like mutation as measured by Sanger assay).<br>ASPEN Cohort 2<br>Cohort 2 enrolled patients with MYD88 wildtype (MYD88WT) or MYD88 mutation unknown WM (N = 26 and 2, respectively) and received BRUKINSA 160 mg twice daily. The median age was 72 years (range: 39 to 87) with 43% > 75 years, 50% were male, 96% were White and 4% were not reported (unknown race). 86% patients had a baseline ECOG performance status 0 or 1 and 14% had a baseline performance status of 2. Twenty-three of the 28 patients in Cohort 2 had relapsed or refractory disease. In Cohort 2, response (CR+VGPR+PR) as assessed by IRC using IWWM-6 or modified IWWM-6 was seen in 50% (13 out of 26 response evaluable patients; 95% CI: 29.9, 70.1).
213217, 04/21/2023	Zanubrutinib (2)	Oncology	Chromosome 17p	Adverse Reactions, Clinical Studies	**6 ADVERSE REACTIONS**<br>*Single-arm cohort: Previously untreated CLL/SLL and 17p deletion*<br>In 111 patients with previously untreated, 17p del CLL/SLL, the median age was 70, 71% were male, 95% were White, and 1% were Asian. Most patients (87%) had an ECOG performance status of 0 to 1. The median duration of exposure to BRUKINSA was 30 months. (See Tables 11 and 12)<br><br>**14 CLINICAL STUDIES**<br>**14.4 Chronic Lymphocytic Leukemia or Small Lymphocytic Lymphoma**<br>The efficacy of BRUKINSA in patients with CLL/SLL was evaluated in two randomized controlled trials.<br>SEQUOIA<br>(…) Additionally, the same BRUKINSA regimen was evaluated in 110 patients with previously untreated, 17p del CLL/SLL in a non-randomized cohort. Efficacy is summarized according to cohort.<br>*Randomized cohort: Previously untreated CLL/SLL without 17p deletion*<br>In the randomized cohort of patients with previously untreated CLL/SLL without 17p deletion, the median age was 70 years; 62% were male, 89% were White, 3% were Asian, 1% were Black. Fifty-three percent of patients had an unmutated IGHV gene and 29% had Binet Stage C disease. Baseline characteristics were generally similar between treatment arms.<br>Efficacy in this cohort was based on progression-free survival as assessed by an IRC. Efficacy results are presented in Table 20 and Figure 1.<br>At the time of analysis, overall survival data were immature. With an estimated median followup of 25.7 months, median overall survival was not reached in either arm, with fewer than 7% of patients experiencing an event.<br>*Single-arm cohort: Previously untreated CLL/SLL with 17p deletion*<br>In this cohort, 110 patients with previously untreated CLL/SLL and centrally confirmed 17p deletion received BRUKINSA 160 mg twice daily until disease progression or unacceptable toxicity. The median age was 70, 71% were male, 95% were White, and 1% were Asian. Sixty percent of patients had an unmutated IGHV gene and 35% had Binet Stage C disease. Efficacy was based on overall response rate and duration of response as assessed by an IRC. Efficacy results are presented in Table 21.<br>ALPINE<br>The efficacy of BRUKINSA in patients with relapsed or refractory CLL/SLL was evaluated in ALPINE, a randomized, multicenter, open-label, actively controlled trial (NCT03734016). The trial enrolled 652 patients with relapsed or refractory CLL/SLL after at least 1 systemic therapy. The patients were randomized in a 1:1 ratio to receive either BRUKINSA 160 mg orally twice daily (n=327) or ibrutinib 420 mg orally once daily (n=325), each administered until disease progression or unacceptable toxicity.<br>Randomization was stratified by age, geographic region, refractoriness to last therapy, and 17p deletion/TP53 mutation status.<br>Baseline characteristics were similar between treatment arms. Overall, the median age was 67 years, 68% were male, 81% were White, 14% were Asian, 1% were Black. Forty-three percent had advanced stage disease, 73% had an unmutated IGHV gene, and 23% had 17p deletion or TP53 mutation. Patients had a median of one prior line of therapy (range: 1-8), 18% of patients had ≥3 prior lines of therapy, 78% had prior chemoimmunotherapy, and 2.3% had prior BCL2 inhibitor.<br>Efficacy was based on overall response rate and duration of response as determined by an IRC. Efficacy results are shown in Table 22.<br>At the time of analysis, overall survival data were immature. With an estimated median followup of 24.7 months, median overall survival was not reached in either arm with 11% of patients experiencing an event.
213217, 04/21/2023	Zanubrutinib (3)	Oncology	TP53	Clinical Studies	**14 CLINICAL STUDIES**<br>**14.4 Chronic Lymphocytic Leukemia or Small Lymphocytic Lymphoma**<br>The efficacy of BRUKINSA in patients with CLL/SLL was evaluated in two randomized controlled trials.<br>ALPINE<br>The efficacy of BRUKINSA in patients with relapsed or refractory CLL/SLL was evaluated in ALPINE, a randomized, multicenter, open-label, actively controlled trial (NCT03734016). The trial enrolled 652 patients with relapsed or refractory CLL/SLL after at least 1 systemic therapy. The patients were randomized in a 1:1 ratio to receive either BRUKINSA 160 mg orally twice daily (n=327) or ibrutinib 420 mg orally once daily (n=325), each administered until disease progression or unacceptable toxicity.<br>Randomization was stratified by age, geographic region, refractoriness to last therapy, and 17p deletion/TP53 mutation status. Baseline characteristics were similar between treatment arms. Overall, the median age was 67 years, 68% were male, 81% were White, 14% were Asian, 1% were Black. Forty-three percent had advanced stage disease, 73% had an unmutated IGHV gene, and 23% had 17p deletion or TP53 mutation. Patients had a median of one prior line of

NDA/ANDA/BLA Number, Label Version Date	Drug	Therapeutic Area*	Biomarker†	Labeling Sections	Labeling Text‡
					therapy (range: 1-8), 18% of patients had ≥3 prior lines of therapy, 78% had prior chemoimmunotherapy, and 2.3% had prior BCL2 inhibitor. Efficacy was based on overall response rate and duration of response as determined by an IRC. Efficacy results are shown in Table 22.<br>At the time of analysis, overall survival data were immature. With an estimated median followup of 24.7 months, median overall survival was not reached in either arm with 11% of patients experiencing an event.
**216834, 10/17/2023**	Zilucoplan	Neurology	ACHR	Indications and Usage, Clinical Studies	**1 INDICATIONS AND USAGE**<br>ZILBRYSQ is indicated for the treatment of generalized myasthenia gravis (gMG) in adult patients who are anti-acetylcholine receptor (AChR) antibody positive.<br><br>**14 CLINICAL STUDIES**<br>The efficacy of ZILBRYSQ for the treatment of generalized myasthenia gravis (gMG) in adult patients who are anti-AChR antibody positive was established in a 12-week, multicenter, randomized, double-blind placebo-controlled study (Study 1; NCT04115293).<br>Study 1 enrolled patients who met the following criteria at screening:<br>• Myasthenia Gravis Foundation of America (MGFA) clinical classification class II to IV,<br>• Positive serology for AChR binding autoantibodies,<br>• MG-Activities of Daily Living (MG-ADL) total score of ≥6,<br>• Those on MG therapy prior to screening (including acetylcholinesterase (AChE) inhibitors, steroids, or non-steroidal immunosuppressive therapies (NSISTs), either in combination or alone), needed to maintain a stable dose. (…)

* Therapeutic areas do not necessarily reflect the CDER review division.
† Representative biomarkers are listed based on standard nomenclature as per the Human Genome Organization (HUGO) symbol and/or simplified descriptors using other common conventions. Listed biomarkers do not necessarily reflect the terminology used in labeling. The term “Nonspecific” is provided when labeling does not explicitly identify the specific biomarker(s) or when the biomarker is represented by a molecular phenotype or gene signature, and in some cases the biomarker was inferred based on the labeling language.
‡ Referenced figures and tables may be found in the labeling available at Drugs@FDA.
Blue text represents the most recent addtions and/or changes since last posted version.
