# Supplementary material for: Opportunities amid complexities in returning genetic results to black precision medicine research participants: Interview themes in context with open all of us data
Source: J Clin Transl Sci. 2025 Apr 11;9(1):e89. doi: 10.1017/cts.2025.67 (PMC12089846; doi:10.1017/cts.2025.67)
Supplement: Hendricks-Sturrup et al. supplementary material 2 — Hendricks-Sturrup et al. supplementary material [file S2059866125000676sup002.pdf]

				ClinVar Significance (as of July 2024)					
Gene	Condition associated with this gene	What it is	Total # of Variants	Risk Factor, Likely Pathogenic, or Pathogenic (%)	Drug Response (%)	Undefined/Uncertain (%)	Variants with Risk Factor, Likely Pathogenic, or Pathogenic Significance & Allele Frequency ≥ 0.10 in African Ancestry Population (Variant Type; Allele Count; Allele Frequency)	Variants with Drug Response Significance & Allele Frequency ≥ 0.10 in African Ancestry Population (Variant Type; Allele Count; Allele Frequency)	FDA PGx Drug Labeling Section (Therapeutic Area; N/A: total % alleles with ClinVar drug response significance is 0; None: total % alleles with ClinVar drug response significance is > 0)
ACTA2	familial thoracic aortic aneurysm and aortic dissection	a blood vessel disorder	22,646	10 (<0.01)	0 (0)	22530 (99.5)	None	None	N/A
ACTC1	hypertrophic cardiomyopathy	a heart disorder	7,139	6 (<0.01)	0 (0)	6979 (97.8)	None	None	N/A
APC	familial adenomatous polyposis	some types of cancers	51303	60 (0.12)	0 (0)	50,115 (97.7)	None	None	Nirogacestat (oncology)
APOB	familial hypercholesterolemia	dangerously high cholesterol	20,132	56 (0.28)	5 (<0.01)	19,507 (96.9)	None	None	None
ATP7B	Wilson disease	a disorder called Wilson disease	35,249	223 (0.63)	0 (0)	34,676 (98.4)	None	None	N/A
BMPR1A	juvenile polyposis syndrome	some types of cancers	75,722	5 (<0.01)	0 (0)	75,408 (99.6)	None	None	N/A
BRCA1	hereditary breast and ovarian cancer syndrome	some types of cancers	52437	219 (0.42)	0 (0)	50,986 (97.2)	None	None	Niraparib, Olaparib, Rucaparib, Sacituzumab Govitecan-hziy, Talazoparib (oncology)
BRCA2	hereditary breast and ovarian cancer syndrome	some types of cancers	34873	372 (1.1)	0 (0)	33,091 (94.9)	None	None	Niraparib, Olaparib, Rucaparib, Sacituzumab Govitecan-hziy, Talazoparib (oncology)
CACNA1S	malignant hyperthermia susceptibility	a disorder called malignant hyperthermia	33,757	36 (0.11)	2 (<0.01)	33,117 (98.1)	1-201112815-C-T (SNV; 73,407; 0.680362)<br>1-201110107-C-T (SNV; 30,531; 0.282972)	None	Desflurane, Isoflurane, Sevoflurane, Succinylcholine (Anesthesiology)
COL3A1	vascular Ehlers-Danlos syndrome (EDS)	a disorder called Ehlers-Danlos syndrome (EDS)	18,232	32 (0.18)	0 (0)	17,682 (97.0)	None	None	N/A
DSC2	arrhythmogenic cardiomyopathy	a heart disorder	17,214	25 (0.15)	0 (0)	16,931 (98.4)	None	None	N/A
DSG2	arrhythmogenic cardiomyopathy	a heart disorder	24,965	53 (0.21)	0 (0)	24,669 (98.8)	None	None	N/A
DSP	arrhythmogenic cardiomyopathy and dilated cardiomyopathy	a heart disorder	21,038	59 (0.28)	0 (0)	20,323 (96.6)	None	None	N/A
FBN1	Marfan syndrome	a blood vessel disorder	90,315	78 (<0.01)	0 (0)	89,456 (99.0)	None	None	N/A
GLA	Fabry disease	a disorder called Fabry disease	5,613	26 (0.46)	12 (0.21)	5,506 (98.1)	None	None	Migalastat (Inborn Errors of Metabolism)
KCNH2	long QT syndrome	a heart disorder	17,863	57 (0.32)	0 (0)	17,375 (97.3)	None	None	N/A
KCNQ1	long QT syndrome	a heart disorder	182,895	119 (<0.01)	0 (0)	182,437 (99.7)	None	None	N/A
LDLR	familial hypercholesterolemia	dangerously high cholesterol	22,739	282 (1.2)	0 (0)	22,197 (97.6)	None	None	Evinacumab-dgnb (endocrinology)
LMNA	dilated cardiomyopathy	a heart disorder	16,734	51 (0.30)	0 (0)	16501 (98.6)	None	None	Lonafarnib (inborn errors of metabolism)
MEN1	multiple endocrine neoplasia type 1	a disorder called multiple endocrine neoplasia (MEN1)	7,493	14 (0.19)	0 (0)	7,218 (96.3)	None	None	N/A
MLH1	Lynch syndrome	some types of cancers	25,876	59 (0.23)	0 (0)	25402 (98.2)	None	None	N/A
MSH2	Lynch syndrome	some types of cancers	72,738	46 (<0.01)	0 (0)	72,184 (99.2)	None	None	N/A
MSH6	Lynch syndrome	some types of cancers	34,268	124 (0.36)	0 (0)	33,438 (97.6)	None	None	N/A
MUTYH	MUTYH-associated polyposis	some types of cancers	8,260	134 (1.6)	0 (0)	7,880 (95.4)	None	None	N/A
MYBPC3	hypertrophic cardiomyopathy	a heart disorder	14,270	164 (1.1)	0 (0)	13,658 (95.7)	None	None	N/A
MYH11	familial thoracic aortic aneurysm and aortic dissection	a blood vessel disorder	70,141	17 (<0.01)	0 (0)	69,365 (98.9)	None	None	N/A
MYH7	dilated cardiomyopathy and hypertro phic cardiomyopathy	a heart disorder	12,454	135 (1.1)	0 (0)	11,735 (94.2)	None	None	N/A
MYL2	hypertrophic cardiomyopathy	a heart disorder	8,283	20 (0.24)	0 (0)	8,150 (98.4)	None	None	N/A
MYL3	hypertrophic cardiomyopathy	a heart disorder	11,647	10 (<0.01)	0 (0)	11,576 (99.4)	None	None	N/A

				ClinVar Significance (as of July 2024)					
Gene	Condition associated with this gene	What it is	Total # of Variants	Risk Factor, Likely Pathogenic, or Pathogenic (%)	Drug Response (%)	Undefined/Uncertain (%)	Variants with Risk Factor, Likely Pathogenic, or Pathogenic Significance & Allele Frequency ≥ 0.10 in African Ancestry Population (Variant Type; Allele Count; Allele Frequency)	Variants with Drug Response Significance & Allele Frequency ≥ 0.10 in African Ancestry Population (Variant Type; Allele Count; Allele Frequency)	FDA PGx Drug Labeling Section (Therapeutic Area; N/A: total % alleles with ClinVar drug response significance is 0; None: total % alleles with ClinVar drug response significance is > 0)
NF2	neurofibromatosis type 2	a disorder called neurofibromatosis	38,021	3 (<0.01)	0 (0)	37,729 (99.2)	None	None	N/A
OTC	ornithine carbamoyltransferase (OTC) deficiency	a disorder called ornithine carbamoyltransferase (OTC) deficiency	20,223	17 (<0.01)	0 (0)	20,080 (99.3)	None	None	N/A
PCSK9	familial hypercholesterolemia	dangerously high cholesterol	13,711	31 (0.23)	0 (0)	13,464 (98.2)	None	None	N/A
PKP2	arrhythmogenic cardiomyopathy	a heart disorder	43,146	65 (0.15)	0 (0)	42,794 (99.2)	None	None	N/A
PMS2	Lynch syndrome	some types of cancers	16,311	88 (0.54)	0 (0)	15,958 (97.8)	None	None	N/A
PRKAG2	hypertrophic cardiomyopathy	a heart disorder	137,152	3 (<0.01)	0 (0)	136,885 (99.8)	None	None	N/A
PTEN	PTEN hamartoma tumor syndrome	some types of cancer	46,291	30 (<0.01)	0 (0)	46,035 (99.4)	None	None	Capivasertib (oncology)
RB1	retinoblastoma	some types of cancer	63,987	11 (<0.01)	0 (0)	63,525 (99.3)	None	None	N/A
RET	multiple endocrine neoplasia type 2 (MEN2)	a disorder called multiple endocrine neoplasia (MEN2)	25,419	38 (0.15)	0 (0)	24,892 (97.9)	None	None	Cabozantinib, Selpercatinib (oncology)
RYR1	malignant hyperthermia	a disorder called malignant hyperthermia	66,889	252 (0.38)	16 (<0.01)	65,132 (97.4)	None	None	Desflurane, Isoflurane, Sevoflurane, Succinylcholine (Anesthesiology)
RYR2	catecholaminergic polymorphic ventricular tachycardia	a heart disorder	311,796	24 (<0.01)	0 (0)	310,202 (99.5)	None	None	N/A
SCN5A	Brugada syndrome and long QT syndrome 3	a heart disorder	41,494	138 (0.33)	0 (0)	40,804 (98.3)	3-38603929-T-C (SNV; 31,805; 0.294802)	None	N/A
SDHAF2	paragangliomas 2	some type of non-cancer growths	11,403	10 (<0.01)	0 (0)	11,335 (99.4)	None	None	N/A
SDHB	paragangliomas 4	some type of non-cancer growths	17,891	38 (0.21)	0 (0)	17,722 (99.1)	None	None	N/A
SDHC	paragangliomas 3	some type of non-cancer growths	27,437	12 (<0.01)	0 (0)	27,294 (99.5)	None	None	N/A
SDHD	paragangliomas 1	some type of non-cancer growths	28,557	15 (<0.01)	0 (0)	28,469 (99.7)	None	None	N/A
SMAD3	Loeys-Dietz syndrome	a blood vessel disorder	55,866	7 (<0.01)	0 (0)	55,647 (99.6)	None	None	N/A
SMAD4	juvenile polyposis syndrome	some types of cancers	27,243	2 (<0.01)	0 (0)	26,951 (98.9)	None	None	N/A
STK11	Peutz-Jeghers syndrome	some types of cancers	23,351	6 (<0.01)	0 (0)	22,981 (98.4)	None	None	N/A
TGFBR1	Loeys-Dietz syndrome	a blood vessel disorder	22,505	8 (<0.01)	0 (0)	22,353 (99.3)	None	None	N/A
TGFBR2	Loeys-Dietz syndrome	a blood vessel disorder	37,948	14 (<0.01)	0 (0)	37,749 (99.5)	None	None	N/A
TMEM43	arrhythmogenic cardiomyopathy	a heart disorder	12,208	5 (<0.01)	0 (0)	11,961 (98.0)	None	None	N/A
TNNI3	hypertrophic cardiomyopathy	a heart disorder	8,038	30 (0.37)	0 (0)	7,815 (97.2)	None	None	N/A
TNNT2	dilated cardiomyopathy and hypertro phic cardiomyopathy	a heart disorder	11,391	32 (0.28)	0 (0)	11,186 (98.2)	None	None	N/A
TP53	Li-Fraumeni syndrome	some types of cancers	14,403	76 (0.53)	1 (<0.01)	14,023 (97.4)	17-7676154-G-C (SNV; 41,433; 0.384037)	None	Pirtobrutinib, Venetoclax, Zanubrutinib (Oncology)
TPM1	hypertrophic cardiomyopathy	a heart disorder	16,207	12 (<0.01)	0 (0)	16,002 (98.7)	None	None	N/A
TSC1	tuberous sclerosis complex	a disorder called tuberous sclerosis complex	23,167	7 (<0.01)	0 (0)	22,558 (97.4)	None	None	N/A
TSC2	tuberous sclerosis complex	a disorder called tuberous sclerosis complex	30,105	59 (0.20)	0 (0)	28,200 (93.7)	None	None	N/A

				ClinVar Significance (as of July 2024)					
Gene	Condition associated with this gene	What it is	Total # of Variants	Risk Factor, Likely Pathogenic, or Pathogenic (%)	Drug Response (%)	Undefined/Uncertain (%)	Variants with Risk Factor, Likely Pathogenic, or Pathogenic Significance & Allele Frequency ≥ 0.10 in African Ancestry Population (Variant Type; Allele Count; Allele Frequency)	Variants with Drug Response Significance & Allele Frequency ≥ 0.10 in African Ancestry Population (Variant Type; Allele Count; Allele Frequency)	FDA PGx Drug Labeling Section (Therapeutic Area; N/A: total % alleles with ClinVar drug response significance is 0; None: total % alleles with ClinVar drug response significance is > 0)
VHL	von Hippel-Lindau syndrome	some types of cancer	10,030	29 (0.29)	0 (0)	9,817 (97.9)	None	None	N/A
WT1	Wilms tumor	a type of cancer called Wilms tumor	33,255	5 (<0.01)	1 (<0.01)	32,987 (99.2)	None	None	Belzutifan (oncology)

SNV = single nuvleotide variant
