# Supplementary material for: Opportunities amid complexities in returning genetic results to black precision medicine research participants: Interview themes in context with open all of us data
Source: J Clin Transl Sci. 2025 Apr 11;9(1):e89. doi: 10.1017/cts.2025.67 (PMC12089846; doi:10.1017/cts.2025.67)
Supplement: Hendricks-Sturrup et al. supplementary material 3 — Hendricks-Sturrup et al. supplementary material [file S2059866125000676sup003.pdf]

PGx Biomarker	Drug interactions considered	Total # of Variants	ClinVar Significance (as of July 2024)<br>Drug Response (%)	Undefined/Uncertain (%)	Variants with <u>Drug Response Significance</u> & <u>Allele Frequency ≥ 0.10</u> in African Ancestry Population (Variant Type; Allele Count; Allele Frequency)
	amitriptyline (Elavil®)				
	citalopram (Celexa®)*				
	clobazam (Onfi®)*				
	clomipramine (Anafranil®)				
	clopidogrel (Plavix®)*				
	doxepin (Sinequan®)*				
	escitalopram (Lexapro®)*				
	imipramine (Tofranil®)				
	sertraline (Zoloft®)				10-94761900-C-T (SNV; 107,890; 0.222523)
	trimipramine (Surmontil®)				10-94775367-A-G (SNV; 107,886; 0.198450)
CYP2C19	voriconazole (Vfend®)*	47,056	34 (0.07)	47018 (99.9)	10-94781859-G-A (SNV; 107,890; 0.177876)
	capecitabine (Xeloda®)*				1-97883329-A-G (SNV; 44,373; 0.411265)
DPYD	fluorouracil (Adrucil®)*	340,350	18 (0.01)	340235 (99.9)	1-97515839-T-C (SNV; 17,056; 0.158081)
	chloramphenicol				
	dabrafenib (Tafinlar®)				
	dapsone				
	hydroxychloroquine (Plaquenil®				
	local anesthetic-containing drug				
	mafenide (Sulfamylon®)				
	methylene blue				
	nalidixic acid (NegGram®)				
	nitrofurantoin (Macrobid®, Mac				
	pegloticase (Krystexxa®)				
	phenazopyridine				
	primaquine				
	probenecid (Col-Benemid®)				
	rasburicase (Elitek®)				
	sodium nitrite				
	sulfacetamide				
	sulfamethoxazole/trimethoprim				
	sulfanilamide				
	sulfasalazine (Azulfidine®)				
	Sulfonylurea drugs [chlorpropar				
G6PD	tafenoquine (Krintafel®)	8,788	0 (0)	8646 (98.4)	None
	azathioprine (Imuran®)				
	mercaptopurine (Purinethol®)				
NUDT15	thioguanine	7,907	4 (0.05)	7903 (99.9)	None
SLCO1B1	simvastatin (Zocor®)	45,383	1 (0.002)	45316 (99.9)	None
	azathioprine (Imuran®)				
	mercaptopurine (Purinethol®)				
TPMT	thioguanine	13,623	11 (0.08)	13,610 (99.9)	None
	atazanavir (Reyataz®)				
	belinostat (Beleodaq®)				2-233760233-C-CAT (insertion; 107,882; 0.401207)
UGT1A1	irinotecan (Camptosar®)	8,755	5 (0.06)	8697 (99.3)	2-233757136-G-A (SNV; 107,828; 0.305394)

*Contains FDA PGx Biomarker Drug Label

SNV = single nuvleotide variant
